# The intrinsic dependence structure of peak, volume, duration, and average intensity of hyetographs and hydrographs

**DOI:** 10.1002/wrcr.20221

**Published:** 2013-06-17

**Authors:** Francesco Serinaldi, Chris G Kilsby

**Affiliations:** 1 School of Civil Engineering and Geosciences, Newcastle UniversityNewcastle Upon Tyne, UK; 2 Willis Research NetworkLondon, UK

**Keywords:** hydrographs, hyetographs, dependence structures, copulas, bootstrap, multivariate distributions

## Abstract

[1] The information contained in hyetographs and hydrographs is often synthesized by using key properties such as the peak or maximum value *X_p_*, volume *V*, duration *D*, and average intensity *I*. These variables play a fundamental role in hydrologic engineering as they are used, for instance, to define design hyetographs and hydrographs as well as to model and simulate the rainfall and streamflow processes. Given their inherent variability and the empirical evidence of the presence of a significant degree of association, such quantities have been studied as correlated random variables suitable to be modeled by multivariate joint distribution functions. The advent of copulas in geosciences simplified the inference procedures allowing for splitting the analysis of the marginal distributions and the study of the so-called dependence structure or copula. However, the attention paid to the modeling task has overlooked a more thorough study of the true nature and origin of the relationships that link 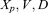, and *I*. In this study, we apply a set of ad hoc bootstrap algorithms to investigate these aspects by analyzing the hyetographs and hydrographs extracted from 282 daily rainfall series from central eastern Europe, three 5 min rainfall series from central Italy, 80 daily streamflow series from the continental United States, and two sets of 200 simulated universal multifractal time series. Our results show that all the pairwise dependence structures between 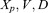, and *I* exhibit some key properties that can be reproduced by simple bootstrap algorithms that rely on a standard univariate resampling without resort to multivariate techniques. Therefore, the strong similarities between the observed dependence structures and the agreement between the observed and bootstrap samples suggest the existence of a numerical generating mechanism based on the superposition of the effects of sampling data at finite time steps and the process of summing realizations of independent random variables over random durations. We also show that the pairwise dependence structures are weakly dependent on the internal patterns of the hyetographs and hydrographs, meaning that the temporal evolution of the rainfall and runoff events marginally influences the mutual relationships of 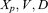, and *I*. Finally, our findings point out that subtle and often overlooked deterministic relationships between the properties of the event hyetographs and hydrographs exist. Confusing these relationships with genuine stochastic relationships can lead to an incorrect application of multivariate distributions and copulas and to misleading results.

## 1. Introduction

[2] In hydrologic engineering, several design and modeling problems are tackled by using a so-called event-based approach. For example, in flood risk assessment, the floodplain corresponding to a given return period *T* is obtained by driving flow routing models with design hydrographs whose shape synthesizes the temporal evolution of the observed flood events and the peak *X_p_* assumes the value corresponding to a prescribed probability of exceedance or return period resulting from a univariate frequency analysis [e.g., *Di Baldassarre et al*., 2010; *Grimaldi et al*., 2012a; *Serinaldi and Grimaldi*, 2011]. When the hydrographs do not result from the simulation of rainfall series and a subsequent continuous rainfall-runoff transformation [*Grimaldi et al*., 2012], they are defined from design hyetographs that synthesize the temporal evolution of rainfall storms and are often characterized by a peak value resulting from a univariate frequency analysis. Both hyetographs and hydrographs are complex objects that are characterized by several properties, such as *X_p_*, volume *V*, duration *D*, and average intensity *I*, which can be of interest in practical applications. These properties are commonly treated as random variables owing to the inherent variability of their values and the complexity of the rainfall and runoff processes. In practical applications, this study is often limited to a univariate frequency analysis of *X_p_* or *I* summarized by intensity-duration-frequency curves [*Chow et al*., 1988] for rainfall and flow-duration-frequency curves for discharge [e.g., *Meunier*, 2001]. However, as 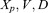, and *I* can all be of interest [*Salvadori and De Michele*, 2006; *Karmakar and Simonovic*, 2008, 2009; *Serinaldi and Grimaldi*, 2011; *Vandenberghe et al*., 2012], more refined multivariate techniques have been proposed in the literature. In more detail, as these variables can exhibit significant values of indices of association such as the Pearson product moment correlation coefficient 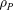, Kendall rank correlation coefficient 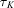, or Spearman correlation 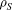, they have been deemed suitable to be modeled by joint distributions.

[3] The first attempts relied on the use of the meta-Gaussian framework under the hypothesis that the transformation of the marginal distributions into Gaussian can guarantee that the joint distribution is multivariate Gaussian. As is well known, this hypothesis is hardly ever fulfilled by real-world data; however, the difficulty of splitting the analysis and modeling of the marginal distributions and joint behavior (as well as computing limitations) limited the applications in that early stage. Since the late 1990s, a series of papers by Yue and coworkers [*Yue et al*., 1999; *Yue*, 2000a,2000b, 2000c, 2001a, 2001b, 2002] has revitalized this research area by showing the application of a set of suitable bivariate non-Gaussian distributions to analyze hyetograph and hydrograph properties. However, the literature on the topic has actually grown fast after the introduction of copulas in geosciences by the seminal paper of *De Michele and Salvadori* [2003]. The up-to-date list of references provided by the International Commission of Statistics in Hydrology of the International Association of Hydrological Sciences acknowledges this research activity (http://www.stahy.org/Activities/STAHYReferences/ReferencesonCopulaFunctiontopic/tabid/78/ Default.aspx).

[4] As copulas allow splitting the analyses of the marginal distribution and the so-called structure of dependence or copula, they provide a virtually infinite set of multivariate distributions with arbitrary marginals and dependence structure that fall outside the field of the meta-Gaussian and metaelliptical multivariate distributions. However, the increased ease of modeling and the simplified inference procedures as well as the availability of free statistical software has led to a focus on the inference procedures and applications overlooking to some extent a more thorough understanding of the variables at hand.

[5] In this study, we attempt to fill this gap. Instead of trying to find the best fitting copula that describes the hyetograph and hydrograph properties, we try to interpret the true nature of the dependence structures exhibited by 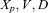, and *I* and their generating mechanism. The analysis is based on a large data set of rainfall and streamflow time series in order to support the generality of the results. We use some simple bootstrap techniques that can be easily implemented to repeat the analysis on other data sets without requiring any specific knowledge of the multivariate frequency analysis and copulas. These ad hoc bootstrap algorithms allow checking the working hypotheses by a nonparametric framework free from modeling errors and uncertainty. A large set of time series simulated from universal multifractal processes is also used to further support the analysis and conclusions.

[6] This study is organized as follows. In section 2, some basic definitions of dependence structure and copula-related concepts are briefly recalled in order to introduce the subject of this study. Section 3 introduces the data sets used in the analyses. Sections 4 and 5 present the analyses and the results referring to hyetographs and hydrographs, respectively. In these sections, we also introduce the bootstrap algorithms used to test the working hypotheses deduced from theoretical remarks and the preliminary inspection of the pairwise dependence structures of 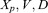, and *I*. Without loss of generality, the discussion is focused on one time series of each data set, whereas the results for all time series are provided as supporting information. A discussion about the relationship between marginal distributions and dependence structure resulting from the hypothesized generating processes is provided in section 6 along with the analysis of the synthetic multifractal time series. Conclusions in section 7 close this study.

## 2. Basic Definitions of Copula and Dependence Structure

[7] In this section, we will outline a few basic concepts concerning joint distributions and copulas. We refer the reader to *Nelsen* [2006], *Genest and Favre* [2007], *De Michele and Salvadori* [2007], and *Salvadori et al*. [2007], among others, for thorough introductions to copula theory, applications, and inference procedures. Denoting the marginal and joint distributions of 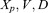, and *I* as 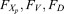, and *F_I_*, respectively, and 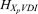, under some suitable conditions, *Sklar*'s [1959] theorem states that 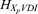 can be written as 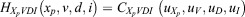, where 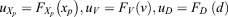, and 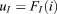 and 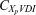 denote a copula function, namely, a distribution function with uniform marginals. As this study is not aimed at finding the best parametric model but rather at understanding the mechanism of generation of the observed dependence structures, we only need the empirical counterpart of the marginal distributions and copulas. In more detail, the analysis is based on the study of the pairwise scatterplots of the pairs of the transformed variables 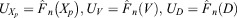, and 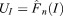, where

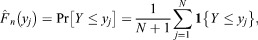
1where *Y* denotes a generic random variable, *N* is the sample size, and 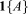 is the indicator function of an event *A*. In order to perform quantitative comparisons between the pairwise dependence structures, we also use the empirical estimator of a bivariate distribution 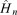, which is the bivariate counterpart of the univariate empirical distribution function:

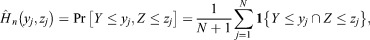
2where *Y* and *Z* denote two generic random variables. The values assumed by 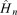 can be seen as the realizations 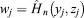 of a random variable *W*, and by Sklar's theorem, they are also an estimate of the copula 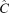 values. Therefore, the empirical distribution function of the empirical copula values can be defined as

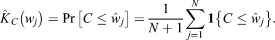
3

[8] This distribution is also known as Kendall distribution (or measure) function [*Genest and Rivest*, 1993, 2001; *Salvadori et al*., 2011] and is used in this study to compute the two-sample Kolmogorov-Smirnov (KS) statistic in order to compare the observed dependence structures and those obtained by the bootstrap algorithms described in the next sections.

## 3. Data Sets and Preprocessing Procedures

### 3.1. Rainfall Data and Hyetograph Selection

**Figure 1:**
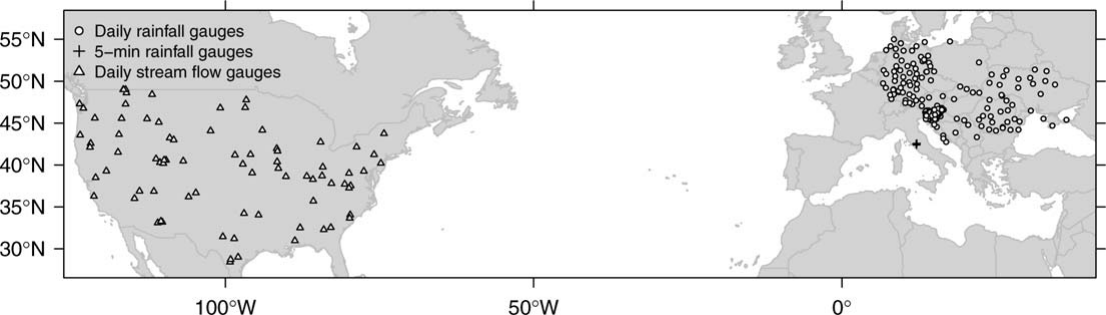
Map of rainfall and stream gauges used in the analyses.

**Figure 2:**
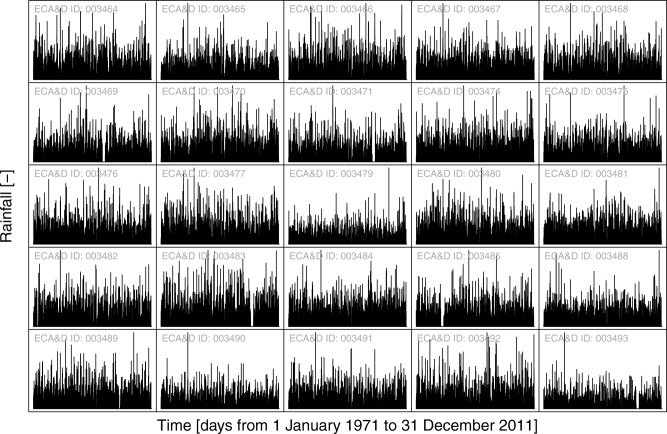
Subset of 25 rainfall series extracted from the 282 ECA&D daily series analyzed in this study. All series have the same length and cover the period from 1 January 1971 to 31 December 2011. The *y* axes have different scales for a better visualization; the range of rainfall values in each part is not reported because the analyses are based on the standardized ranks and the purpose of the diagrams is purely illustrative.

[9] The rainfall data consist of 41 years of daily rainfall records spanning from 1971 to 2011 for 282 stations in central eastern Europe (Figure 1) with less than 5% of missing data. The data are provided by the Royal Netherlands Meteorological Institute through the European Climate Assessment and Dataset (ECA&D) project [*Klein Tank et al*. [2002] and were downloaded from the ECA&D website (http://eca.knmi.nl/dailydata/predefinedseries.php). A subset of 25 series is shown in Figure 2. It is worth noting the actual magnitude of the streamflow observations does not matter in the following analyses, as we deal with rank-based variables ranging in the unit hypercube. The automatic checks based on the ECA&D data flagging codes were complemented by a visual inspection of each time series. The daily data set is complemented by three rainfall series at 5 min time resolution previously studied and modeled by *Serinaldi* [2010] in order to assess the effect of temporal resolution and seasonality.

[10] Following *Yue* [2000a, 2000c, 2001a, 2002] the event hyetographs of the daily rainfall time series are defined as continuous sequences of positive daily rainfall values separated by one or more dry days. This definition is coherent with the short memory that is often exhibited by daily rainfall data [e.g., *Serinaldi*, 2009, and references therein].

[11] For the 5 min rainfall data, storm events are commonly selected by algorithms devised to identify independent storm events such as the *Restrepo-Posada and Eagleson* [1982] method or by experts' considerations based on the climate of the area under study. In order to study the intrinsic properties of clusters of positive rainfall data recorded at different time scales, our analyses focus on consecutive sequences of positive 5 min rainfall values. Therefore, for the 5 min data we apply the same definition of event hyetograph used for the daily data, keeping in mind that we could not cope with physically consistent storm events, and the effect of dry intervals within an event is not accounted for as it requires further extensive analyses beyond the scope of this study.

### 3.2. Streamflow Data and Hydrograph Selection

**Figure 3:**
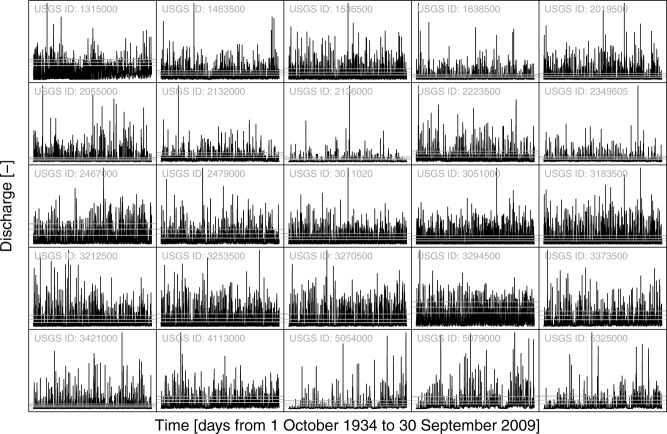
Subset of 25 streamflow series extracted from the 80 USGS daily series analyzed in this study. All series have the same length and cover the period from 1 October 1934 to 30 September 2009. The *y* axes have different scales for a better visualization; the range of streamflow values in each part is not reported because the analyses are based on the standardized ranks and the purpose of the diagrams is purely an illustration of the different regimes. The horizontal gray lines denote the thresholds at the 80th, 90th, and 95th percentile that are used to select the upper part of the hydrographs.

[12] The data consist of 74 water years of daily streamflow records spanning from 1935 to 2009 for 80 stations in the continental United States (Figure 1). The data set was retrieved from the US Geological Survey (USGS) website (http://waterdata.usgs.gov/nwis) along with the corresponding metadata. Almost all rivers and creeks are regulated by lakes, reservoirs, power plants, and diversions for irrigation, industrial, and municipal supply, thus influencing, to various degrees, the properties of the streamflow records. In this data set, 19 series show zero streamflow values at times. Figure 3 shows 25 examples of time series exhibiting a wide range of streamflow regimes.

[13] The possible lack of stationarity related to human regulations can be also neglected as the aim is to select the part of the hydrographs exceeding a given threshold and collect a wide range of heterogeneous cases. For instance, the selected hydrographs can be rather similar along a time series when the series is reasonably stationary and dominated by the seasonality, or rather dissimilar when the time series shows evident nonstationarity. In other words, since this study does not deal with inference and modeling, if the magnitude of the events increases over time or small and large events alternate along the time series, it does not matter in the present analyses as these events are simply treated as independent clusters of numbers (streamflow values). On the other hand, nonstationarity may generate possible exotic dependence structures which make the results more general. For the sake of completeness, it should be mentioned that nonstationarity can almost always be ascribed to flood control policies, multiple-use reservoir storage plans, abstraction for power plants, diversions for irrigation, or other human activities.

[14] In a similar way as for hyetographs, the analysis of the hydrograph properties requires the selection of the event hydrographs. As this selection requires an accurate analysis to identify the start of the rising limb and the end of the recession limb, and this identification can be rather arbitrary [*Smakhtin*, 2001]; in the present context we adopt a pragmatic approach. The event hydrographs (the upper parts of a streamflow series) are selected by using three different thresholds corresponding to the 80th, 90th, and 95th percentiles of the discharge measurements. In this way, we obtain a full picture of possible scenarios: for the lowest threshold we can select hydrographs that are not so extreme and show longer durations, whereas the highest threshold allows focusing on the extreme events. This threshold analysis on a large and heterogeneous data set extends the results reported by *Grimaldi and Serinaldi* [2006a] and *Karmakar and Simonovic* [2009]. Moreover, since the number of selected events decreases as the threshold increases, the effect of the sample size is also taken into account.

[15] Finally, it should be mentioned that in dealing with rank transformed data, a problem we face is the presence of the so-called “ties,” namely, sets of identical values resulting from the finite resolution of the measurement instruments and the sampling time intervals. The measurement resolution can affect 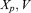, and the time resolution influences *D*, whereas both impact on 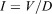. In this respect, ties are treated by using the method proposed by *Vandenberghe et al*. [2010] and subsequently applied by *Gyasi-Agyei* [2011a,[Bibr b20]].

## 4. Hyetograph Analysis

### 4.1. Preliminary Remarks

**Figure 4:**
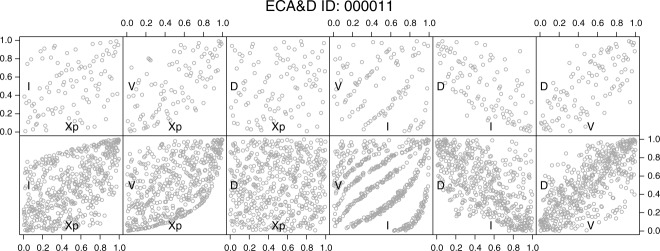
Pairwise scatterplots of the standardized ranks of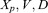, and *I* for the ECA&D station number 000011. Properties of the first (top) 100 and (bottom) 500 hyetographs that are most extreme in terms of *X_p_*.

[16] This study is motivated by the observation of some particular properties exhibited by the pairwise scatterplots of 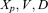, and *I* displayed in the literature and some subsequent conceptual considerations. We introduce the discussion by analyzing the properties of the hyetographs extracted from one daily rainfall series. As previously mentioned, we work with standardized ranks 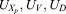, and *U_I_*, but for ease of notation and without ambiguity, the variables in the diagrams are denoted as 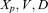, and *I*. The top row of Figure 4 shows the pairwise scatterplots of the hyetograph properties of the ECA&D station number 000011 (Kremsmuenster, Austria). The points refer to the 100 hyetographs with the highest peaks: this choice corresponds to the selection of about two events per year in a peak-over-threshold approach. The events can be considered independent based on the discussion reported in the previous sections; however, as is discussed later, this hypothesis does not influence the analysis. Figure 4 highlights some properties generally recognized in the literature such as the positive correlation of the pairs 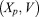 and 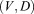 and the negative correlation of the pairs 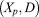 and 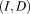. However, a closer look at the top 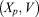 pair highlights the existence of an apparent lower boundary in the bottom right area of the unit square. This boundary appears more clearly by considering the first 500 most extreme events in term of *X_p_* (bottom row in Figure 4) and characterizes the pairs 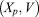 and 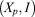. In particular, the almost uniform concentration of points (events) that lies along the boundary corresponds to the 1 day events for which 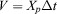, where 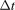 is the time resolution (suitably rescaled to obtain the required measure unit for the volume). The recognition of these patterns allows drawing further remarks. In a discrete sequence of 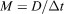 positive values 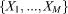, we have 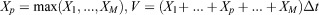, and 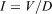. Therefore, it follows that 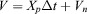, where 
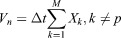
, denotes the “net” volume. The values of *V* cannot be smaller than 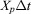, thus introducing a boundary condition that tends to be more prominent when the duration is short and 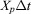 is large compared to *V_n_*. *Grimaldi and Serinaldi* [2006b] and *Serinaldi* [2013] already highlighted this aspect mentioning its physical/geometrical nature. The existence of such relationships between the characteristics of sequences of observations that define a hyetograph (and a hydrograph) raises a question about their true origin and the nature of the observed dependence structures between 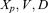, and *I*. These aspects are studied in the next sections.

### 4.2. Analysis of Daily Data

**Figure 5:**
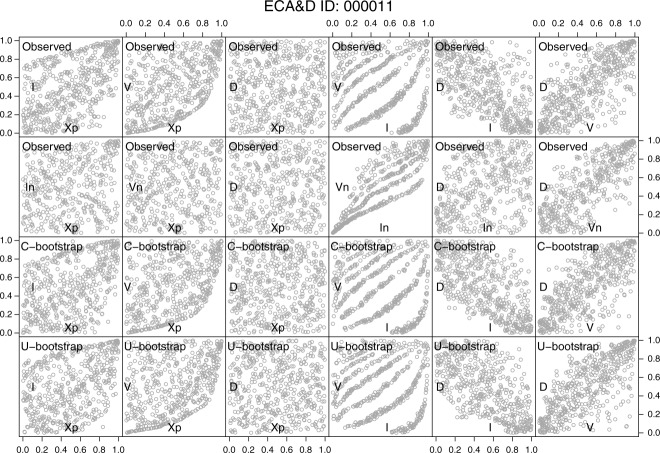
Pairwise scatterplots of the standardized ranks of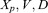, and *I* for the ECA&D station number 000011. The first row refers to the properties of the first 500 hyetographs that are most extreme in terms of *X_p_*. The second row corresponds to the “net” properties obtained by removing *X_p_* from the computation of *V*. The third and fourth rows refer to the hyetograph properties obtained by the C-boot and U-boot algorithms described in the text.

[17] Based on the previous remarks, we formulate the working hypothesis that the mutual relationships between 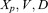, and *I* can be explained as a general and natural result of taking the maximum and summation of positive random variables over random durations. To test this assumption, we first assess the pairwise relationships of the net characteristics defined as 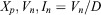, and *D*. For 1 day events, 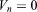 and 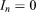, thus introducing ties in the normalized ranks that reflect the discrete-continuous nature of the marginal distributions of *V_n_* and *I_n_*. In order to provide a clear comparison, we focus on the continuous part of the bivariate relationships by removing the pairs corresponding to 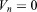. The pairwise scatterplots of the original 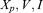, and *D* (already reported in the bottom row of Figure 4) and those of 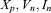, and *D* are shown in the first two rows of Figure 5.

[18] The pairs 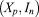 and 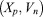 no longer exhibit any lower boundary in the bottom right corner and show an almost uniform scatter in the unit square denoting a weak correlation. The pair 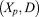 is obviously almost unchanged as the only difference with the corresponding pair in the first line is the removal of the pairs corresponding to 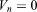.

[19] The pair 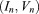 shows a stronger association compared to the original 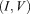 because of the removal of the peak record of each event. Indeed, since a hyetograph (especially at daily scale) is usually characterized by a spike and a number of mid-low records, after removing the peak value, the distribution function of the remaining observations (within each event) is rather uniform (or, at least, less skewed), and the functional relationship 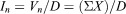 is more evident because the summation is taken over values that are not affected by the high variability of the peak. The stripe-like shape of the dependence structures of 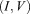 and 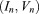 depends upon the discrete nature of *D*. Specifically, the treatment of ties applied in this study (which is a type of jittering) is effective if the relationship between the variables is purely stochastic, whereas the effect of the discretization of *D* is still evident for variables that are functionally linked to each other such as *V* and 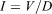. In other words, *I* results from a simple transformation of *V* through *D*: if *D* is discrete (or jittered), this property emerges in the dependence structure of 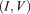 and 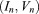.

[20] The pair 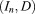 shows that the boundary in the bottom-left corner of the original pair 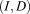 is removed, thus resulting in more weakly correlated random variables (an almost uniform scattering in the unit square). This behavior depends on the peak removal as well. The negative correlation between *I* and *D* is usually ascribed to the nature of rain storms; namely, long events with low intensity are associated with frontal systems, whereas short events with high intensity to convective phenomena. The pair 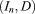 shows that this behavior is dominated by the peak rather than by the remaining observations. Once the largest observation is removed, shorter (longer) events exhibit lower (higher) average intensity, thus reverting the sign of the correlation. In other words, the higher average intensity exhibited by short events seems to be more related to the highest observation than to a process that is really more intense throughout the whole event duration. The diagrams discussed in the next section highlight that this property is even more evident at 5 min time scale.

[21] The pair 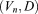 shows that the positive relationship between the original *V* and *D* values is preserved. Indeed, unlike the pair 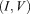, these variables are not linked by any explicit functional relationship, and the removal of the peak record keeps the ranks of *V* and *D* and their mutual association almost unchanged.

[22] The previous preliminary visual analysis indicates that 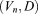 exhibit an evident and genuine stochastic correlation, whereas the other pairwise dependence structures are influenced by the presence of *X_p_* within the computation of *V*. When the geometrical boundary conditions related to the discrete sampling are removed, the relationships of the pairs 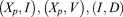 weaken, whereas the association between *I* and *V* strengthens because *X_p_*, which is weakly related to *V_n_*, does not influence the relationship 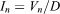. It is worth noting that this behavior is general as is shown by the 282 analogous diagrams reported in the supporting information.

[23] To further study the mechanism of generation of the pairwise dependence structures and provide a quantitative assessment, two different bootstrap algorithms named C-boot (conditional bootstrap) and U-boot (unconditional bootstrap) have been set up as follows:

[24] 1. Take *N* hyetographs that are the most extreme in terms of *X_p_* (or another property such as *V* or *I*) and build three data sets, namely, a vector with the *N* event durations, a vector with the *N* values of *X_p_* and a vector of all the observations 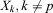 (i.e., the values to be used to compute *V_n_*). The observations of the *X_p_* and *X_k_* data sets must be flagged in order to retain the information concerning the duration;

[25] 2. Sample with replacement from the duration vector to obtain a new set of *N* values of *D*;

[26] 3. For each resampled *D* value, sample with replacement one value of *X_p_* (from the *X_p_* vector) corresponding to one of the events with duration equal to the resampled *D*. In this way, the sampling procedure of *X_p_* is conditioned to the event duration;

[27] 4. For each resampled value of *D*, sample *M* − 1 values of *X_k_* (from the vector *X_k_*) whose flag corresponds to the resampled duration *D*. In this way, the *X_k_* values are sampled from events with duration *D*;

[28] 5. For each resampled value *D*, compute 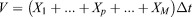 and 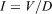 using the values obtained from the steps 2–4.

[29] The U-boot algorithm is similar to the C-boot, but the sampling procedure of *X_p_* and *X_k_* in the steps 3 and 4 is not conditioned to the duration flag. The two algorithms provide new sets of 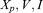, and *D* values by simply resampling from three vectors (i.e., the vectors in which 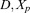, and *X_k_* are stored) without introducing any parametric or nonparametric copula and without accounting for the internal structure of the resampled sequences 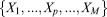.

[30] The pairwise scatterplots of the standardized ranks of one C-boot and U-boot simulation are shown in the third and fourth rows of Figure 5. The similarity between these diagrams and the corresponding diagrams shown in the first row is remarkable and holds for all 282 daily rainfall time series analyzed in this study (see supporting information). The algorithms can reproduce accurately all the pairwise dependence structures between 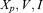, and *D*. In particular, the simulation mechanism can mimic the boundary that characterizes the pairs 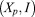 and 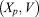.

**Figure 6:**
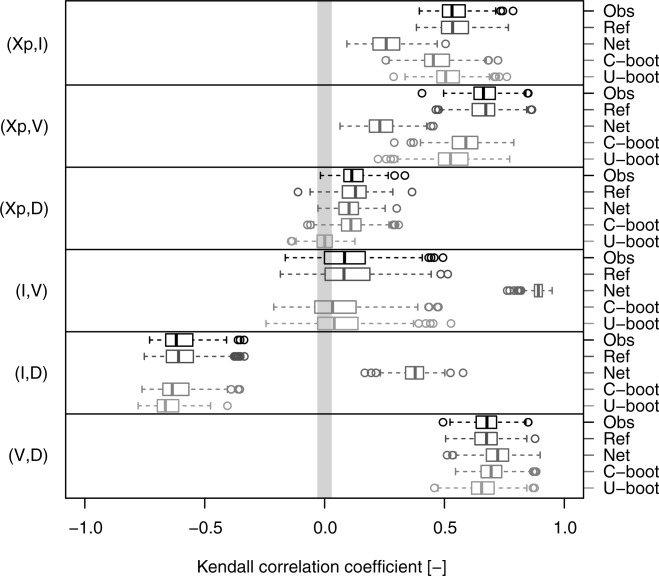
Box plots of pairwise Kendall correlation values referring to the hyetograph properties extracted from the 282 ECA&D daily rainfall series. Five data sets are compared for each pair of variables: (1) “Obs” (observed) refers to the original time series; (2) “Ref” (reference) refers to resampled standardized ranks and provides a picture of the variability of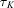 under the null hypothesis that the empirical copula is equal to the observed (see text for further details); (3) “Net” refers to the net properties; (4) “C-boot” refers to conditional bootstrap samples; and (5) “U-boot” refers to unconditional bootstrap samples. The gray stripe denotes the approximate 95% confidence interval of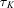 under the null hypothesis of independence.

[31] As a visual comparison is not enough to make inference and draw conclusions, a quantitative comparison was also performed. The agreement between the observed and simulated dependence structures is assessed by comparing their overall strength via the Kendall correlation and computing the KS statistic on the empirical Kendall distributions (equation (3)) of the observed and simulated data. The box plots in Figure 6 show the pairwise Kendall correlation values corresponding to the observed hyetograph properties extracted from the 282 daily rainfall time series (denoted as “Obs”) and the values referring to the net properties (denoted as “Net”), the C-boot and the U-boot data. Figure 6 also shows an additional reference case (denoted as “Ref”) obtained by resampling with replacement from the 4-D samples 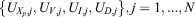 (see, e.g., *Efron and Tibshirani* [1993, pp. 49–50], for an example of bootstrap of 2-D samples). Thus, the Ref case describes the variability of the Kendall correlation under the null hypothesis that the simulated (bootstrapped) dependence structures are equal to those of the observed data unless an intrinsic statistical fluctuation. The box plots highlight the significant and systematic difference between the original and the net observations as well as the ability of the proposed algorithms to reproduce the pairwise correlation values and their variability. In particular, the C-boot performs well for the pairs 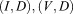, while both the algorithms (C-boot and U-boot) tend to slightly underestimate the correlation of the pairs 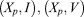, and 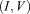. It should be noted that the box plot of the pair 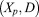 for U-boot falls within the gray stripe that denotes the approximate 95% confidence interval of 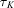 under the null hypothesis of independence as these variables are independently sampled by definition.

**Figure 7:**
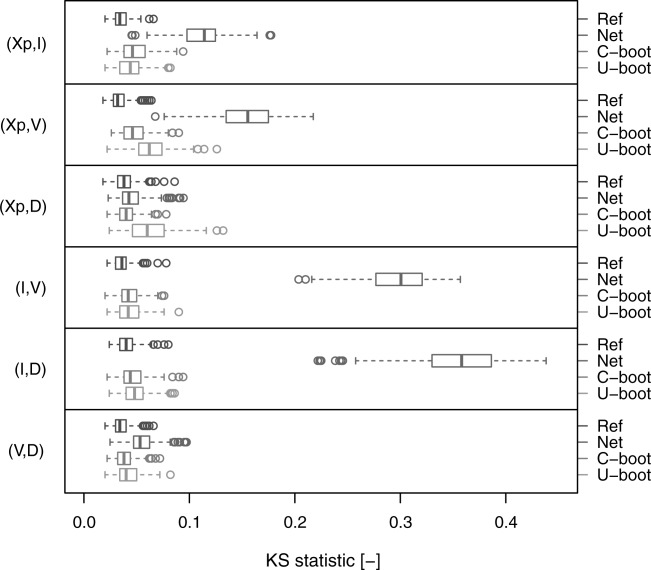
Box plots of the values of the KS statistic computed on the Kendall distributions referring to the pairwise empirical dependence structures. Each box plot describes the KS statistics obtained from 282 comparisons between the Kendall distributions corresponding to the observed samples and to the simulated samples (“C-boot” and “U-boot”). “Ref” provides a picture of the variability of the KS statistic under the null hypothesis that the empirical copula is preserved and represents the reference for the other box plots. “Net” refers to the comparison between the observed and net dependence structures.

[32] Figure 7 shows the box plots of the KS statistic computed by comparing the Kendall distribution corresponding to the observed data and the Kendall distributions of the Ref, Net, C-boot, and U-boot data sets. The Ref case provides a picture of the KS distribution under the null hypothesis (the data come from the observed empirical copulas). The C-boot and U-boot algorithms return pairwise dependence structures that are coherent with the observed ones. As for the Kendall correlation, some discrepancy can be observed for the pairs 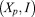 and 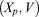. C-boot performs better than U-boot for 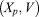 and 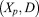. However, the C-boot and U-boot mechanisms generate copulas that are very close to the observed ones, thus explaining the dominant processes responsible of these dependence structures.

[33] These results confirm our working hypothesis about the nature of the dependence structures that link the hyetograph characteristics: they can be adequately explained by the intrinsic properties of sequences of independent random variables defined on a positive support and summed over random durations. On the other hand, the physical properties and internal structure of the rainfall events seem to play a marginal role. Distinguishing between *X_p_* values and the remaining observations 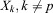, is sufficient to create sequences whose dependence structure is indistinguishable from that of the observed hyetographs without introducing further assumptions.

### 4.3. Analysis of 5 Min Data

[34] Three 5 min time series from central Italy are analyzed to explore the effect of the time scale and the seasonality on the dependence structures between 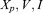, and *D*. The geographical location also allows accounting for a typical Mediterranean climate regime. As mentioned in section 3.1, the hyetographs were selected as continuous sequences of positive rainfall values, even though from a physical point of view, 6–7 h of no rain are commonly used to distinguish independent storm events in this area [*Grimaldi and Serinaldi*, 2006b; *Serinaldi*, 2010]. We stress again that our aim is to show that the key properties of the apparently heterogeneous dependence structures of 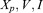, and *D* can be explained by a unique generating mechanism.

**Figure 8:**
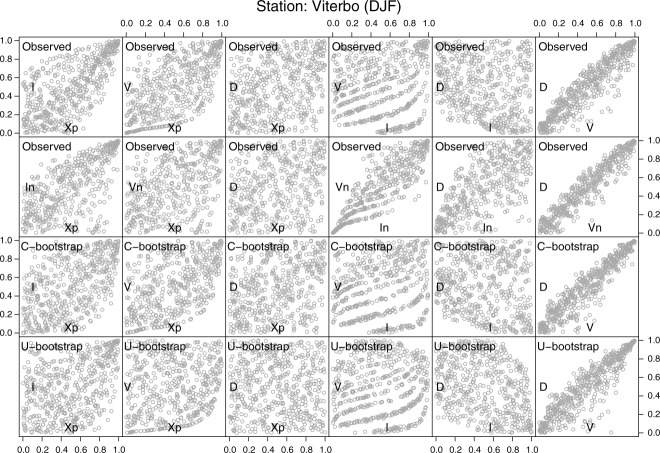
As in Figure 5 but for the winter 5 min rainfall series of Viterbo (Italy).

**Figure 9:**
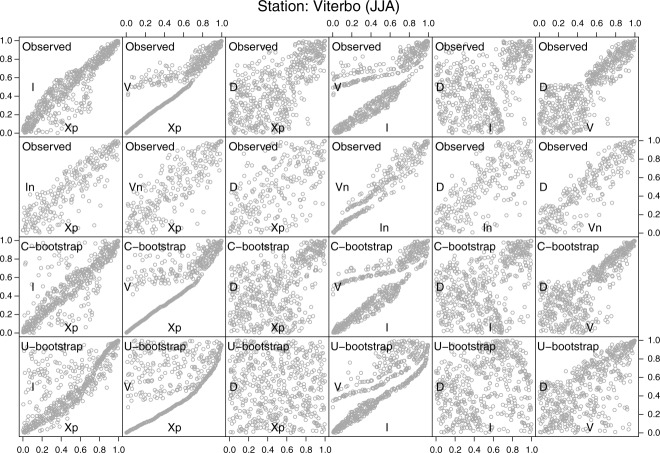
As in Figure 5 but for the summer 5 min rainfall series of Viterbo (Italy).

[35] Figures 8 and 9 show the pairwise scatterplots of the hyetograph properties extracted from the winter and summer subseries. For the summer season, it should be noted that many isolated events spanning only 5 min can be extracted as is shown by the boundary in the bottom left corner of the pair 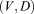, resulting from the randomization of the *D* ties. From these diagrams, one can draw the same conclusions discussed for the daily data set. In particular, it is worth noting the seasonal differences between the shapes of the clouds of points (and then follows, of the dependence structures), and the overall ability of the C-boot of reproducing them.

[36] As already mentioned, discrepancies are allowed as the diagrams compare the observations with just one bootstrap sample, and the algorithms are not intended to exactly reproduce the observed behavior but to show that the main characteristics of the observed dependence structures are substantially related to the hypothesized mechanism. Finally, we note that the stripe-like behavior exhibited by the pair 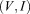 was already recognized by *Vandenberghe et al*. [2010] for the storm events extracted from a long 10 min rainfall series. In that case, the behavior is less evident because the authors selected the storm events by the *Restrepo-Posada and Eagleson* [1982] method, thus including dry intervals within each storm and obtaining a wider range of event durations.

## 5. Hydrograph Analysis

[37] Unlike the hyetograph analysis, the nature of the streamflow process and the threshold selection do not allow extracting a fixed number of hydrographs for all stations. For the rivers characterized by a strong seasonal pattern, the number of events is often close to the number of years, whereas a large number of events can be extracted for time series with weak seasonality. The drainage area plays an important role along with the perennial or ephemeral nature of the streamflow regime. The heterogeneous behavior of the 80 streamflow time series considered in this study allows the exploration of a variety of dependence structures apparently very different, thus providing a wide catalog of cases.

**Figure 10:**
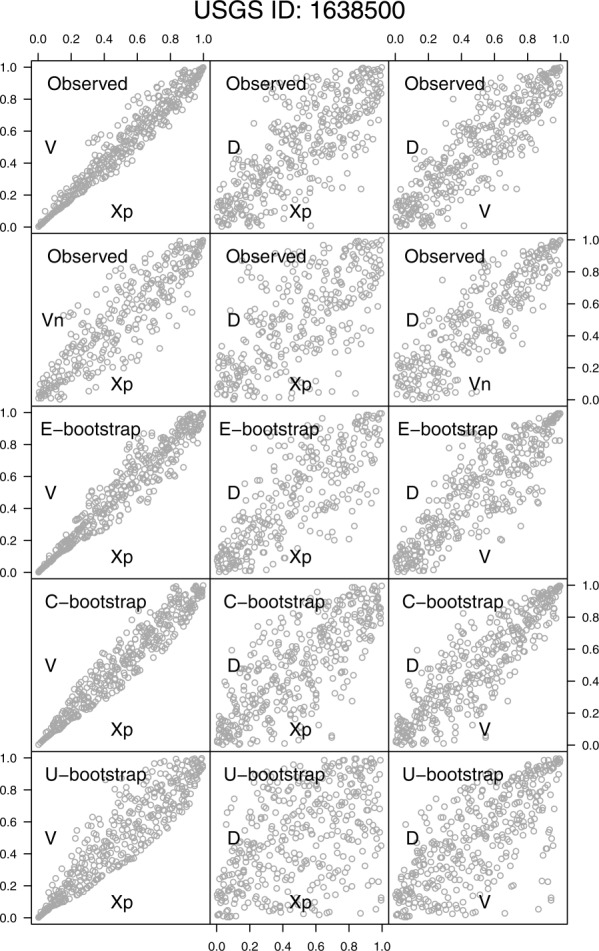
Pairwise scatterplots of the standardized ranks of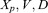 for the USGS station number 1638500. The first row refers to the properties of the hydrographs extracted by using the 95th percentile threshold. The second row corresponds to the “net” properties obtained by removing *X_p_* from the computation of *V*. The third, fourth, and fifth rows refer to the hydrograph properties obtained by the E-boot, C-boot, and U-boot algorithms, respectively, described in the text.

[38] As mentioned in section 3.2, the hydrograph analysis is performed on events extracted by using three different threshold values. The first row in Figure 10 shows the pairwise scatterplots of 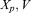, and *D* for the USGS station number 1638500 (Potomac River at Point of Rocks, Maryland) and the 95% threshold. The analysis focuses on these three variables since they are commonly used in the multivariate frequency analysis [*Yue et al*., 1999; *Yue*, 2000b,2001b; *Grimaldi and Serinaldi*, 2006a; *Shiau et al*., 2006; *Zhang and Singh*, 2007; *Karmakar and Simonovic*, 2008,^2009^; *Chebana and Ouarda*, 2009,^2011^; *Chowdhary et al*., 2011; *Aissia et al*., 2012; *Ganguli and Reddy*, 2013]. As for the hyetographs, we refer to the supporting information for the graphical results concerning the whole data set and the three thresholds.

[39] The pair 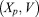 shows an apparent lower bound for low-mid values of *X_p_*. Analogous to the hyetographs, this behavior can be associated with the relationship 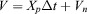, whose effect is more evident for streamflow series of rivers with weak seasonality and possibly ephemeral; in these cases, many events can be extracted, and some of them have quite a short duration and a *V* value close to *X_p_*. It is worth noting that this behavior is also exhibited by the hydrographs studied by *Klein et al*. [2010,2011] and the hydrographs simulated by *Vandenberghe et al*. [2012], corresponding to a small river located in central Italy [*Grimaldi et al*., 2012a]. Similar to the hyetograph analysis, the working hypothesis is that these dependence structures are the outcome of a process of summation of positive random variables over random durations. To test this assumption, the bootstrap algorithms used for the hyetograph analysis are slightly modified. In particular, we use three approaches: E-boot (event-based bootstrap), C-boot (bootstrap conditioned on duration), and U-boot (unconditioned bootstrap). As a hydrograph profile is generally smoother than a hyetograph owing to the persistence of the runoff process (at least, at the daily time scale), the modified bootstrap algorithms do not distinguish between the distributions of *X_p_* and 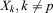. The E-boot algorithm is as follows:

[40] 1. Given a set of *N* events extracted from a time series, build two data sets, namely, a vector with the *N* event durations and a vector with the *X_k_* values. The observations of the *X_k_* data set are flagged in order to retain the information concerning the particular event and the duration of the event which they come from (this information is used in the E-boot and C-boot algorithms for hydrographs);

[41] 2. Sample with replacement from the vector of indices 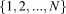 to obtain a new vector *L* of resampled indices;

[42] 3. For each index *l* in *L*, sample with replacement the *X_k_* values (from the vector *X_k_*) corresponding to the *l*th event (e.g., if the first element of *L* is 6, resample from the 6th event in the original sequence of events). In this way, the sampling procedure of *X_k_* is conditioned to the event, thus preserving the discharge distribution function within each event, but removing the internal temporal dependence;

[43] 4. For each resampled event, compute *V* and *X_p_*.

[44] The E-boot algorithm is devised to check the impact of the internal persistence (temporal dependence) of the discharge sequence on the dependence structure of the summary statistics 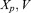, and *D*. This algorithm implicitly assumes that each event is characterized by a specific distribution function of the discharge values. This hypothesis is partly relaxed in the C-boot algorithm, whereby *X_k_* is sampled from all events with a given duration. The U-boot algorithm allows for sampling from the entire *X_k_* data set without any conditioning, thus assuming a unique distribution for all the discharge values *X_k_*. As for hyetographs, the dependence structures corresponding to the net volume *V_n_* are studied as well.

[45] The results are summarized in Figure 10. Moving from *V* to *V_n_* the lower bound in the pair 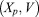 tends to disappear, and the association degree weakens. The spread of 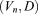 is slightly tighter than that of 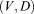. The E-boot provides a rather accurate reproduction of the observed scatterplots, thus denoting that the impact of the internal structure of the hydrographs does not influence the dependence structures very much. Some piece of information is lost when we move from the E-boot to C-boot; namely, the relationships between *V* and *D* seem to be slightly stronger than the observed, whereas the shape of the clouds 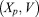 tends to change. The U-boot results show the importance of conditioning the sampling procedures to events with similar duration. Since the duration can be seen as an index to classify the events, the three algorithms highlight that the dependence structures between 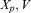, and *D* are mainly related to the process of sampling and summing up sets of independent random variables from a pool of suitable distribution functions over random durations.

**Figure 11:**
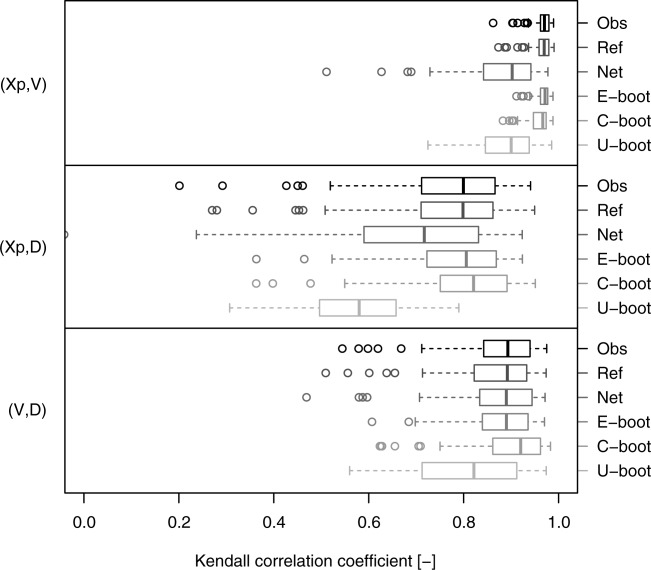
Box plots of pairwise Kendall correlation values referring to the hydrograph properties extracted from the 80 USGS daily streamflow series. Six data sets are compared for each pair of variables: (1) “Obs” (observed) refers to the original time series; (2) “Ref” (reference) refers to resampled standardized ranks and provides a picture of the variability of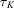 under the null hypothesis that the empirical copula is equal to the observed (see text for further details); (3) “Net” refers to the net properties; (4) “E-boot” to event-based bootstrap samples; (5) “C-boot” to conditional bootstrap samples; and (6) “U-boot” to unconditional bootstrap samples.

**Figure 12:**
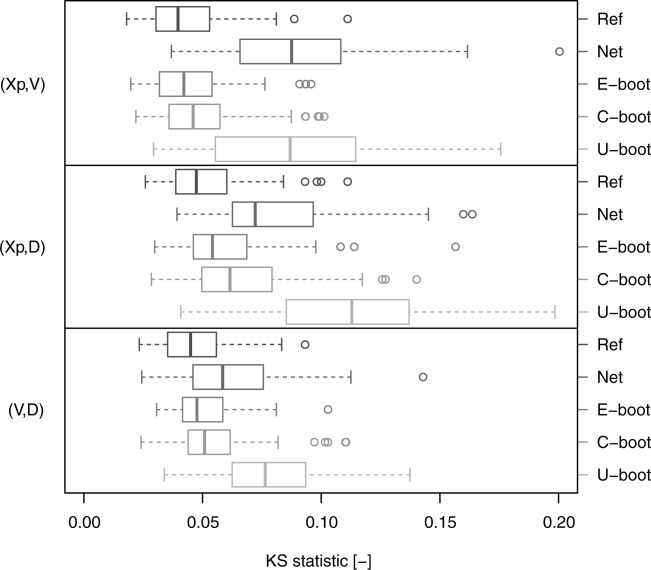
Box plots of the values of the KS statistic computed on the Kendall distributions referring to the pairwise empirical dependence structures. Each box plot describes the KS statistics obtained from 80 comparisons between the Kendall distributions corresponding to the observed samples and to the simulated samples (“E-boot,” “C-boot,” and “U-boot”). “Ref” provides a picture of the variability of the KS statistic under the null hypothesis that the empirical copula is preserved and represents the reference for the other box plots. “Net” refers to the comparison between the observed and net dependence structures.

[46] A quantitative assessment on the whole data set is performed by computing the Kendall correlation coefficient and the KS statistic. Figure 11 confirms the remarks drawn from Figure 10. The relationships of the pair 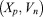 tend to be weaker compared to 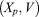, whereas the opposite holds for the pairs 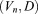 and 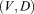. The E-boot yields the best reproduction of the Kendall correlation, whereas the C-boot samples are characterized by a slight positive bias for the pairs 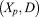 and 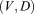. The U-boot algorithm produces a significant underestimation of the Kendall correlation for all pairs. The KS statistic computed on the Kendall distributions better highlights the agreement between the observed, net, and simulated dependence structures. Figure 12 confirms the significant difference between the observations and the net and U-boot samples. These results are coherent across the different thresholds (see diagrams in the supporting information). Small departures and discrepancies are expected and can be likely ascribed to the memory removal operated by the bootstrap procedures. However, these findings show that the suggested generating mechanism might explain the nature and shape of the dependence structures that link the hydrograph properties 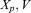, and *D*. The good performance of the bootstrap algorithms on a wide set of heterogeneous dependence structures further corroborates the generality of the conclusions.

## 6. Discussion

### 6.1. Relationship Between Marginal Distributions and Dependence Structures

[47] Even though the summation of independent random variable over random durations can explain several key features of the relationships between 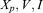, and *D*, the variety of dependence structures emerging in the observed data suggests that some additional factors act and specialize the shape of the dependence structures themselves. Copulas have been introduced as a mathematical representation able to split the marginal and joint behavior. Actually, they allow writing a joint distribution by making explicit the expression of the marginal distributions into the formula of the joint distribution and allow splitting the inference procedure by separating the analysis of marginals and dependence structure. However, these mathematical and inferential properties do not imply that the observed dependence structures of geophysical variables are not related to the marginal distributions at all, since the full joint distribution is the result of unique and coherent physical processes. This topic was the object of a debate in the scientific community summarized in a special issue of Extremes journal (see *Mikosch* [2006], *Genest and Rémillard* [2006], and the other contribution in that journal issue). In this study, the problem is treated from a pragmatic point of view distinguishing between making inference and understanding the underlying mechanism that generates the dependence structures. We use a simple Monte Carlo (MC) simulation to illustrate the impact of the generation process on the dependence structures by using an algorithm similar to the U-boot but fed with different parametric distributions for *X_k_* and *D*, which are fictitious variables in the present context. The MC algorithm is as follows:

[48] 1. Simulate *N* samples from a skewed distribution (e.g., a J-shaped exponential distribution) mimicking, for example, the hydrograph durations *D*. The values may be rounded to the first upper integer in order to obtain the discretization effect due to the time resolution (e.g., daily time steps);

[49] 2. For each simulated value of *D*, simulate a sample of length *D* from a skewed distribution defined in 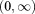 (e.g., the Weibull distribution). These values mimic the discharges *X_k_*. Each group of pseudodischarge values can be seen as a pseudohydrograph with no internal persistence. It should be noted that the parameters of the distribution of *X_k_* are chosen with no reference to real-world data;

[50] 3. Select the maximum value for each pseudohydrograph 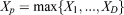;

[51] 4. Compute the sum of the elements of each pseudohydrograph 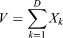 (the multiplicative effect of 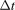 is not accounted for in this illustrative algorithm);

[52] 5. Compute the rescaled ranks of the simulated *X_p_* and *V* and draw the scatterplots.

[53] In this experiment, we used three different configurations, namely,

[54] 1. 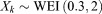, 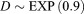;

[55] 2. 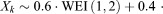 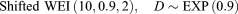;

[56] 3. 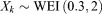, 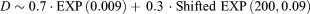;

[57] where the symbol “∼” denotes “distributed as,” “WEI” denotes “Weibull,” and “EXP” denotes “exponential.” Cases (2) and (3) involve mixtures of distributions for *X_k_* and *D*, respectively. Case (2) can mimic a bimodal streamflow distribution resulting from heterogeneous forcing causes (e.g., storms and snow melt) or different responses of a basin related to soil moisture thresholds that generate ordinary and extraordinary extreme events. Case (3) is less related to real-world situations but help understanding the impact of the *D* distribution.

**Figure 13:**
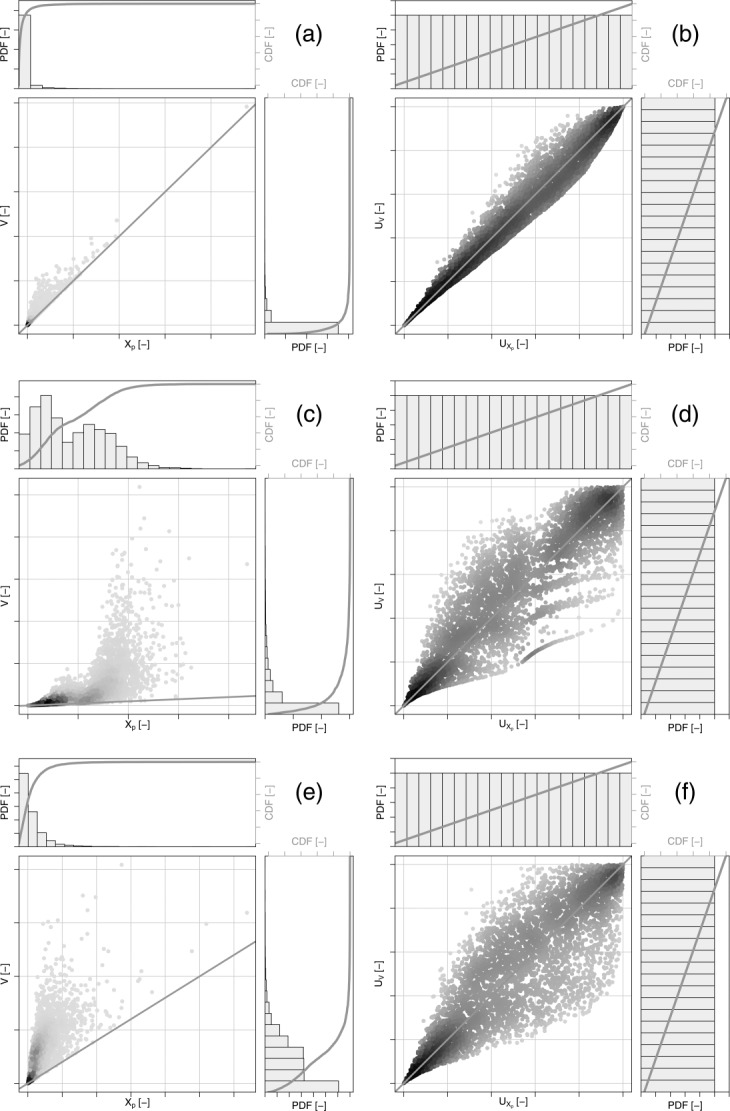
Joint density functions, marginal distributions, and dependence structures (empirical copulas) of 5000 pairs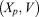 simulated by the MC algorithm described in the text. (a and b) “Case (1),” (c and d) “Case (2),” and (e and f) “Case (3)” (see text for further details). (a, c, and e) Joint densities in the main subfigures and the marginal PDFs and CDFs in the side subfigures. (b, d, and f) Dependence structures (empirical copula densities) corresponding to (a, c, and e). The side subfigures show the standard uniform PDFs and CDFs of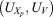.

[58] The joint density functions, the marginal distributions, and dependence structures (empirical copulas) corresponding to *N* = 5000 simulated pairs 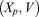 are shown in Figure 13. Figure 13a shows the cloud of data corresponding to Case (1) along with the marginal empirical probability density functions (PDFs) and cumulative distribution functions (CDFs). Figure 13b displays the relationships between the standardized ranks 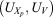 as well as their uniform marginal PDFs and CDFs. Figures 13c and 13d refer to Case 2, whereas Figures 13e and 13f refer to Case 3. Figures 13a, 13c, and 13e clearly show the presence of the boundary corresponding to 1:1 line and the rather different shapes of the marginal distributions of *X_p_* and *V*. The right side of Figure 13 focuses on the dependence structures once the marginals are filtered out. The different shapes of these dependence structures come from using different parent distributions for *X_k_* and *D* in the same generating algorithm and are intrinsically related to the marginal distributions of *X_p_* and V. This effect is evident in Figure 13c, where the mixed parent CDF for *X_k_* and a mixed marginal for *D* generate a bimodal marginal distribution of *X_p_* and a rather complex dependence structure which is locally clustered and asymmetric in all directions. The three dependence structures are characterized by a well defined lower boundary in the lower part of the clouds of points that is not stochastic but purely numerical (resulting from the condition 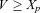). According to the copula theory, these dependence structures can be studied independently of the marginal distributions (reported in the left); however, without knowing the shape of the parent distribution of *X_k_*, the marginal distribution of *D*, and the generating mechanism, some physical and numerical relationships between the variables might be easily confused with stochastic relationships and modeled with copulas that provide an incorrect representation and interpretation of the phenomenon under study. In other words, even though copulas allow coupling arbitrary marginals and dependence structures, this does not mean that this is the most appropriate method, and it may overlook important properties. When the preliminary analyses highlight a plausible generating mechanism, this introduces further information, and the selection of the joint distribution is no longer only a matter of minimization of some performance criteria but requires coherent choices of marginals and copulas that fulfill the numerical and/or physical constraints related to the underlying process.

[59] In this context, the size of the sample plays a key role in the correct analysis of the data. As hydrological analyses are often focused on either annual maxima or a limited number of peak-over-threshold observations per year, the available sample size is commonly rather small and can easily hide the actual nature of the relationships between the studied variables and fundamental aspects such as the numerical boundary discussed previously. Figure 4 provides an example of such a situation and highlights the importance of an adequate understanding of the processes before performing statistical analyses and modeling.

### 6.2. Dependence Structures Resulting From Theoretical Signals

**Figure 14:**
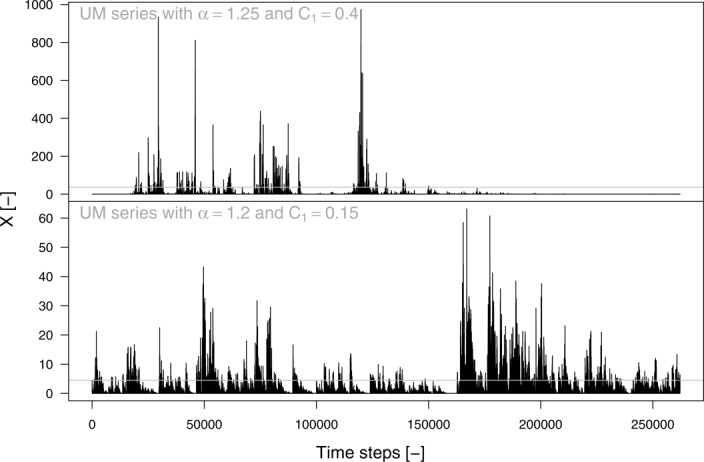
Examples of time series simulated from a universal multifractal process. (top) Universal multifractal time series with parameters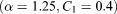. Gray line denotes the threshold at the 99.5th percentile used to select pseudoevents (see text for further details). (bottom) Universal multifractal signal with parameters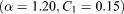. Gray line denotes the threshold at the 95th percentile used to select pseudoevents.

[60] The generating mechanism discussed throughout this study raises another fundamental question: is this mechanism general? or in other words, is it linked to physical features of the rainfall and runoff data or does it characterize other types of signals? To answer this question we have simulated 200 time series with size 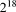 from a universal multifractal model [*Schertzer and Lovejoy*, 1987] with two different sets of parameters 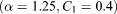 and 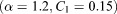. These processes were chosen to test our hypotheses against synthetic events (which can be seen as pseudohyetographs or pseudohydrographs) extracted from a signal with a rather complex temporal structure, which is expected to impact on the dependence structures. For each time series, the pseudoevents are selected as continuous sequences of values that exceed the 99.5th percentile for the first set of parameters and the 95th percentile for the second set. Two examples of these time series are shown in Figure 14 along with the selected thresholds. We have considered the pairwise relationships between 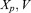, and *D* and the same bootstrap algorithms applied in the hydrograph analysis.

**Figure 15:**
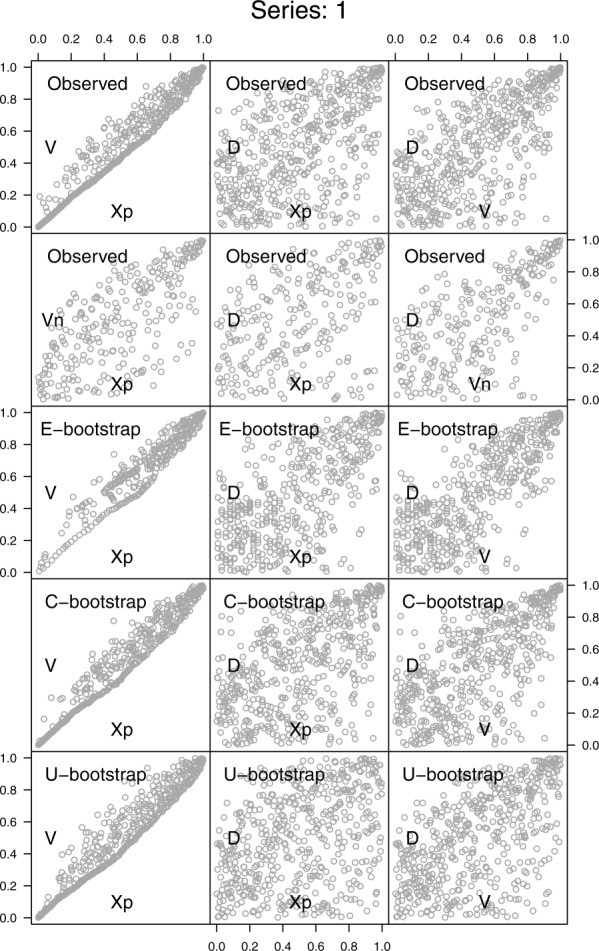
Pairwise scatterplots of the standardized ranks of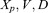 for one time series simulated from a universal multifractal process with parameters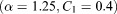. The first row refers to the properties of the pseudoevents extracted by using the 99.5th percentile threshold. The second row corresponds to the “net” properties obtained by removing *X_p_* from the computation of *V*. The third, fourth, and fifth rows refer to the hydrograph properties obtained by the E-boot, C-boot, and U-boot algorithms, respectively, described in the text.

[61] Figure 15 shows the pairwise scatterplots of the properties of the pseudoevents extracted from a time series following a universal multifractal process with the first set of parameters (this figure is analogous to Figure 10). Nearly half of the selected events has unit duration. The selection highlights the impact of removing *X_p_* from the computation of *V*. As for the hydrographs, the bootstrap algorithms reproduce key features of the pairwise relationships.

[62] A closer look at the performance of the bootstrap procedures is provided by the Kendall correlation and the KS statistic shown in Figures 6 and 7 (analogous to Figures 1 and 2). The Kendall correlation is best reproduced by the C-boot algorithm, which performs rather well also in terms of KS statistic. Obviously, consistent discrepancies are present in light of the complex nature of the signal; however, since the aim is not to reproduce exactly the dependence structures, the agreement is satisfactory if we keep in mind the unavoidable influence of the multifractal properties of the signal.

**Figure 16:**
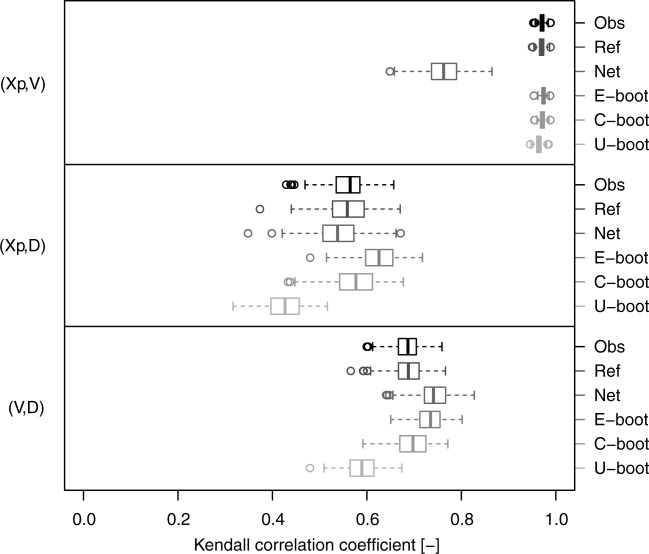
Box plots of pairwise Kendall correlation values referring to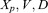 extracted from 200 time series simulated from a universal multifractal process with parameters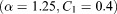. Six data sets are compared for each pair of variables: (1) “Obs” (observed) refers to the original time series; (2) “Ref” (reference) refers to resampled standardized ranks; (3) “Net” refers to the net properties; (4) “E-boot” refers to event-based bootstrap samples; (5) “C-boot” refers to conditional bootstrap samples; and (6) “U-boot” refers to unconditional bootstrap samples.

**Figure 17:**
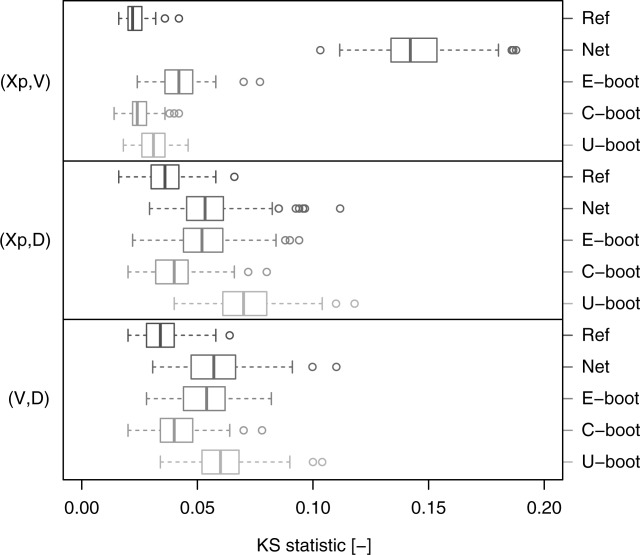
Box plots of the values of the KS statistic computed on the Kendall distributions referring to the pairwise empirical dependence structures. Each box plot describes the KS statistics obtained from 200 comparisons between the Kendall distributions corresponding to the observed samples and to the simulated samples (“E-boot,” “C-boot,” and “U-boot”). “Ref” provides a picture of the variability of the KS statistic under the null hypothesis that the empirical copula is preserved and represents the reference for the other box plots. “Net” refers to the comparison between the observed and net dependence structures.

**Figure 18:**
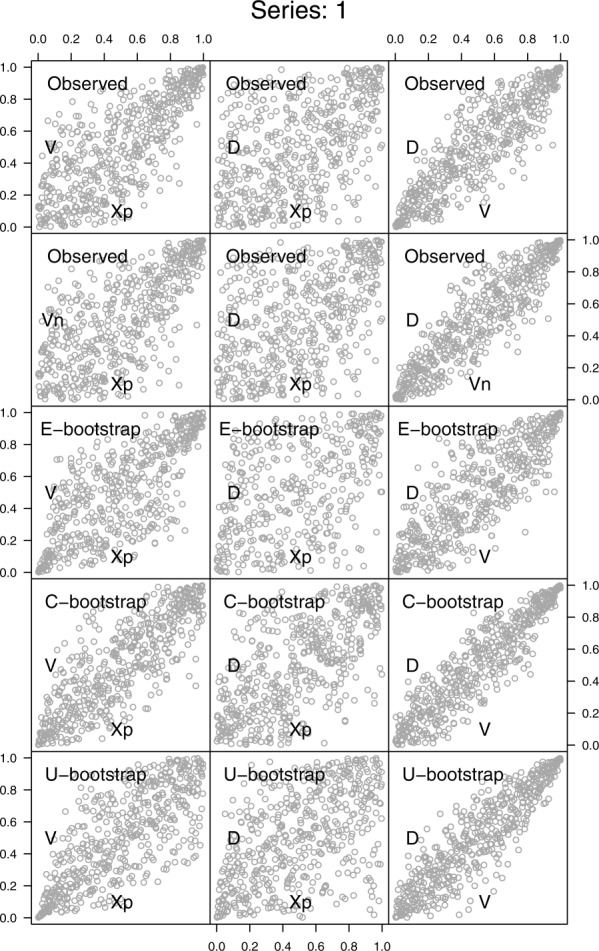
As in Figure 15 but for universal multifractal time series with parameters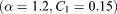 and 95th percentile threshold.

**Figure 19:**
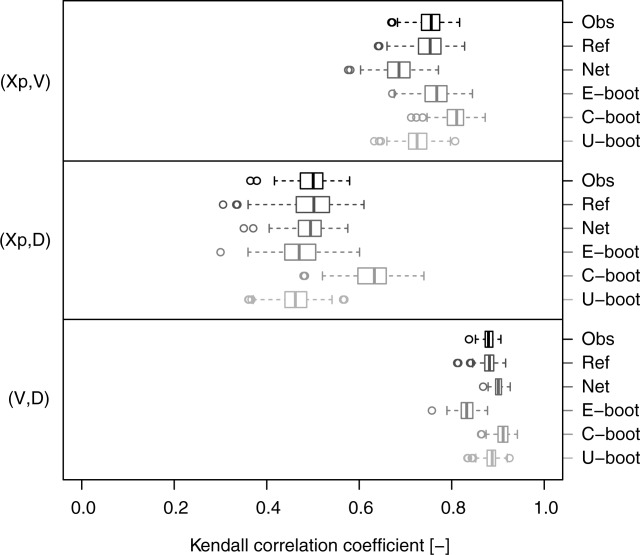
As in Figure 16 but for 200 universal multifractal time series with parameters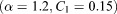.

**Figure 20:**
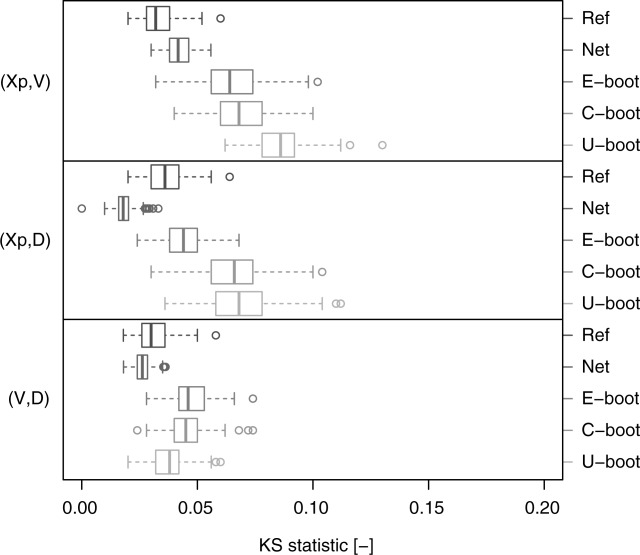
As in Figure 117 but for 200 universal multifractal time series with parameters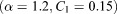.

[63] Analogous diagrams are provided for the pseudoevents corresponding to the second parameterization. In this case, the parameter set up returns events with no unitary duration. The scatterplots in Figure 18 highlight this feature which keeps almost unchanged the relationship between *X_p_* and *D*. This property is reflected in the box plots that summarize the Kendall correlation and the KS statistic (Figures 19 and 20). The bootstrap algorithms are not able to reproduce accurately the observed relationships. However, as already stressed, the hypothesized mechanisms produce Kendall correlation values and empirical copulas which broadly capture the main features of the observed dependence structures. In other words, they are able to explain a significant part of the observed behavior, while recognizing that residual unexplained characteristics must be ascribed to the intrinsic nature of the underlying processes (rainfall, runoff, or multifractal processes).

## 7. Conclusions

[64] In this study, we have investigated the nature of the dependence structures that link the key properties of the event hyetographs and hydrographs, namely, peak, volume, duration, and average intensity. Unlike previous studies that focused on the modeling of these relationships, we tried to shed light on the generating mechanism in order to understand the actual shape of the pairwise dependence structures. We analyzed a large data set of event hyetographs and hydrographs extracted from 282 daily rainfall series from central eastern Europe, three 5 min rainfall series from central Italy, and 80 daily streamflow series from the continental United States corresponding to heterogeneous climate, physical, management, and regulation conditions. In addition, 200 simulated universal multifractal time series has been considered. These data sets allowed highlighting the presence of some general properties of the pairwise dependence structures of 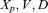, and *I* that were used as a guide to set up a pool of bootstrap algorithms devised to study the origin of these properties. The results of this study can be summarized as follows:

[65] 1. The pairwise relationships between 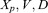, and *I* can be substantially explained as the result of summing independent random variables over random durations. This result implies that the internal structure of the hyetographs and hydrographs (i.e., the internal time dependence) plays only a marginal role, meaning that the underlying rainfall and runoff processes are only marginally responsible for the relationships between 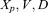, and *I*, which are instead more intrinsically related to the properties of clusters of independent random variables.

[66] 2. The previous result also implies that dependence structures of 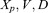, and *I* have a common nature which can be only approximately described by the copulas commonly applied in the literature. Therefore, as the use of different copulas for describing a unique mechanism could not be fully justified, it follows that an appropriate model must be developed to describe the above mentioned dependence structures, keeping in mind that their nature in not purely related to the physical variable under study. In this respect, it is worth noting that the boundary that characterizes the scatterplot of *X_p_* and *V* can be removed by analyzing the “net” variables [e.g., *Gyasi-Agyei and Melching*, 2012]; as these variables exhibit a more genuine purely stochastic behavior, the use of multivariate distributions seems to be more justified and easier.

[67] 3. When the copula methodology is used to perform a multivariate frequency analysis, it is worth distinguishing between inference and process understanding. According to the copula theory, marginals and dependence structures can be studied independently; however, our simulation exercise showed that from a physical or numerical point of view, the shape of the marginal distributions is strictly related to the shape of the dependence structures. Therefore, understanding the generating mechanisms is fundamental to interpretation of the true nature of the dependence structures and choice of appropriate analysis method. Confusing stochastic relationships with numerical or geometrical relationships can lead to misleading conclusions. Thus, our findings further stress the importance of establishing a stronger link between the interpretation of the processes that generate the design variables and the statistical techniques used to summarize them.

[68] 4. The simulation of time series following a theoretical universal multifractal process highlights that the algorithms devised for observed hyetographs and hydrographs are able to explain a relevant part of the dependence structures of the pseudoevents extracted from those signals. Even though the agreement is not perfect (as expected), the analyses confirm that the numerical summation over random durations plays a key role in the resulting dependence structures.

[69] 5. The rationale of the bootstrap simulation described in this study can be used to build algorithms useful to investigate in more depth the properties of objects such as hyetographs and hydrographs. They can also be applied as a base for parametric and nonparametric simulation strategies of the required dependence structure by using univariate concepts.

## Appendix A: R Codes for the MC Algorithm

[70] We report the R [*R Development Core Team*, 2011] implementation of the MC algorithm used to simulate the data shown in Figure 13.

[71] Case (1)

[72] set.seed(666)

[73] d <- ceiling(rexp(5000, 0.09))

[74] v <- numeric()xp <- numeric()

[75] for (i in 1:5000) {

[76] xi <- rweibull(d[i], 0.3, 2)

[77] v[i] <- sum(xi)

[78] xp[i] <- max(xi)}

[79] Case (2)

[80] set.seed(666)

[81] d <- ceiling(rexp(5000, 0.09))

[82] v <- numeric()

[83] xp <- numeric()

[84] for (i in 1:5000) {

[85] u <- runif(1)

[86] if (u > 0.6) xi <- rweibull(d[i], 0.9, 2) + 10

[87] else xi <- rweibull(d[i], 1.0, 2)

[88] v[i] <- sum(xi)

[89] xp[i] <- max(xi)

[90] }

[91] Case (3)

[92] set.seed(666)

[93] d <- ceiling(c(rexp(700 * 5, 0.009), rexp(300 * 5, 0.09) + 200))

[94] v <- numeric()

[95] xp <- numeric()

[96] for (i in 1:5000) {

[97] q <- rweibull(d[i], 0.3, 2)

[98] v[i] <- sum(q)

[99] xp[i] <- max(q)

[100] }
