# Supplementary material for: The intrinsic dependence structure of peak, volume, duration, and average intensity of hyetographs and hydrographs
Source: Water Resour Res. 2013 Jun 17;49(6):3423–42. doi: 10.1002/wrcr.20221 (PMC4303924; doi:10.1002/wrcr.20221)
Supplement: Supplementary file 1 — Supplementary Figures [file wrcr0049-3423-sd1.pdf]

Station: Castel Cellesi (DJF)

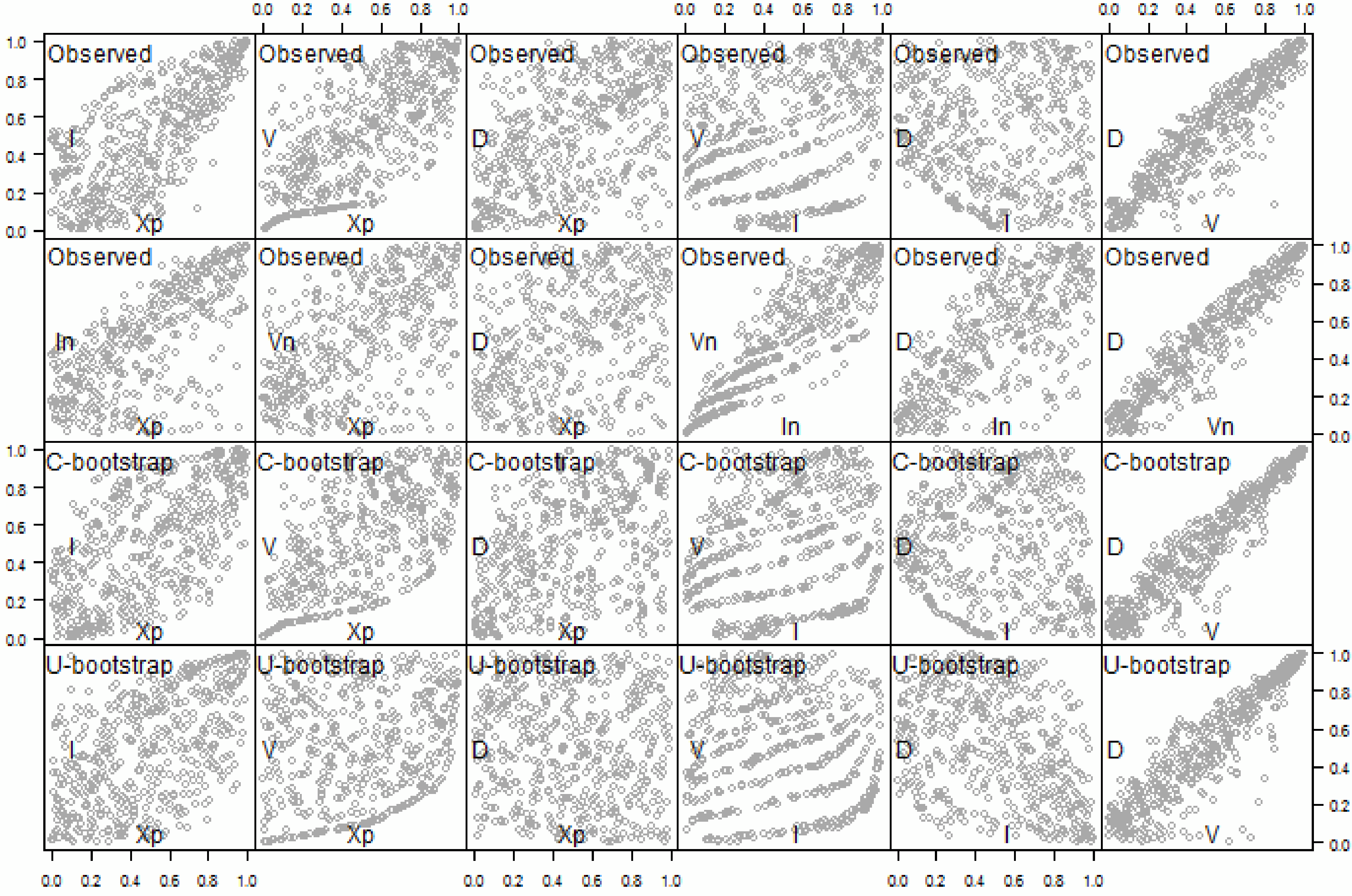

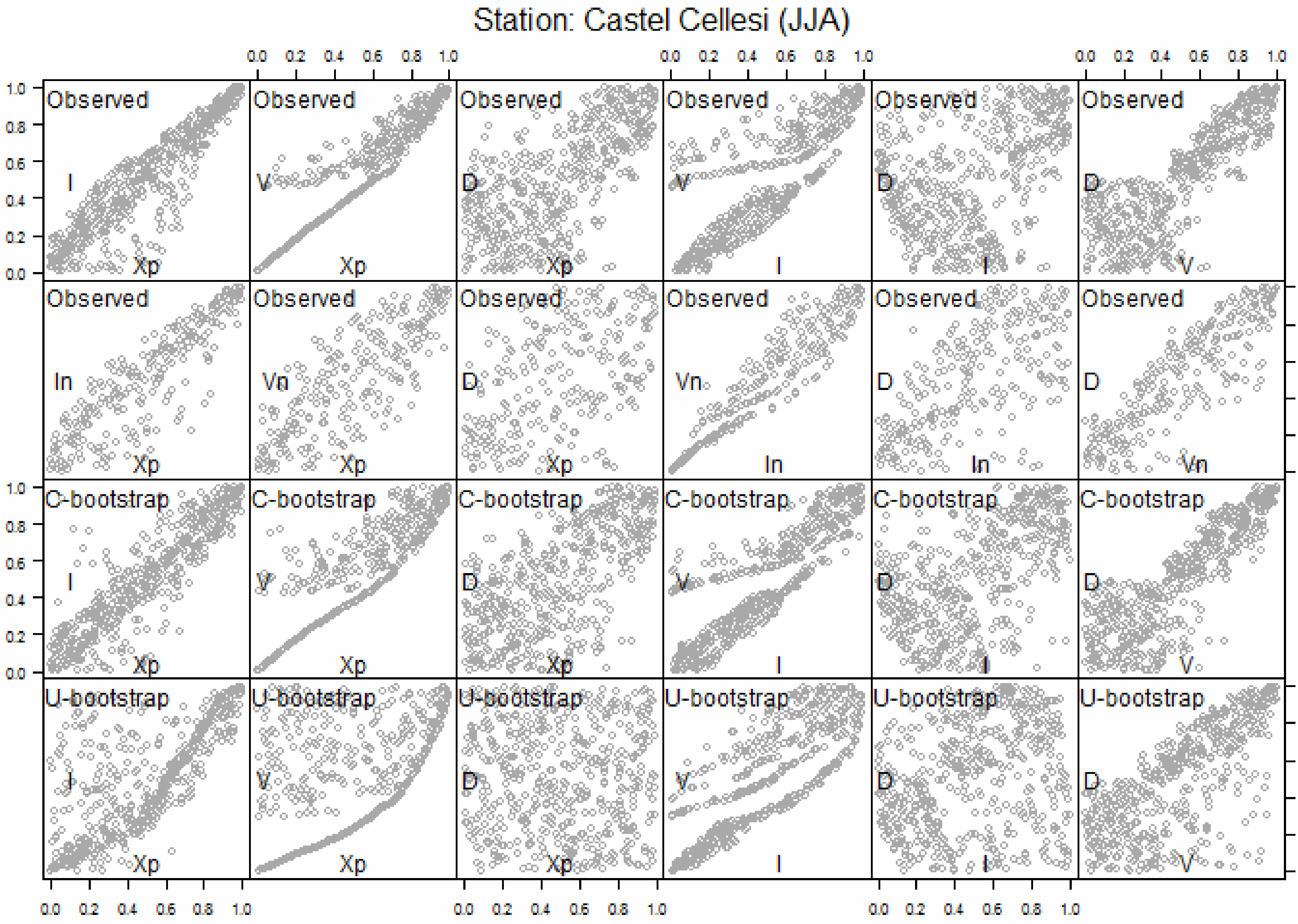
Station: Castel Cellesi (JJA)
Observed
C-bootstrap
U-bootstrap
Xp
I
V
D
In
Vn
0.0
0.2
0.4
0.6
0.8
1.0

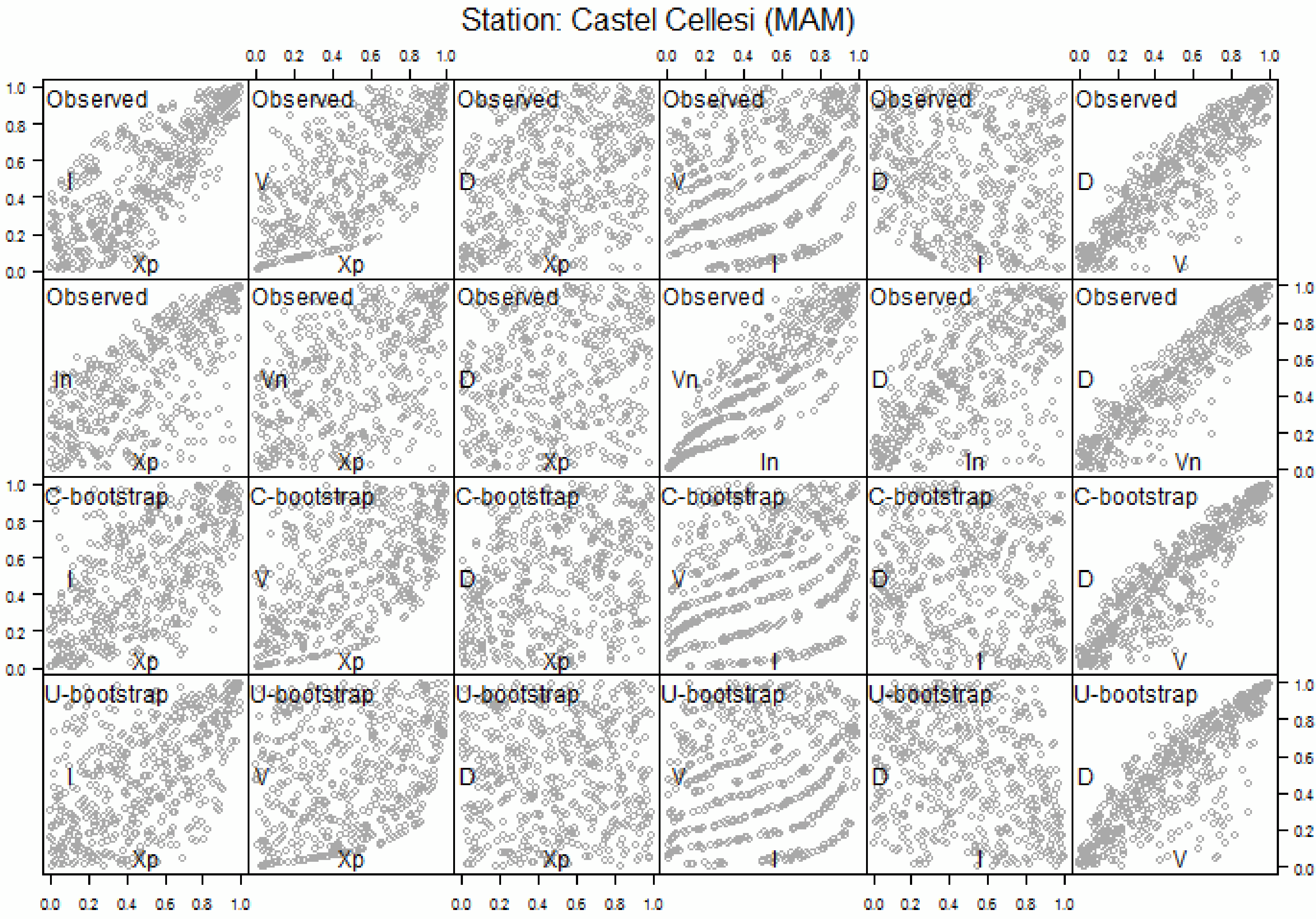
Station: Castel Cellesi (MAM)
Observed
I
Xp
Observed
V
Xp
Observed
D
Xp
Observed
V
I
Observed
D
I
Observed
D
V
Observed
In
Xp
Observed
Vn
Xp
Observed
D
Xp
Observed
Vn
In
Observed
D
In
Observed
D
Vn
C-bootstrap
I
Xp
C-bootstrap
V
Xp
C-bootstrap
D
Xp
C-bootstrap
V
I
C-bootstrap
D
I
C-bootstrap
D
V
U-bootstrap
I
Xp
U-bootstrap
V
Xp
U-bootstrap
D
Xp
U-bootstrap
V
I
U-bootstrap
D
I
U-bootstrap
D
V
0.0
0.2
0.4
0.6
0.8
1.0

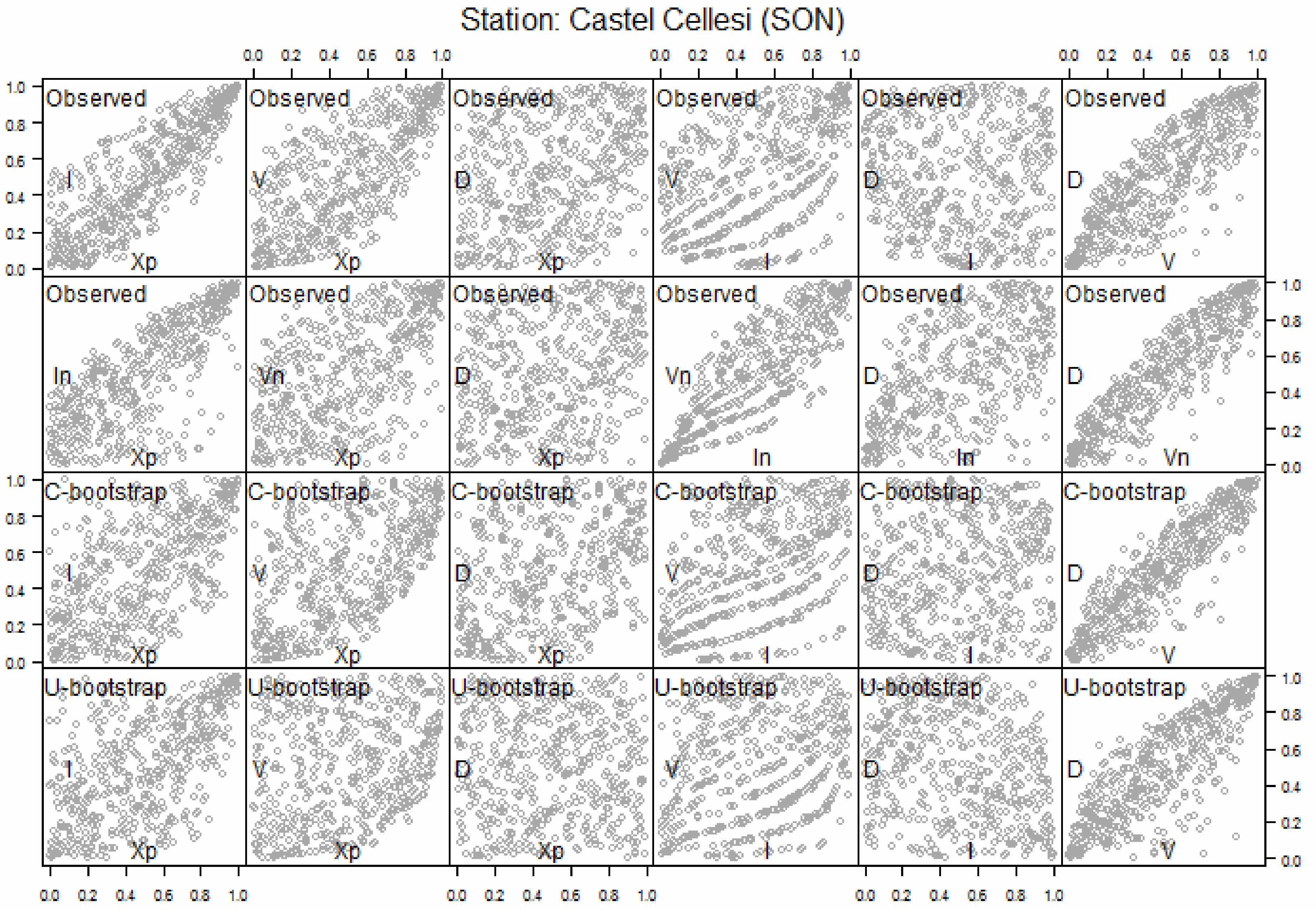
Station: Castel Cellesi (SON)
Observed
C-bootstrap
U-bootstrap
Xp
I
V
D
In
Vn
0.0
0.2
0.4
0.6
0.8
1.0

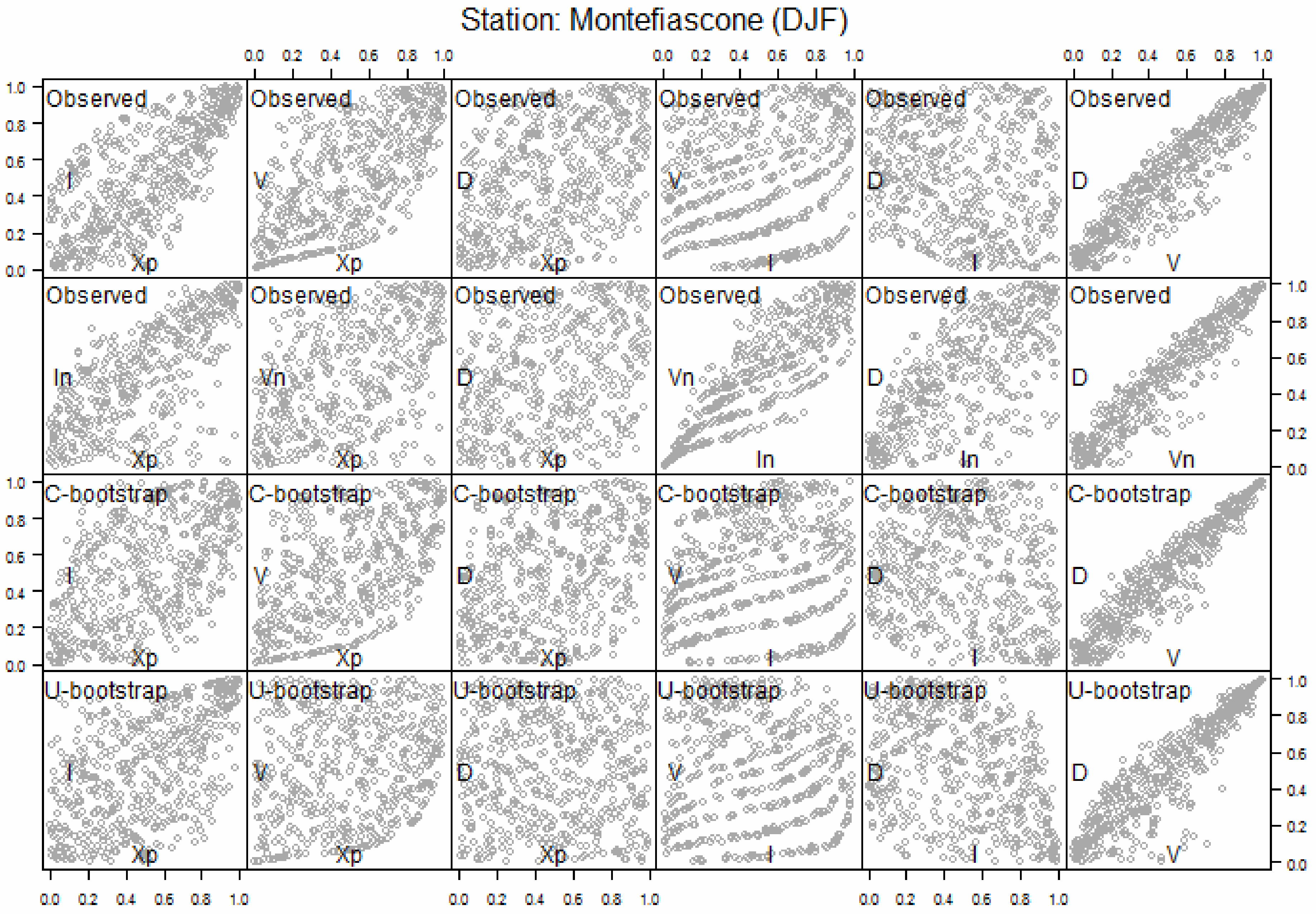
Station: Montefiascone (DJF)
Observed
C-bootstrap
U-bootstrap
Xp
I
V
D
In
Vn
0.0
0.2
0.4
0.6
0.8
1.0

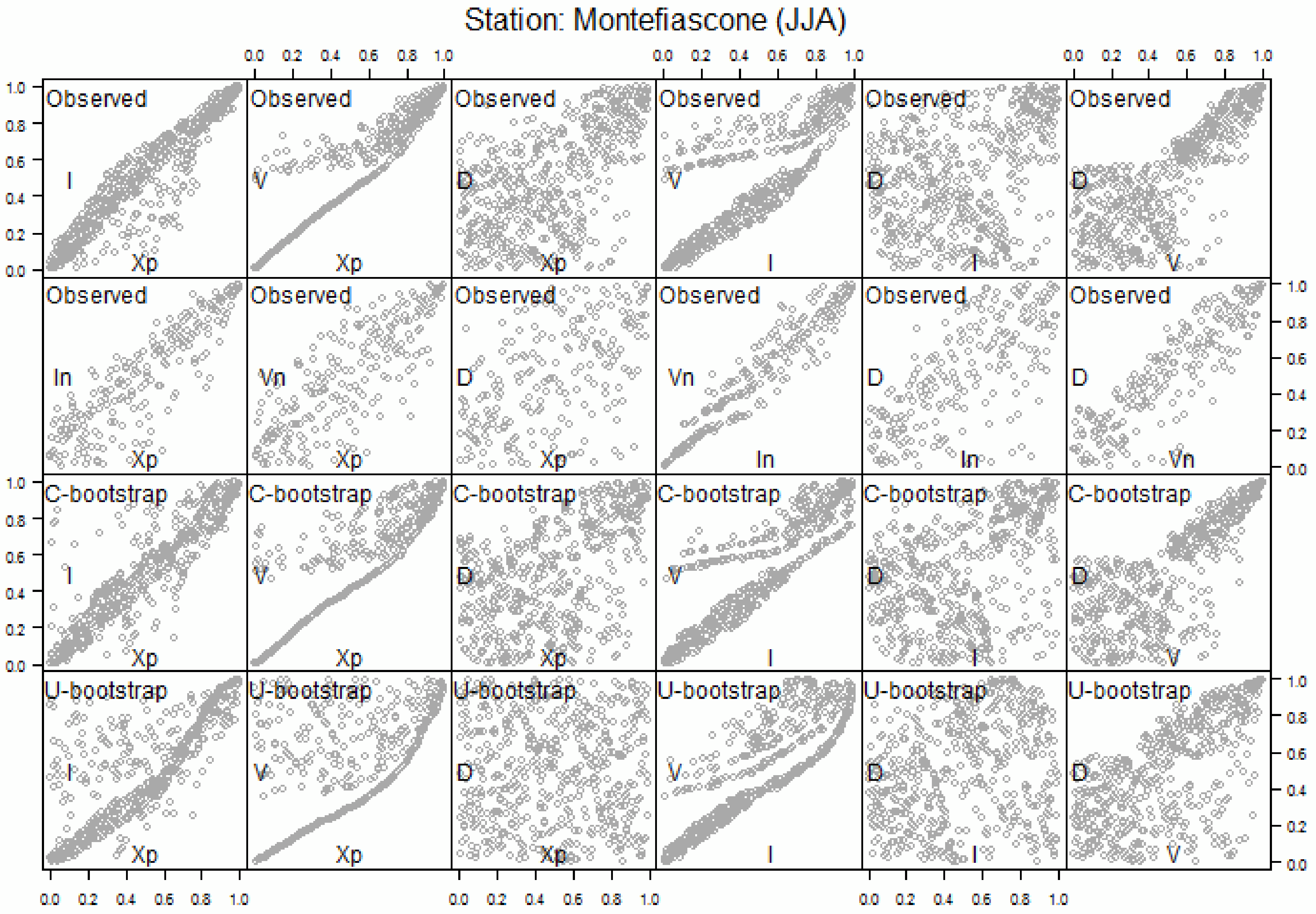
Station: Montefiascone (JJA)
Observed
C-bootstrap
U-bootstrap
Xp
I
V
D
In
Vn
0.0
0.2
0.4
0.6
0.8
1.0

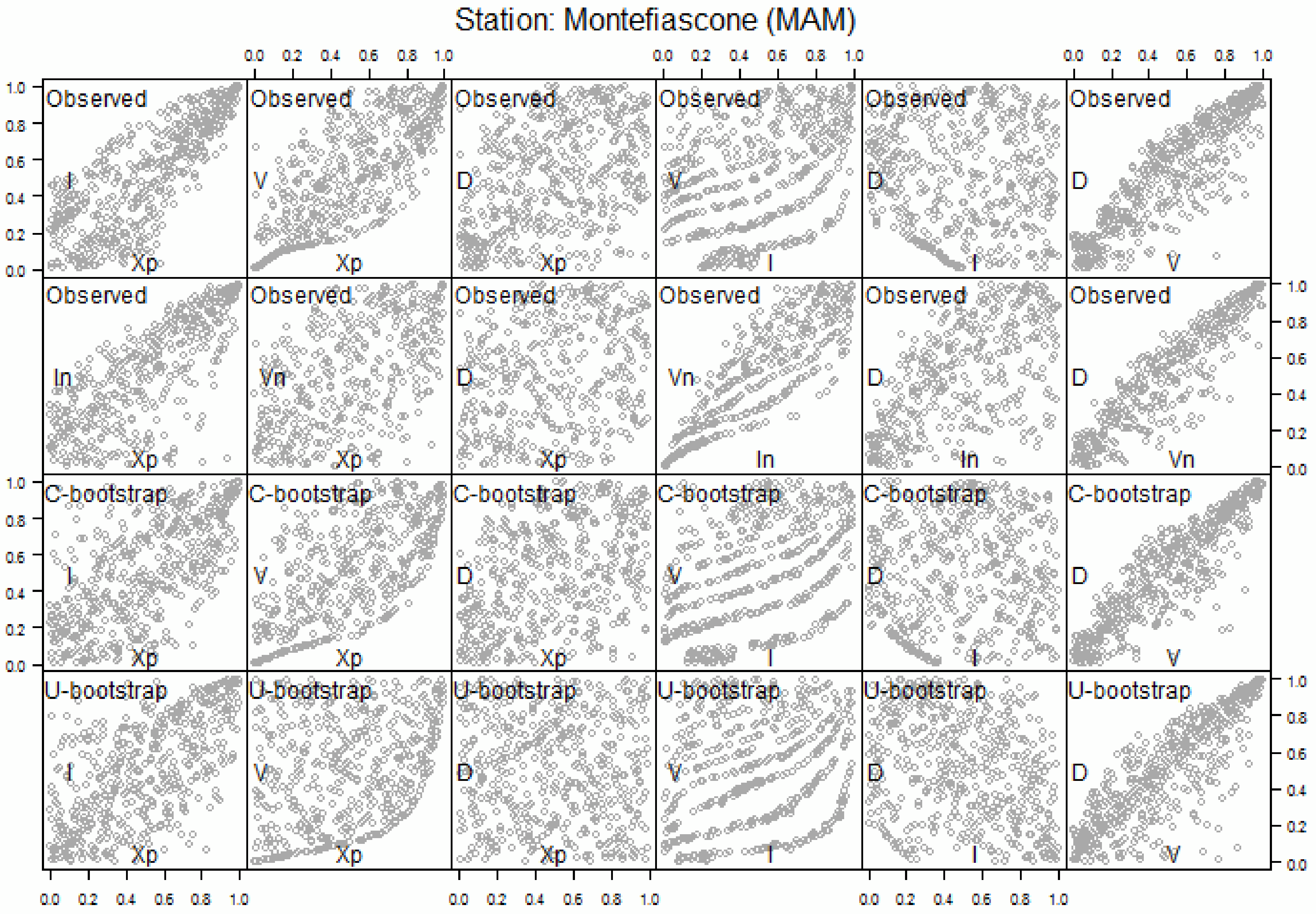
Station: Montefiascone (MAM)
Observed
C-bootstrap
U-bootstrap
Xp
I
V
D
In
Vn
0.0
0.2
0.4
0.6
0.8
1.0

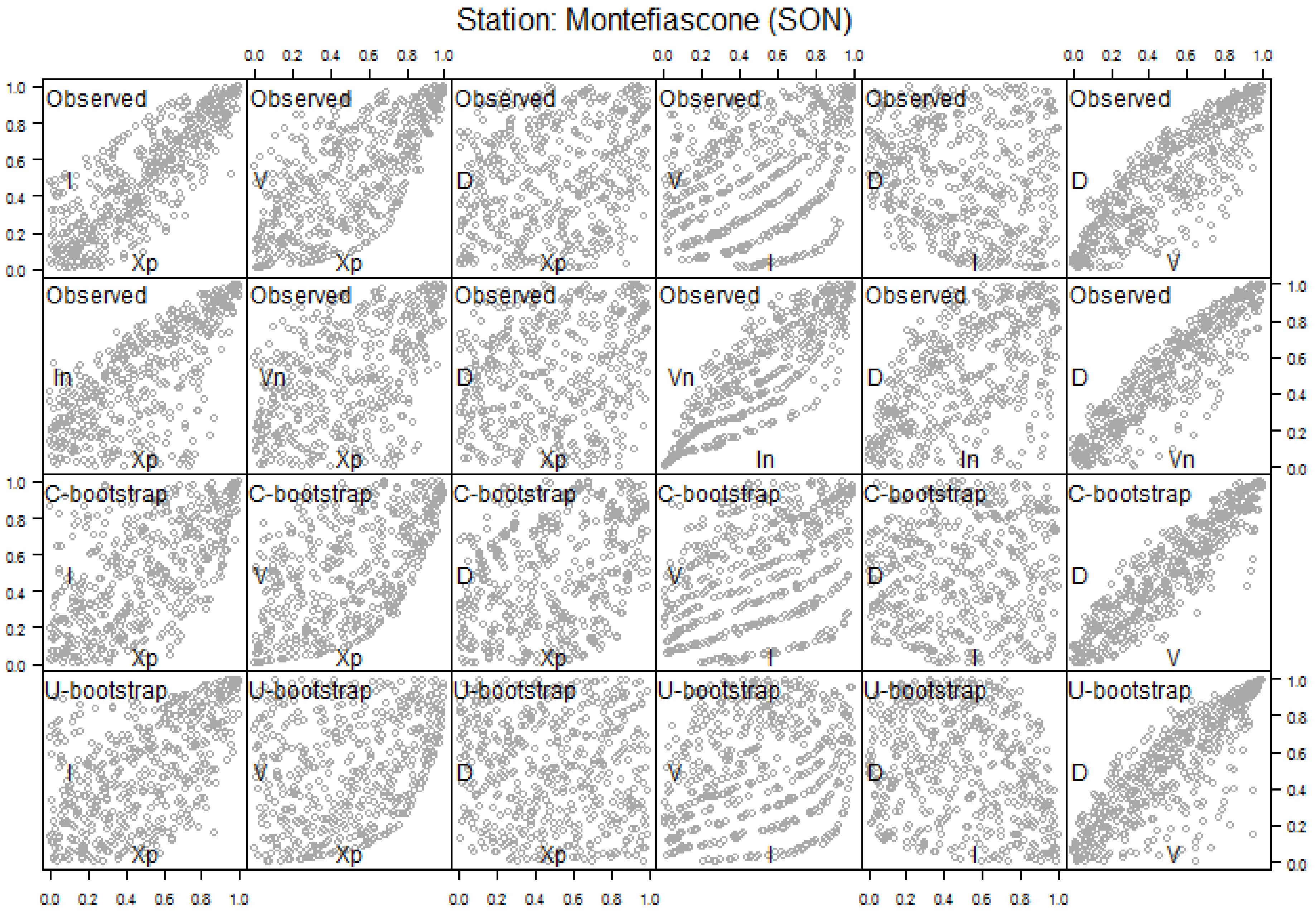
Station: Montefiascone (SON)
Observed
Observed
Observed
Observed
Observed
Observed
C-bootstrap
U-bootstrap
I
V
D
In
Vn
Xp
0.0
0.2
0.4
0.6
0.8
1.0

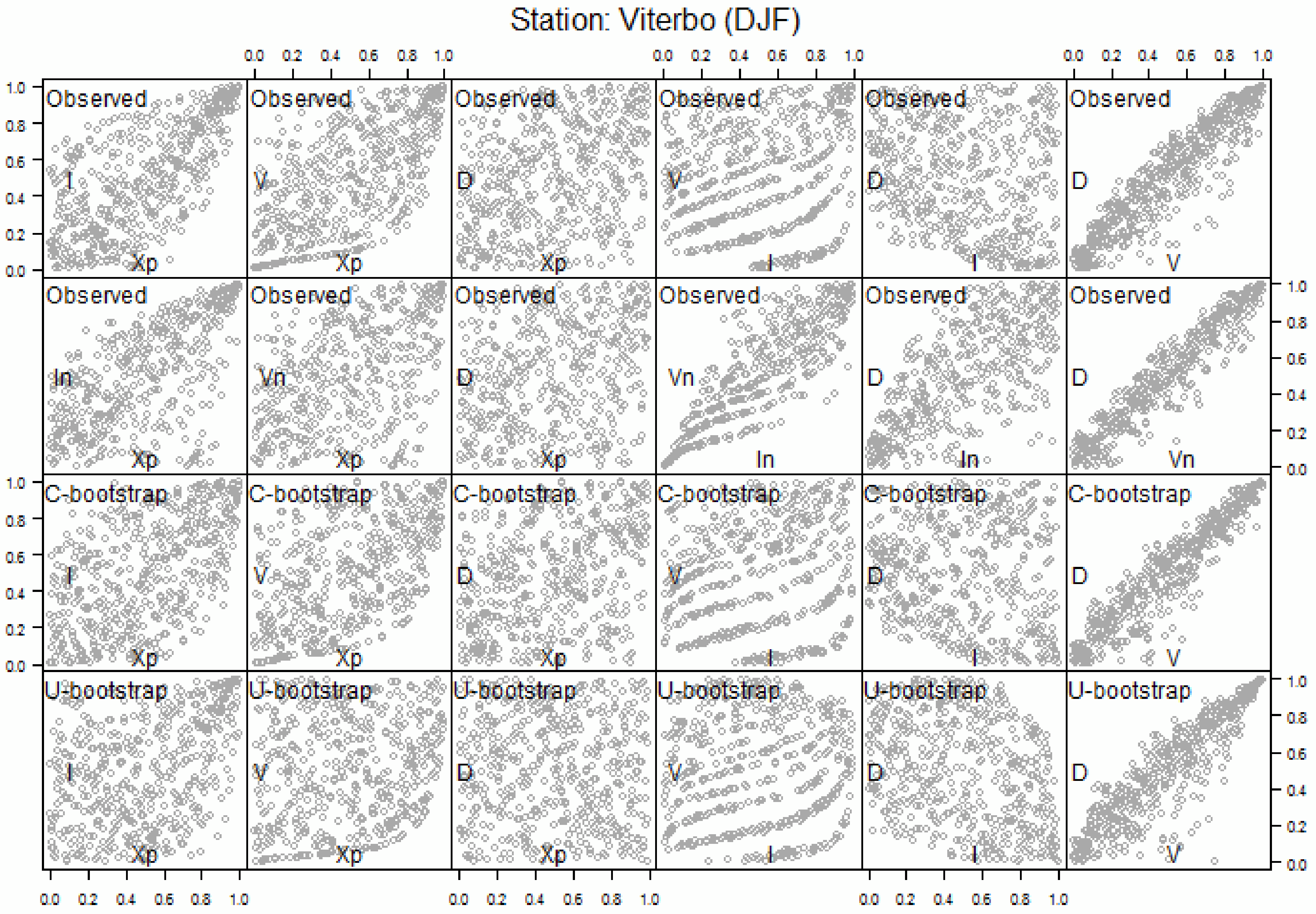

Station: Viterbo (DJF)
Observed
C-bootstrap
U-bootstrap
Xp
I
V
D
In
Vn
0.0
0.2
0.4
0.6
0.8
1.0

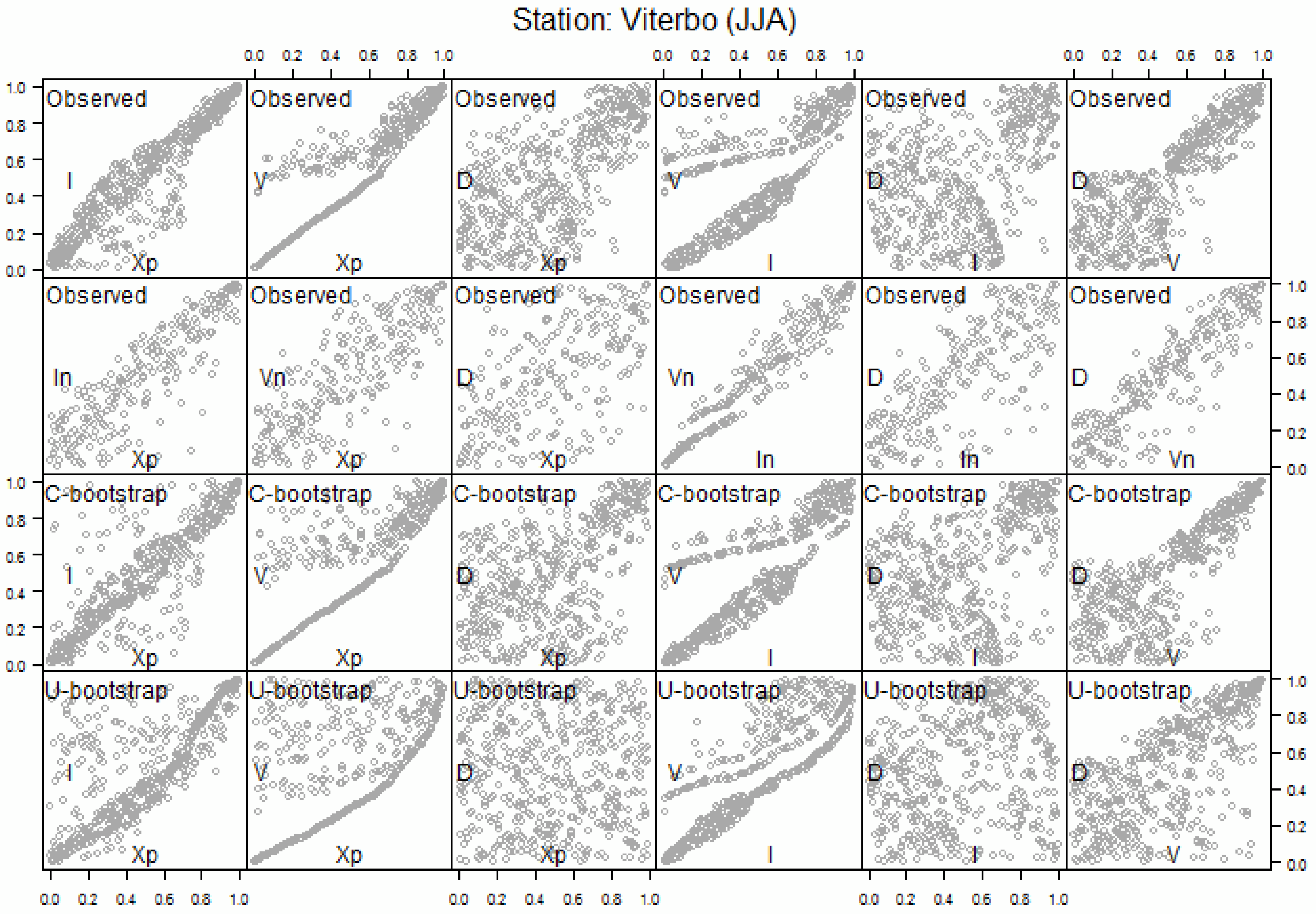
Station: Viterbo (JJA)
Observed
C-bootstrap
U-bootstrap
Xp
I
V
D
In
Vn
0.0
0.2
0.4
0.6
0.8
1.0

Station: Viterbo (MAM)

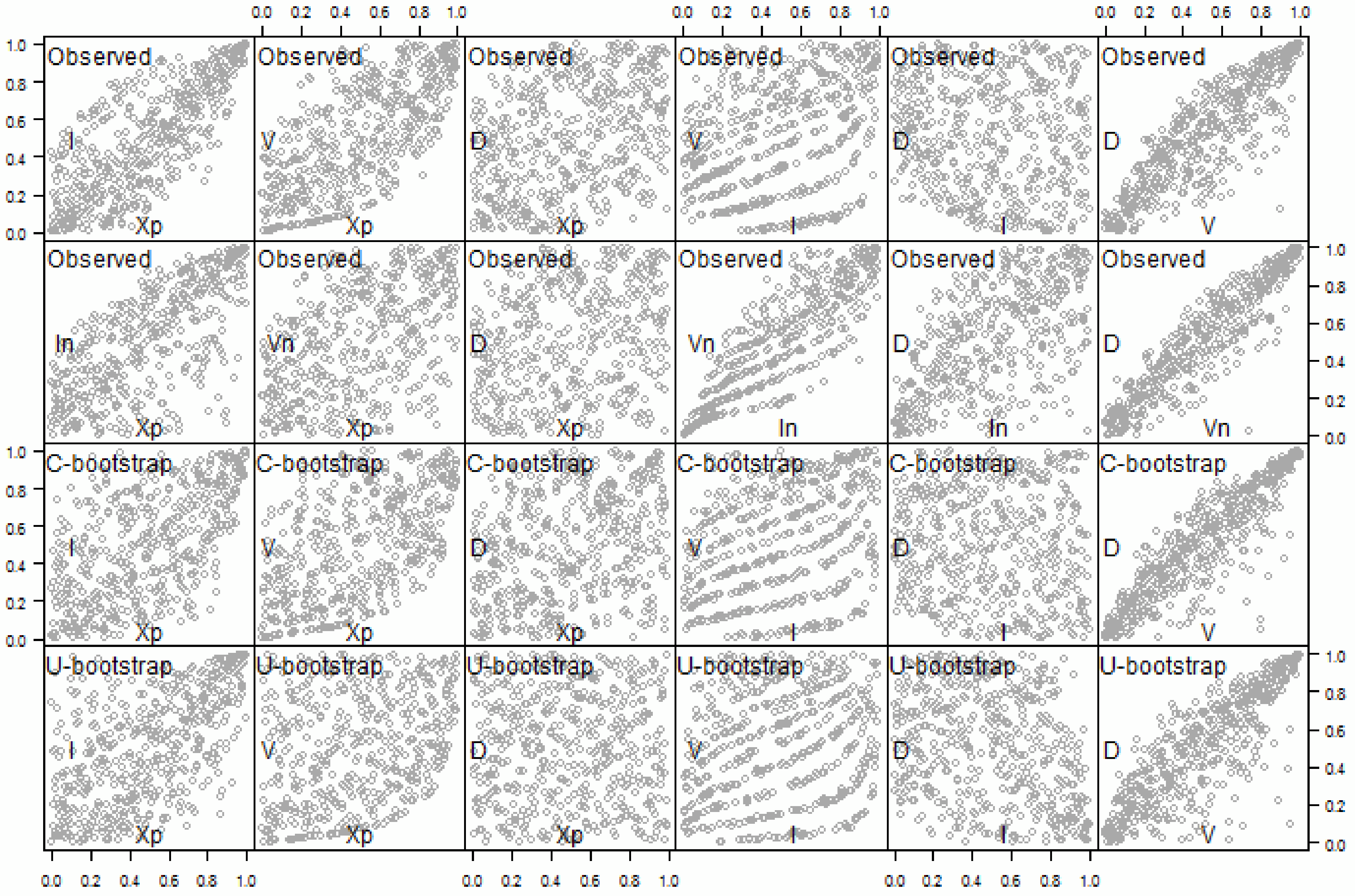

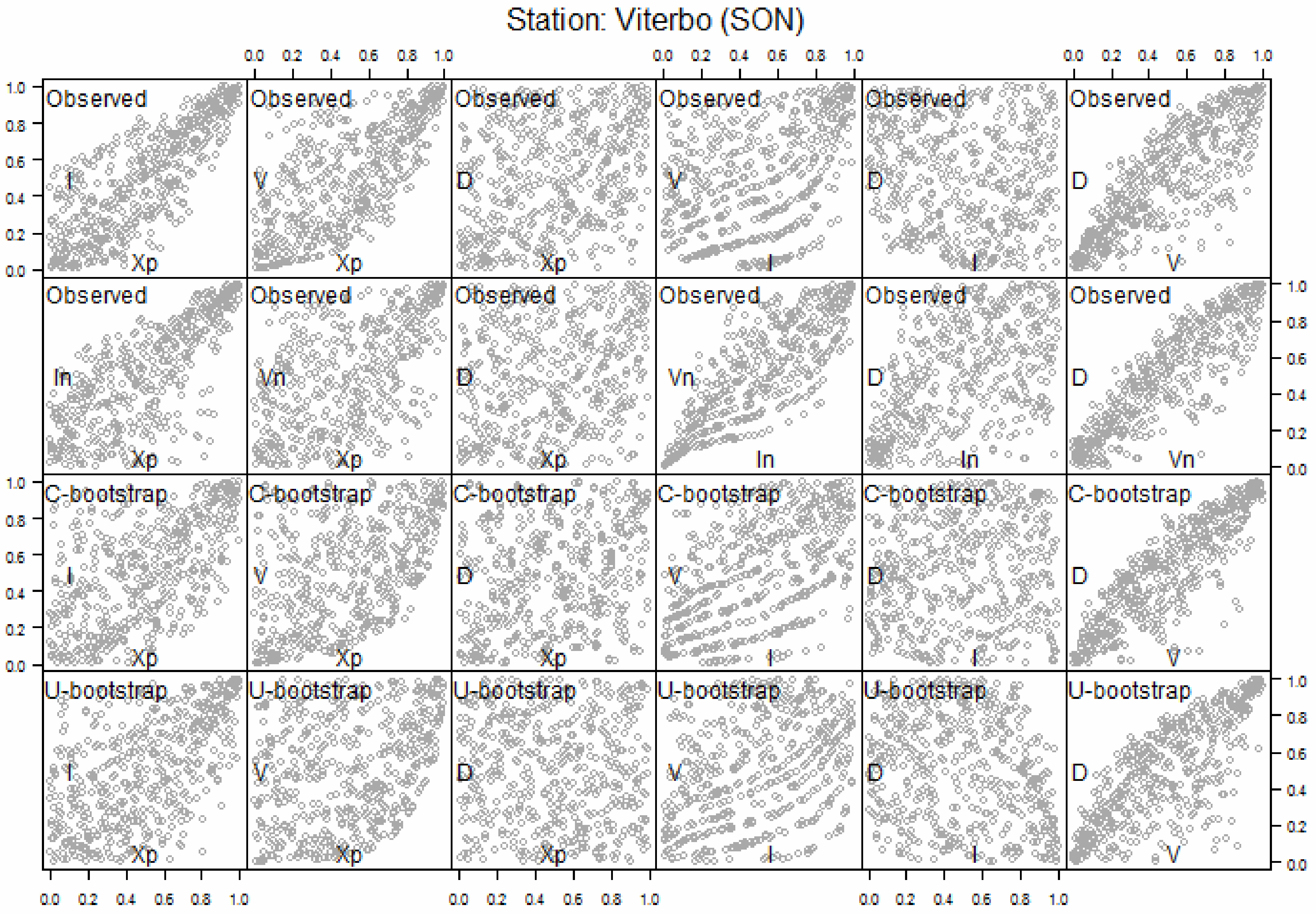

Station: Viterbo (SON)
Observed
C-bootstrap
U-bootstrap
Xp
I
V
D
In
Vn
0.0
0.2
0.4
0.6
0.8
1.0
