# Supplementary figures and images for: The intrinsic dependence structure of peak, volume, duration, and average intensity of hyetographs and hydrographs
Source: Water Resour Res. 2013 Jun 17;49(6):3423–42. doi: 10.1002/wrcr.20221 (PMC4303924; doi:10.1002/wrcr.20221)

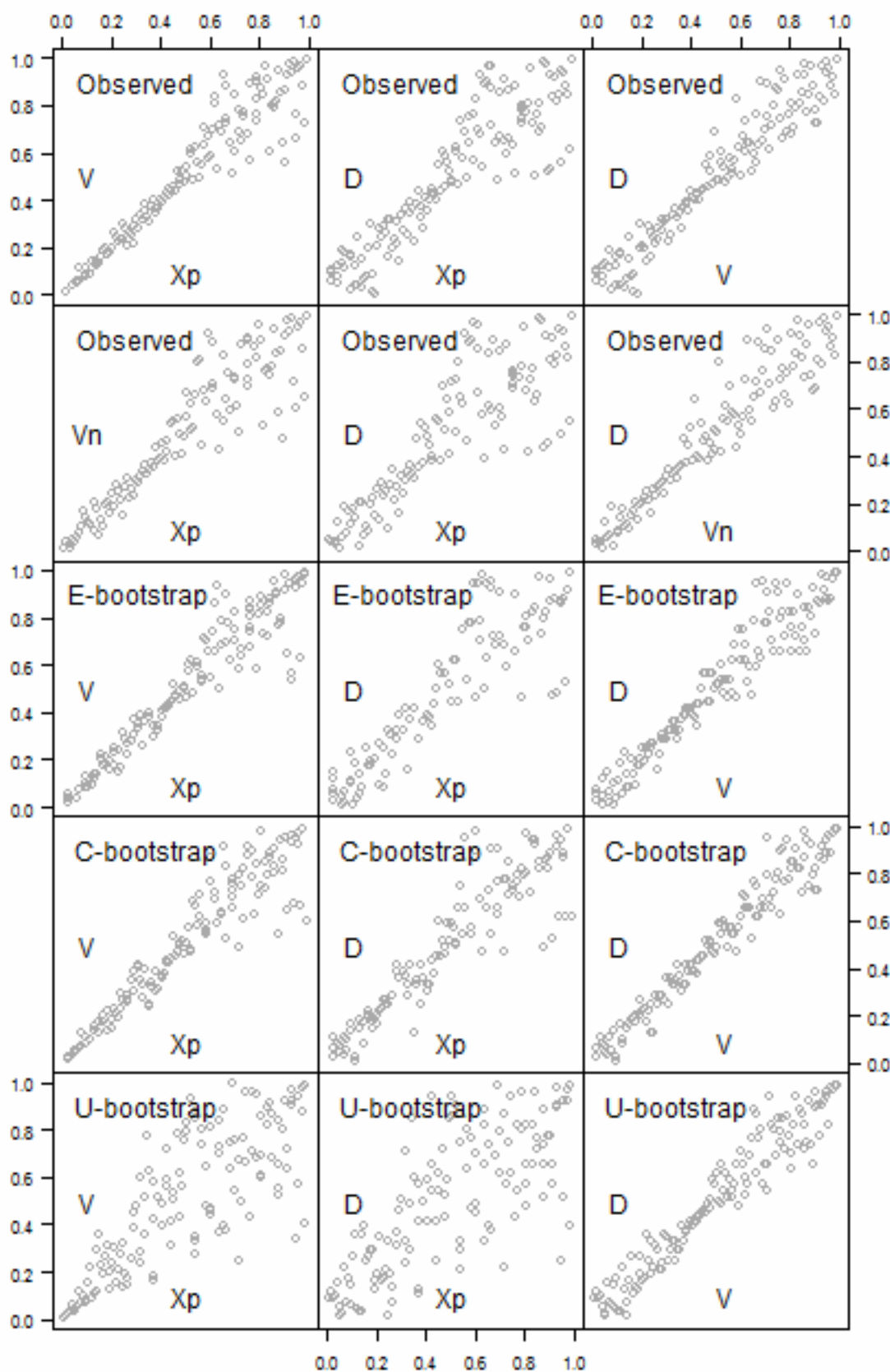

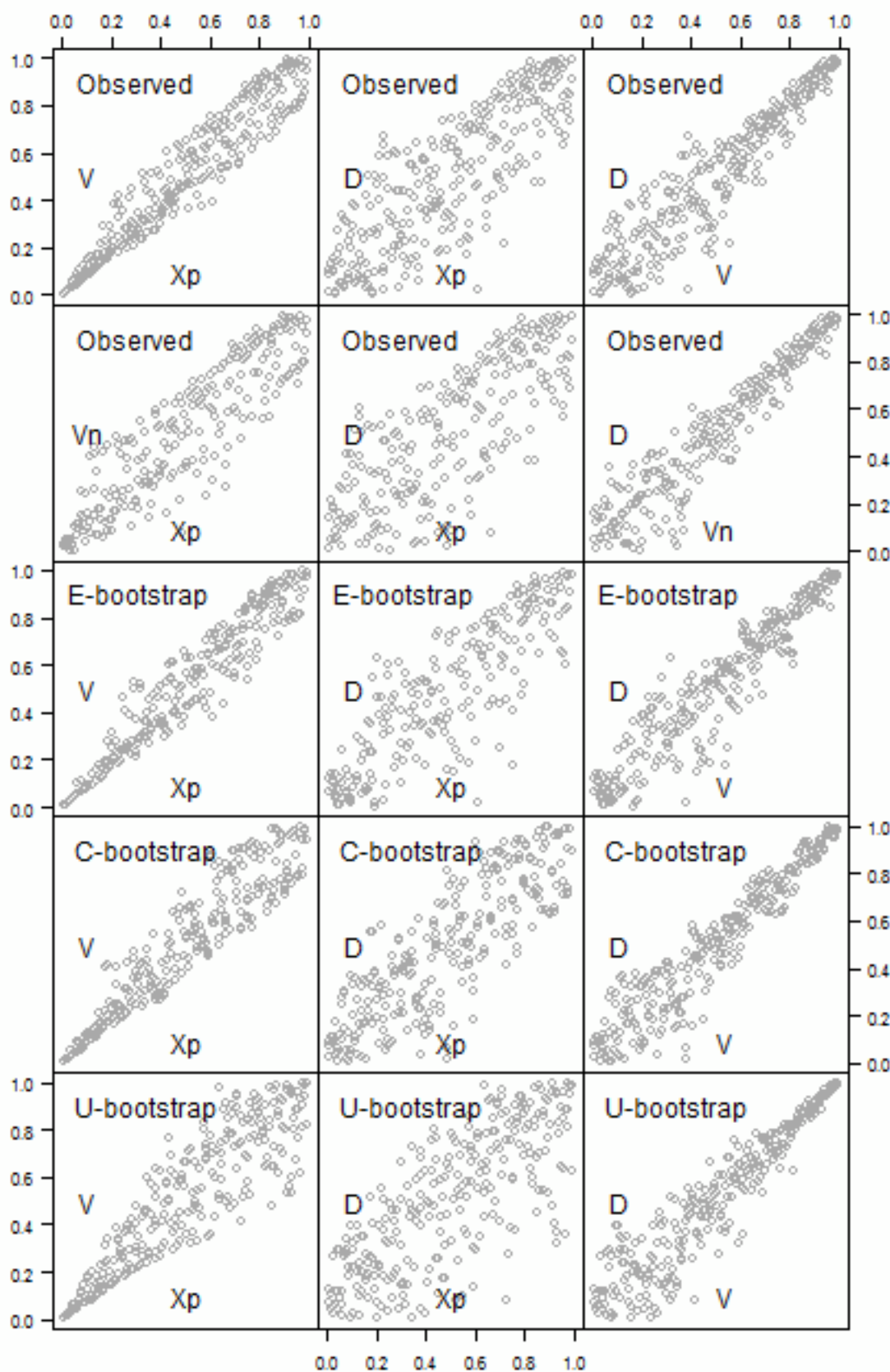

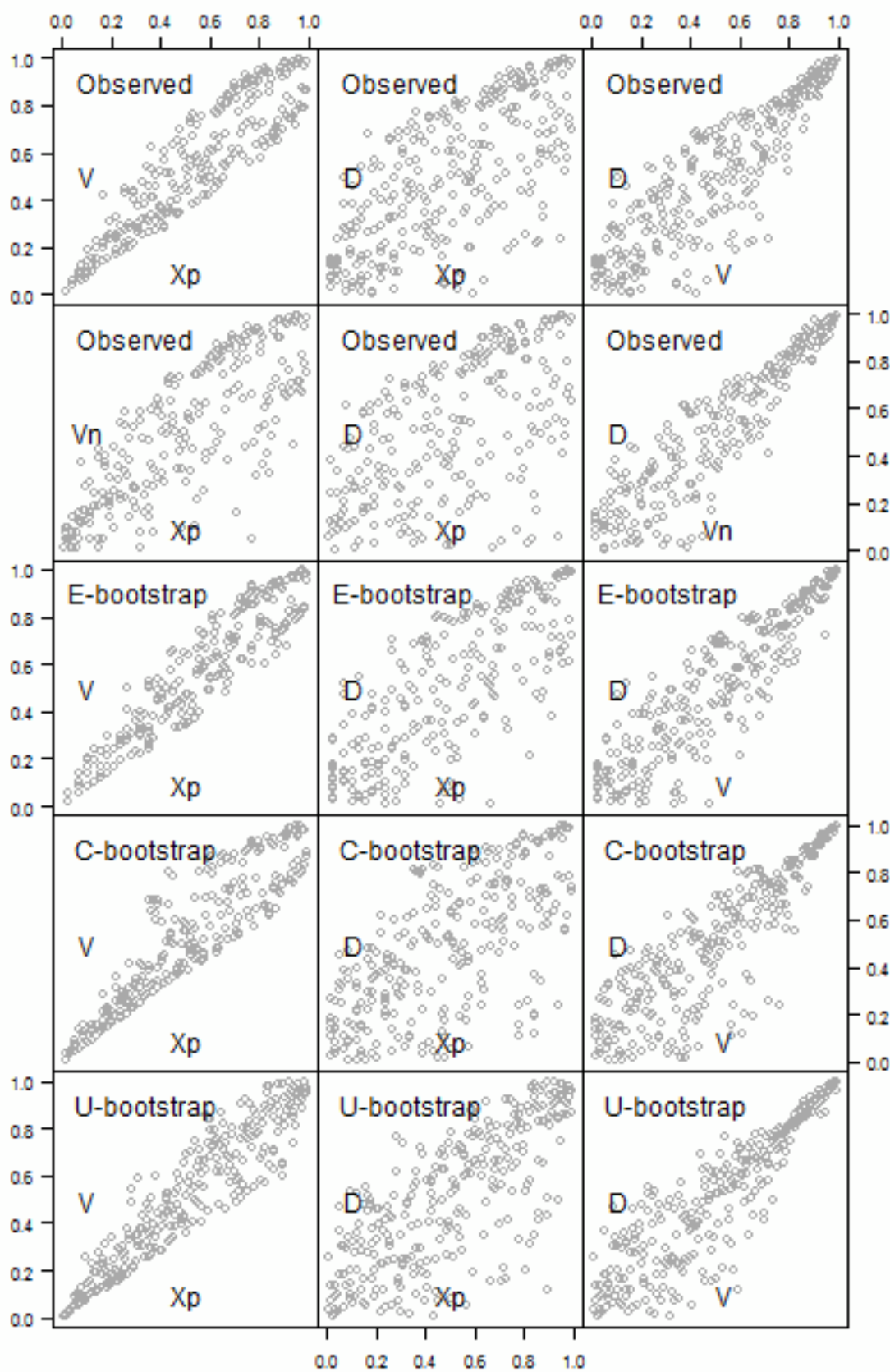

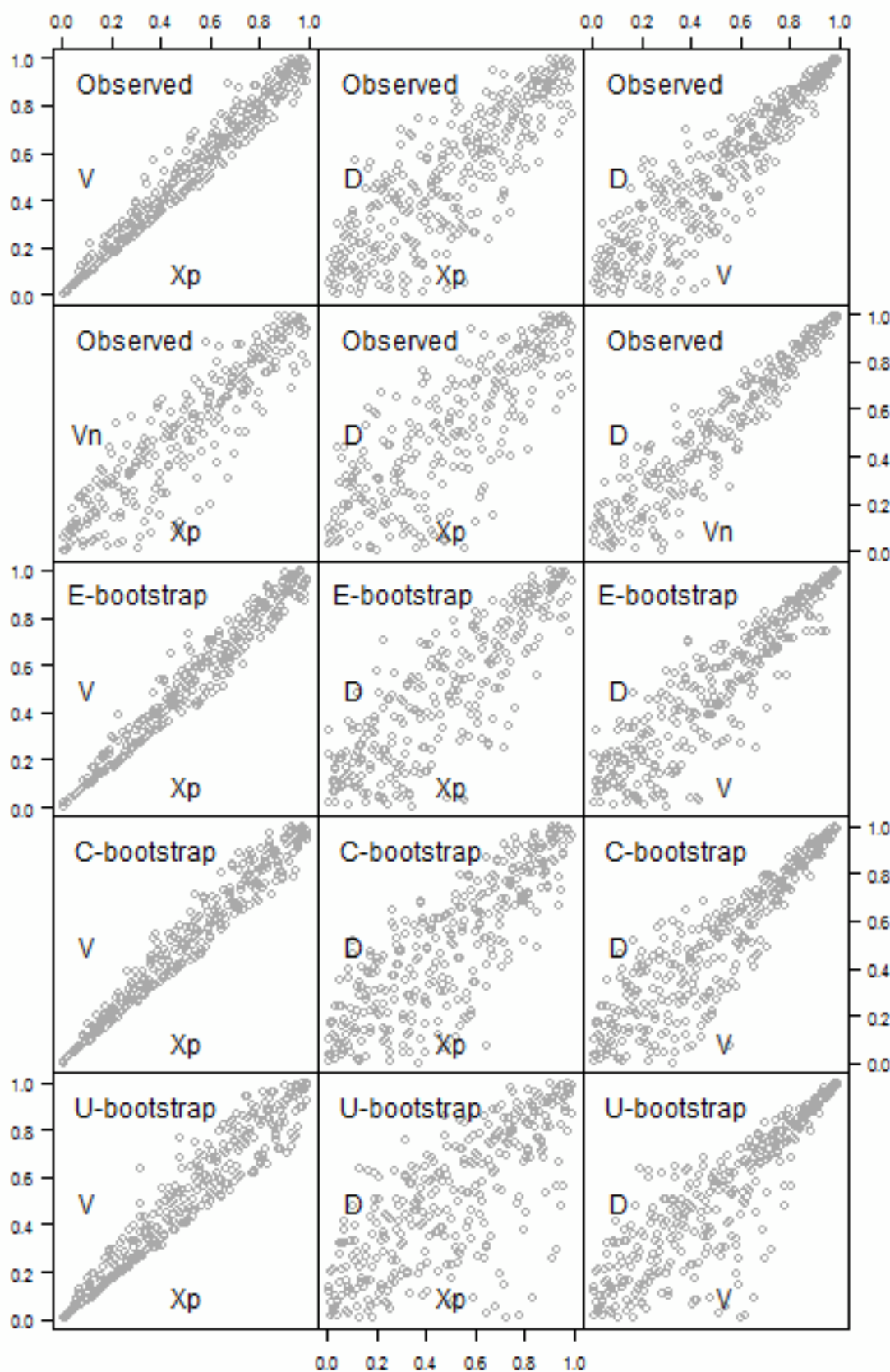

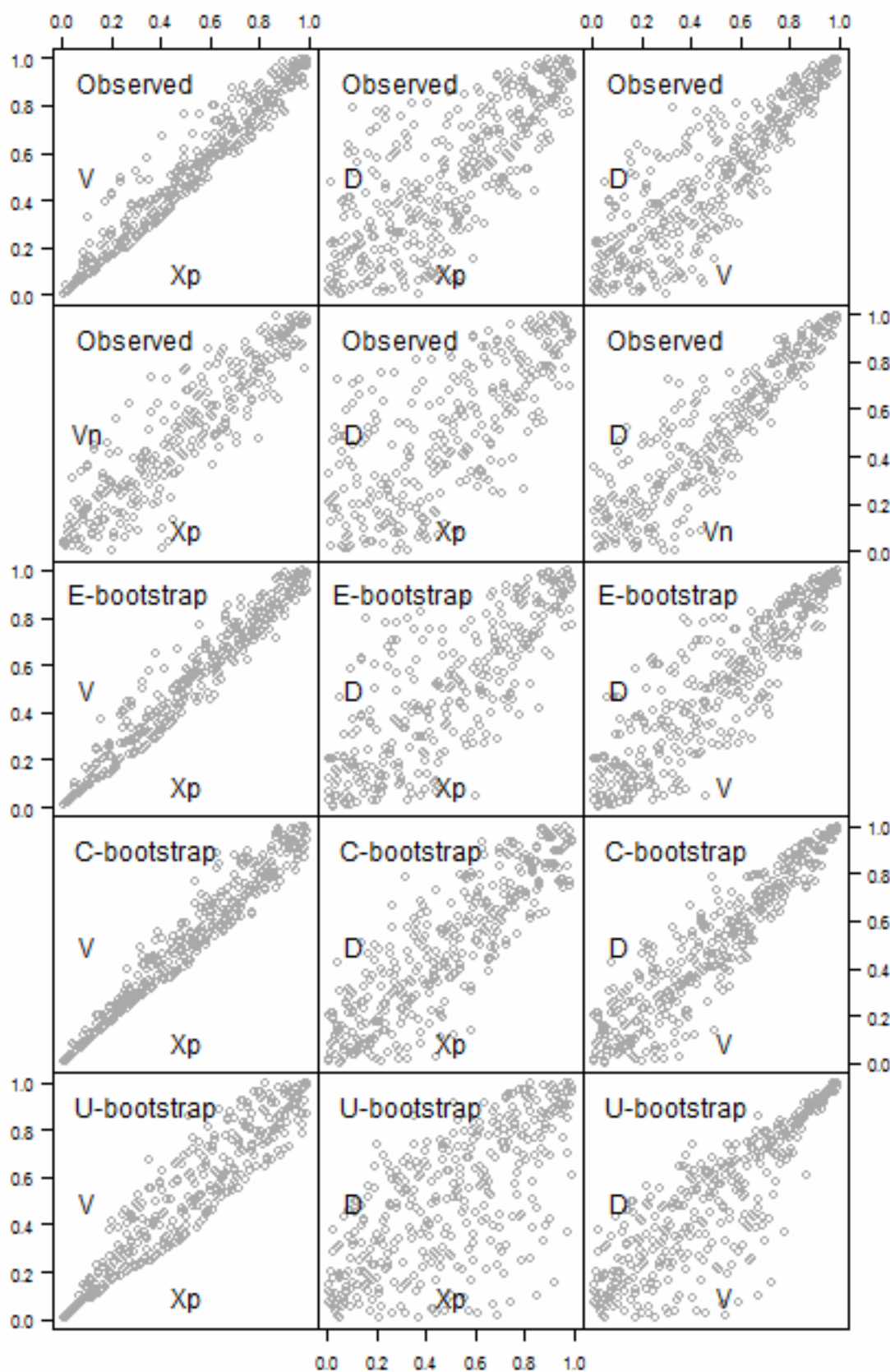

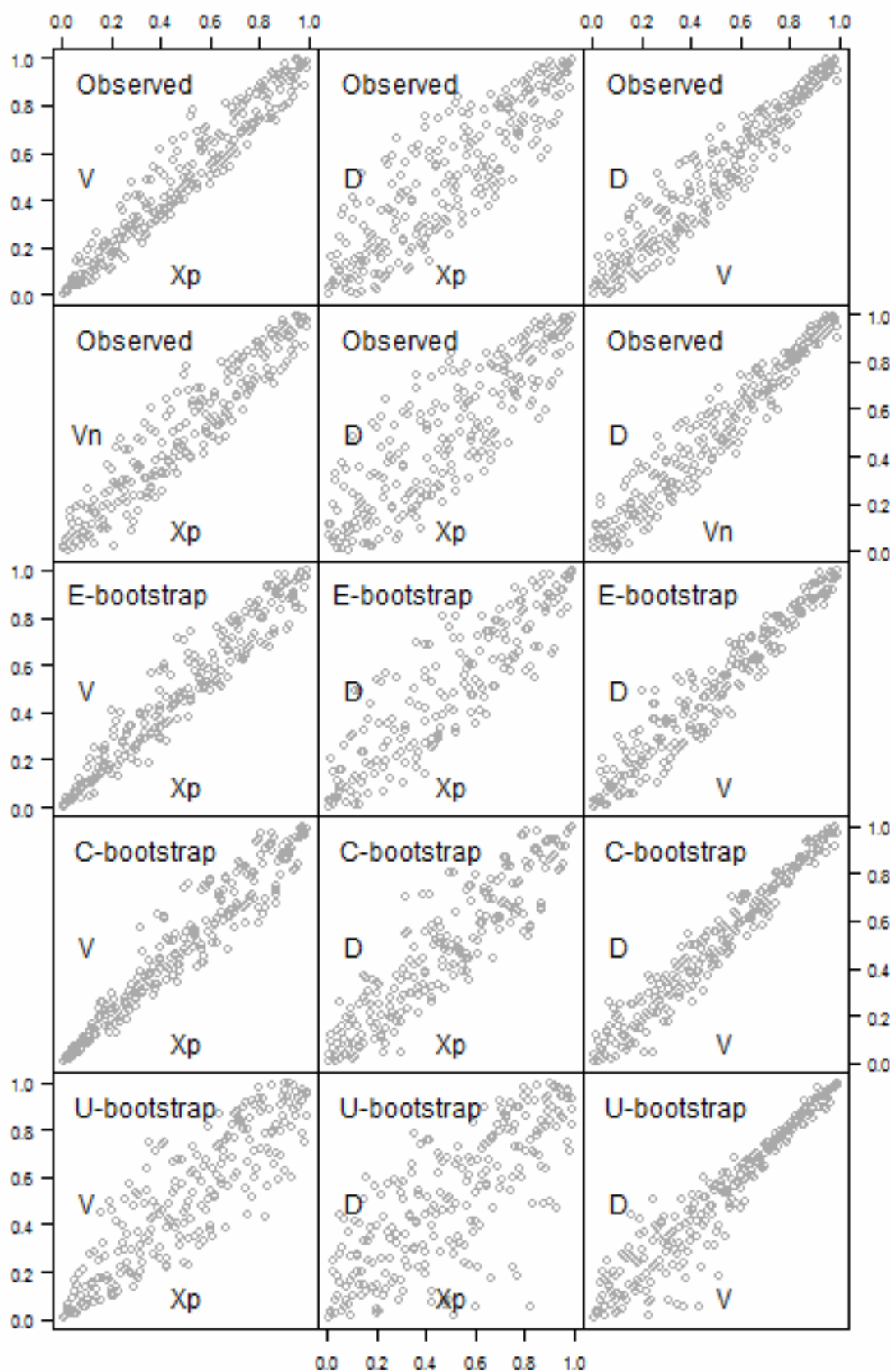

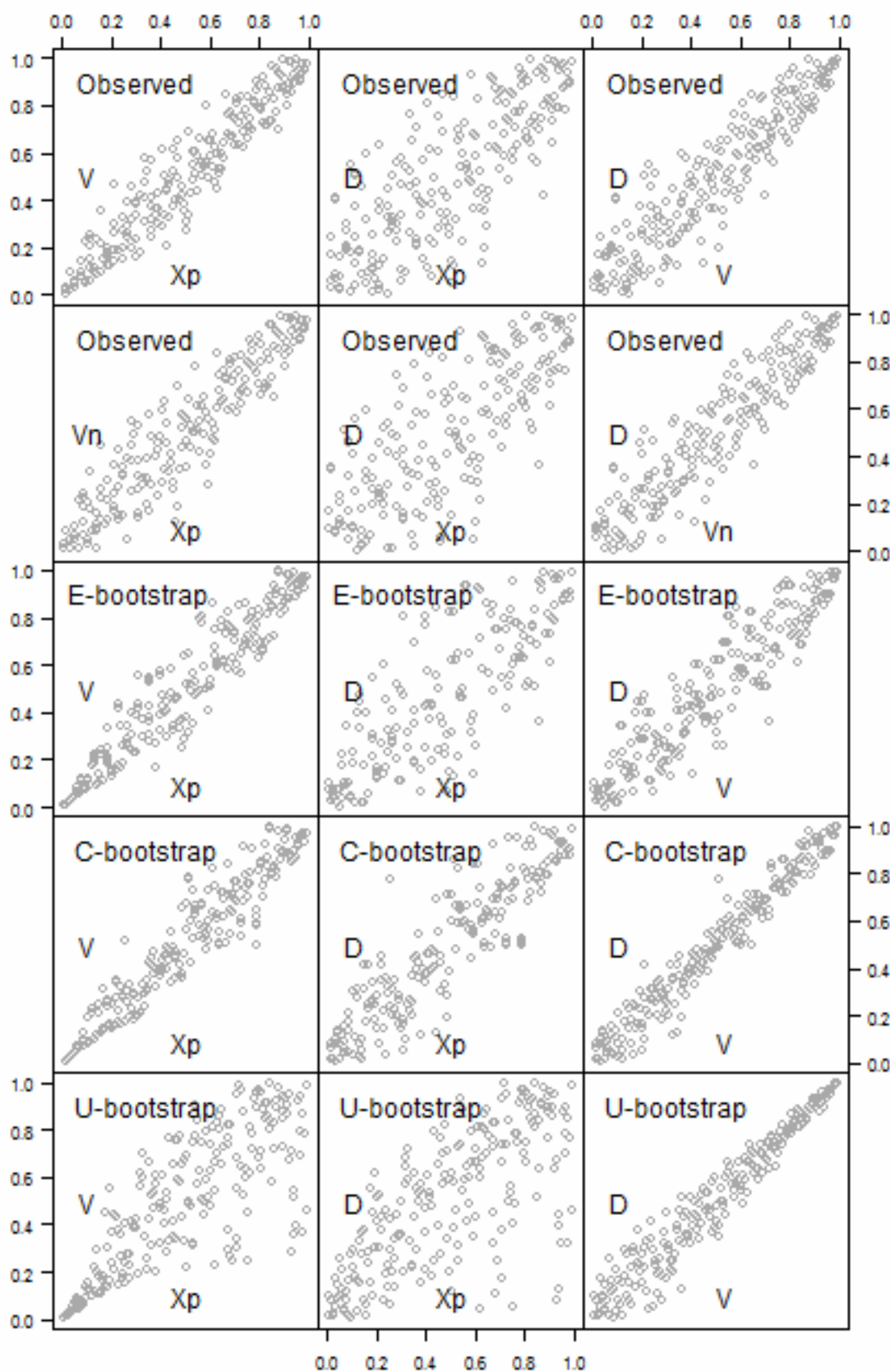

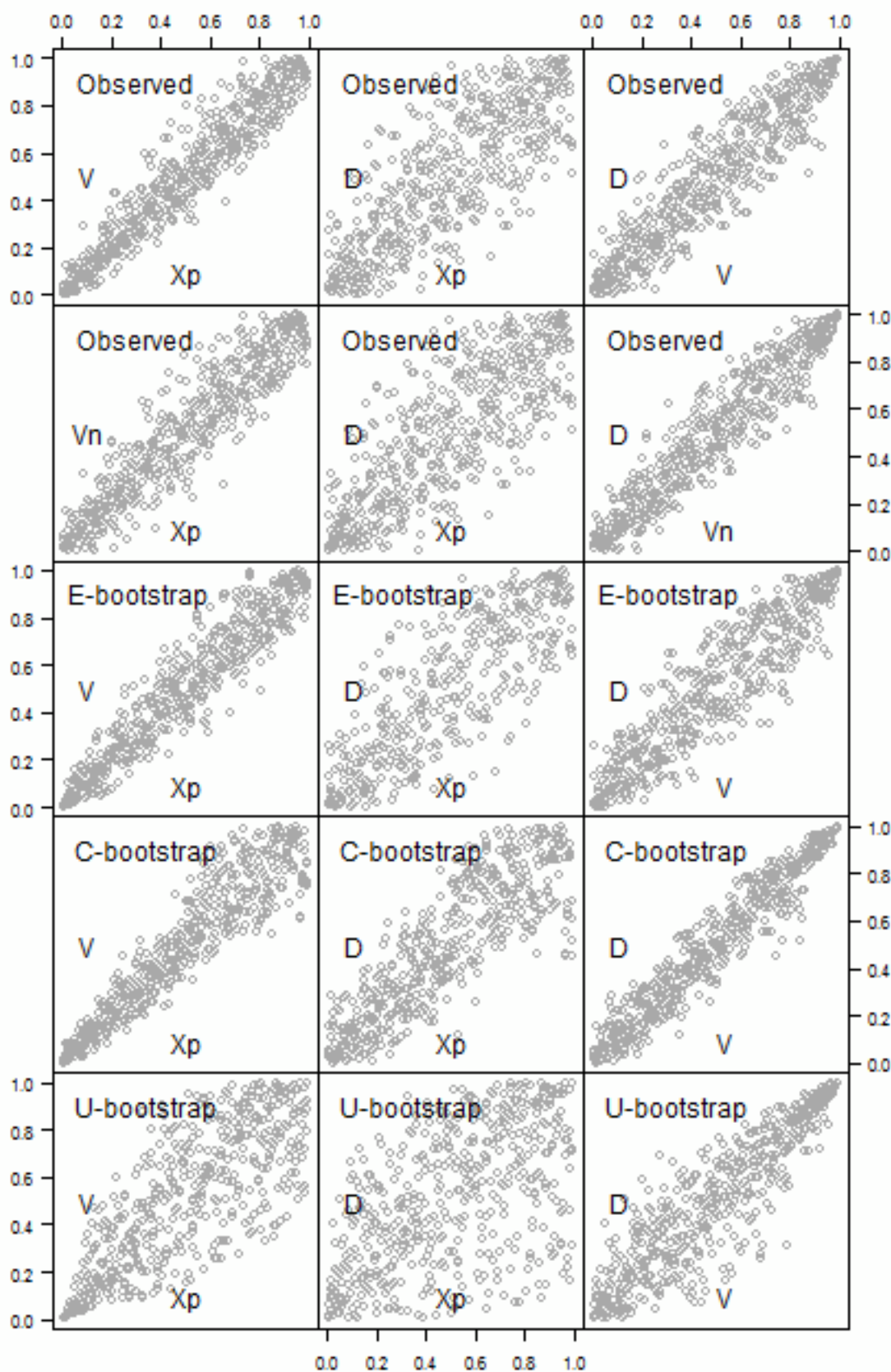

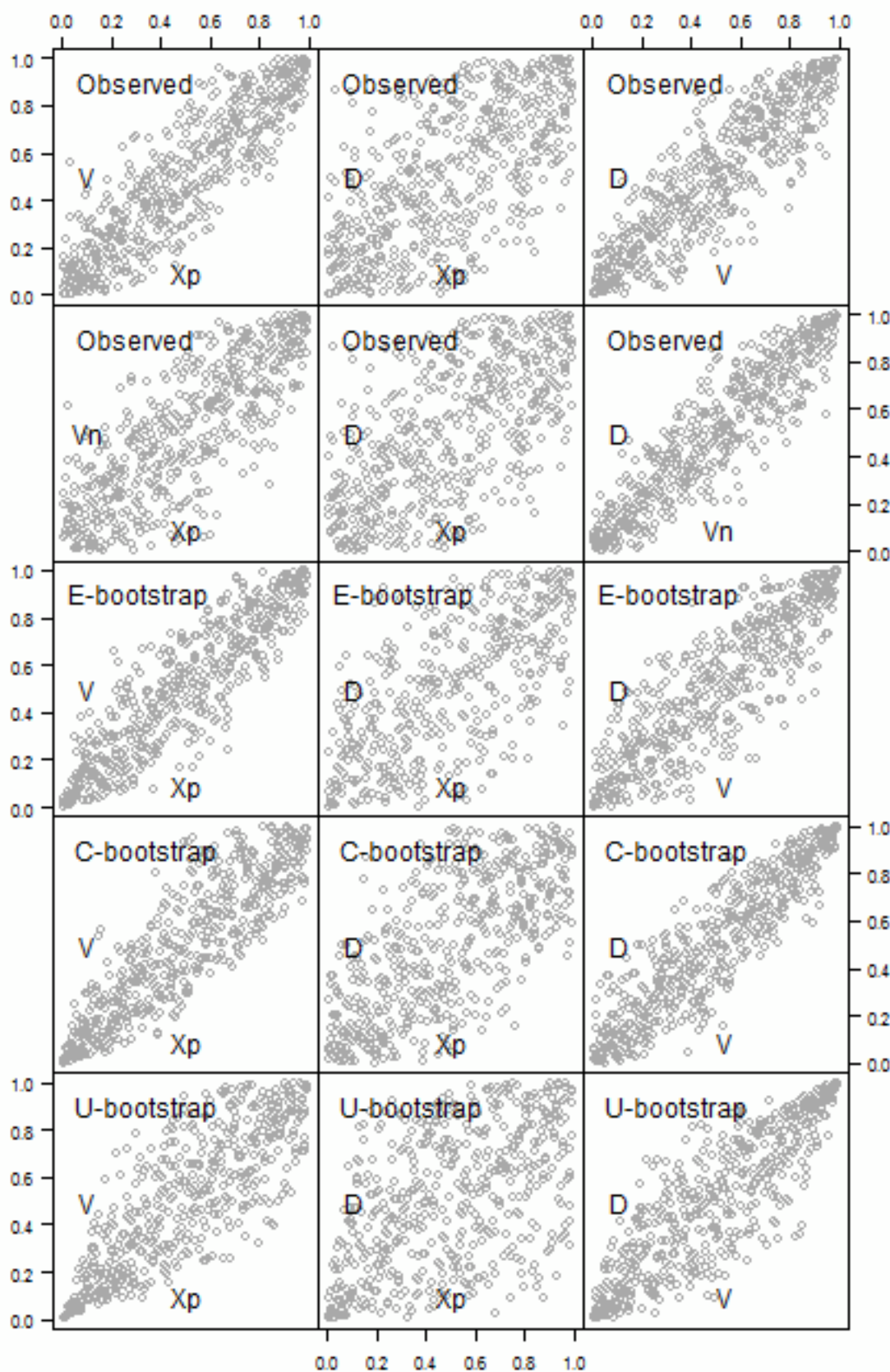

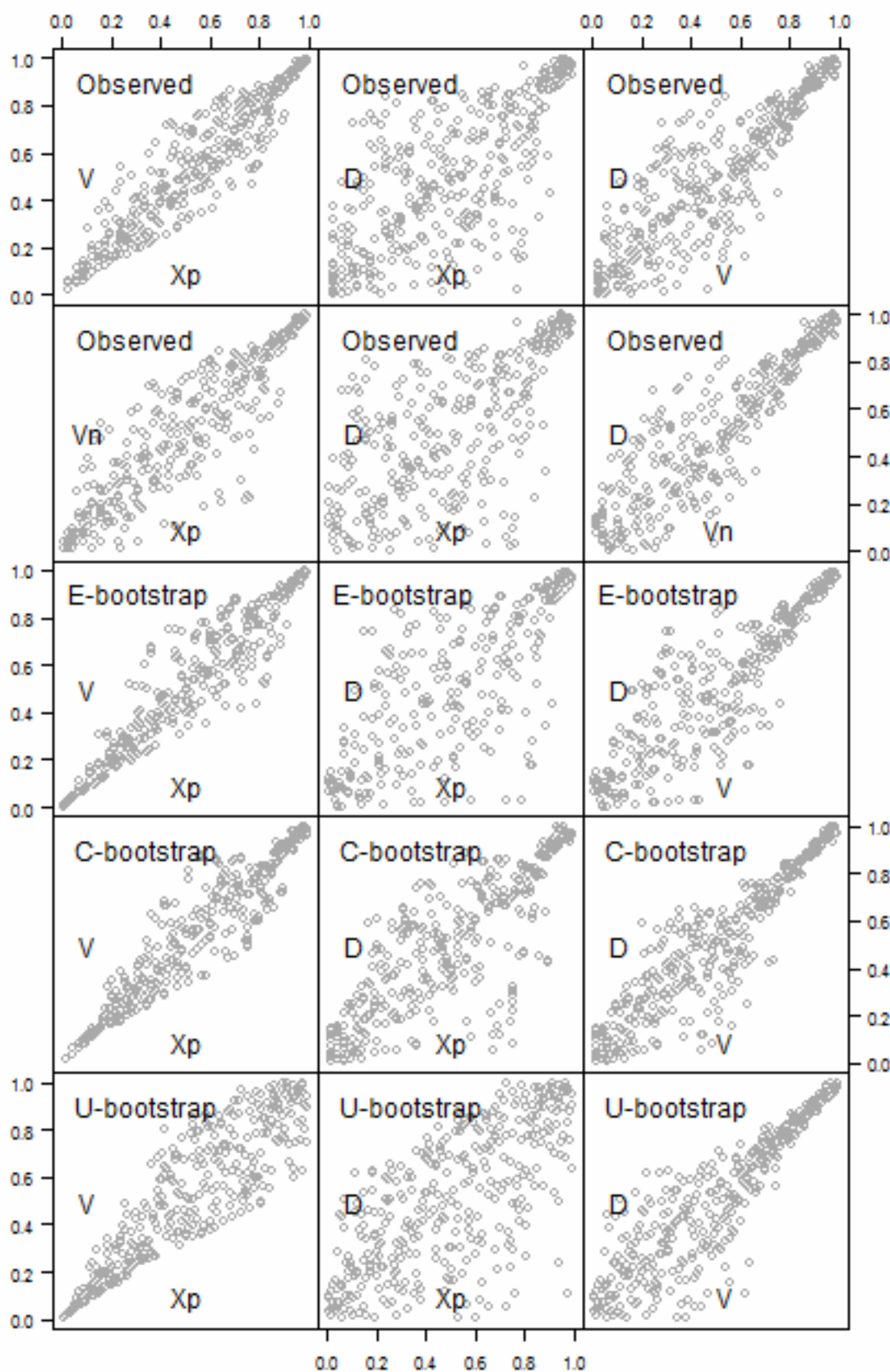

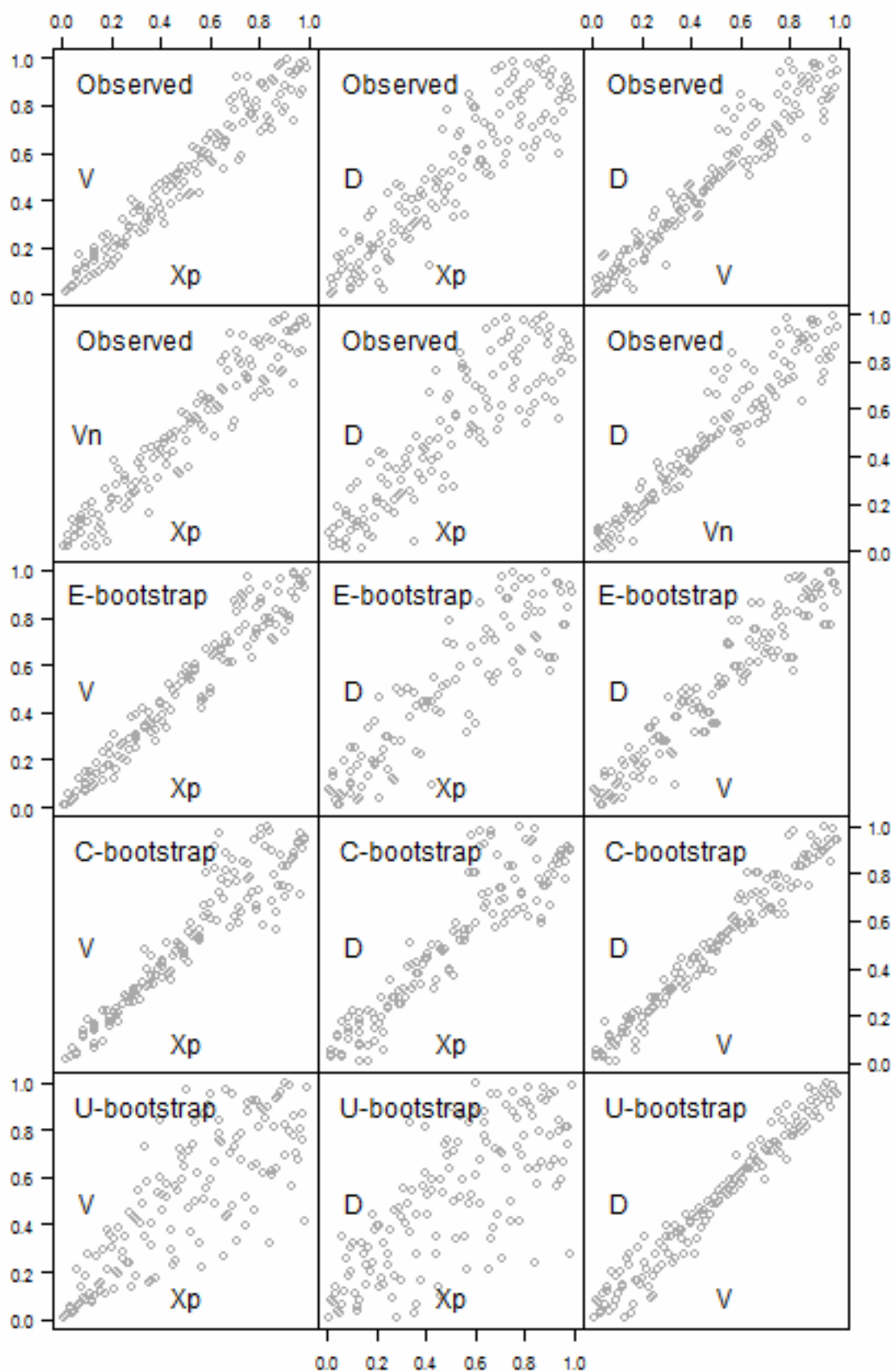

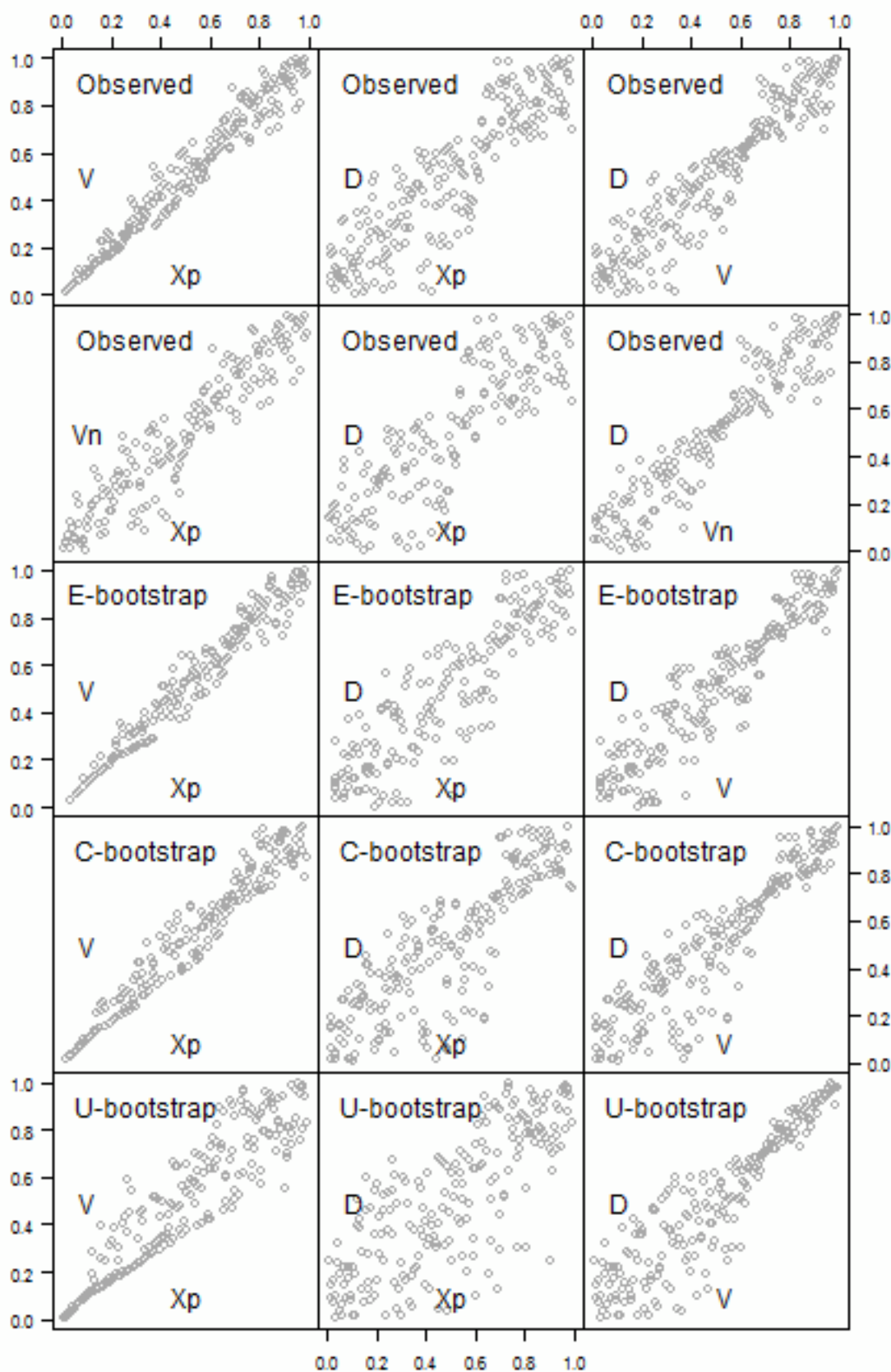

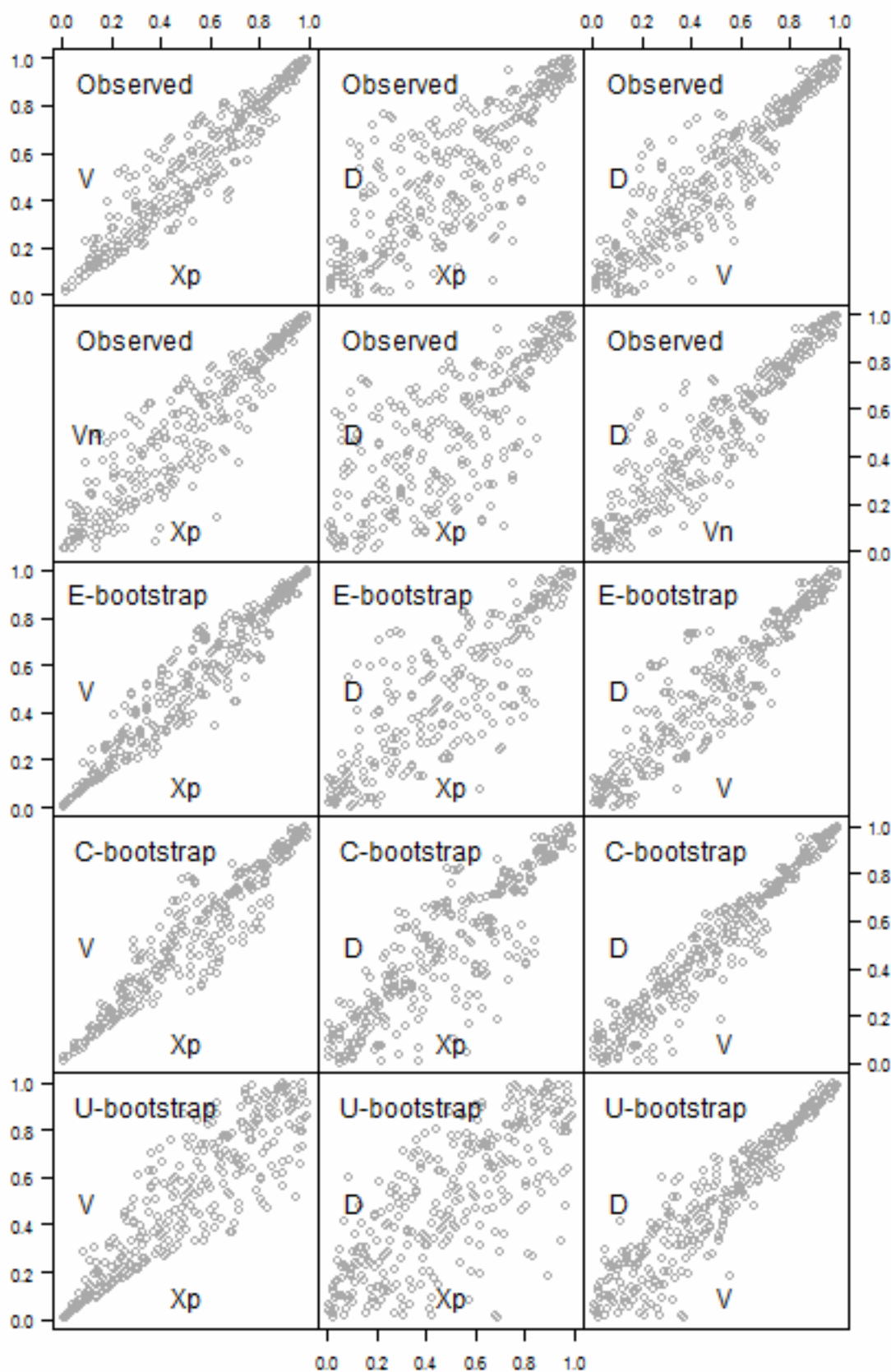

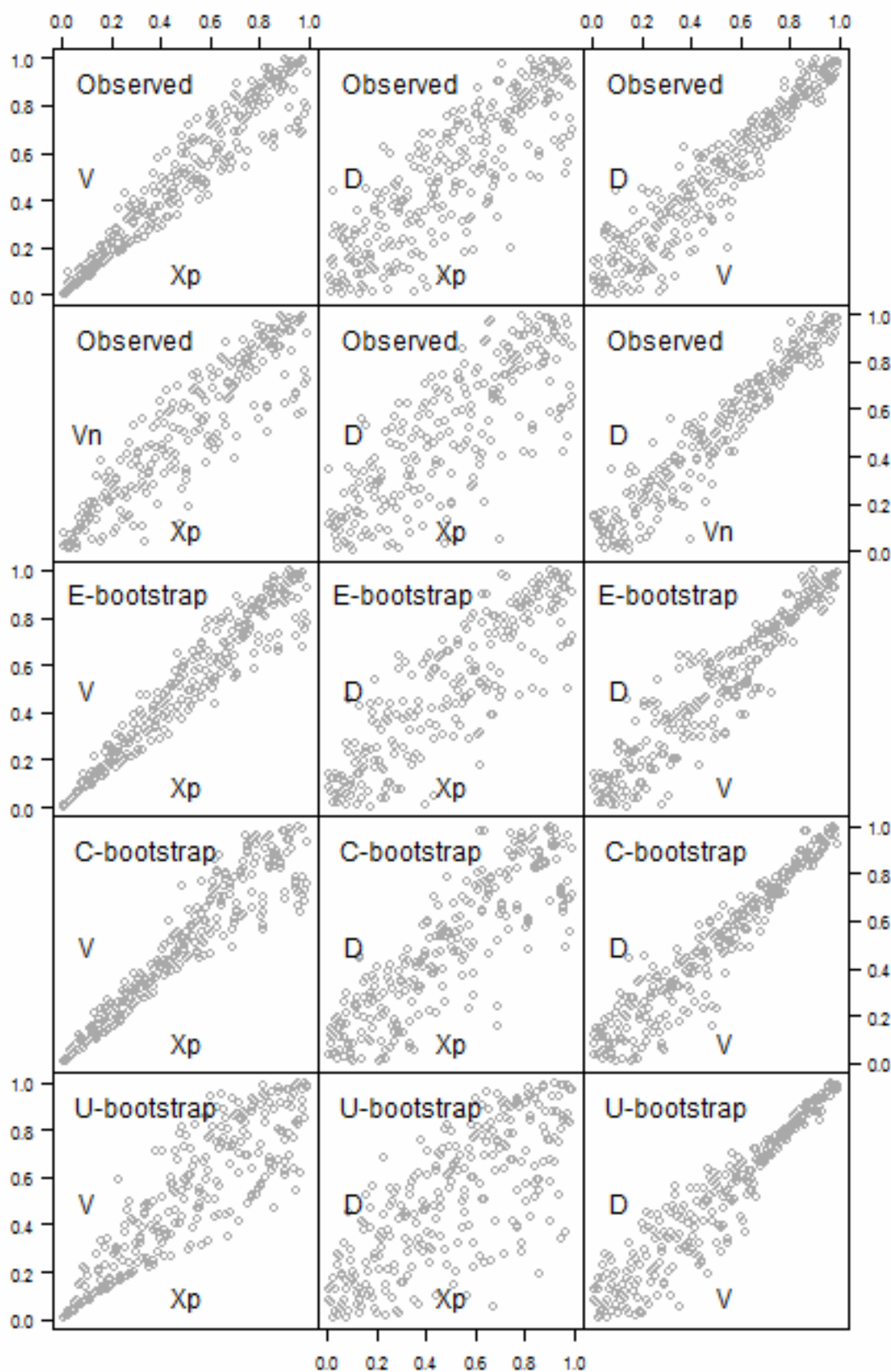

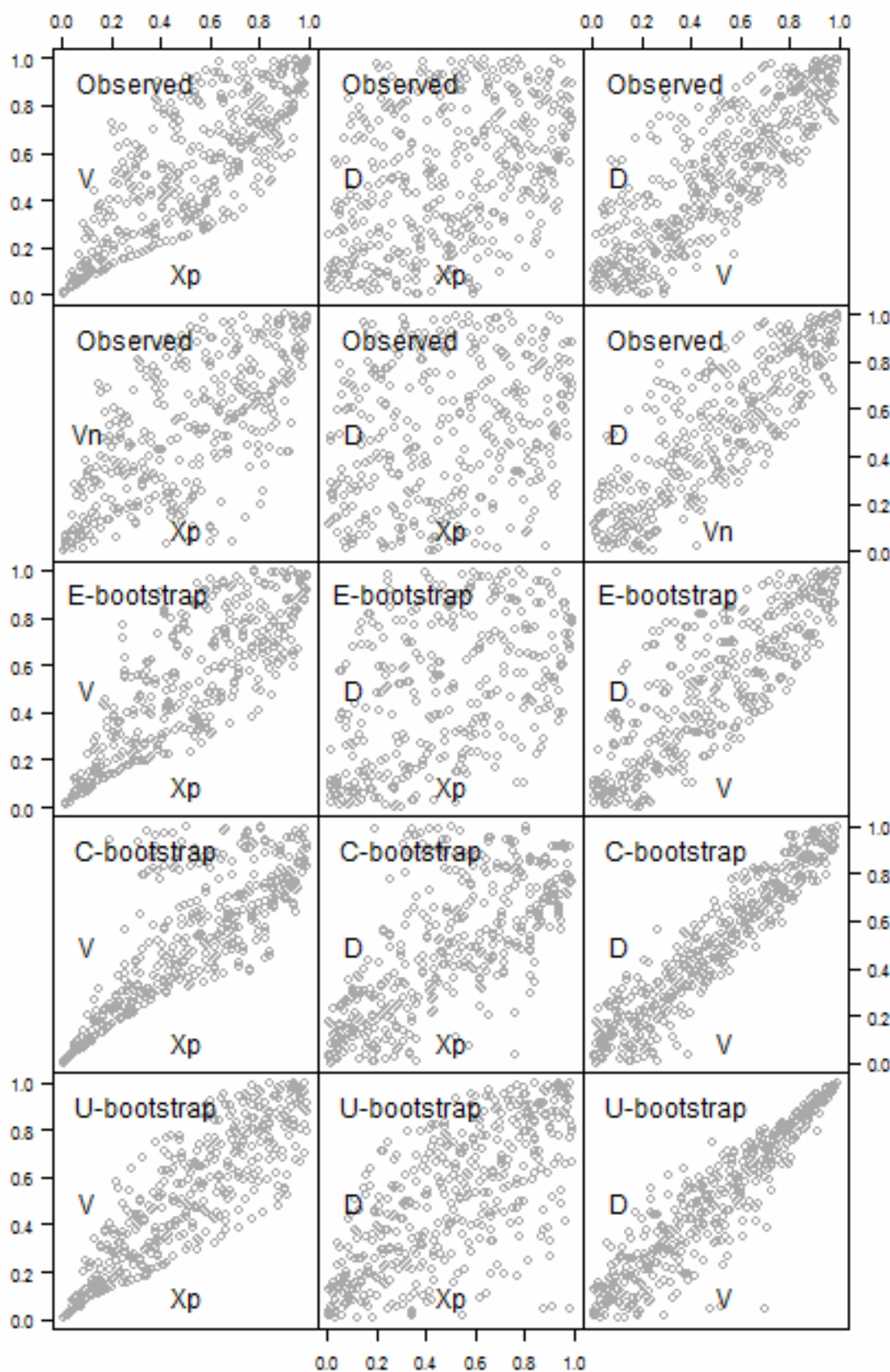

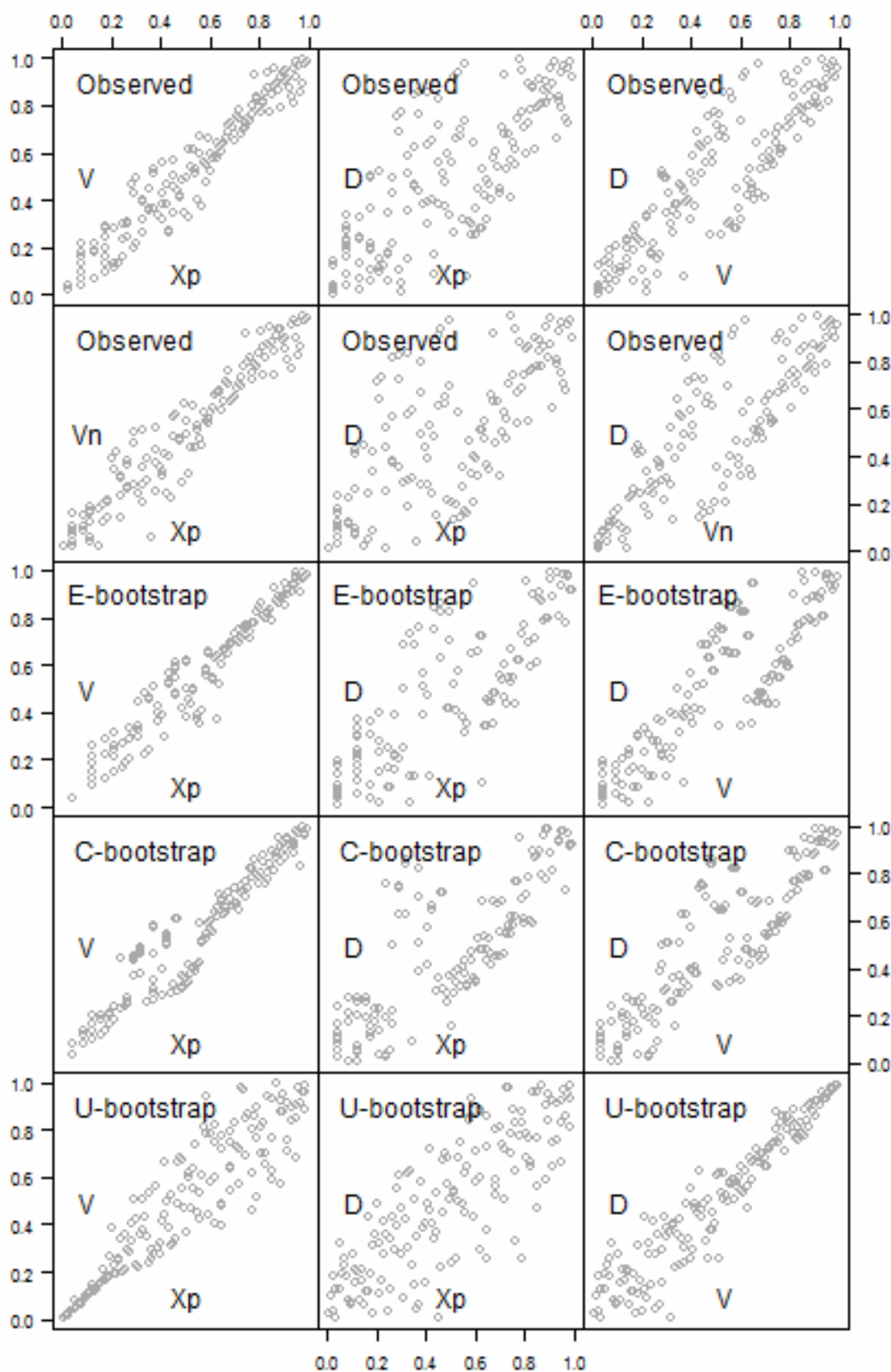

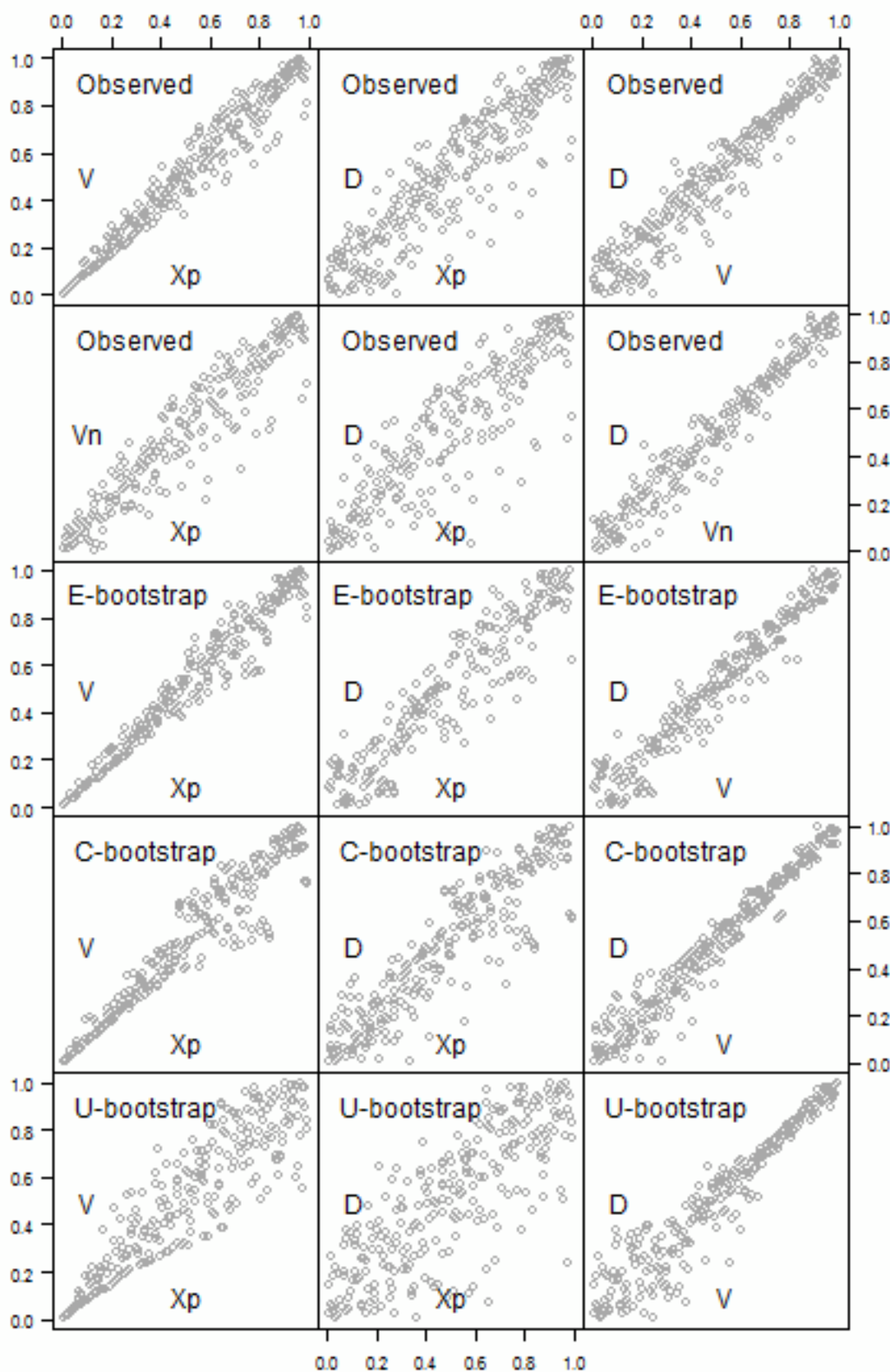

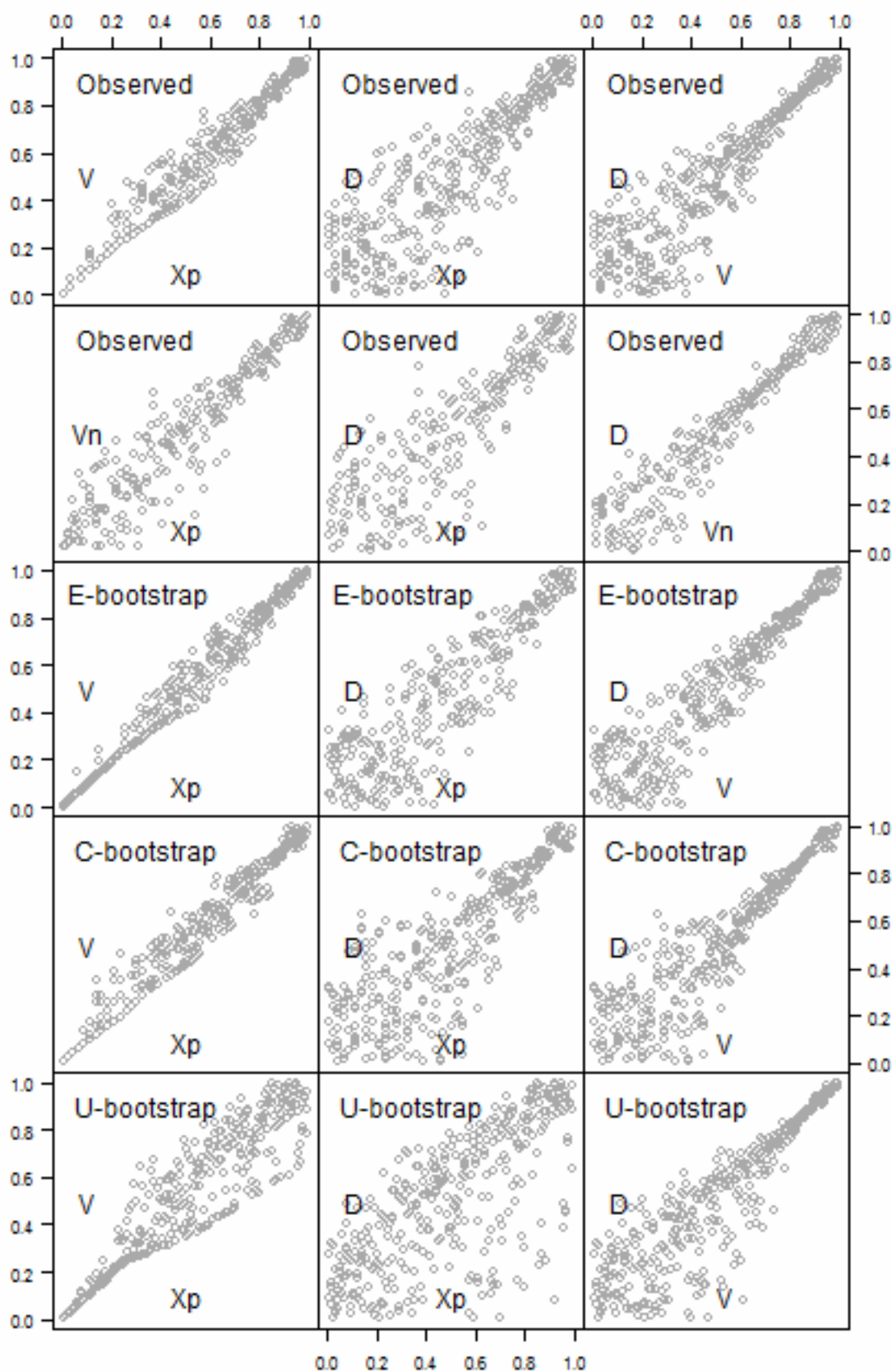

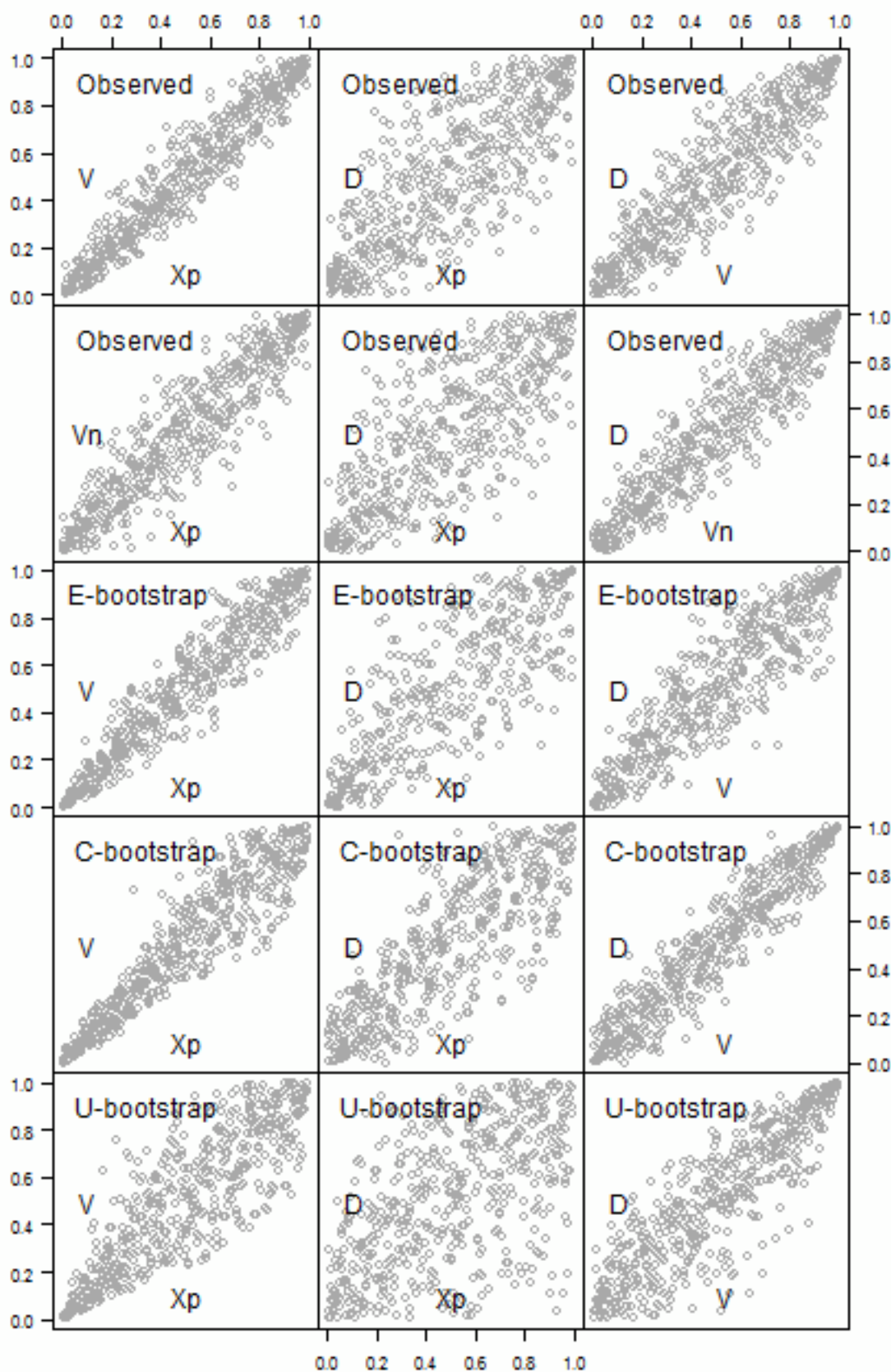

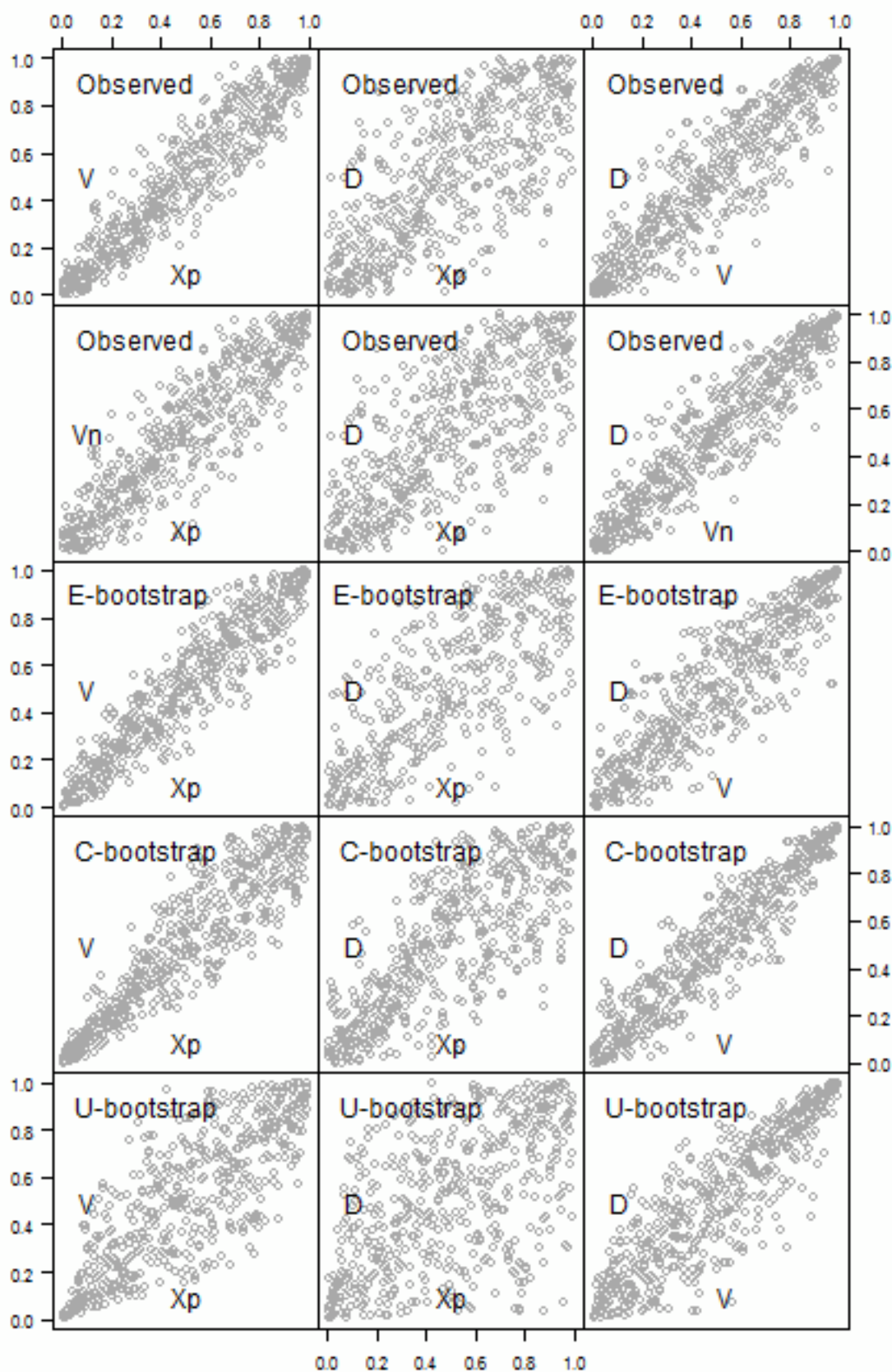

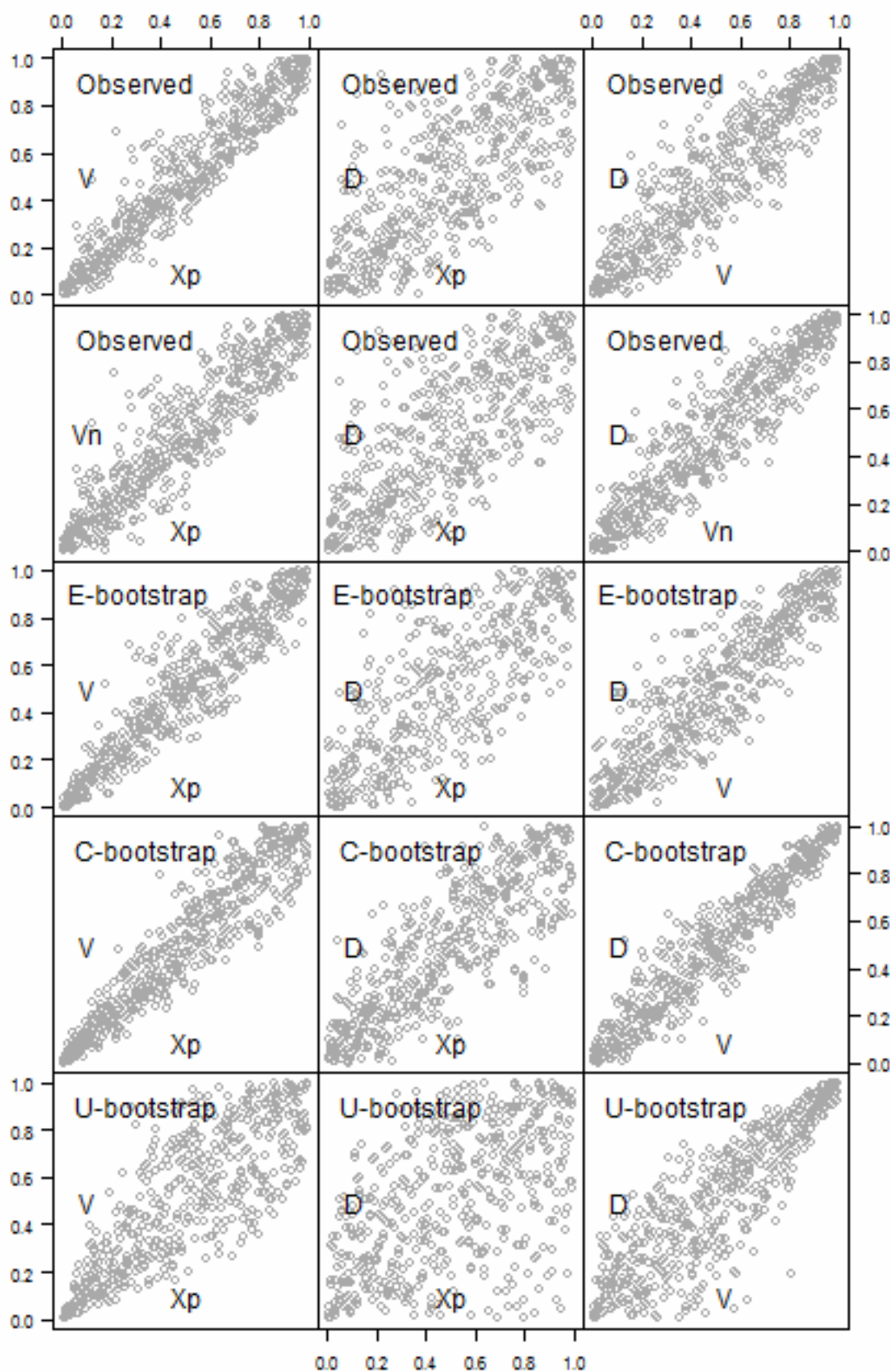

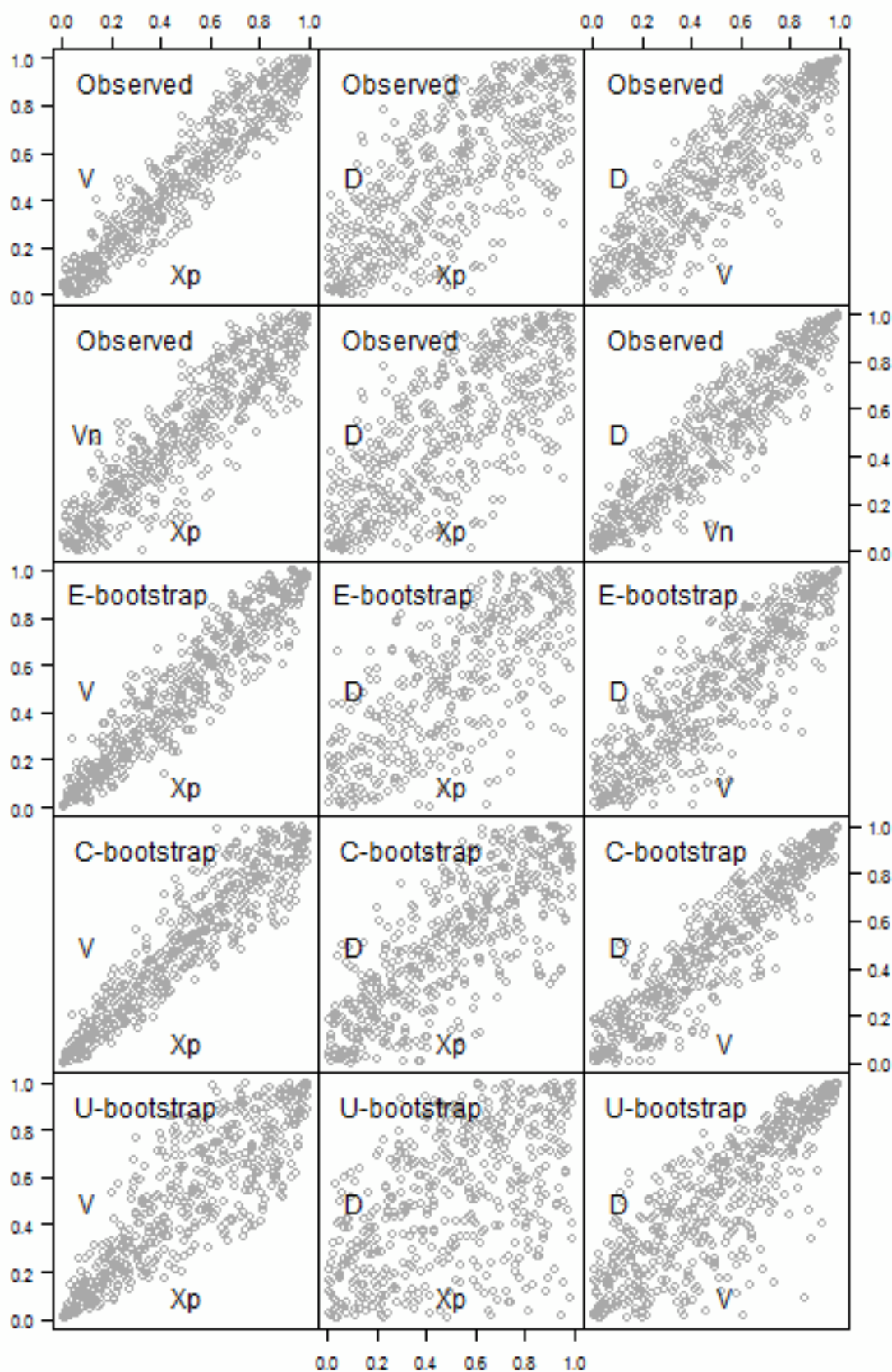

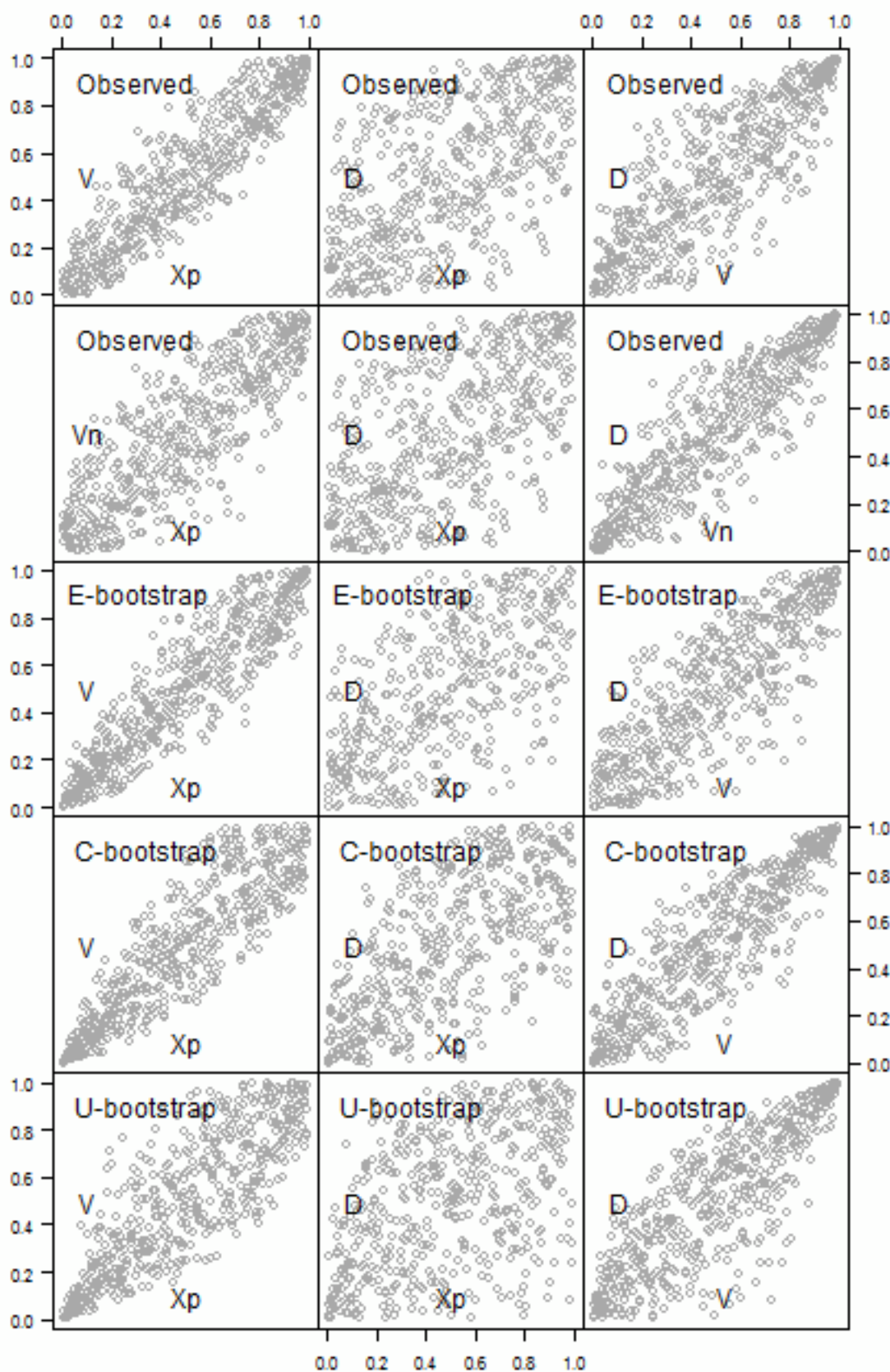

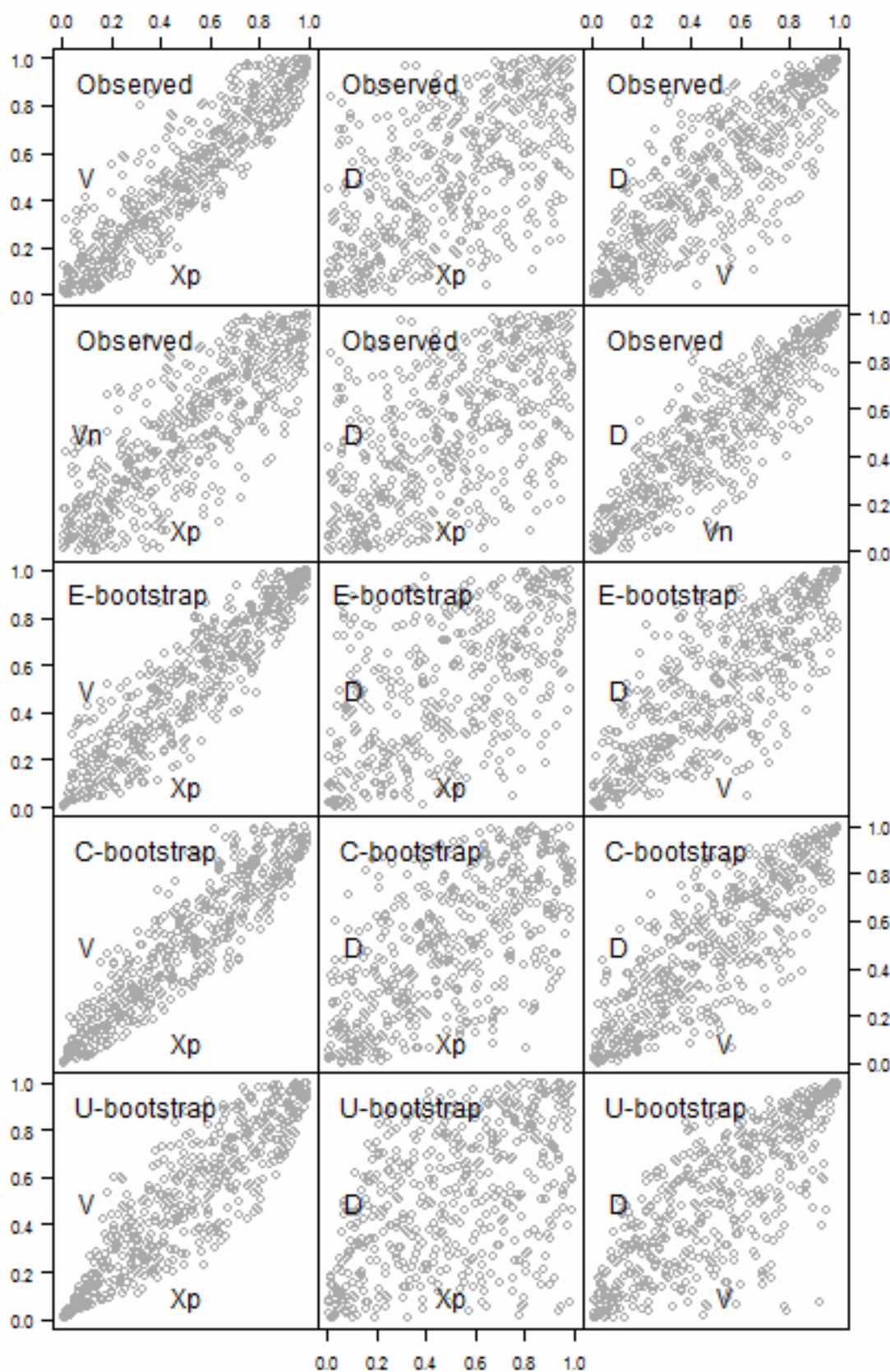

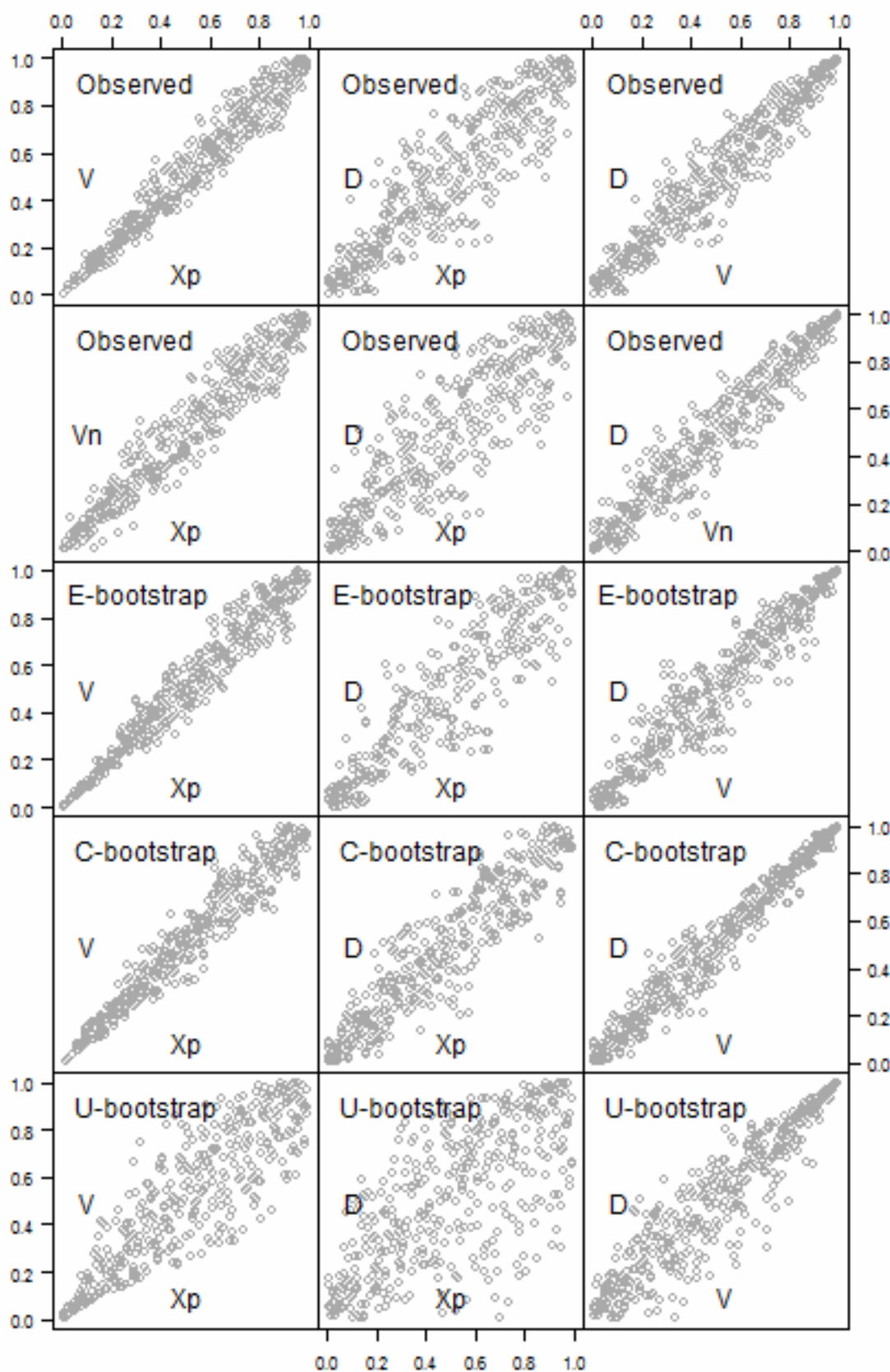

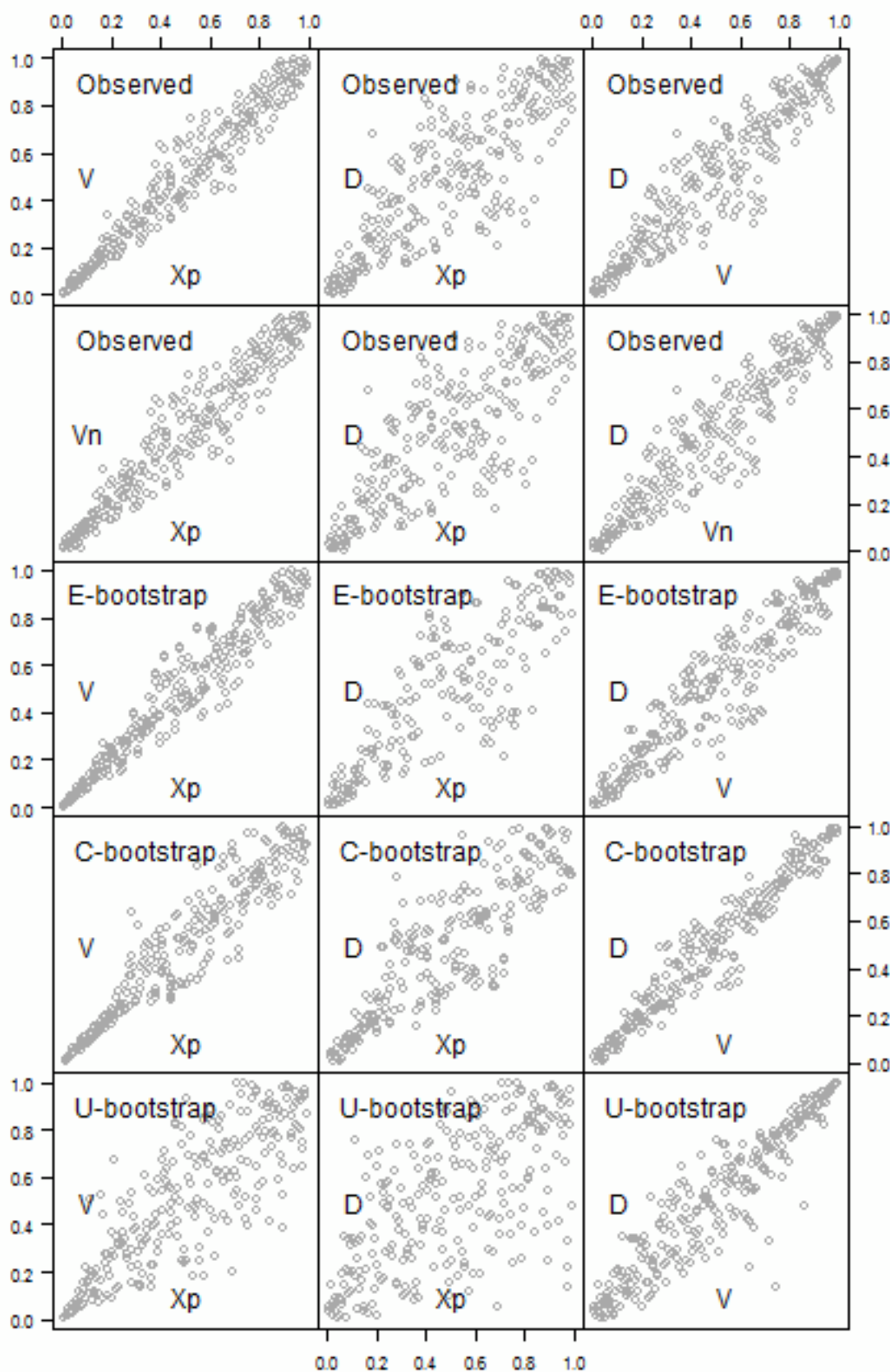

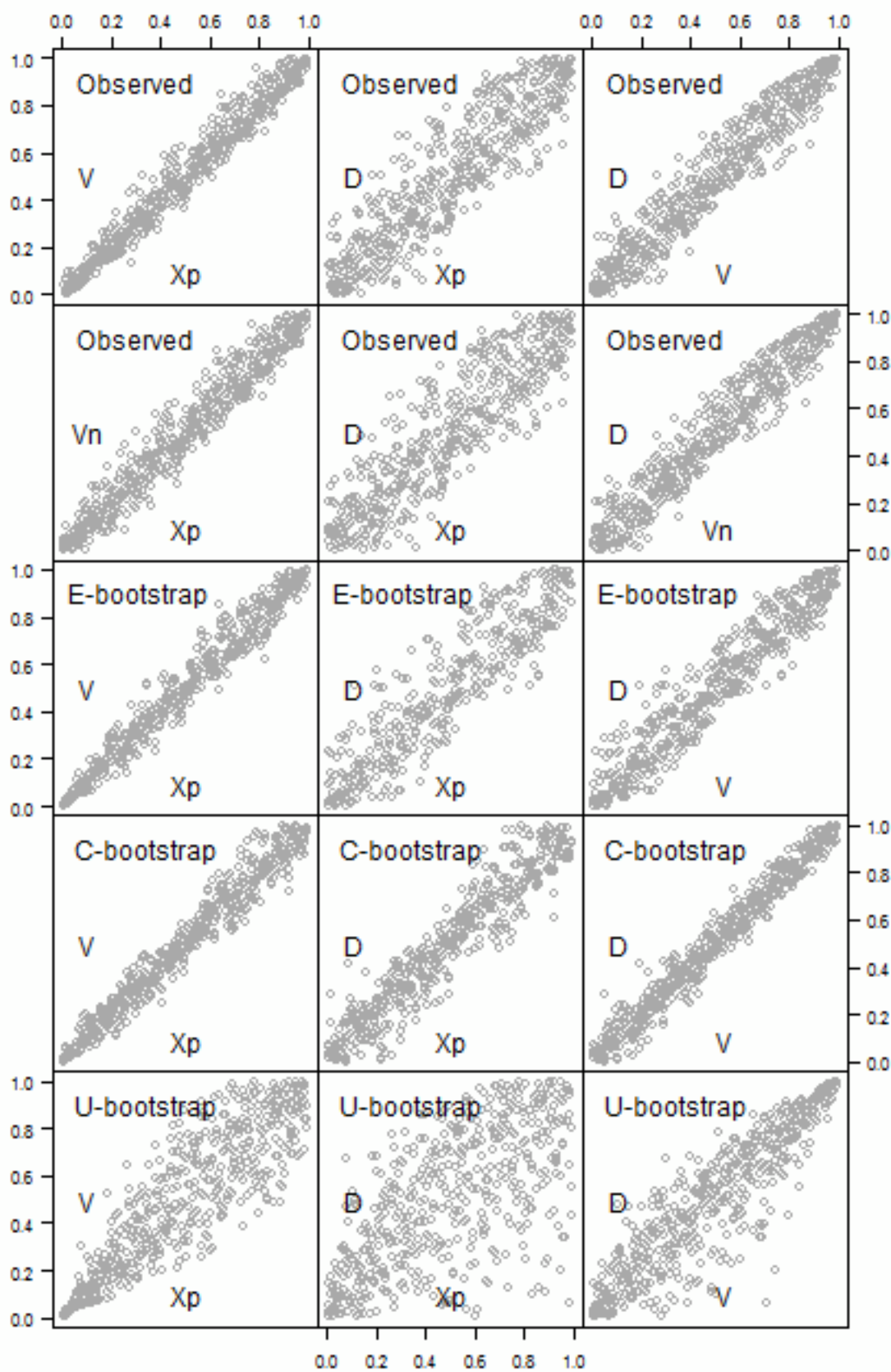

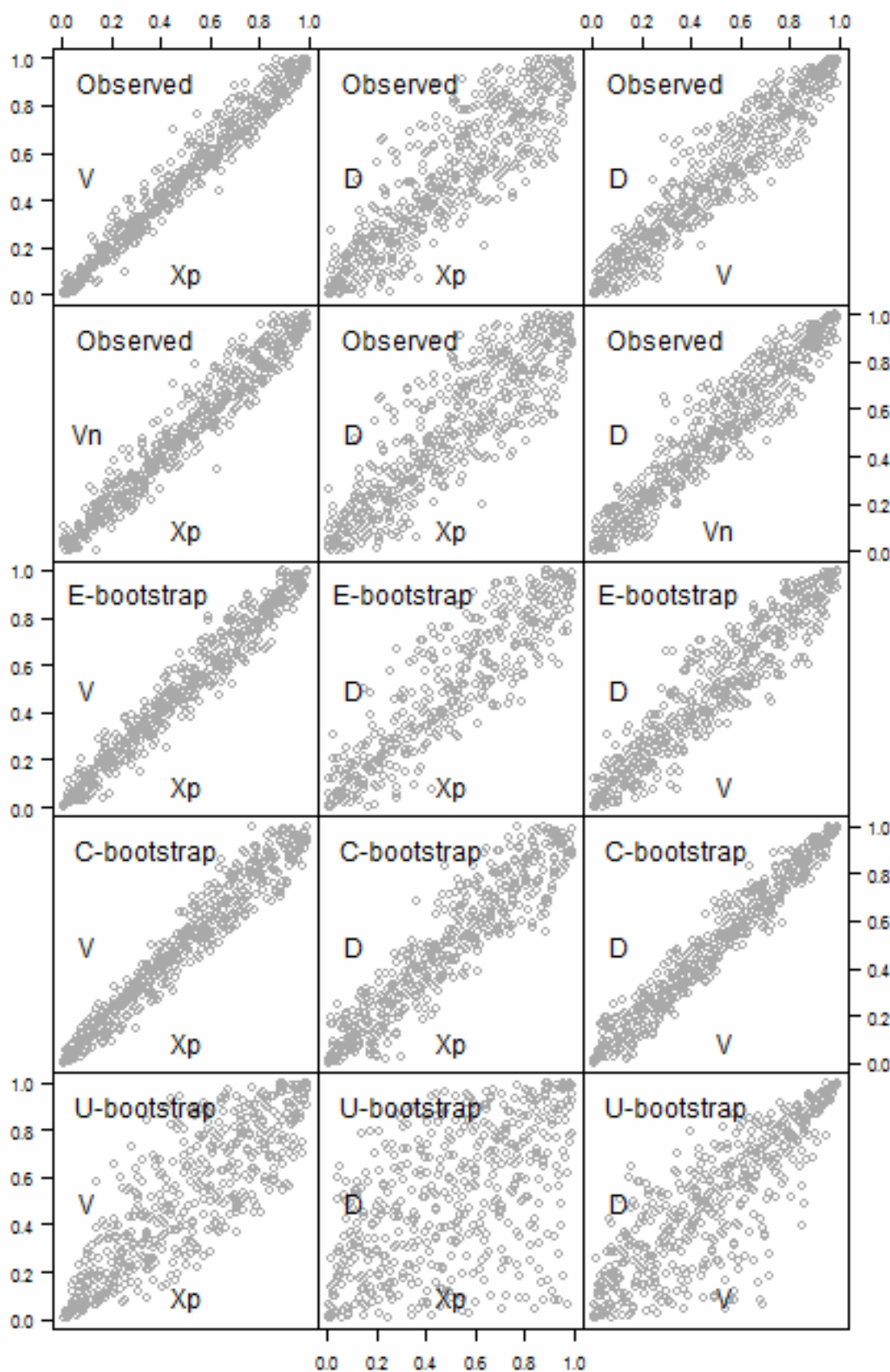

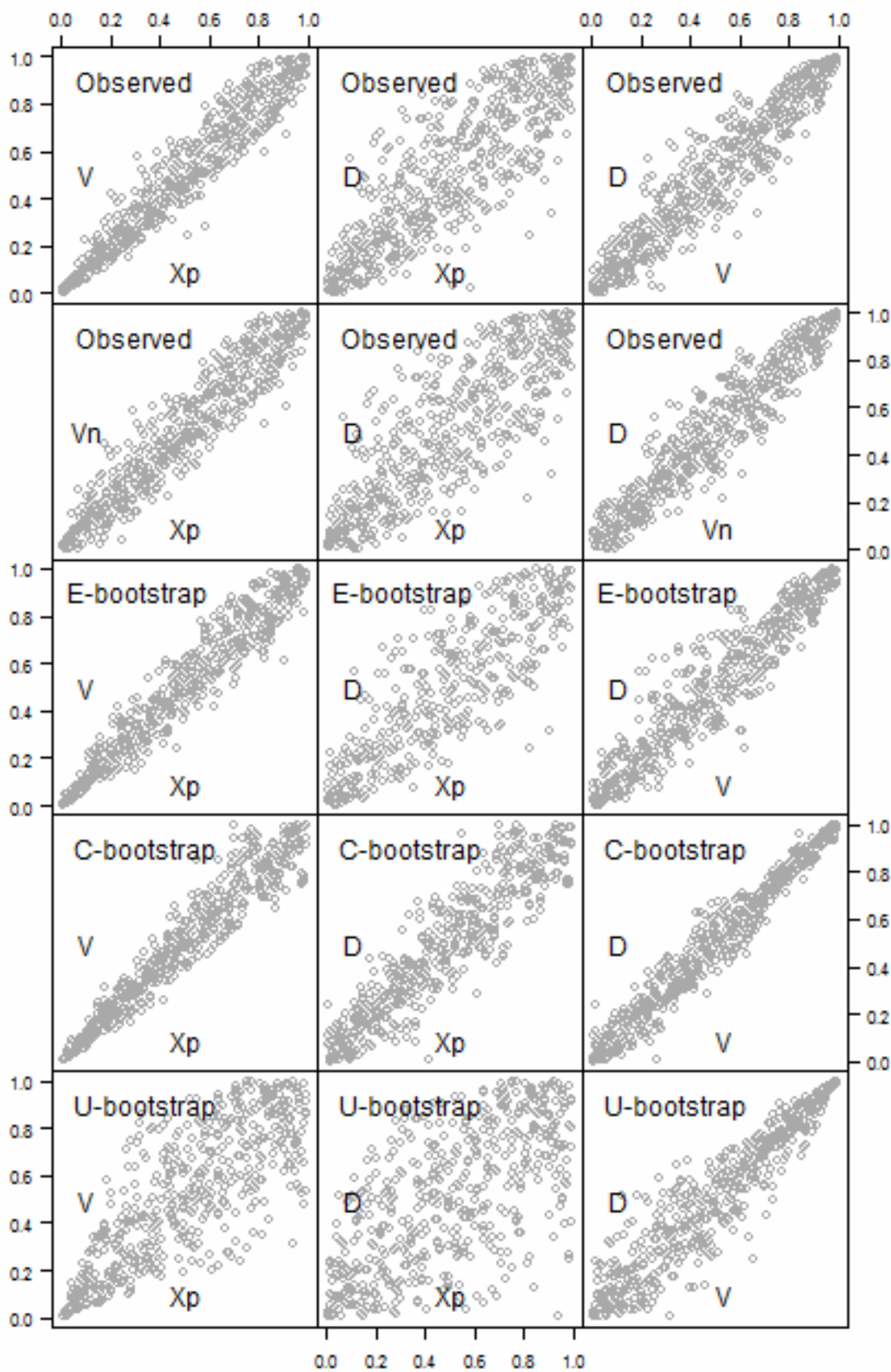

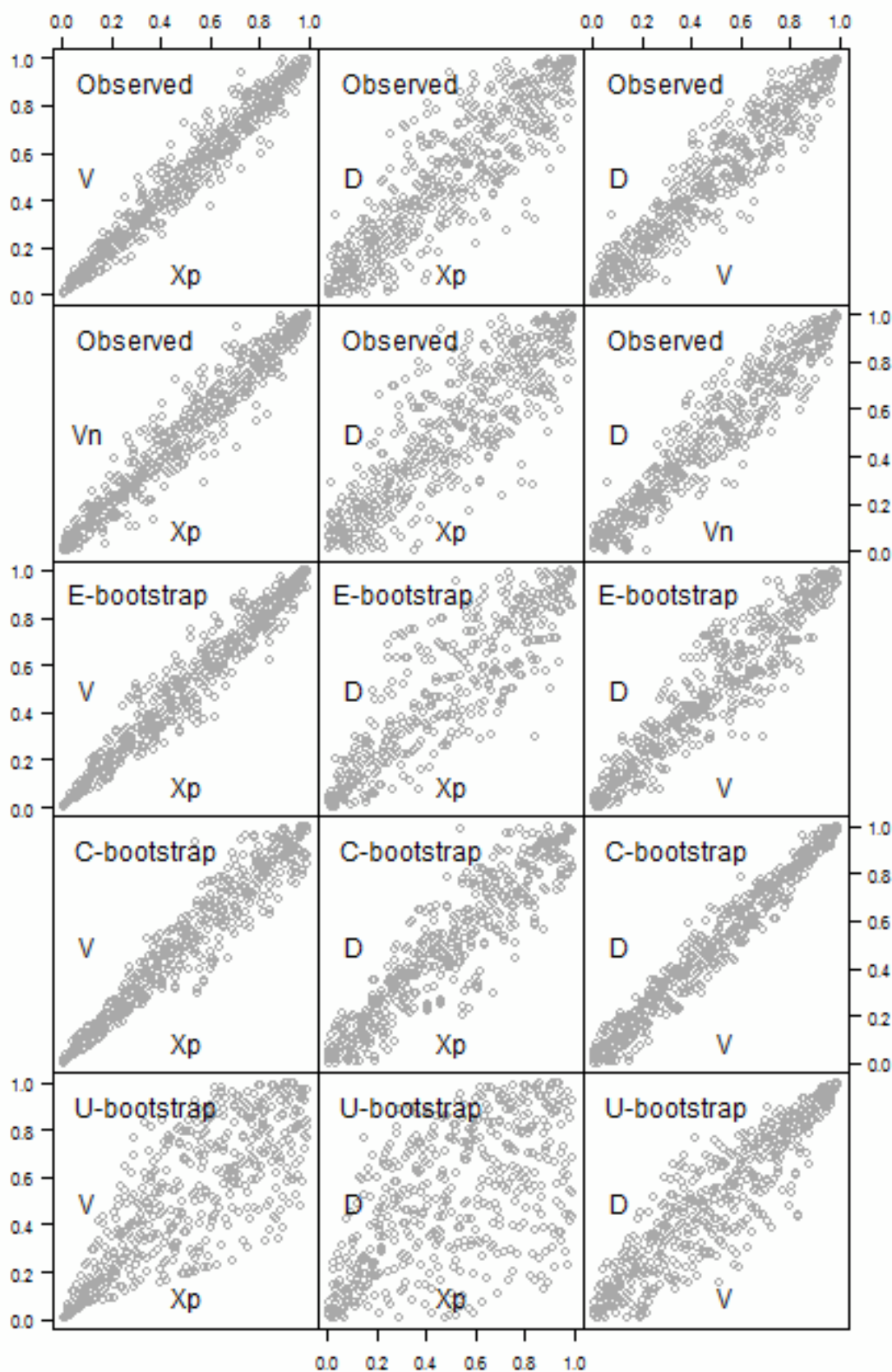

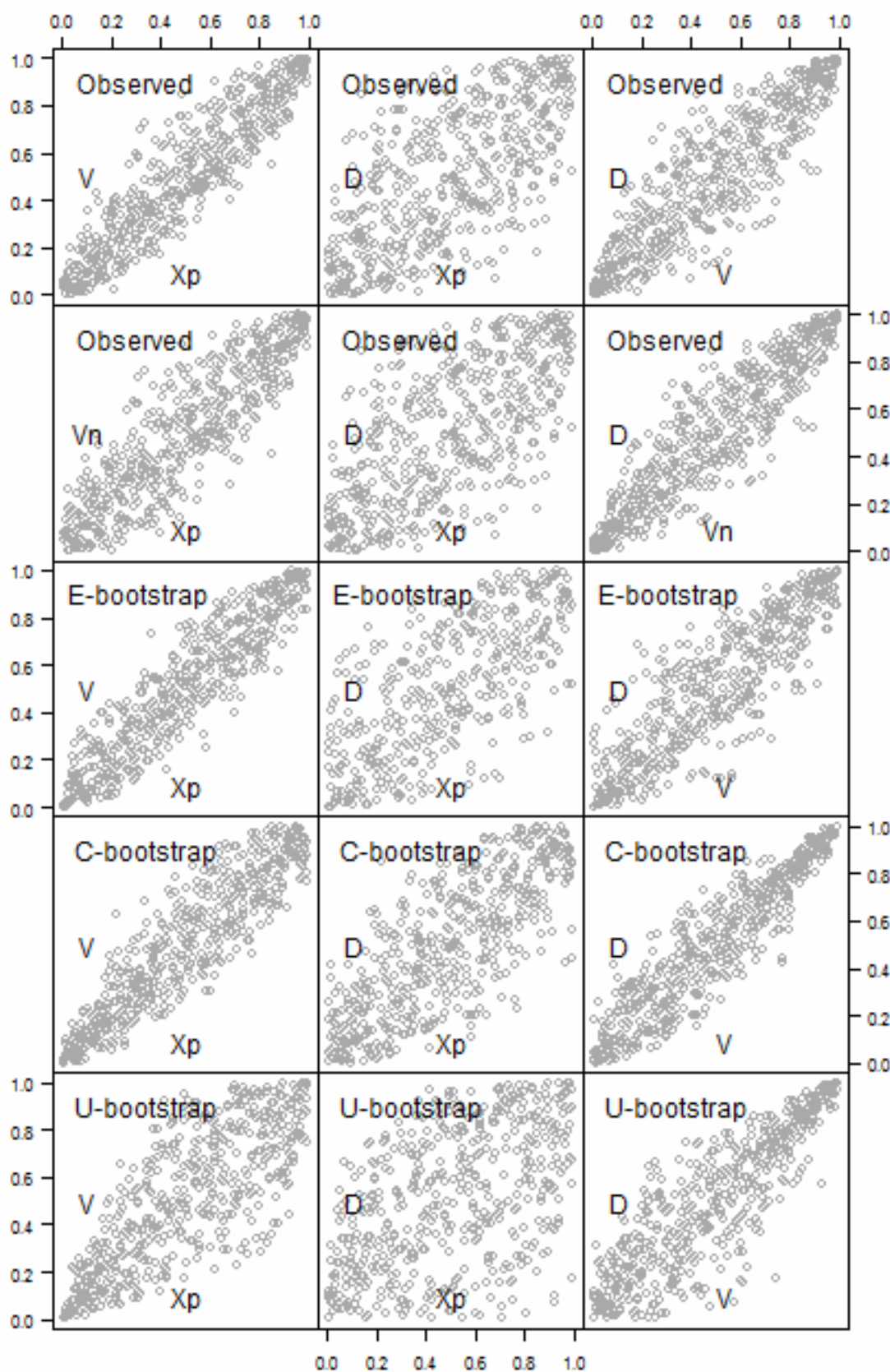

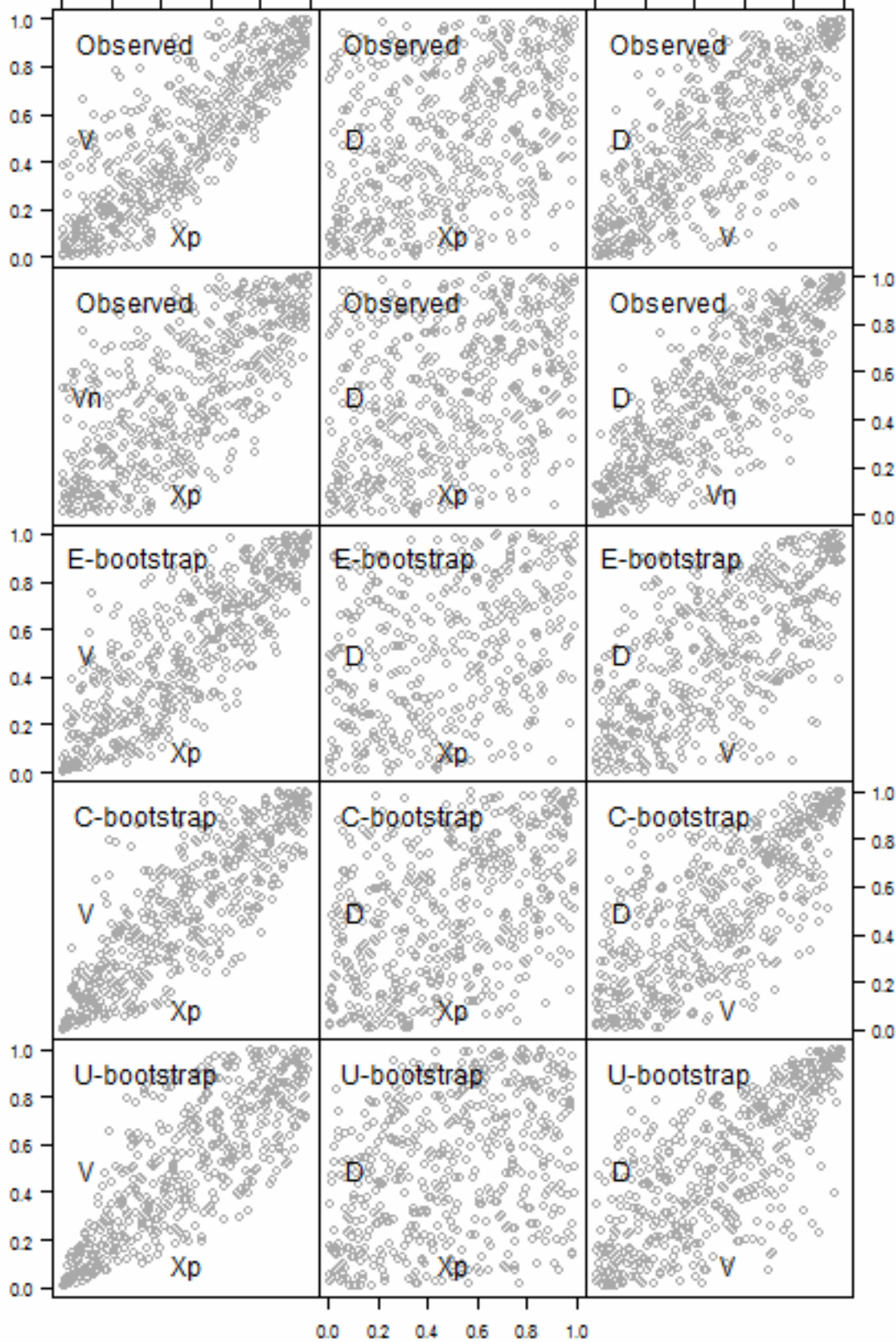

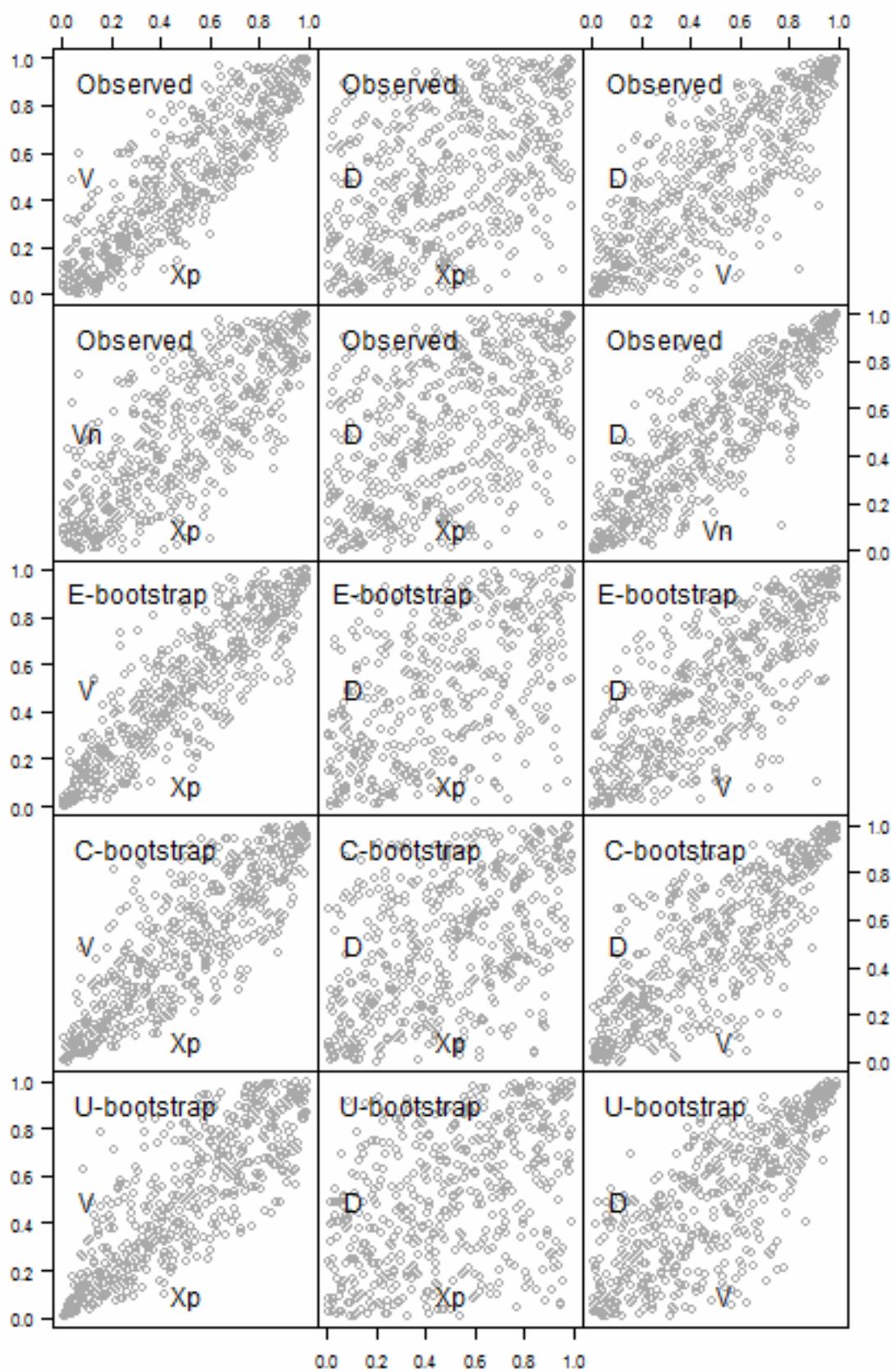

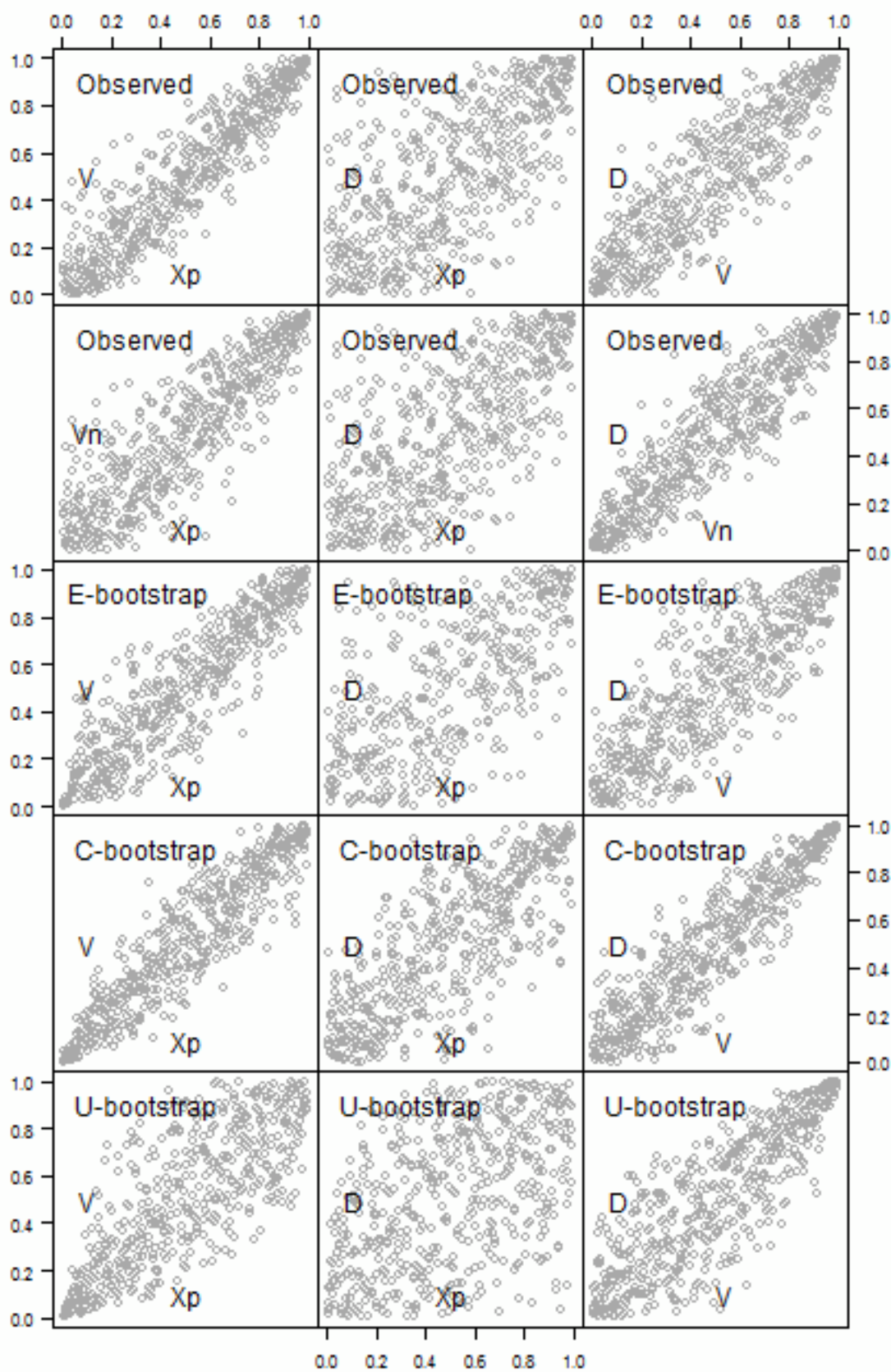

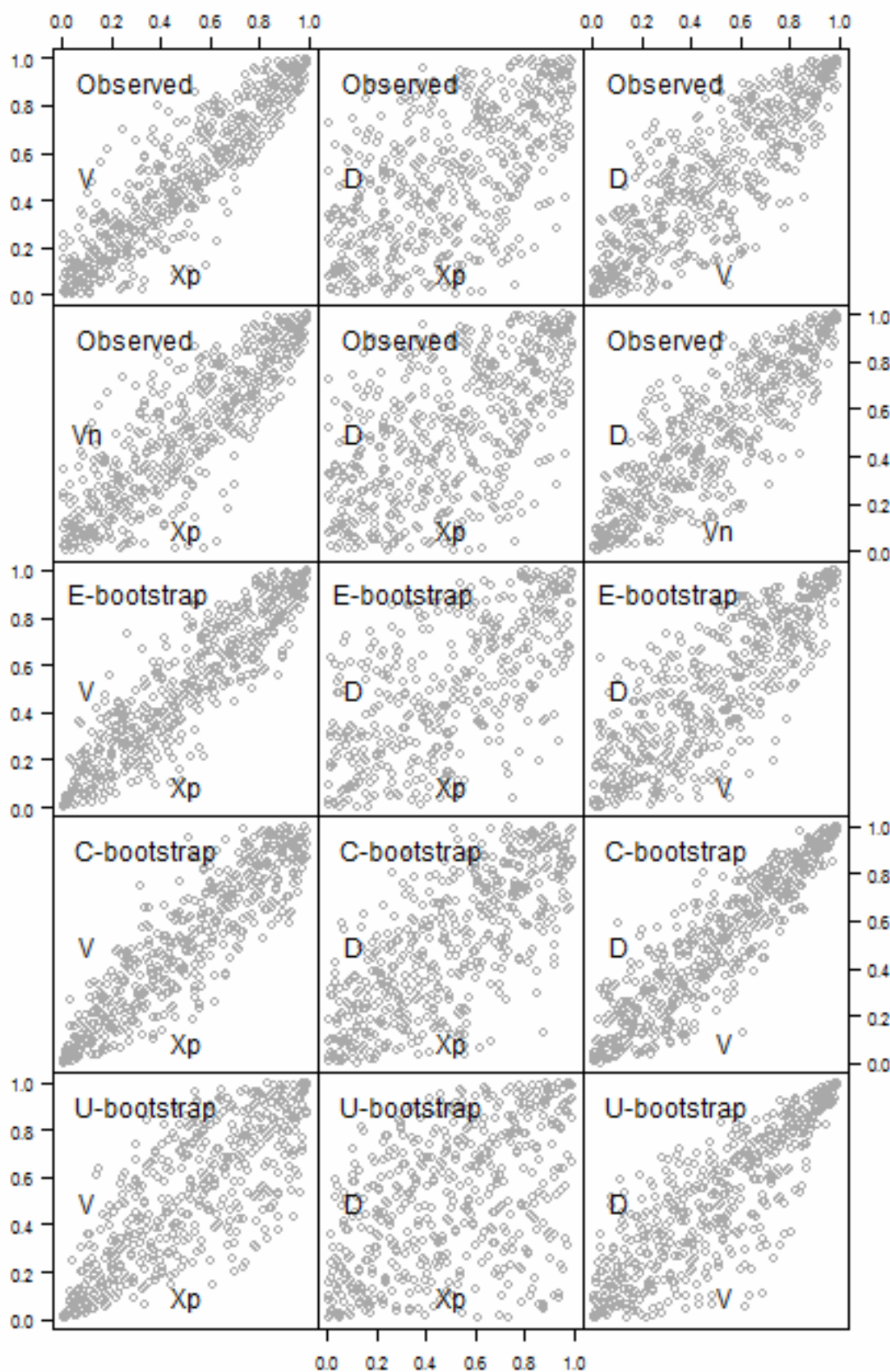

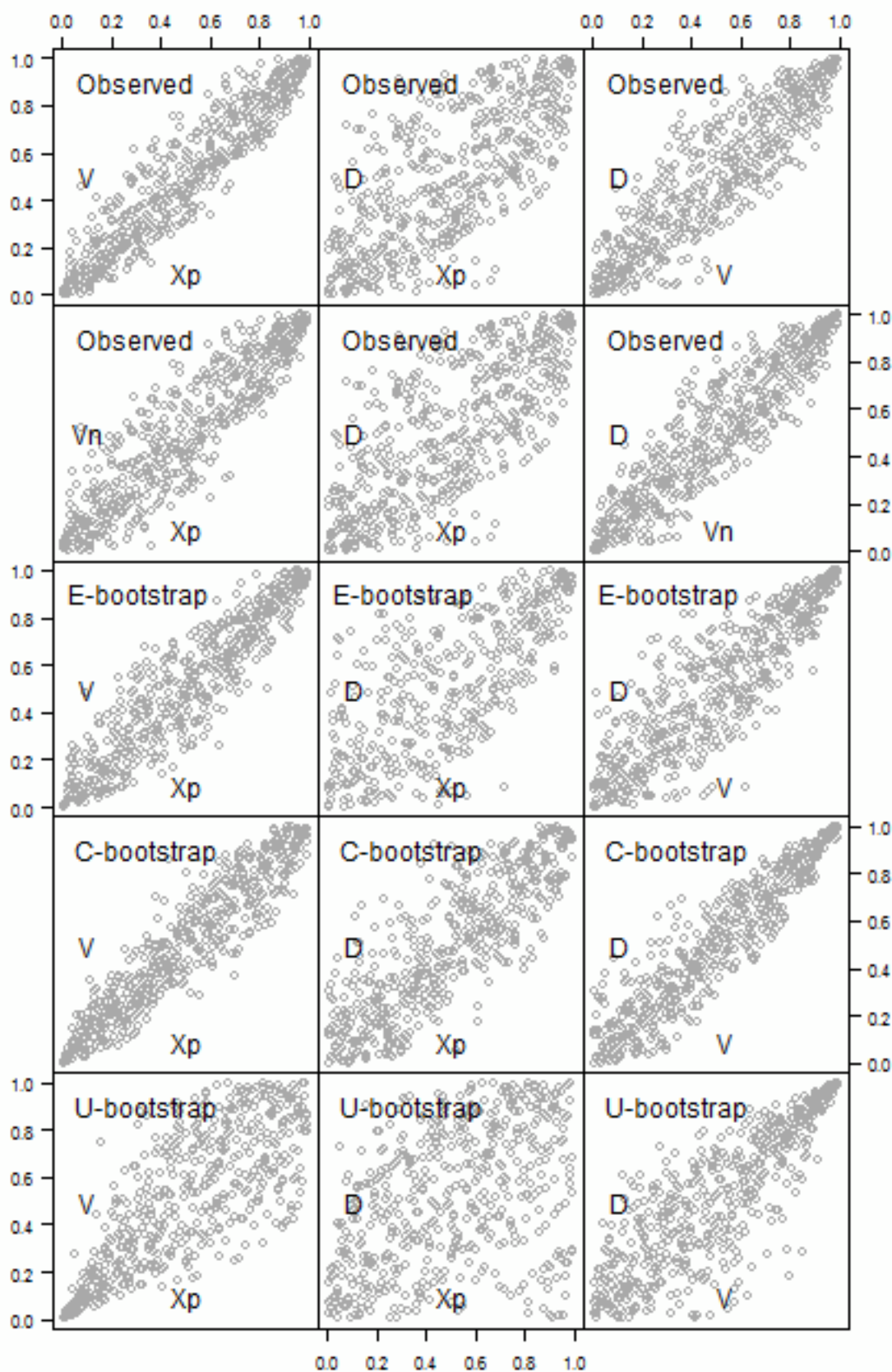

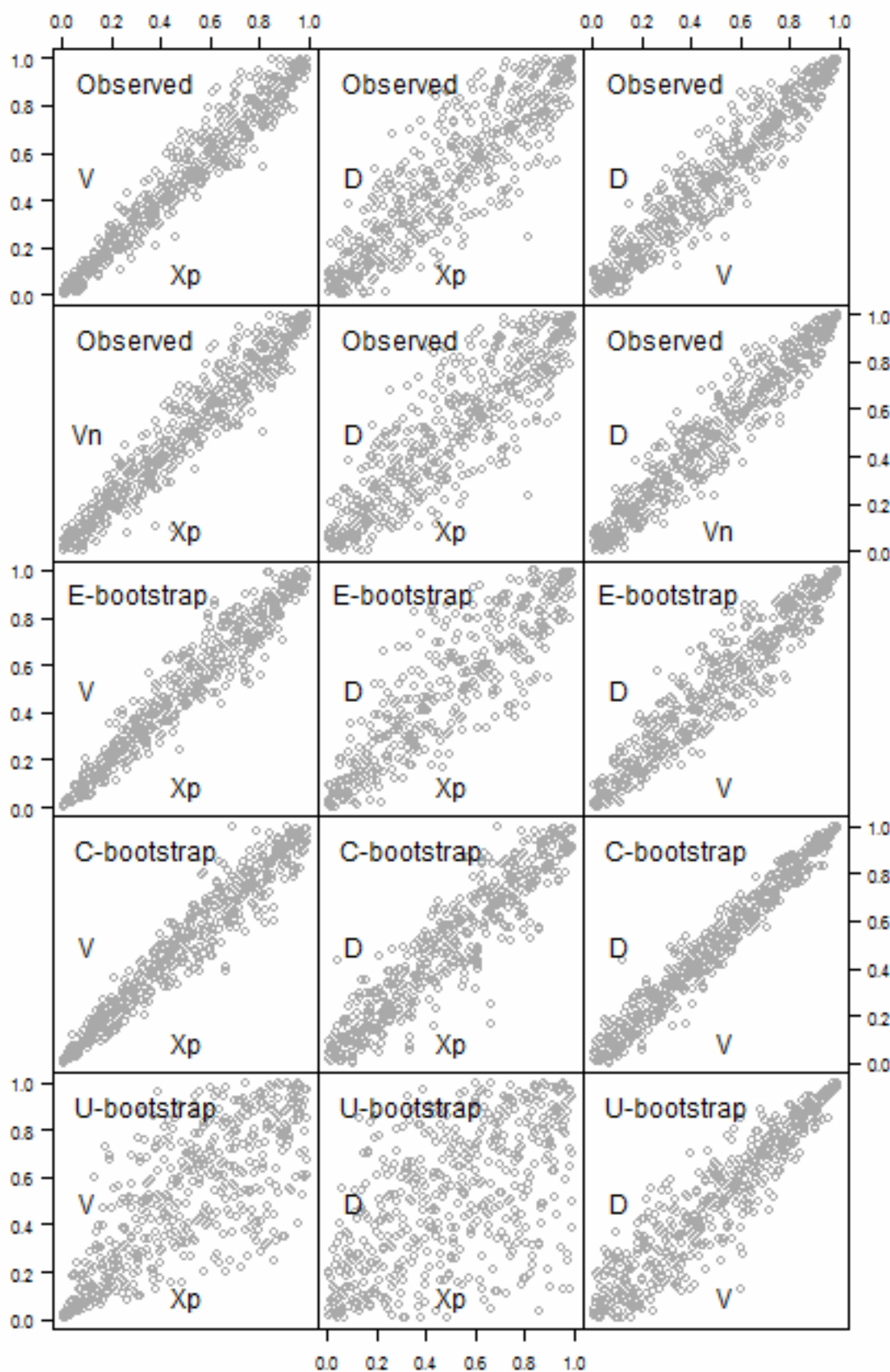

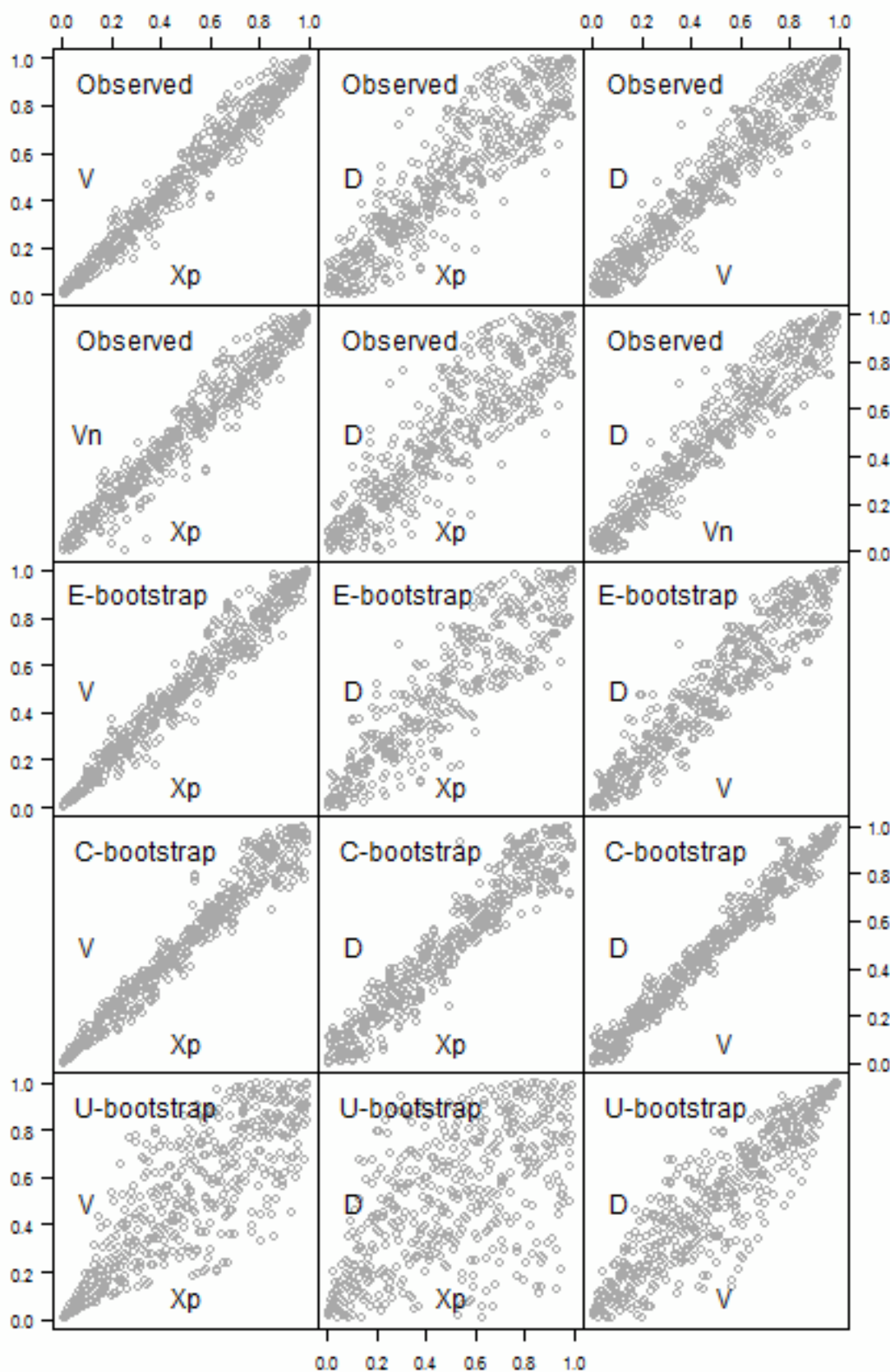

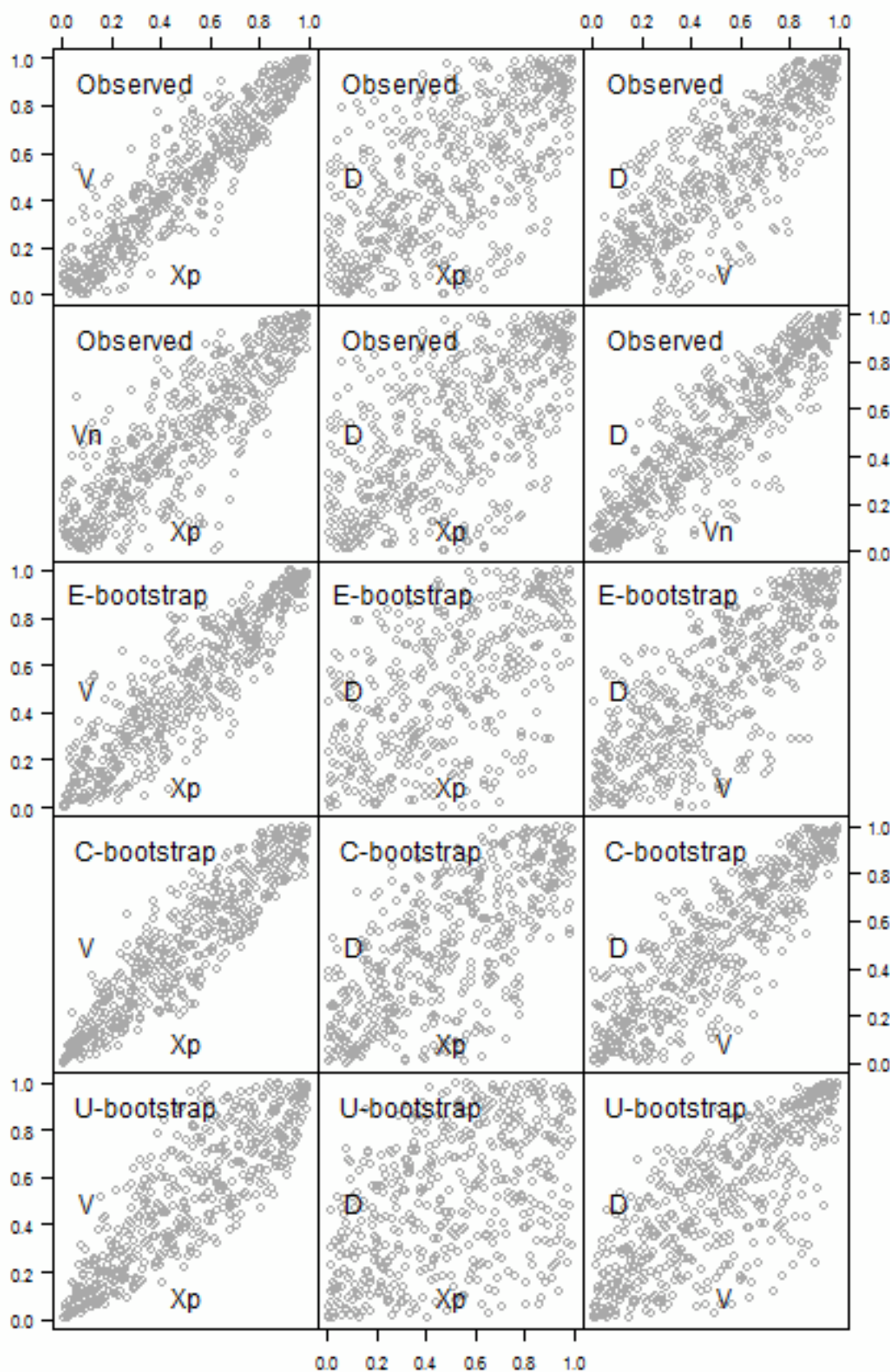

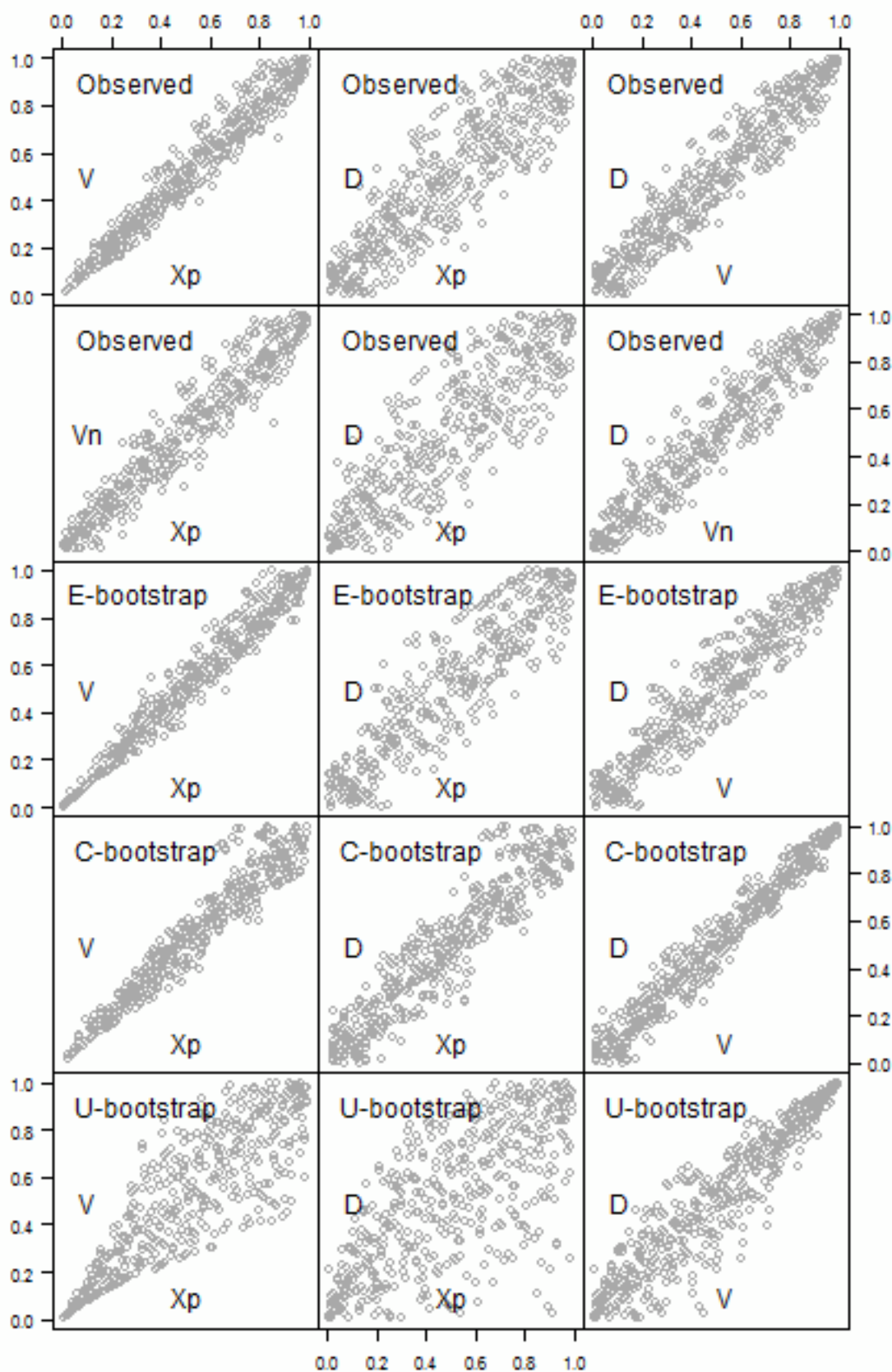

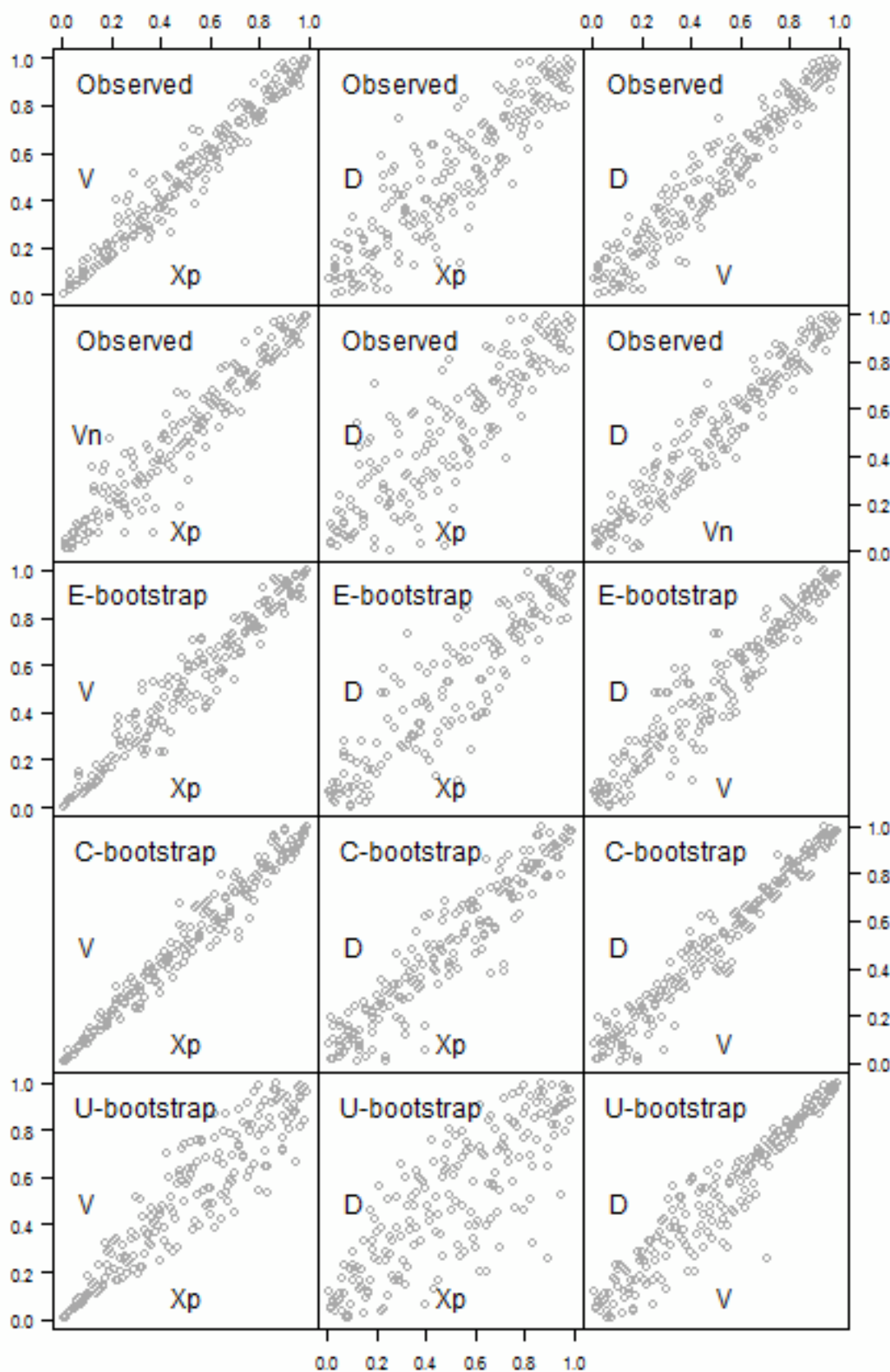

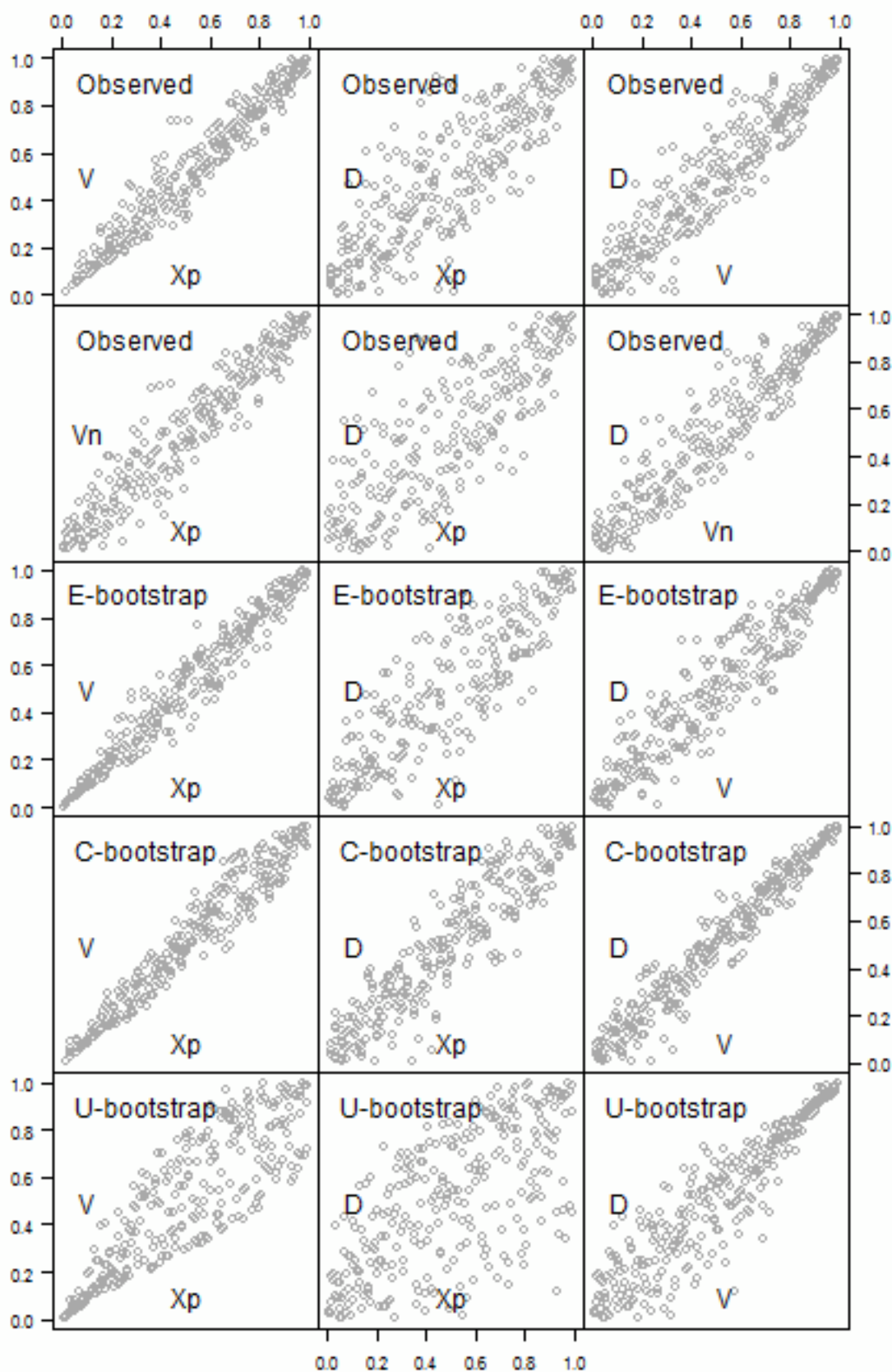

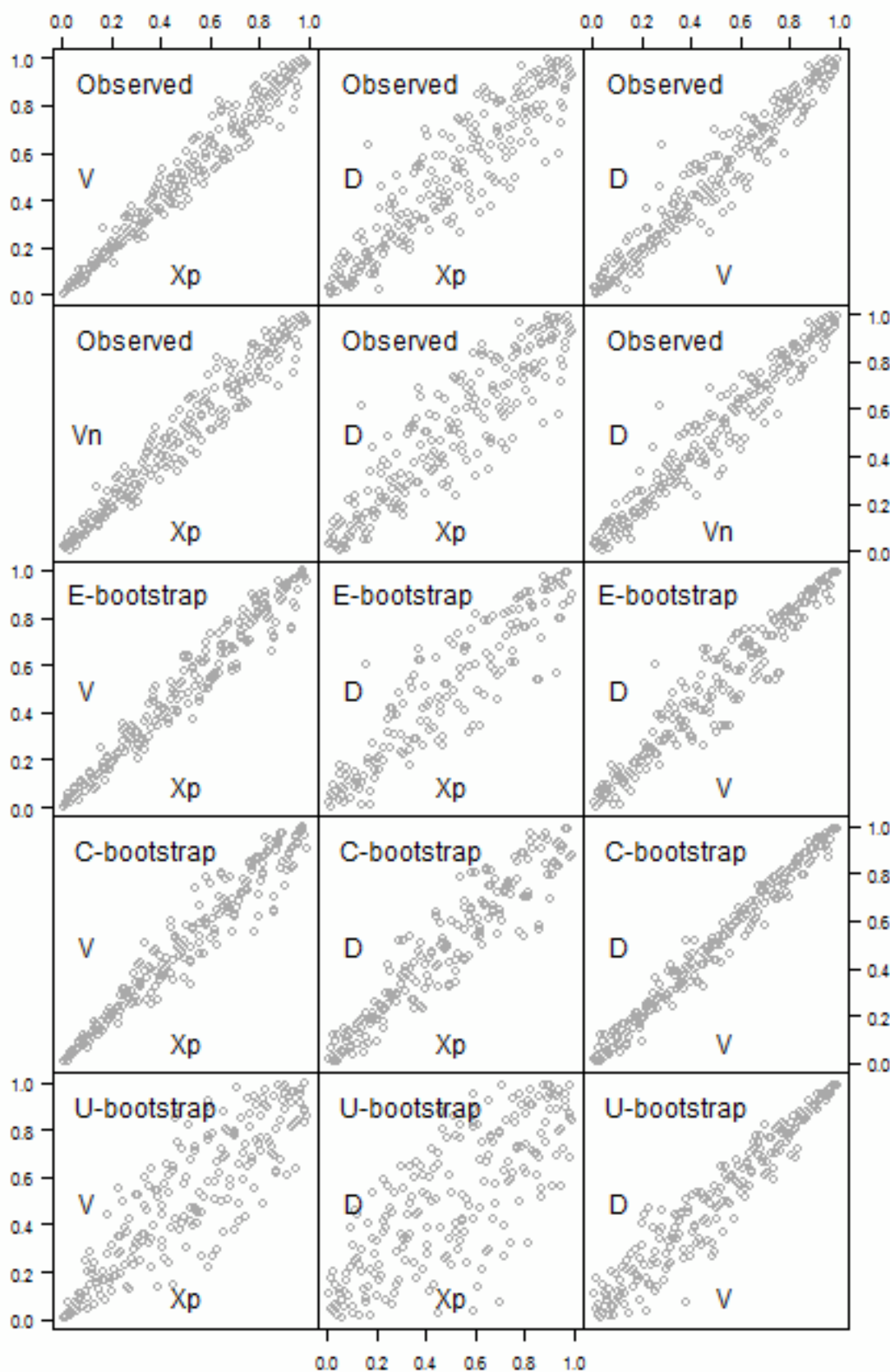

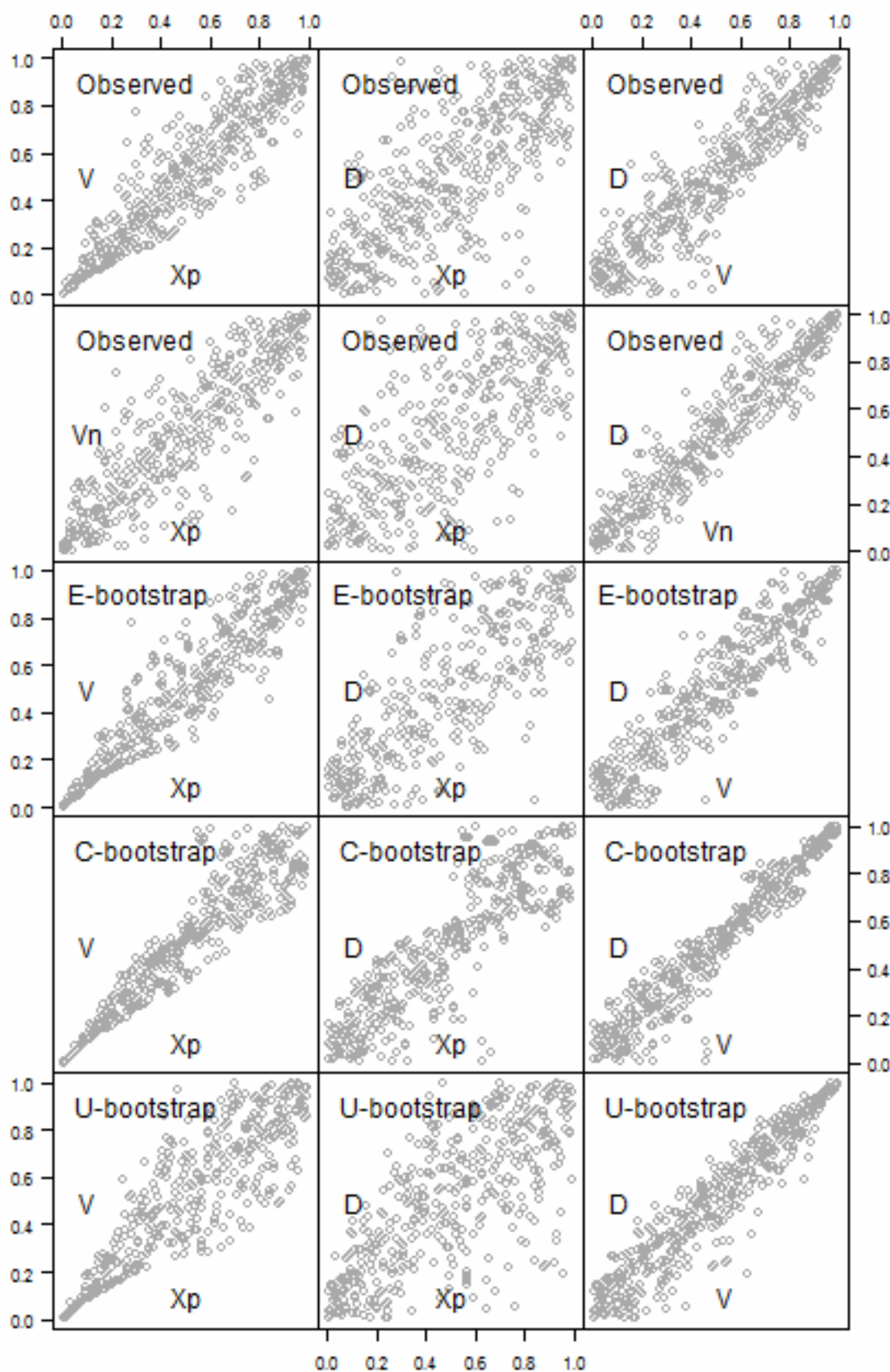

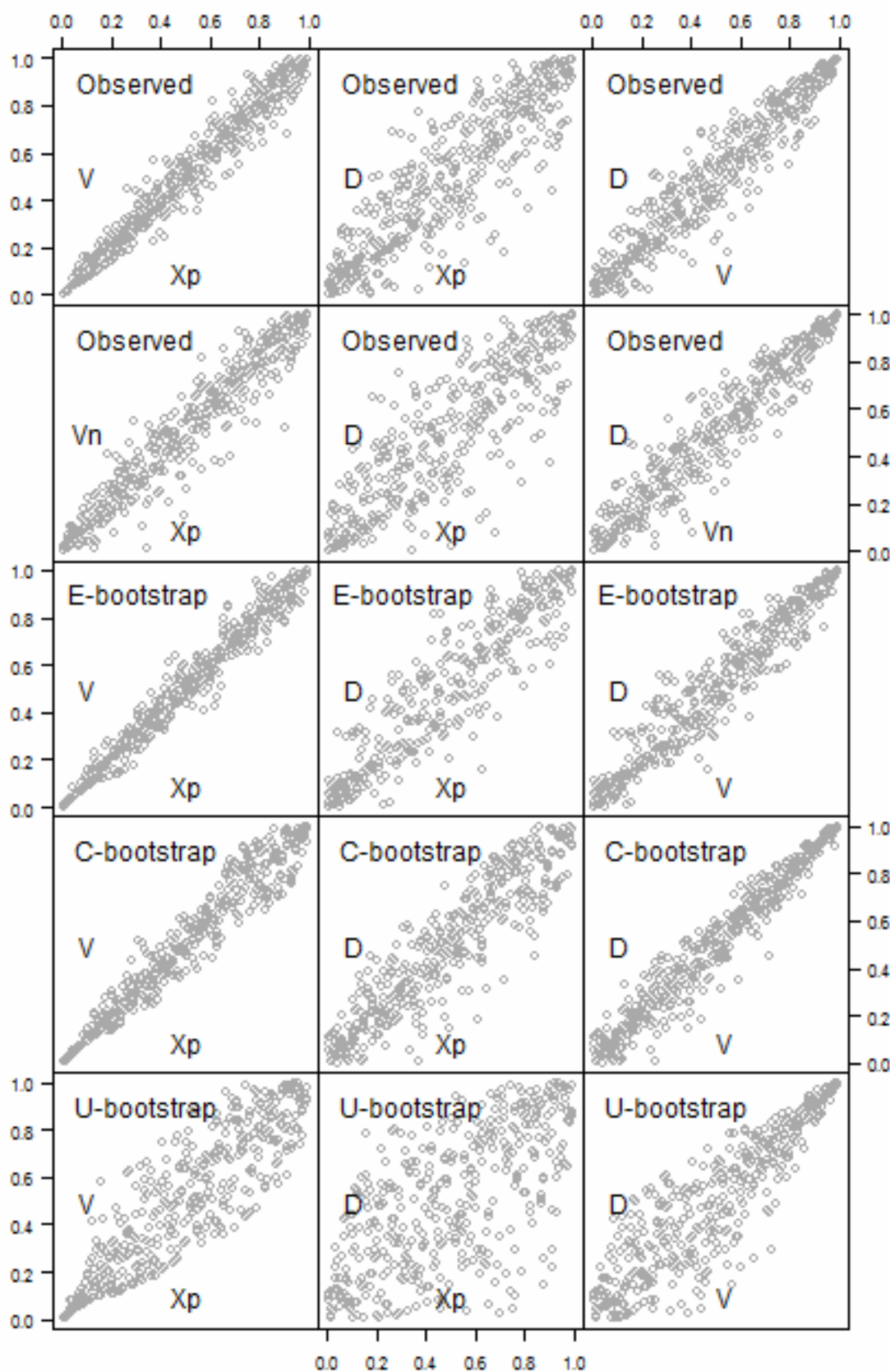

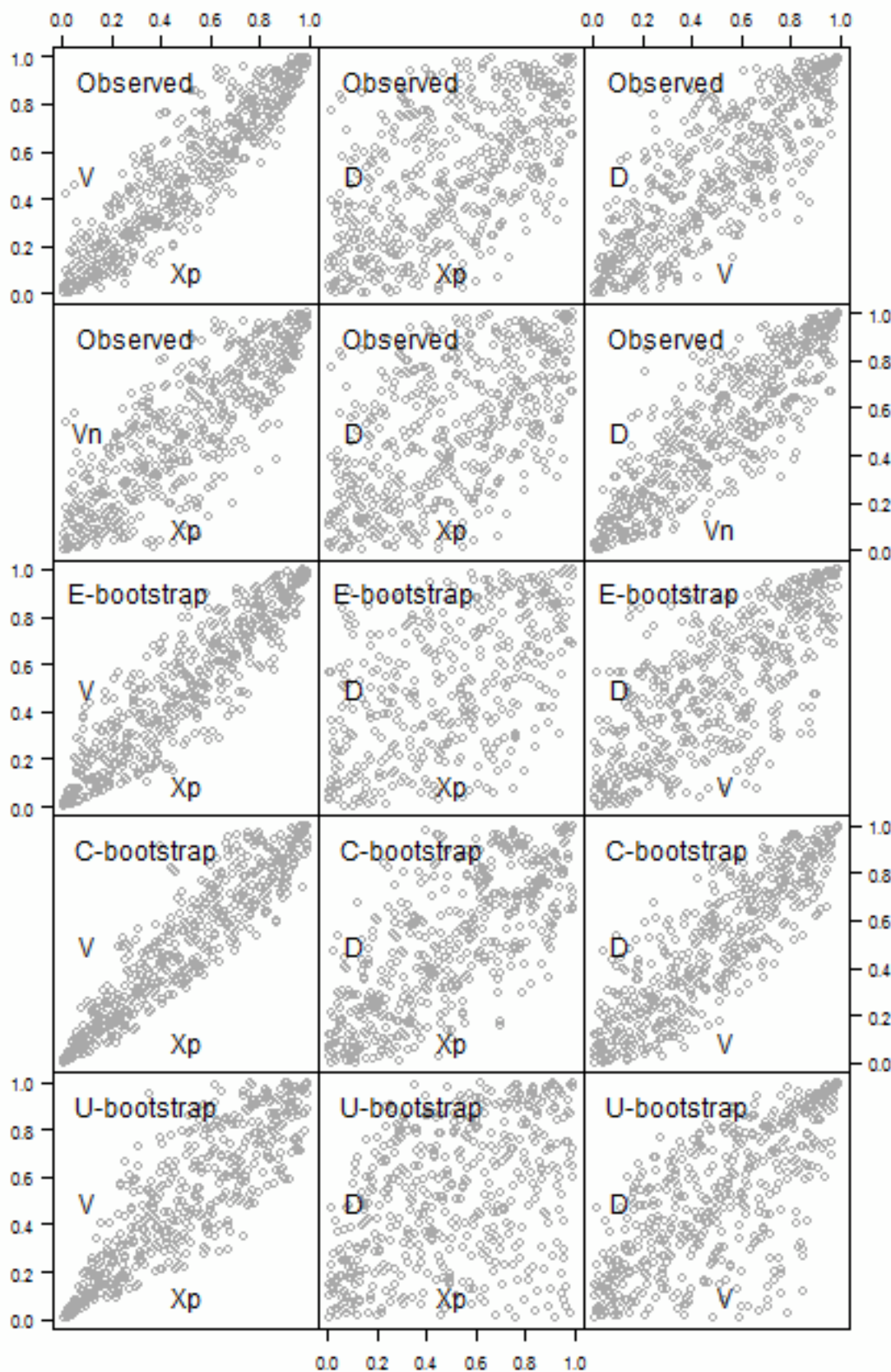

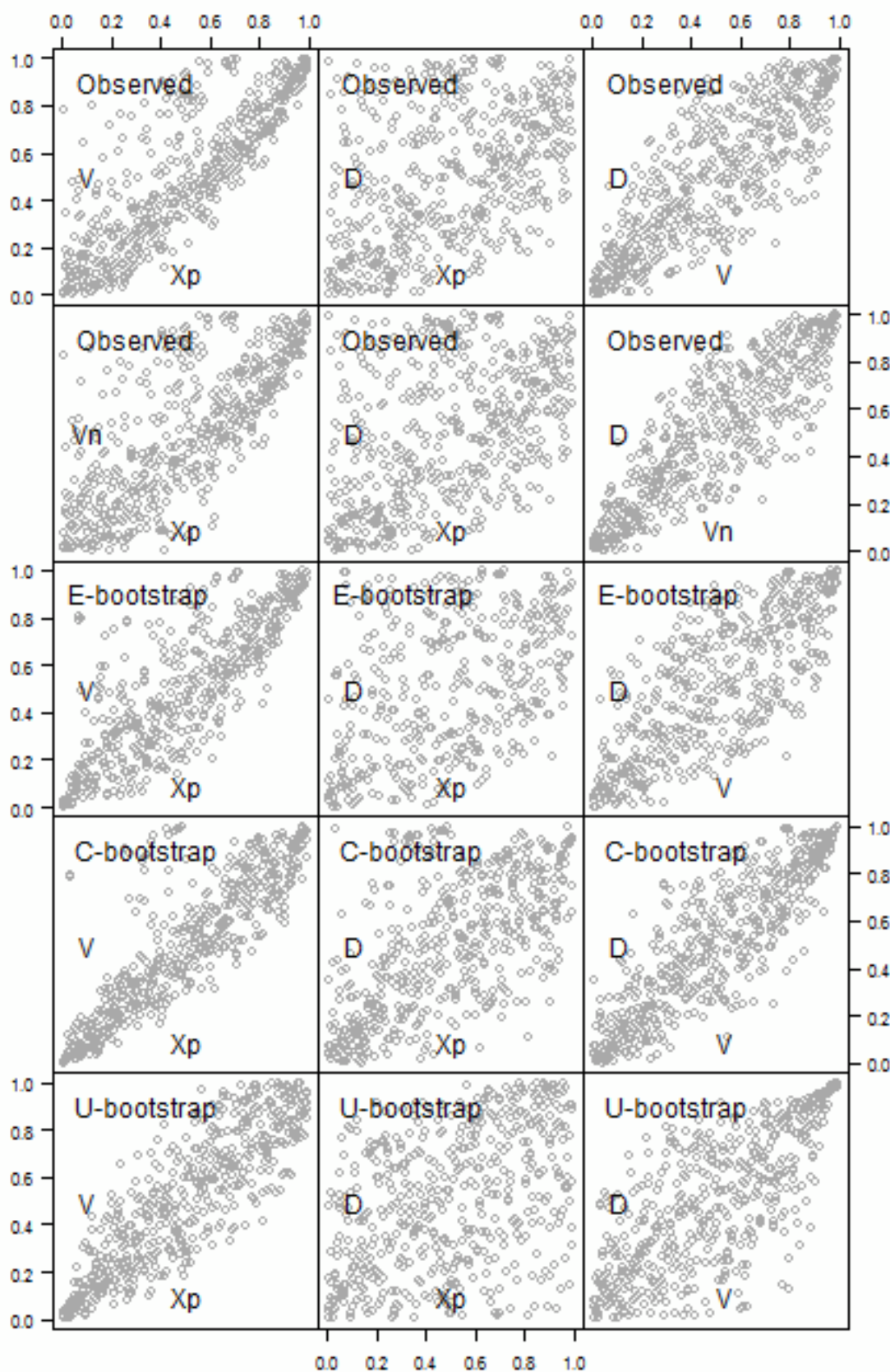

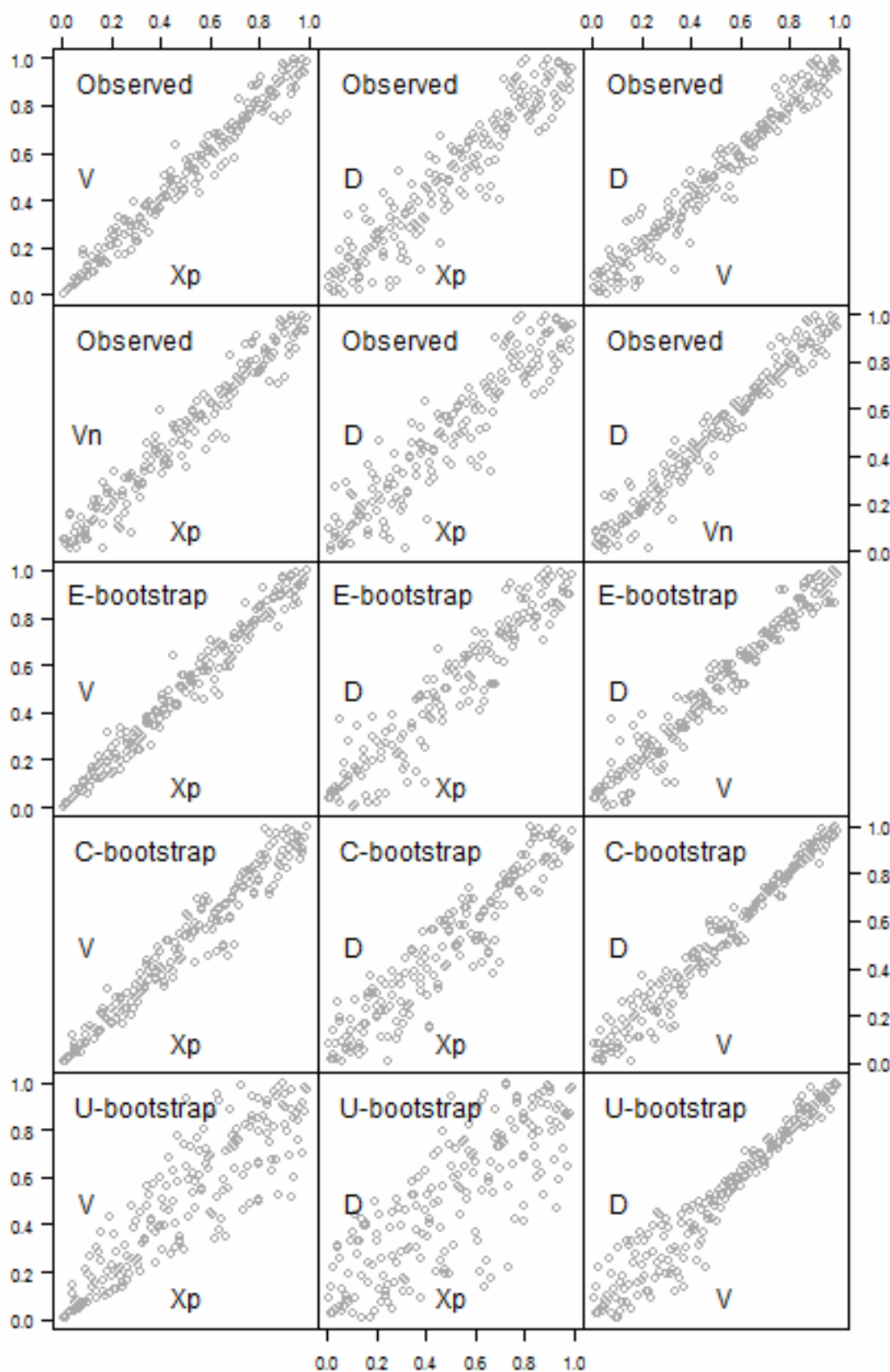

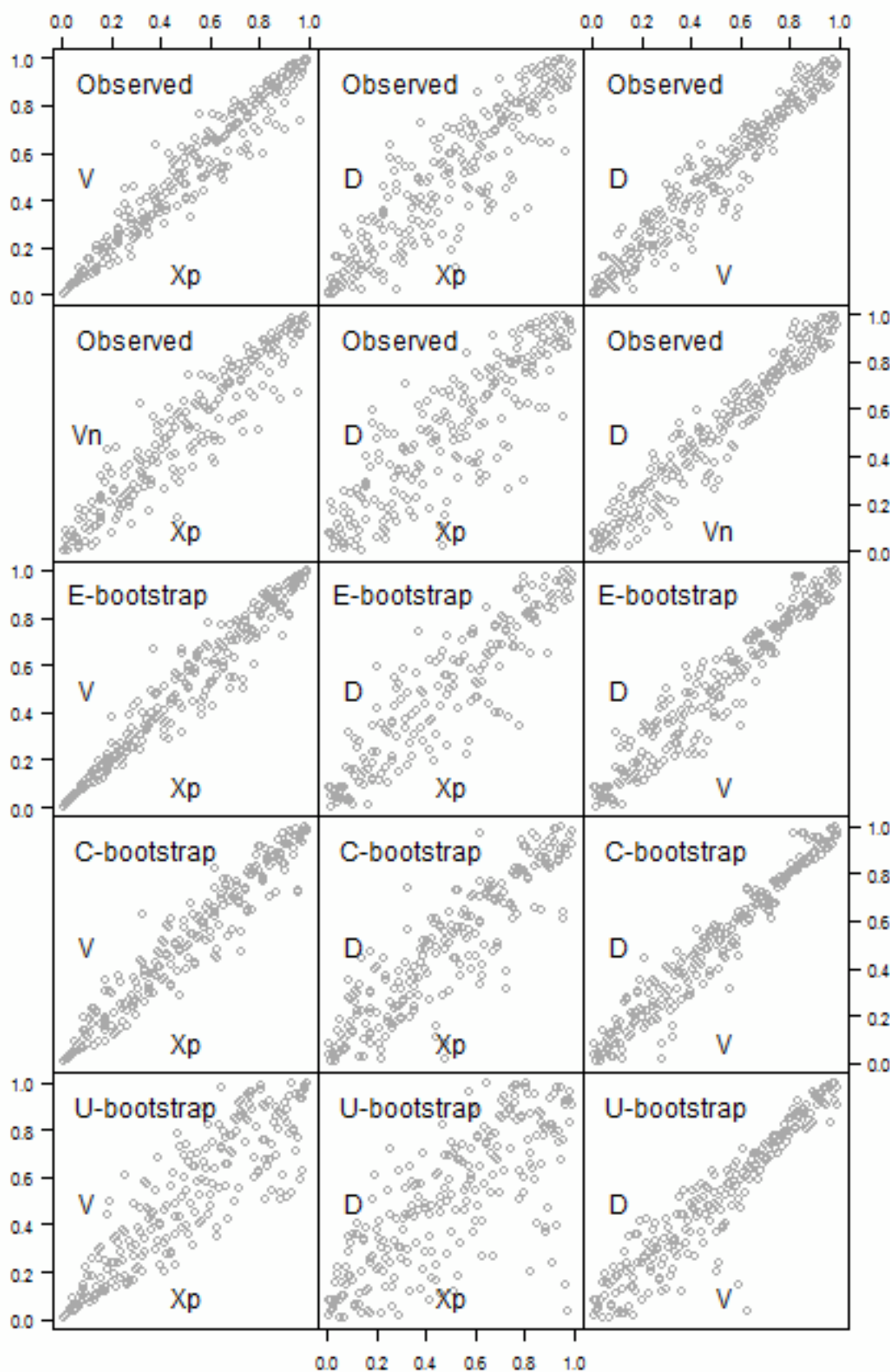

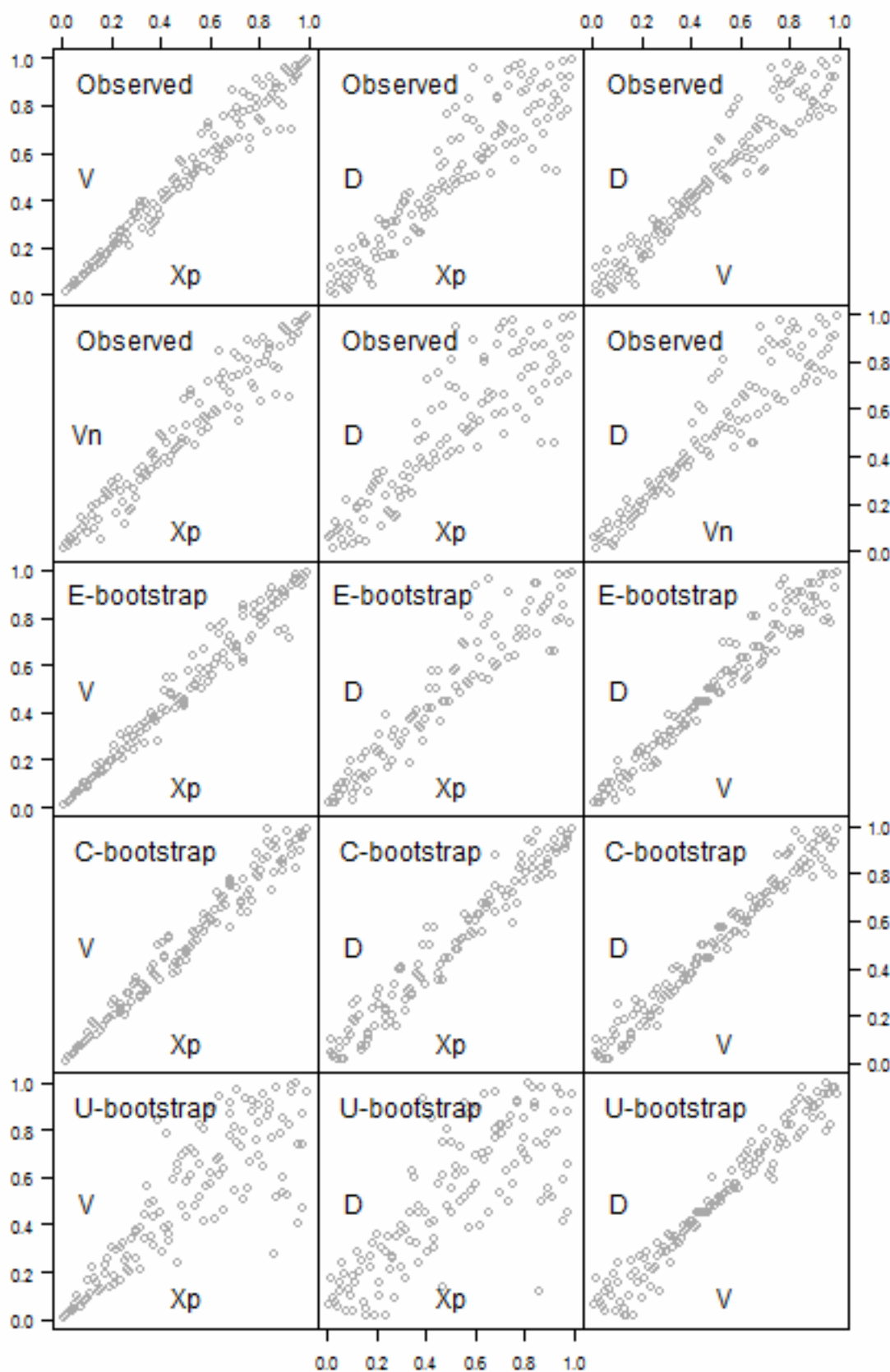

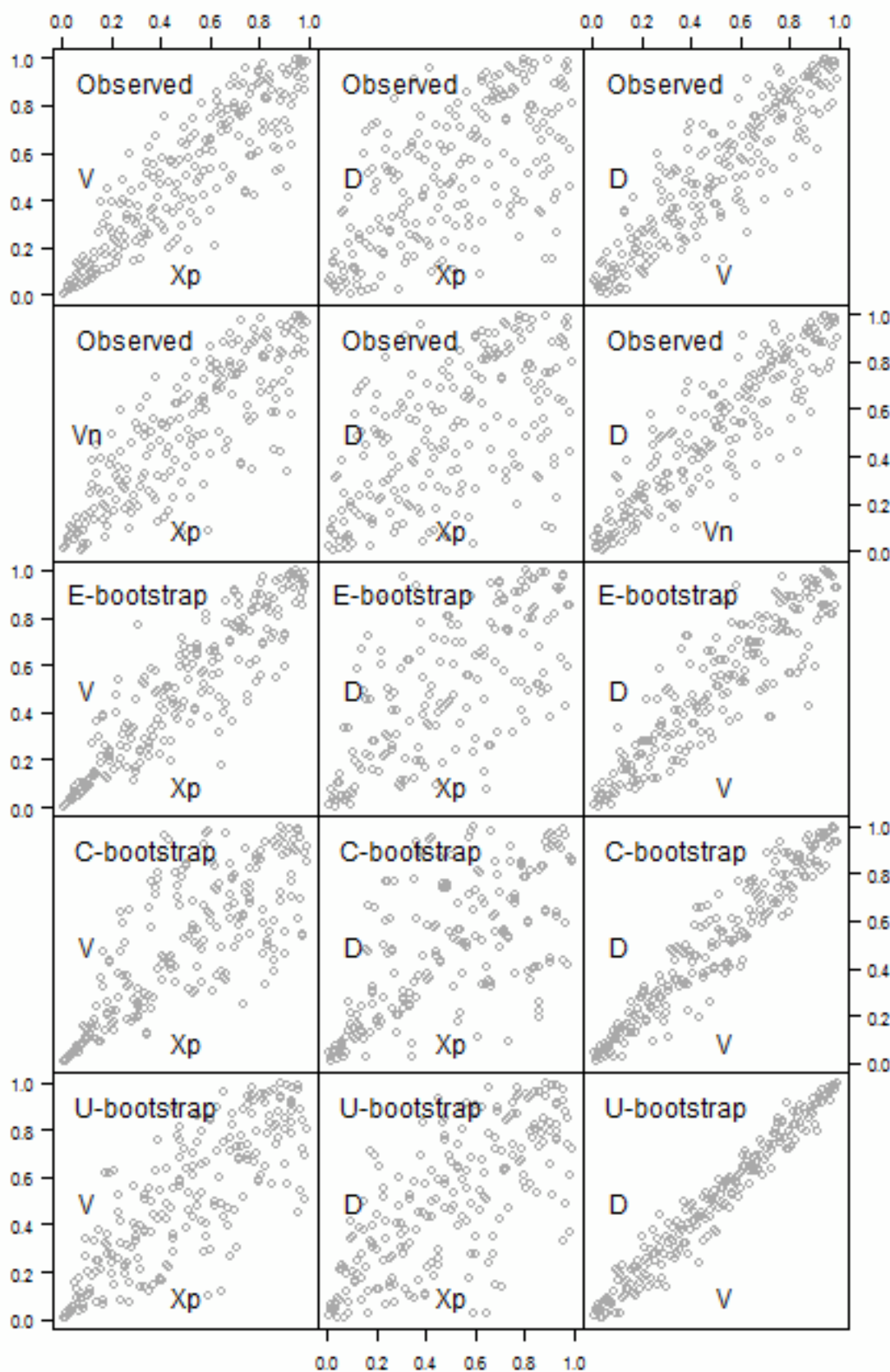

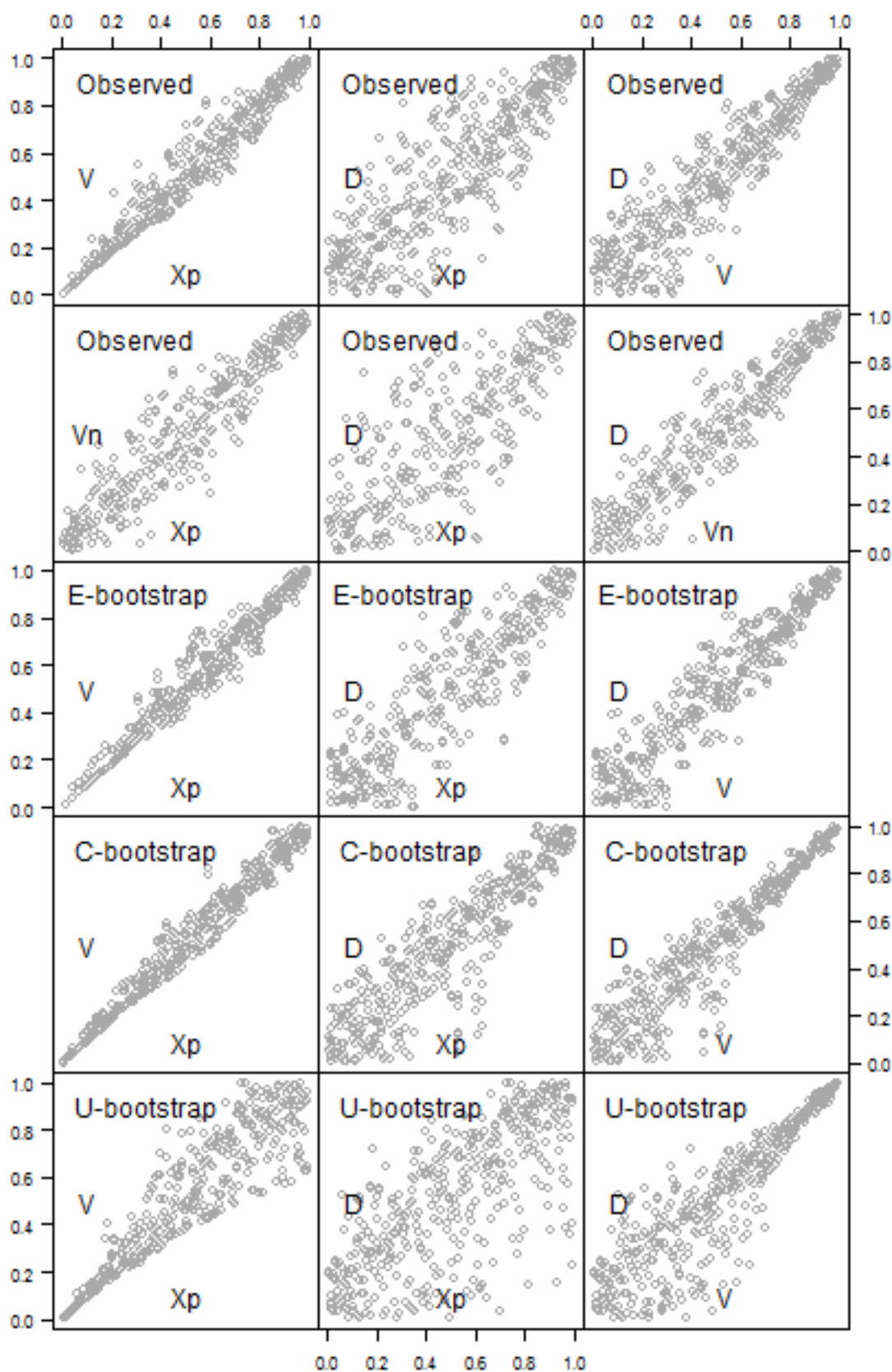

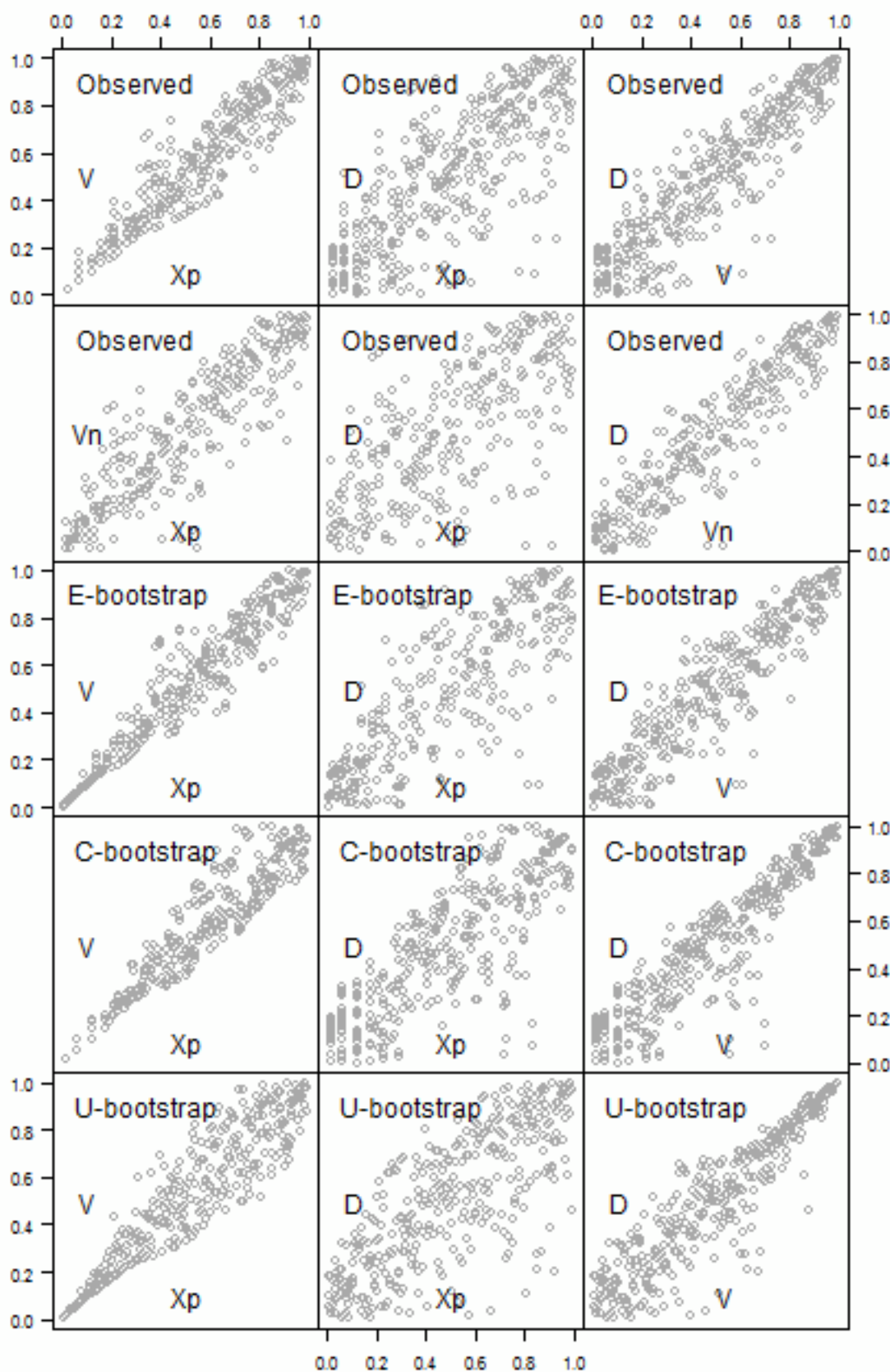

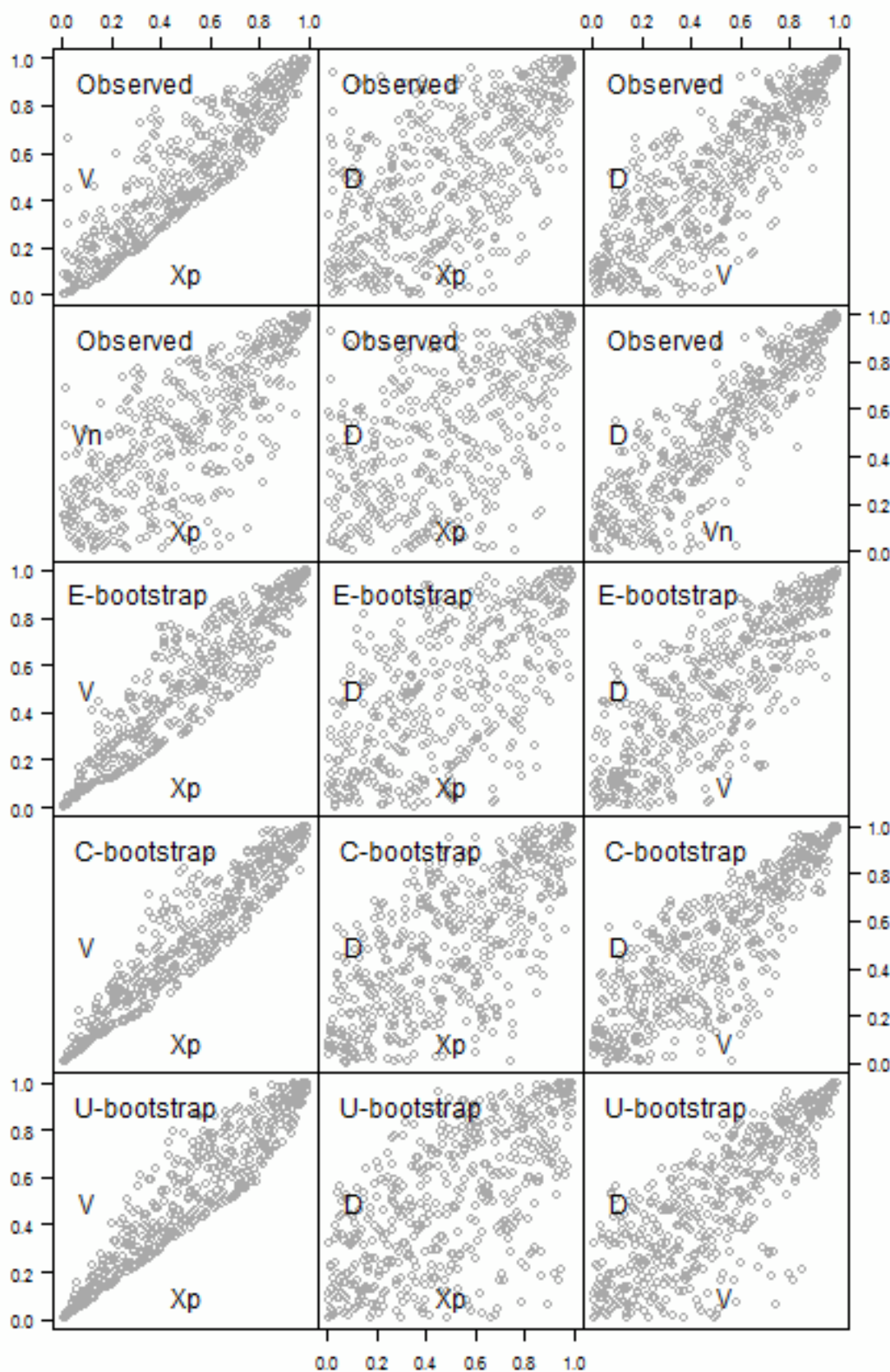

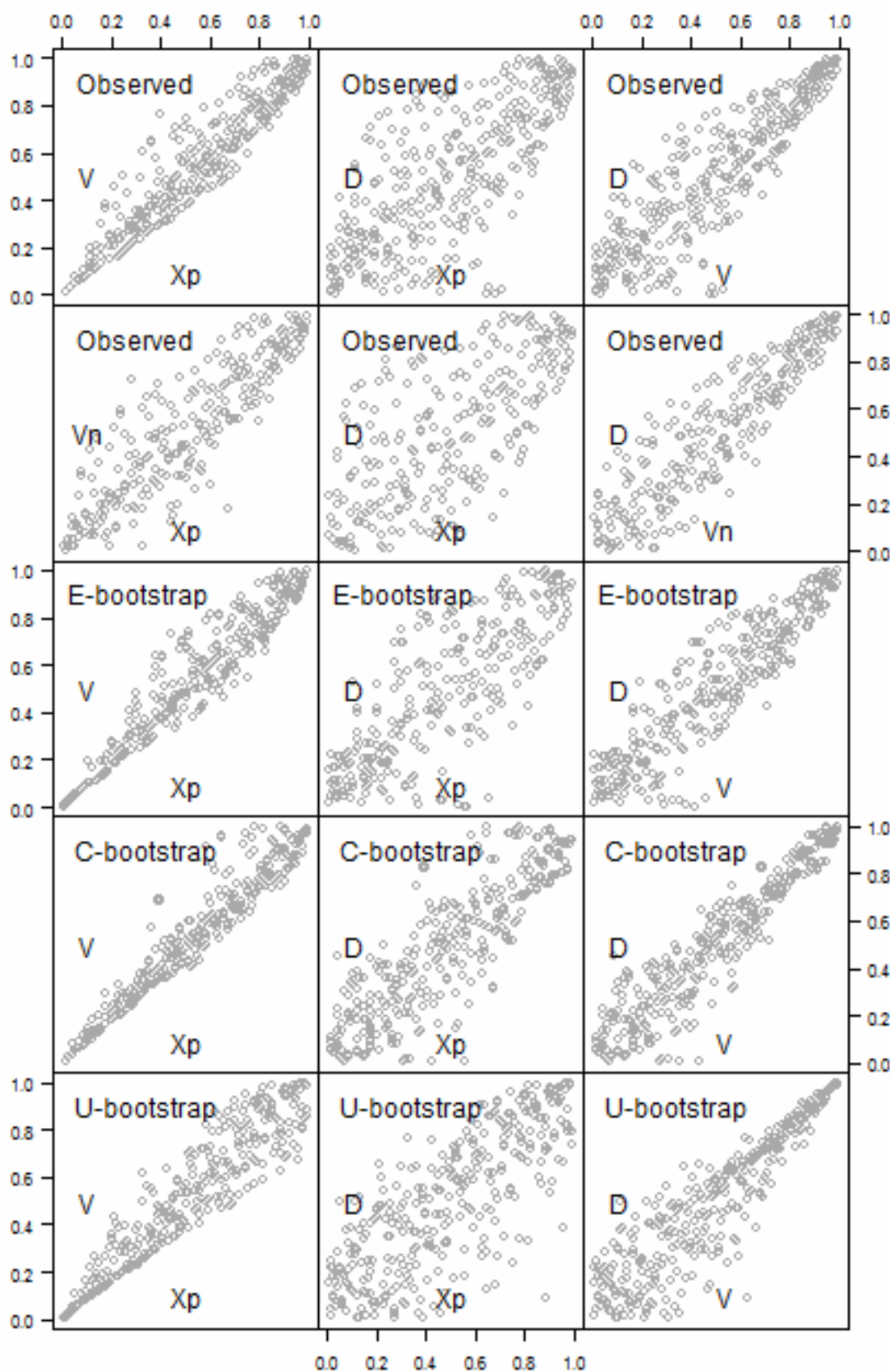

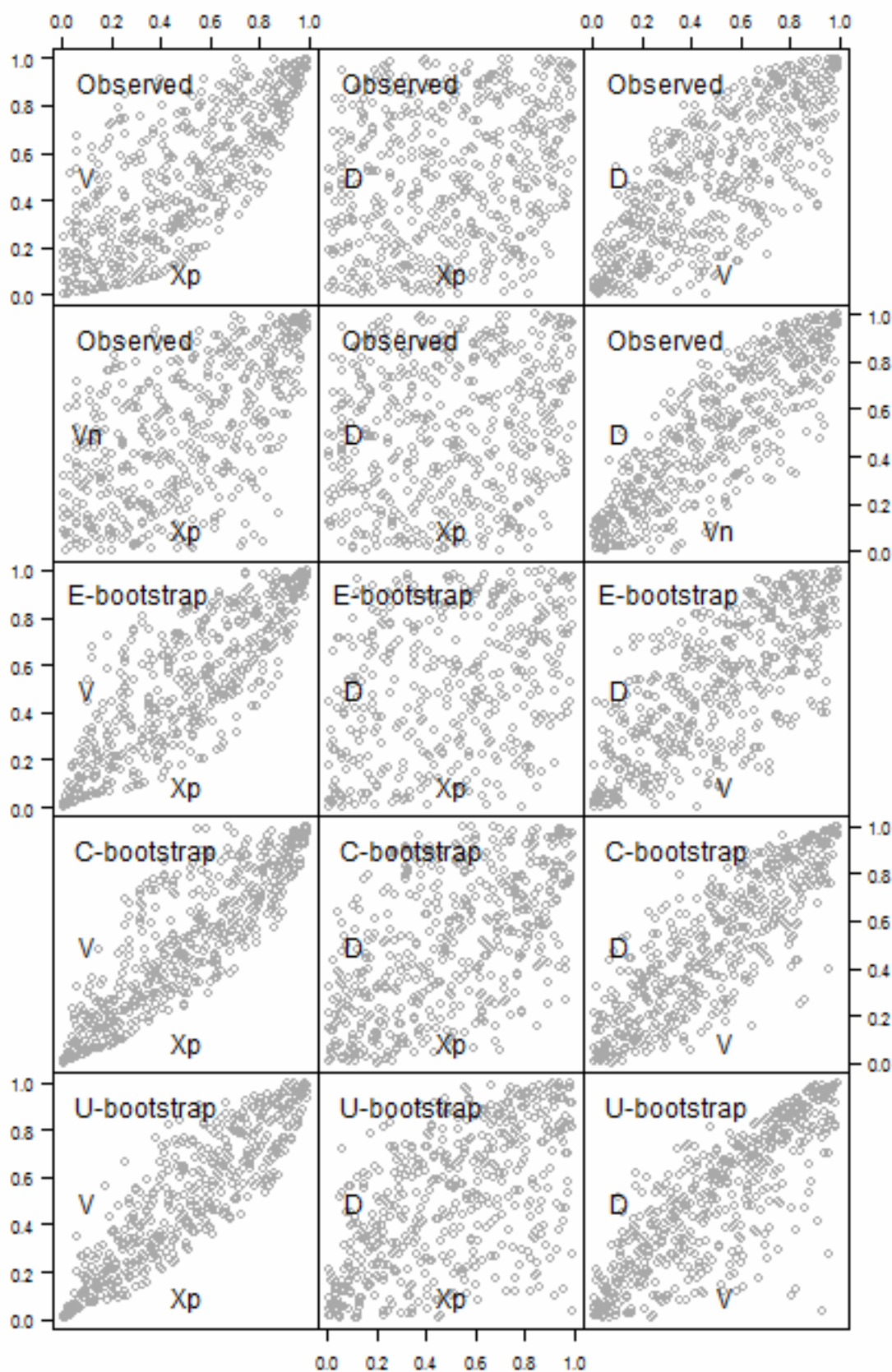

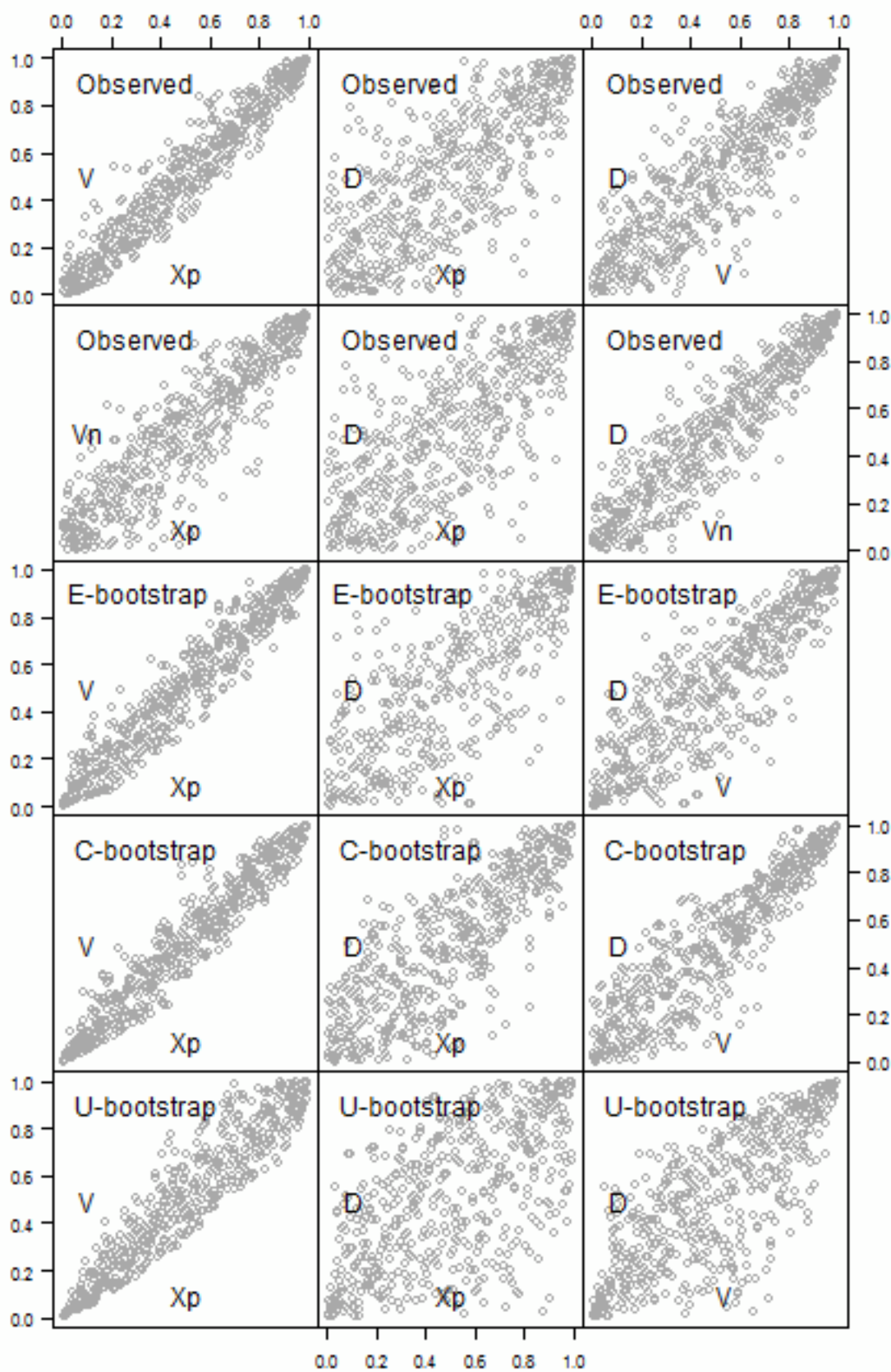

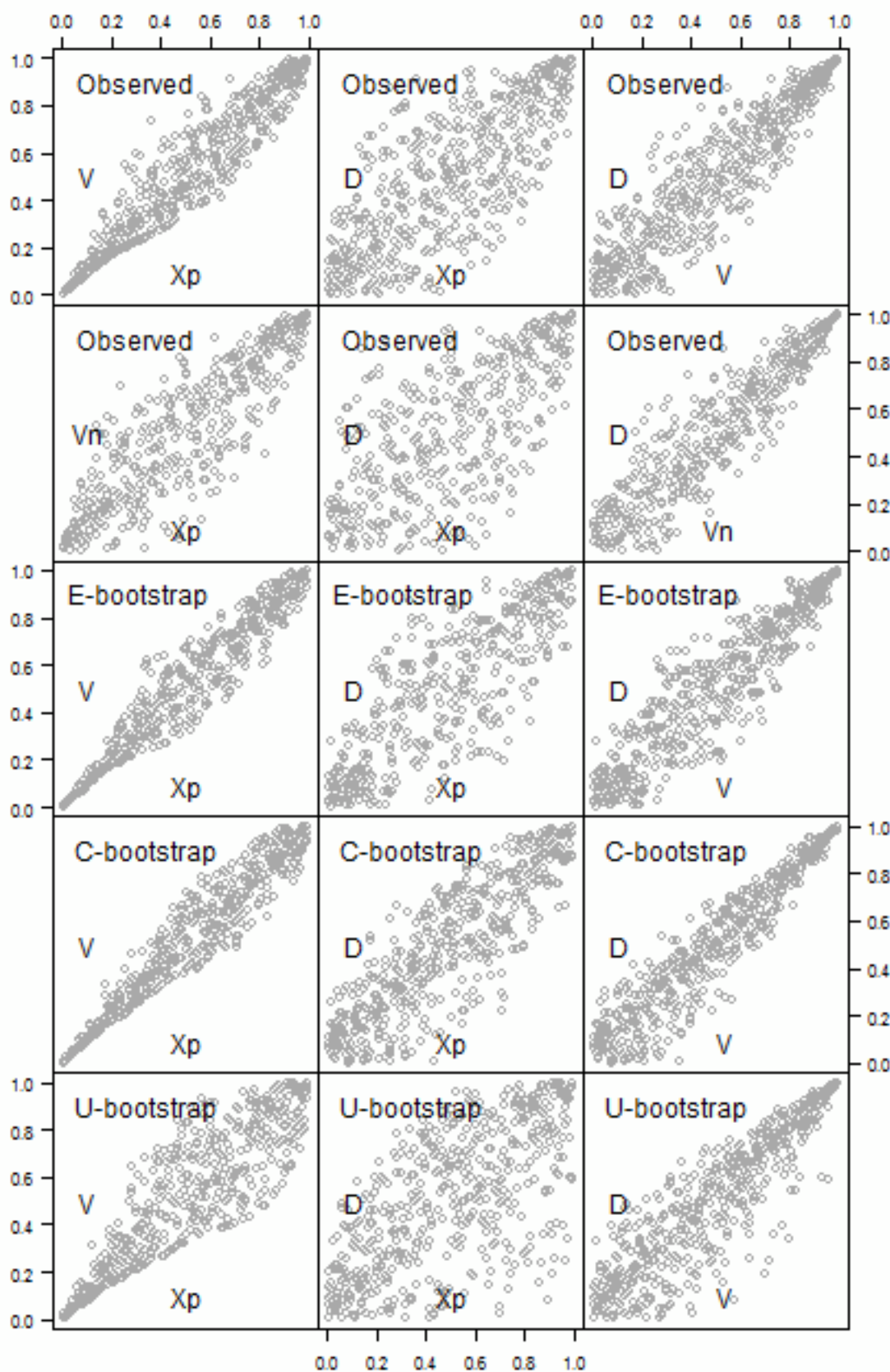

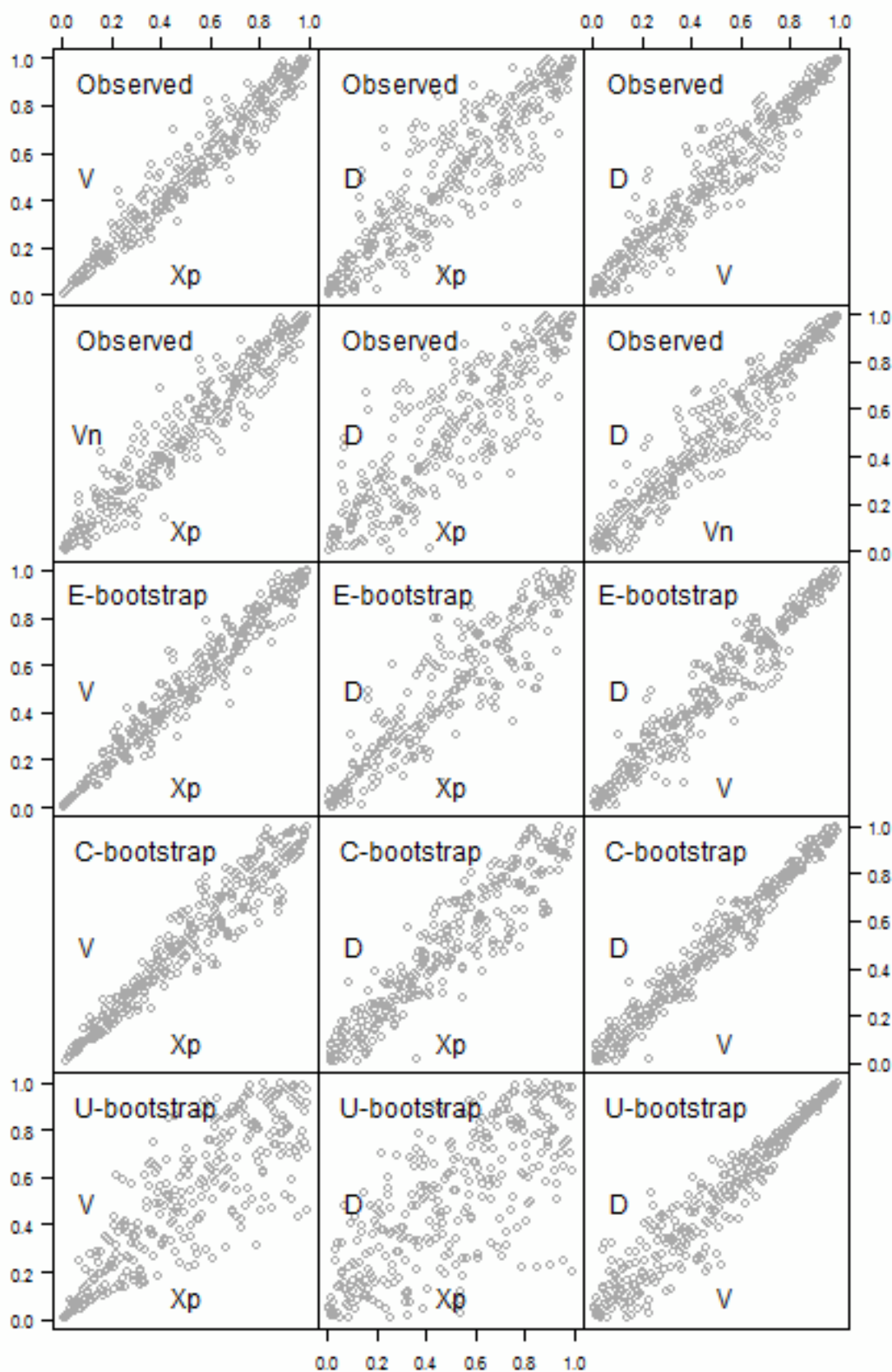

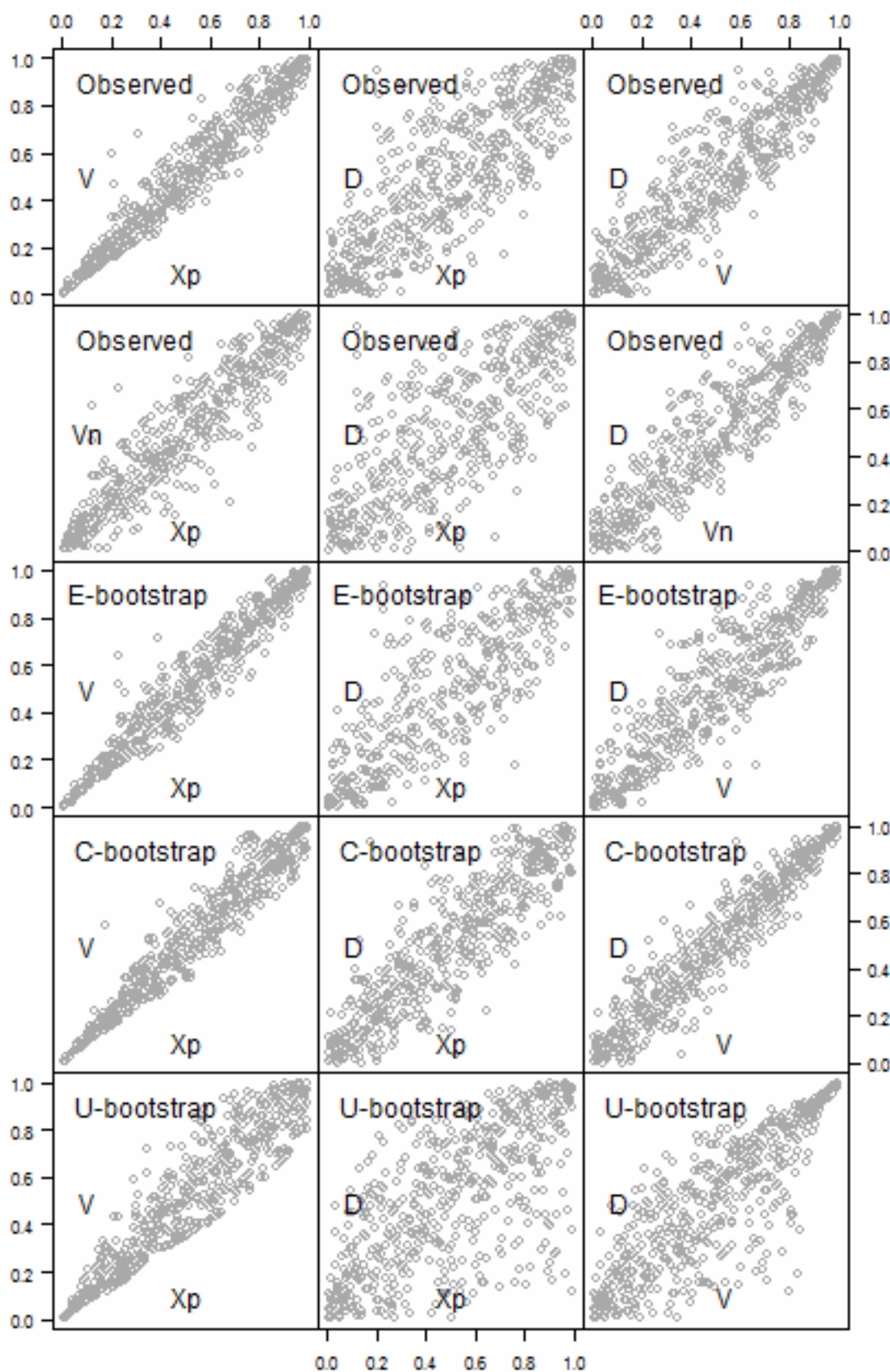

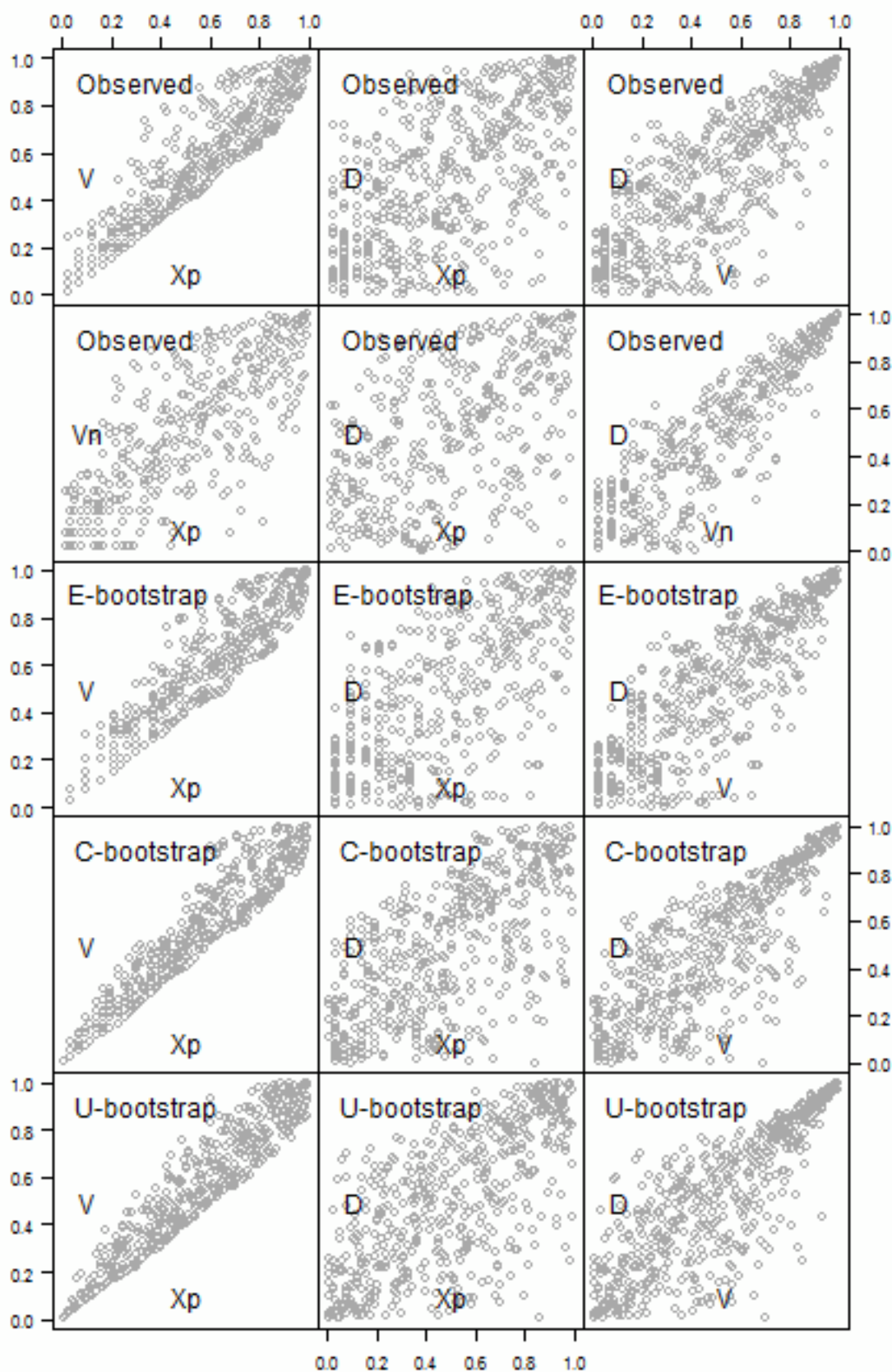

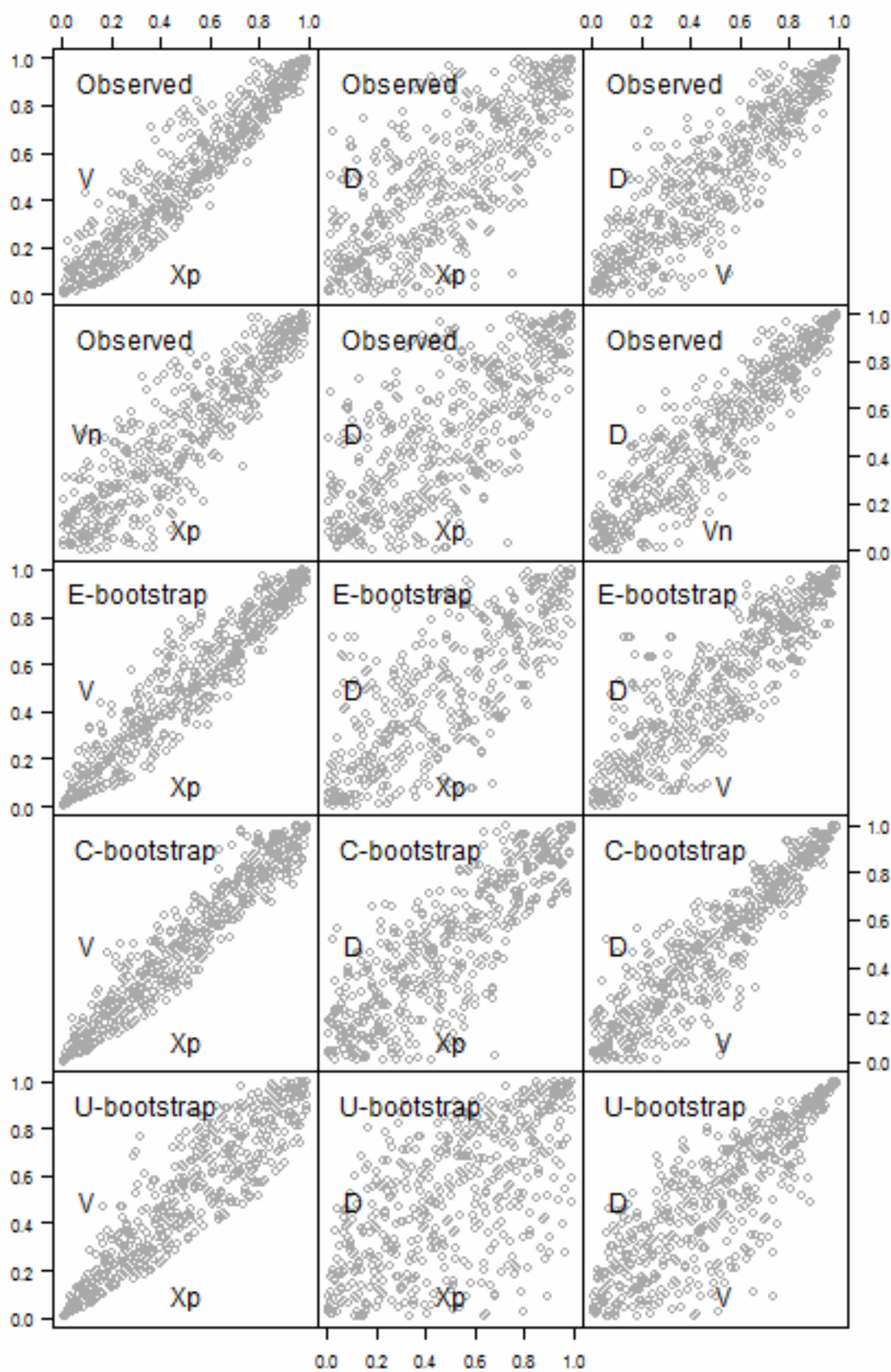

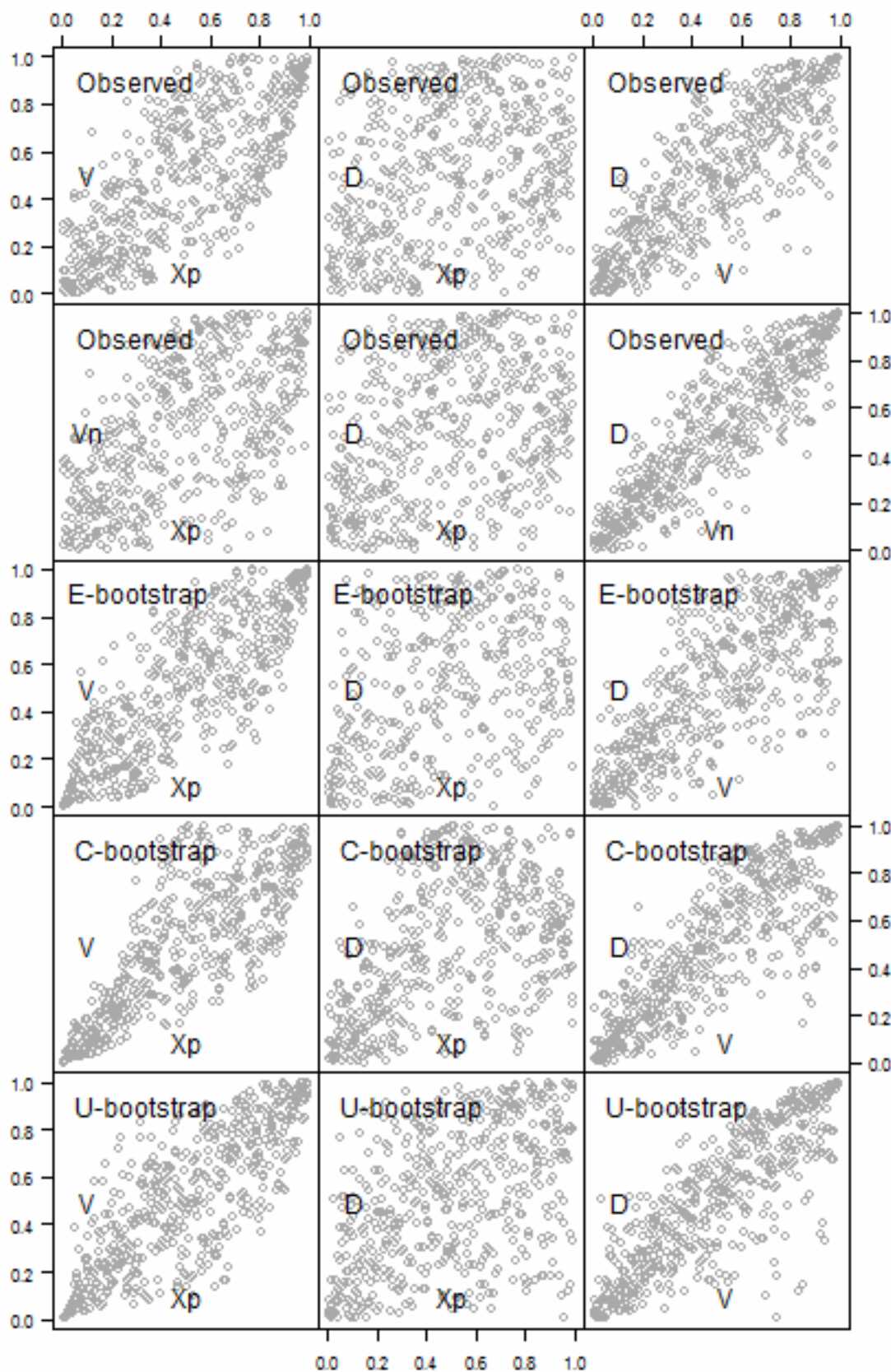

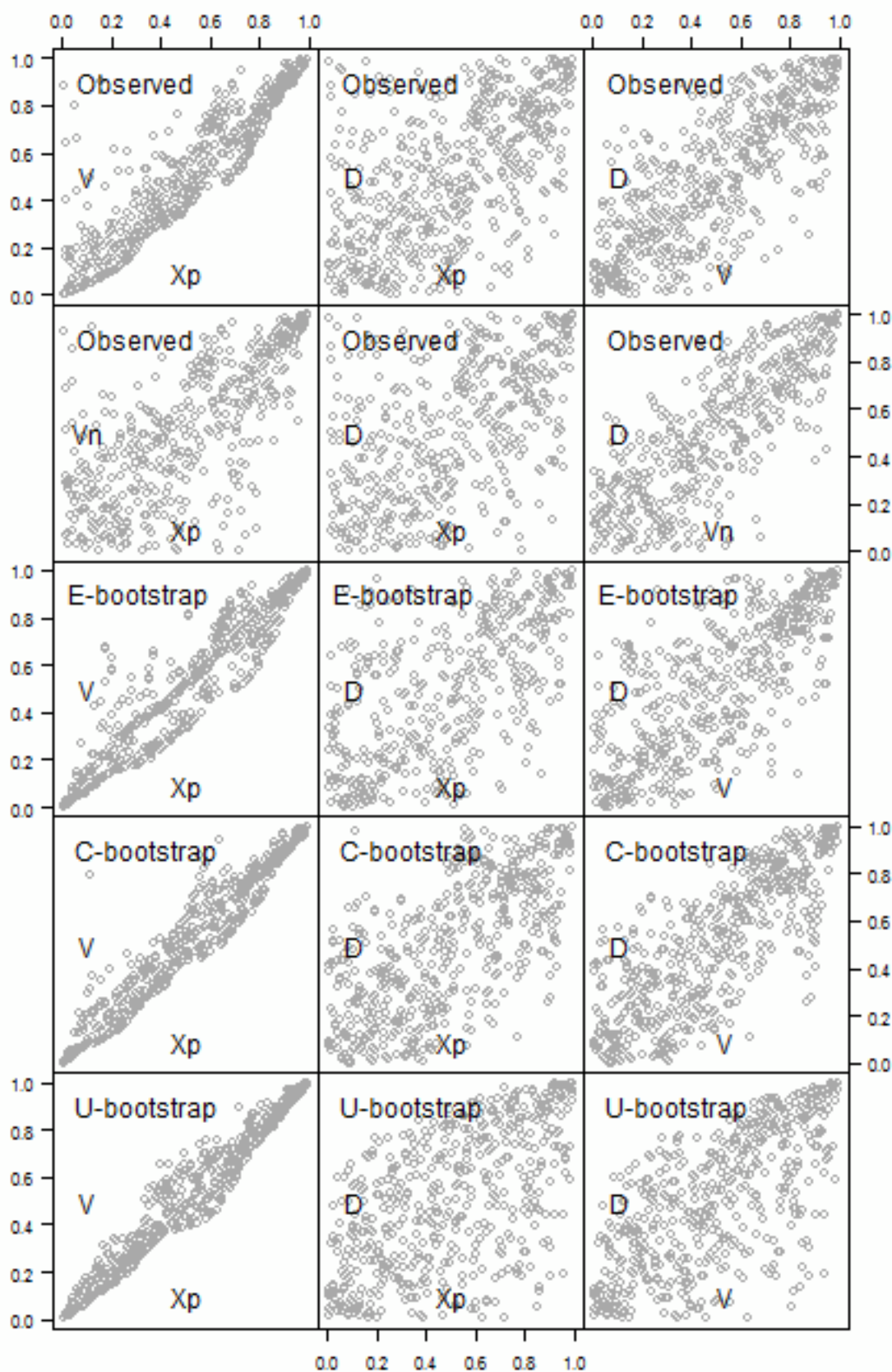

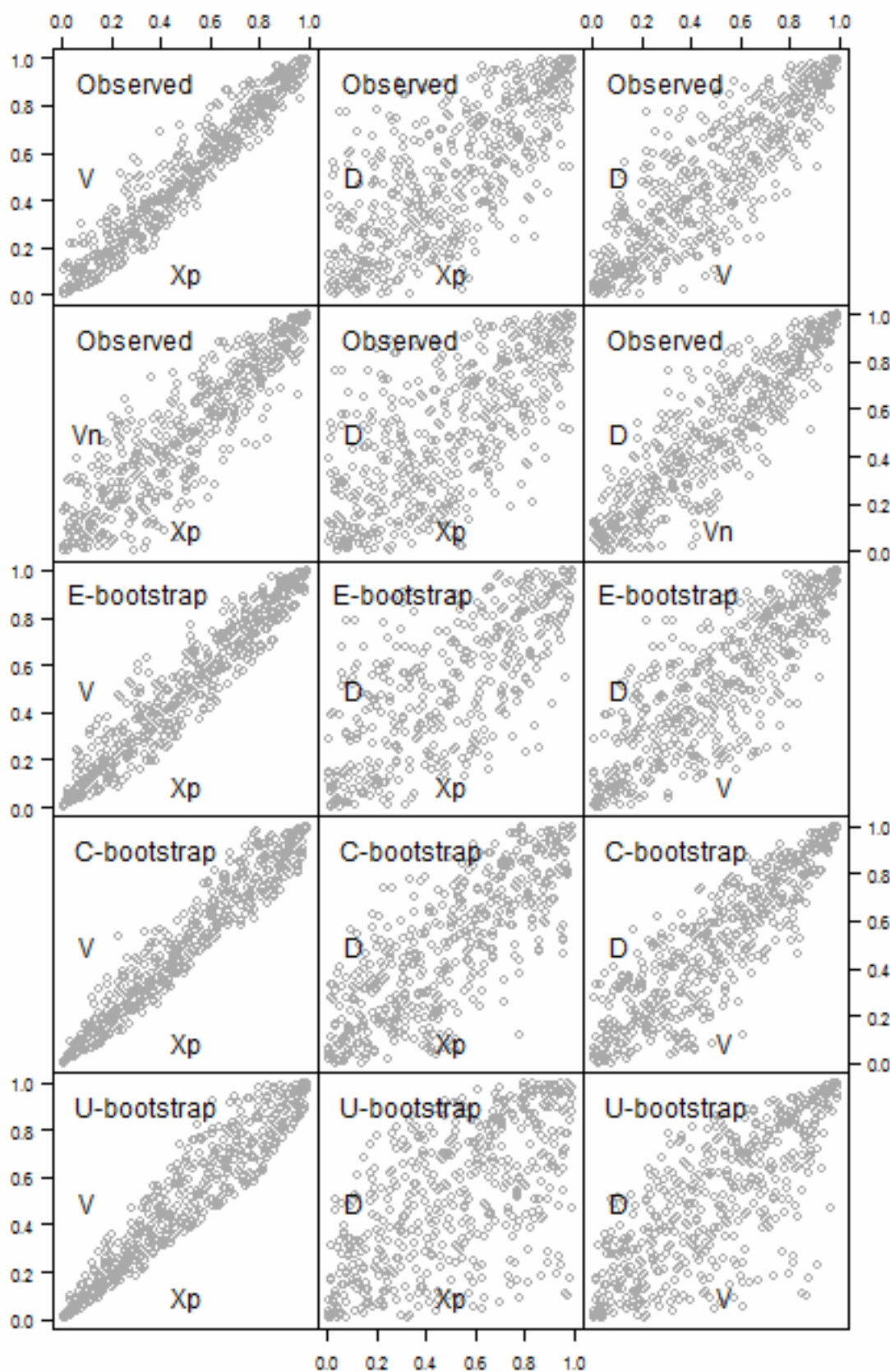

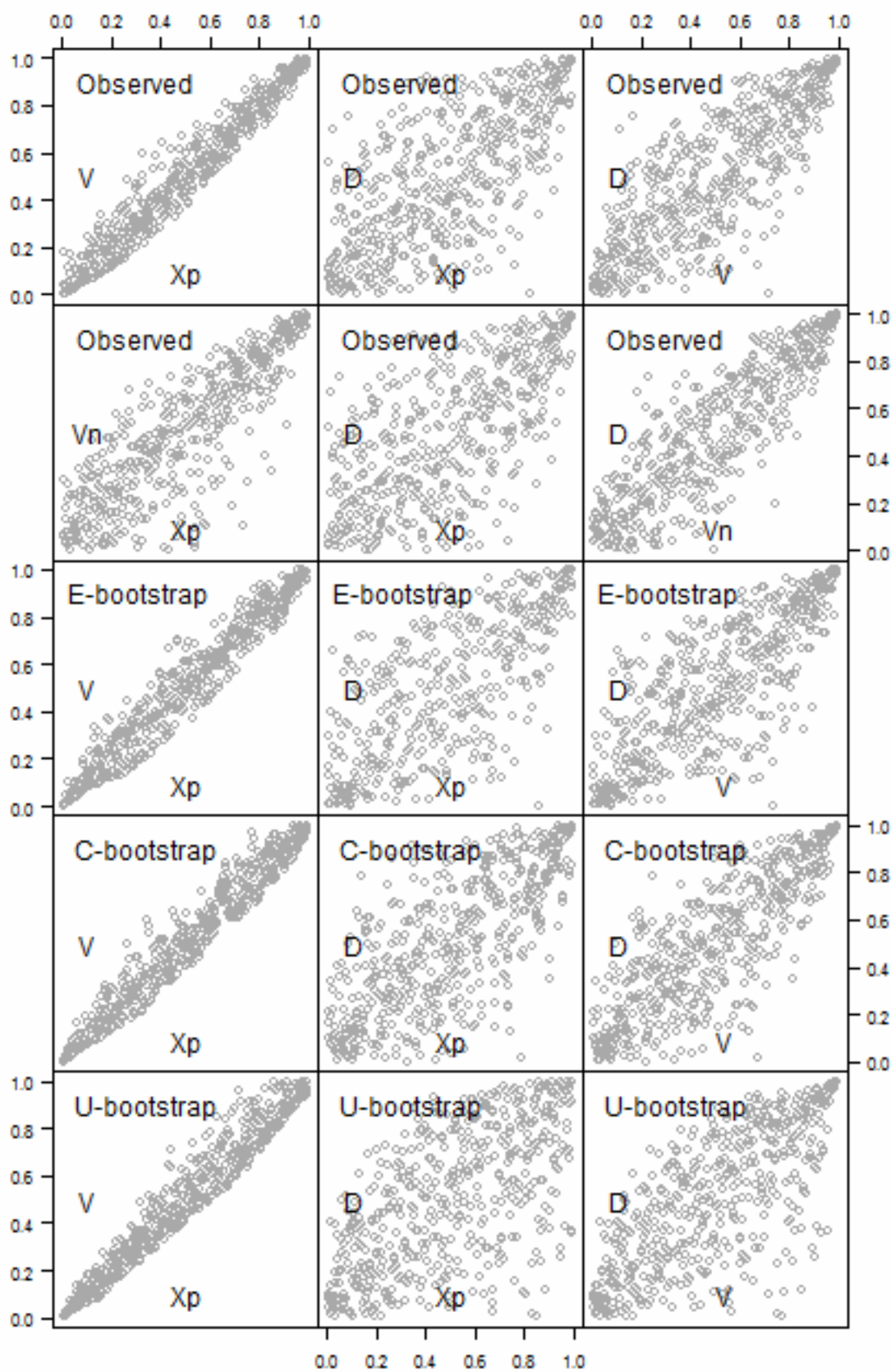

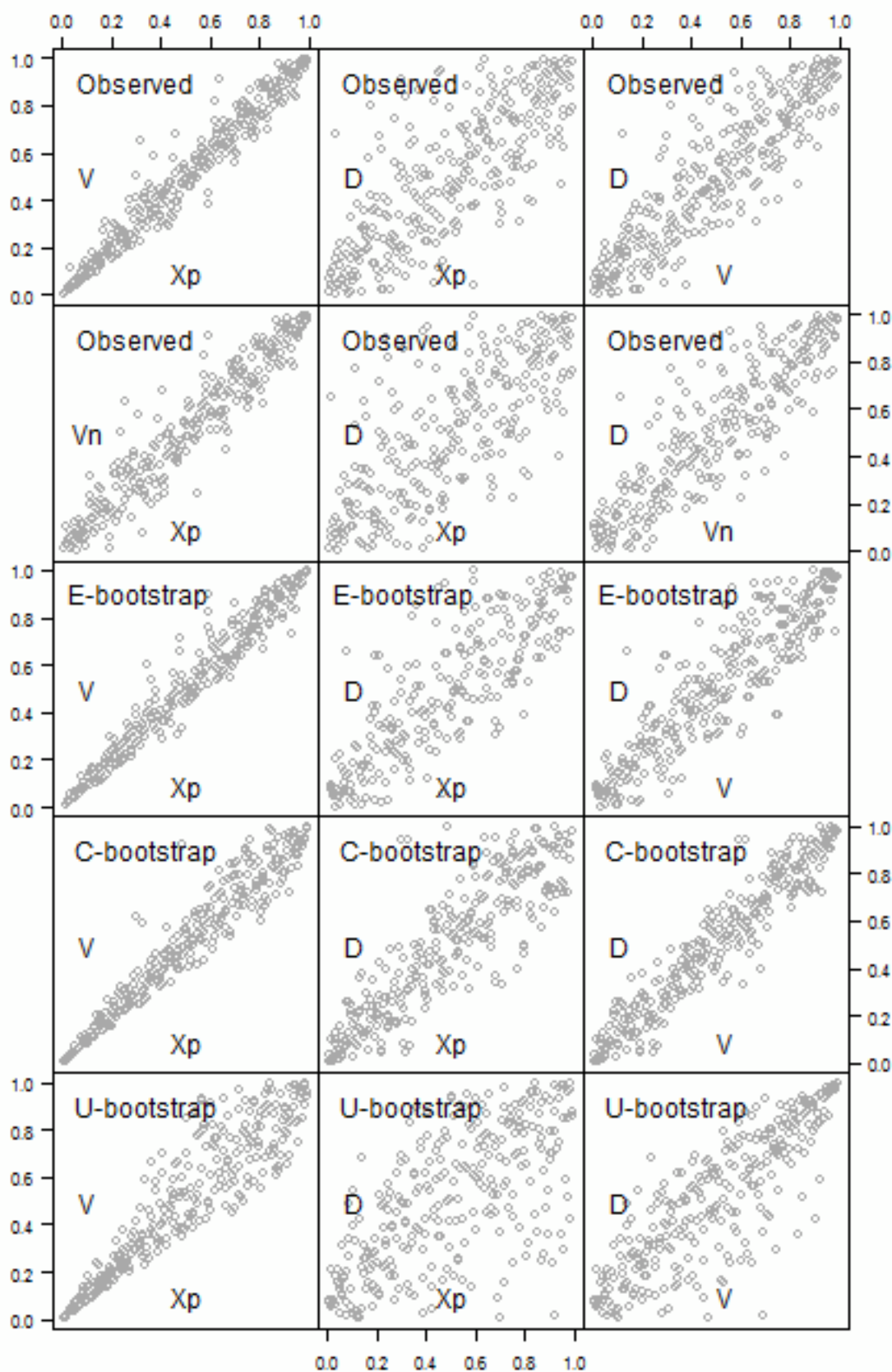

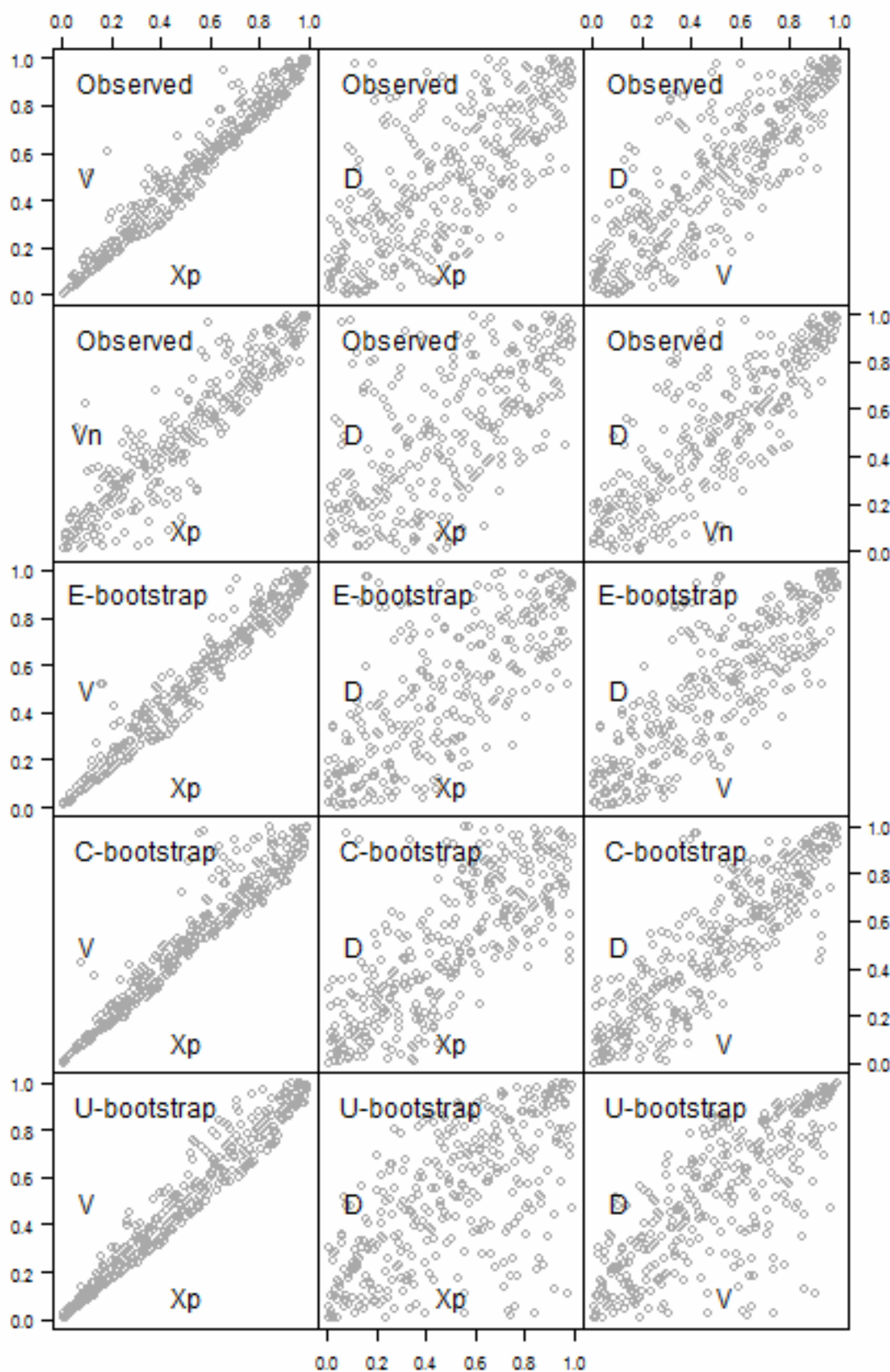

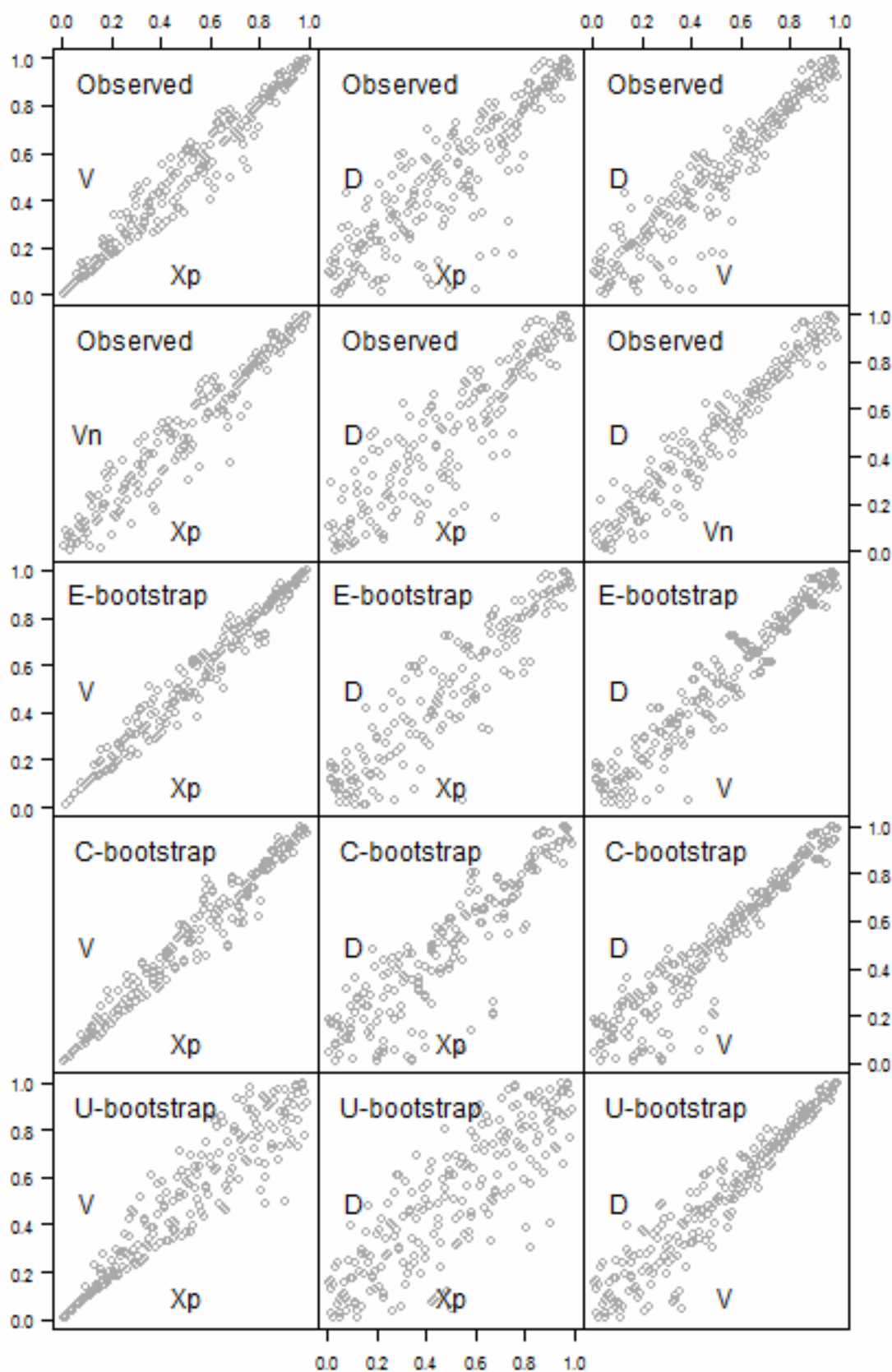

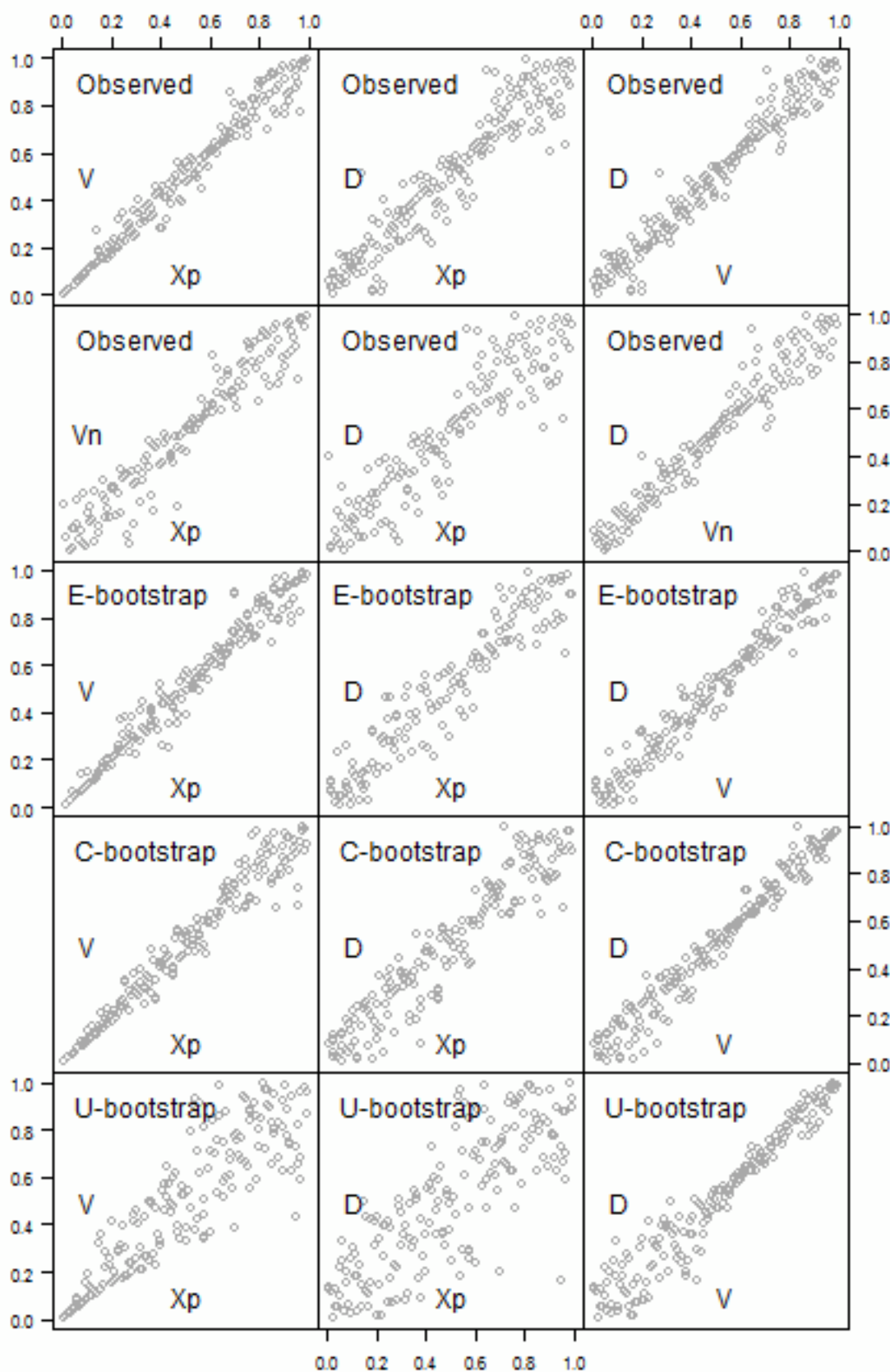

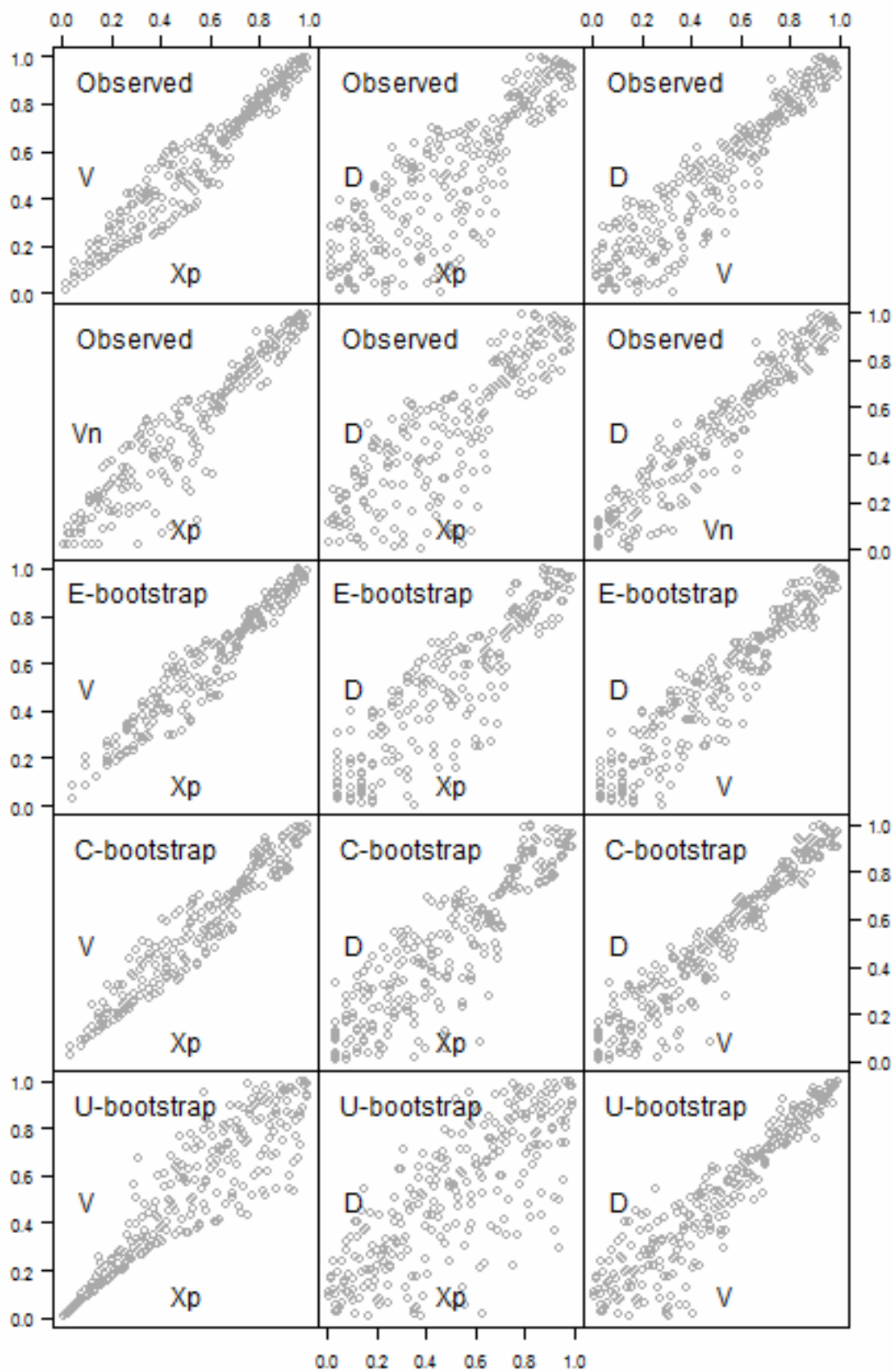

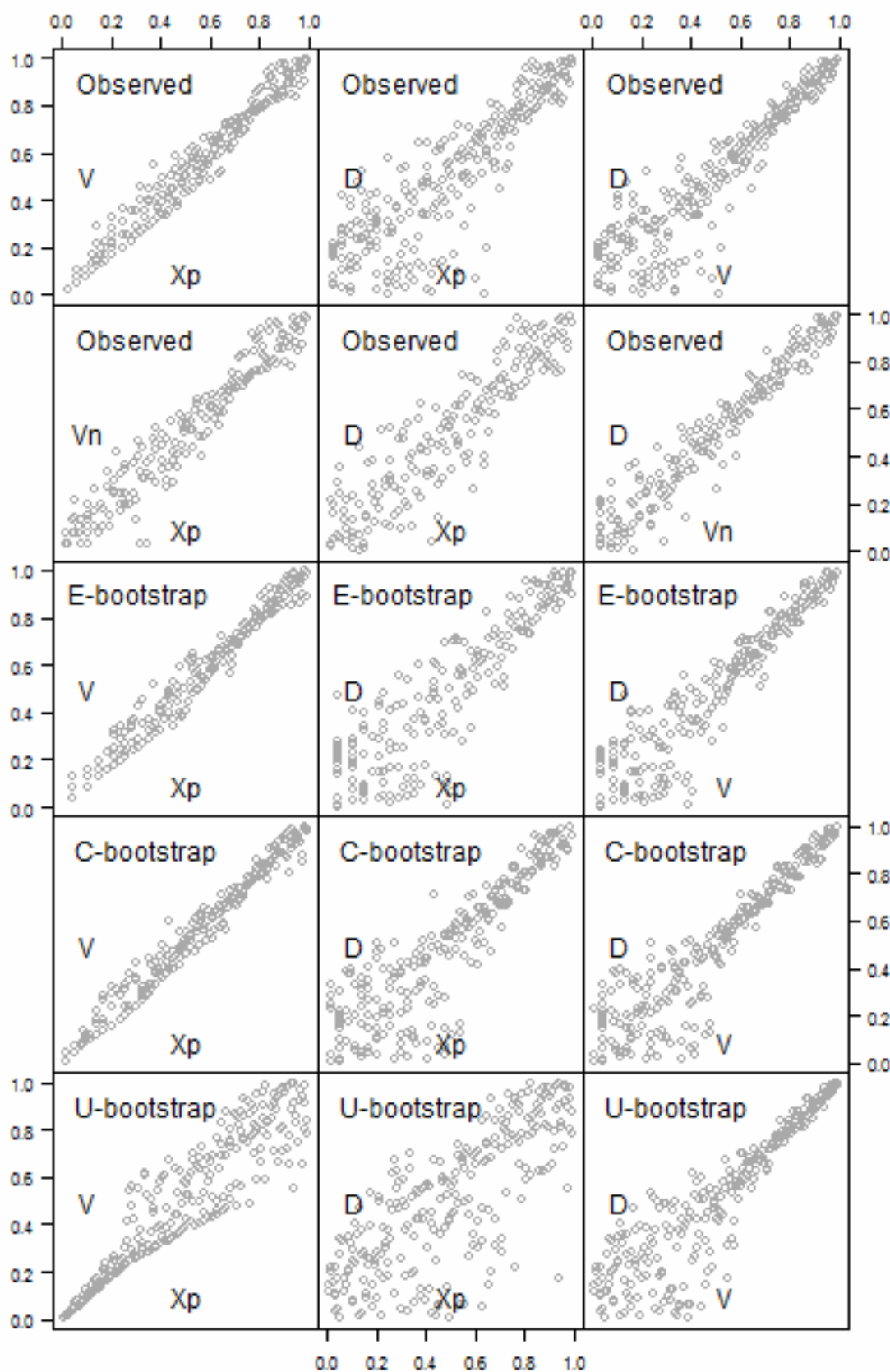

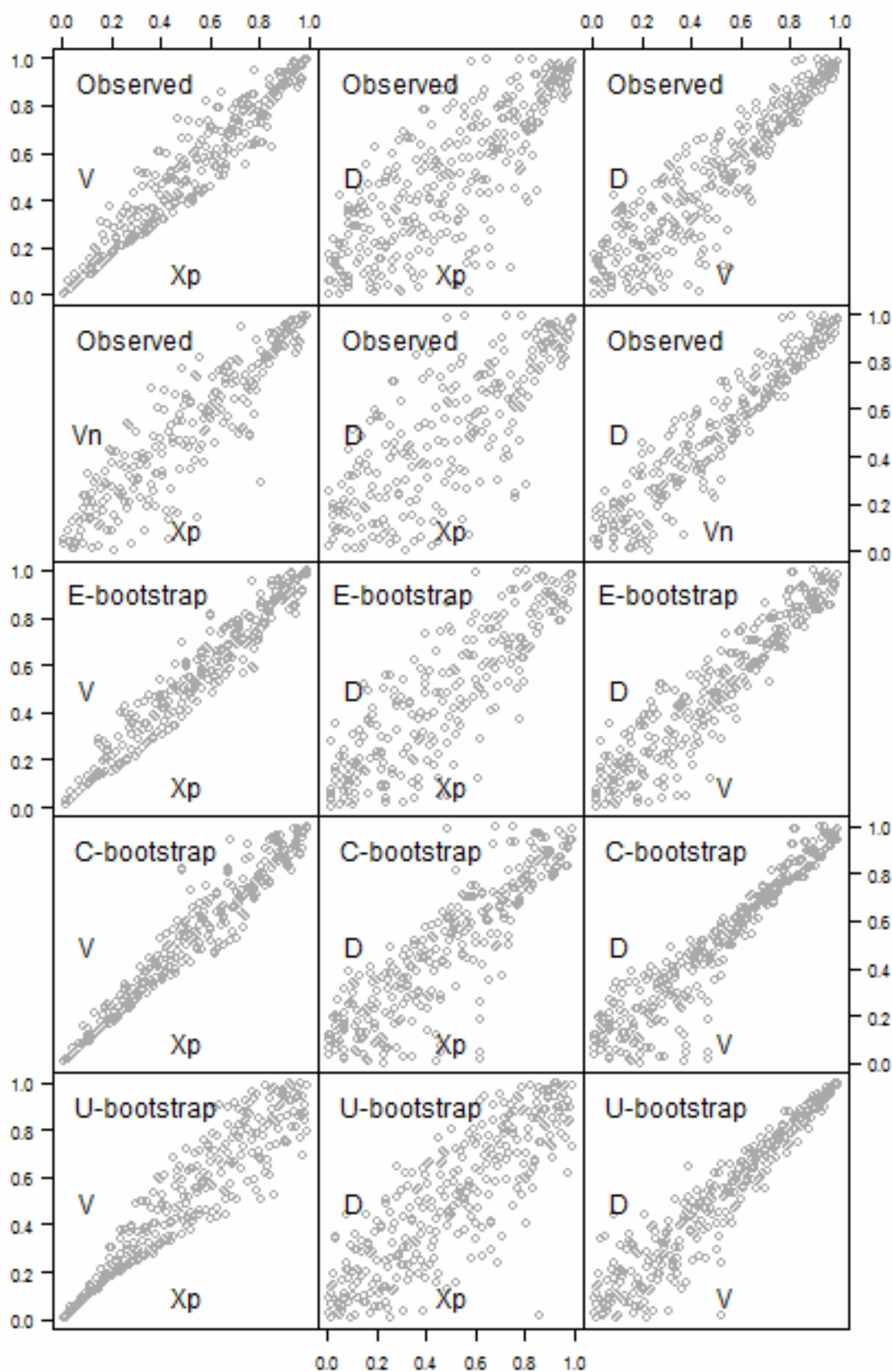

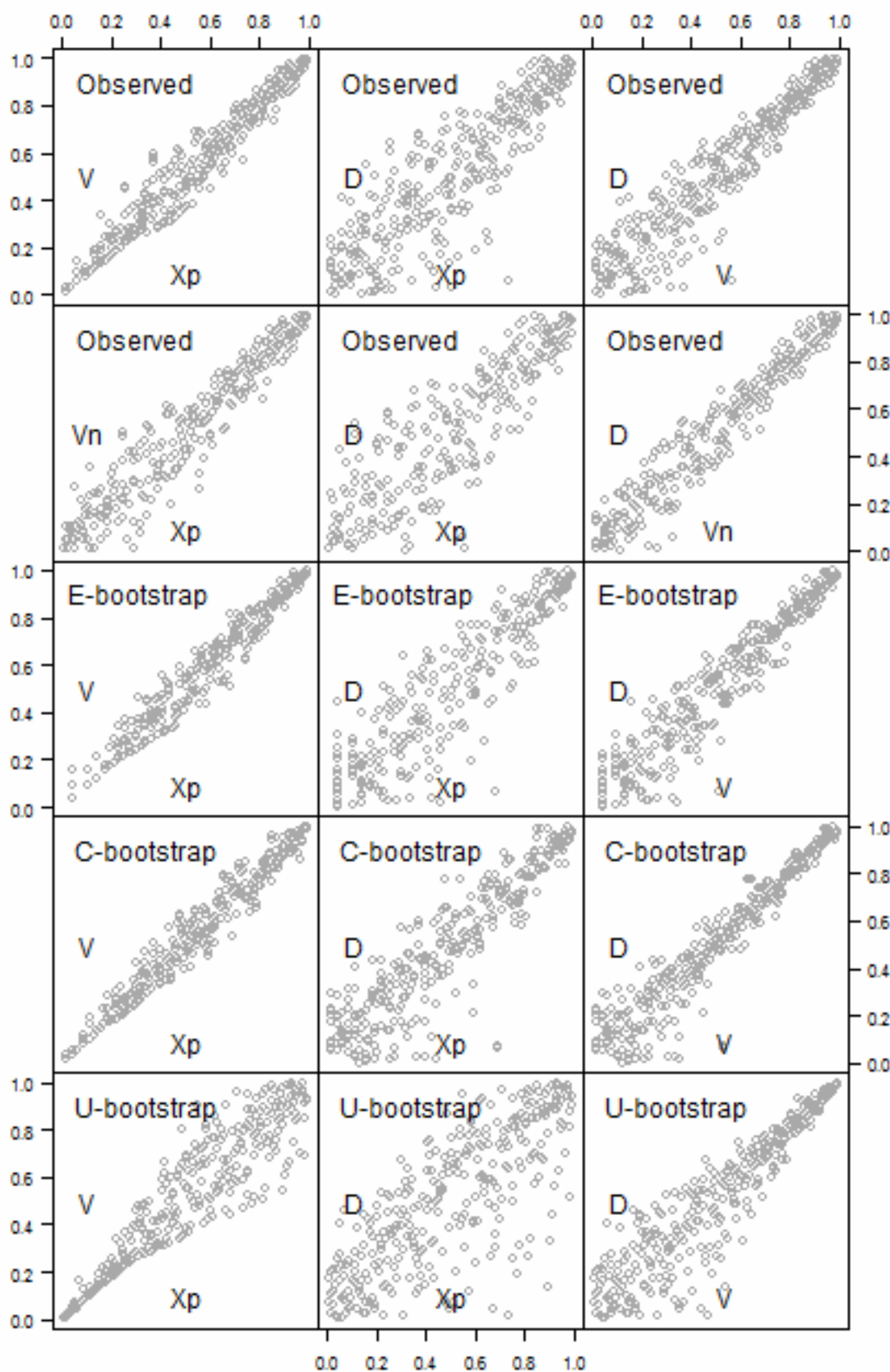

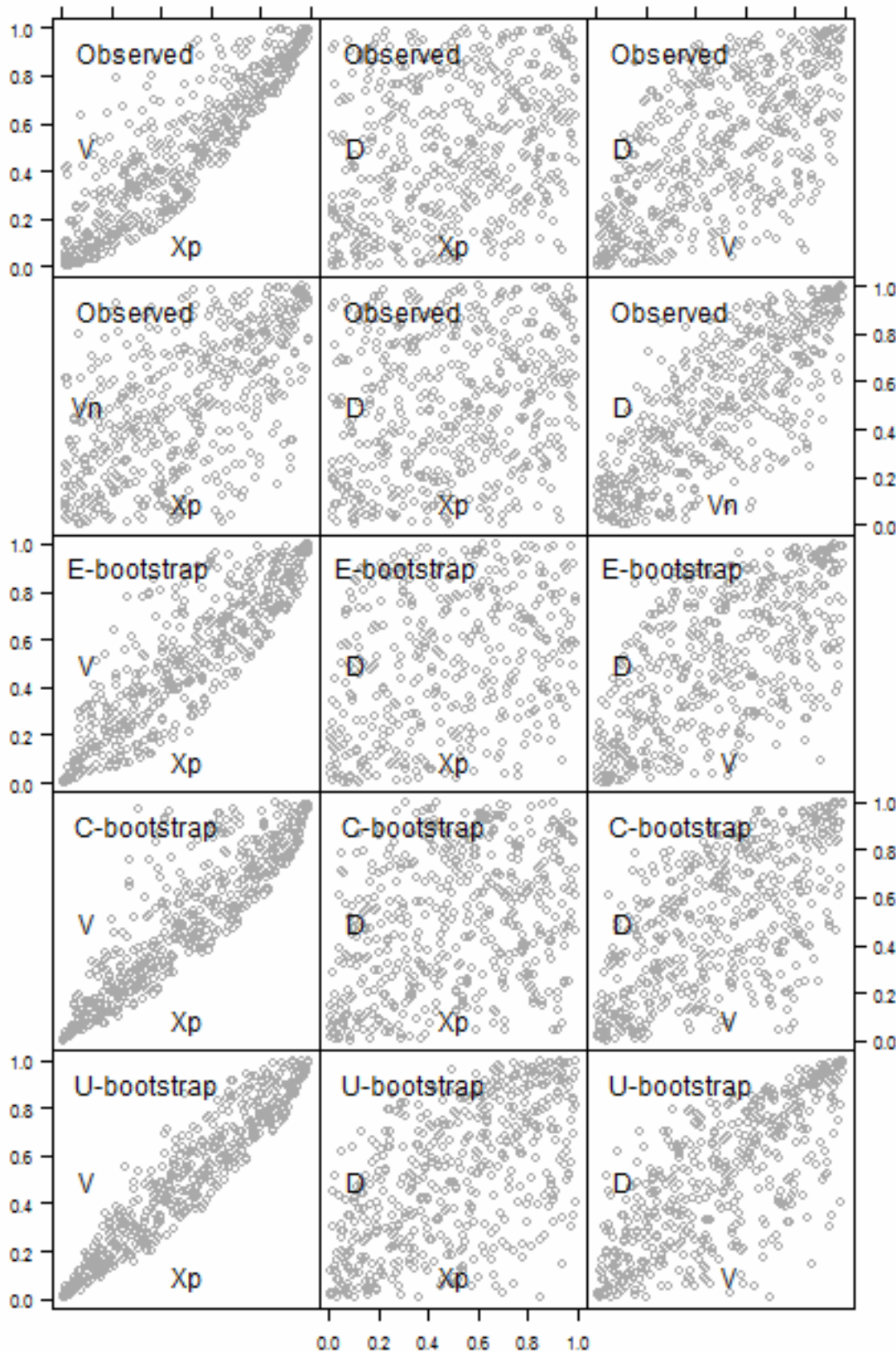

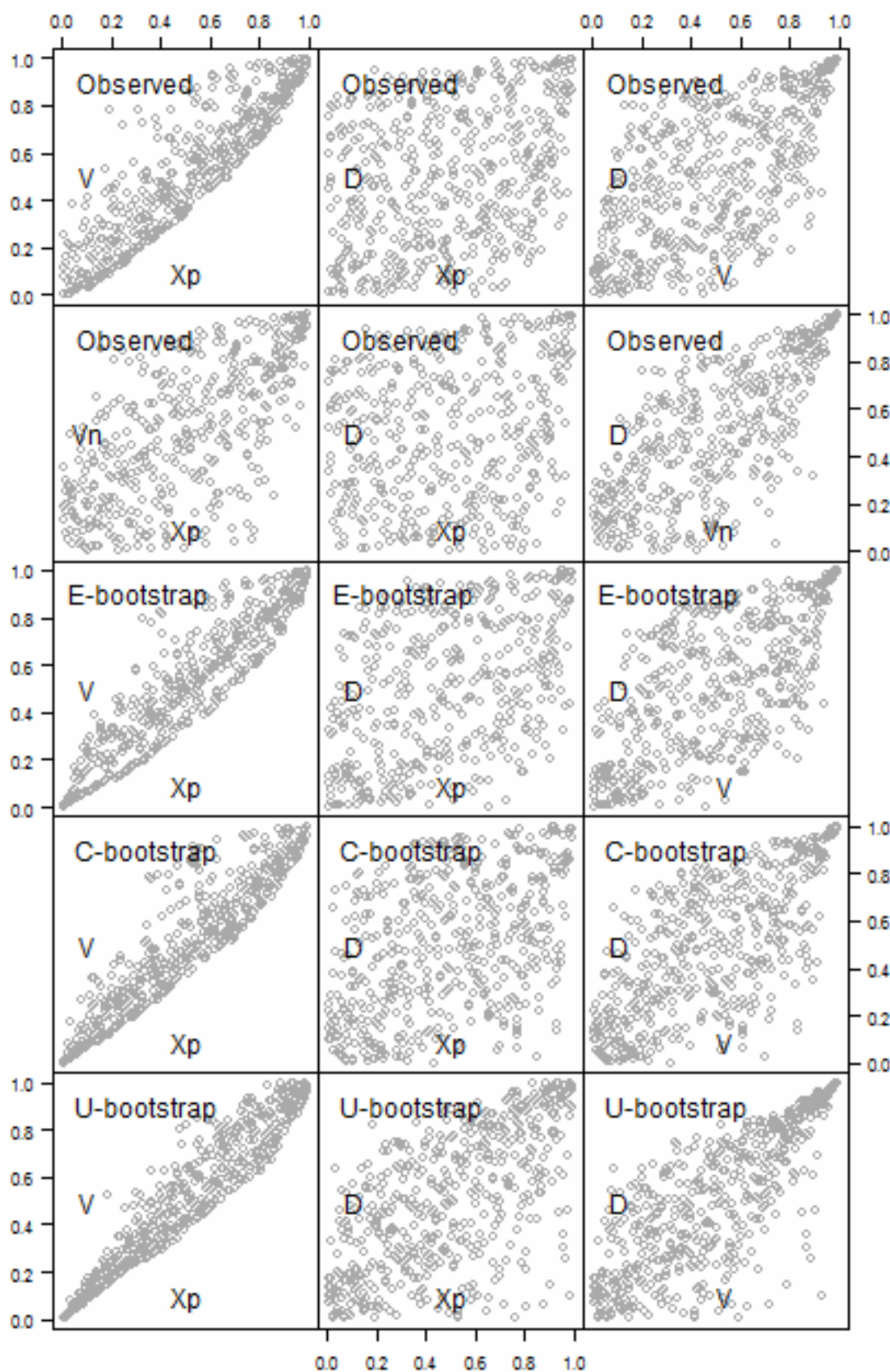

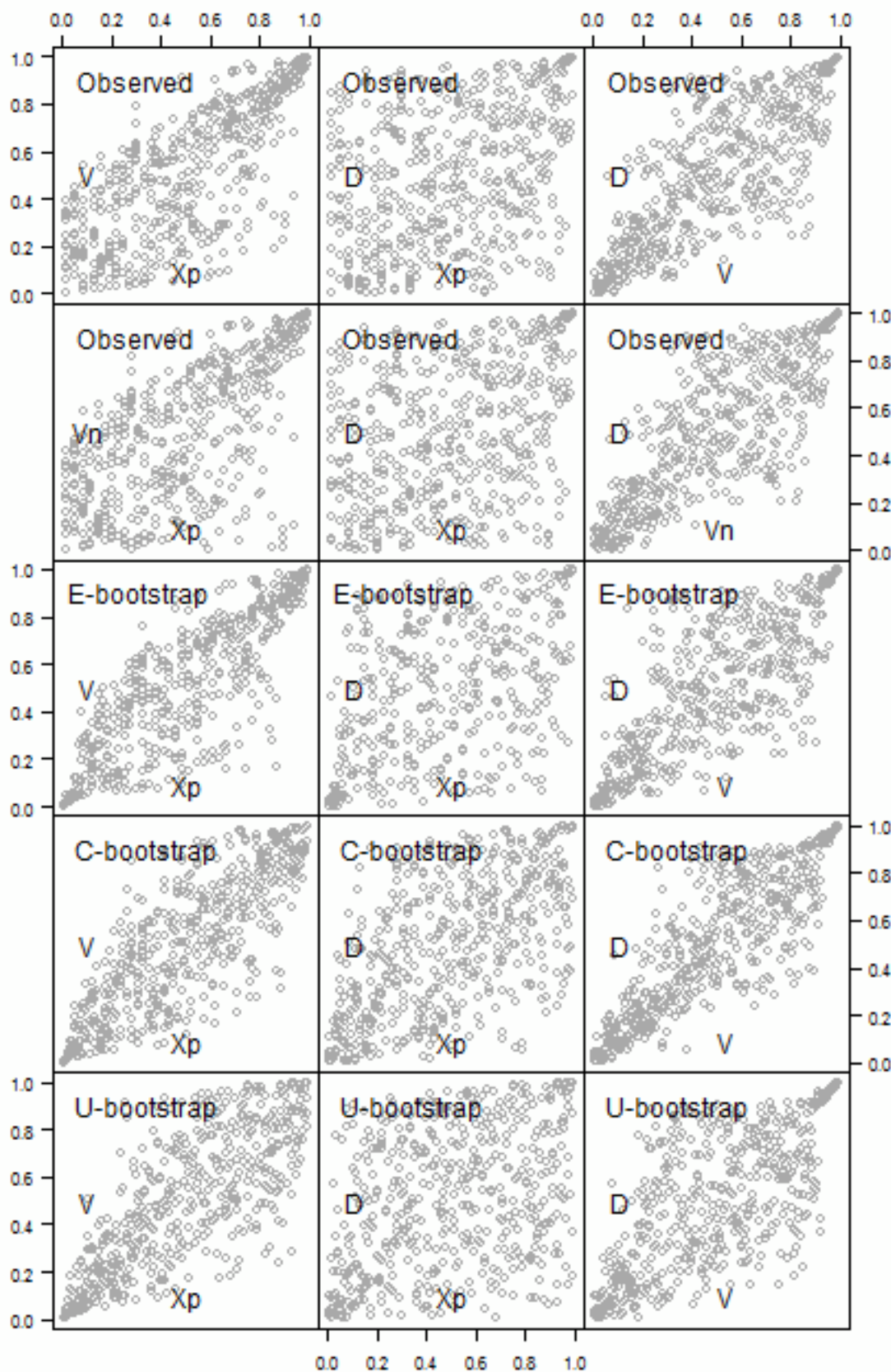

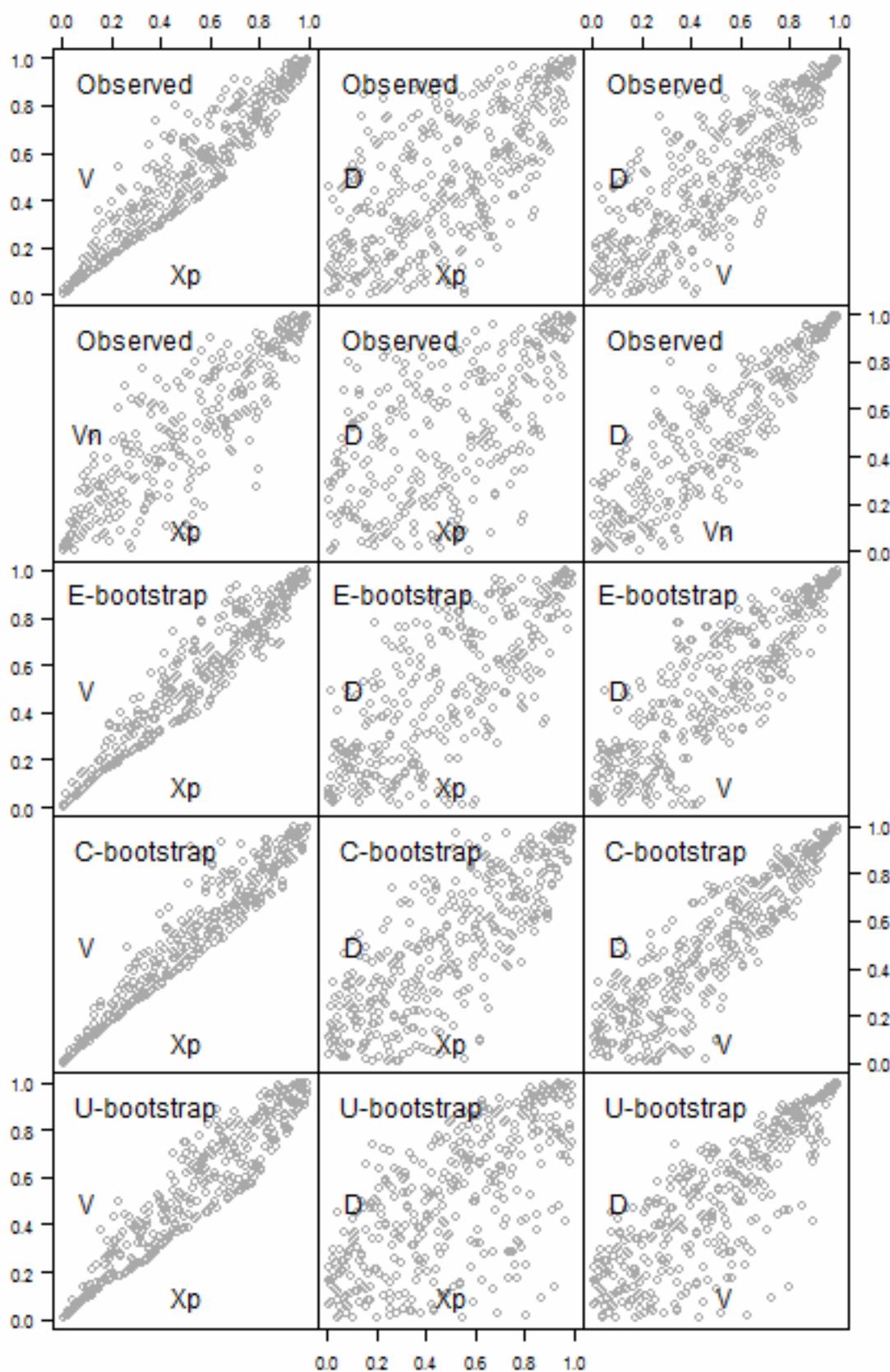

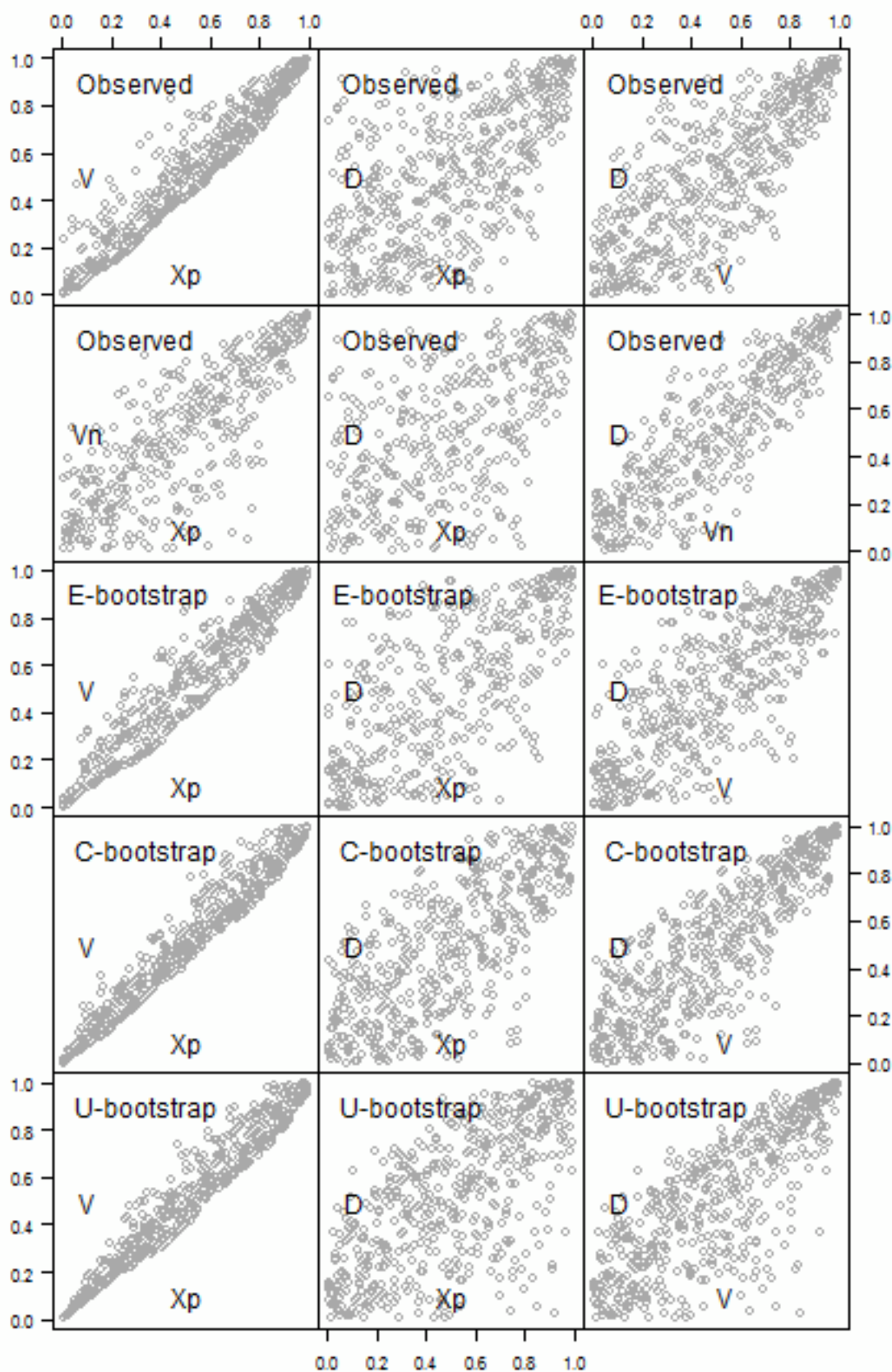

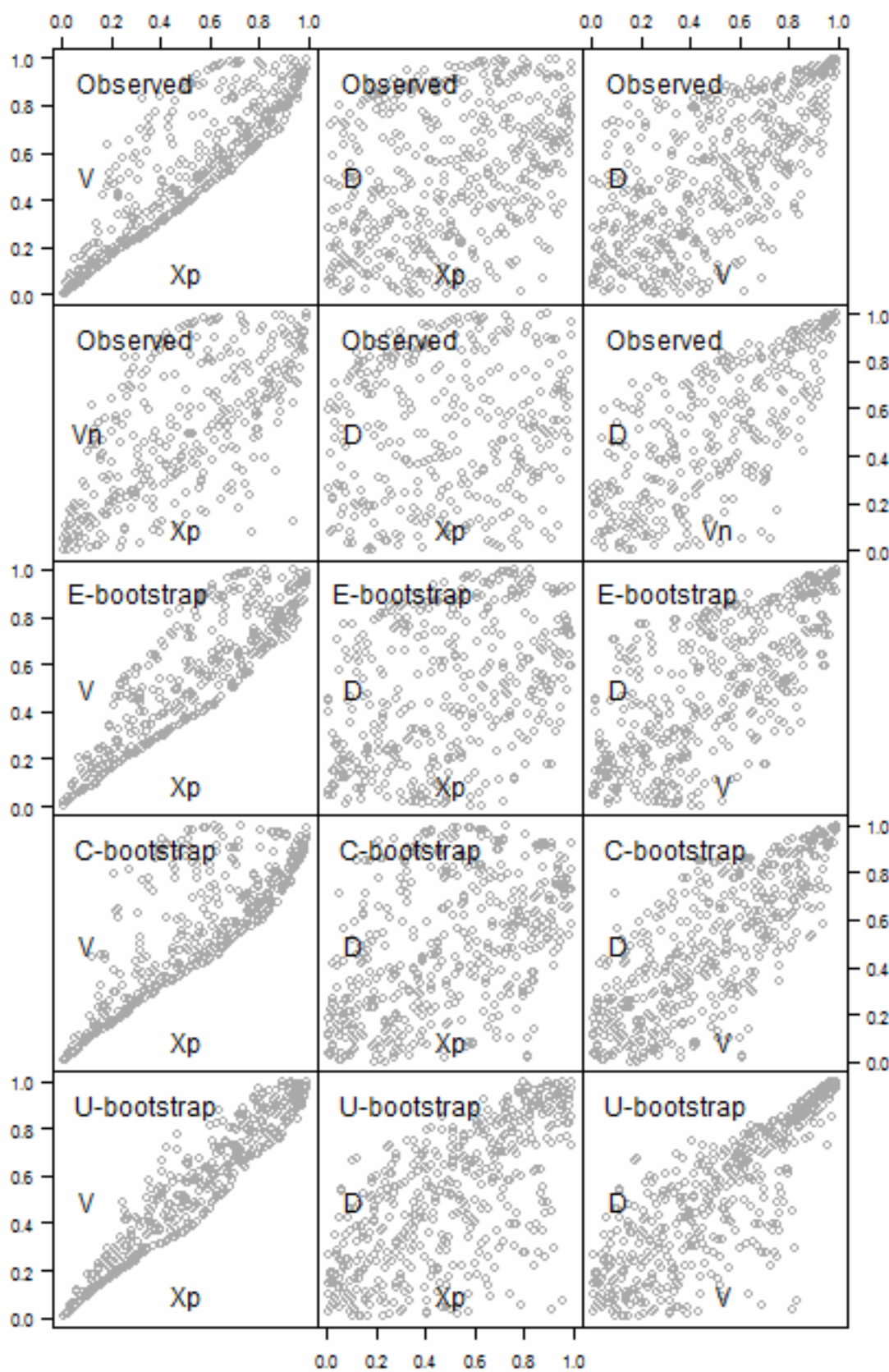

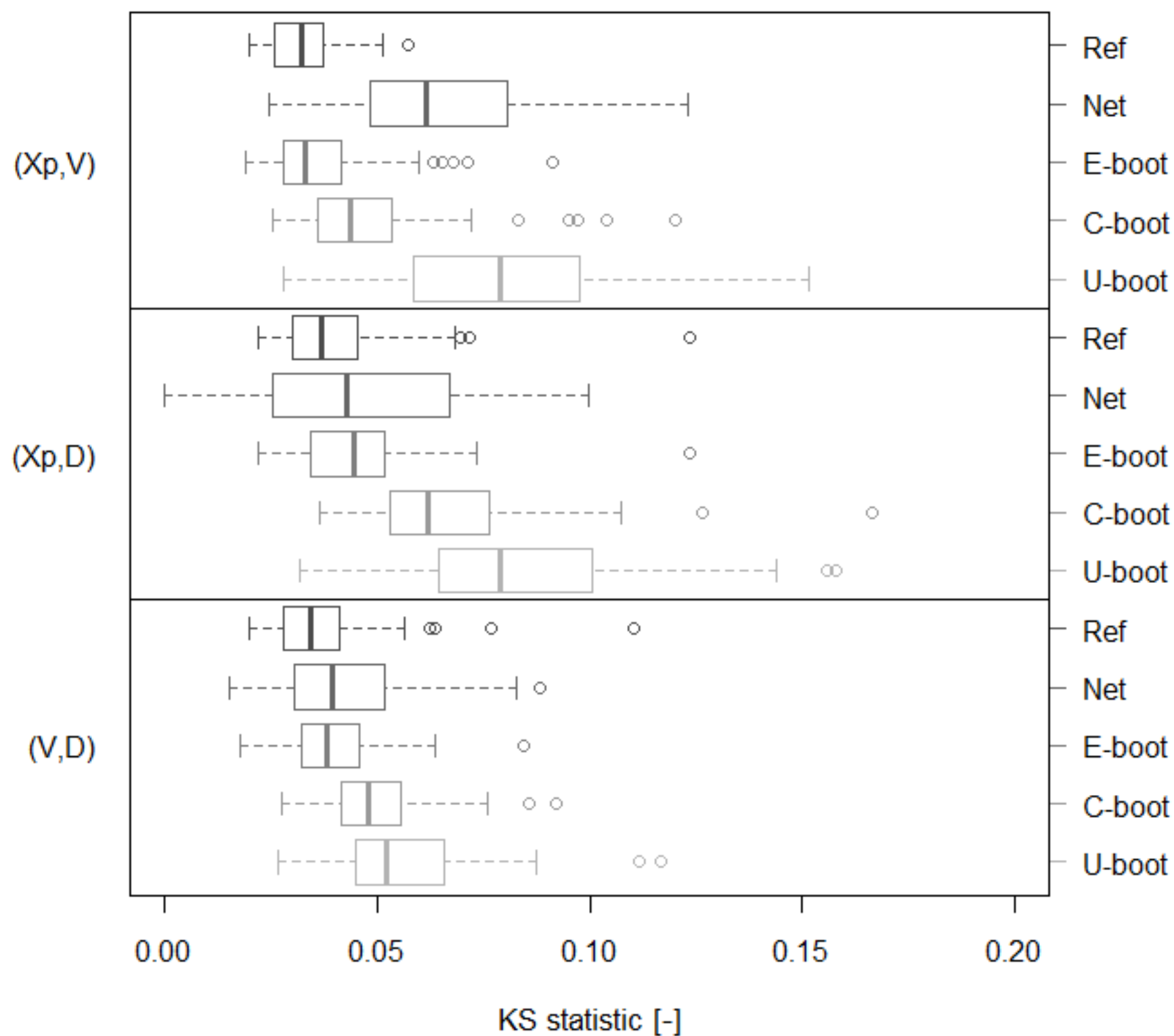

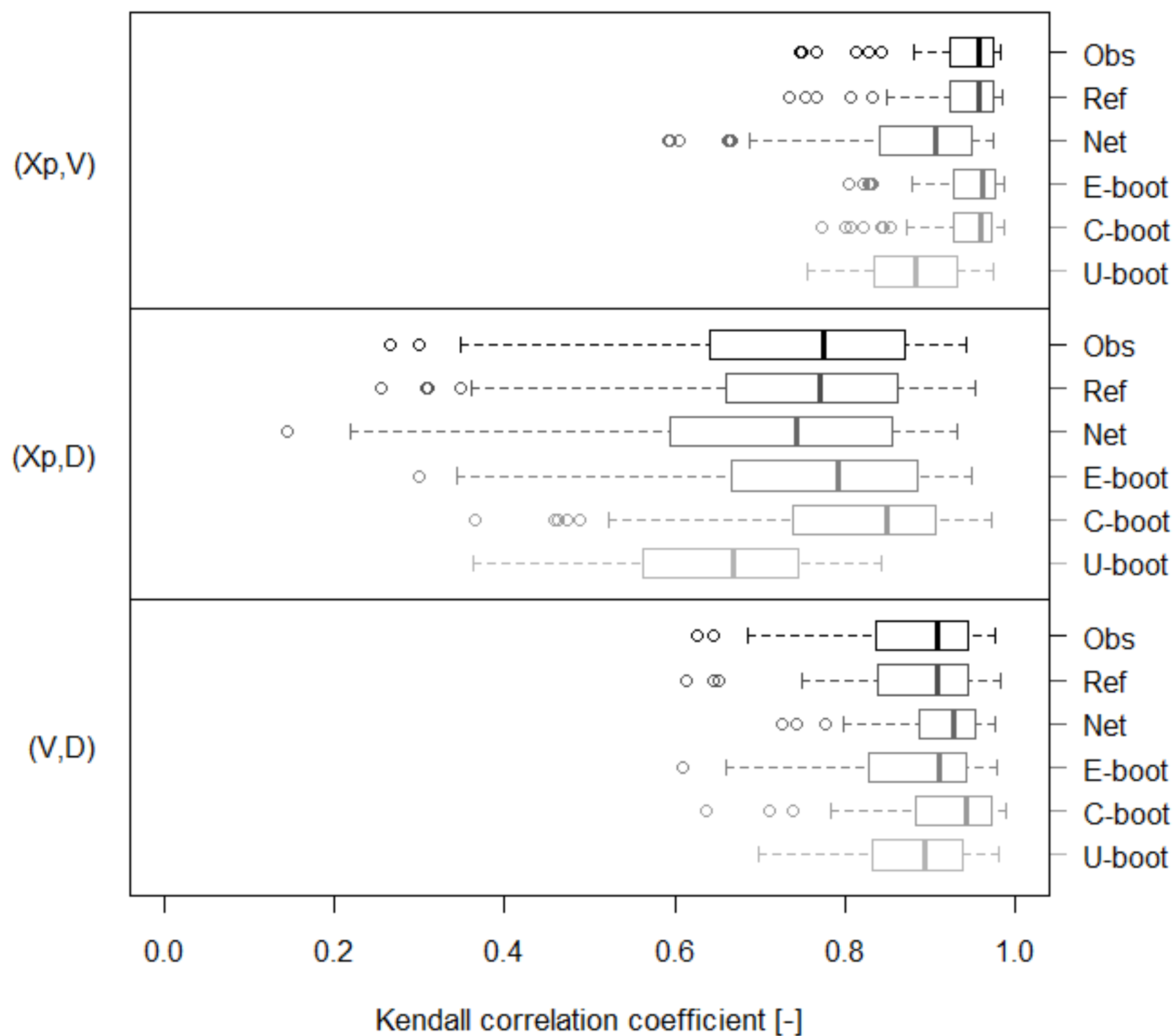

Supplement: Supplementary file 3 — Supplementary Figures [file wrcr0049-3423-sd3.pdf]

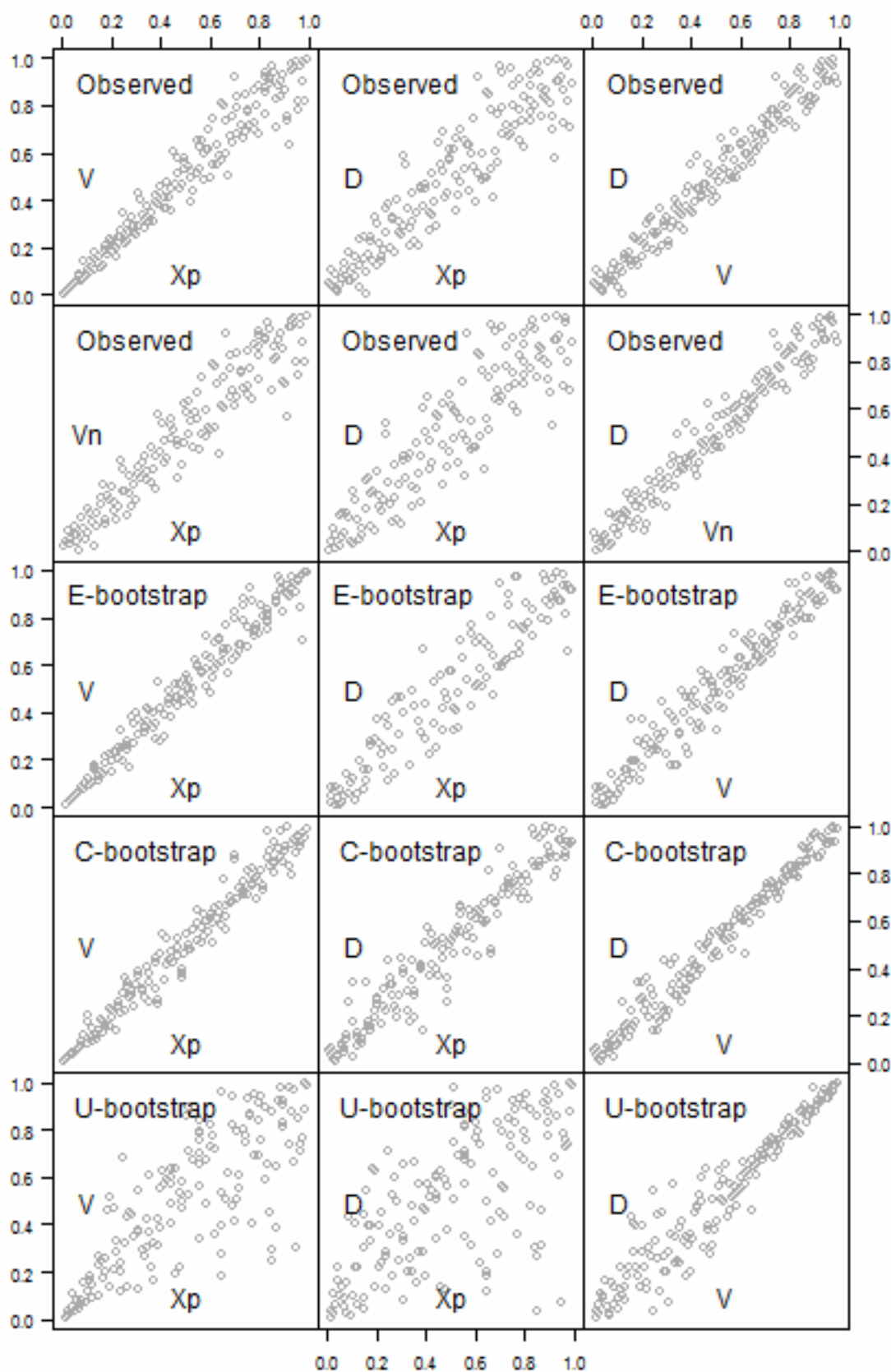

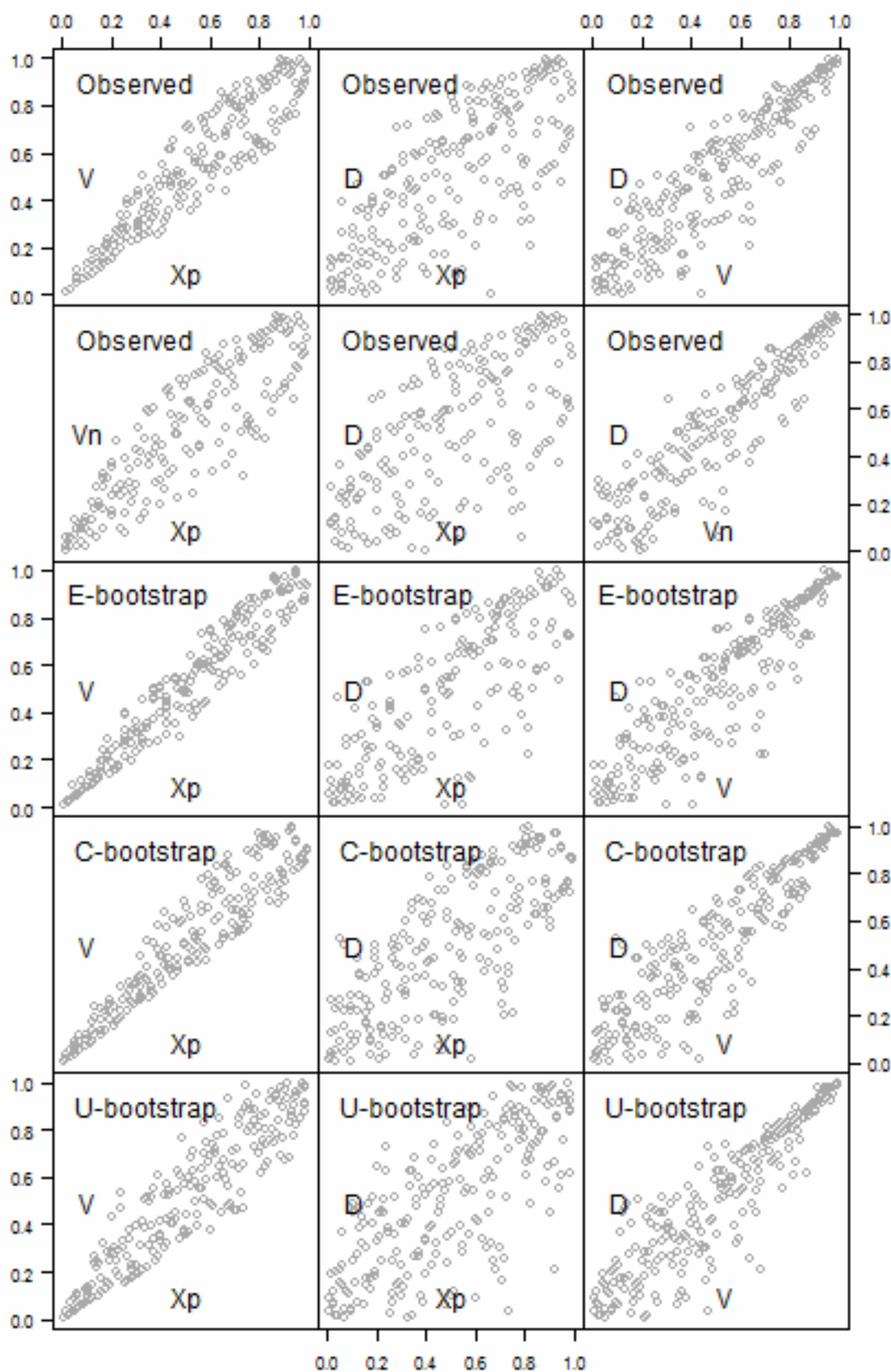

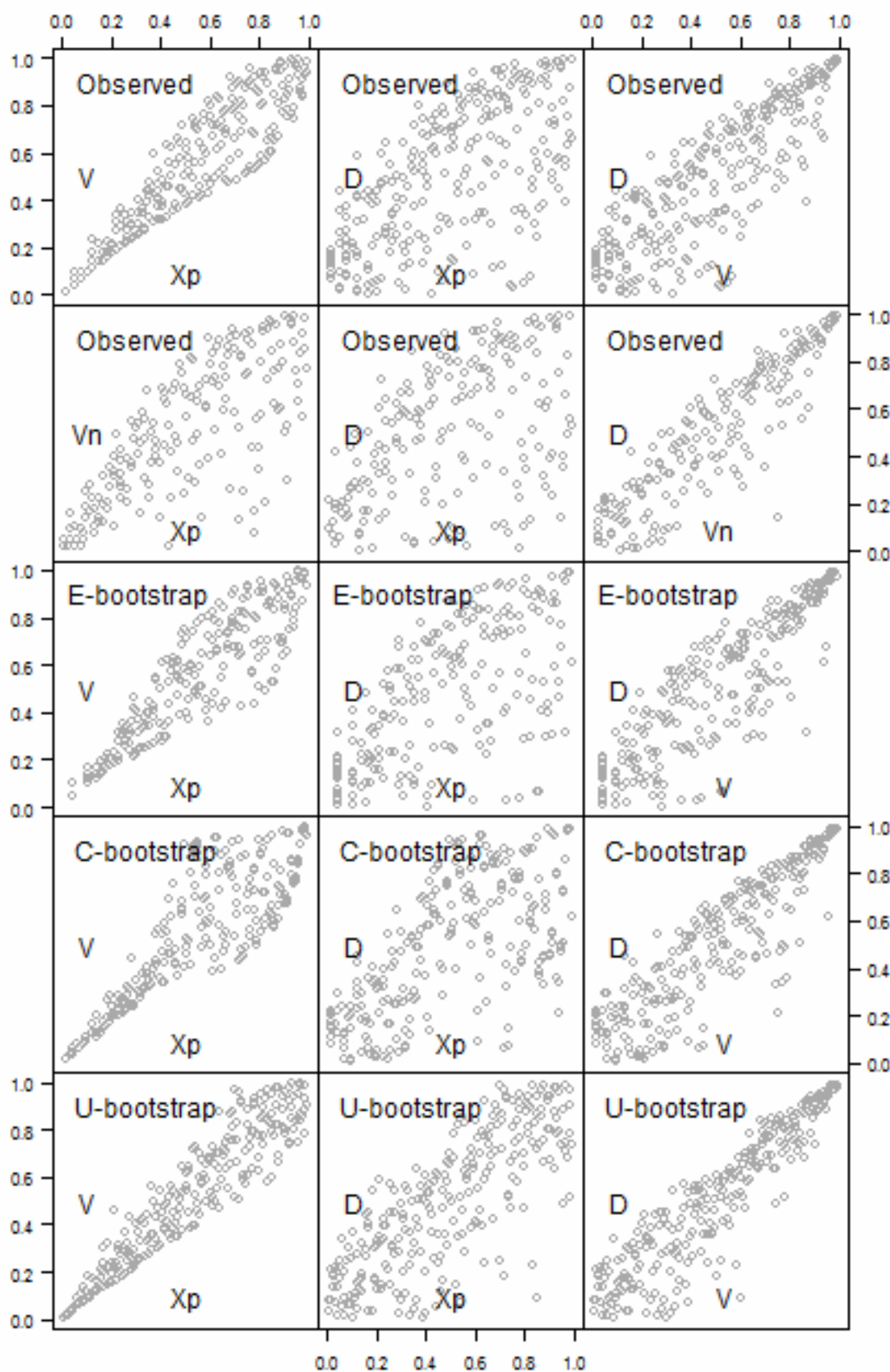

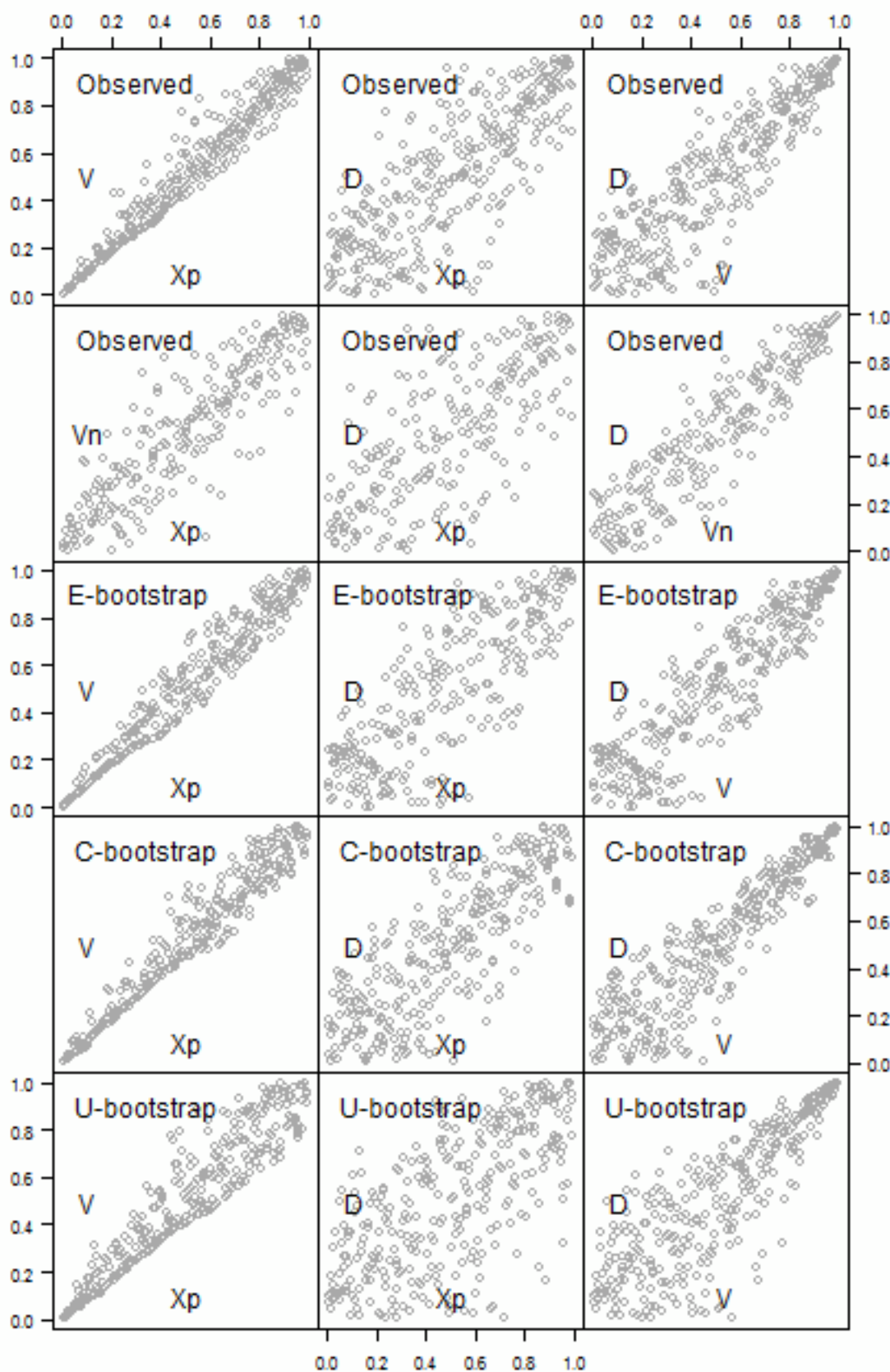

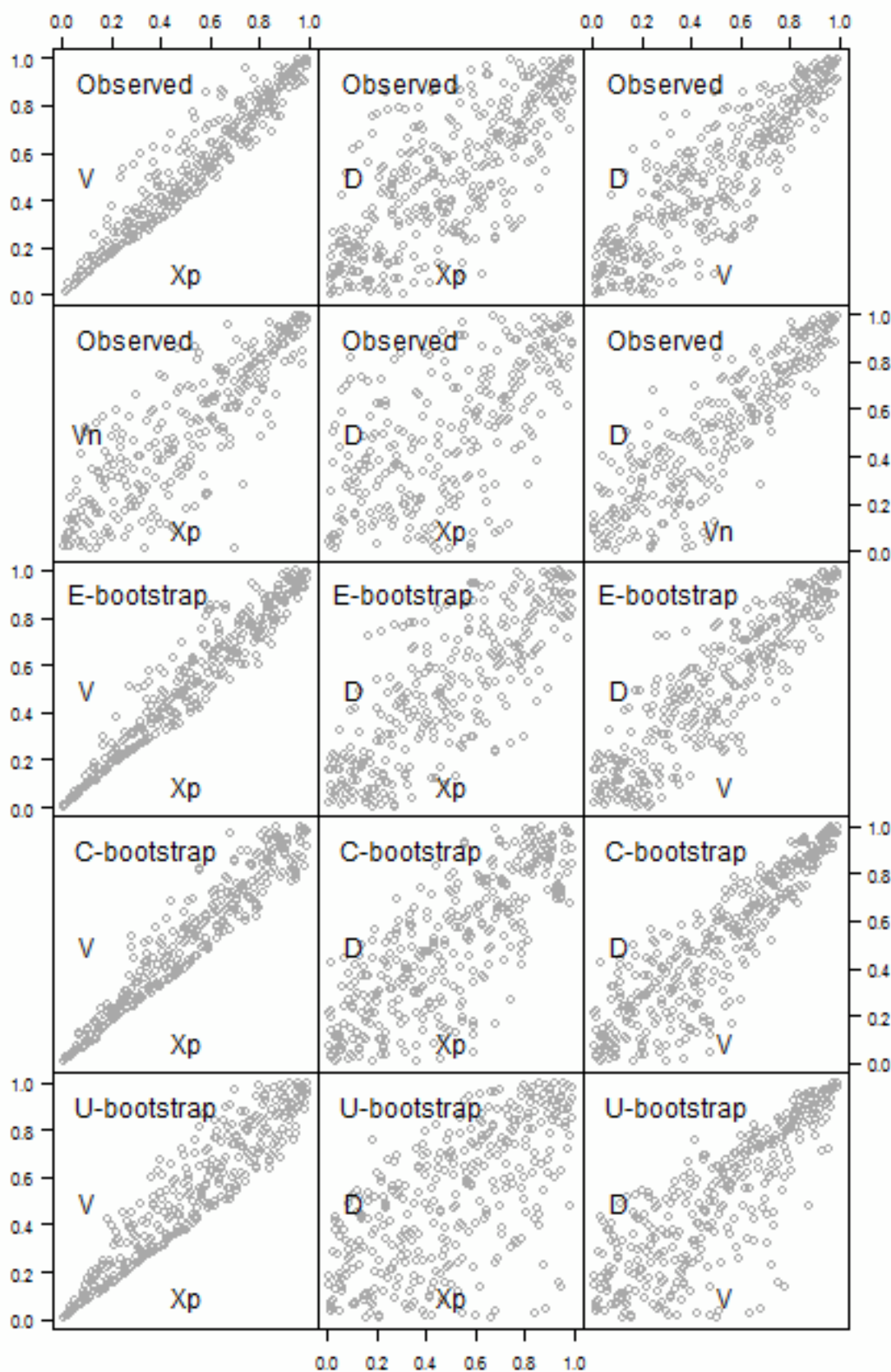

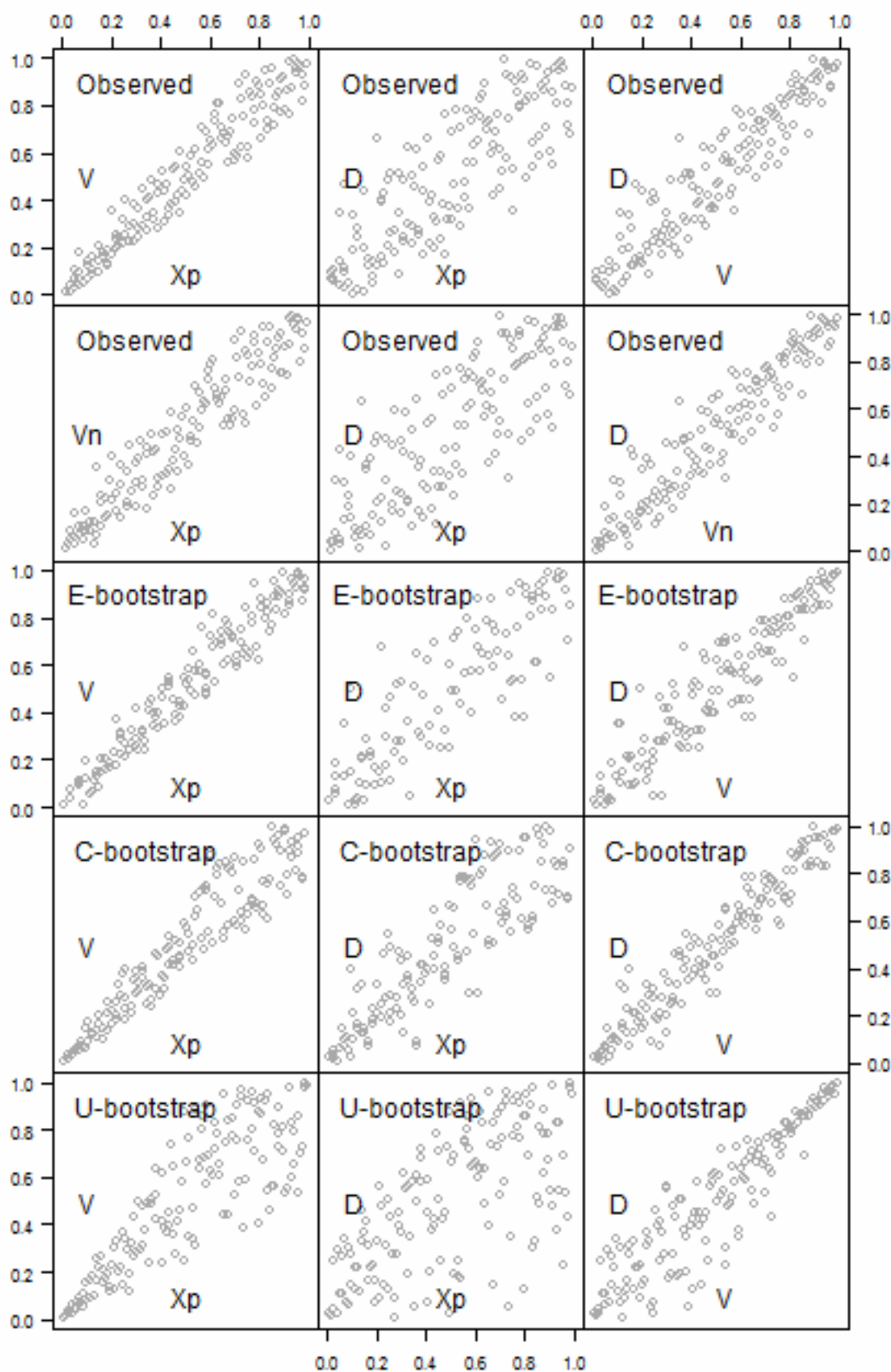

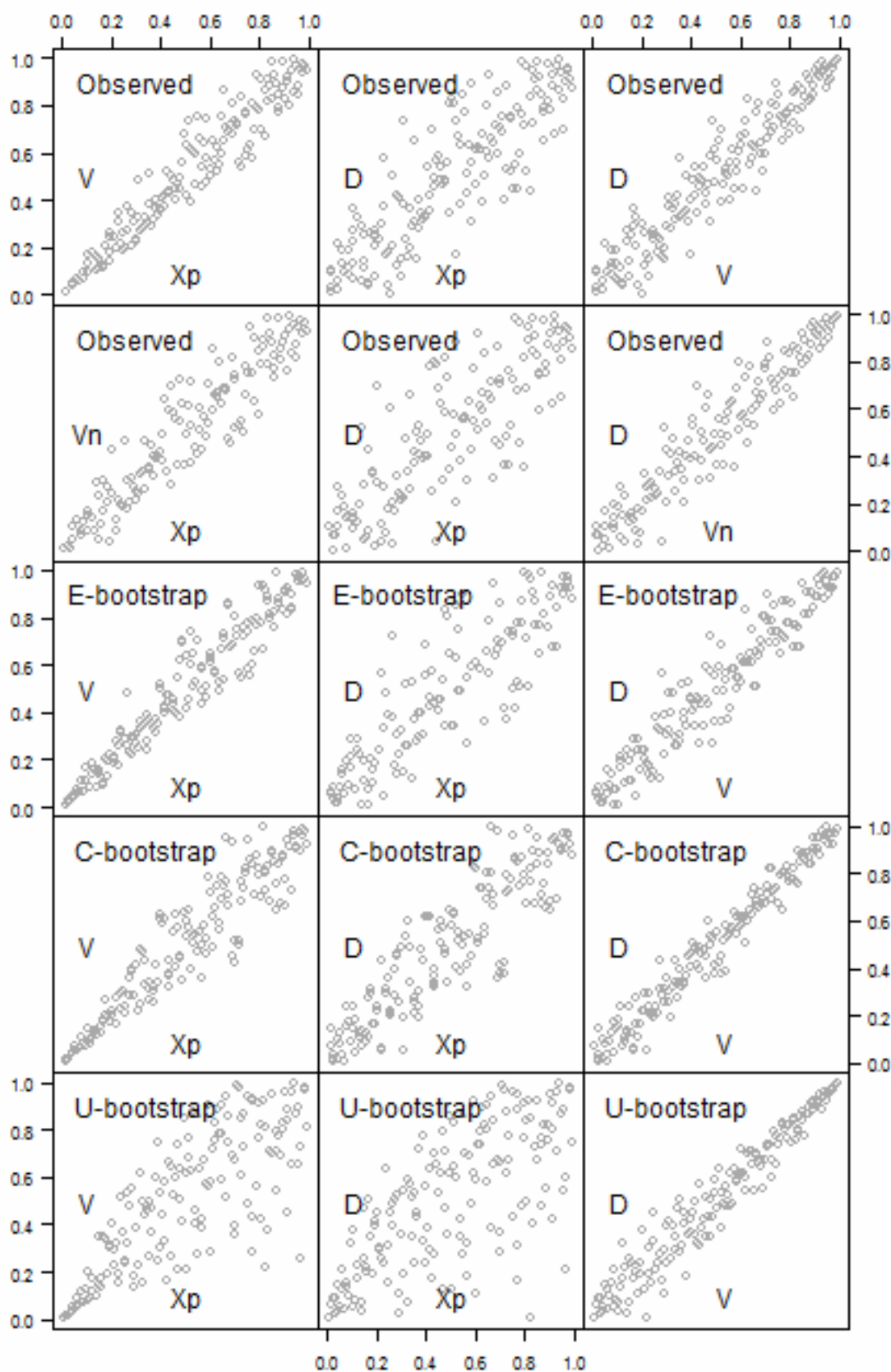

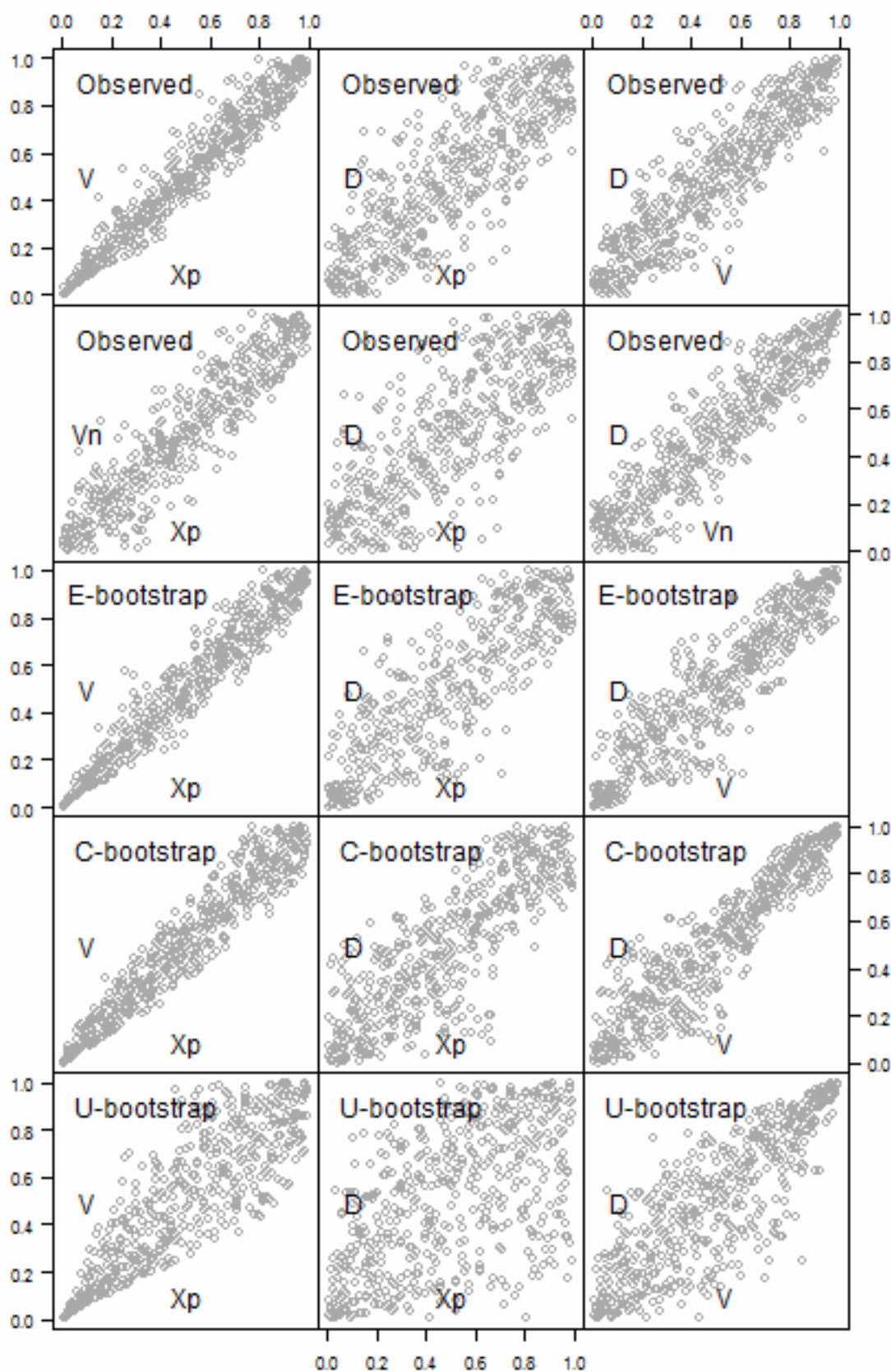

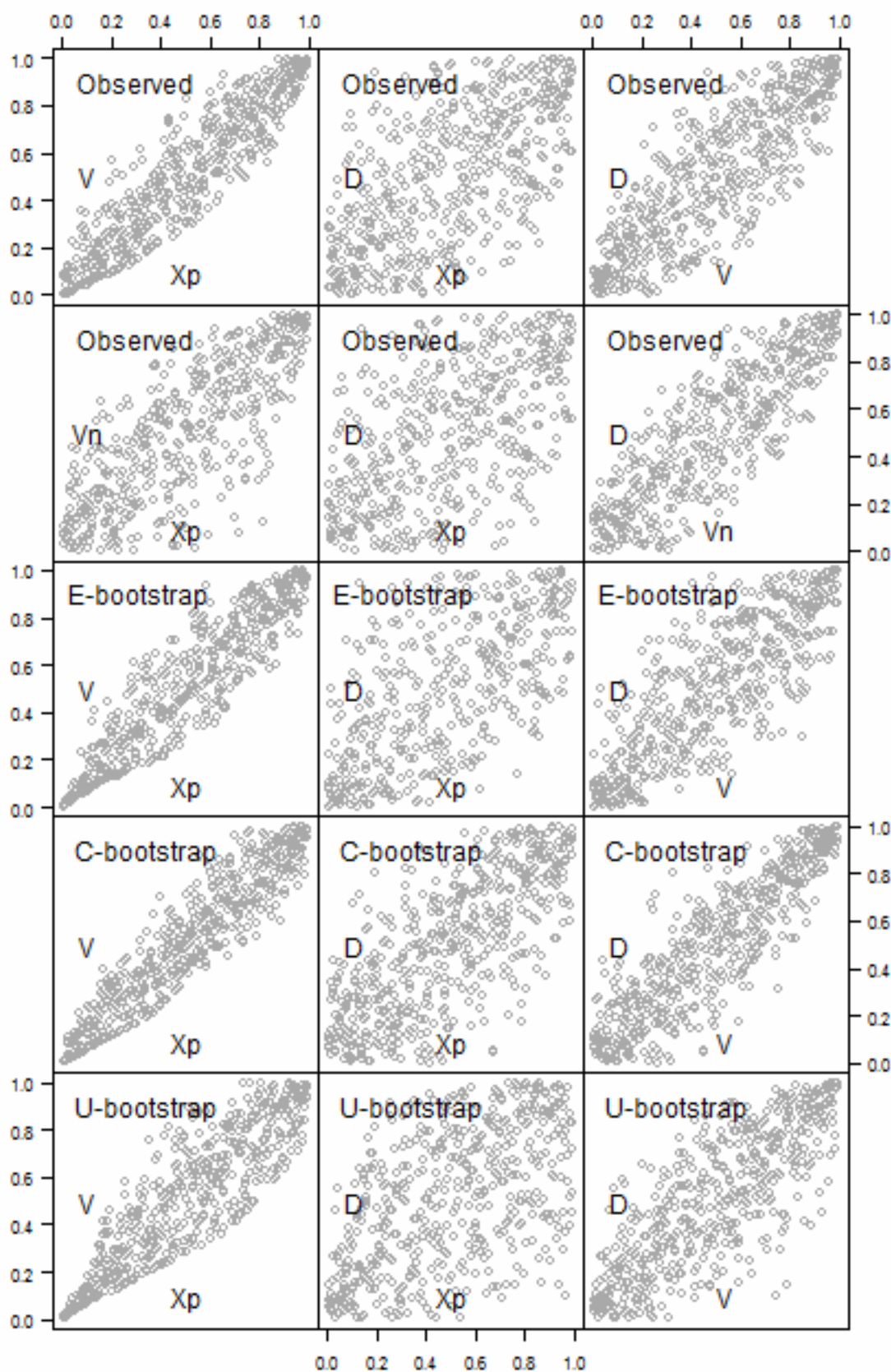

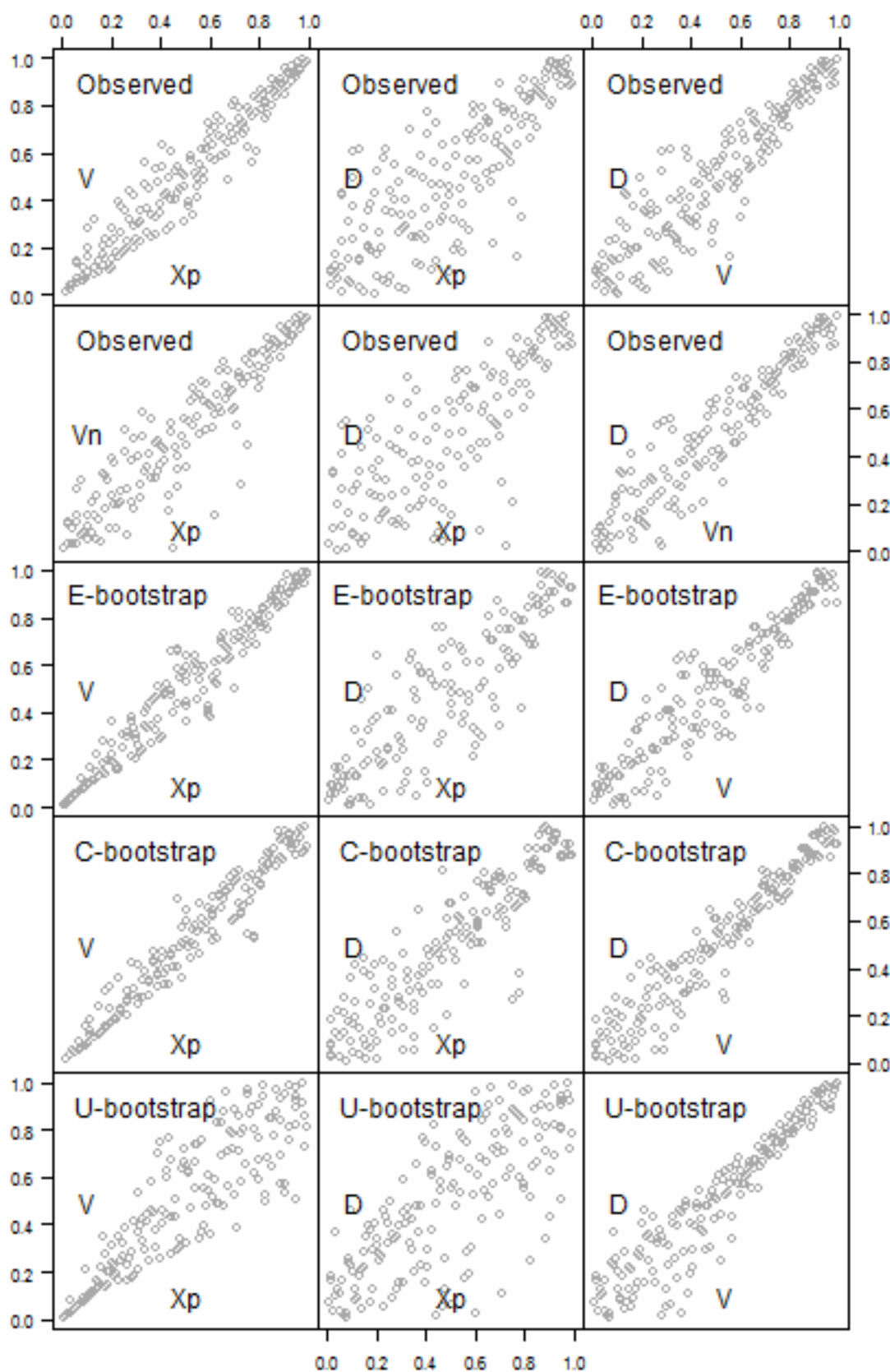

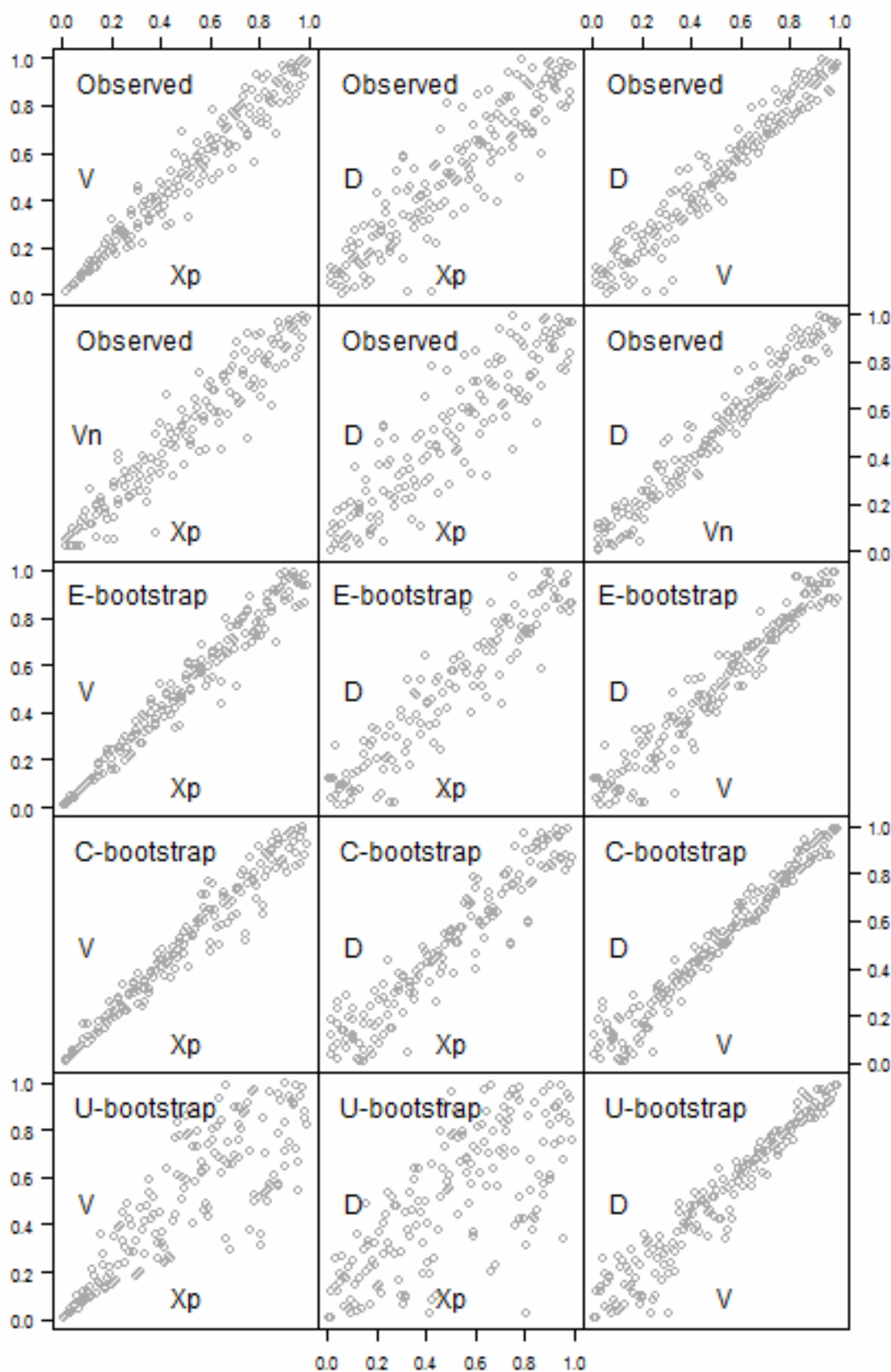

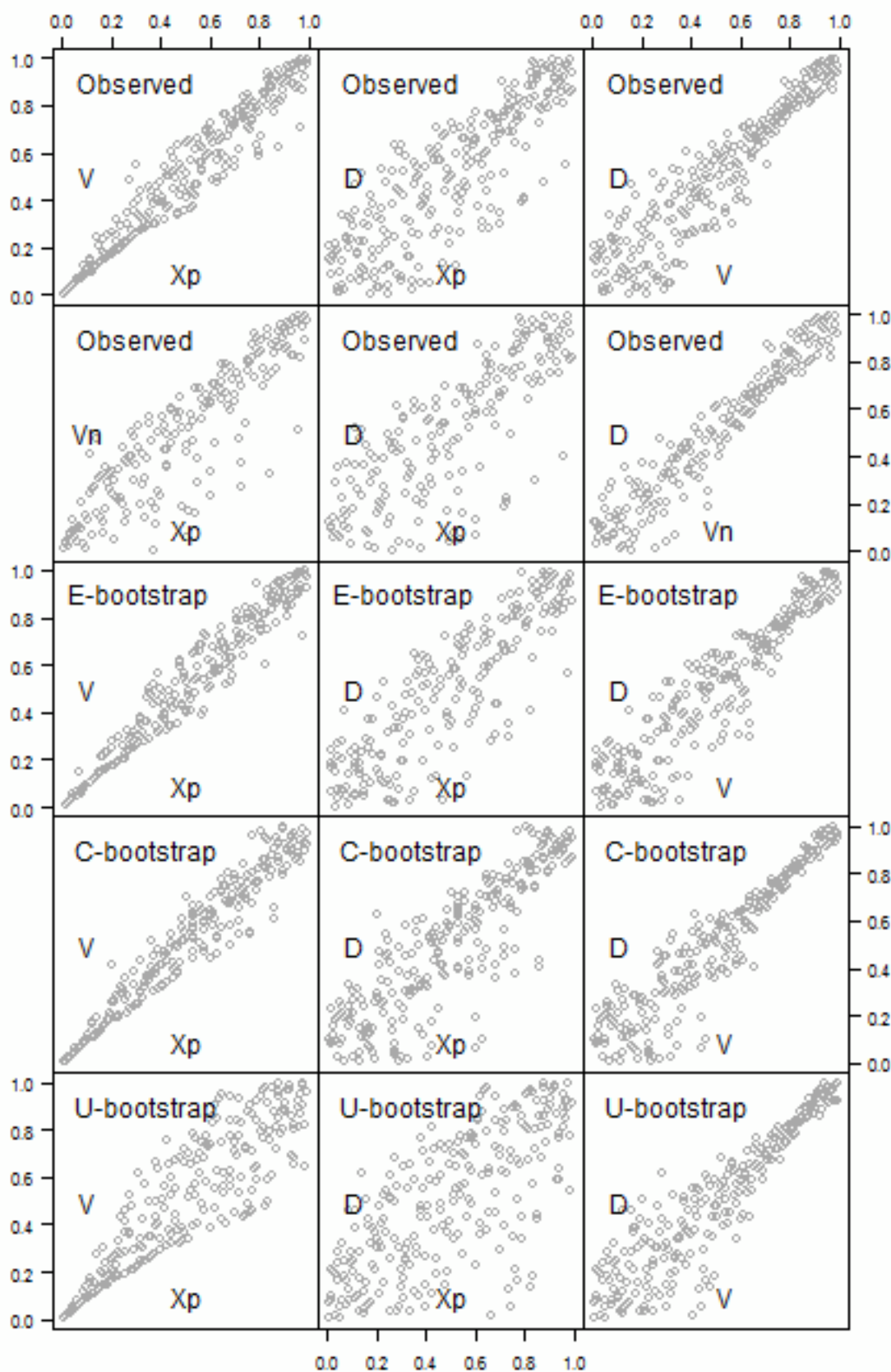

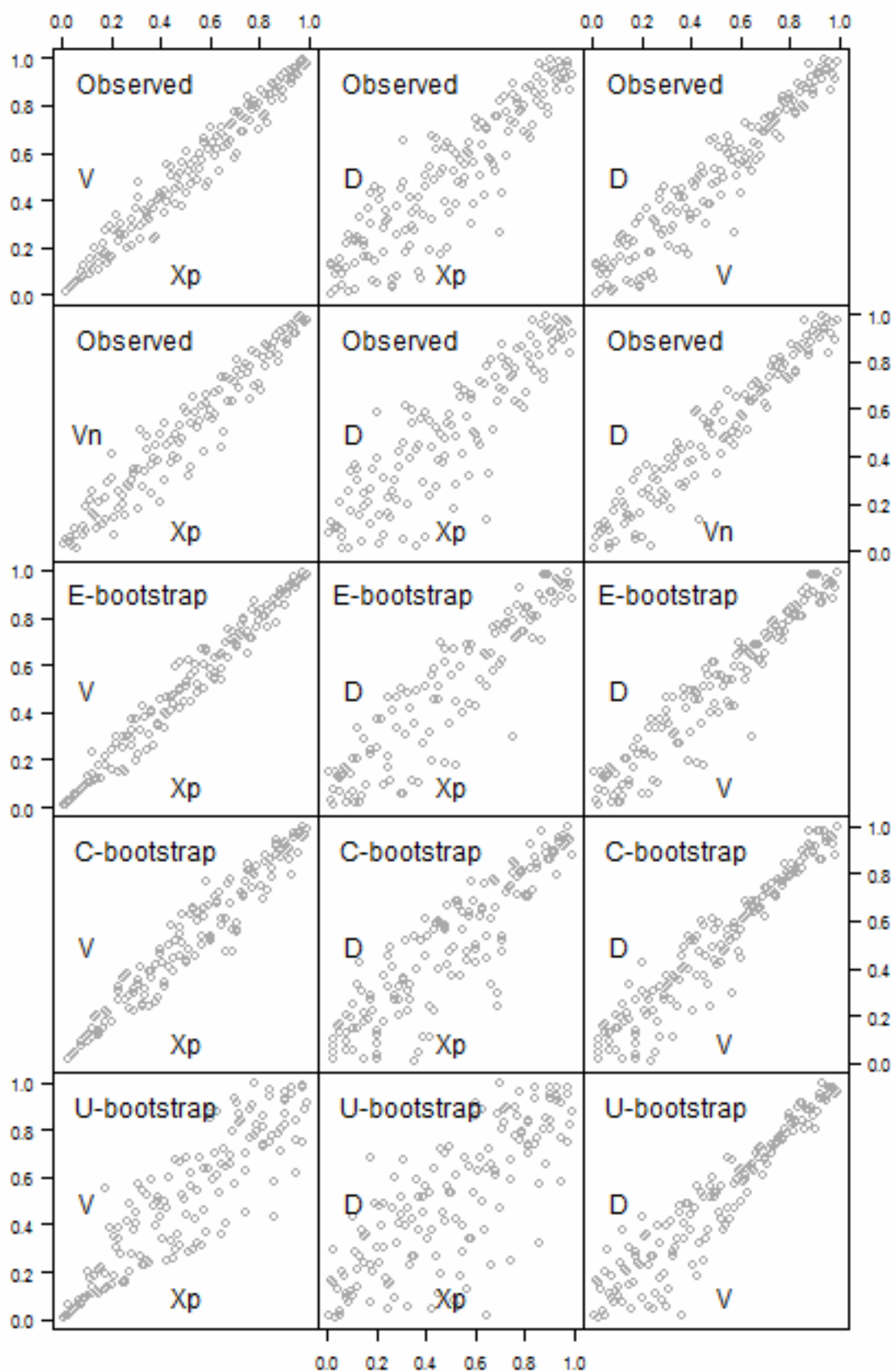

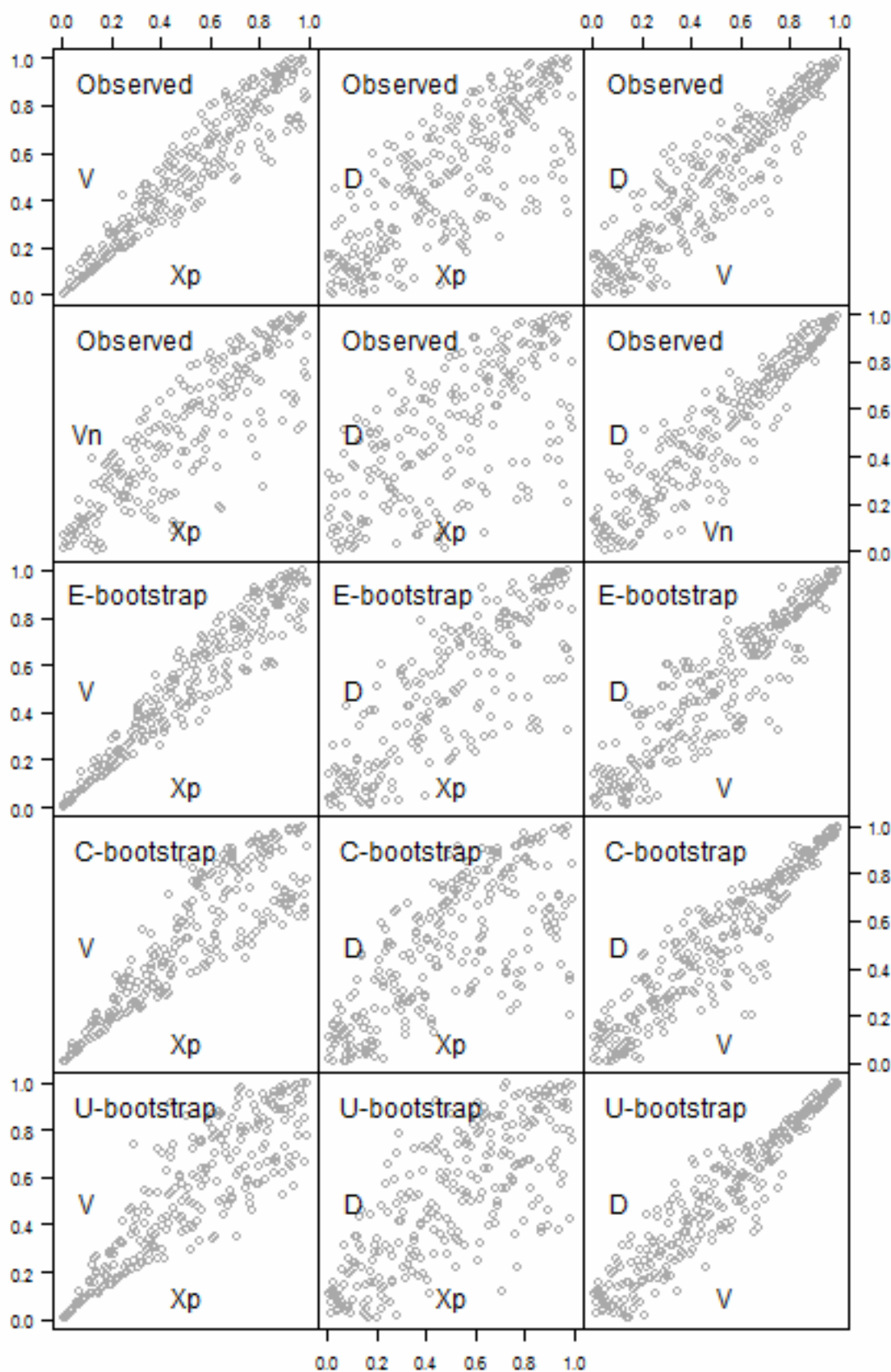

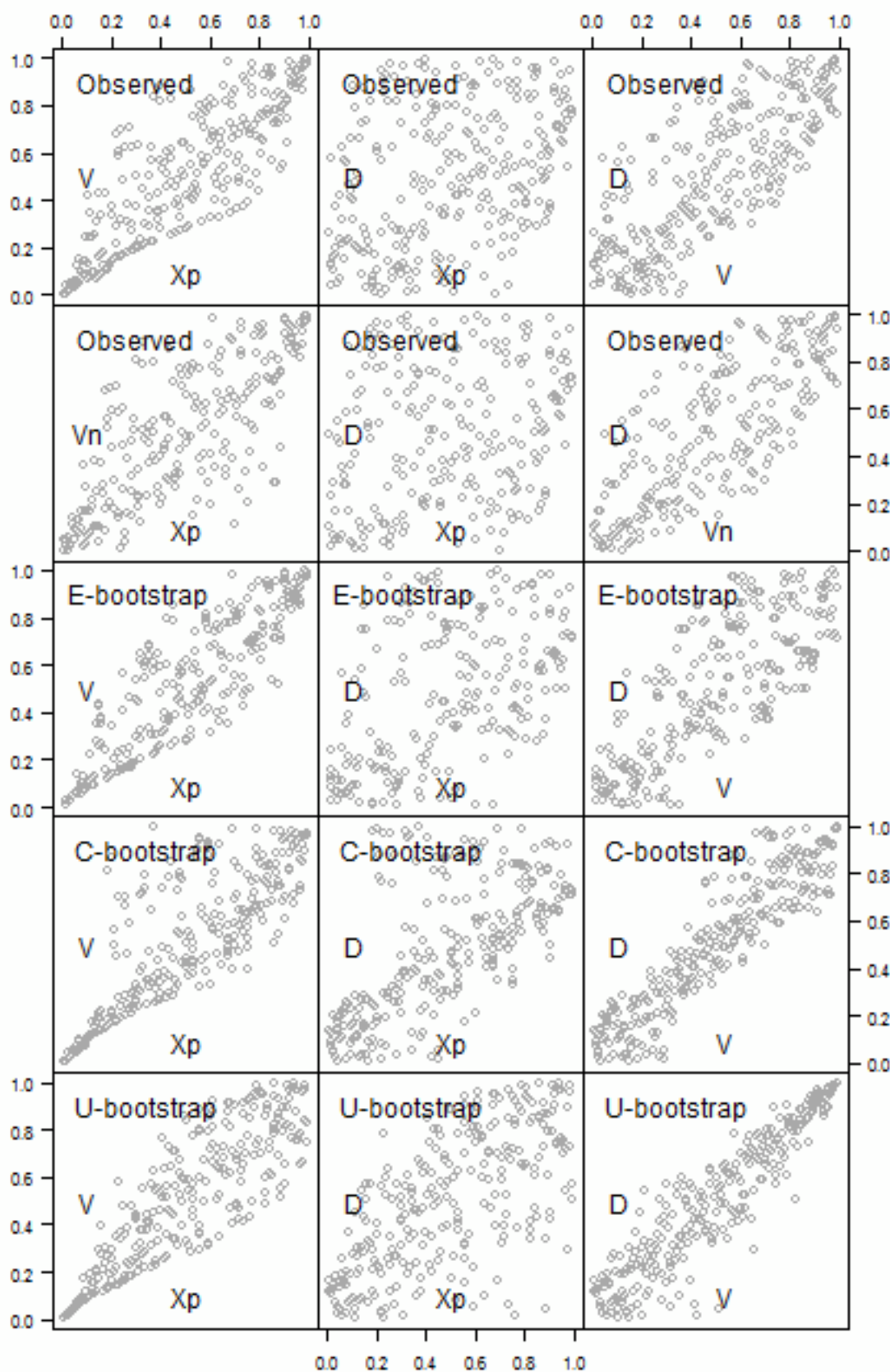

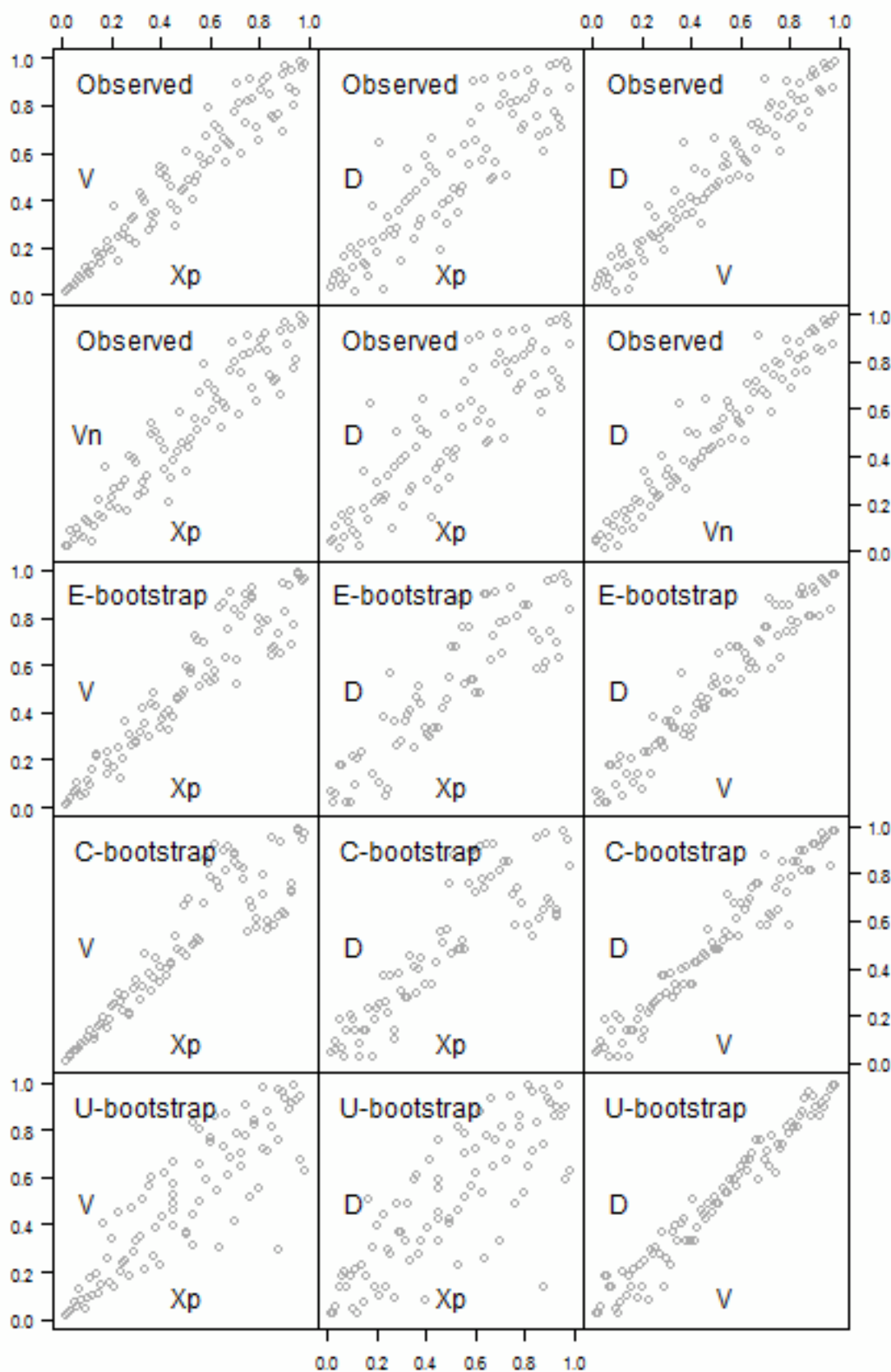

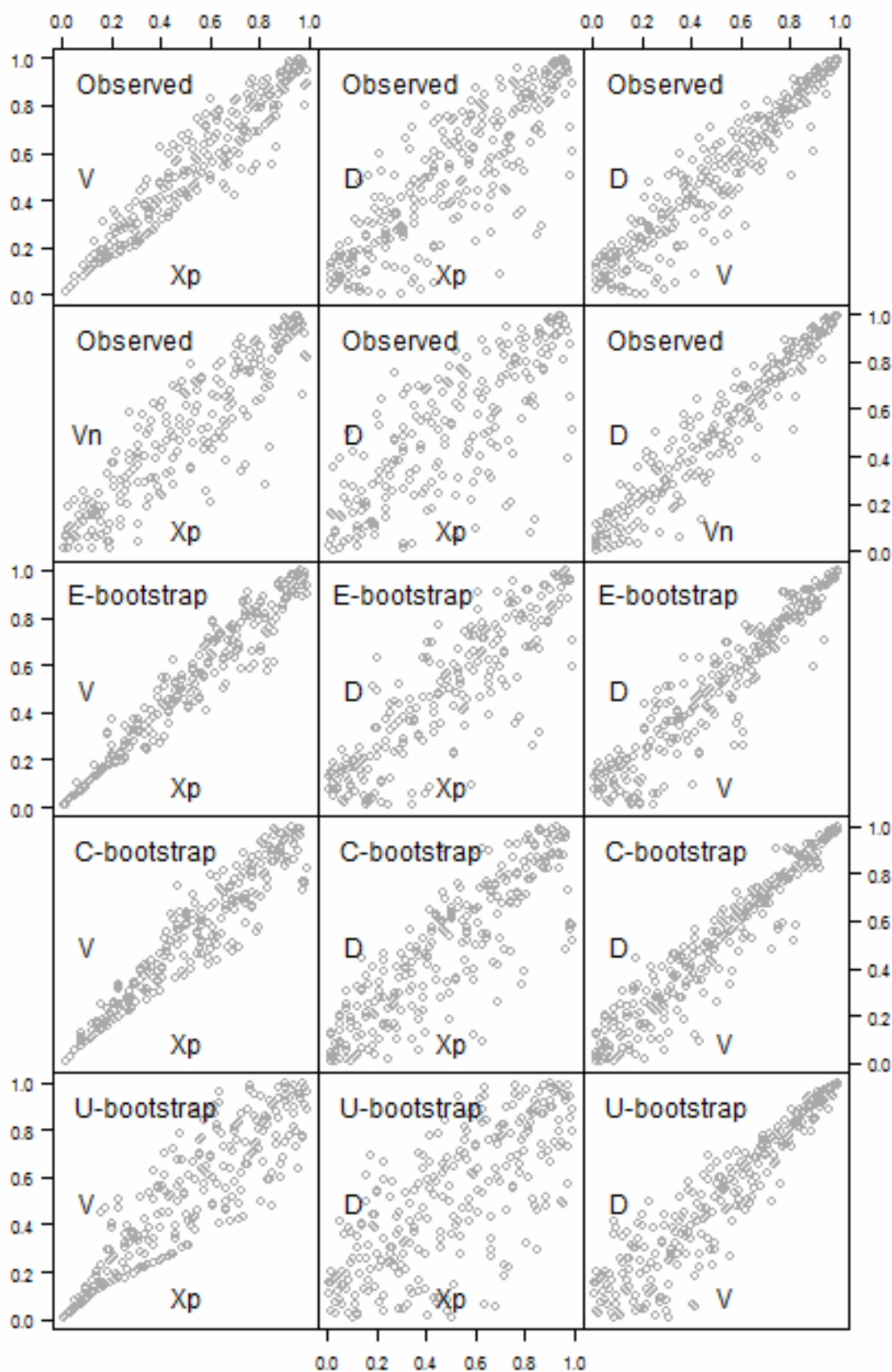

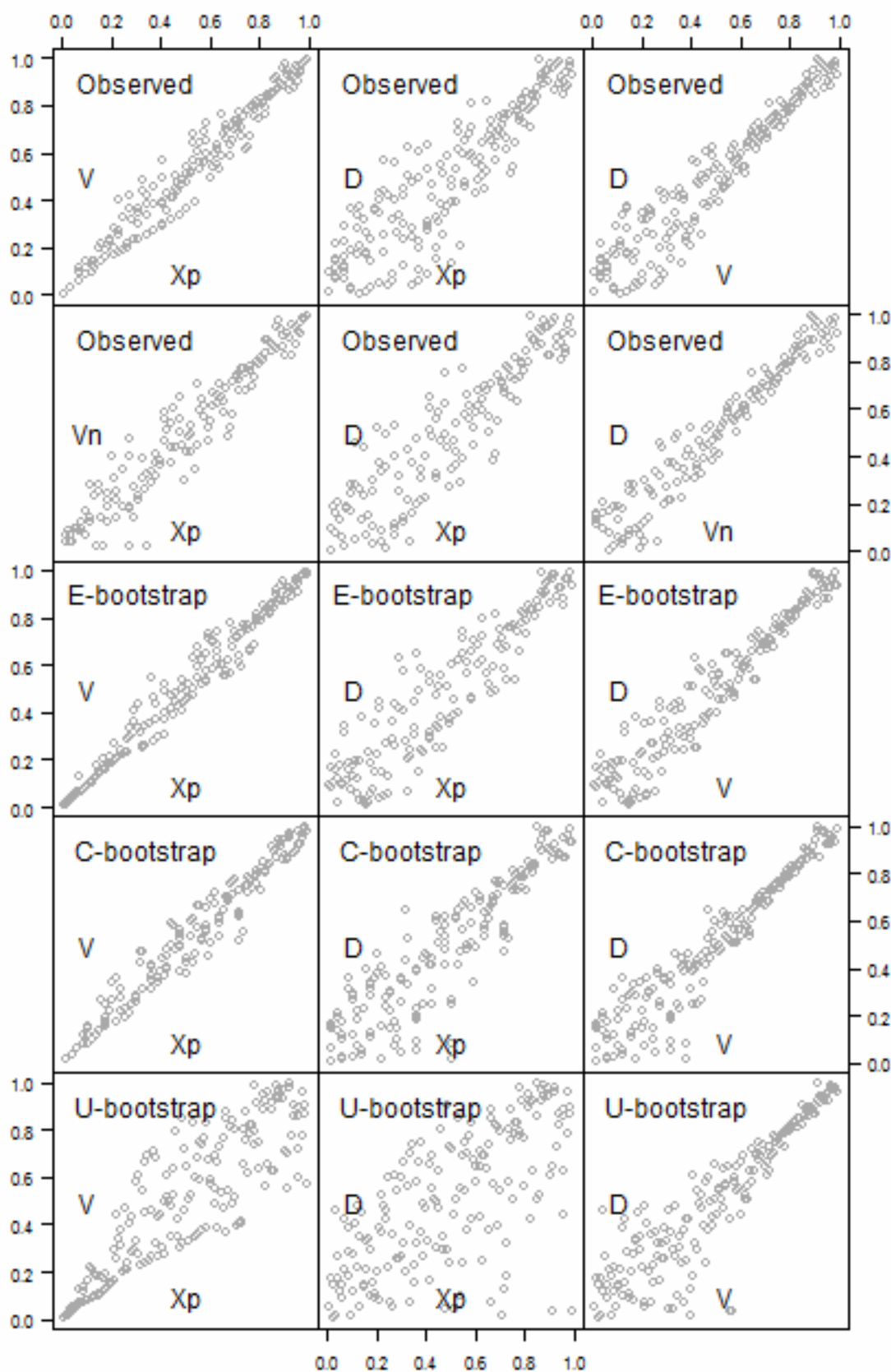

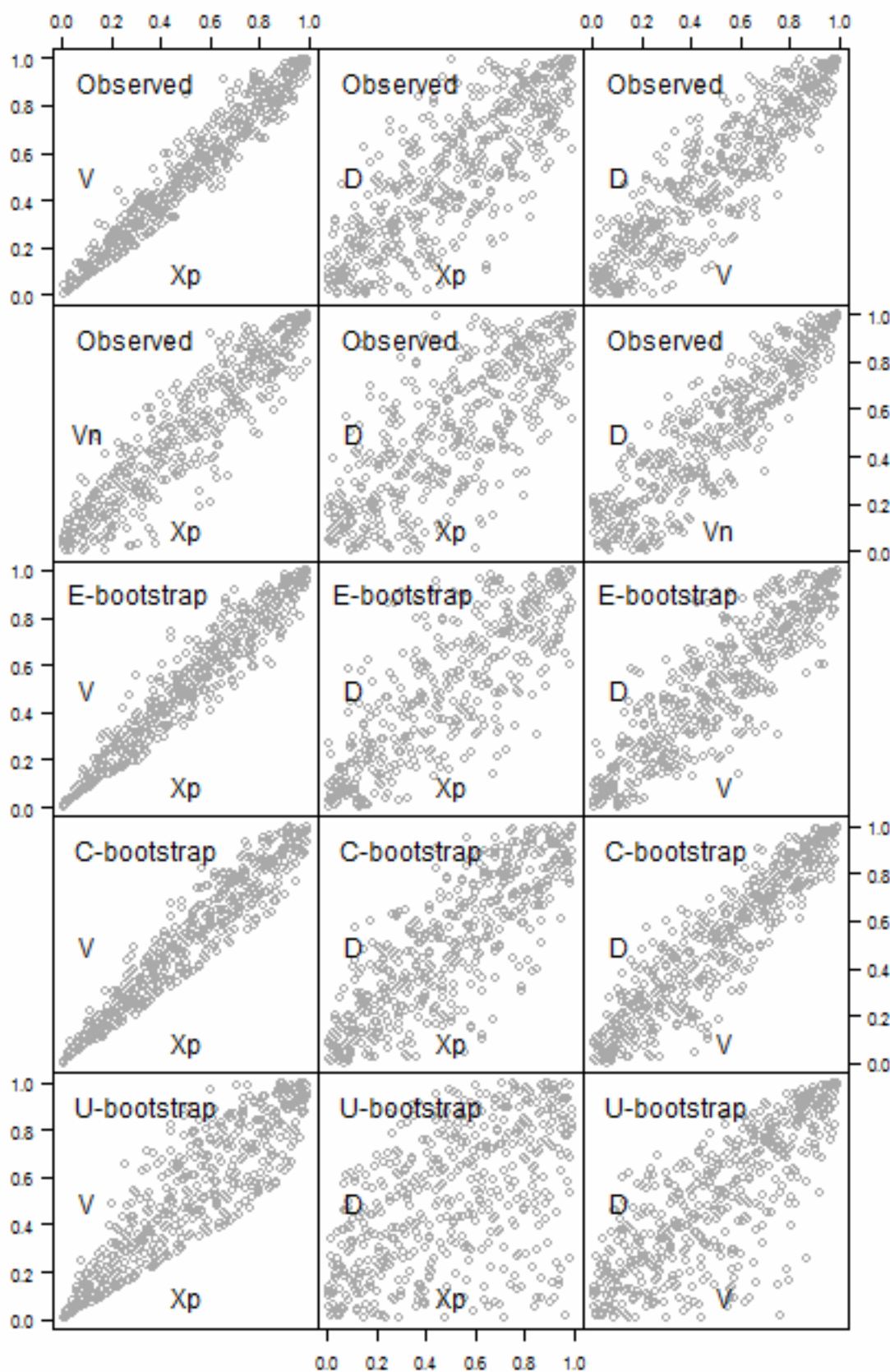

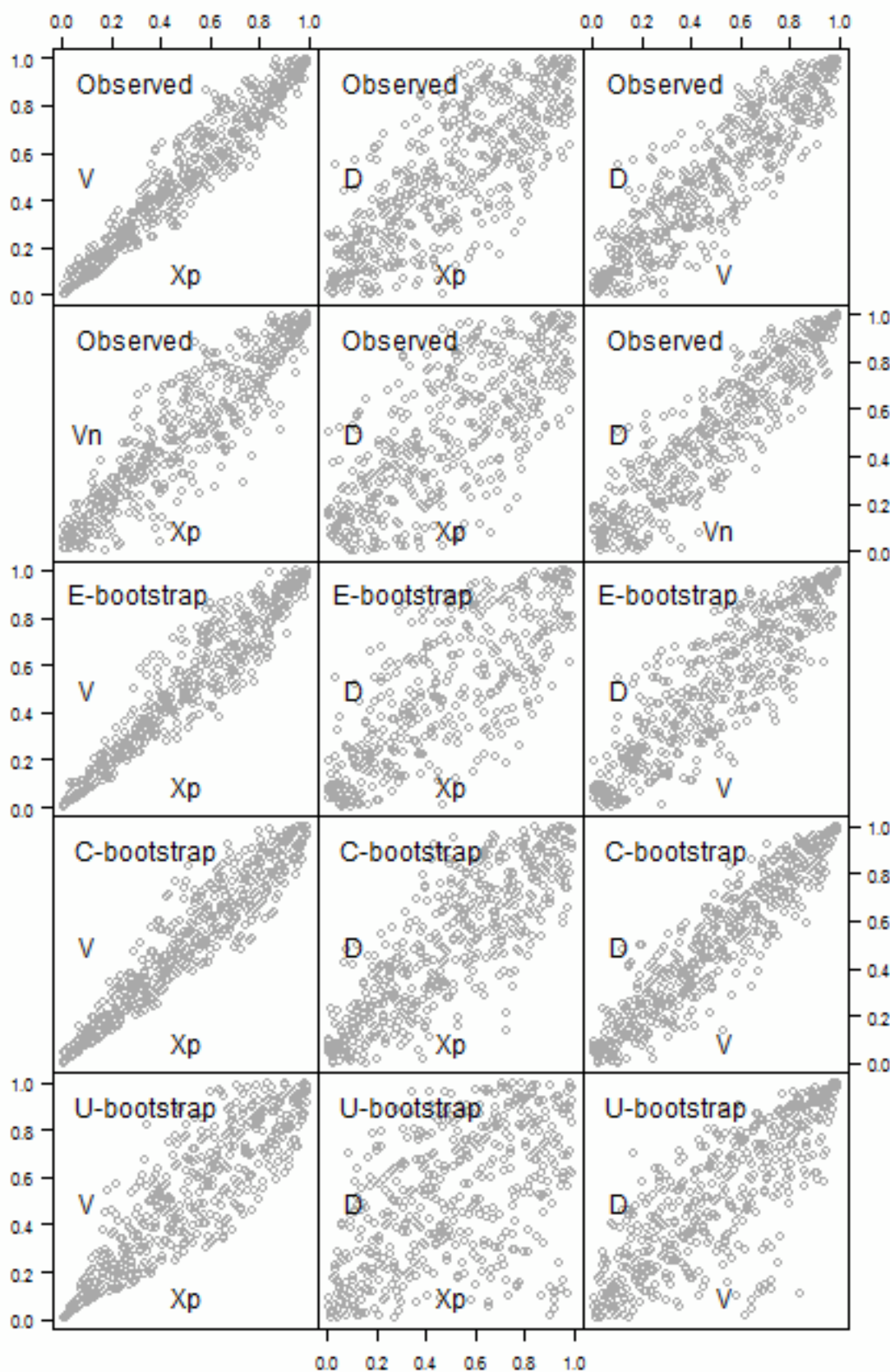

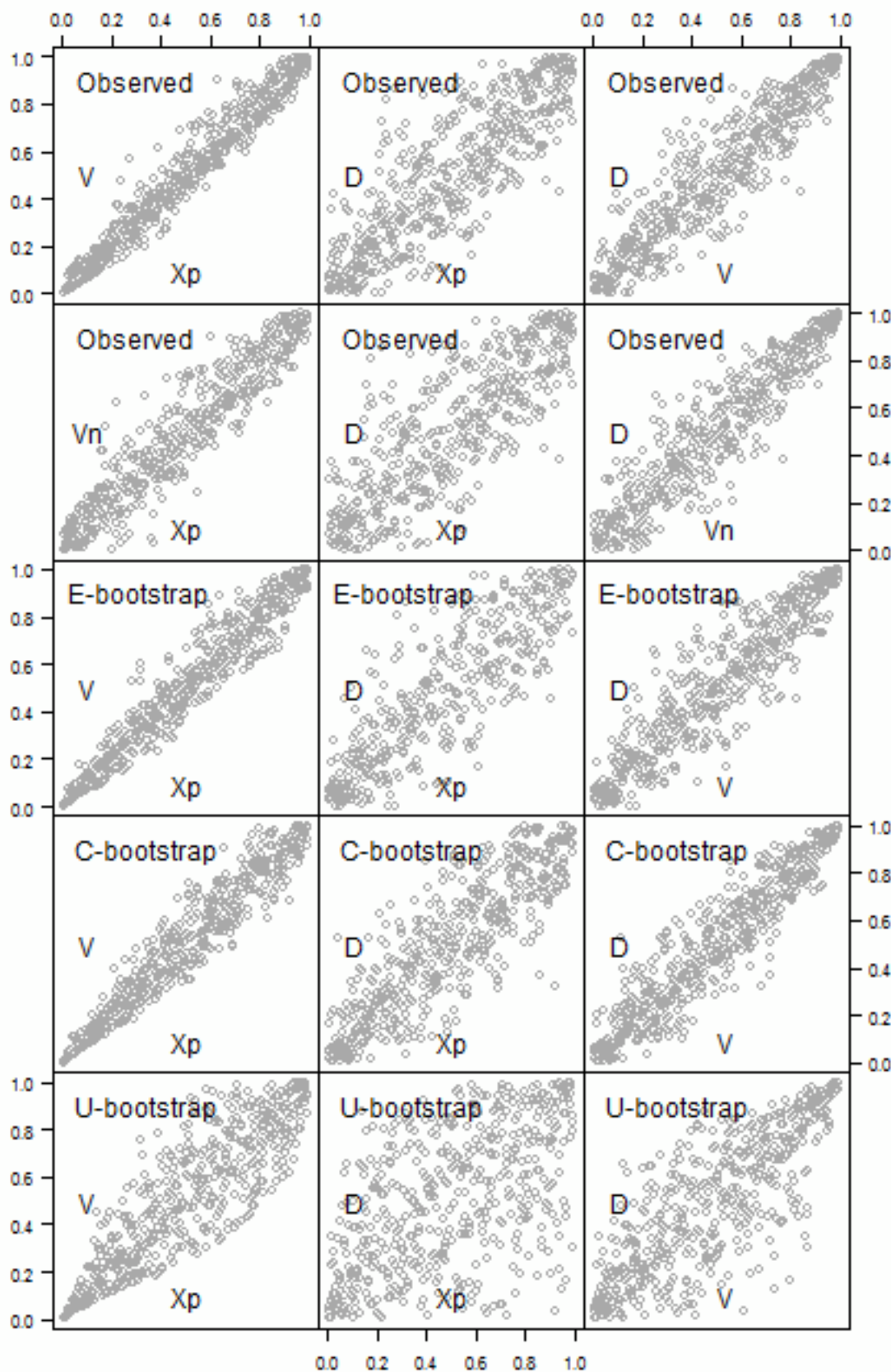

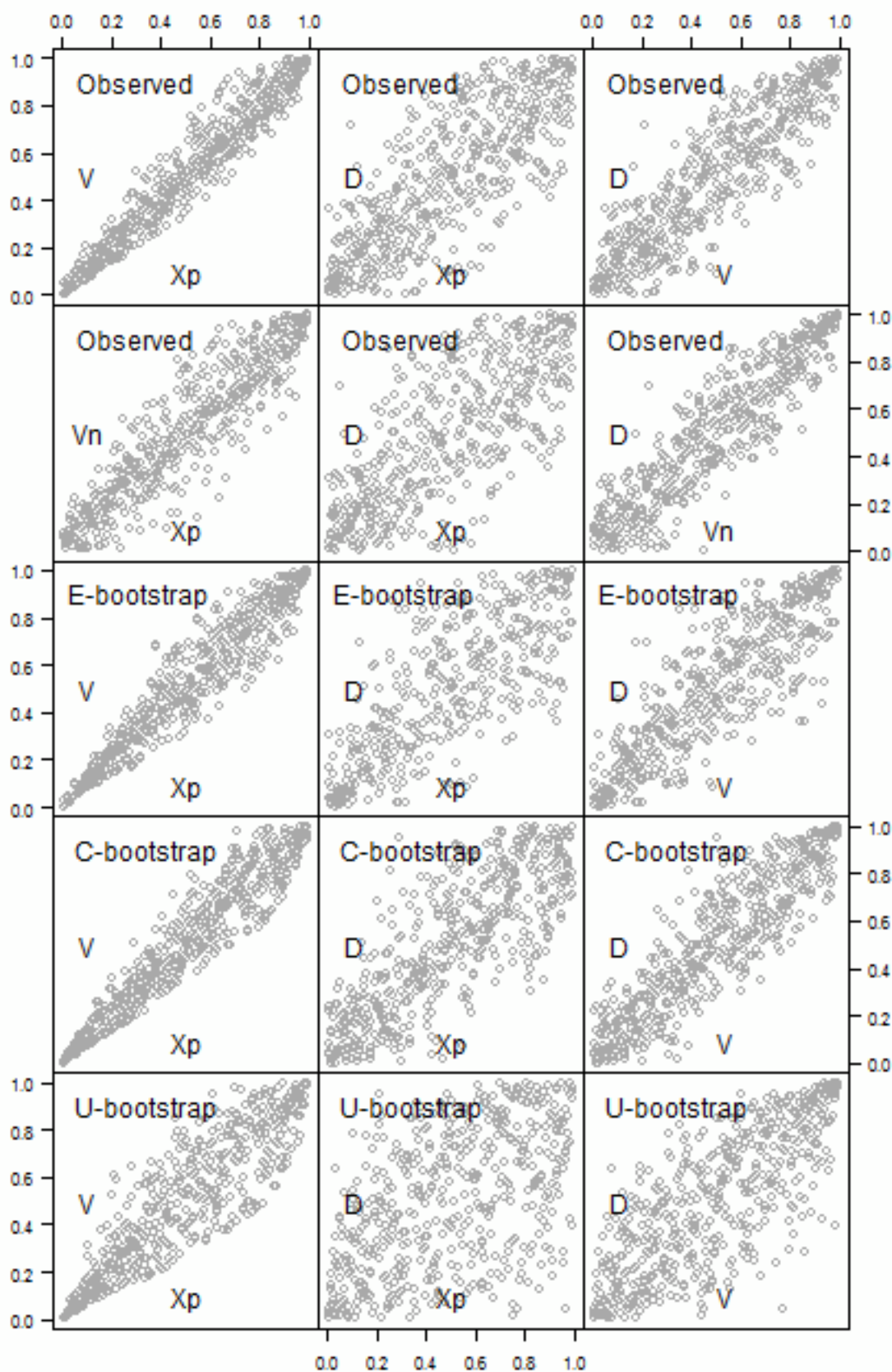

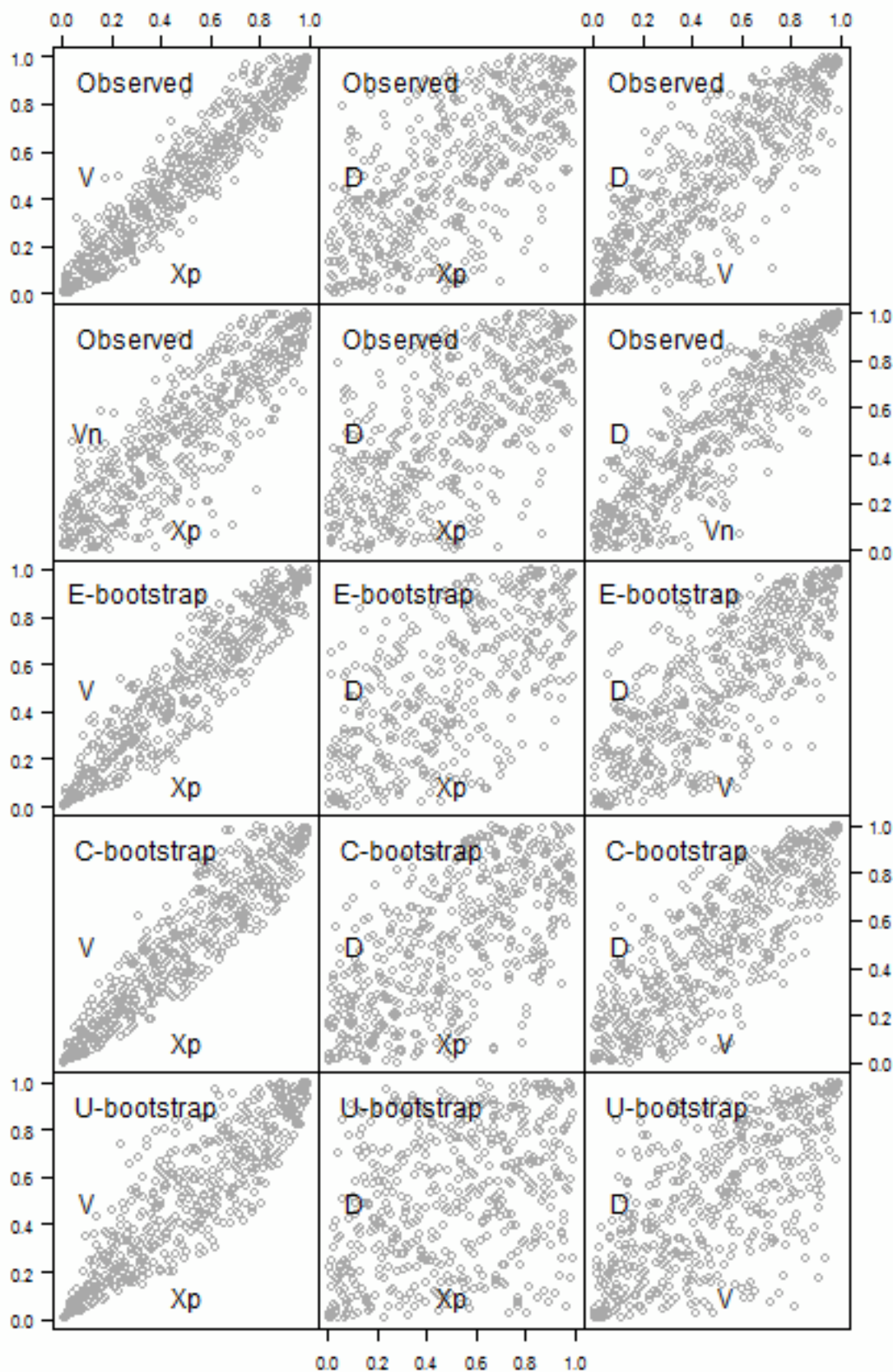

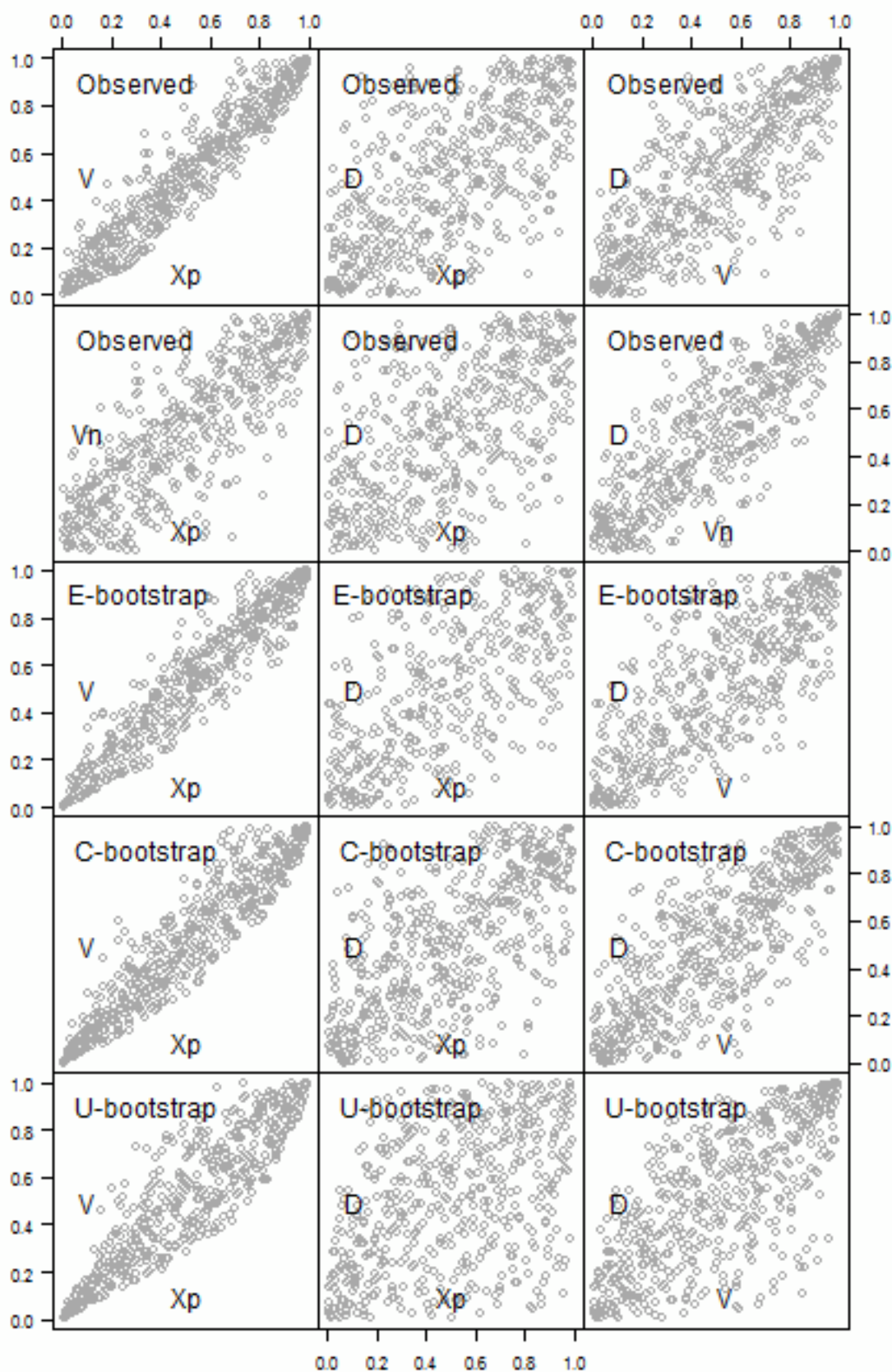

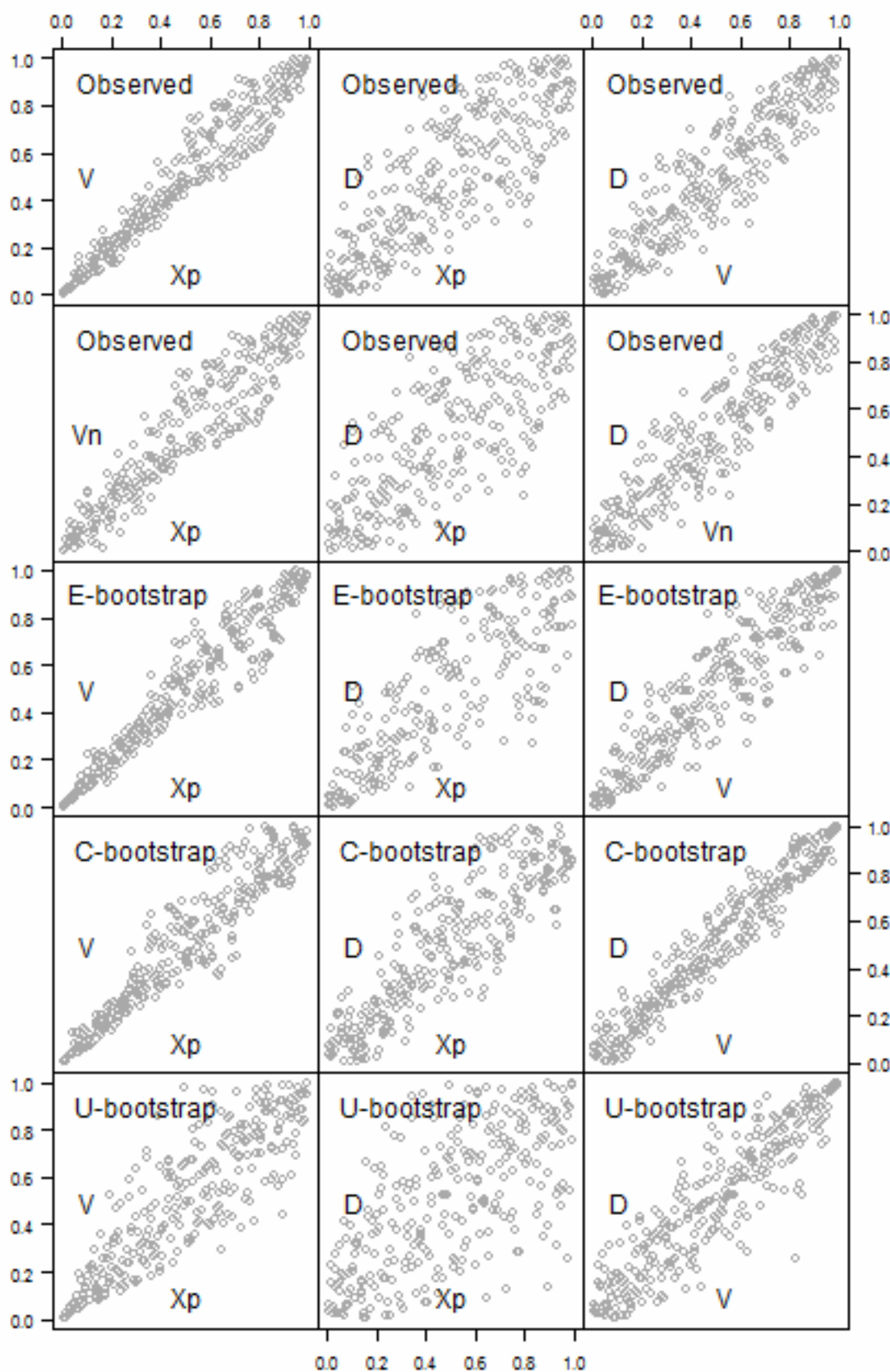

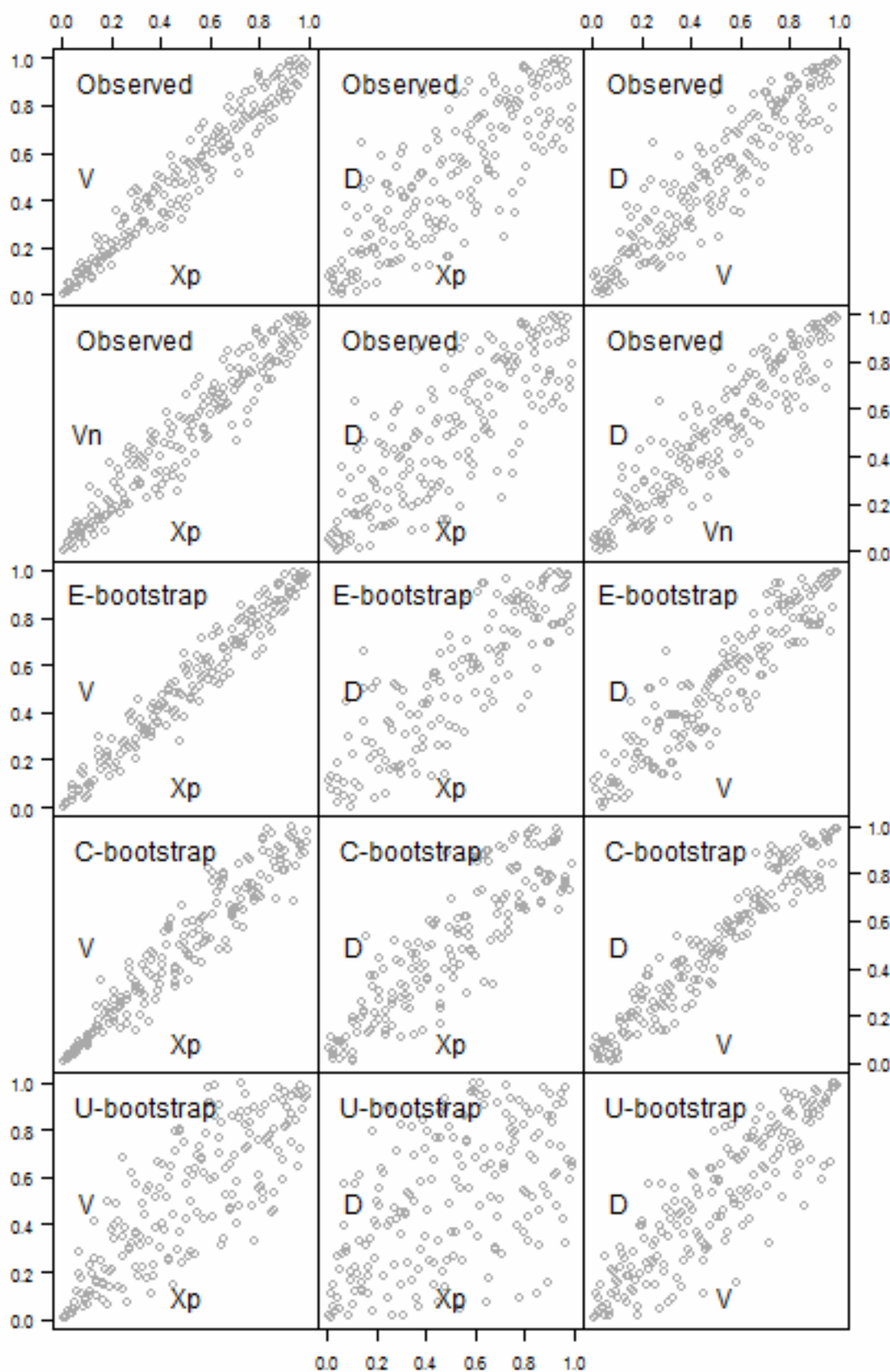

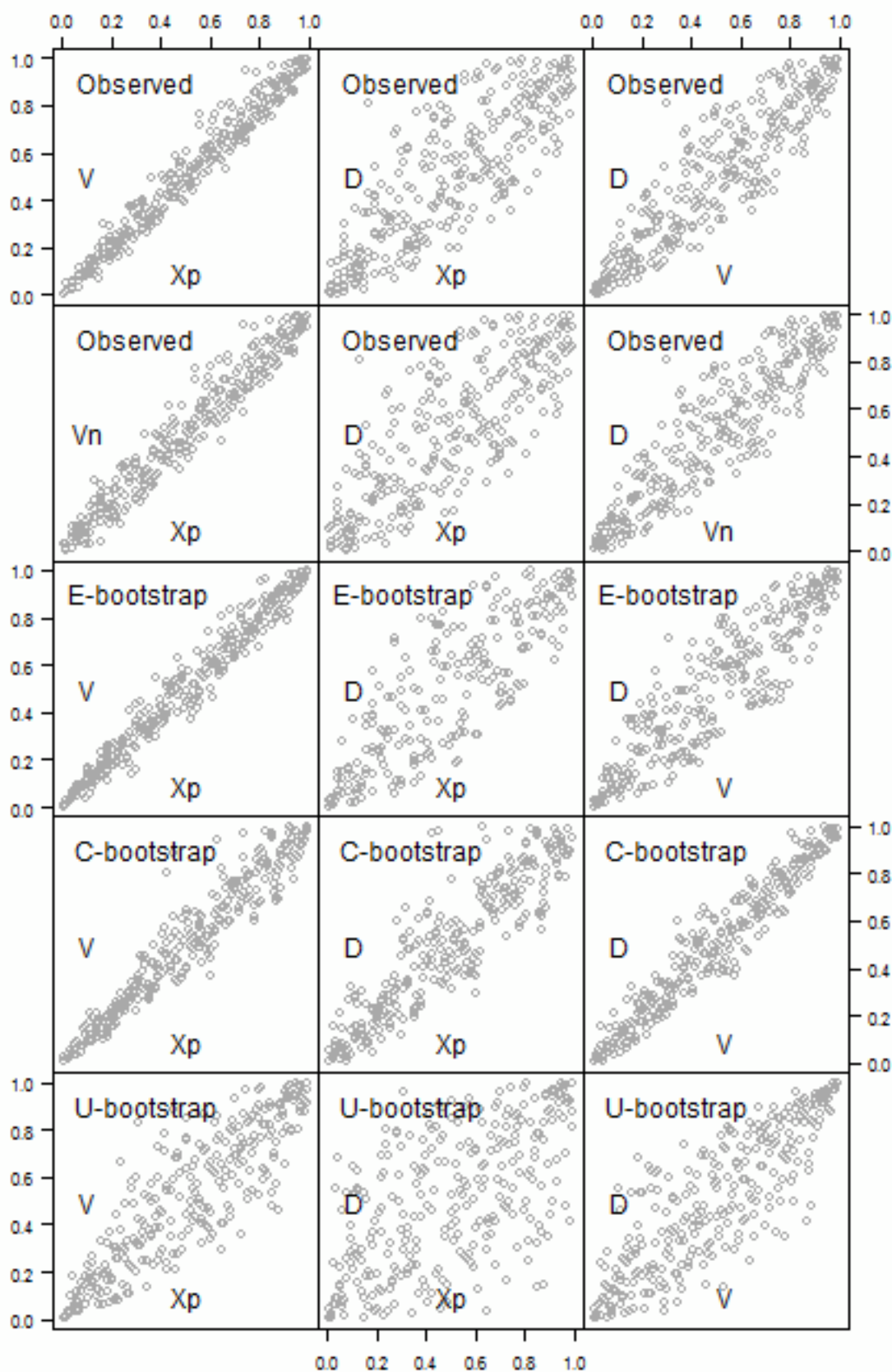

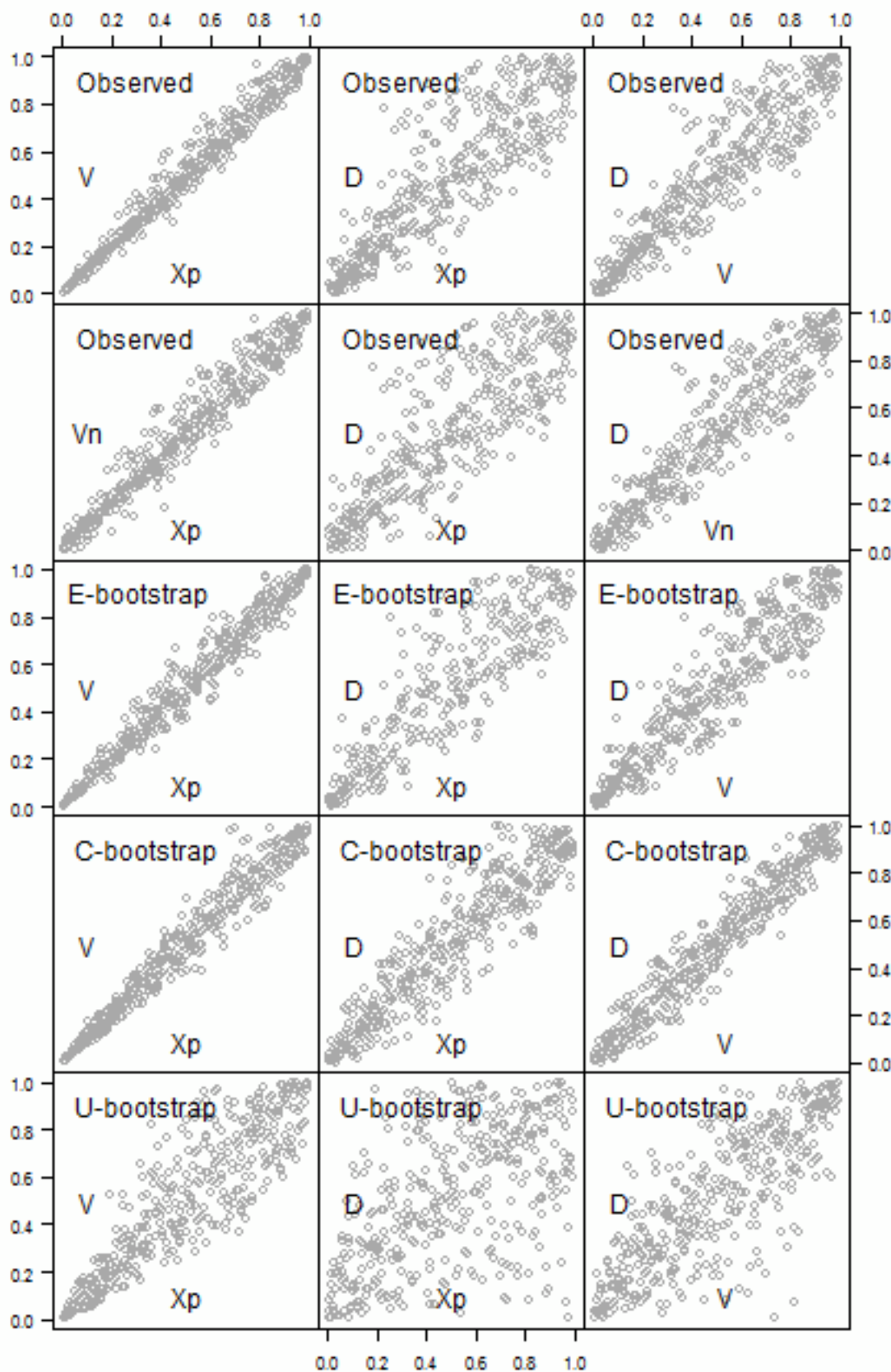

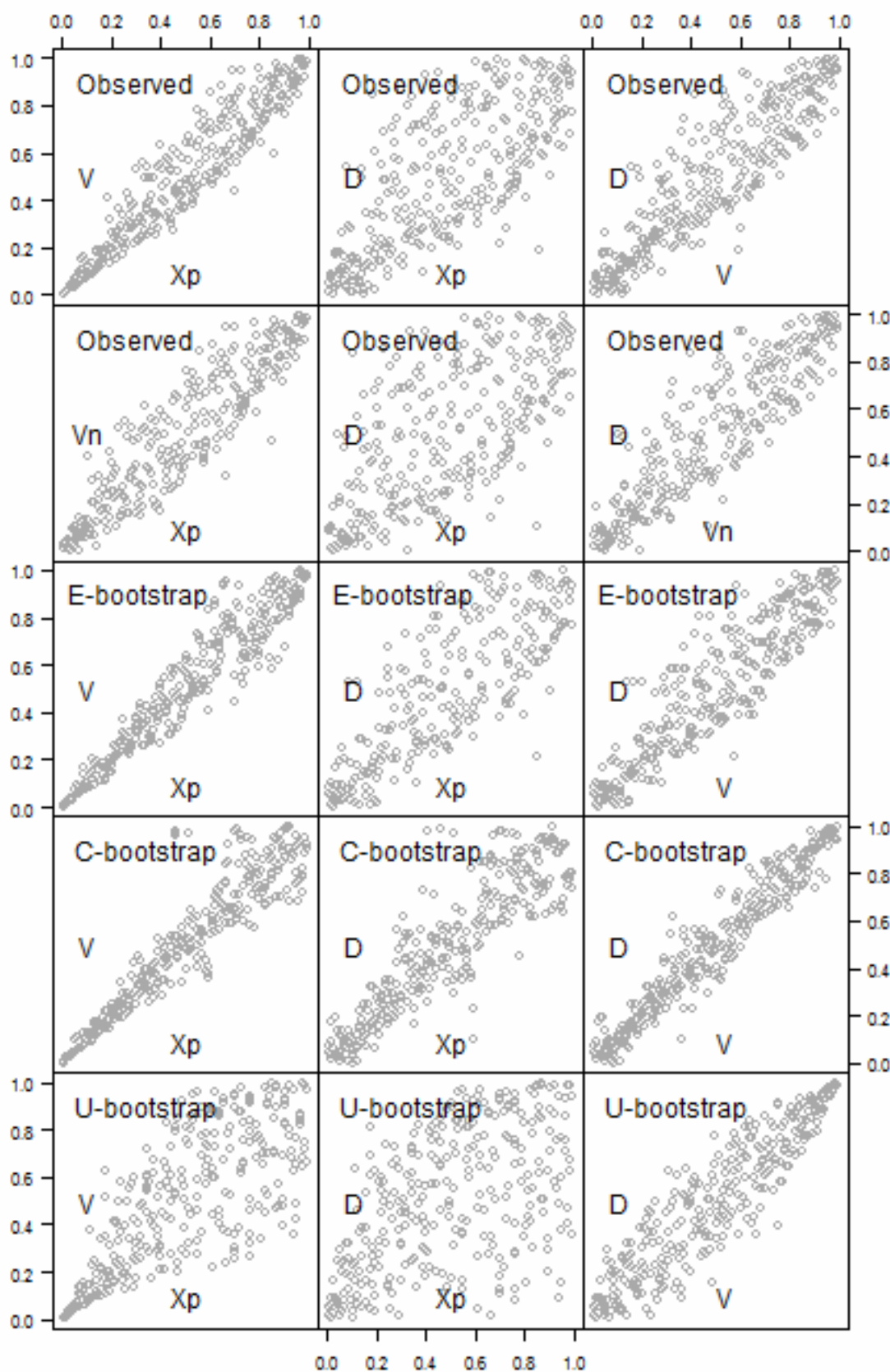

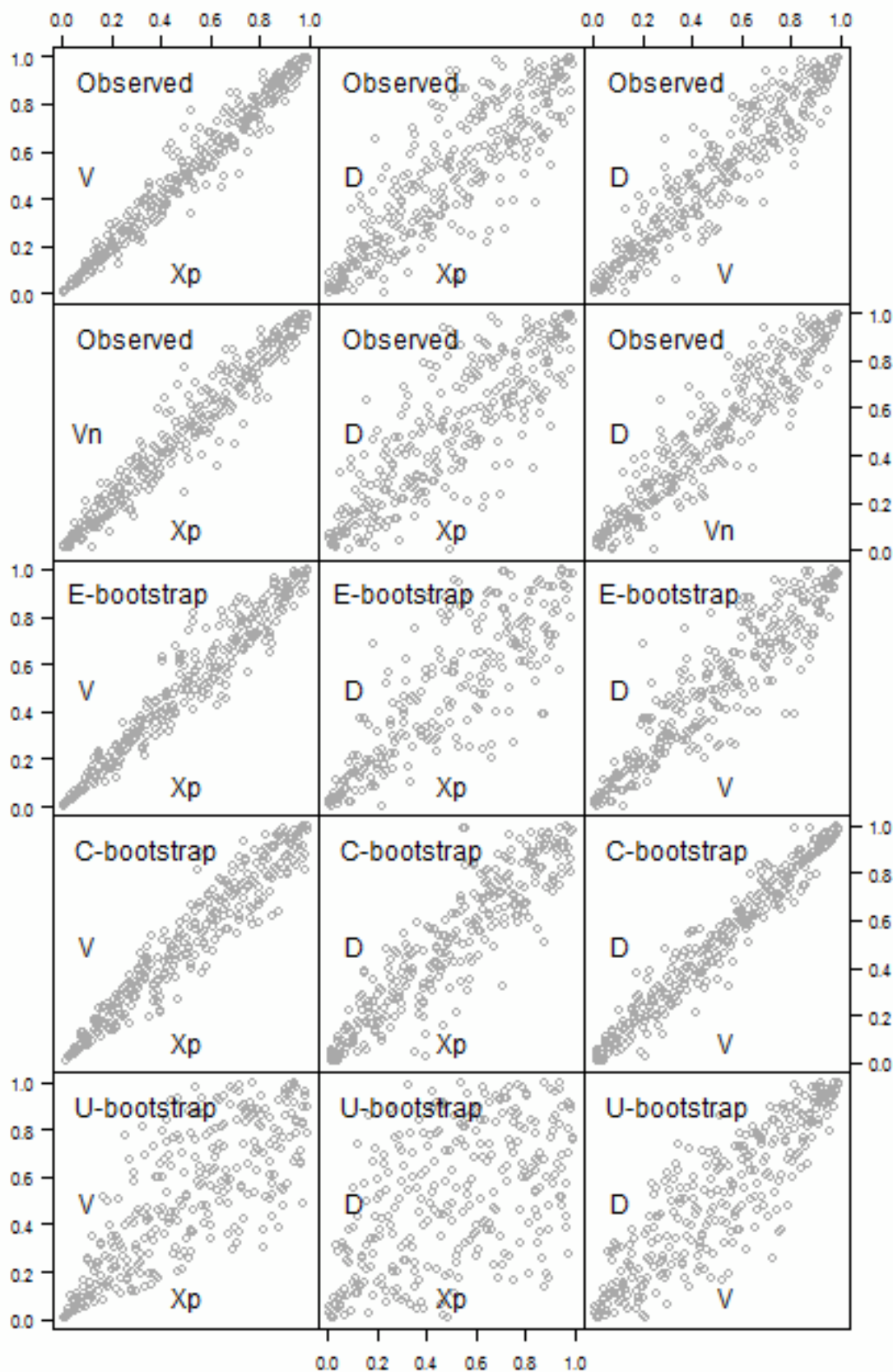

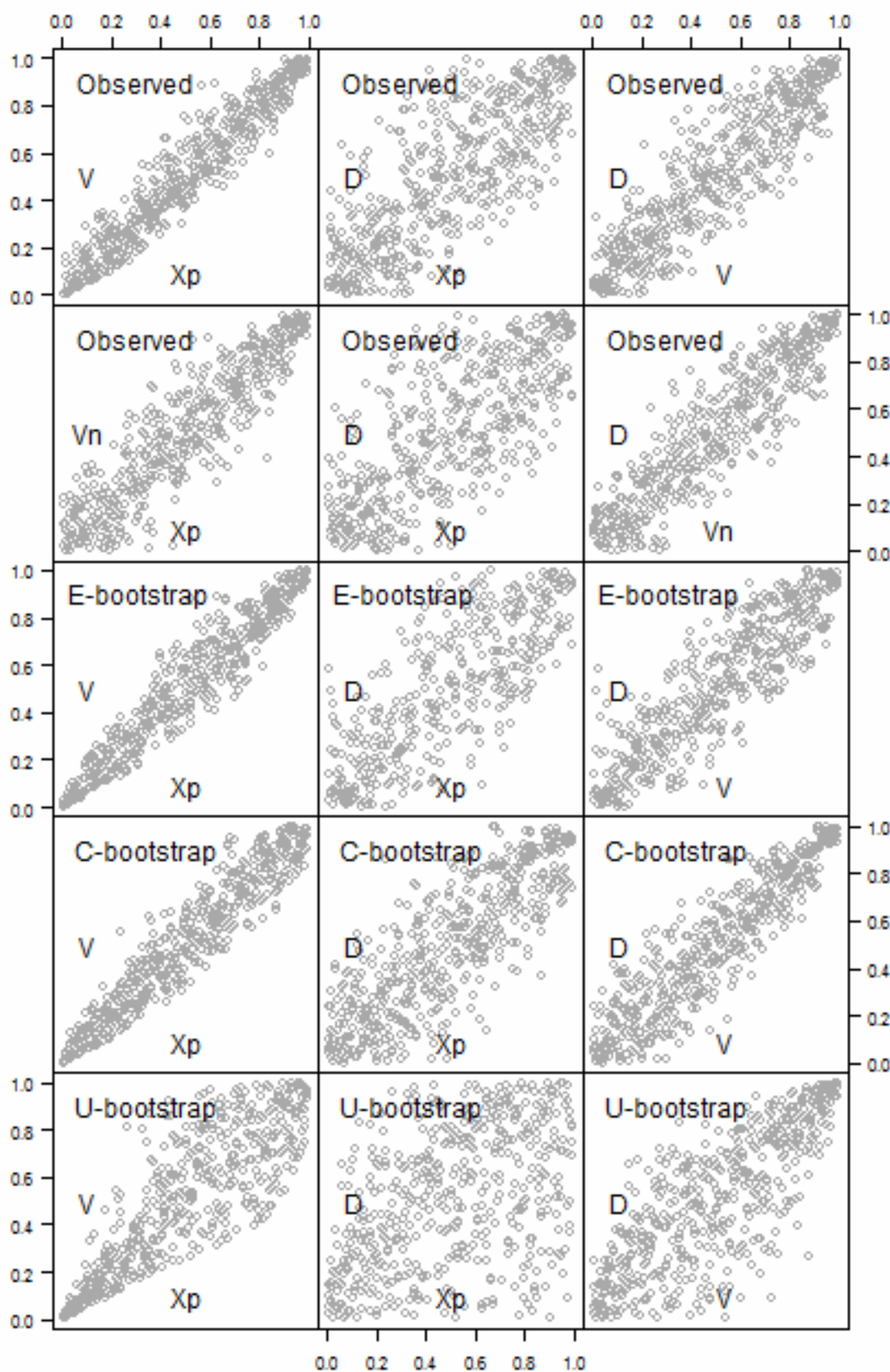

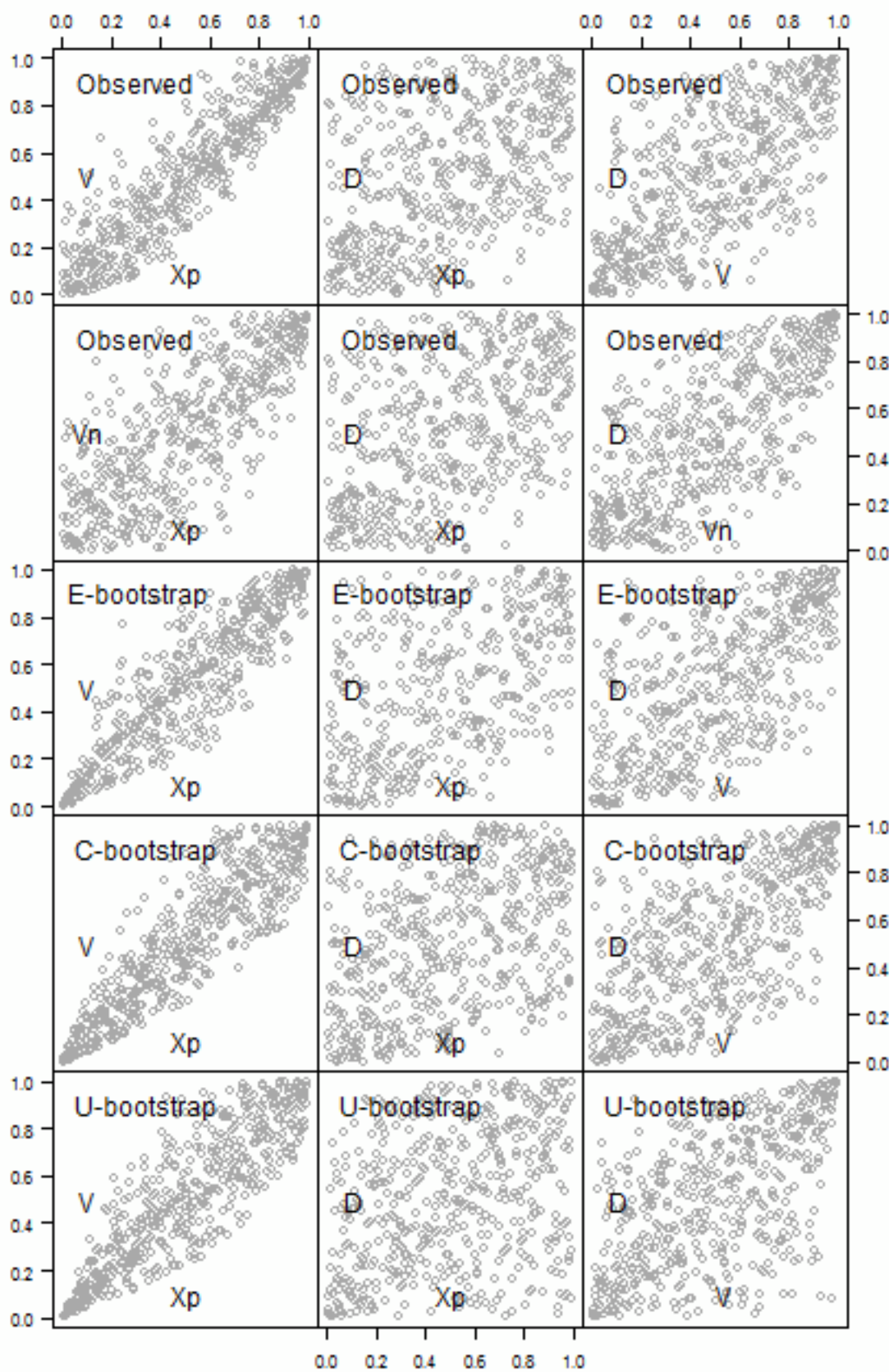

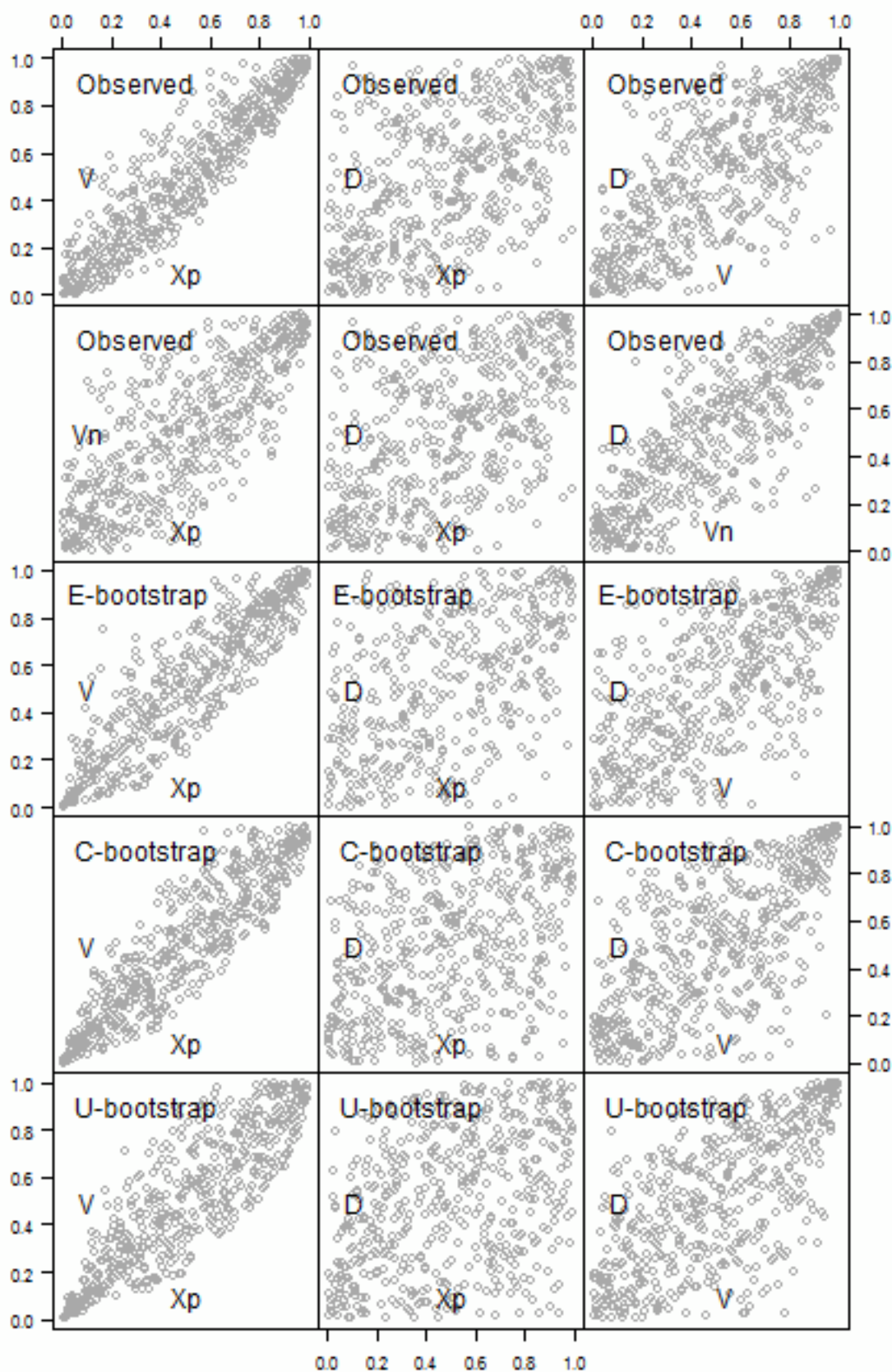

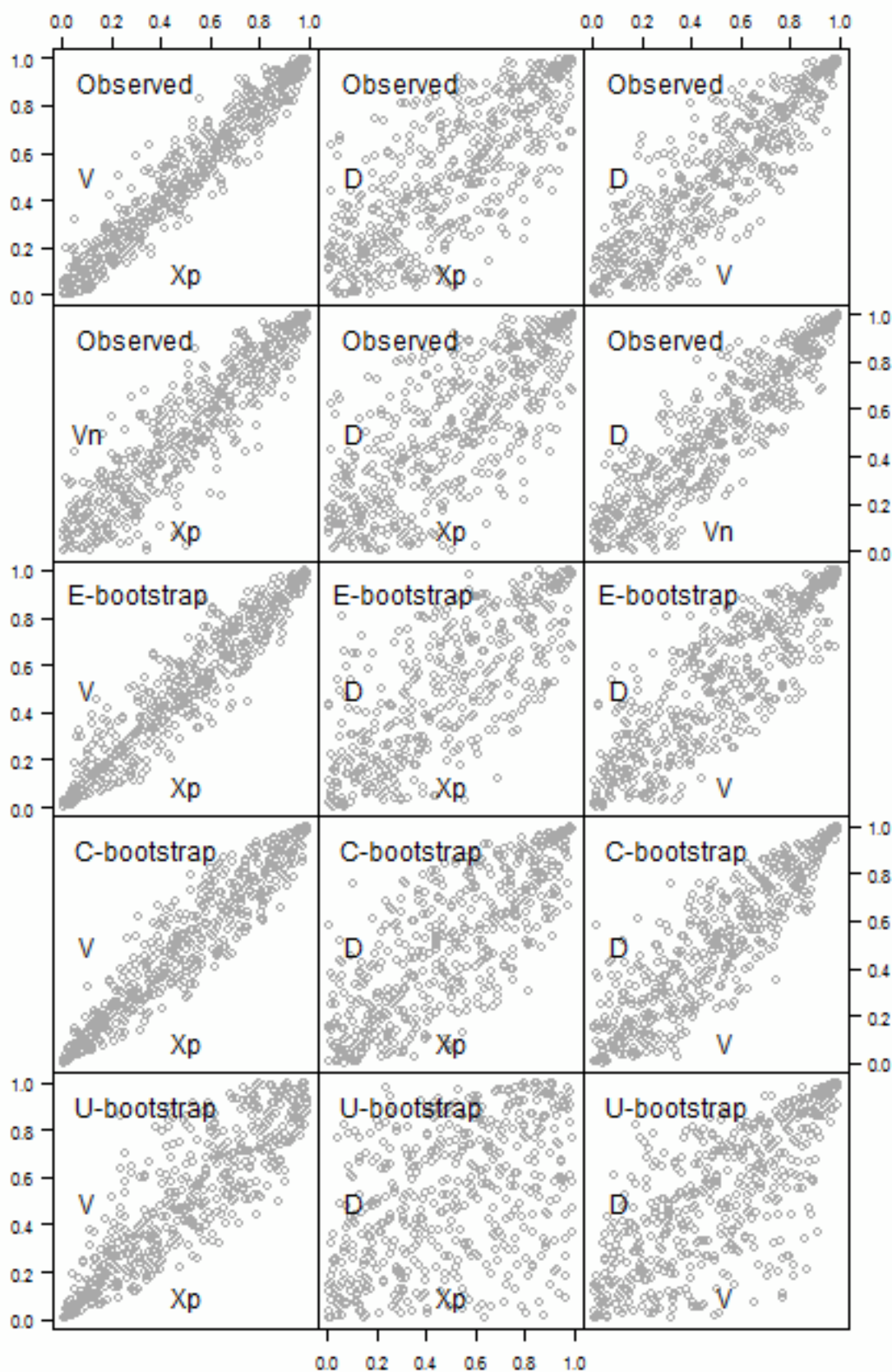

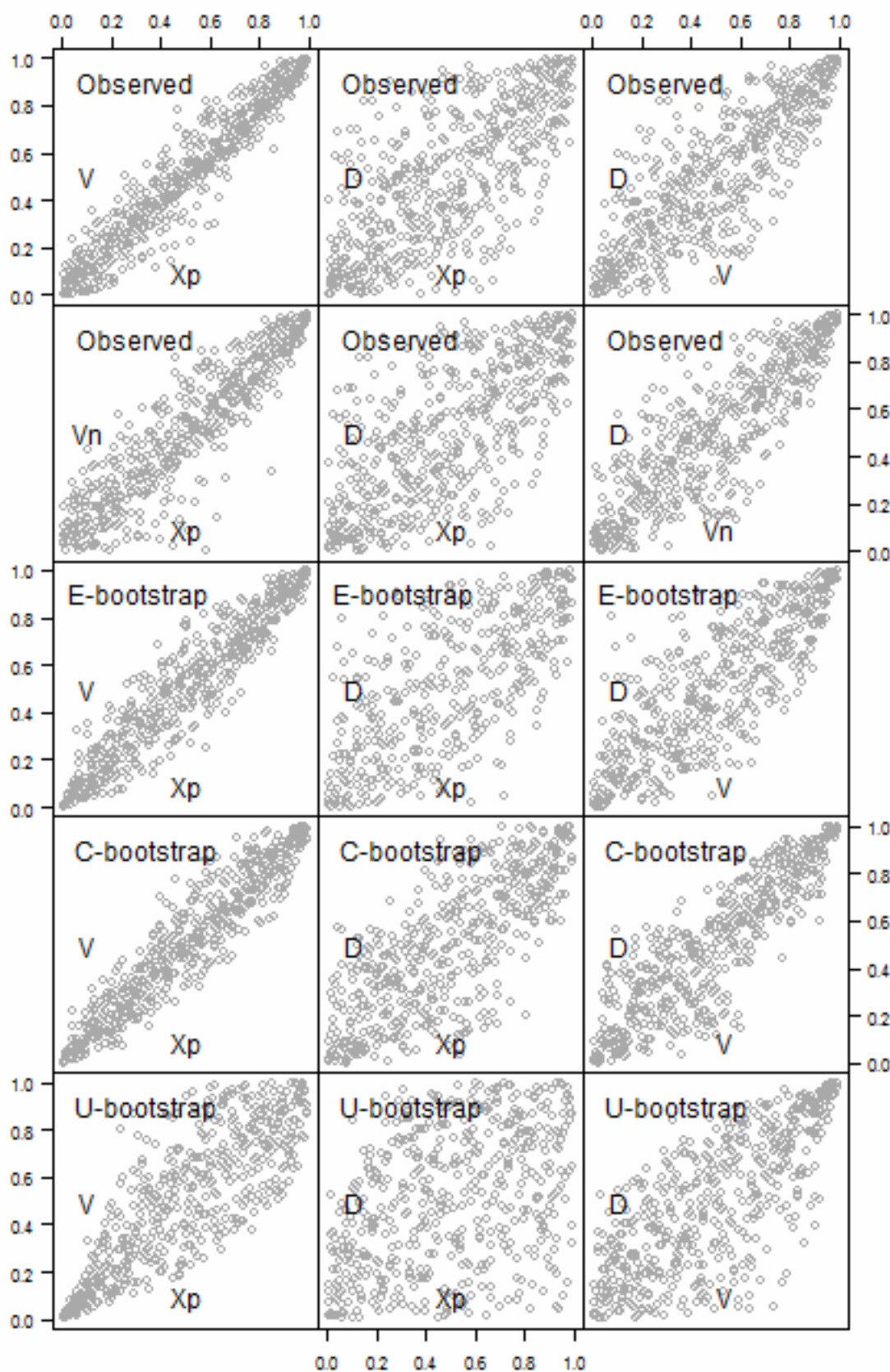

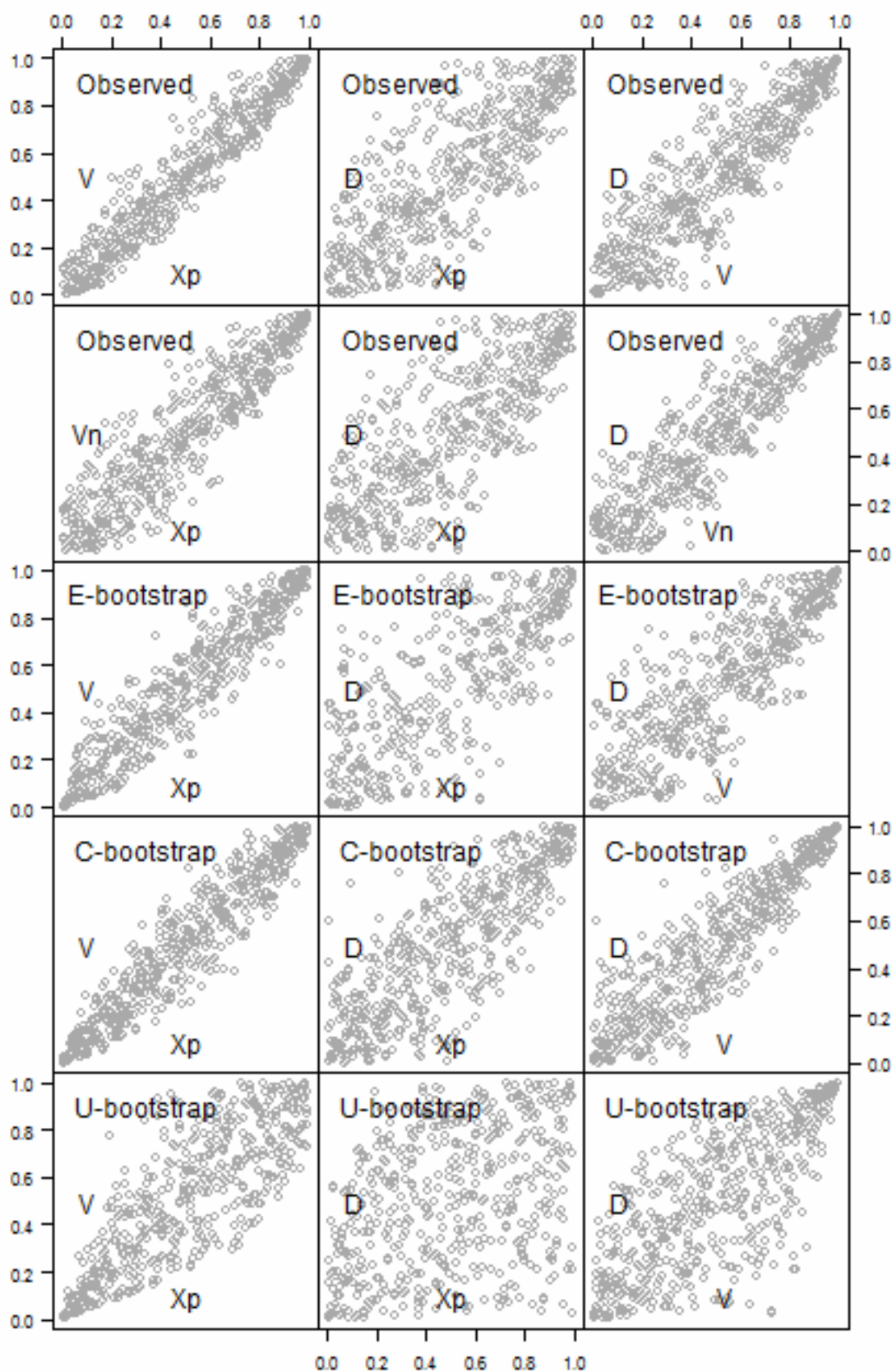

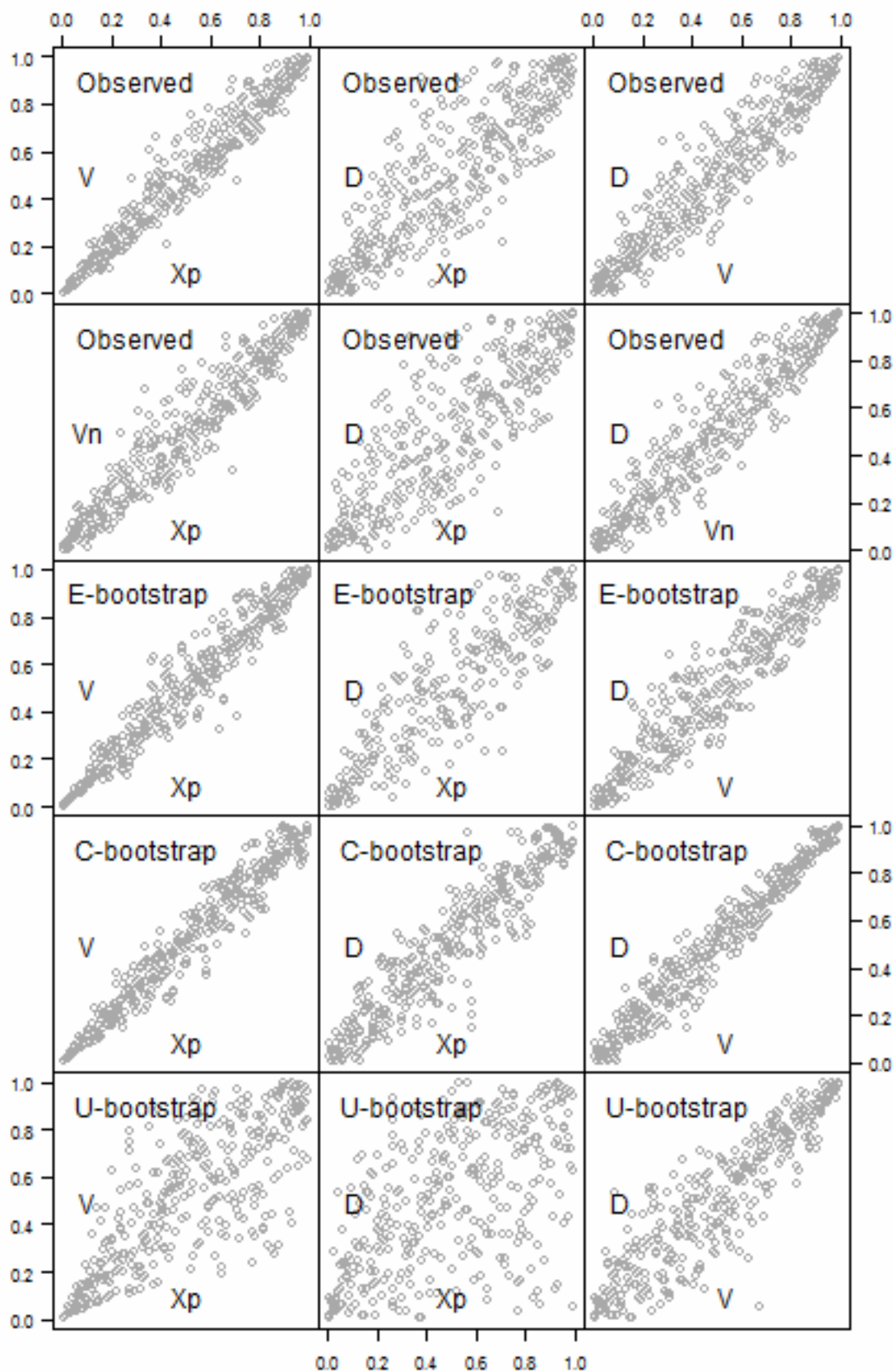

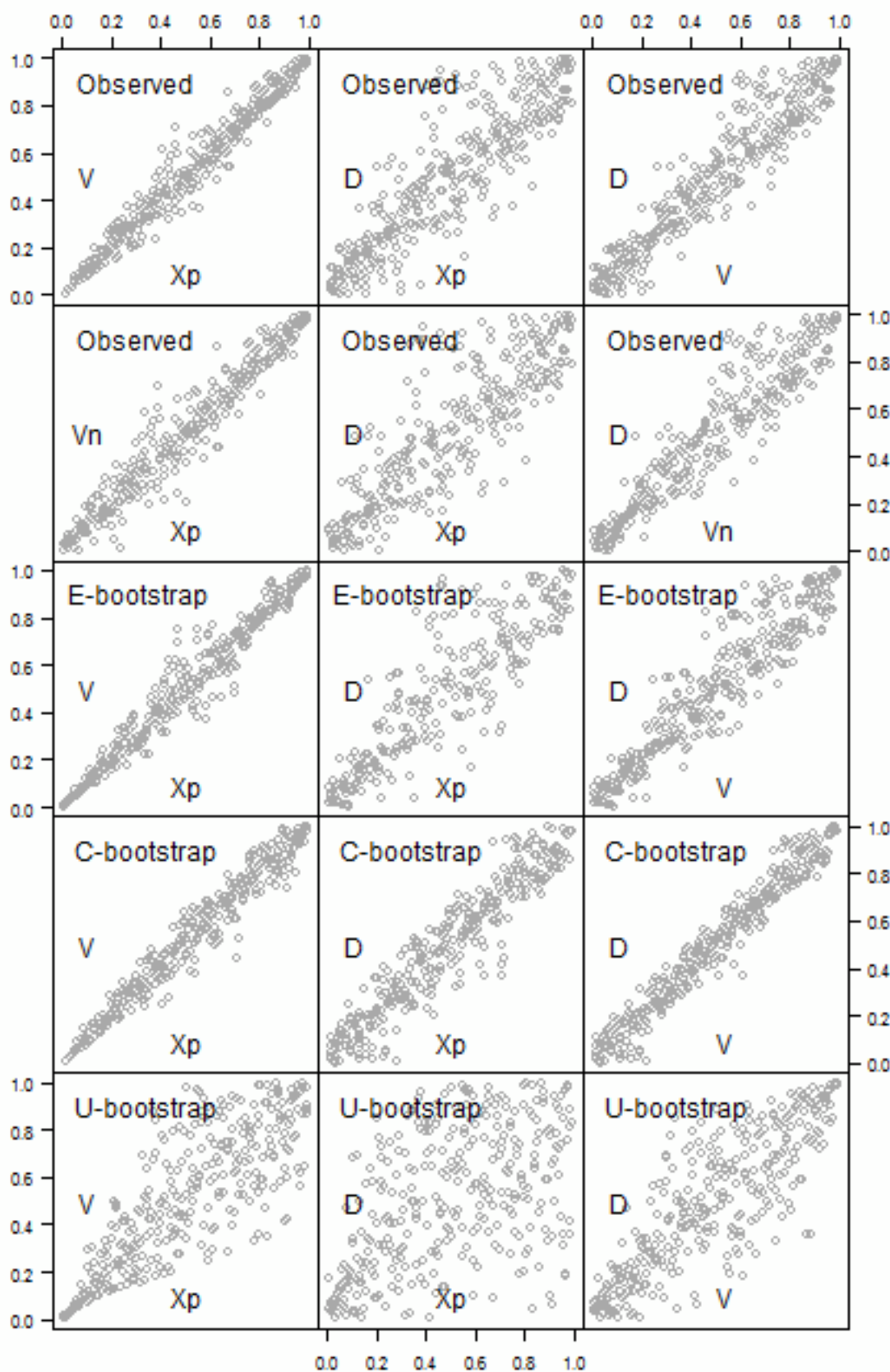

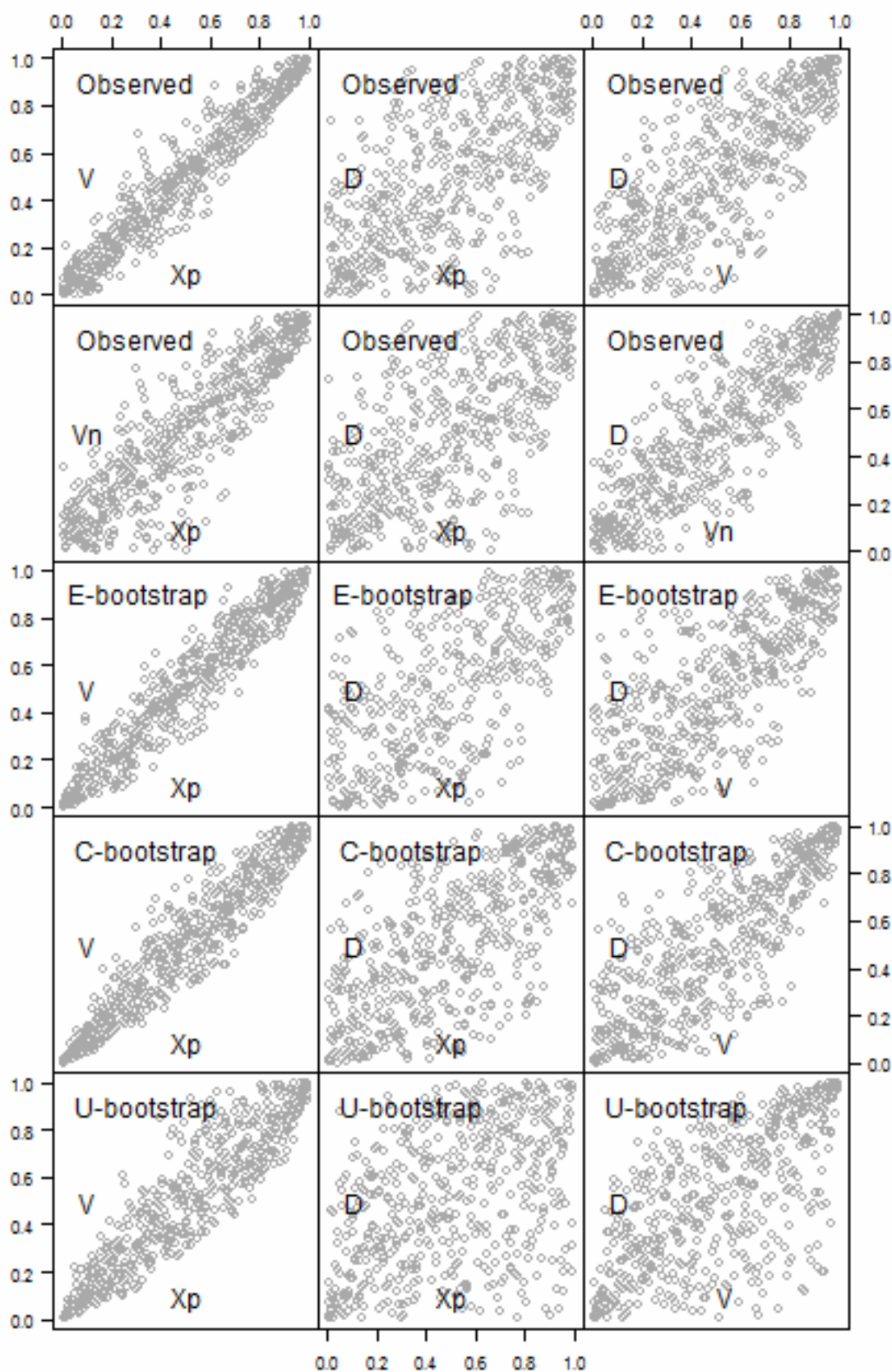

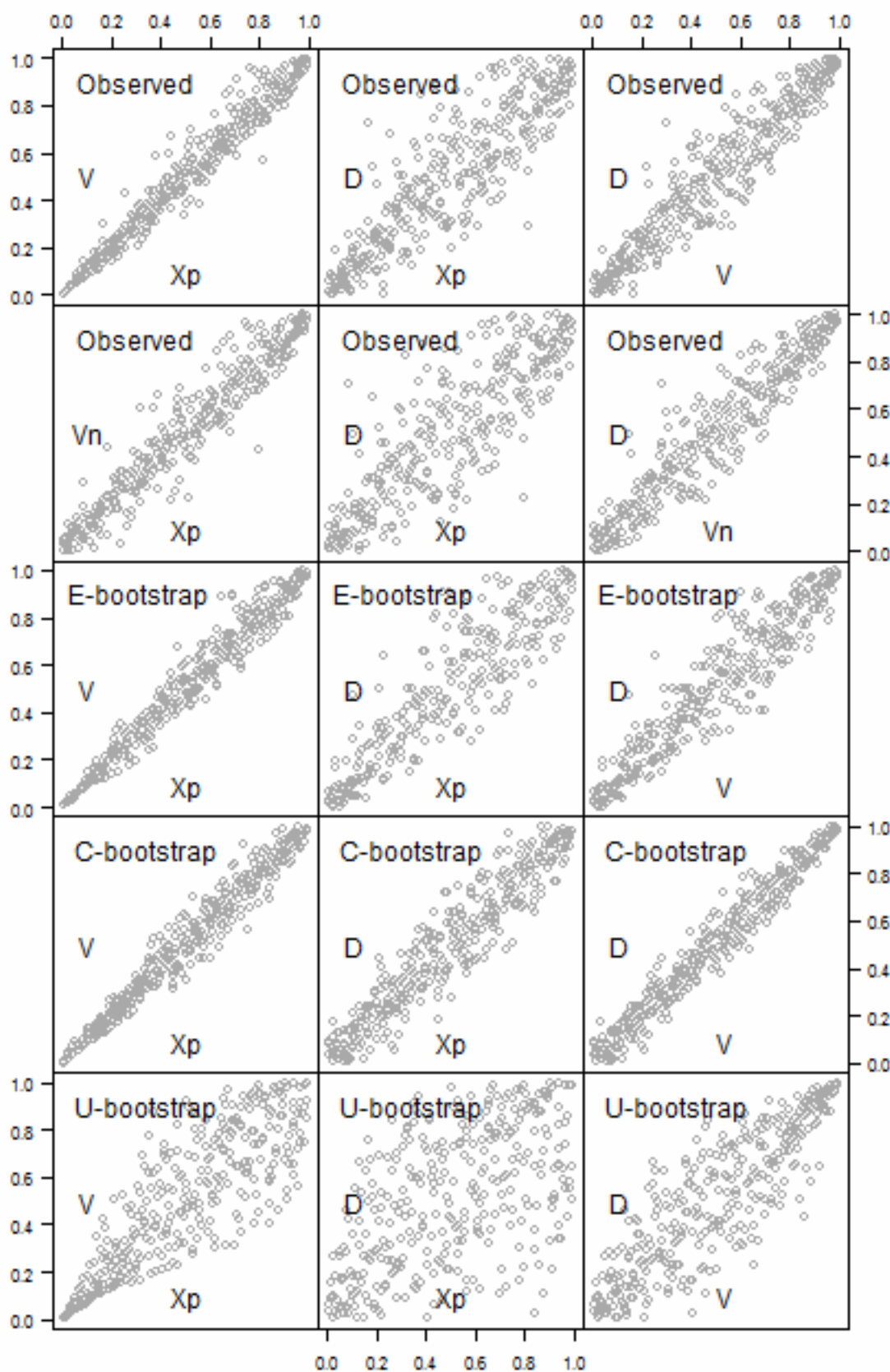

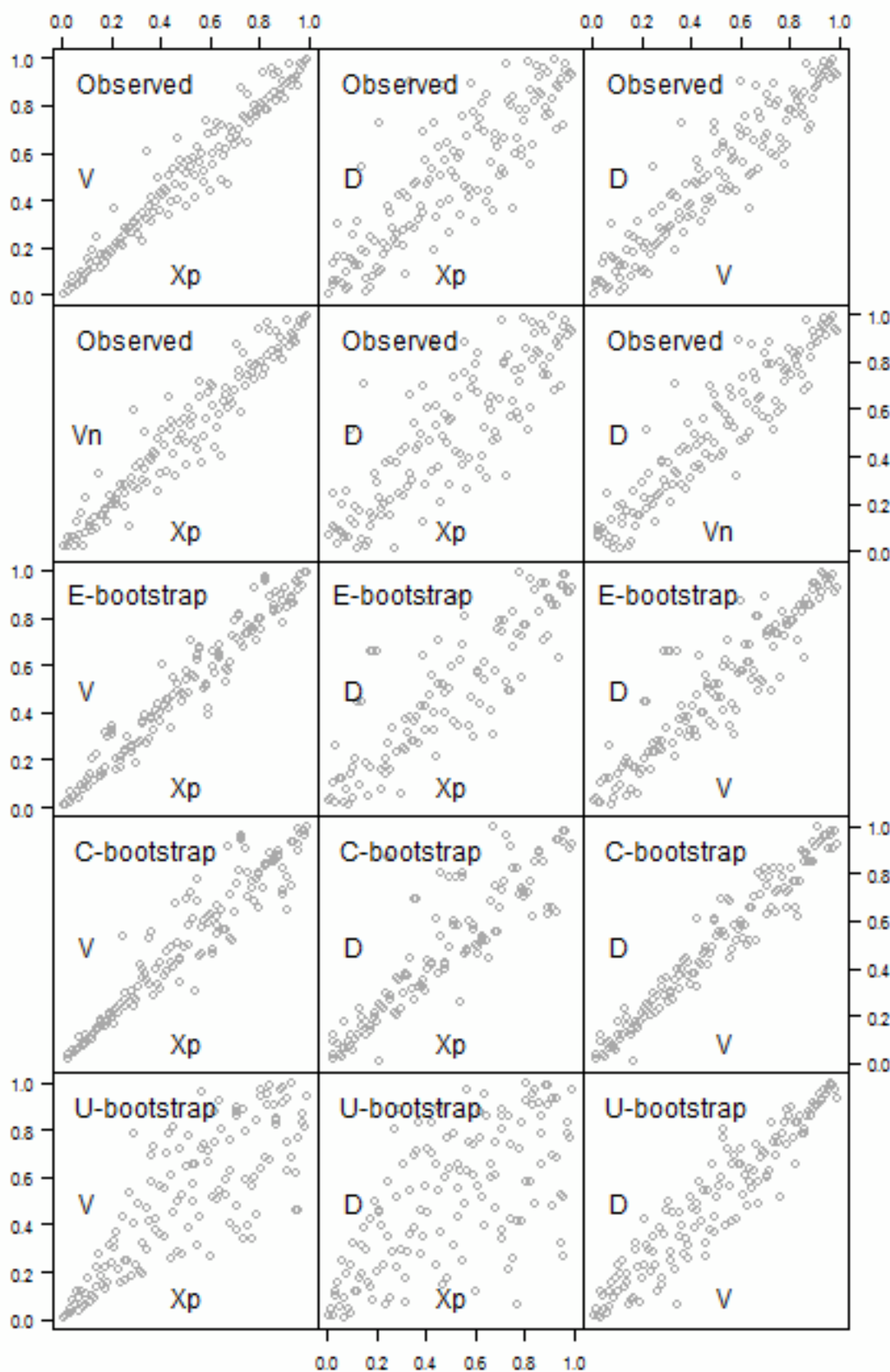

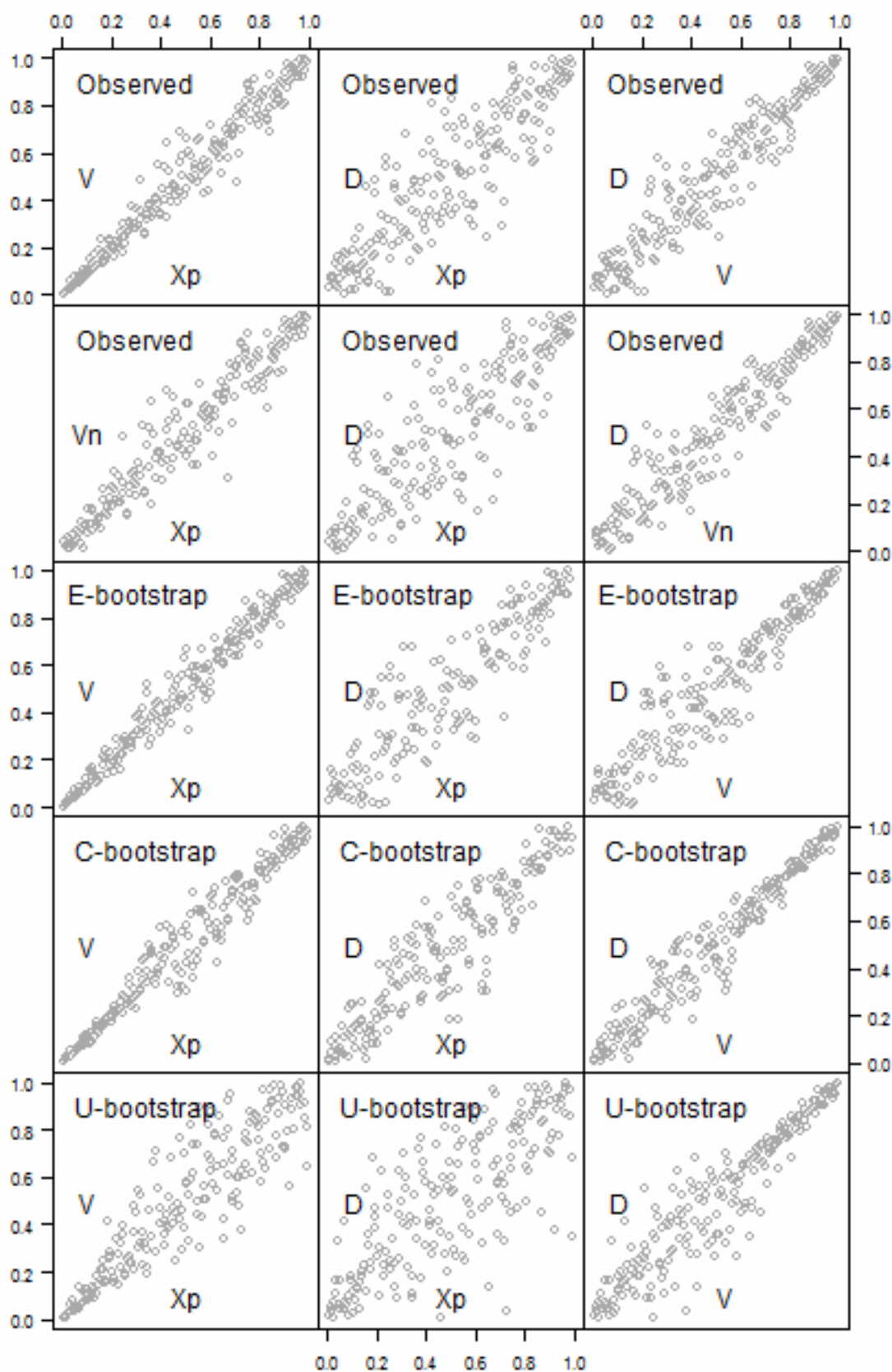

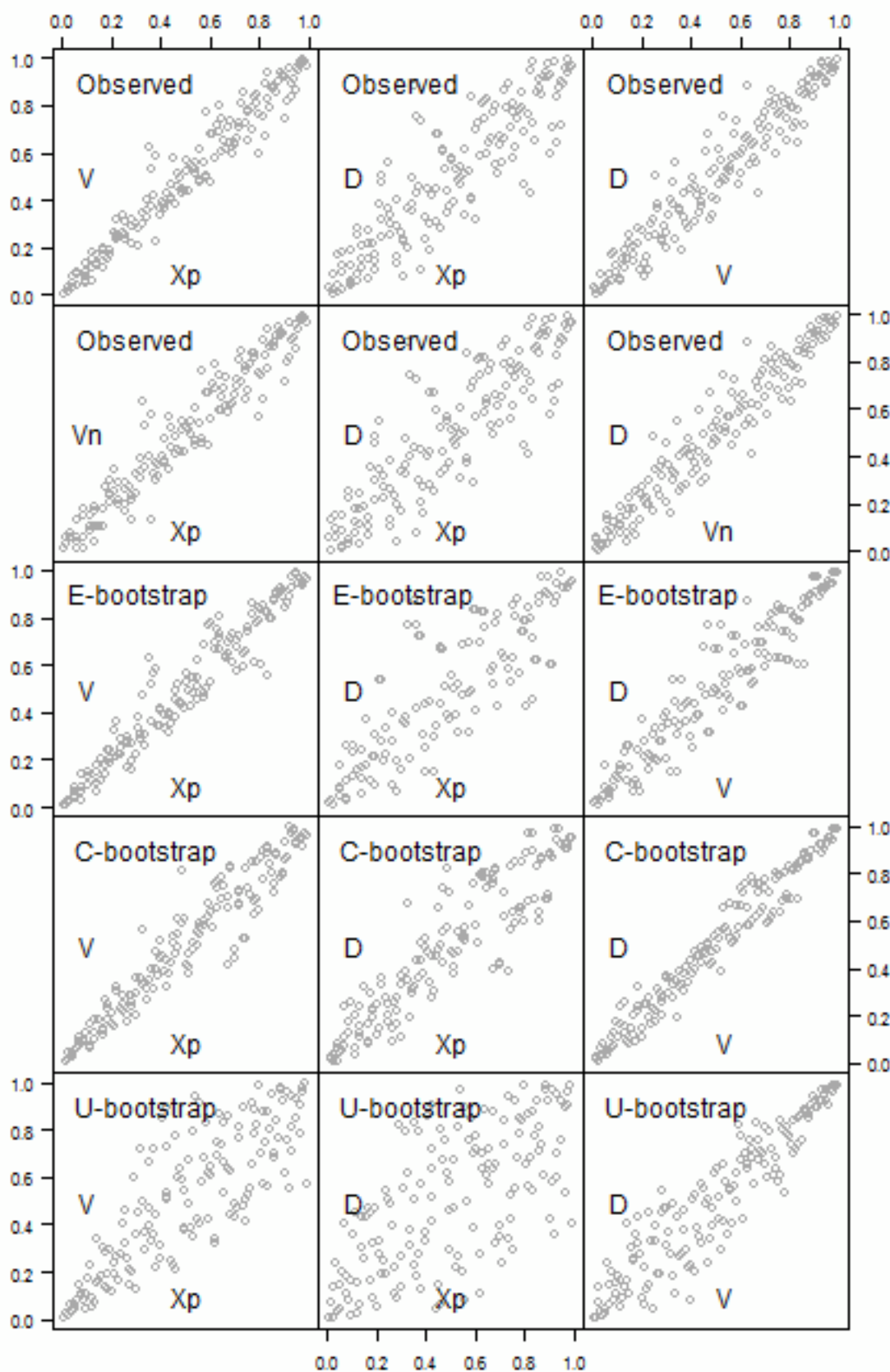

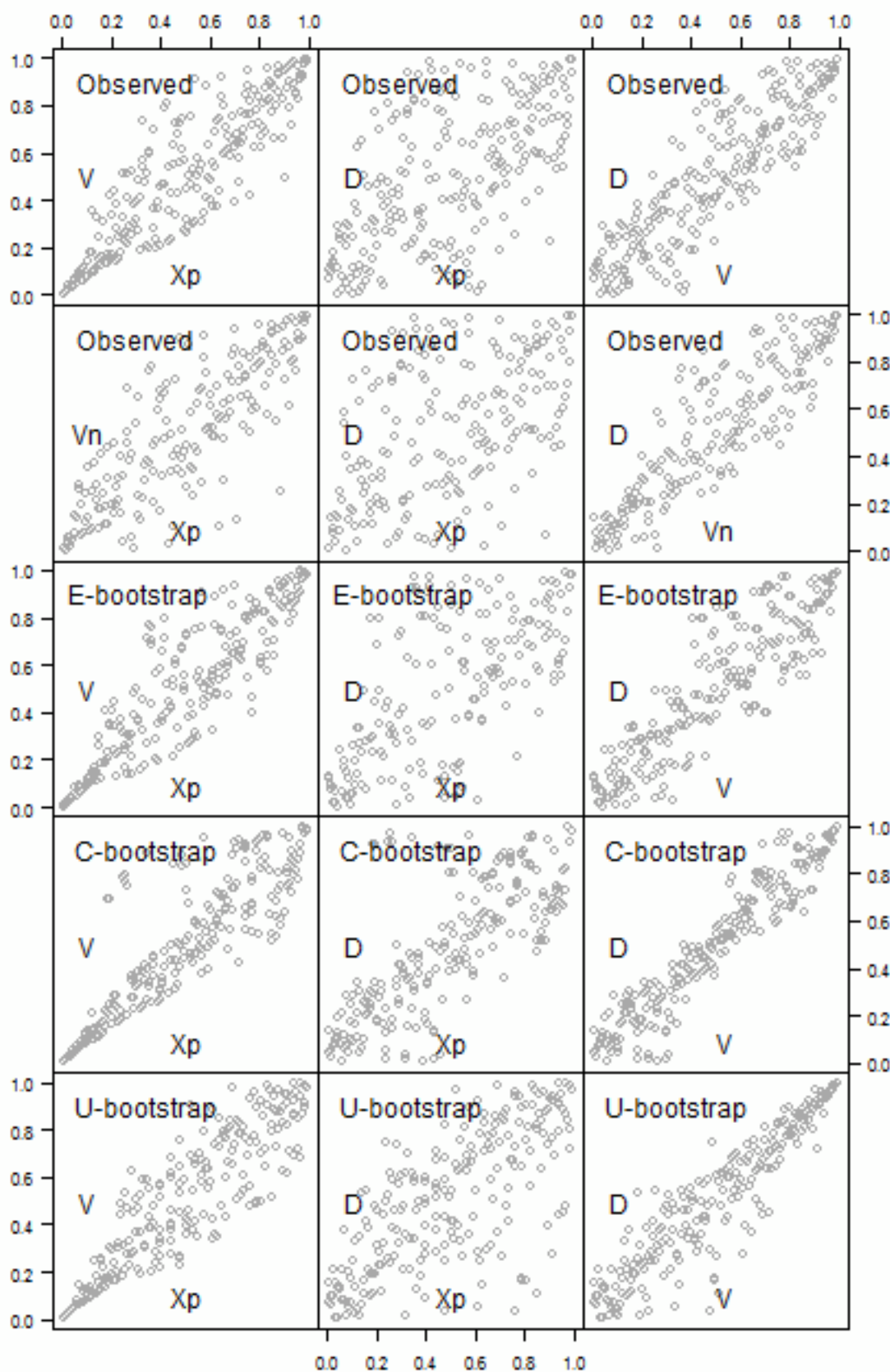

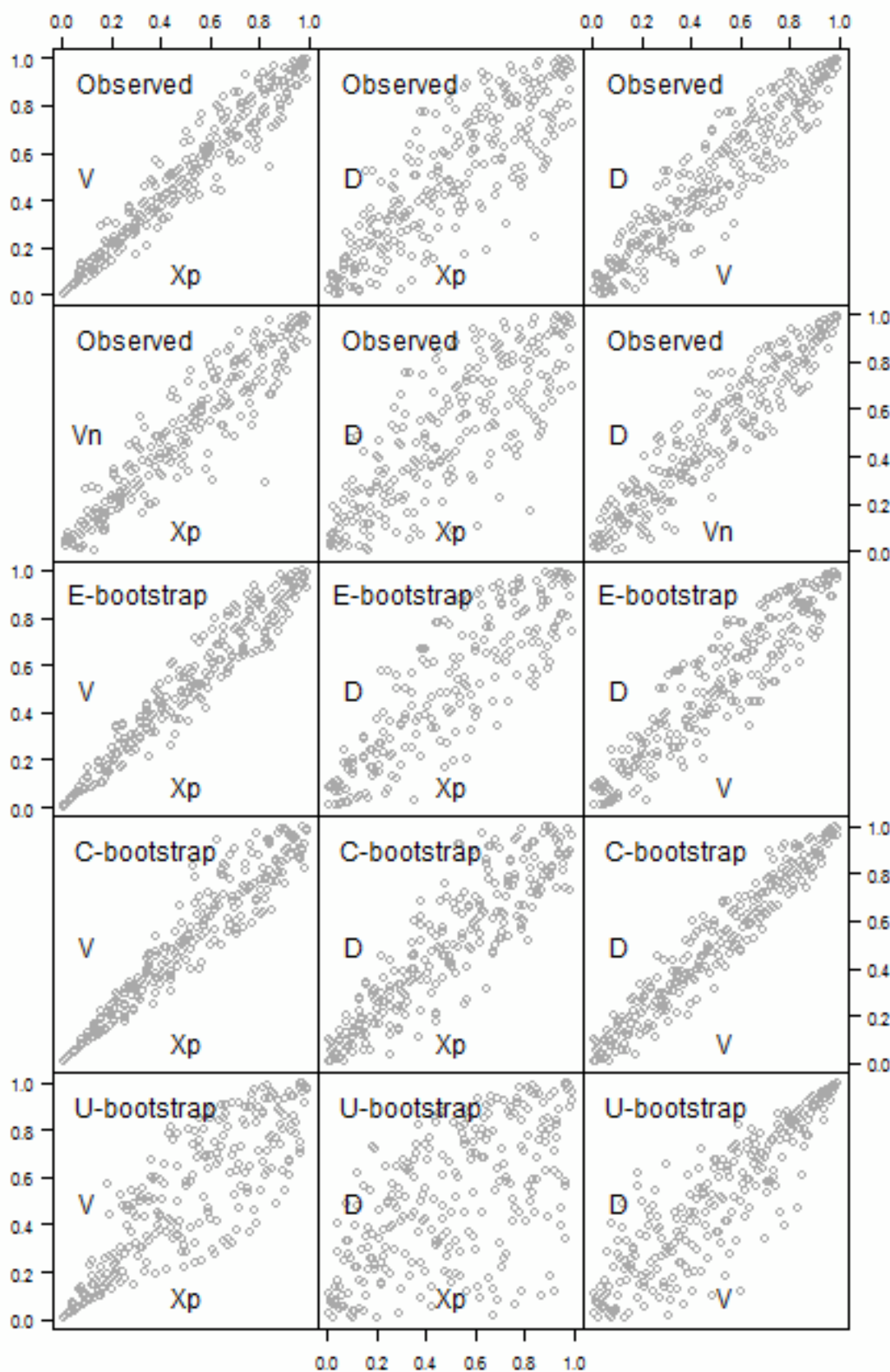

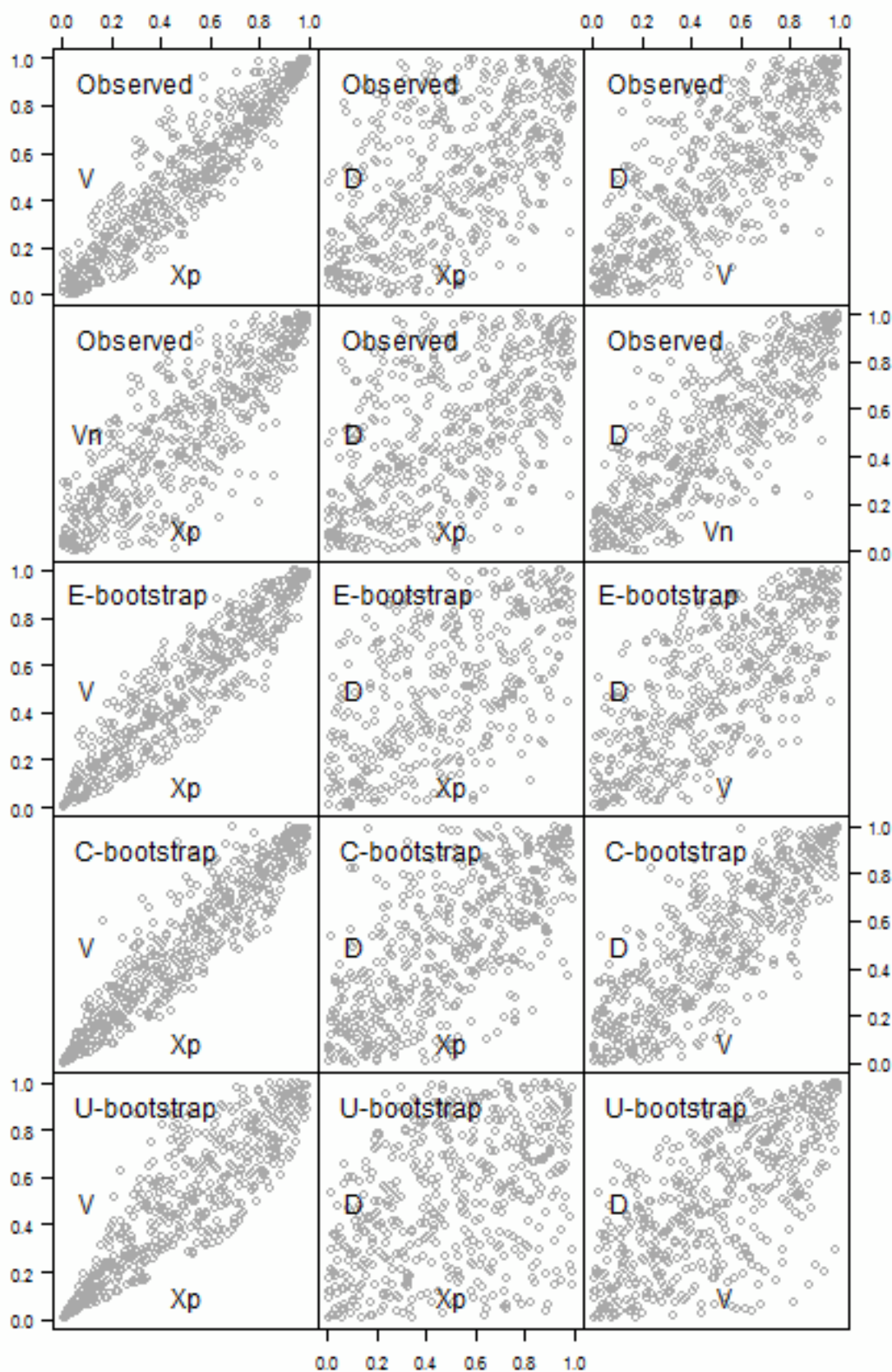

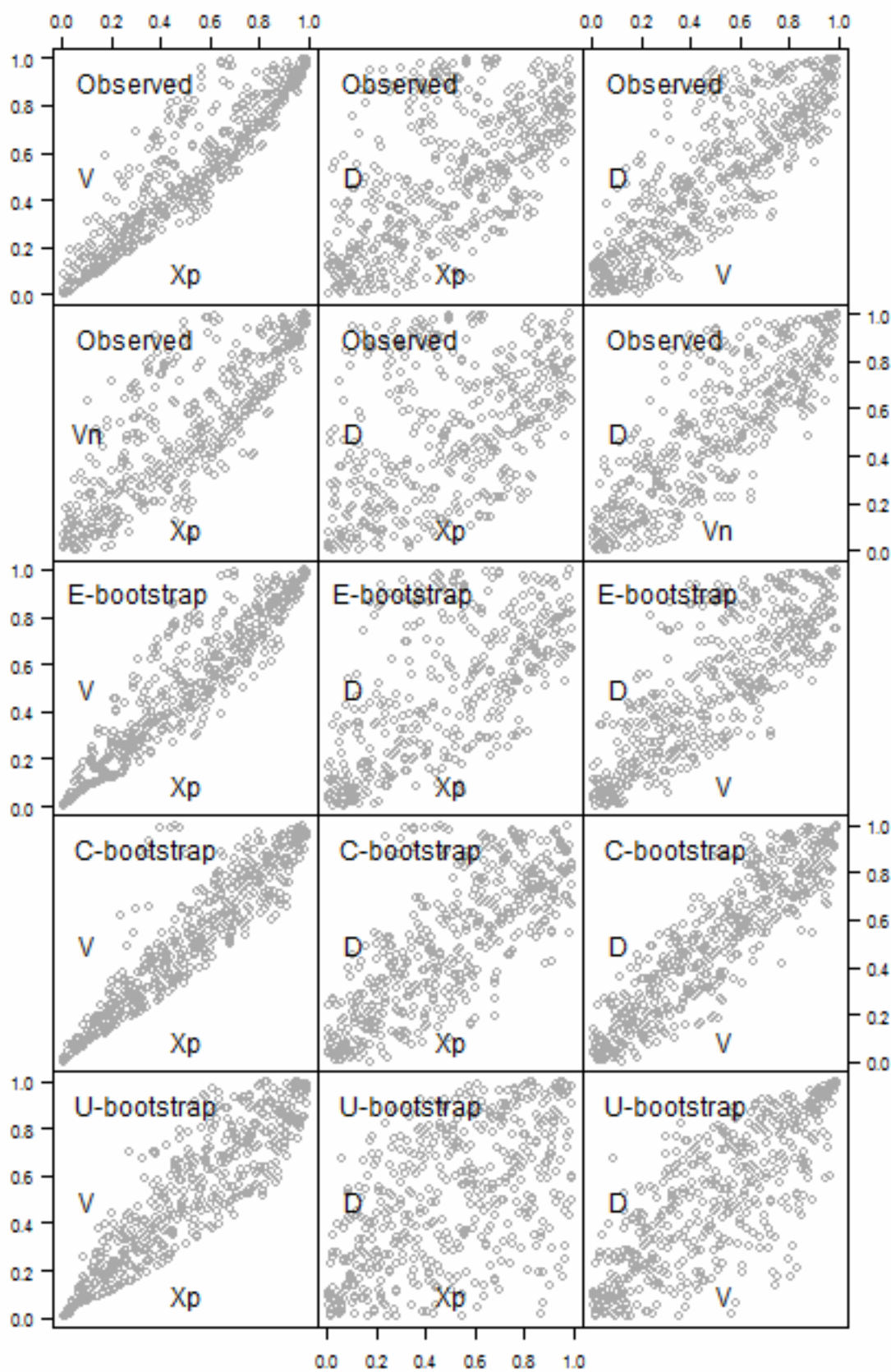

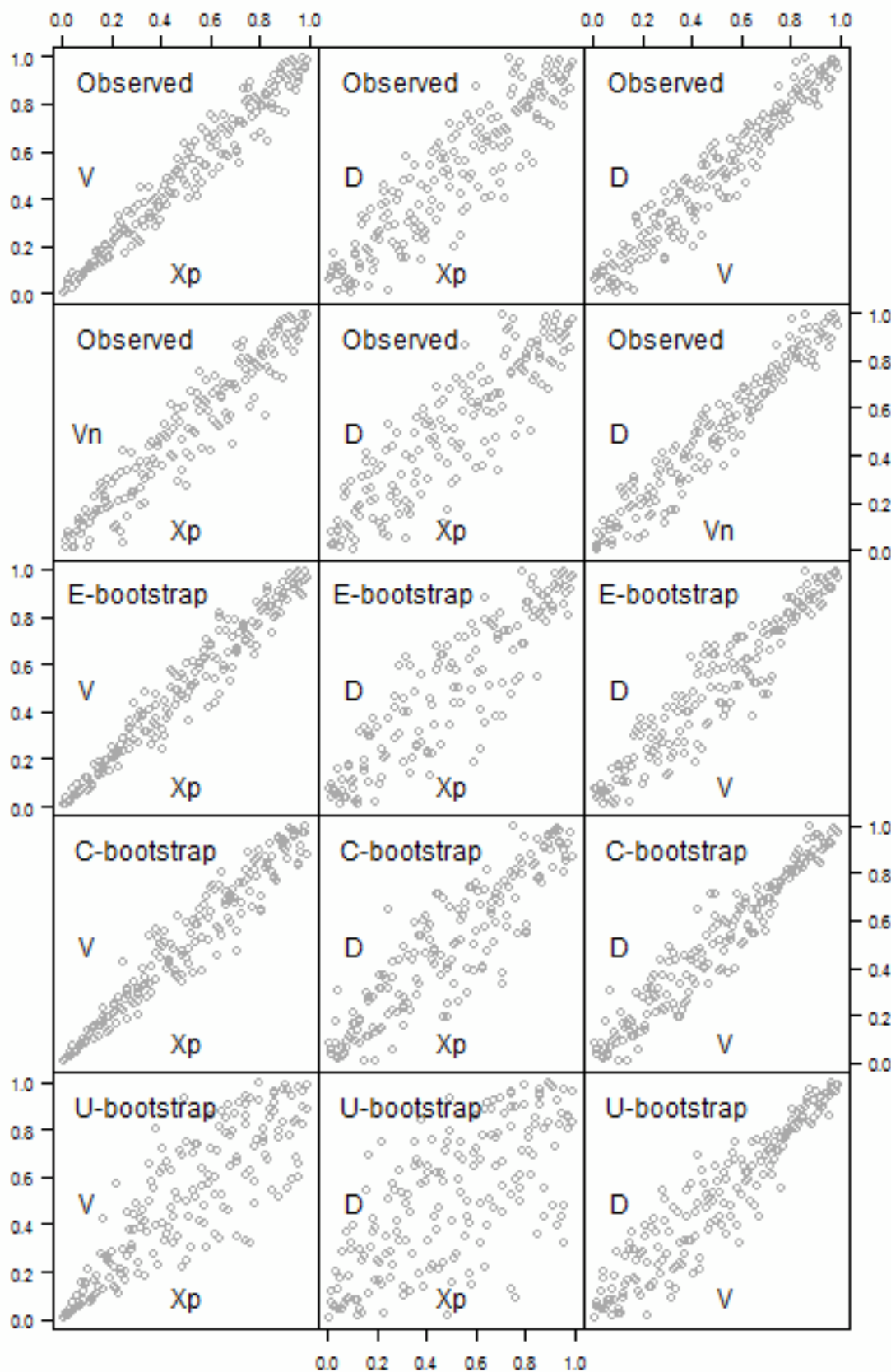

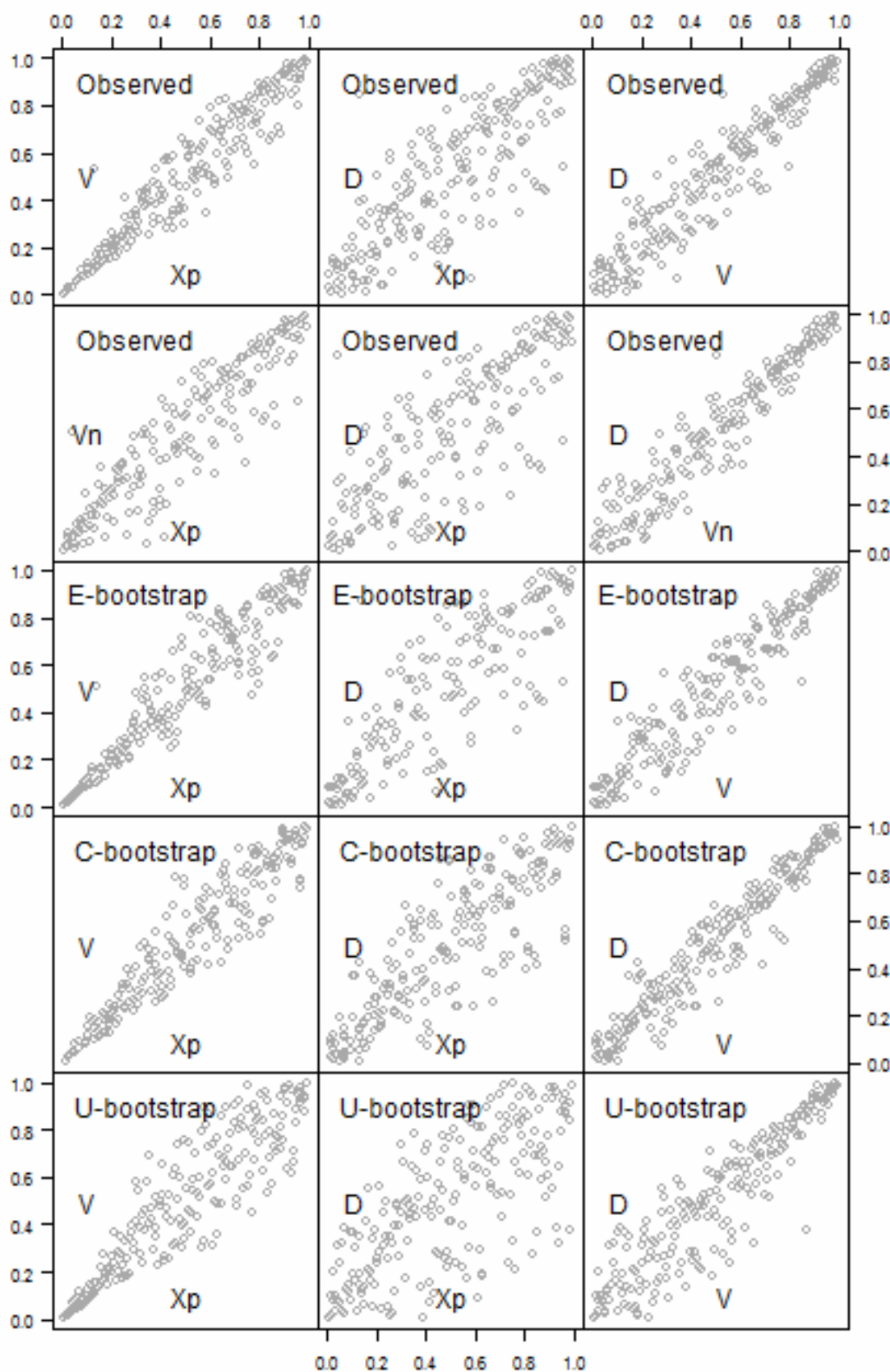

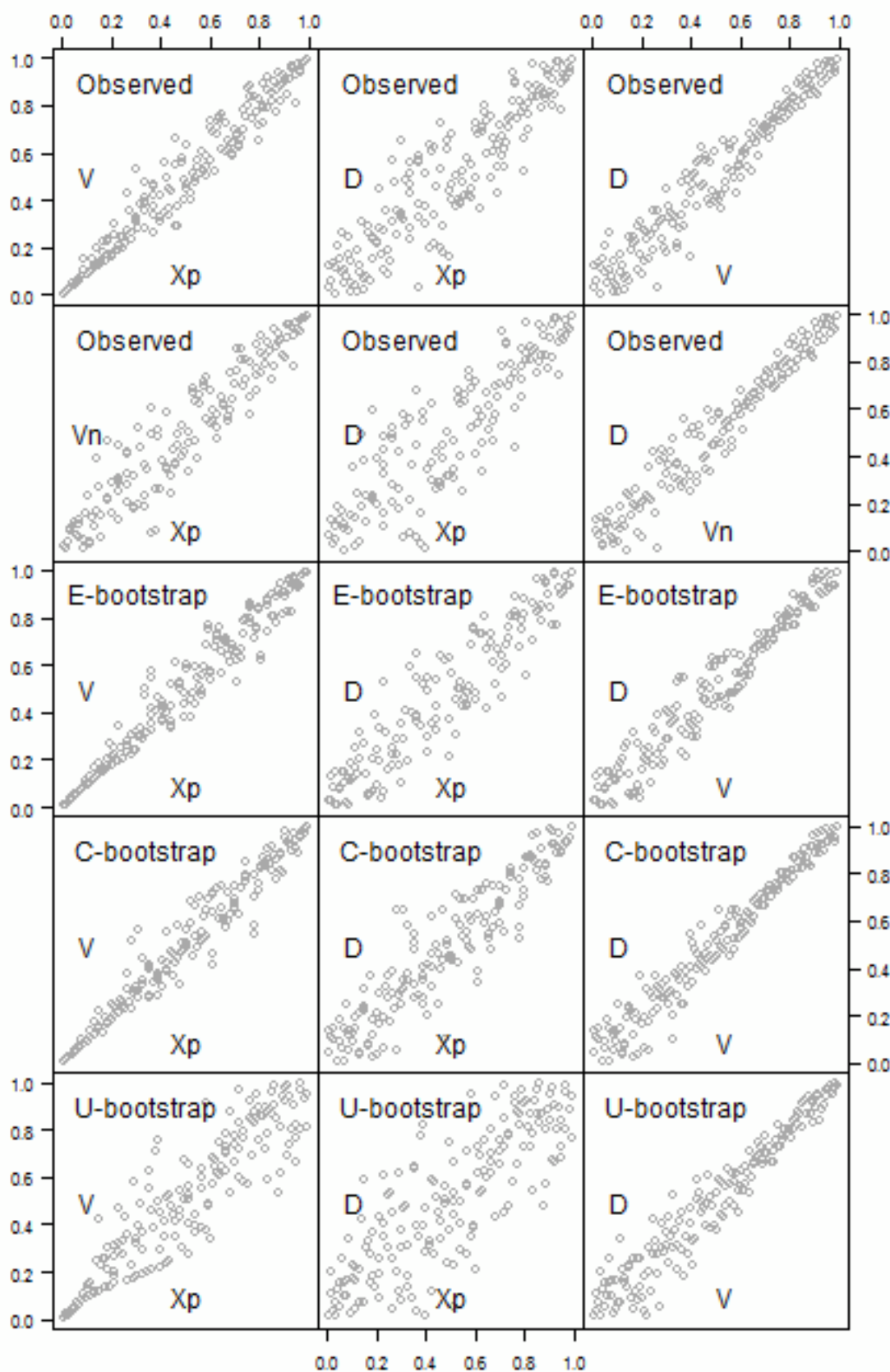

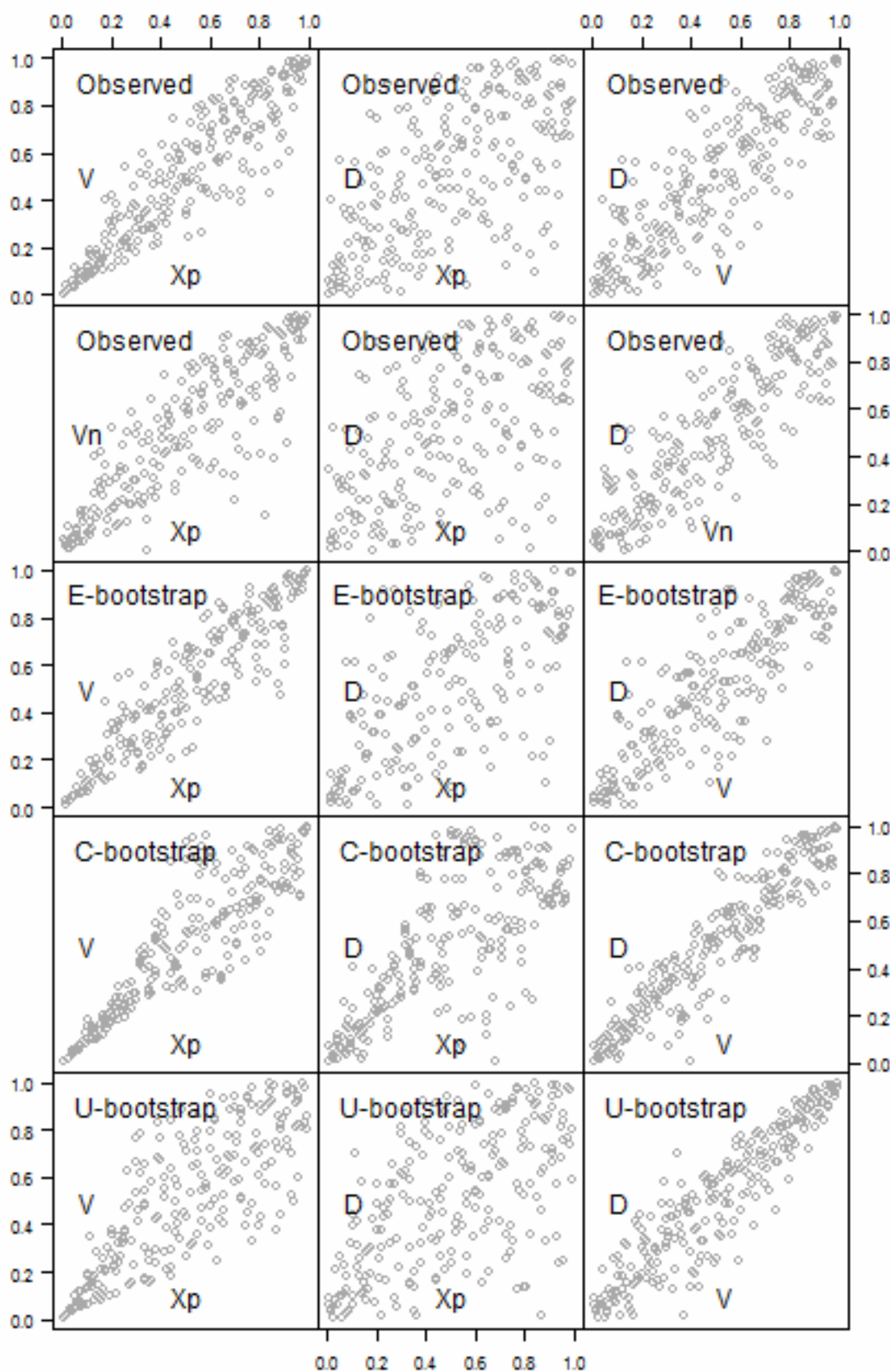

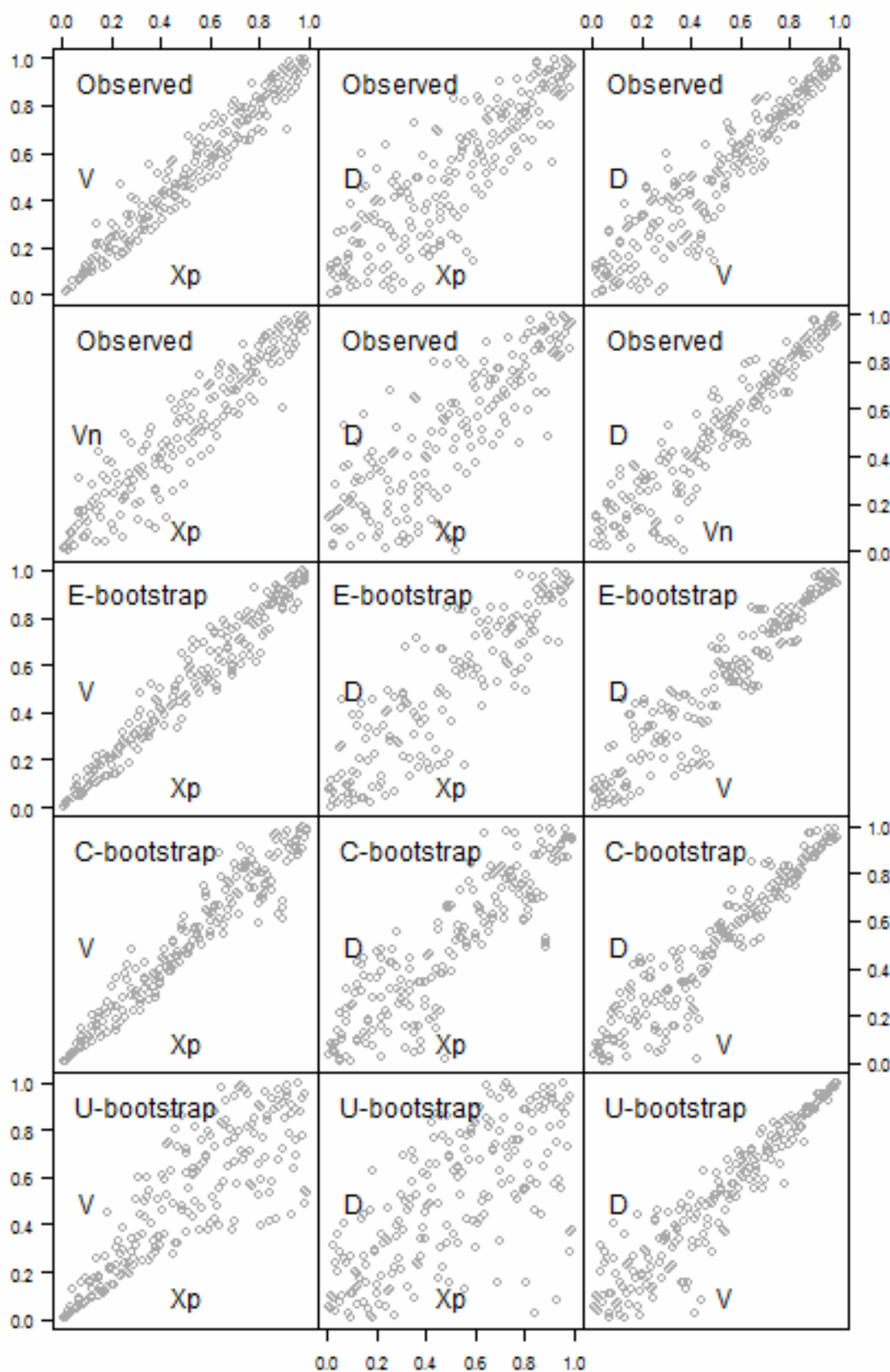

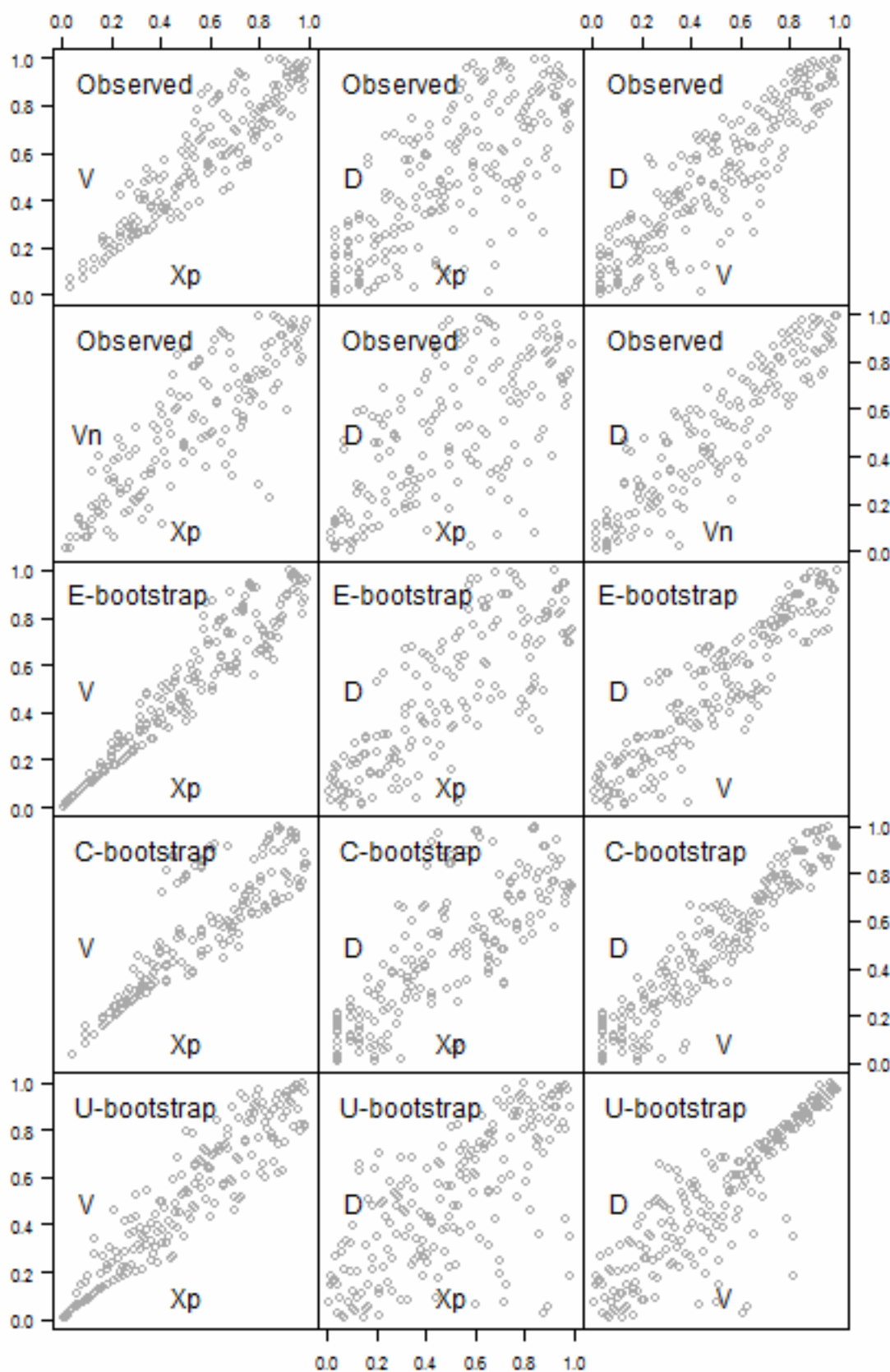

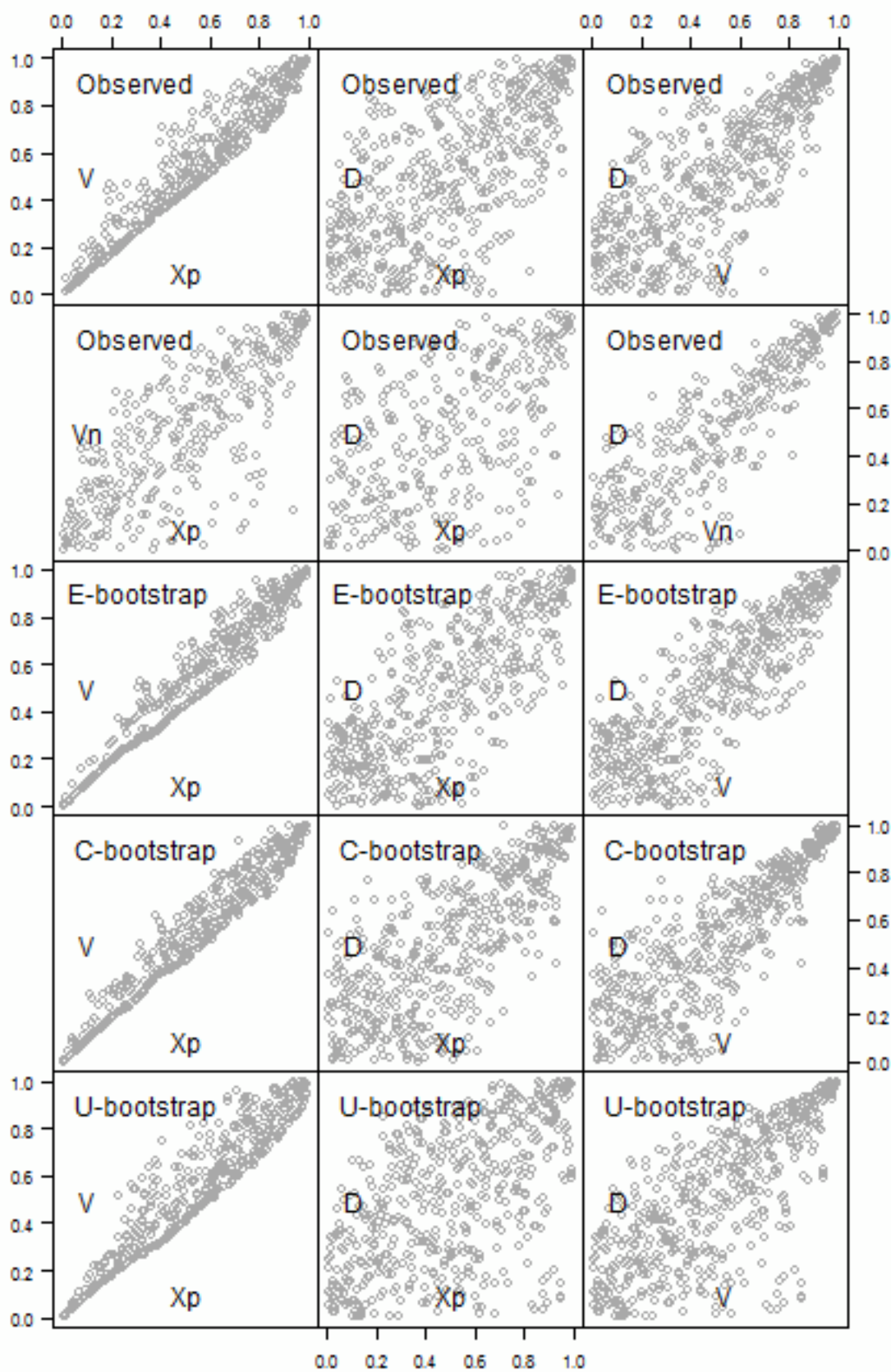

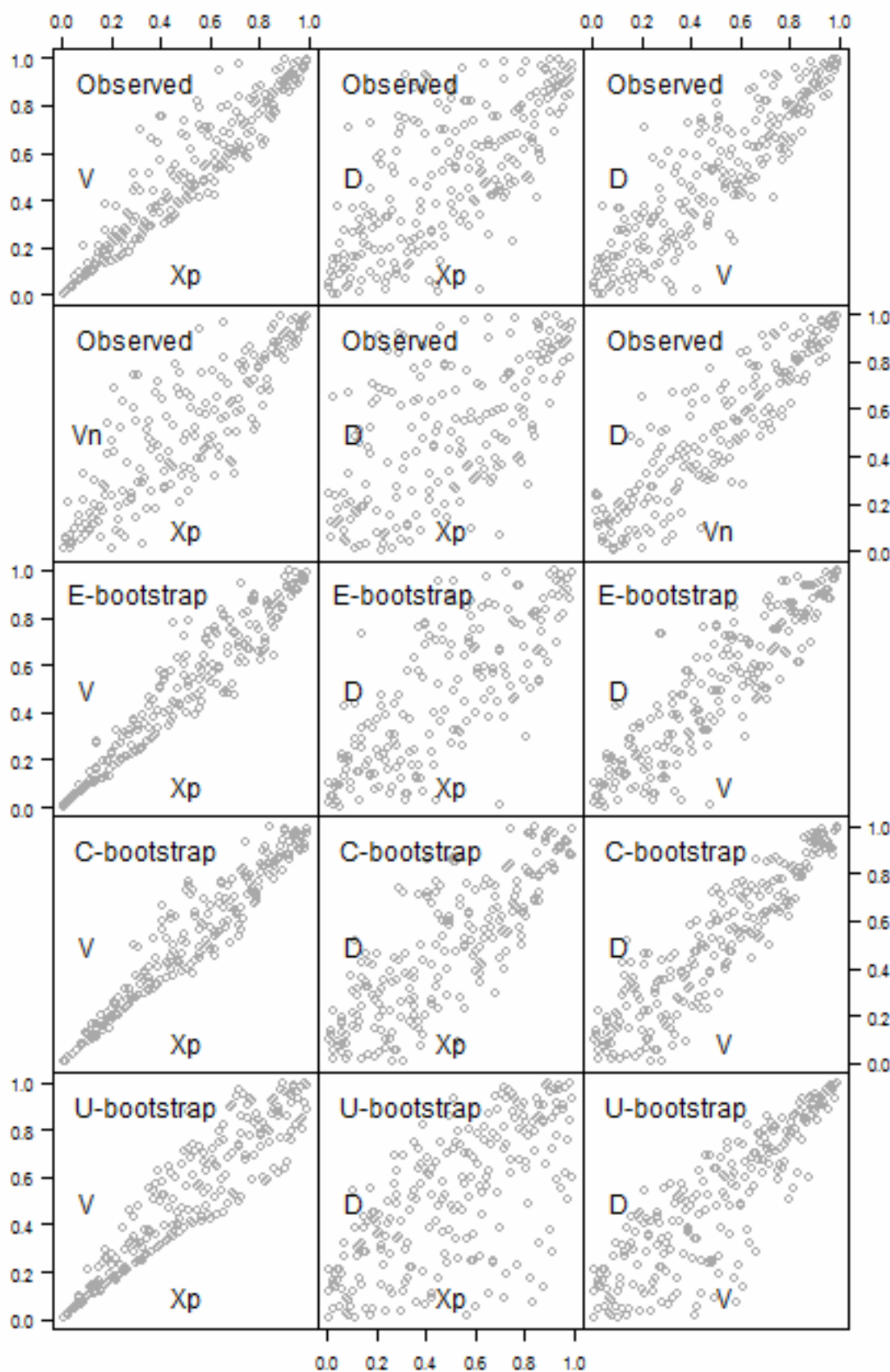

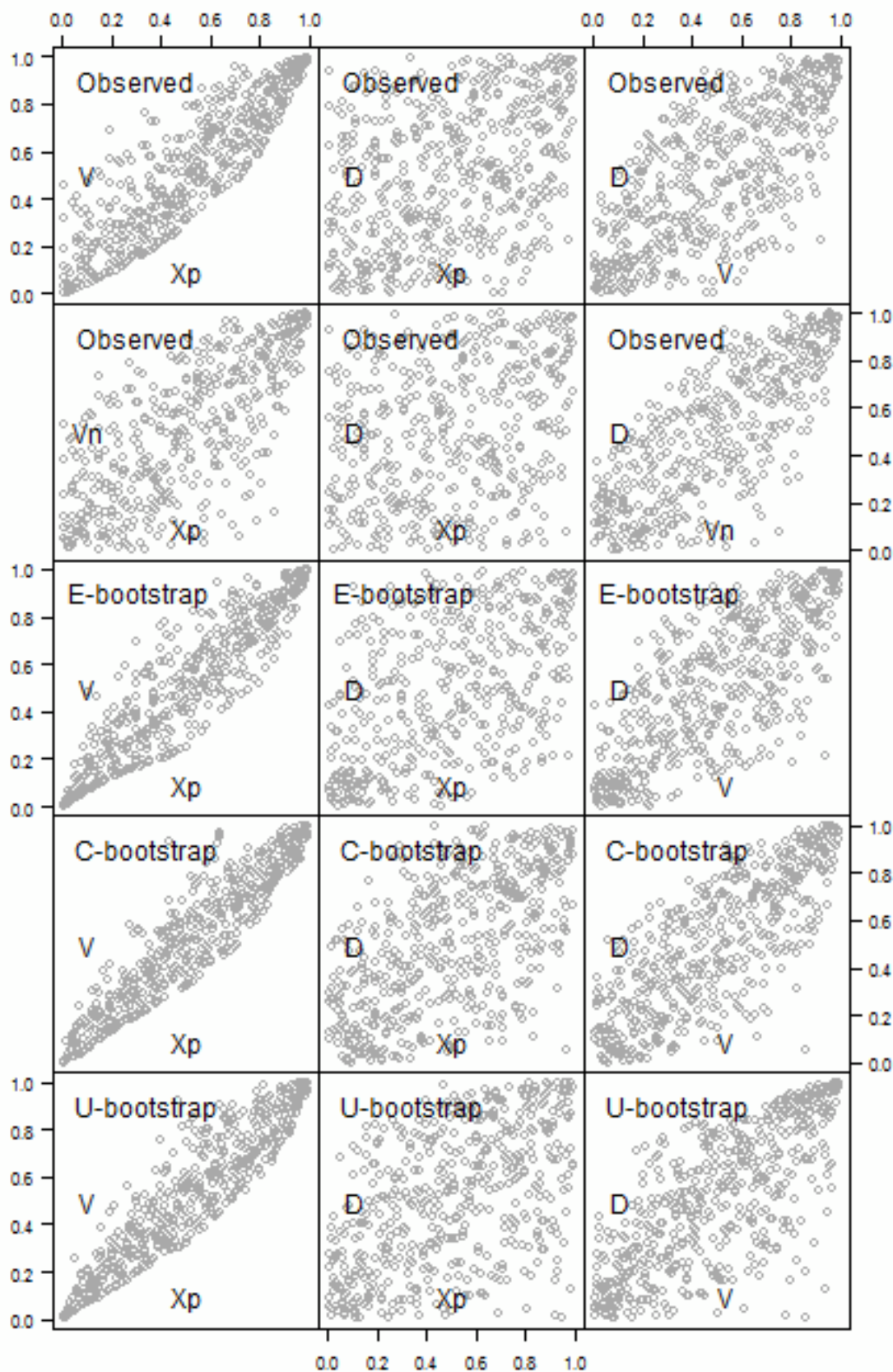

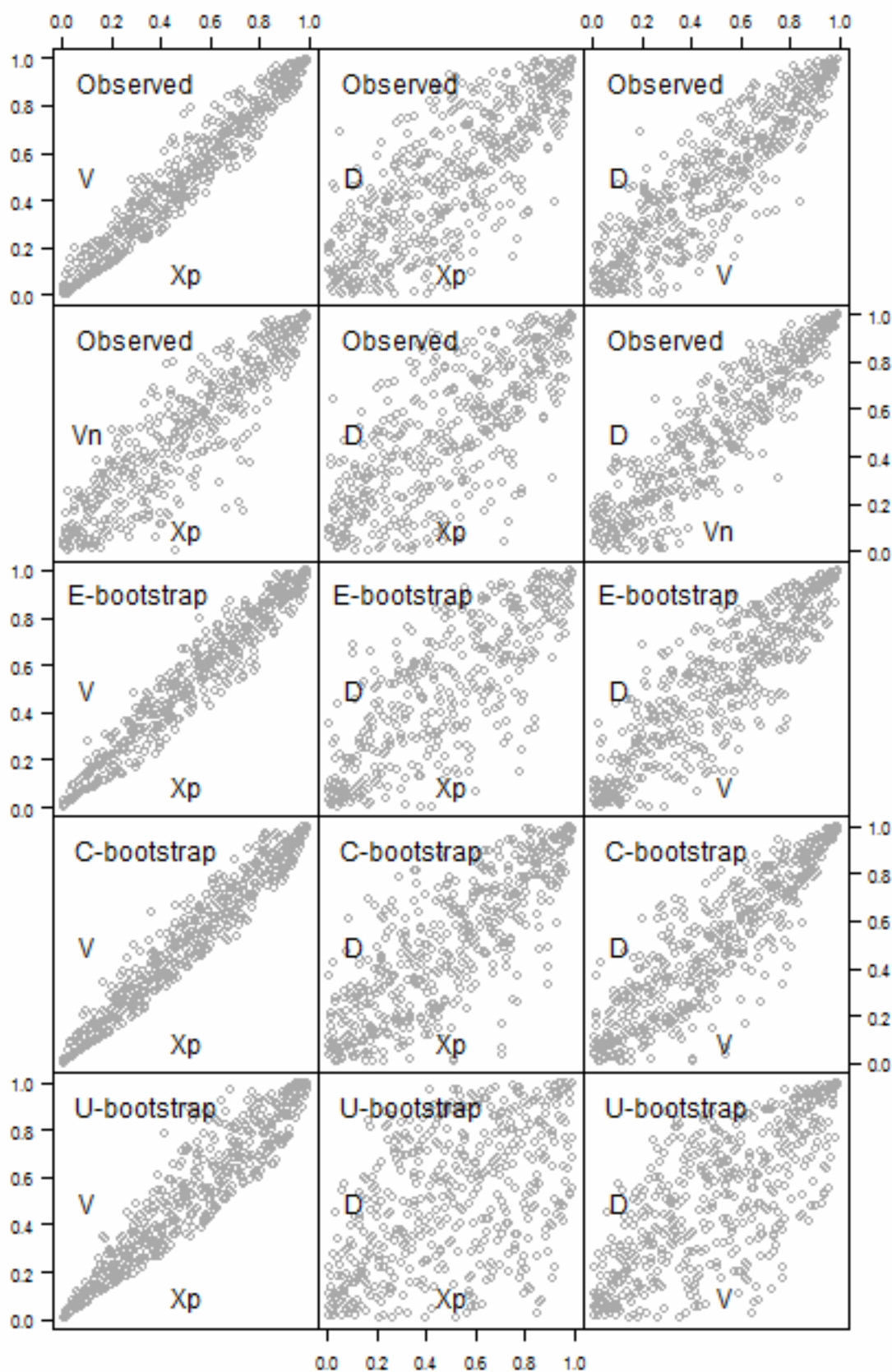

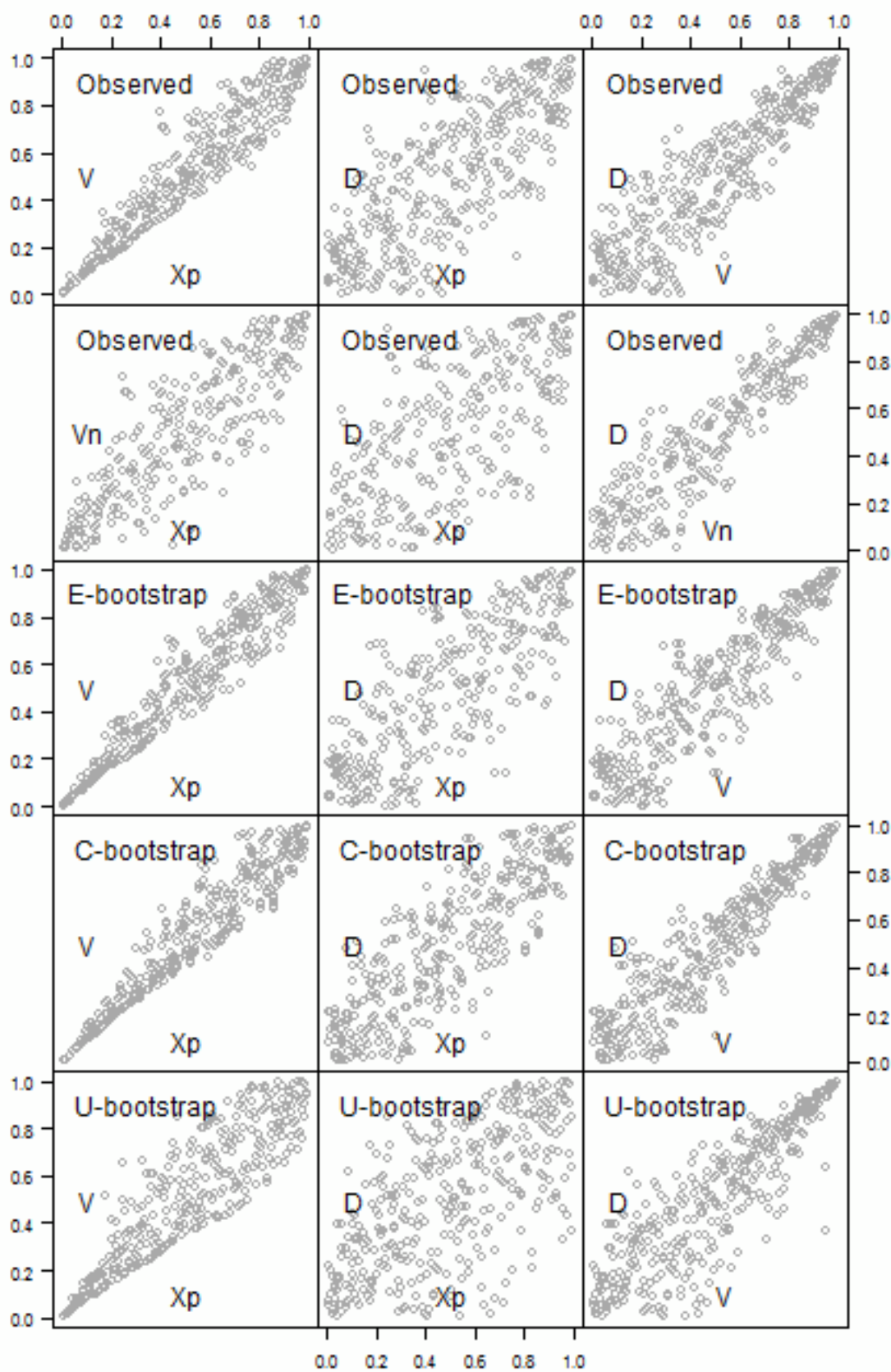

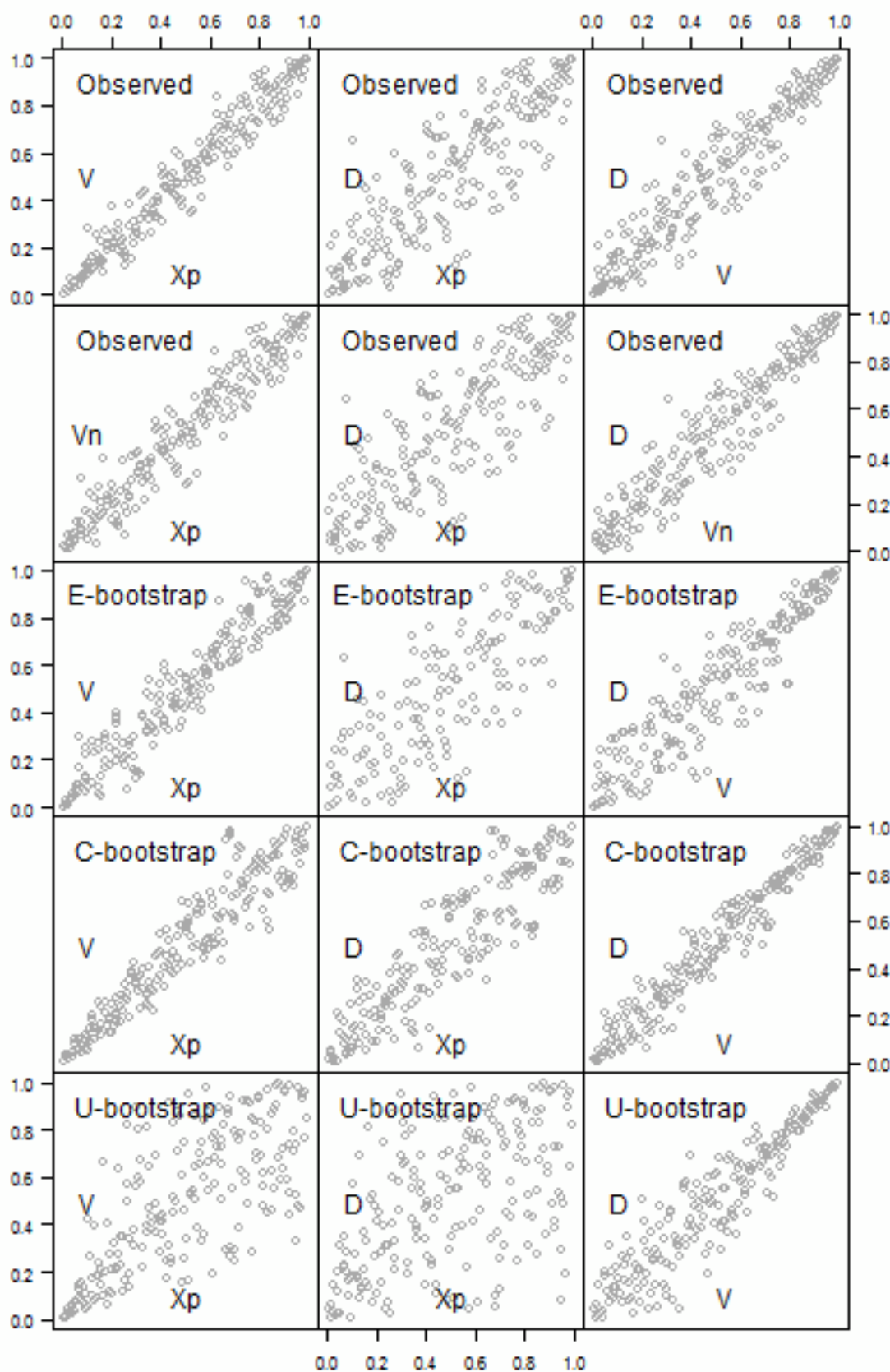

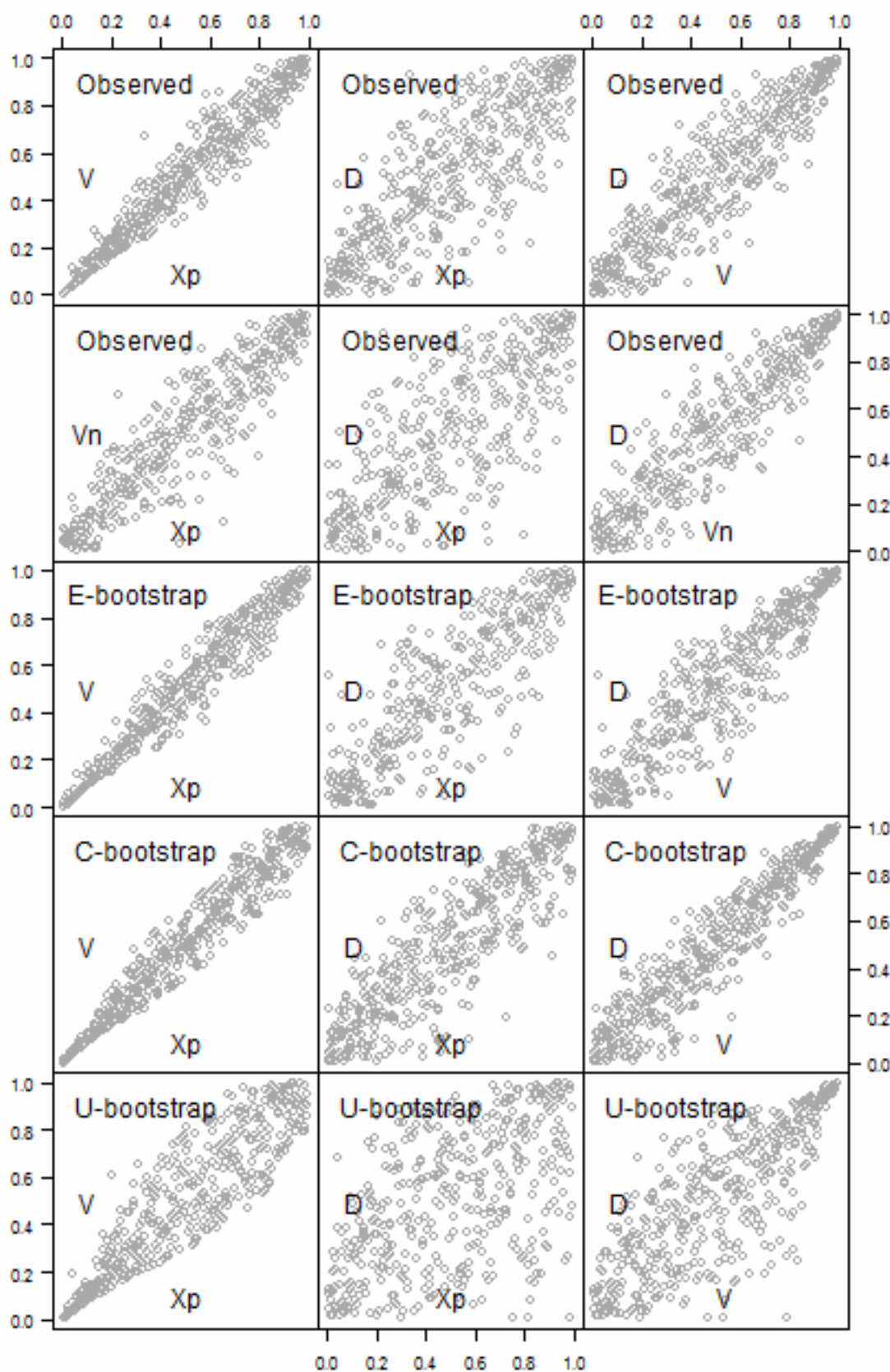

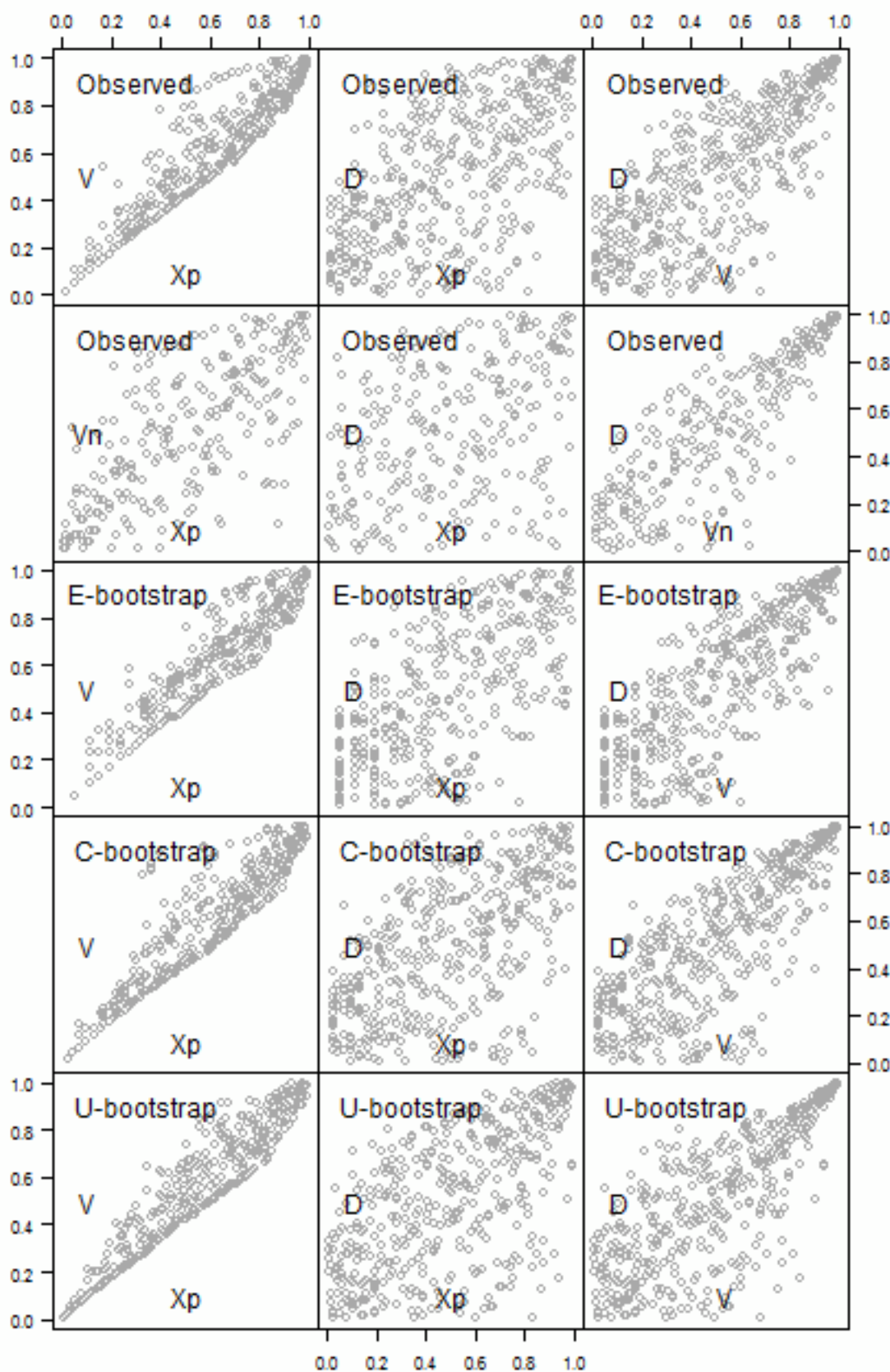

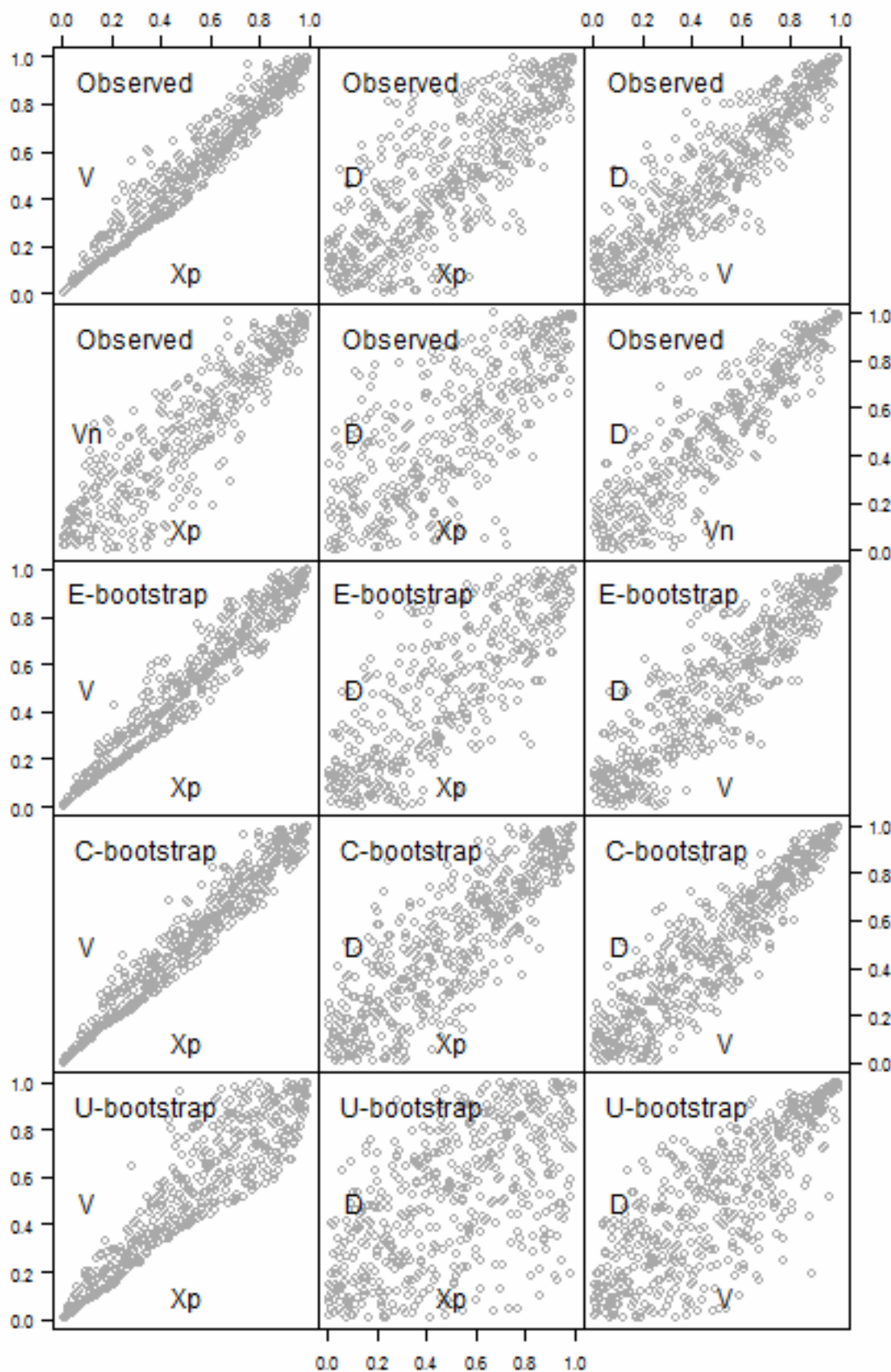

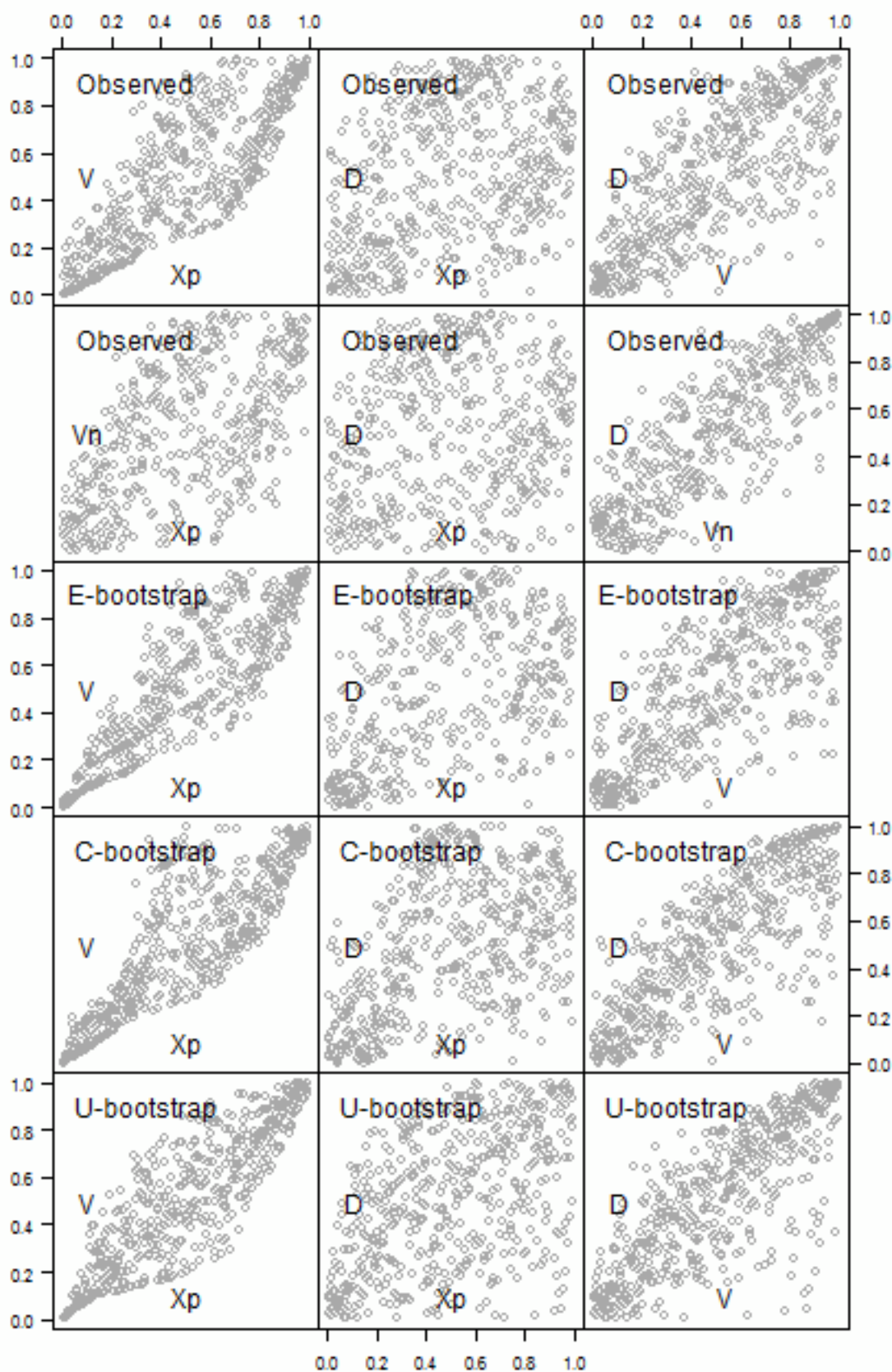

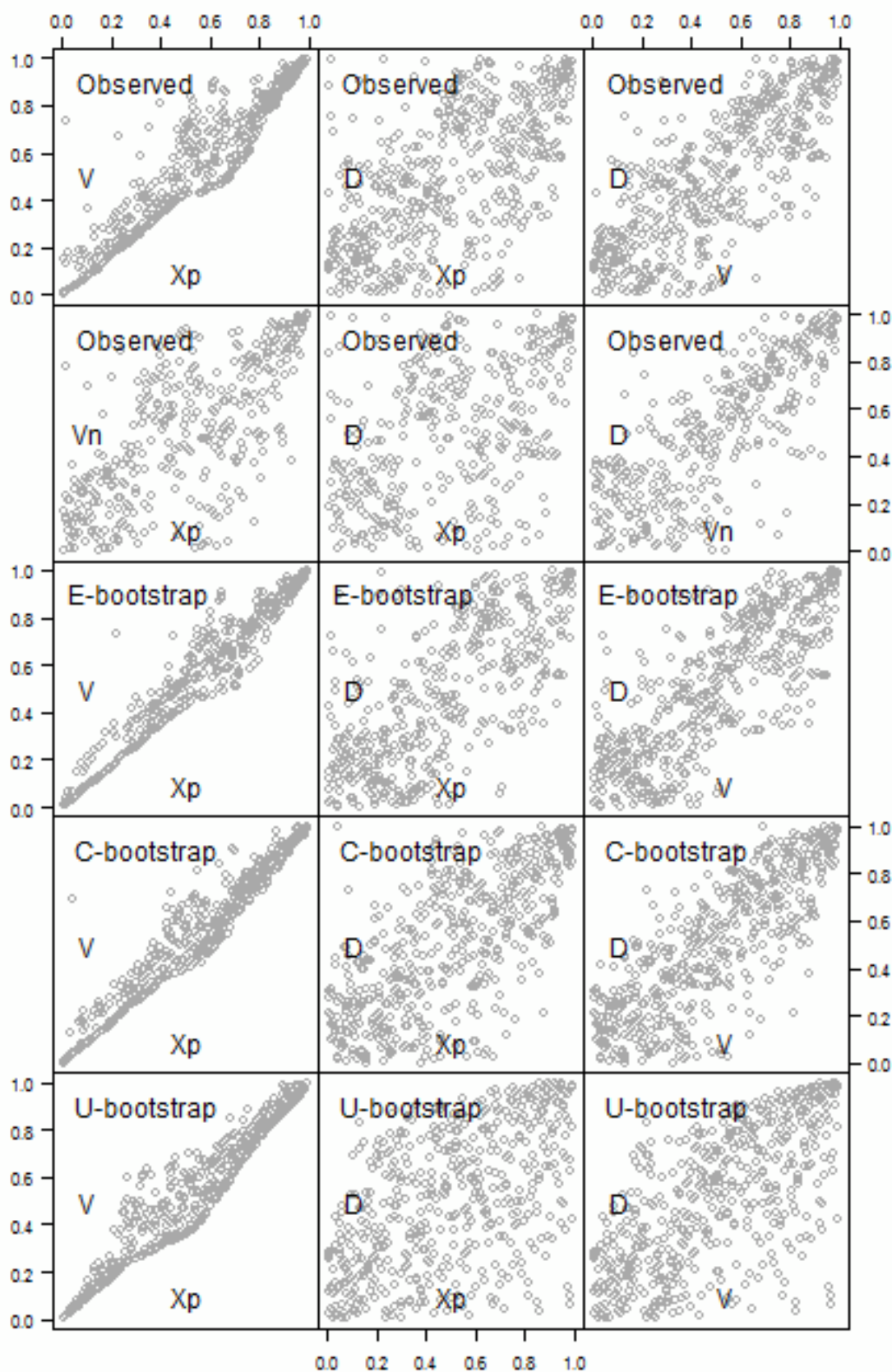

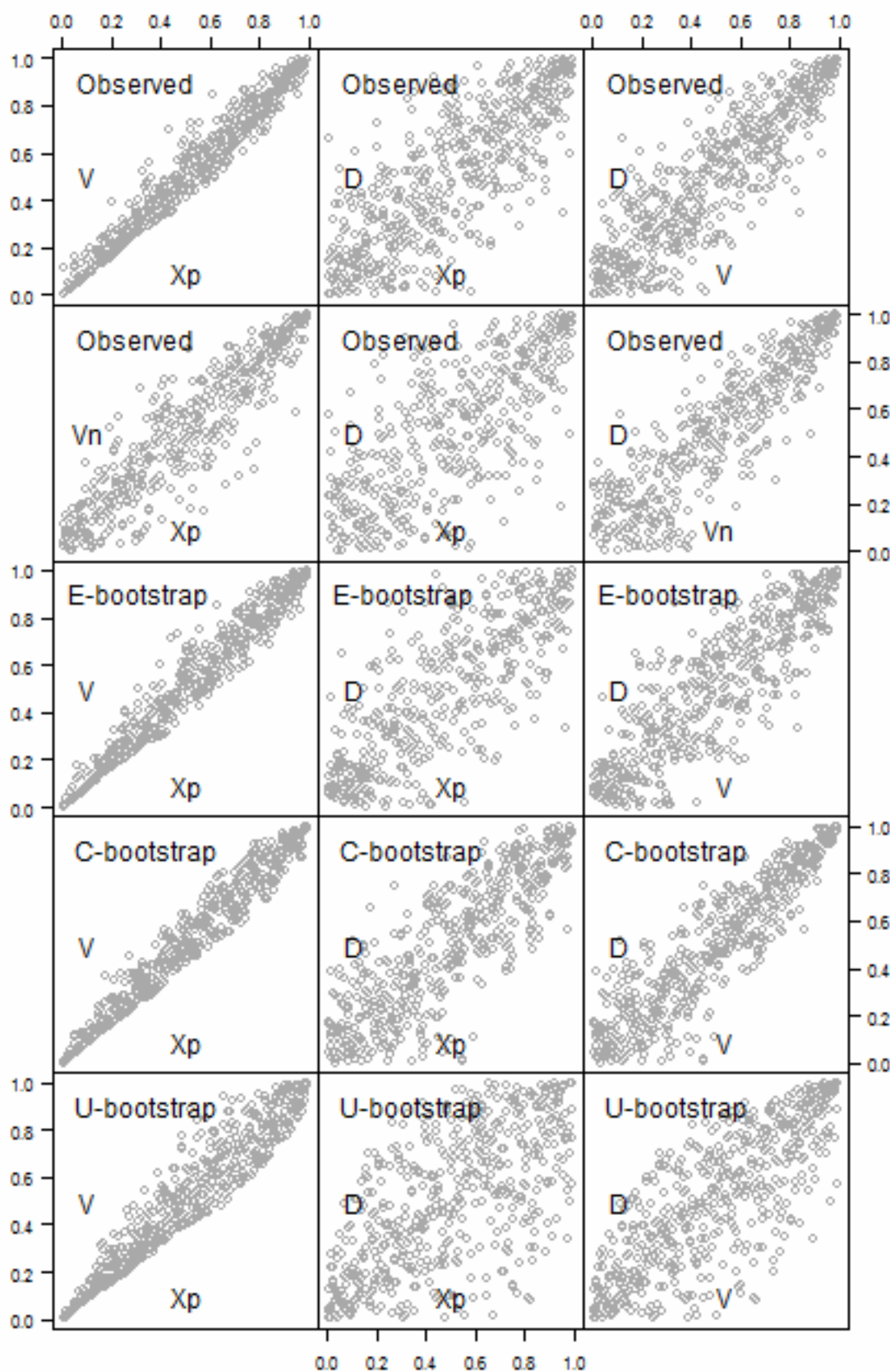

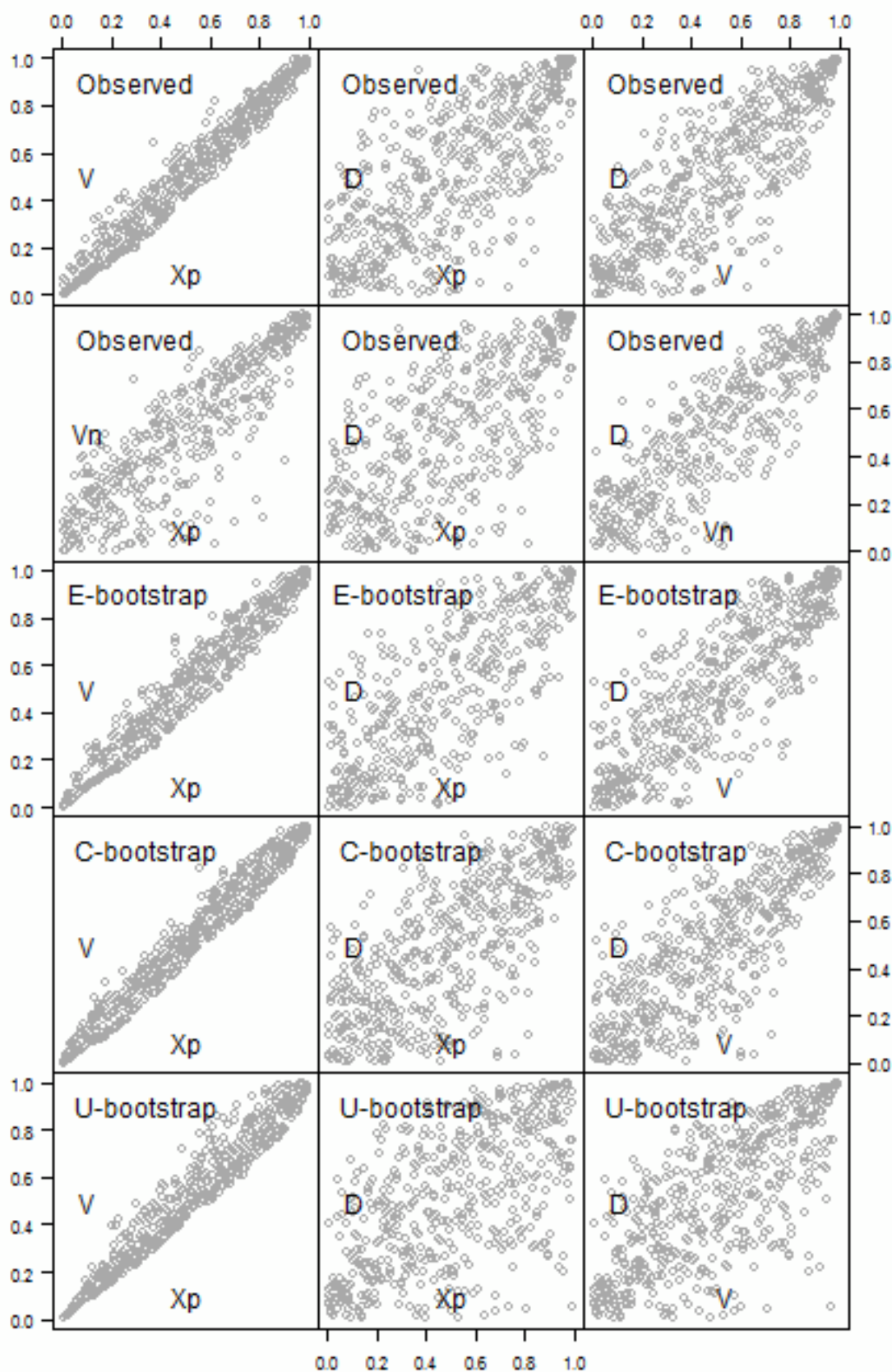

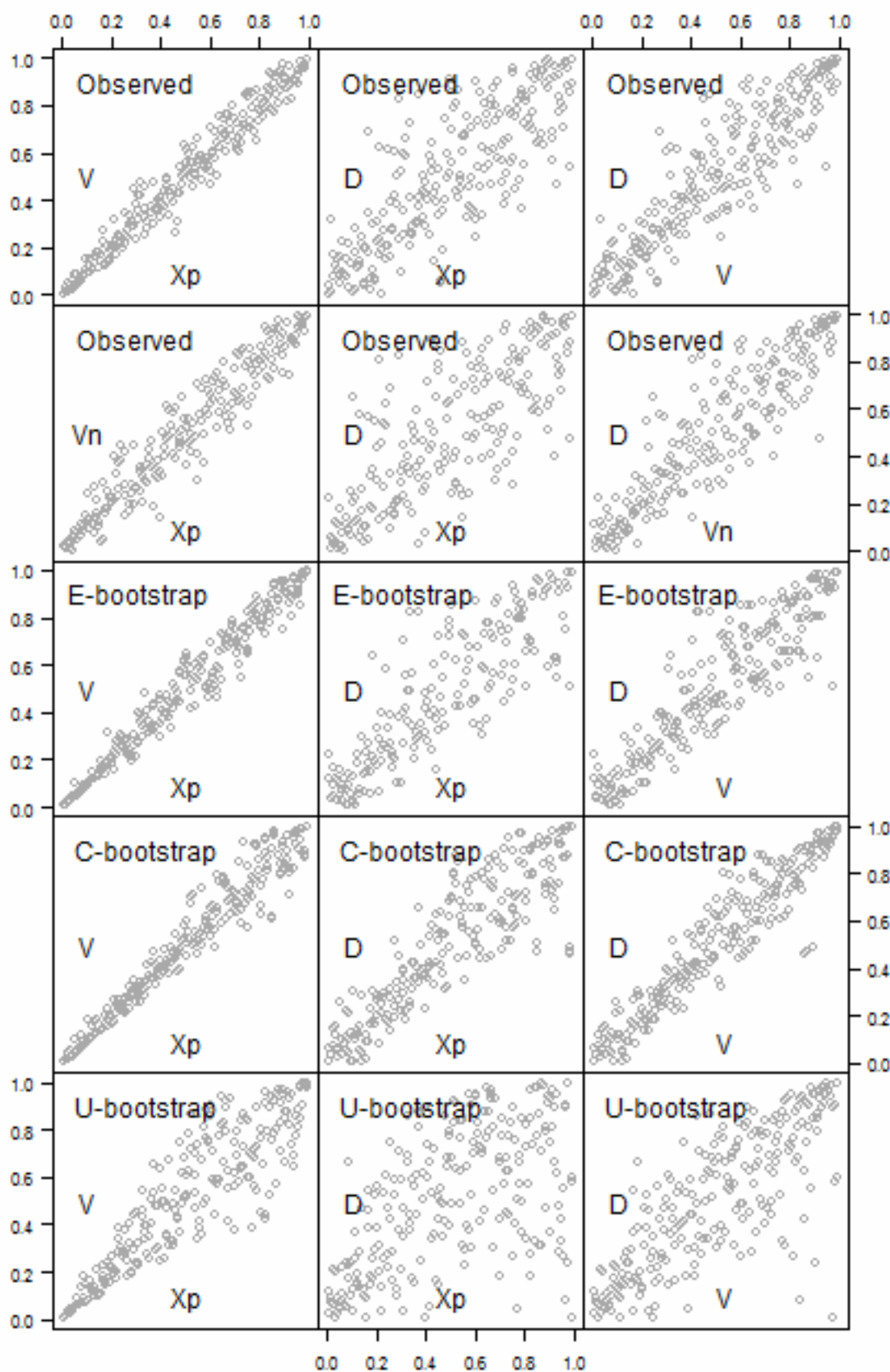

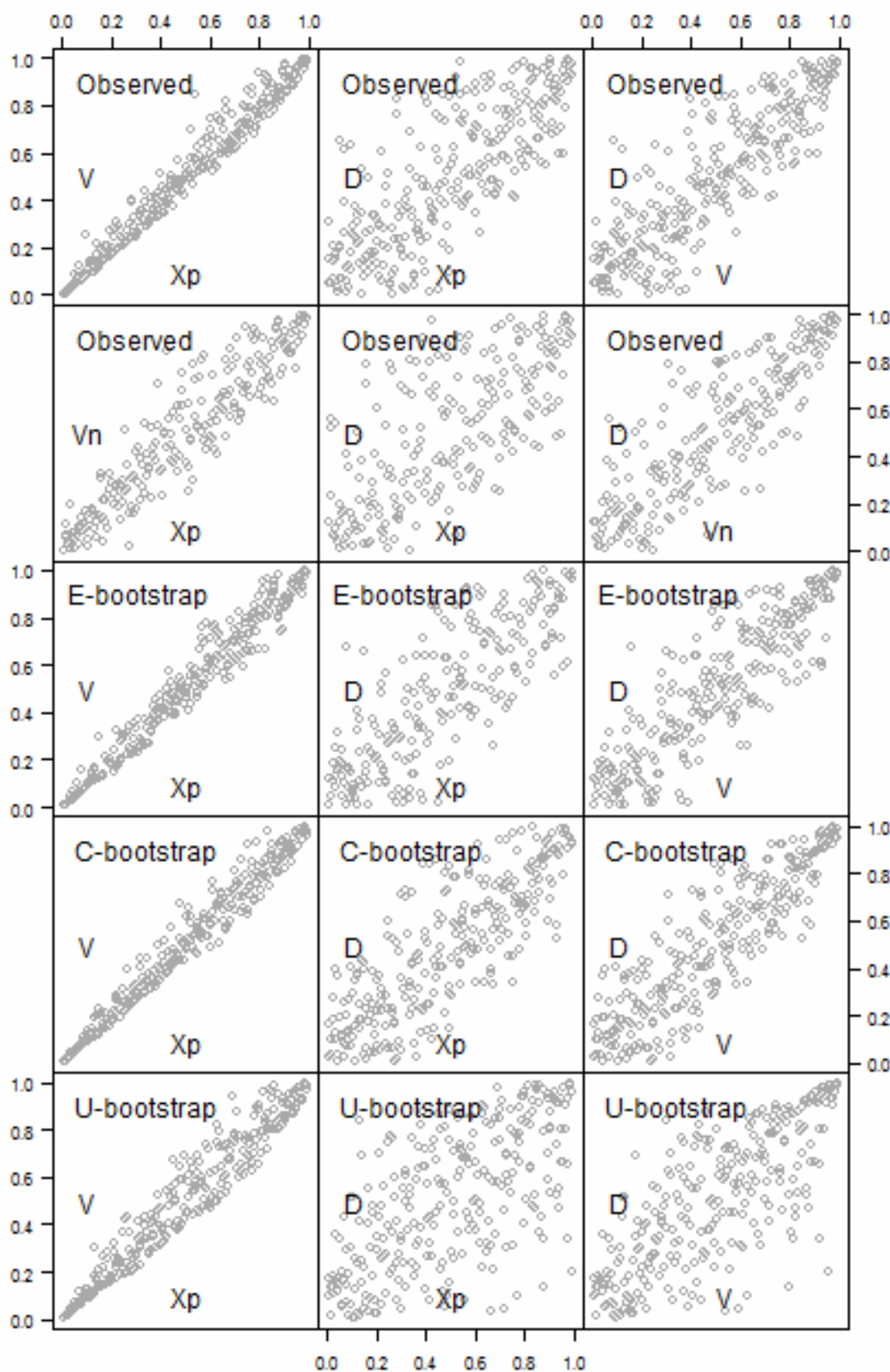

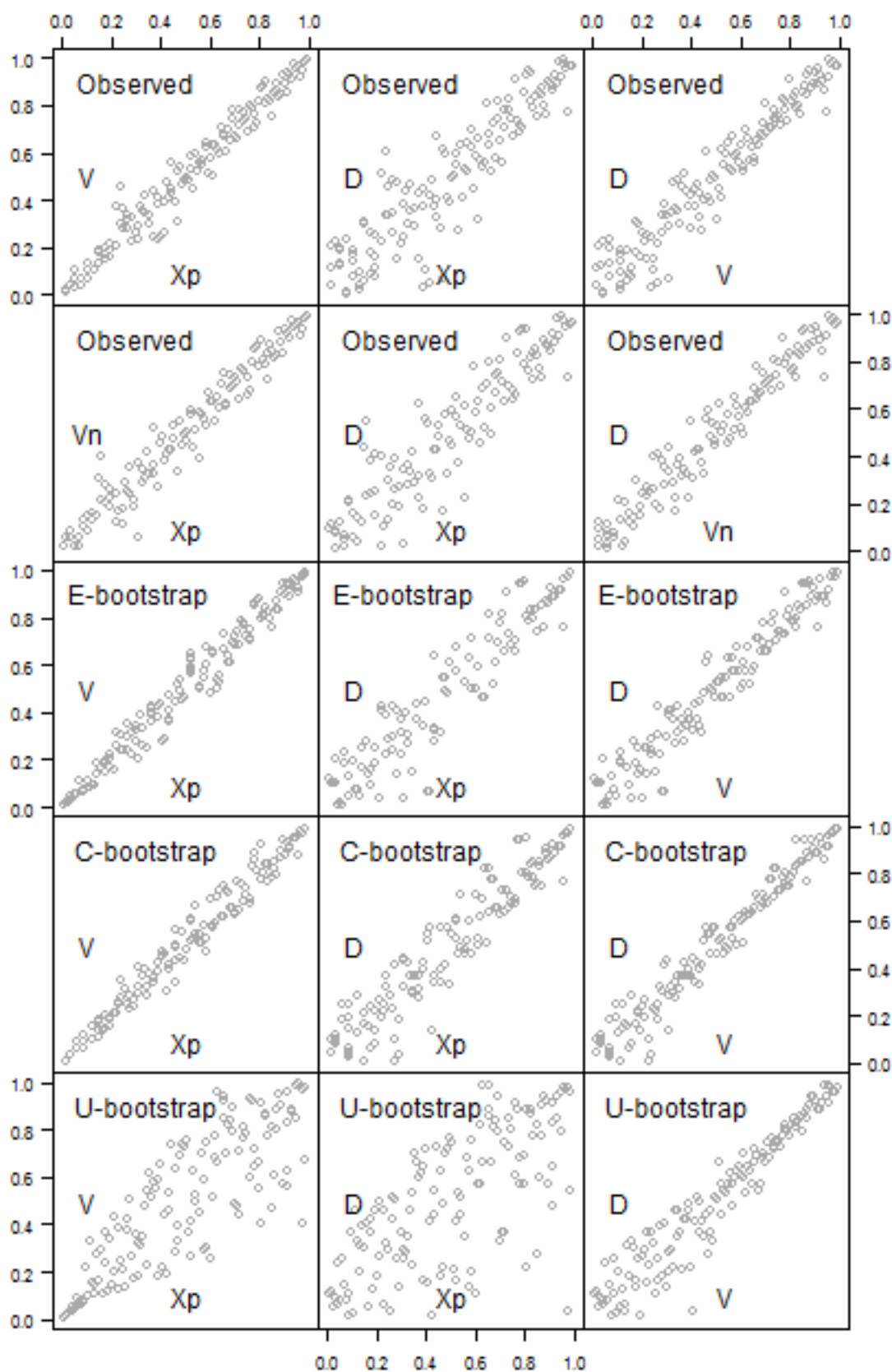

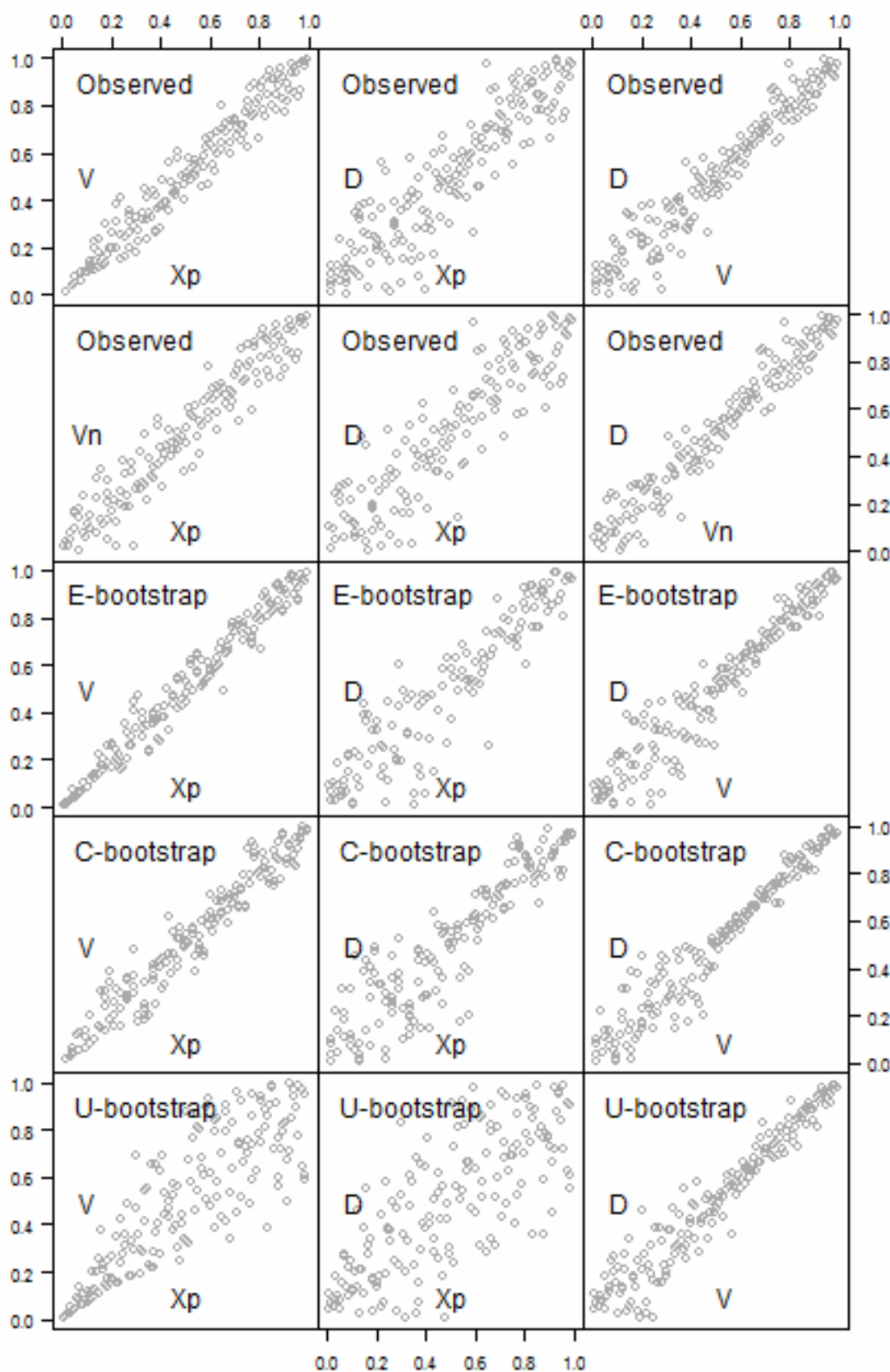

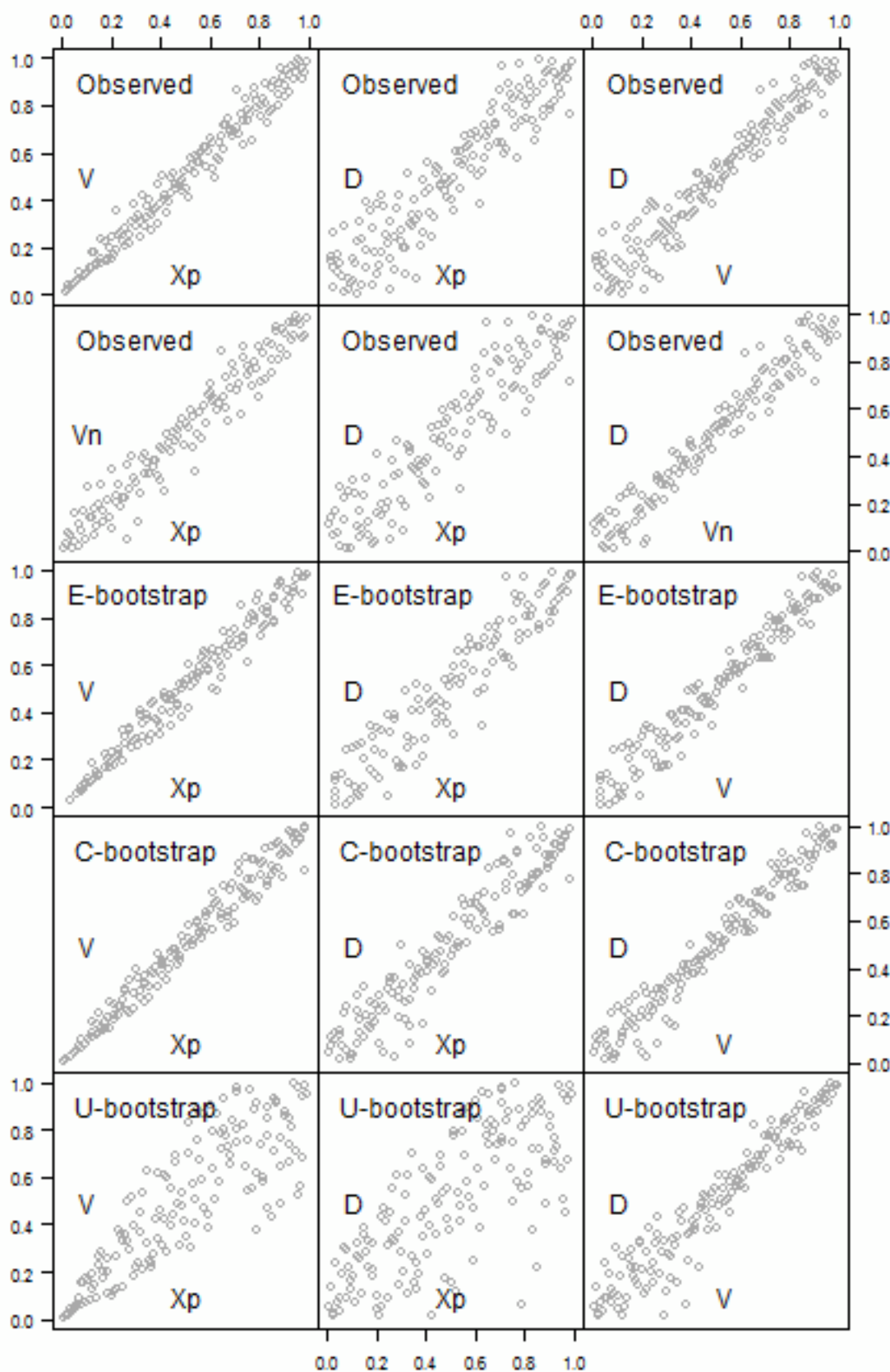

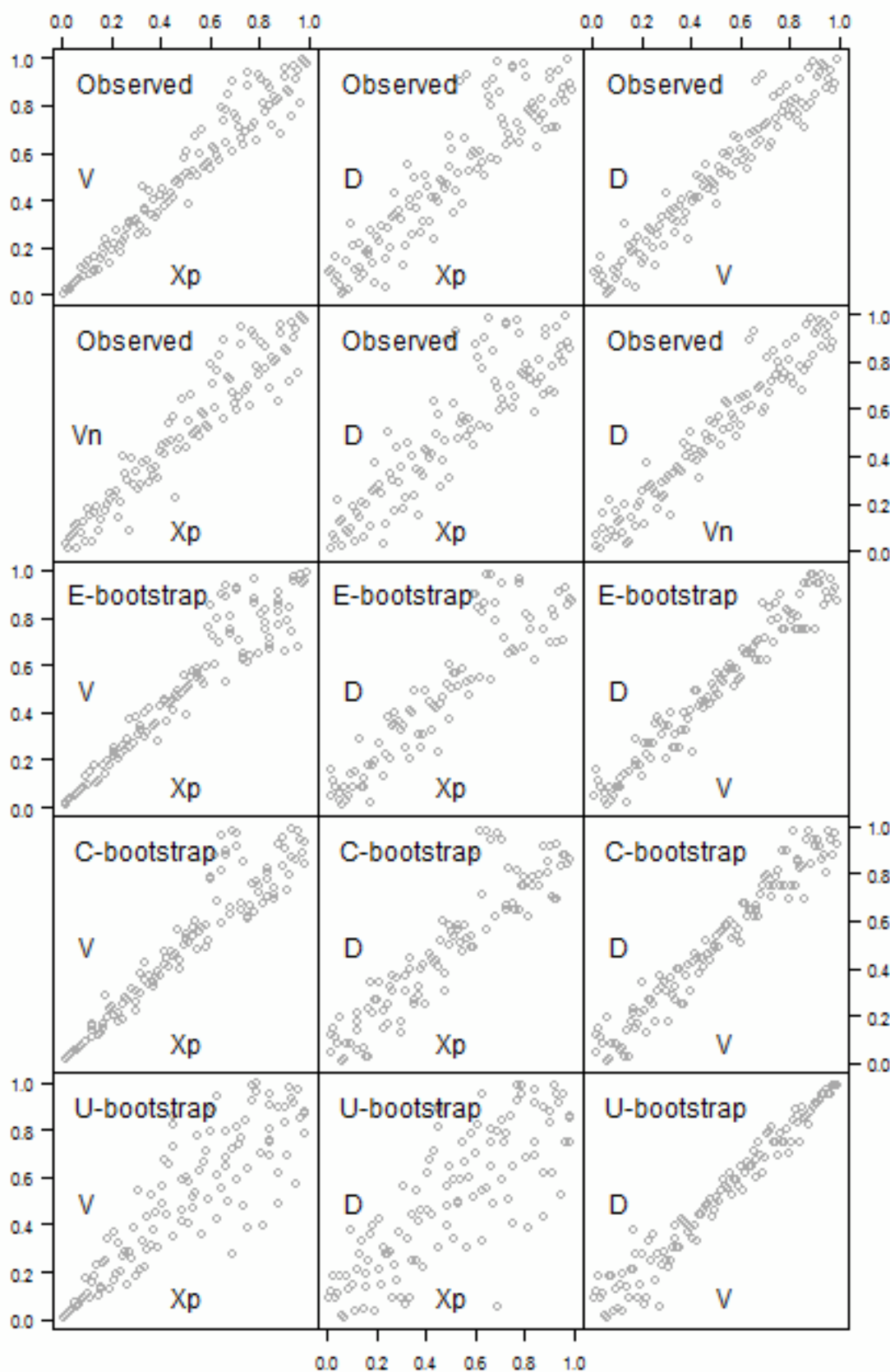

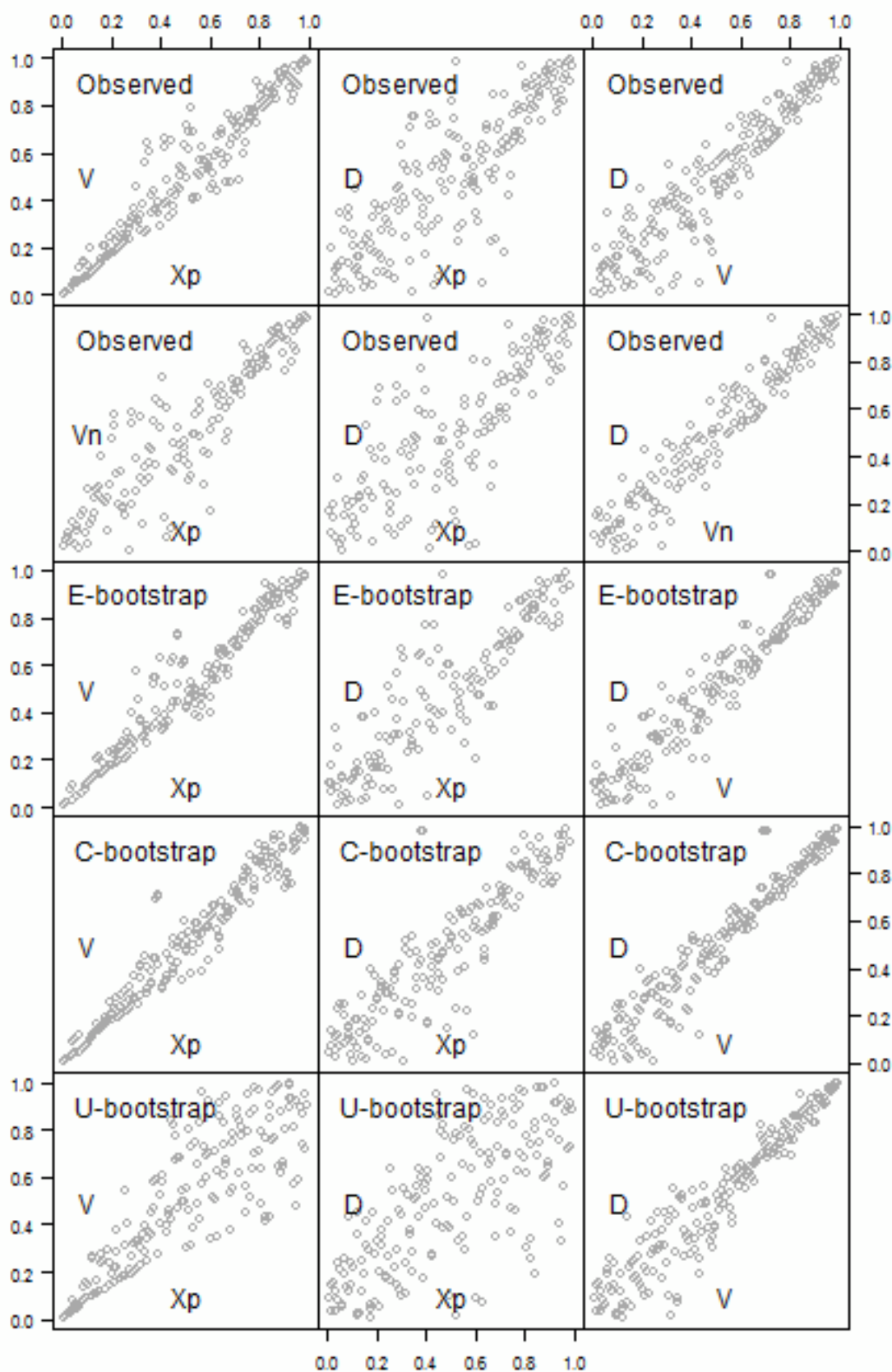

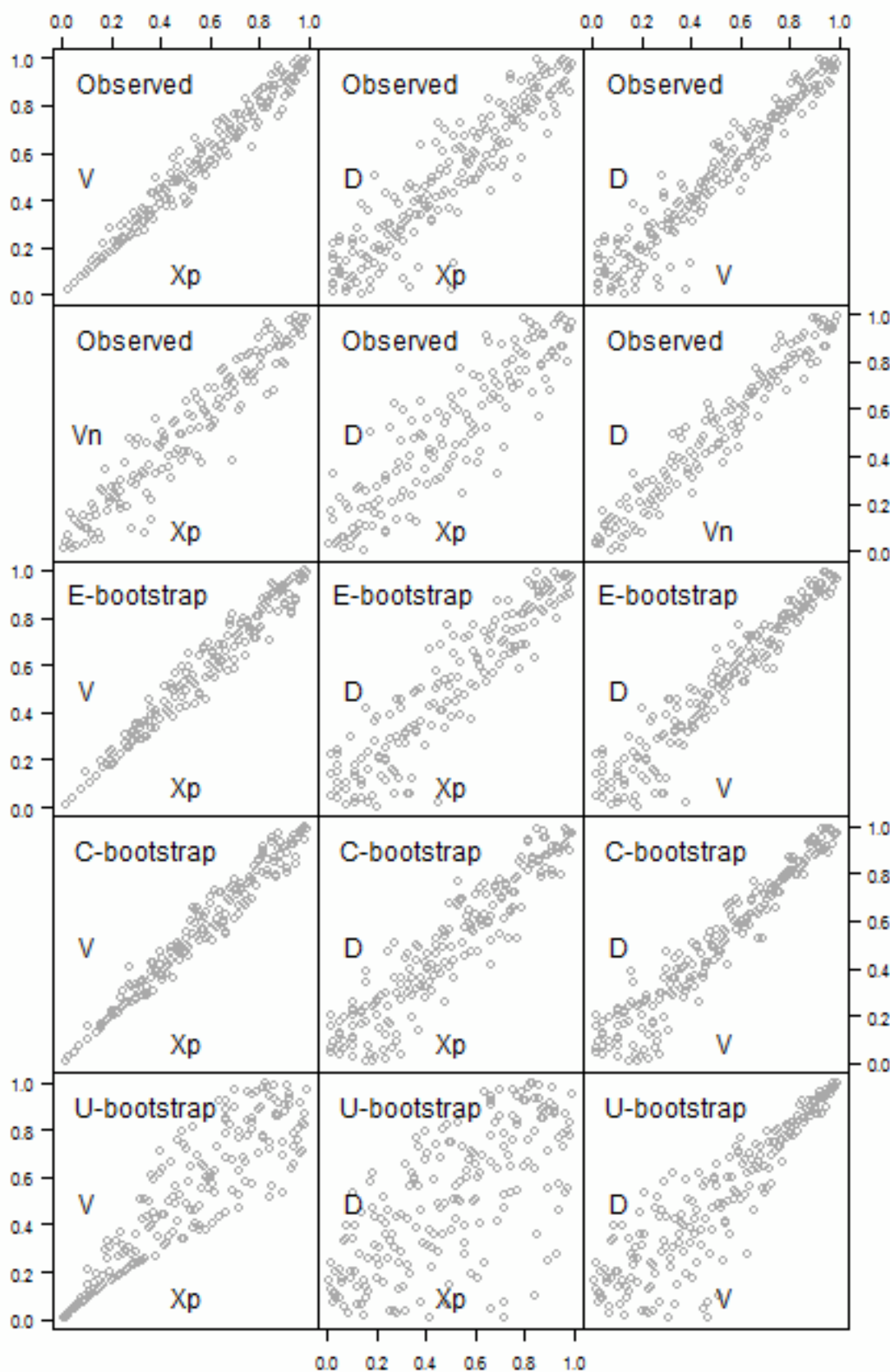

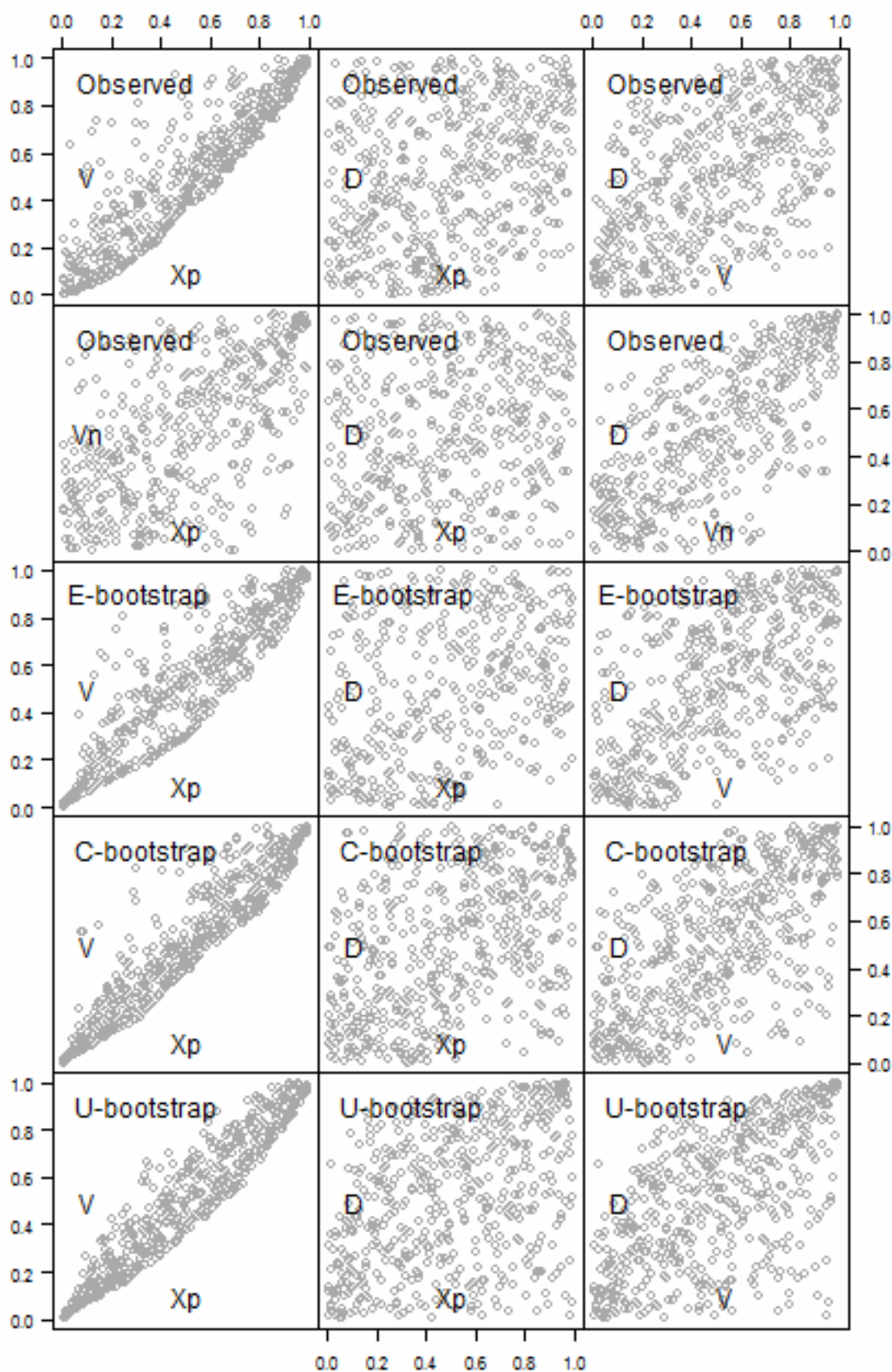

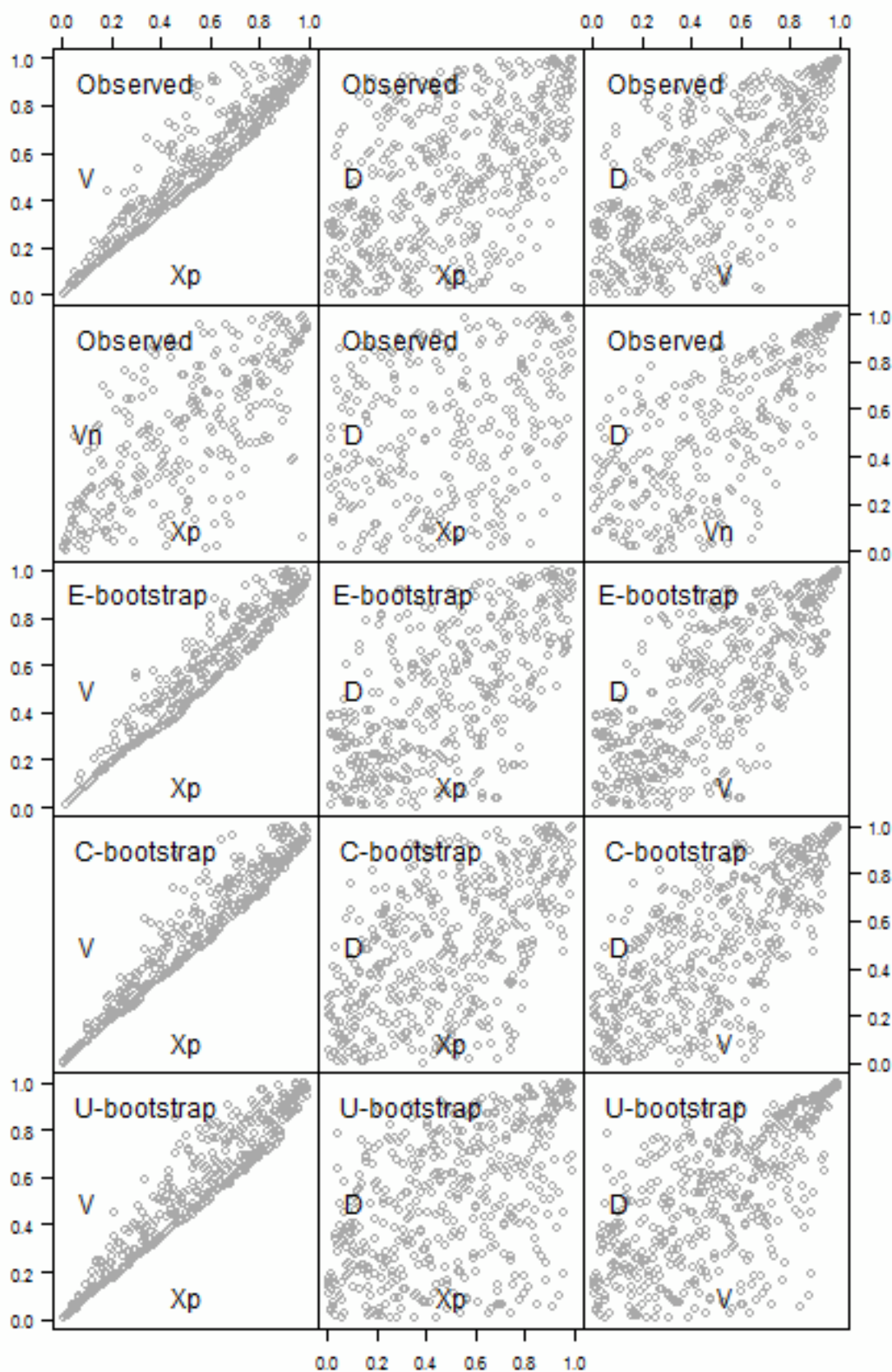

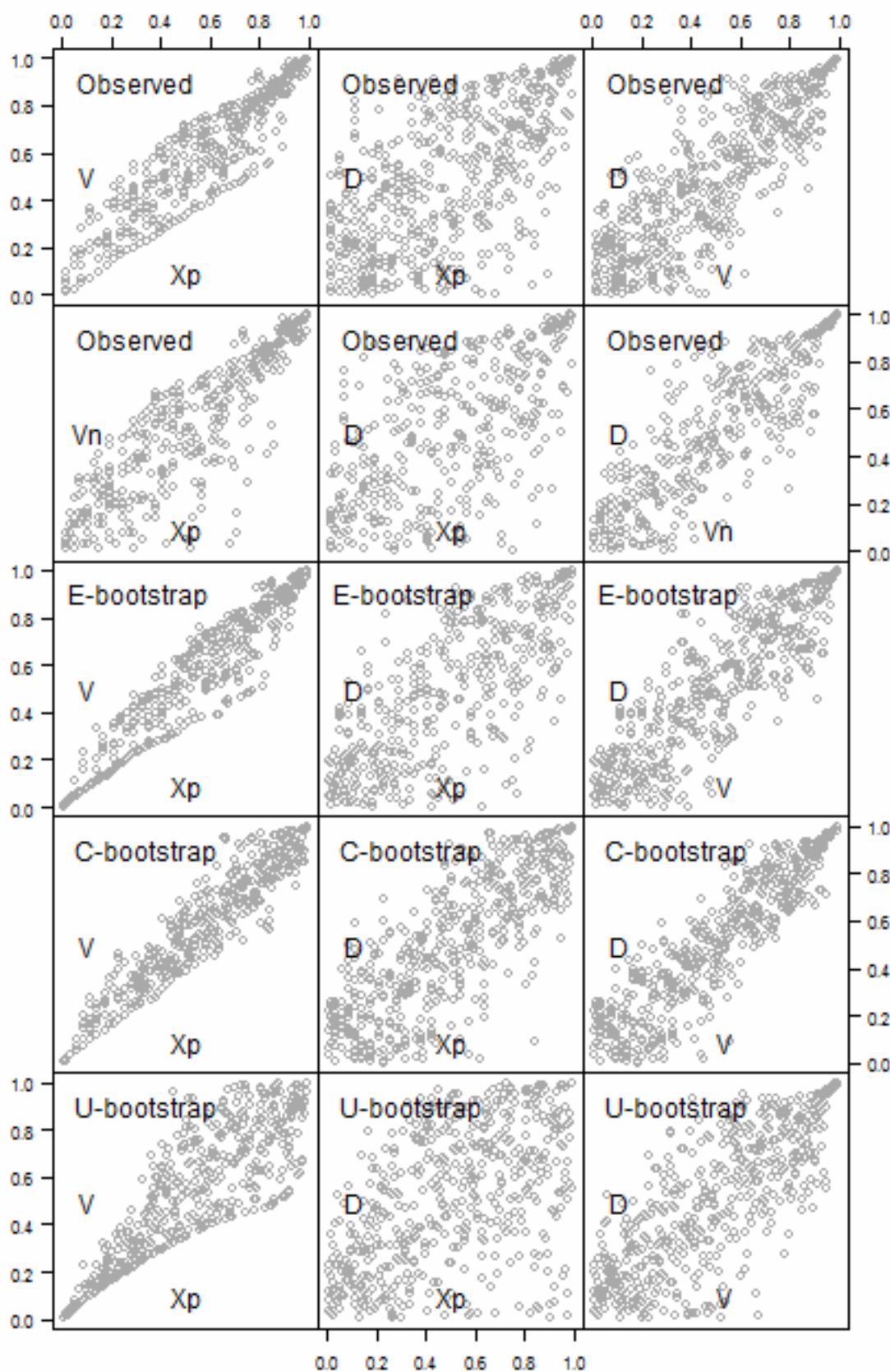

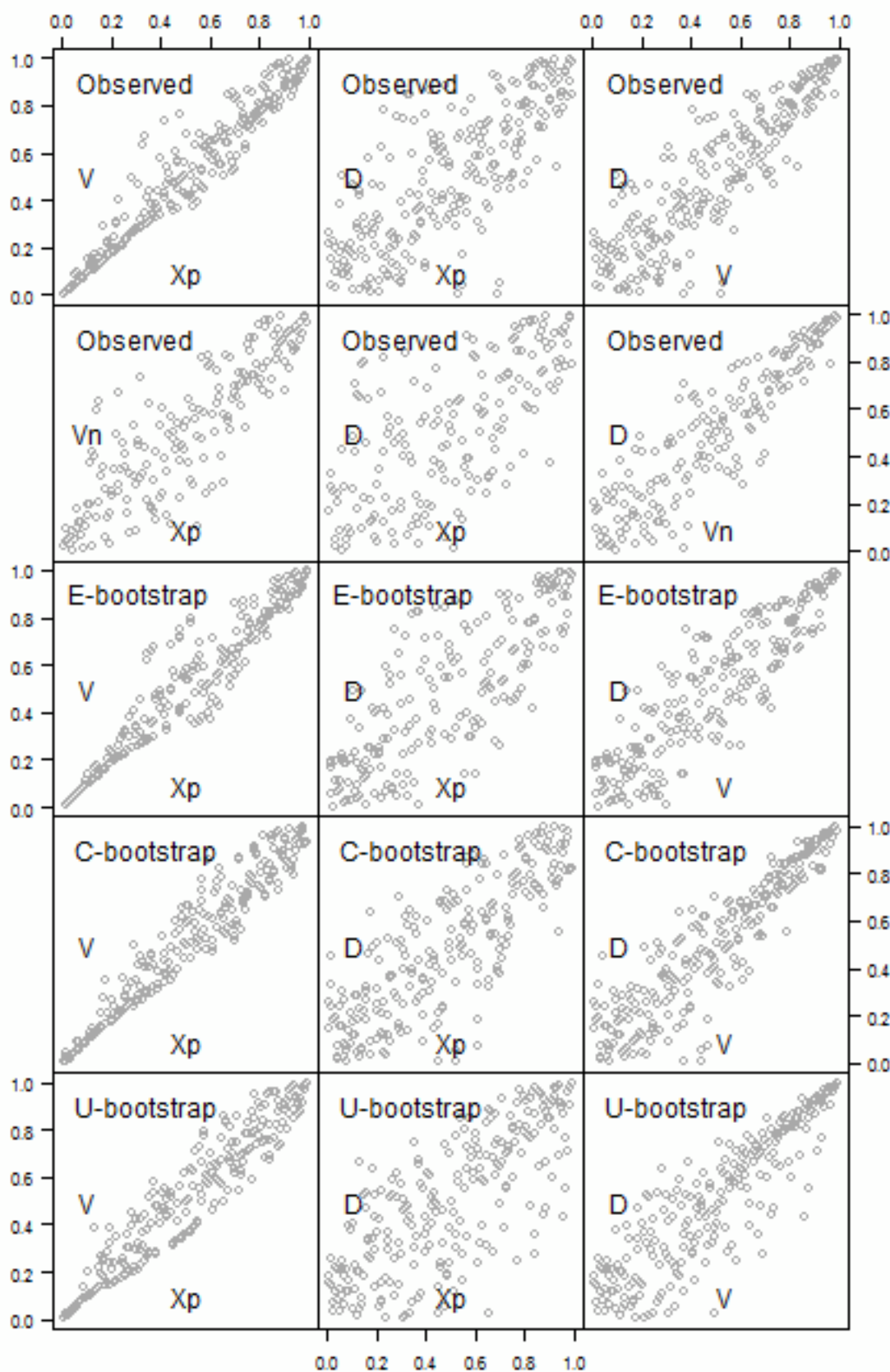

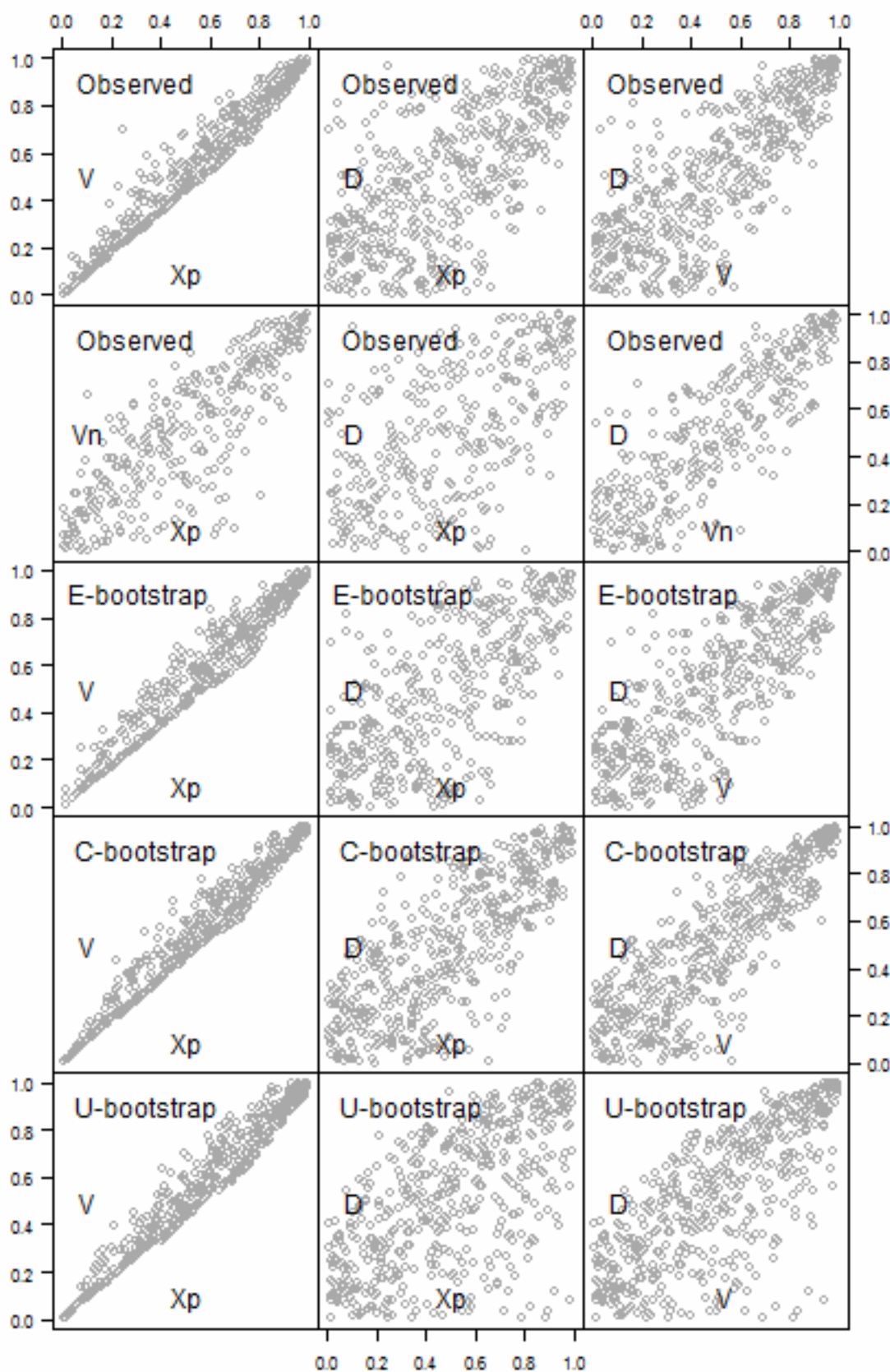

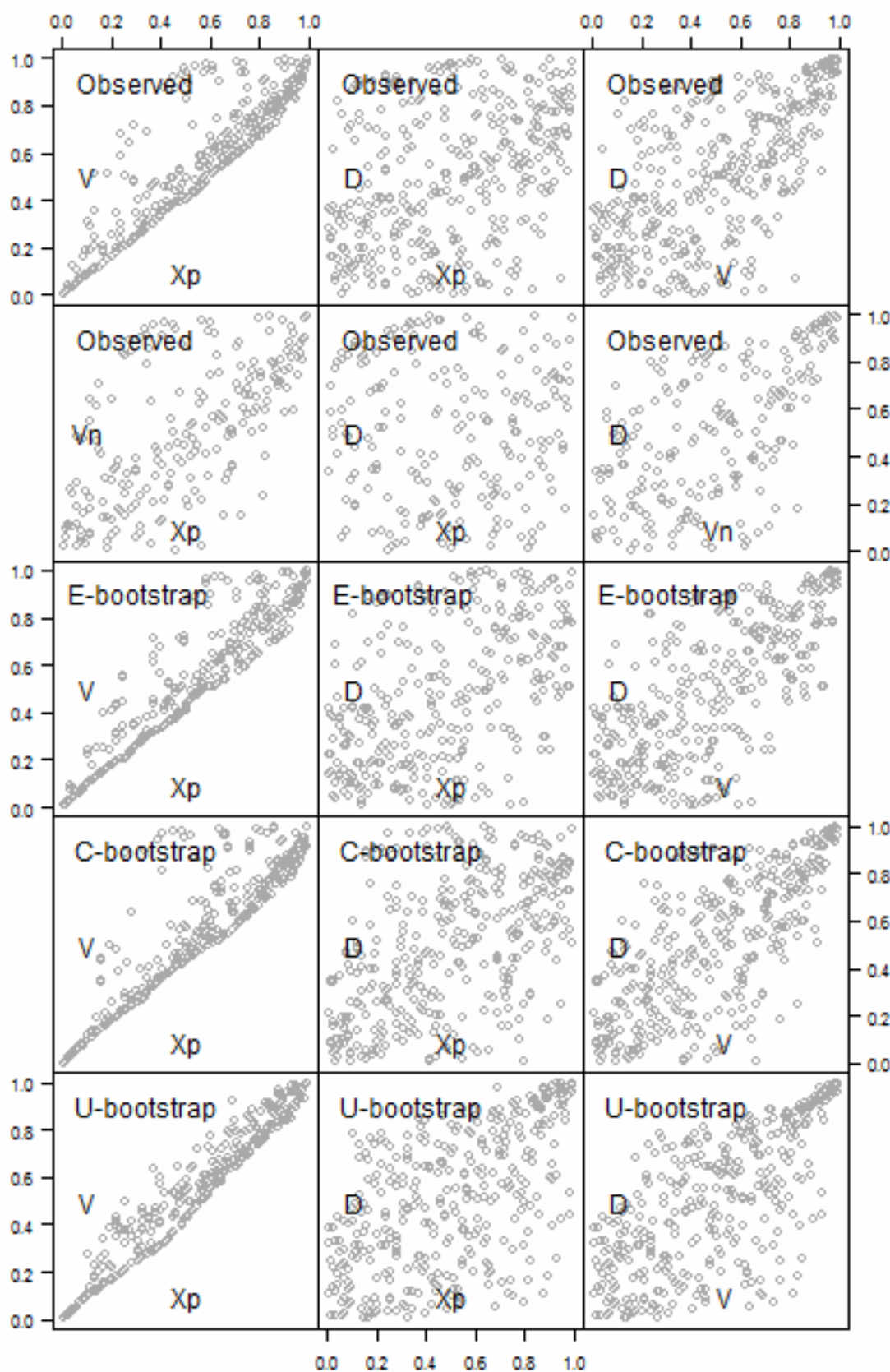

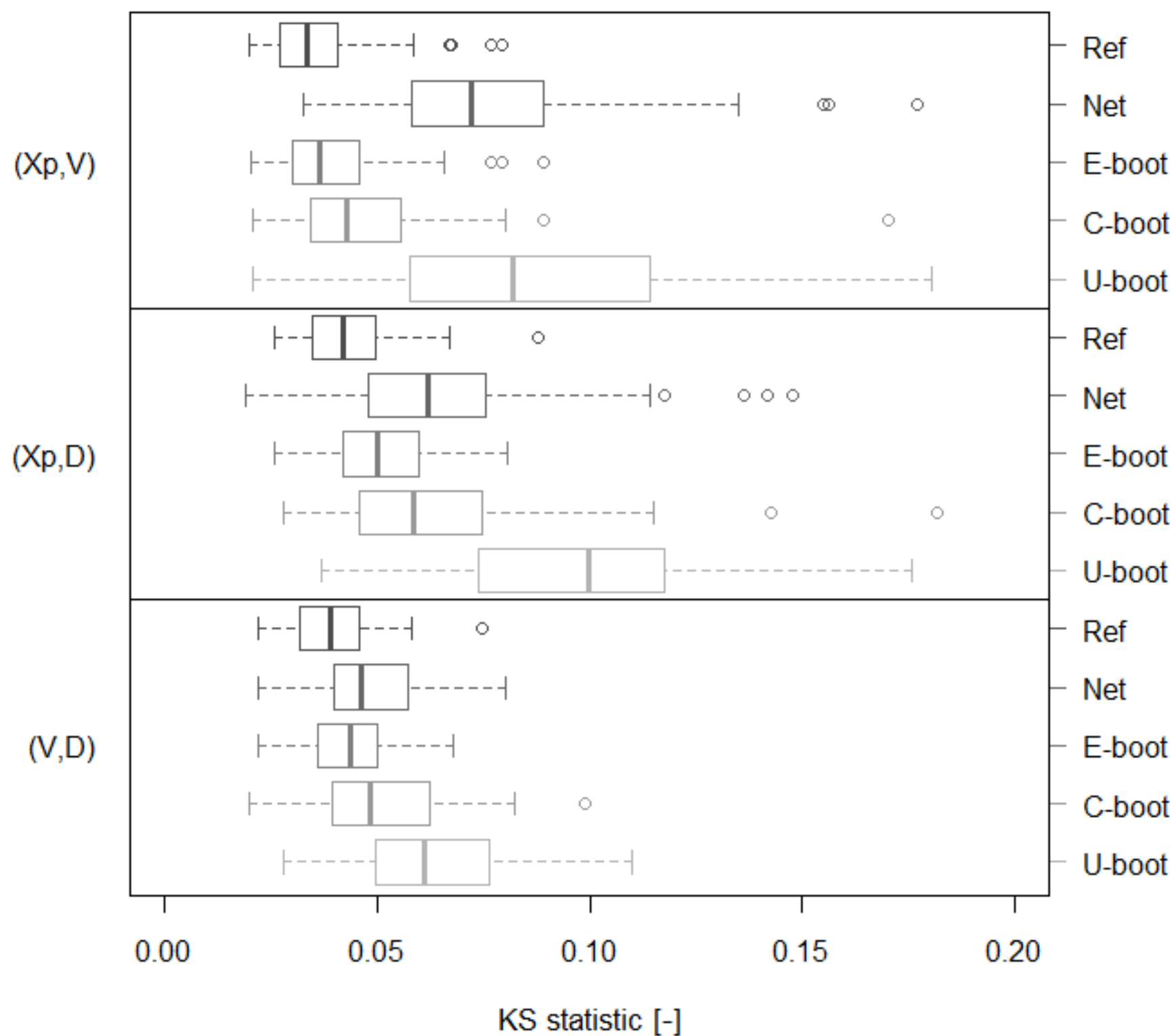

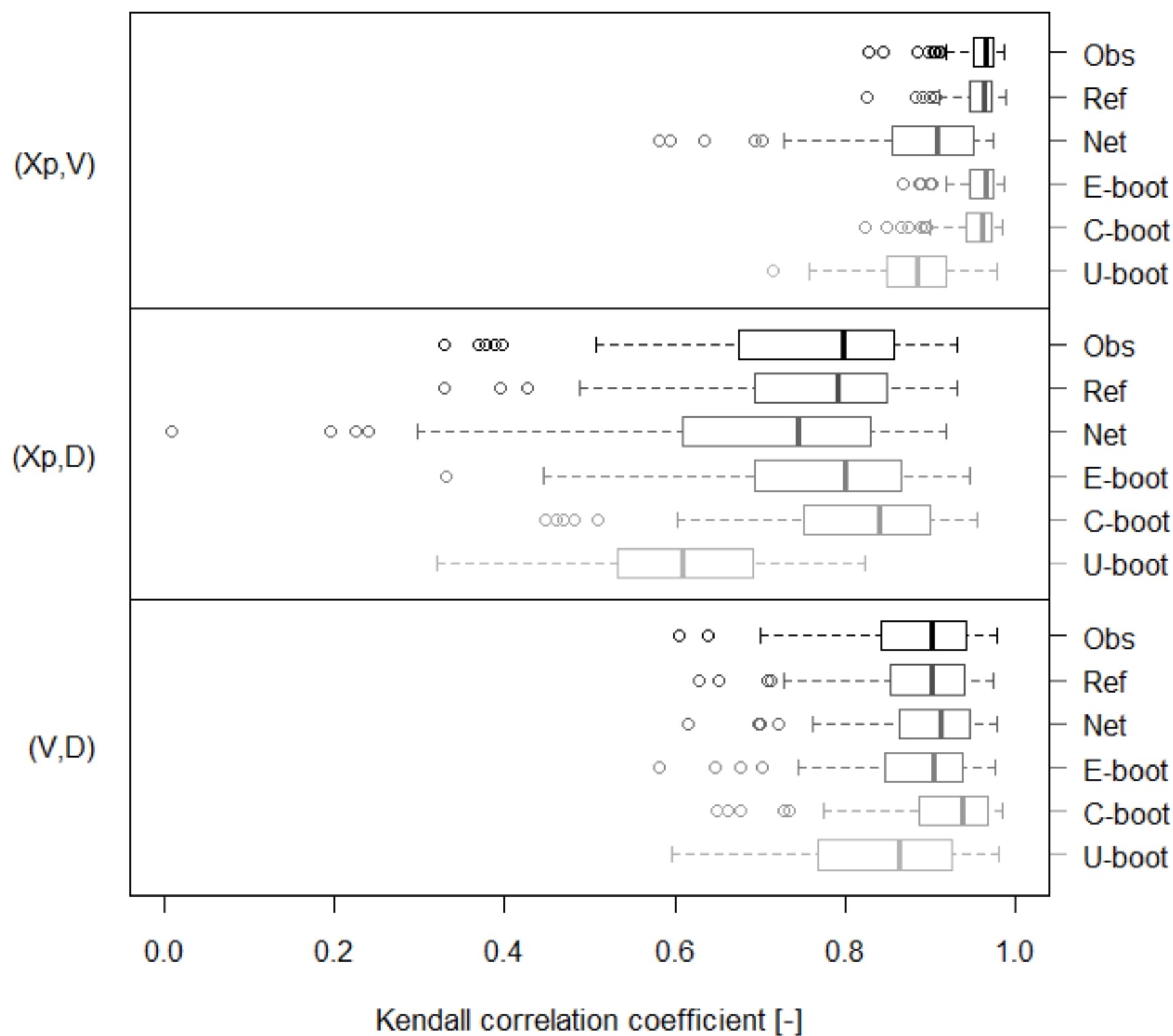

Supplement: Supplementary file 4 — Supplementary Figures [file wrcr0049-3423-sd4.pdf]

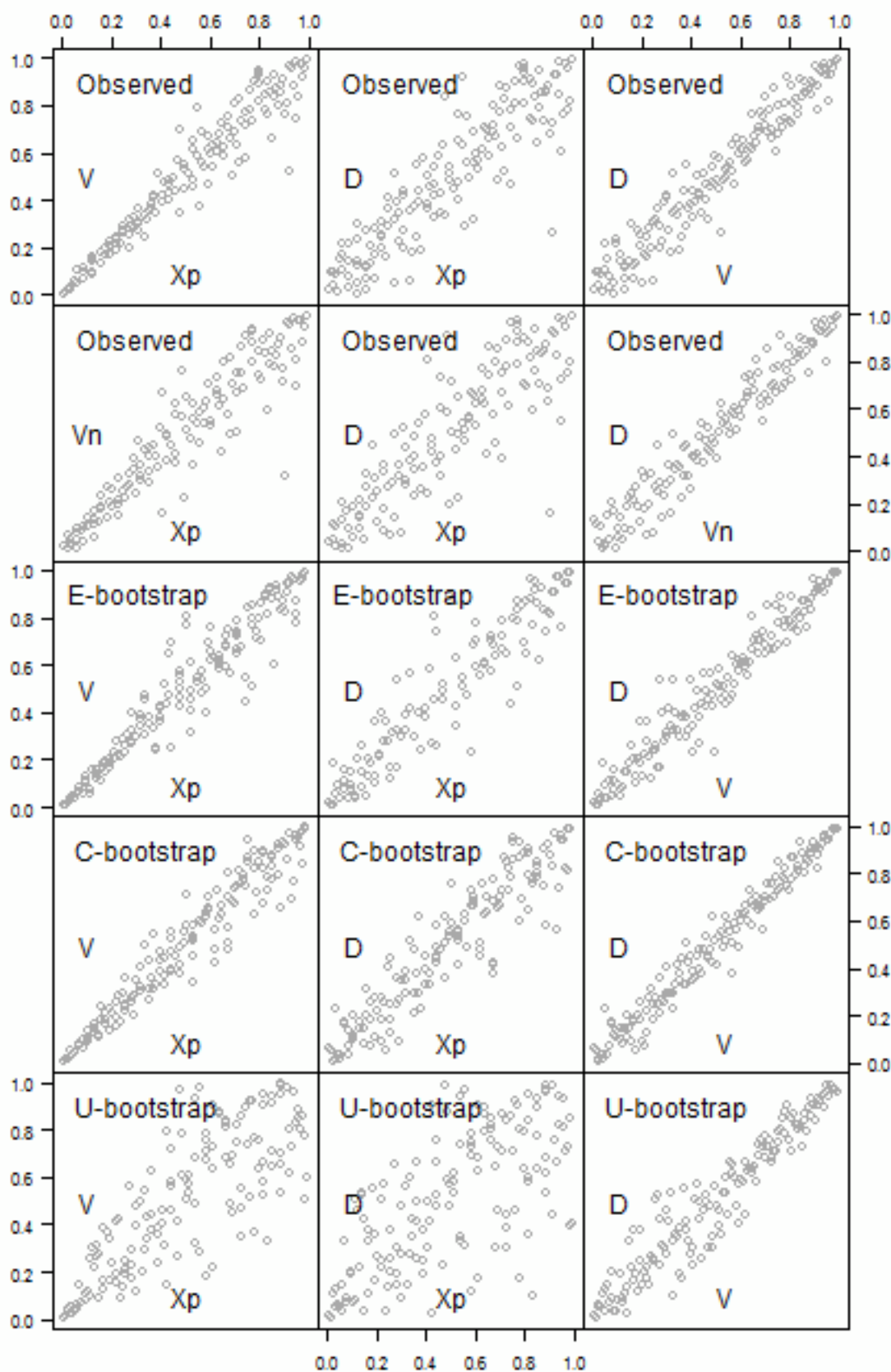

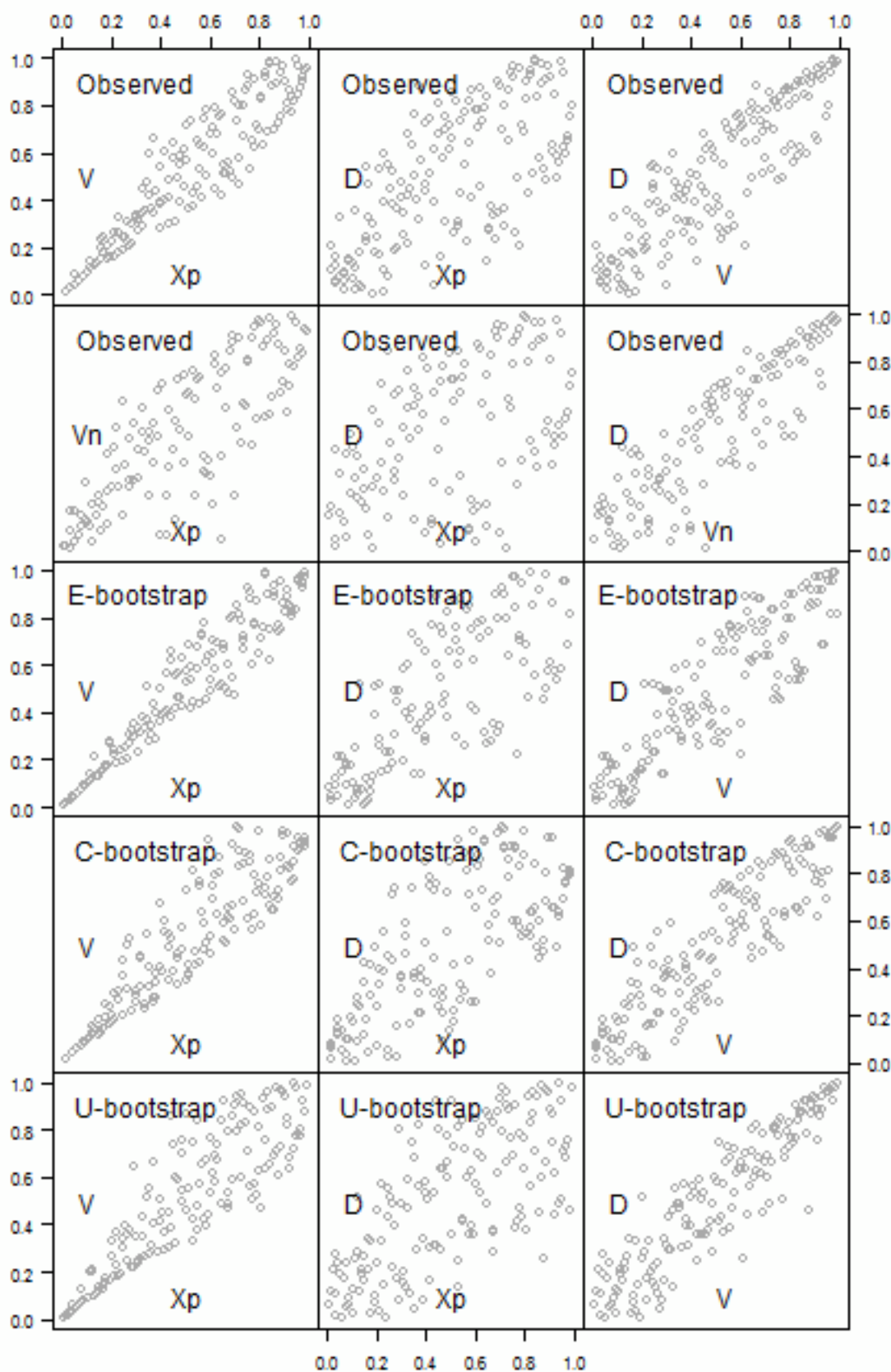

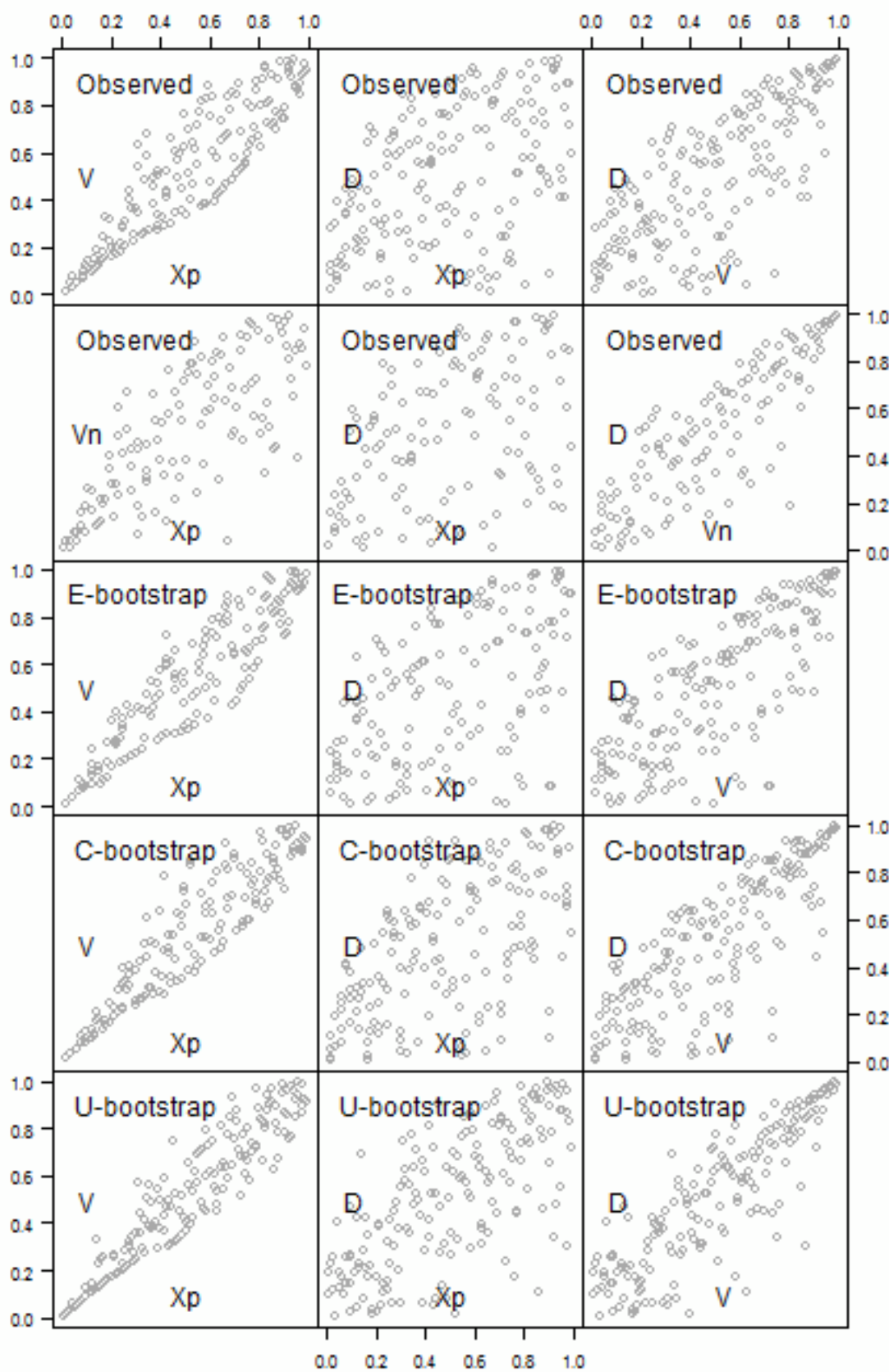

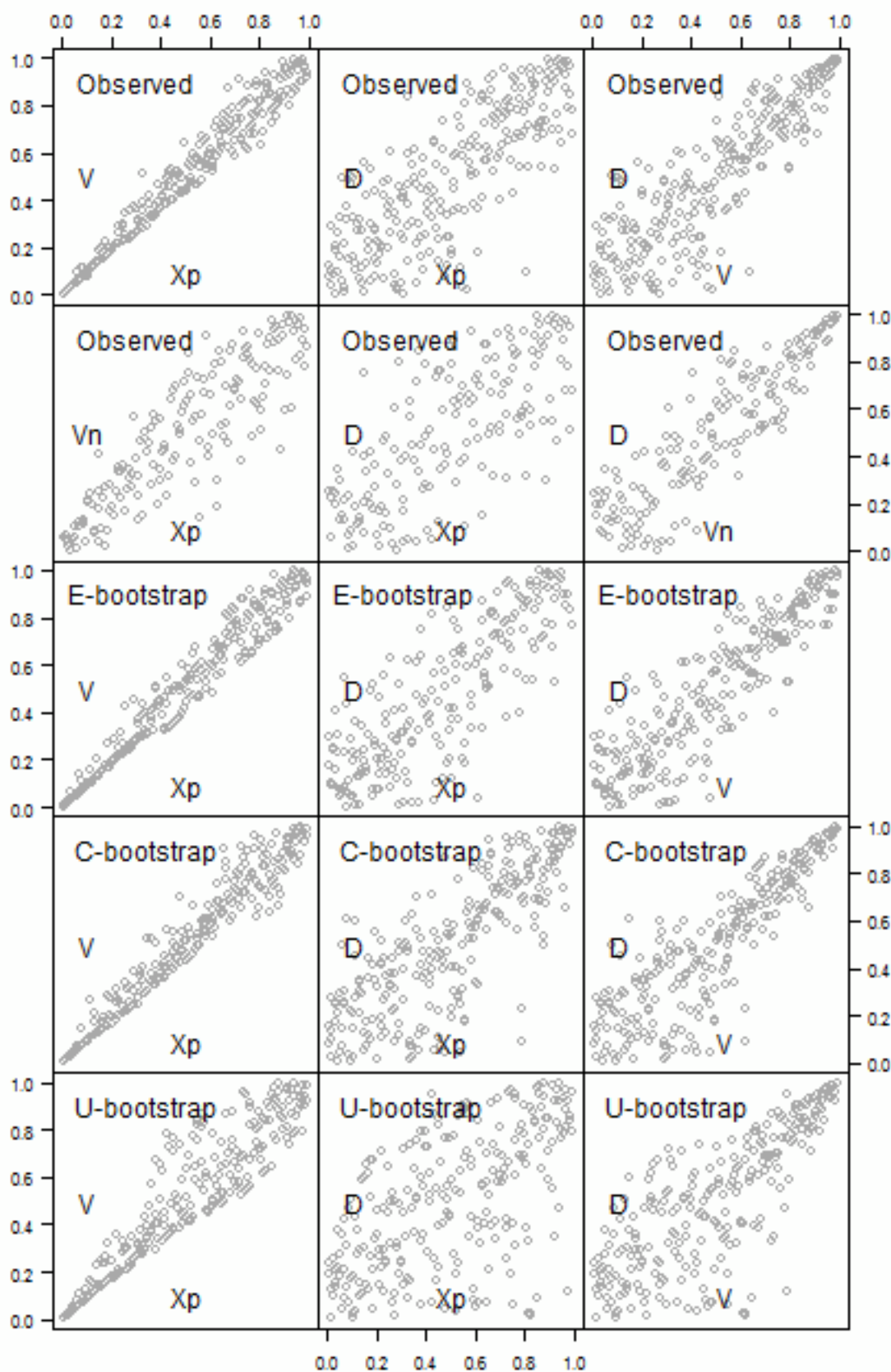

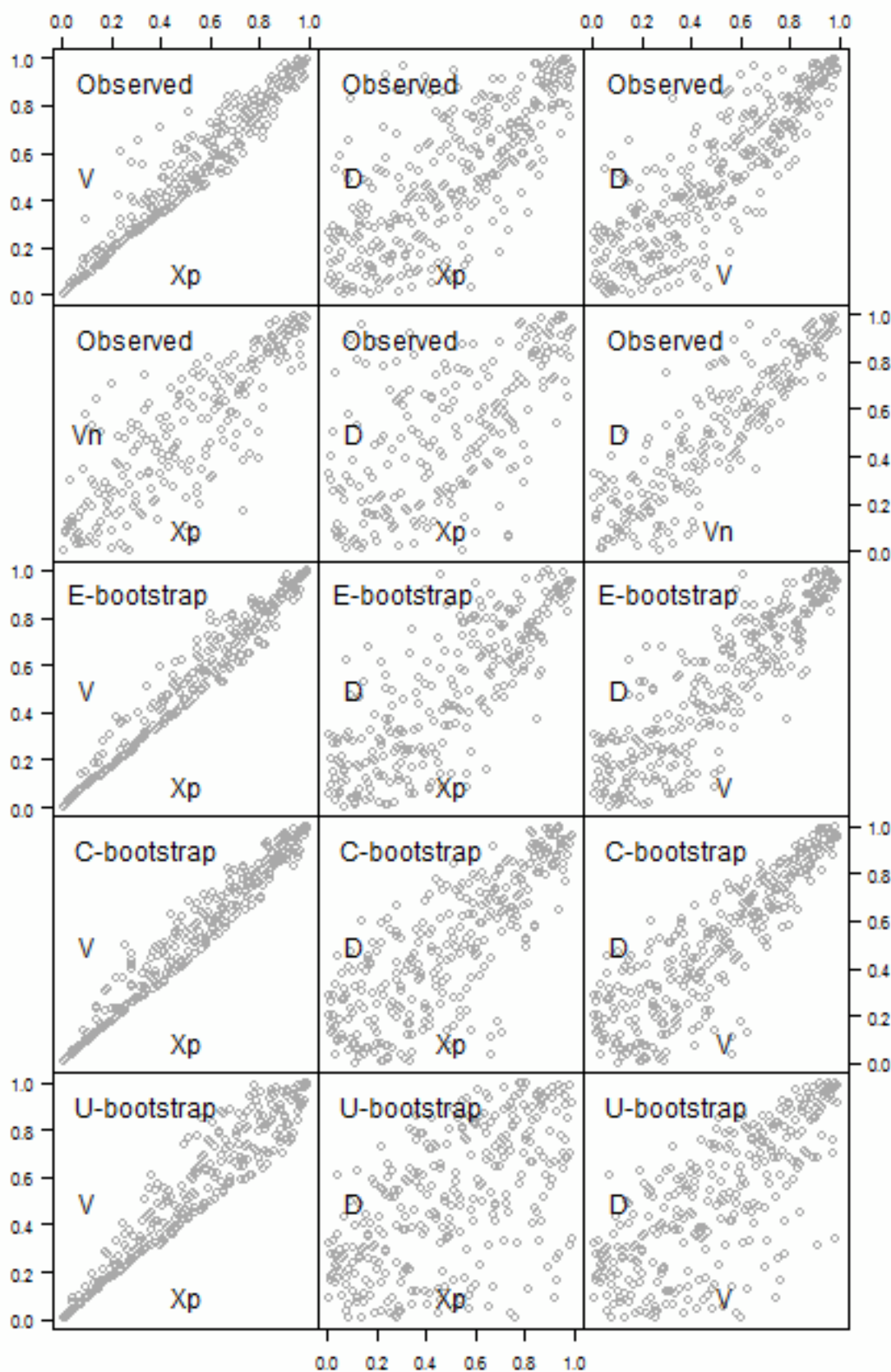

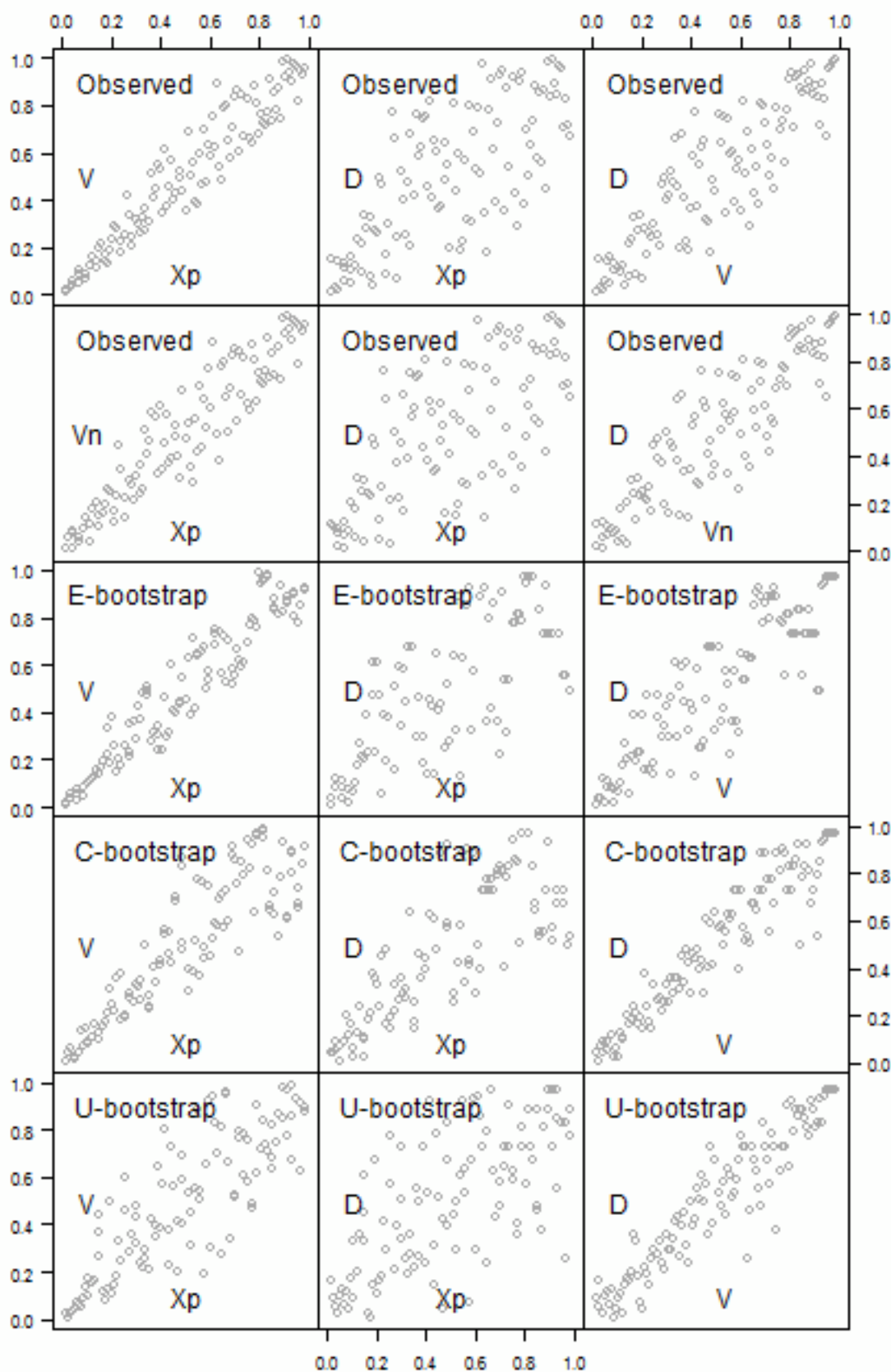

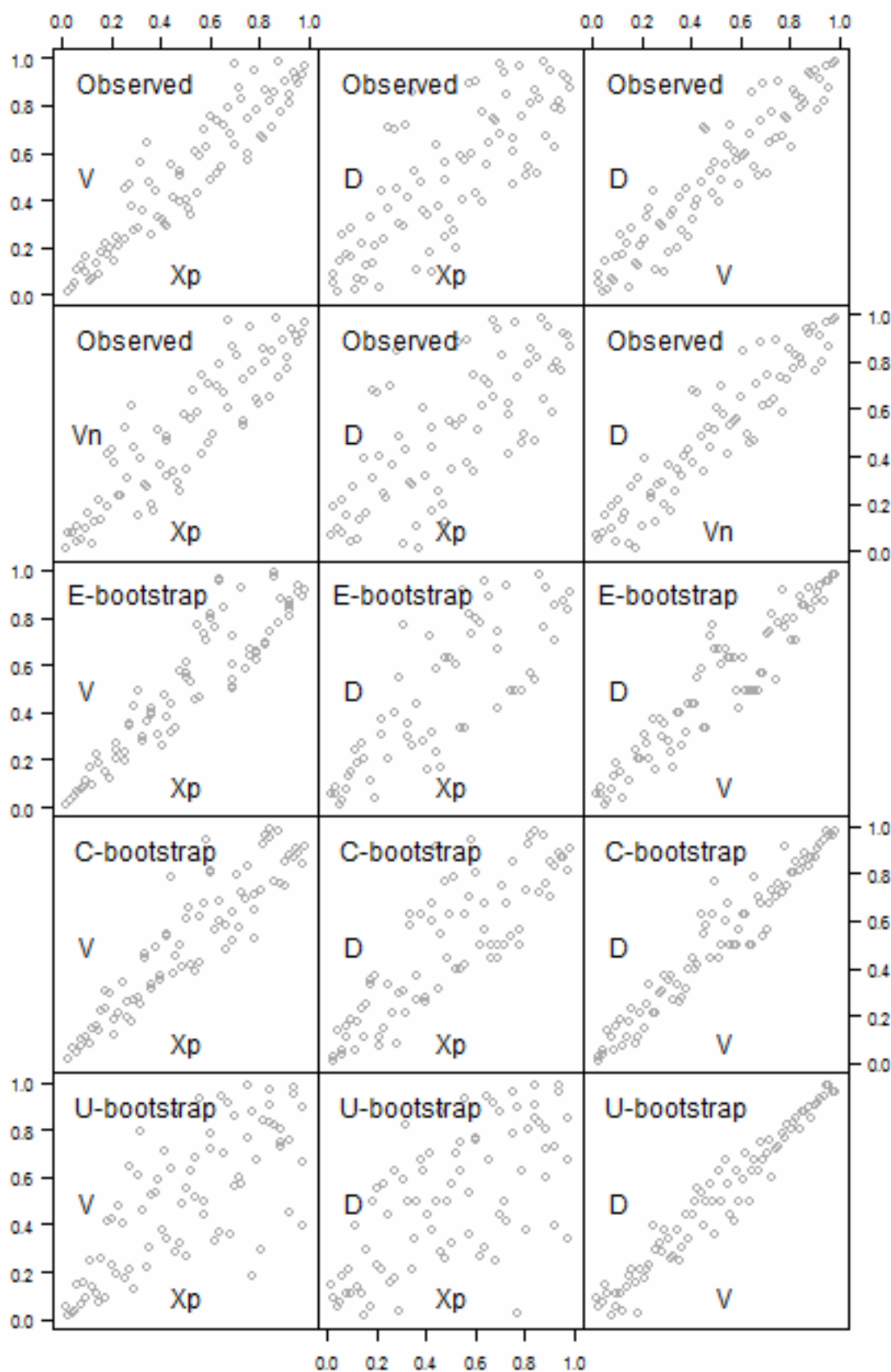

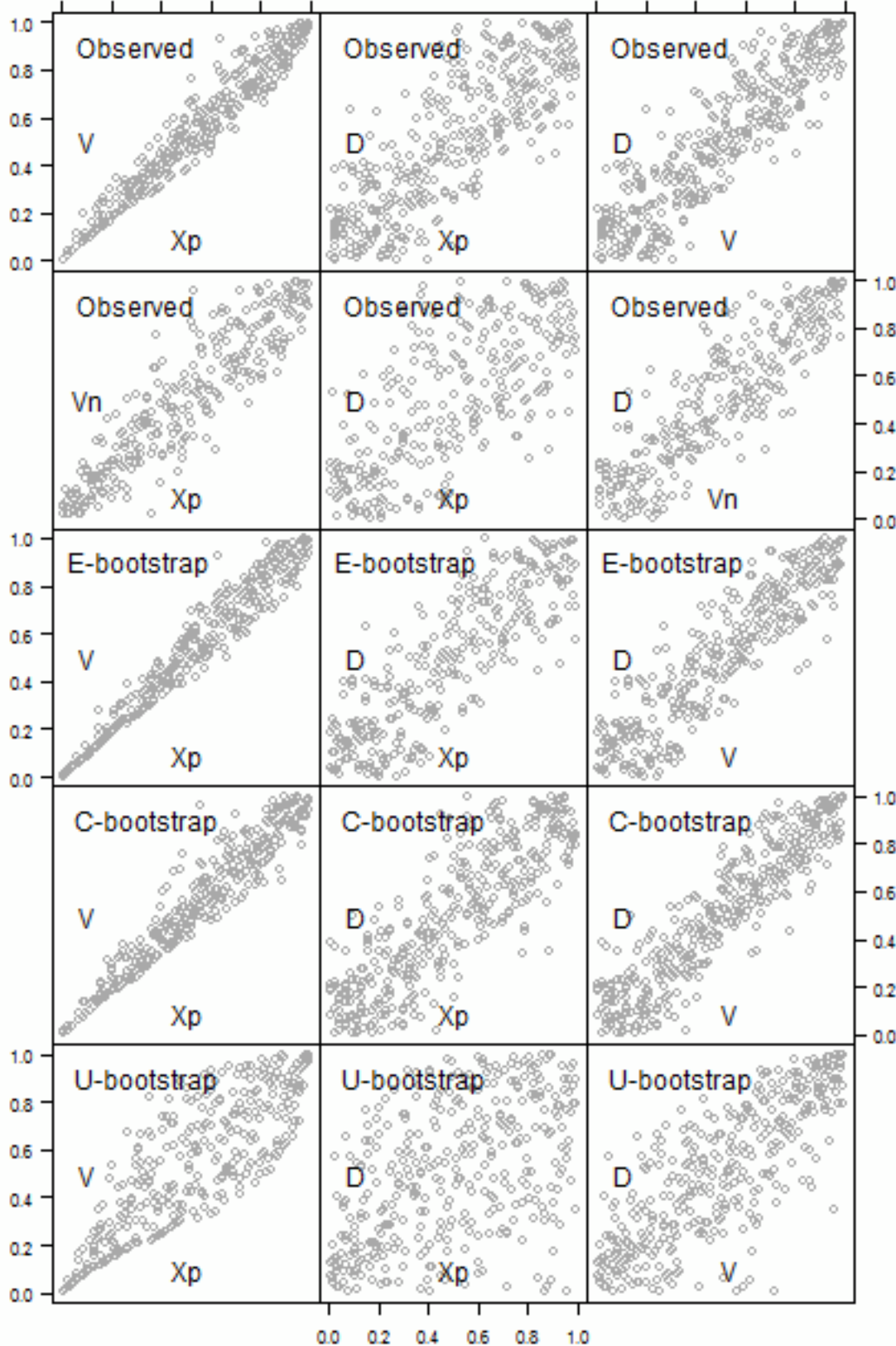

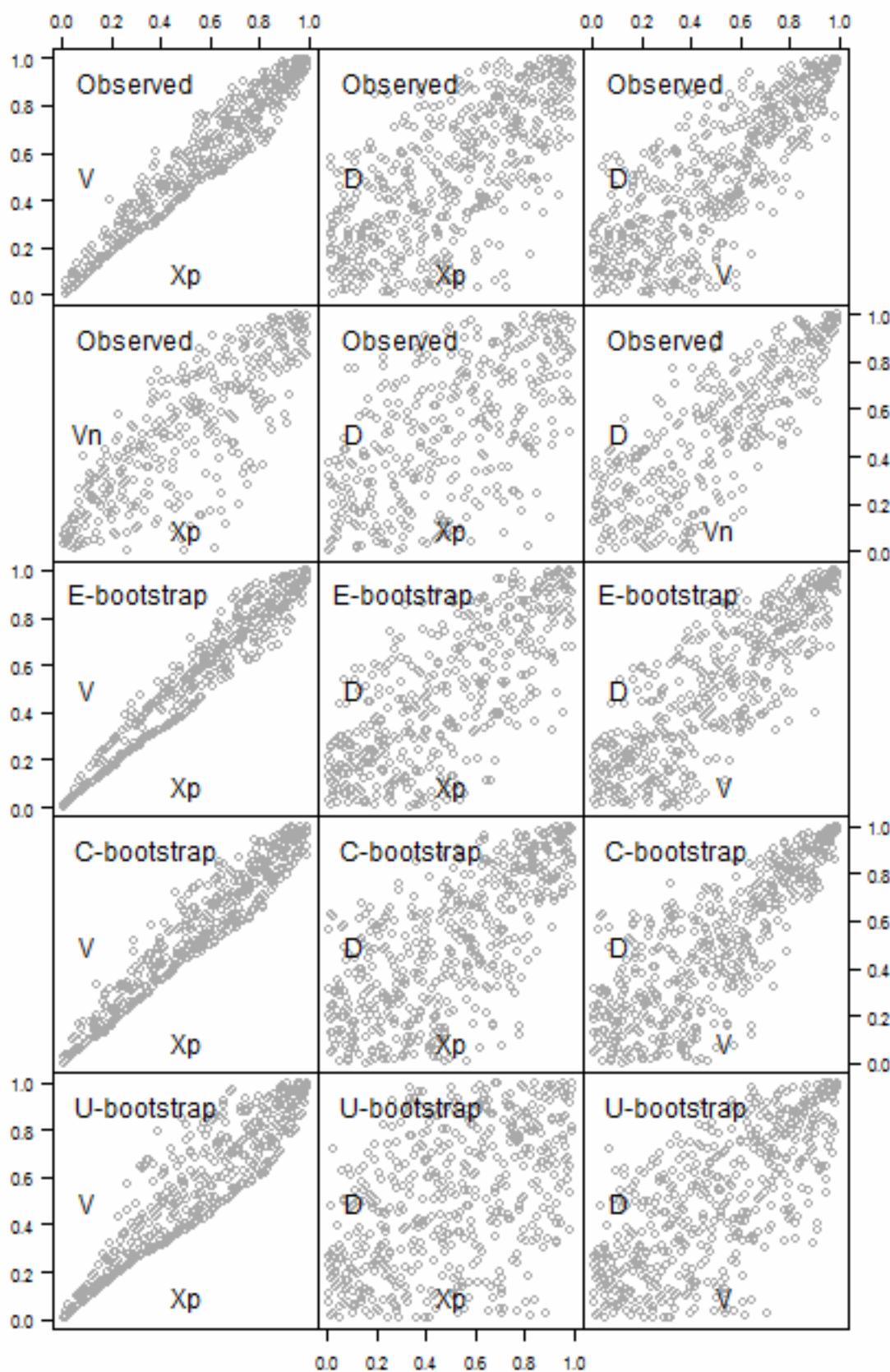

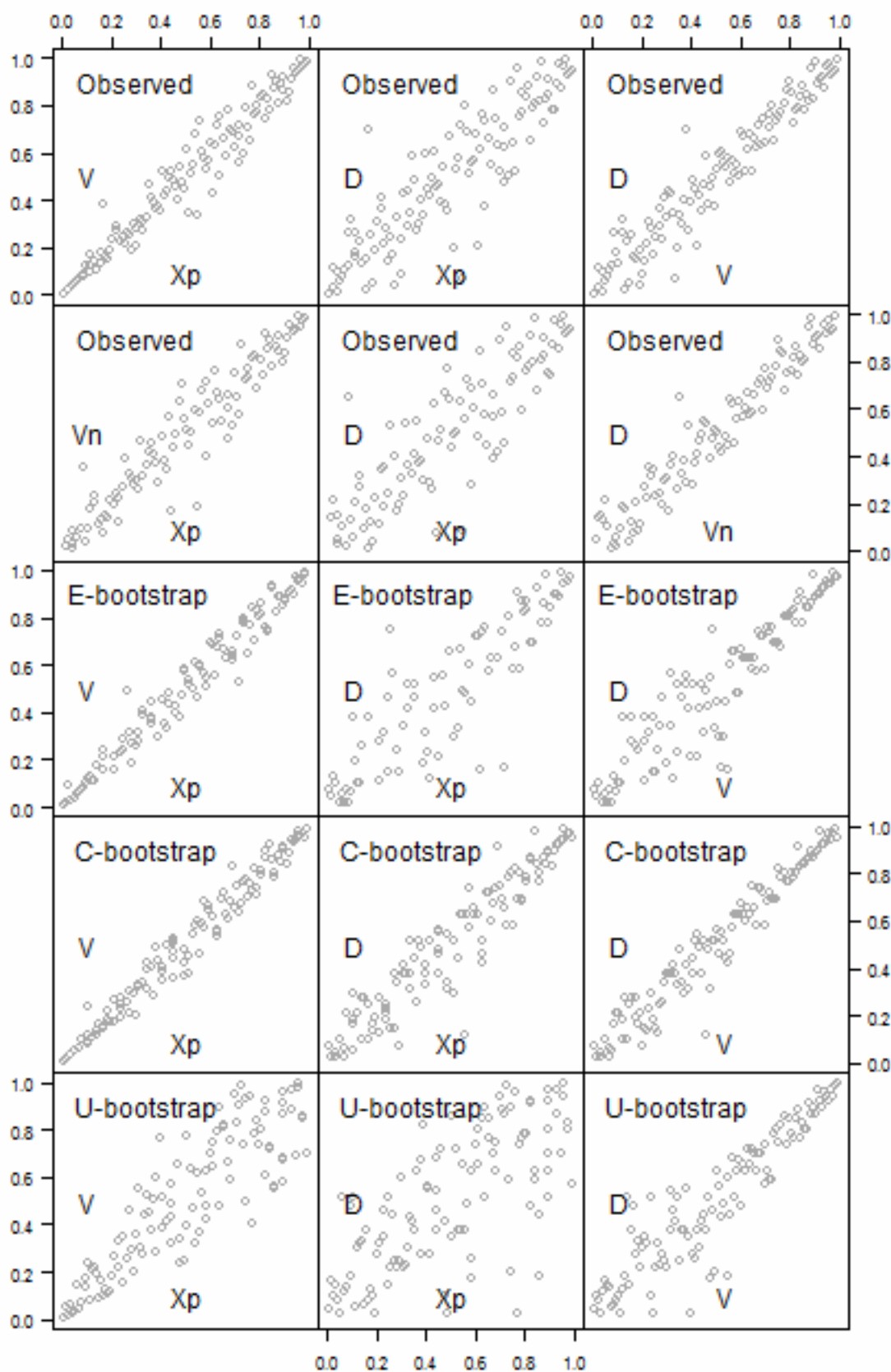

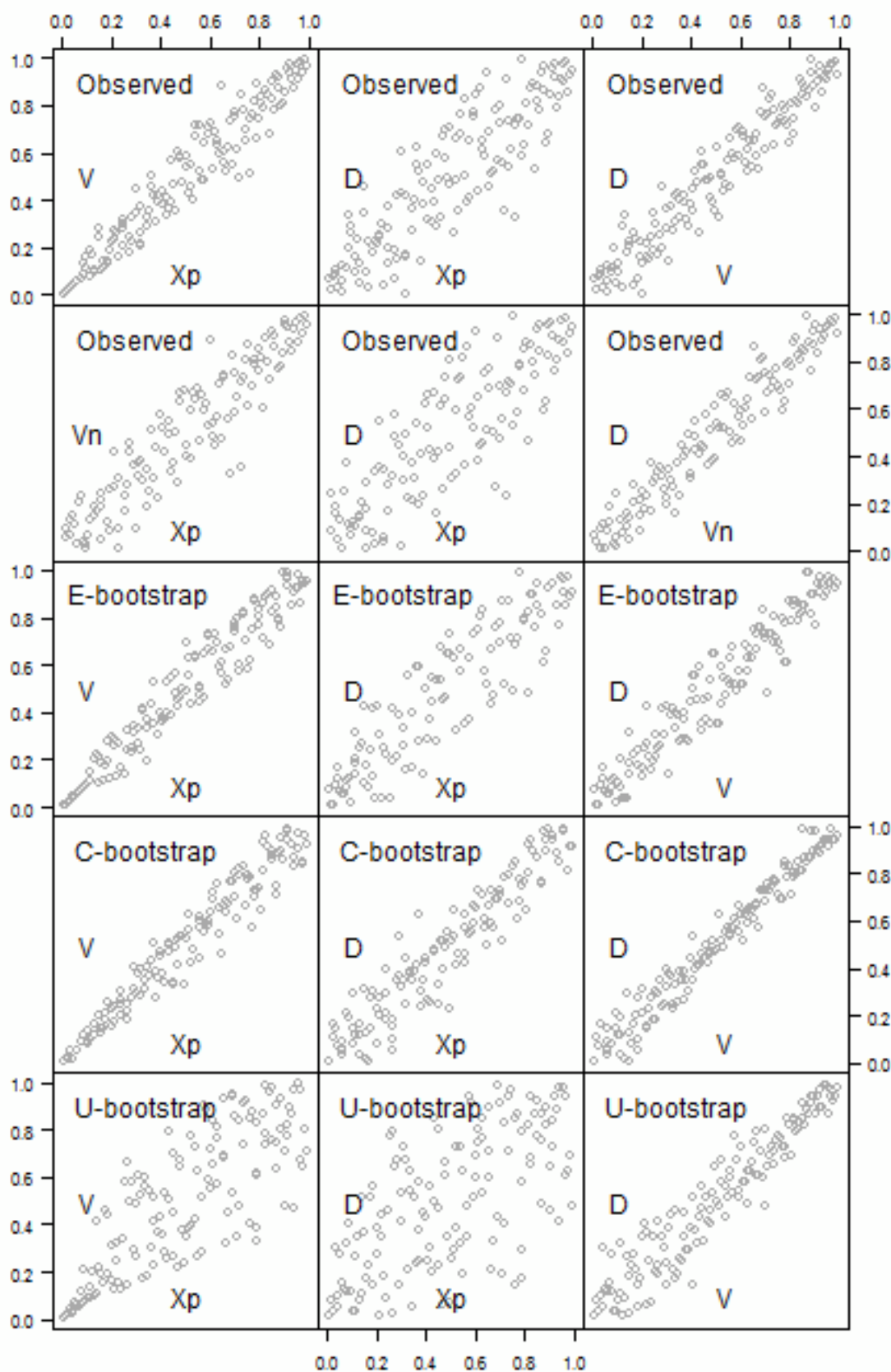

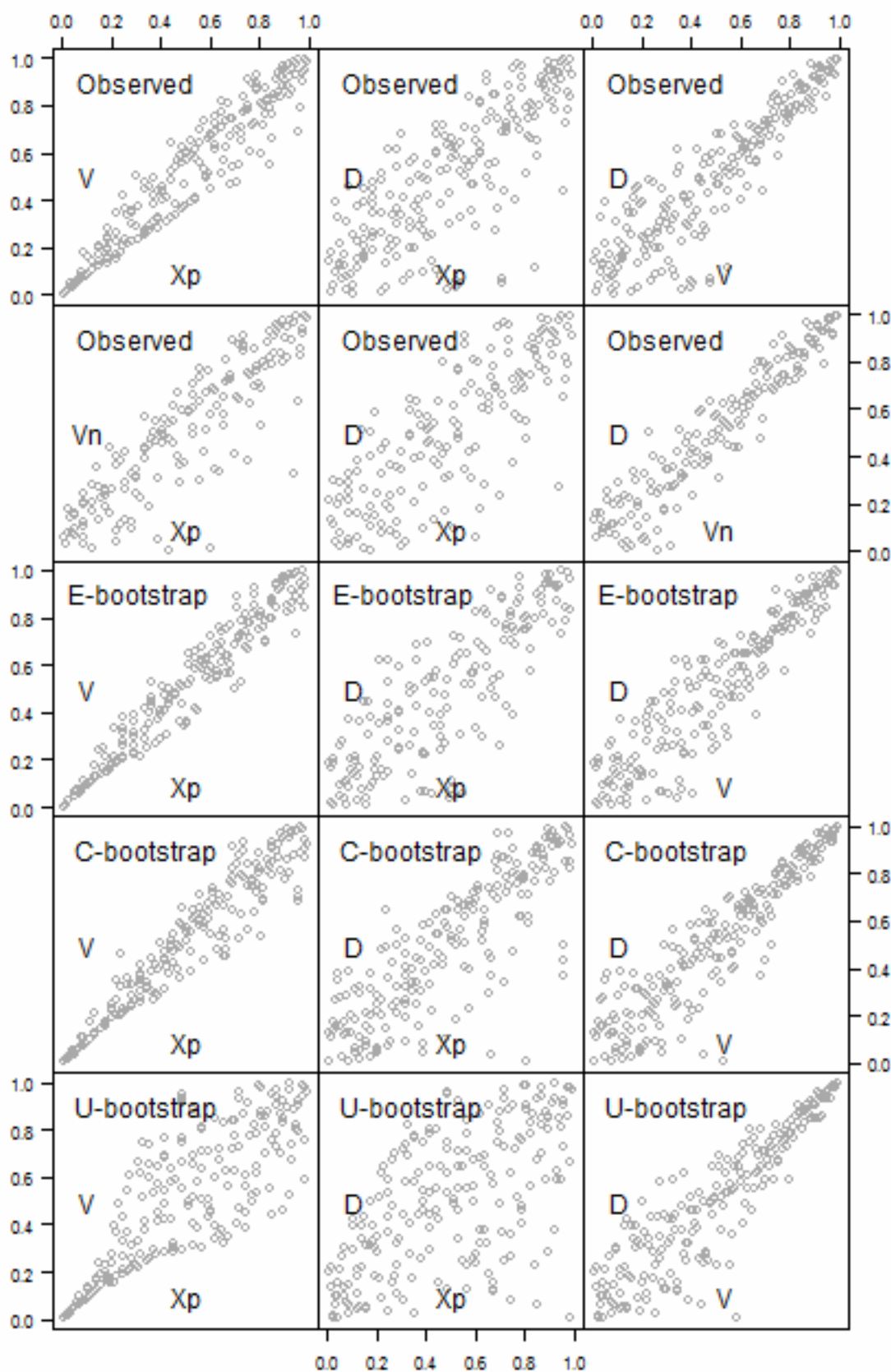

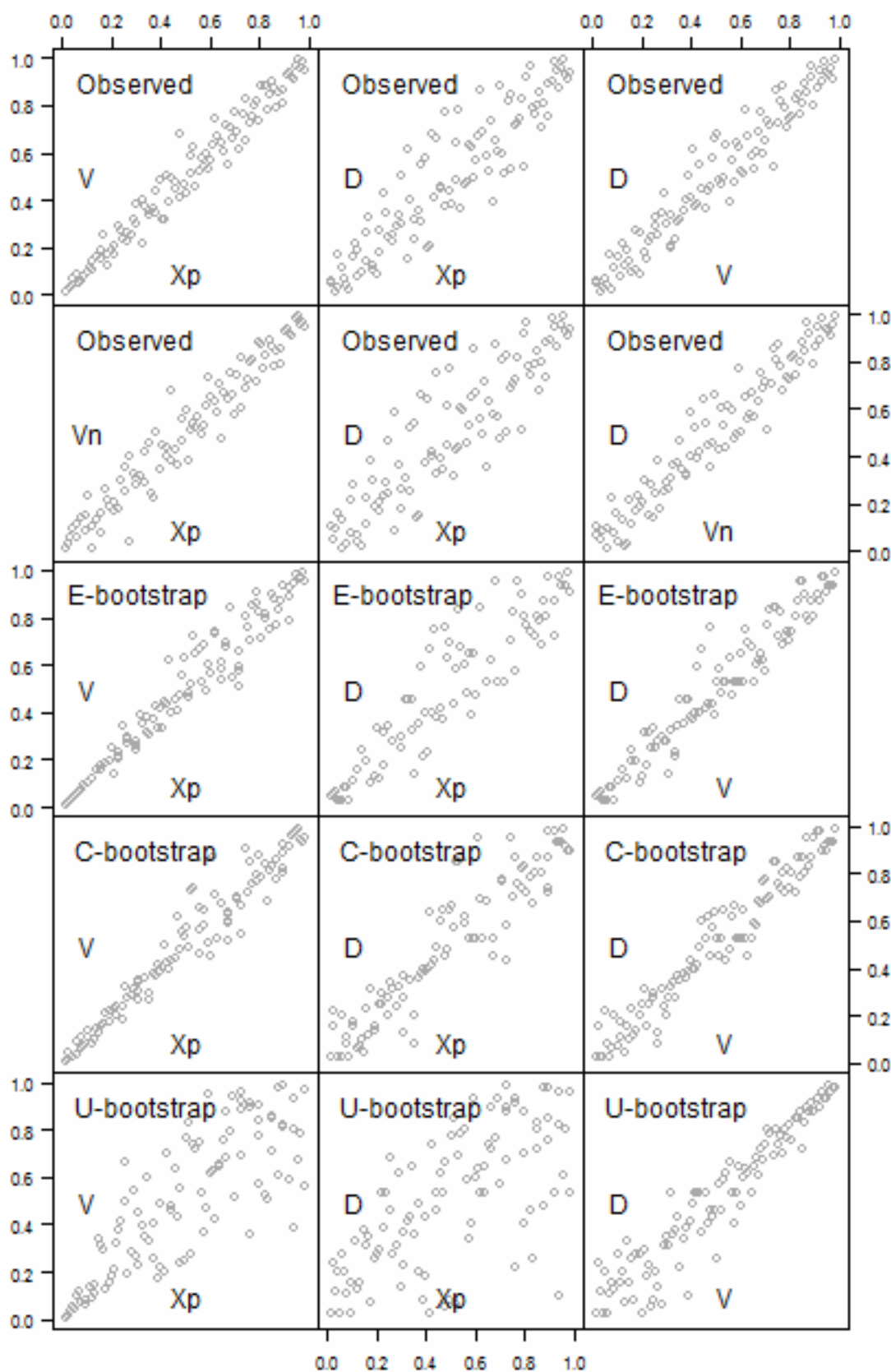

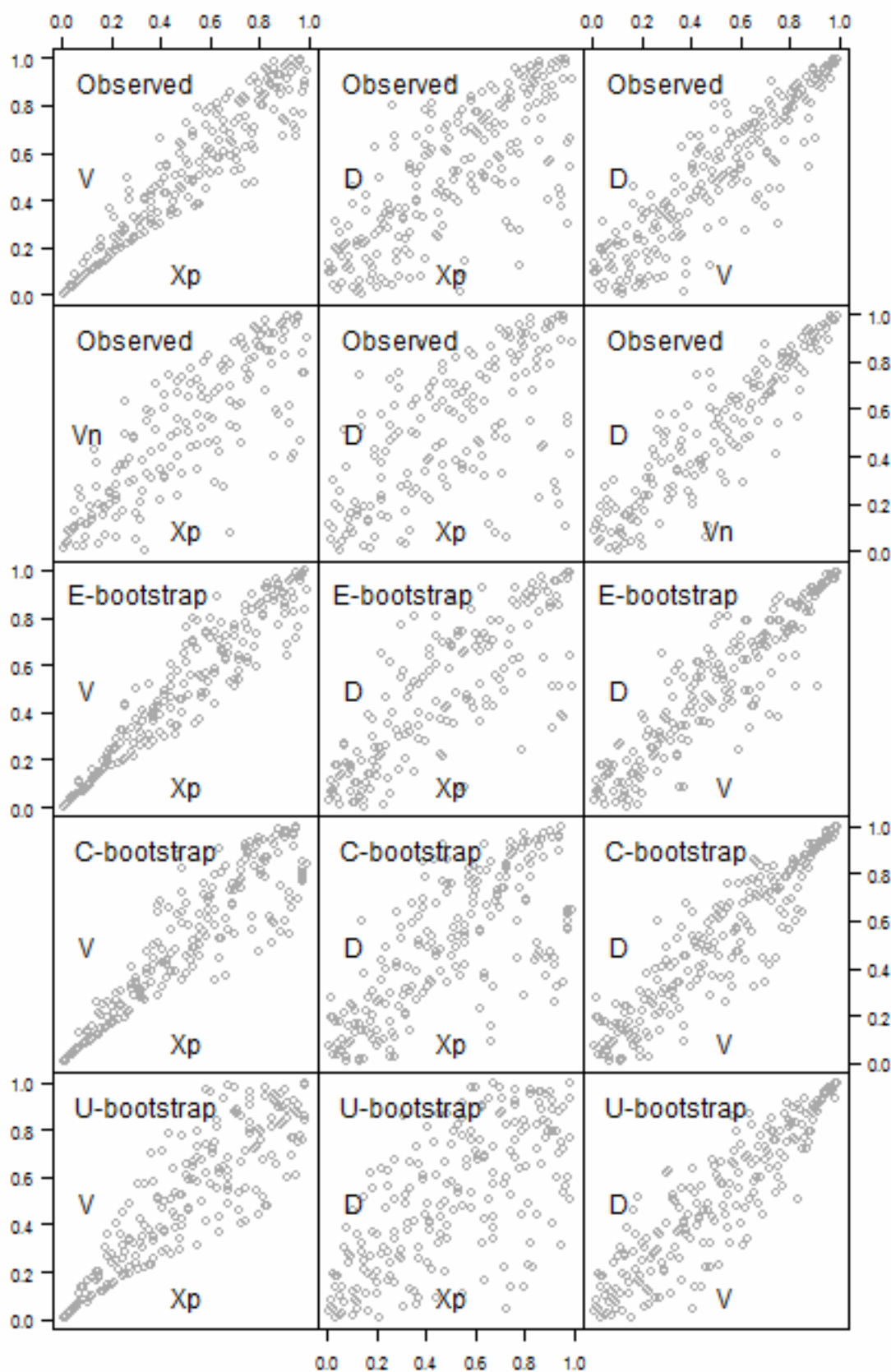

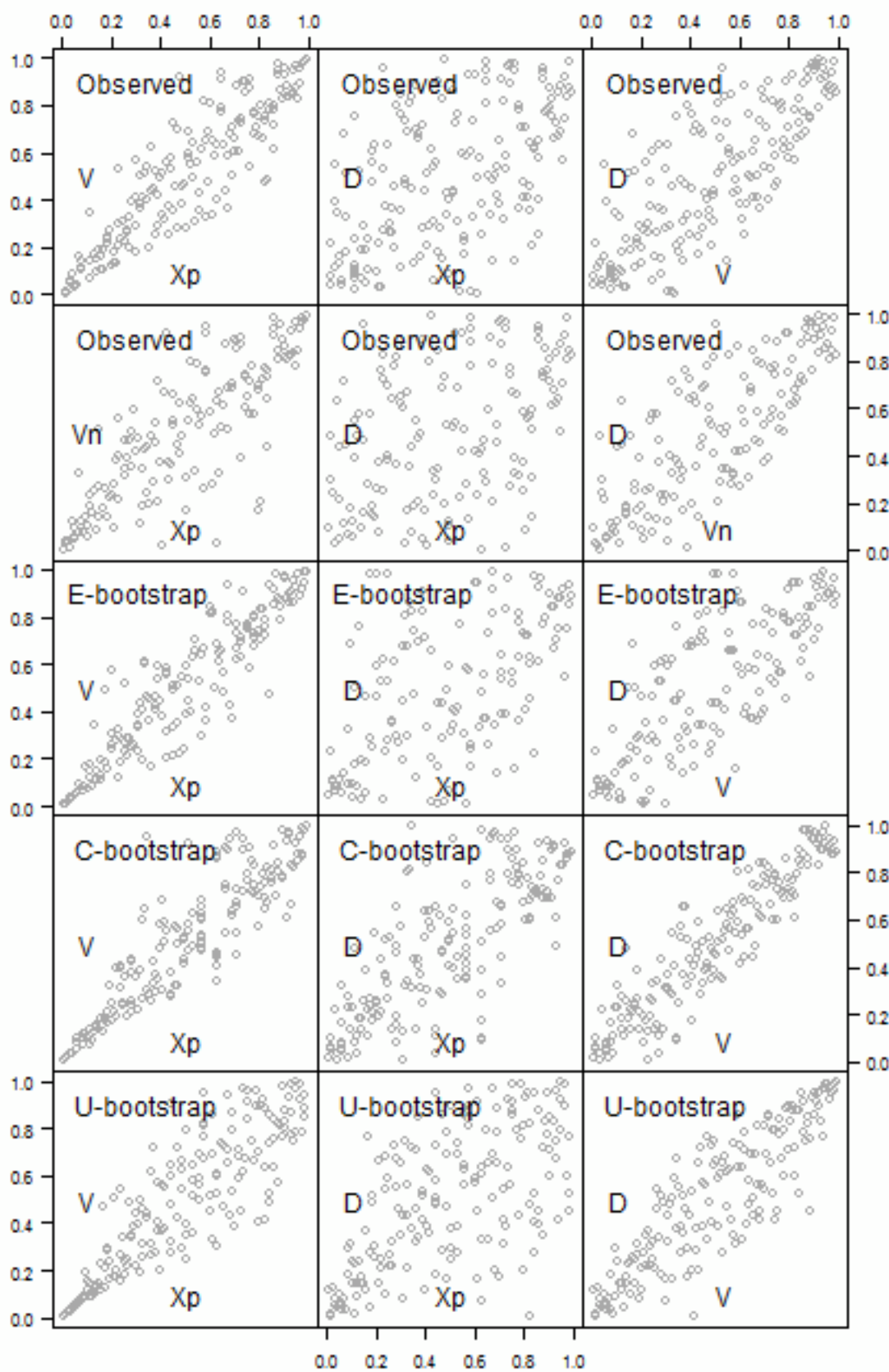

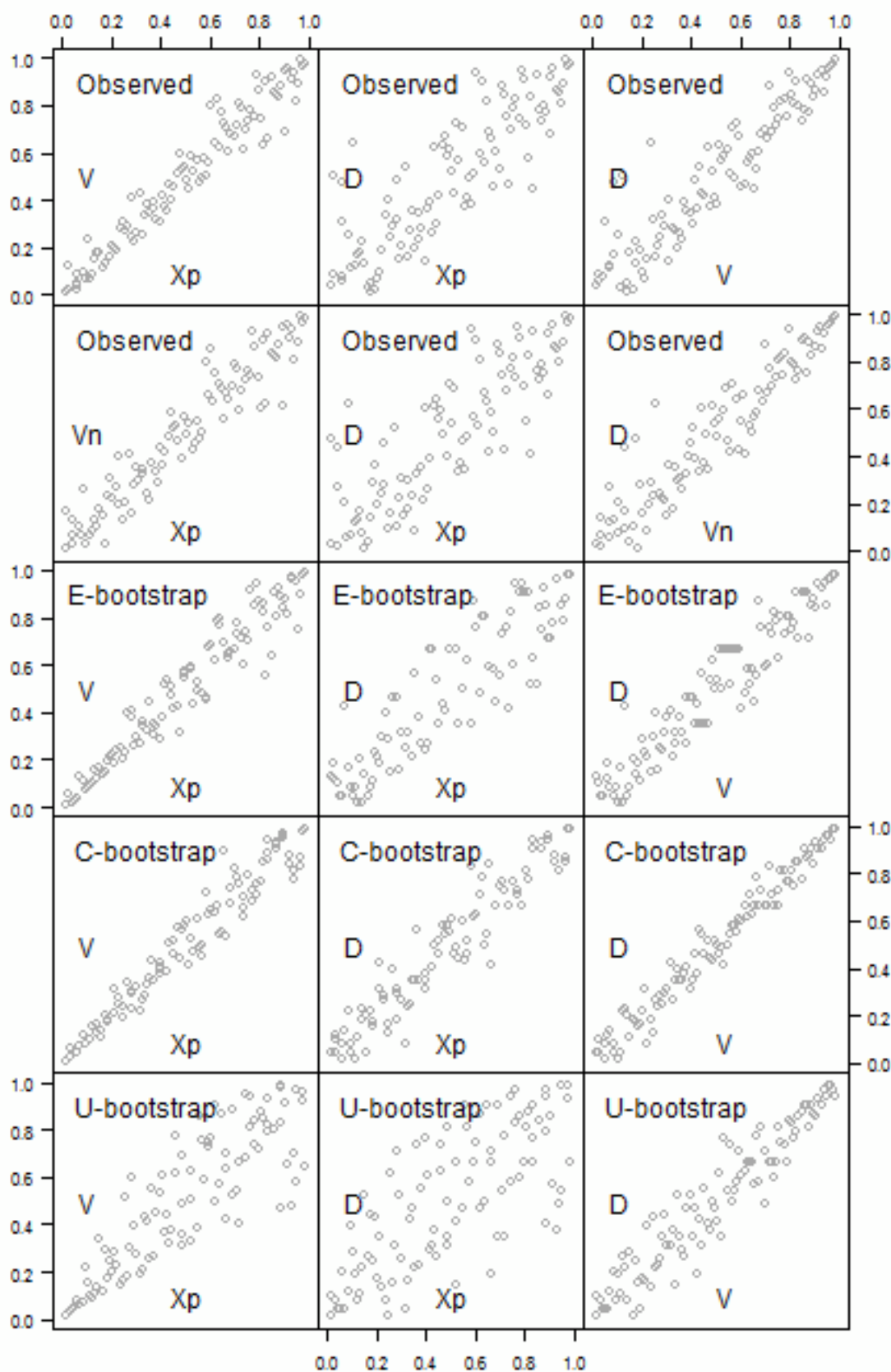

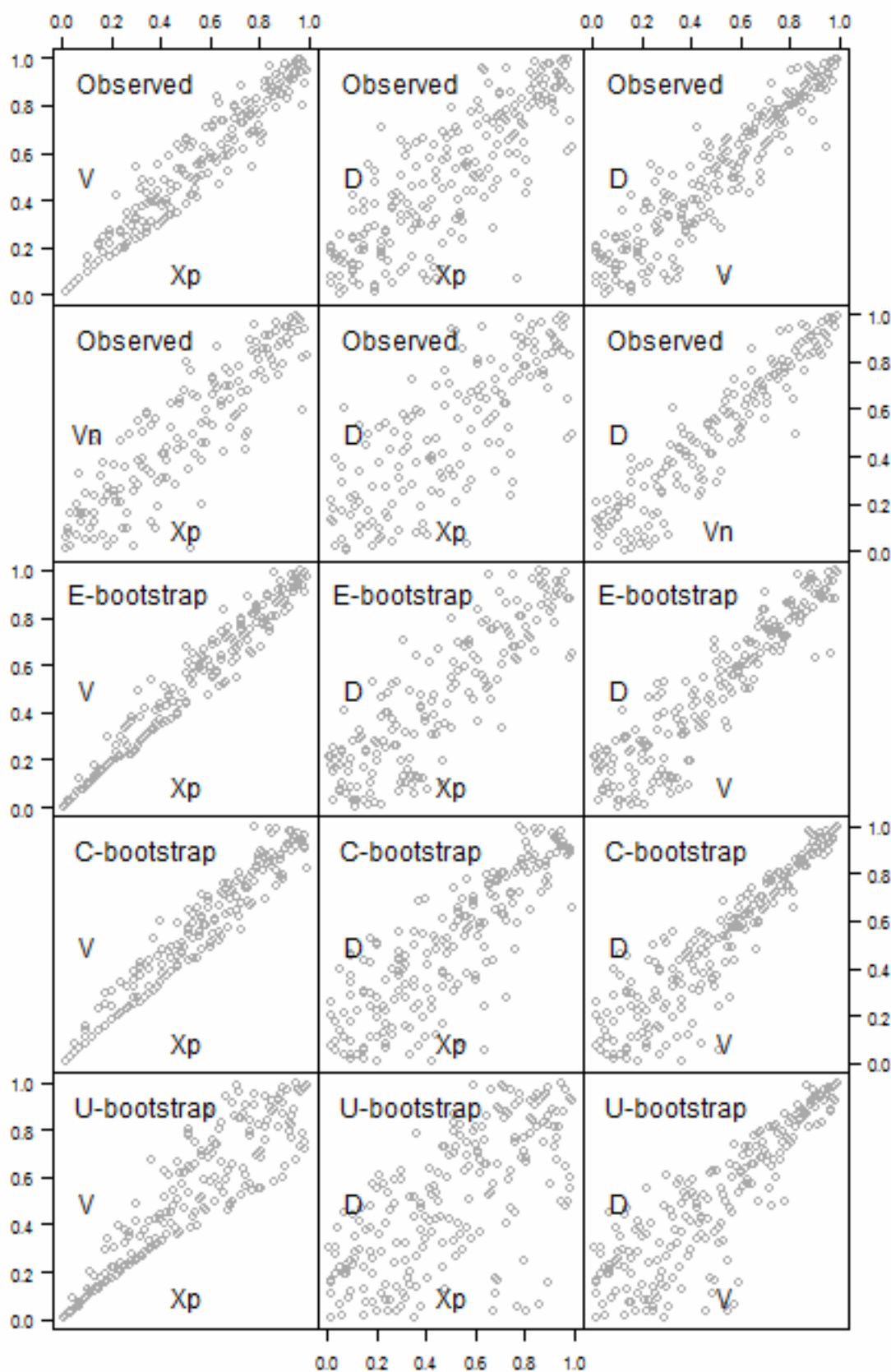

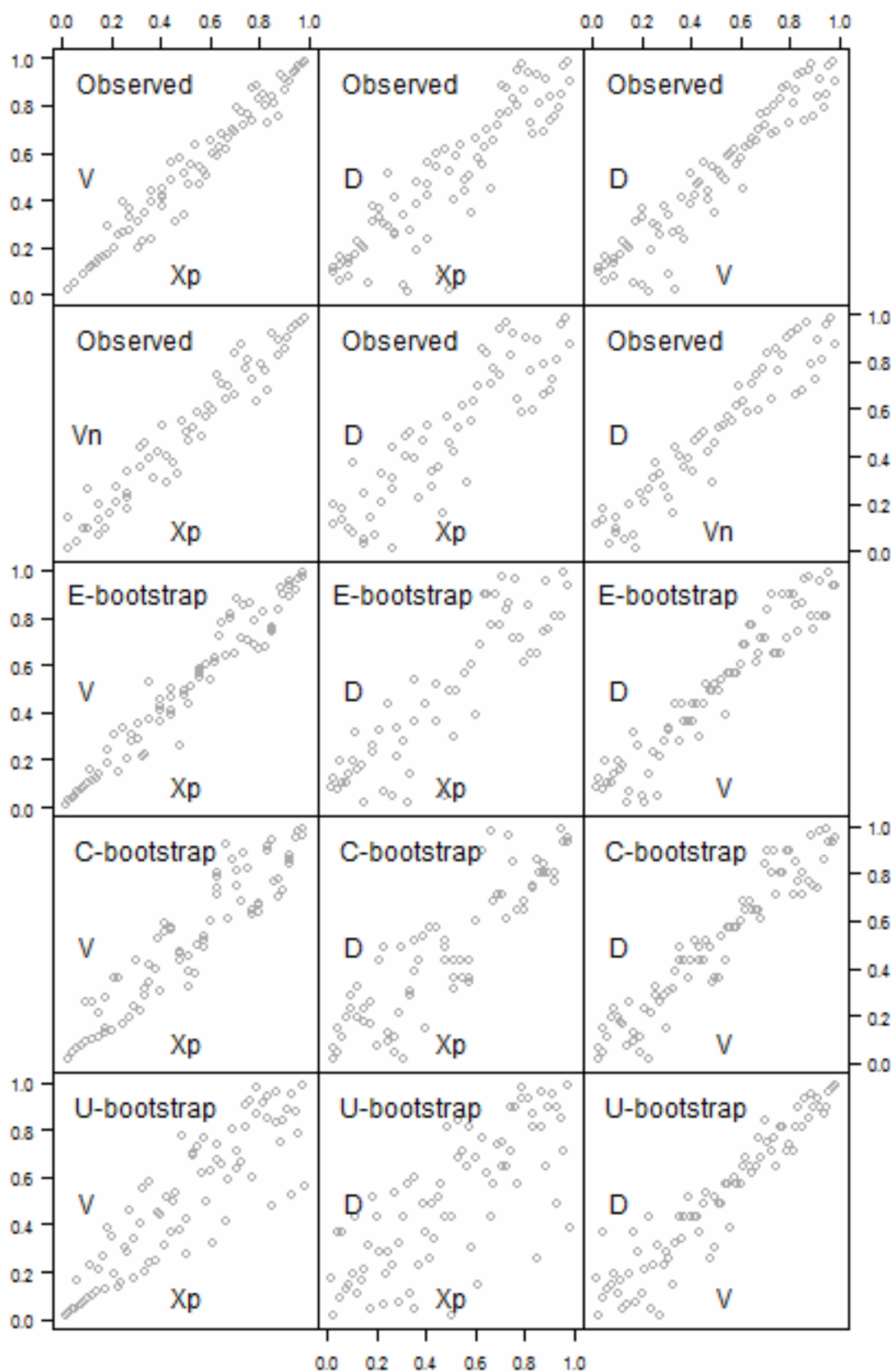

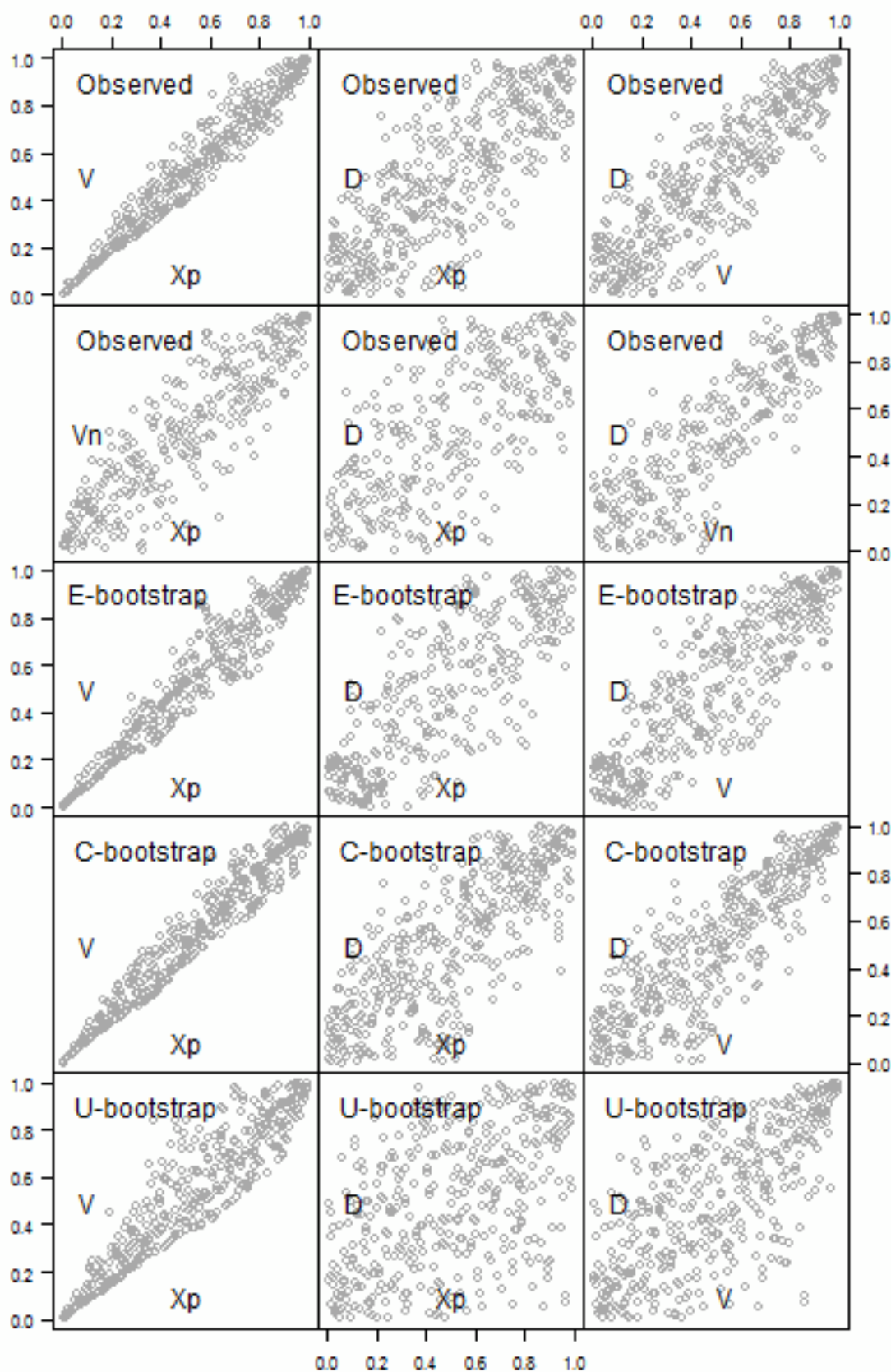

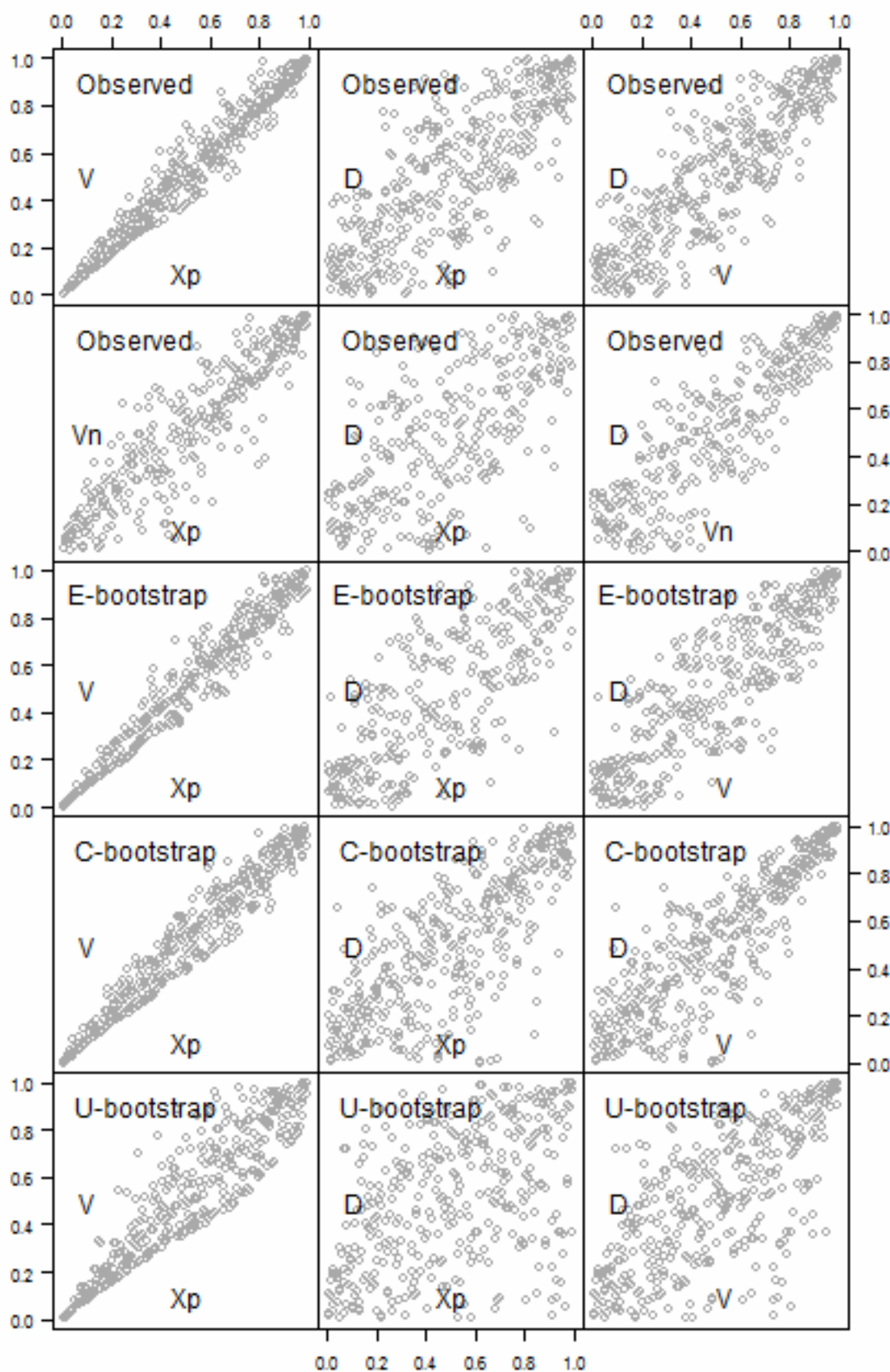

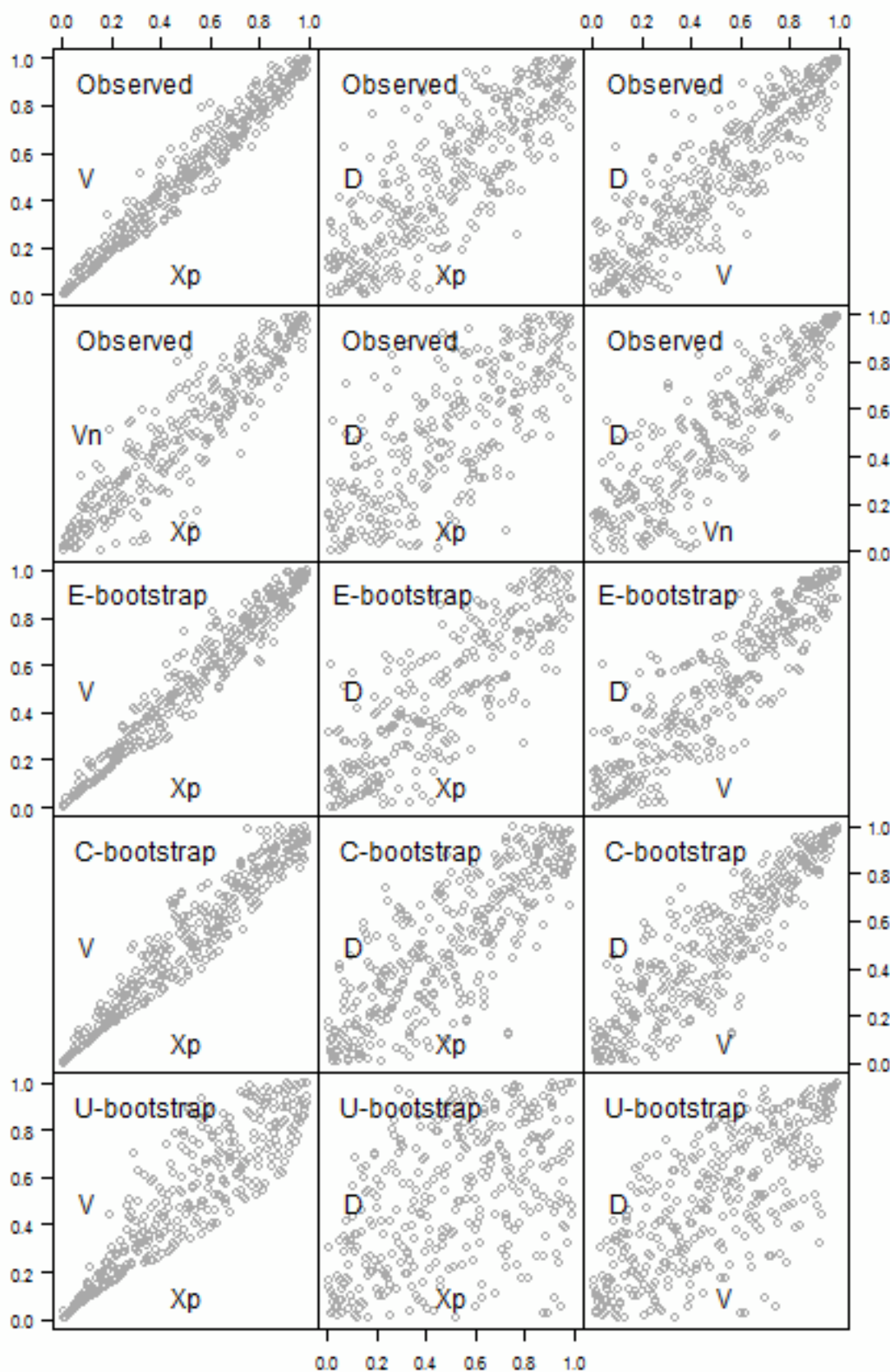

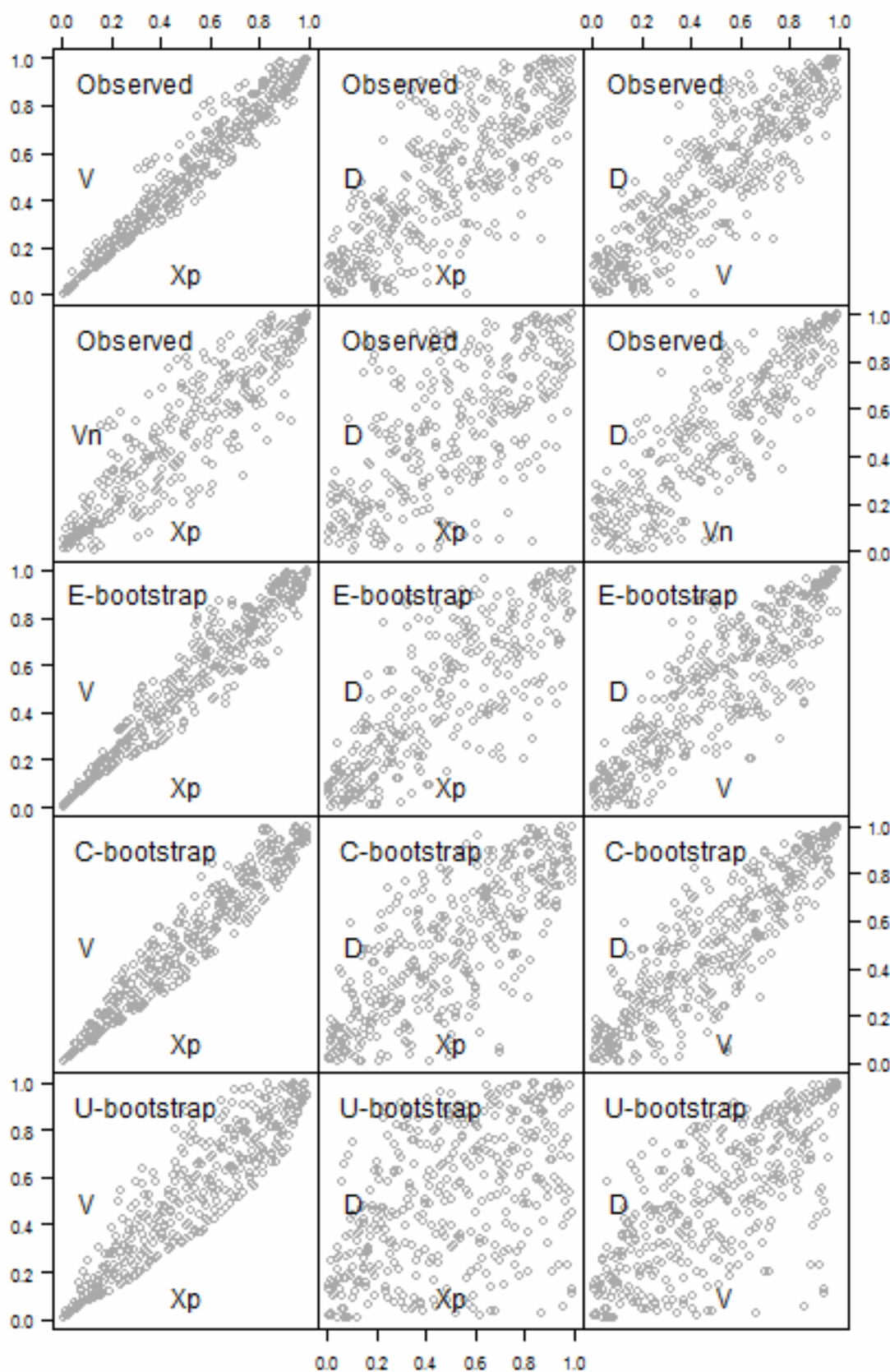

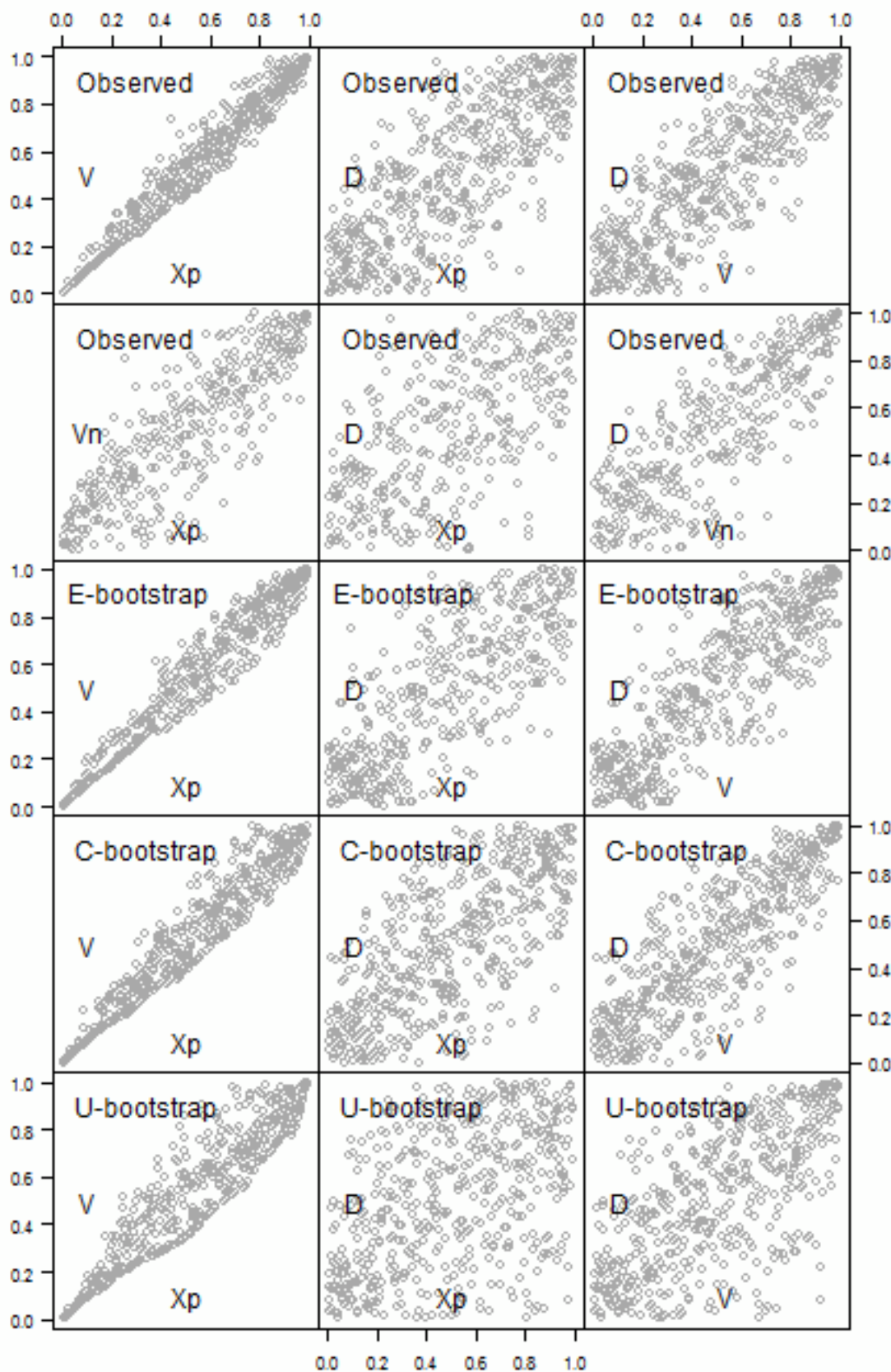

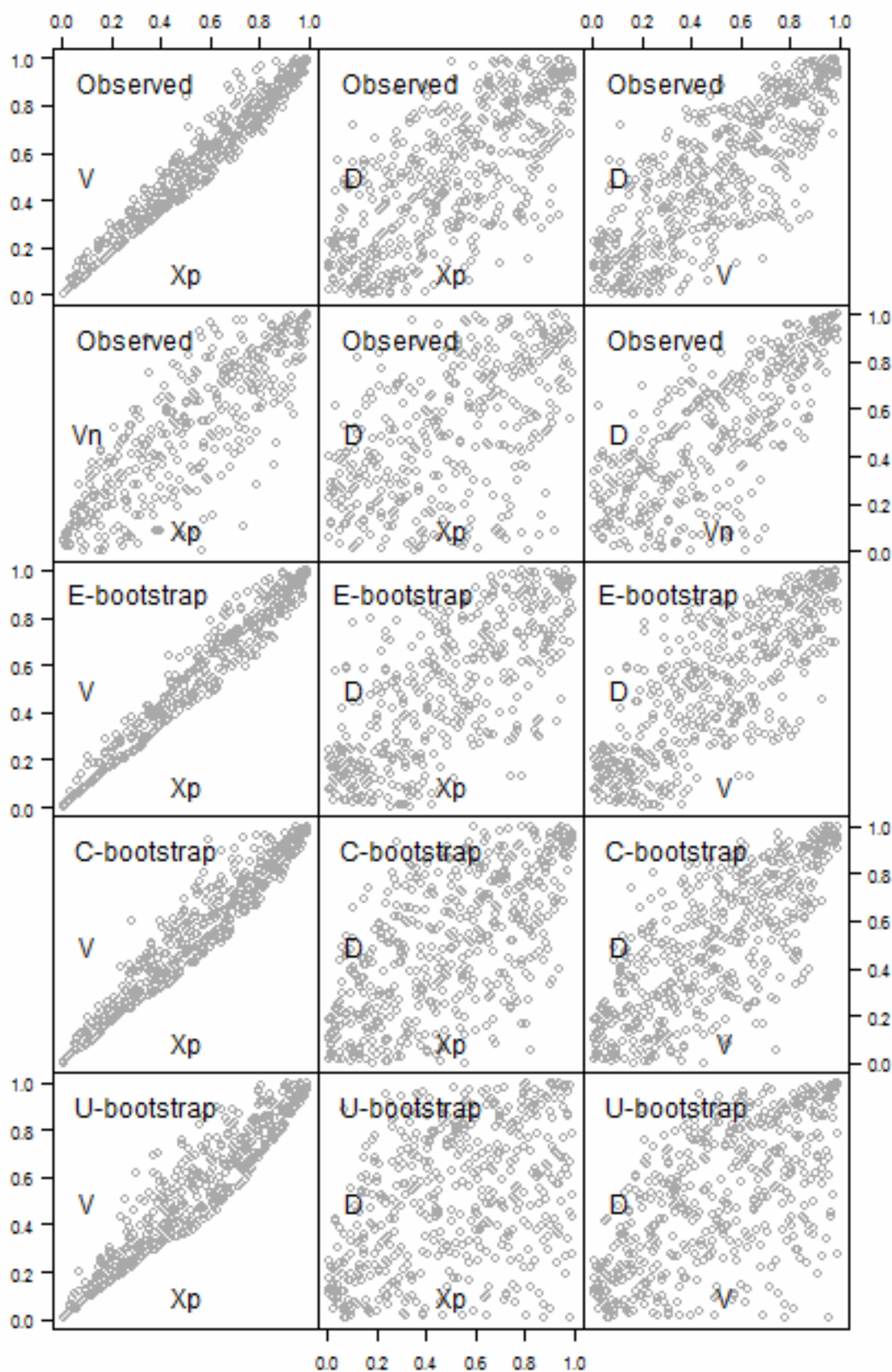

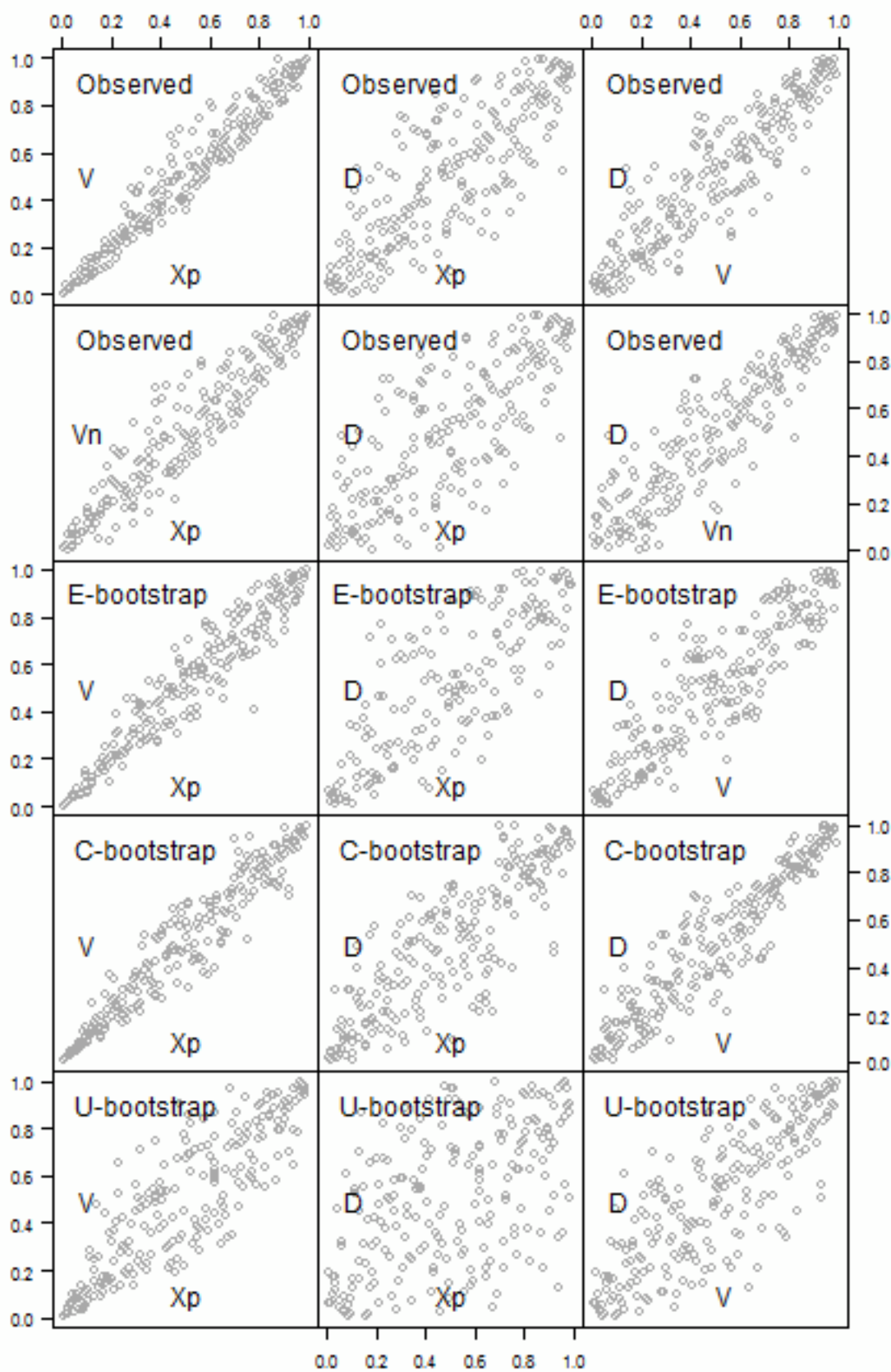

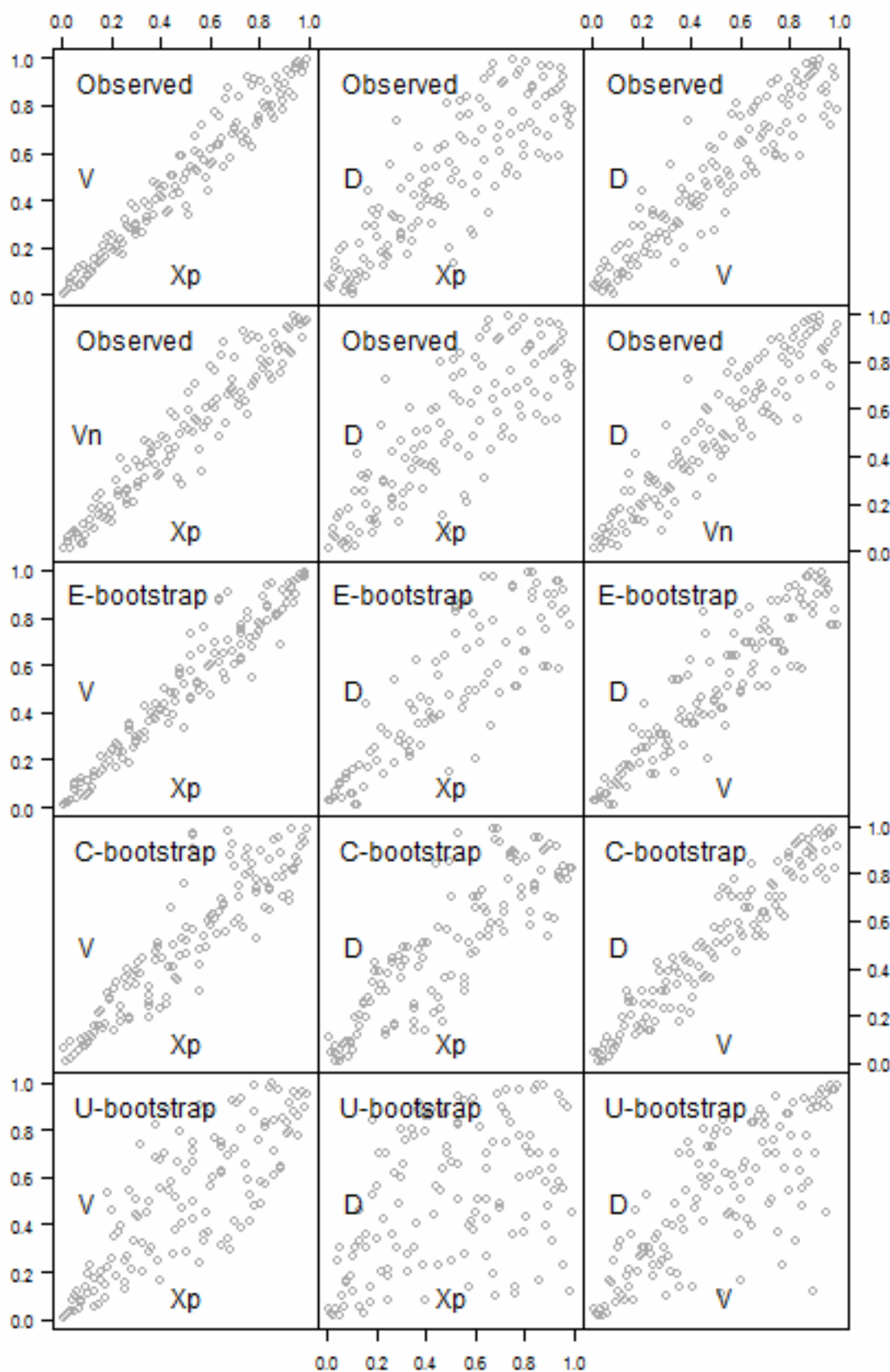

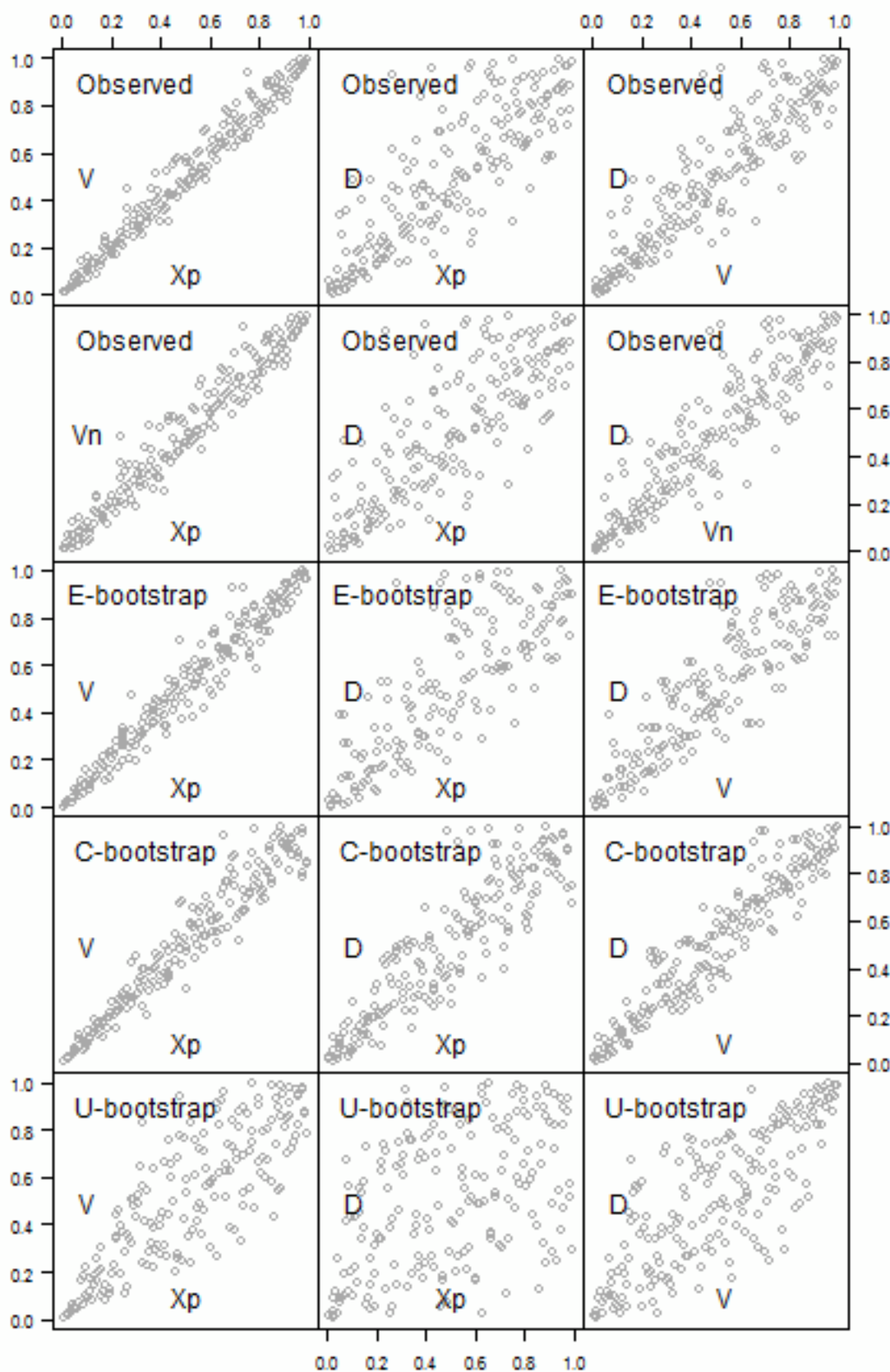

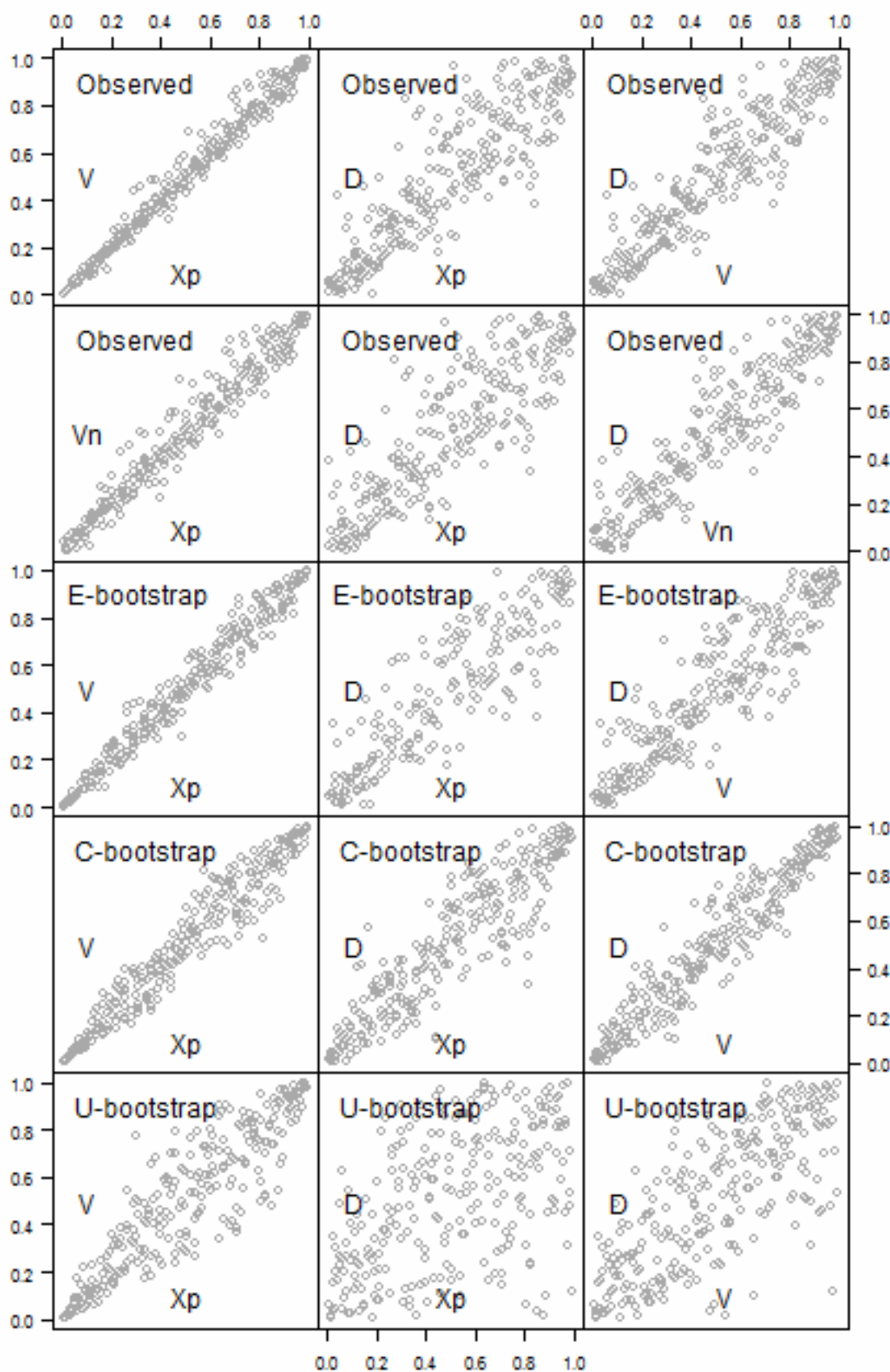

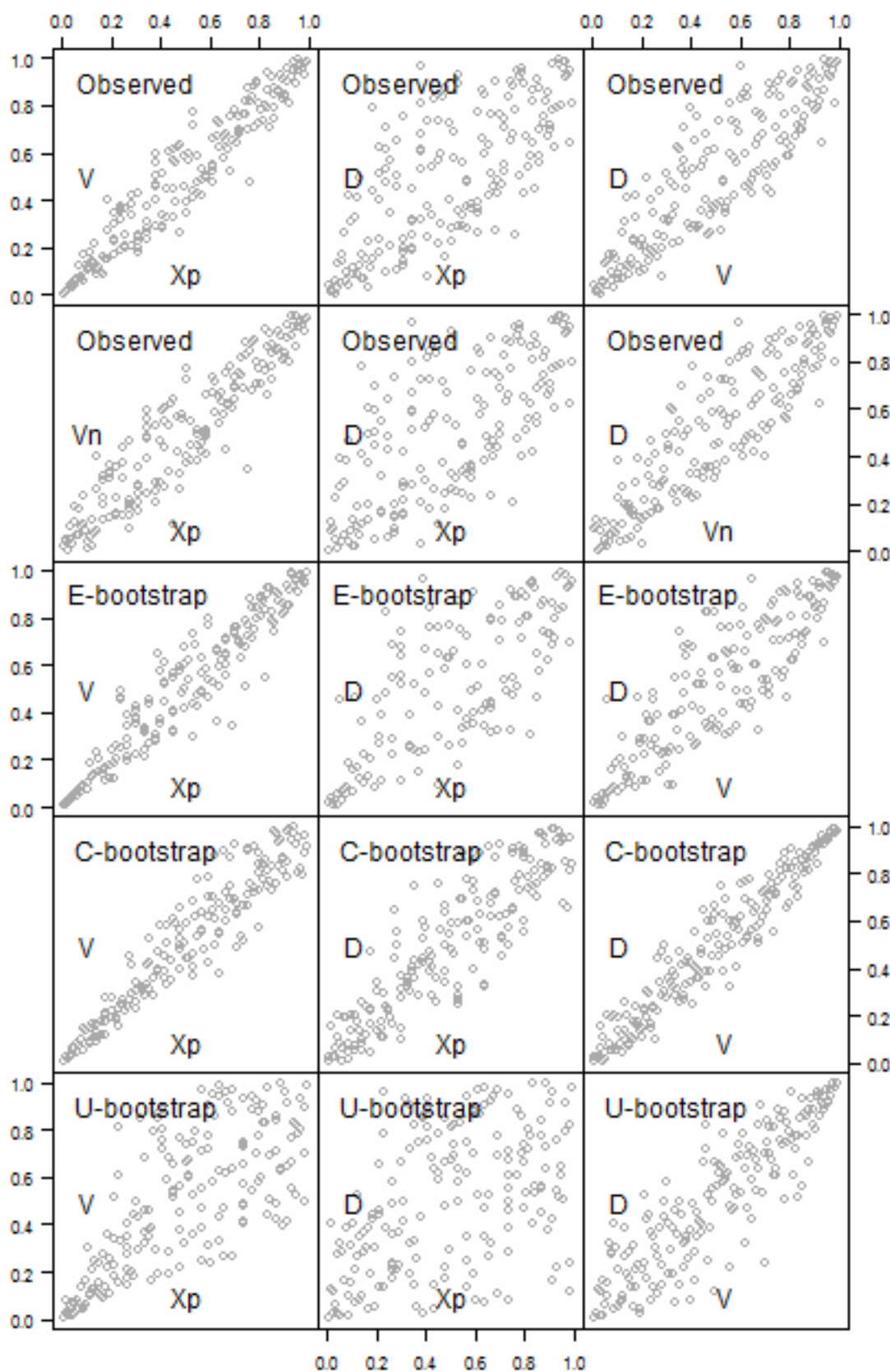

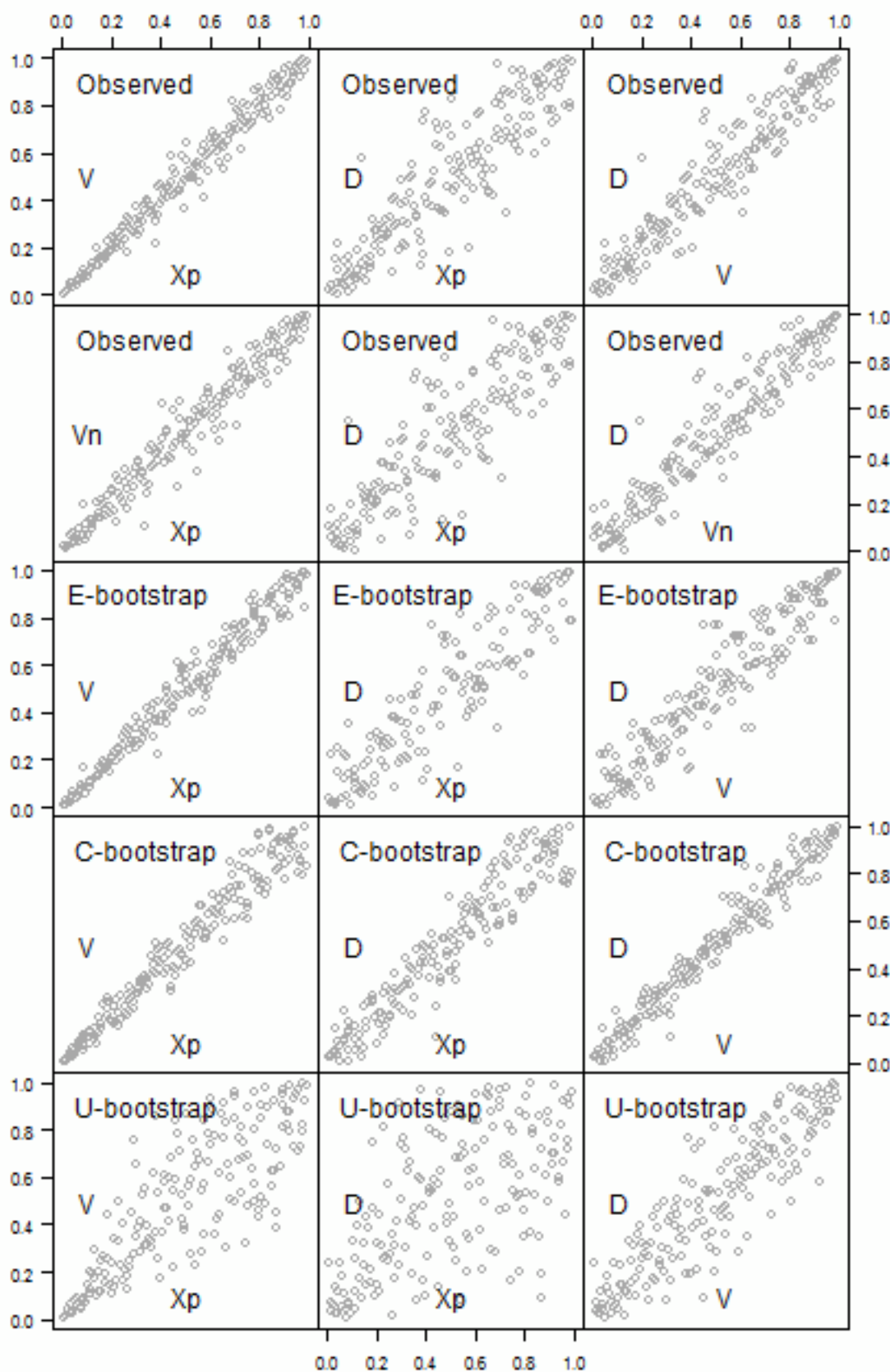

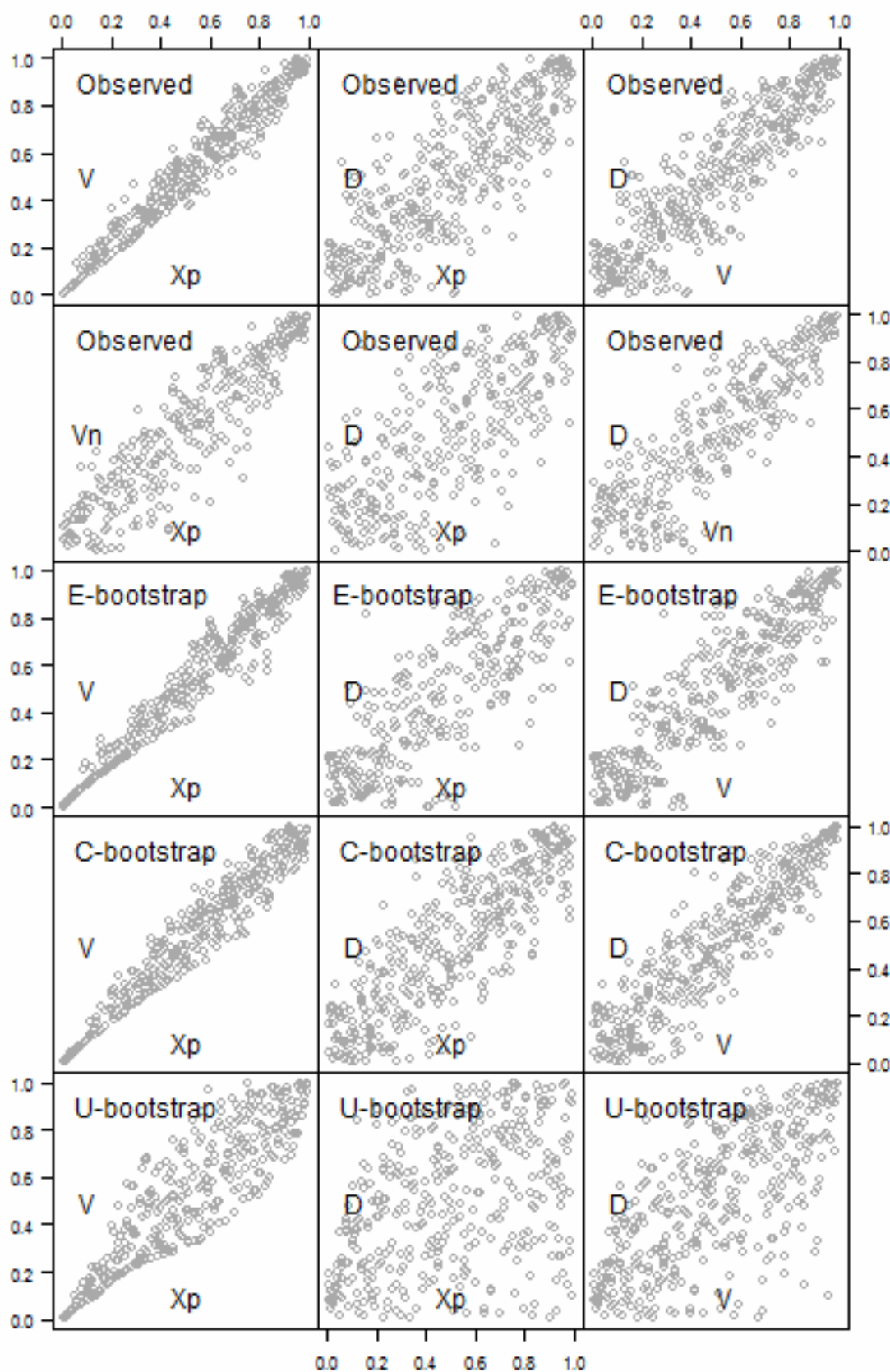

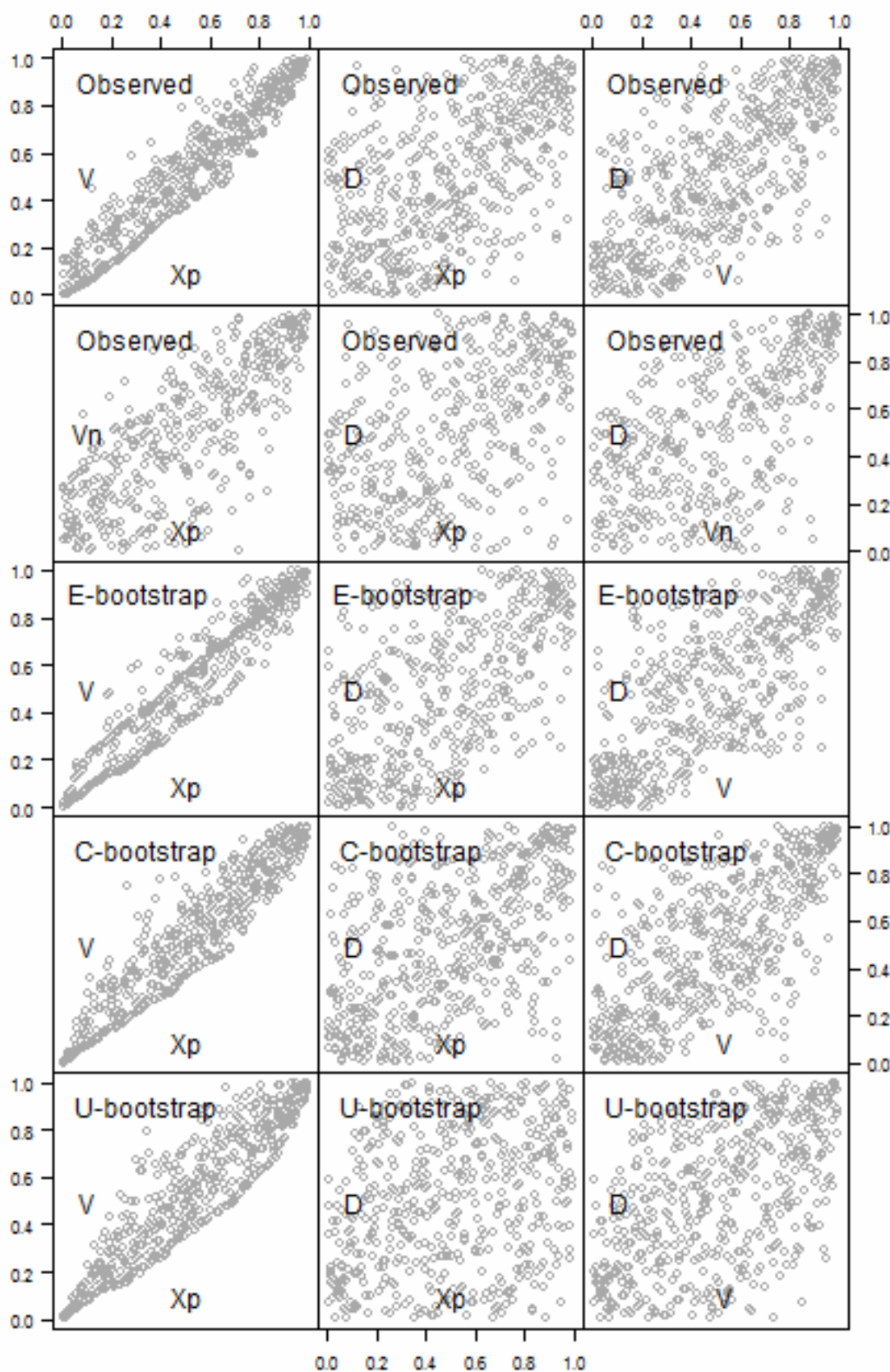

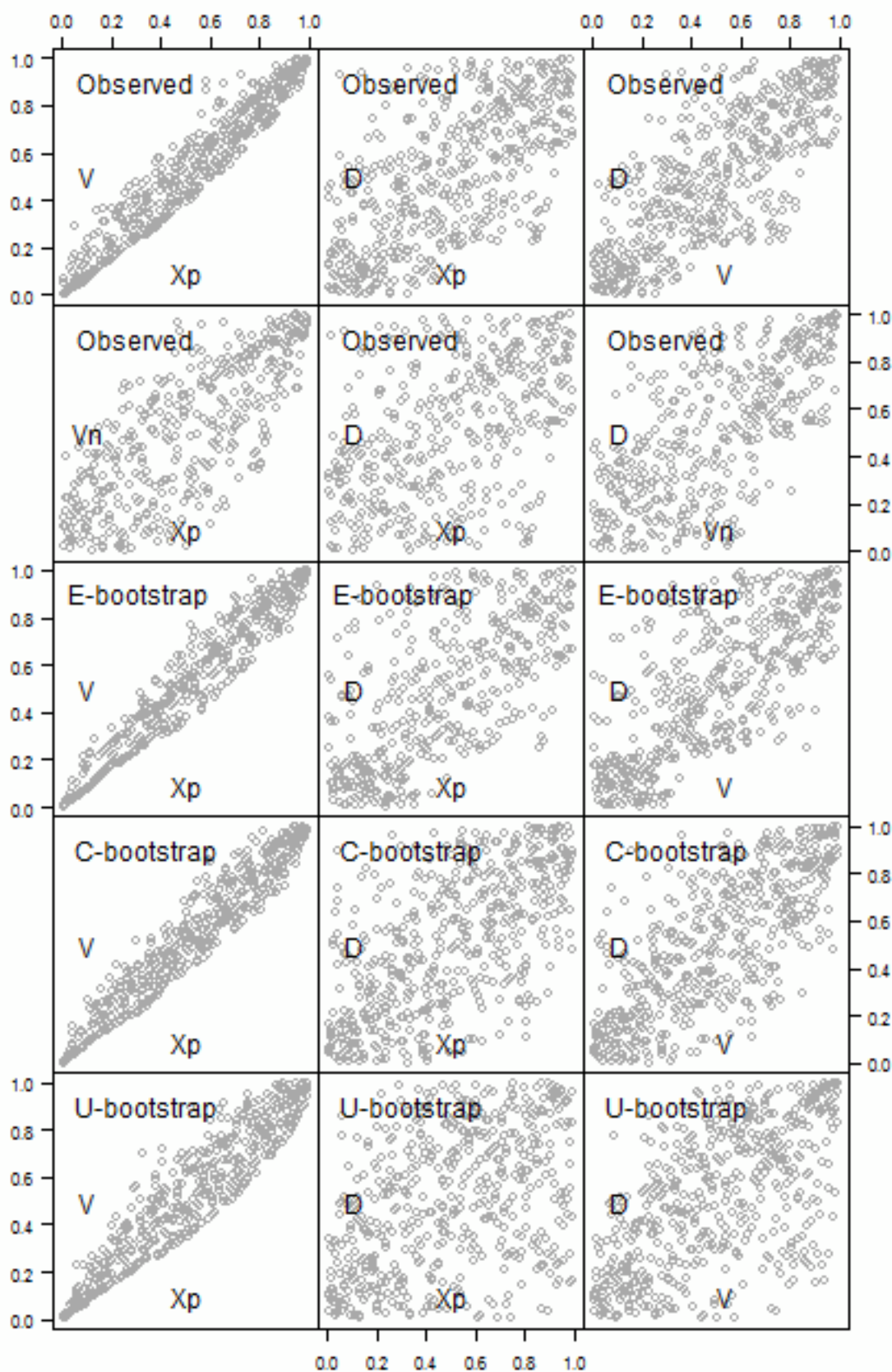

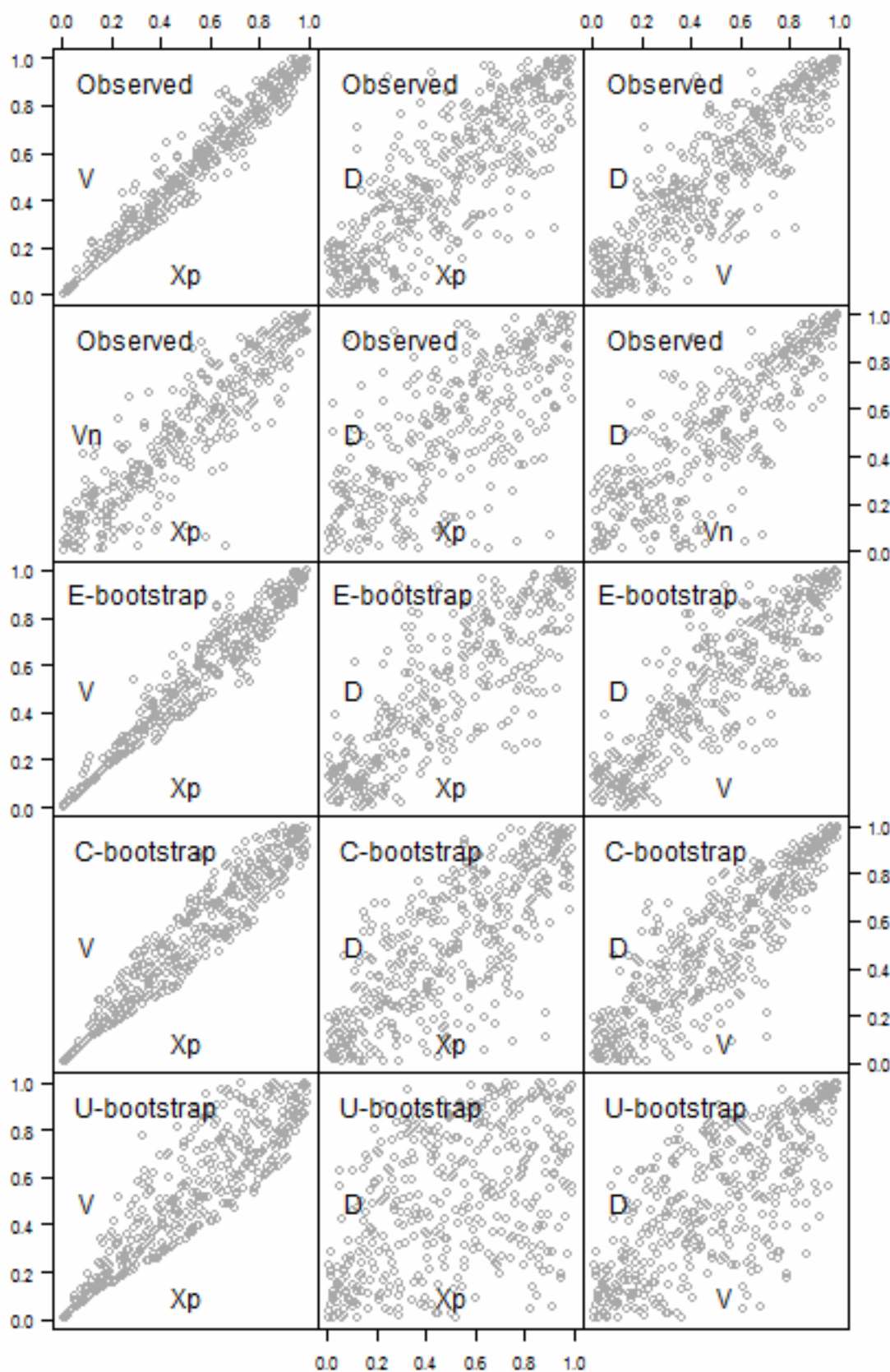

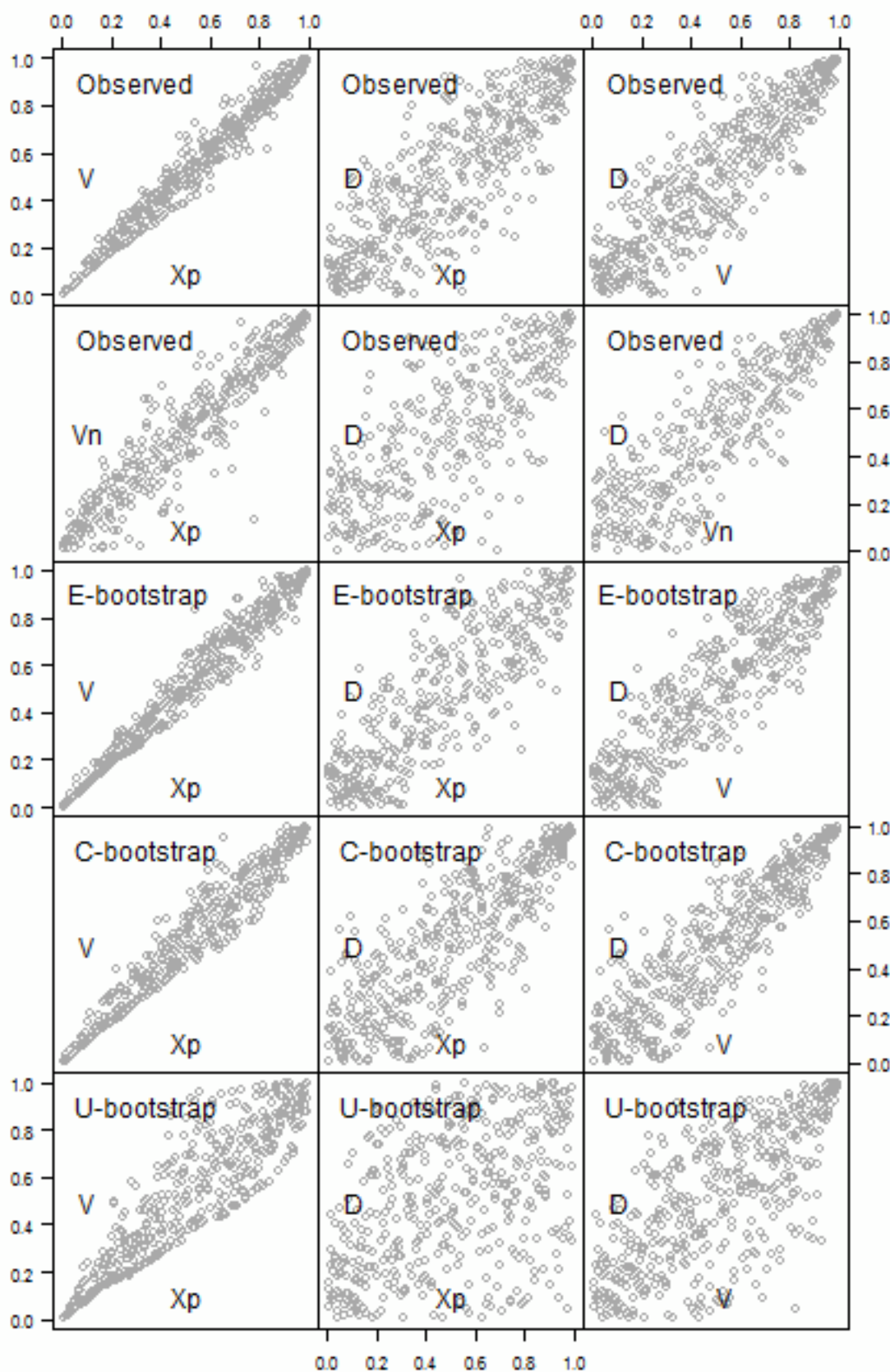

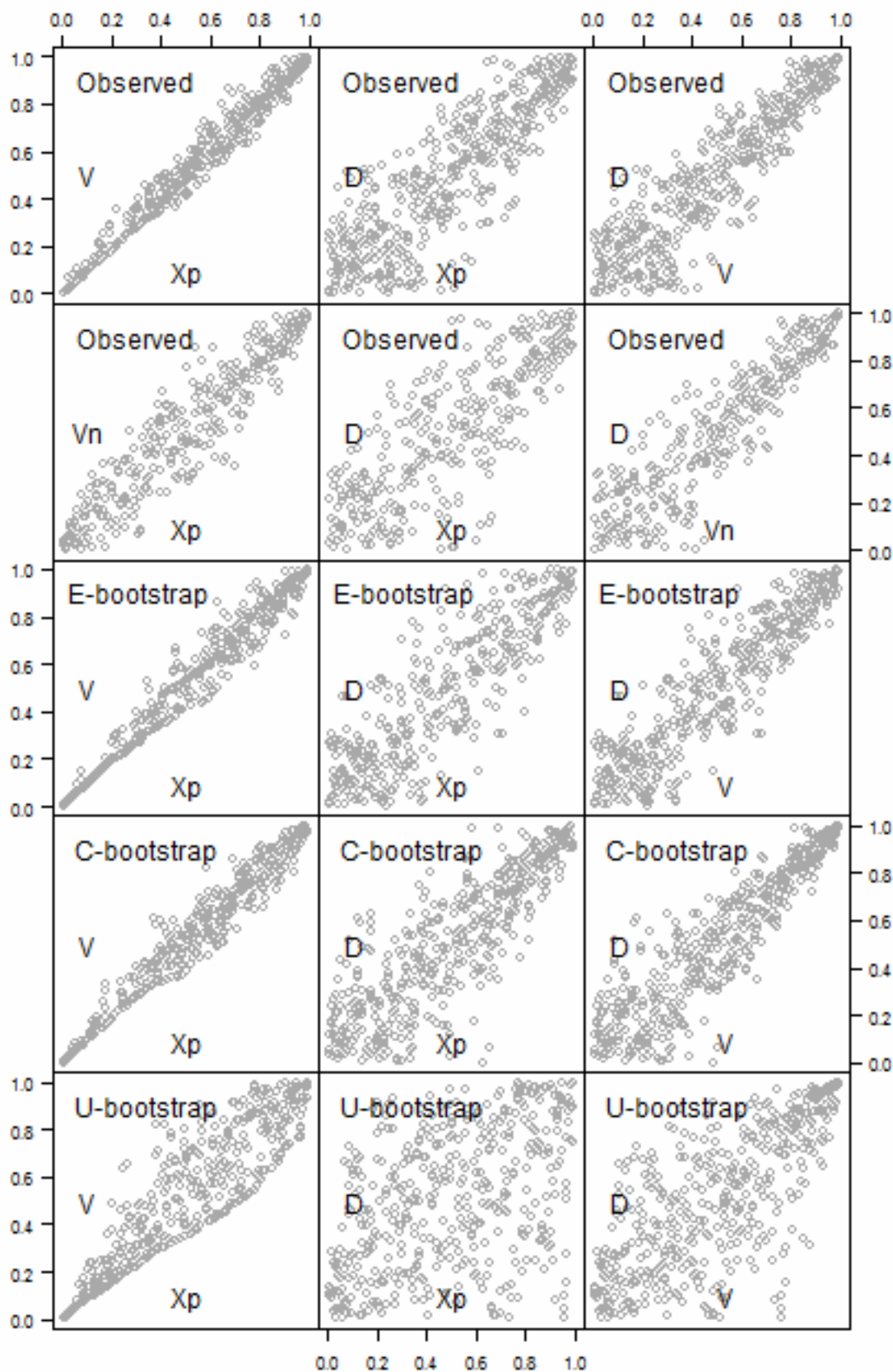

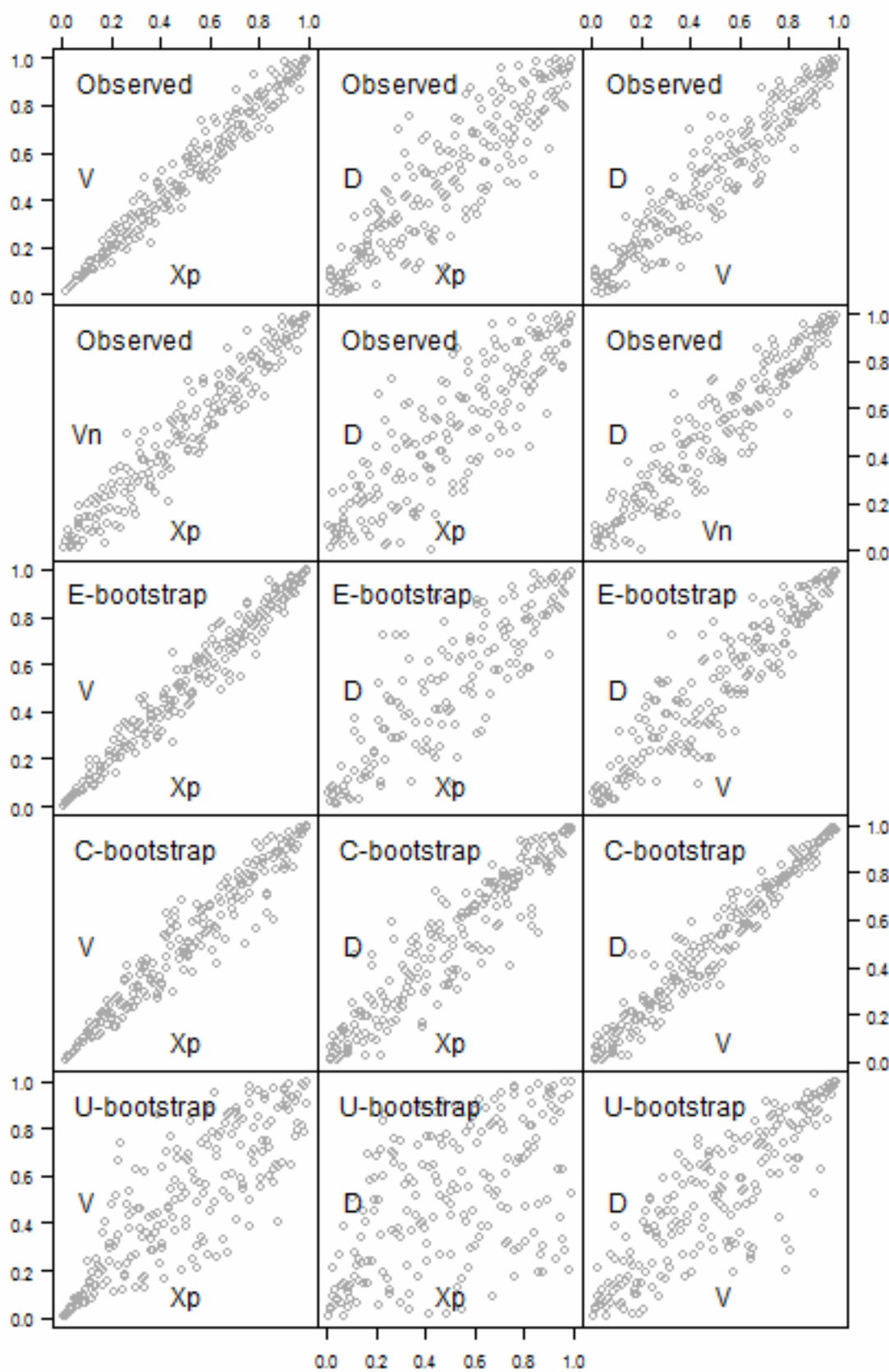

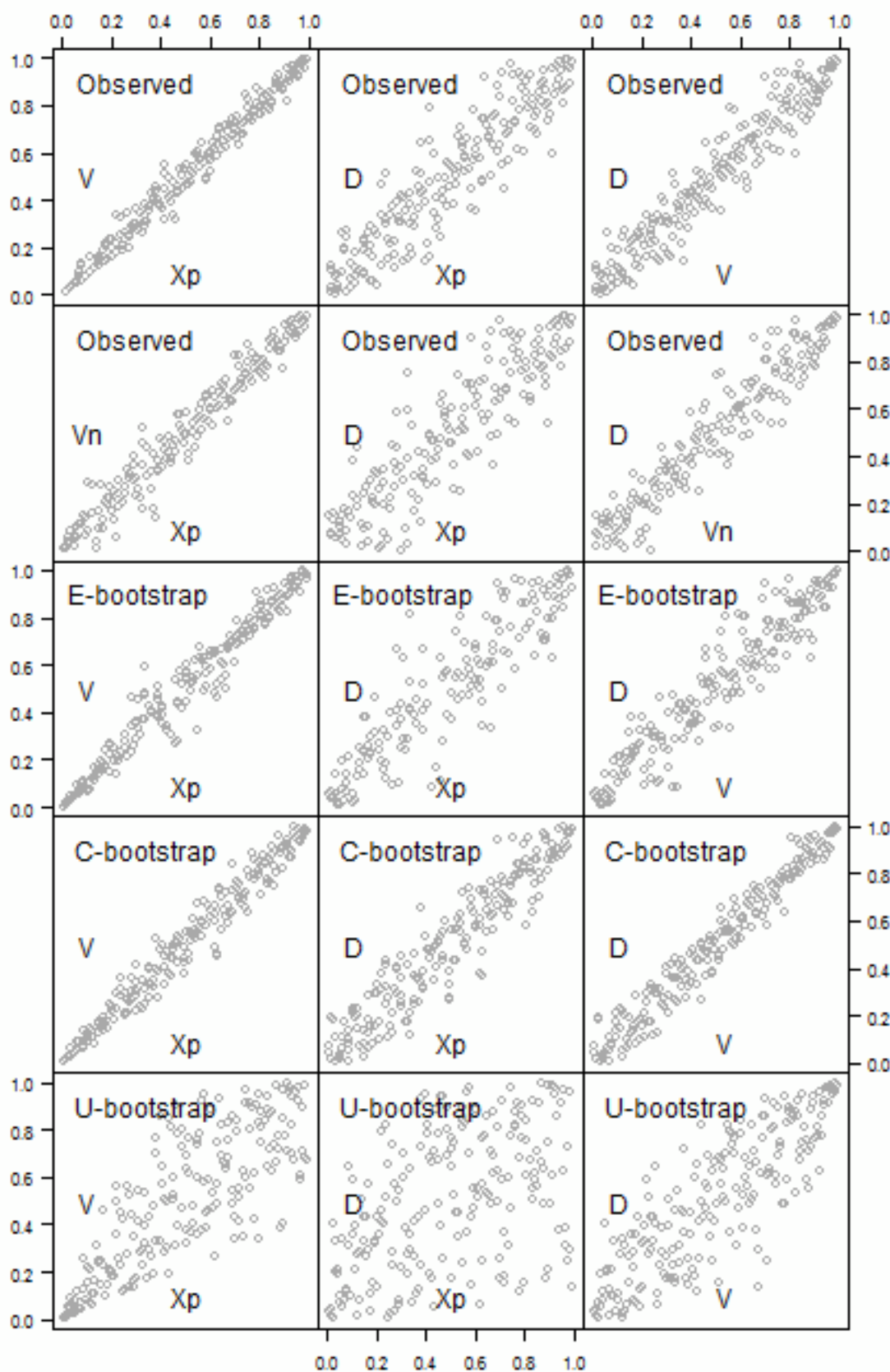

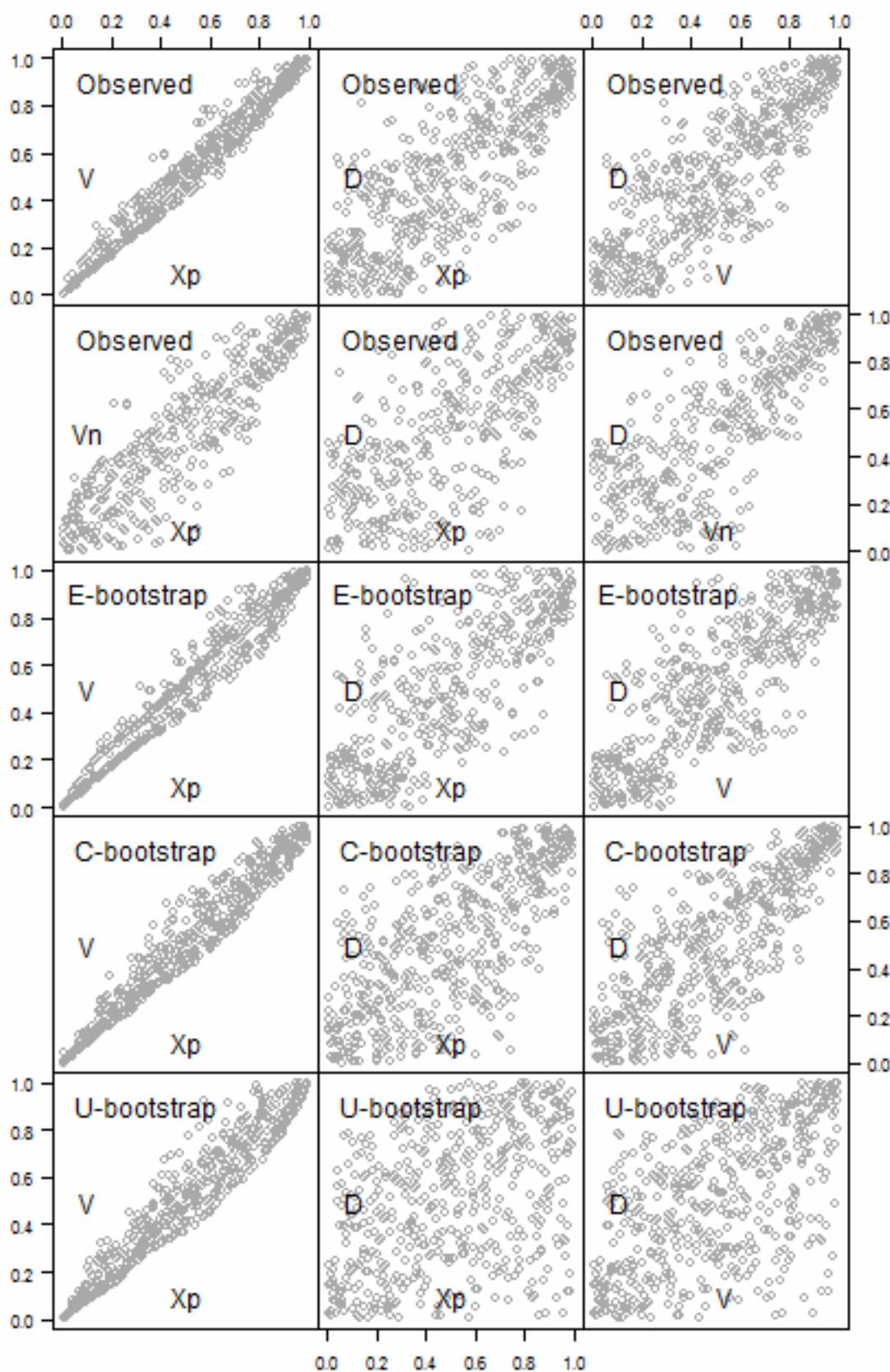

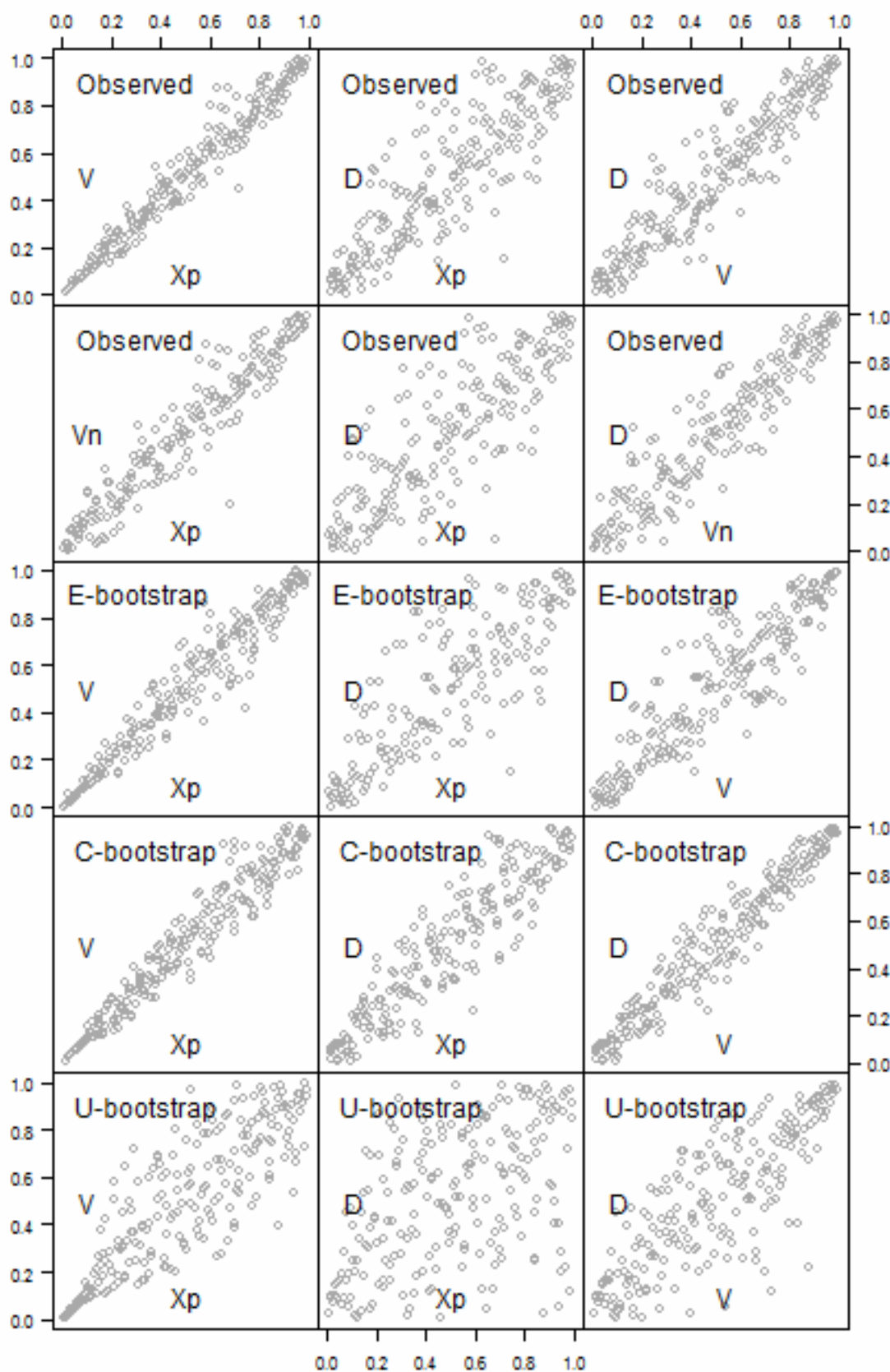

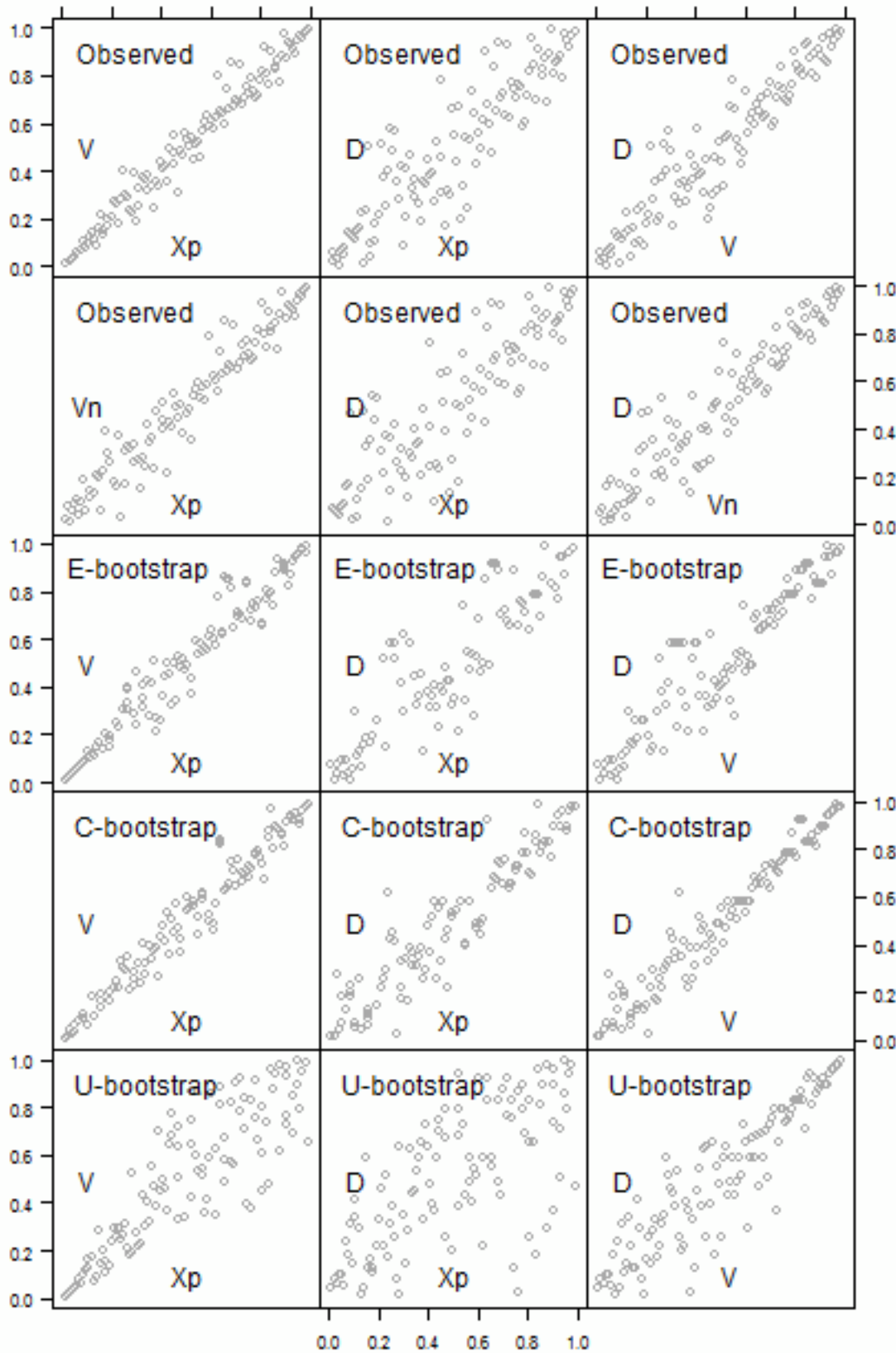

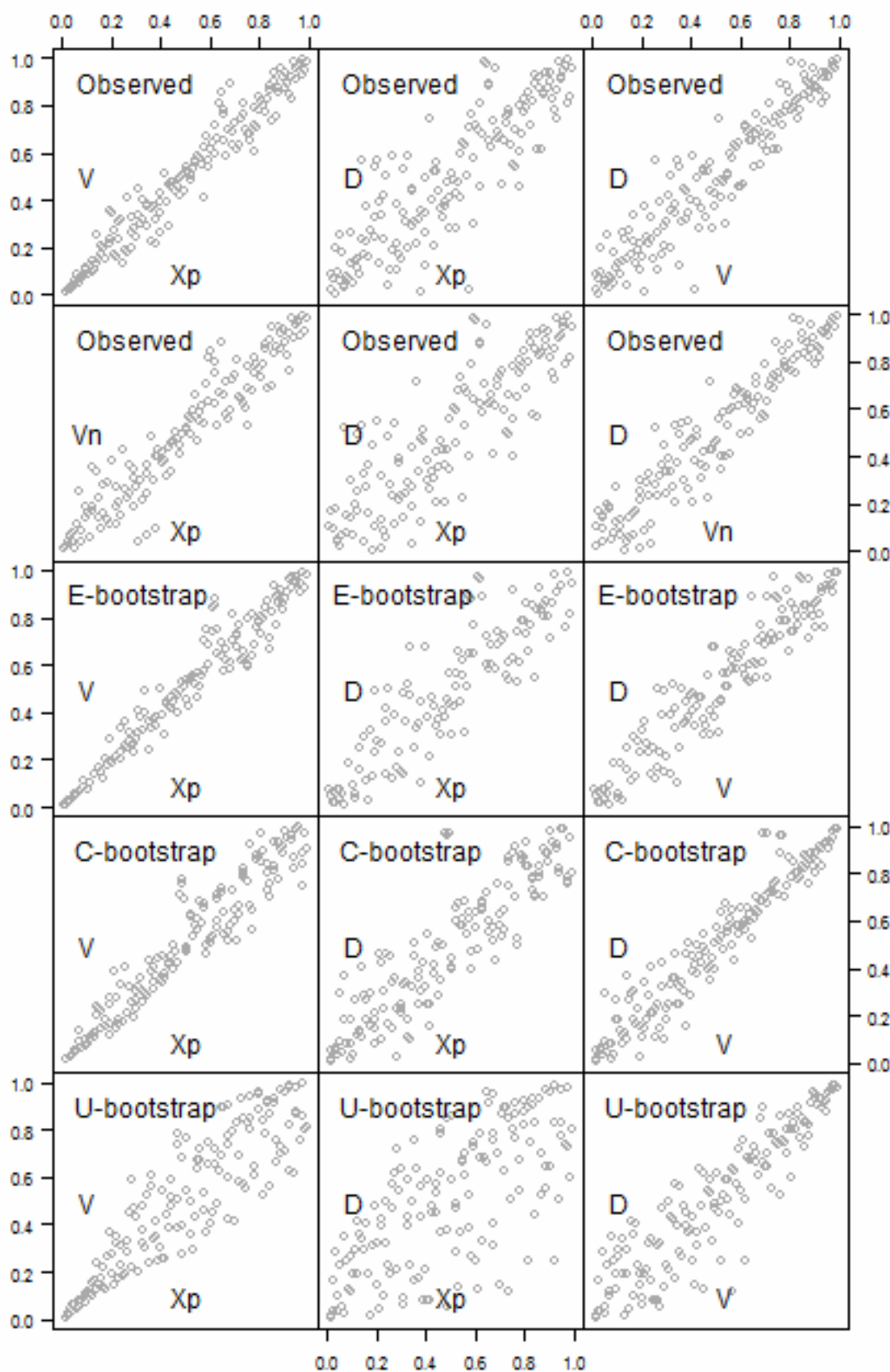

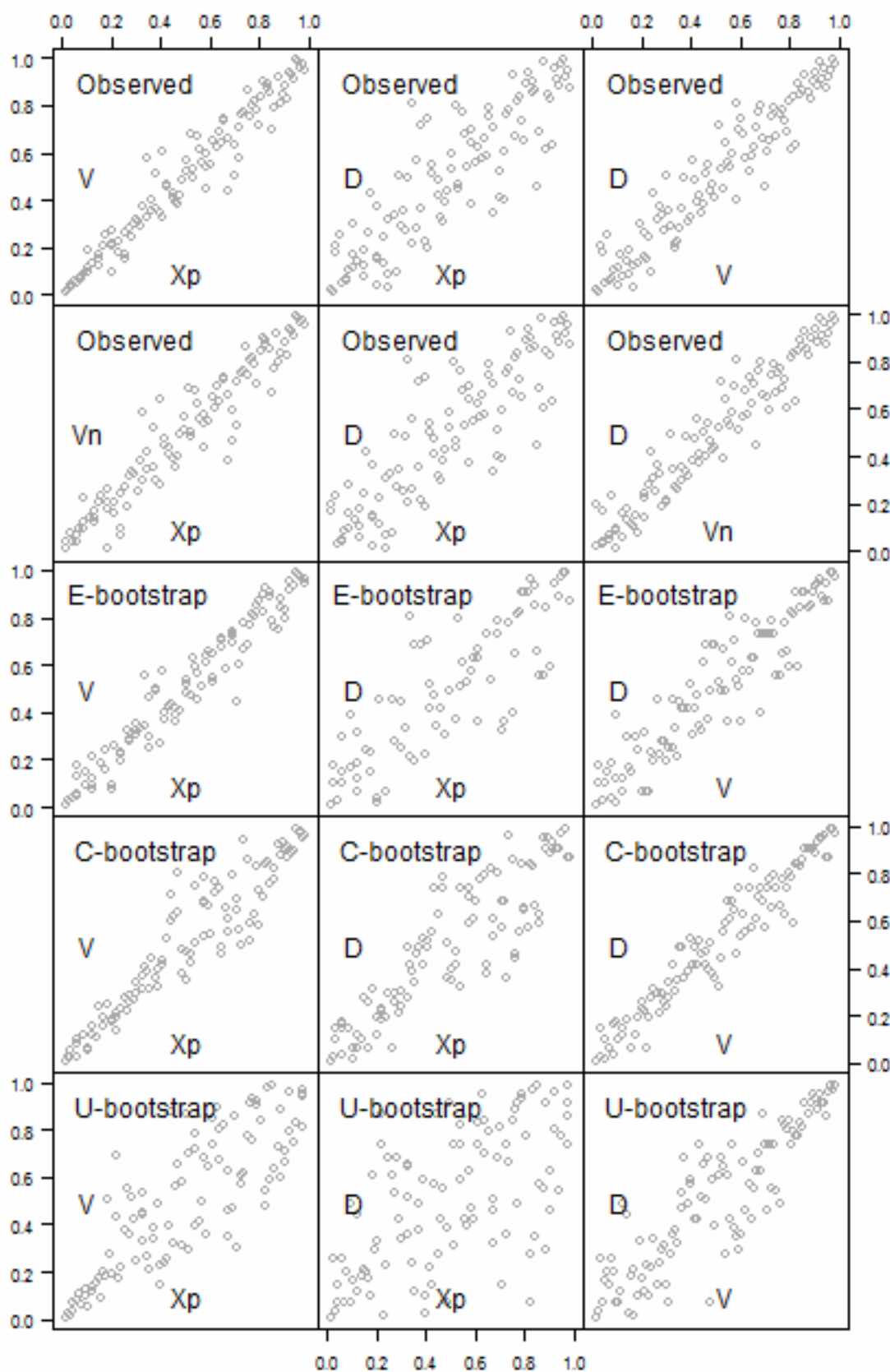

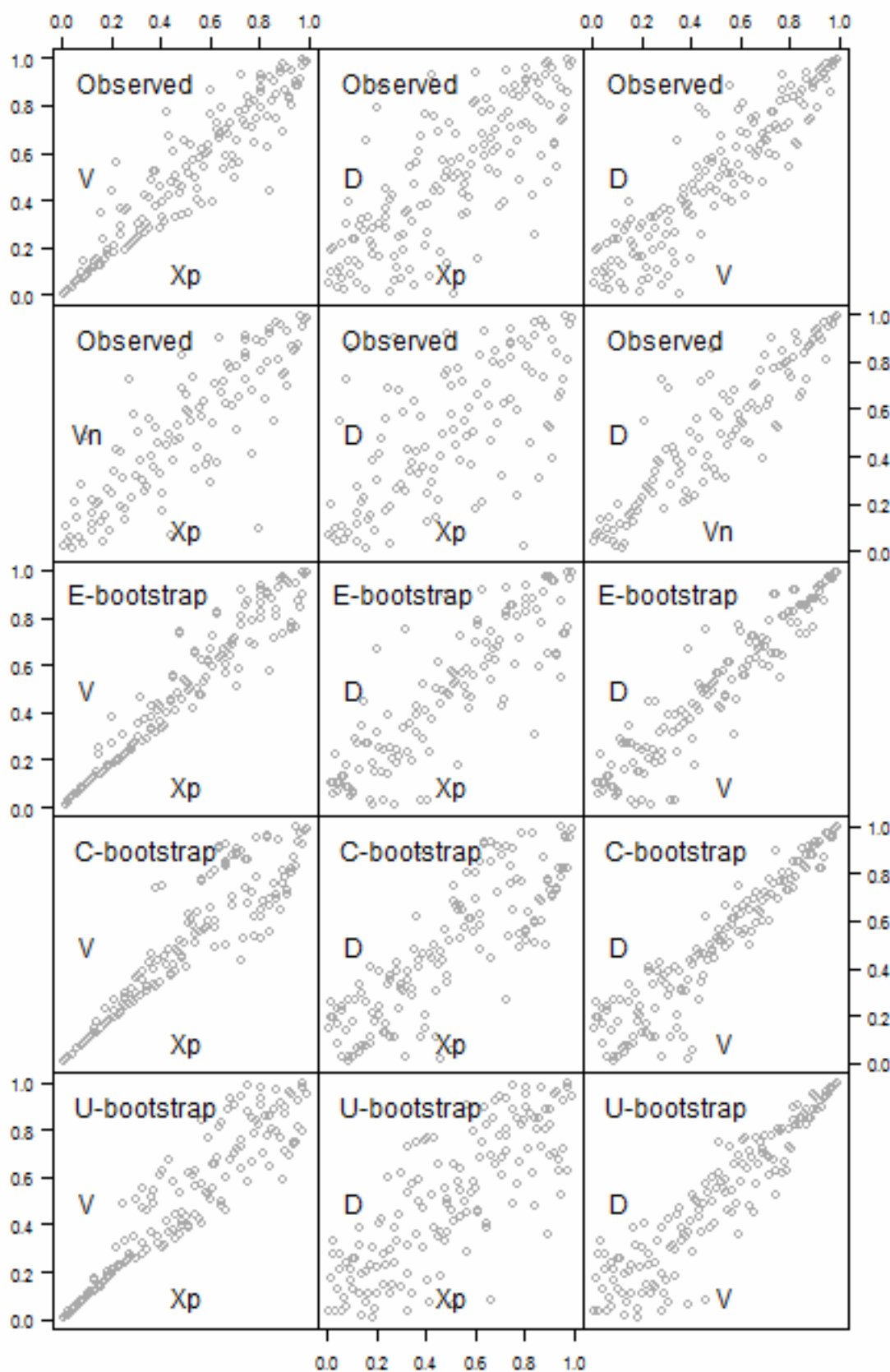

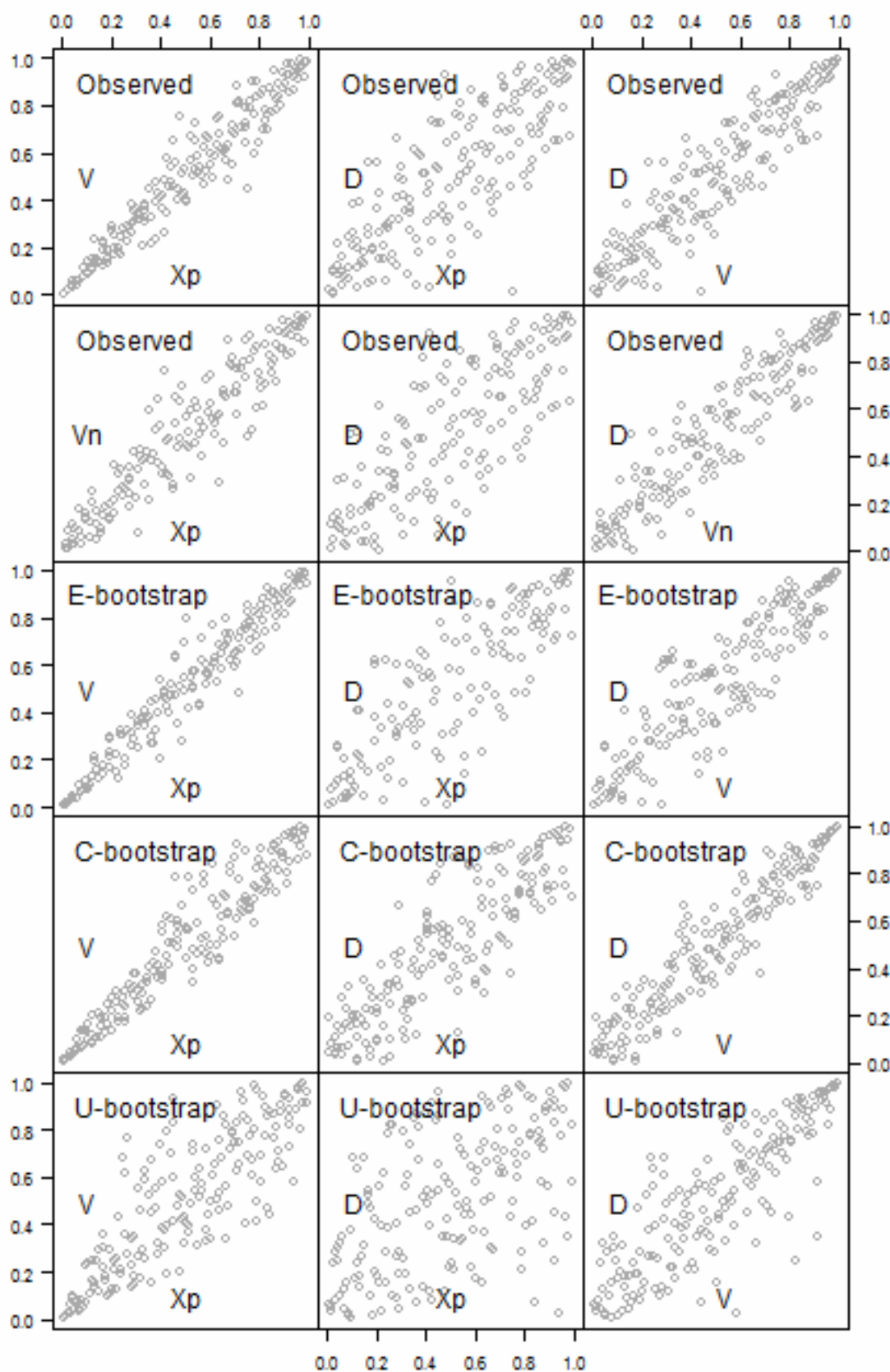

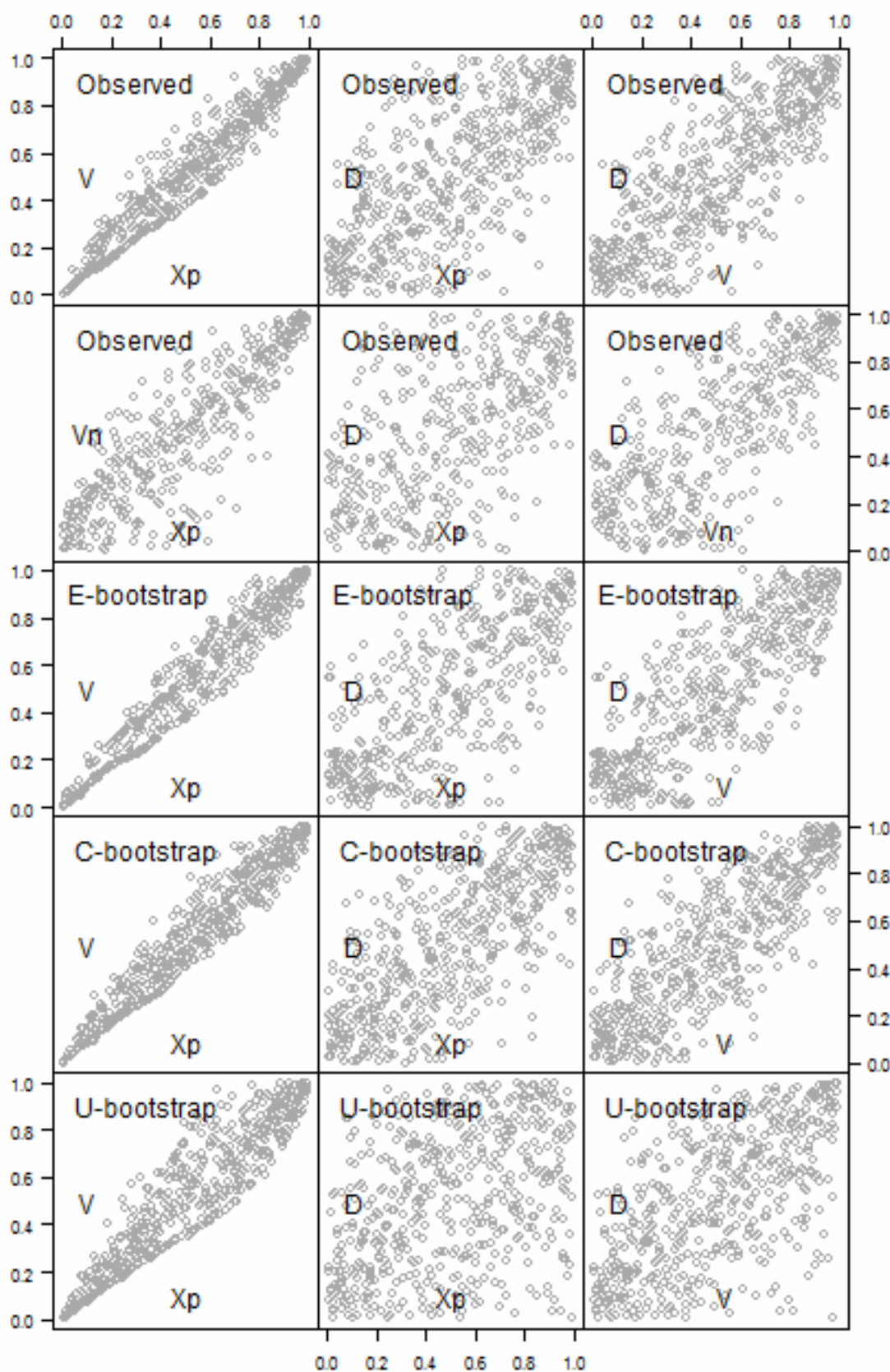

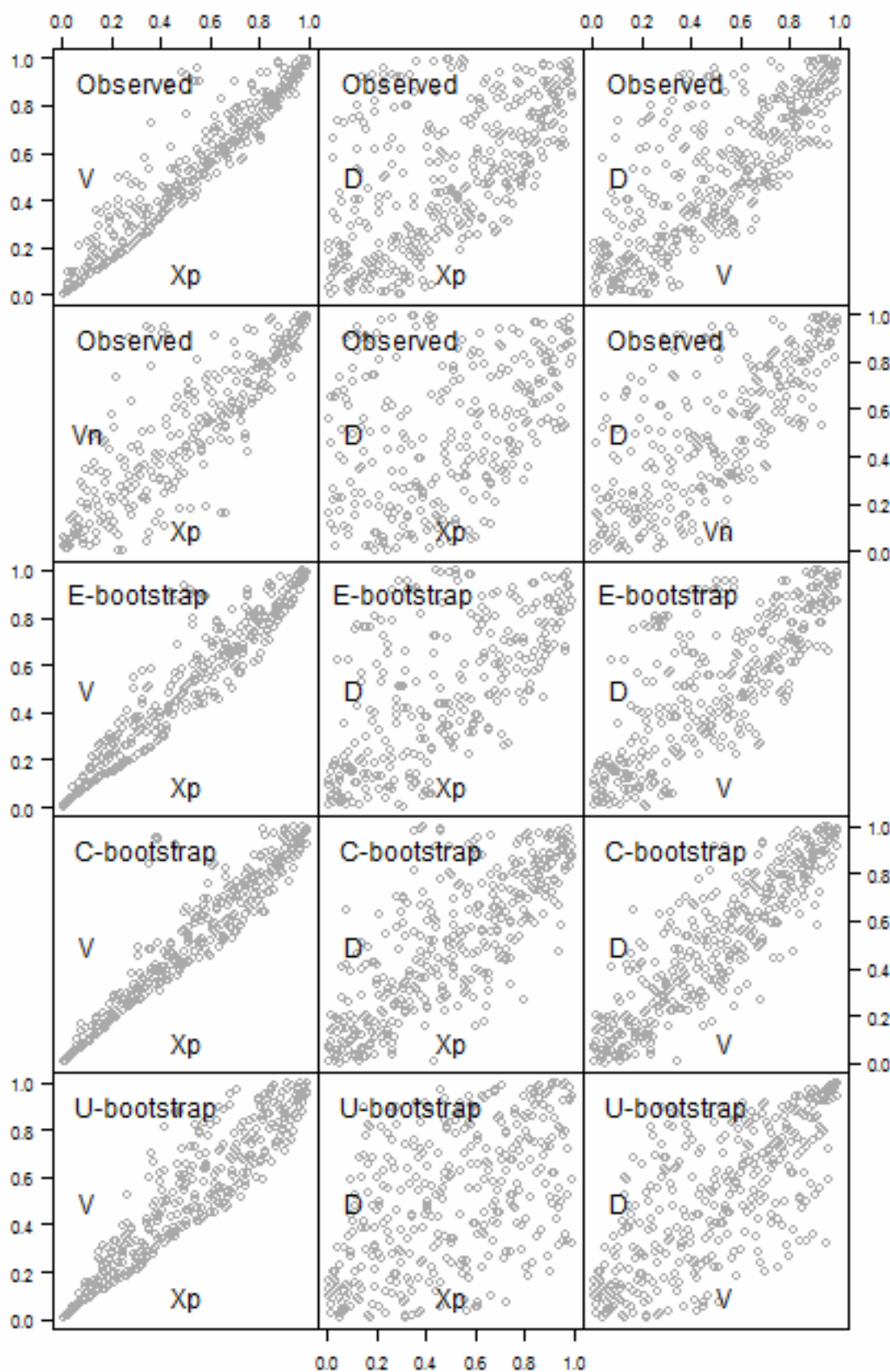

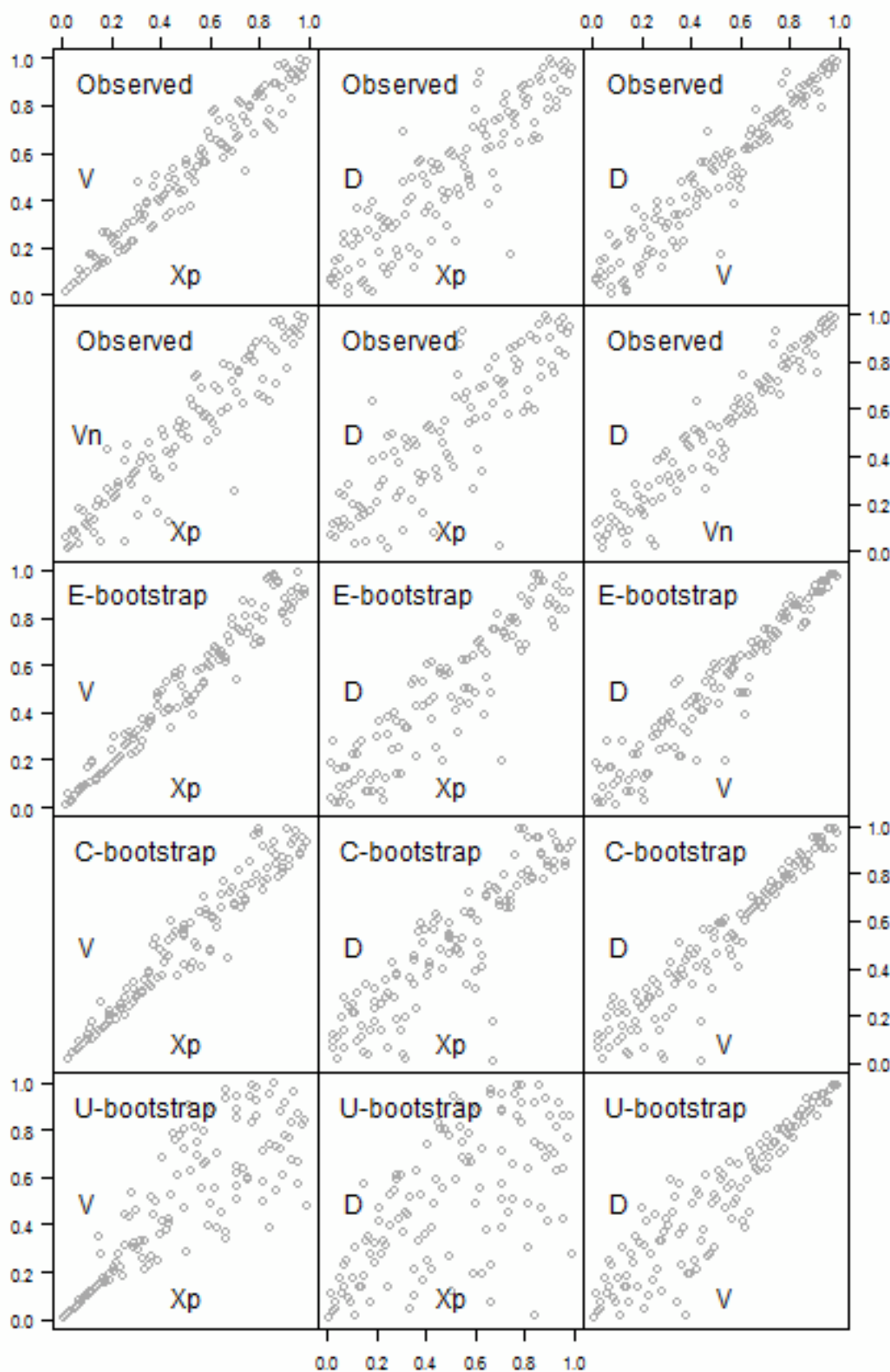

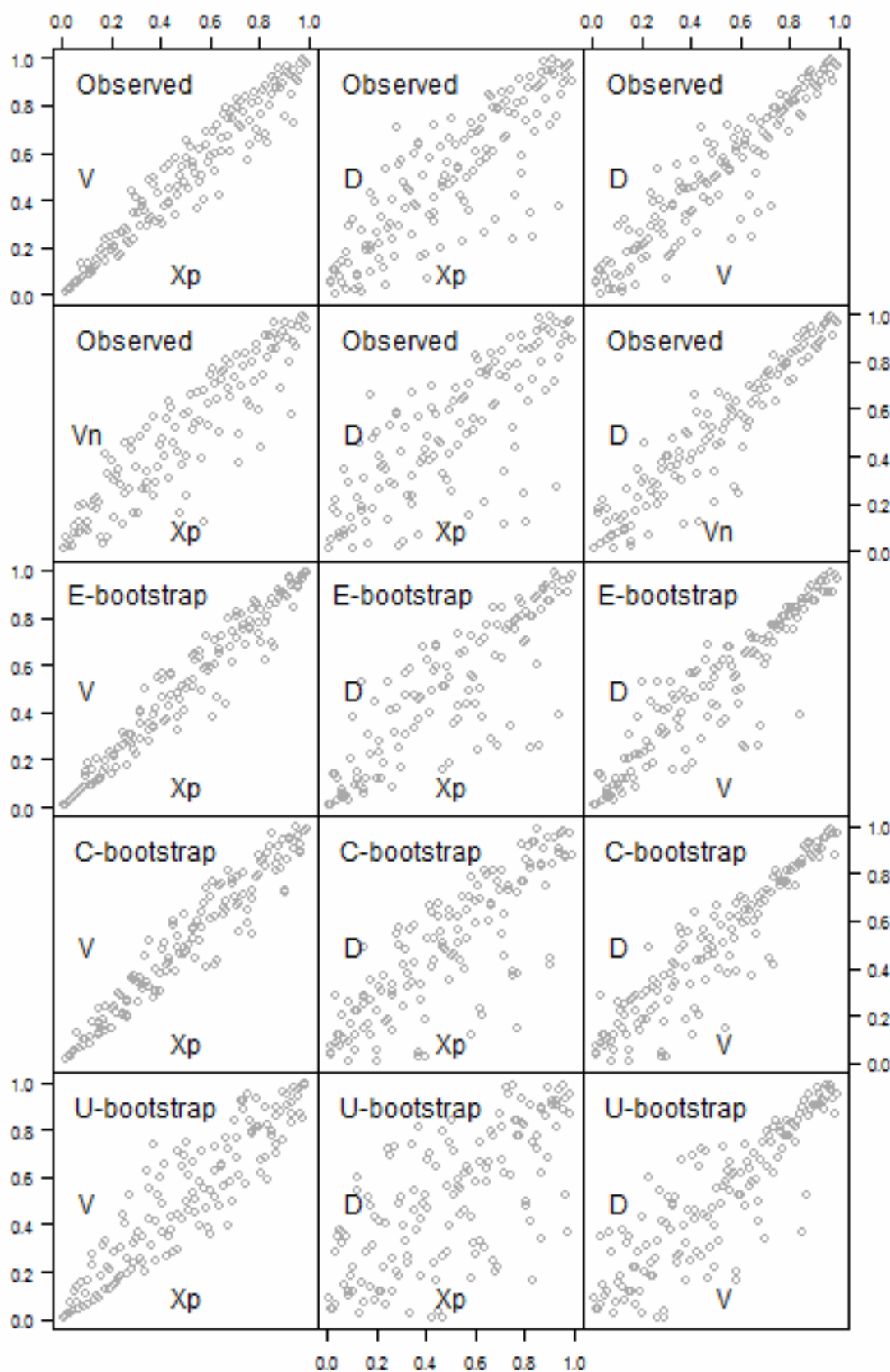

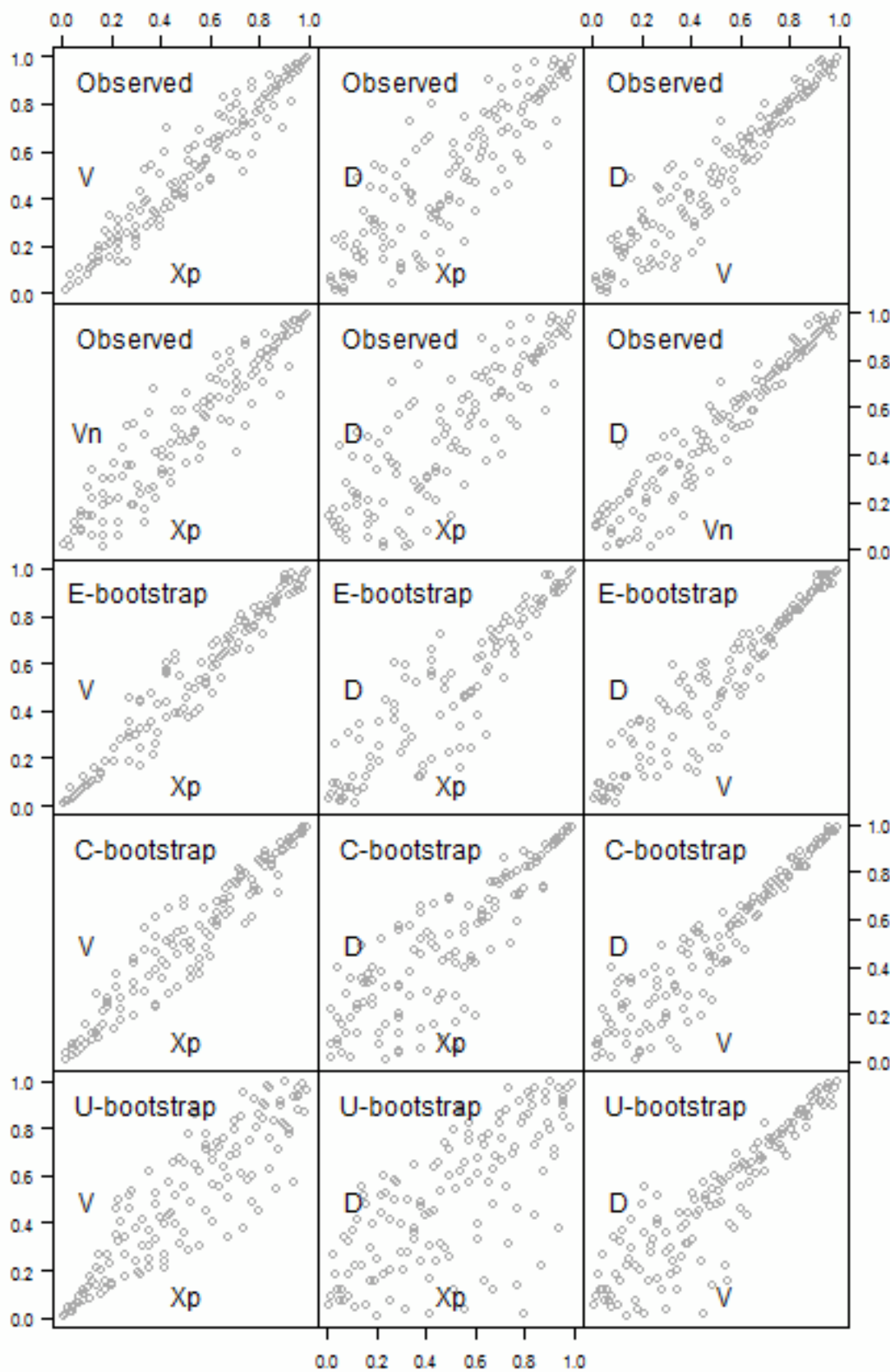

## USGS ID: 6225000

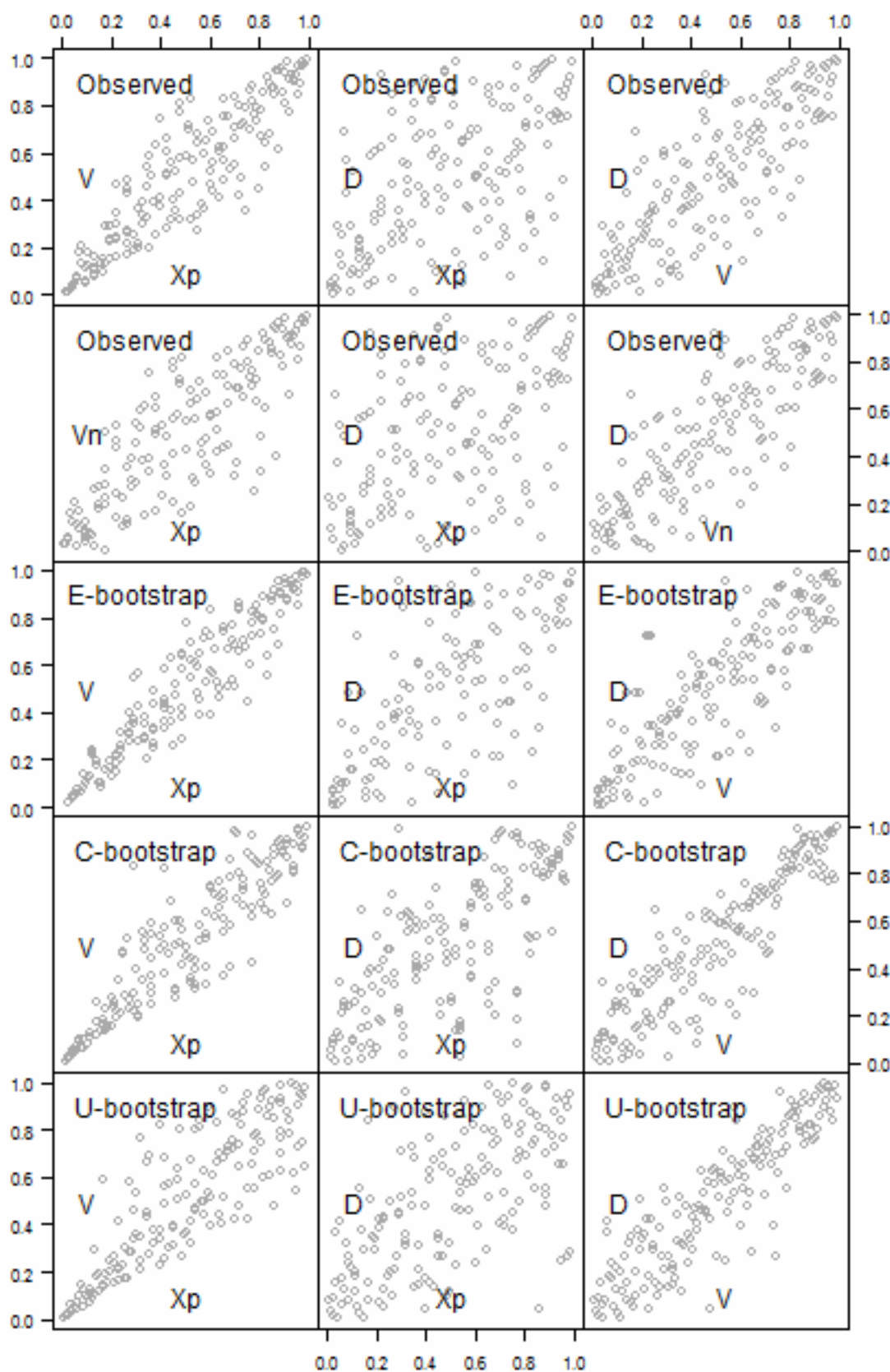

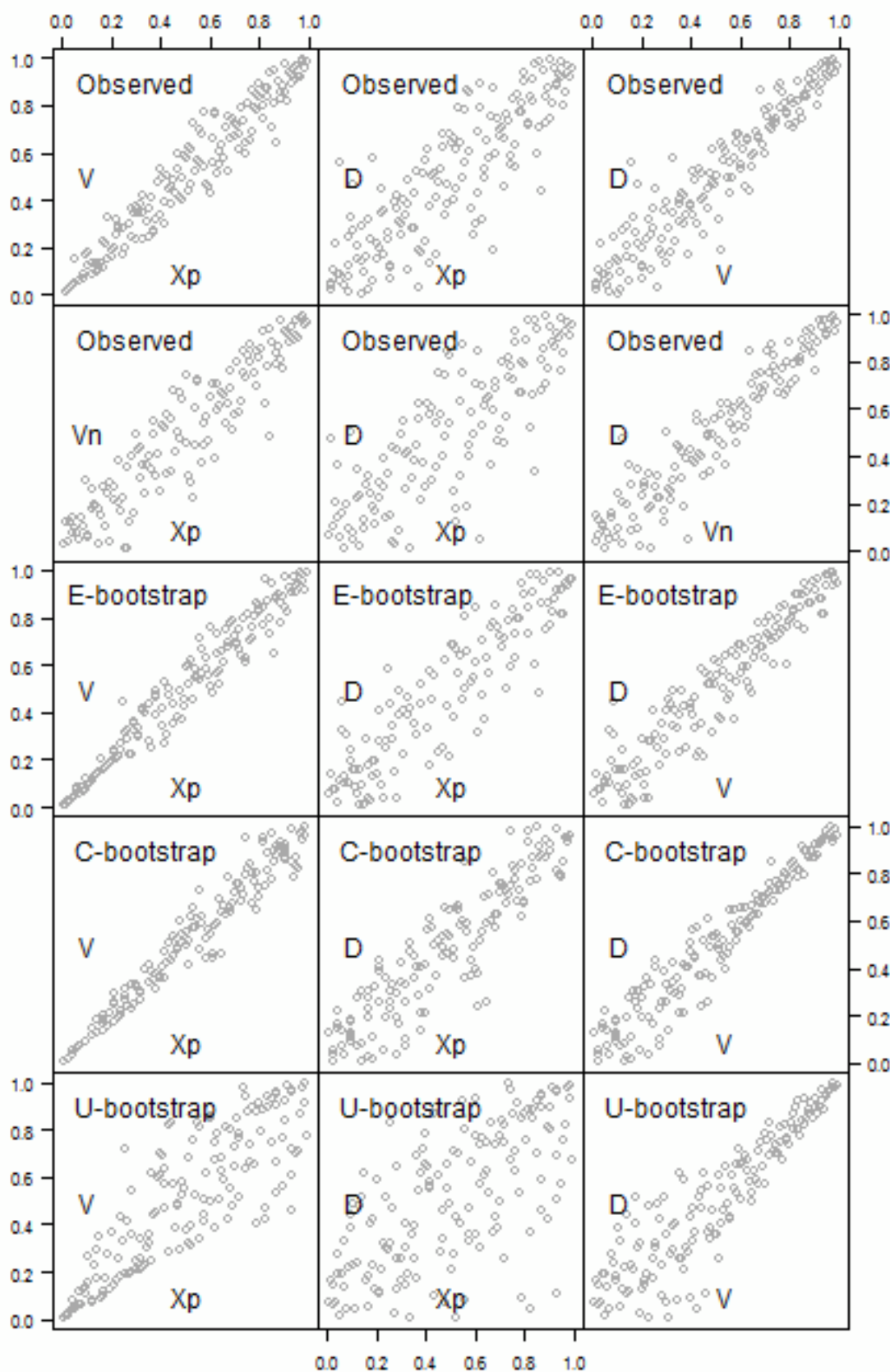

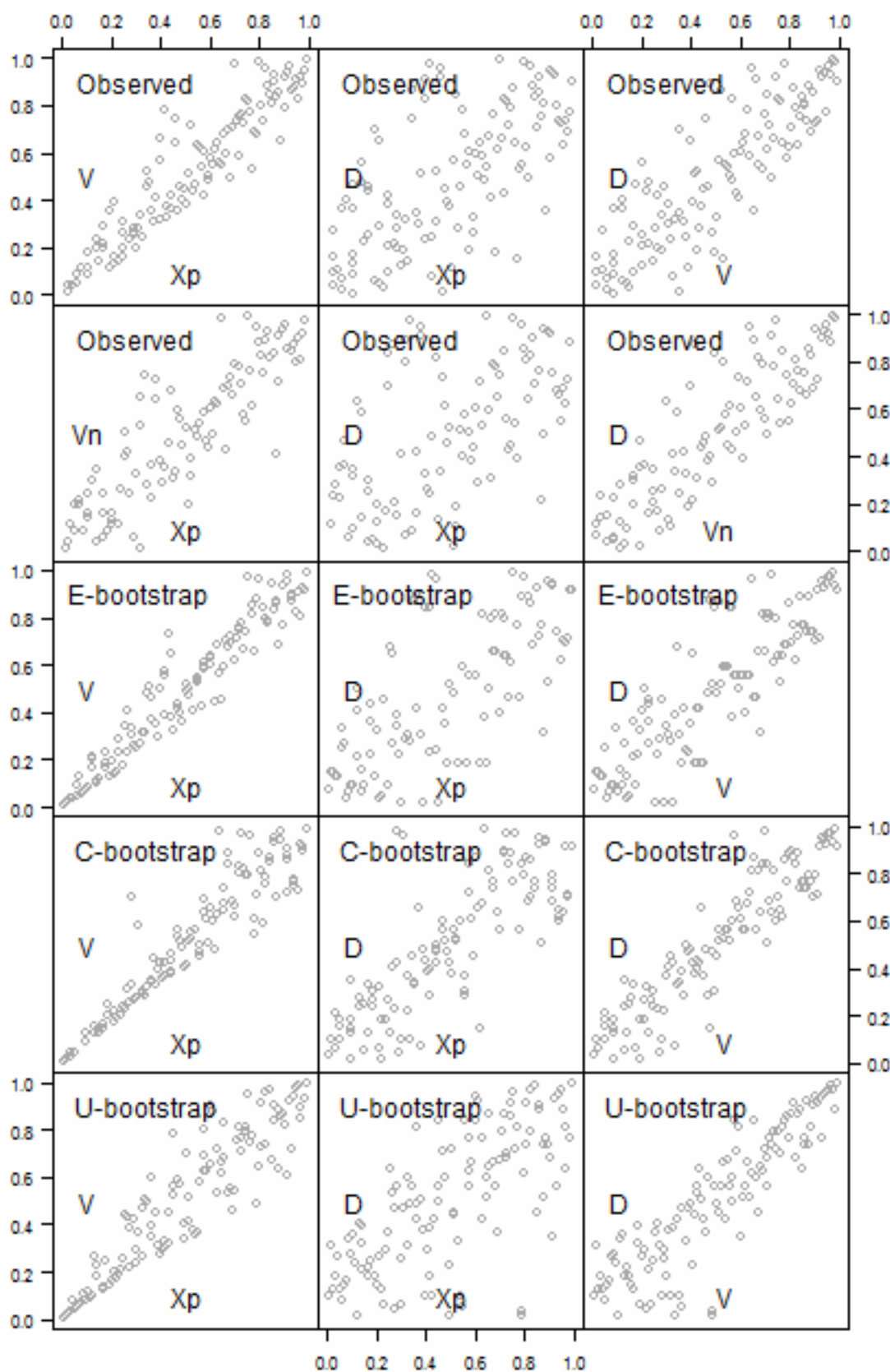

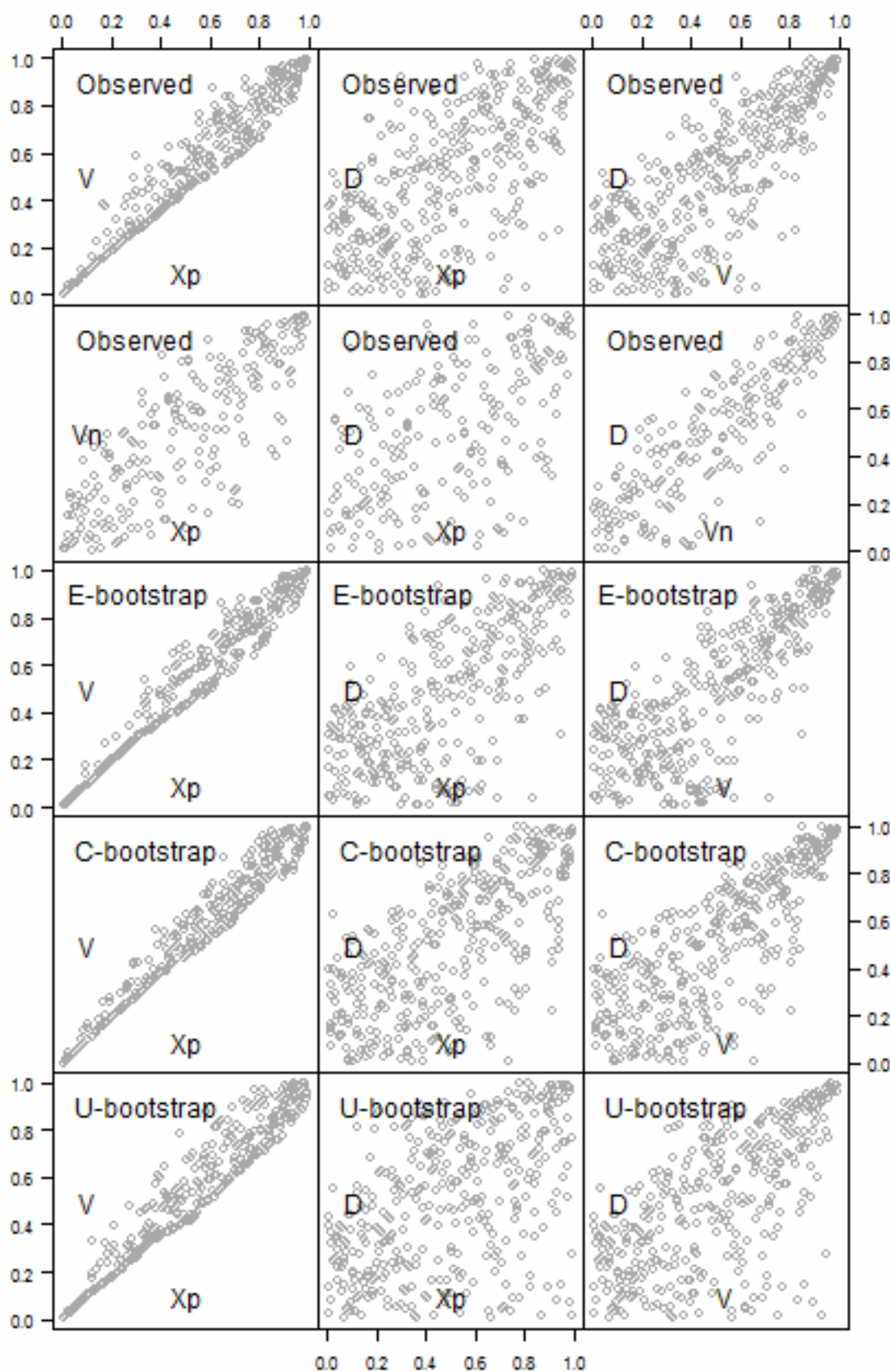

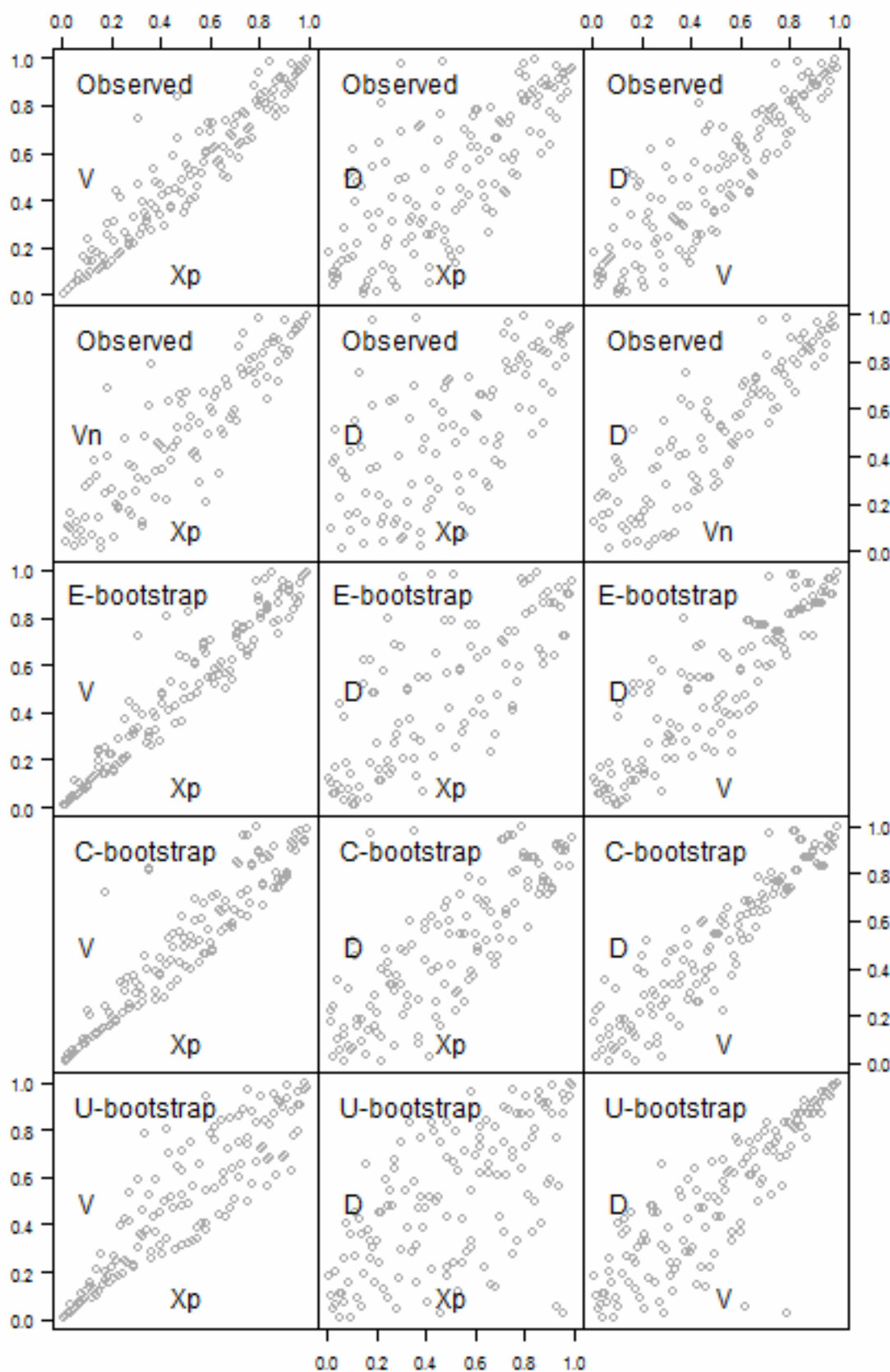

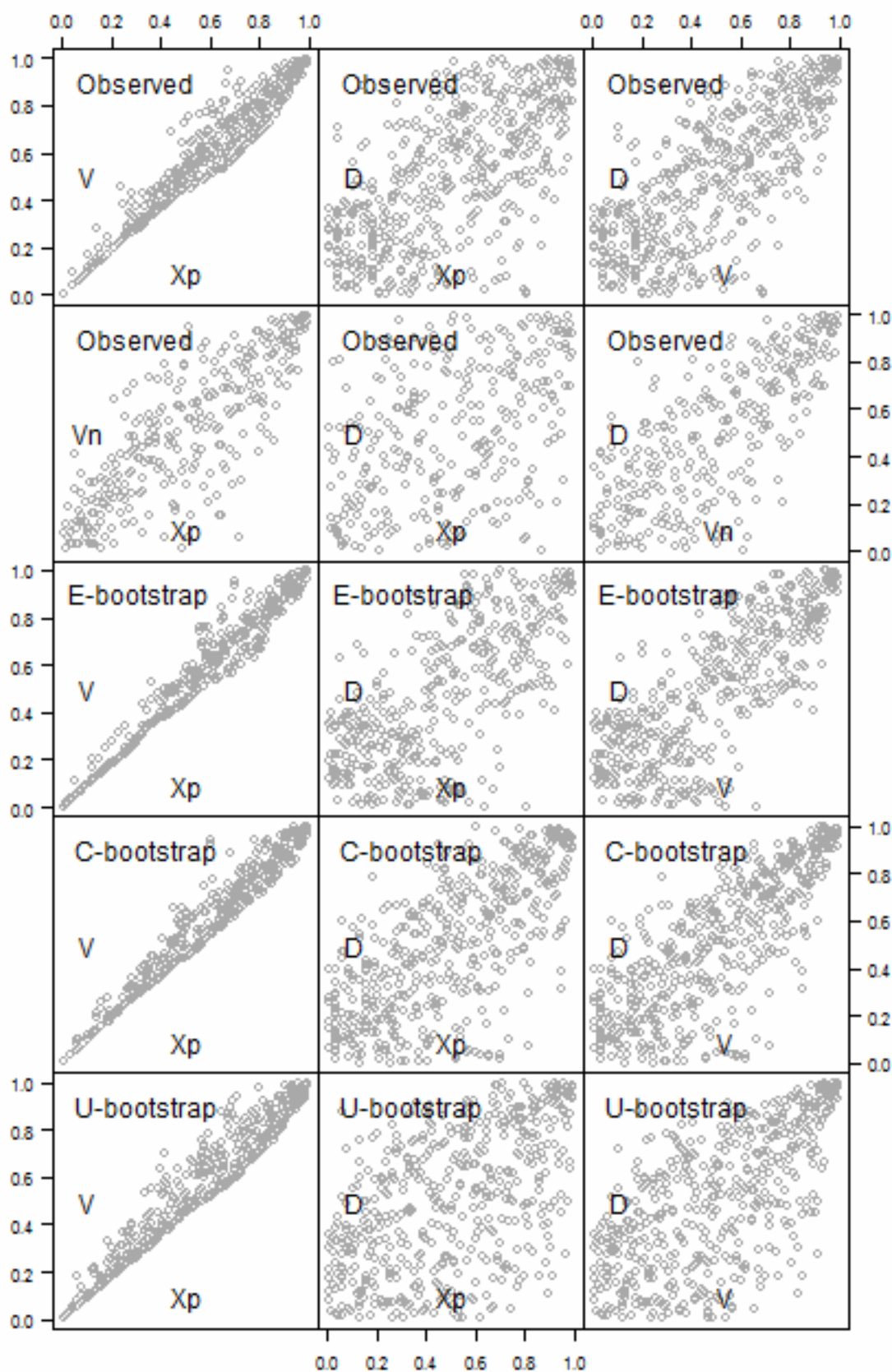

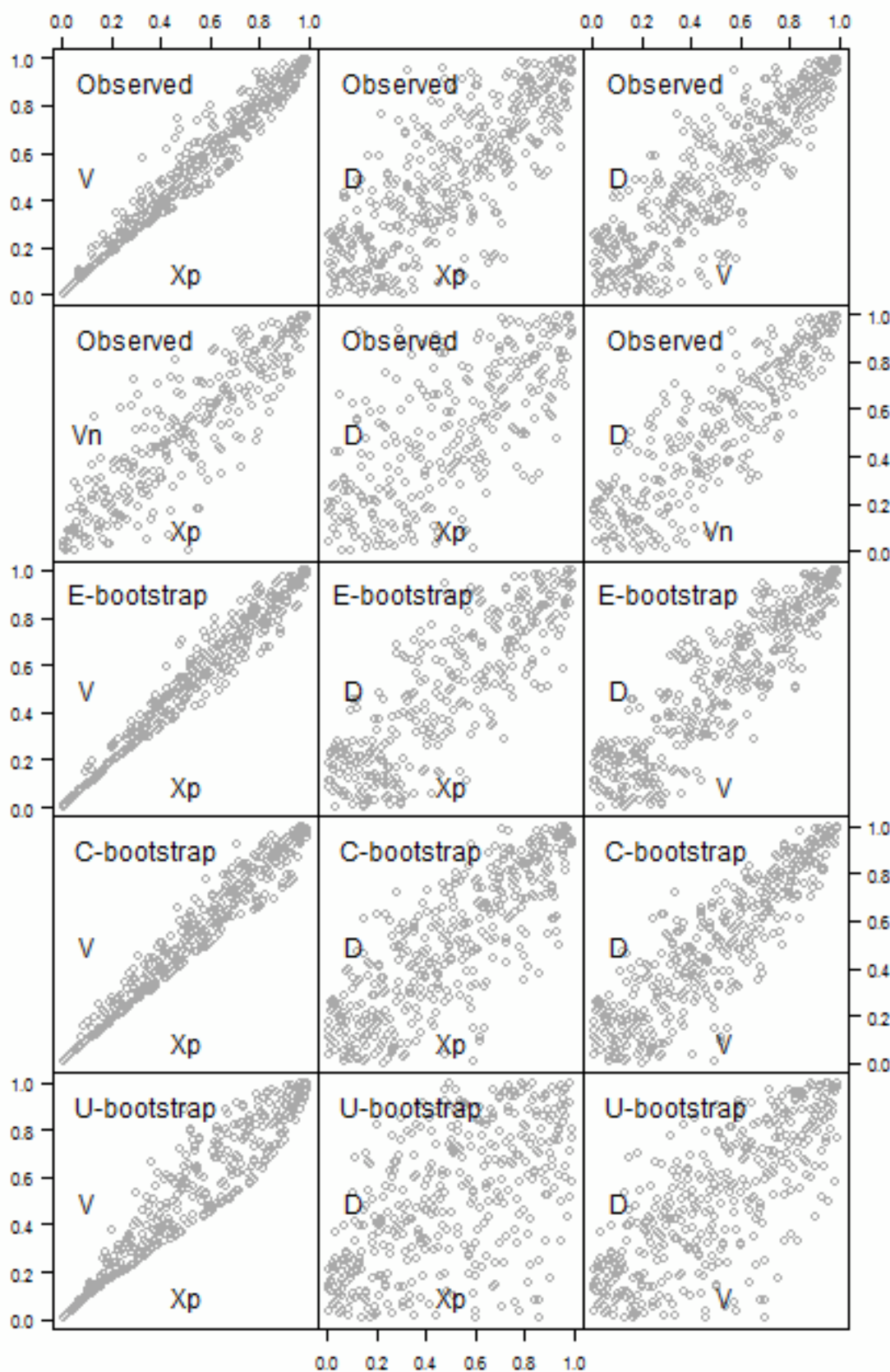

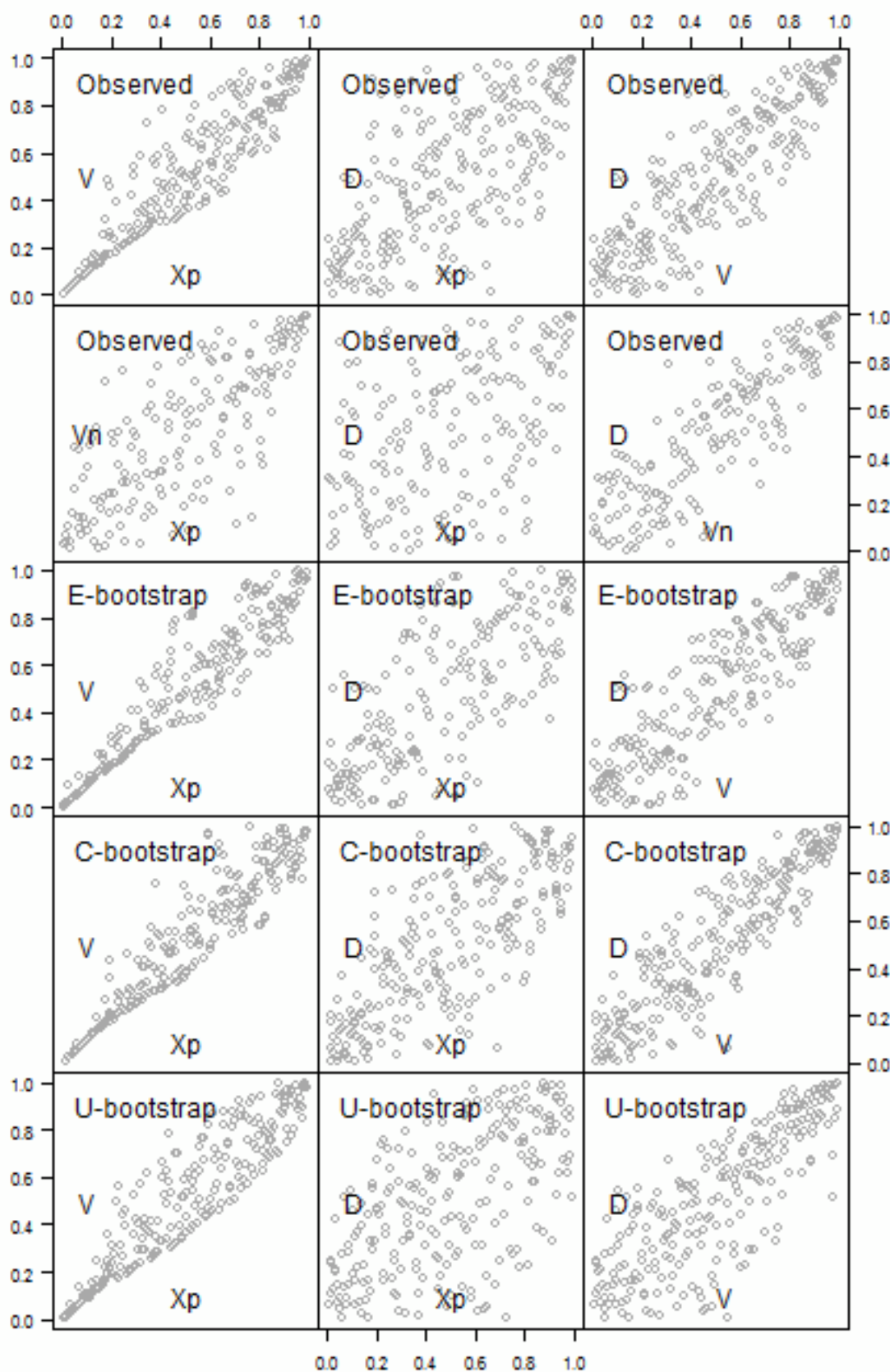

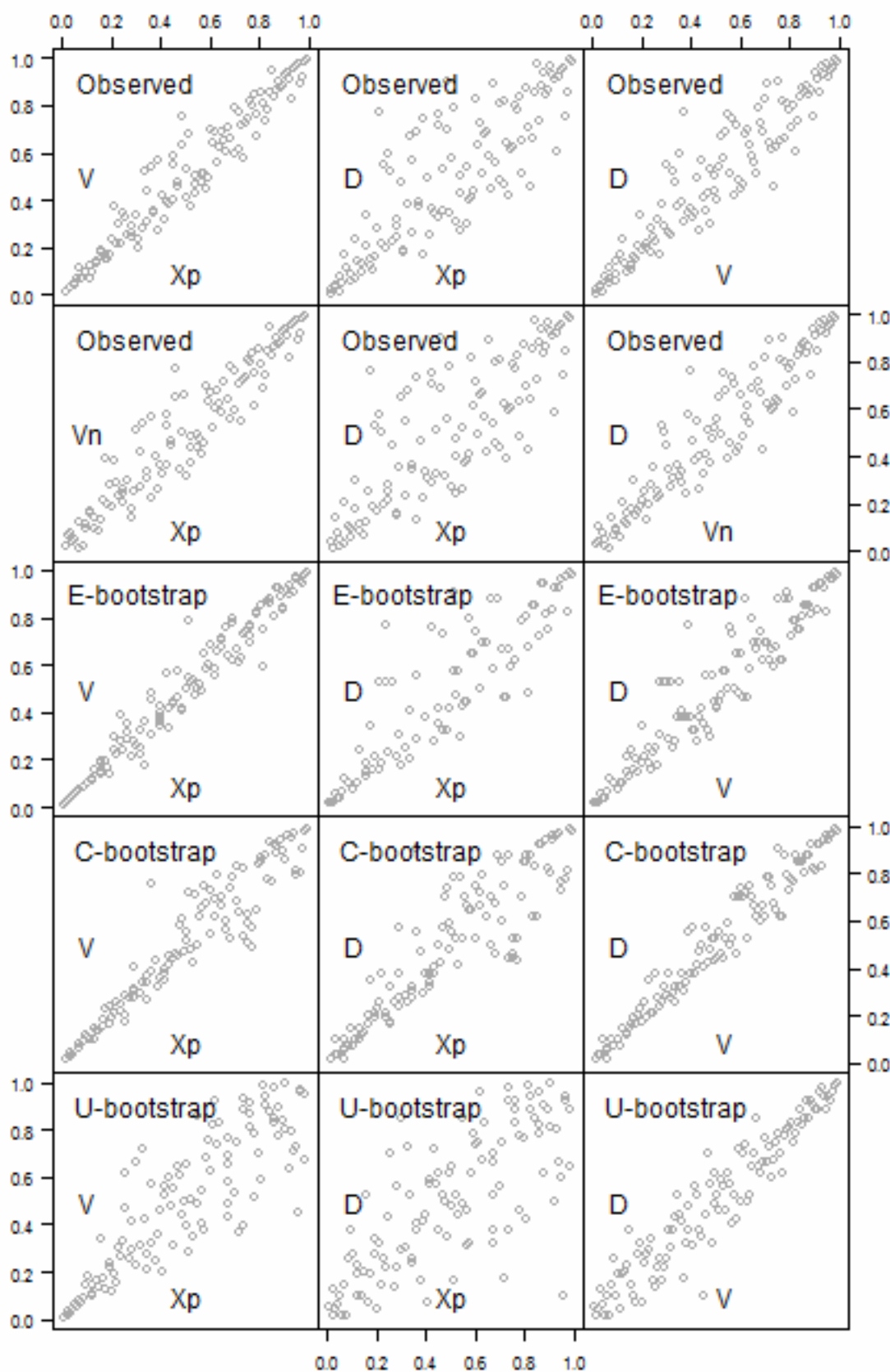

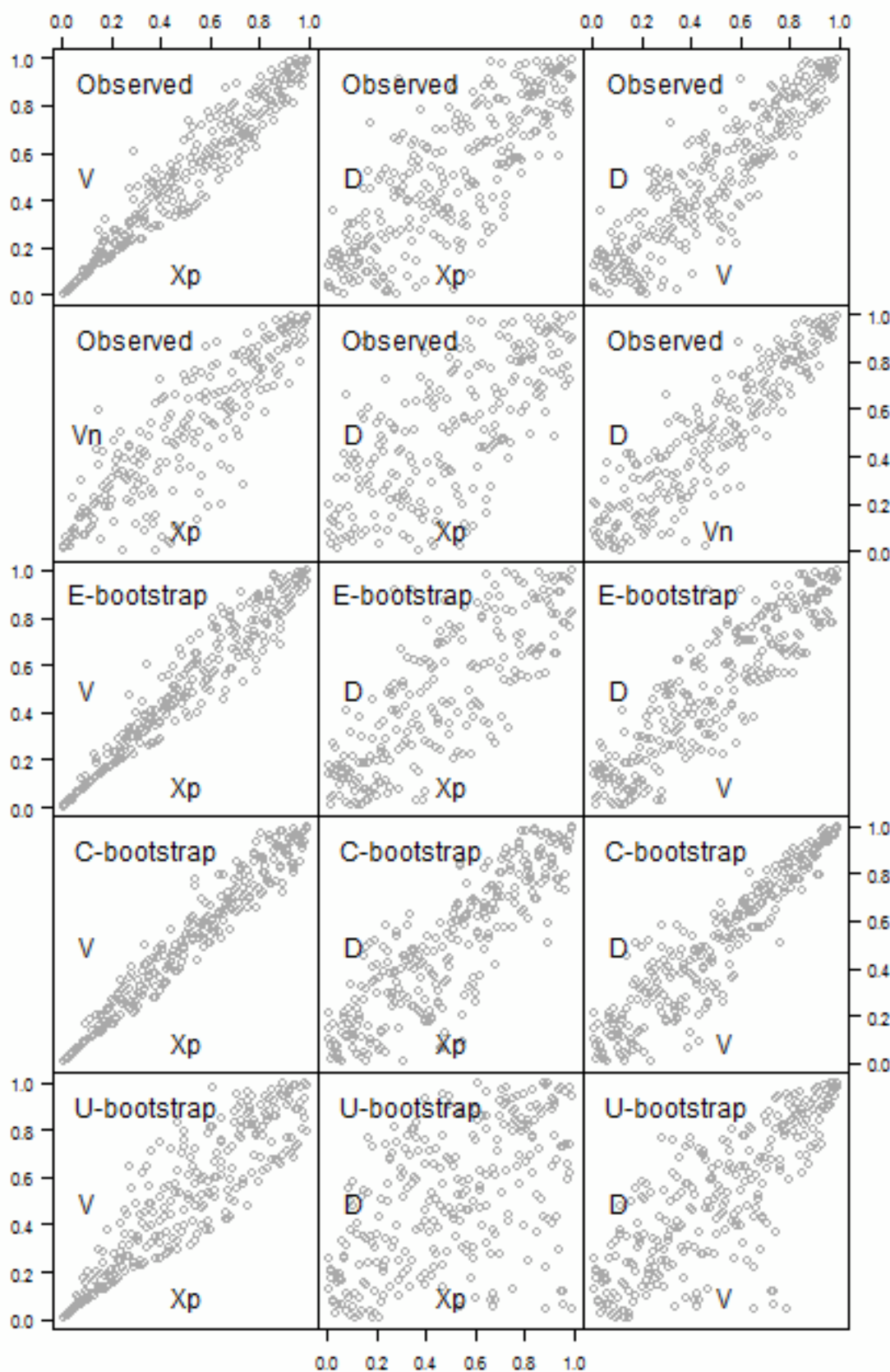

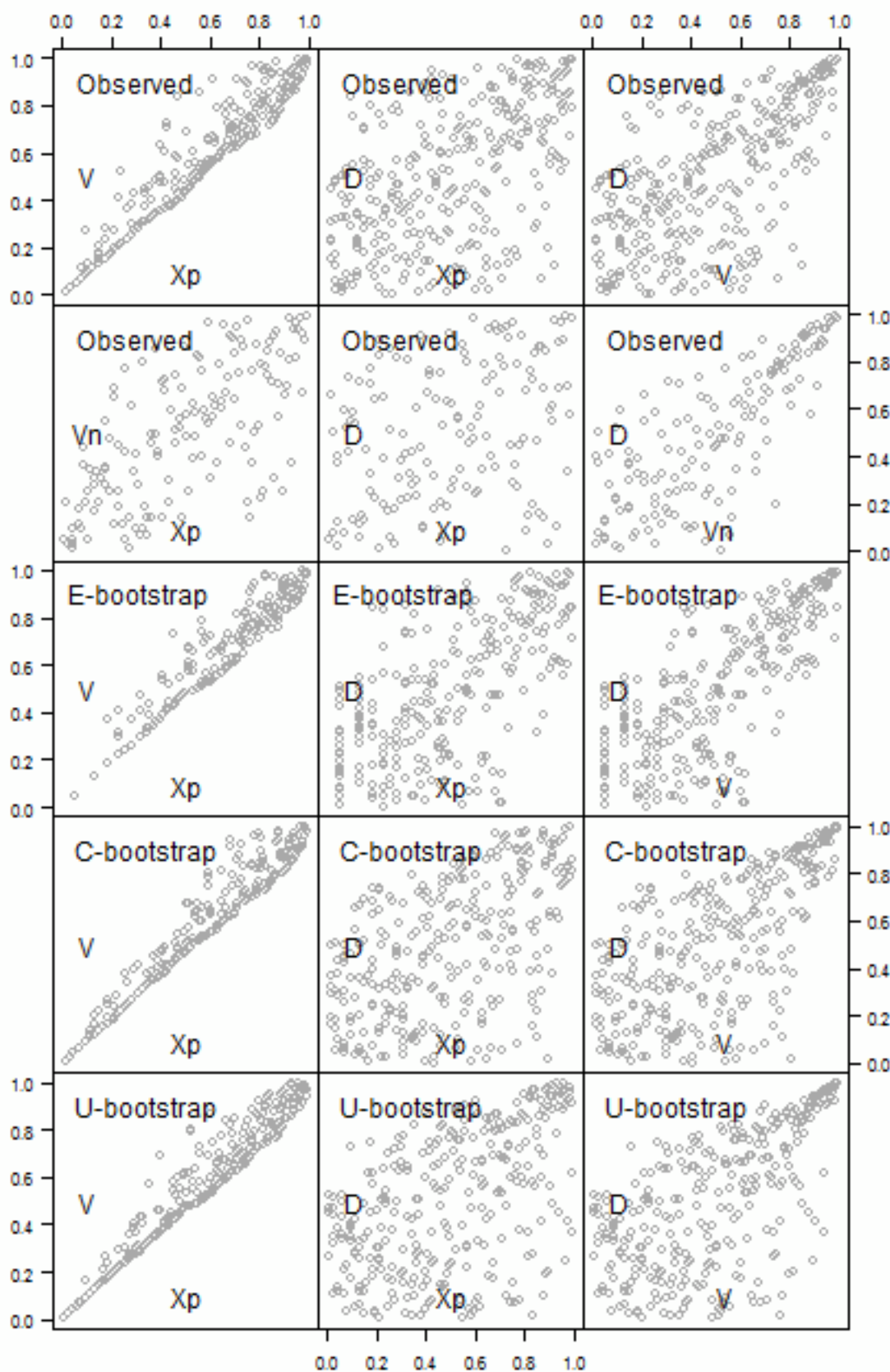

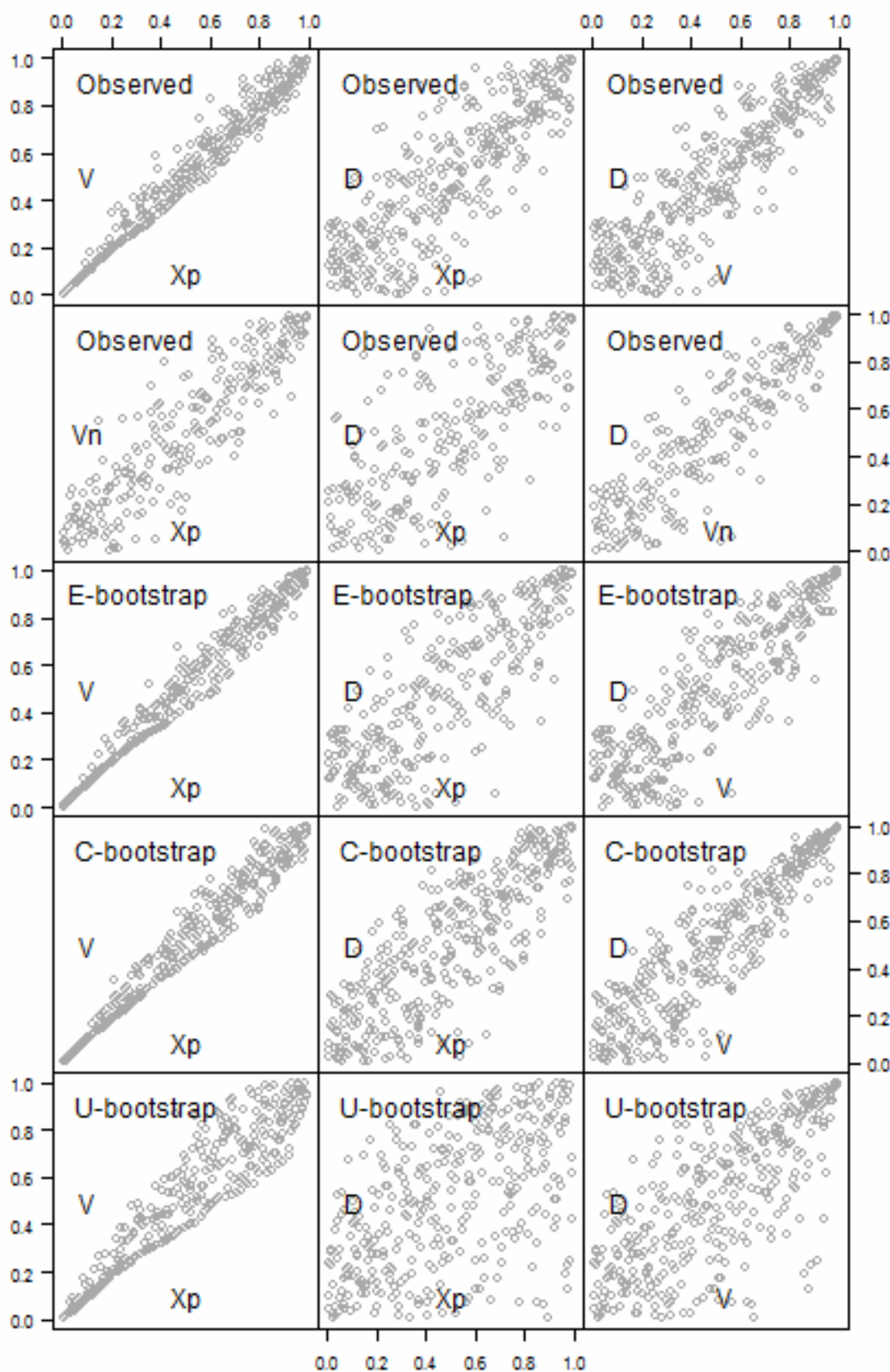

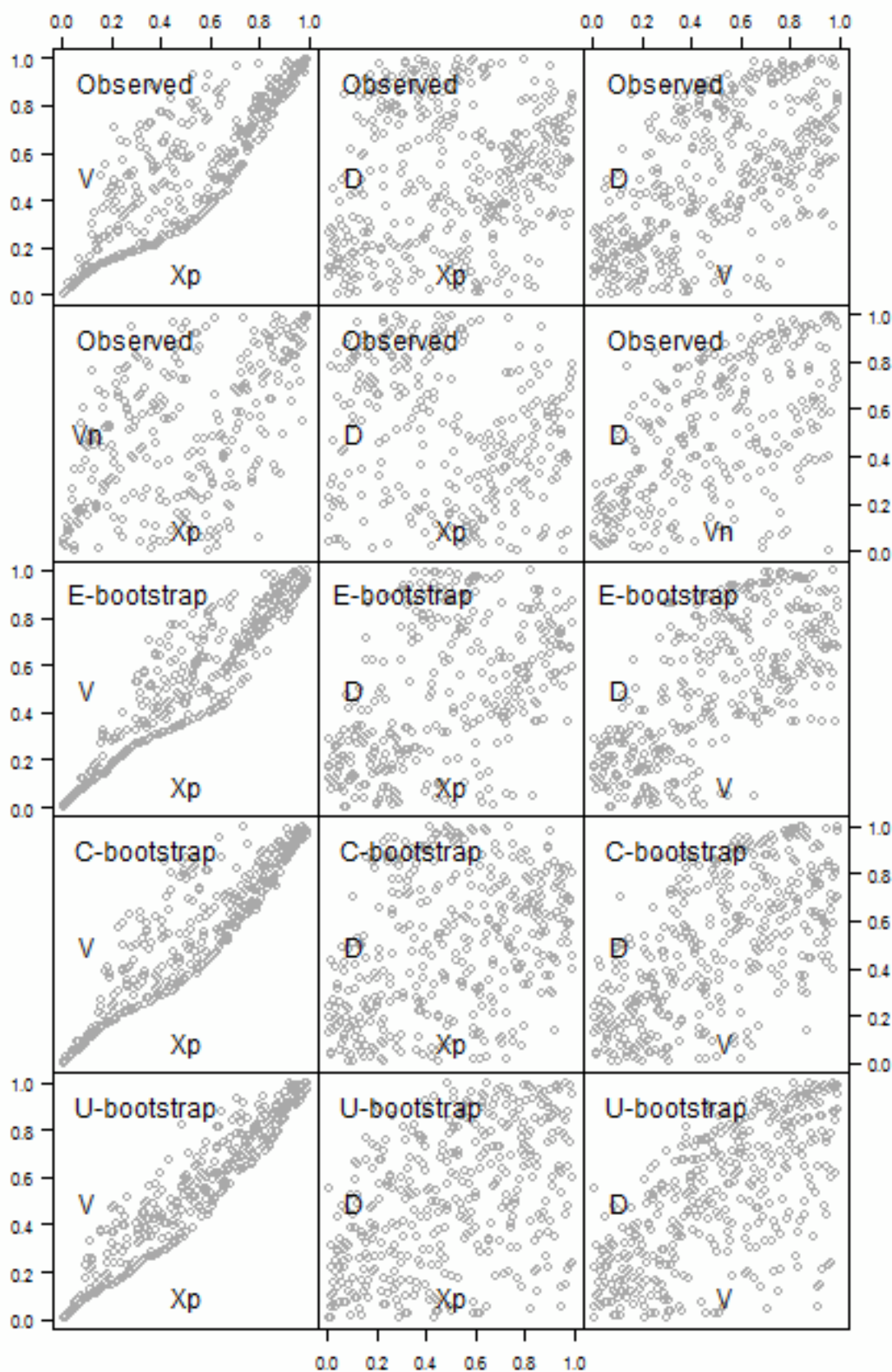

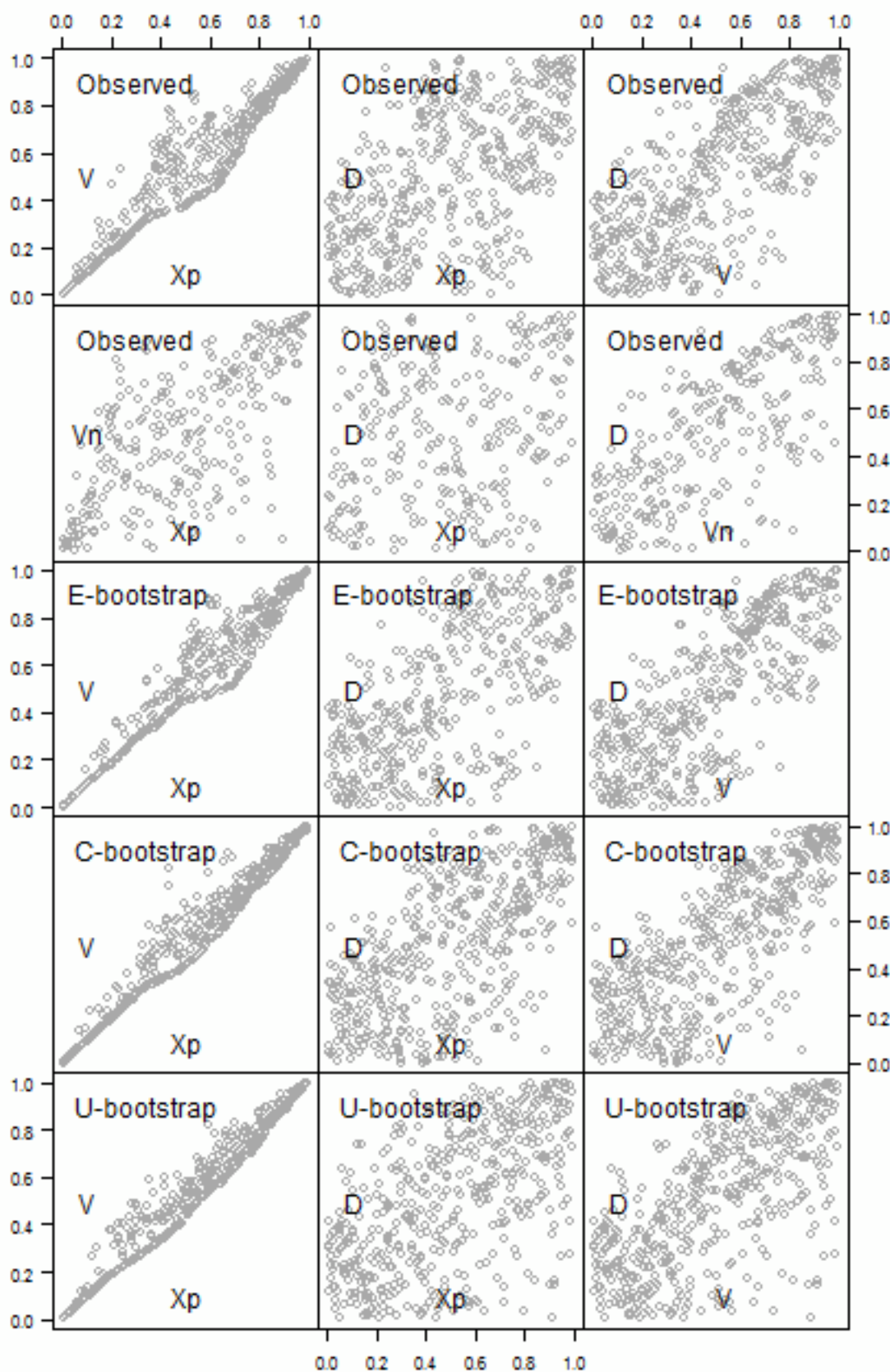

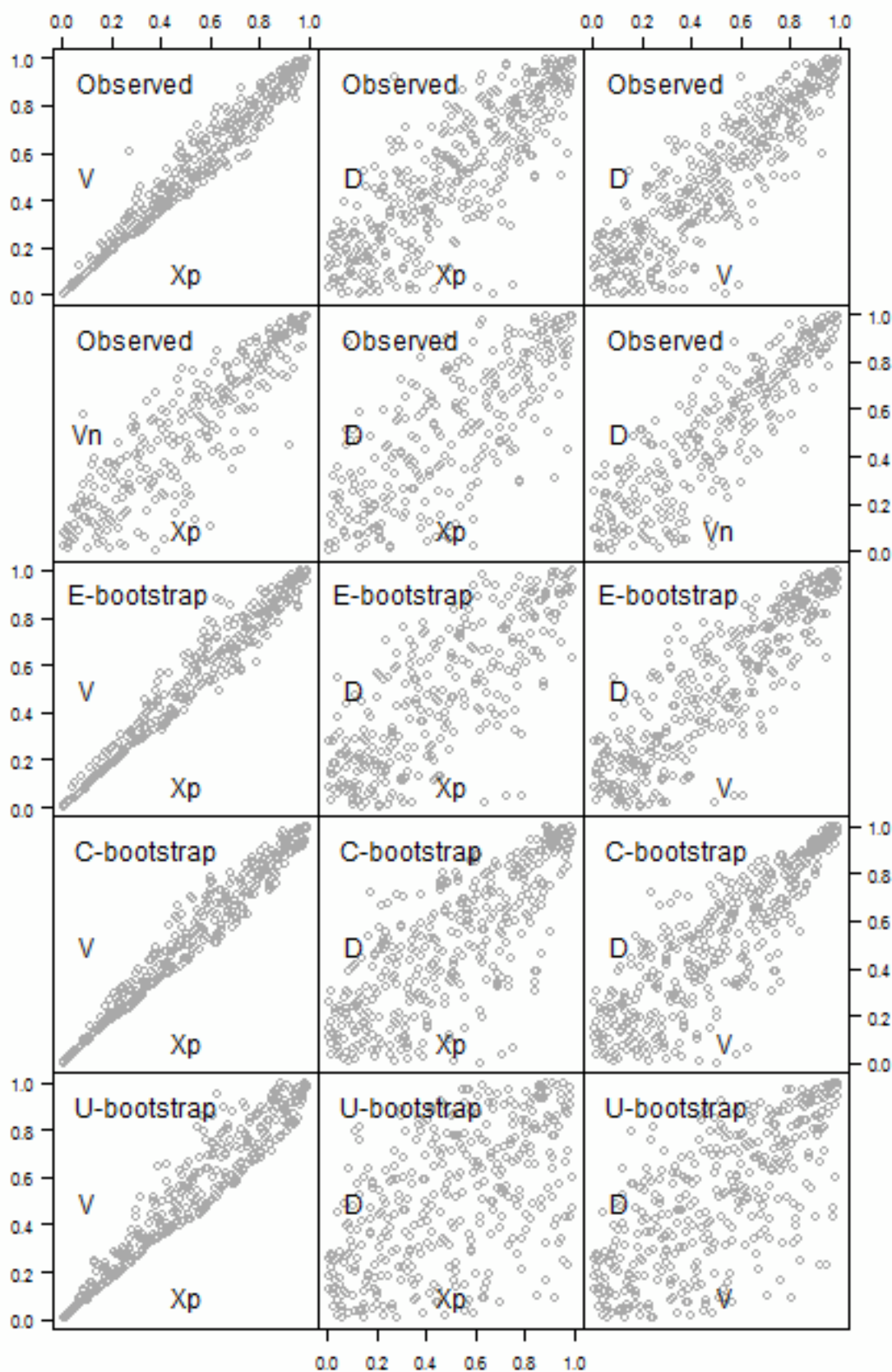

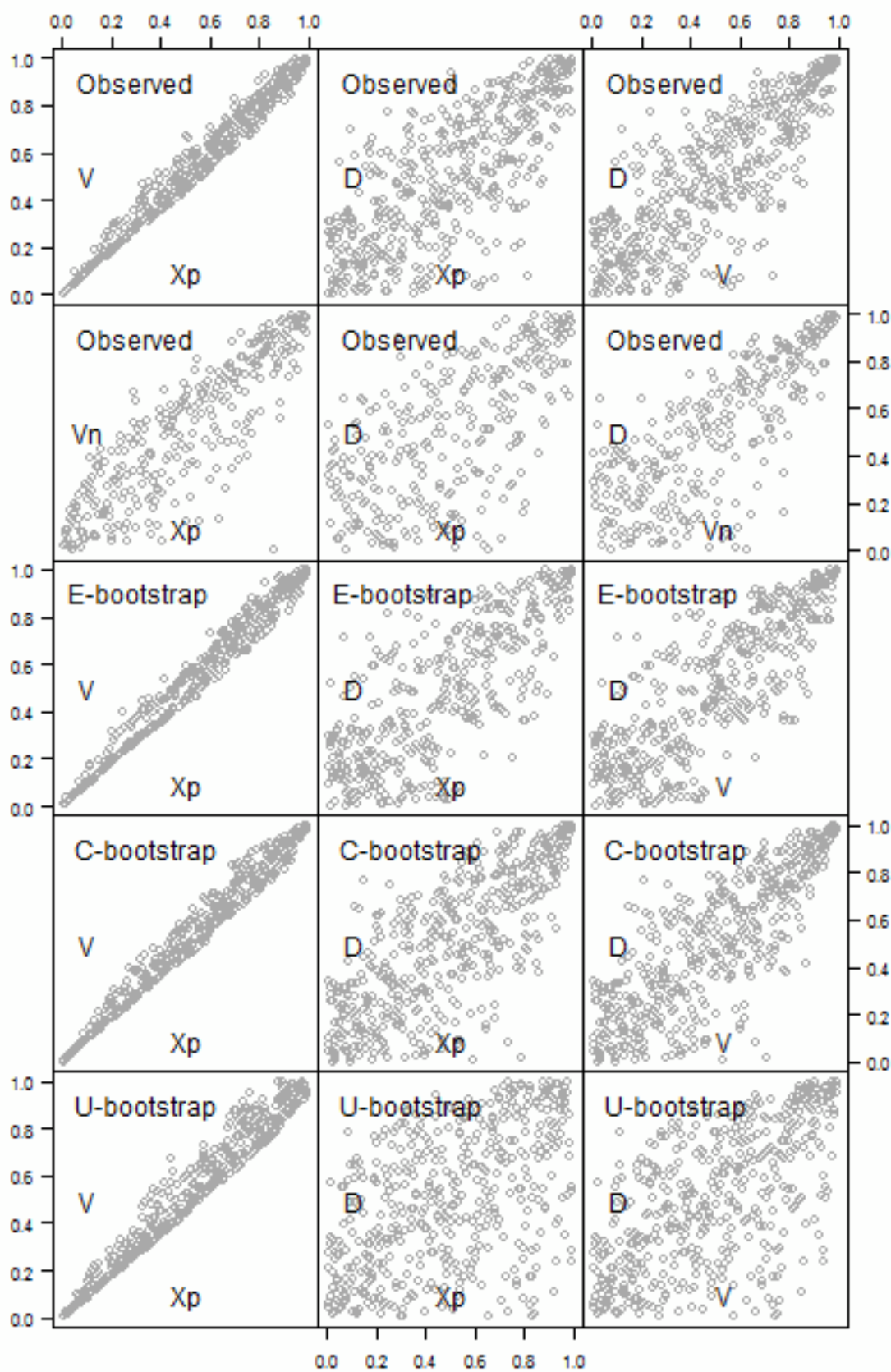

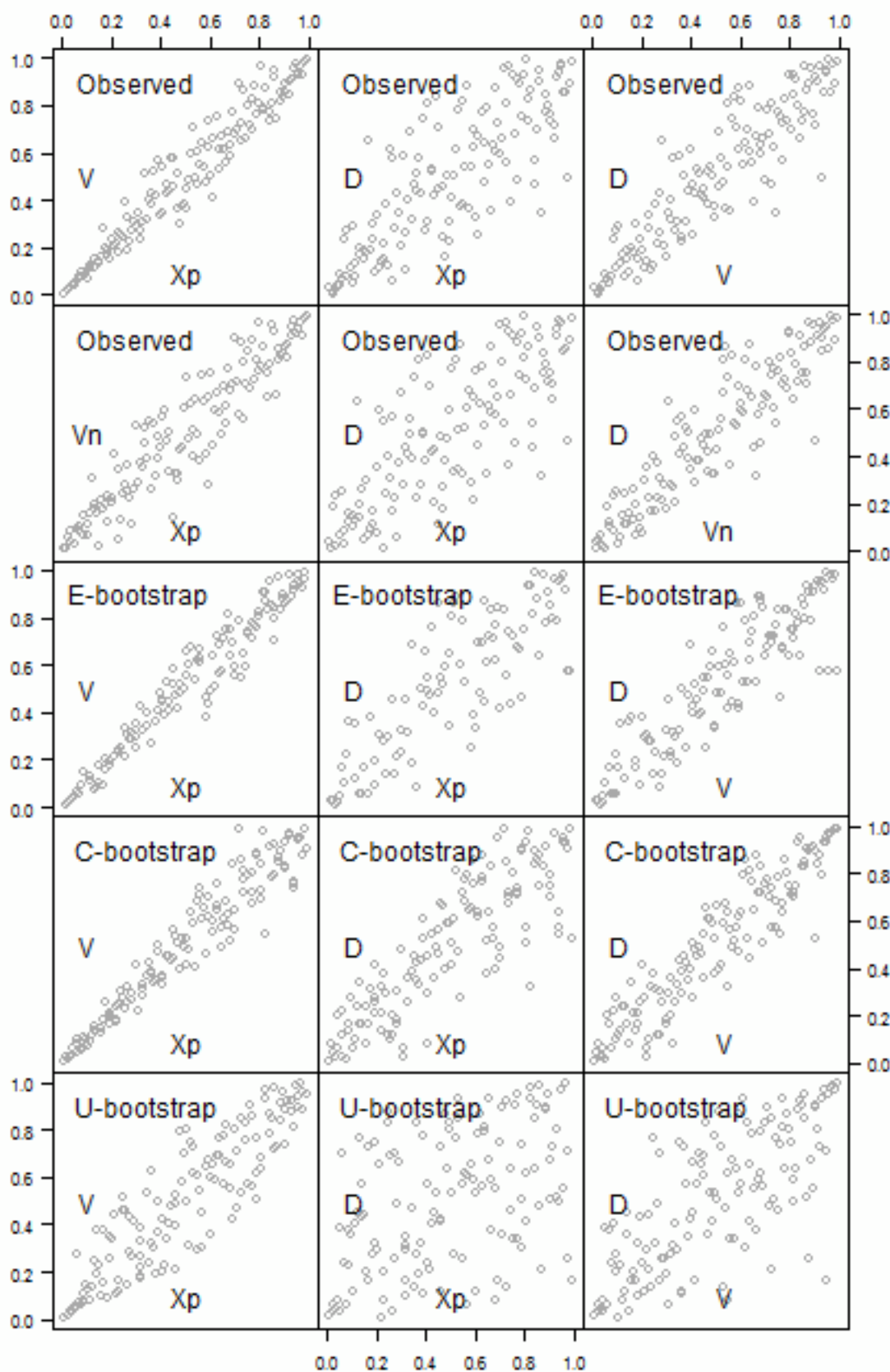

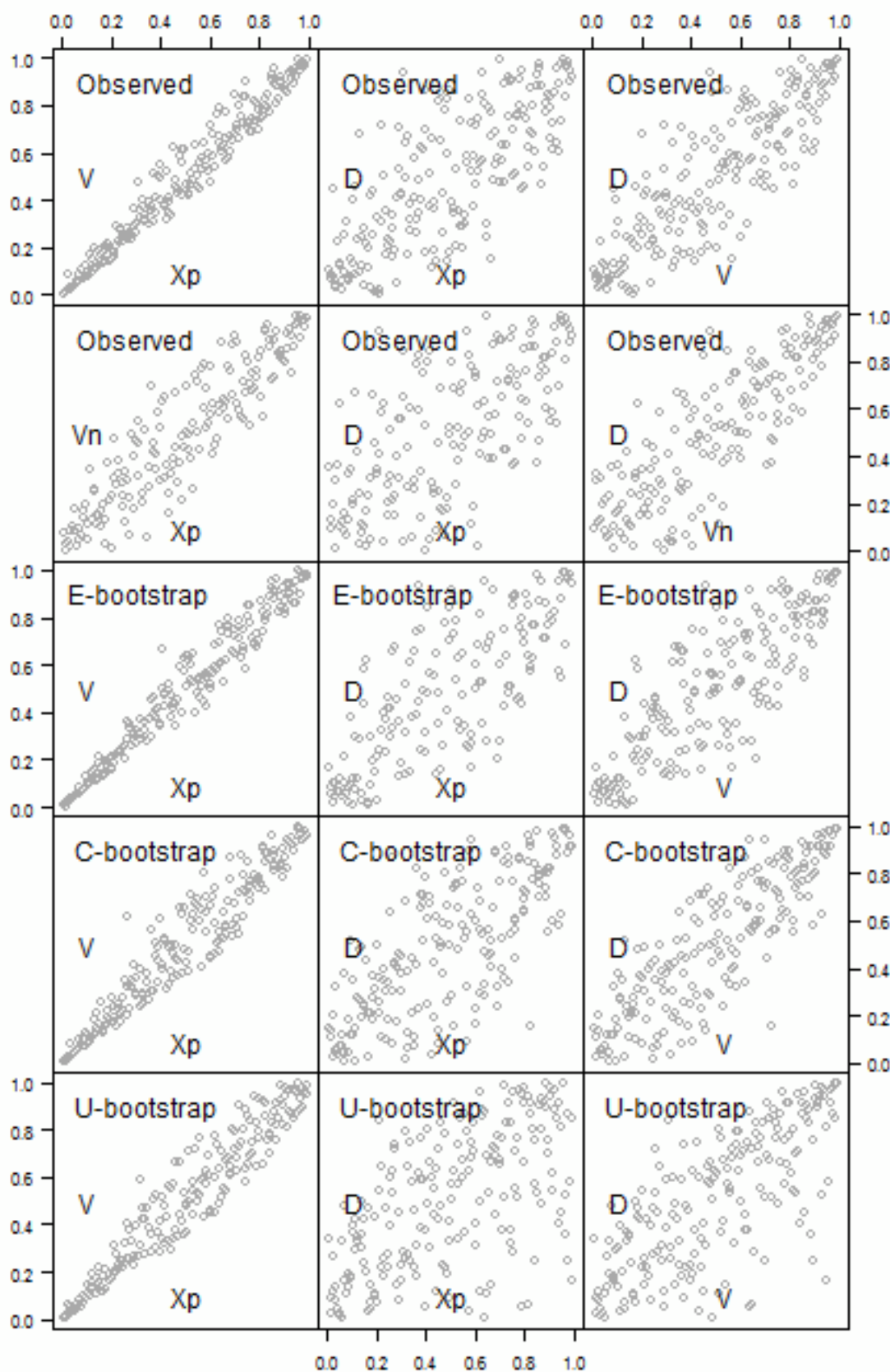

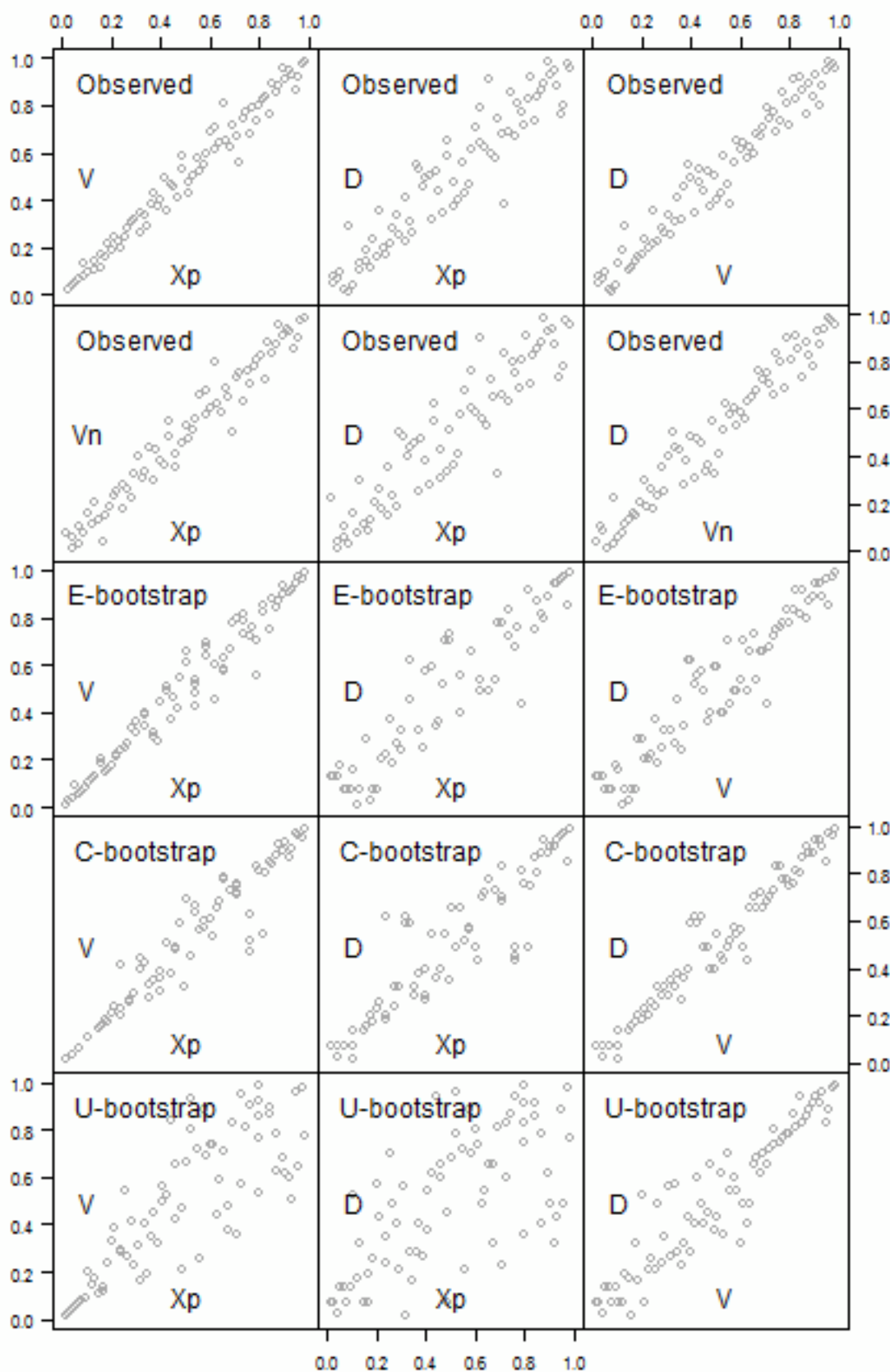

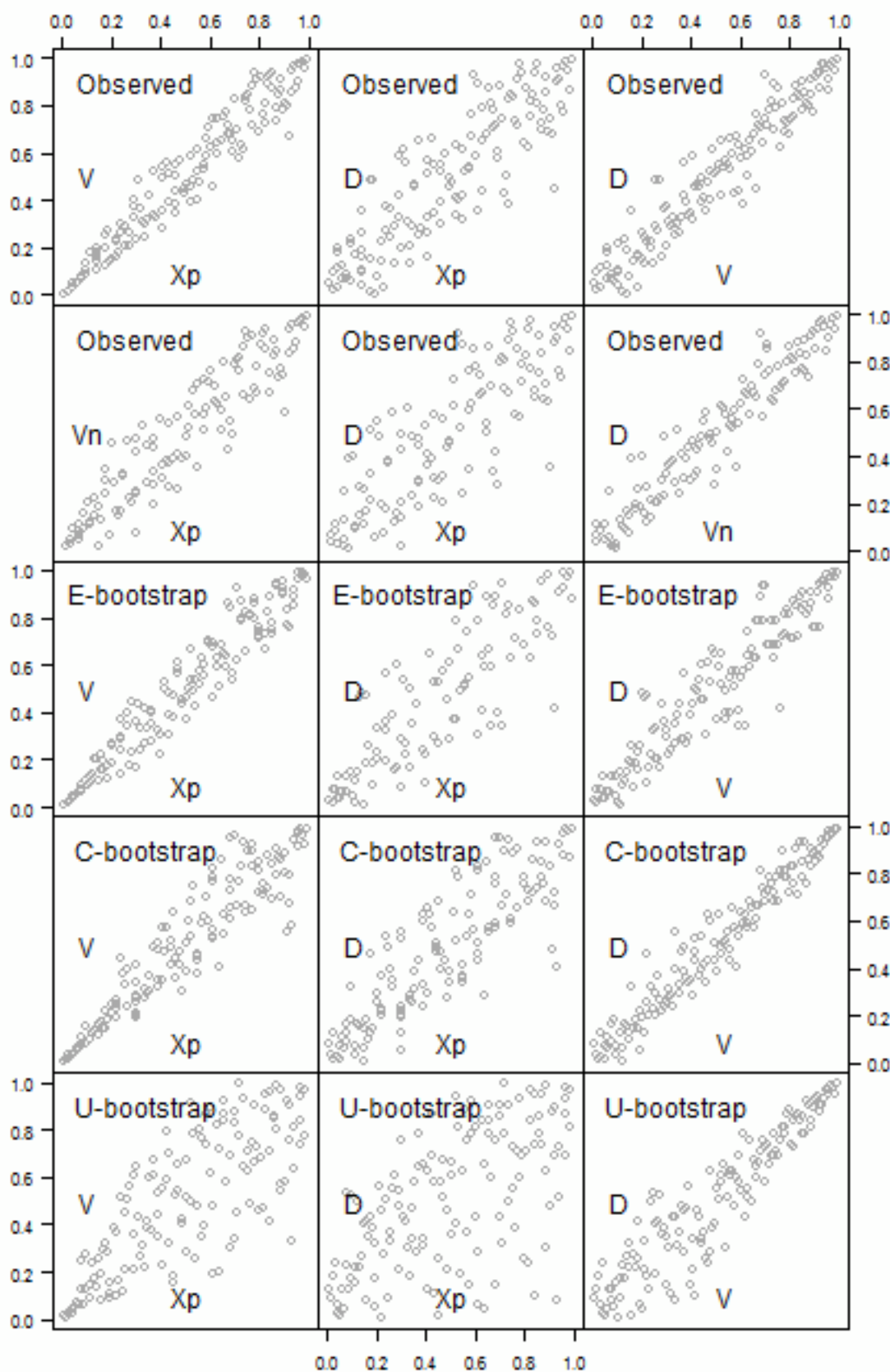

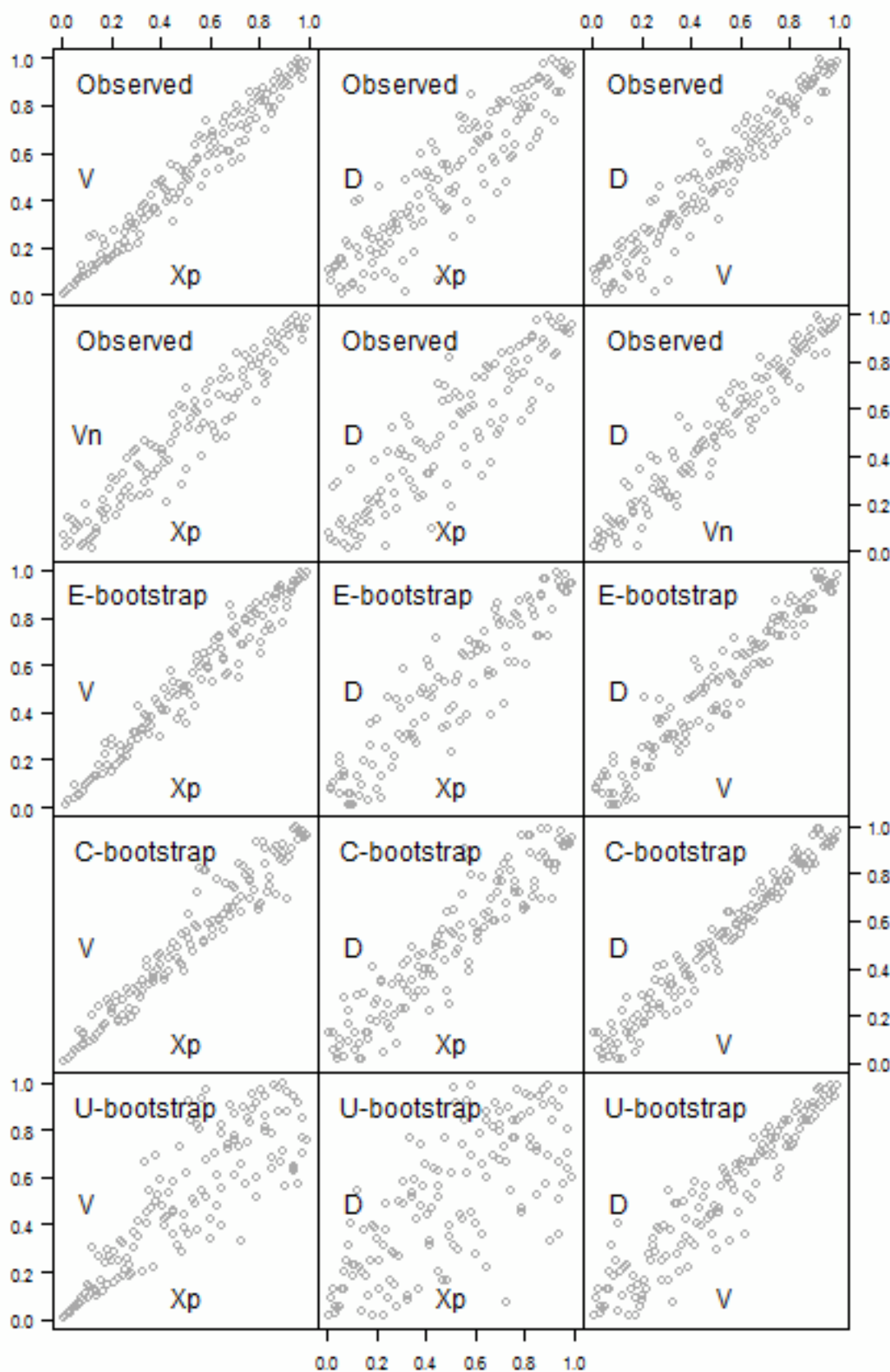

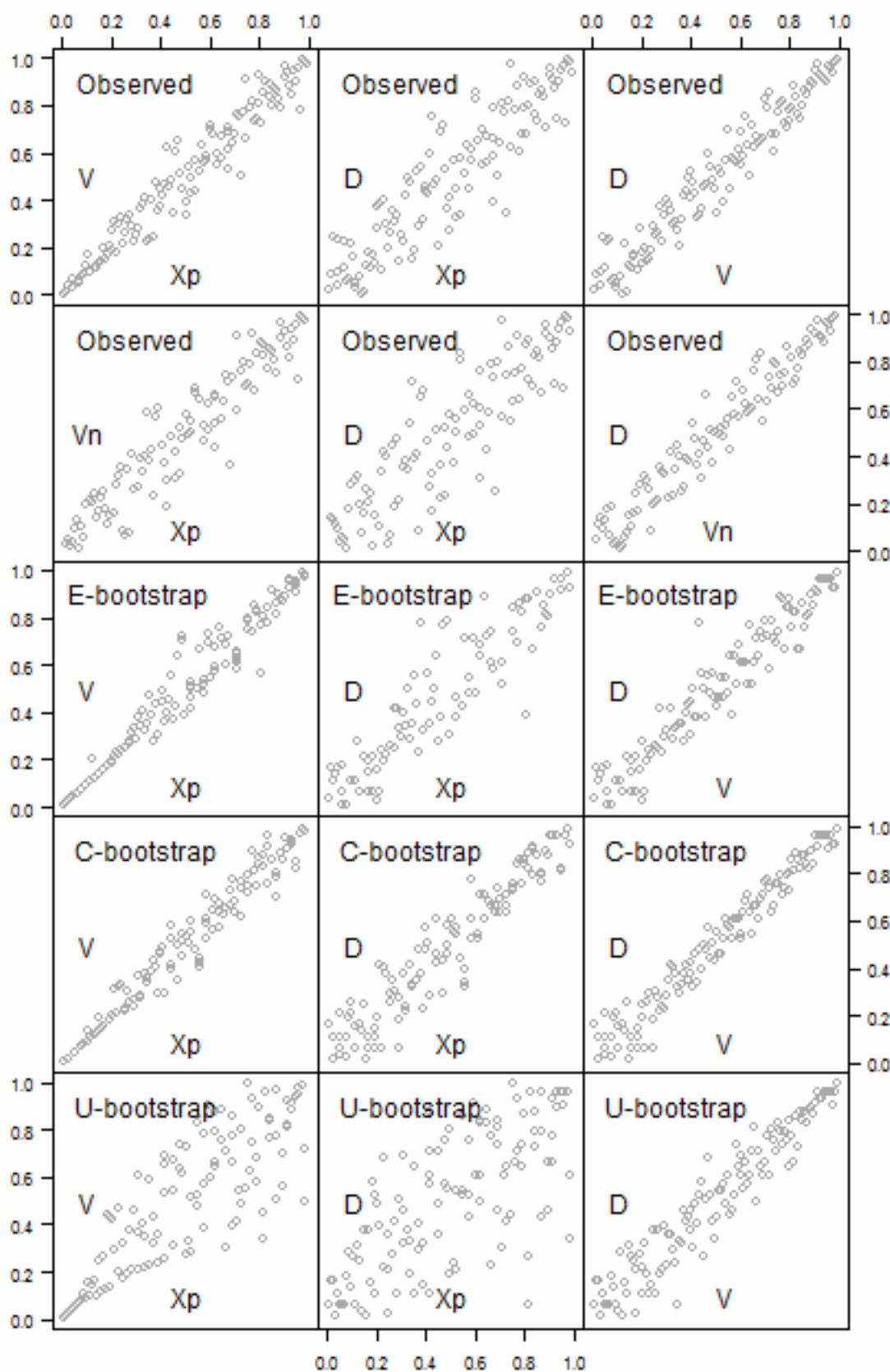

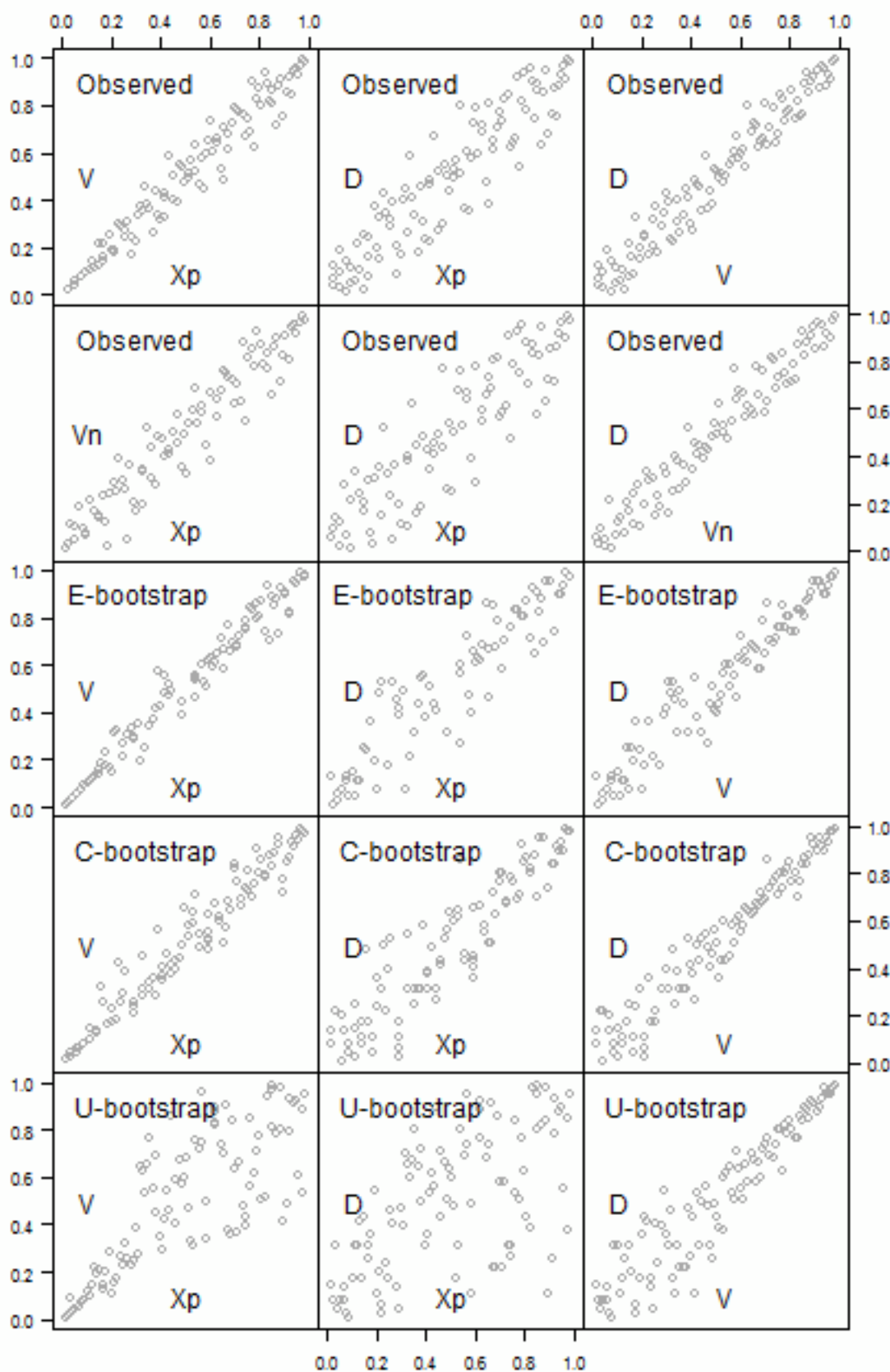

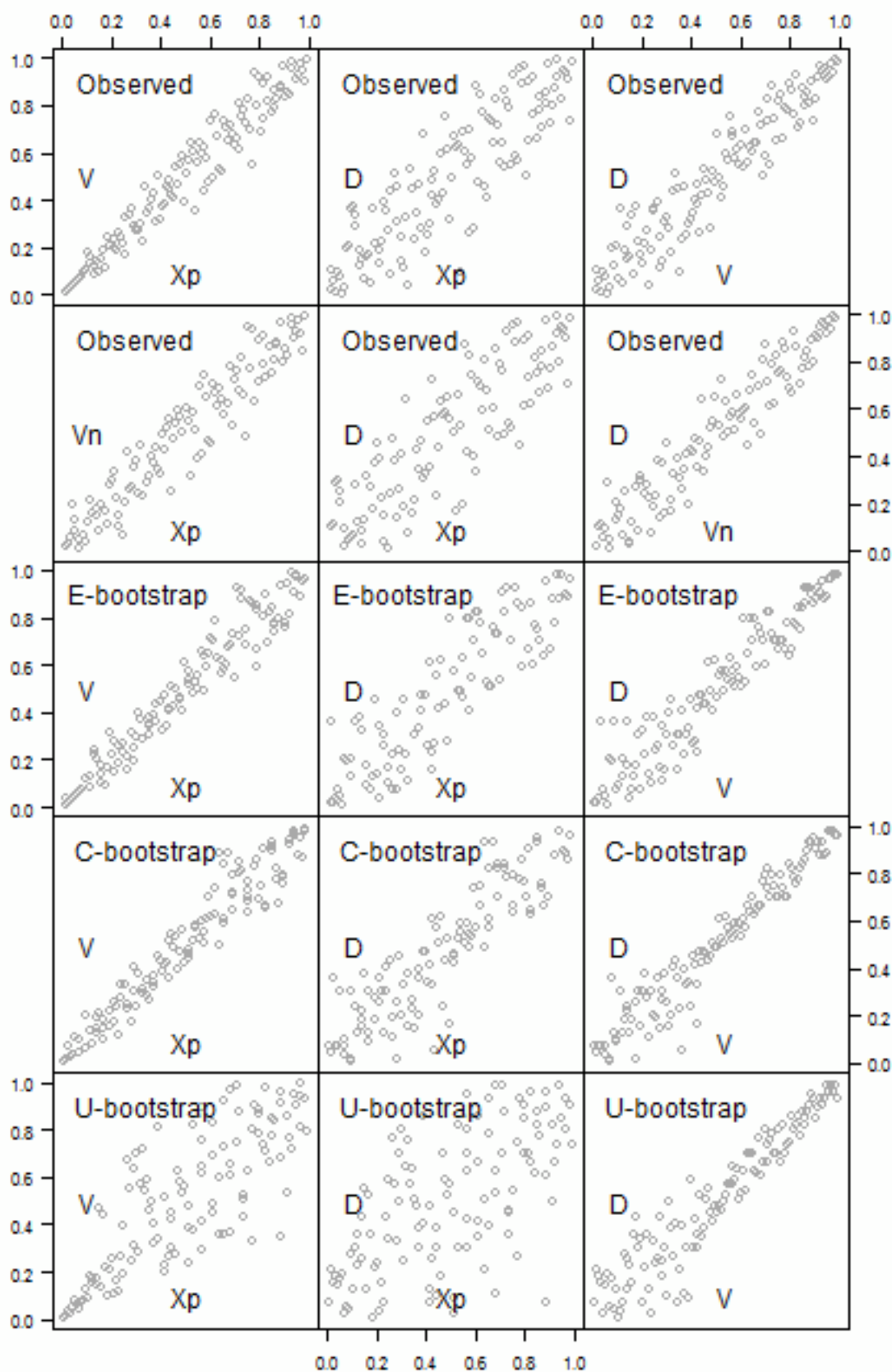

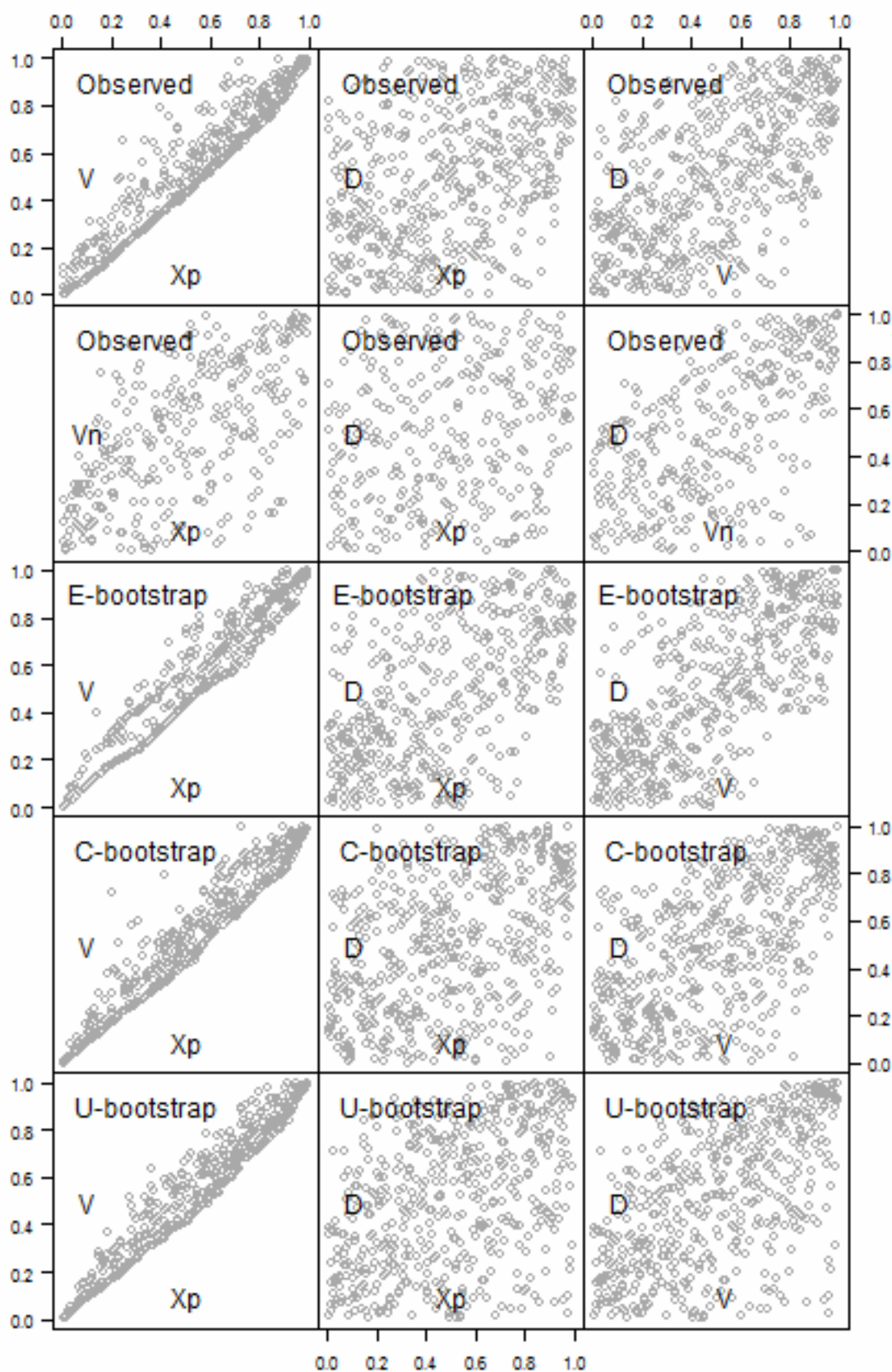

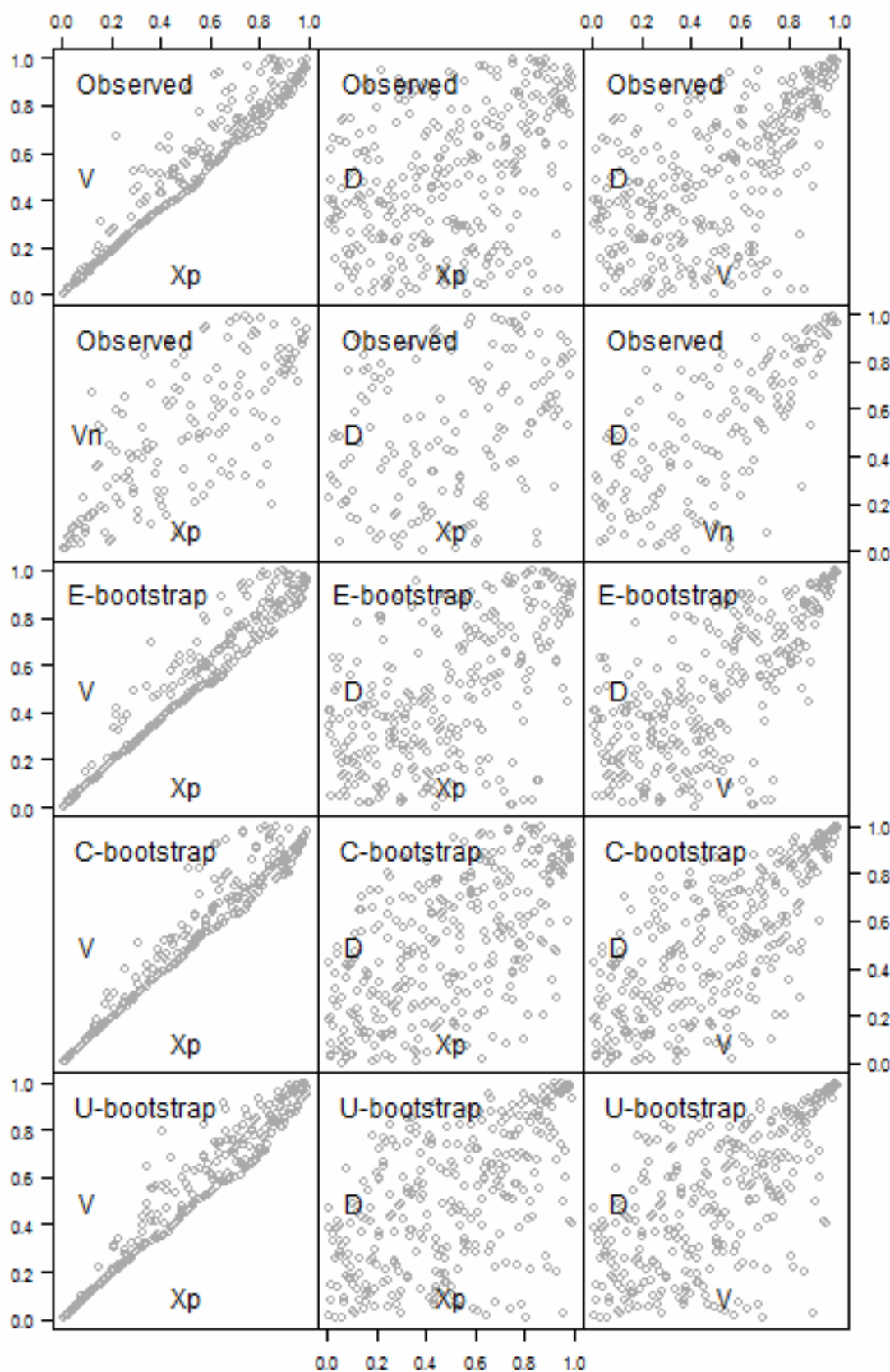

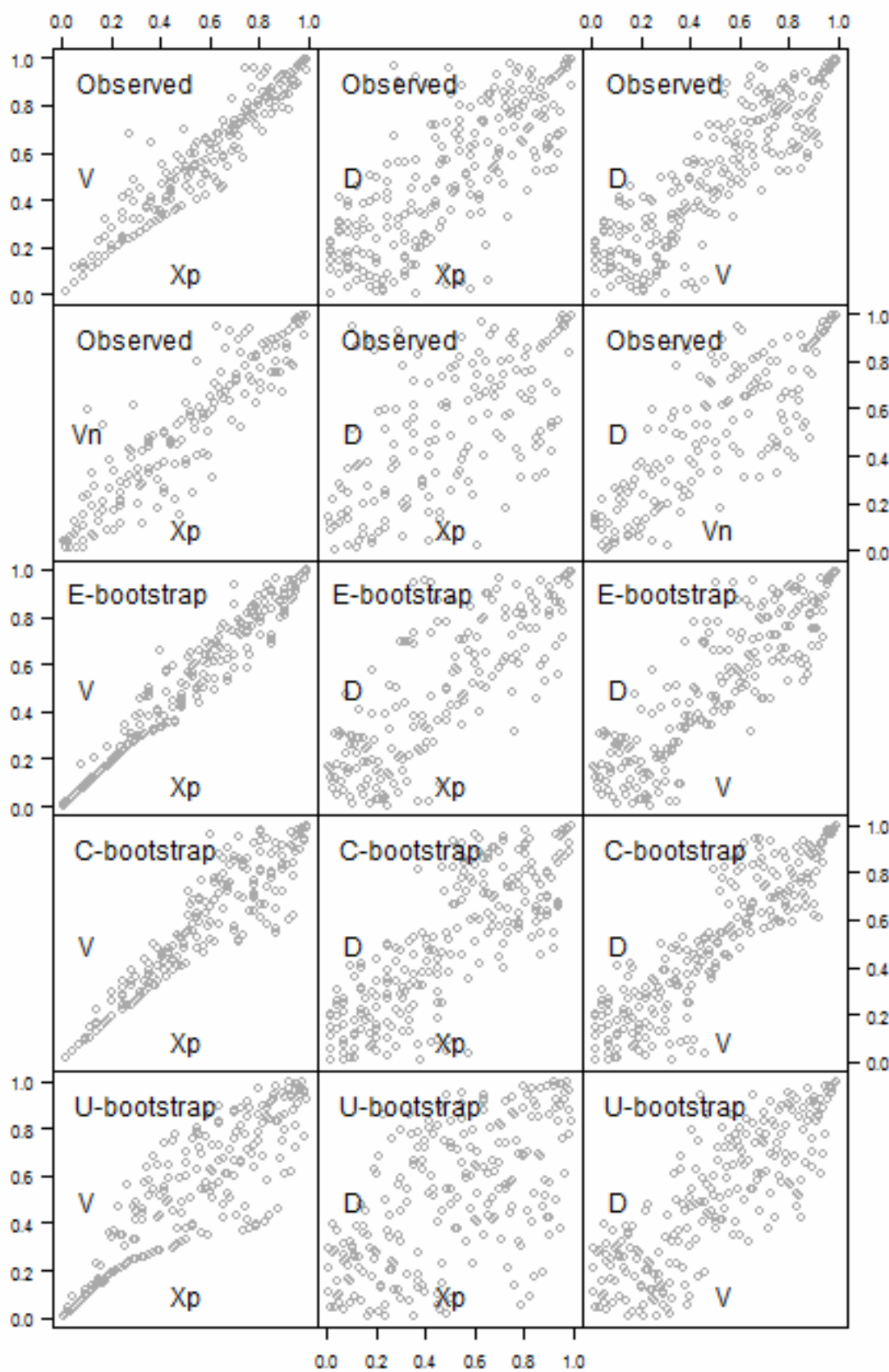

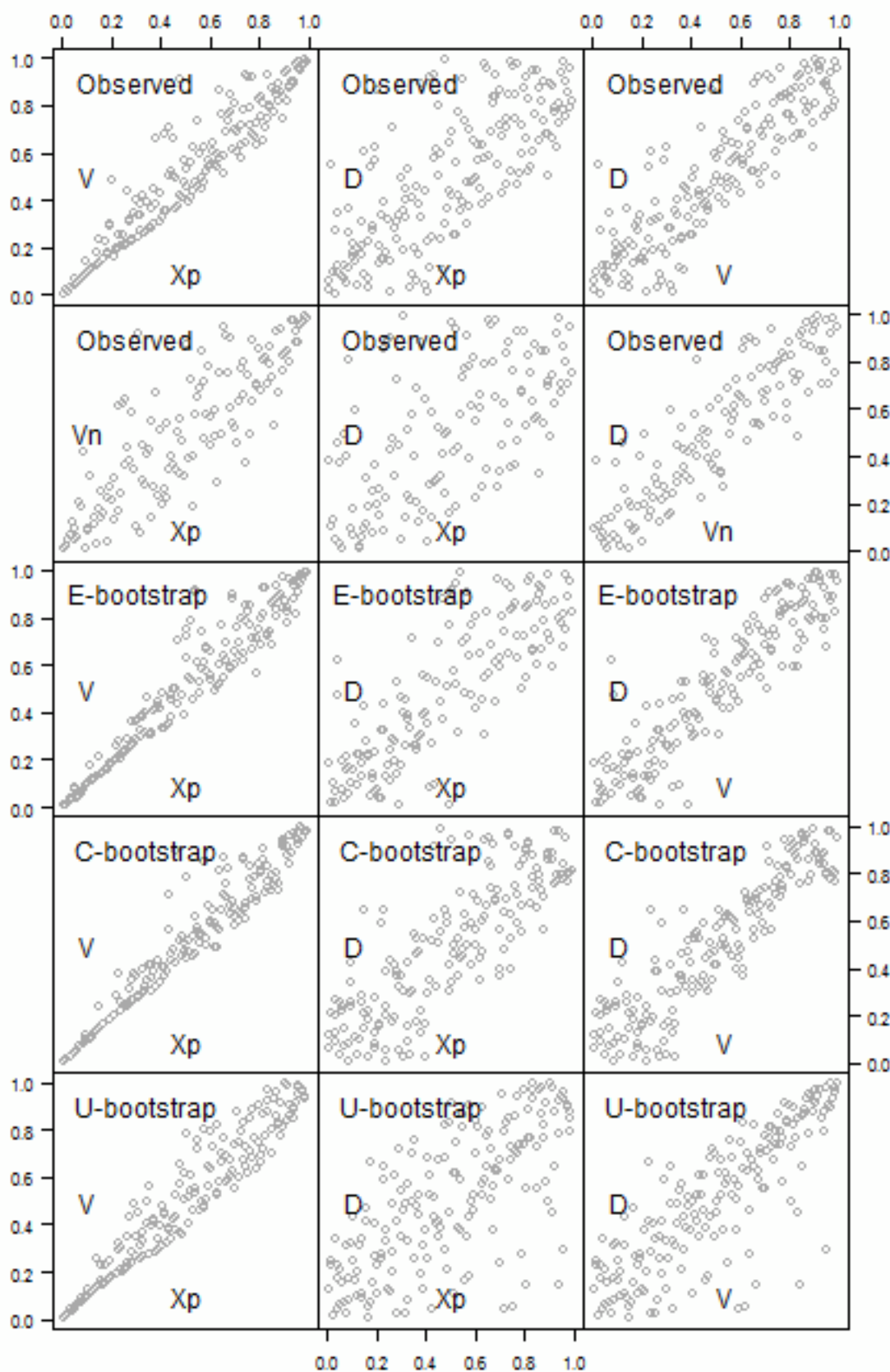

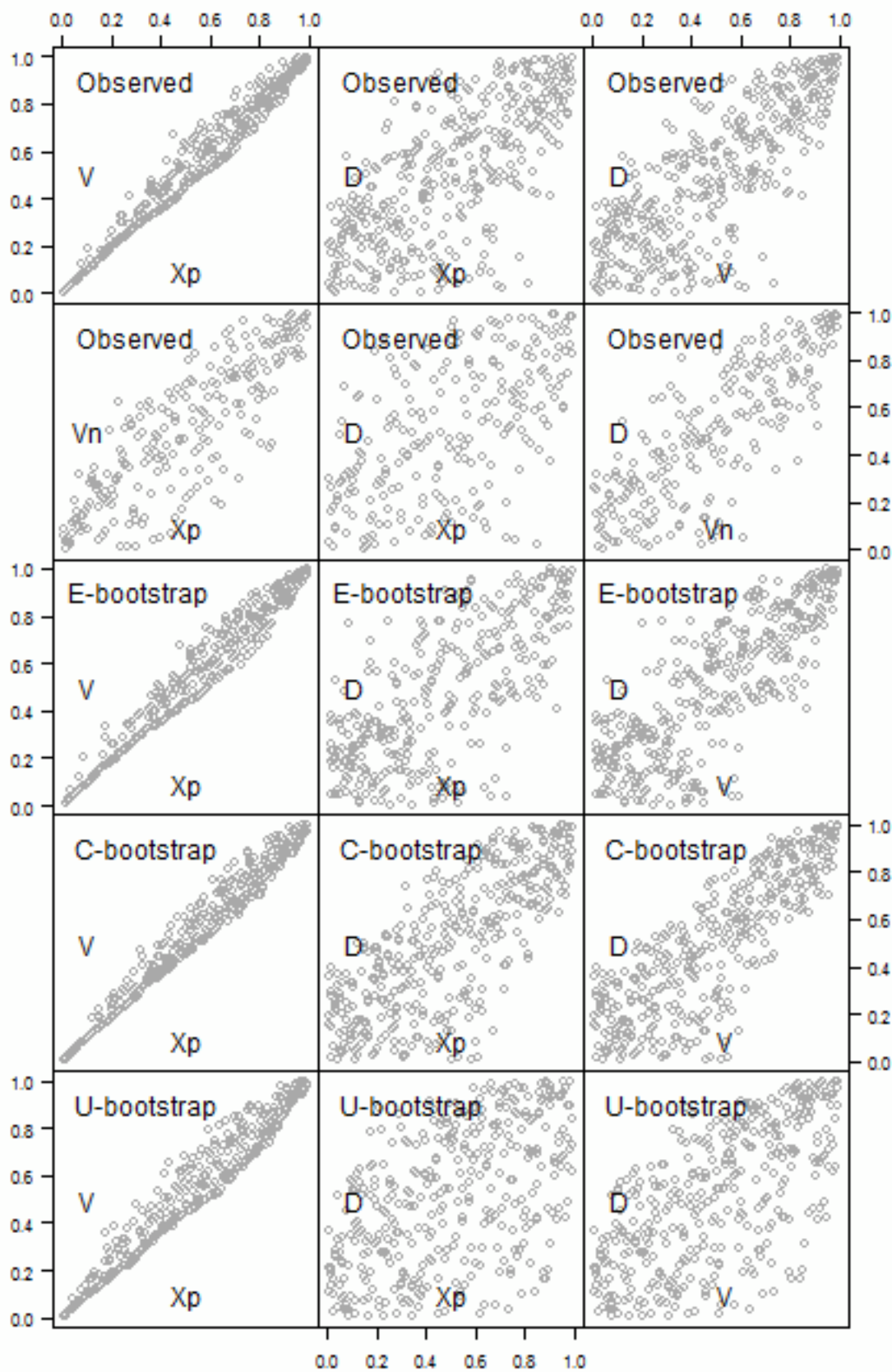

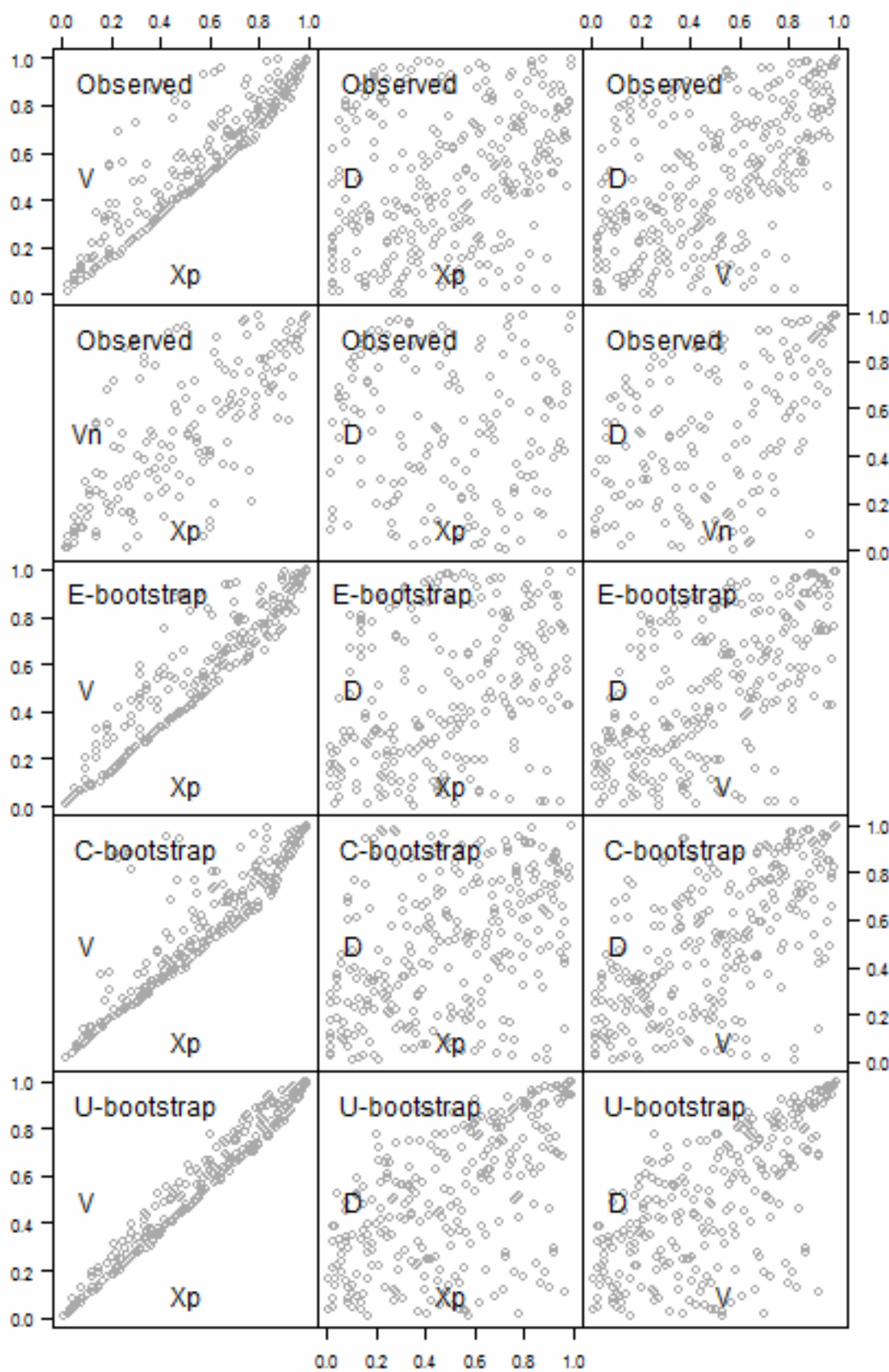

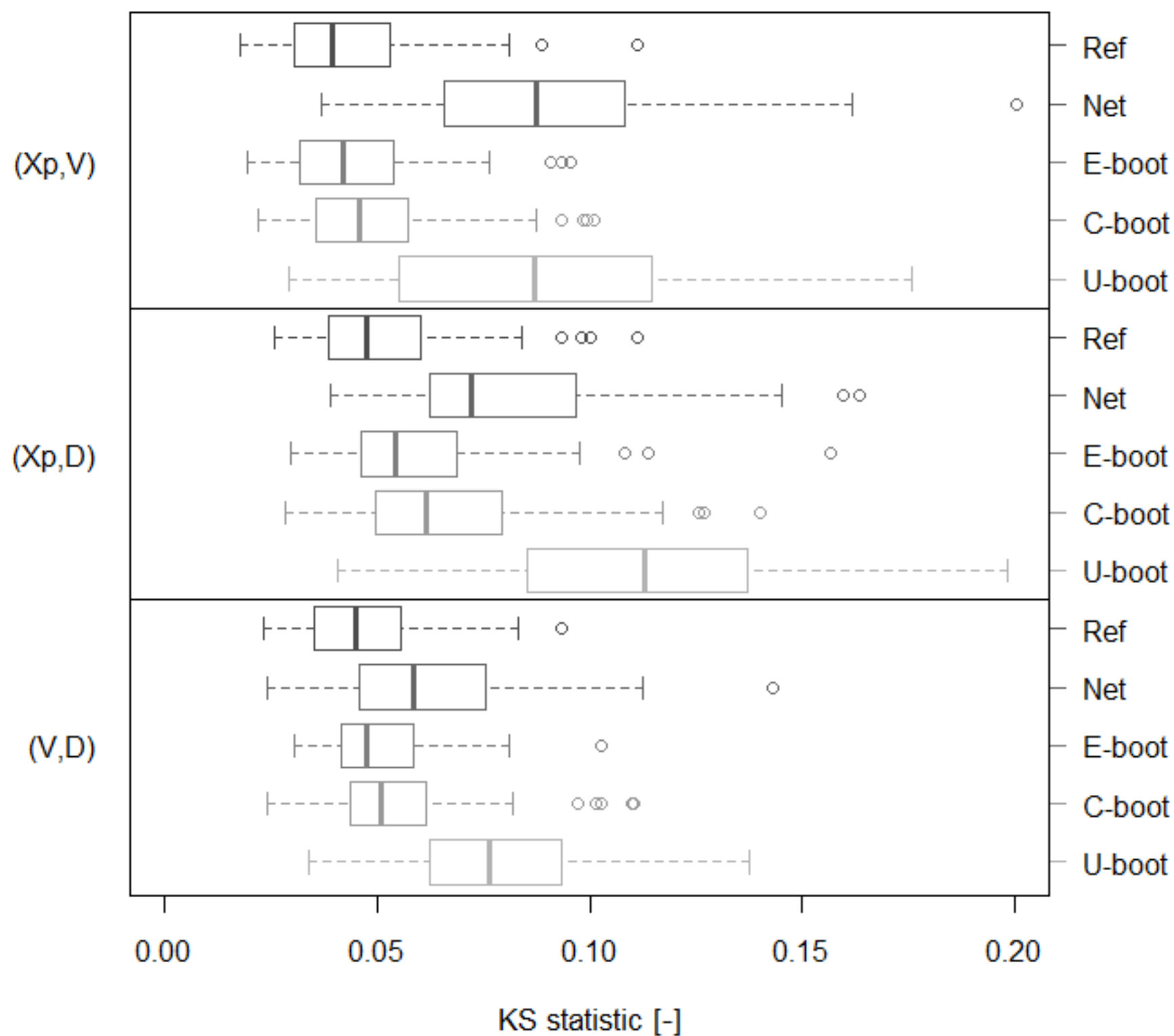

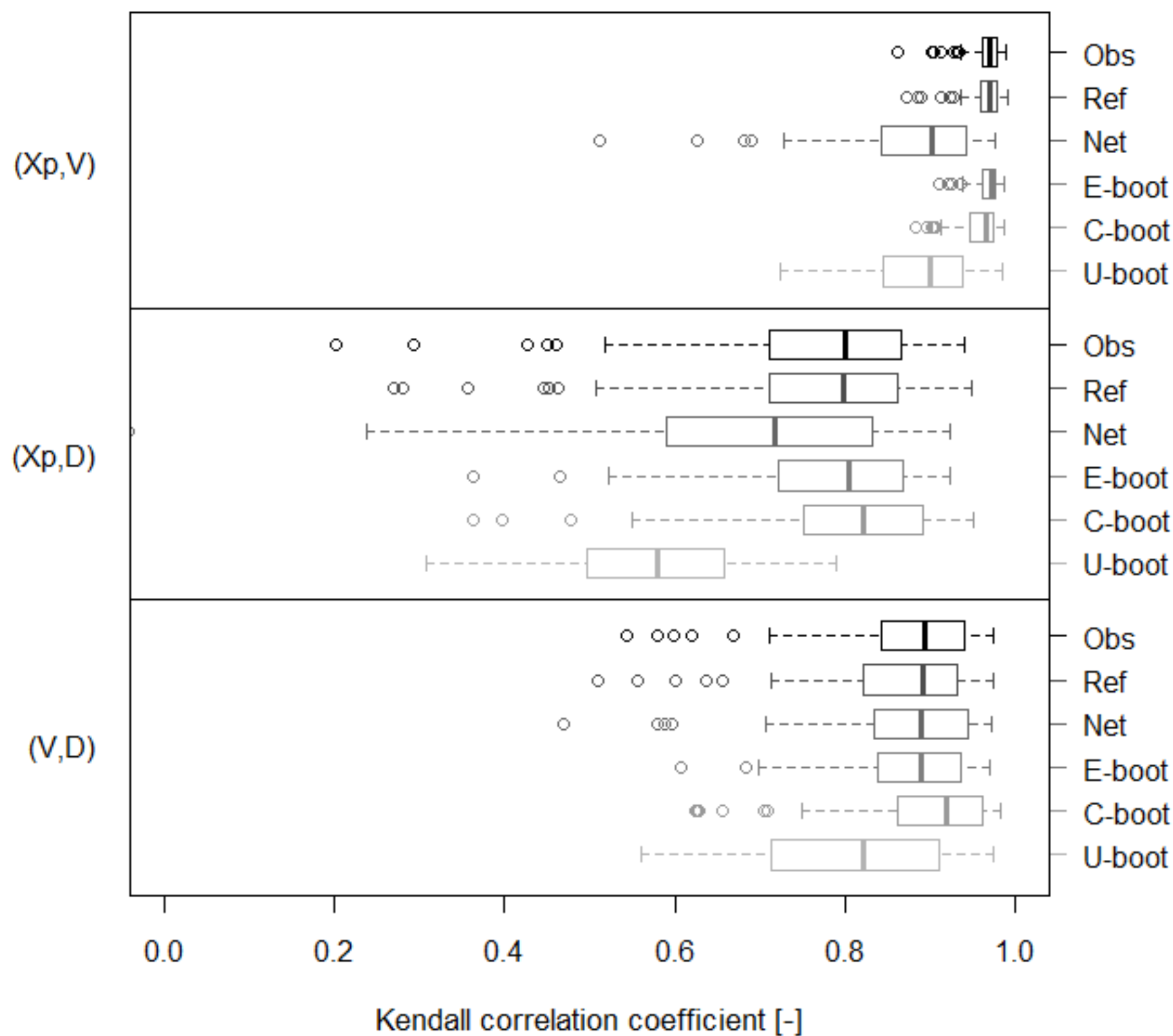

Supplement: Supplementary file 5 — Supplementary Figures [file wrcr0049-3423-sd5.pdf]
