# Supplementary material for: The intrinsic dependence structure of peak, volume, duration, and average intensity of hyetographs and hydrographs
Source: Water Resour Res. 2013 Jun 17;49(6):3423–42. doi: 10.1002/wrcr.20221 (PMC4303924; doi:10.1002/wrcr.20221)
Supplement: Supplementary file 2 — Supplementary Figures [file wrcr0049-3423-sd2.pdf]

ECA&D ID: 000011

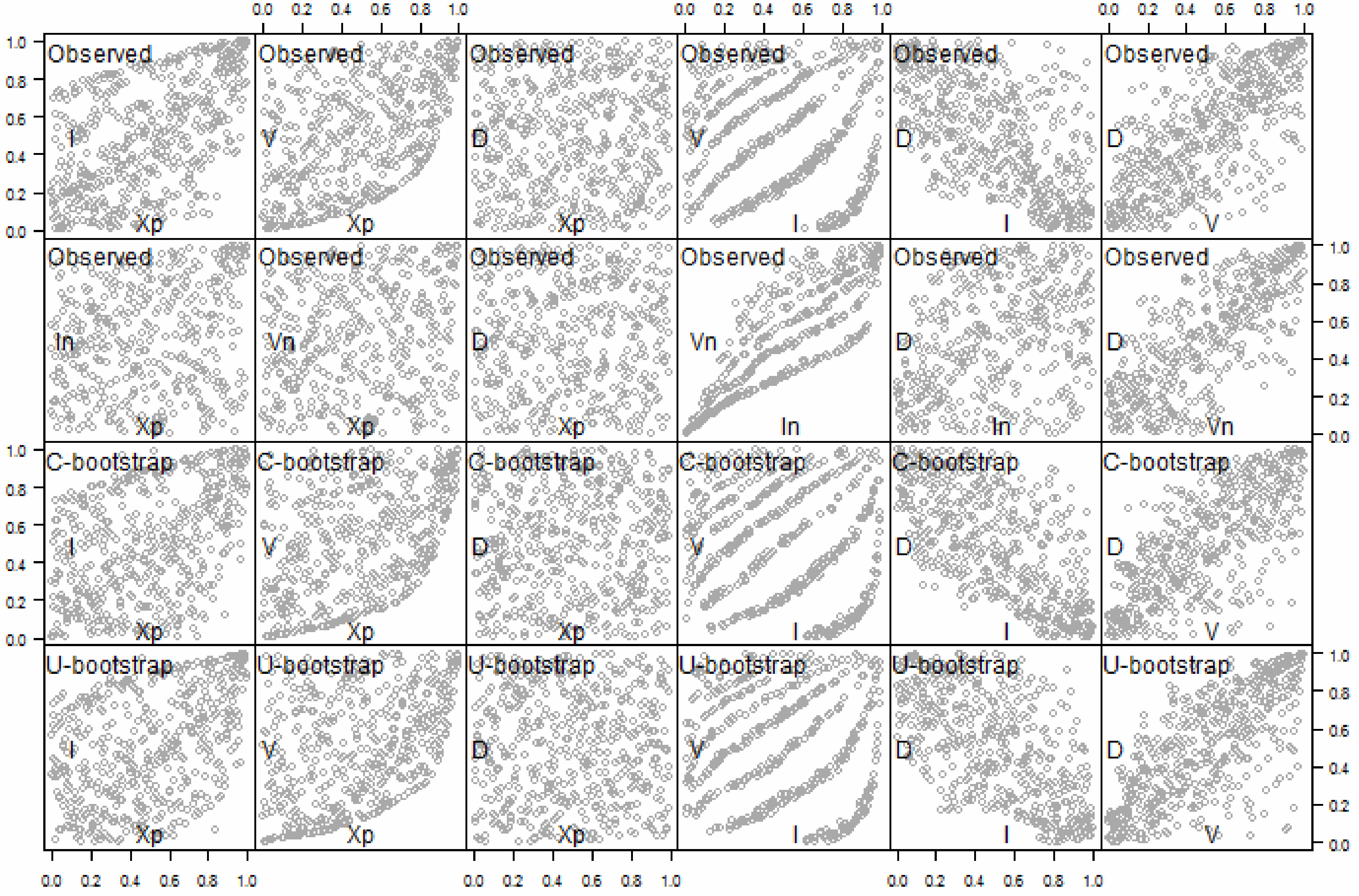

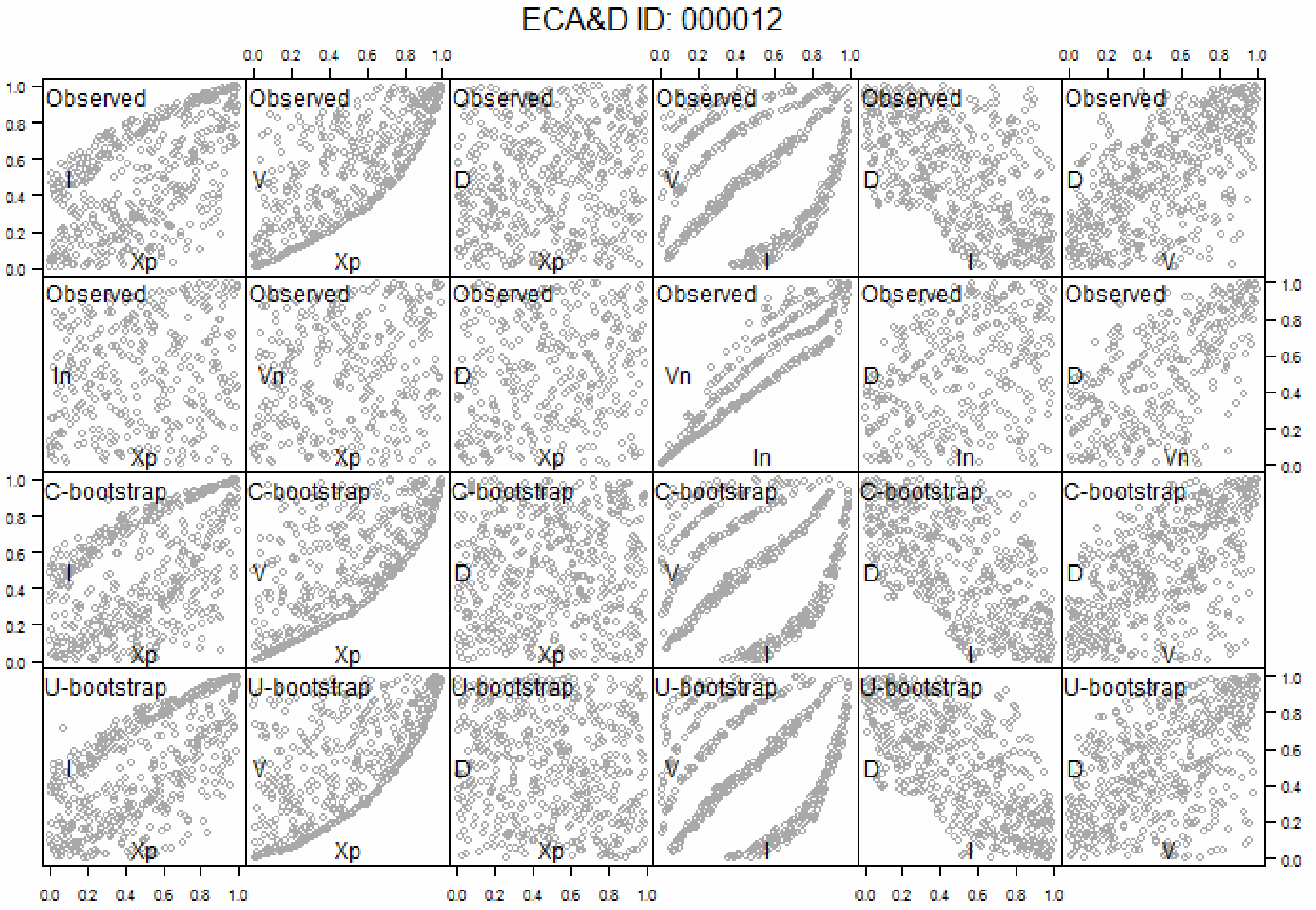
ECA&D ID: 000012
Observed
C-bootstrap
U-bootstrap
Xp
I
V
D
In
Vn
0.0
0.2
0.4
0.6
0.8
1.0

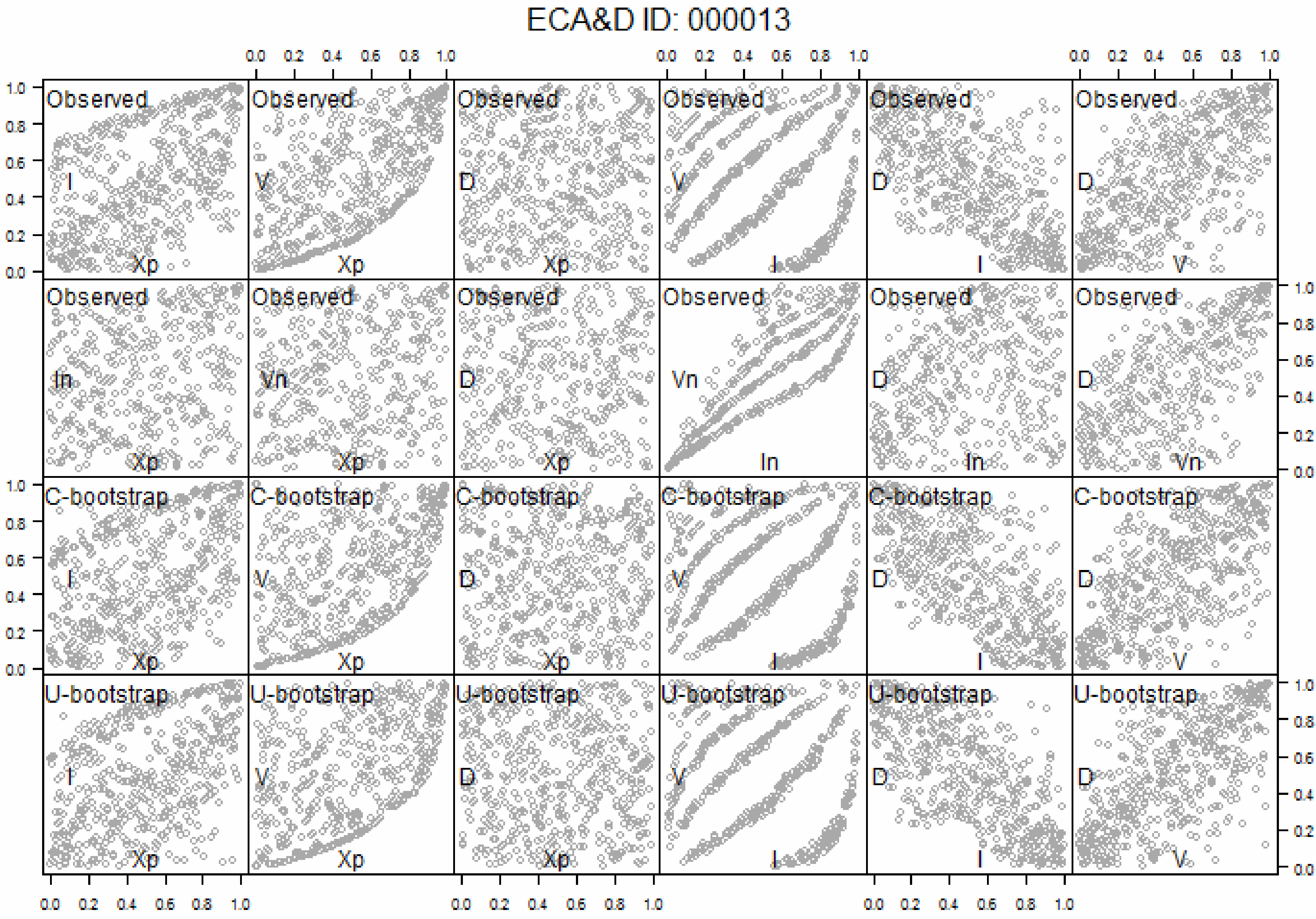
ECA&D ID: 000013
Observed
C-bootstrap
U-bootstrap
Xp
I
V
D
In
Vn
0.0
0.2
0.4
0.6
0.8
1.0

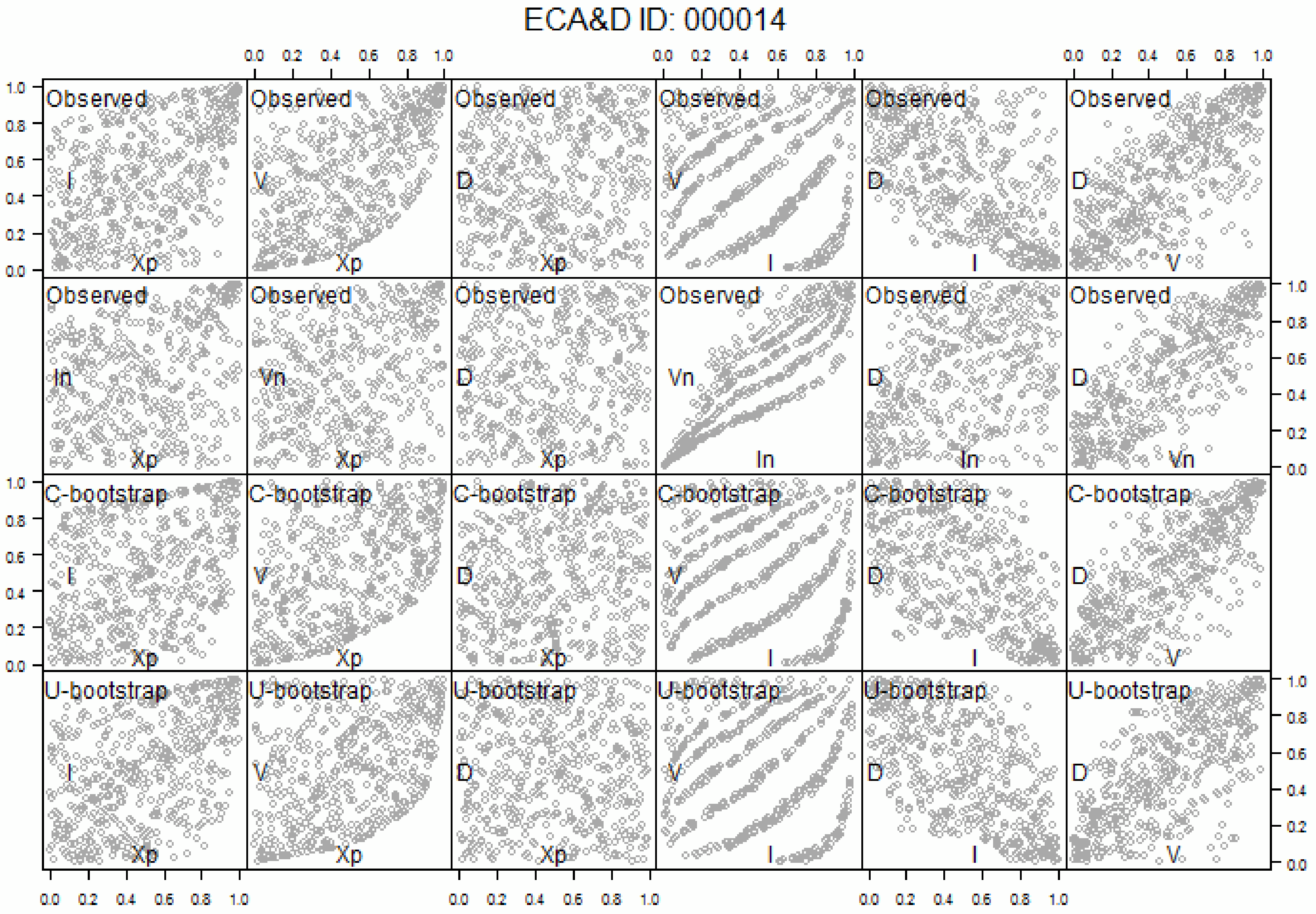
ECA&D ID: 000014
Observed
C-bootstrap
U-bootstrap
Xp
I
V
D
In
Vn
0.0
0.2
0.4
0.6
0.8
1.0

ECA&D ID: 000015

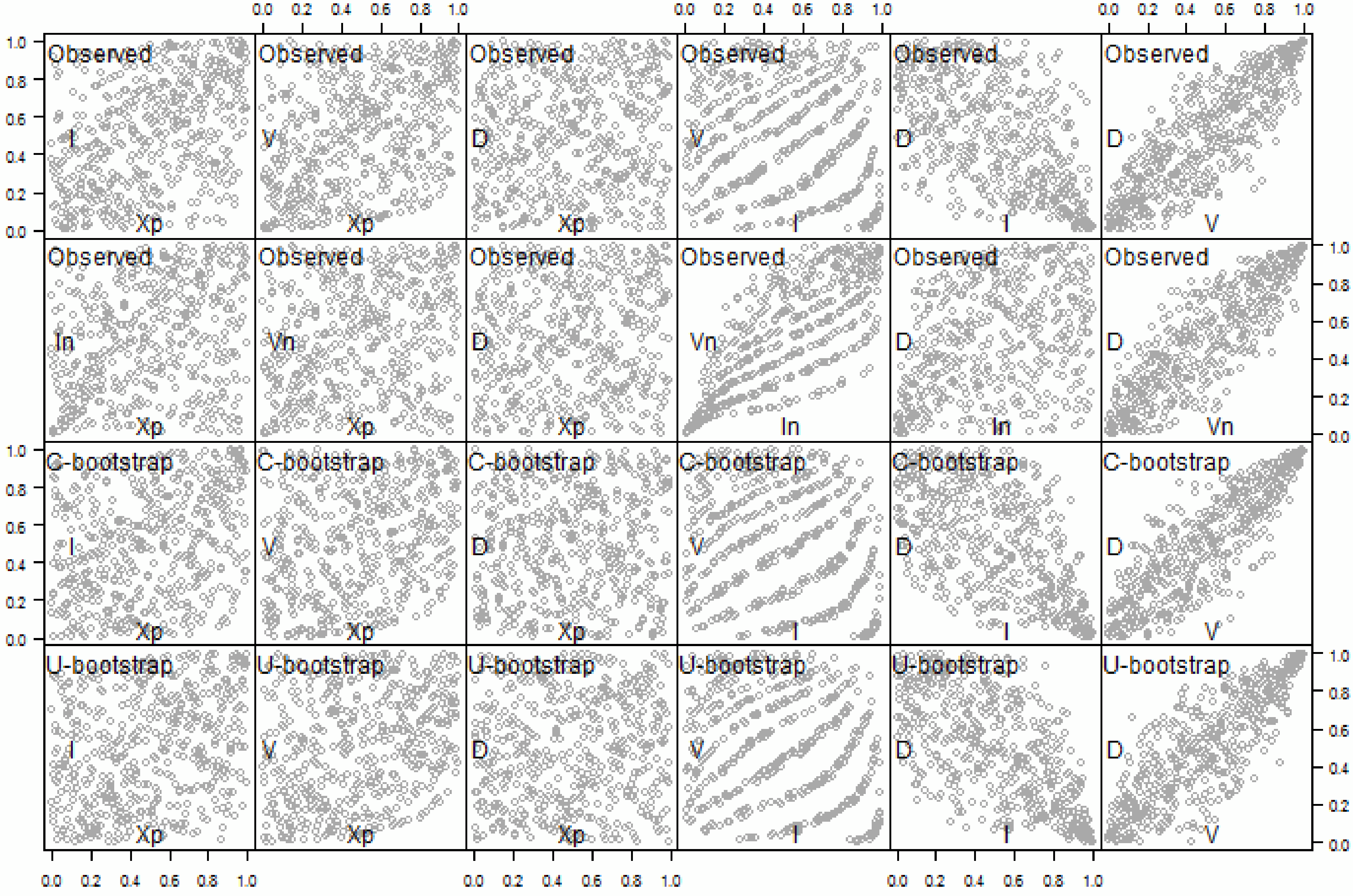

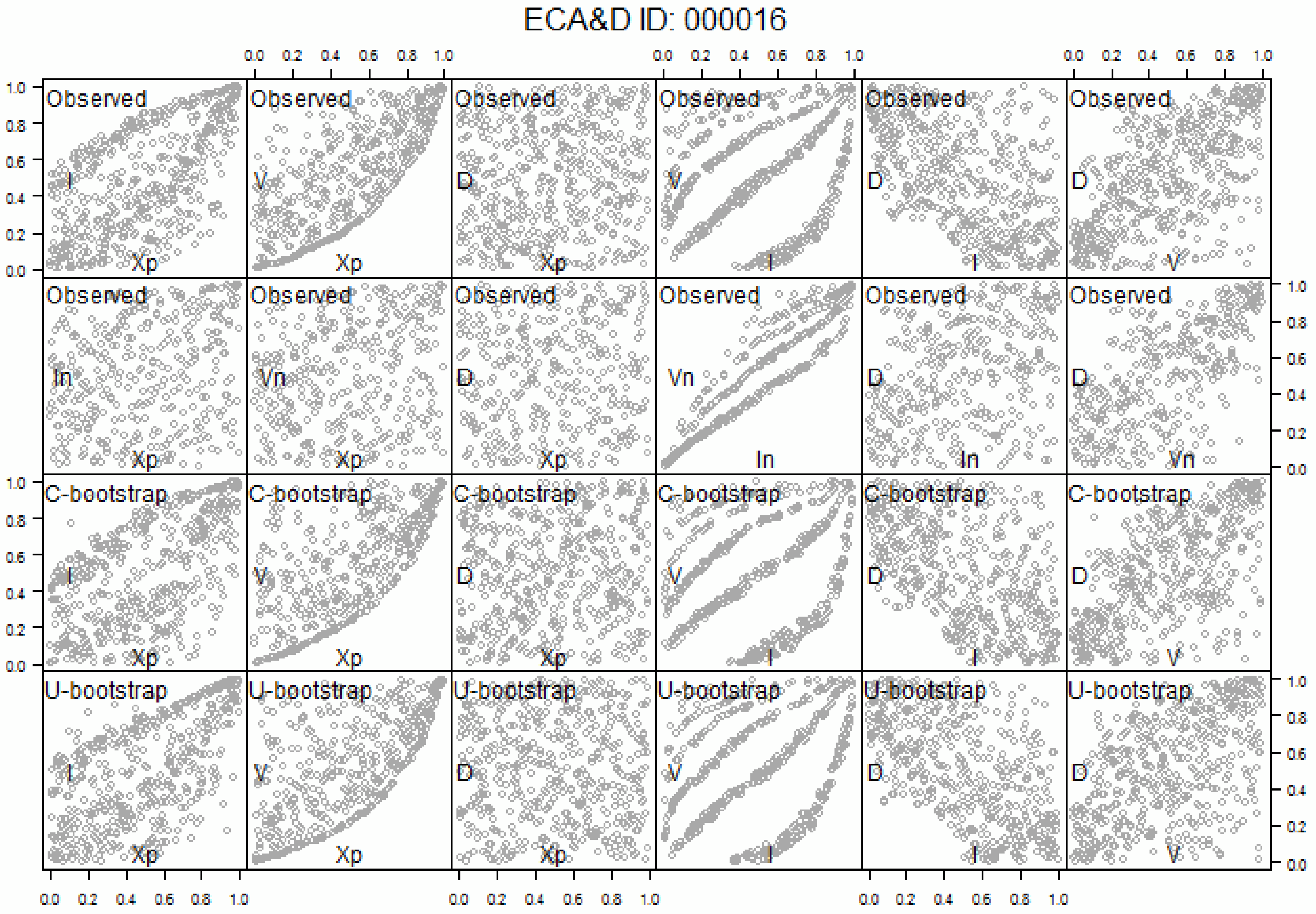
ECA&D ID: 000016
Observed
C-bootstrap
U-bootstrap
Xp
I
V
D
In
Vn
0.0
0.2
0.4
0.6
0.8
1.0

ECA&D ID: 000021

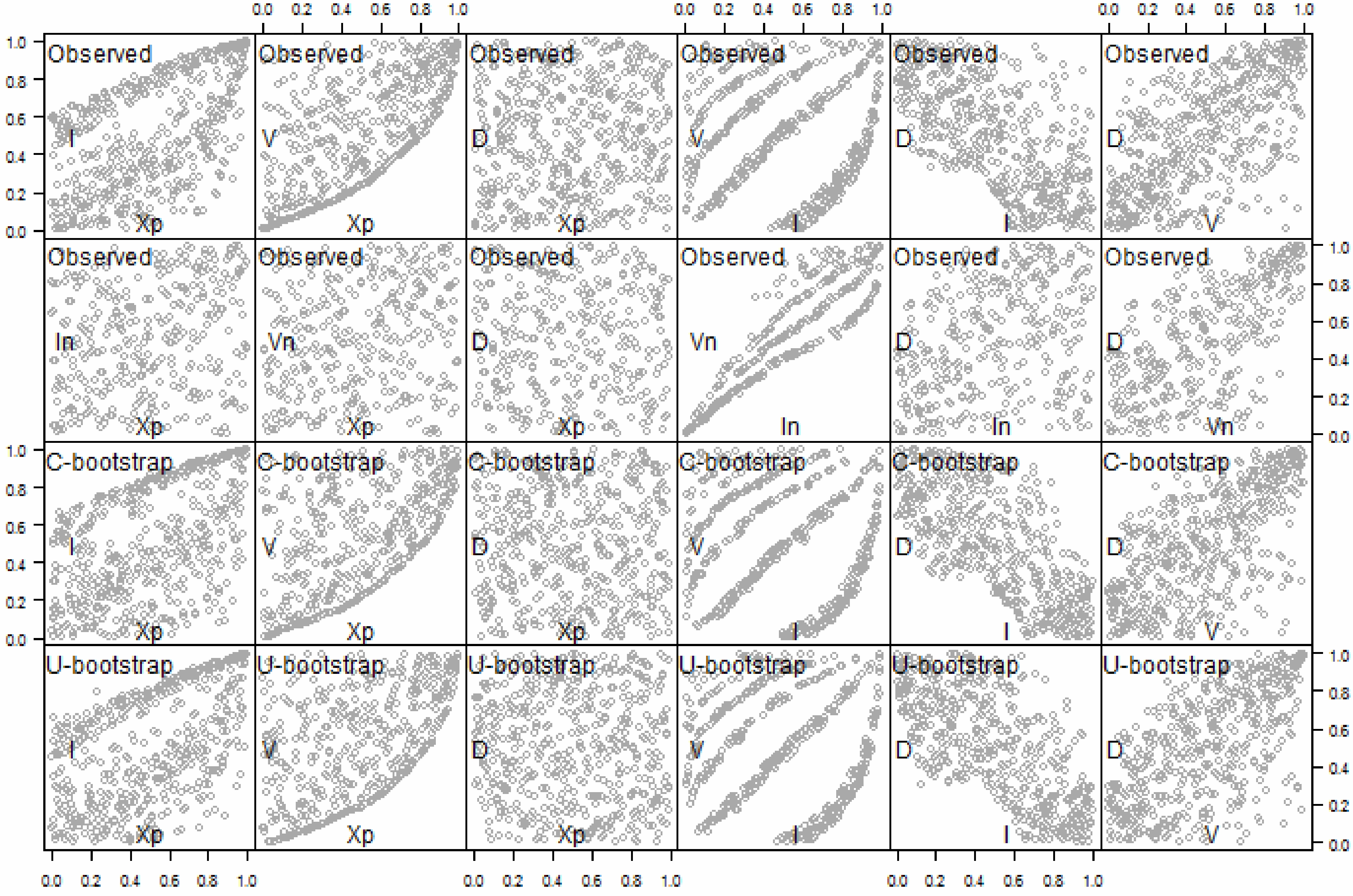

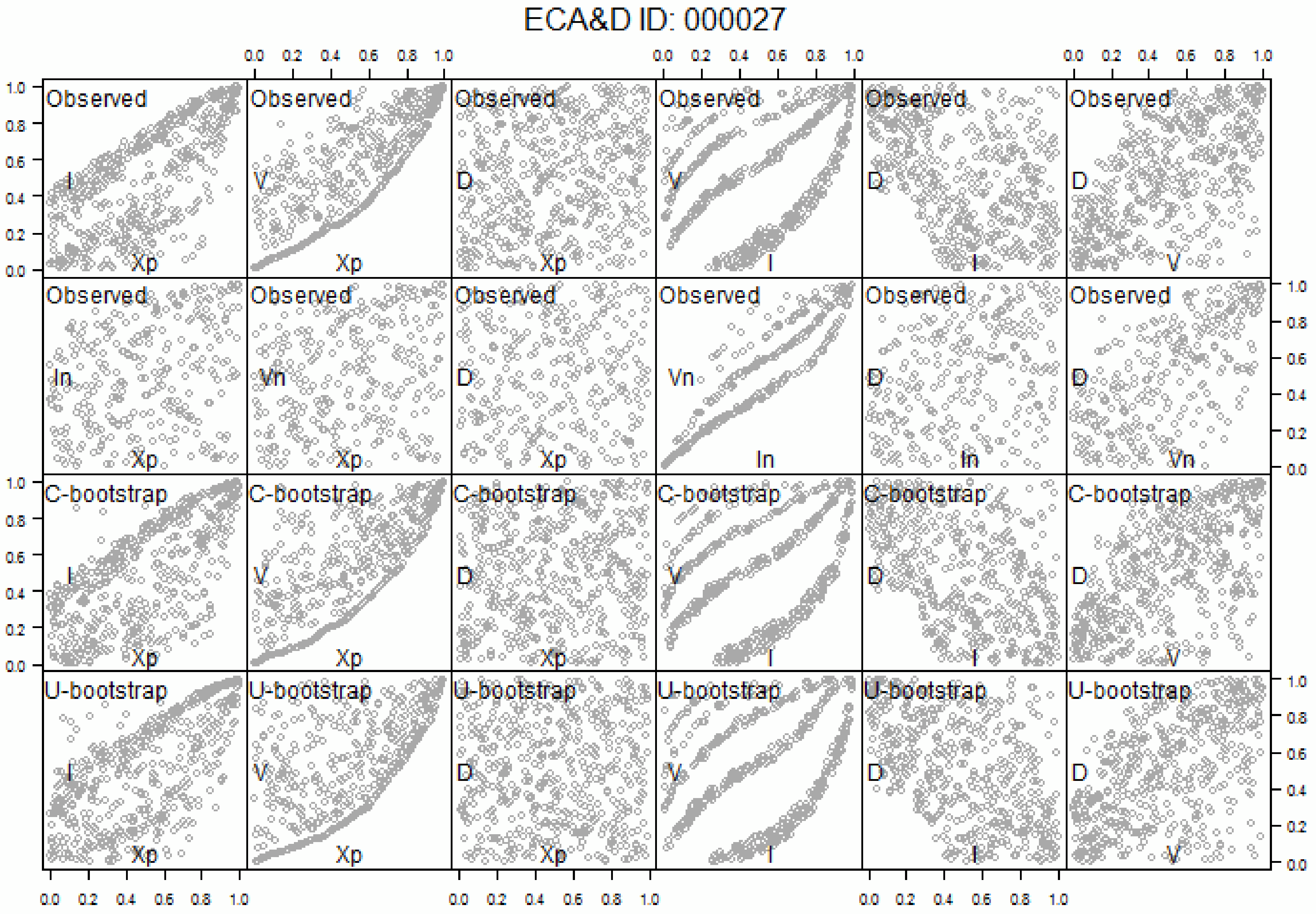
ECA&D ID: 000027
Observed
C-bootstrap
U-bootstrap
Xp
I
V
D
In
Vn
0.0
0.2
0.4
0.6
0.8
1.0

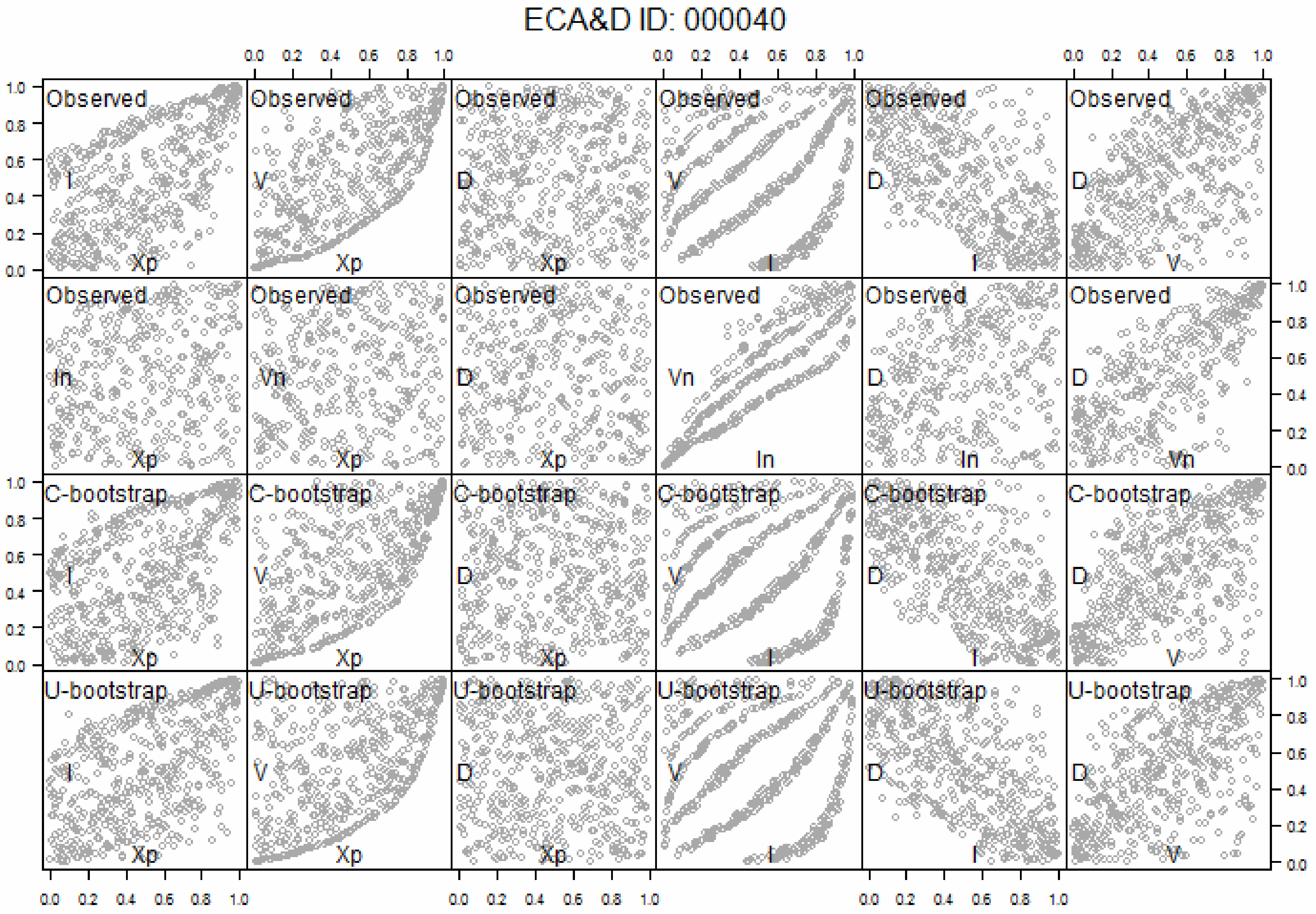
ECA&D ID: 000040
Observed
C-bootstrap
U-bootstrap
Xp
I
V
D
In
Vn
0.0
0.2
0.4
0.6
0.8
1.0

ECA&D ID: 000041

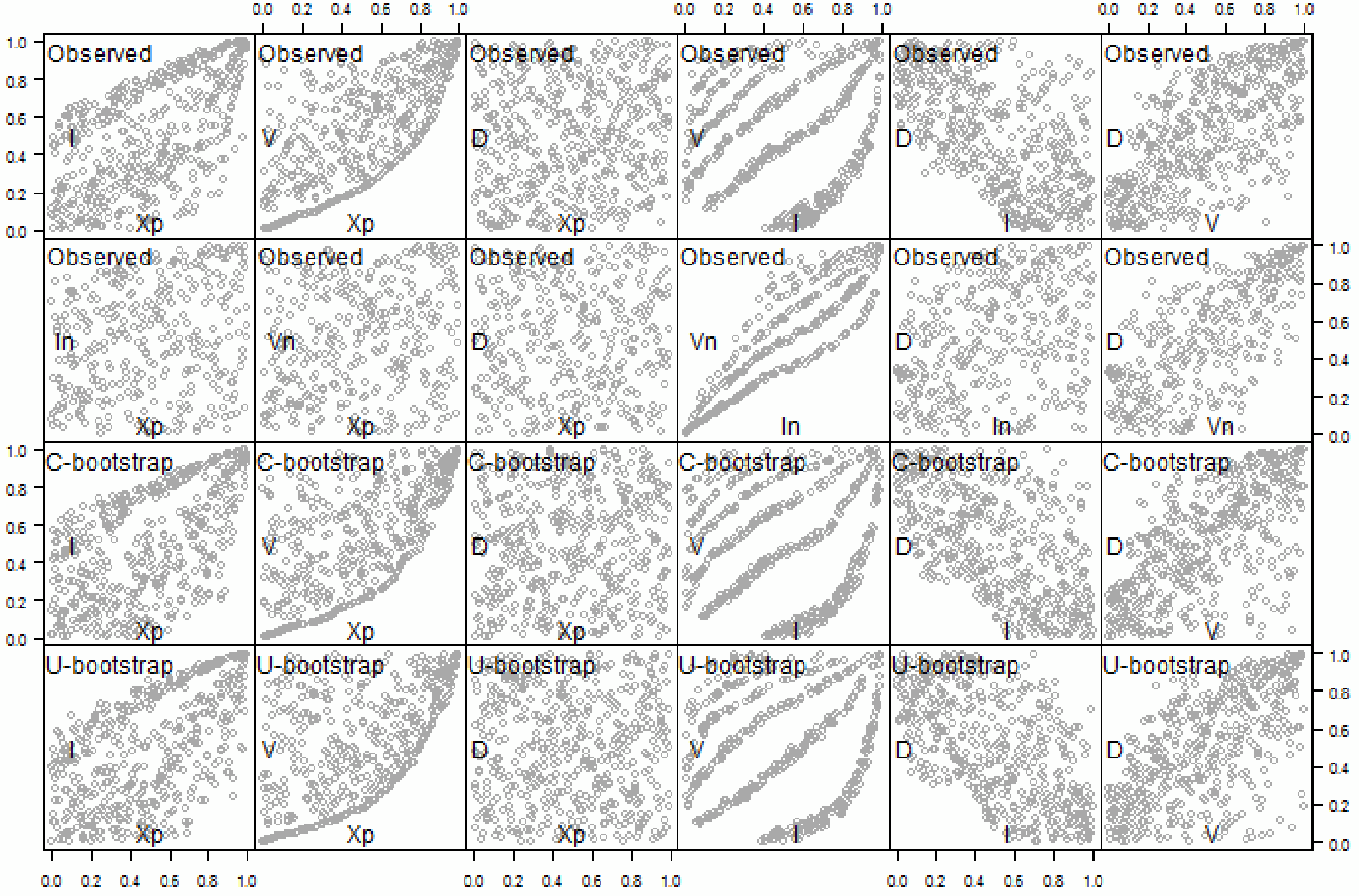

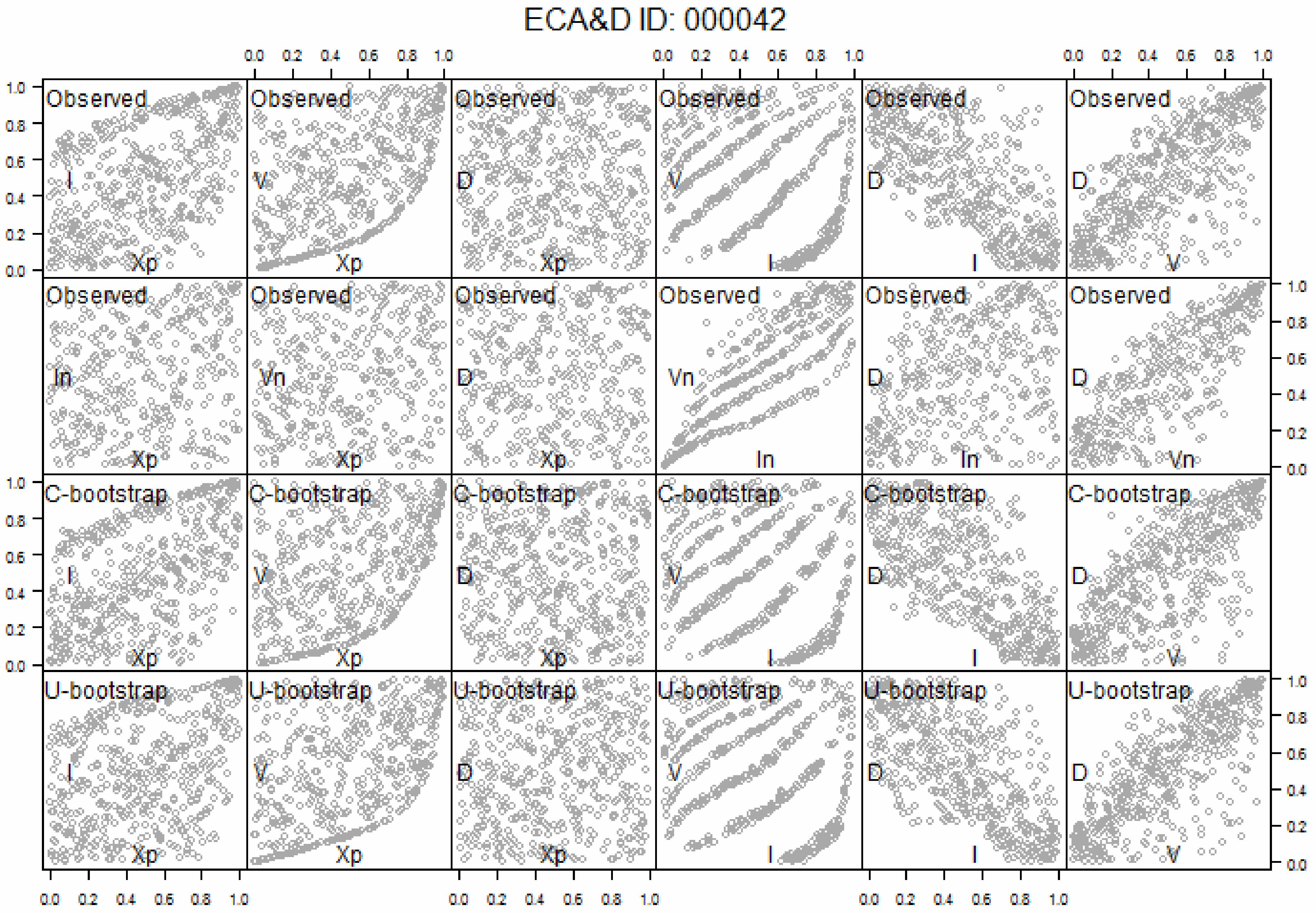
ECA&D ID: 000042
Observed
C-bootstrap
U-bootstrap
Xp
I
V
D
In
Vn
0.0
0.2
0.4
0.6
0.8
1.0

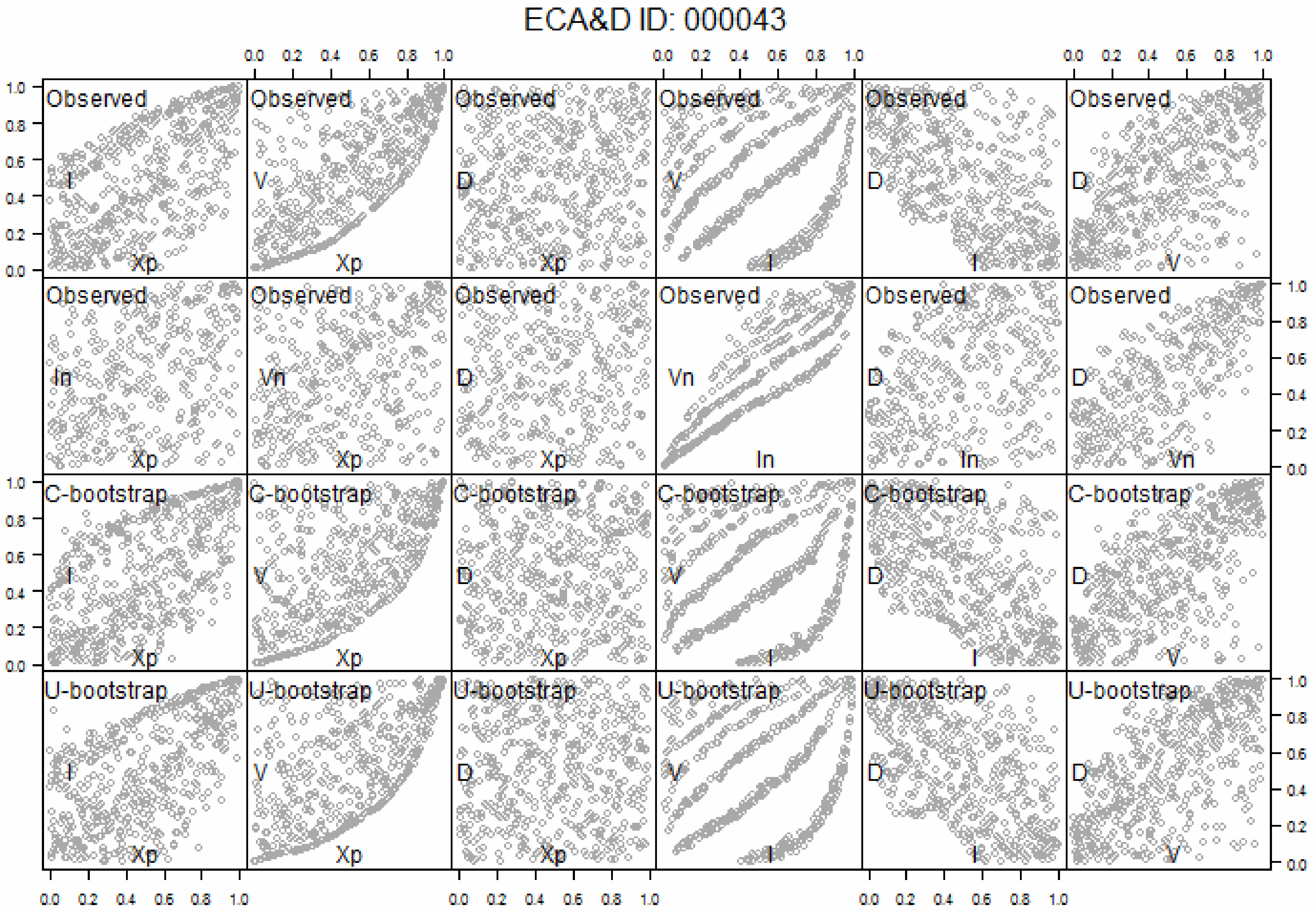
ECA&D ID: 000043
Observed
C-bootstrap
U-bootstrap
Xp
I
V
D
In
Vn
0.0
0.2
0.4
0.6
0.8
1.0

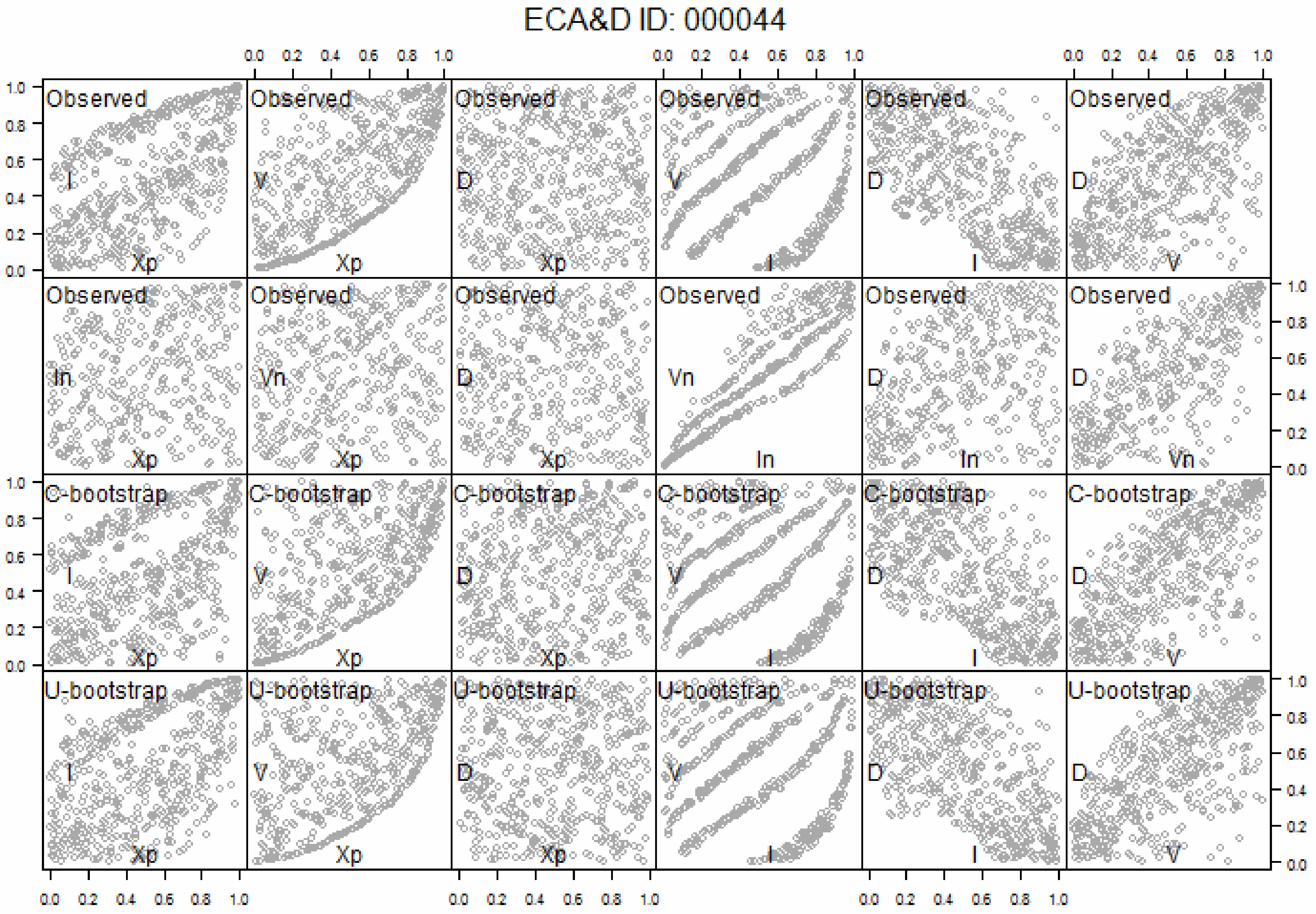
ECA&D ID: 000044
Observed
C-bootstrap
U-bootstrap
Xp
I
V
D
In
Vn
0.0
0.2
0.4
0.6
0.8
1.0

ECA&D ID: 000045

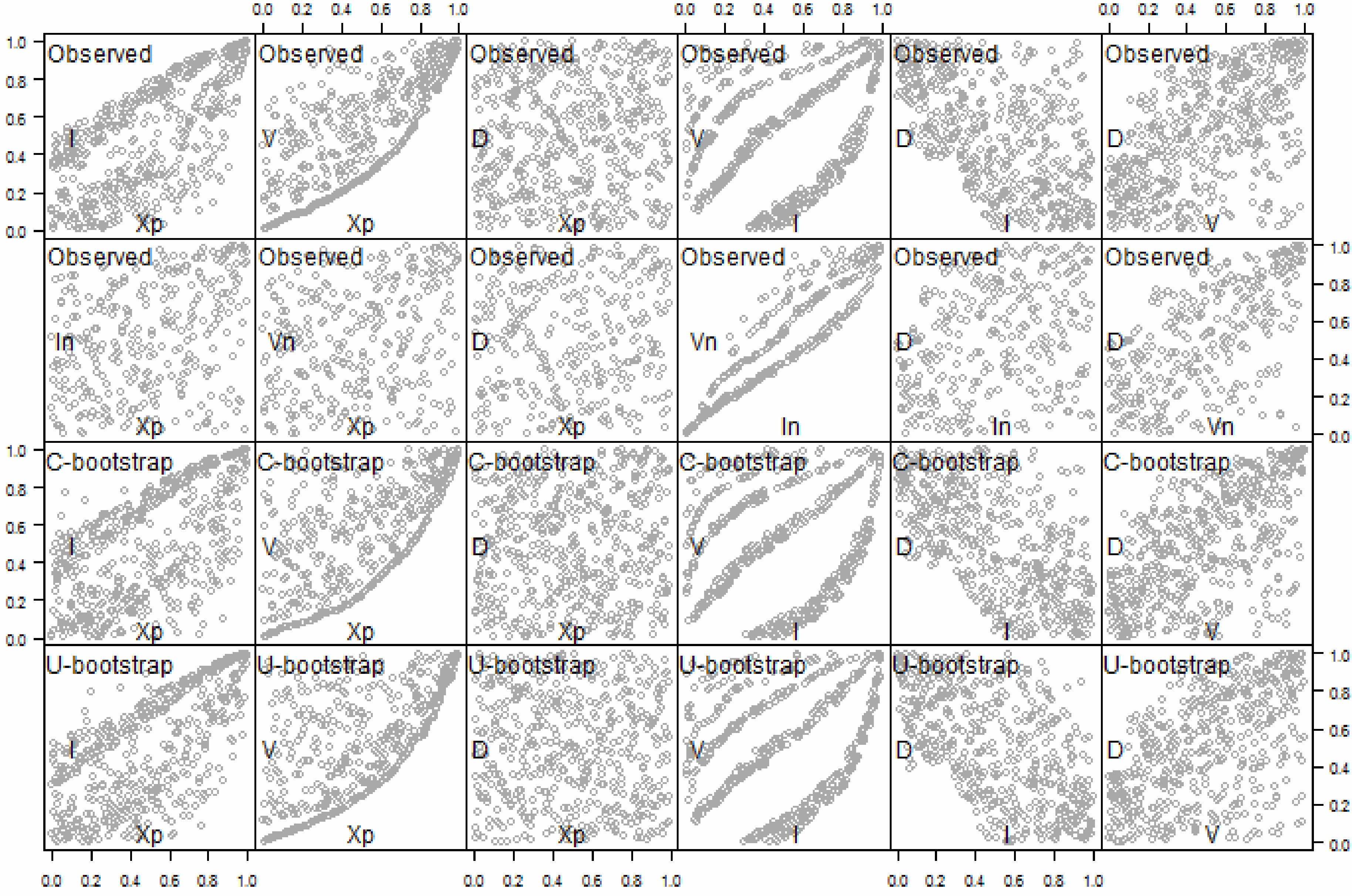

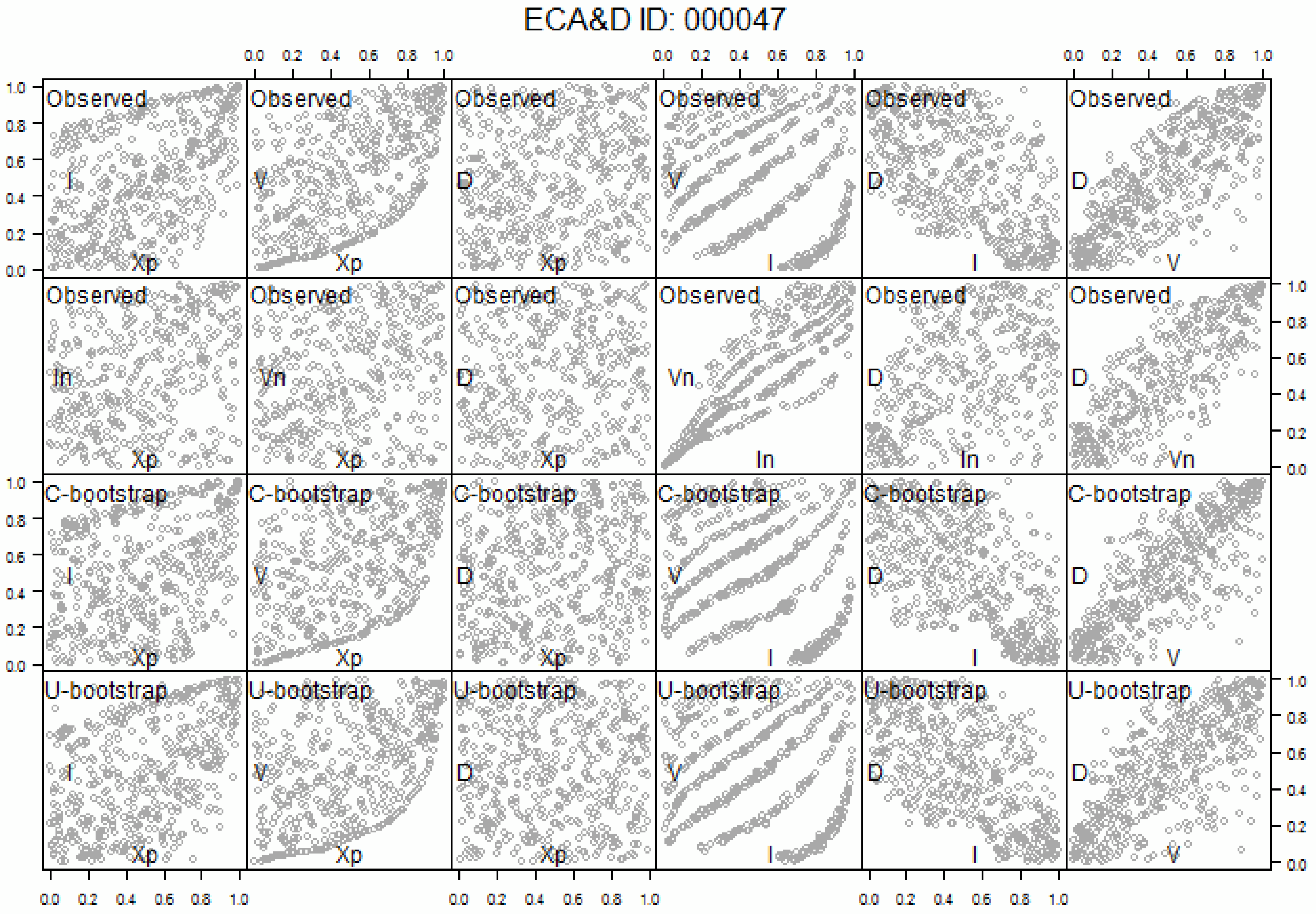
ECA&D ID: 000047
Observed
C-bootstrap
U-bootstrap
Xp
I
V
D
In
Vn
0.0
0.2
0.4
0.6
0.8
1.0

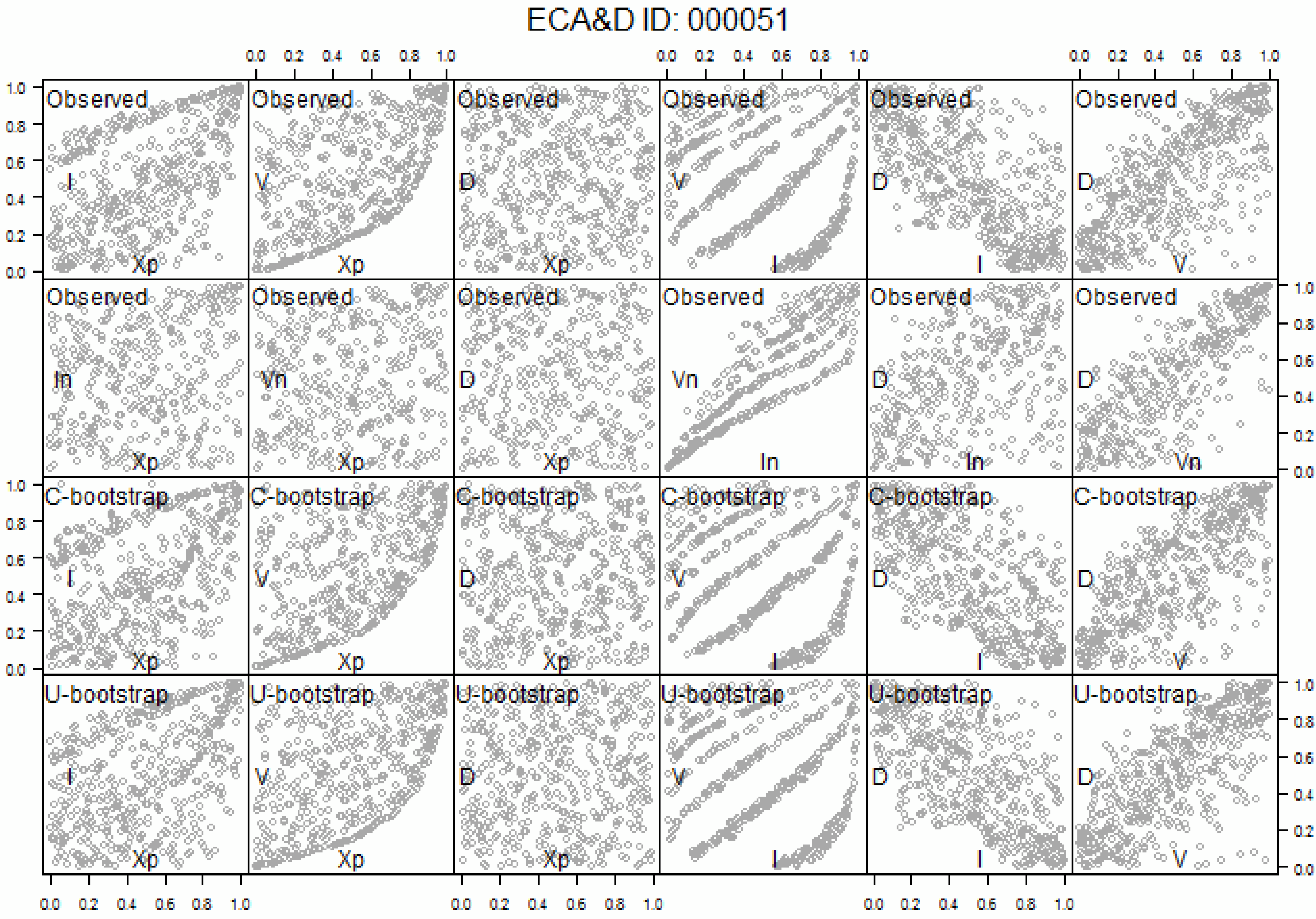
ECA&D ID: 000051
Observed
C-bootstrap
U-bootstrap
Xp
I
V
D
In
Vn
0.0
0.2
0.4
0.6
0.8
1.0

ECA&D ID: 000052

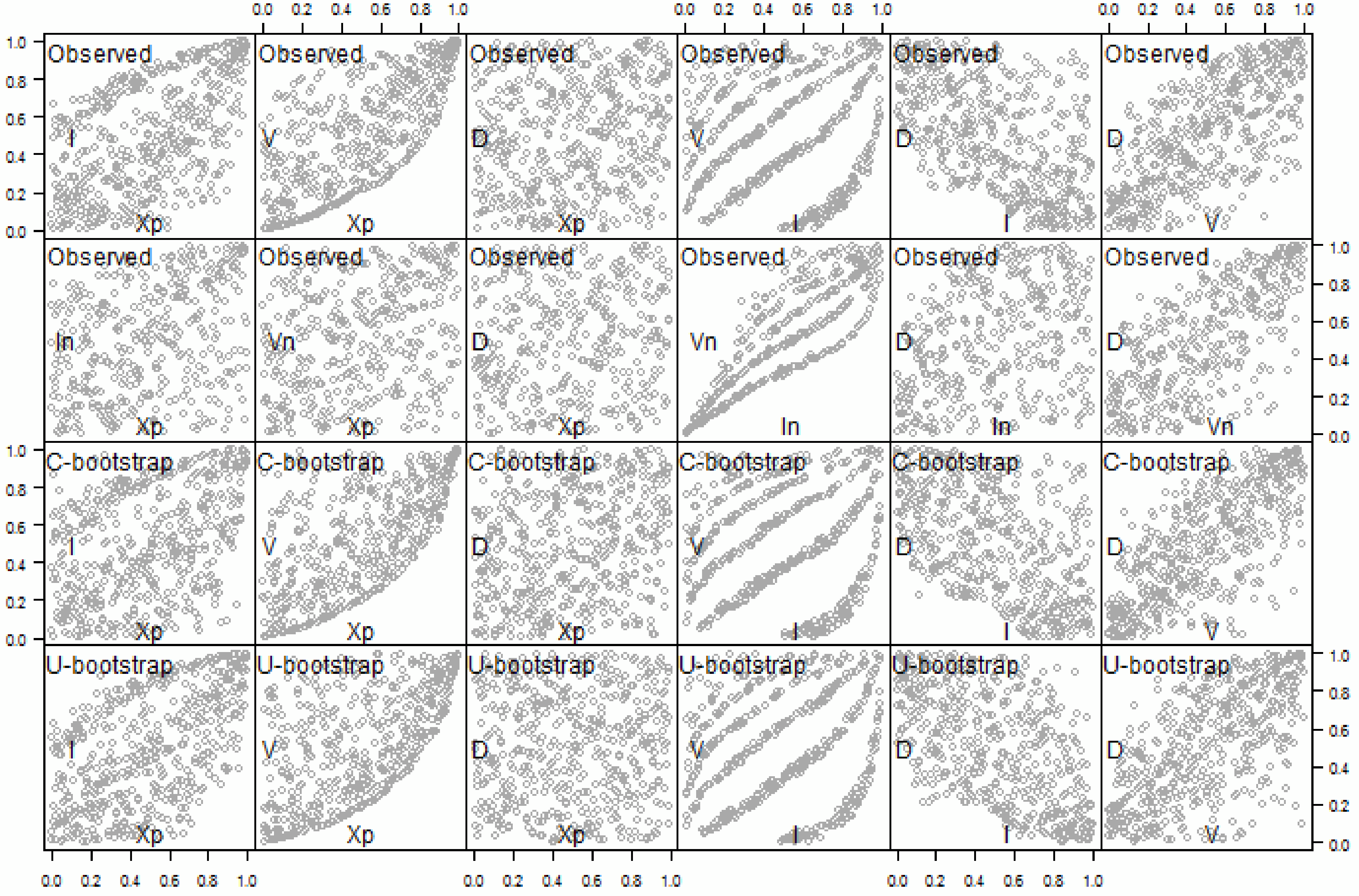

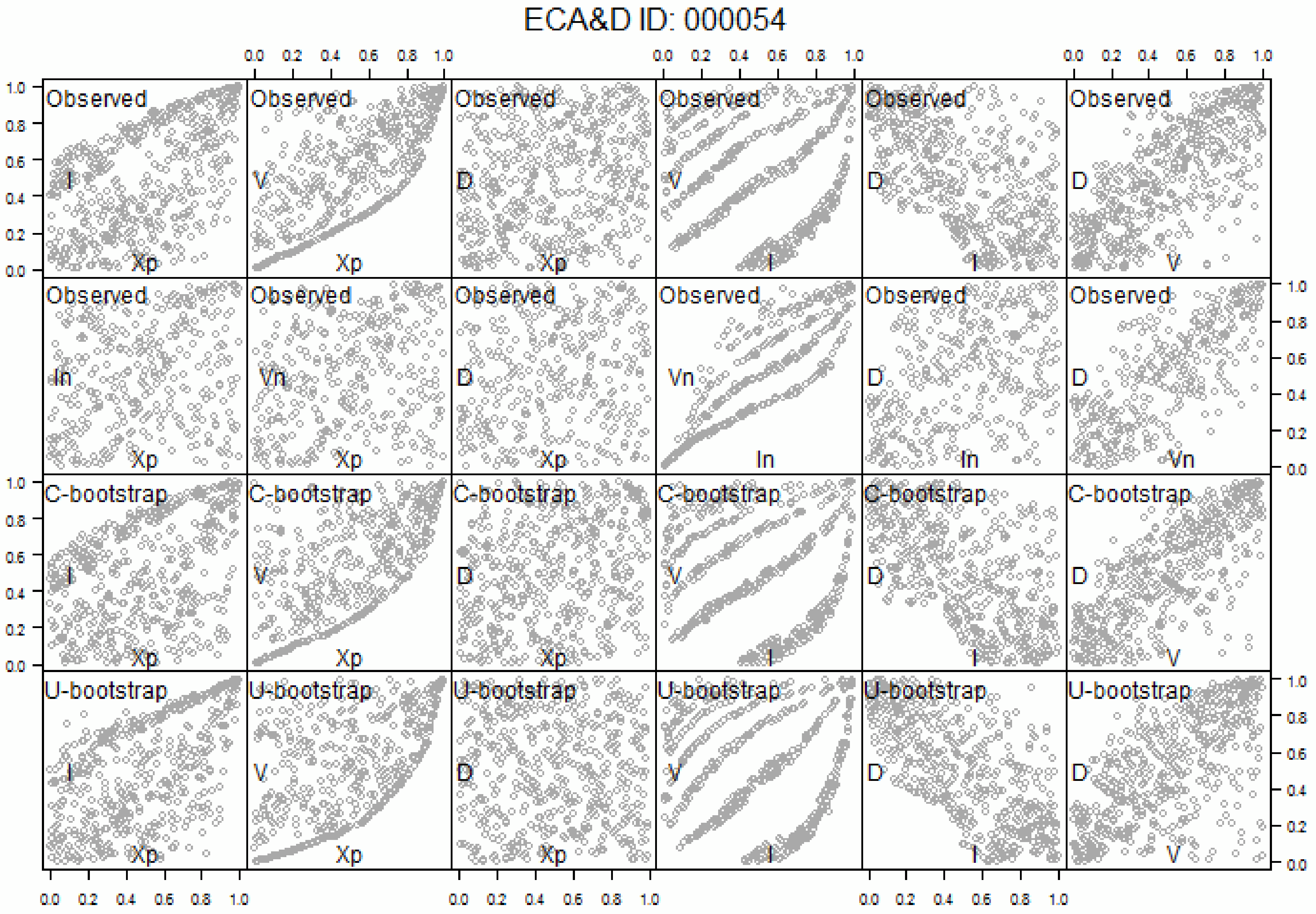
ECA&D ID: 000054
Observed
C-bootstrap
U-bootstrap
Xp
I
V
D
In
Vn
0.0
0.2
0.4
0.6
0.8
1.0

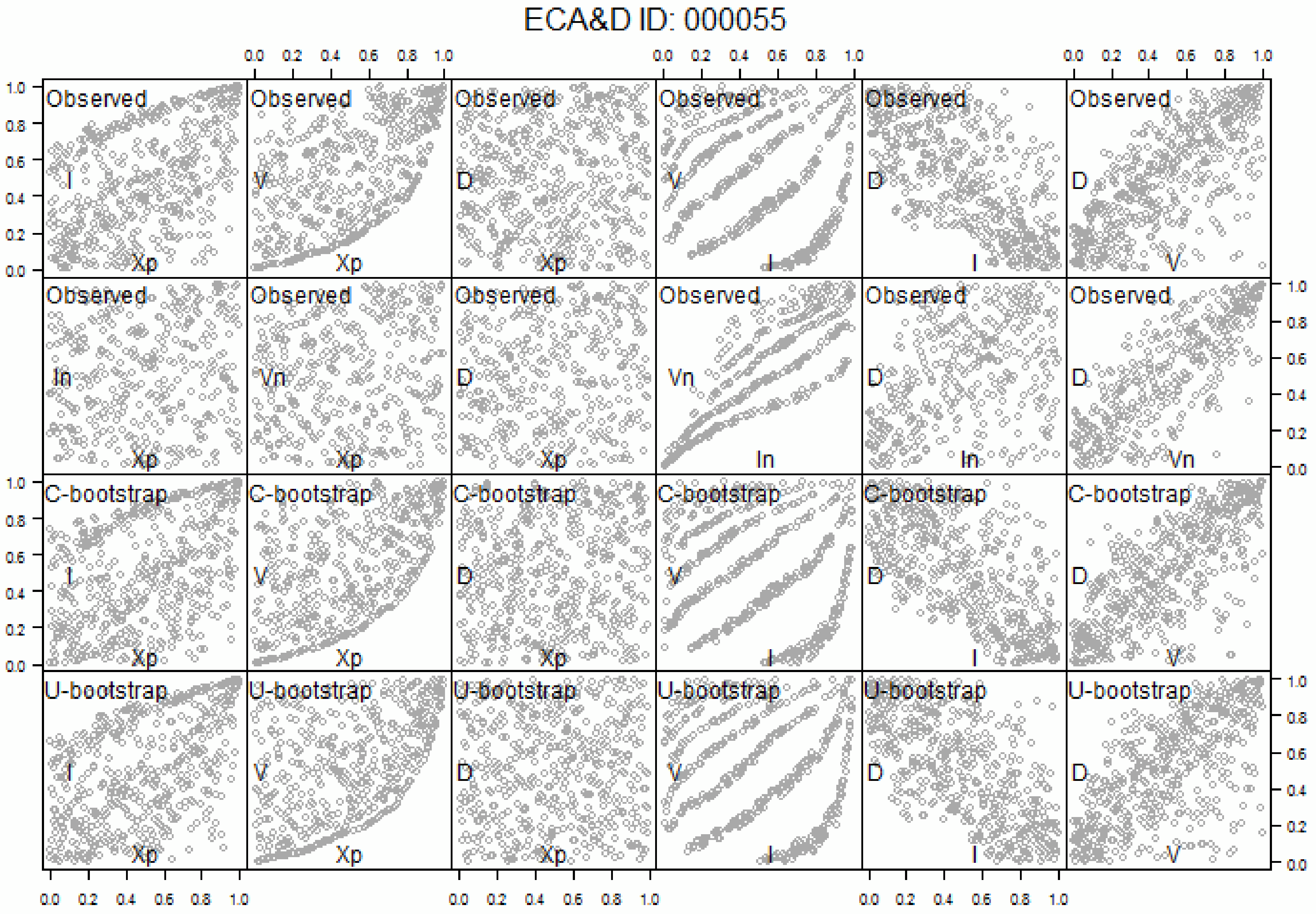
ECA&D ID: 000055
Observed
C-bootstrap
U-bootstrap
Xp
I
V
D
In
Vn
0.0
0.2
0.4
0.6
0.8
1.0

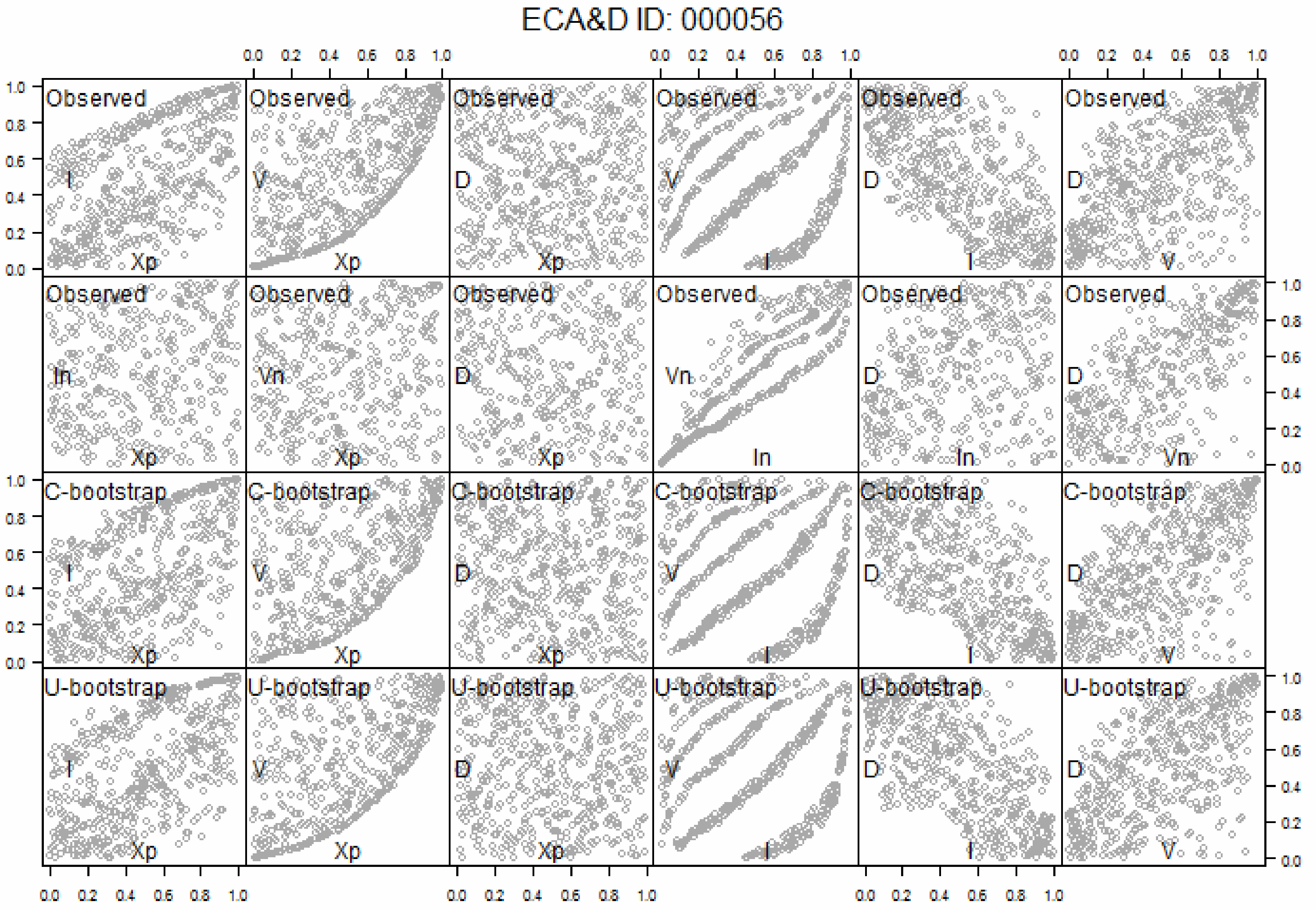
ECA&D ID: 000056
Observed
C-bootstrap
U-bootstrap
Xp
I
V
D
In
Vn
0.0
0.2
0.4
0.6
0.8
1.0

ECA&D ID: 000058

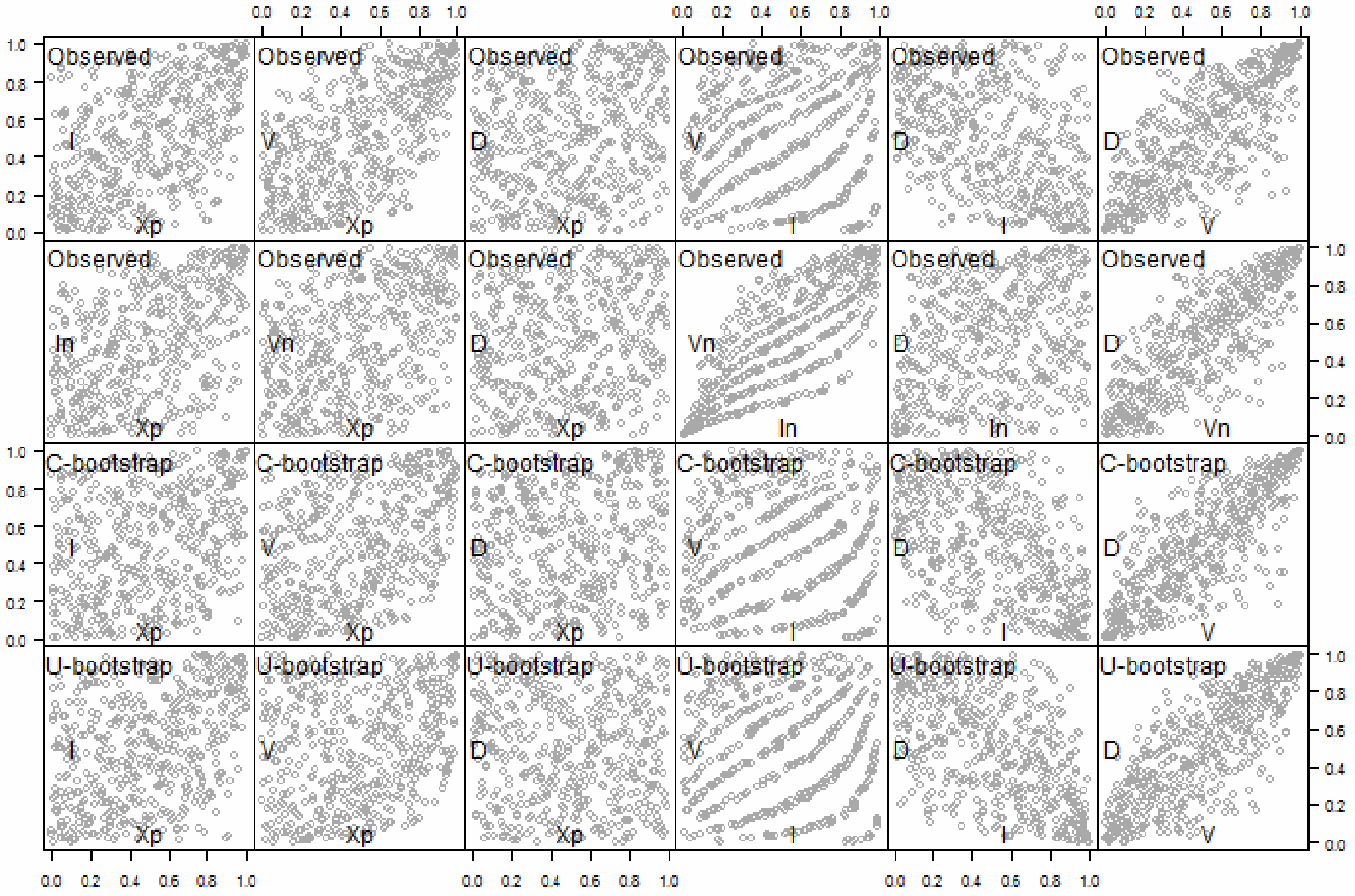

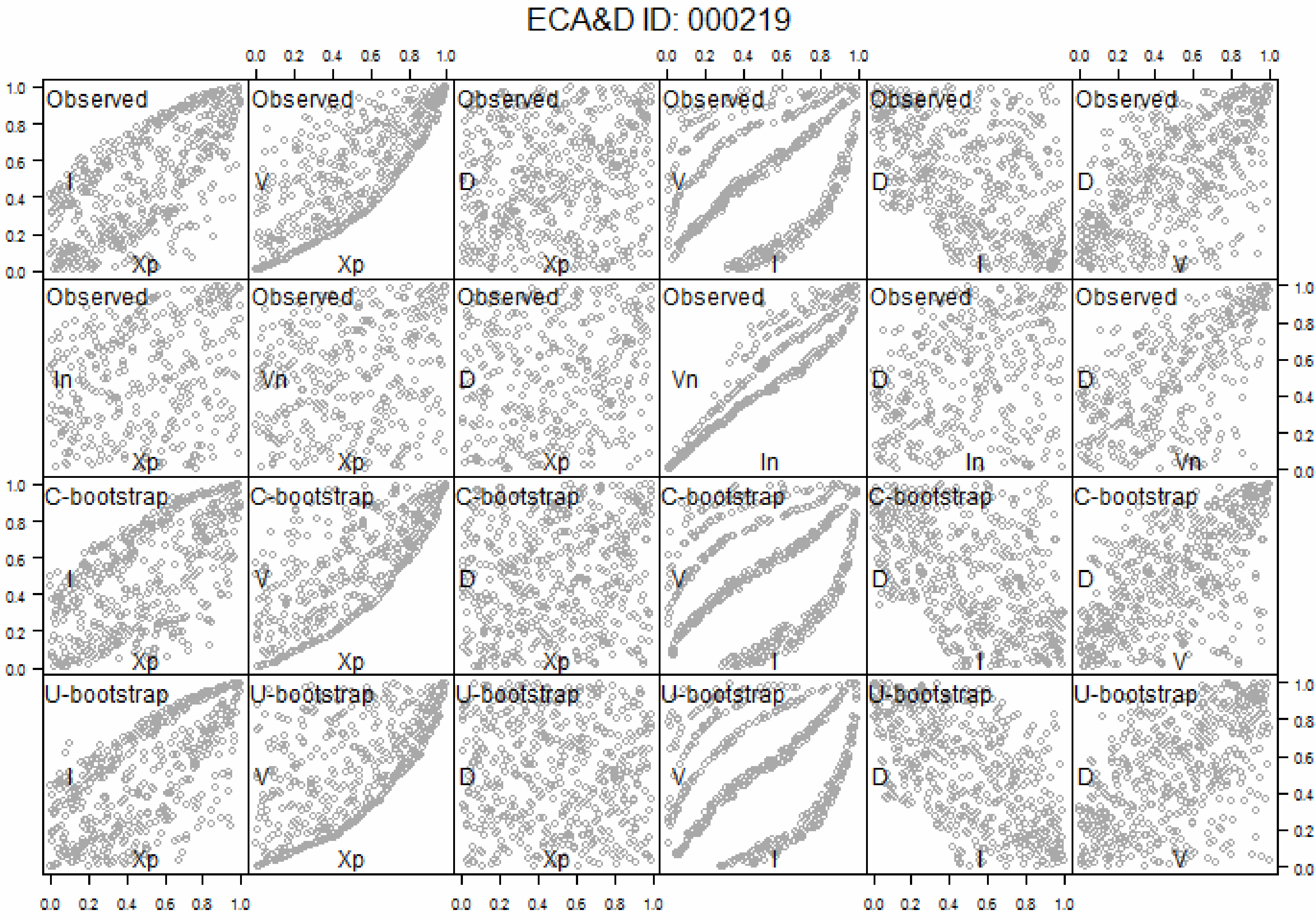
ECA&D ID: 000219
Observed
C-bootstrap
U-bootstrap
Xp
I
V
D
In
Vn
0.0
0.2
0.4
0.6
0.8
1.0

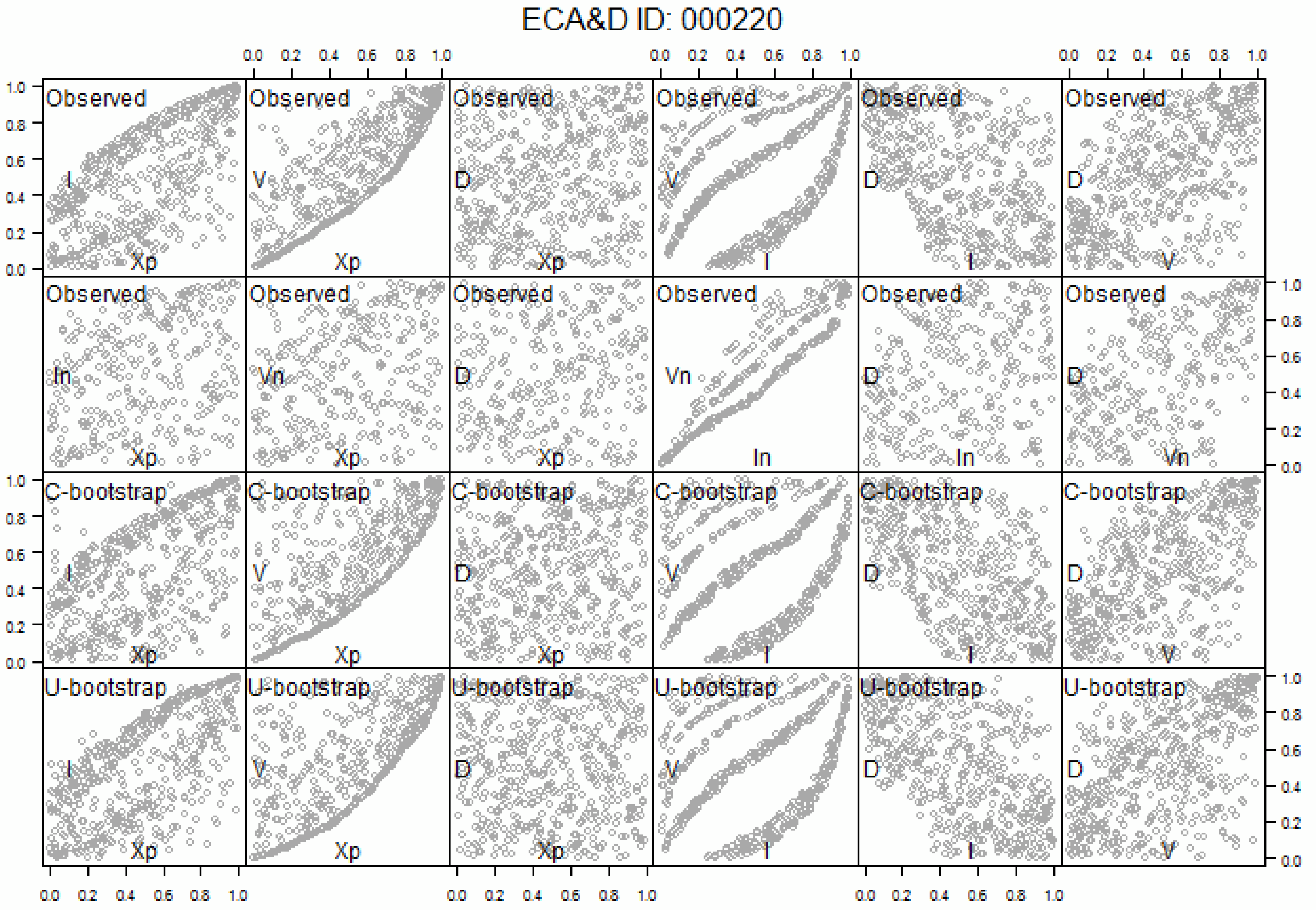

ECA&D ID: 000220
Observed
C-bootstrap
U-bootstrap
Xp
I
V
D
In
Vn
0.0
0.2
0.4
0.6
0.8
1.0

ECA&D ID: 000221

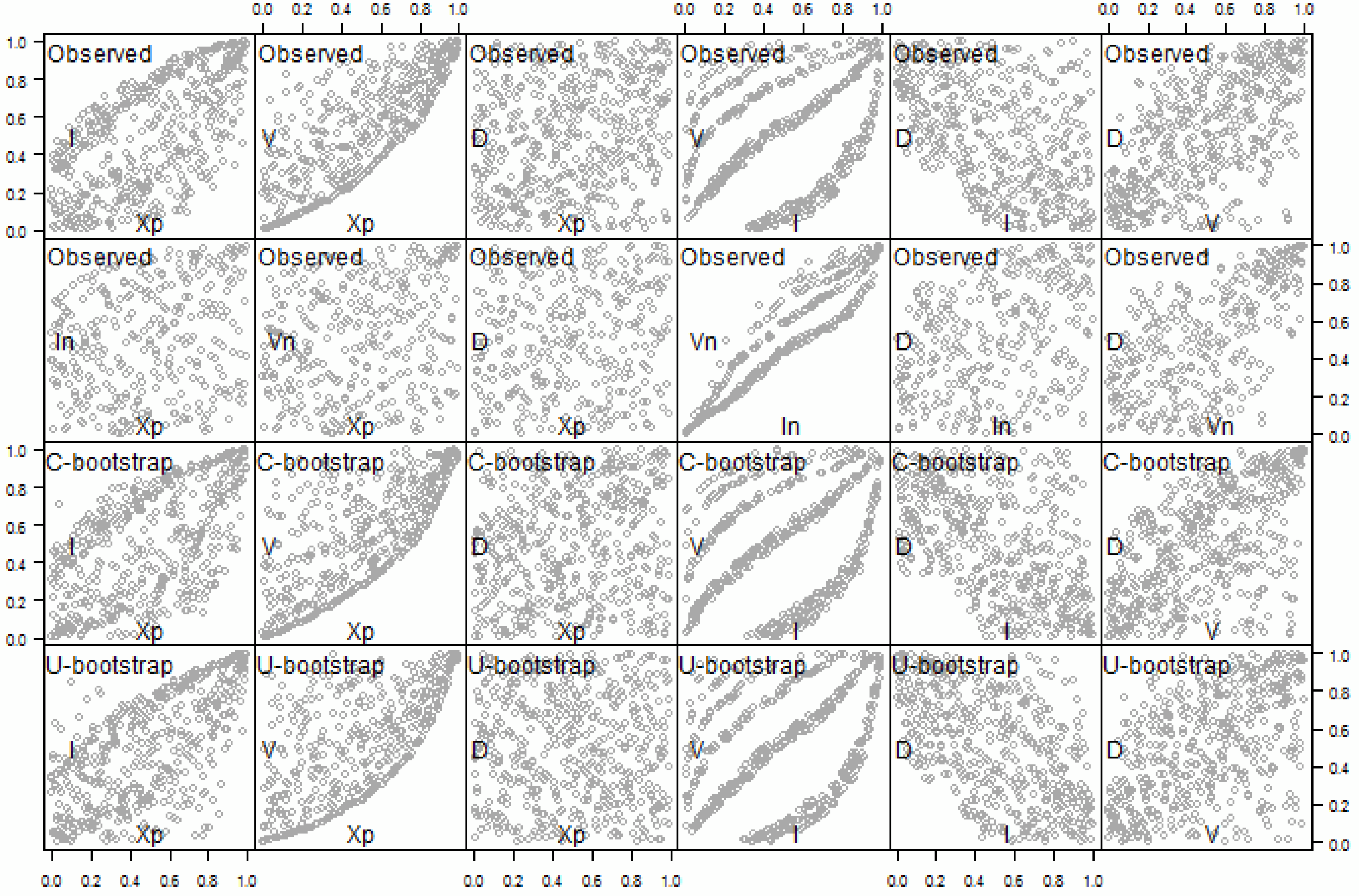

ECA&D ID: 000222

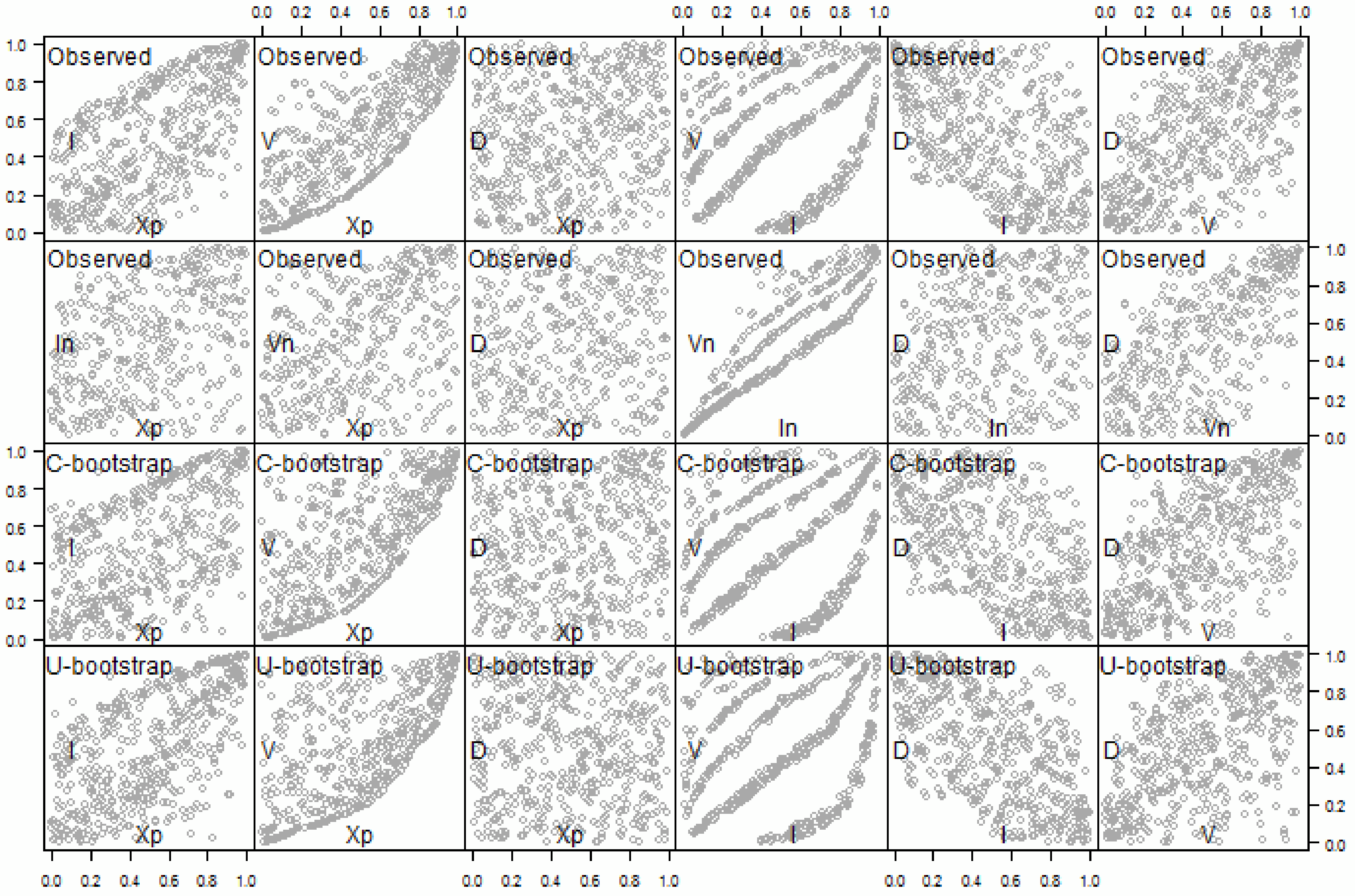

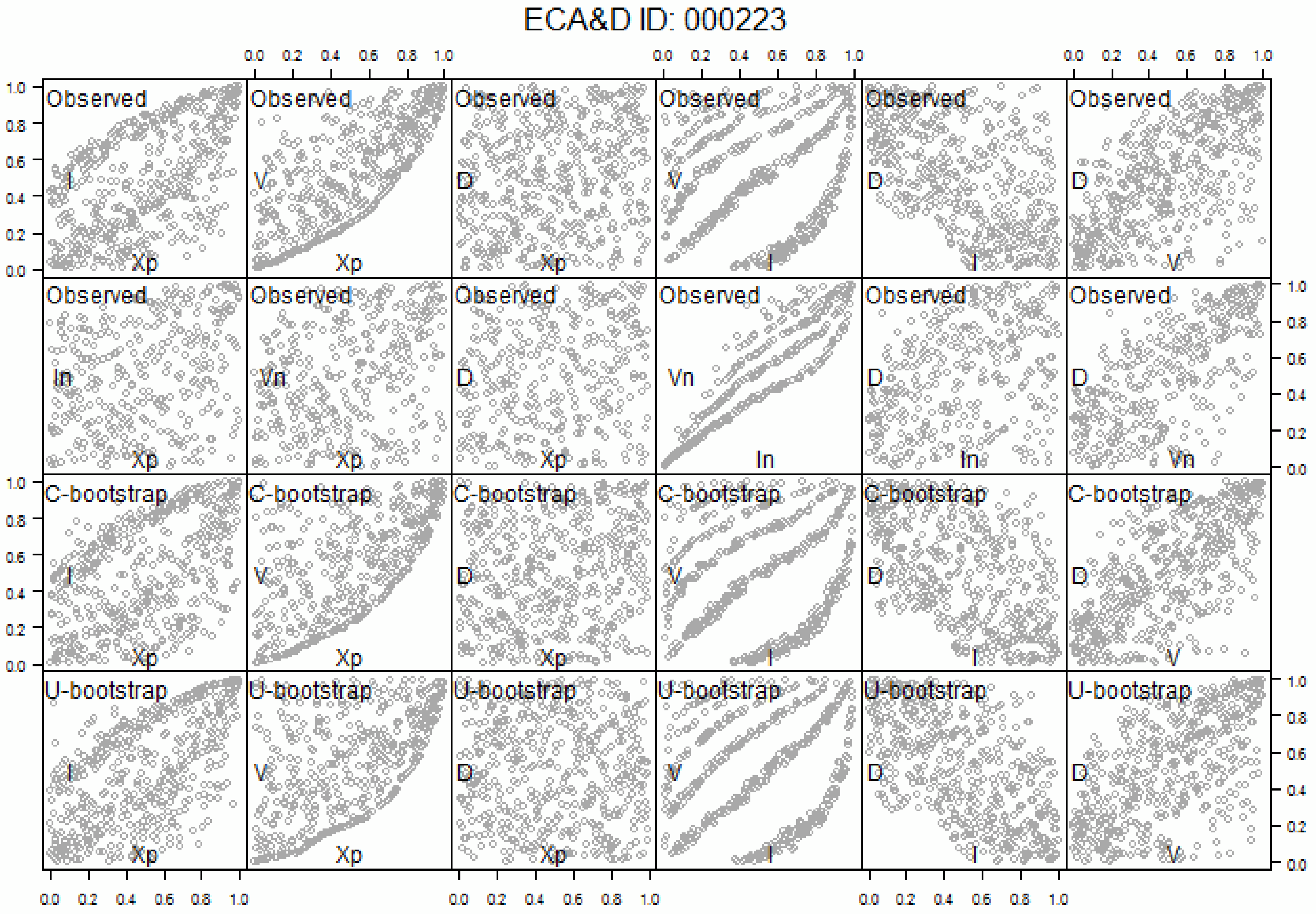
ECA&D ID: 000223
Observed
C-bootstrap
U-bootstrap
Xp
I
V
D
In
Vn

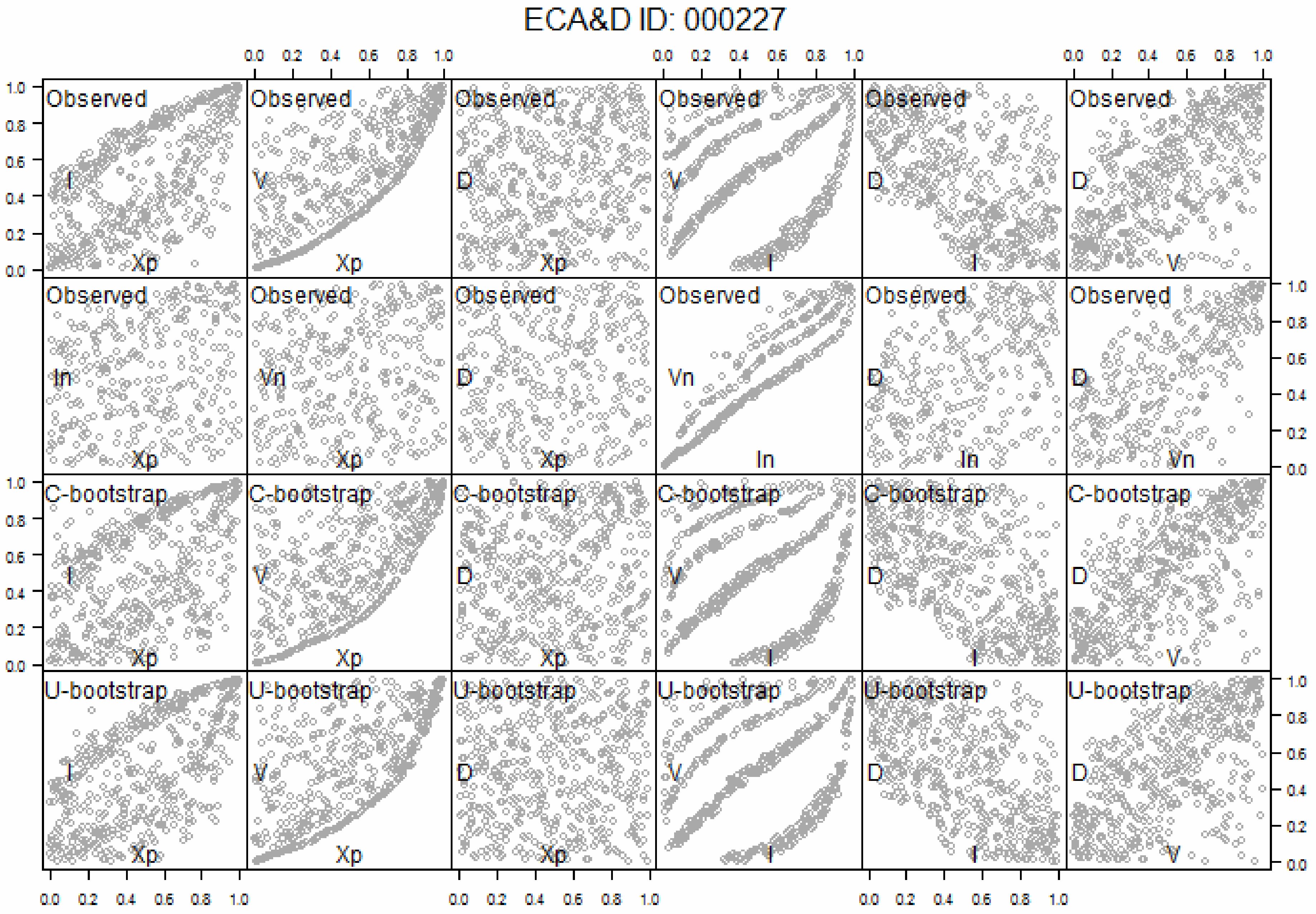

ECA&D ID: 000227
Observed
C-bootstrap
U-bootstrap
Xp
I
V
D
In
Vn
0.0
0.2
0.4
0.6
0.8
1.0

ECA&D ID: 000228

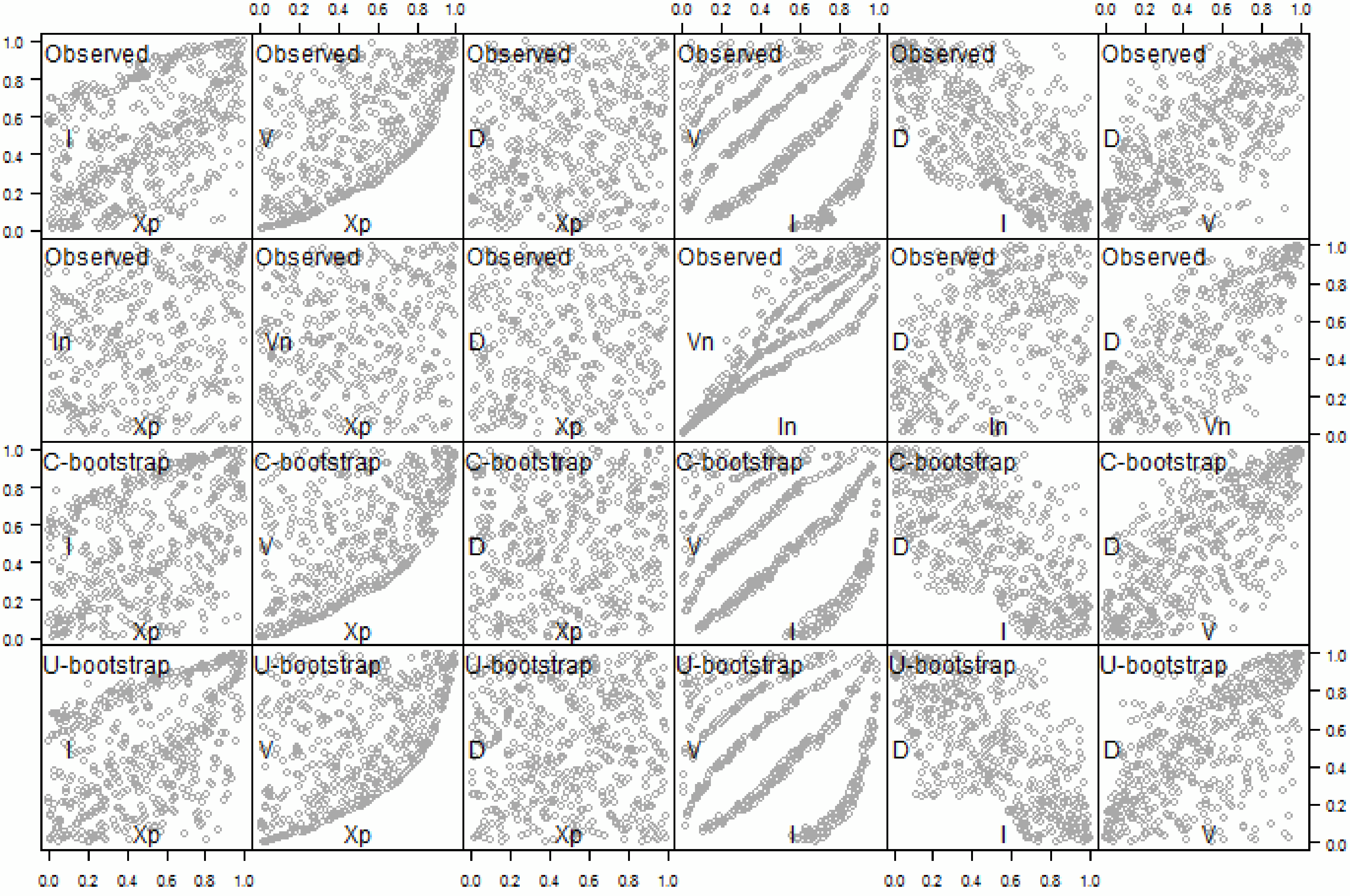

ECA&D ID: 000252

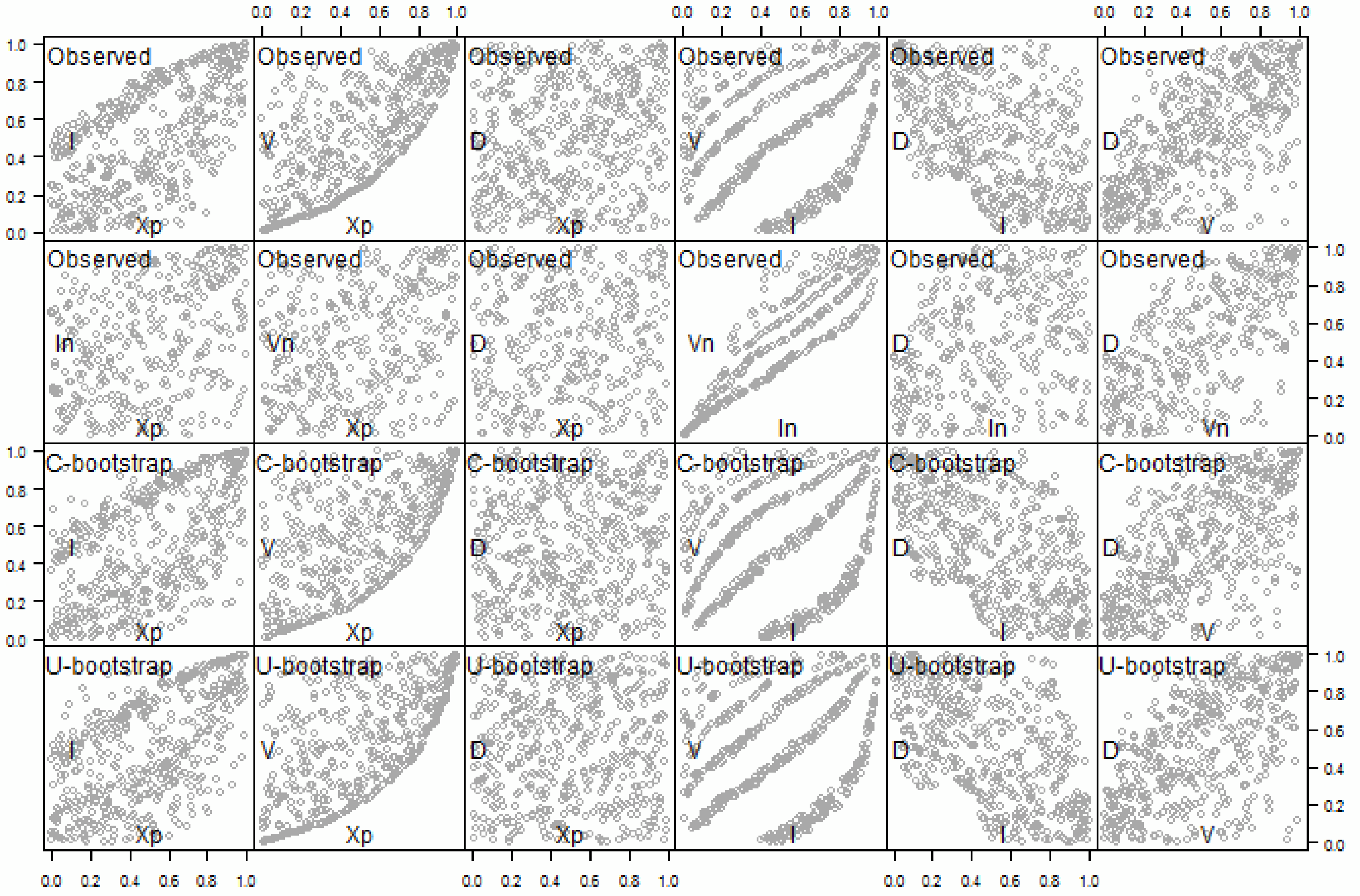

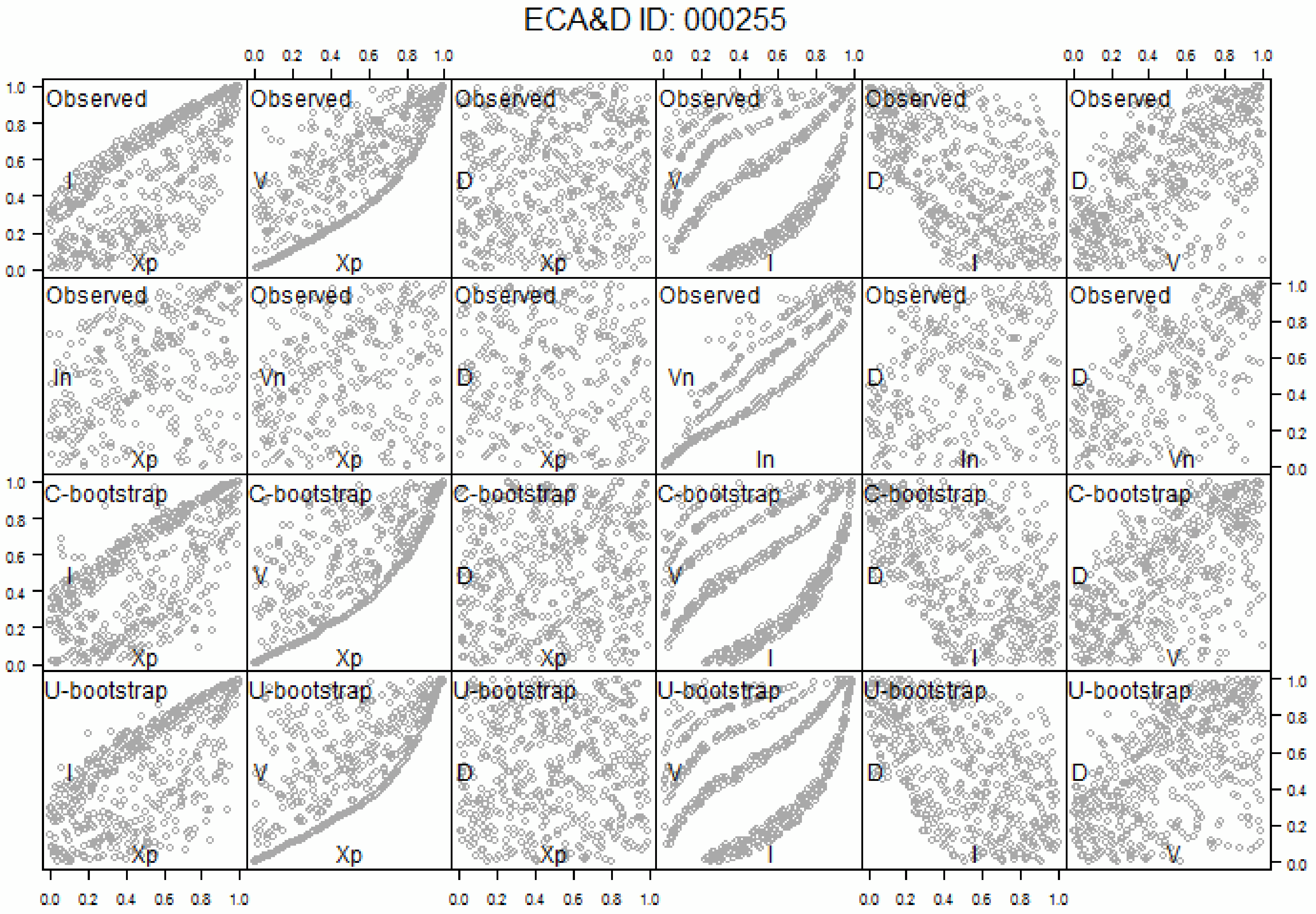
ECA&D ID: 000255
Observed
C-bootstrap
U-bootstrap
Xp
I
V
D
In
Vn
0.0
0.2
0.4
0.6
0.8
1.0

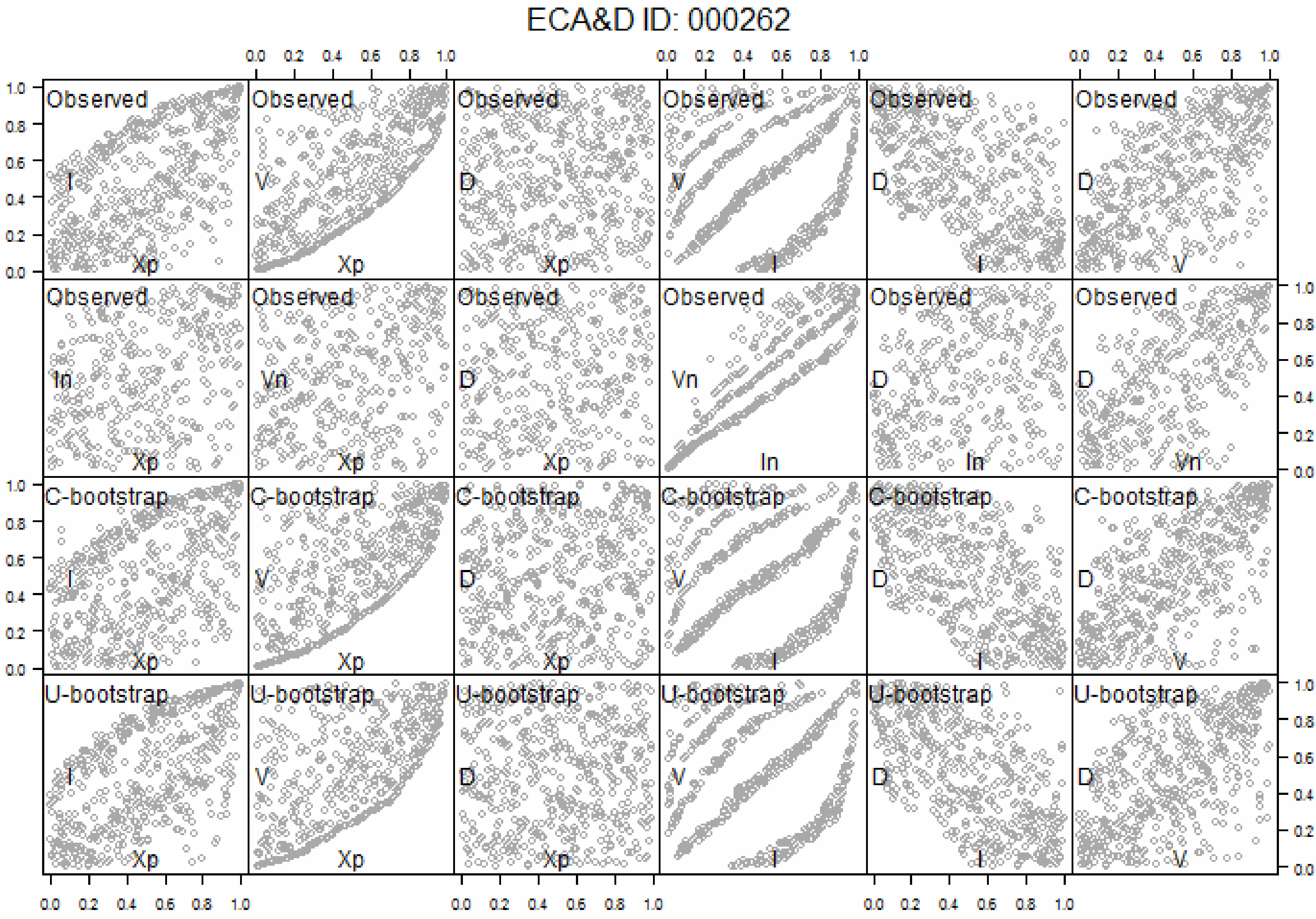

ECA&D ID: 000262
Observed
C-bootstrap
U-bootstrap
I
V
D
Xp
In
Vn
0.0
0.2
0.4
0.6
0.8
1.0

ECA&D ID: 000263

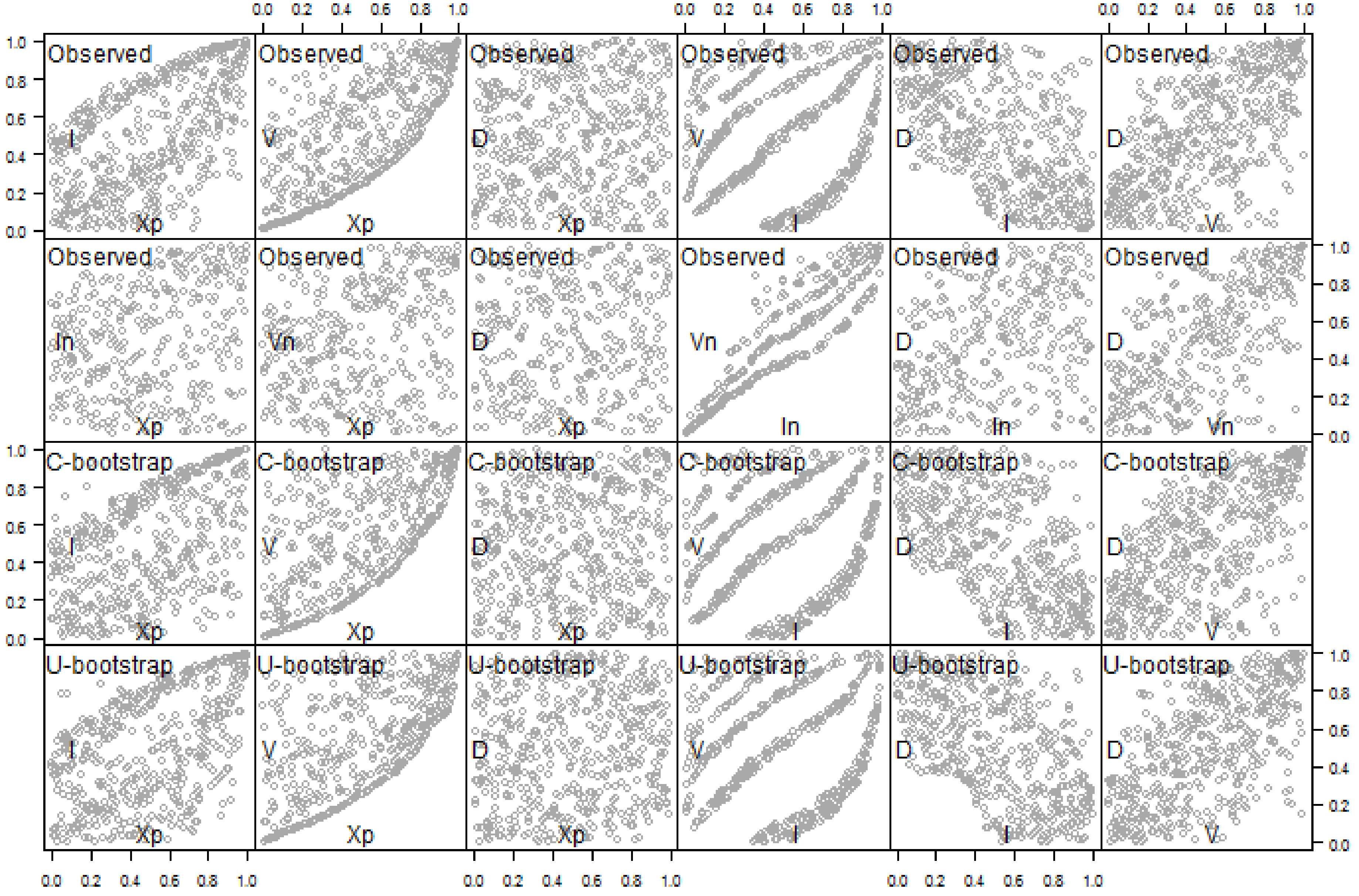

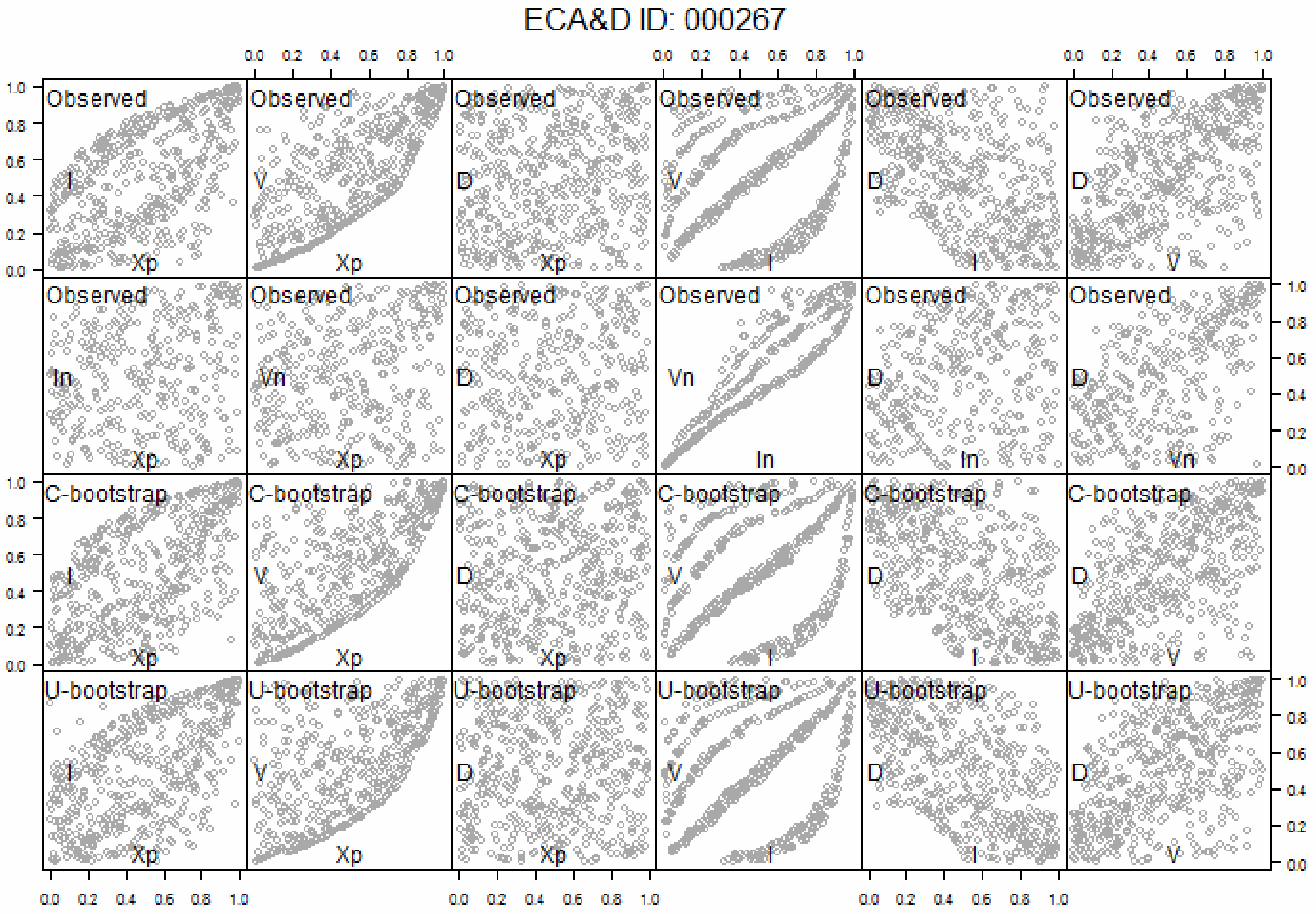
ECA&D ID: 000267
Observed
C-bootstrap
U-bootstrap
Xp
I
V
D
In
Vn
0.0
0.2
0.4
0.6
0.8
1.0

ECA&D ID: 000276

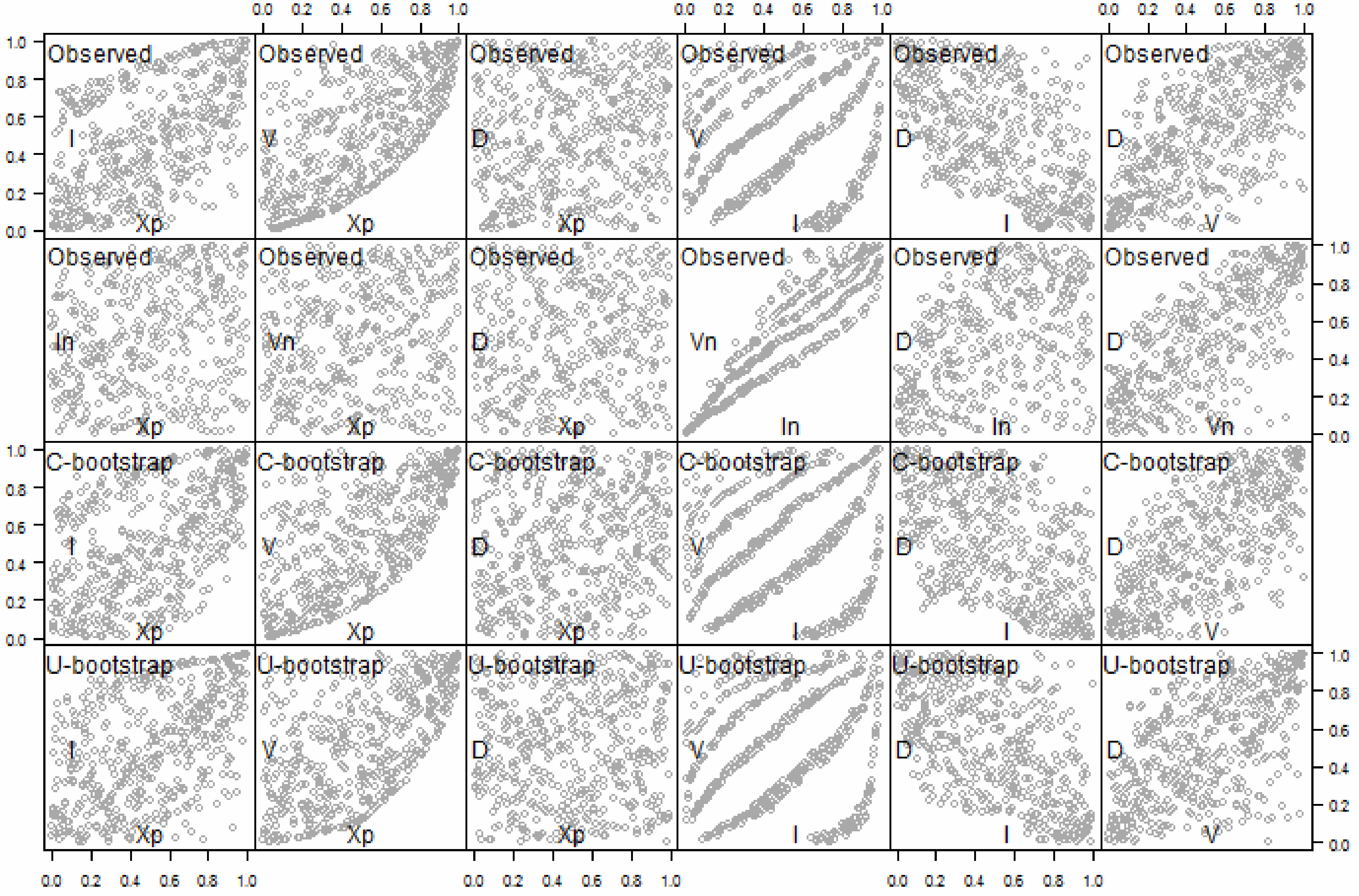

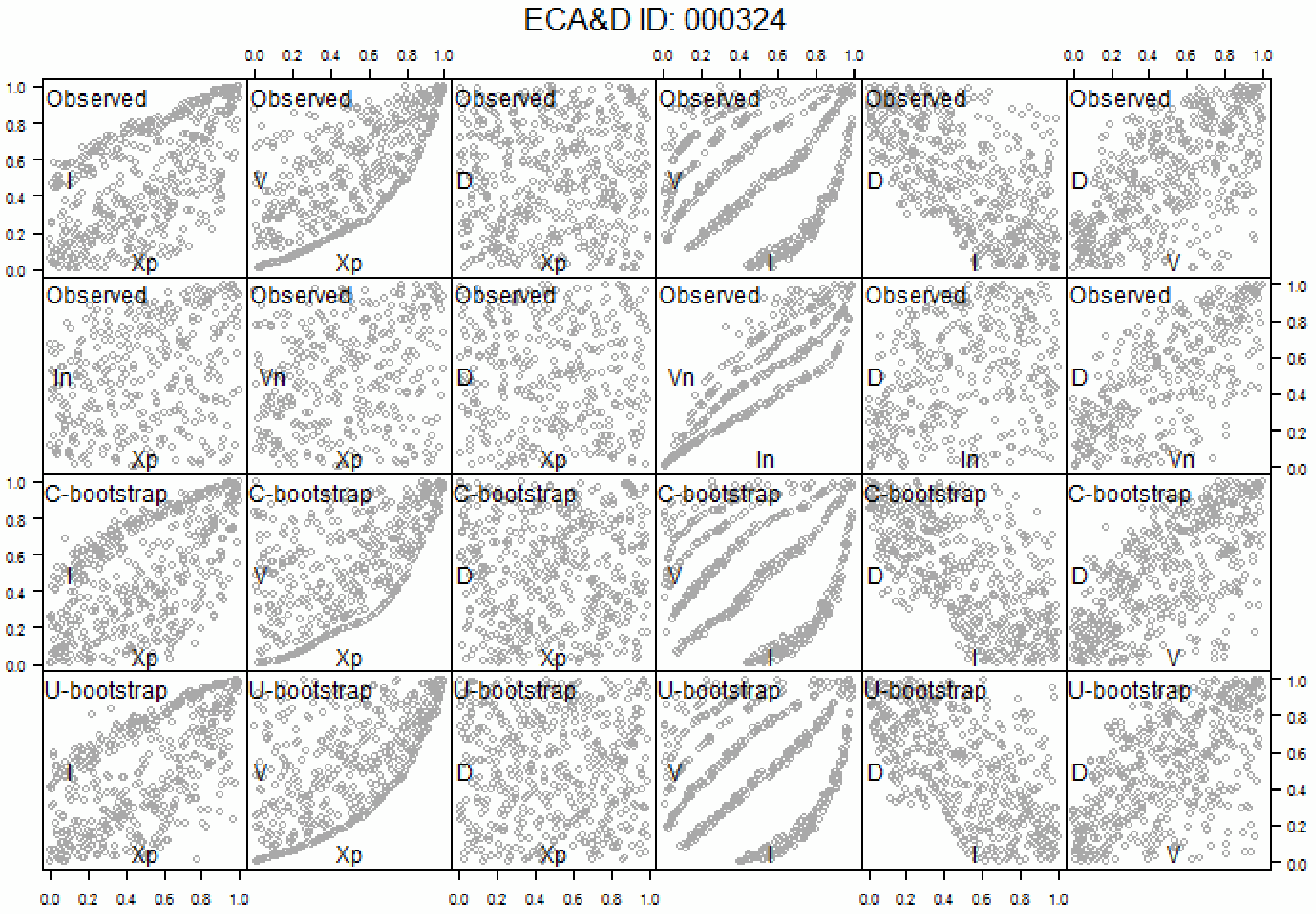
ECA&D ID: 000324
Observed
C-bootstrap
U-bootstrap
Xp
I
V
D
In
Vn
0.0
0.2
0.4
0.6
0.8
1.0

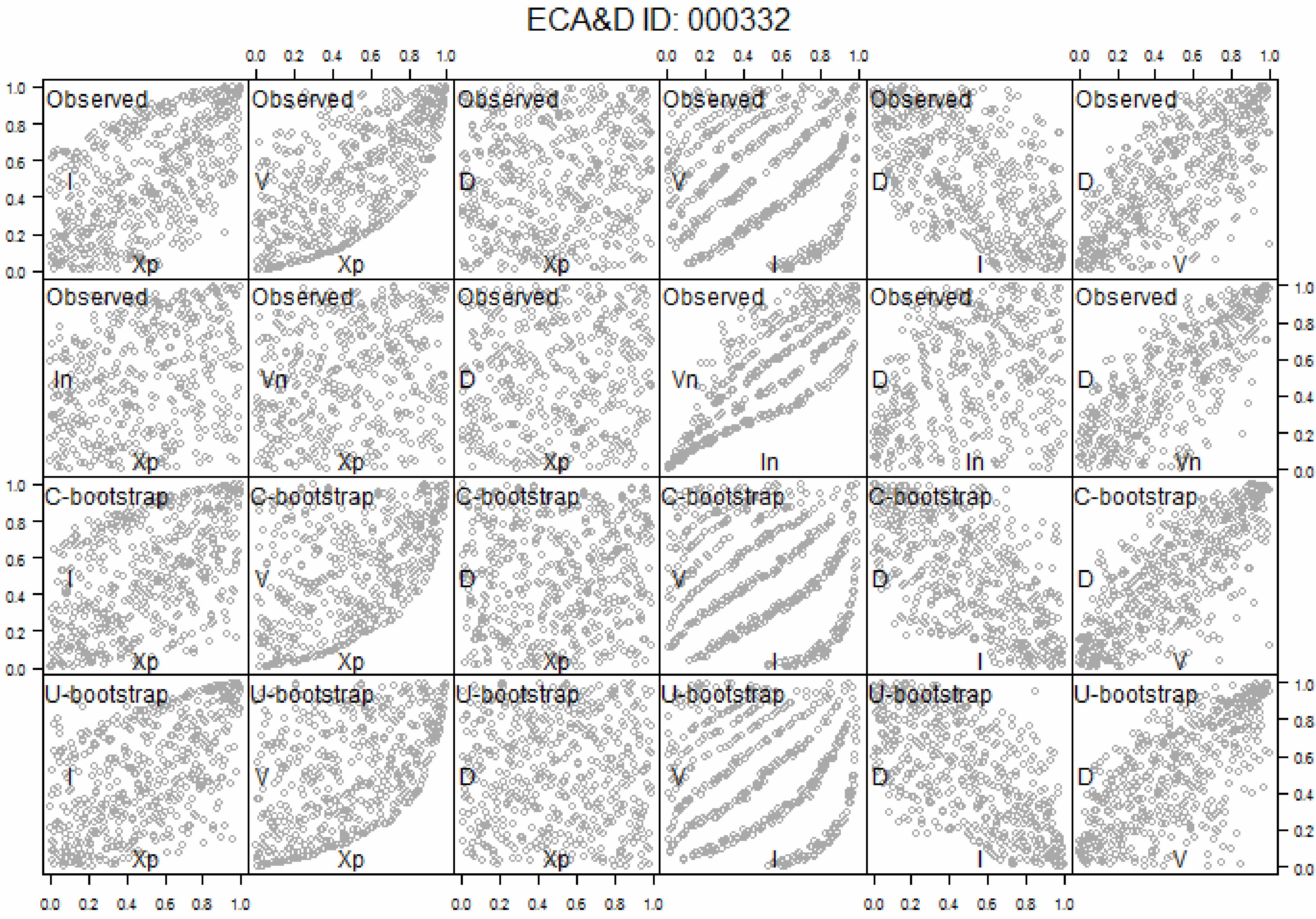
ECA&D ID: 000332
Observed
C-bootstrap
U-bootstrap
Xp
I
V
D
In
Vn
0.0
0.2
0.4
0.6
0.8
1.0

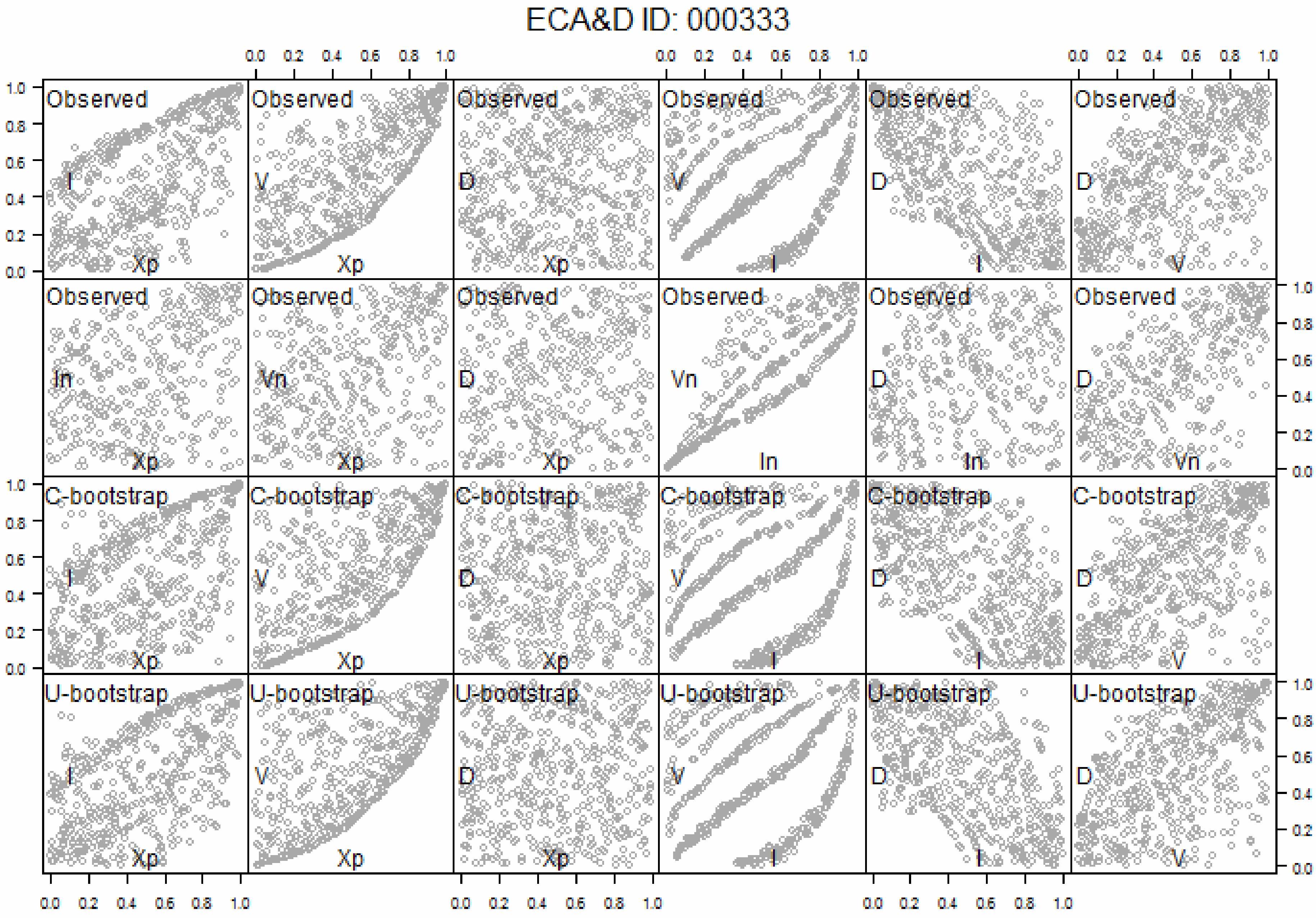

ECA&D ID: 000333
Observed
C-bootstrap
U-bootstrap
Xp
I
V
D
In
Vn
0.0
0.2
0.4
0.6
0.8
1.0

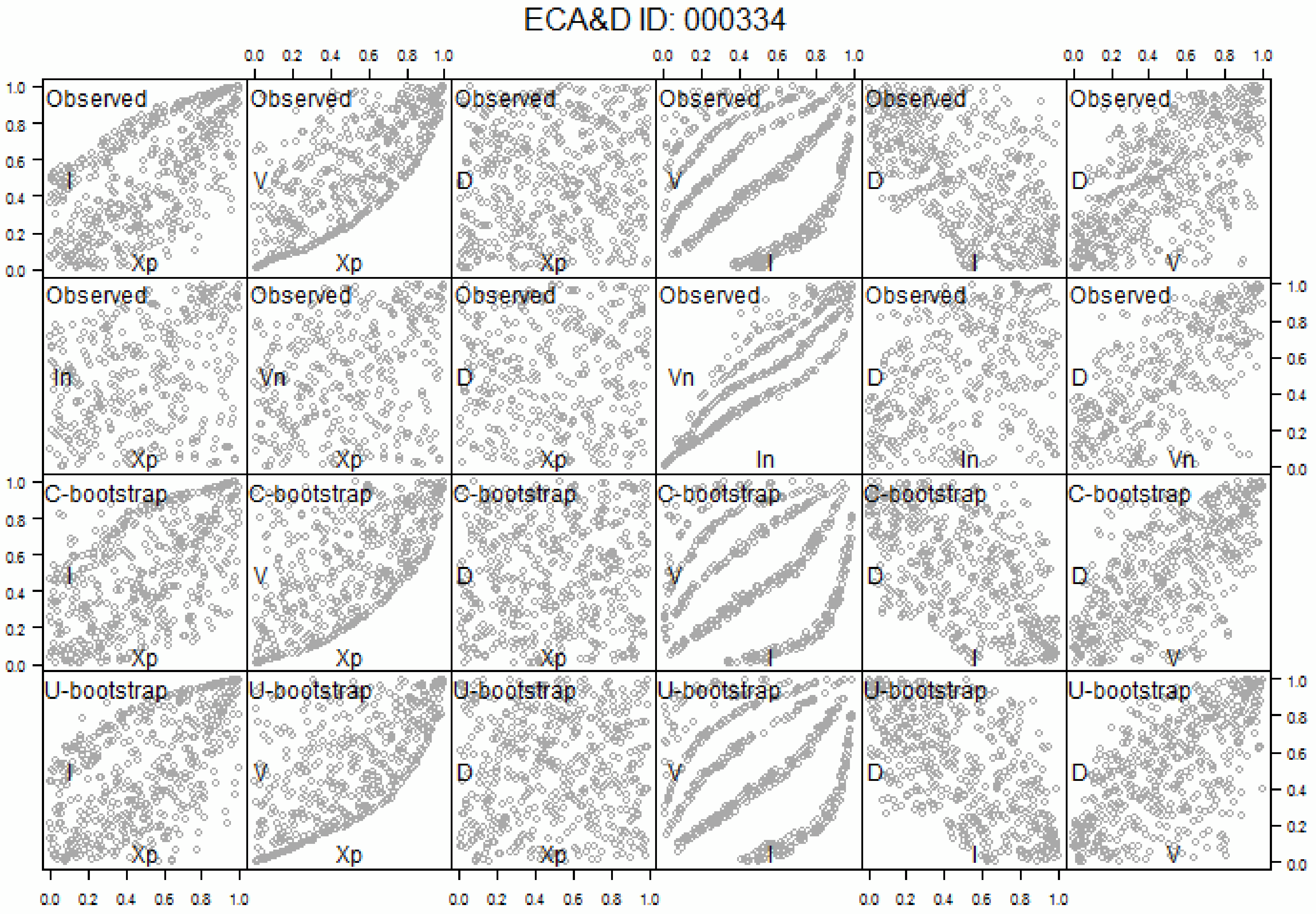

ECA&D ID: 000334
Observed
C-bootstrap
U-bootstrap
Xp
I
V
D
In
Vn
0.0
0.2
0.4
0.6
0.8
1.0

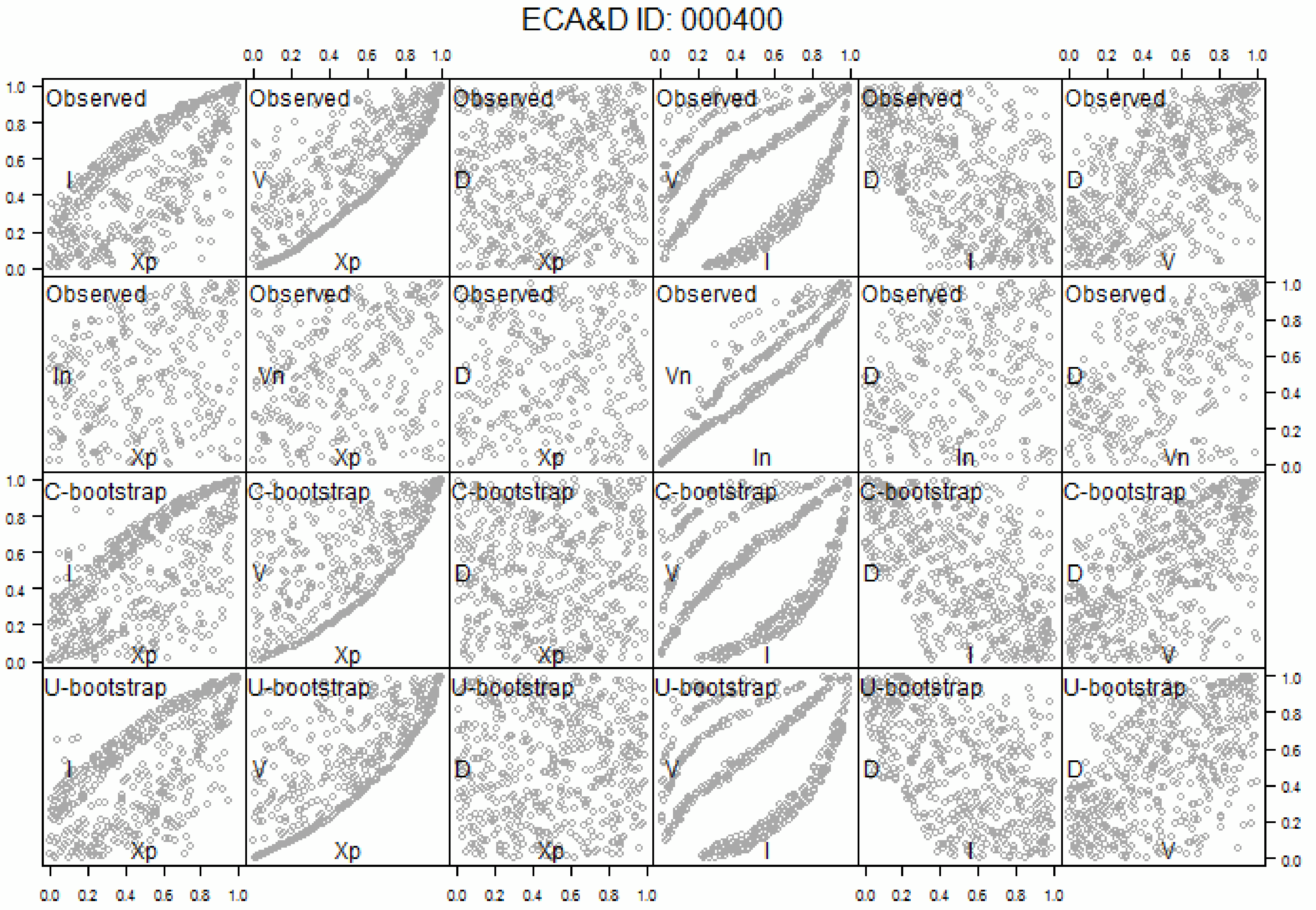
ECA&D ID: 000400
Observed
C-bootstrap
U-bootstrap
Xp
I
V
D
In
Vn
0.0
0.2
0.4
0.6
0.8
1.0

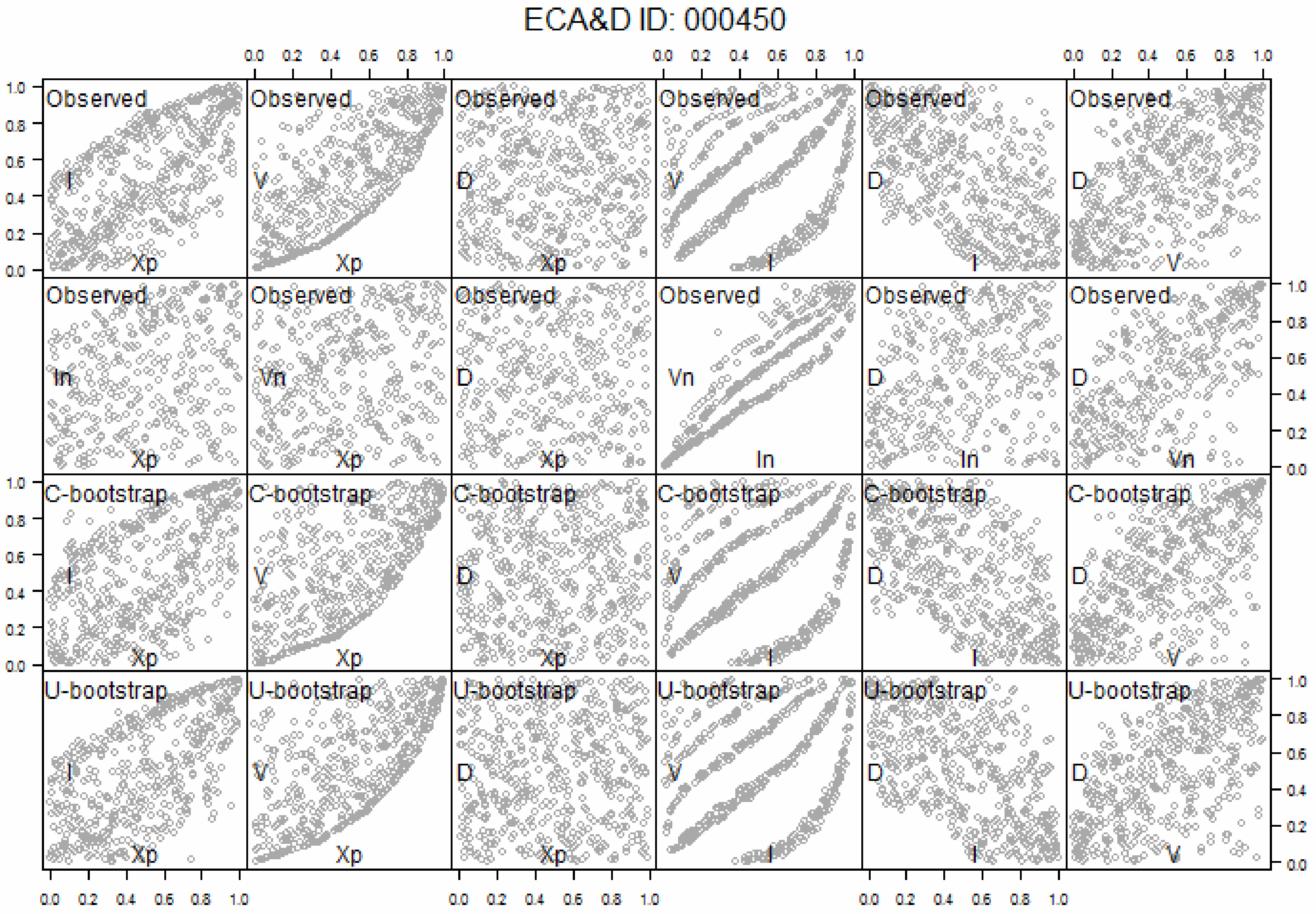
ECA&D ID: 000450
Observed
C-bootstrap
U-bootstrap
Xp
I
V
D
In
Vn
0.0
0.2
0.4
0.6
0.8
1.0

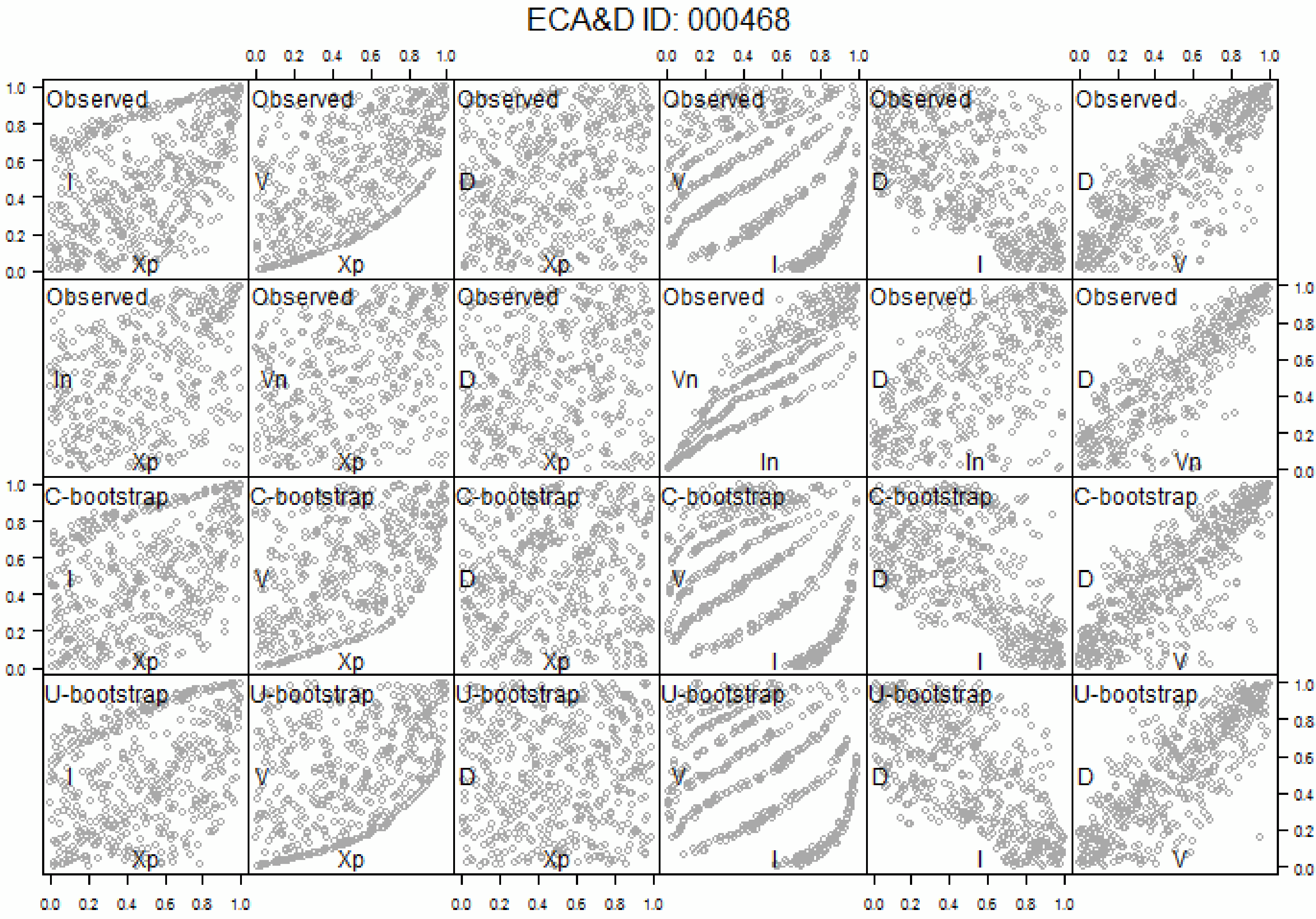
ECA&D ID: 000468
Observed
C-bootstrap
U-bootstrap
Xp
I
V
D
In
Vn
0.0
0.2
0.4
0.6
0.8
1.0

ECA&D ID: 000469

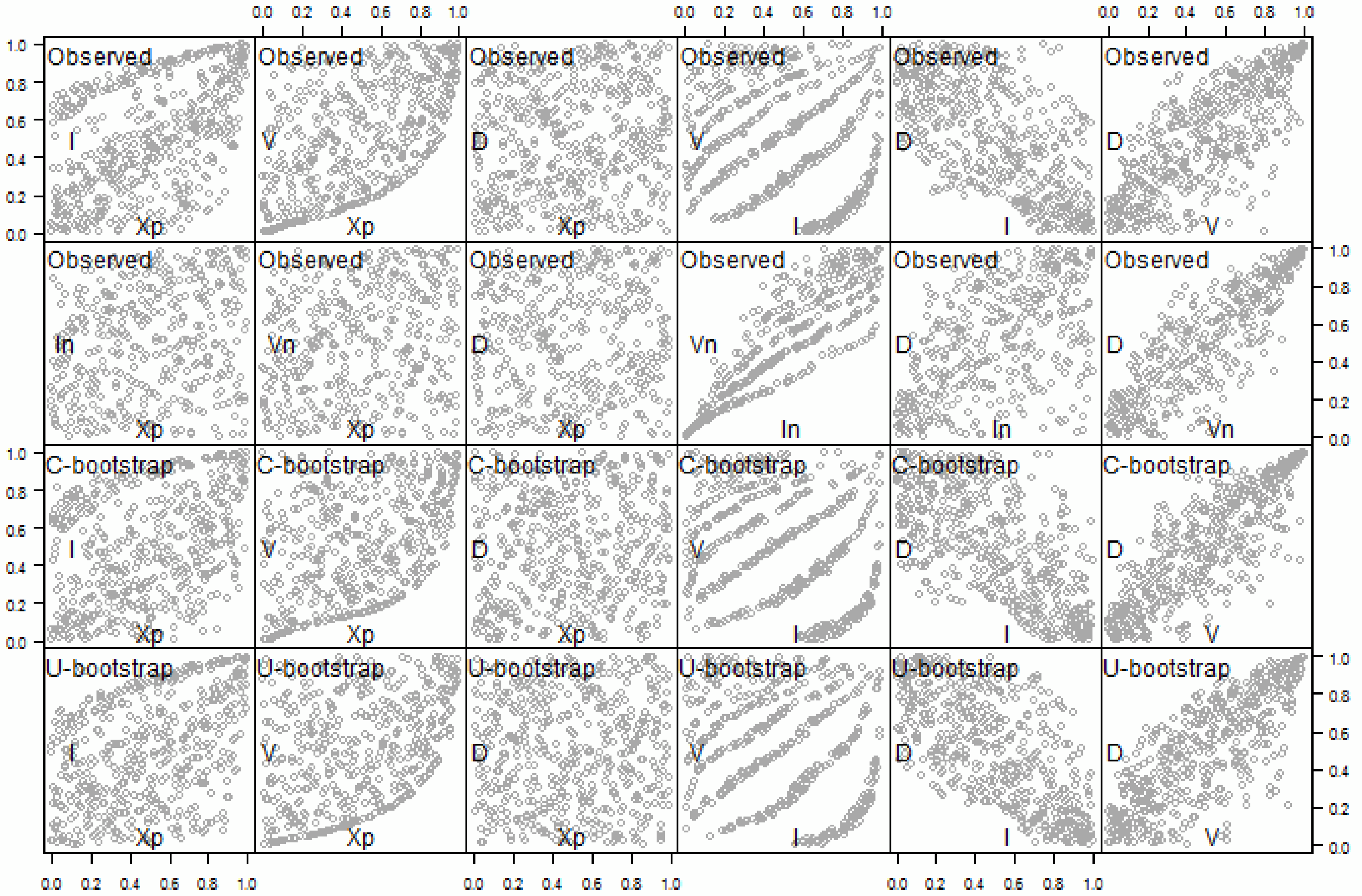

ECA&D ID: 000470

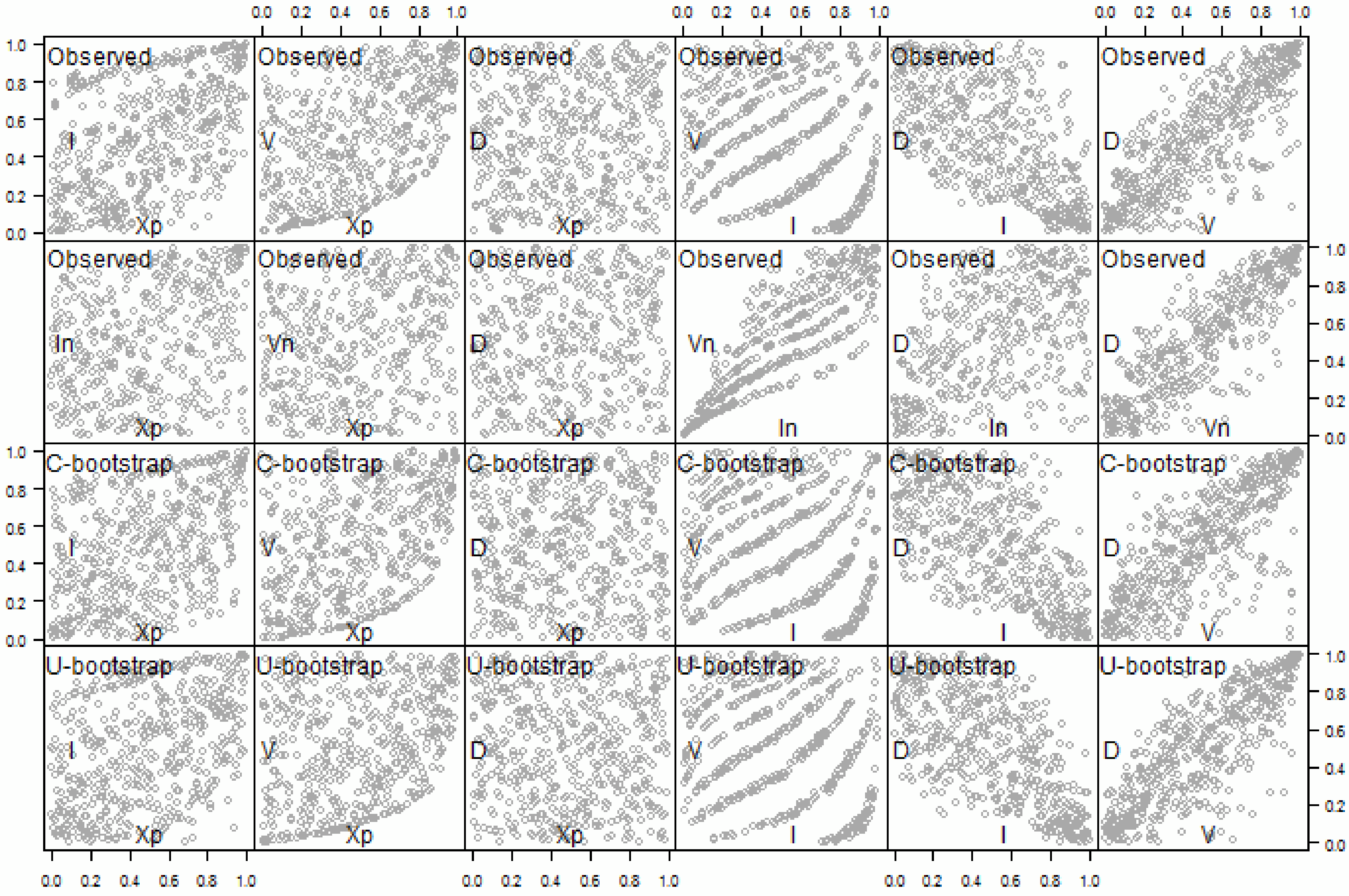

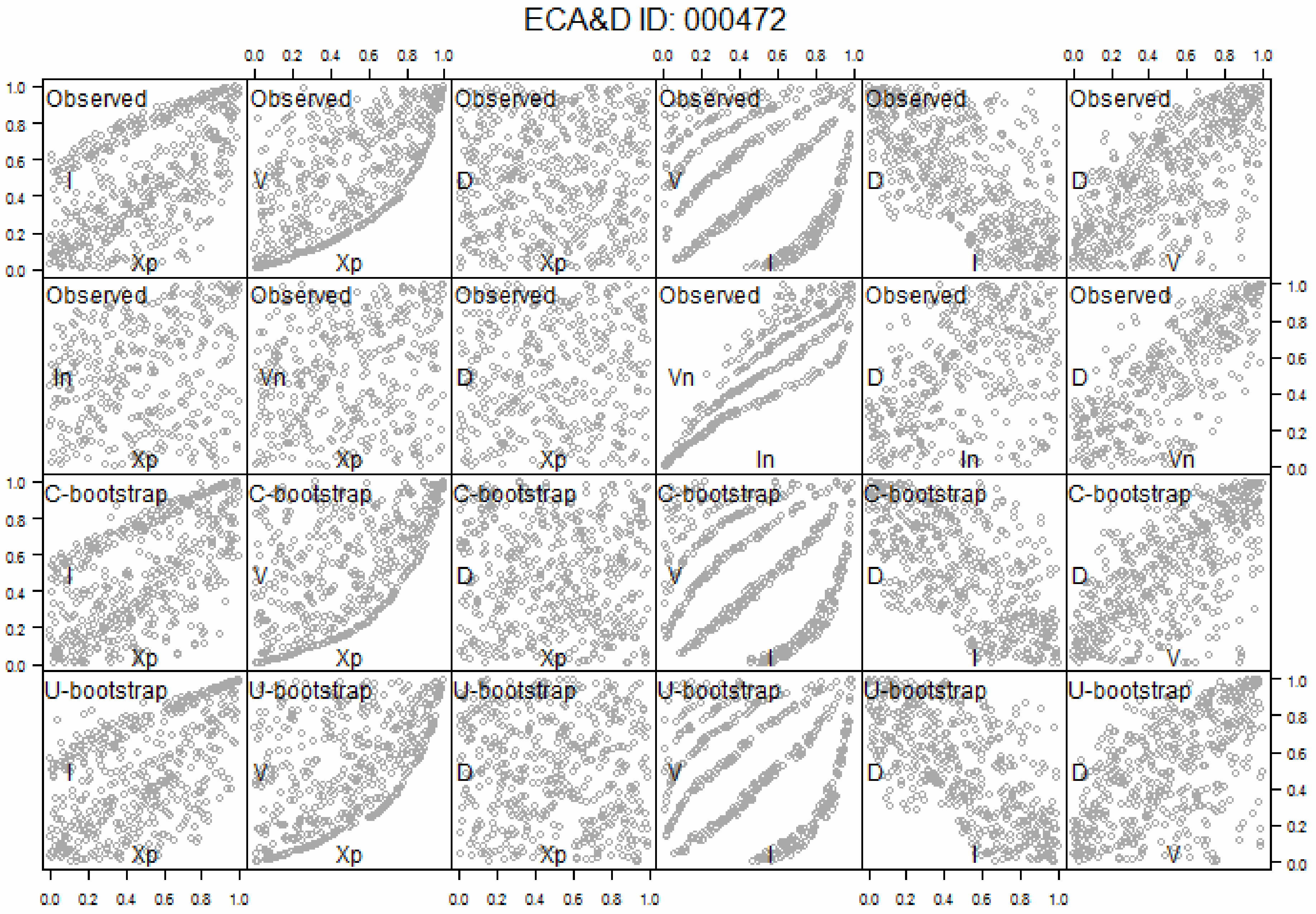
ECA&D ID: 000472
Observed
C-bootstrap
U-bootstrap
Xp
I
V
D
In
Vn
0.0
0.2
0.4
0.6
0.8
1.0

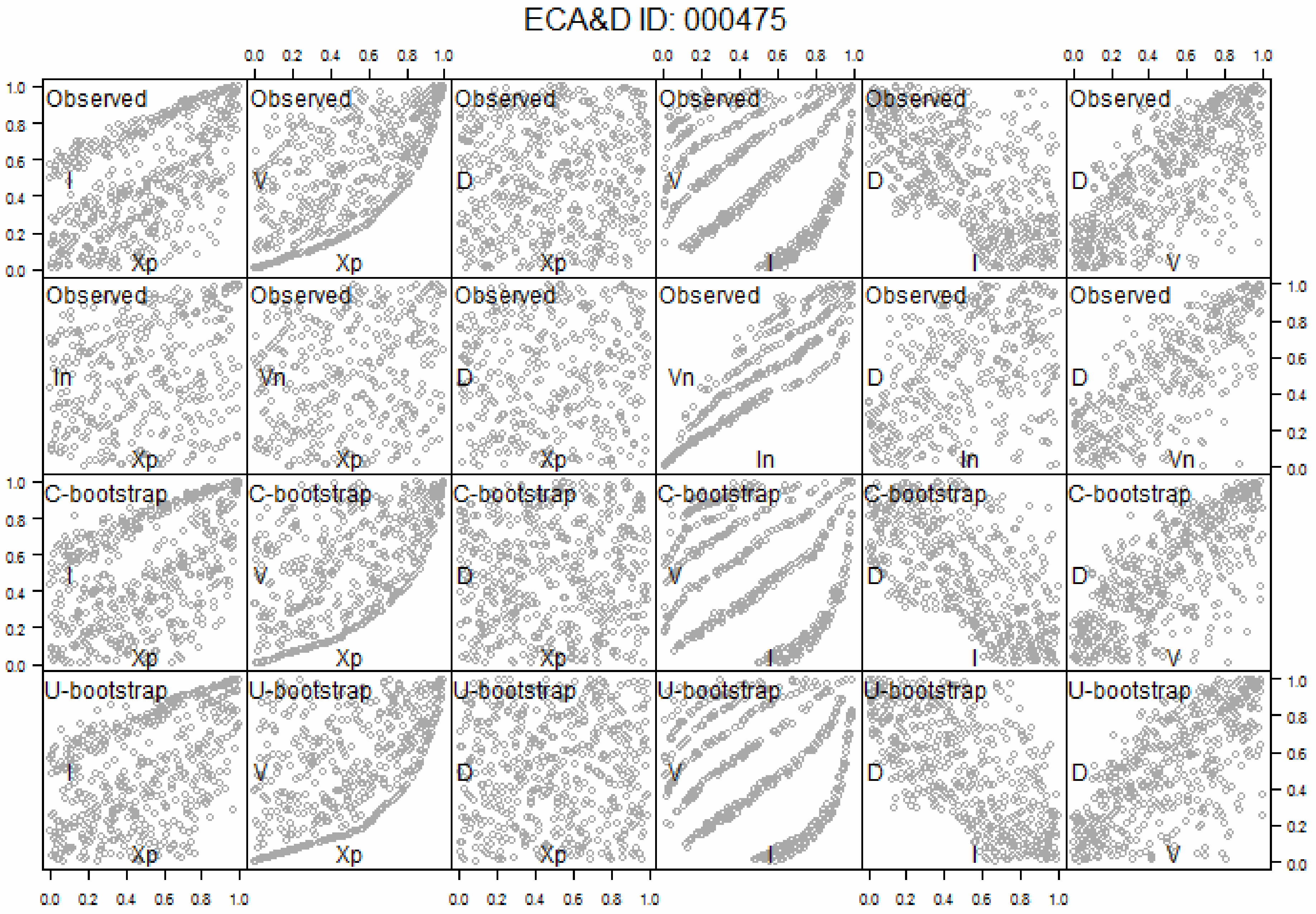
ECA&D ID: 000475
Observed
C-bootstrap
U-bootstrap
Xp
I
V
D
In
Vn
0.0
0.2
0.4
0.6
0.8
1.0

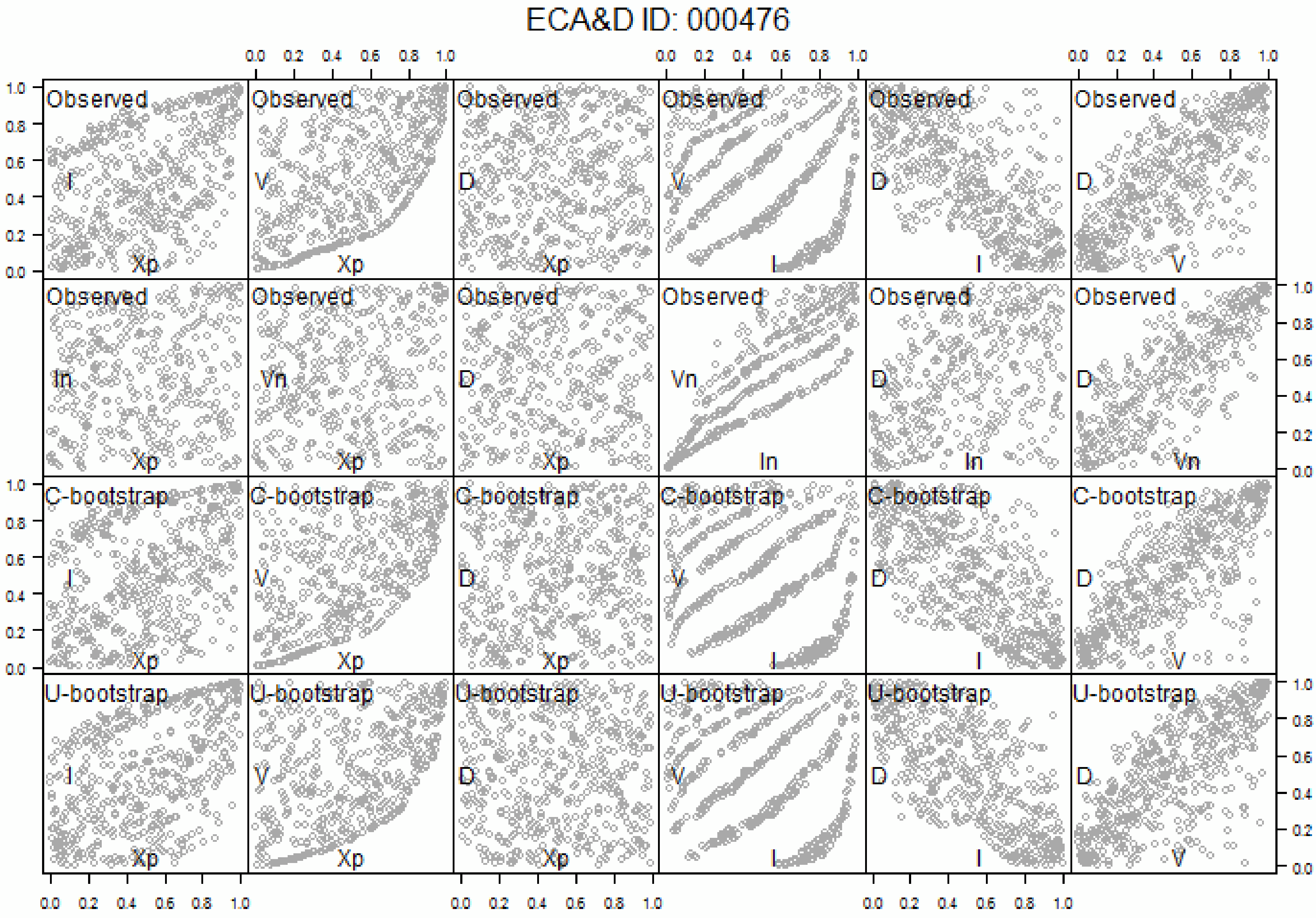
ECA&D ID: 000476
Observed
C-bootstrap
U-bootstrap
Xp
I
V
D
In
Vn
0.0
0.2
0.4
0.6
0.8
1.0

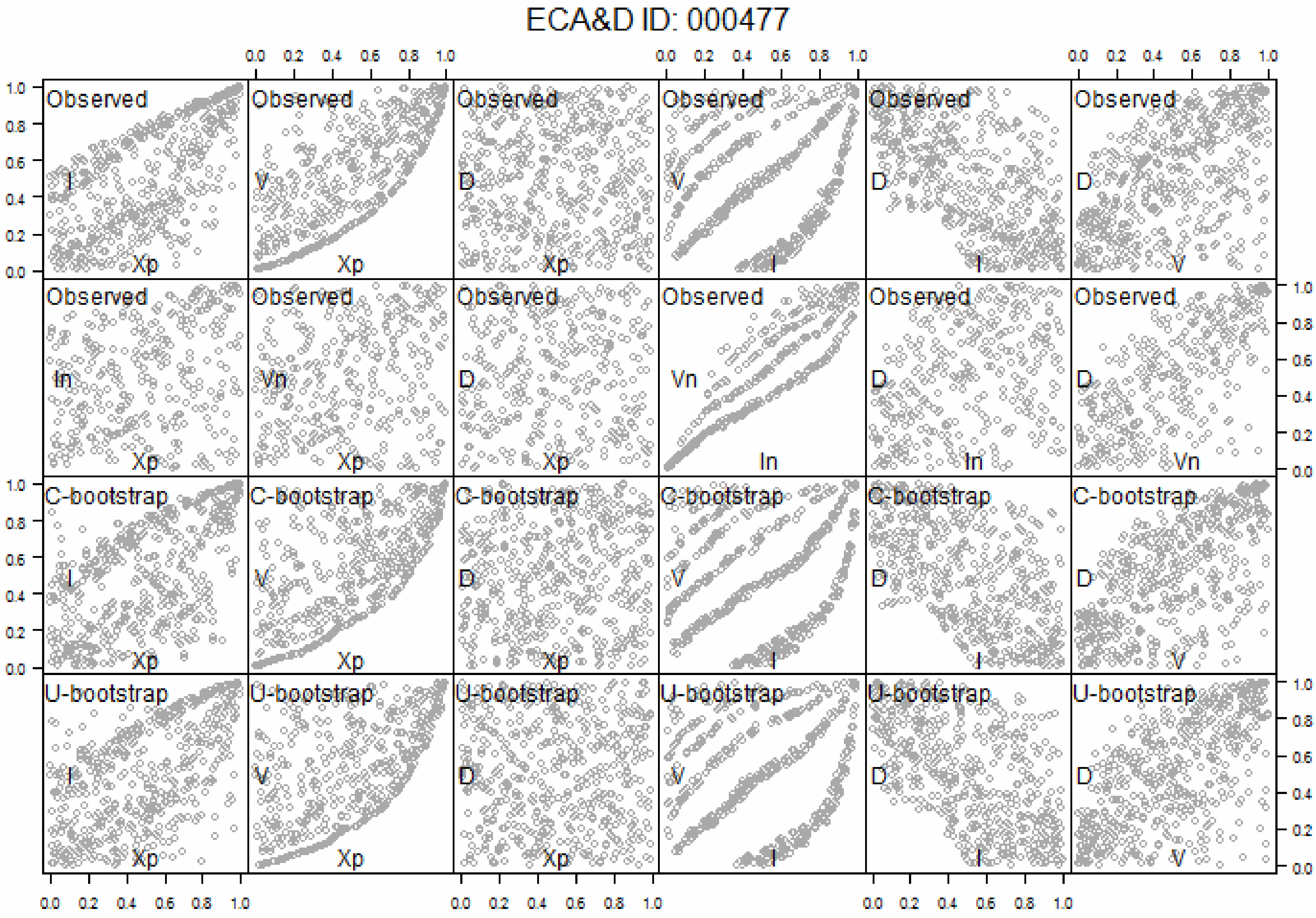
ECA&D ID: 000477
Observed
C-bootstrap
U-bootstrap
Xp
I
V
D
In
Vn
0.0
0.2
0.4
0.6
0.8
1.0

ECA&D ID: 000479

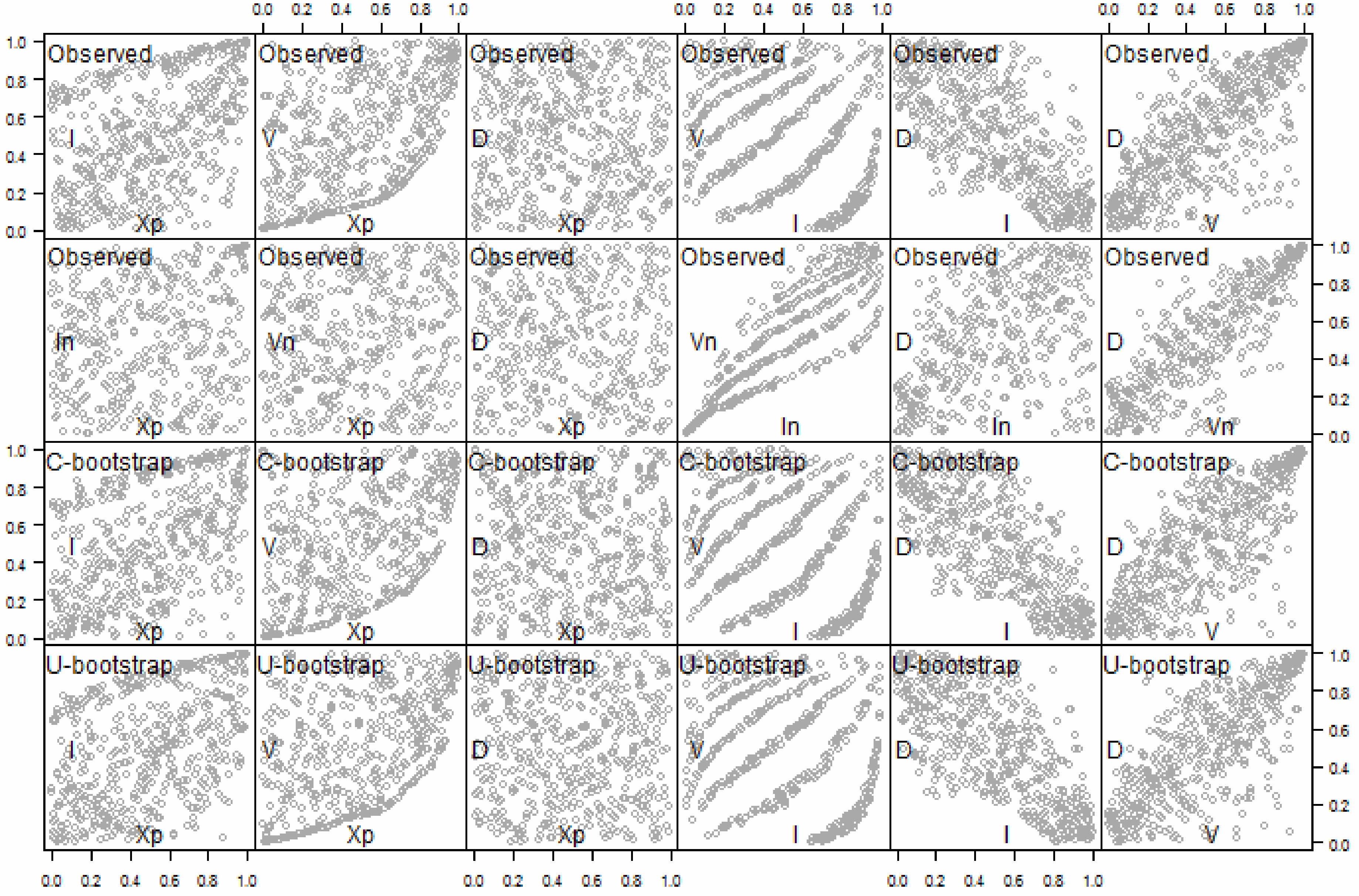

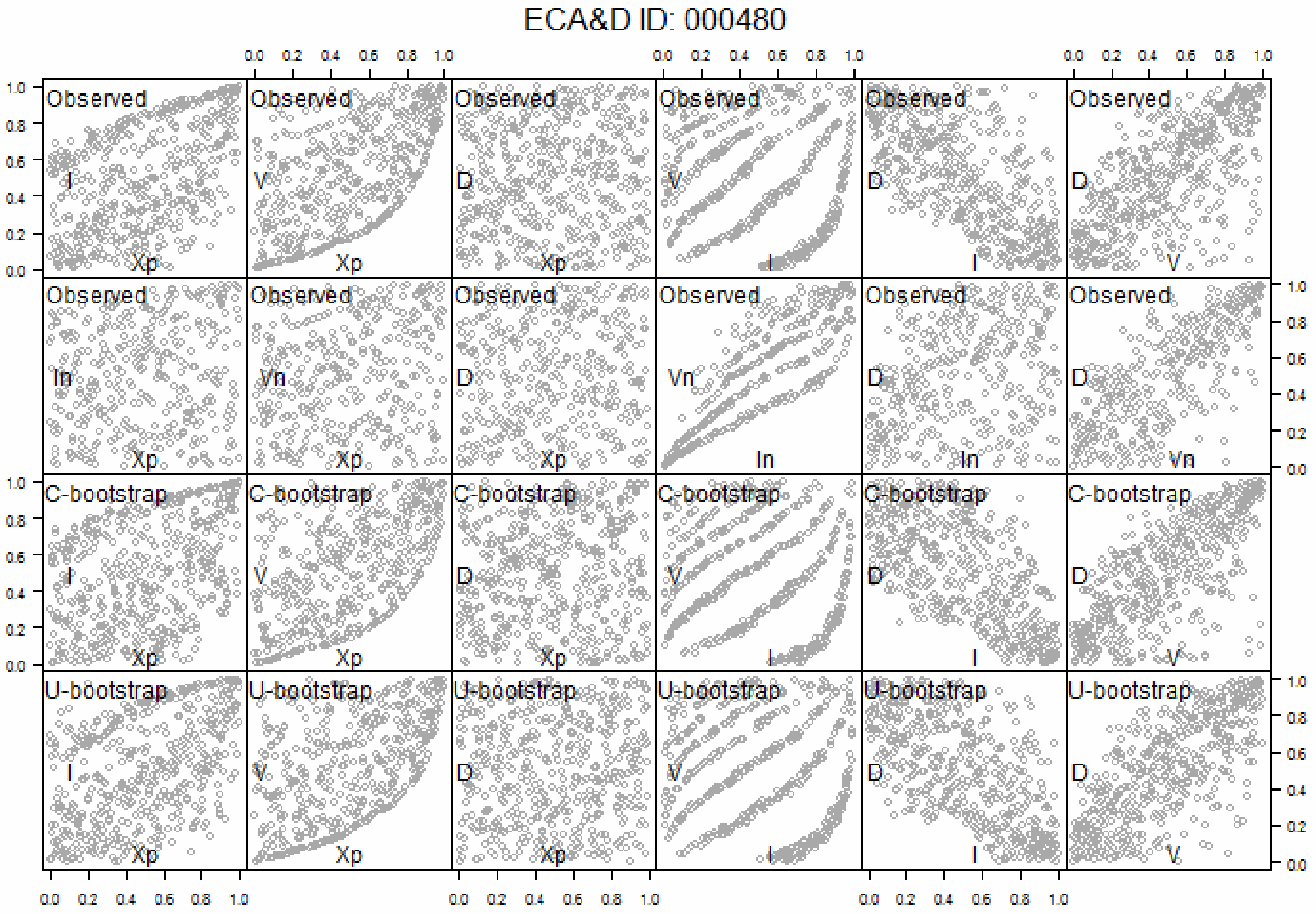
ECA&D ID: 000480
Observed
C-bootstrap
U-bootstrap
Xp
I
V
D
In
Vn
0.0
0.2
0.4
0.6
0.8
1.0

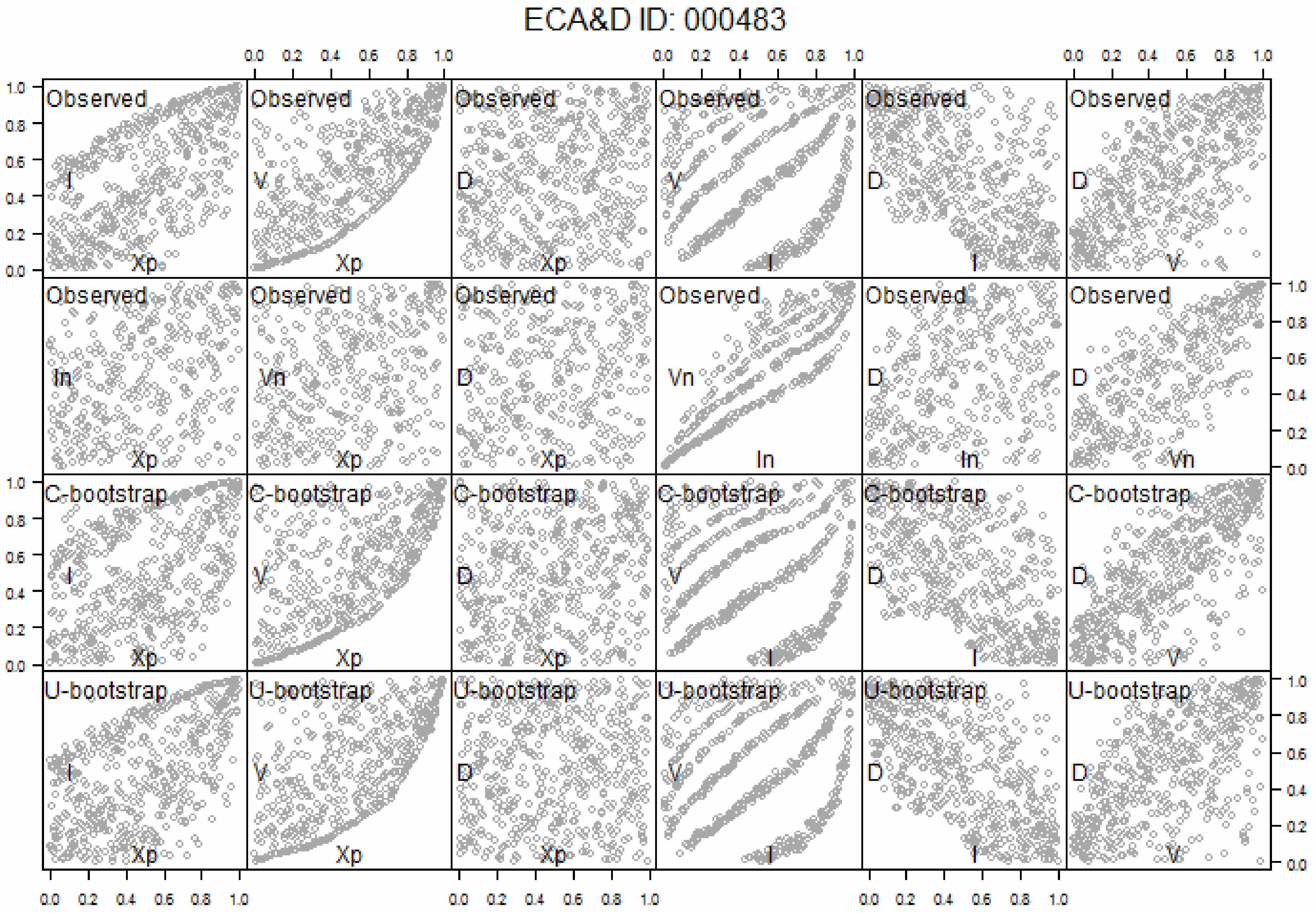

ECA&D ID: 000483
Observed
C-bootstrap
U-bootstrap
I
V
D
Xp
In
Vn
0.0
0.2
0.4
0.6
0.8
1.0

ECA&D ID: 000484

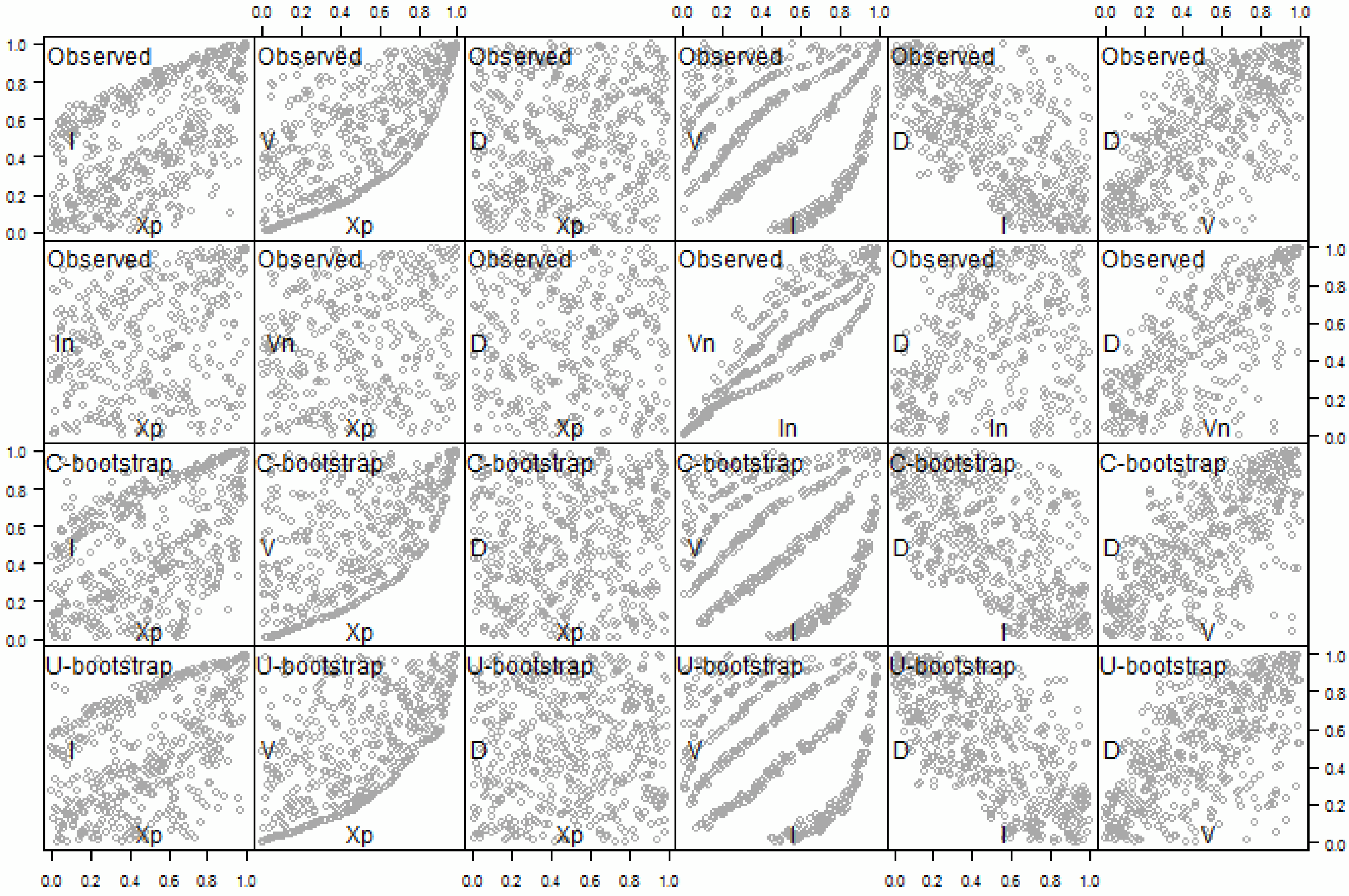

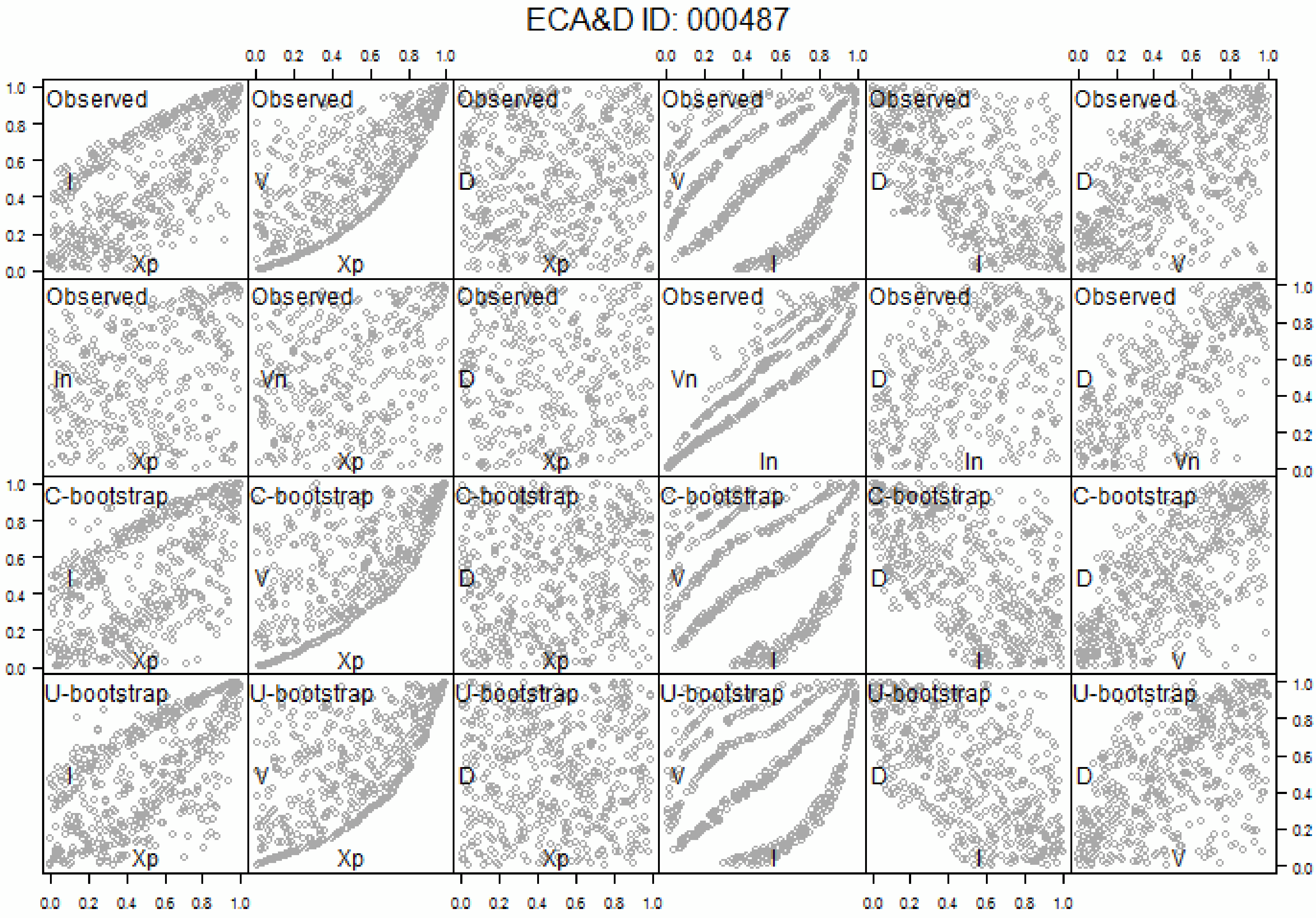
ECA&D ID: 000487
Observed
C-bootstrap
U-bootstrap
Xp
I
V
D
In
Vn
0.0
0.2
0.4
0.6
0.8
1.0

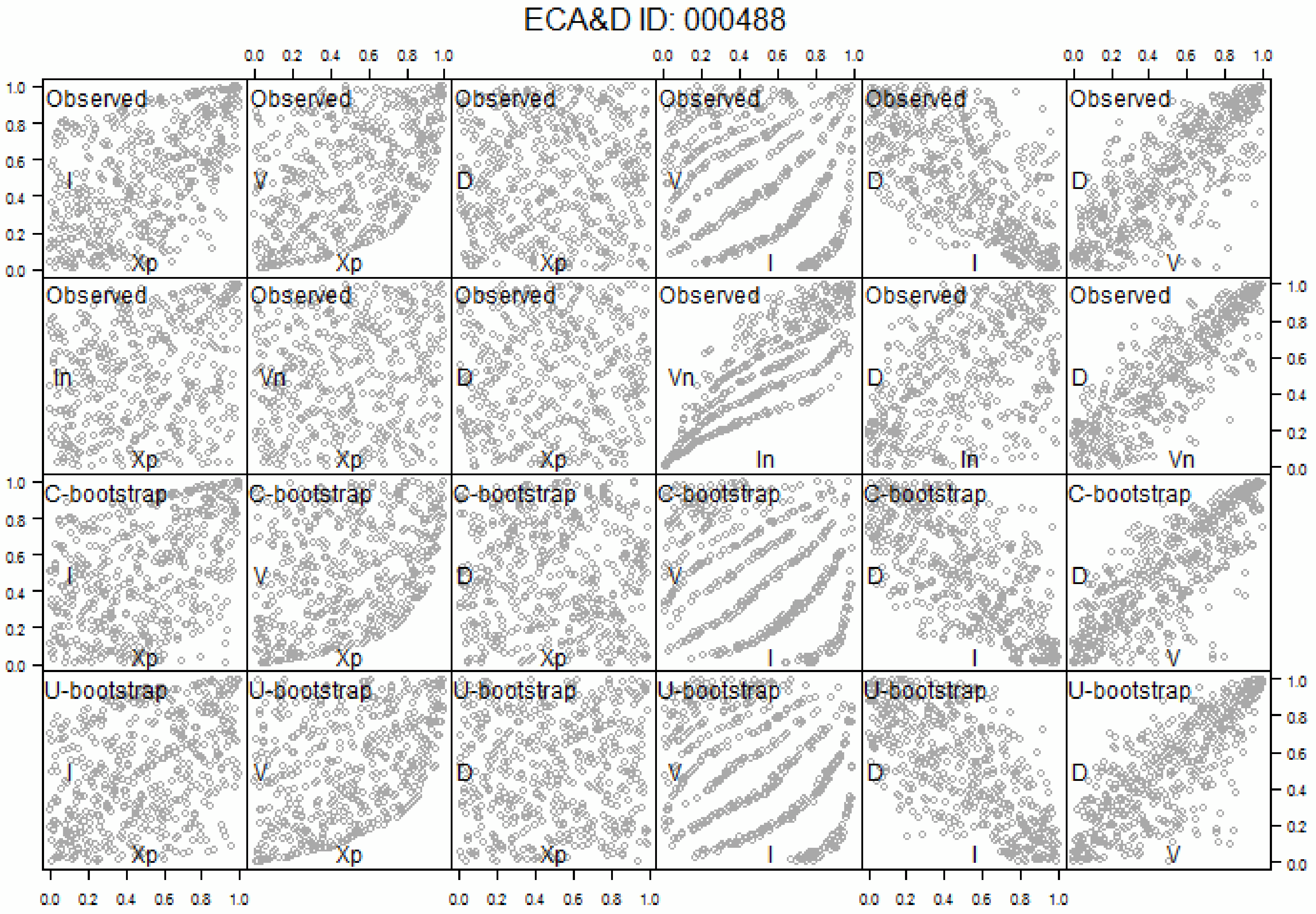
ECA&D ID: 000488
Observed
C-bootstrap
U-bootstrap
Xp
I
V
D
In
Vn
0.0
0.2
0.4
0.6
0.8
1.0

ECA&D ID: 000489

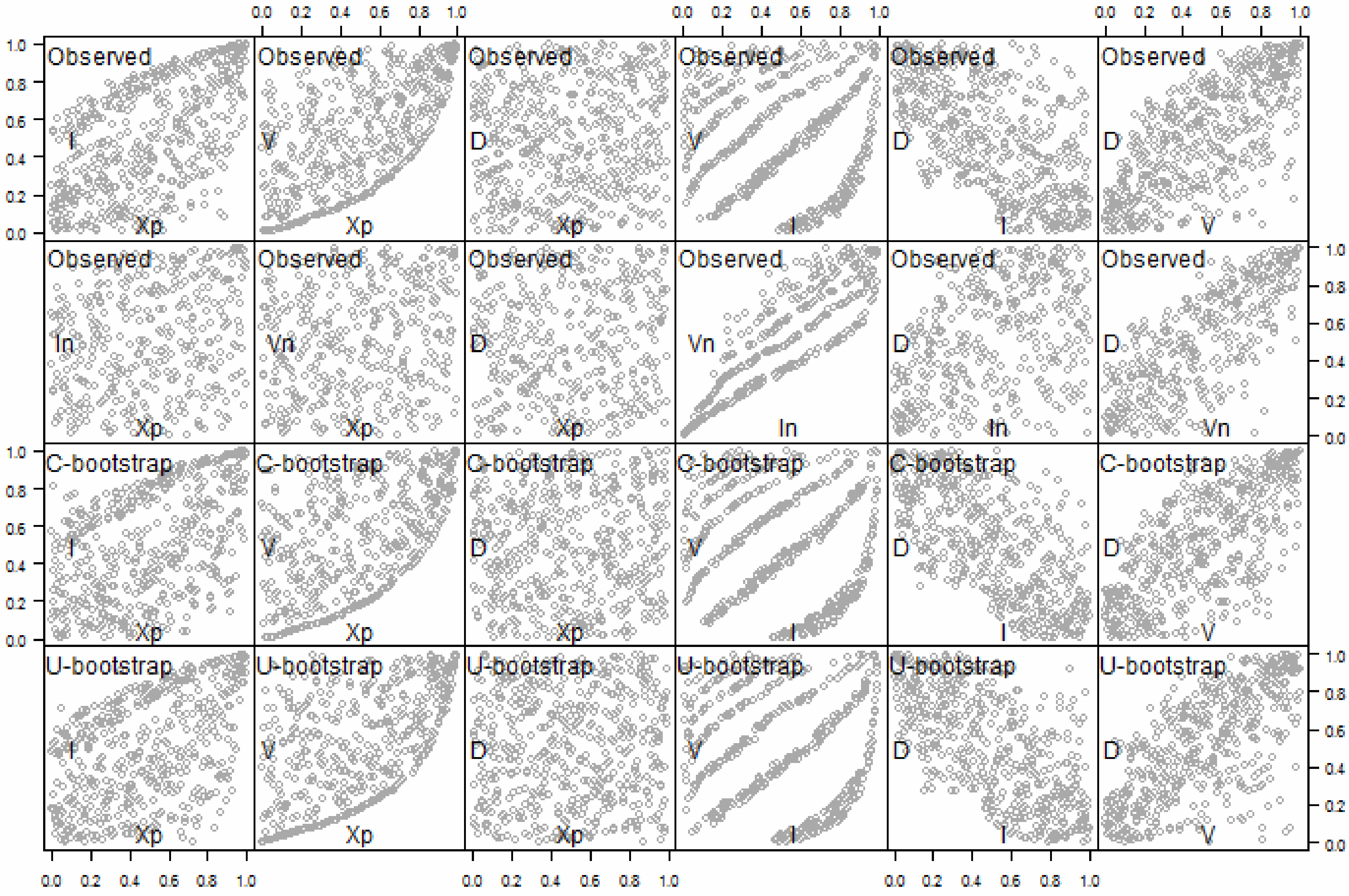

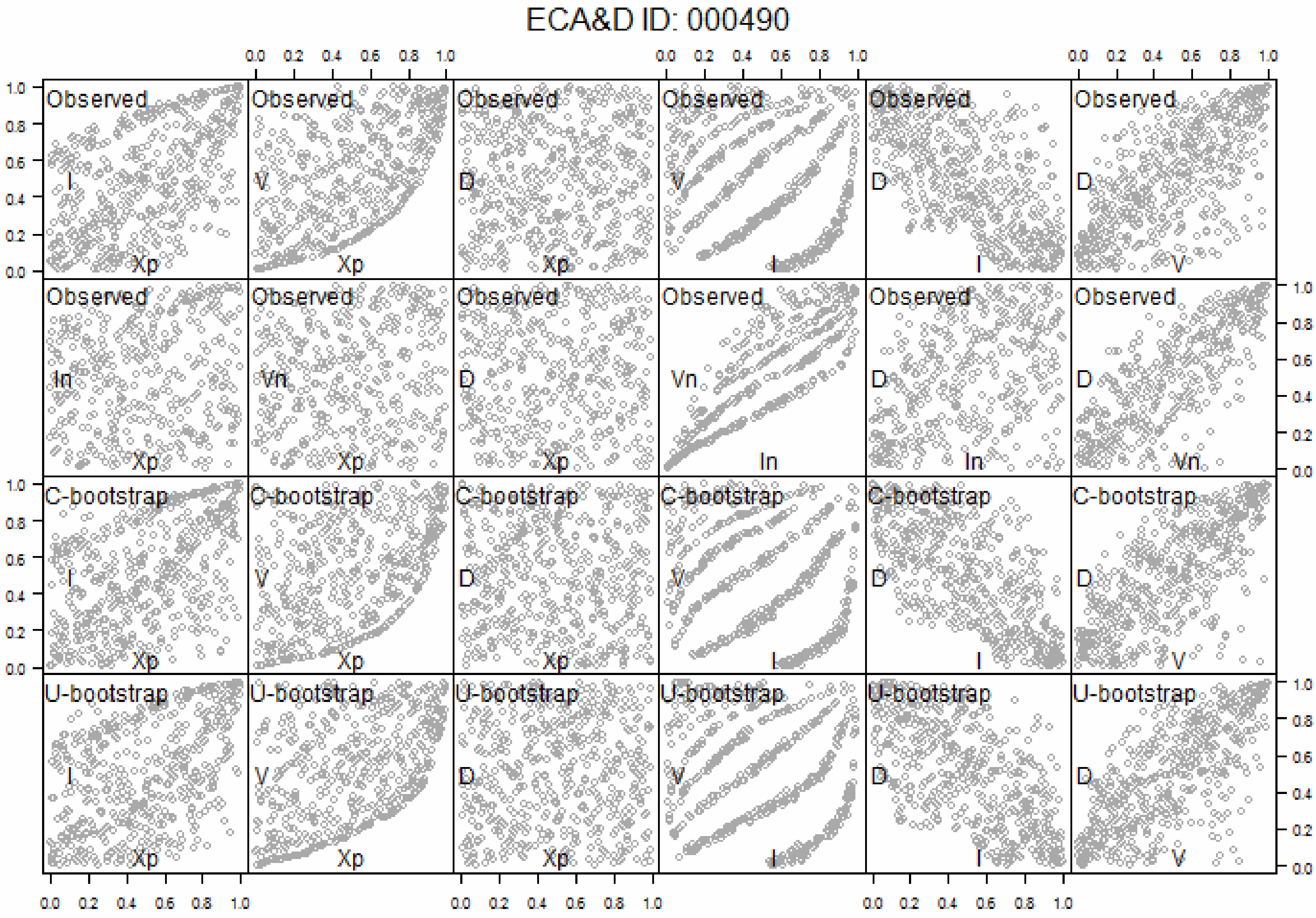

ECA&D ID: 000490
Observed
C-bootstrap
U-bootstrap
Xp
I
V
D
In
Vn
0.0
0.2
0.4
0.6
0.8
1.0

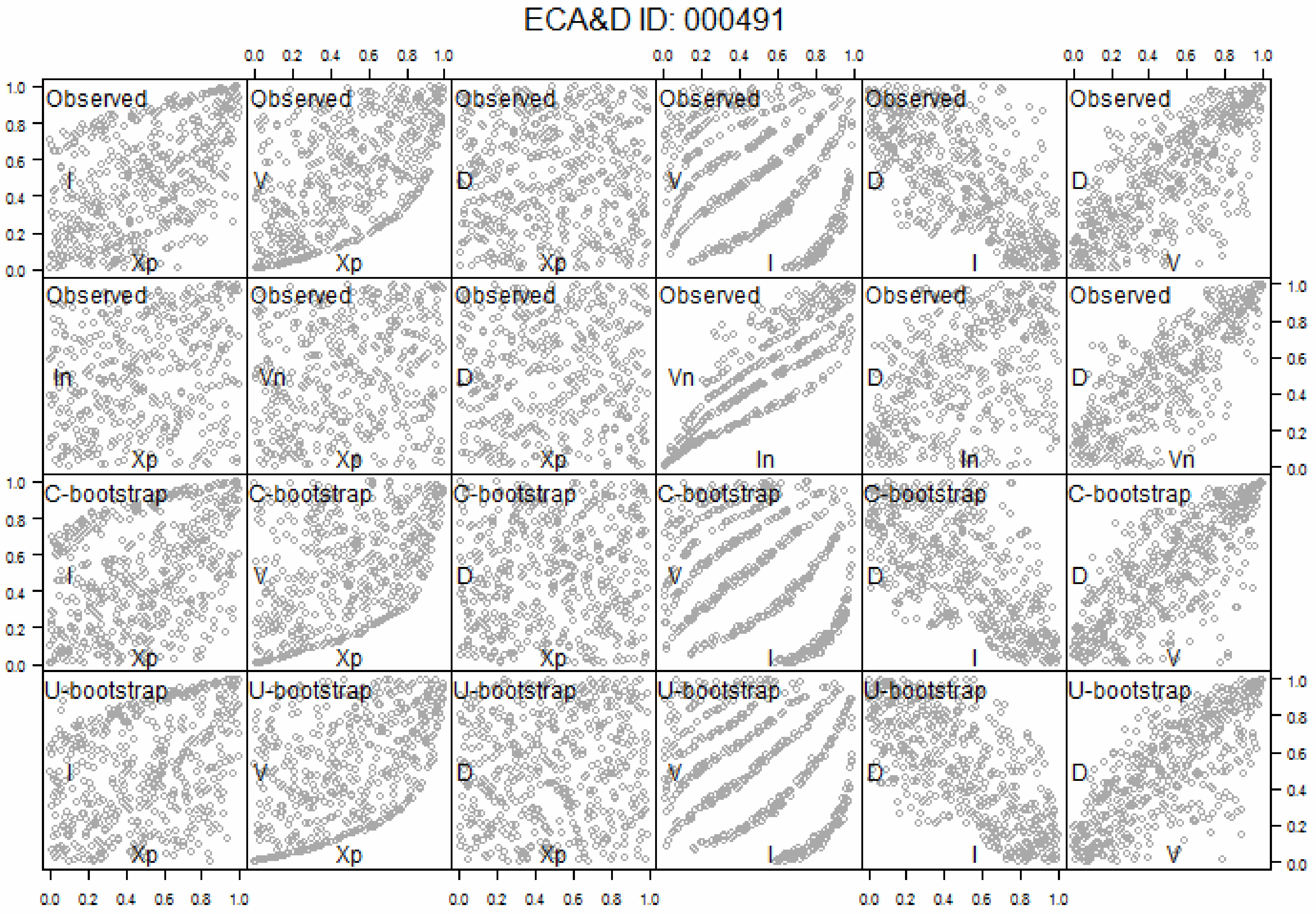
ECA&D ID: 000491
Observed
C-bootstrap
U-bootstrap
Xp
I
V
D
In
Vn
0.0
0.2
0.4
0.6
0.8
1.0

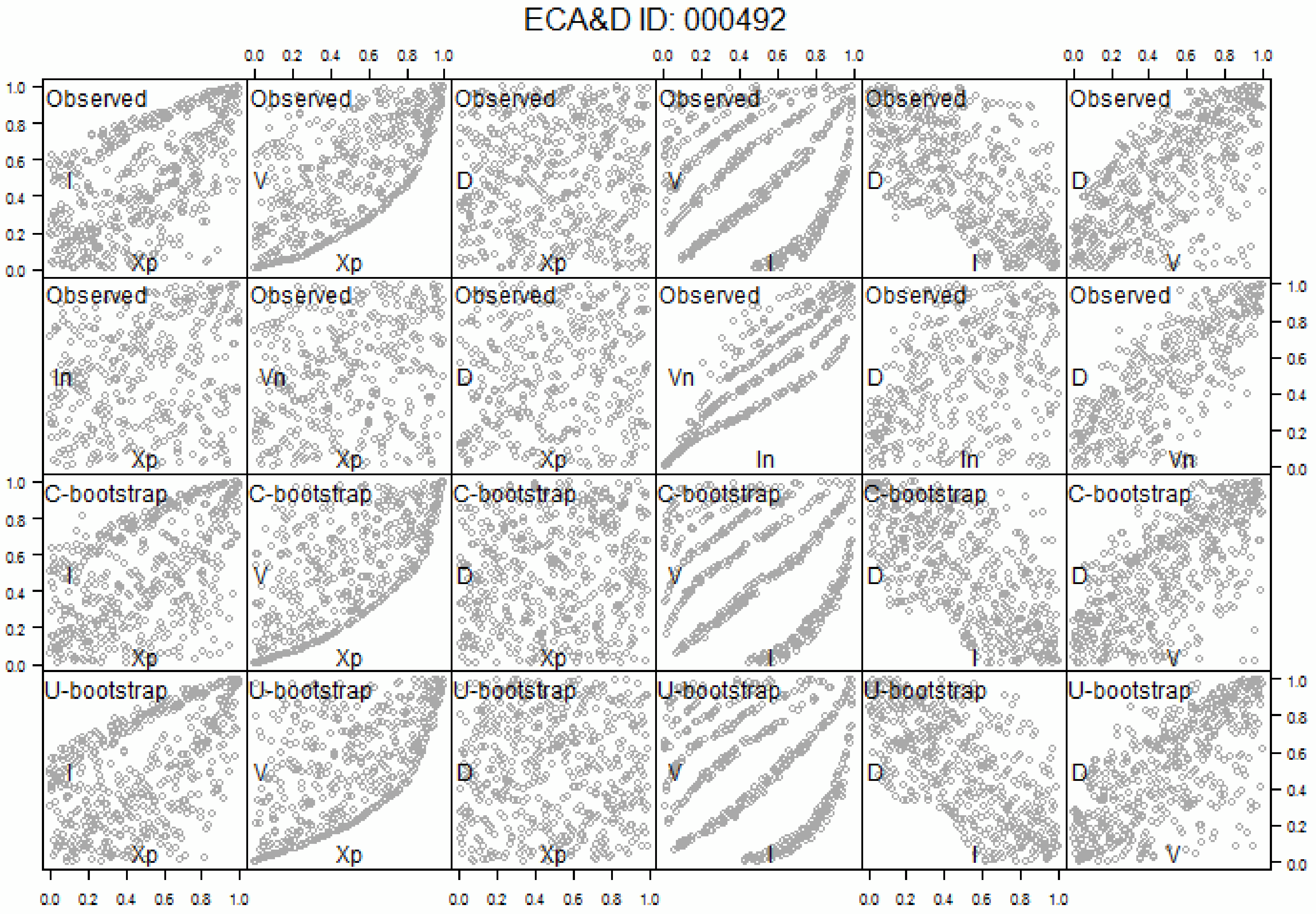
ECA&D ID: 000492
Observed
C-bootstrap
U-bootstrap
Xp
I
V
D
In
Vn
0.0
0.2
0.4
0.6
0.8
1.0

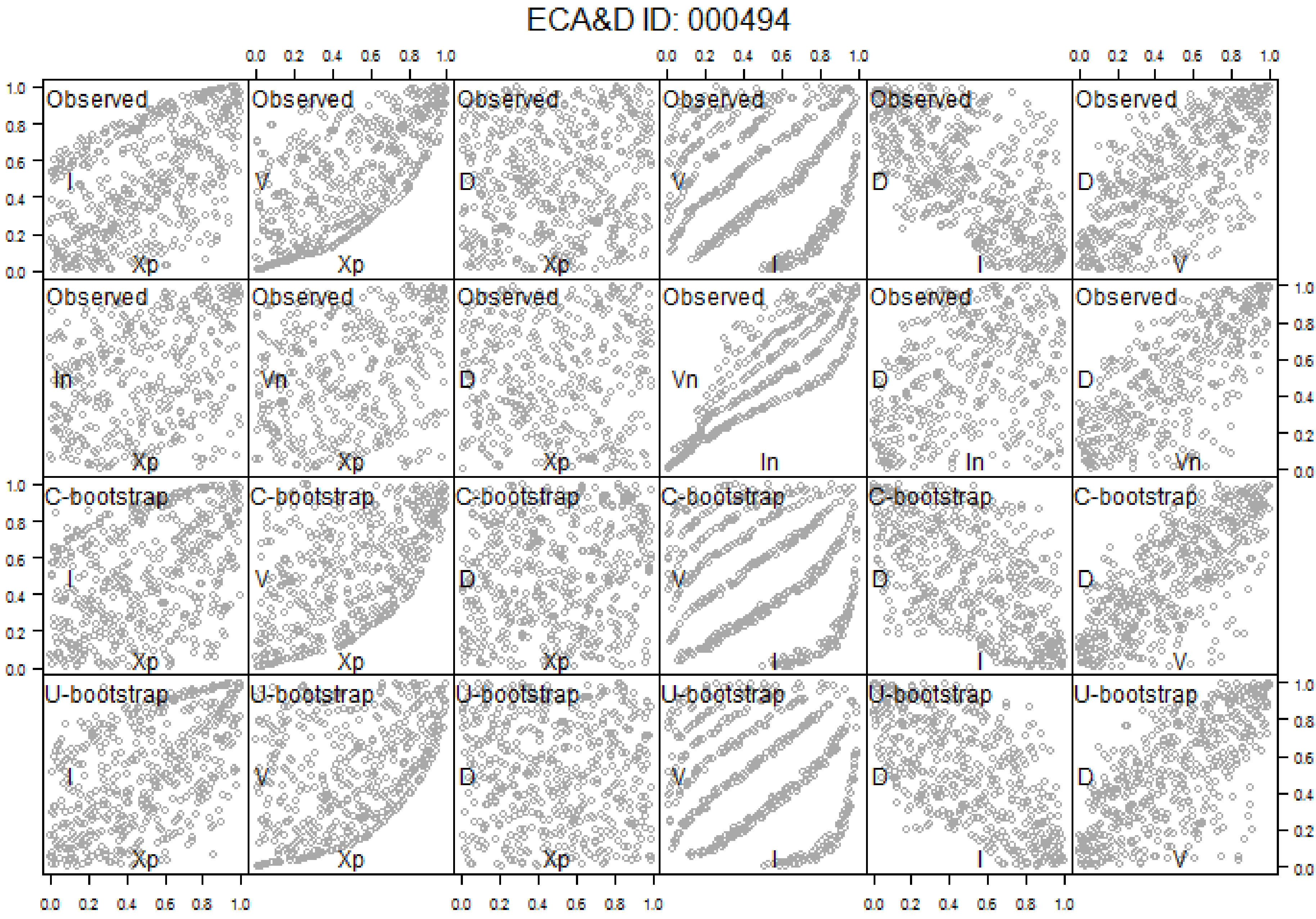

ECA&D ID: 000494
Observed
C-bootstrap
U-bootstrap
Xp
I
V
D
In
Vn
0.0
0.2
0.4
0.6
0.8
1.0

ECA&D ID: 000496

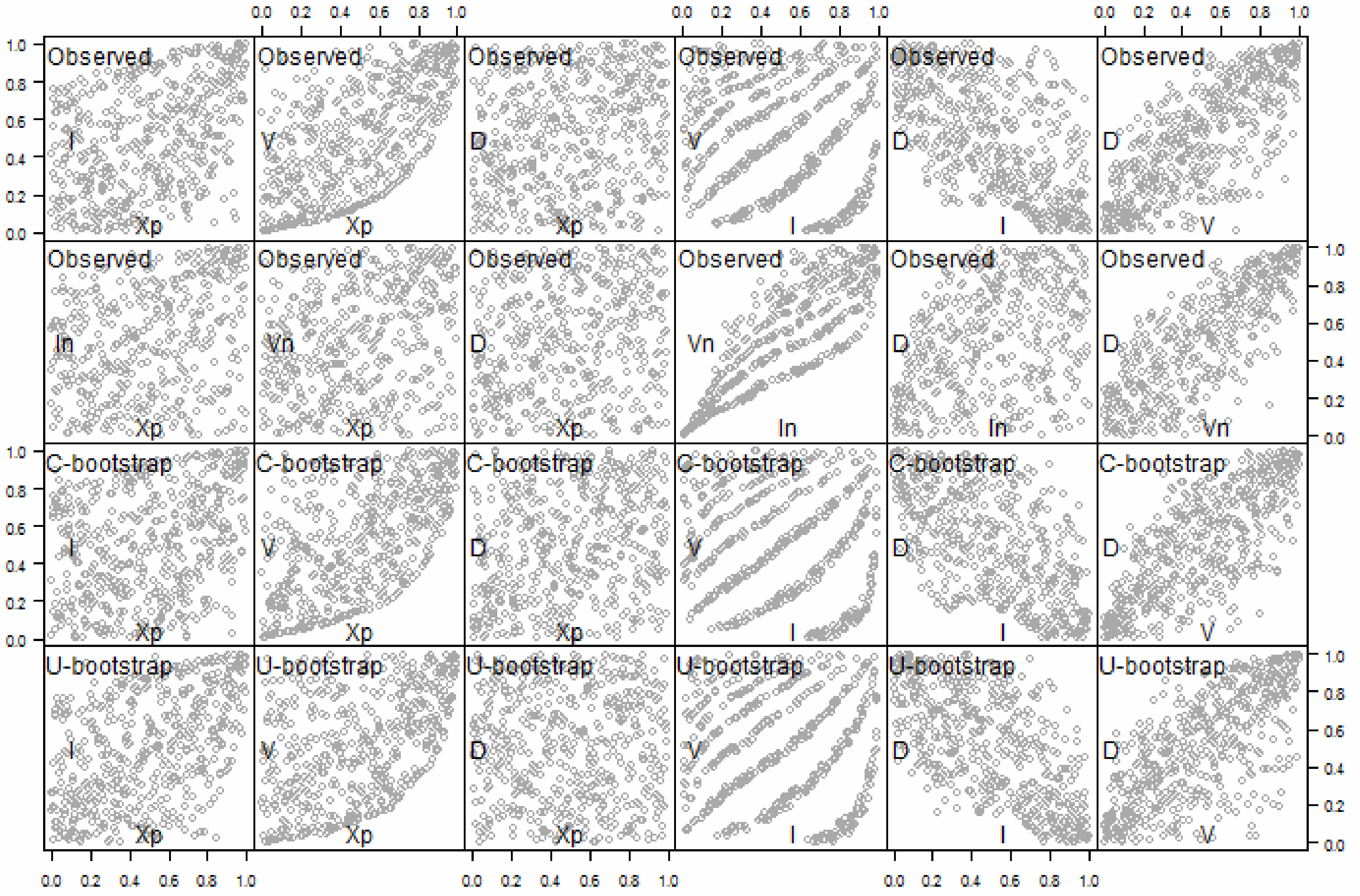

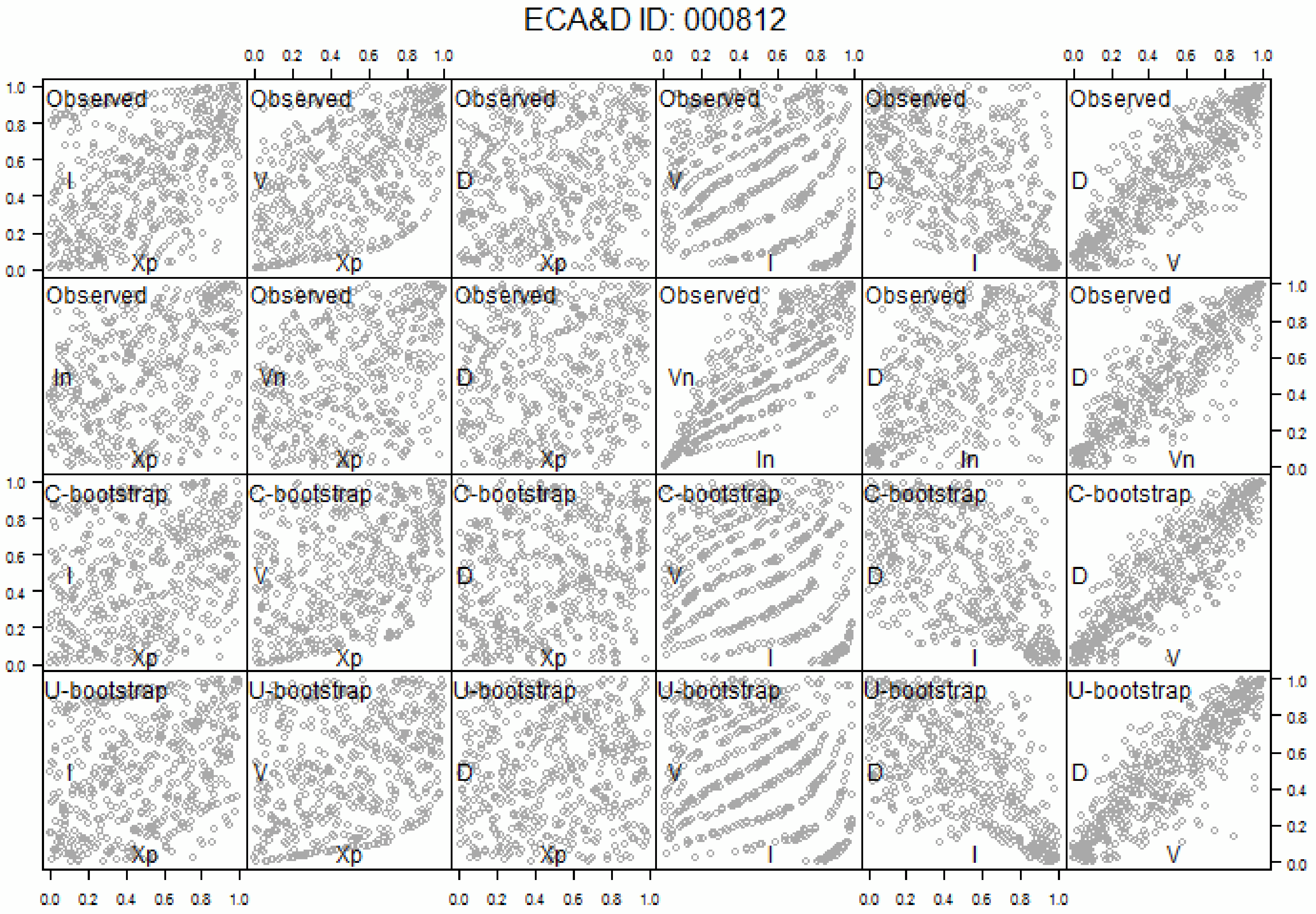
ECA&D ID: 000812
Observed
C-bootstrap
U-bootstrap
Xp
I
V
D
In
Vn
0.0
0.2
0.4
0.6
0.8
1.0

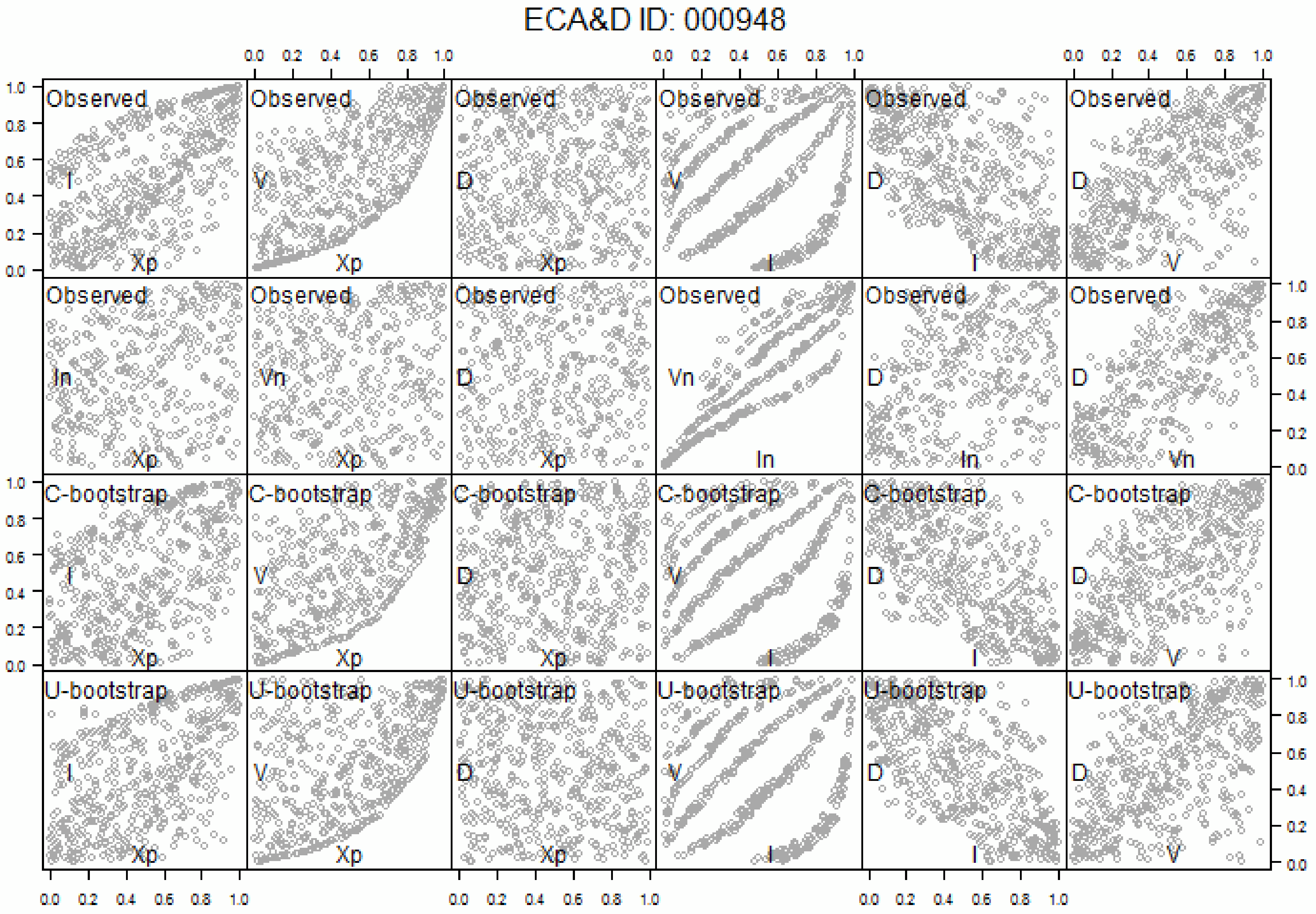
ECA&D ID: 000948
Observed
C-bootstrap
U-bootstrap
Xp
I
V
D
In
Vn
0.0
0.2
0.4
0.6
0.8
1.0

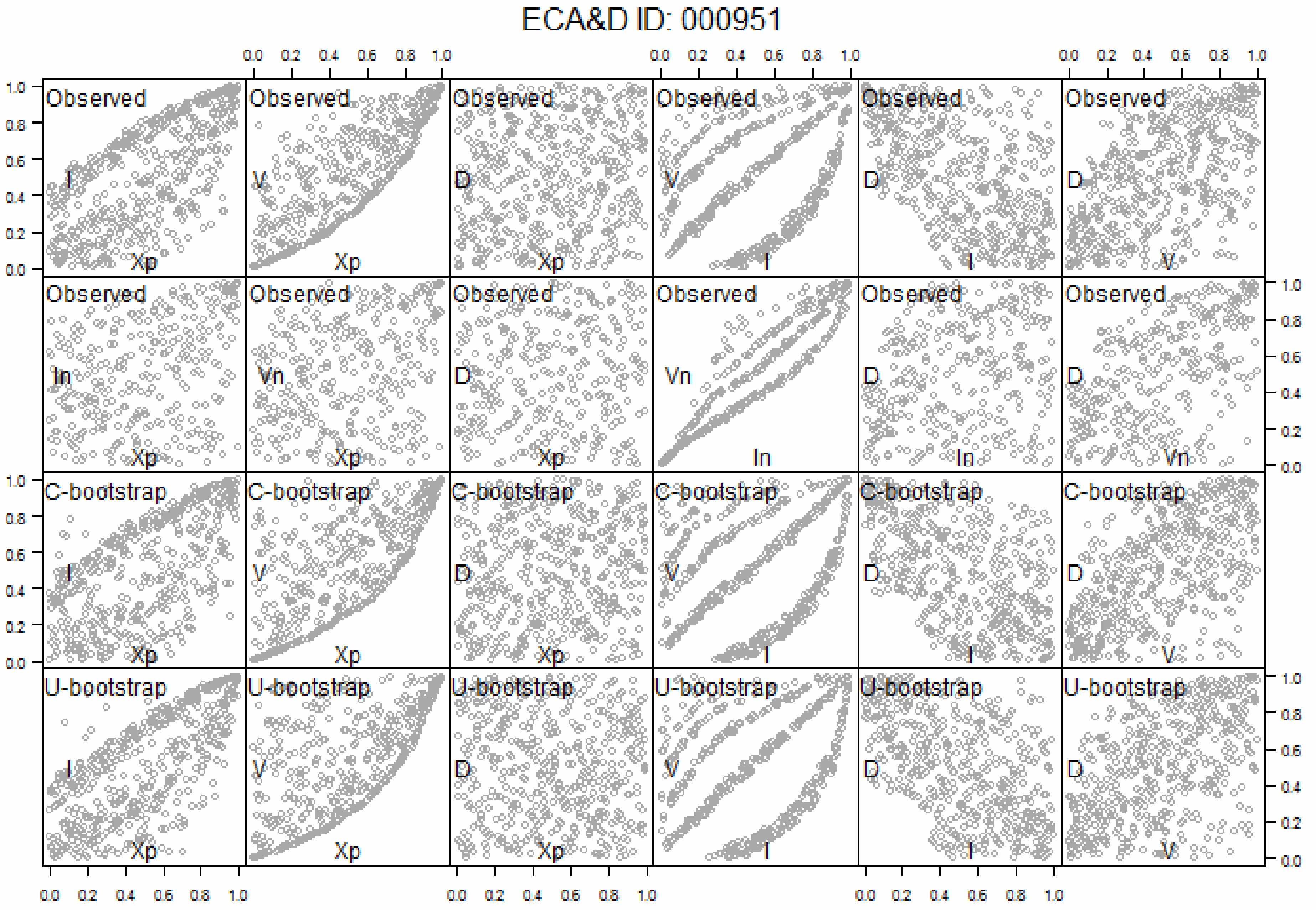
ECA&D ID: 000951
Observed
C-bootstrap
U-bootstrap
Xp
I
V
D
In
Vn

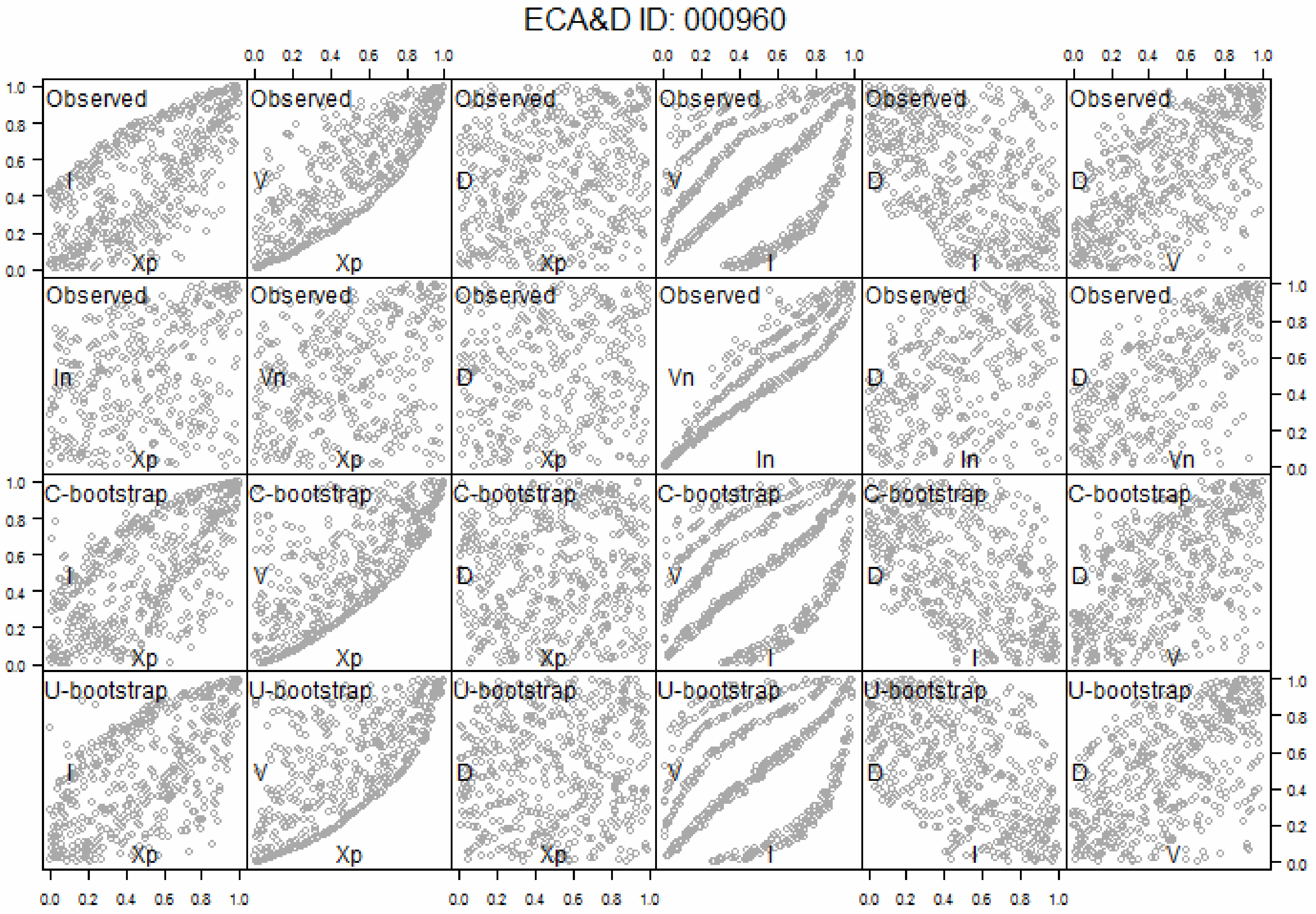
ECA&D ID: 000960
Observed
C-bootstrap
U-bootstrap
Xp
I
V
D
In
Vn
0.0
0.2
0.4
0.6
0.8
1.0

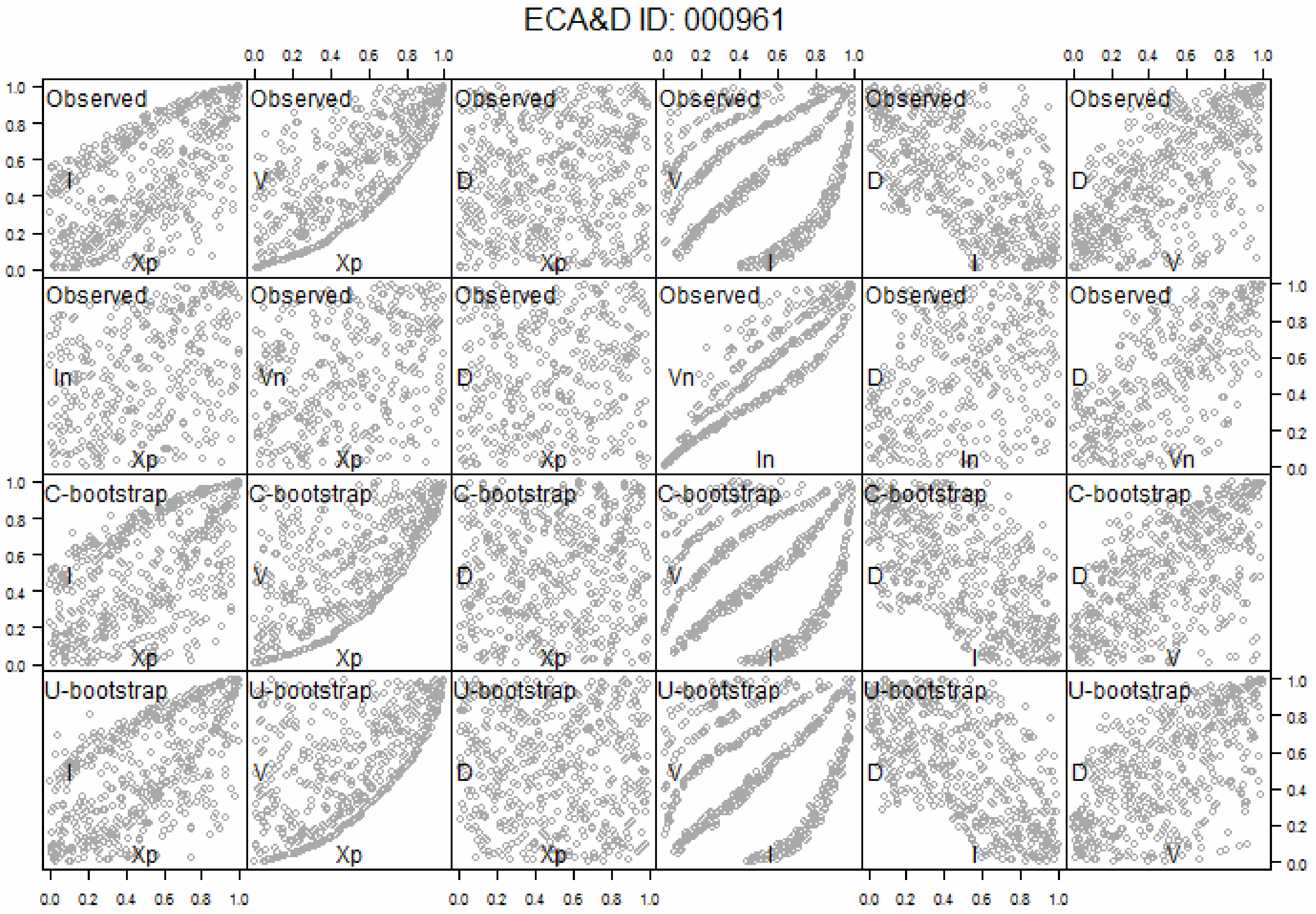
ECA&D ID: 000961
Observed
C-bootstrap
U-bootstrap
Xp
I
V
D
In
Vn
0.0
0.2
0.4
0.6
0.8
1.0

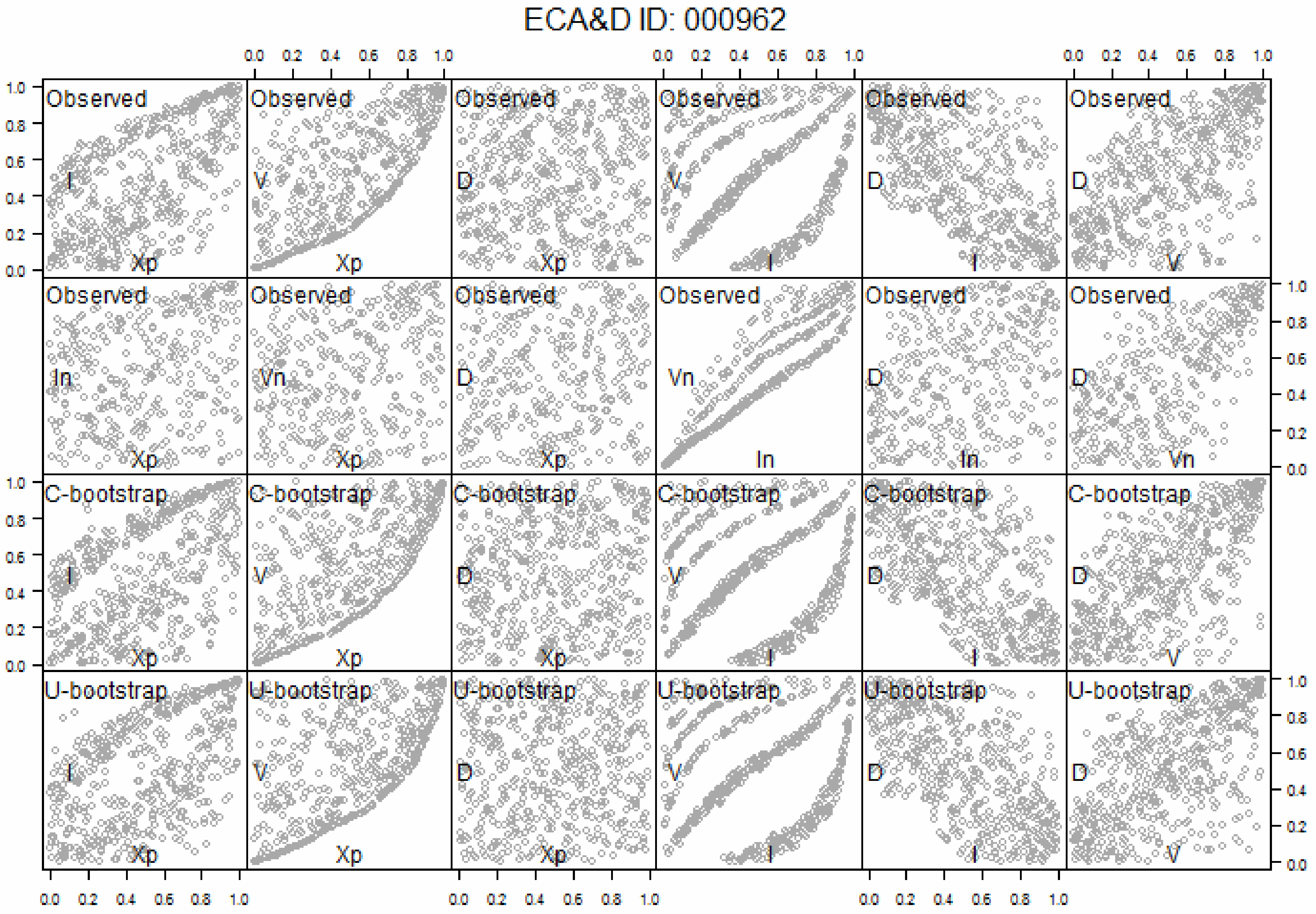
ECA&D ID: 000962
Observed
C-bootstrap
U-bootstrap
Xp
I
V
D
In
Vn
0.0
0.2
0.4
0.6
0.8
1.0

ECA&D ID: 000967

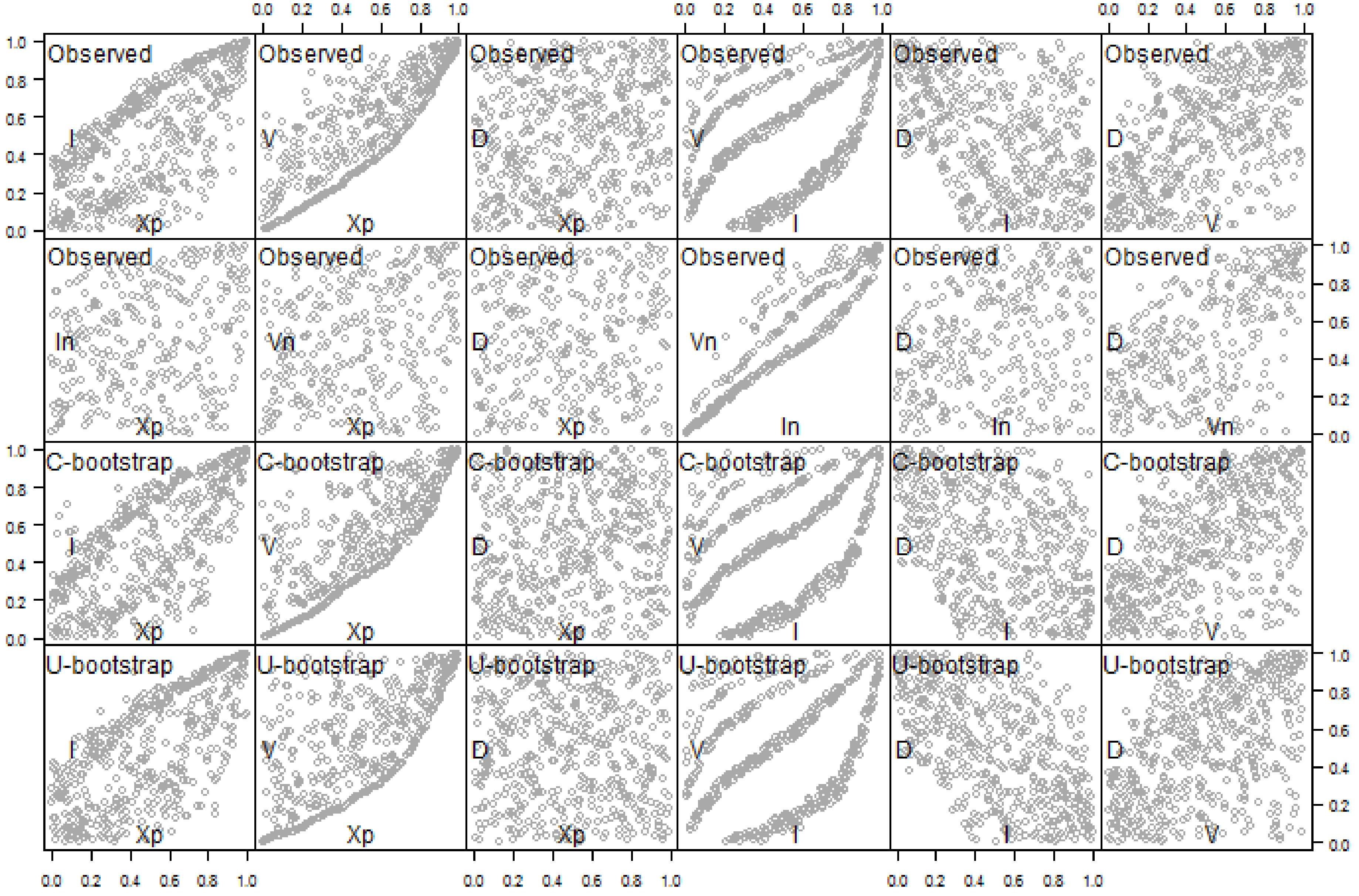

ECA&D ID: 000972

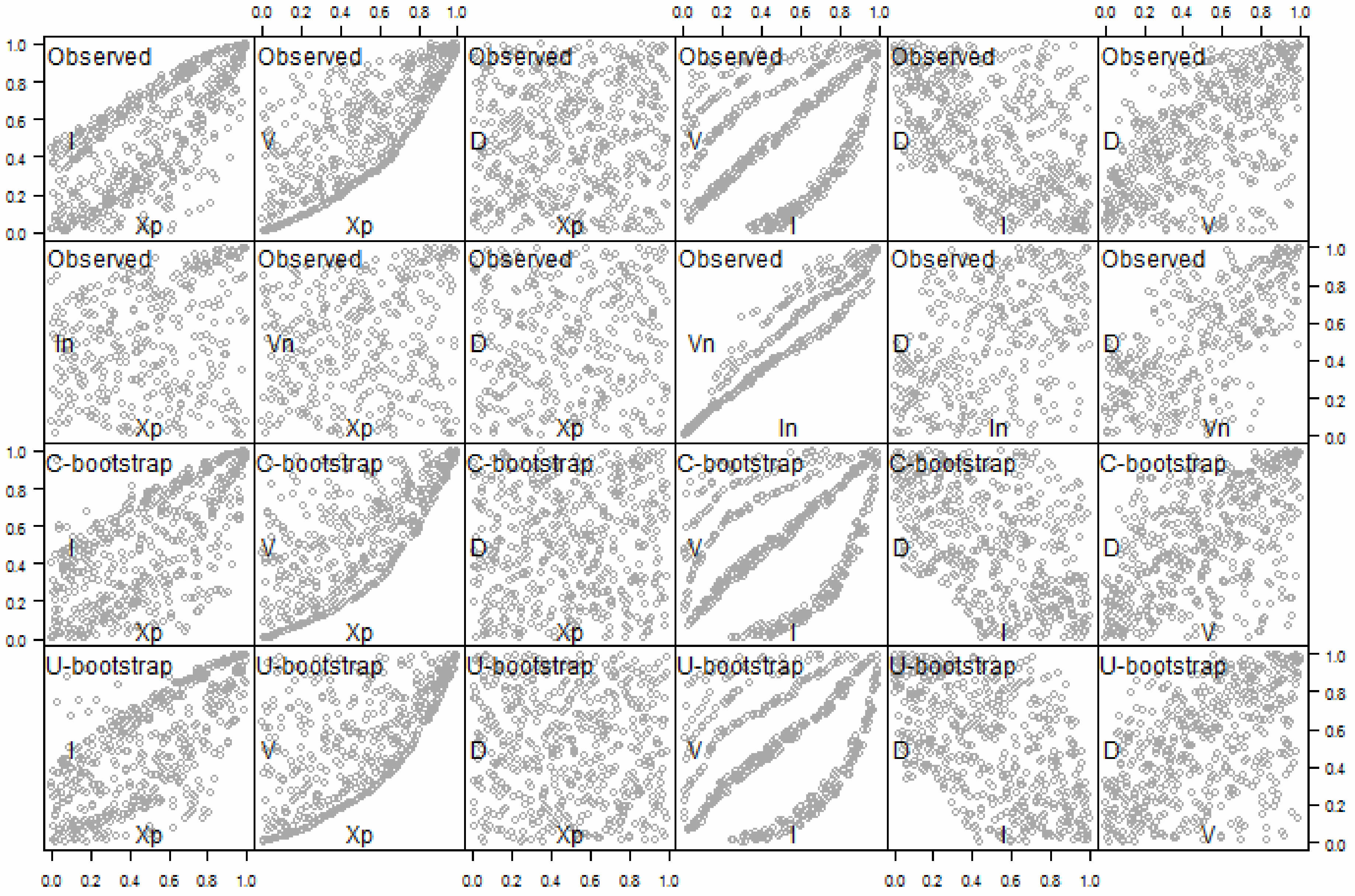

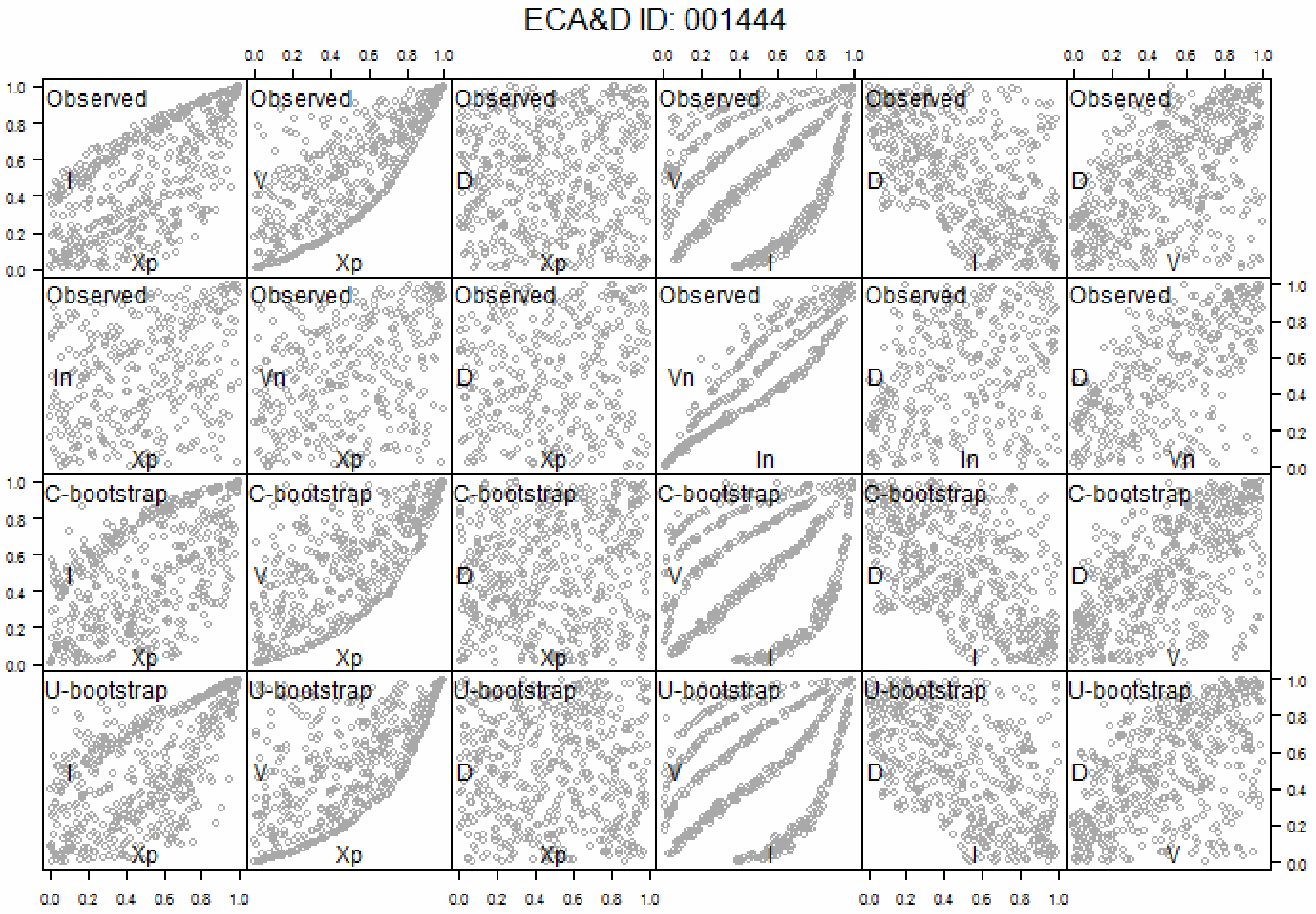
ECA&D ID: 001444
Observed
C-bootstrap
U-bootstrap
Xp
I
V
D
In
Vn
0.0
0.2
0.4
0.6
0.8
1.0

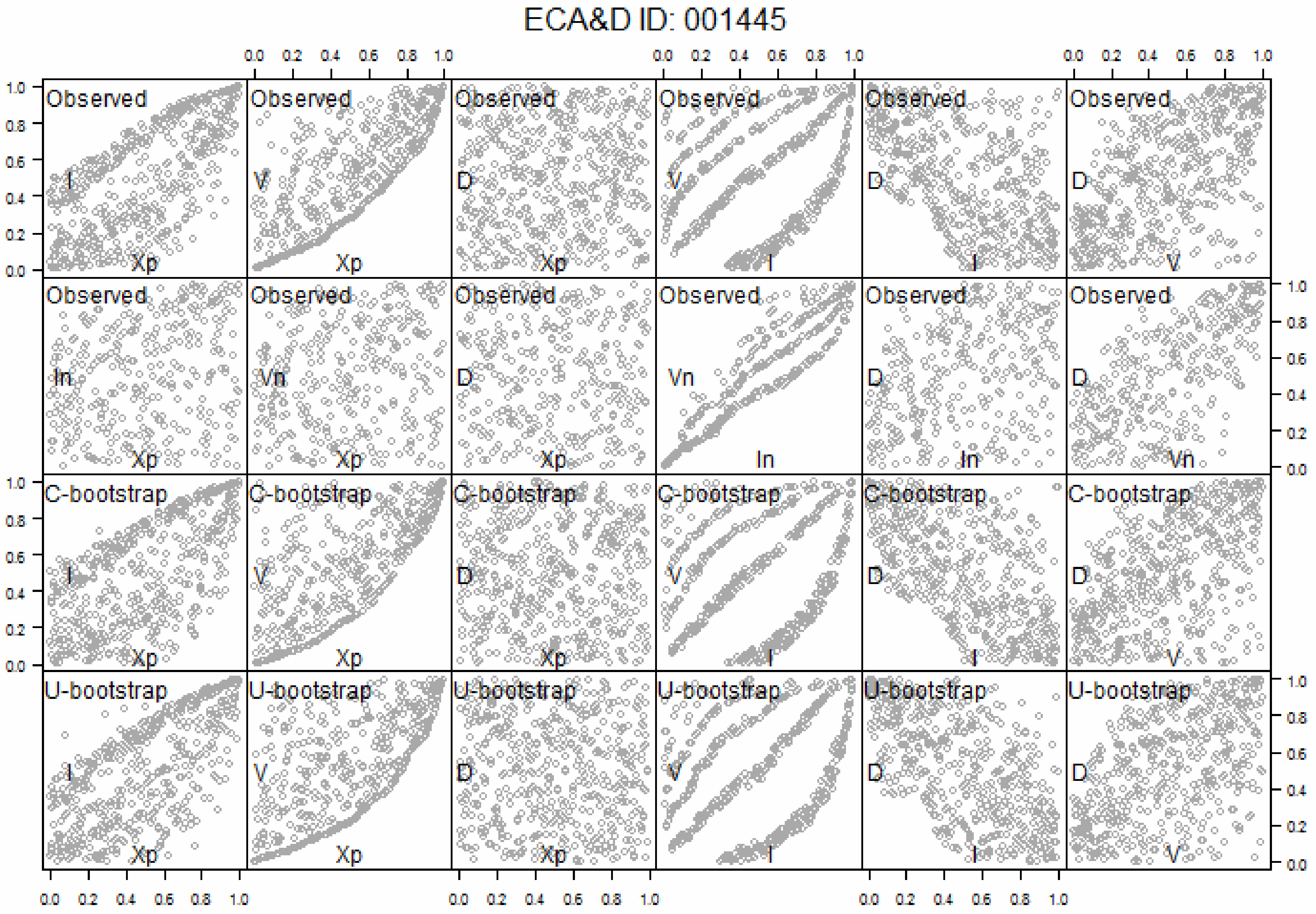
ECA&D ID: 001445
Observed
C-bootstrap
U-bootstrap
Xp
I
V
D
In
Vn
0.0
0.2
0.4
0.6
0.8
1.0

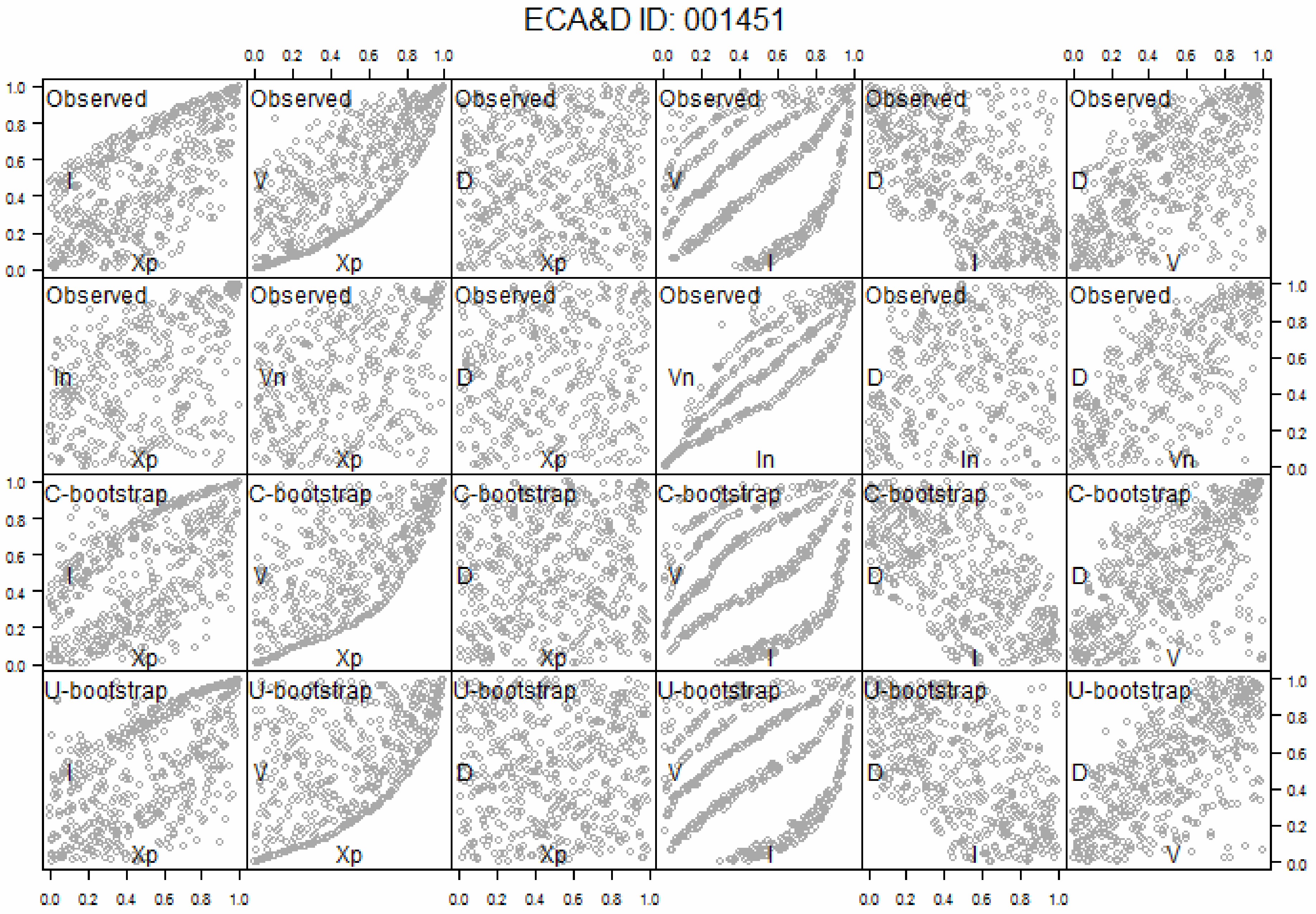
ECA&D ID: 001451
Observed
C-bootstrap
U-bootstrap
Xp
I
V
D
In
Vn
0.0
0.2
0.4
0.6
0.8
1.0

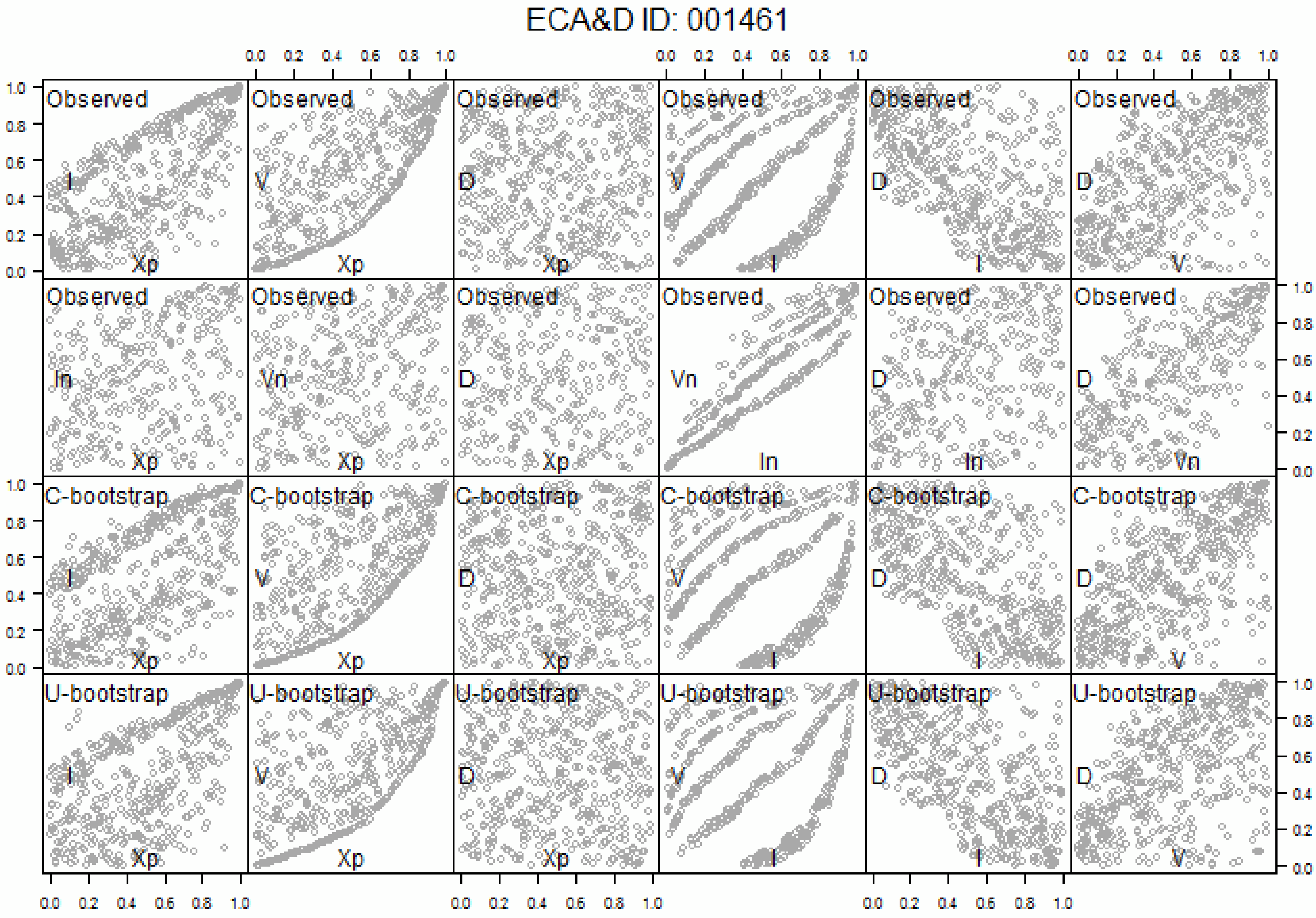

ECA&D ID: 001461
Observed
C-bootstrap
U-bootstrap
Xp
I
V
D
In
Vn
0.0
0.2
0.4
0.6
0.8
1.0

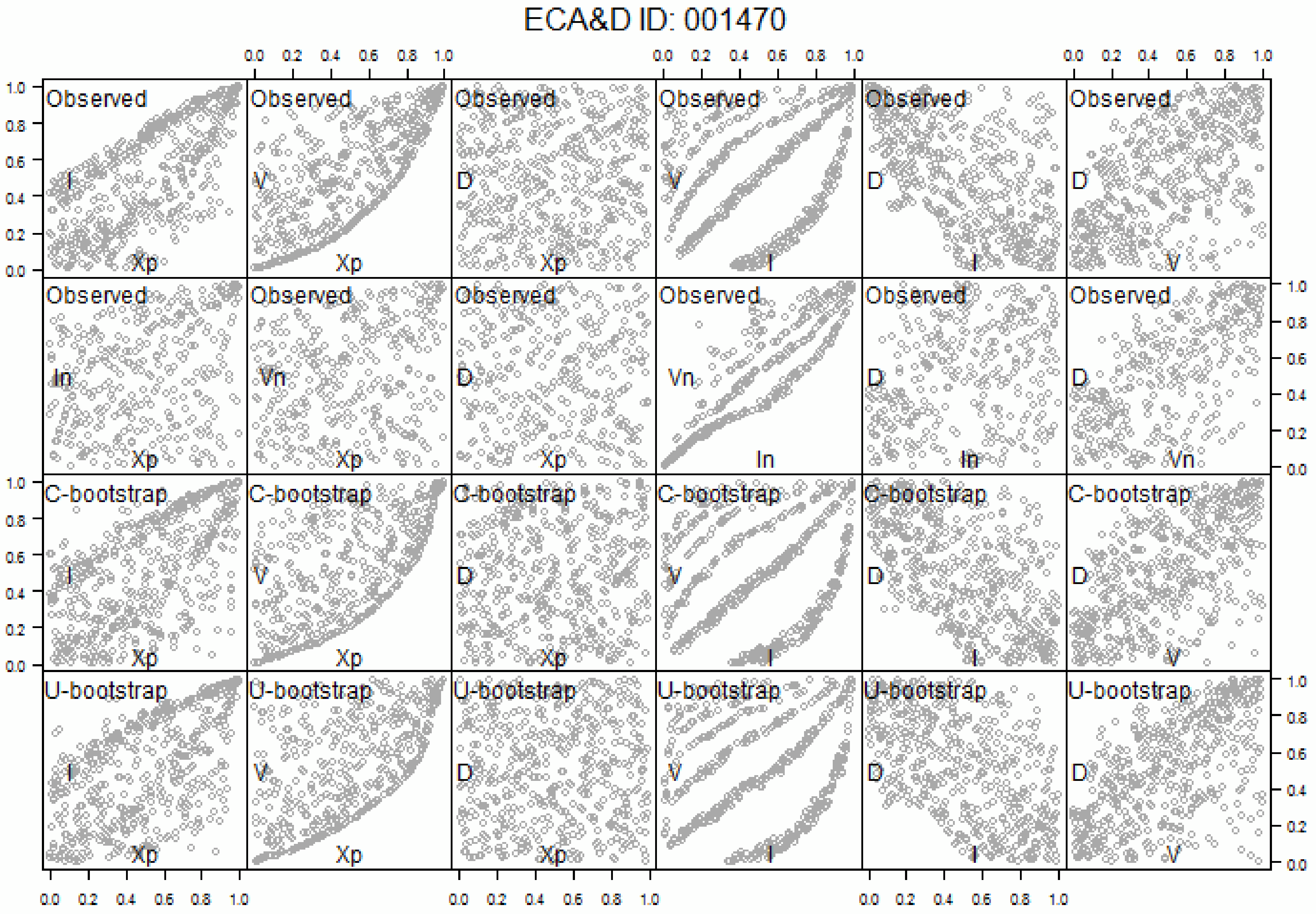

ECA&D ID: 001470
Observed
C-bootstrap
U-bootstrap
Xp
I
V
D
In
Vn
0.0
0.2
0.4
0.6
0.8
1.0

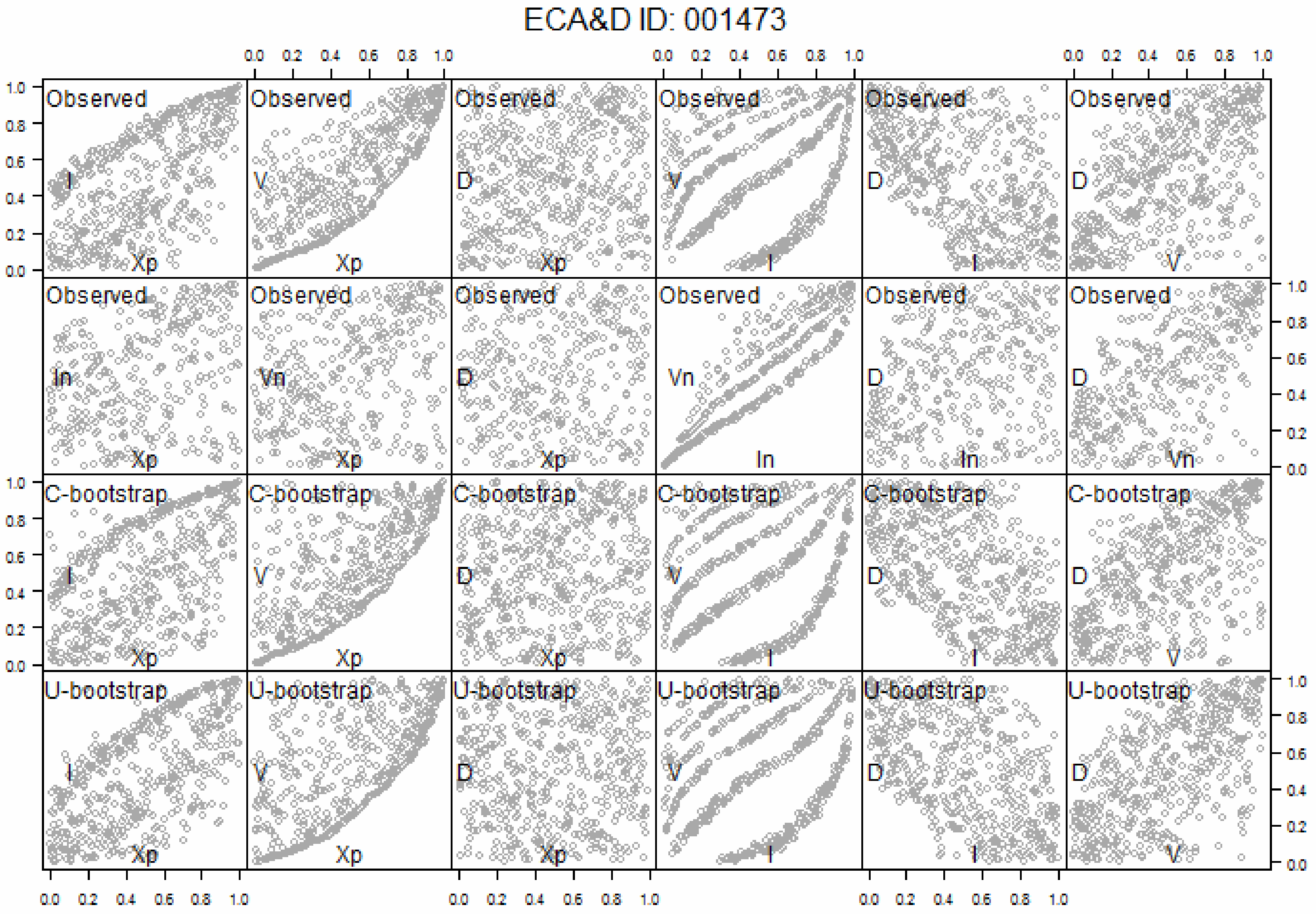
ECA&D ID: 001473
Observed
C-bootstrap
U-bootstrap
Xp
I
V
D
In
Vn
0.0
0.2
0.4
0.6
0.8
1.0

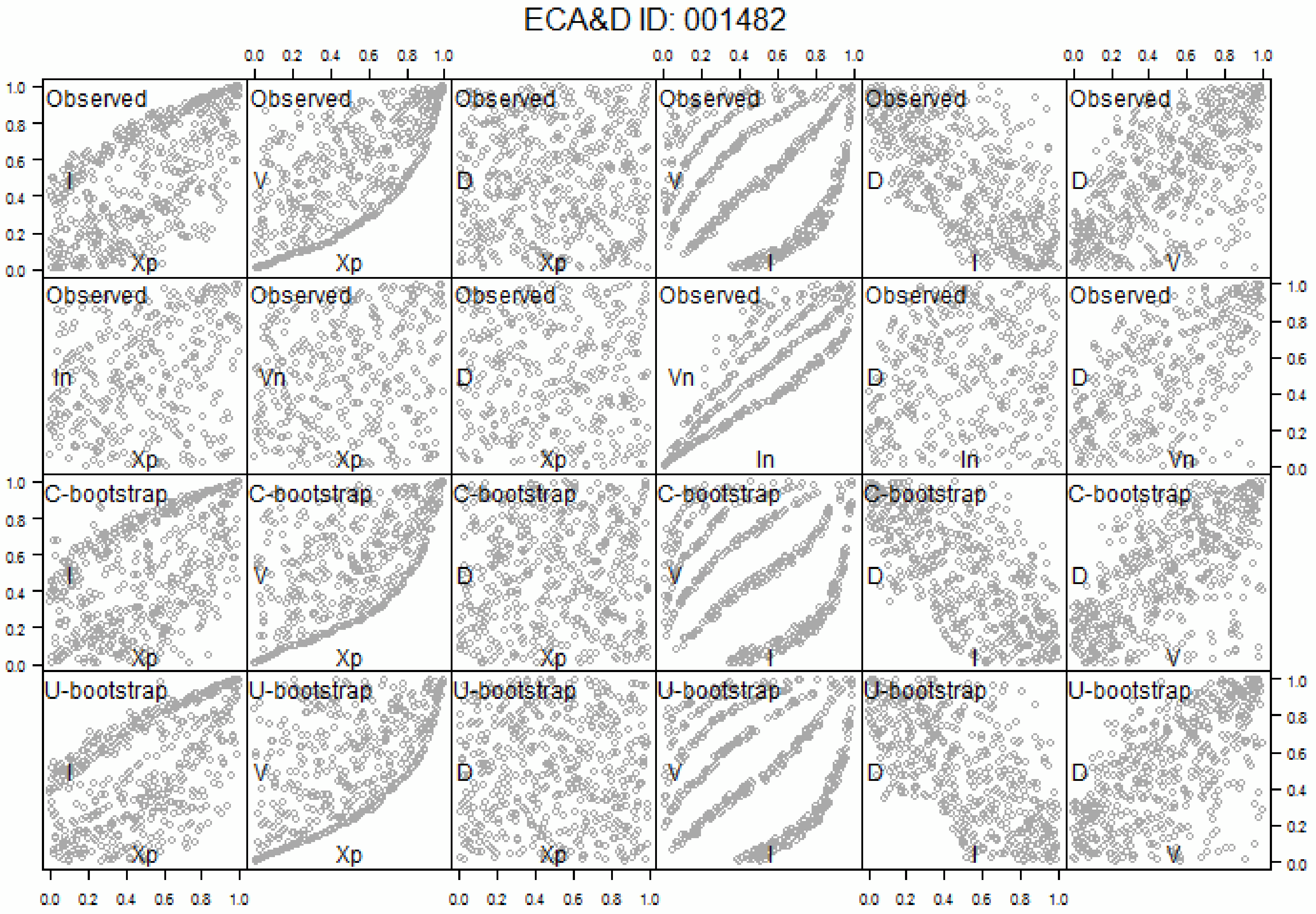

ECA&D ID: 001482
Observed
C-bootstrap
U-bootstrap
Xp
I
V
D
In
Vn
0.0
0.2
0.4
0.6
0.8
1.0

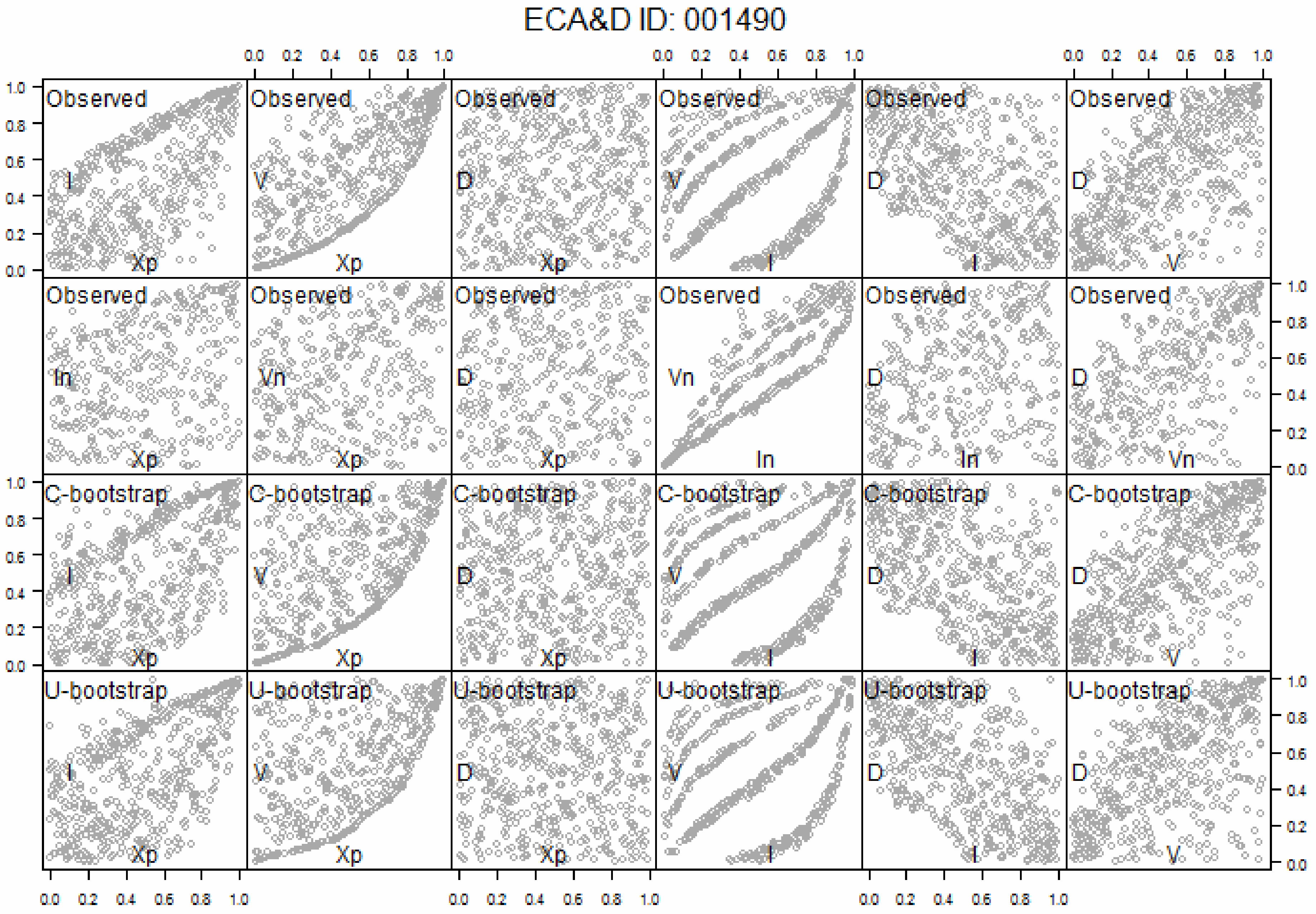

ECA&D ID: 001490
Observed
C-bootstrap
U-bootstrap
Xp
I
V
D
In
Vn
0.0
0.2
0.4
0.6
0.8
1.0

ECA&D ID: 001496

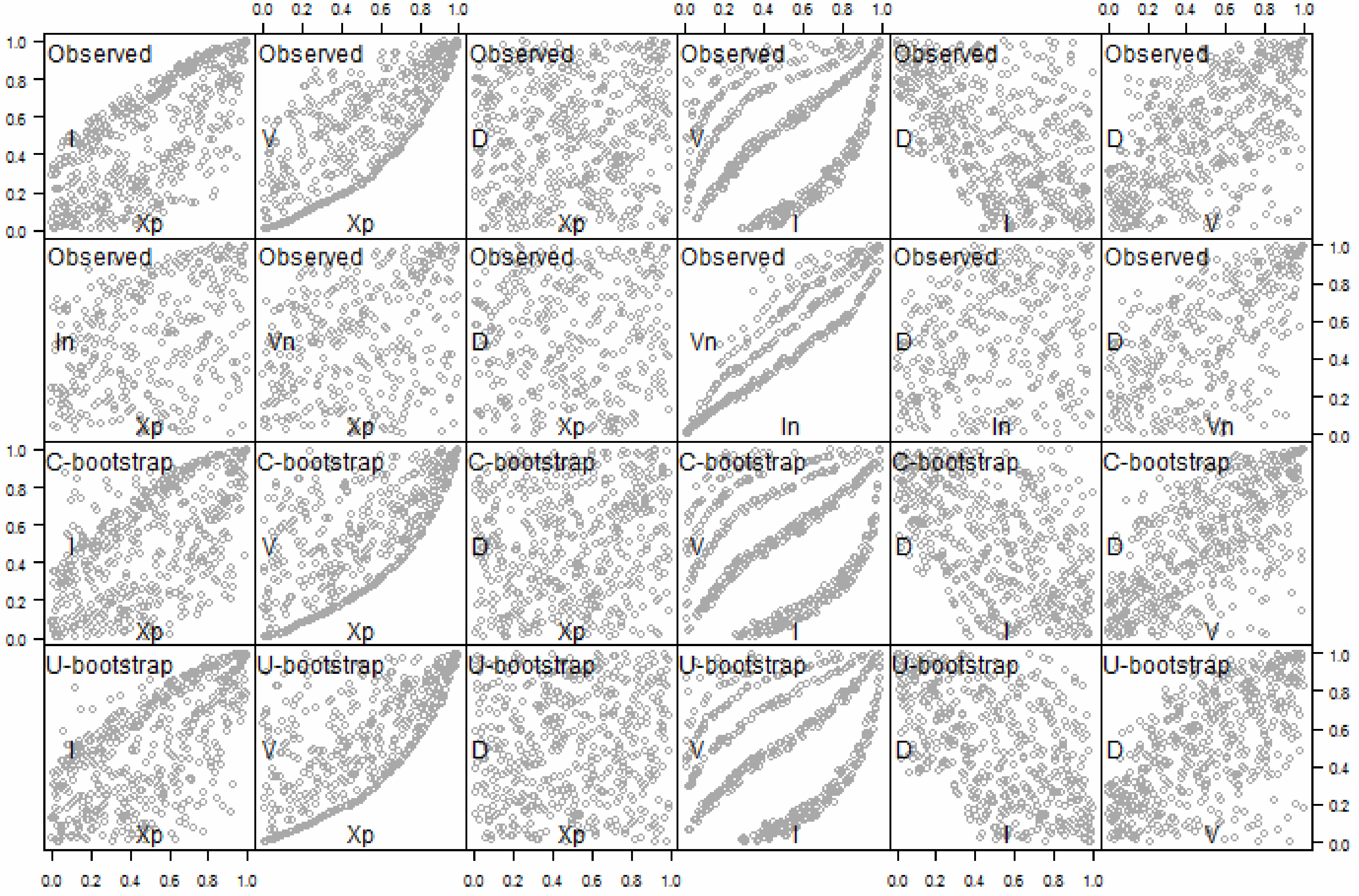

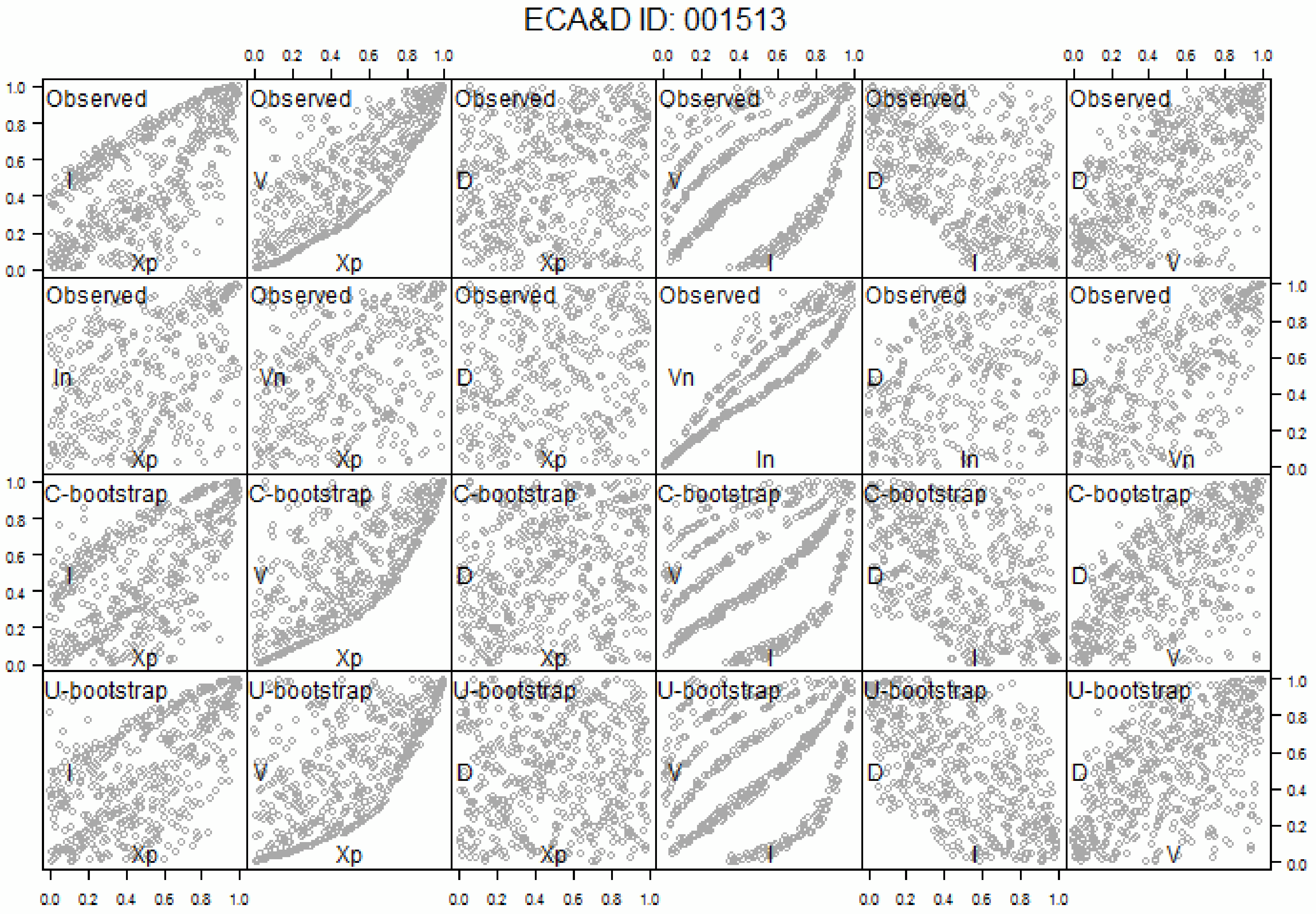
ECA&D ID: 001513
Observed
C-bootstrap
U-bootstrap
Xp
I
V
D
In
Vn
0.0
0.2
0.4
0.6
0.8
1.0

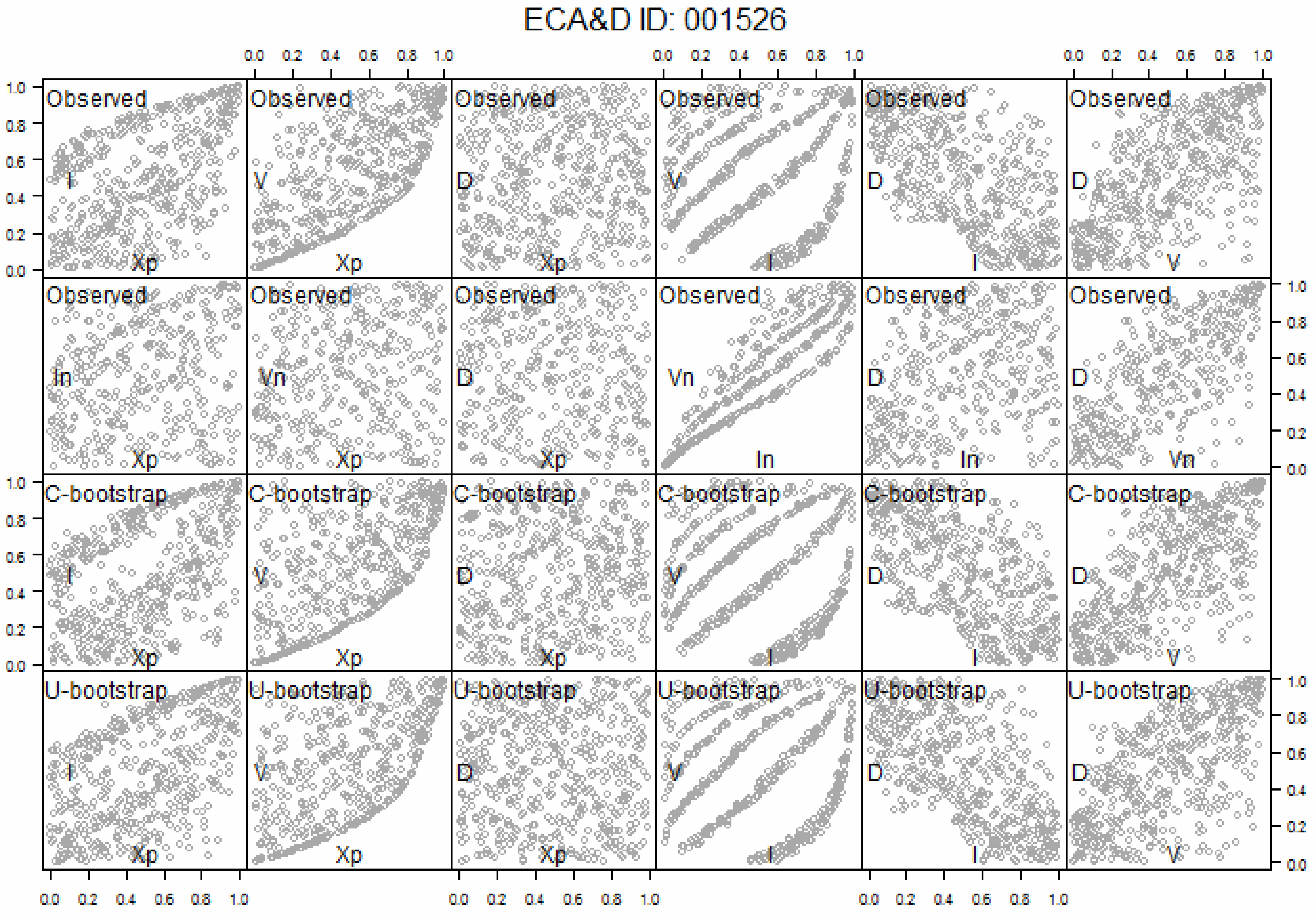
ECA&D ID: 001526
Observed
C-bootstrap
U-bootstrap
Xp
I
V
D
In
Vn
0.0
0.2
0.4
0.6
0.8
1.0

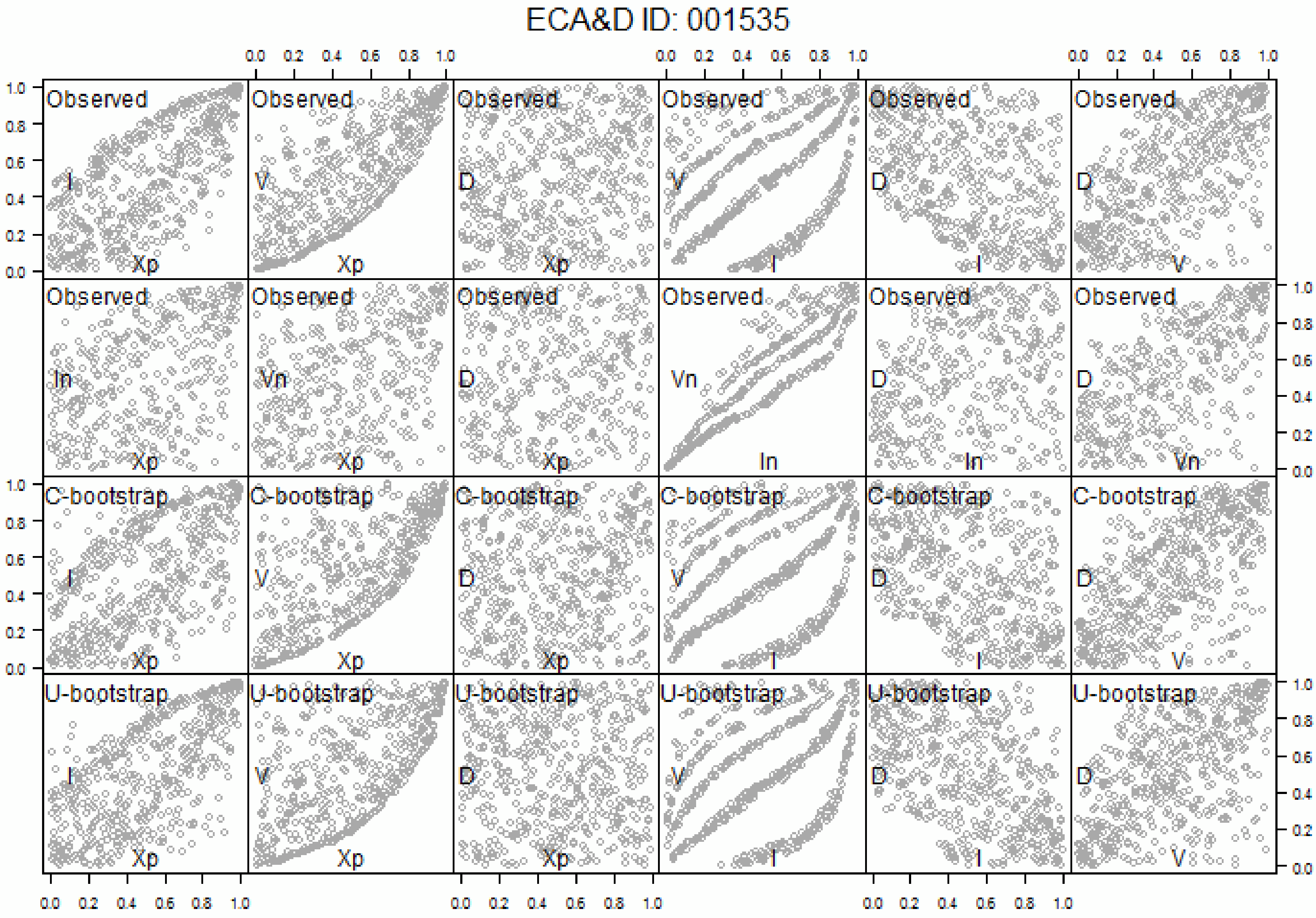

ECA&D ID: 001535
Observed
C-bootstrap
U-bootstrap
Xp
I
V
D
In
Vn
0.0
0.2
0.4
0.6
0.8
1.0

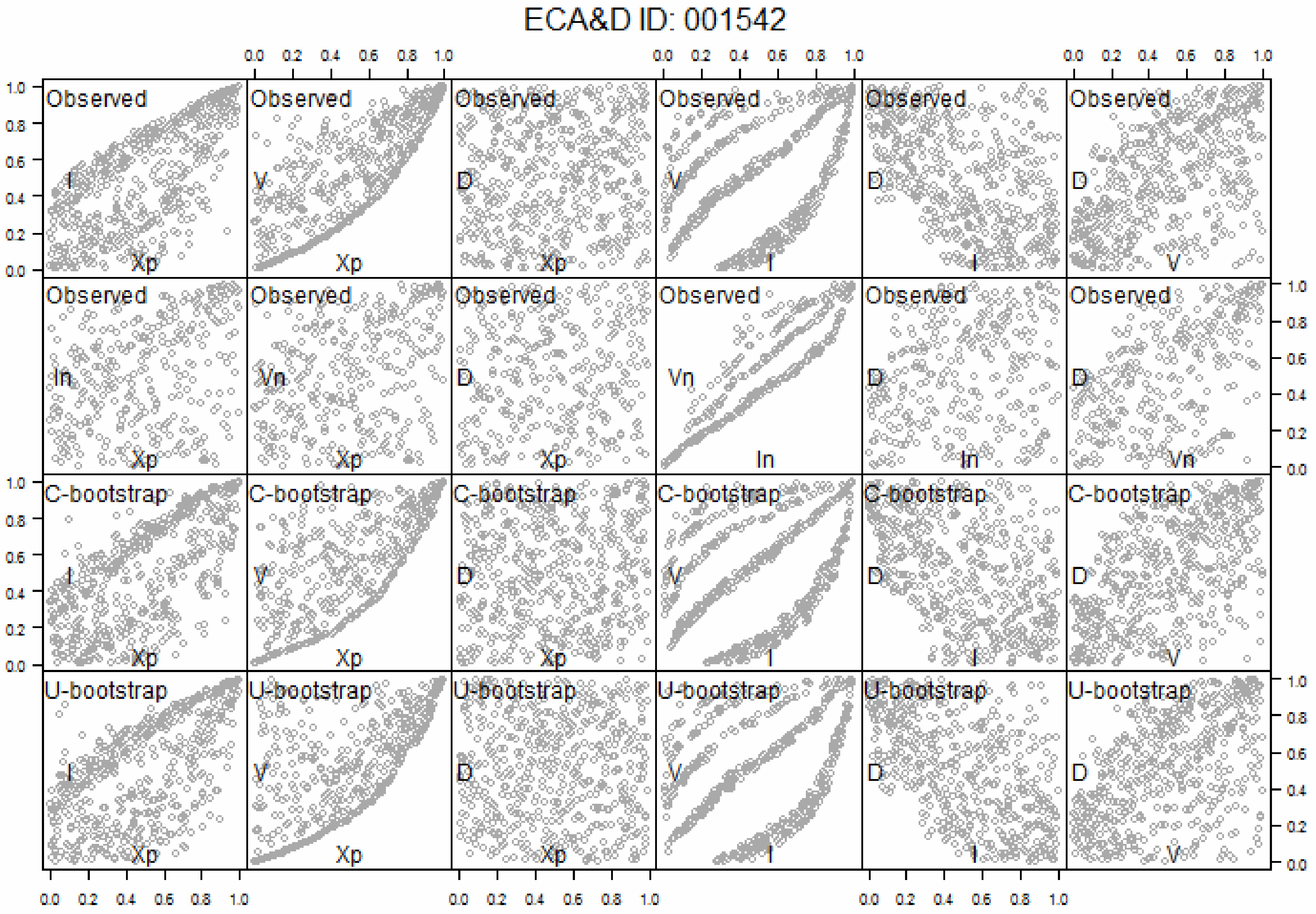

ECA&D ID: 001542
Observed
C-bootstrap
U-bootstrap
Xp
I
V
D
In
Vn
0.0
0.2
0.4
0.6
0.8
1.0

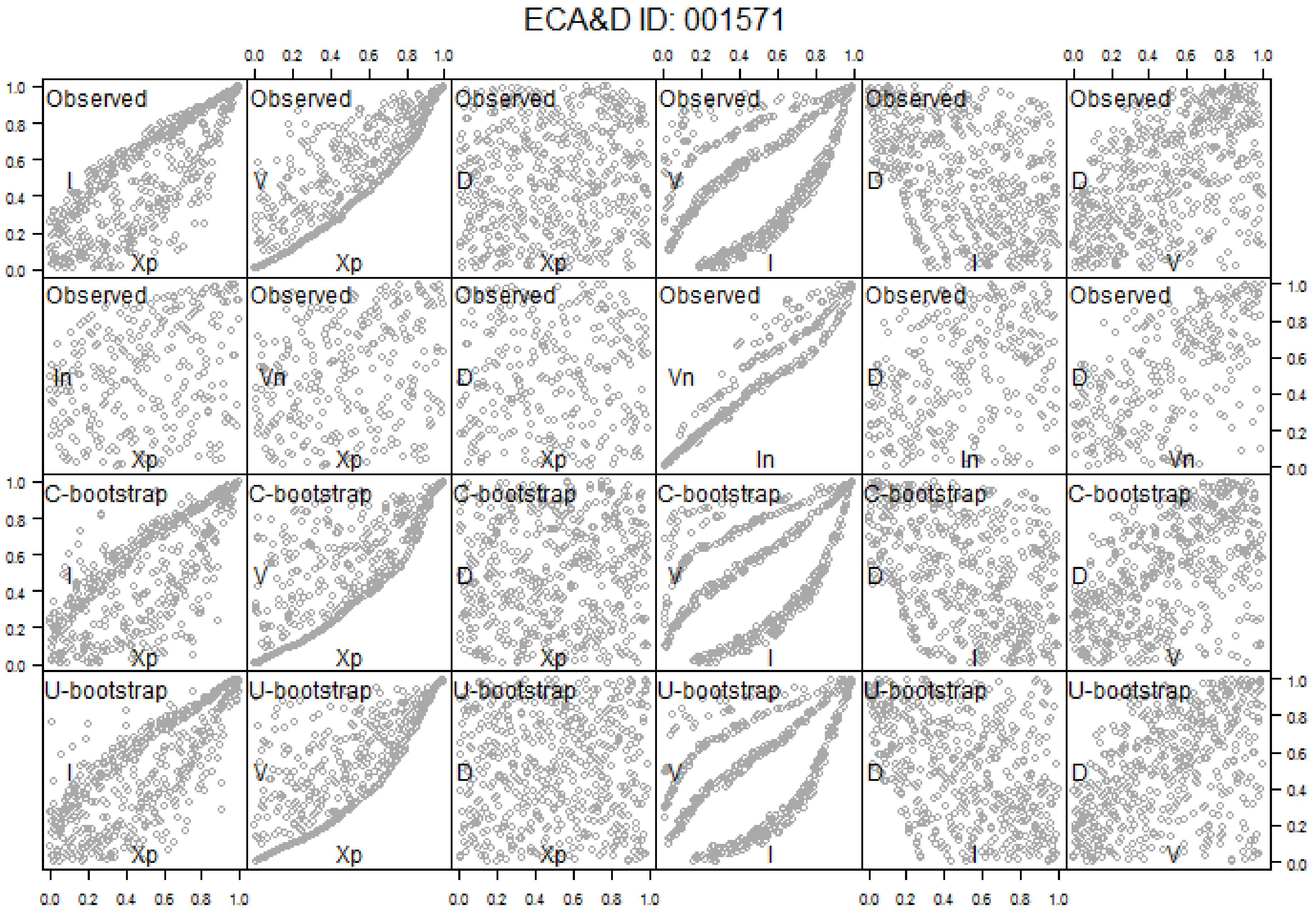
ECA&D ID: 001571
Observed
C-bootstrap
U-bootstrap
Xp
I
V
D
In
Vn
0.0
0.2
0.4
0.6
0.8
1.0

ECA&D ID: 001577

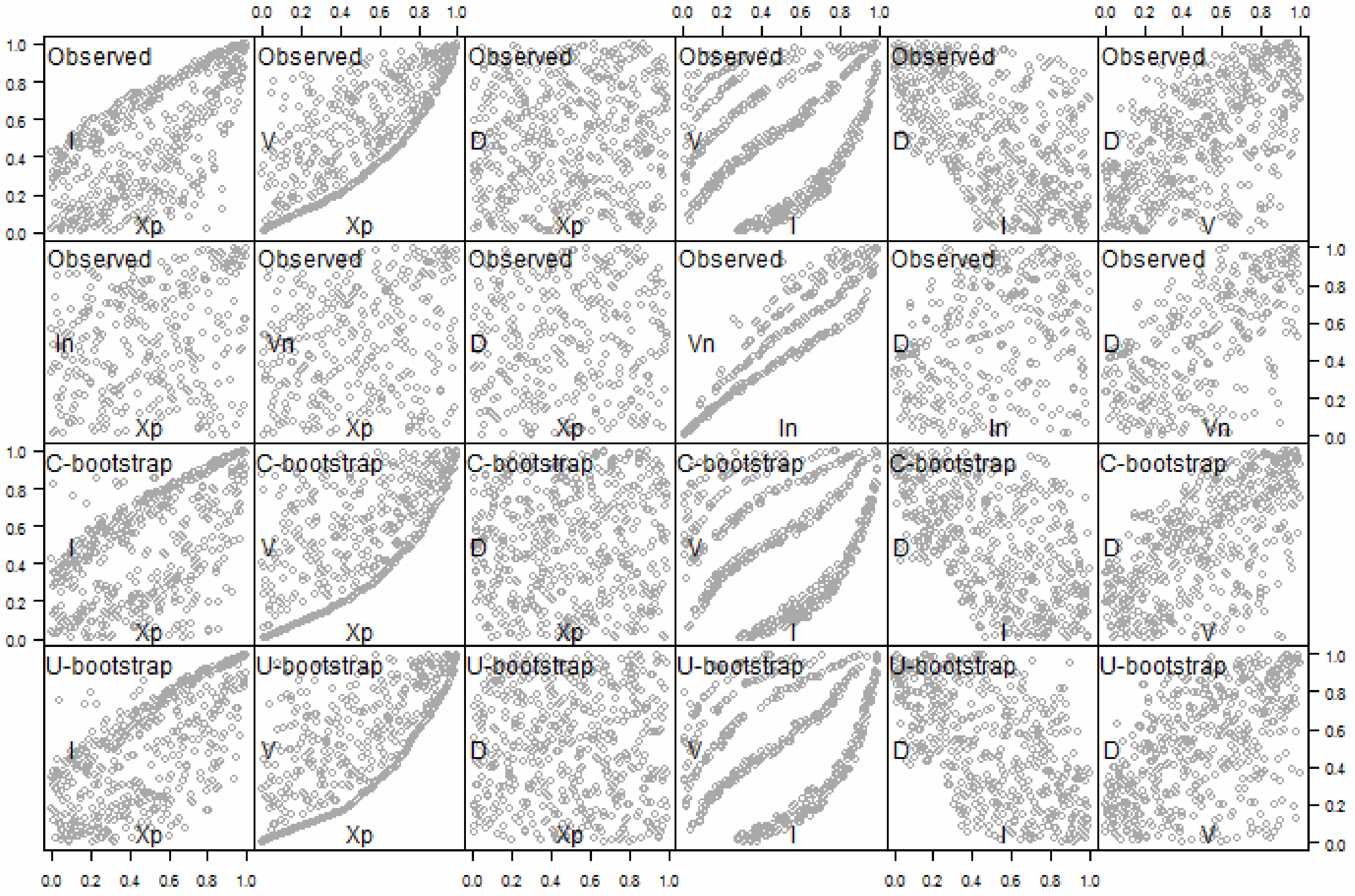

ECA&D ID: 001585

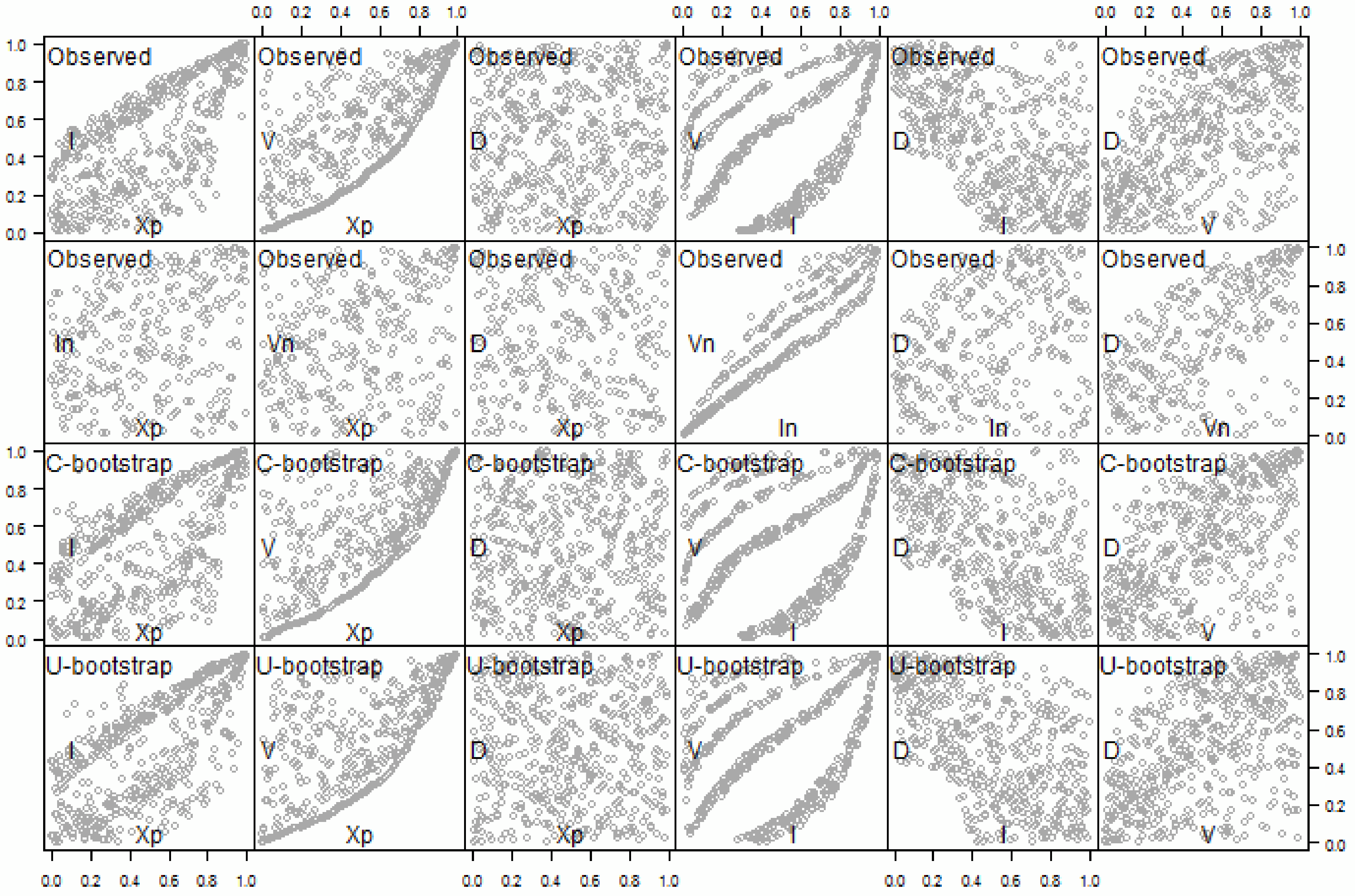

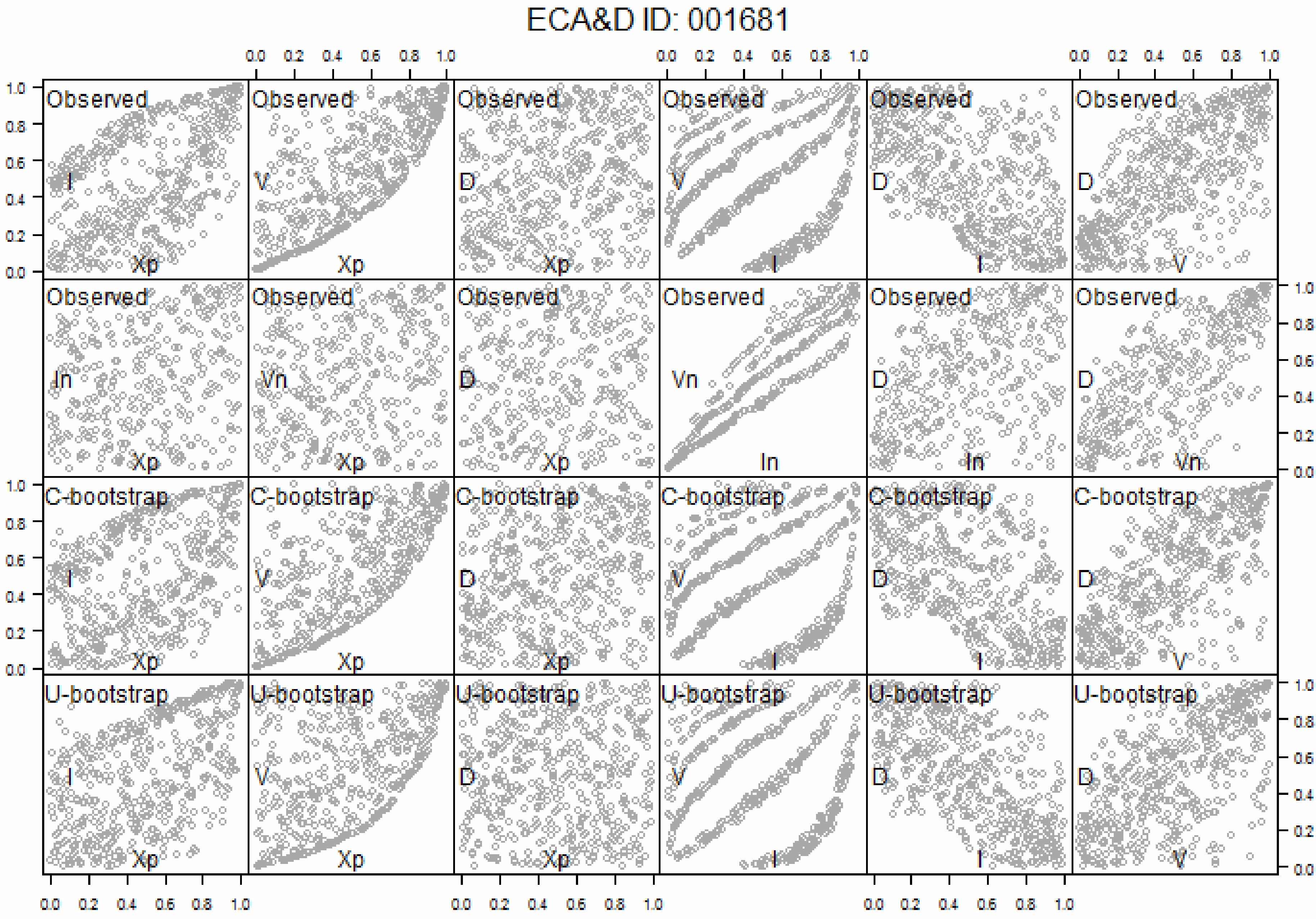

ECA&D ID: 001681
Observed
C-bootstrap
U-bootstrap
Xp
I
V
D
In
Vn
0.0
0.2
0.4
0.6
0.8
1.0

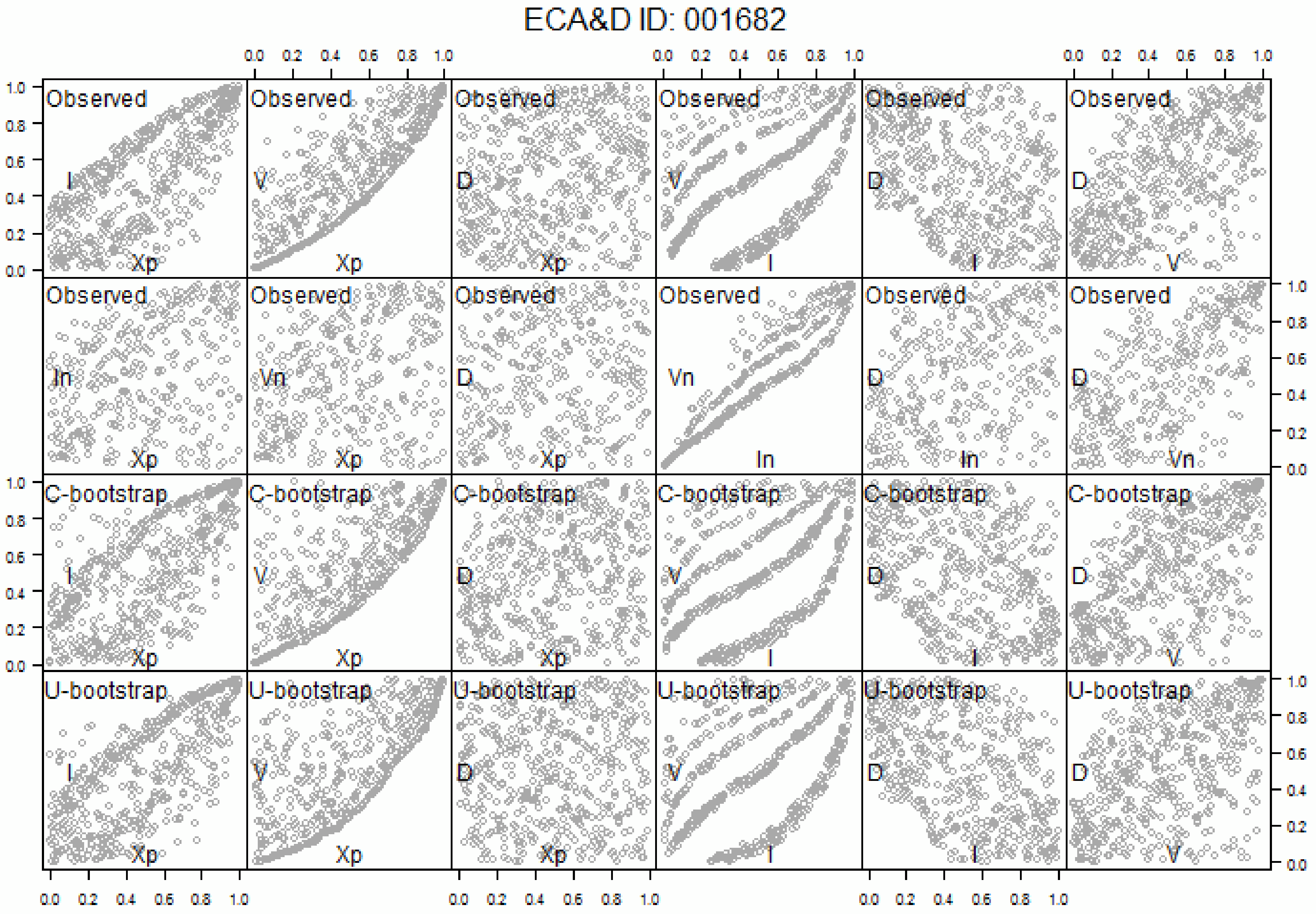
ECA&D ID: 001682
Observed
C-bootstrap
U-bootstrap
Xp
I
V
D
In
Vn
0.0
0.2
0.4
0.6
0.8
1.0

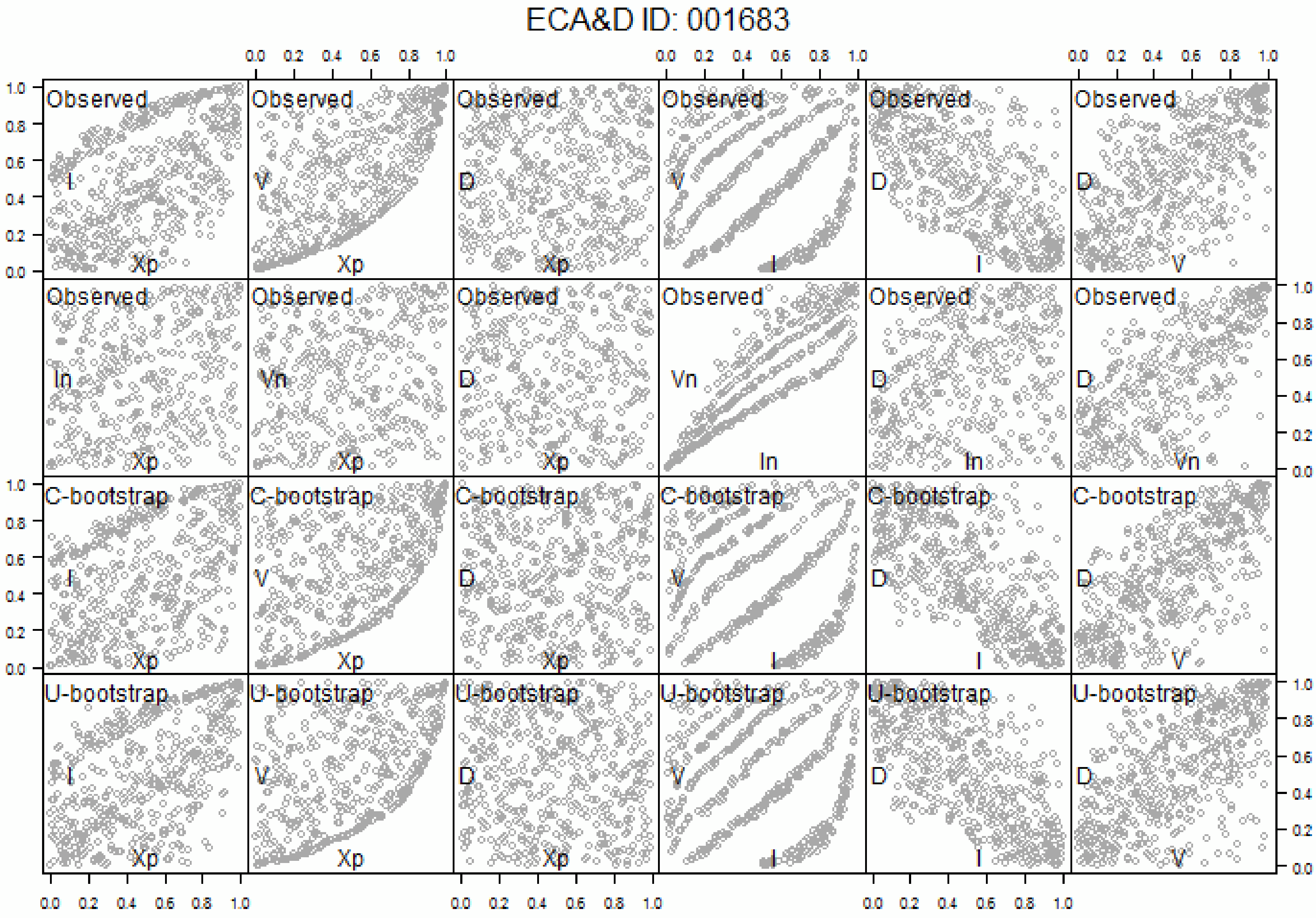
ECA&D ID: 001683
Observed
C-bootstrap
U-bootstrap
Xp
I
V
D
In
Vn
0.0
0.2
0.4
0.6
0.8
1.0

ECA&D ID: 001684

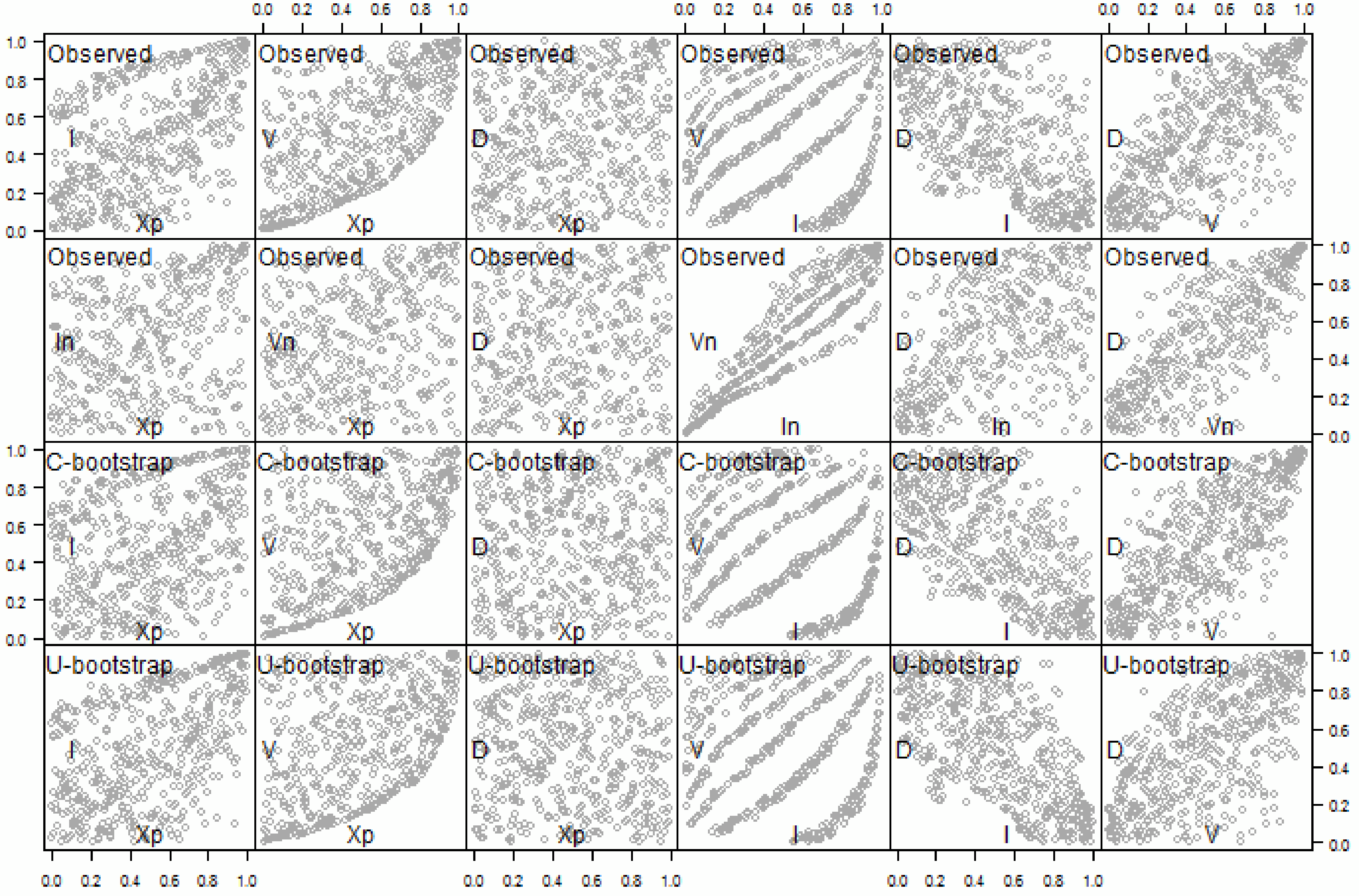

ECA&D ID: 001685

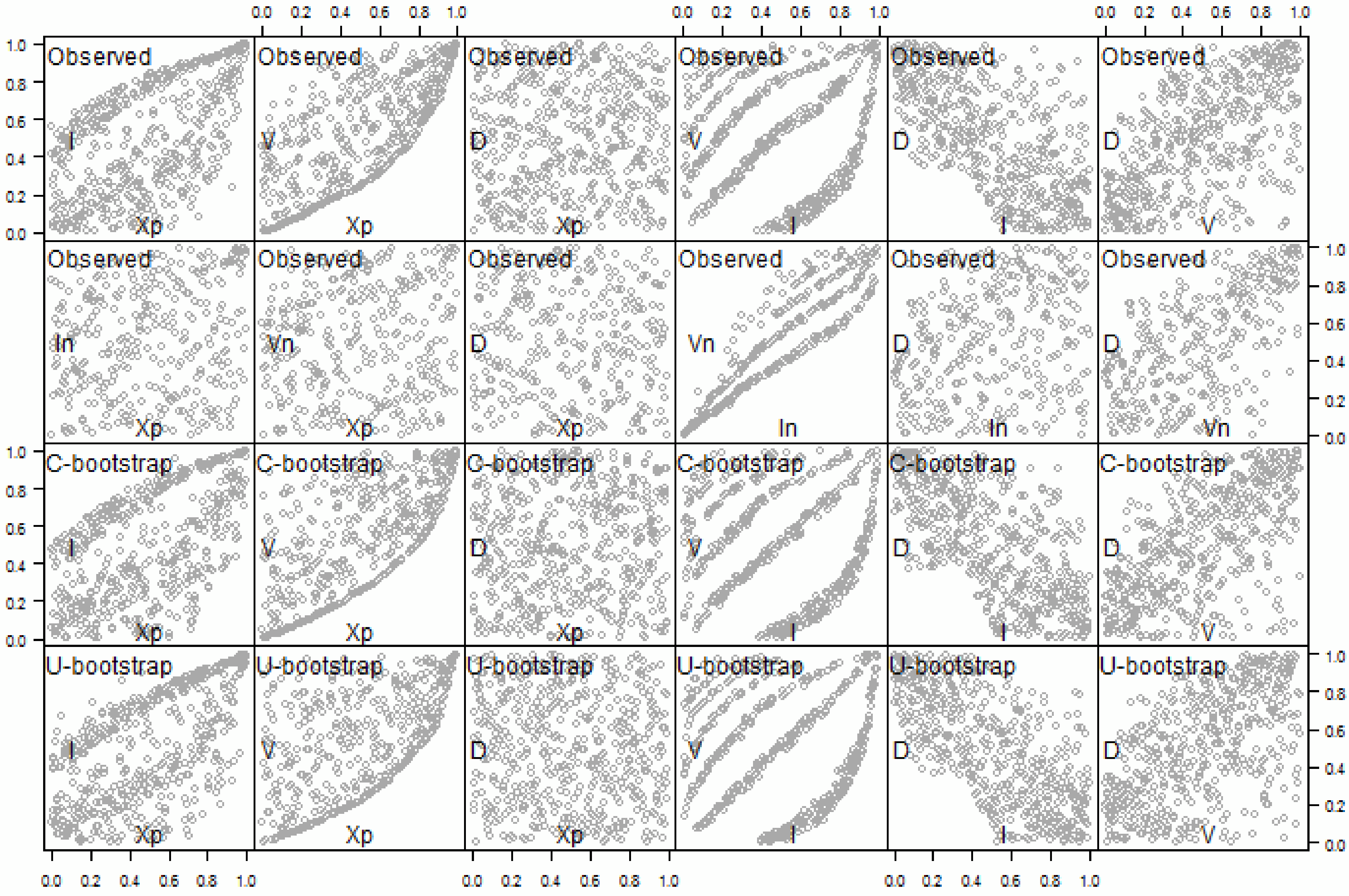

ECA&D ID: 001686

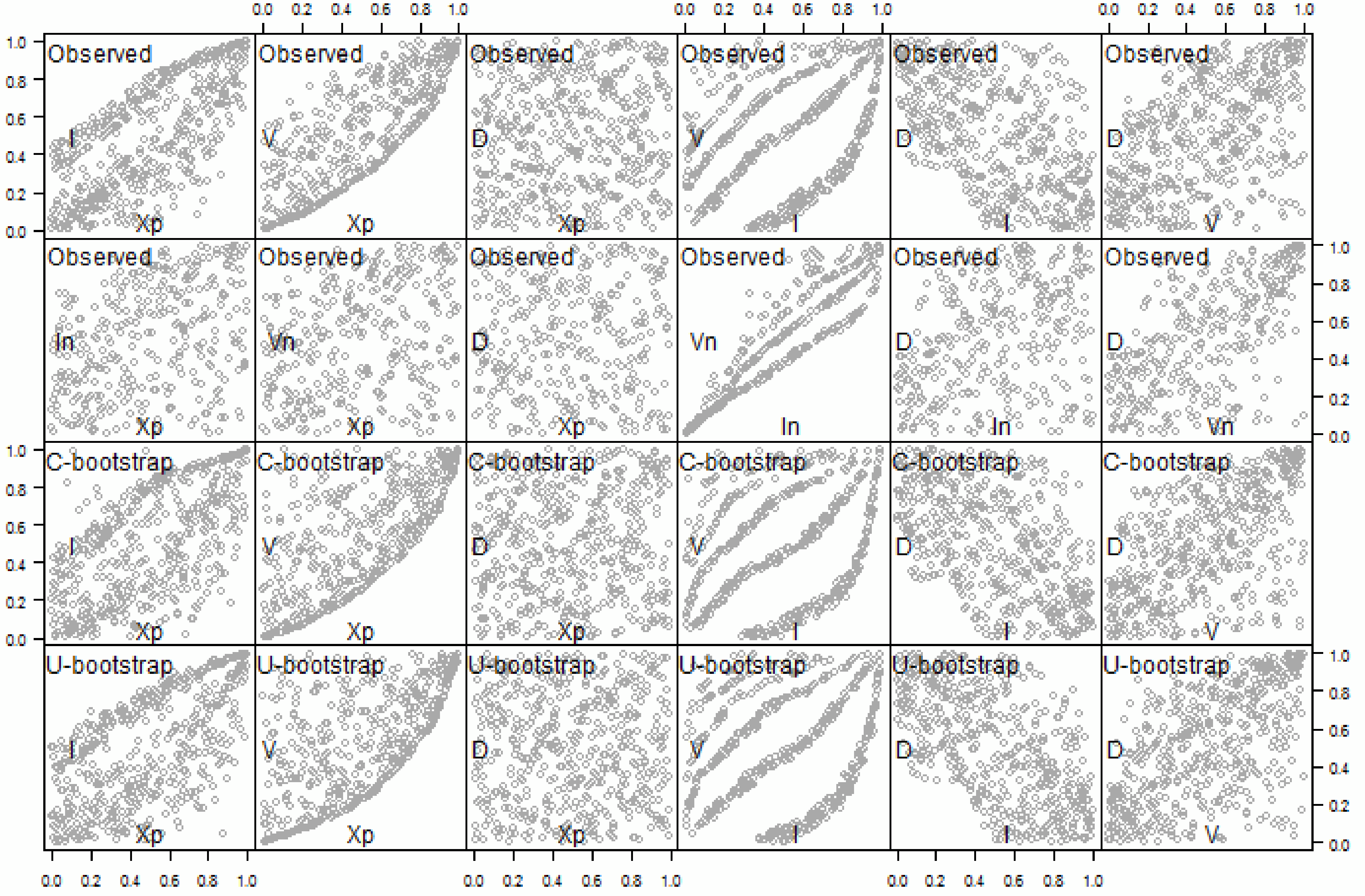

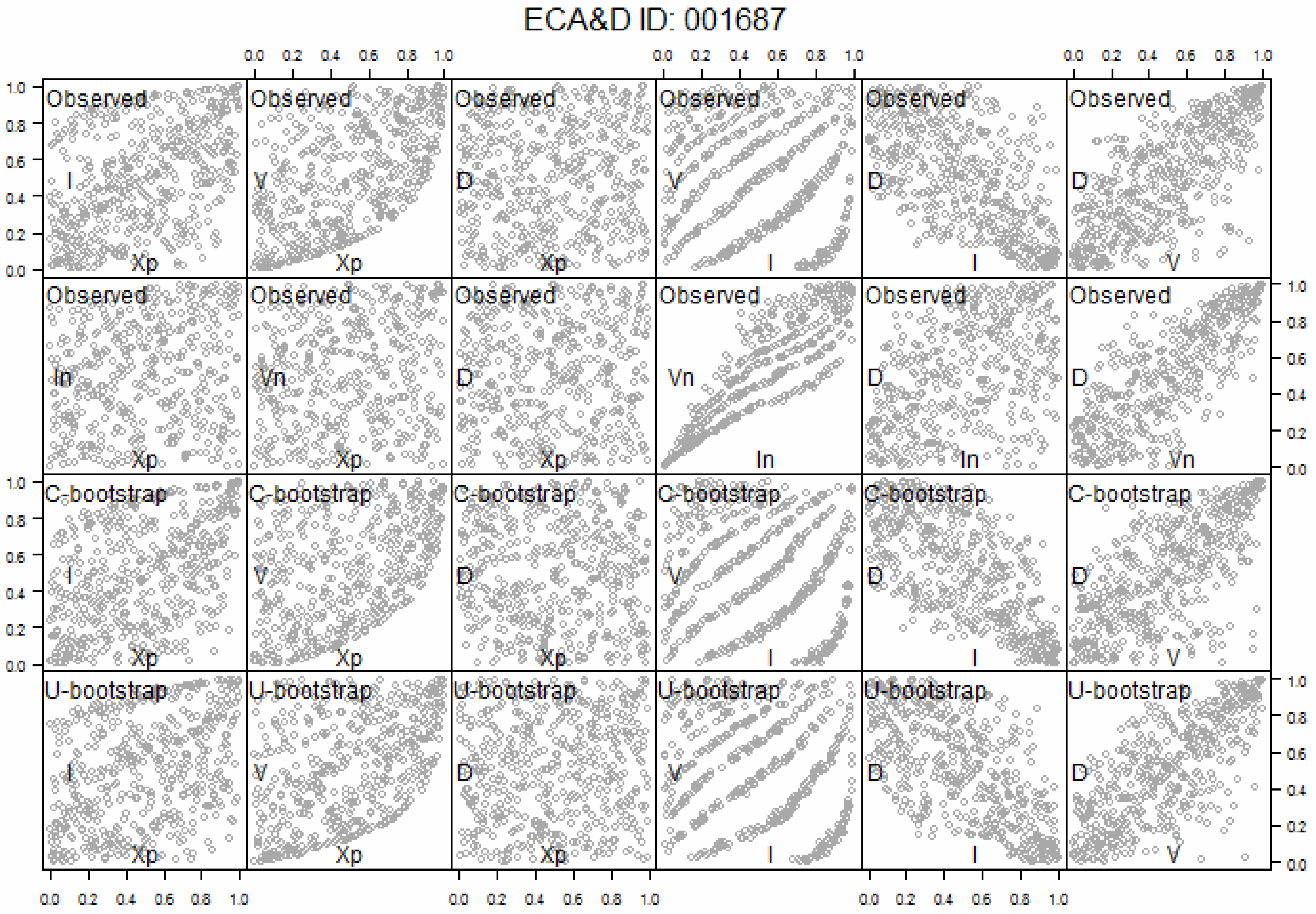
ECA&D ID: 001687
Observed
C-bootstrap
U-bootstrap
Xp
I
V
D
In
Vn
0.0
0.2
0.4
0.6
0.8
1.0

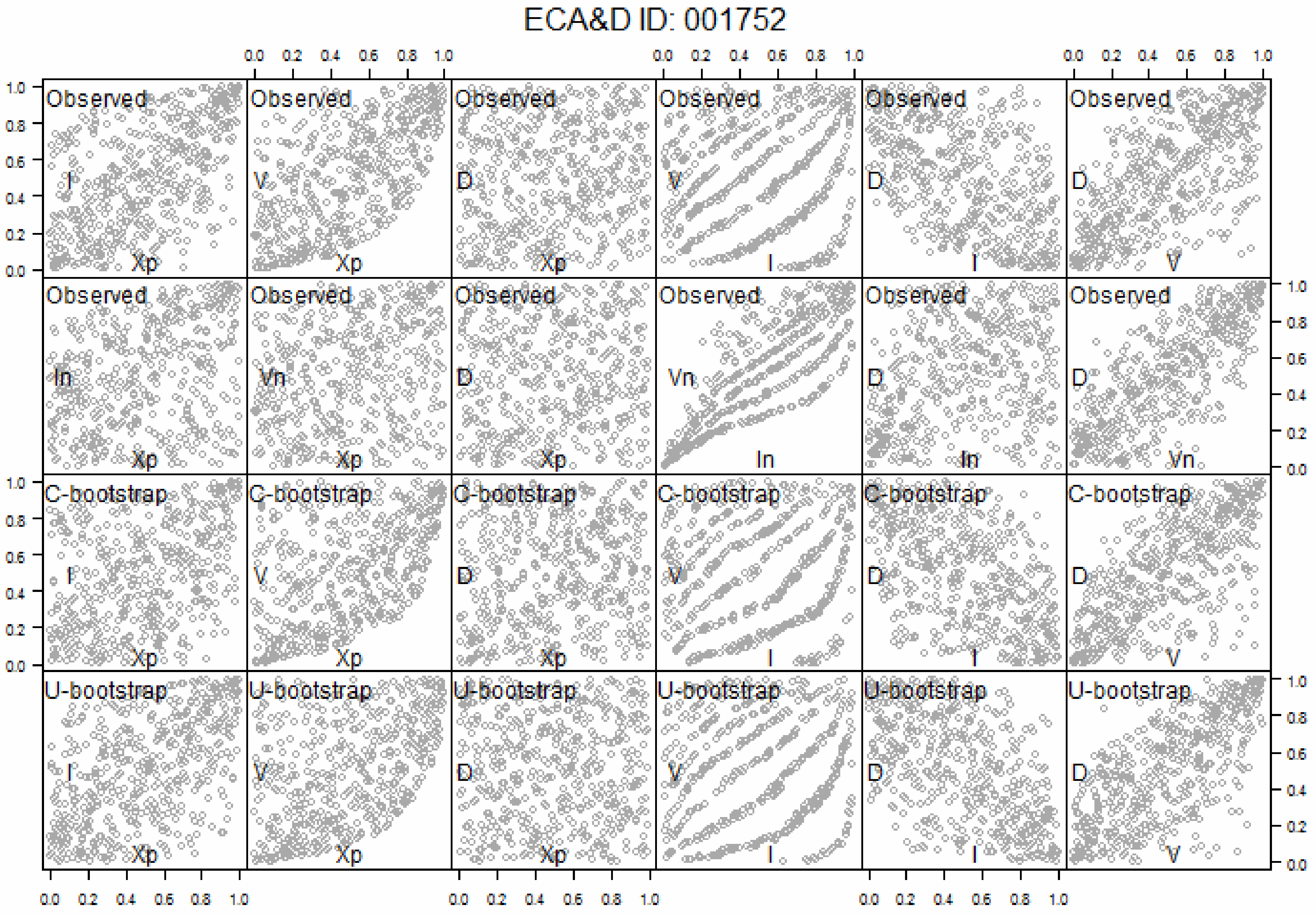
ECA&D ID: 001752
Observed
C-bootstrap
U-bootstrap
Xp
I
V
D
In
Vn
0.0
0.2
0.4
0.6
0.8
1.0

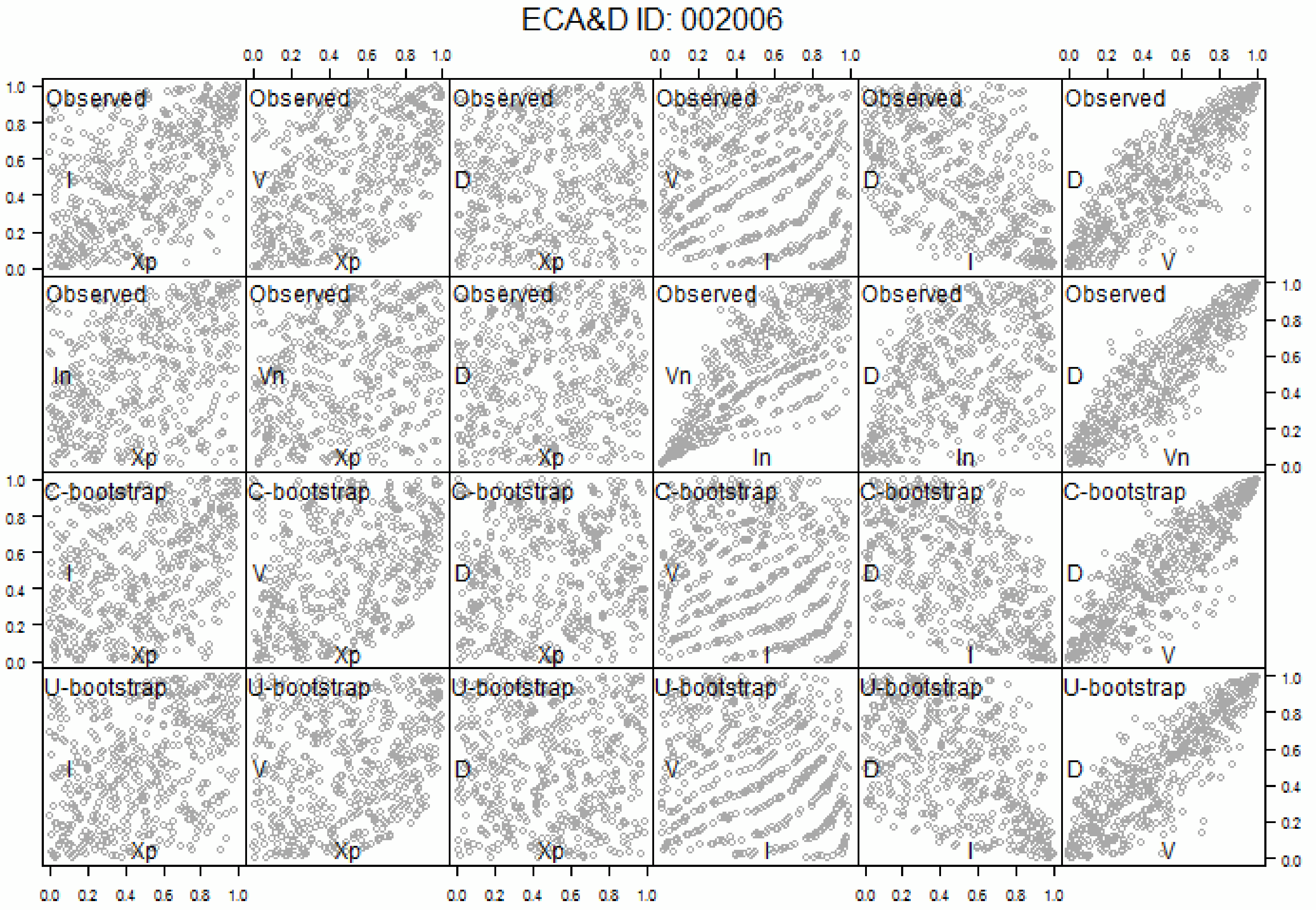
ECA&D ID: 002006
Observed
C-bootstrap
U-bootstrap
Xp
I
V
D
In
Vn
0.0
0.2
0.4
0.6
0.8
1.0

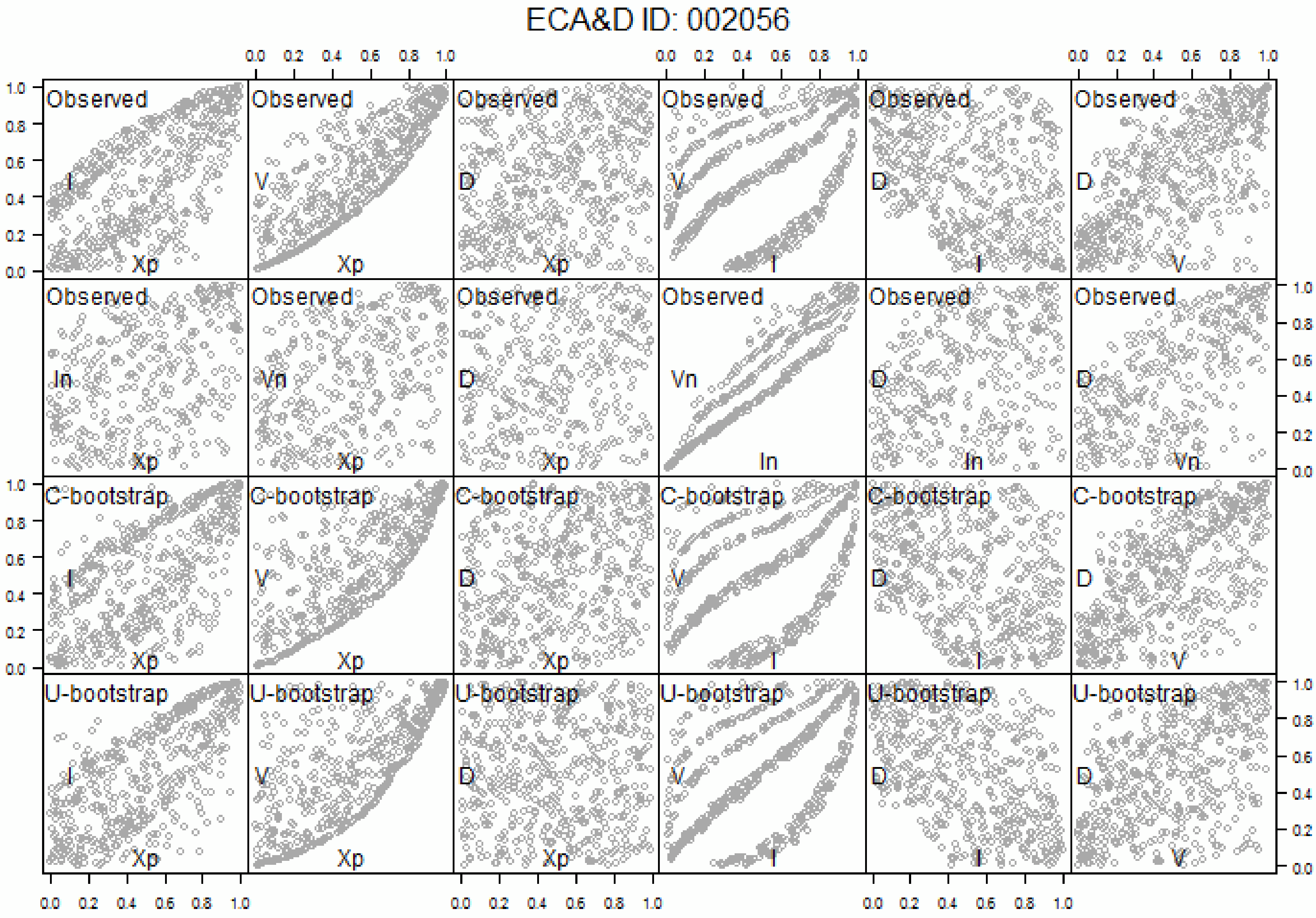
ECA&D ID: 002056
Observed
C-bootstrap
U-bootstrap
Xp
I
V
D
In
Vn
0.0
0.2
0.4
0.6
0.8
1.0

ECA&D ID: 002058

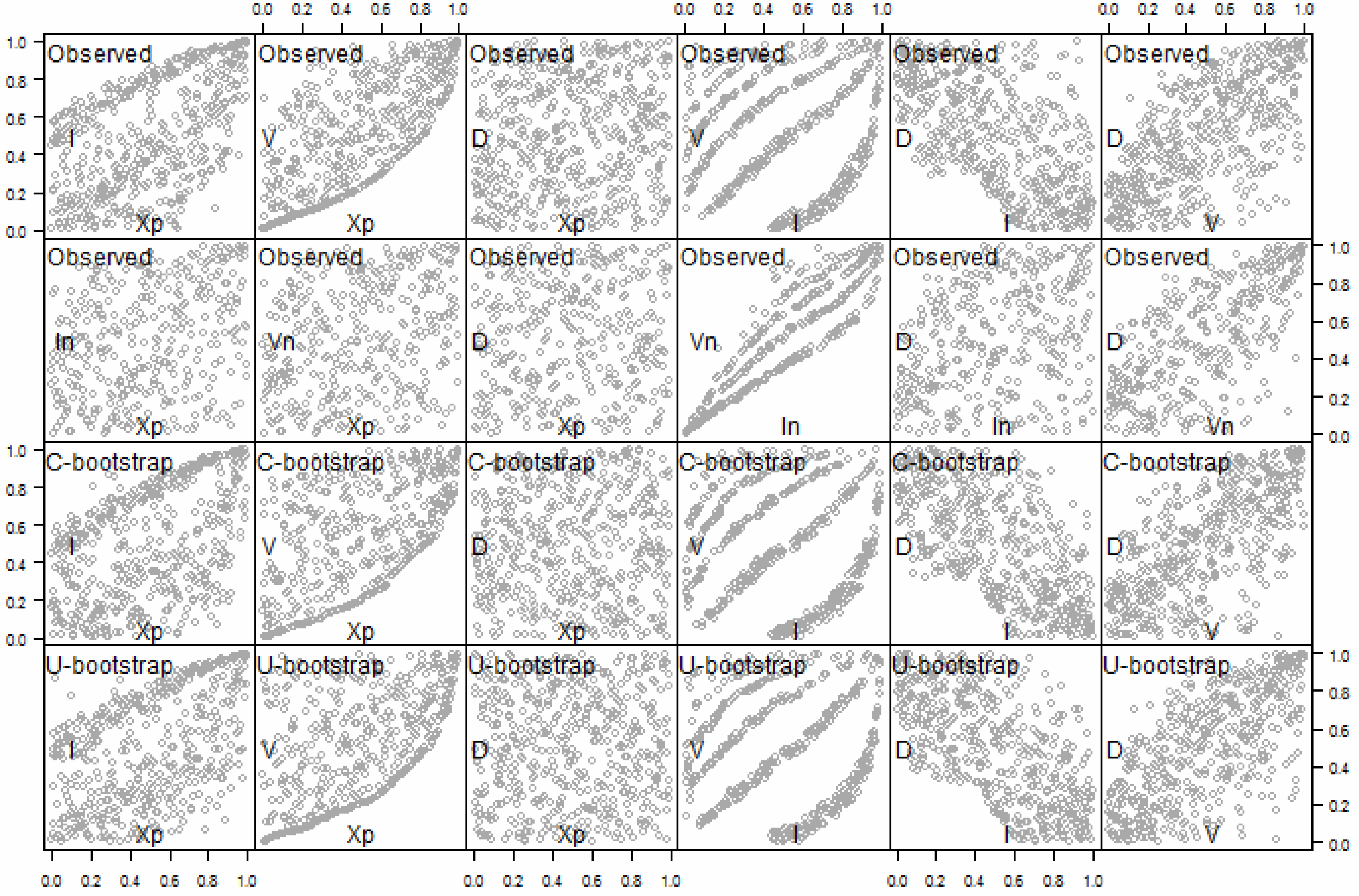

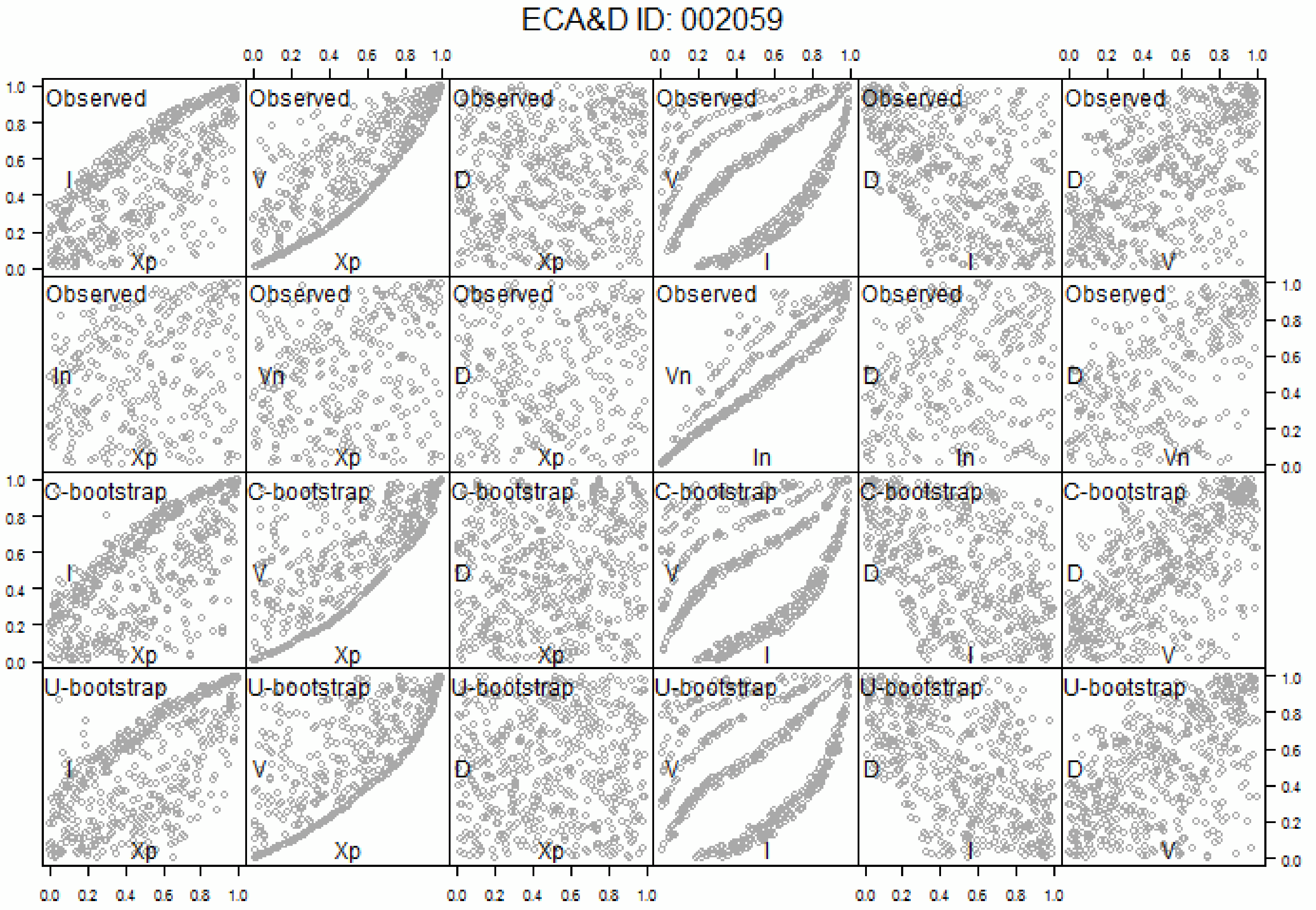

ECA&D ID: 002059
Observed
C-bootstrap
U-bootstrap
Xp
I
V
D
In
Vn
0.0
0.2
0.4
0.6
0.8
1.0

ECA&D ID: 002060

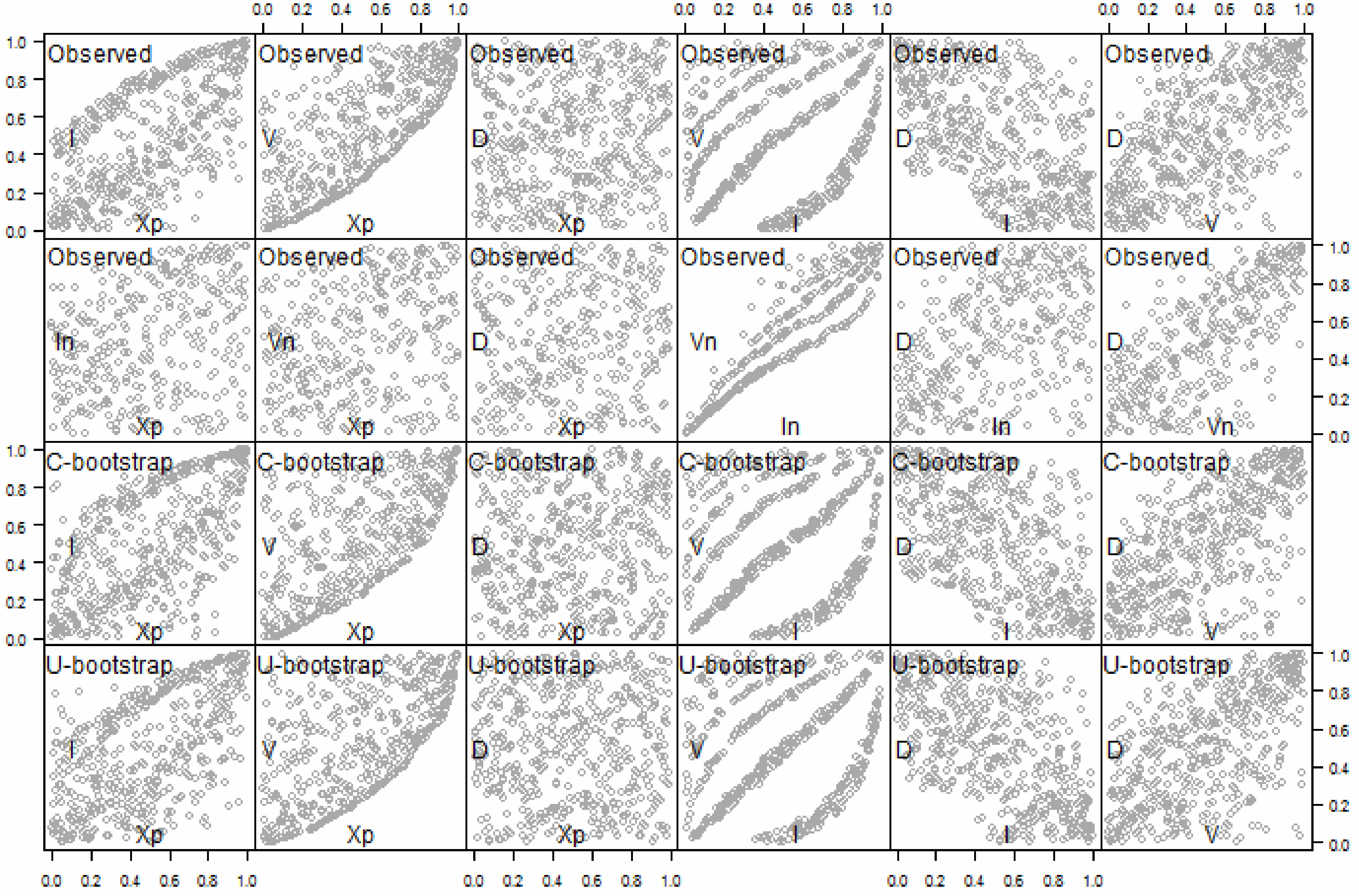

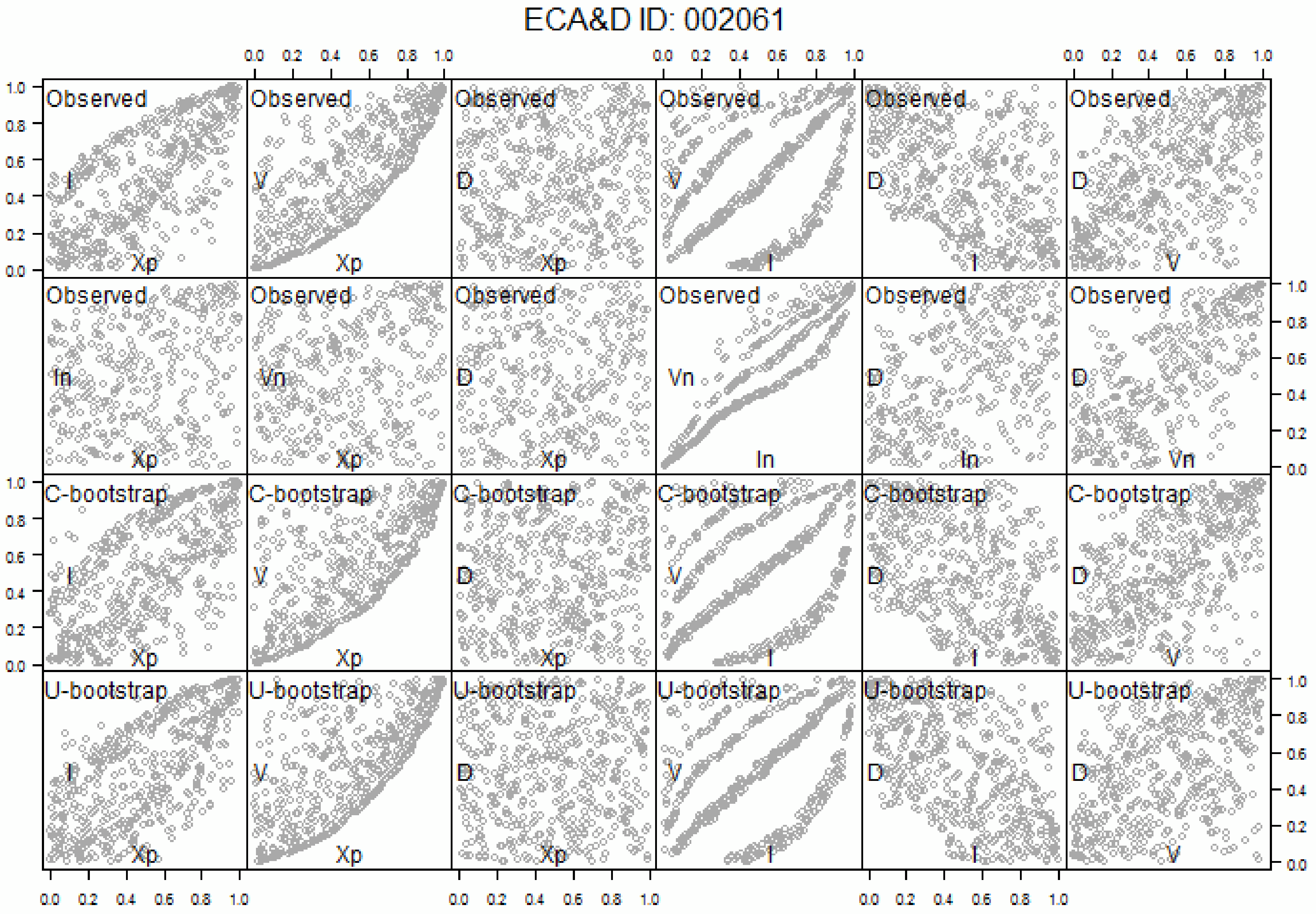

ECA&D ID: 002061
Observed
C-bootstrap
U-bootstrap
Xp
I
V
D
In
Vn
0.0
0.2
0.4
0.6
0.8
1.0

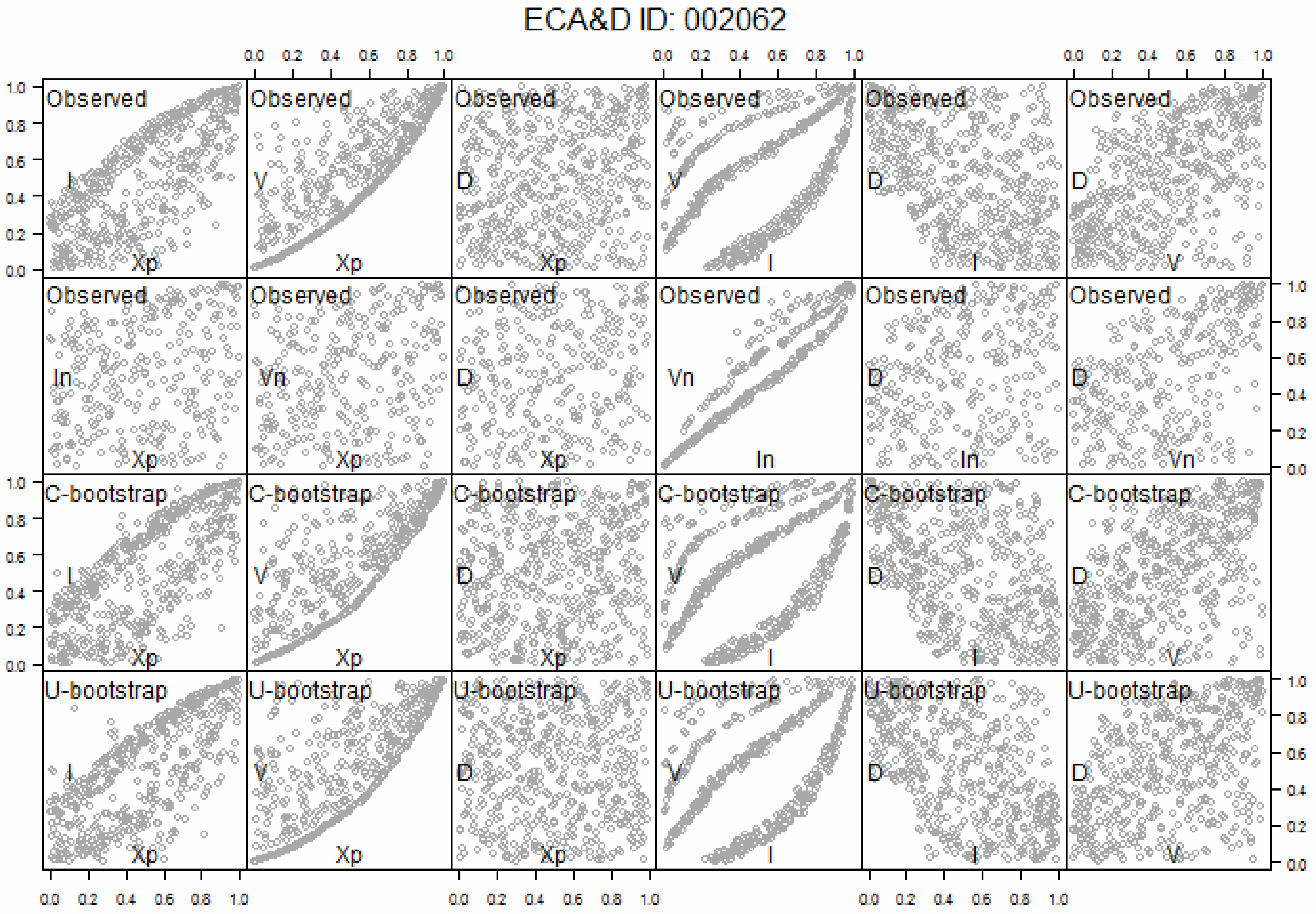

ECA&D ID: 002062
Observed
C-bootstrap
U-bootstrap
Xp
I
V
D
In
Vn
0.0
0.2
0.4
0.6
0.8
1.0

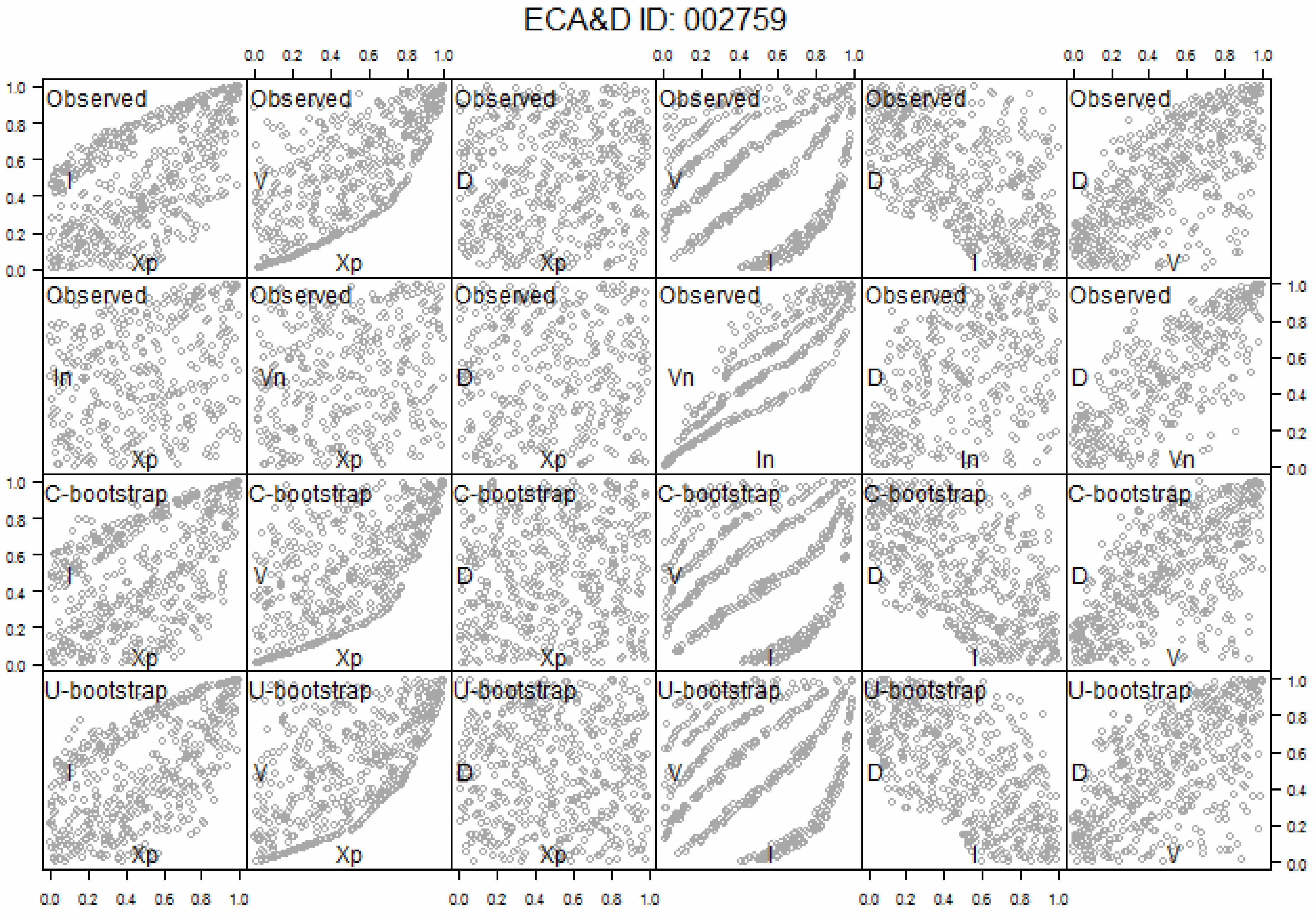
ECA&D ID: 002759
Observed
C-bootstrap
U-bootstrap
Xp
I
V
D
In
Vn
0.0
0.2
0.4
0.6
0.8
1.0

ECA&D ID: 002760

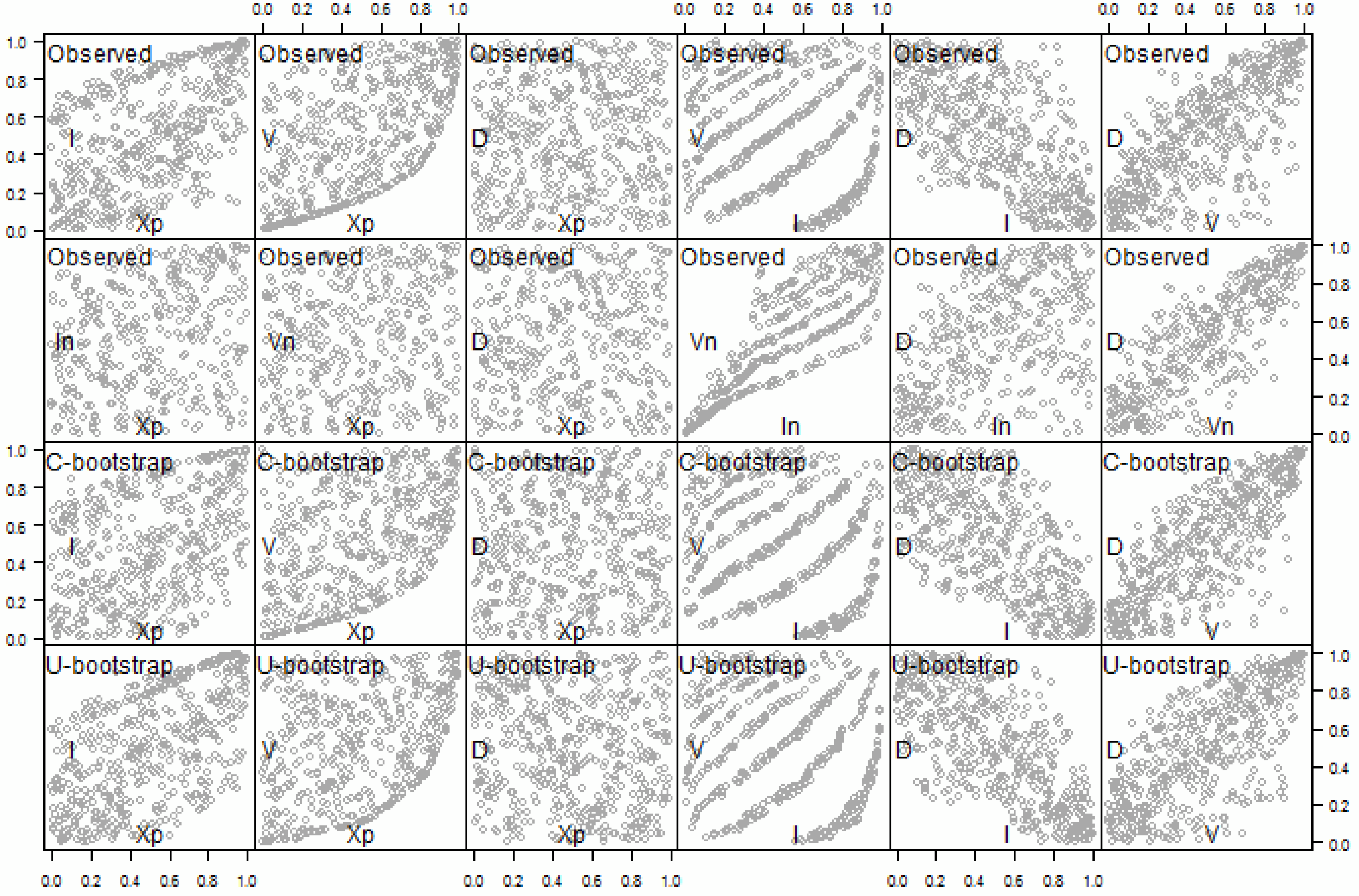

ECA&D ID: 002761

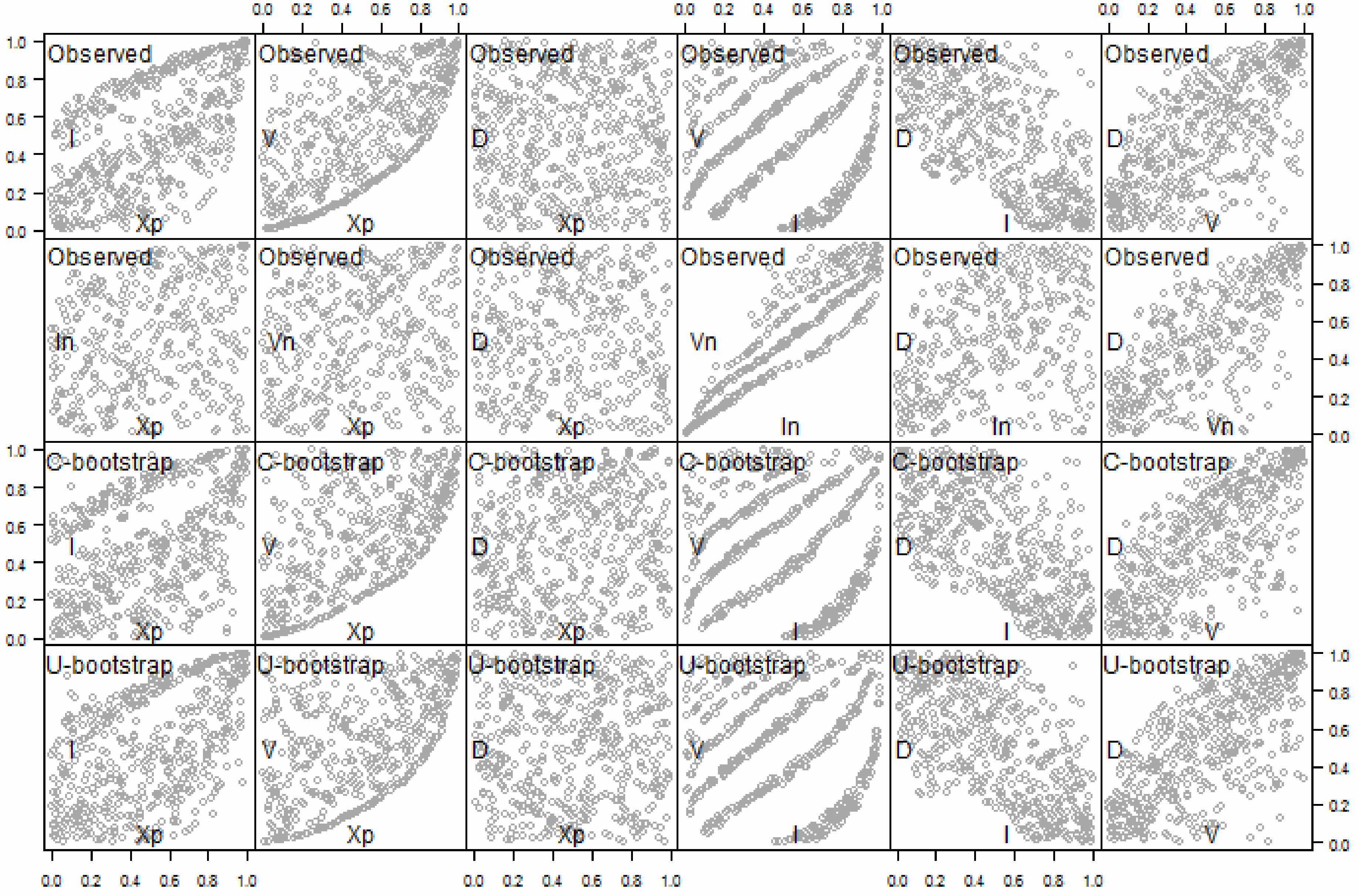

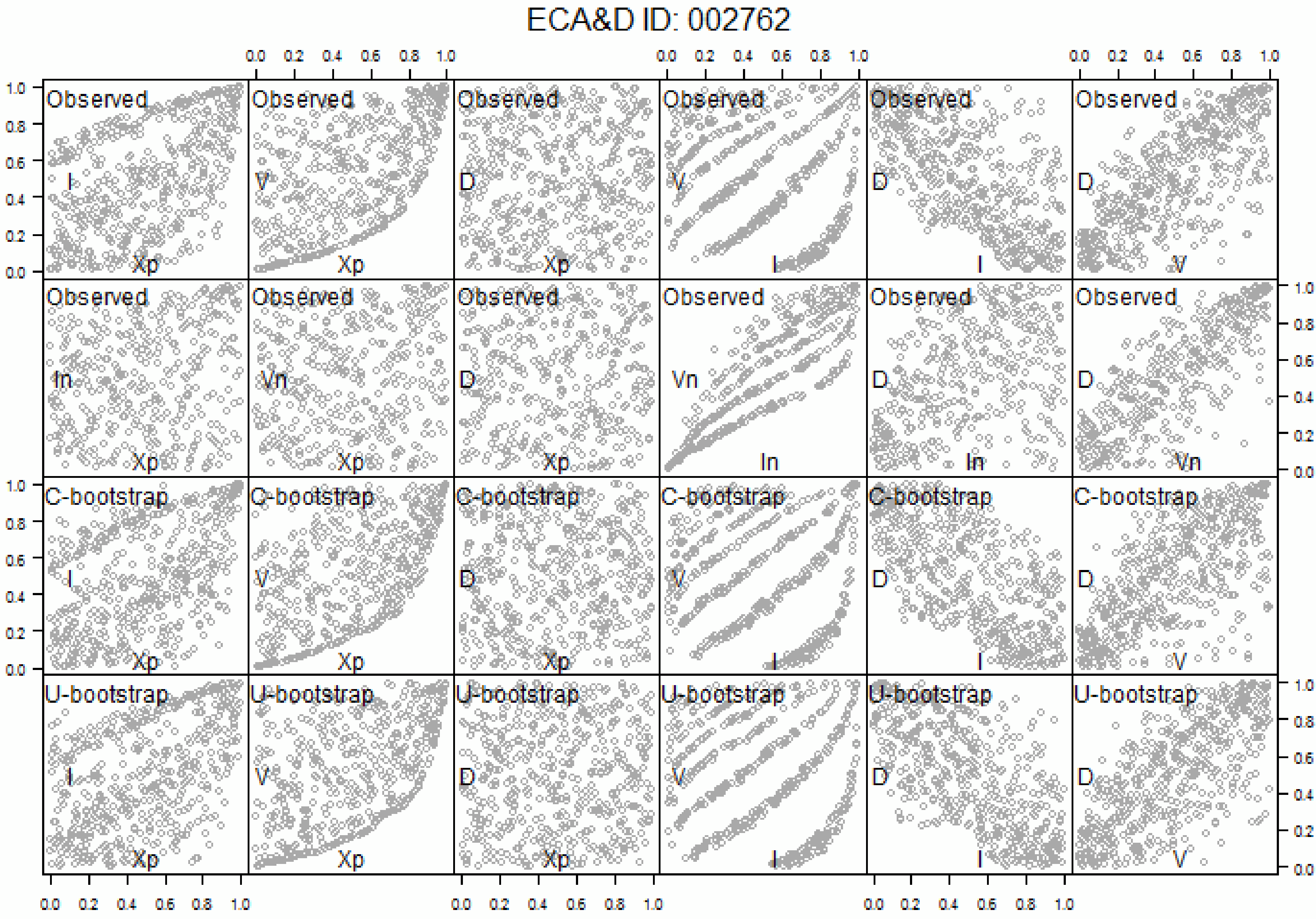

ECA&D ID: 002762
Observed
C-bootstrap
U-bootstrap
I
V
D
In
Vn
Xp
0.0
0.2
0.4
0.6
0.8
1.0

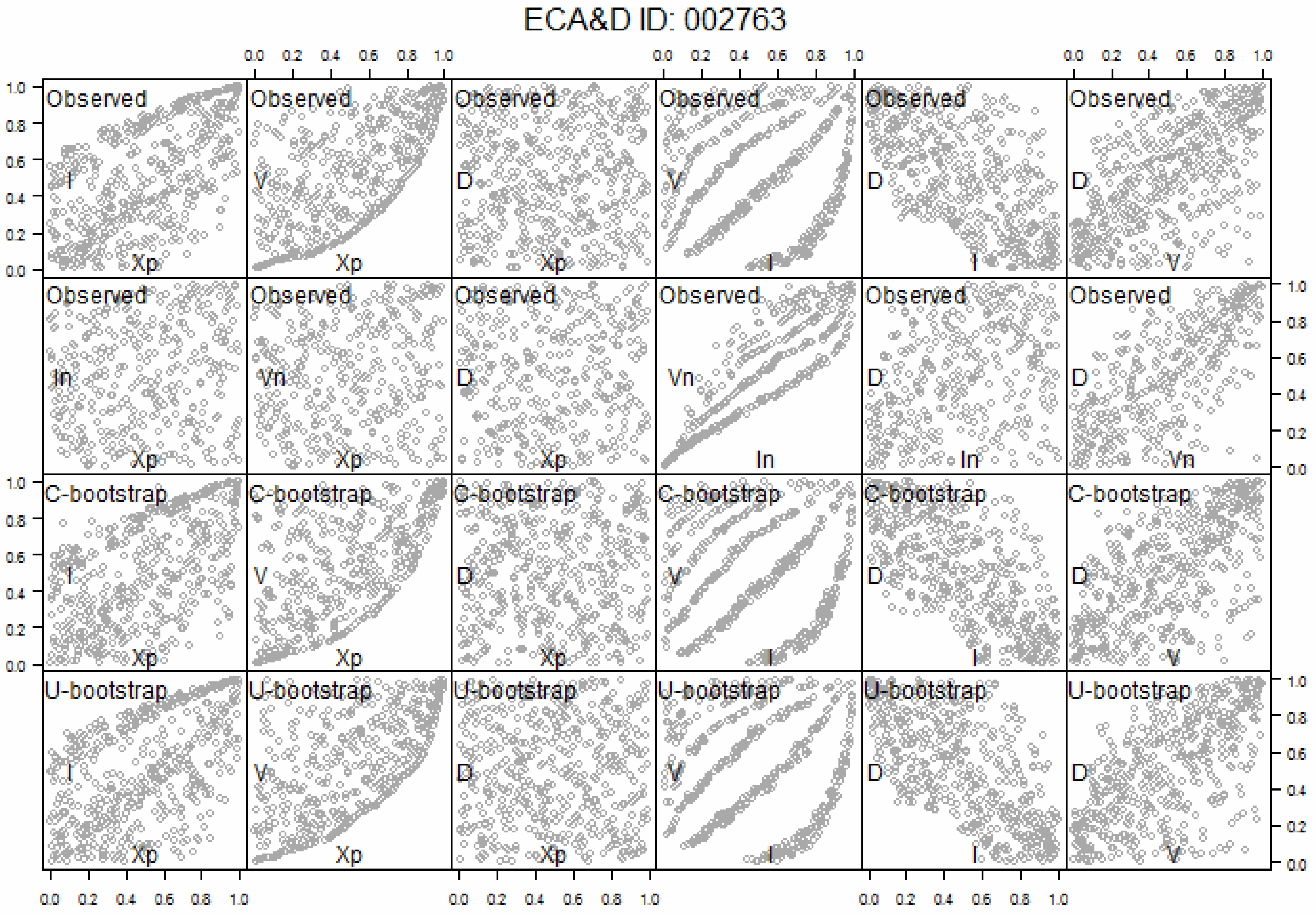
ECA&D ID: 002763
Observed
C-bootstrap
U-bootstrap
Xp
I
V
D
In
Vn
0.0
0.2
0.4
0.6
0.8
1.0

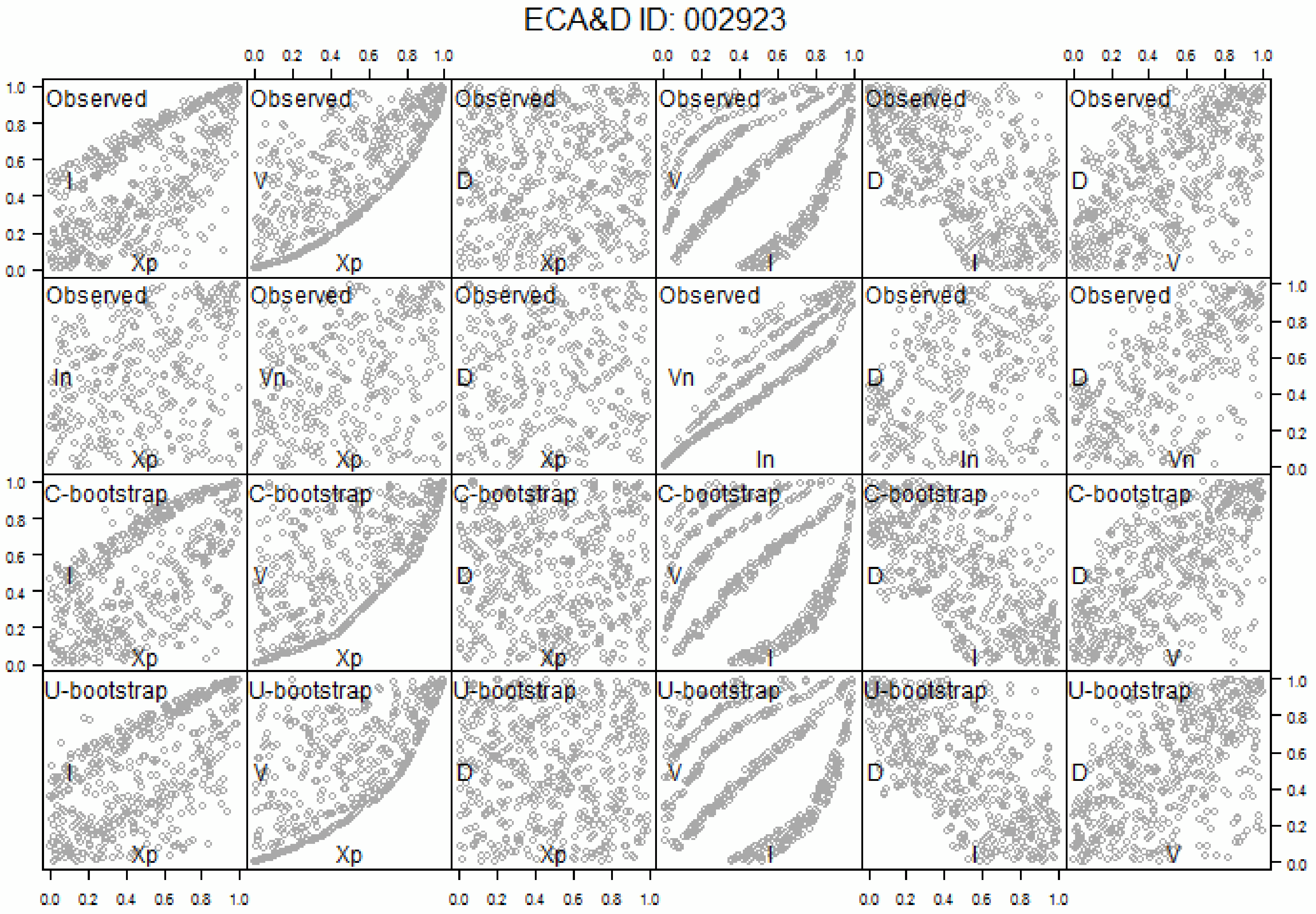

ECA&D ID: 002923
Observed
C-bootstrap
U-bootstrap
I
V
D
In
Vn
Xp
0.0
0.2
0.4
0.6
0.8
1.0

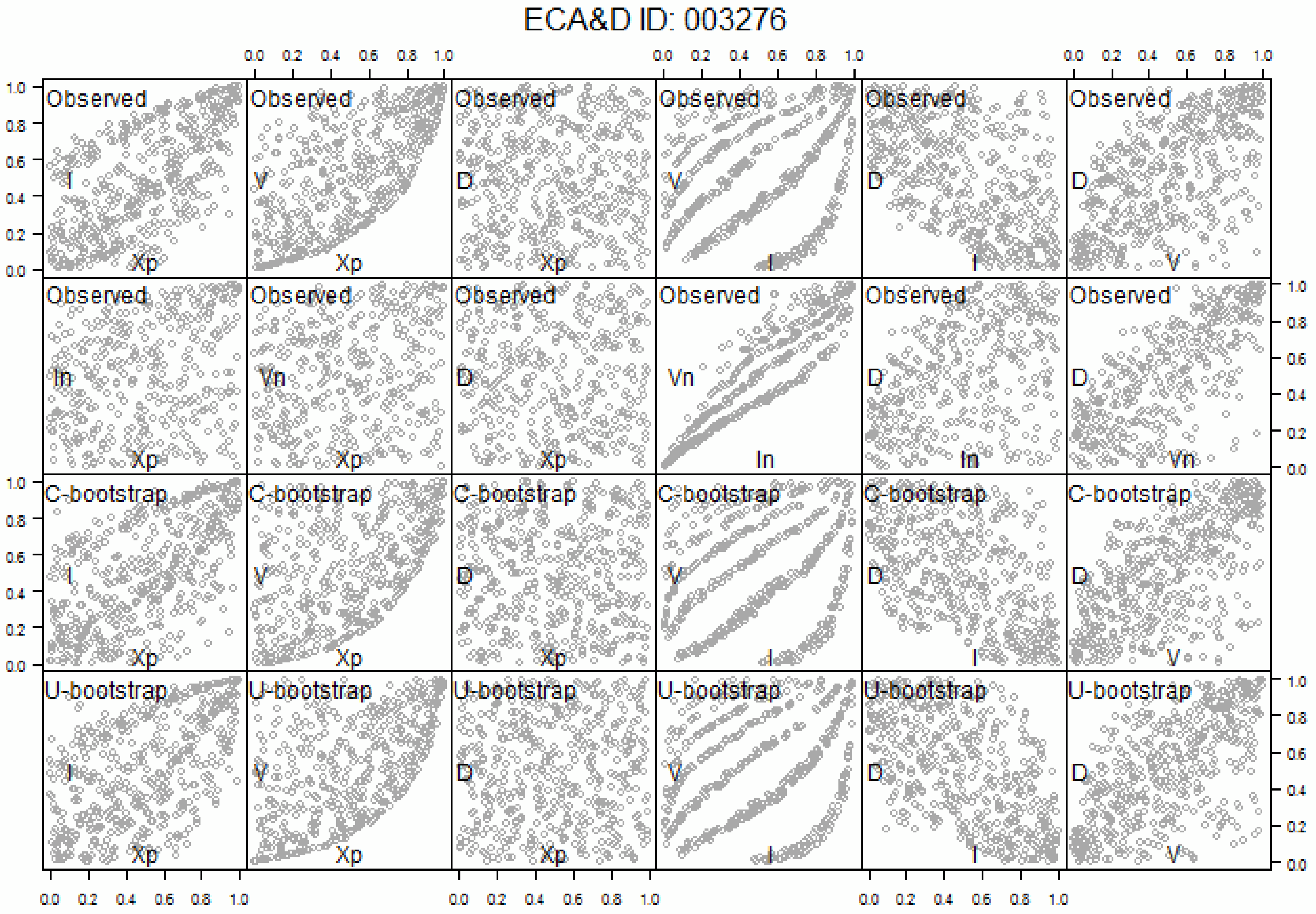
ECA&D ID: 003276
Observed
C-bootstrap
U-bootstrap
Xp
I
V
D
In
Vn
0.0
0.2
0.4
0.6
0.8
1.0

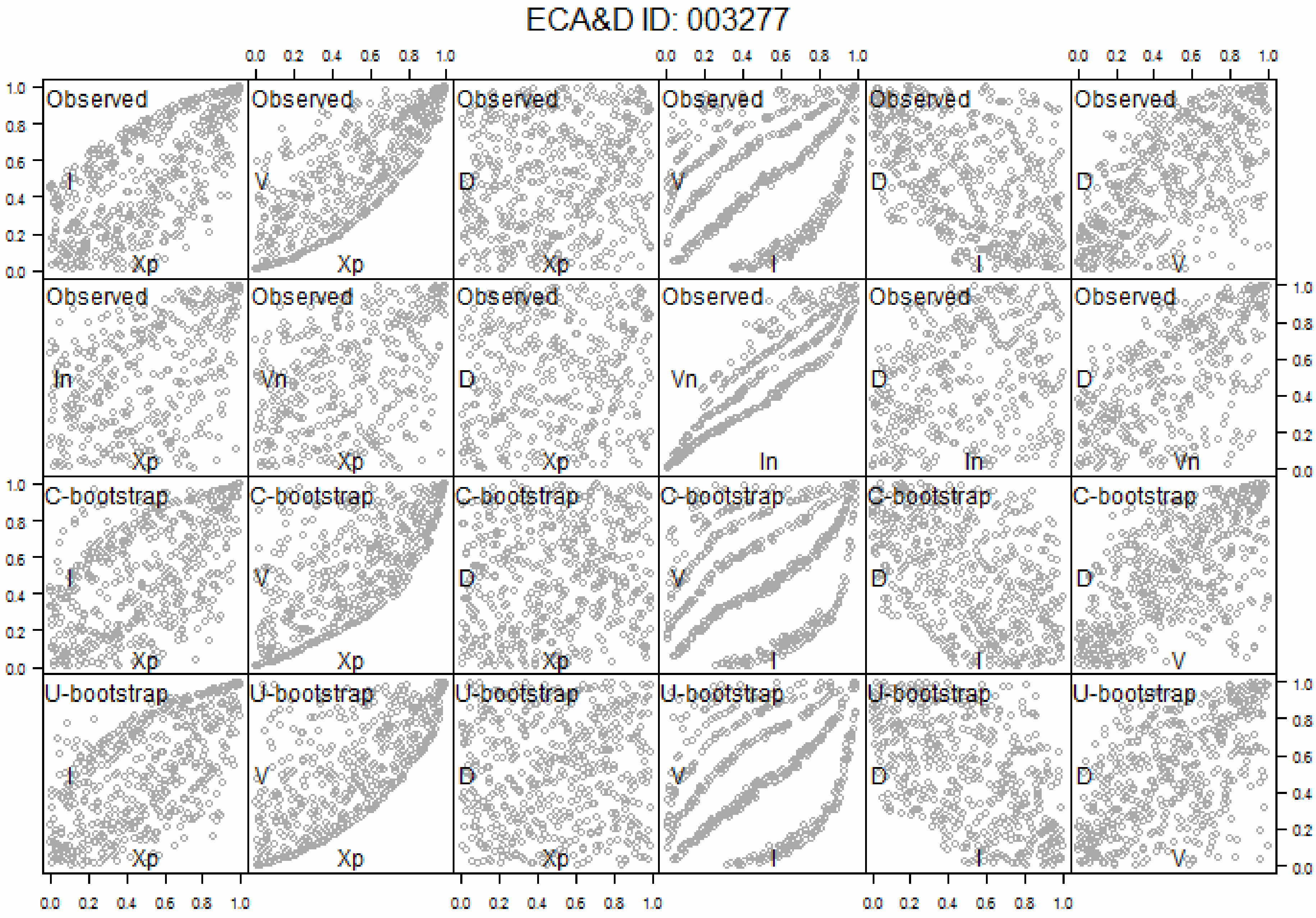
ECA&D ID: 003277
Observed
C-bootstrap
U-bootstrap
Xp
I
V
D
In
Vn
0.0
0.2
0.4
0.6
0.8
1.0

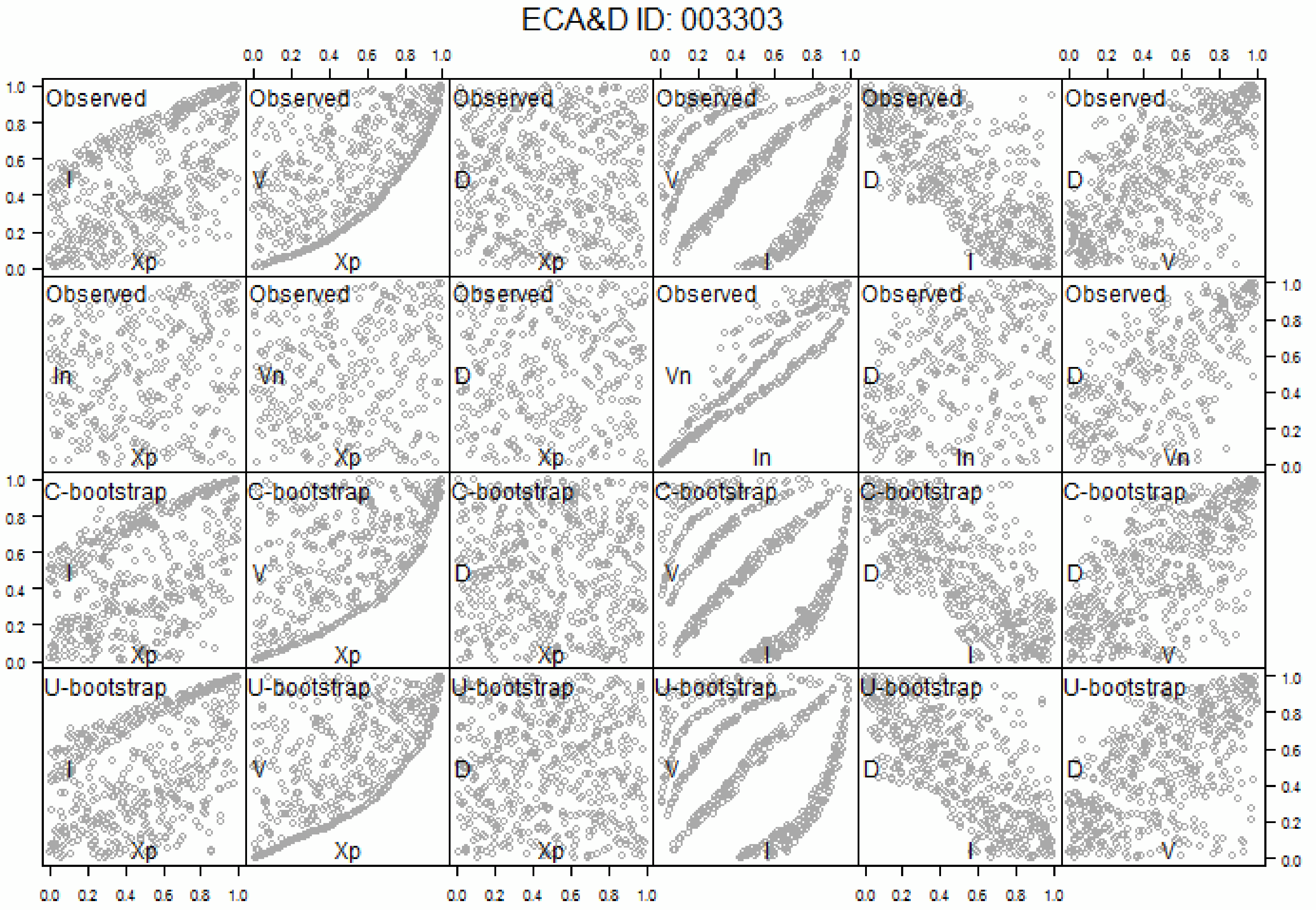
ECA&D ID: 003303
Observed
C-bootstrap
U-bootstrap
Xp
I
V
D
In
Vn
0.0
0.2
0.4
0.6
0.8
1.0

ECA&D ID: 003305

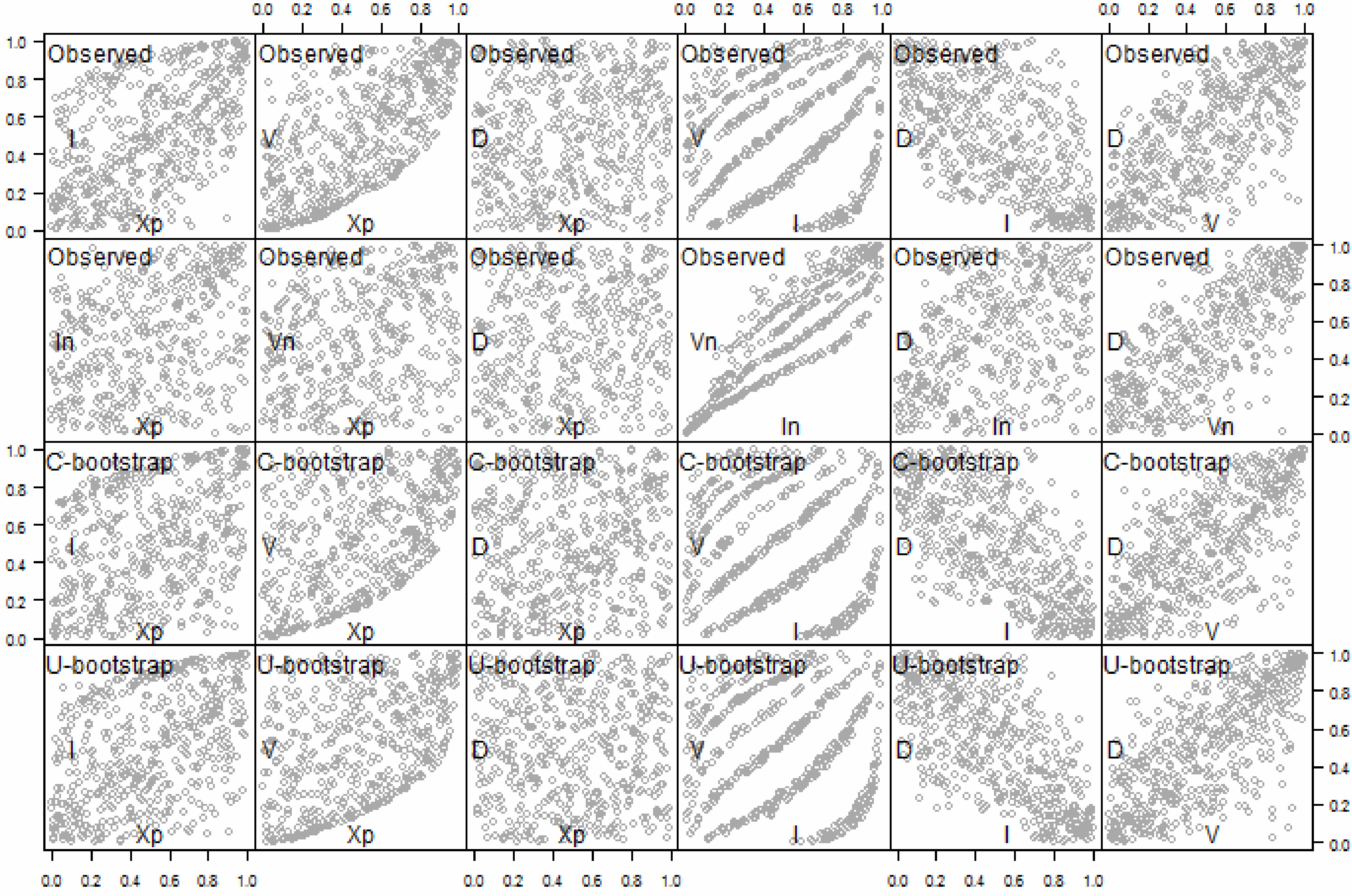

ECA&D ID: 003306

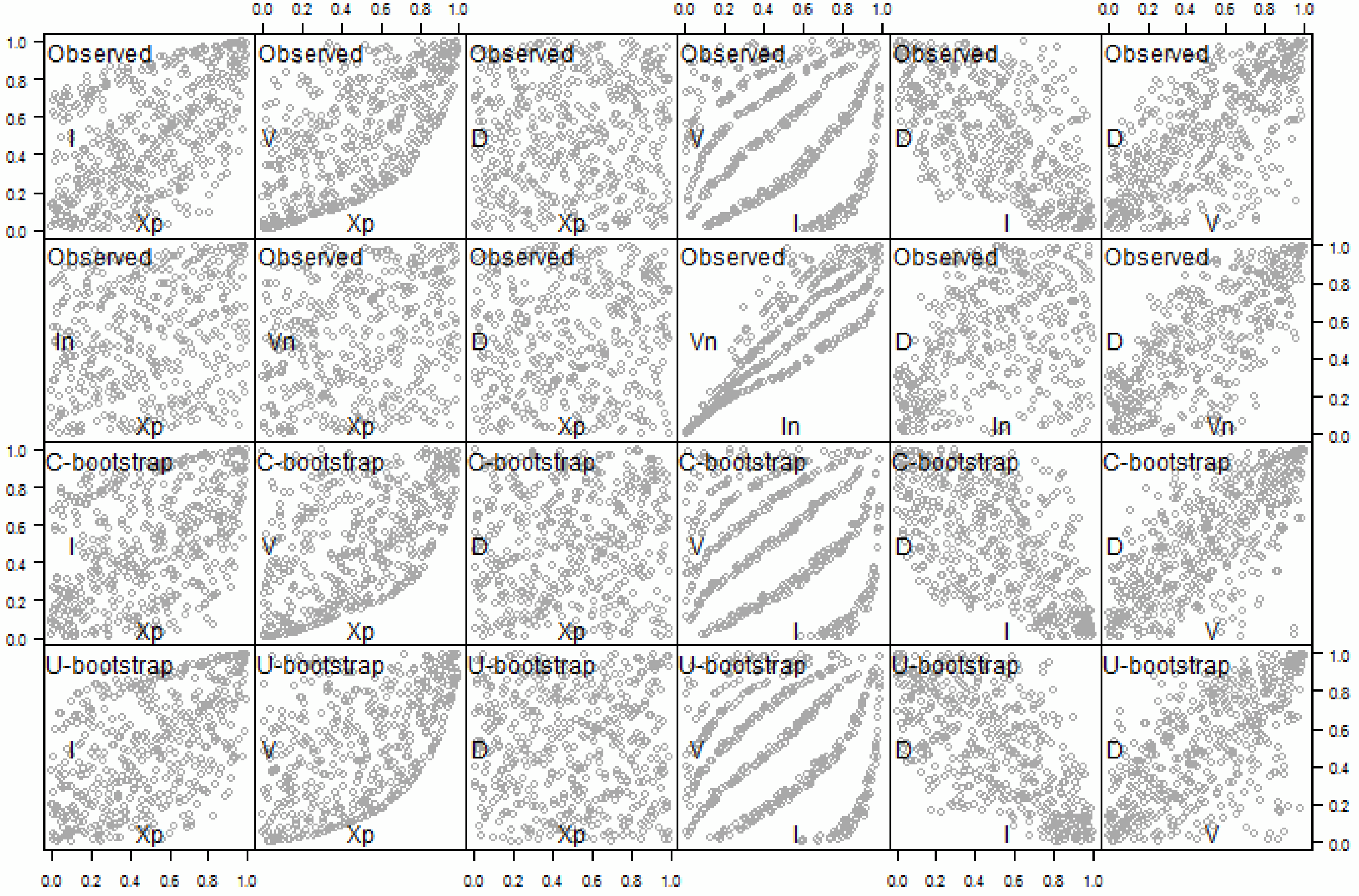

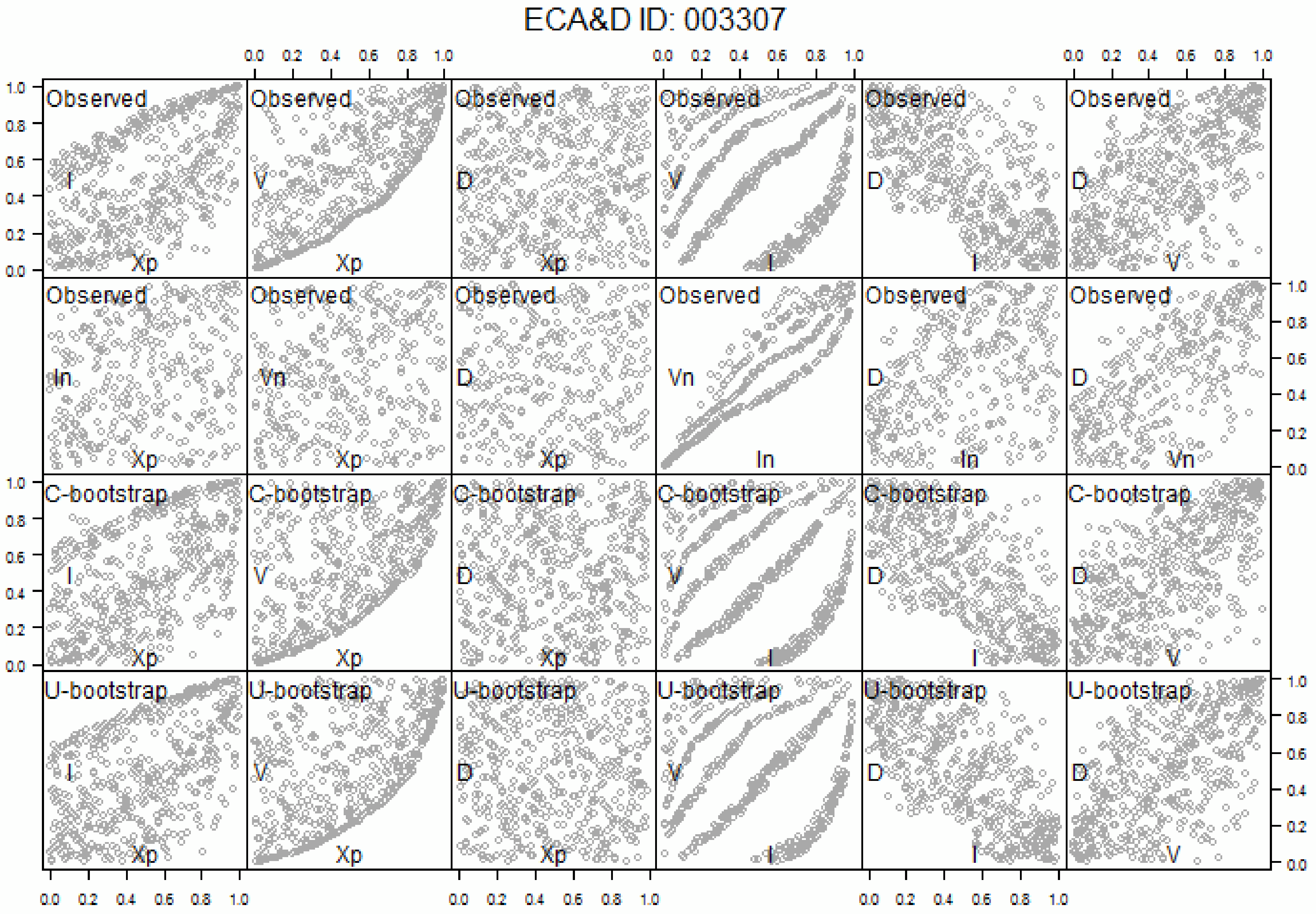
ECA&D ID: 003307
Observed
C-bootstrap
U-bootstrap
Xp
I
V
D
In
Vn
0.0
0.2
0.4
0.6
0.8
1.0

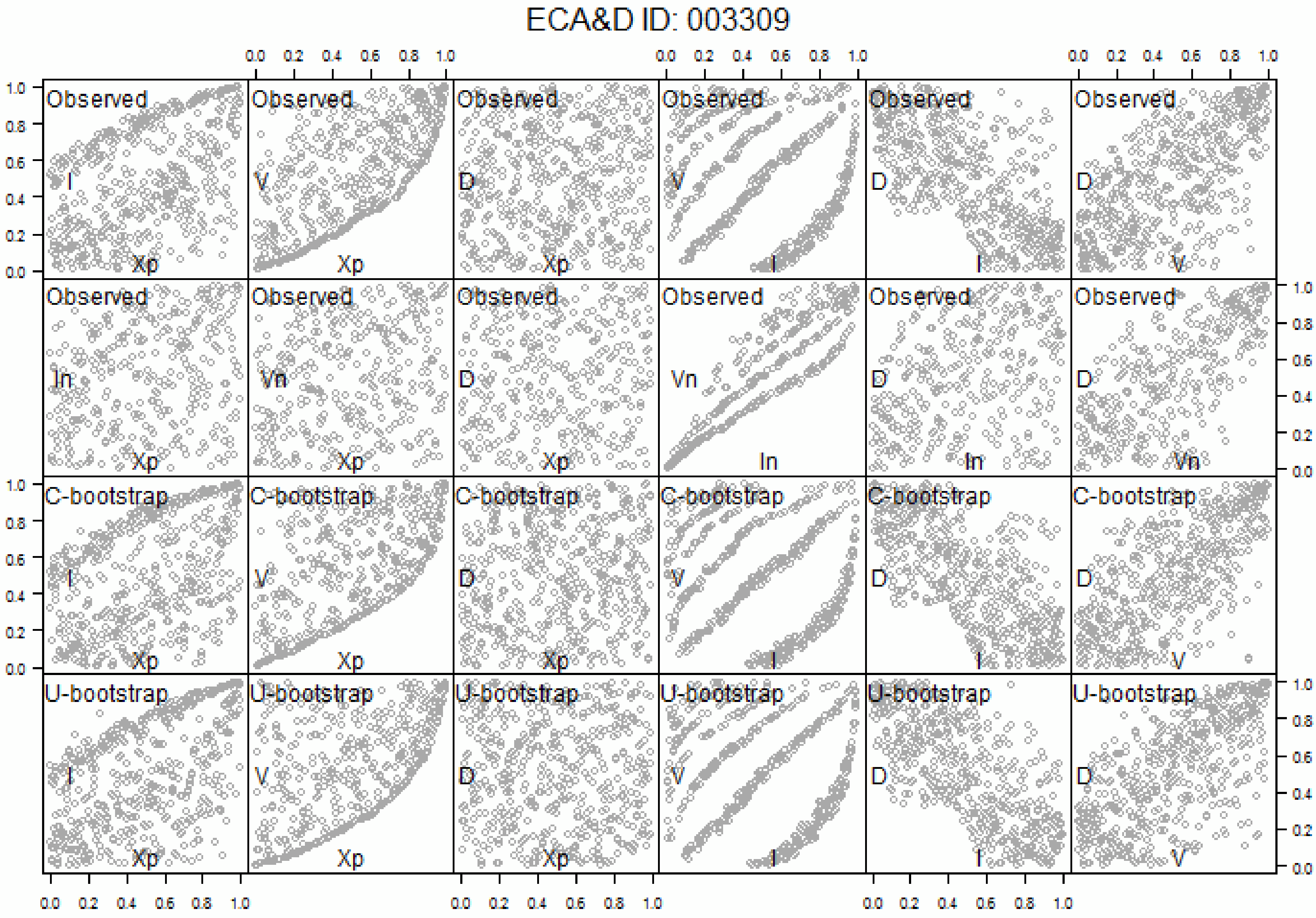
ECA&D ID: 003309
Observed
C-bootstrap
U-bootstrap
Xp
I
V
D
In
Vn
0.0
0.2
0.4
0.6
0.8
1.0

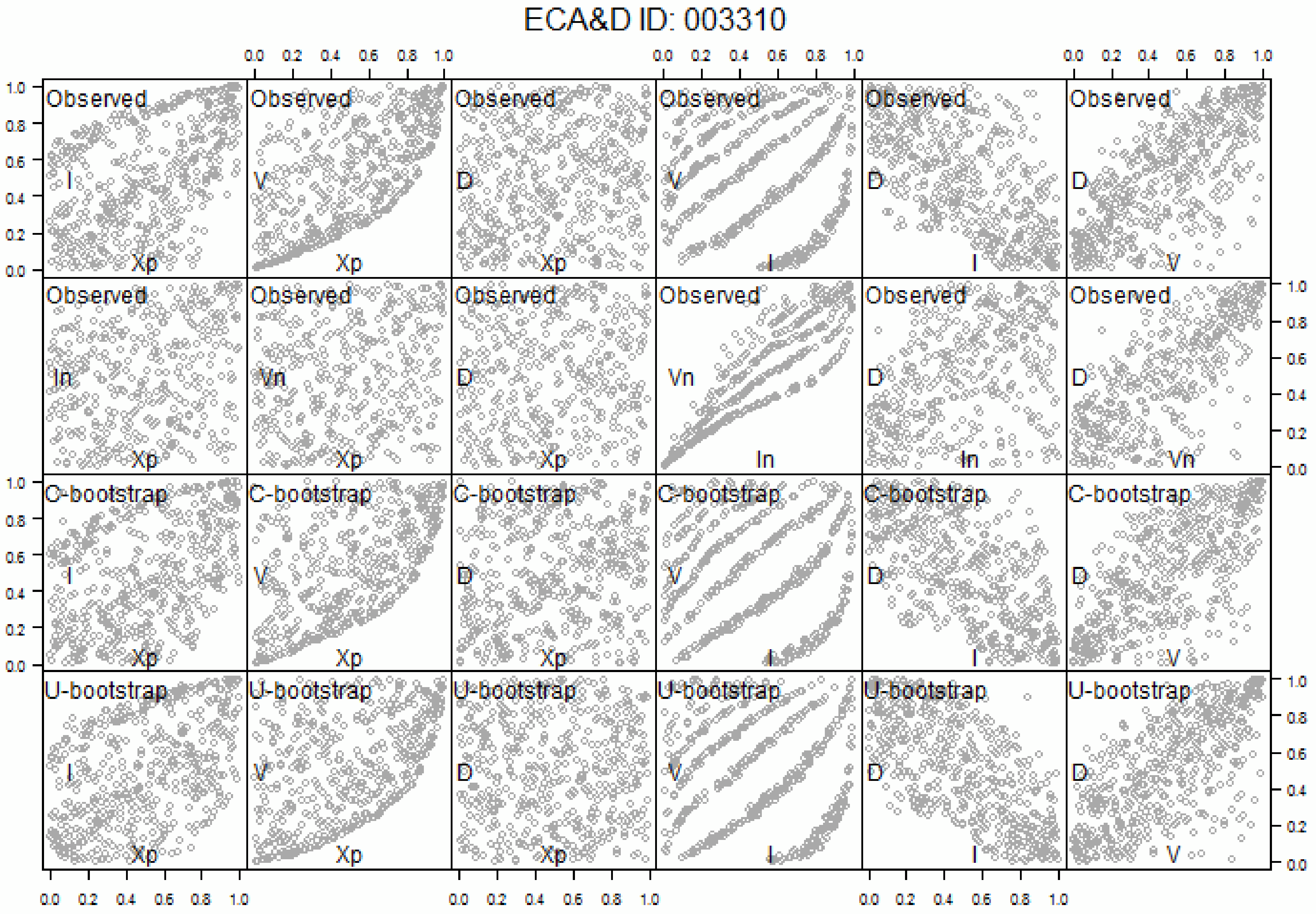

ECA&D ID: 003310
Observed
C-bootstrap
U-bootstrap
Xp
I
V
D
In
Vn
0.0
0.2
0.4
0.6
0.8
1.0

ECA&D ID: 003311

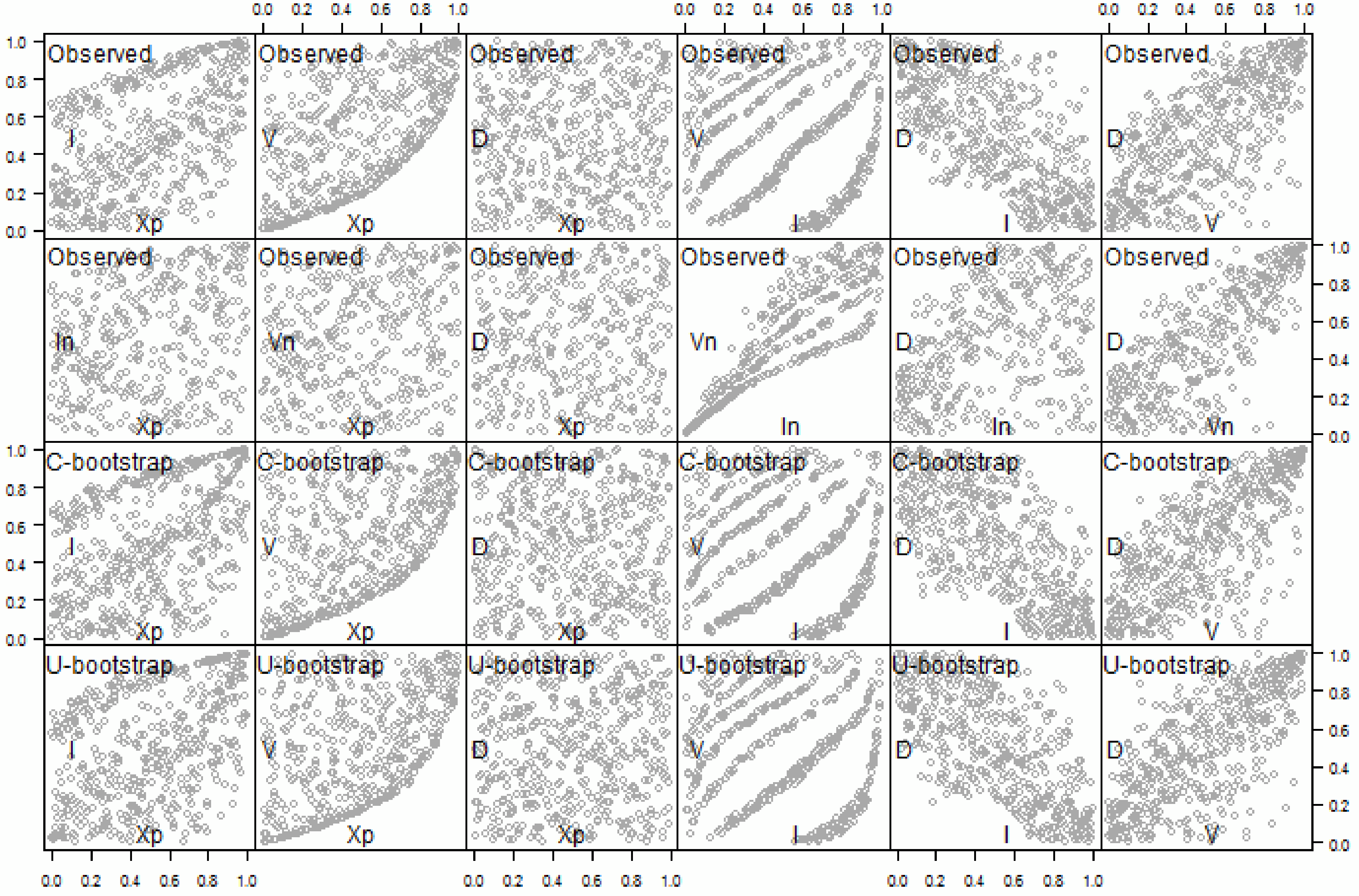

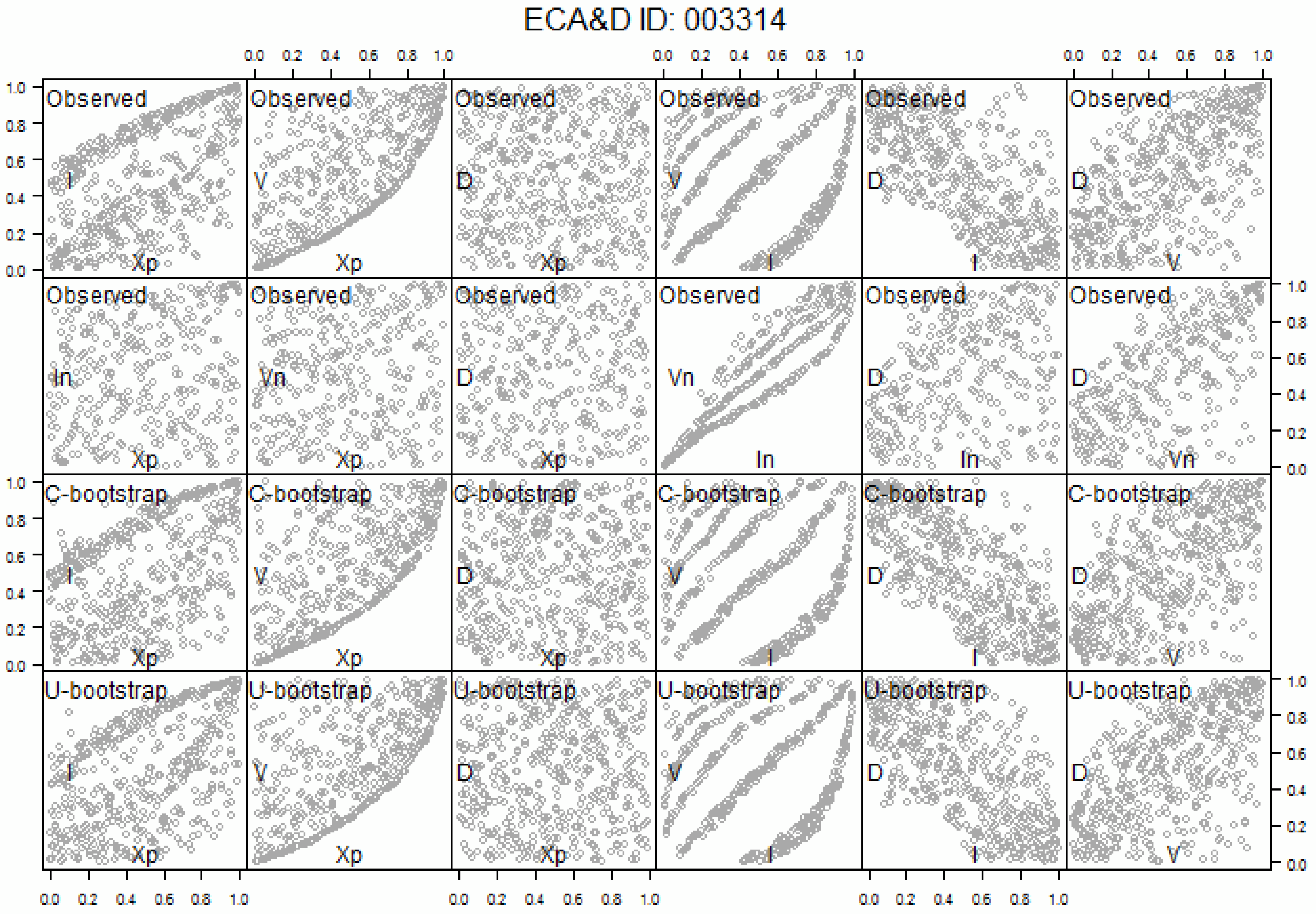

ECA&D ID: 003314
Observed
C-bootstrap
U-bootstrap
Xp
I
V
D
In
Vn
0.0
0.2
0.4
0.6
0.8
1.0

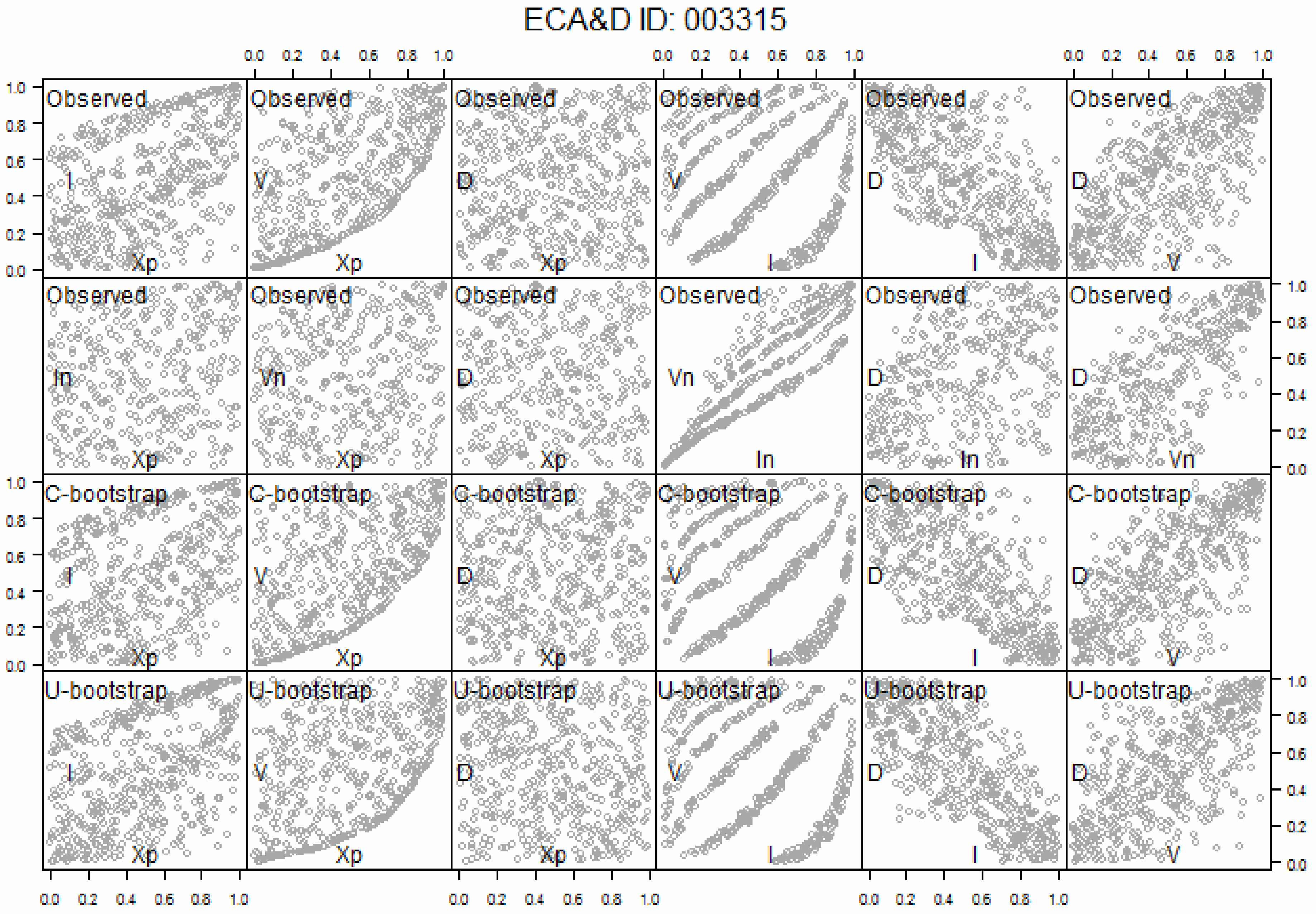

ECA&D ID: 003315
Observed
C-bootstrap
U-bootstrap
Xp
I
V
D
In
Vn
0.0
0.2
0.4
0.6
0.8
1.0

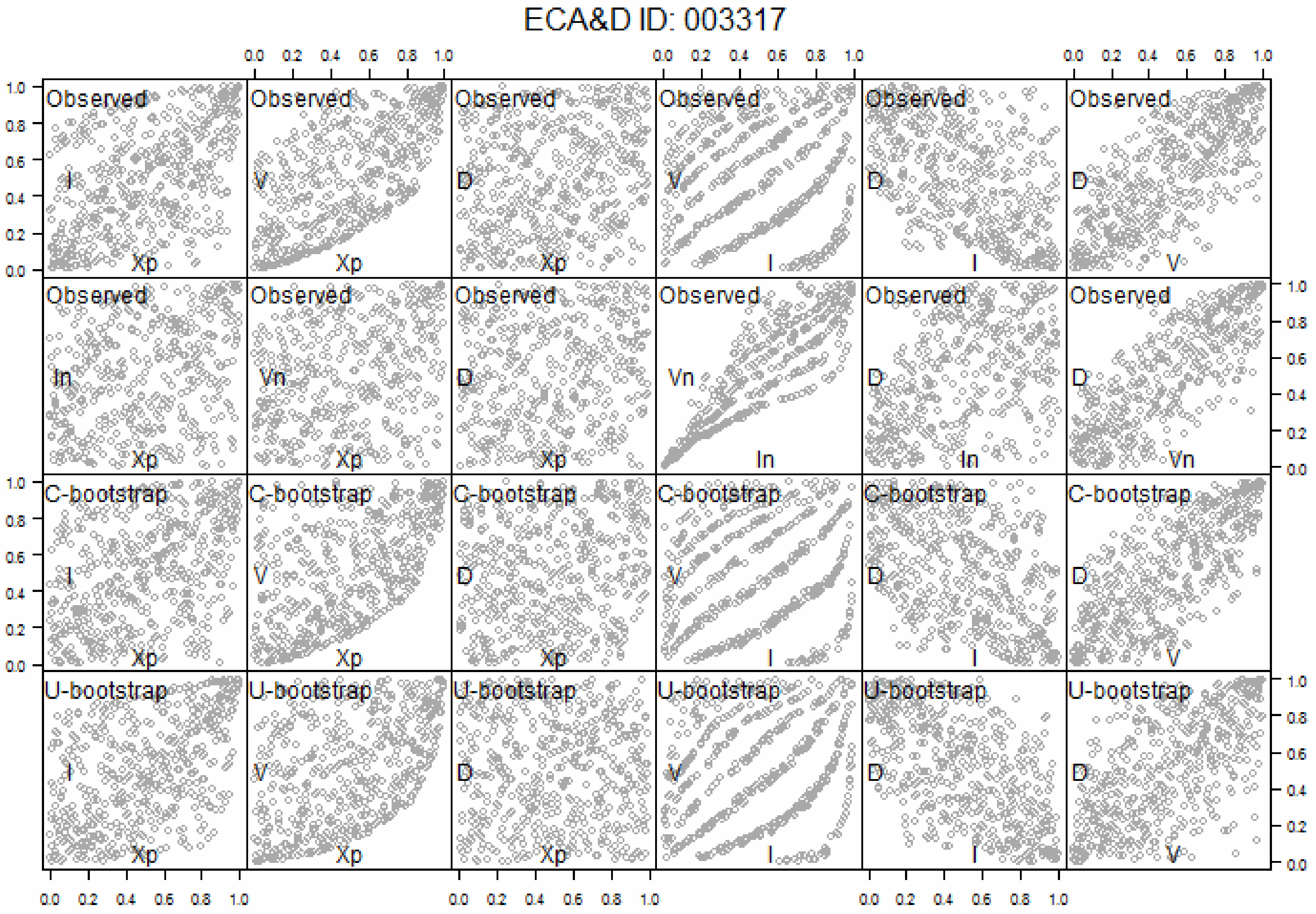

ECA&D ID: 003317
Observed
C-bootstrap
U-bootstrap
I
V
D
In
Vn
Xp
0.0
0.2
0.4
0.6
0.8
1.0

ECA&D ID: 003320

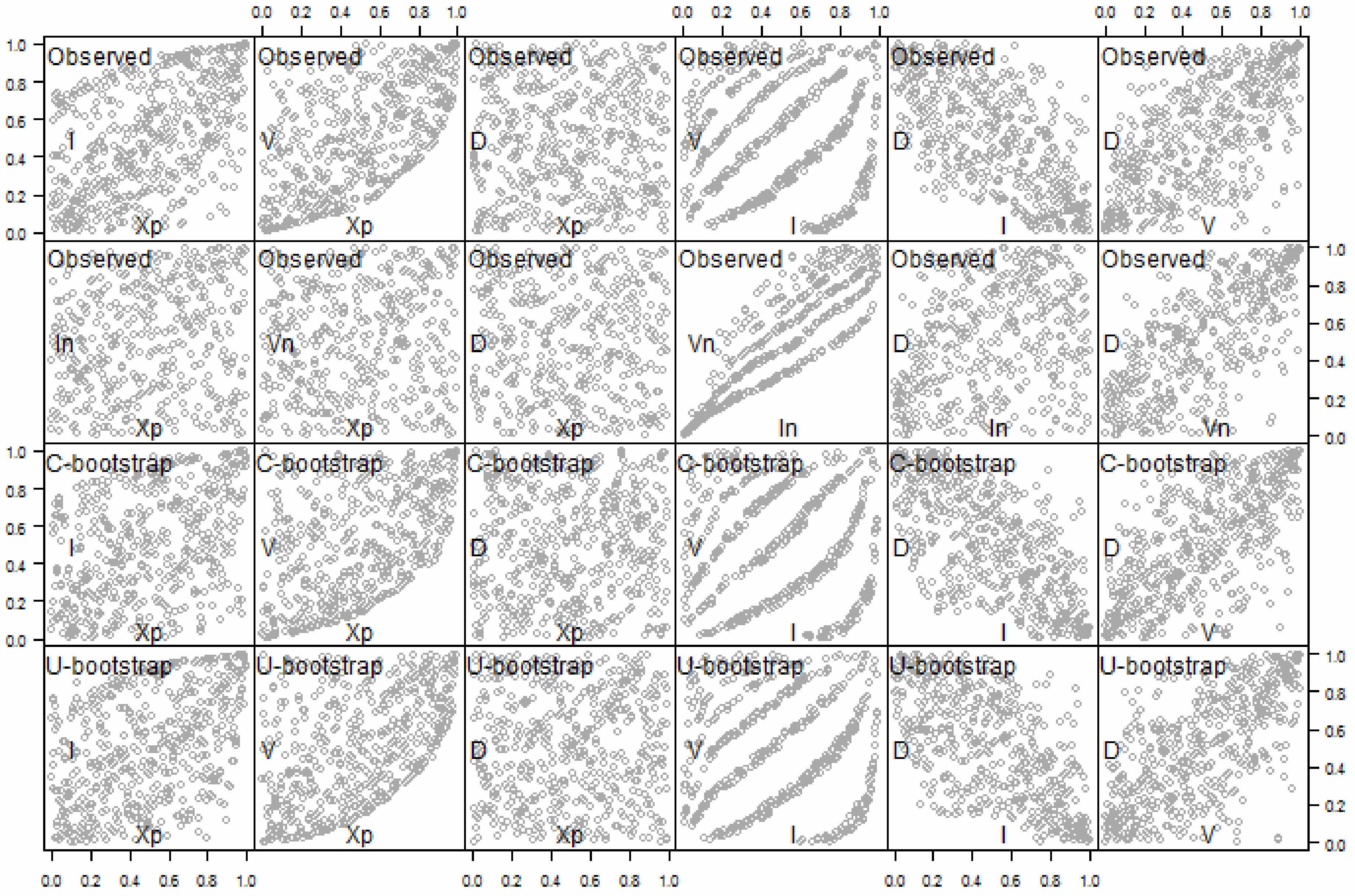

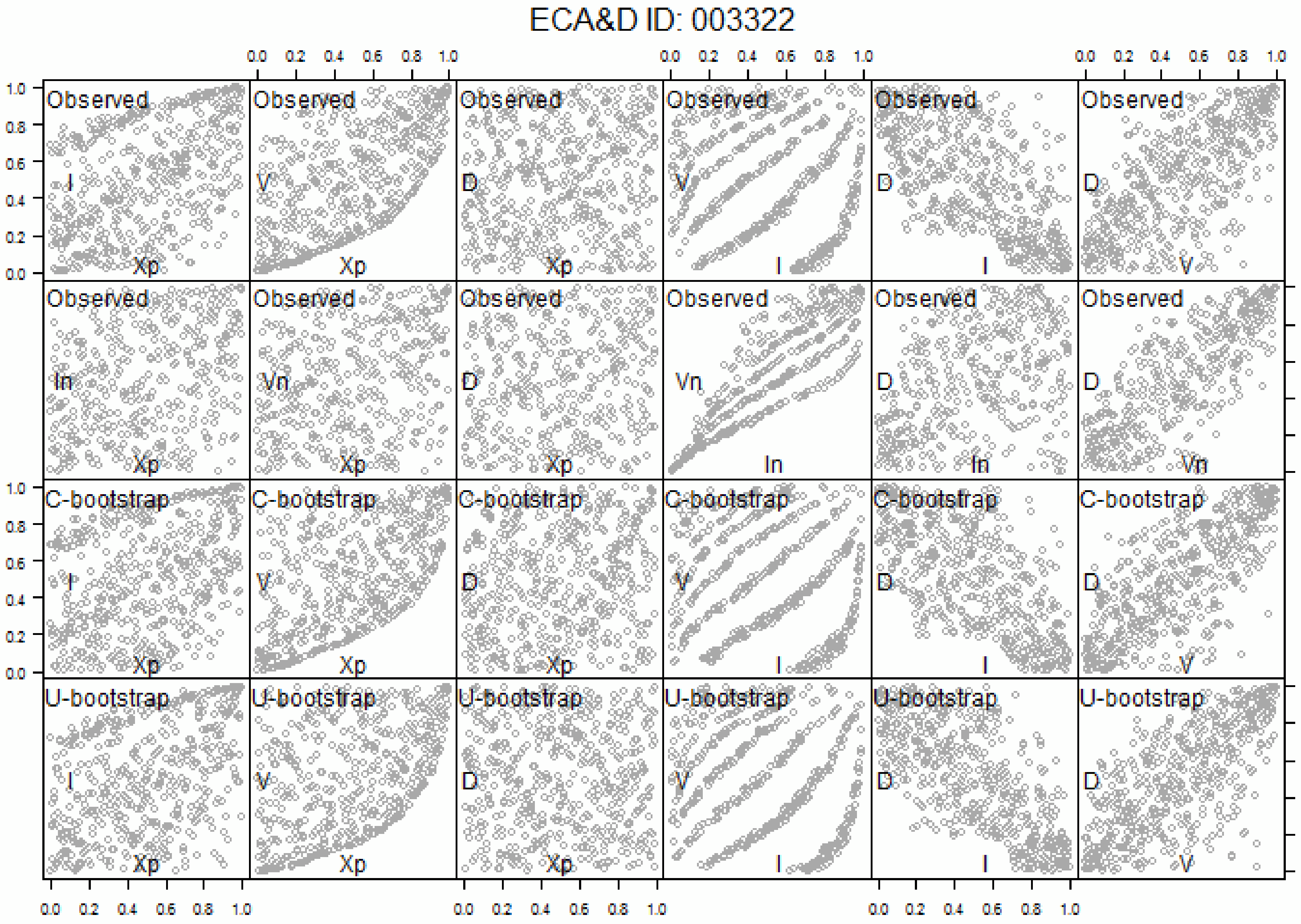

ECA&D ID: 003322
Observed
C-bootstrap
U-bootstrap
I
V
D
In
Vn
Xp
0.0
0.2
0.4
0.6
0.8
1.0

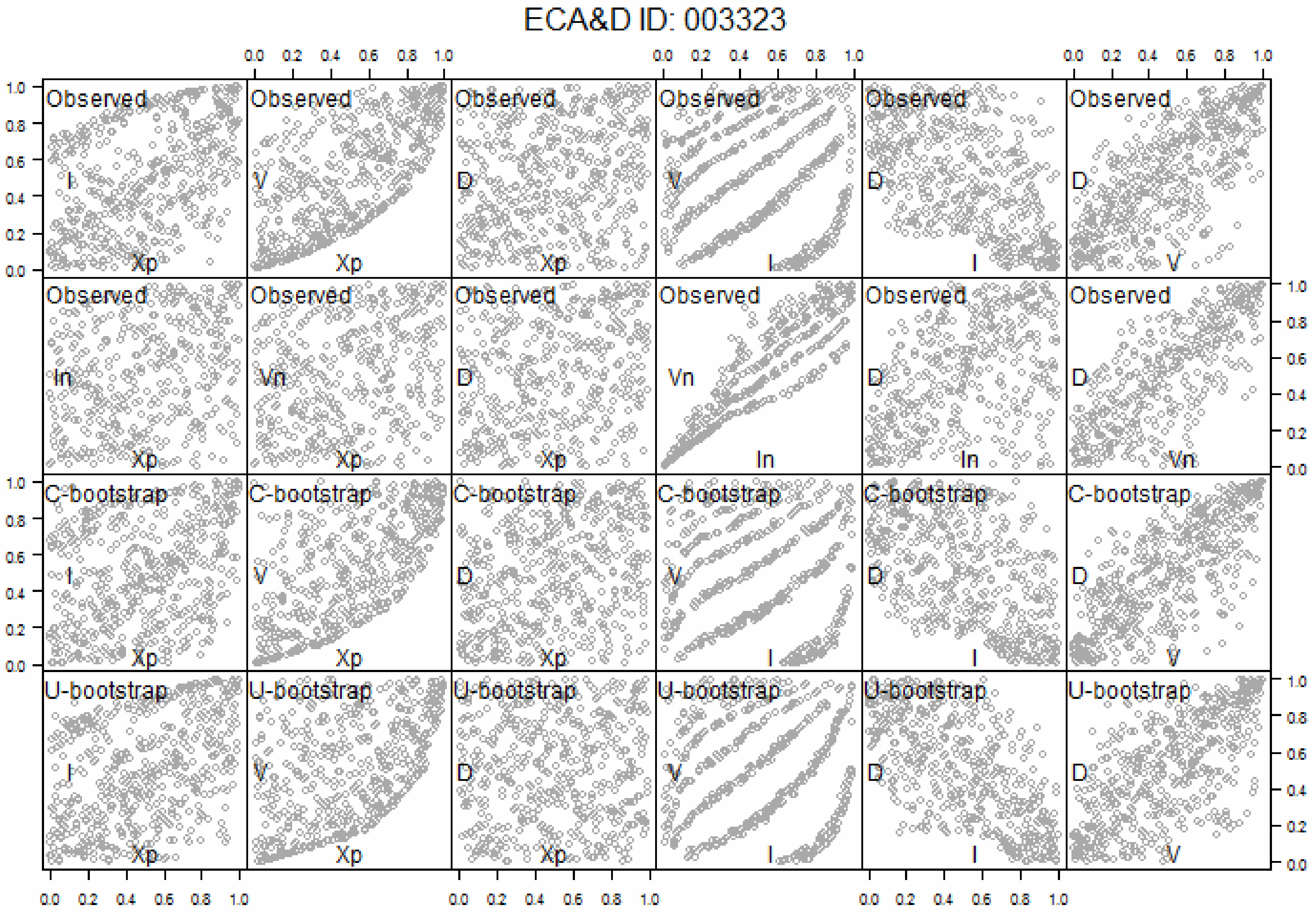
ECA&D ID: 003323
Observed
C-bootstrap
U-bootstrap
Xp
I
V
D
In
Vn
0.0
0.2
0.4
0.6
0.8
1.0

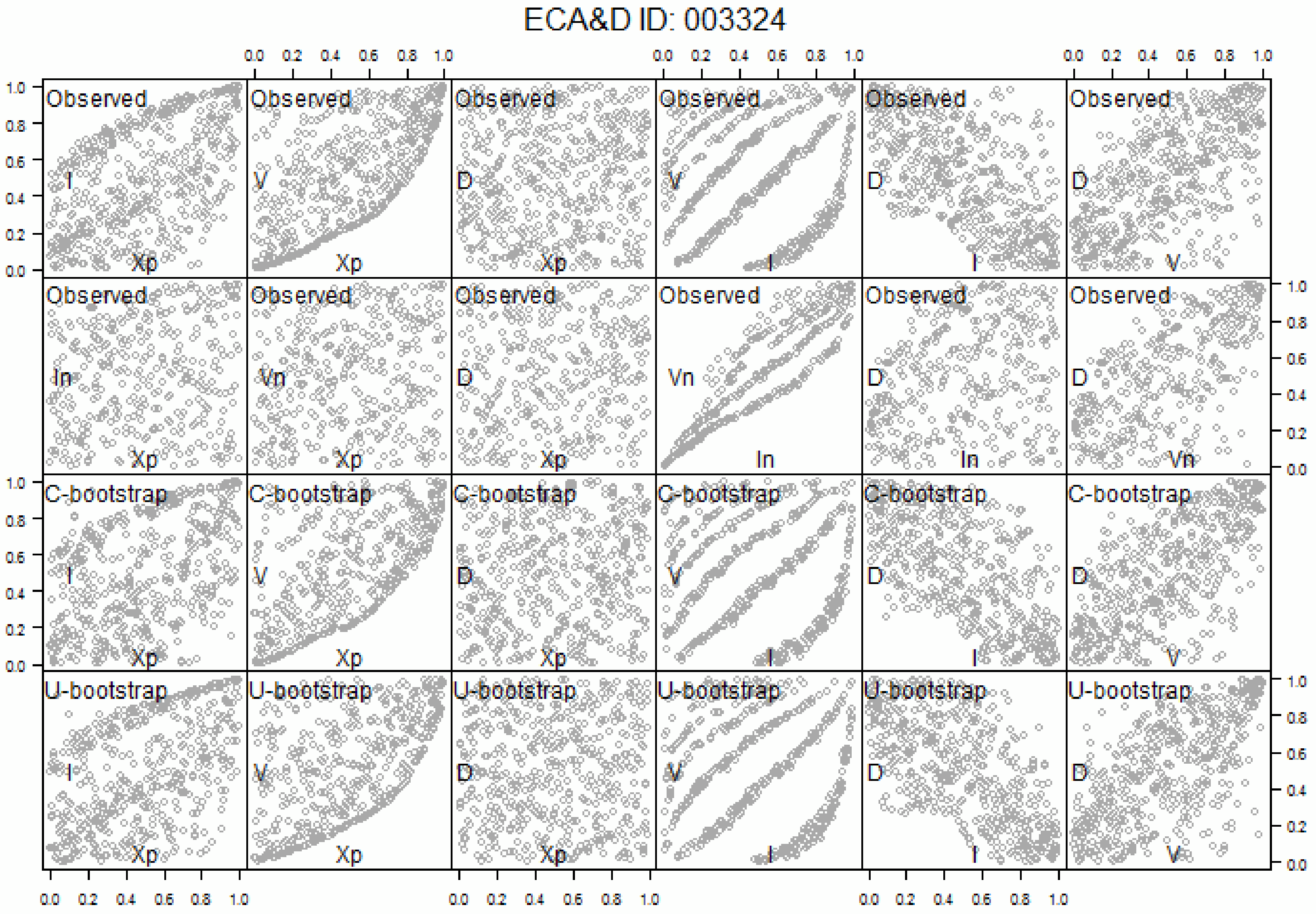
ECA&D ID: 003324
Observed
C-bootstrap
U-bootstrap
Xp
I
V
D
In
Vn
0.0
0.2
0.4
0.6
0.8
1.0

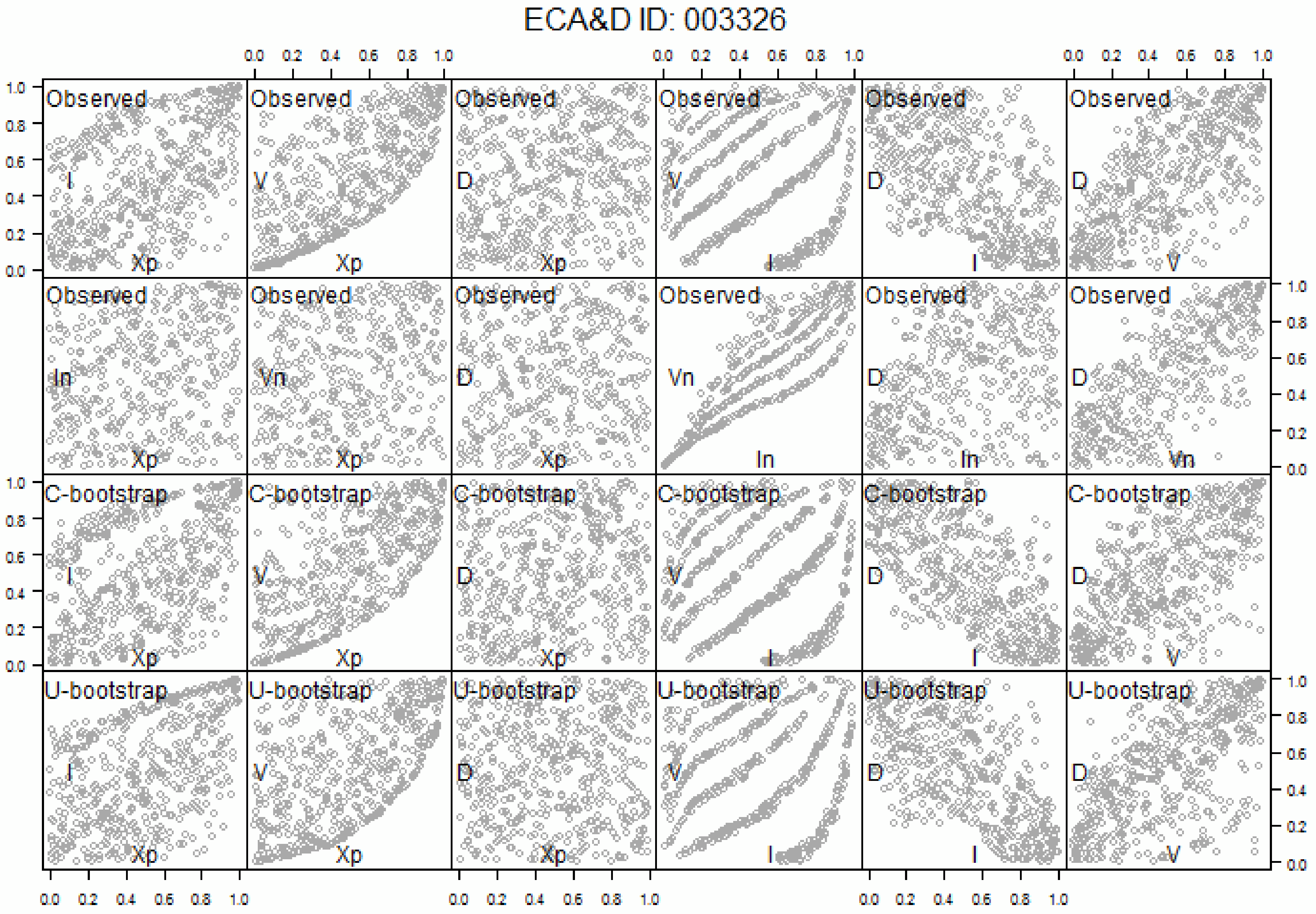
ECA&D ID: 003326
Observed
C-bootstrap
U-bootstrap
Xp
I
V
D
In
Vn
0.0
0.2
0.4
0.6
0.8
1.0

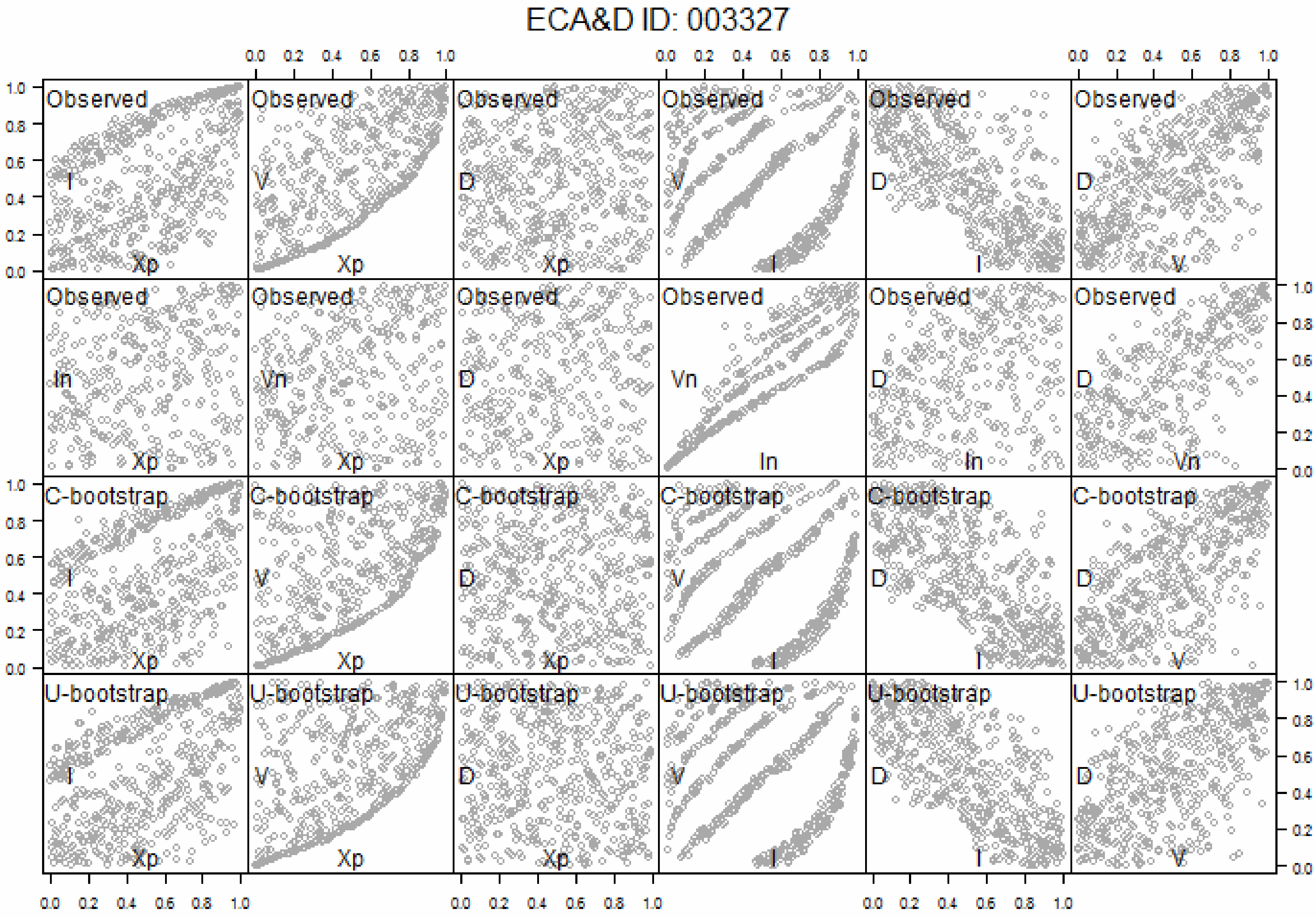
ECA&D ID: 003327
Observed
C-bootstrap
U-bootstrap
Xp
I
V
D
In
Vn
0.0
0.2
0.4
0.6
0.8
1.0

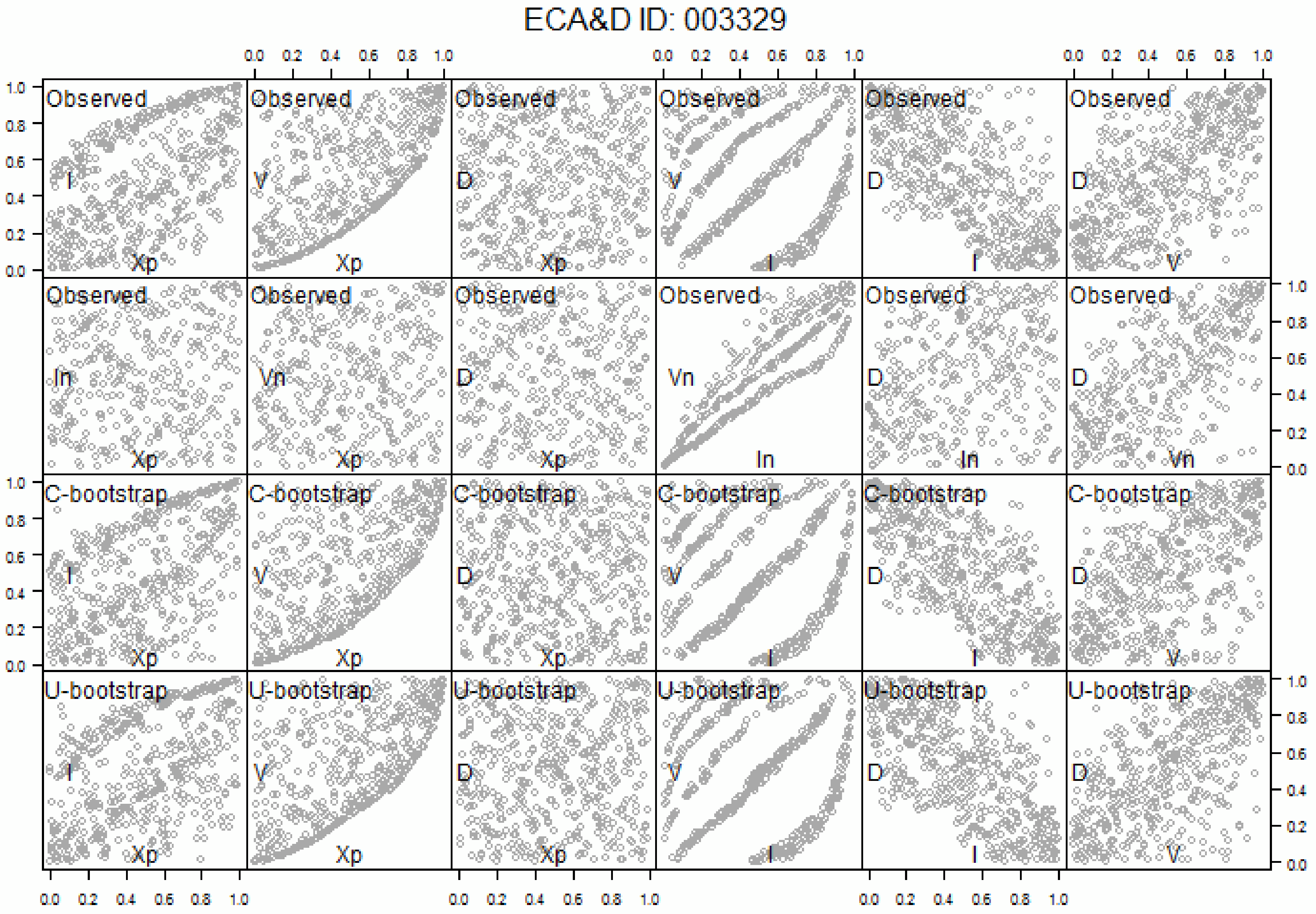
ECA&D ID: 003329
Observed
C-bootstrap
U-bootstrap
Xp
I
V
D
In
Vn
0.0
0.2
0.4
0.6
0.8
1.0

ECA&D ID: 003330

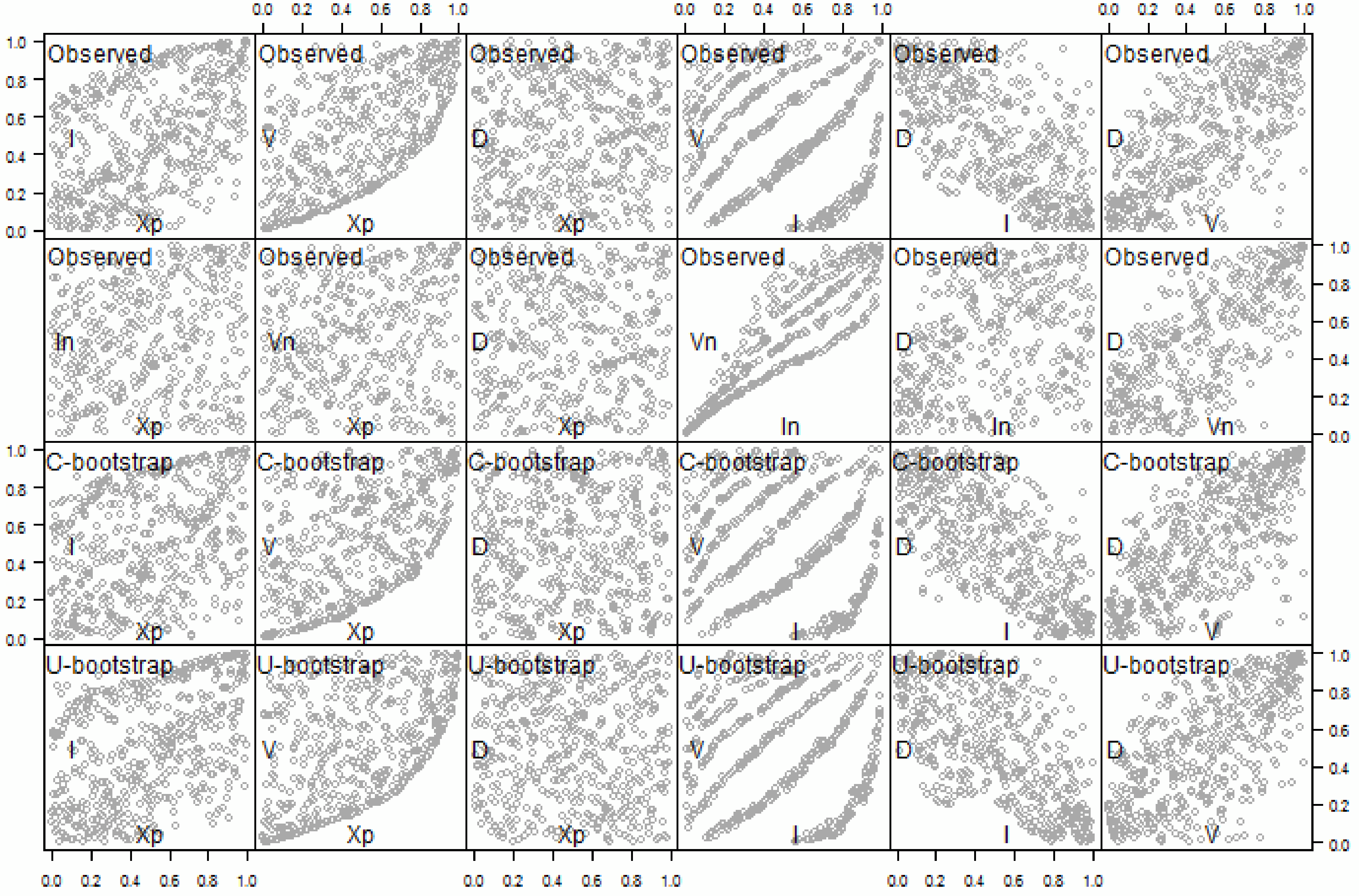

ECA&D ID: 003331

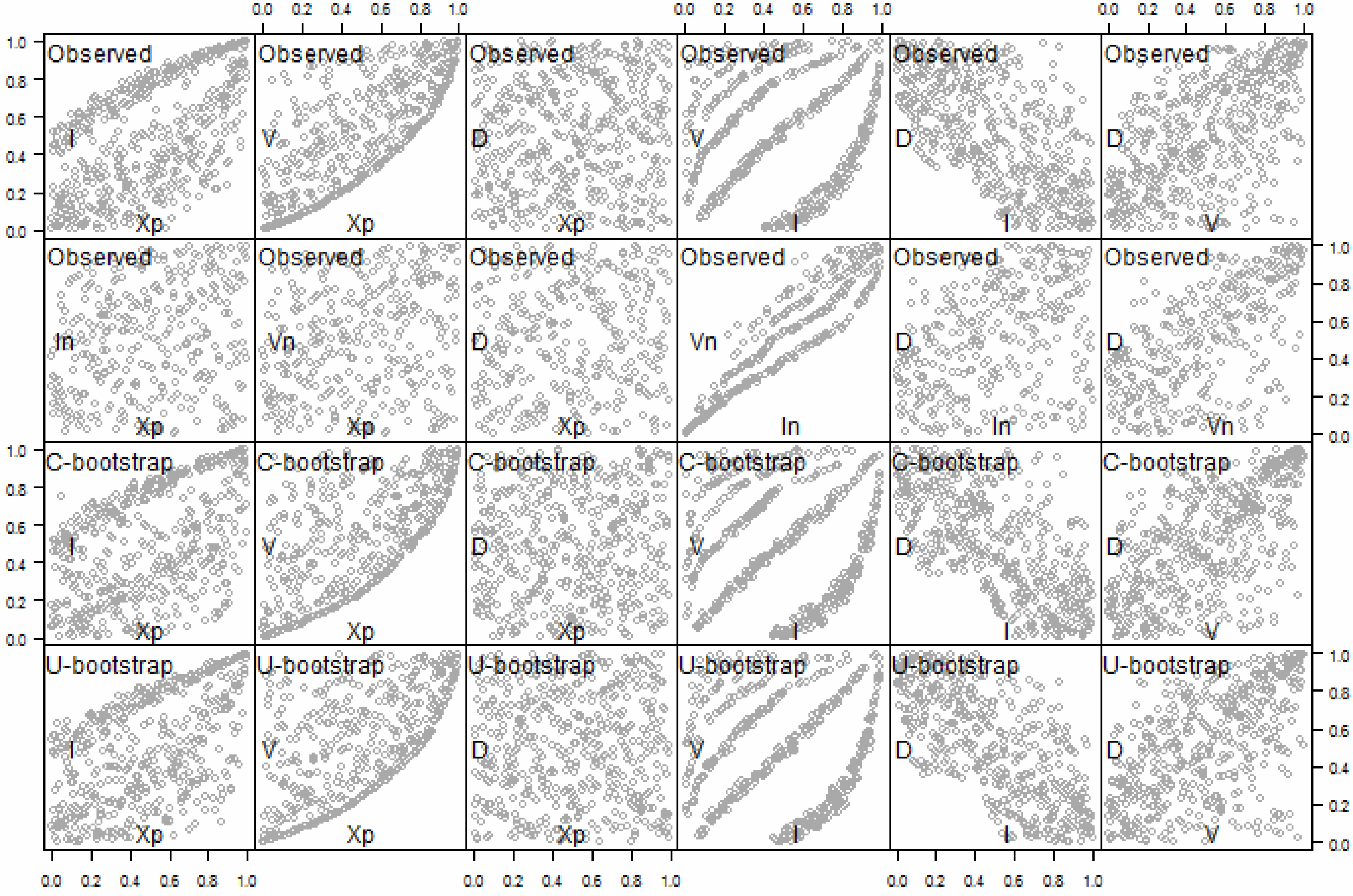

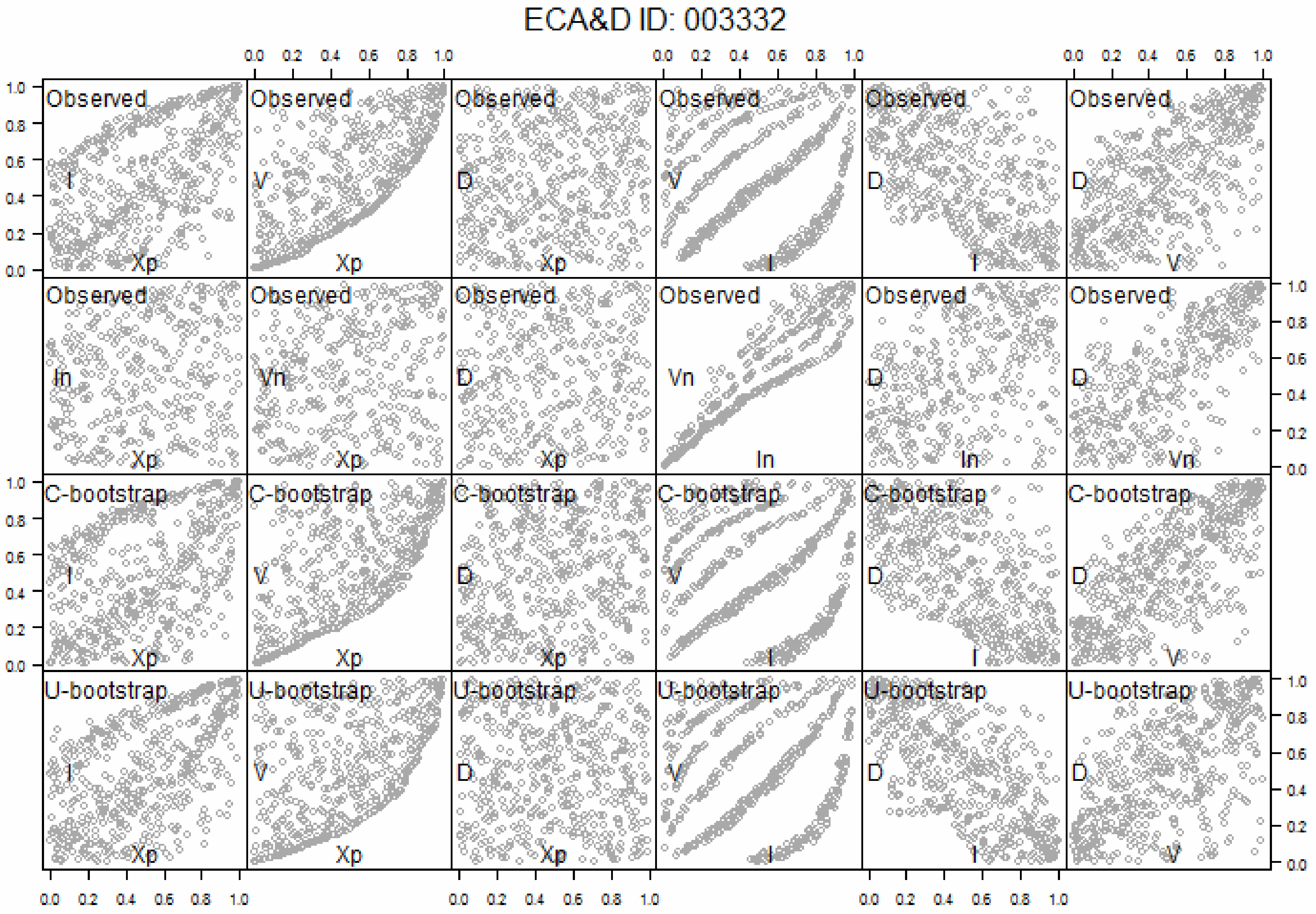

ECA&D ID: 003332
Observed
C-bootstrap
U-bootstrap
Xp
I
V
D
In
Vn
0.0
0.2
0.4
0.6
0.8
1.0

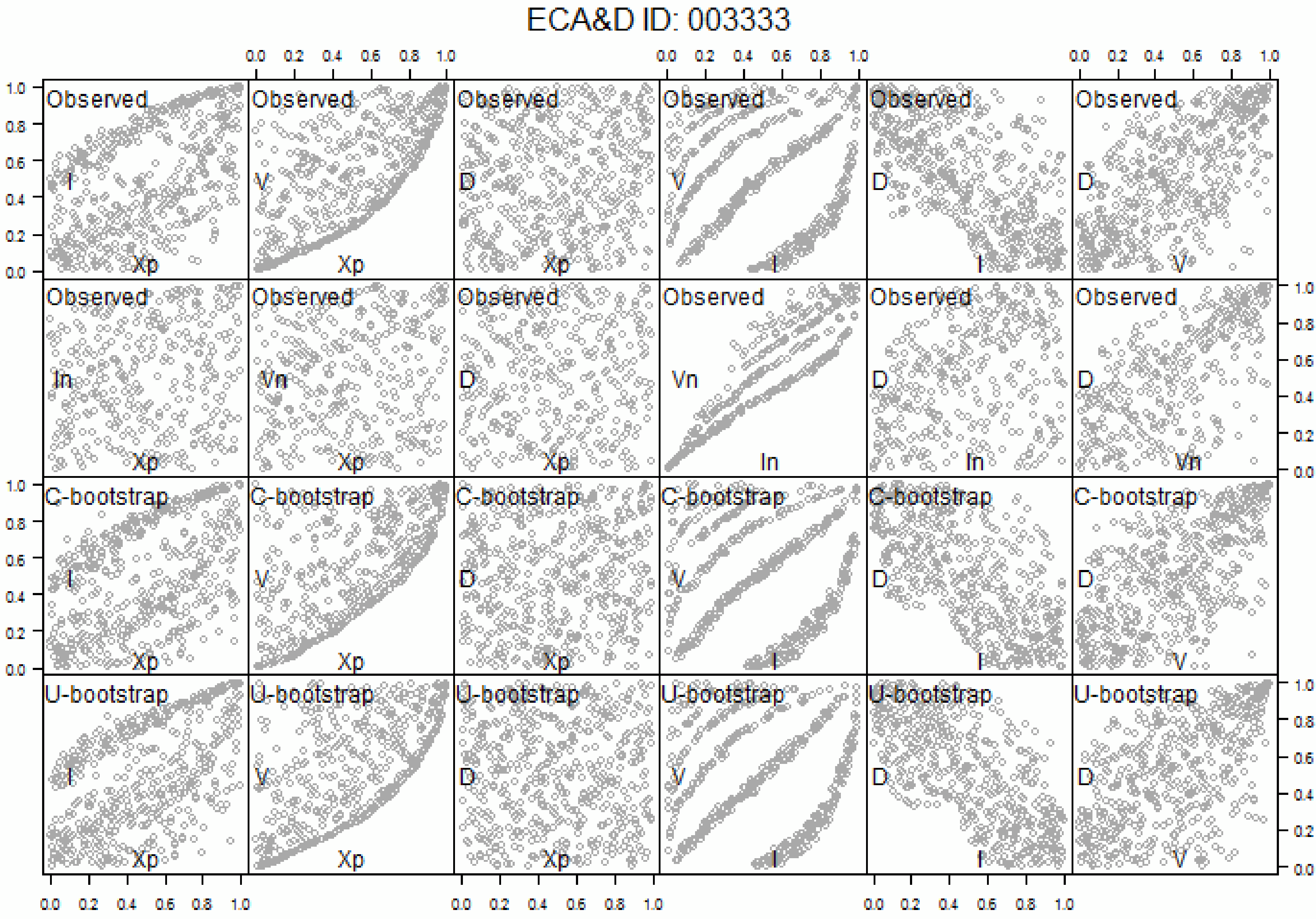
ECA&D ID: 003333
Observed
C-bootstrap
U-bootstrap
Xp
I
V
D
In
Vn
0.0
0.2
0.4
0.6
0.8
1.0

ECA&D ID: 003335

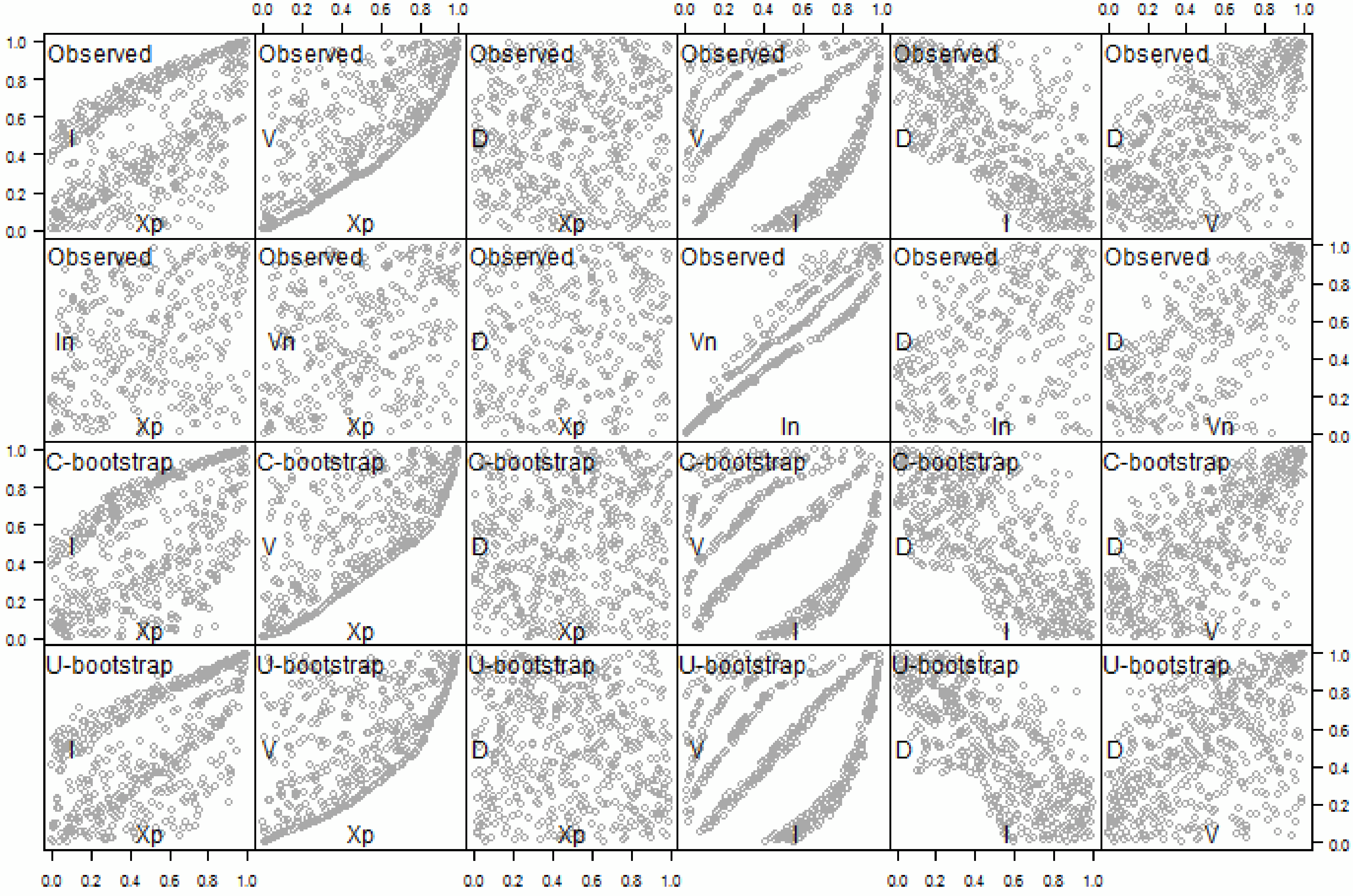

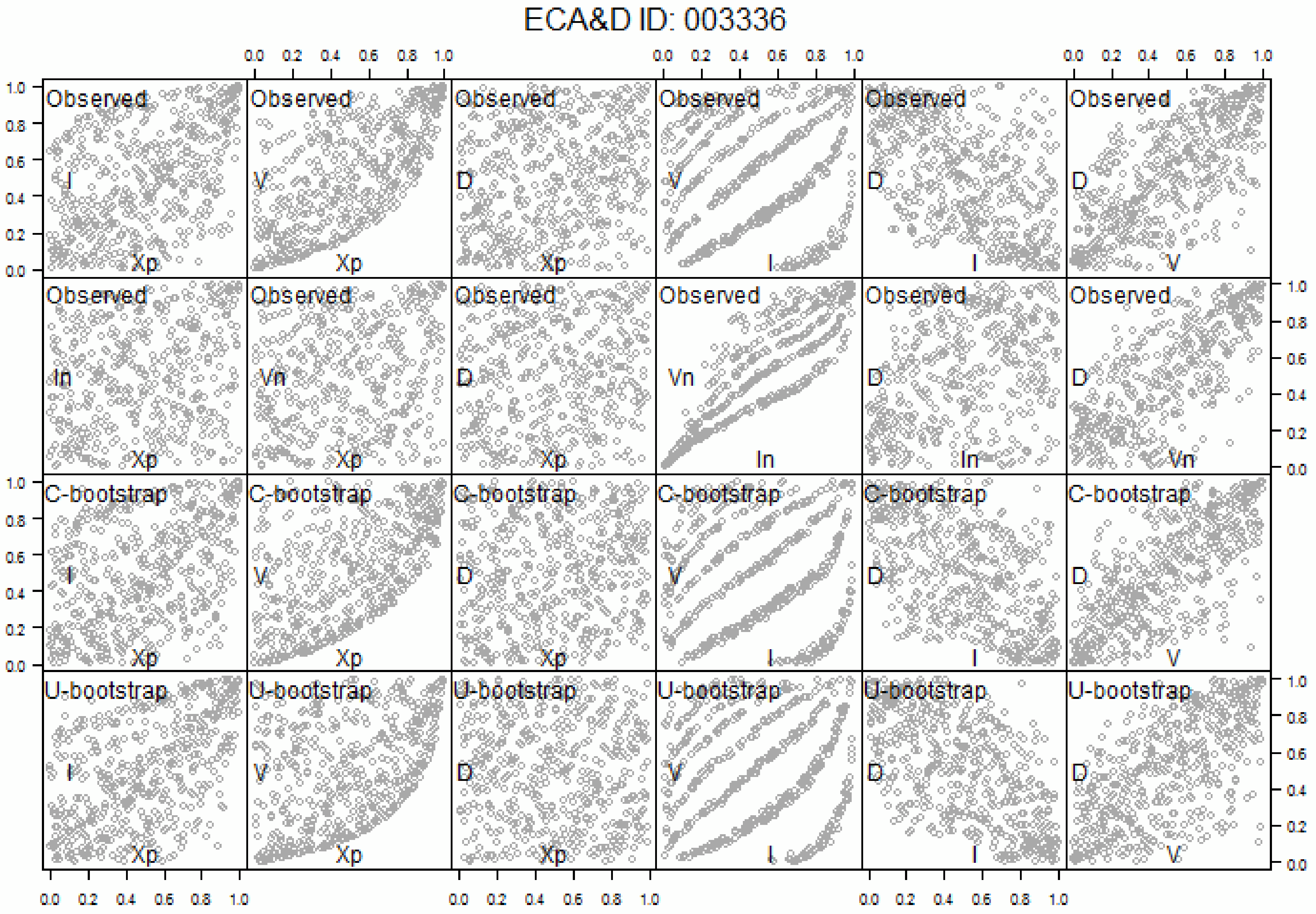
ECA&D ID: 003336
Observed
C-bootstrap
U-bootstrap
Xp
I
V
D
In
Vn
0.0
0.2
0.4
0.6
0.8
1.0

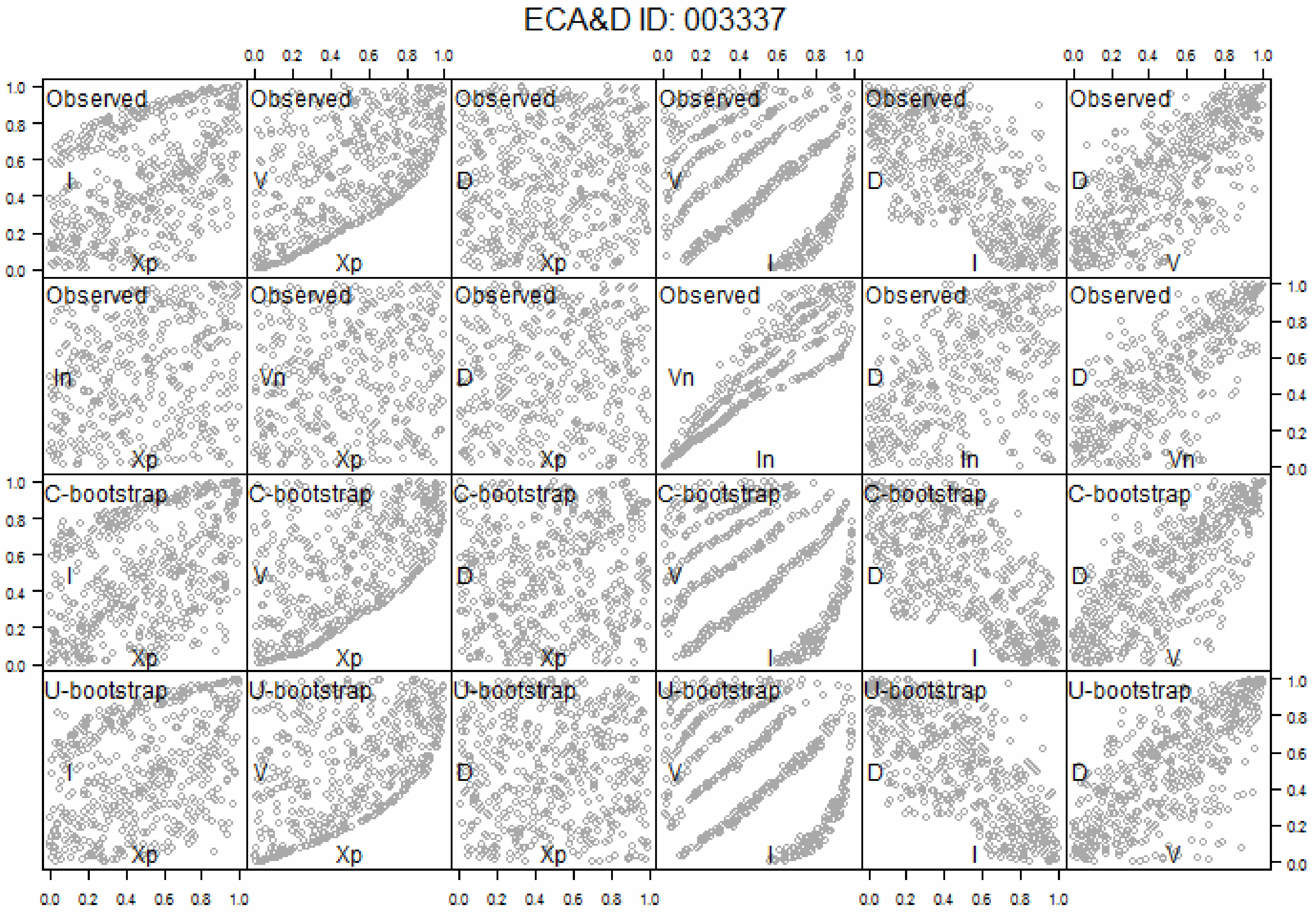

ECA&D ID: 003337
Observed
C-bootstrap
U-bootstrap
Xp
I
V
D
In
Vn
0.0
0.2
0.4
0.6
0.8
1.0

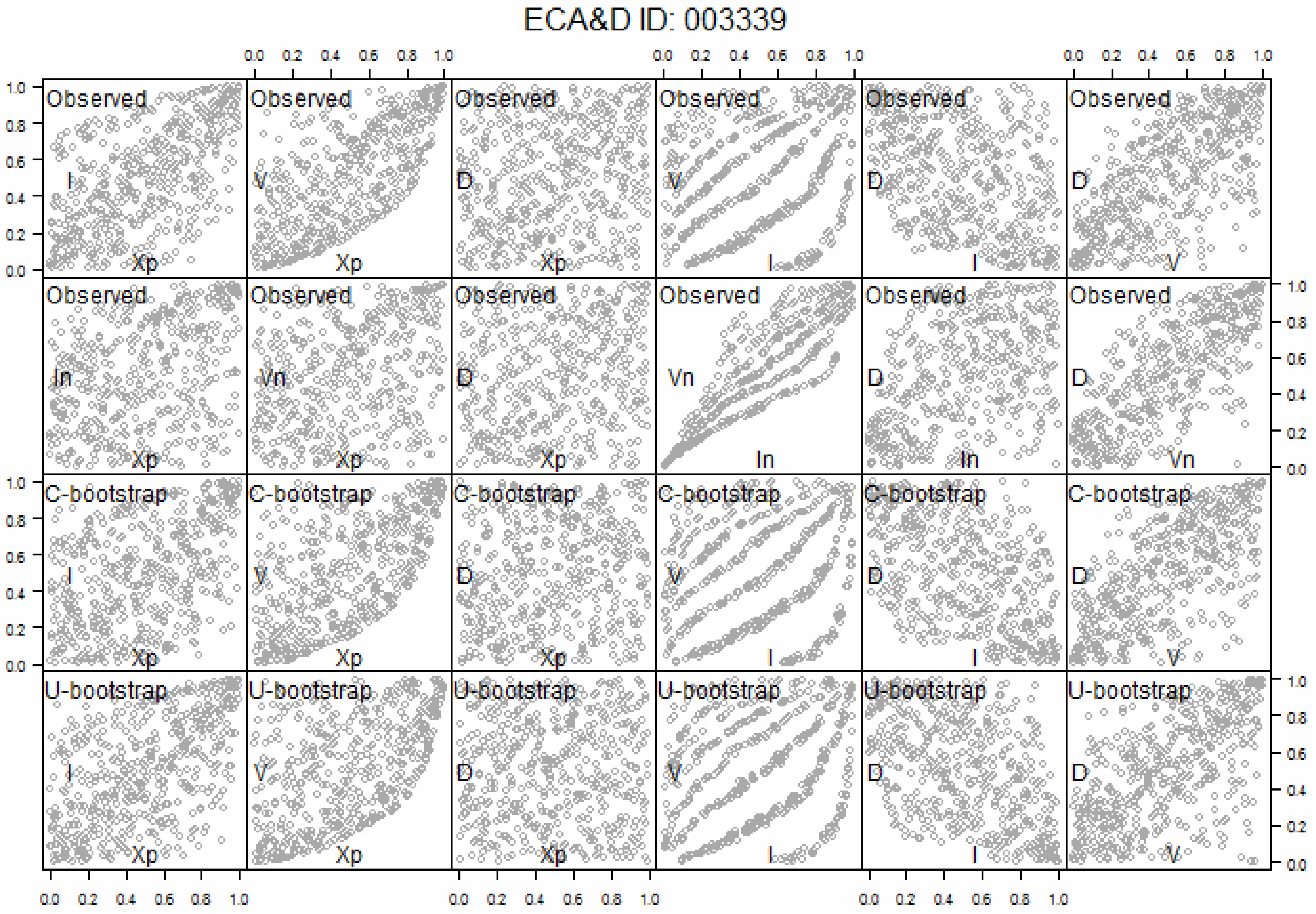
ECA&D ID: 003339
Observed
C-bootstrap
U-bootstrap
Xp
I
V
D
In
Vn
0.0
0.2
0.4
0.6
0.8
1.0

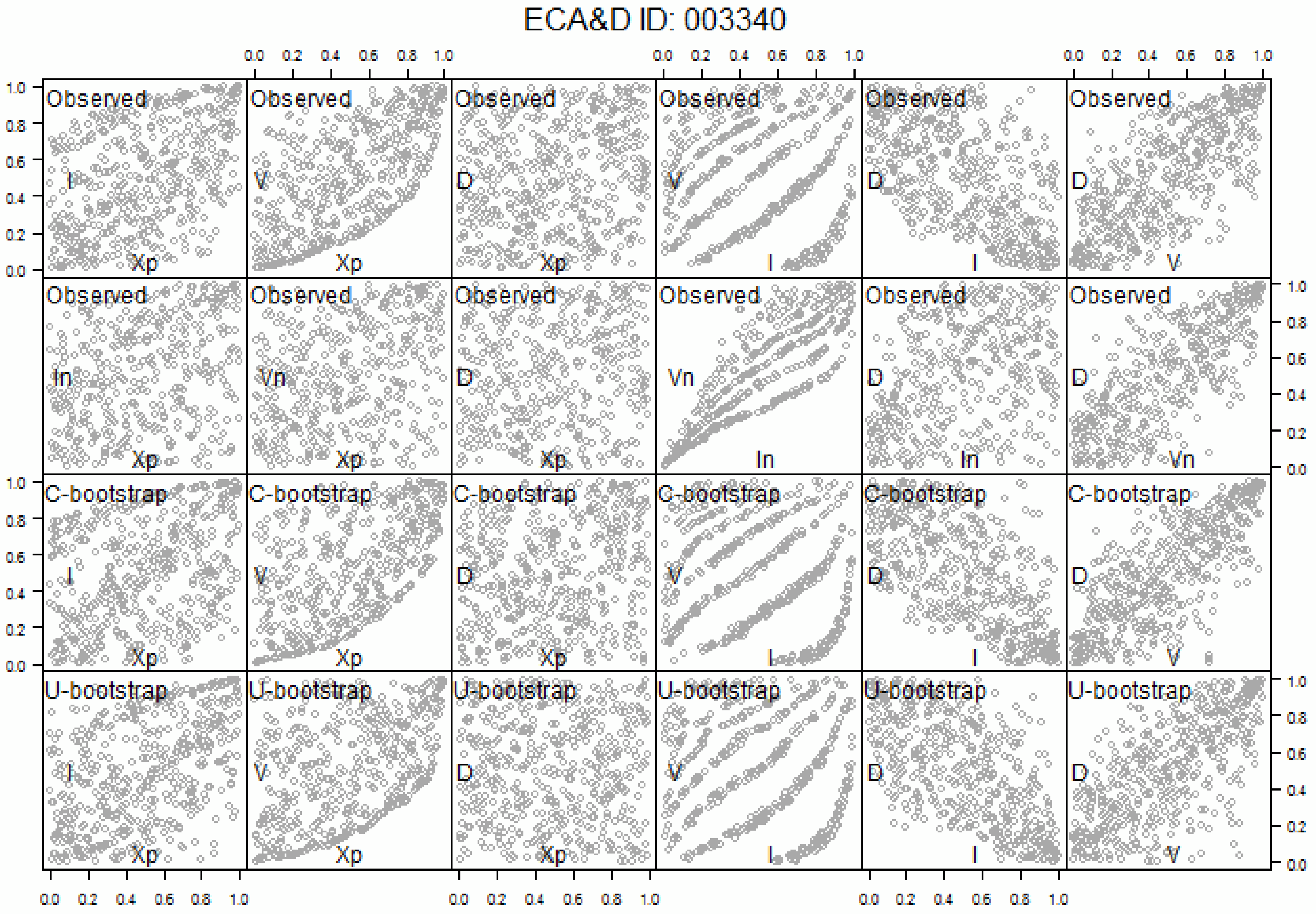
ECA&D ID: 003340
Observed
C-bootstrap
U-bootstrap
Xp
I
V
D
In
Vn
0.0
0.2
0.4
0.6
0.8
1.0

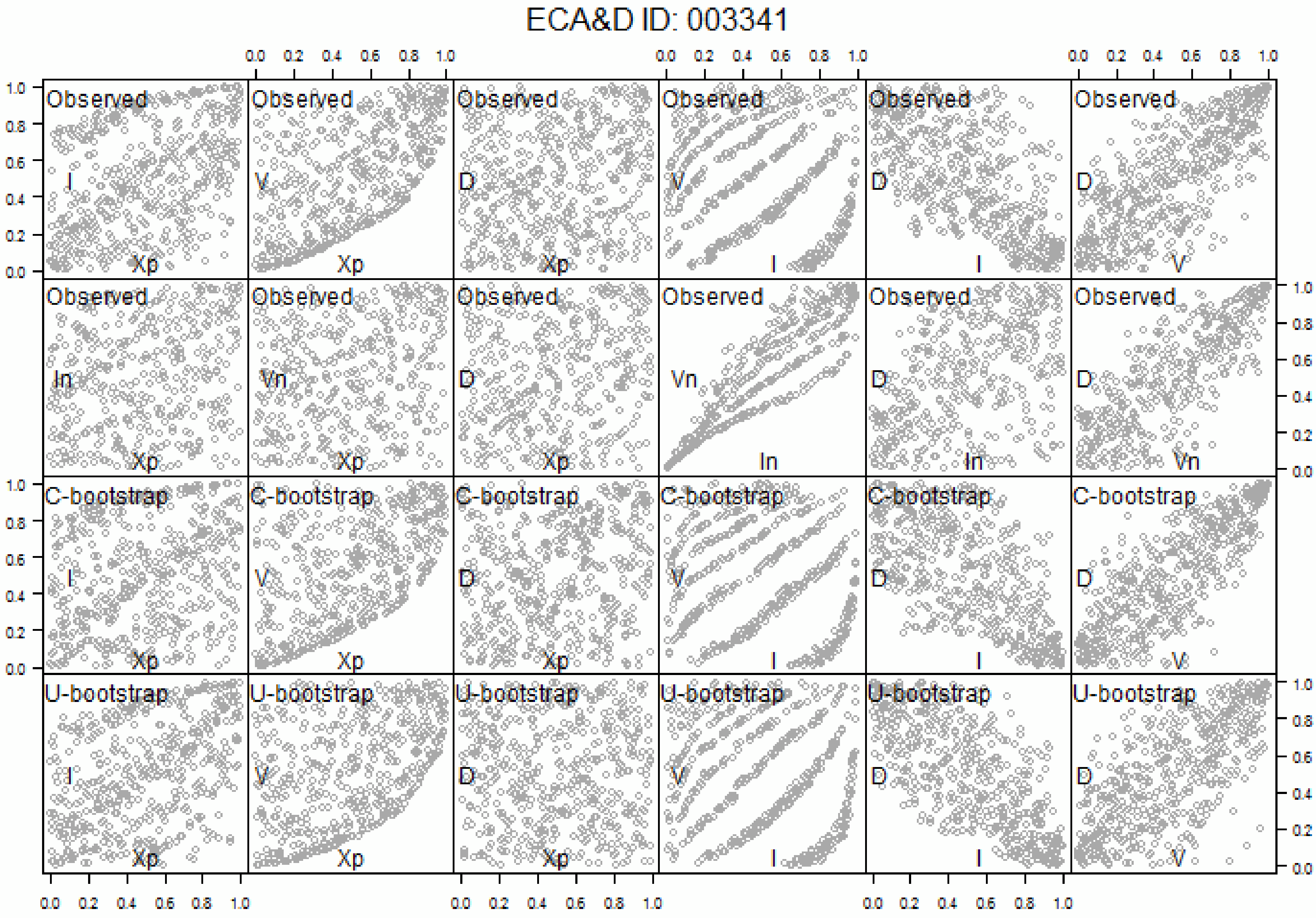
ECA&D ID: 003341
Observed
C-bootstrap
U-bootstrap
Xp
I
V
D
In
Vn
0.0
0.2
0.4
0.6
0.8
1.0

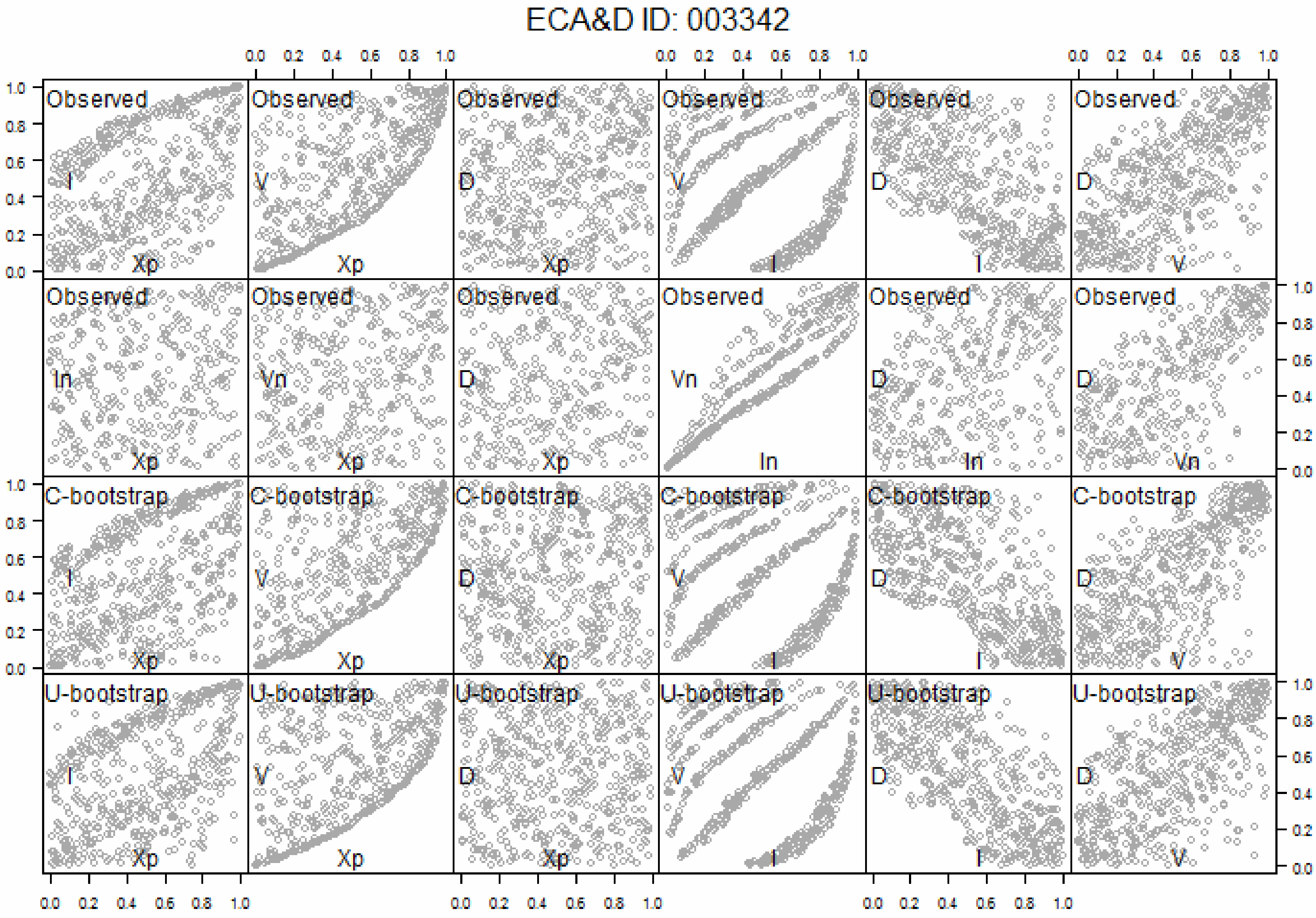
ECA&D ID: 003342
Observed
C-bootstrap
U-bootstrap
Xp
I
V
D
In
Vn
0.0
0.2
0.4
0.6
0.8
1.0

ECA&D ID: 003343

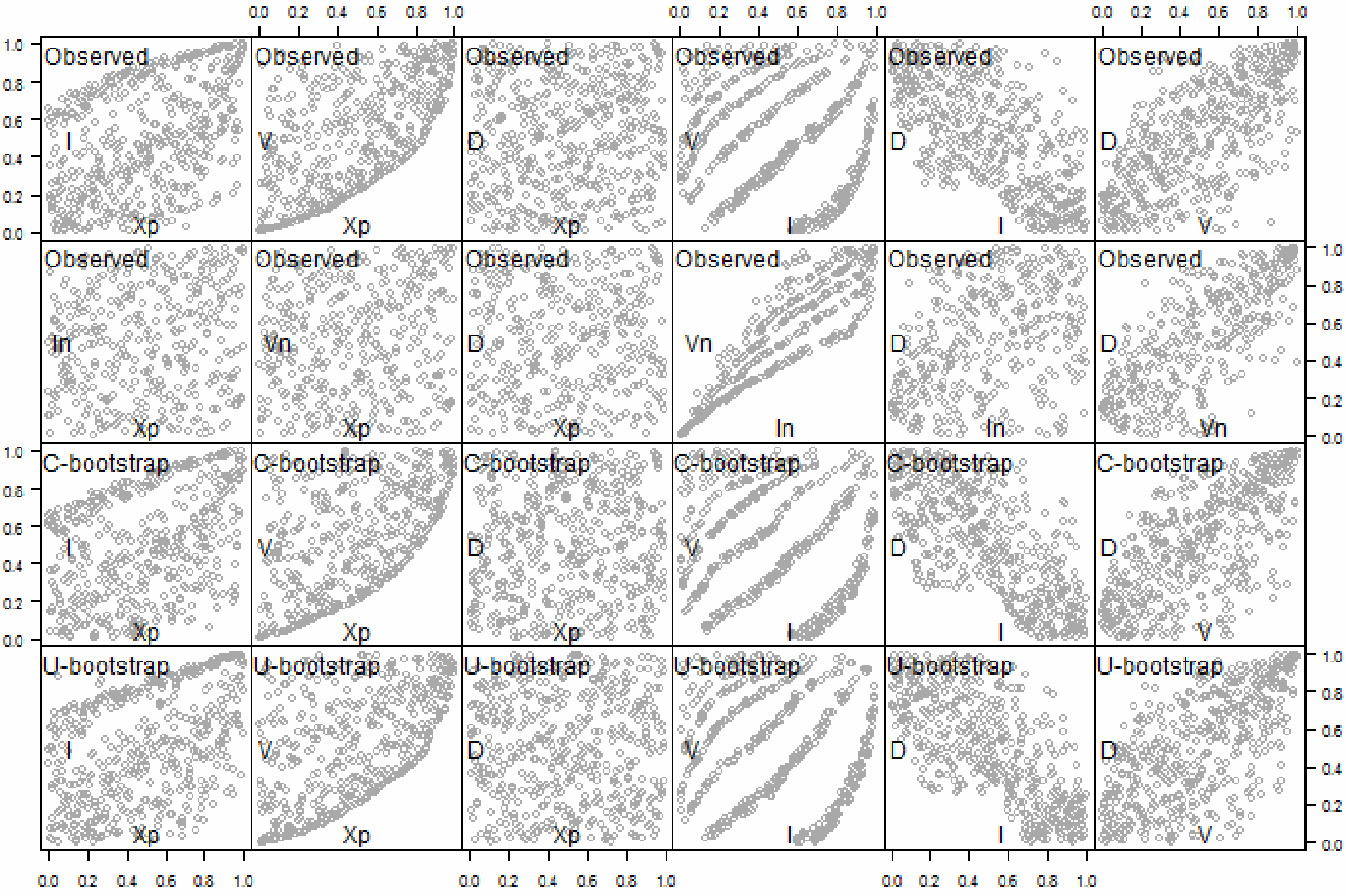

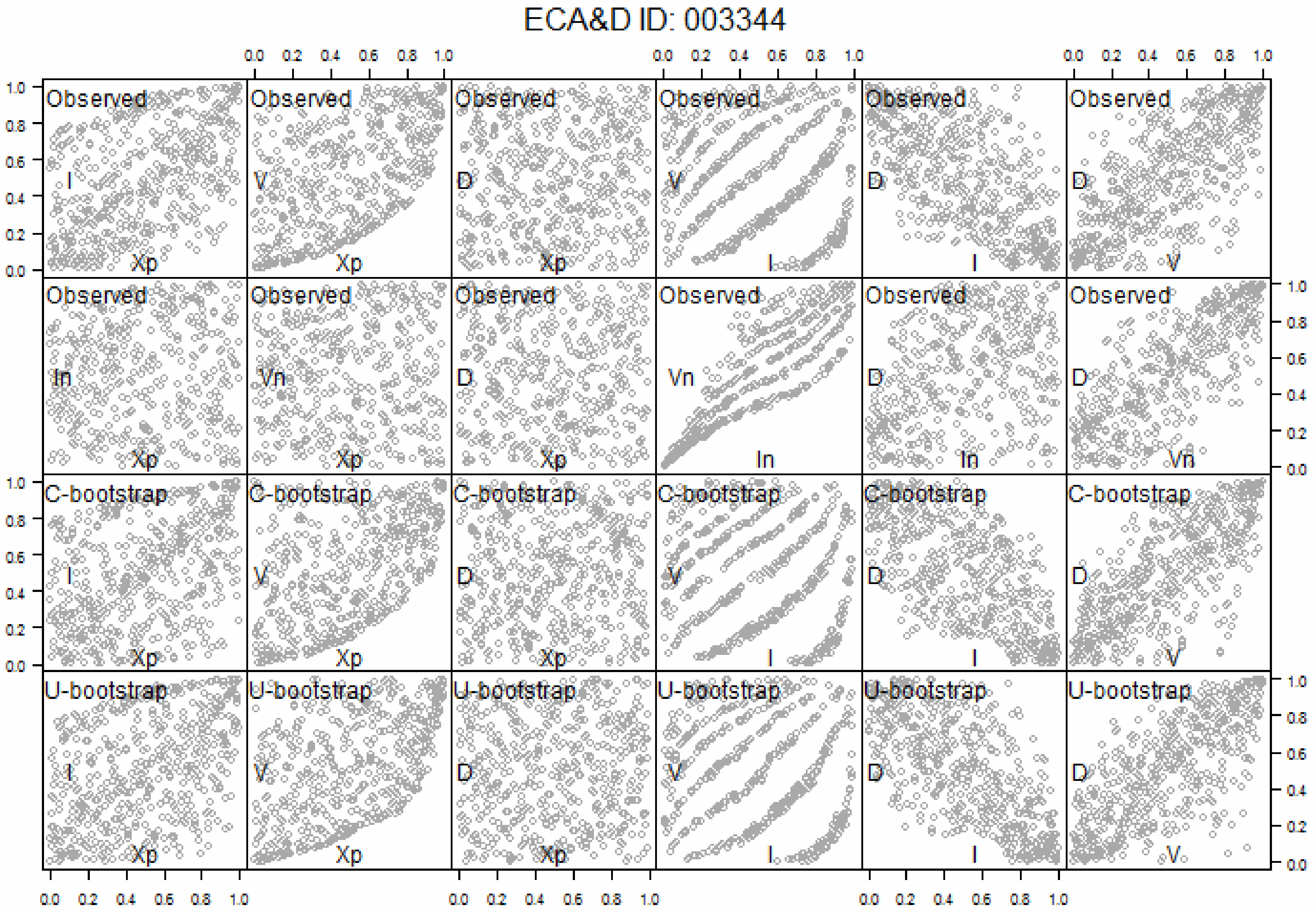
ECA&D ID: 003344
Observed
C-bootstrap
U-bootstrap
Xp
I
V
D
In
Vn
0.0
0.2
0.4
0.6
0.8
1.0

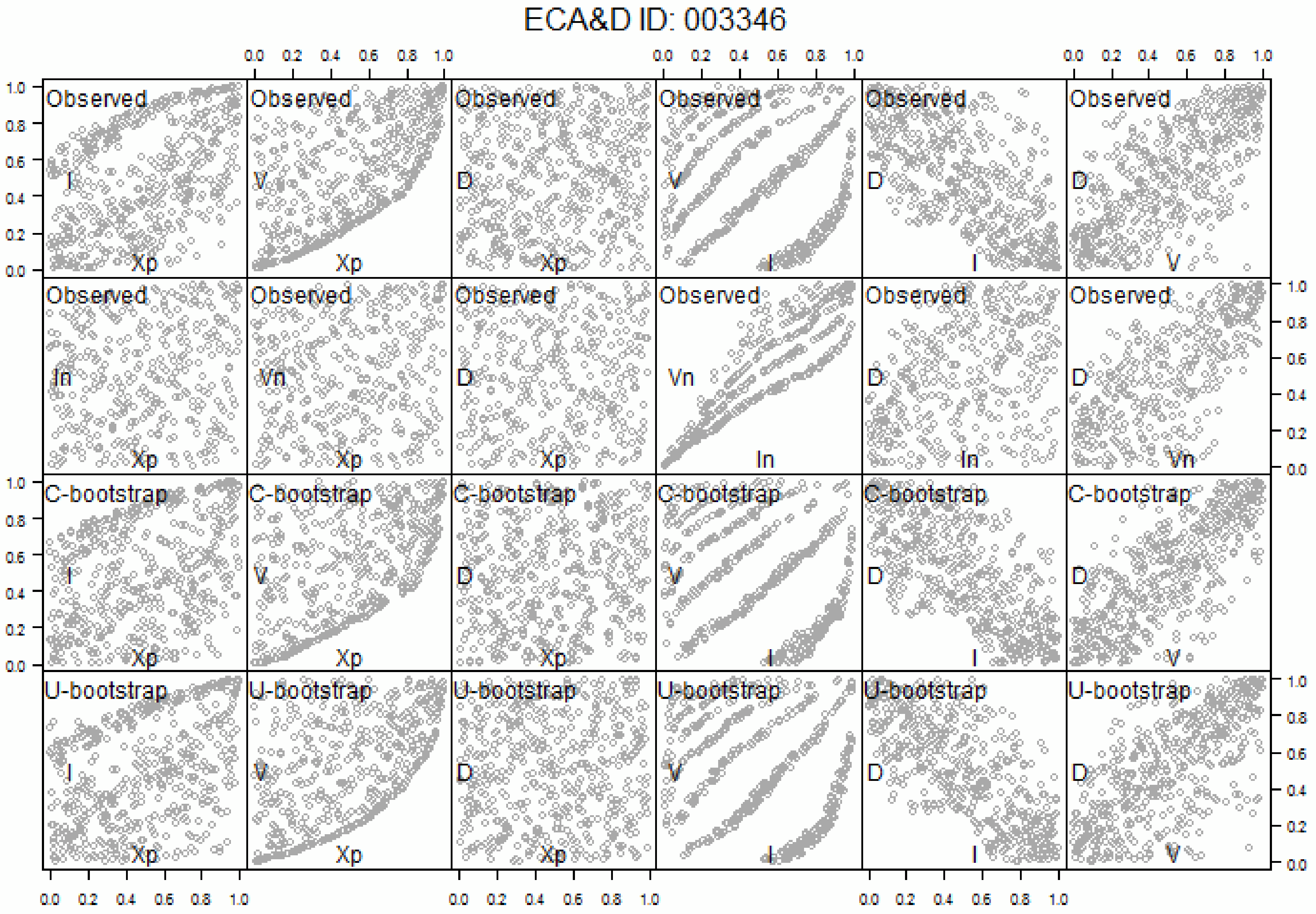
ECA&D ID: 003346
Observed
C-bootstrap
U-bootstrap
Xp
I
V
D
In
Vn
0.0
0.2
0.4
0.6
0.8
1.0

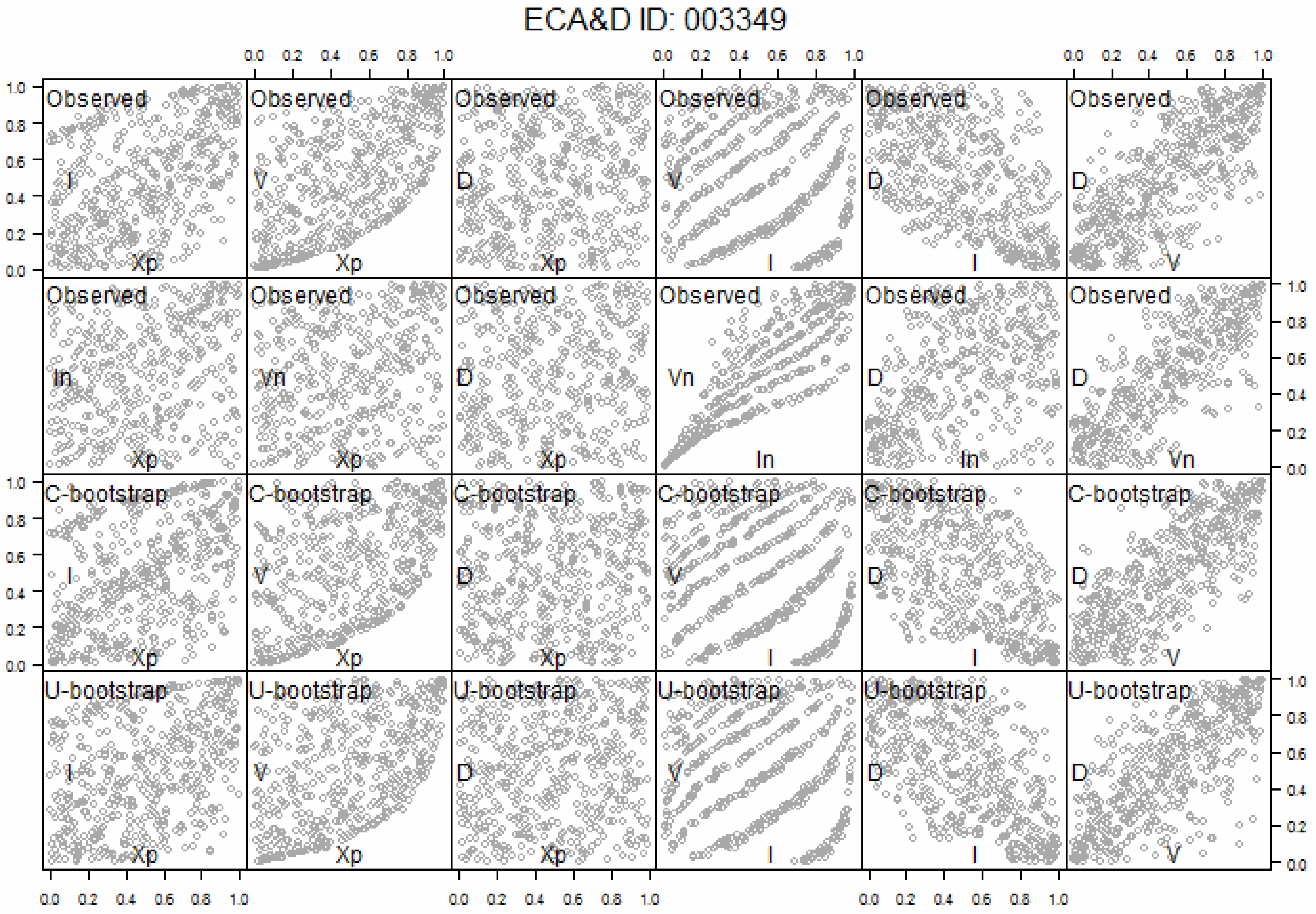
ECA&D ID: 003349
Observed
C-bootstrap
U-bootstrap
Xp
I
V
D
In
Vn
0.0
0.2
0.4
0.6
0.8
1.0

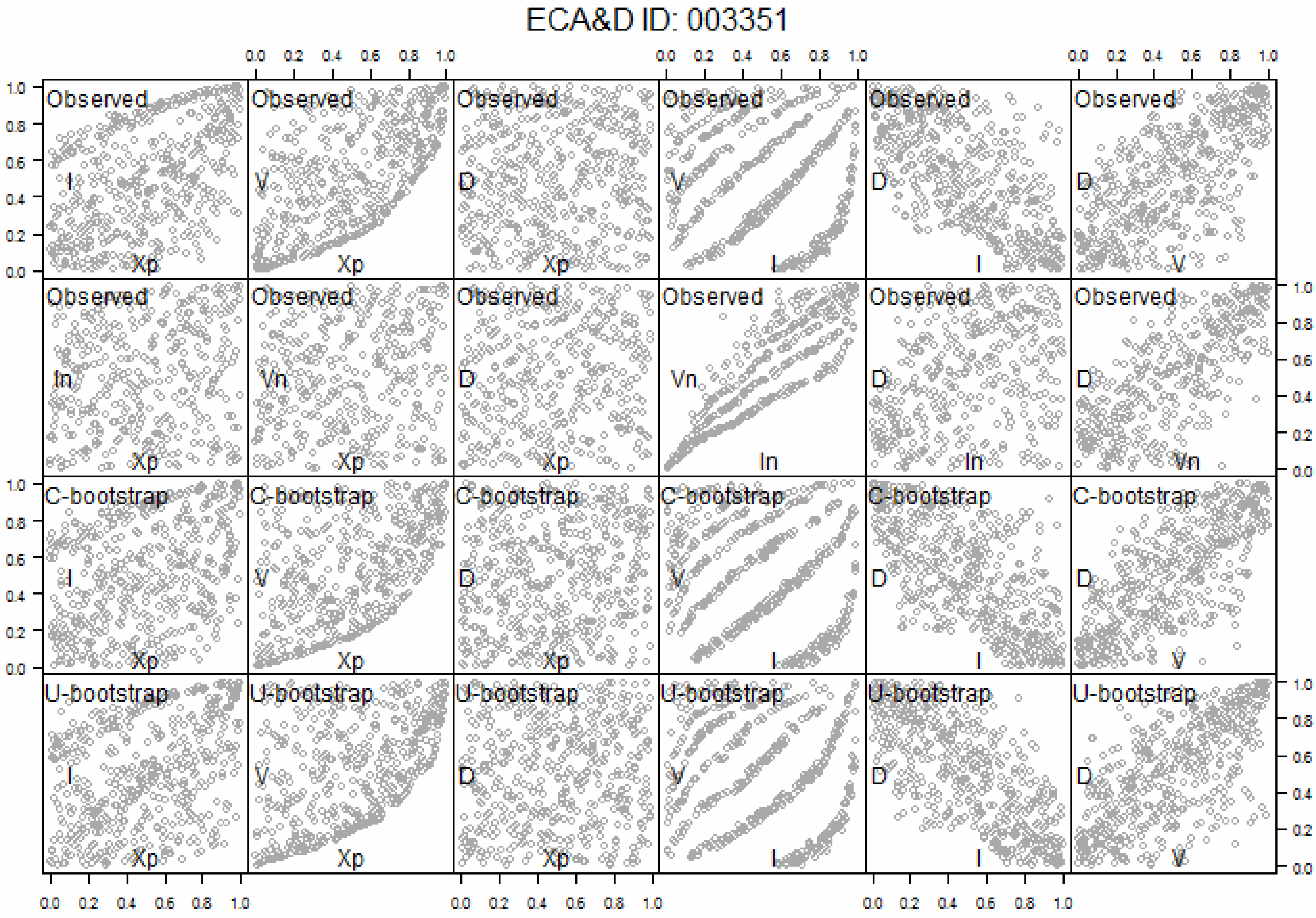
ECA&D ID: 003351
Observed
C-bootstrap
U-bootstrap
Xp
I
V
D
In
Vn
0.0
0.2
0.4
0.6
0.8
1.0

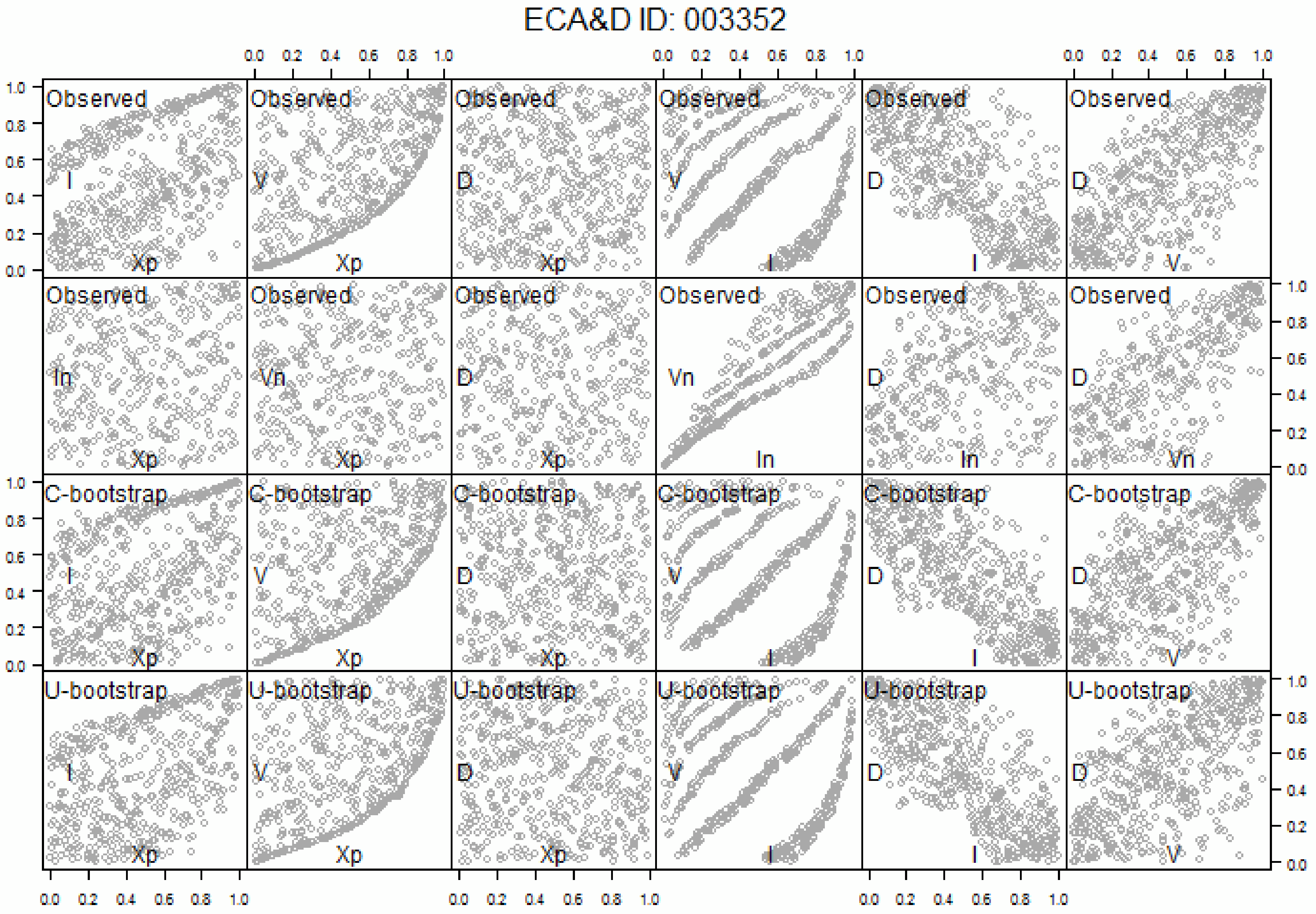
ECA&D ID: 003352
Observed
C-bootstrap
U-bootstrap
Xp
I
V
D
In
Vn
0.0
0.2
0.4
0.6
0.8
1.0

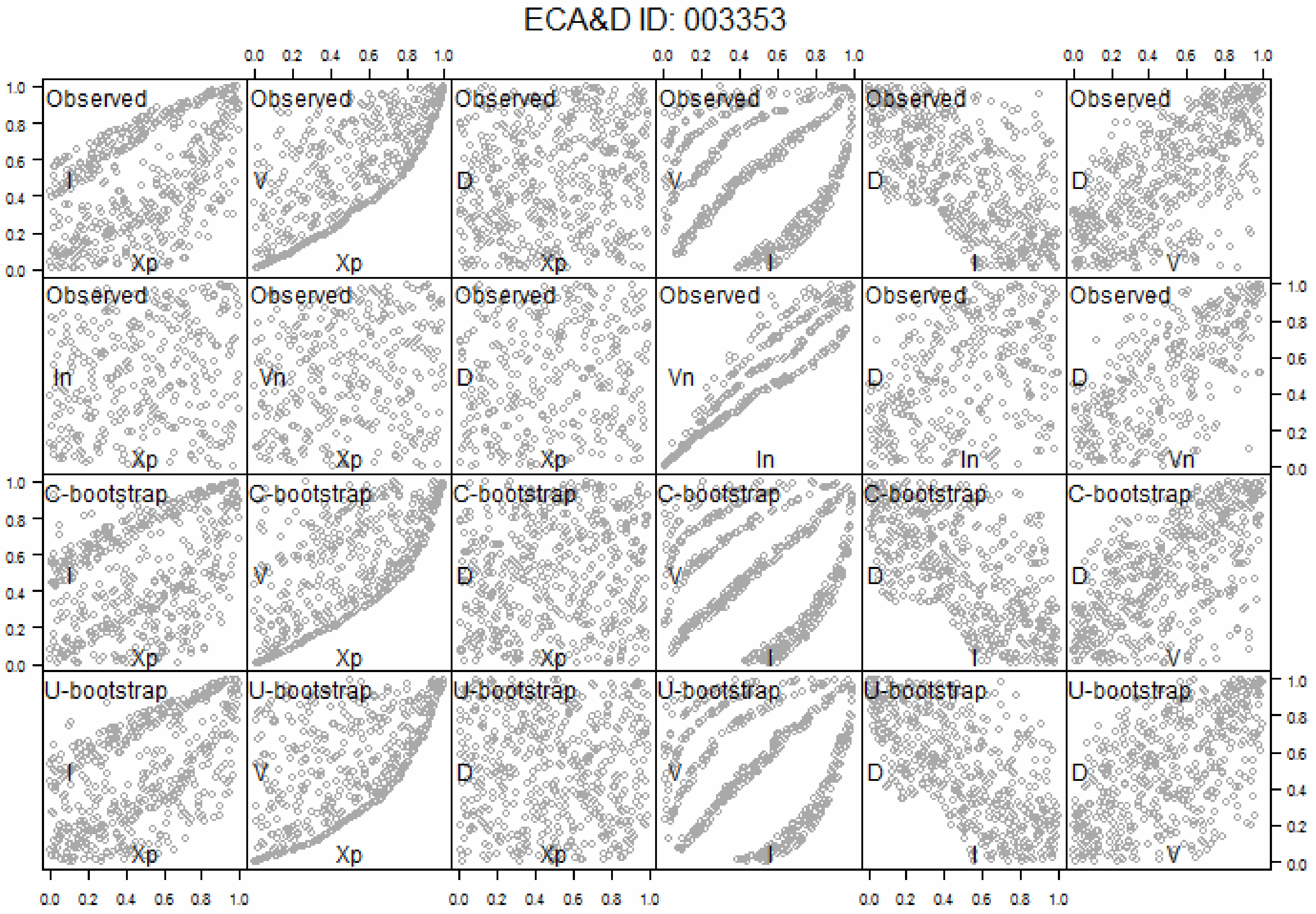
ECA&D ID: 003353
Observed
C-bootstrap
U-bootstrap
Xp
I
V
D
In
Vn
0.0
0.2
0.4
0.6
0.8
1.0

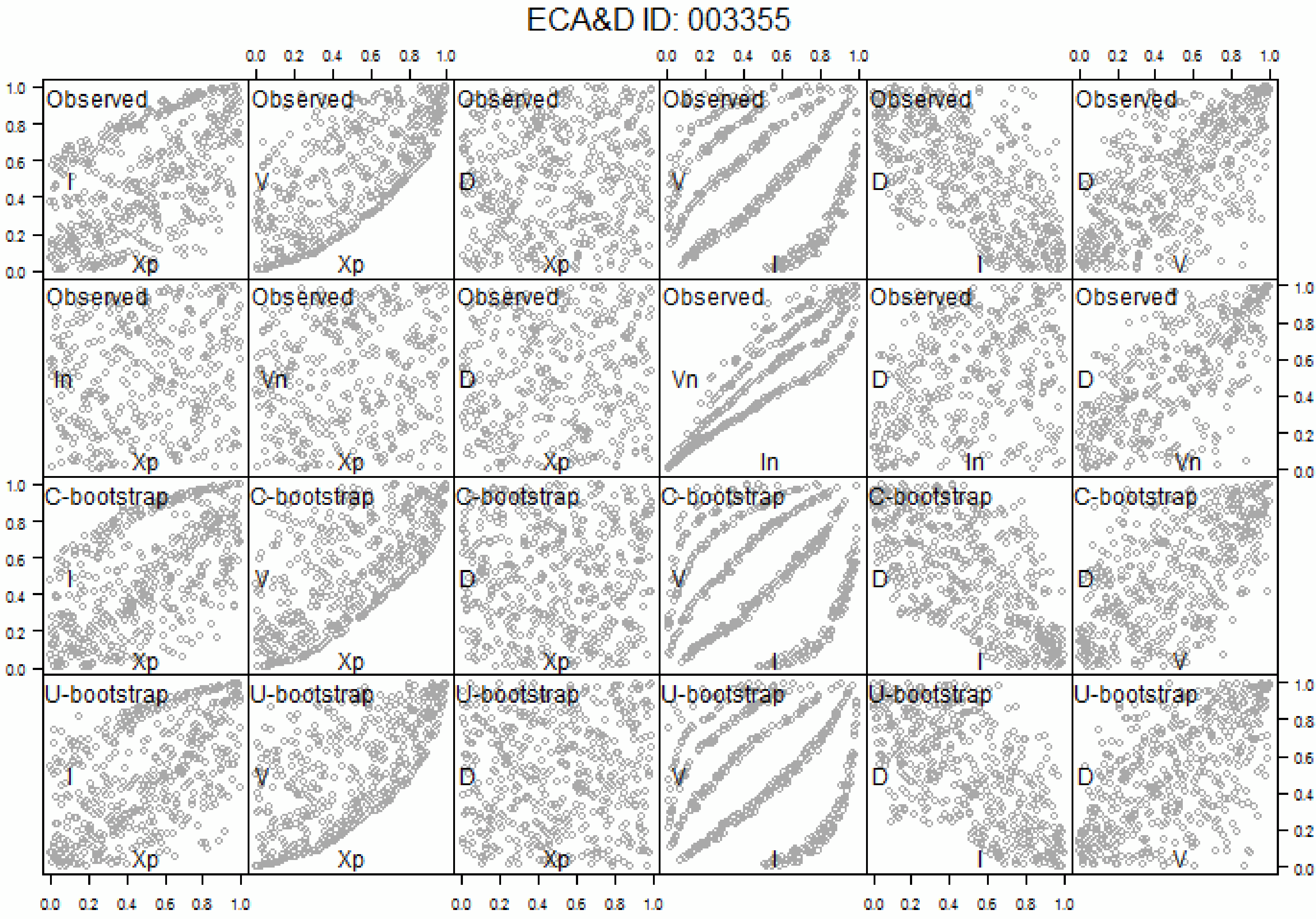

ECA&D ID: 003355
Observed
C-bootstrap
U-bootstrap
Xp
I
V
D
In
Vn
0.0
0.2
0.4
0.6
0.8
1.0

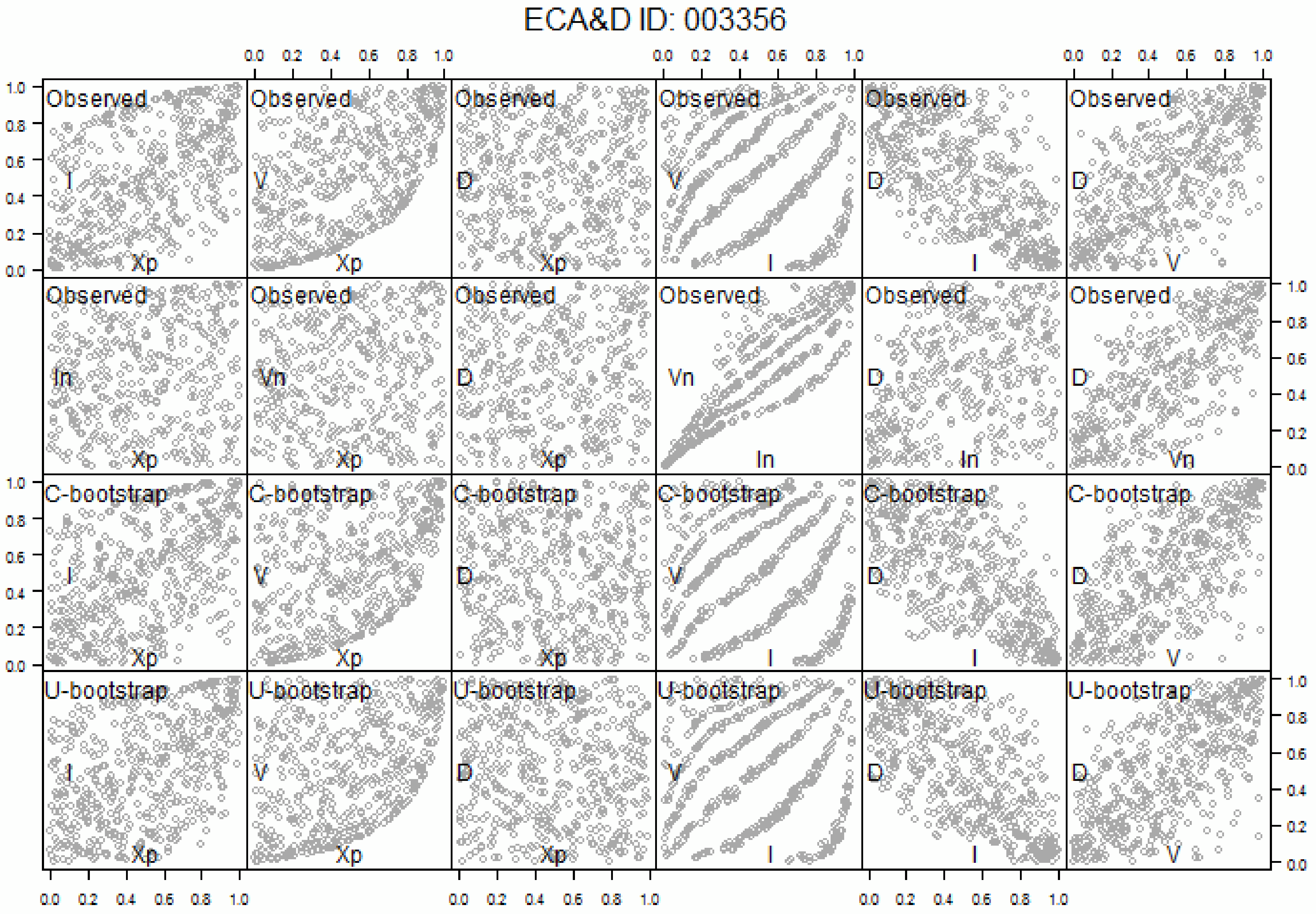
ECA&D ID: 003356
Observed
C-bootstrap
U-bootstrap
Xp
I
V
D
In
Vn
0.0
0.2
0.4
0.6
0.8
1.0

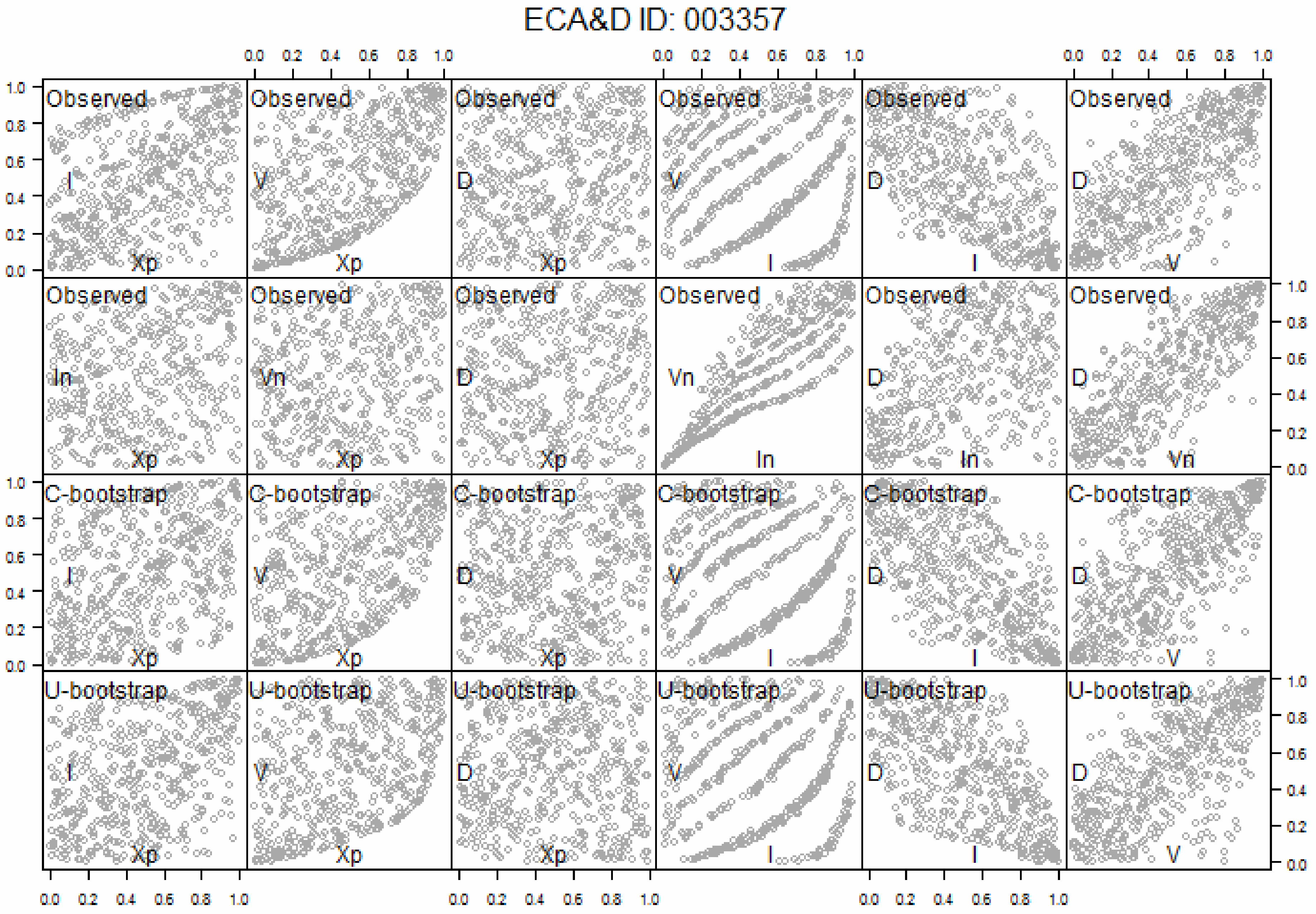
ECA&D ID: 003357
Observed
C-bootstrap
U-bootstrap
Xp
I
V
D
In
Vn
0.0
0.2
0.4
0.6
0.8
1.0

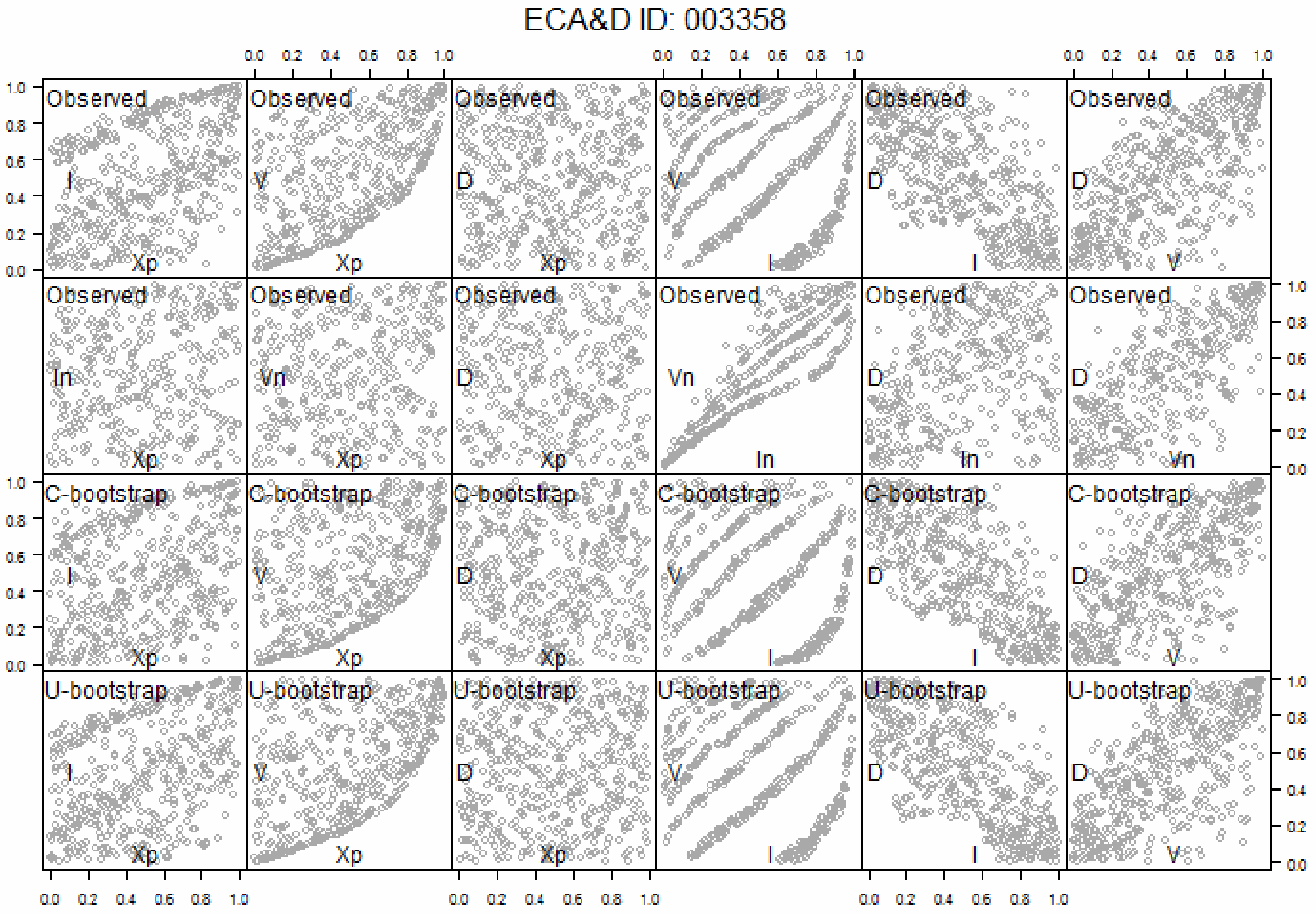
ECA&D ID: 003358
Observed
C-bootstrap
U-bootstrap
Xp
I
V
D
In
Vn
0.0
0.2
0.4
0.6
0.8
1.0

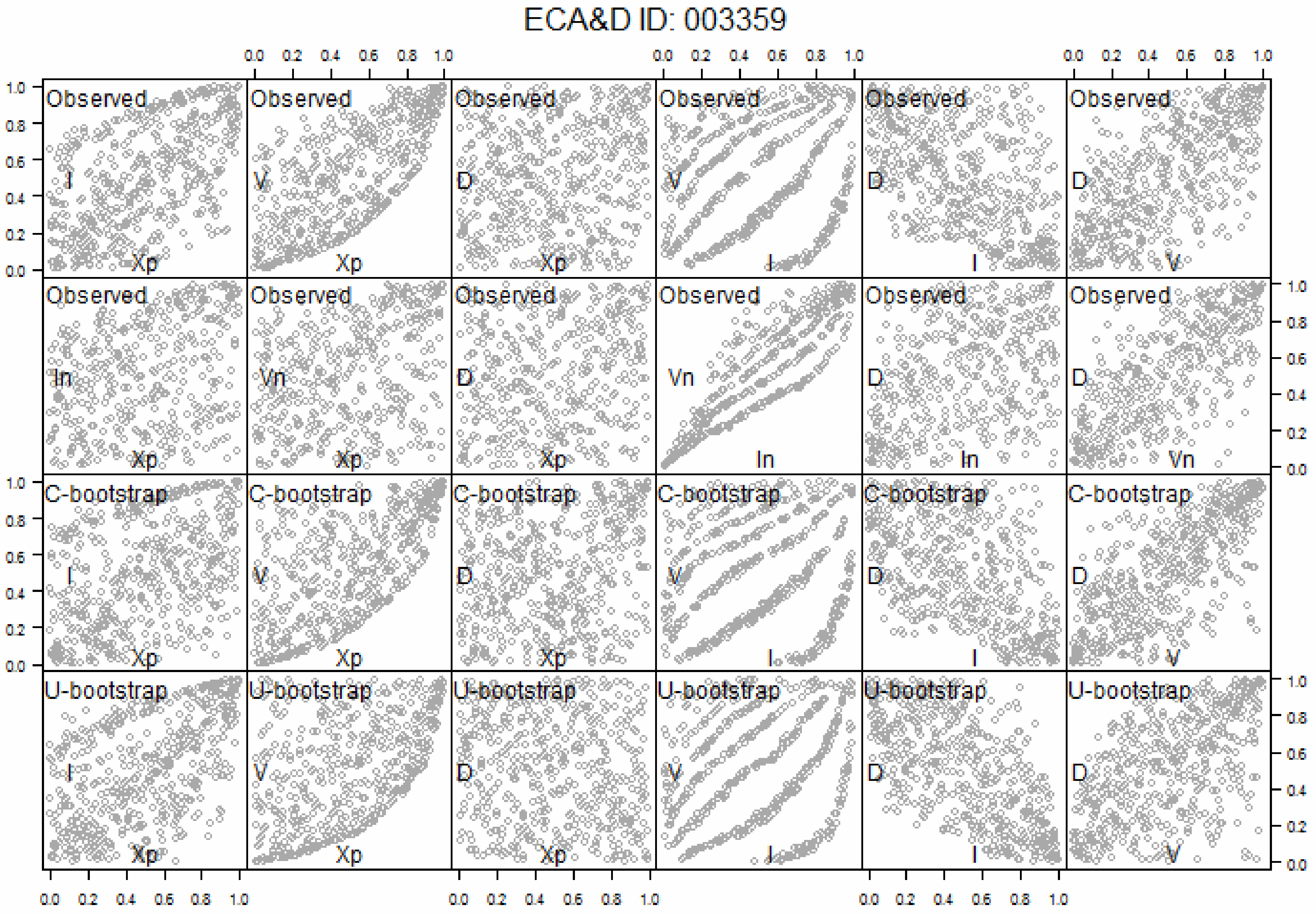

ECA&D ID: 003359
Observed
C-bootstrap
U-bootstrap
Xp
I
V
D
In
Vn
0.0
0.2
0.4
0.6
0.8
1.0

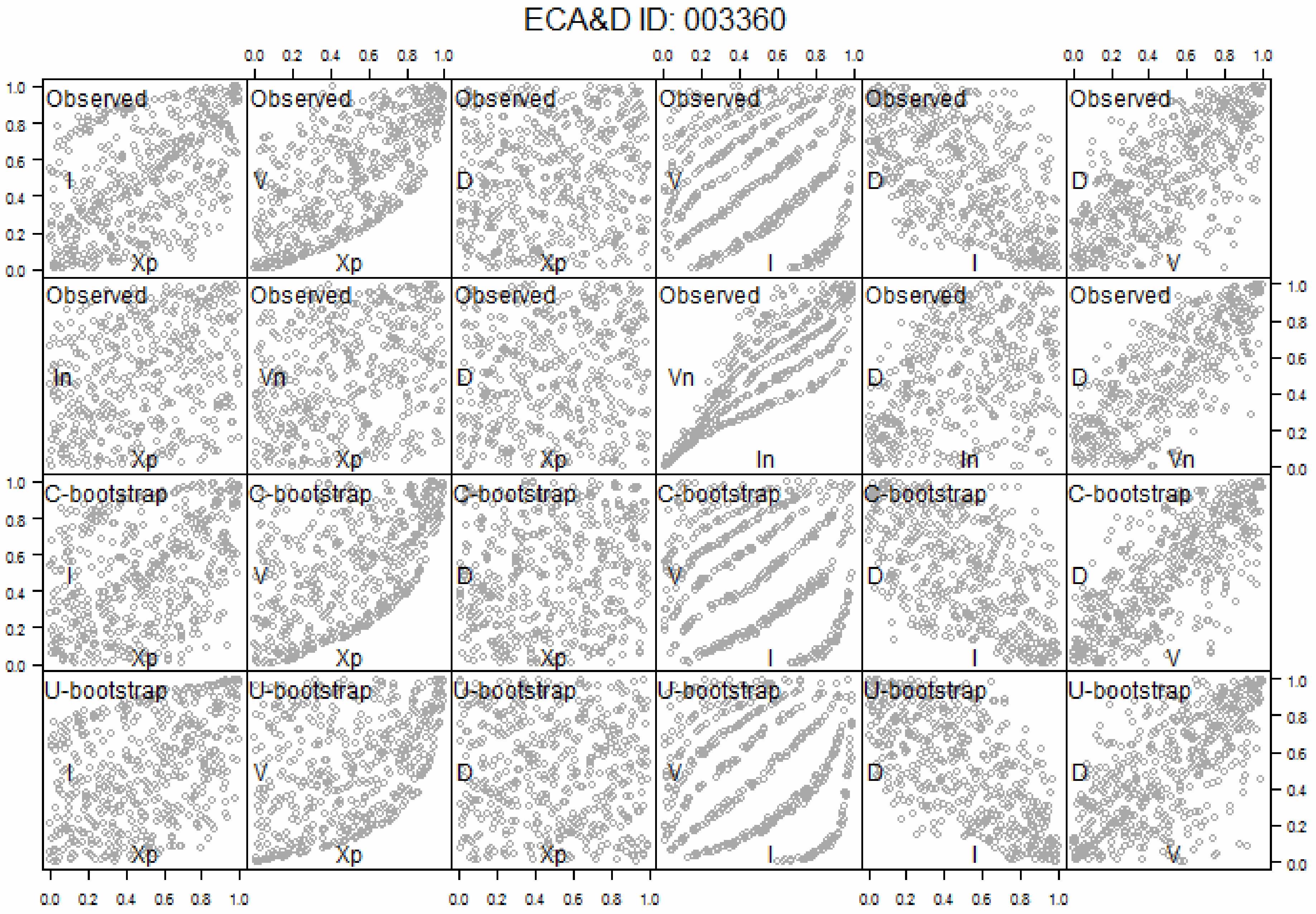

ECA&D ID: 003360
Observed
C-bootstrap
U-bootstrap
Xp
I
V
D
In
Vn
0.0
0.2
0.4
0.6
0.8
1.0

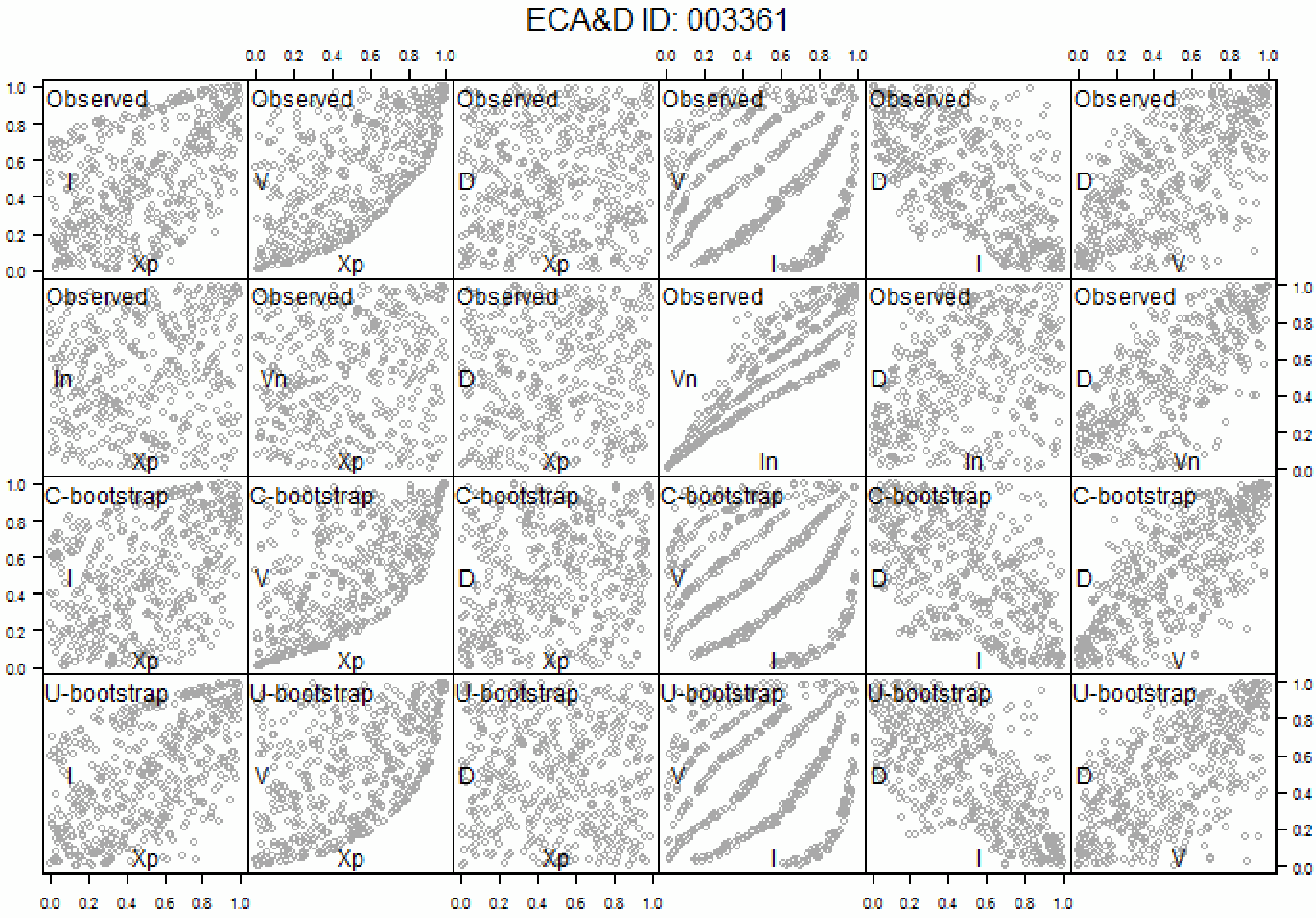
ECA&D ID: 003361
Observed
C-bootstrap
U-bootstrap
Xp
I
V
D
In
Vn
0.0
0.2
0.4
0.6
0.8
1.0

ECA&D ID: 003362

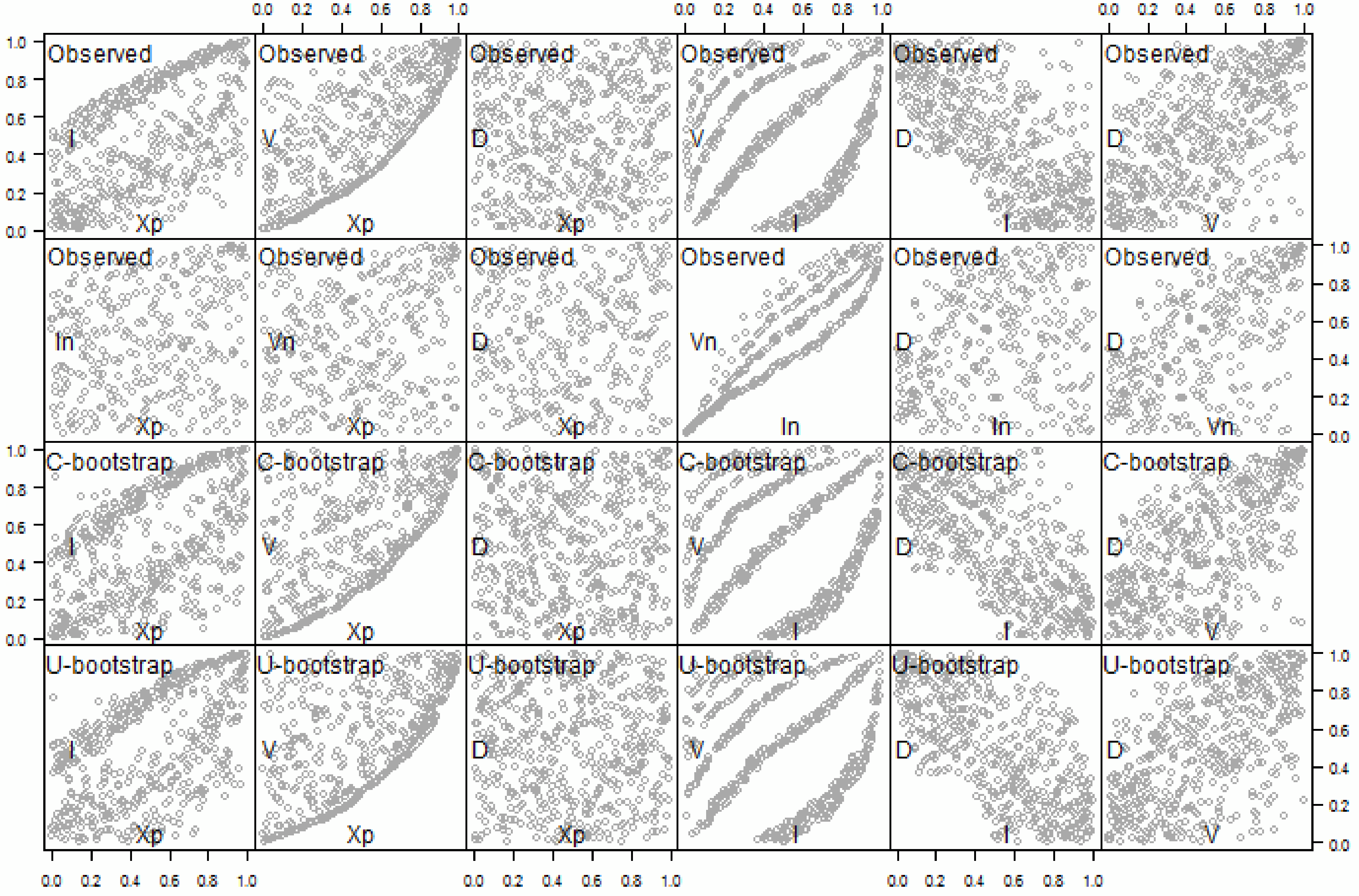

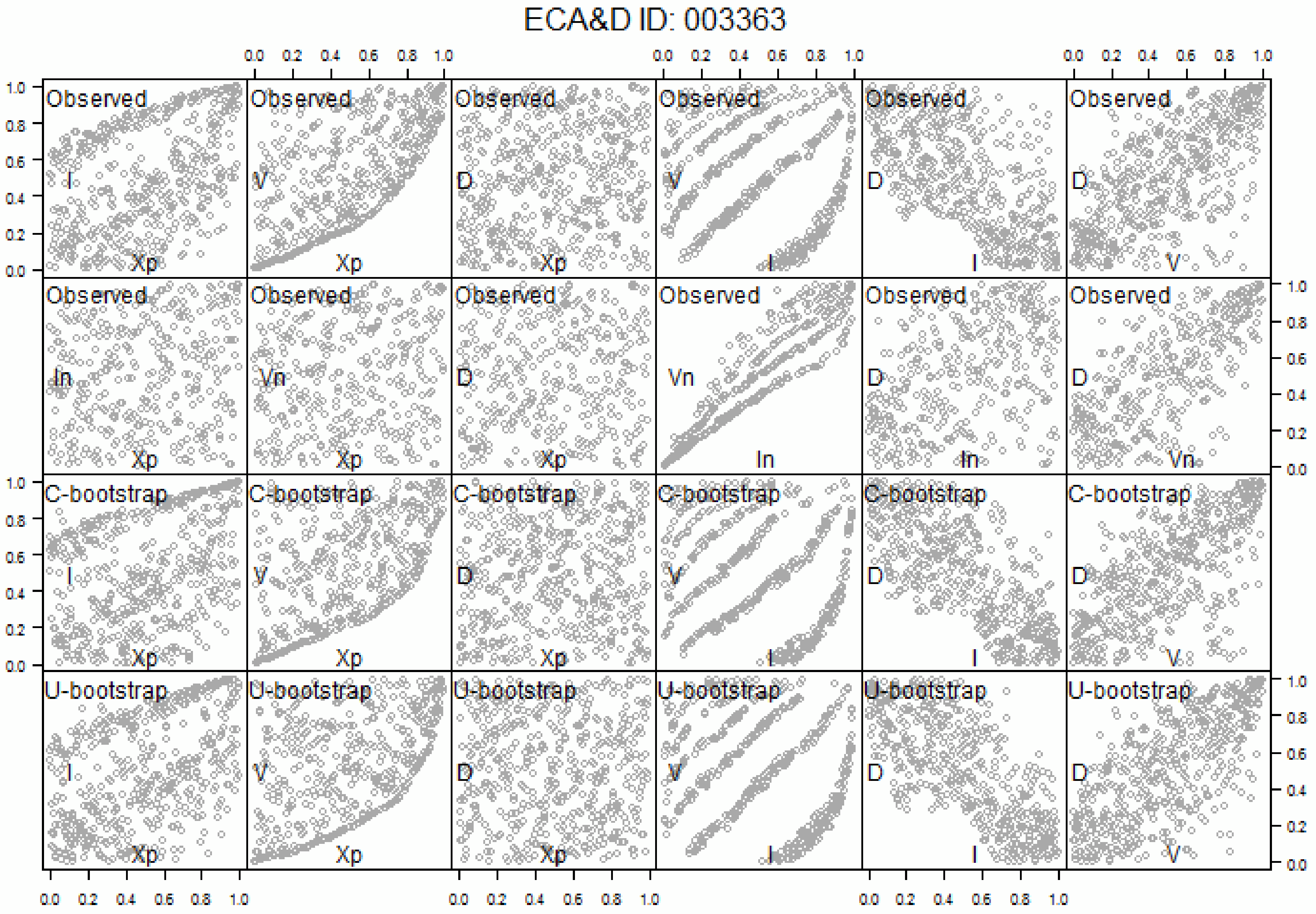
ECA&D ID: 003363
Observed
C-bootstrap
U-bootstrap
Xp
I
V
D
In
Vn
0.0
0.2
0.4
0.6
0.8
1.0

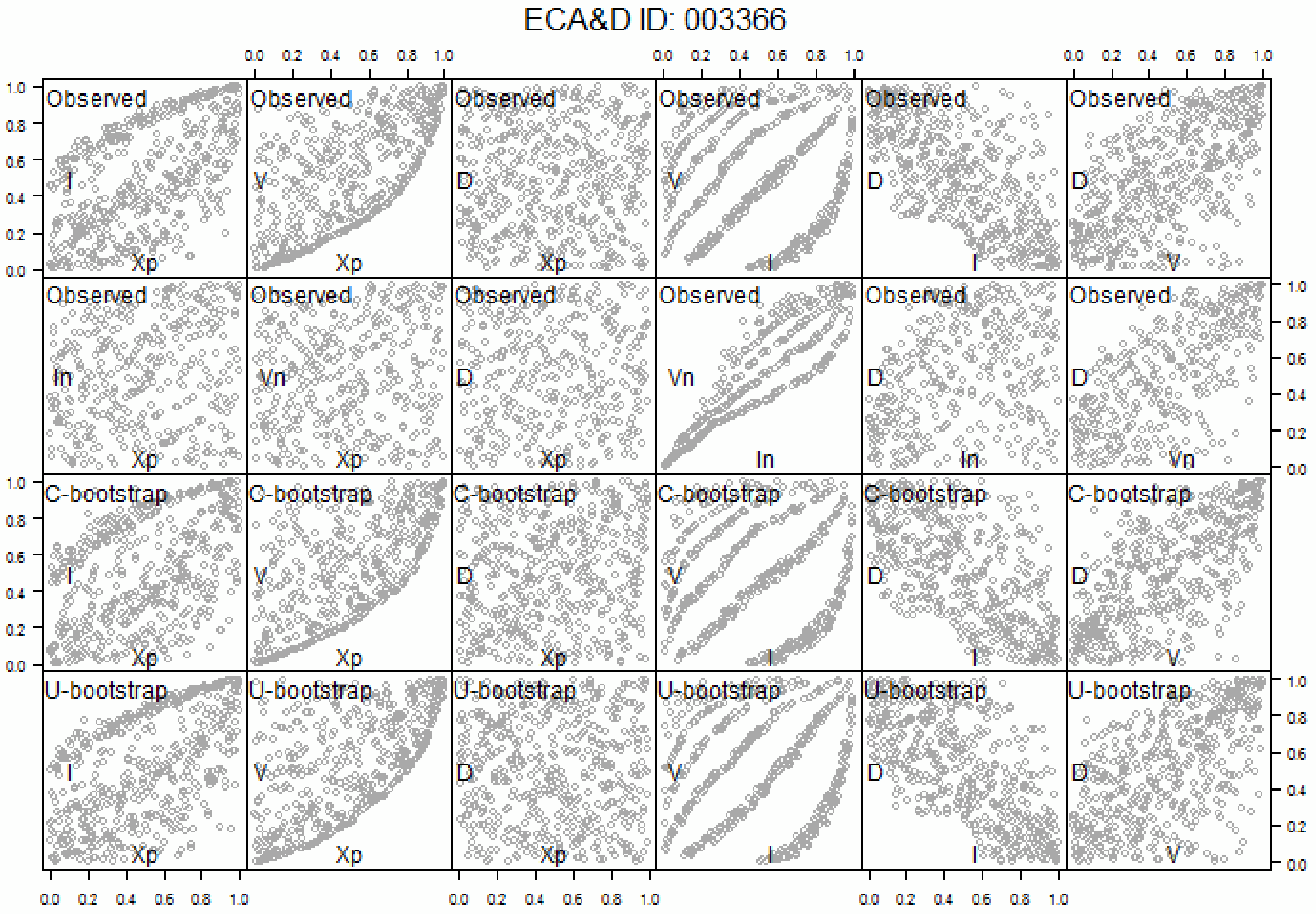

ECA&D ID: 003366
Observed
C-bootstrap
U-bootstrap
I
V
D
In
Vn
Xp
0.0
0.2
0.4
0.6
0.8
1.0

ECA&D ID: 003369

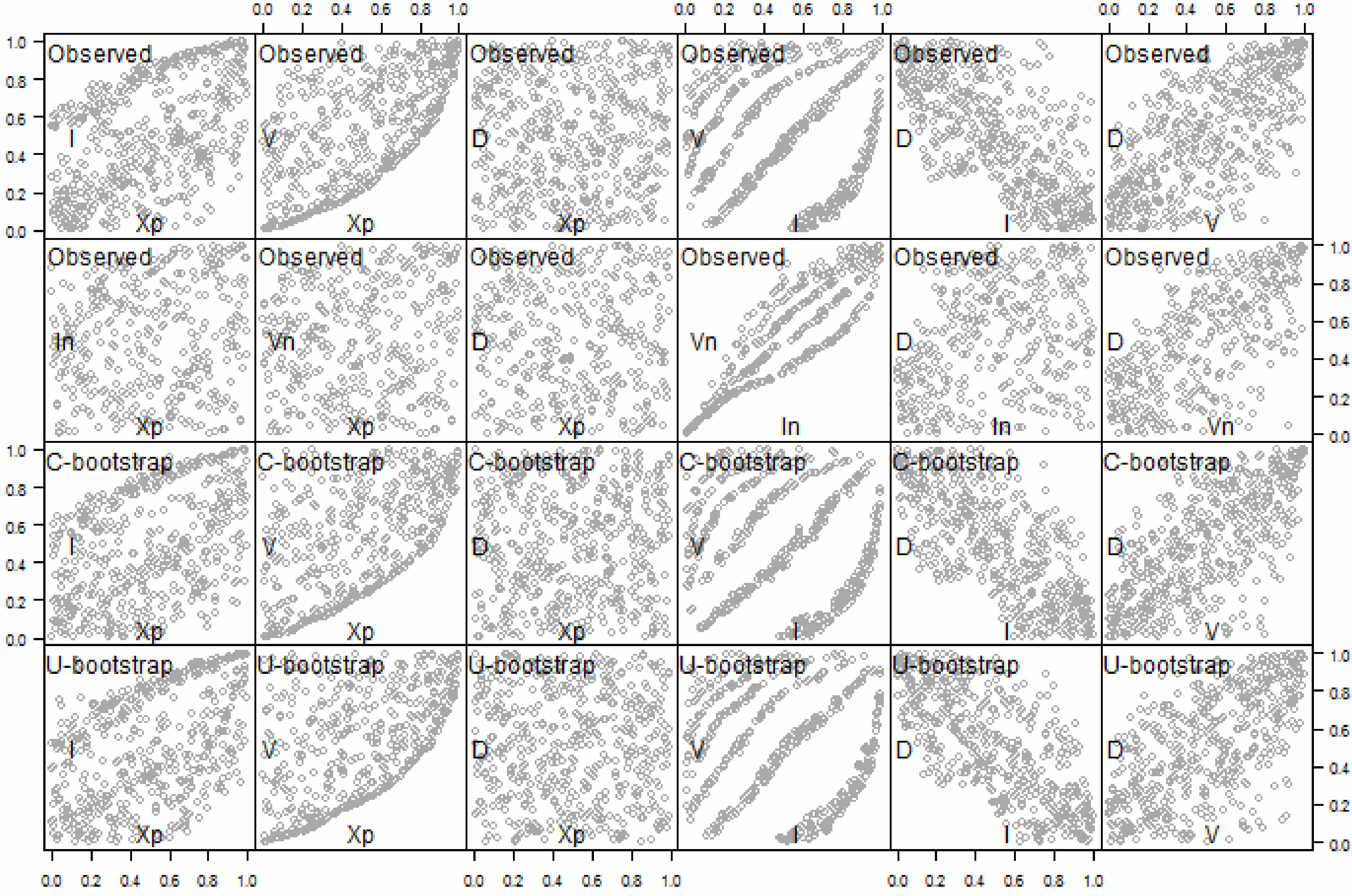

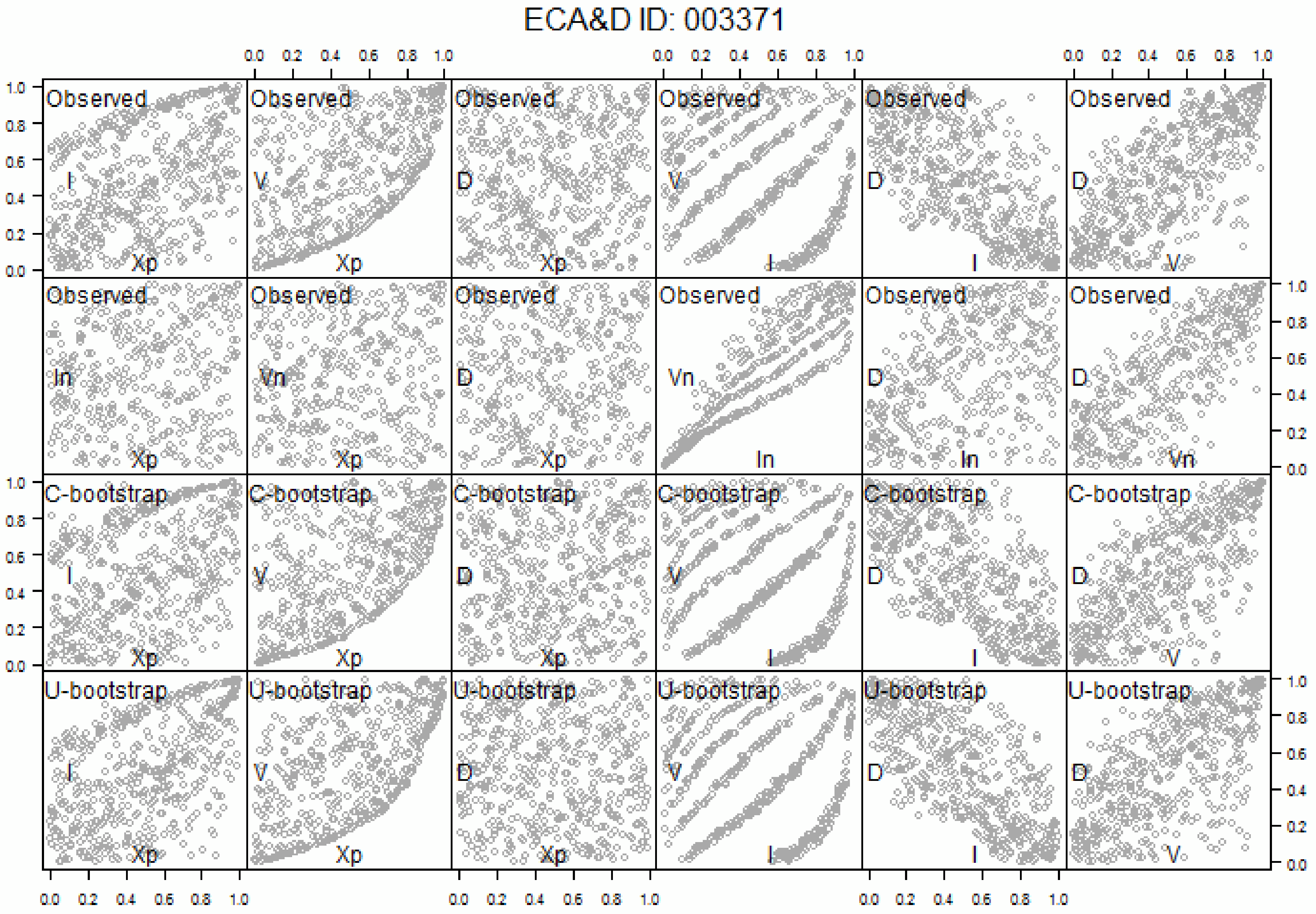
ECA&D ID: 003371
Observed
Xp
I
V
D
In
Vn
C-bootstrap
U-bootstrap
0.0
0.2
0.4
0.6
0.8
1.0

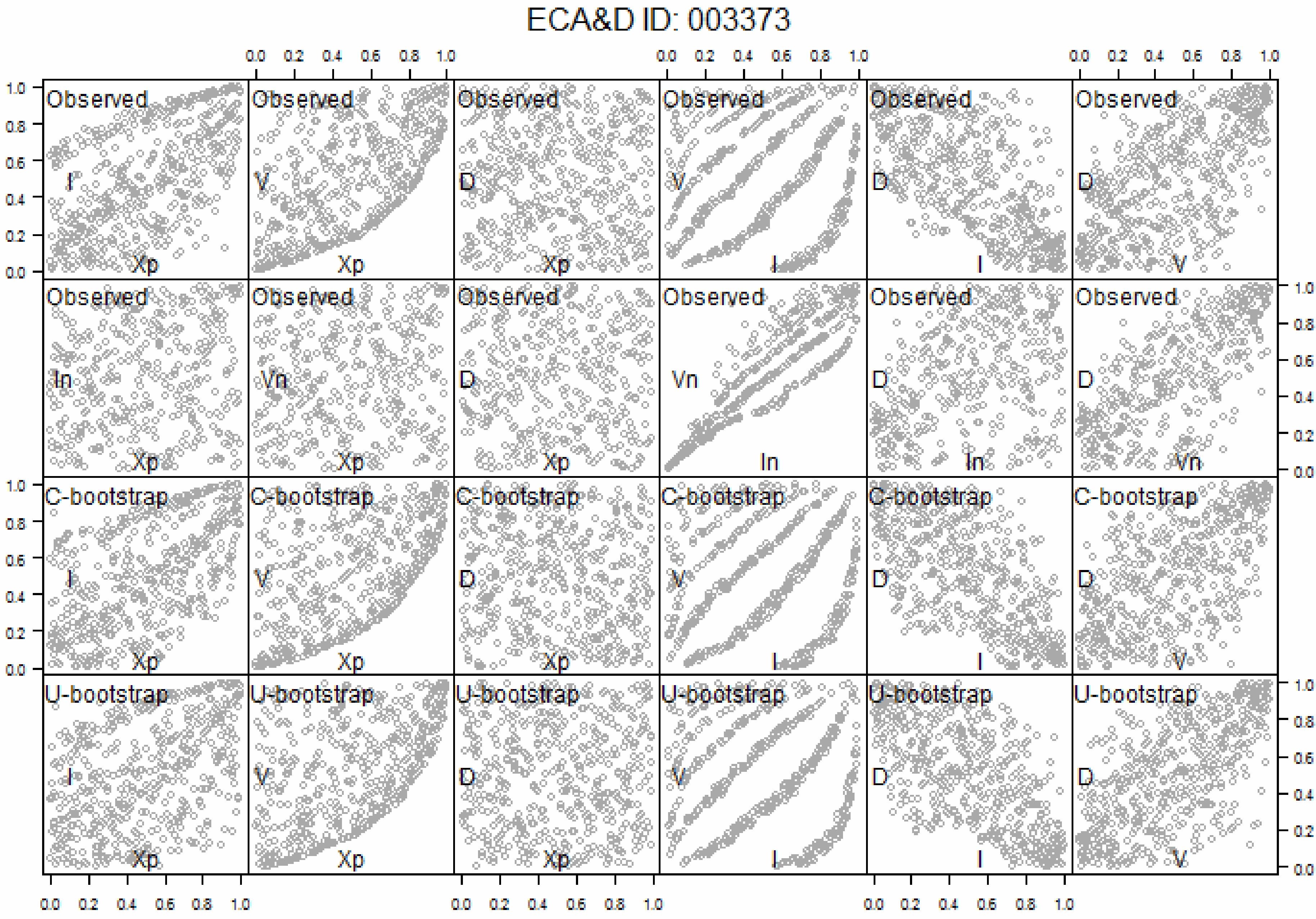
ECA&D ID: 003373
Observed
C-bootstrap
U-bootstrap
Xp
I
V
D
In
Vn
0.0
0.2
0.4
0.6
0.8
1.0

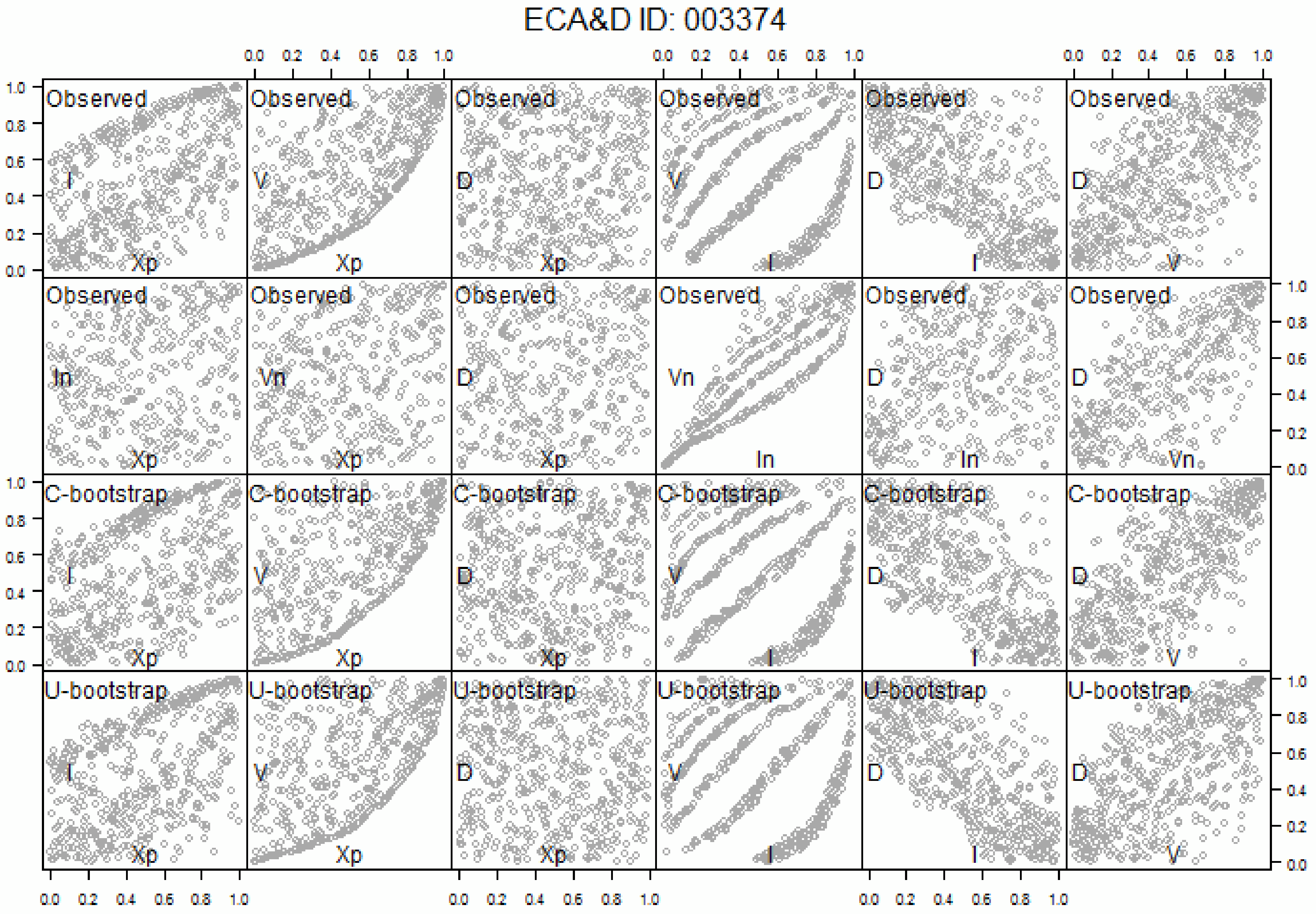
ECA&D ID: 003374
Observed
C-bootstrap
U-bootstrap
Xp
I
V
D
In
Vn
0.0
0.2
0.4
0.6
0.8
1.0

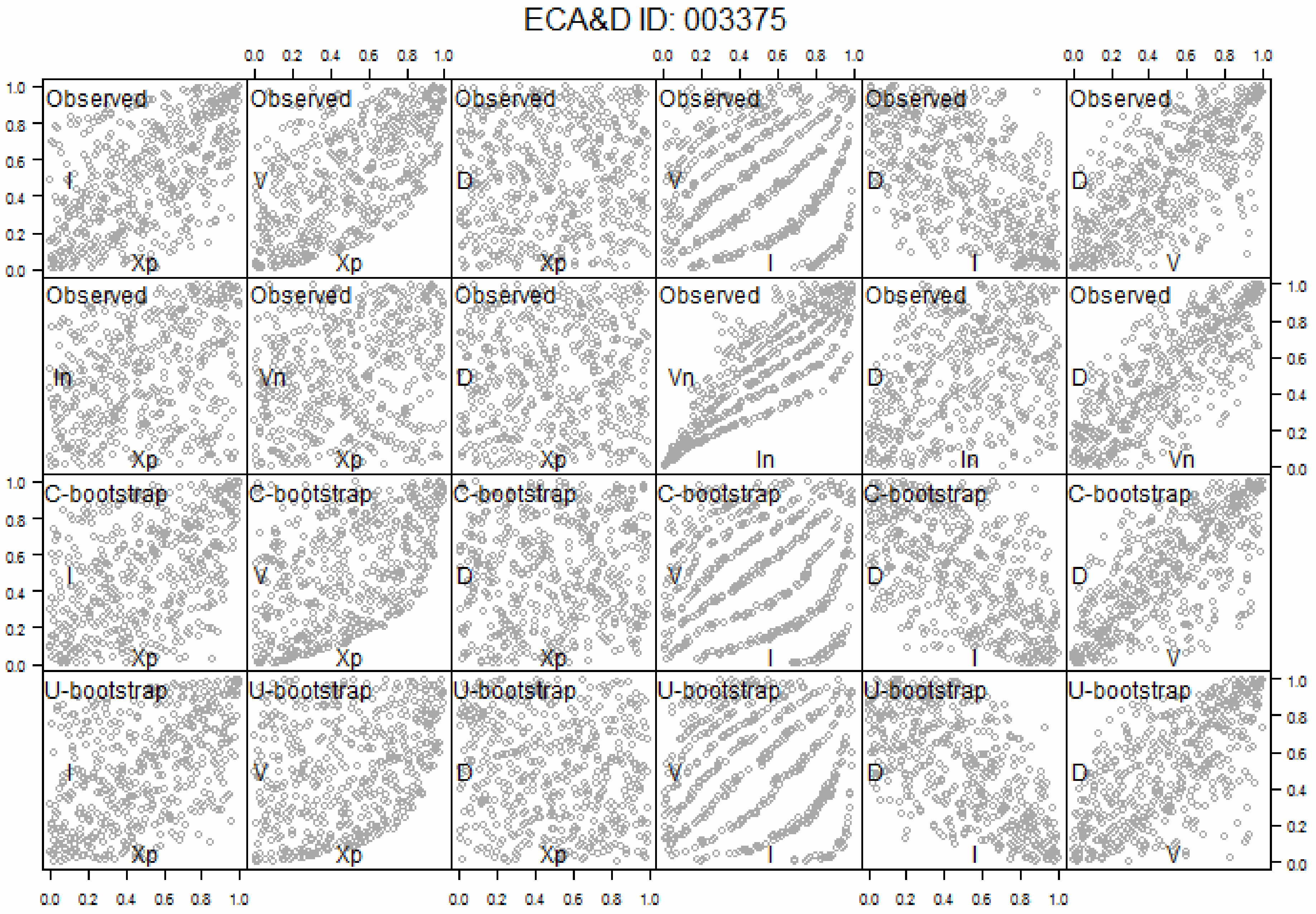
ECA&D ID: 003375
Observed
C-bootstrap
U-bootstrap
Xp
I
V
D
In
Vn
0.0
0.2
0.4
0.6
0.8
1.0

ECA&D ID: 003377

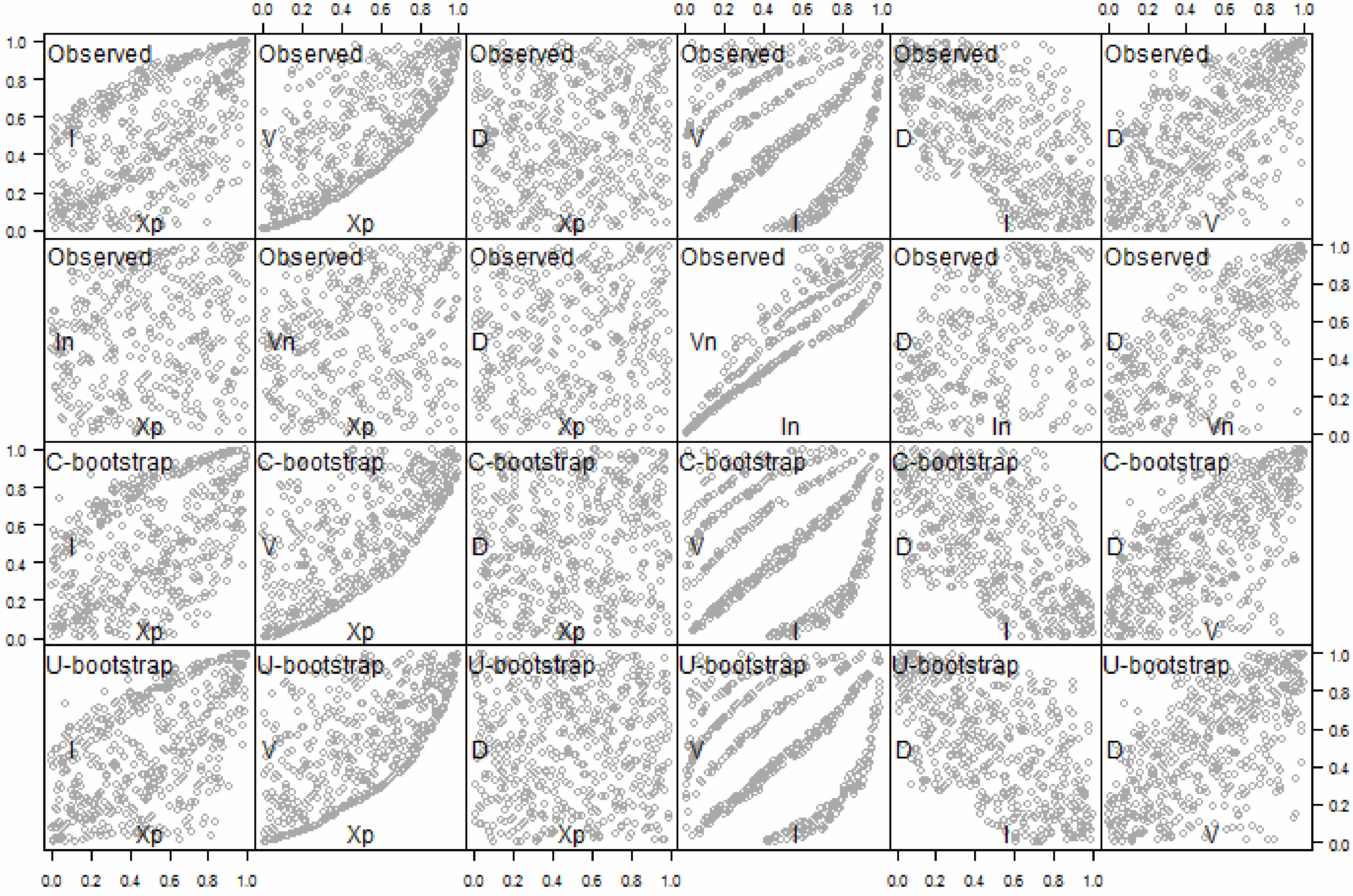

ECA&D ID: 003378

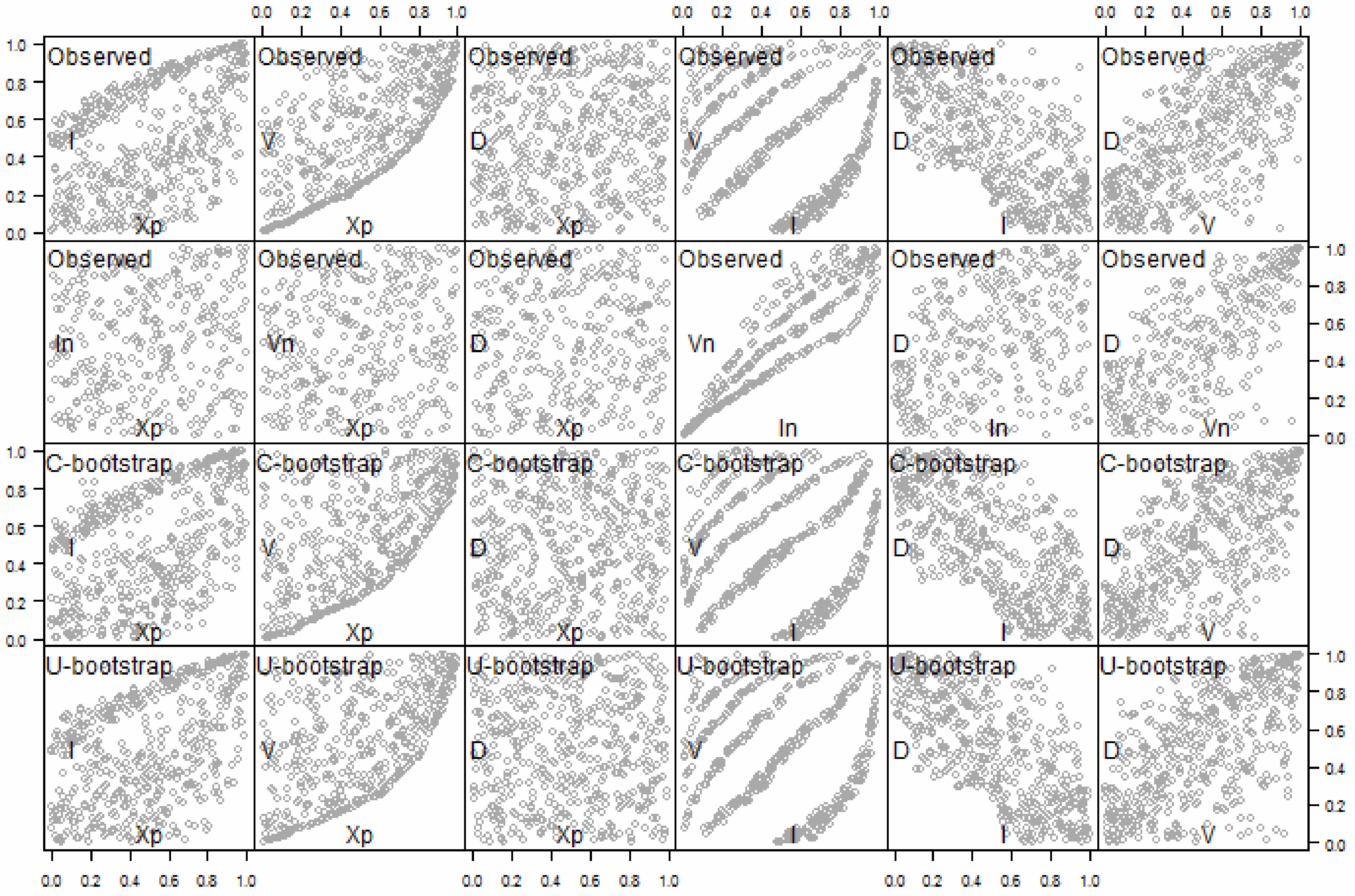

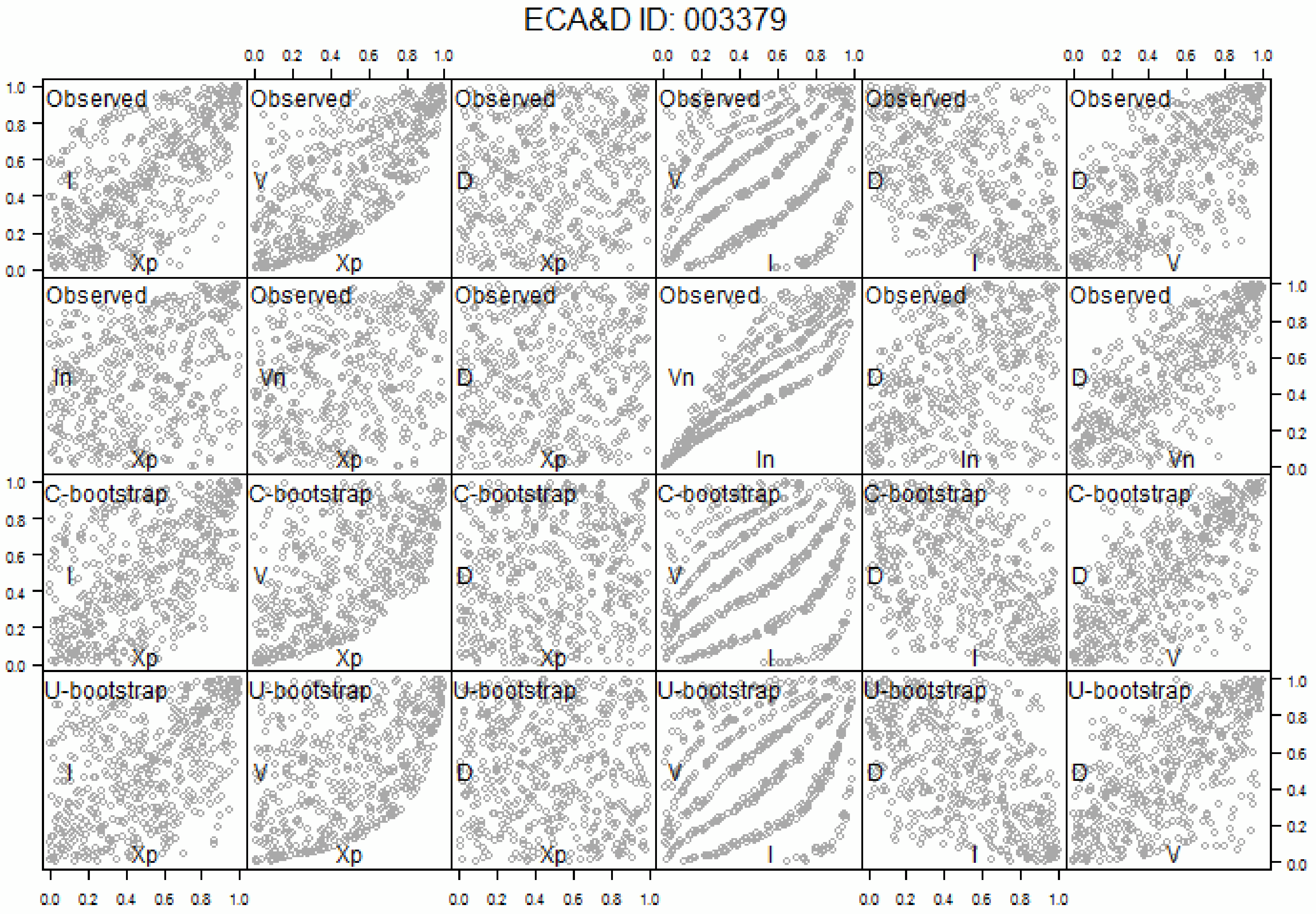
ECA&D ID: 003379
Observed
C-bootstrap
U-bootstrap
Xp
I
V
D
In
Vn
0.0
0.2
0.4
0.6
0.8
1.0

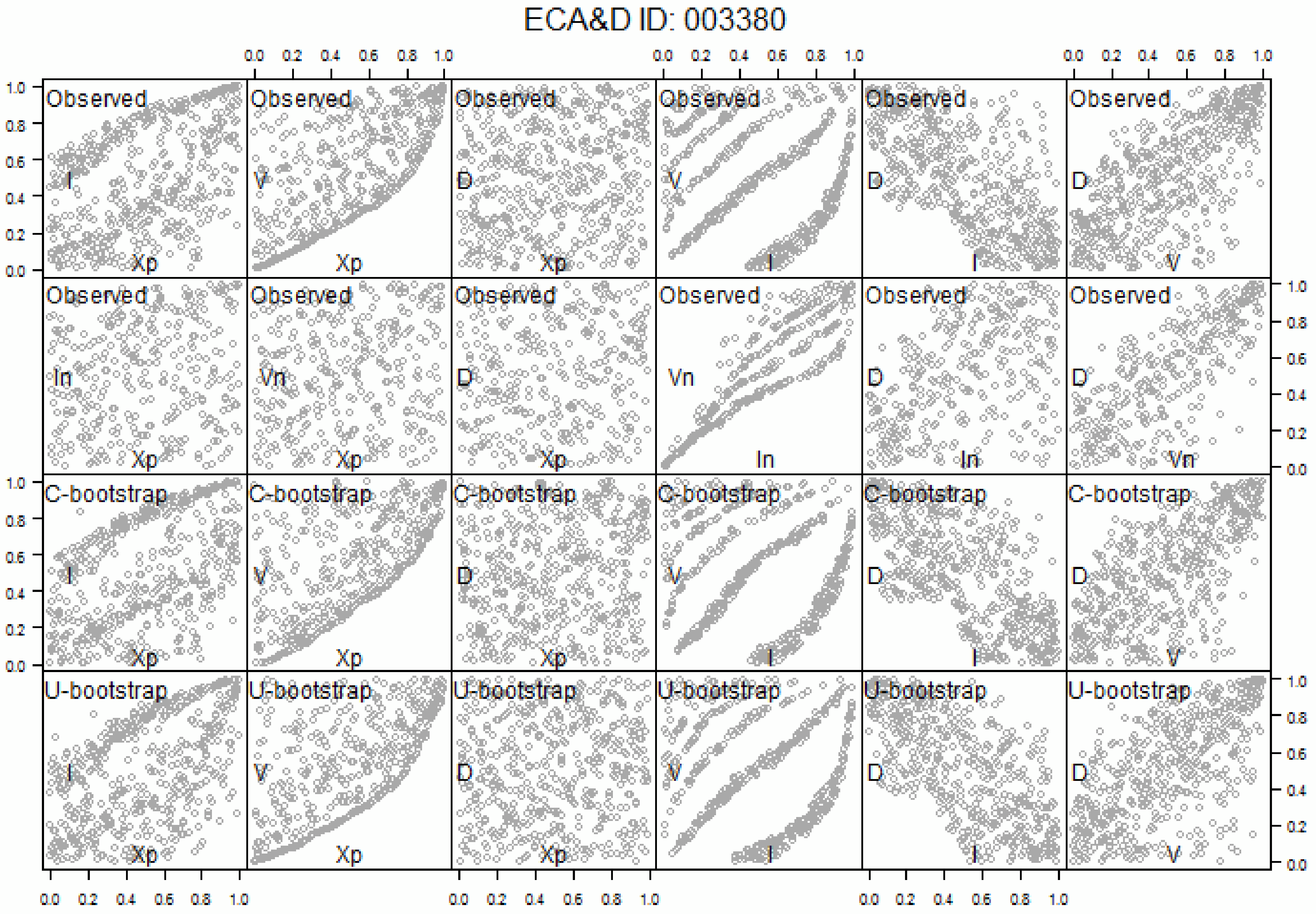
ECA&D ID: 003380
Observed
C-bootstrap
U-bootstrap
Xp
I
V
D
In
Vn
0.0
0.2
0.4
0.6
0.8
1.0

ECA&D ID: 003382

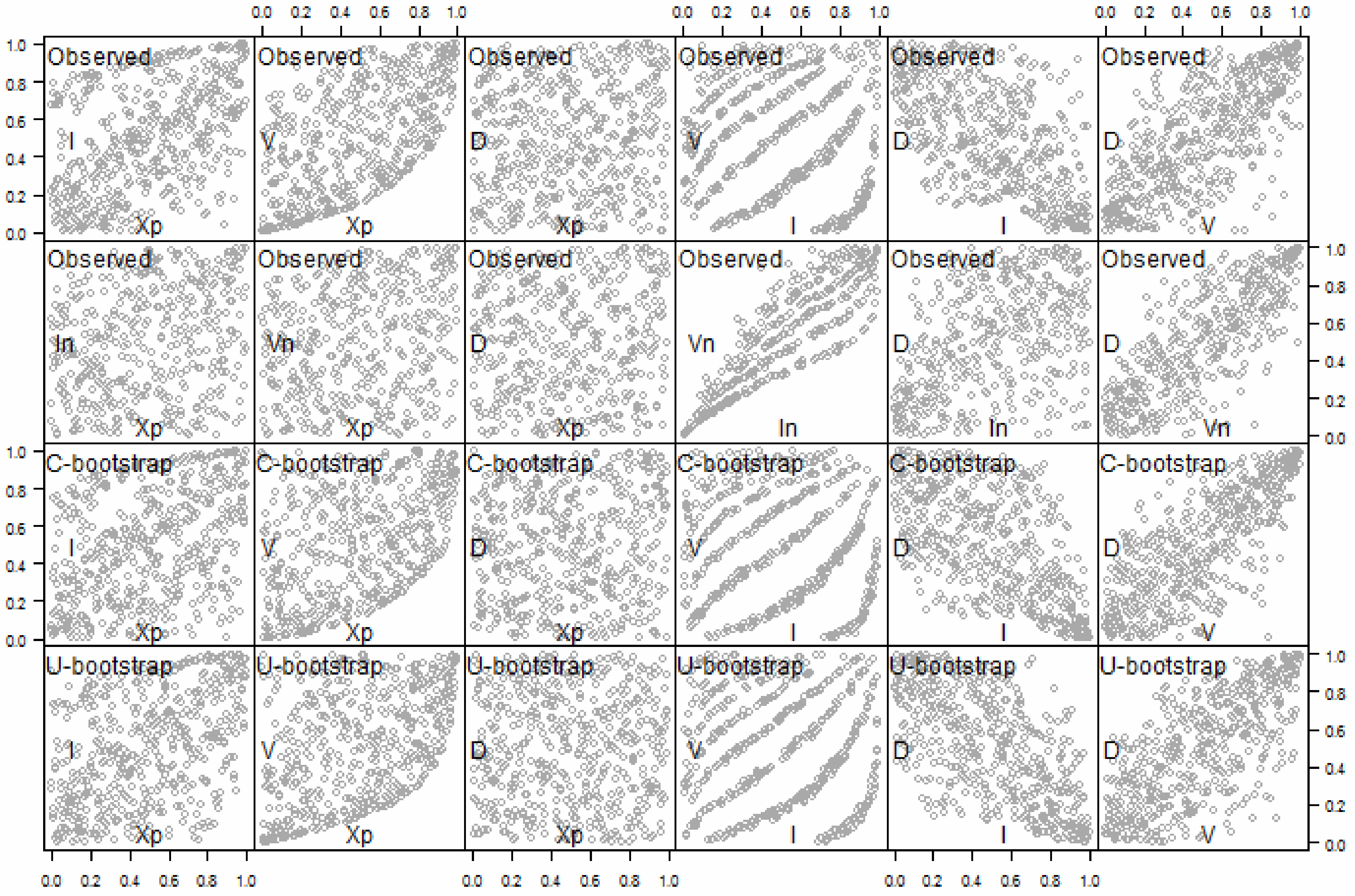

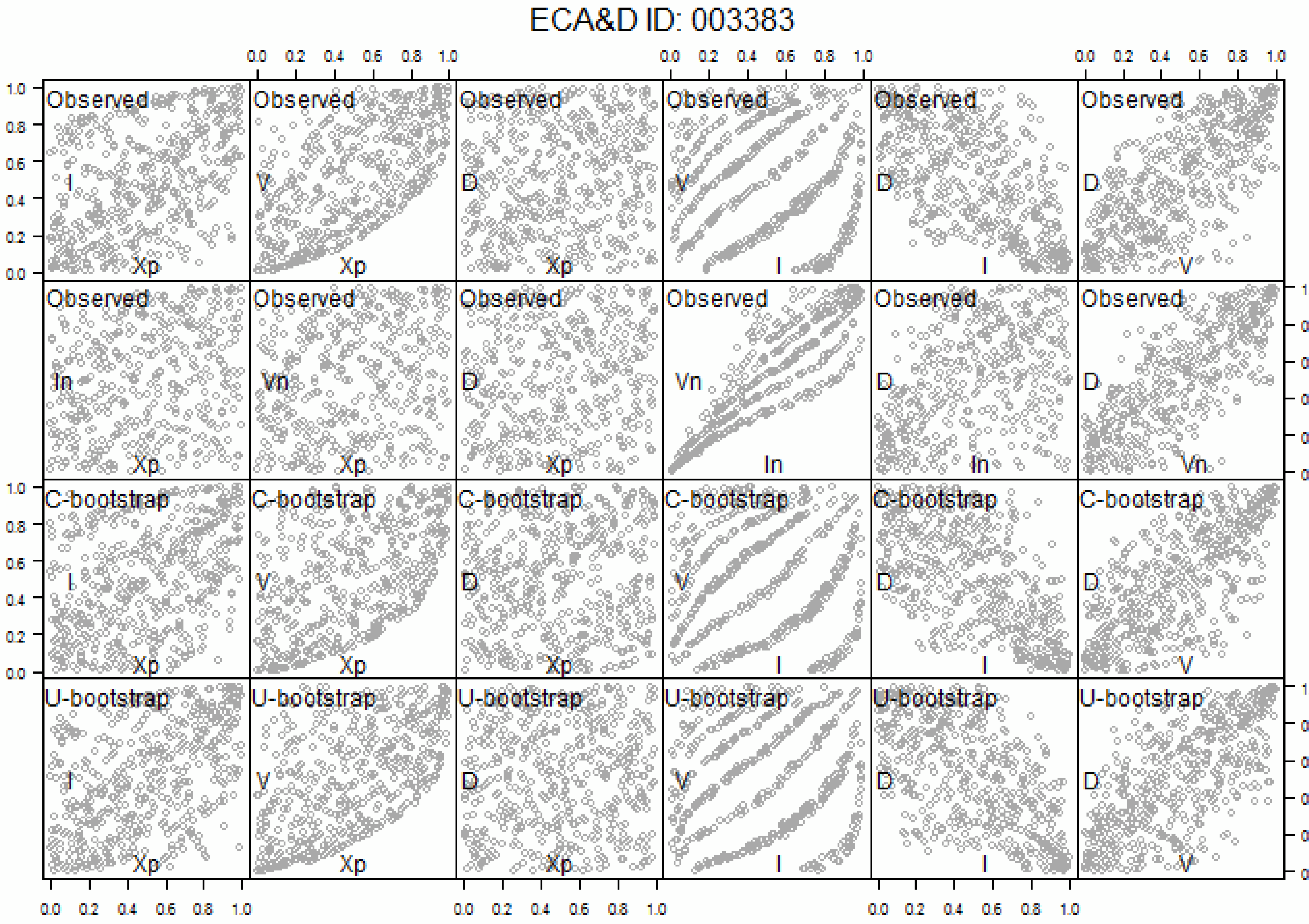

ECA&D ID: 003383
Observed
C-bootstrap
U-bootstrap
Xp
I
V
D
In
Vn
0.0
0.2
0.4
0.6
0.8
1.0

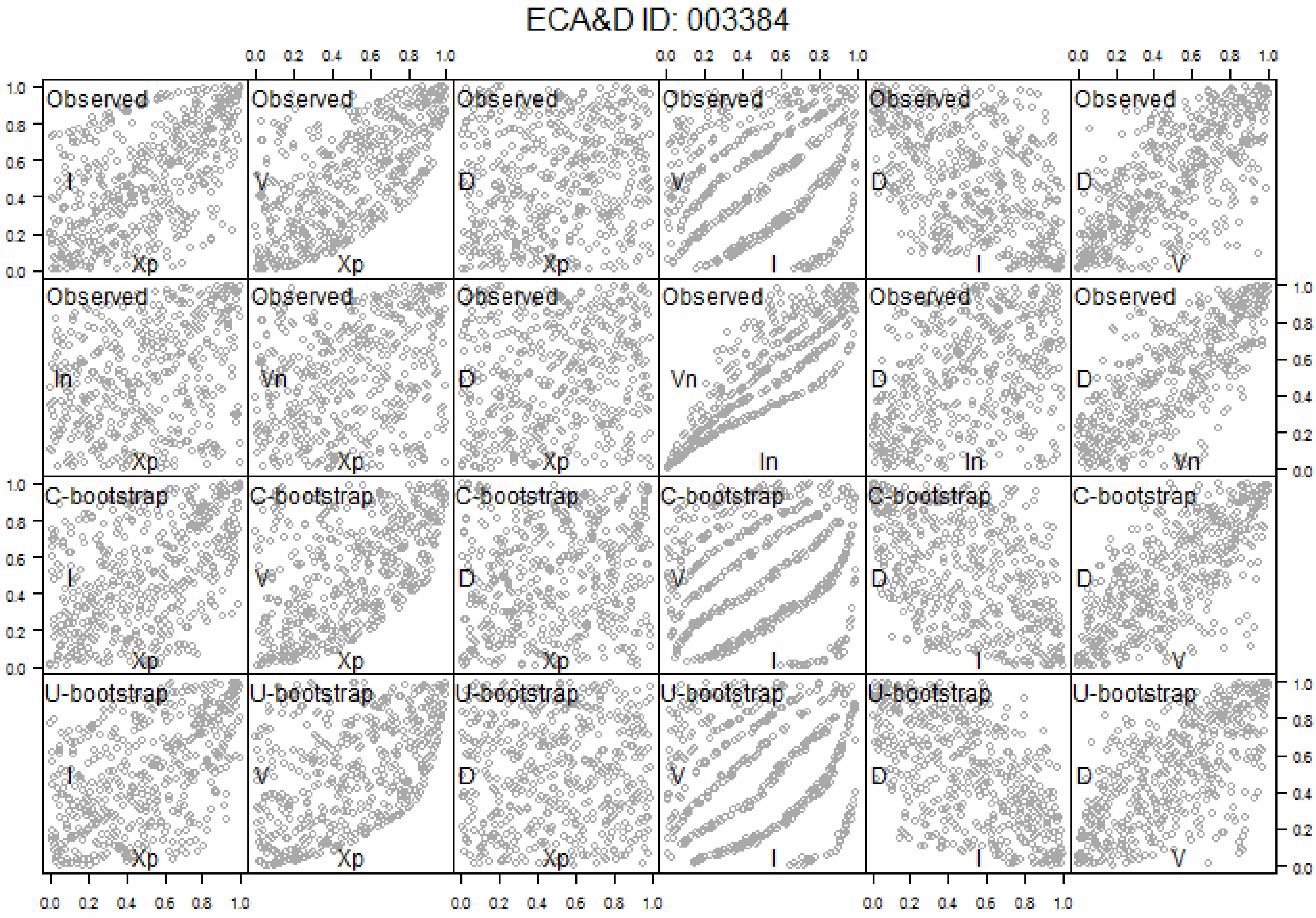
ECA&D ID: 003384
Observed
C-bootstrap
U-bootstrap
Xp
I
V
D
In
Vn
0.0
0.2
0.4
0.6
0.8
1.0

ECA&D ID: 003385

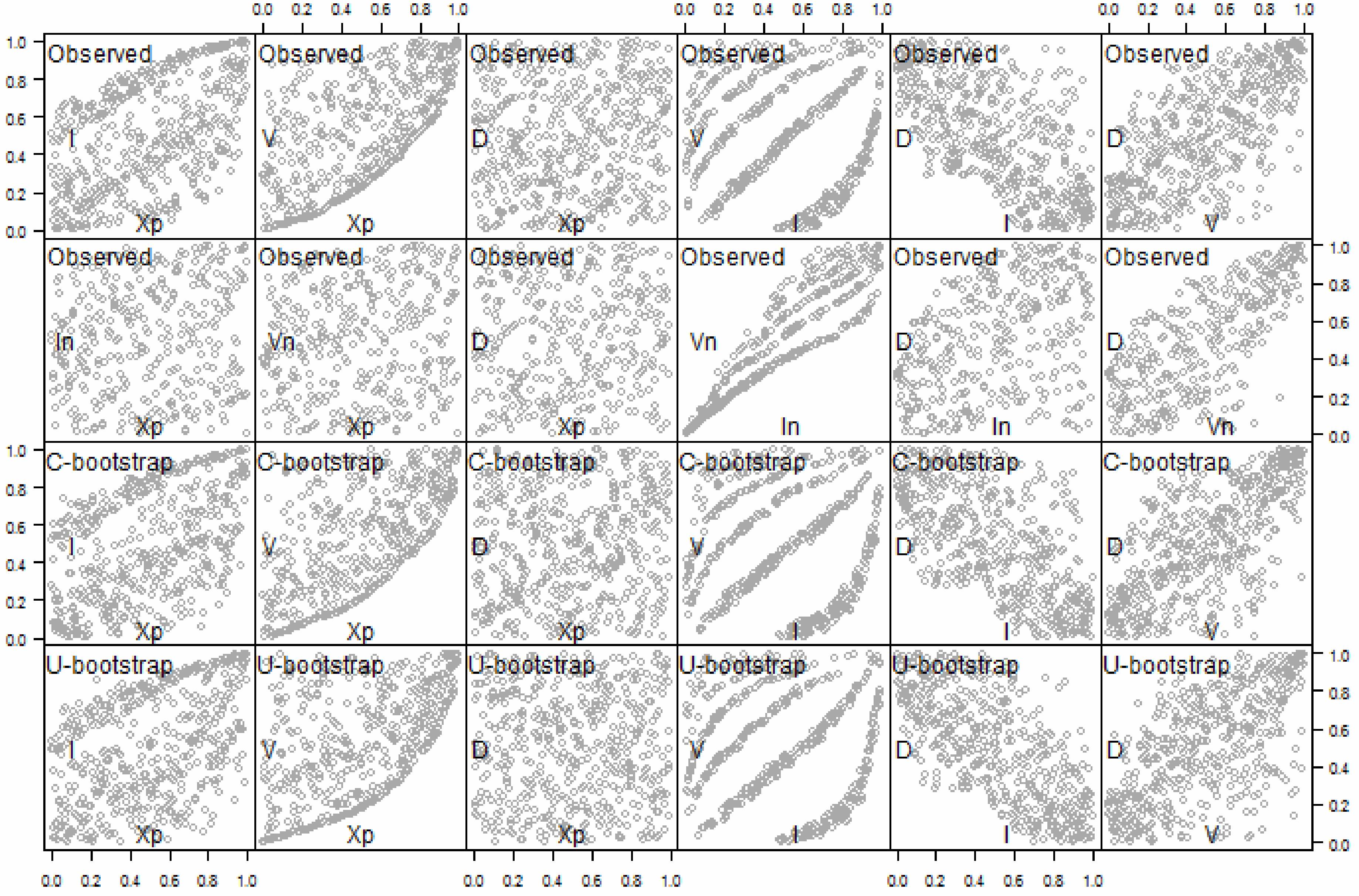

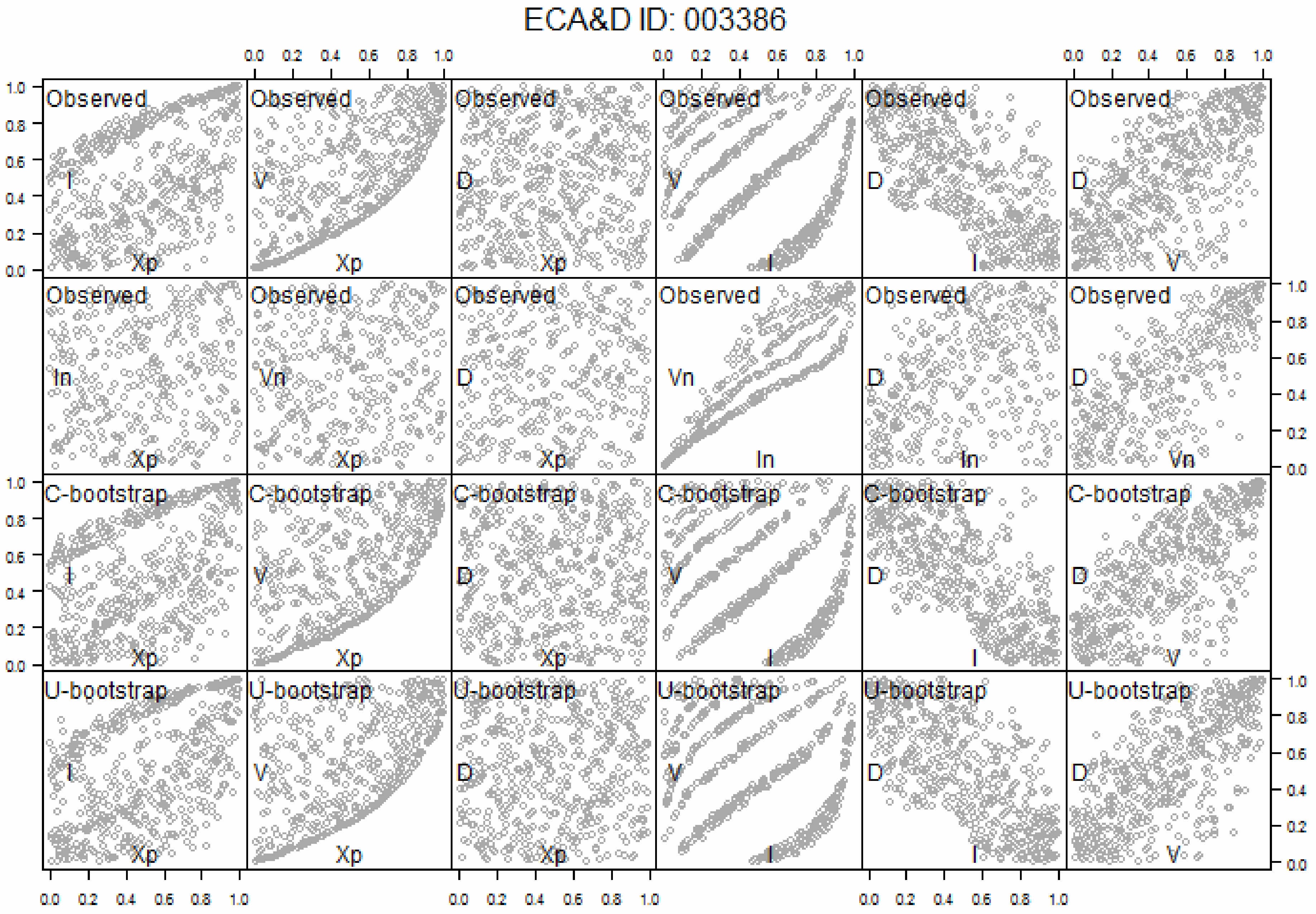
ECA&D ID: 003386
Observed
C-bootstrap
U-bootstrap
Xp
I
V
D
In
Vn
0.0
0.2
0.4
0.6
0.8
1.0

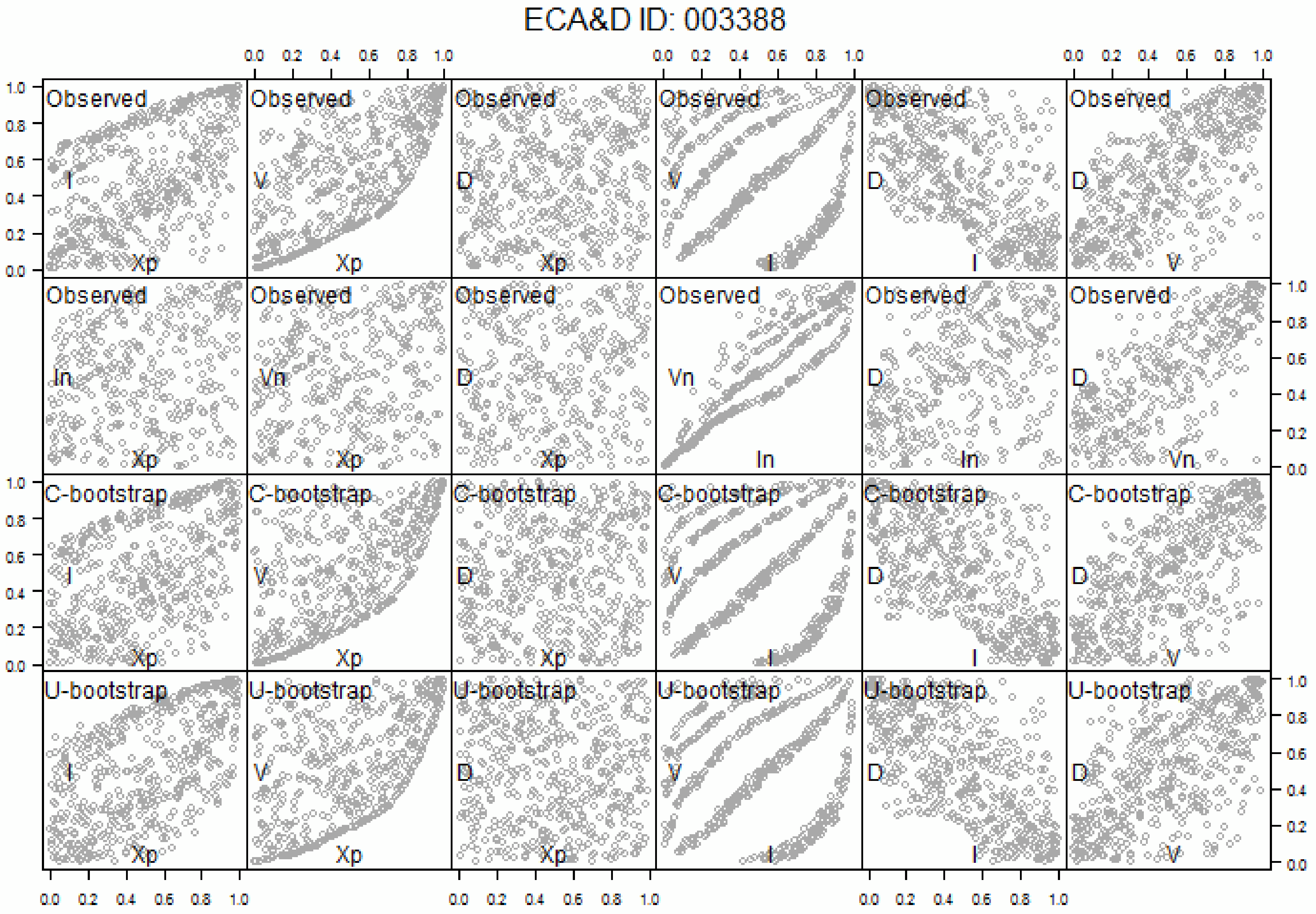

ECA&D ID: 003388
Observed
C-bootstrap
U-bootstrap
Xp
I
V
D
In
Vn
0.0
0.2
0.4
0.6
0.8
1.0

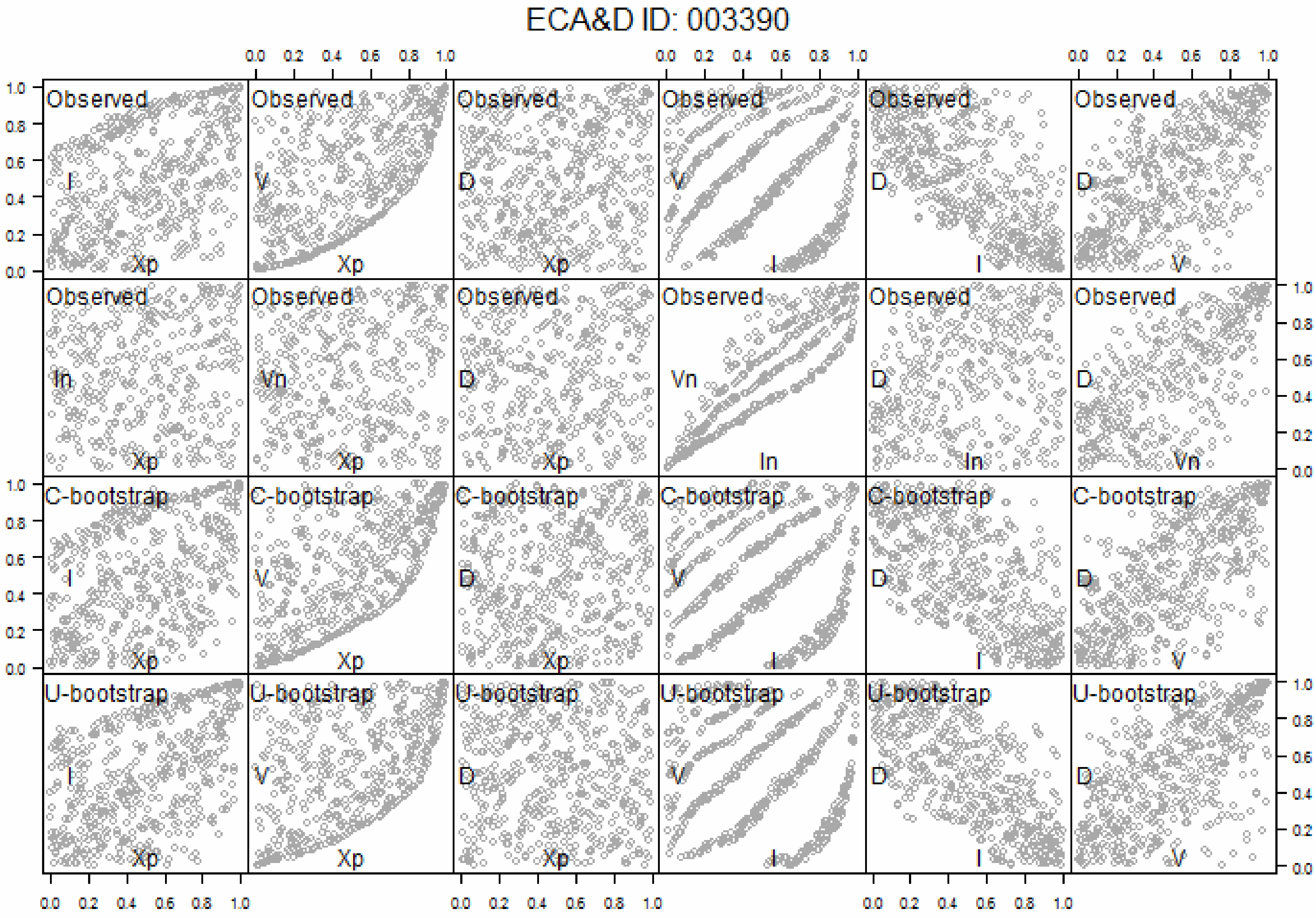
ECA&D ID: 003390
Observed
C-bootstrap
U-bootstrap
I
V
D
In
Vn
Xp
0.0
0.2
0.4
0.6
0.8
1.0

ECA&D ID: 003391

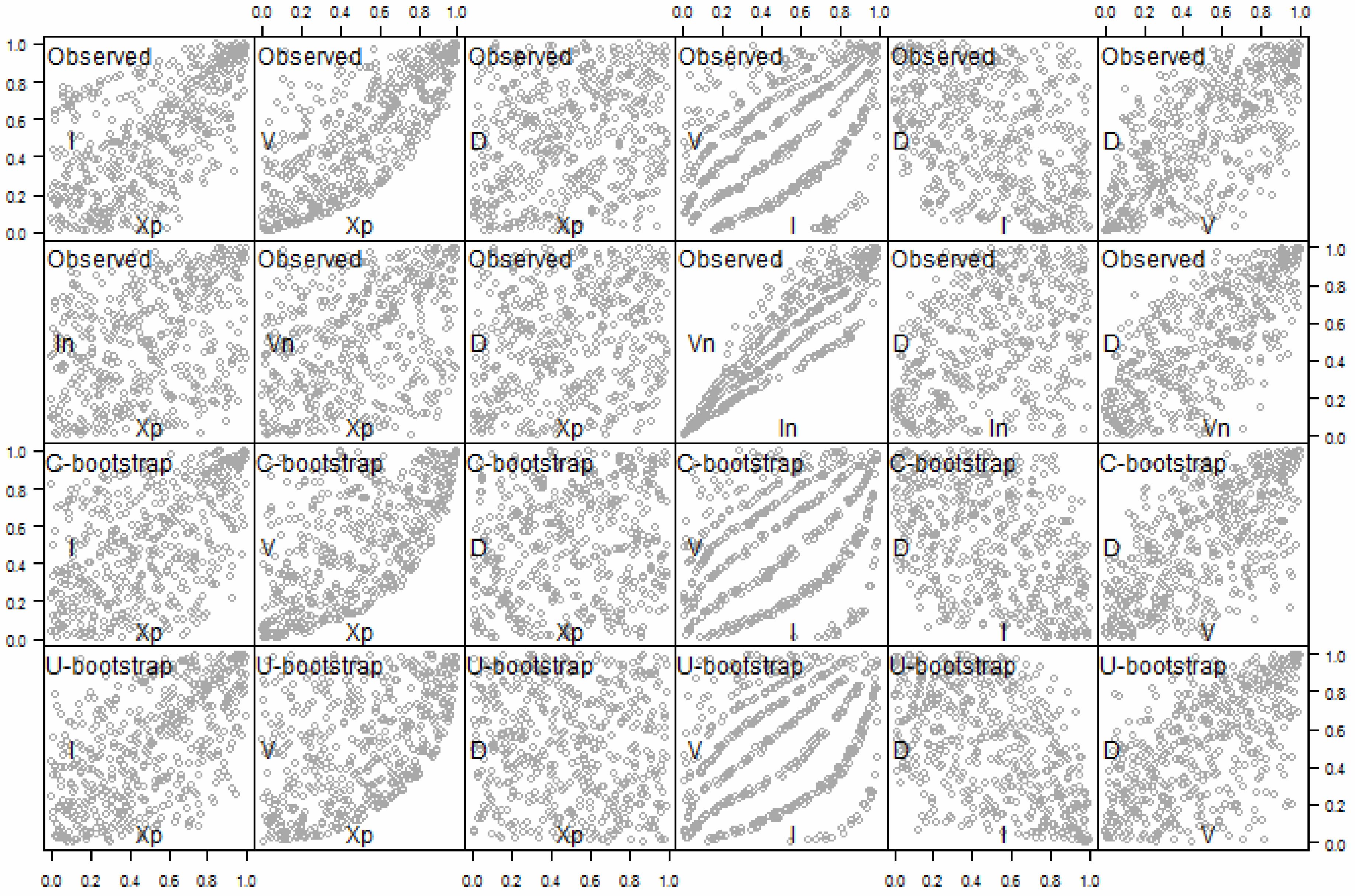

ECA&D ID: 003392

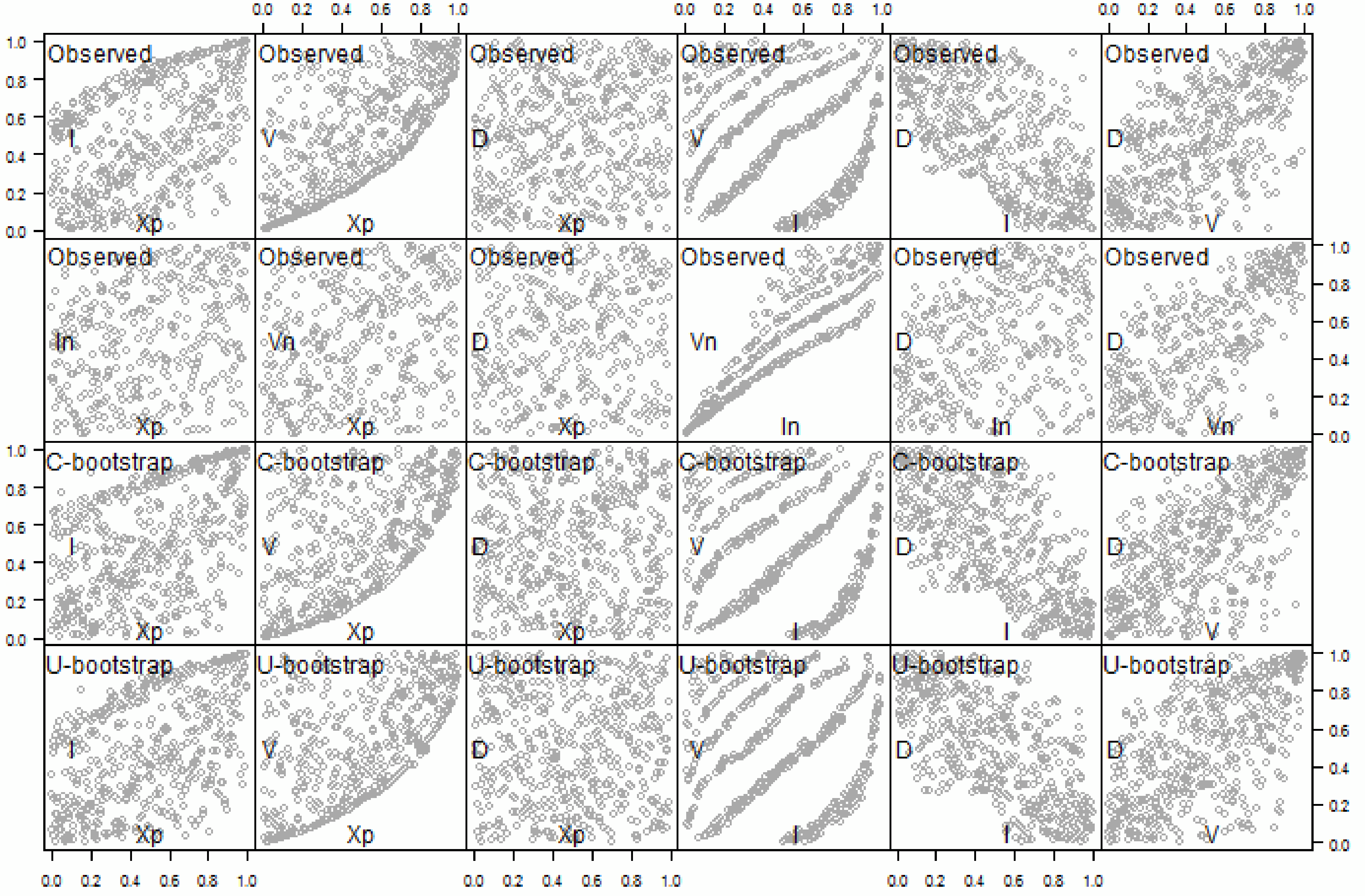

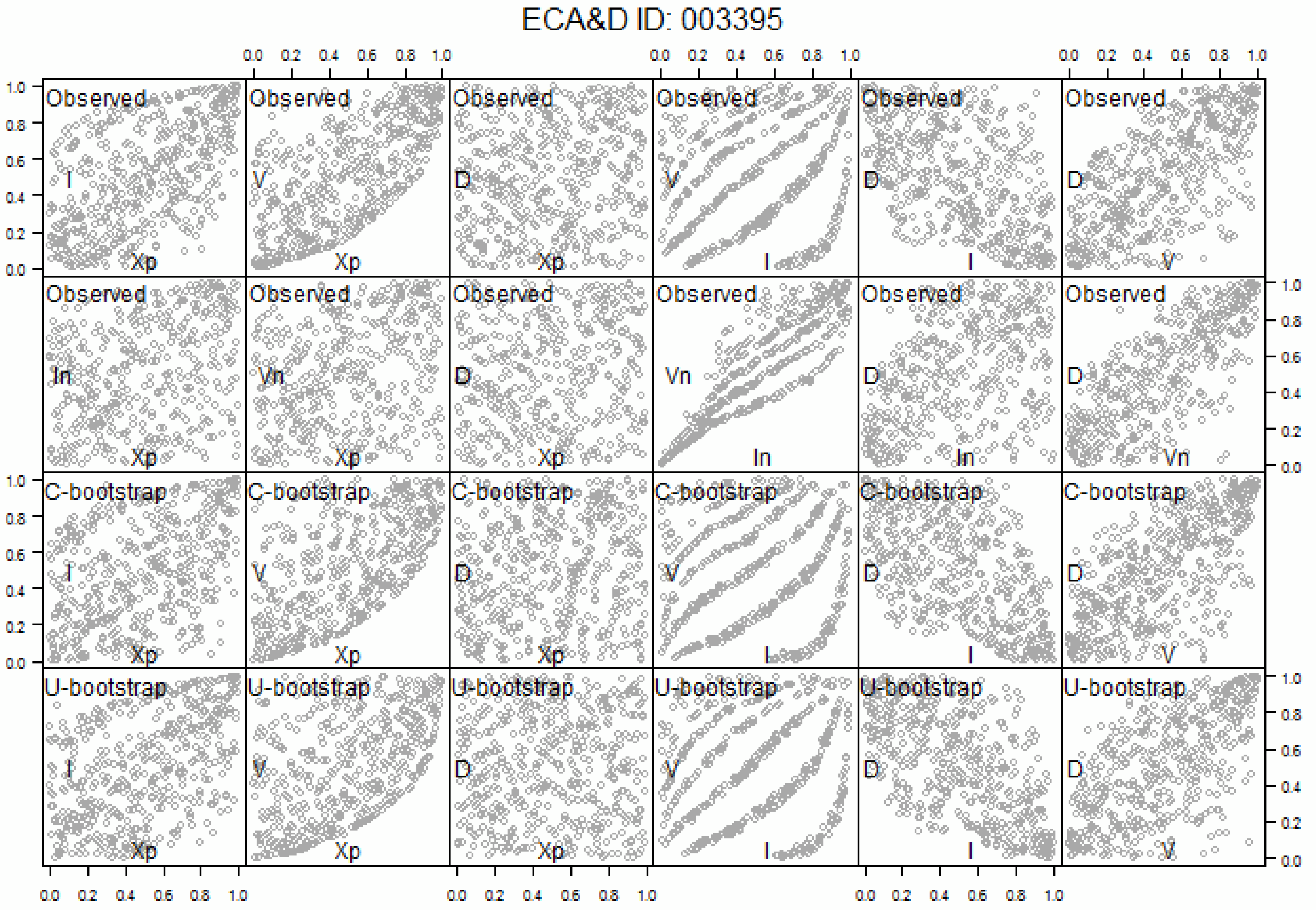
ECA&D ID: 003395
Observed
C-bootstrap
U-bootstrap
Xp
I
V
D
In
Vn
0.0
0.2
0.4
0.6
0.8
1.0

ECA&D ID: 003396

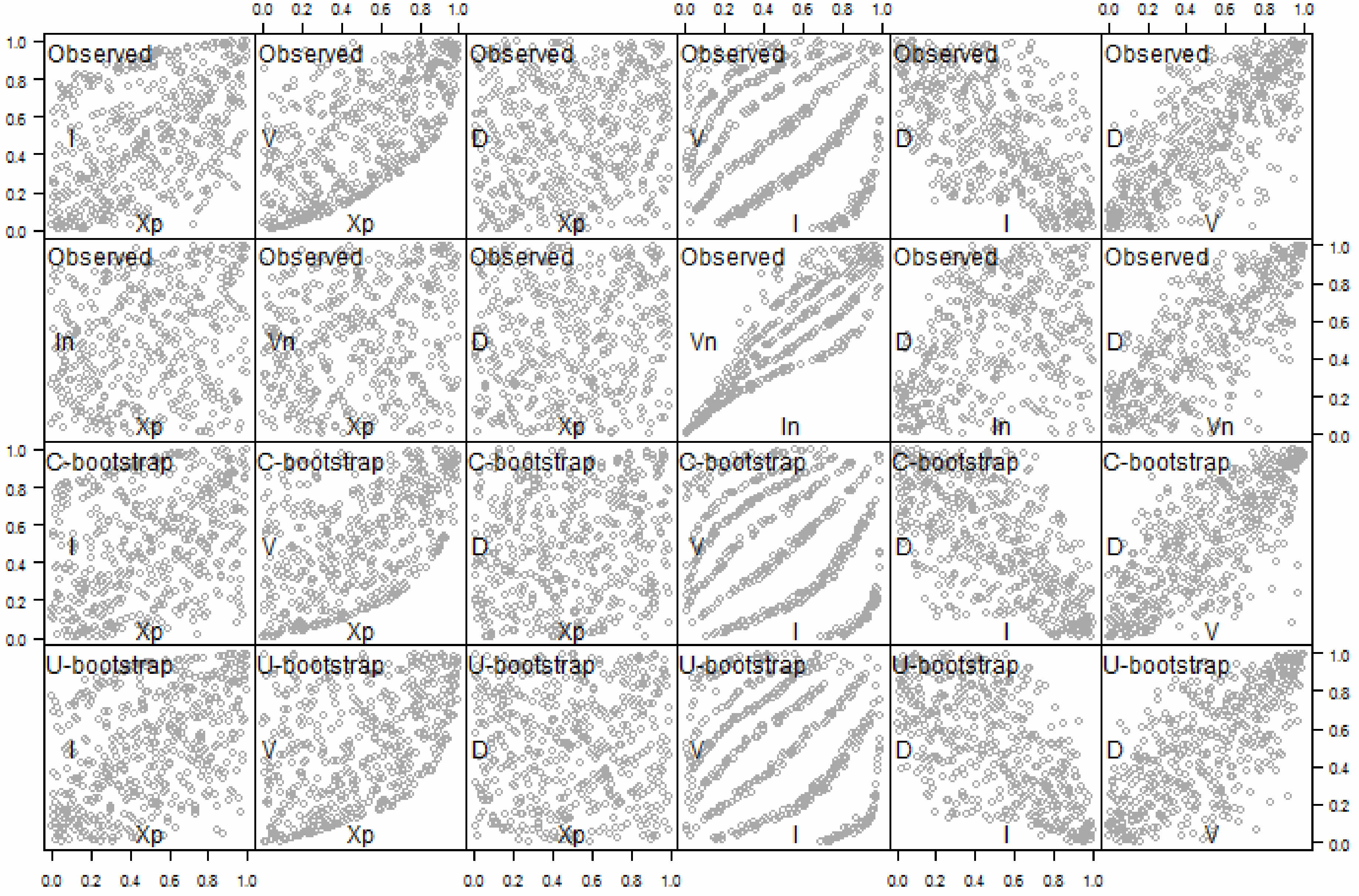

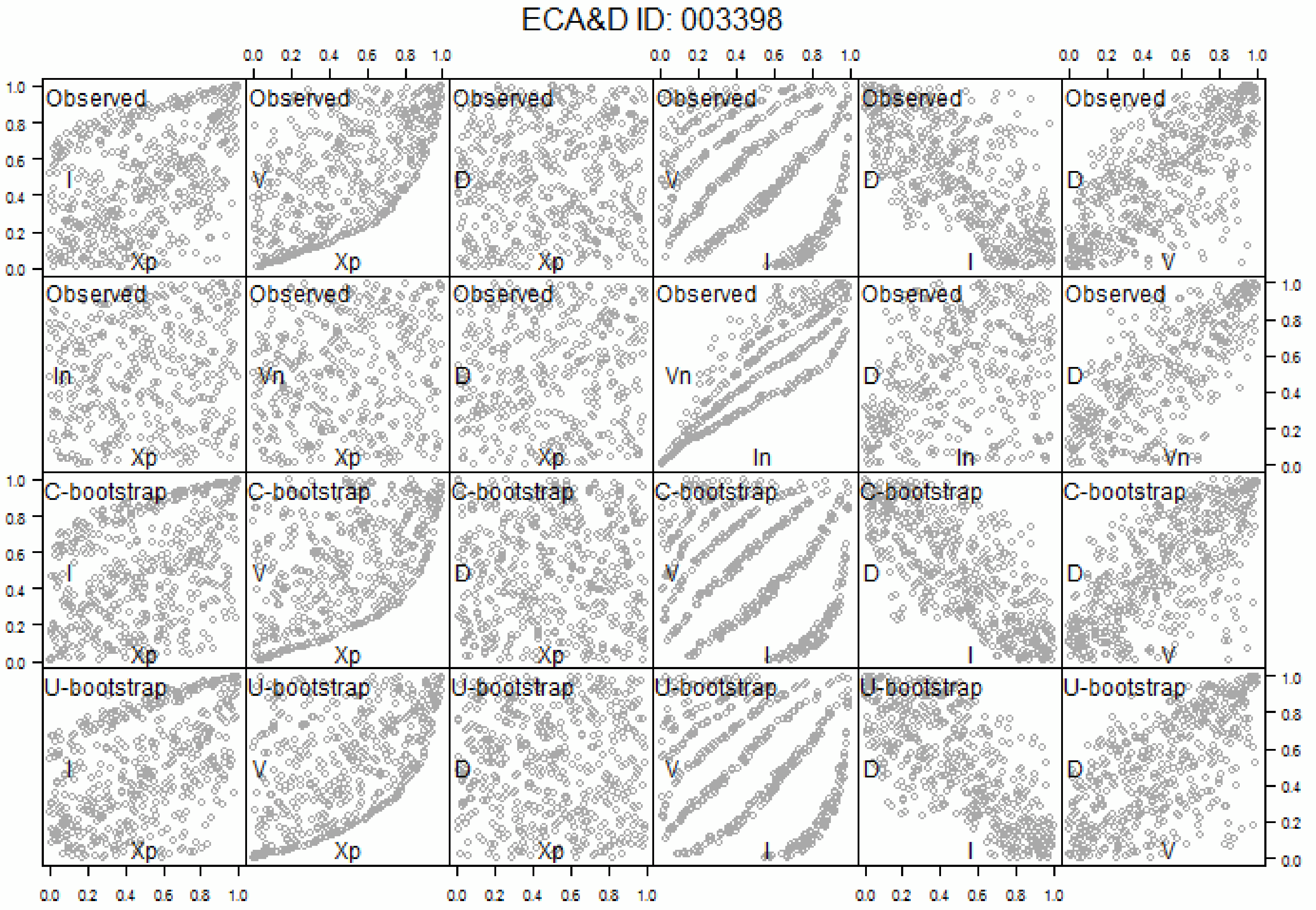
ECA&D ID: 003398
Observed
C-bootstrap
U-bootstrap
Xp
I
V
D
In
Vn
0.0
0.2
0.4
0.6
0.8
1.0

ECA&D ID: 003399

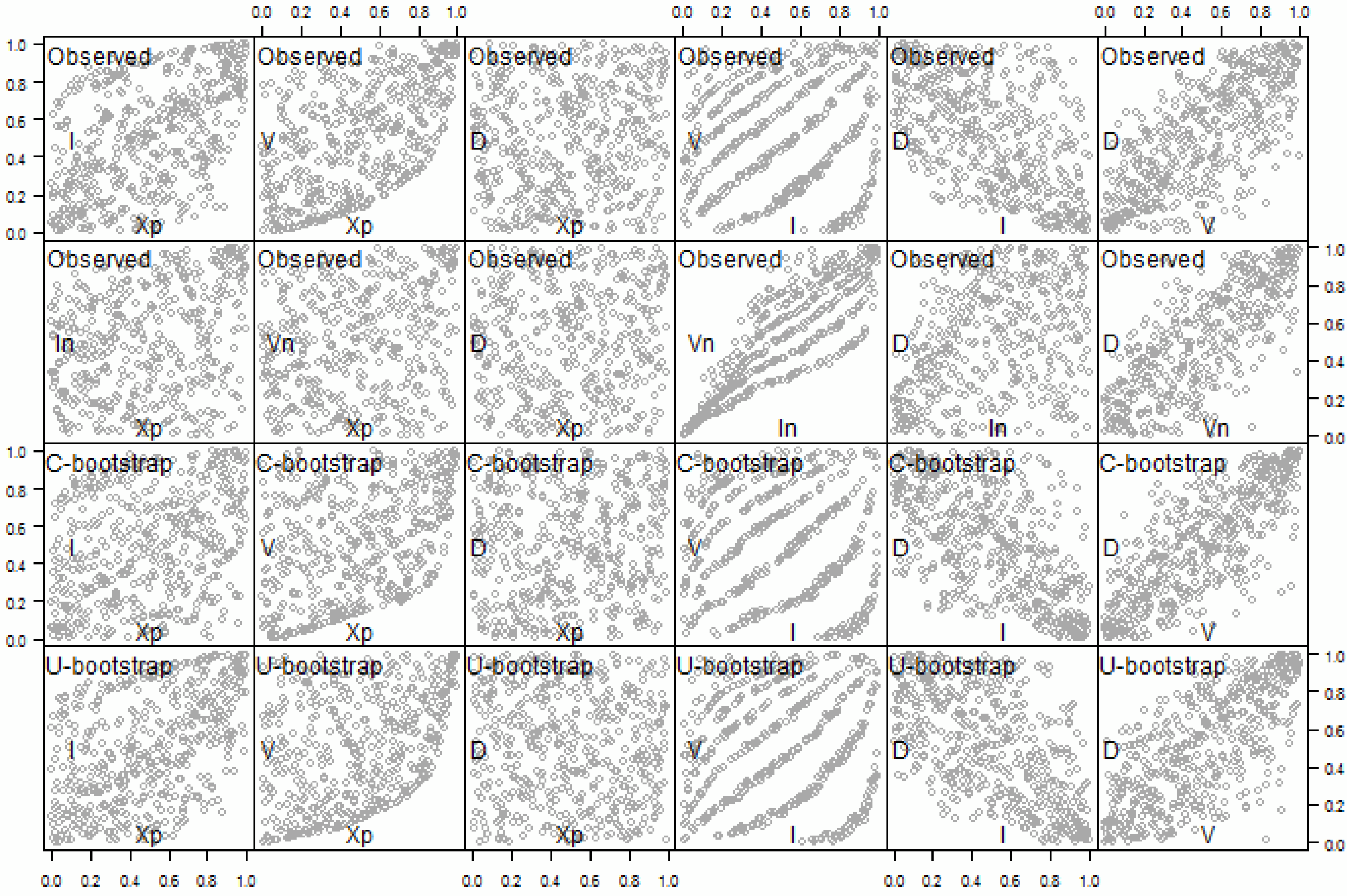

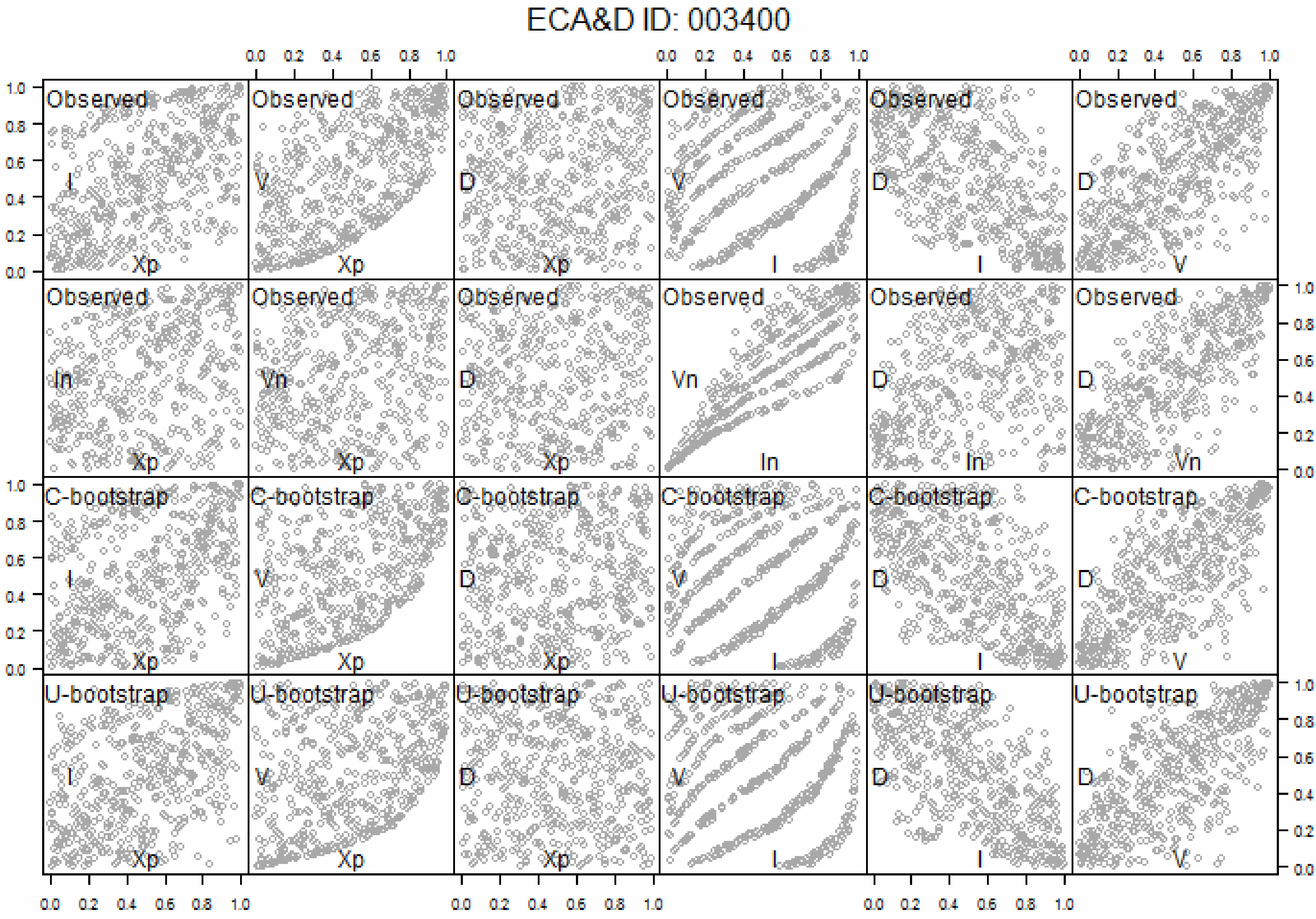
ECA&D ID: 003400
Observed
C-bootstrap
U-bootstrap
Xp
I
V
D
In
Vn
0.0
0.2
0.4
0.6
0.8
1.0

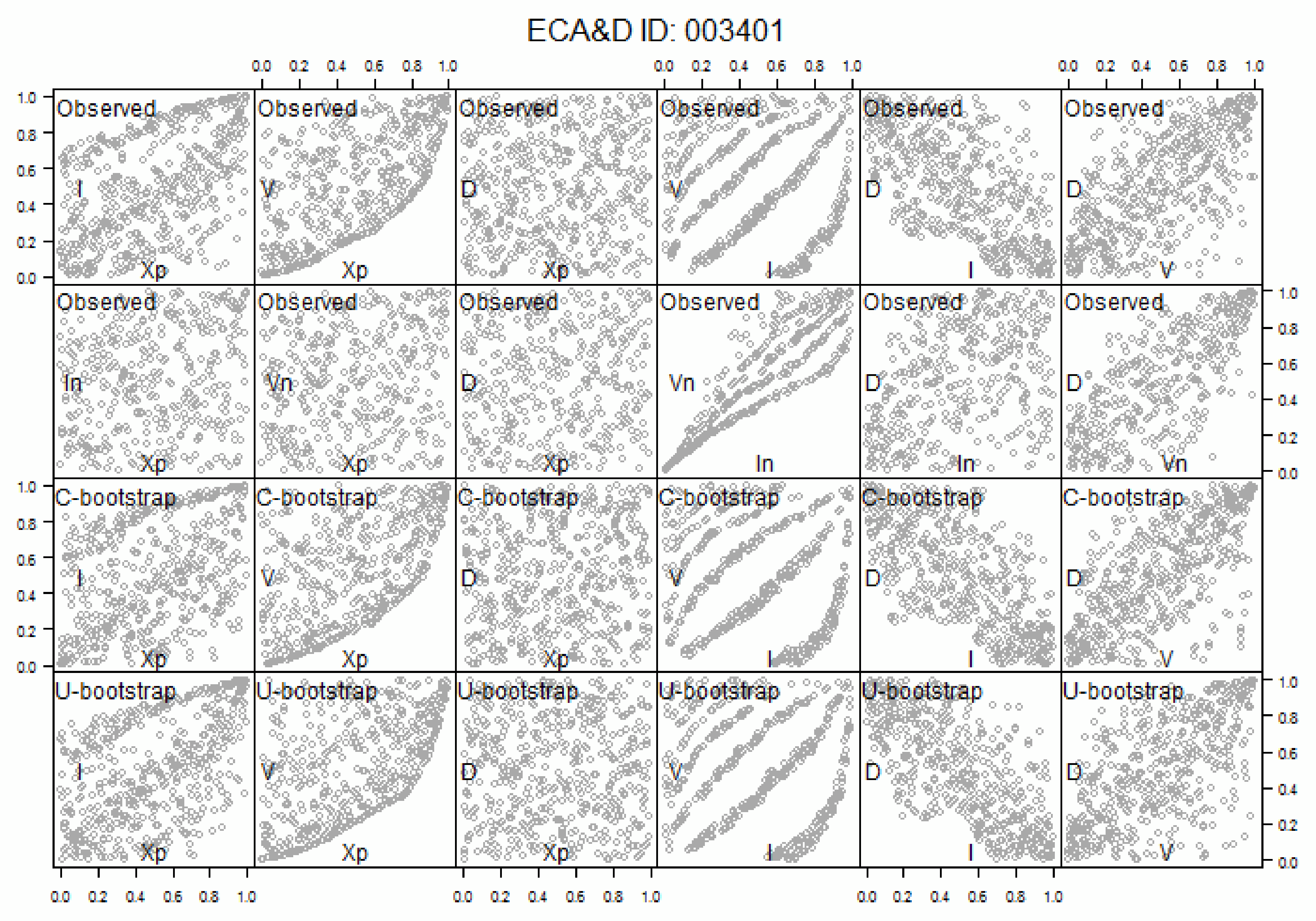
ECA&D ID: 003401
Observed
C-bootstrap
U-bootstrap
Xp
I
V
D
In
Vn
0.0
0.2
0.4
0.6
0.8
1.0

ECA&D ID: 003402

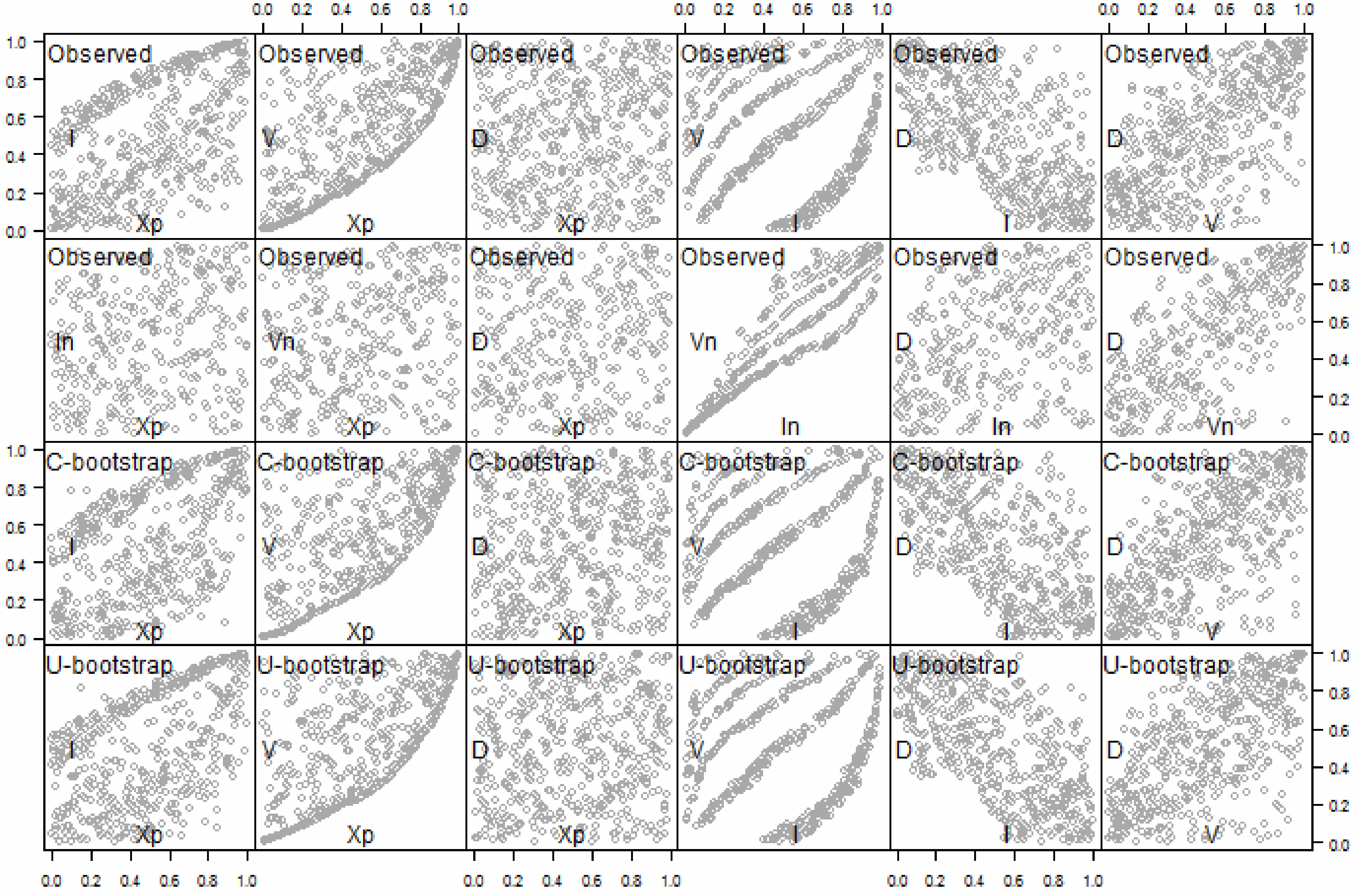

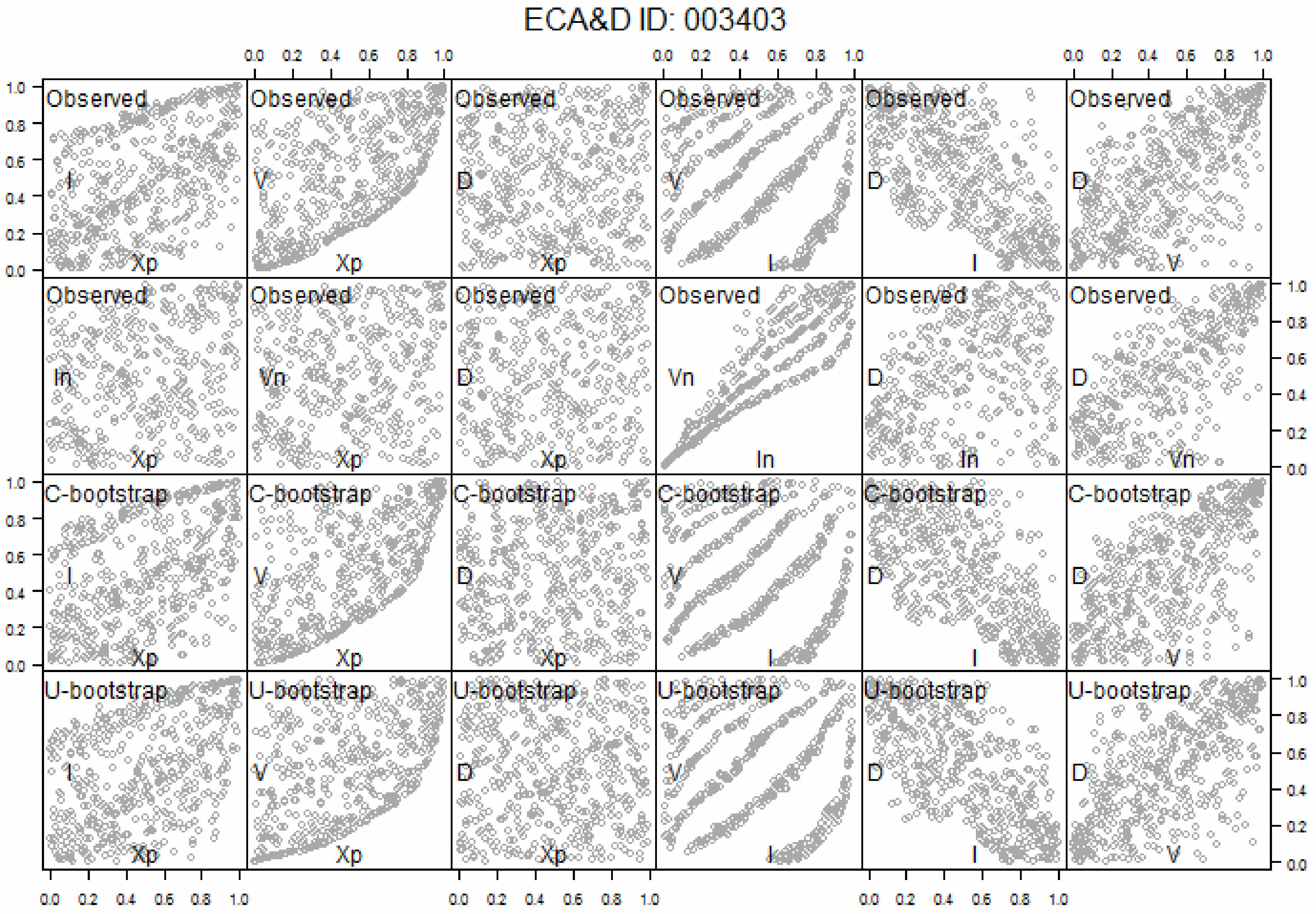
ECA&D ID: 003403
Observed
C-bootstrap
U-bootstrap
Xp
I
V
D
In
Vn
0.0
0.2
0.4
0.6
0.8
1.0

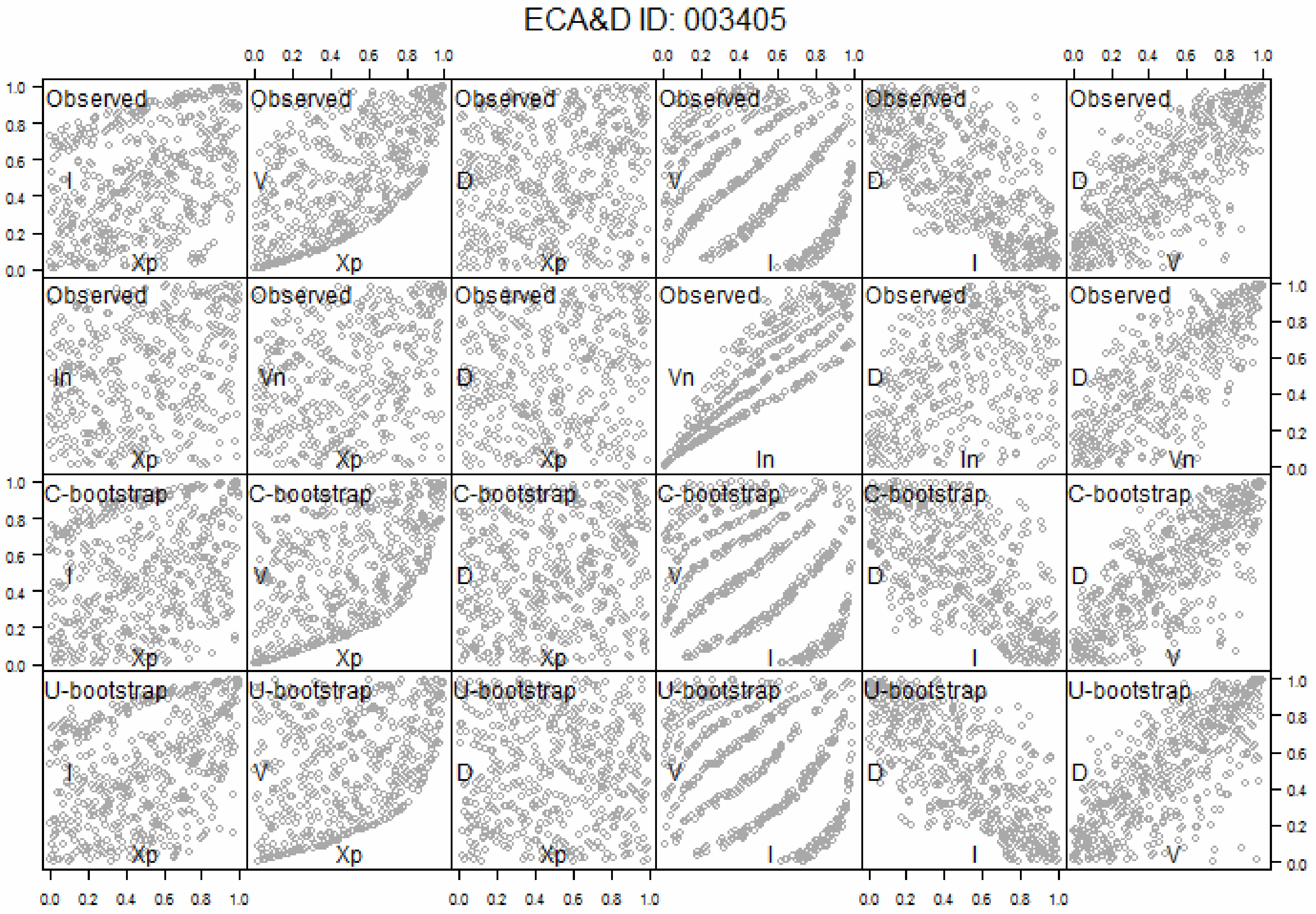
ECA&D ID: 003405
Observed
C-bootstrap
U-bootstrap
Xp
I
V
D
In
Vn
0.0
0.2
0.4
0.6
0.8
1.0

ECA&D ID: 003406

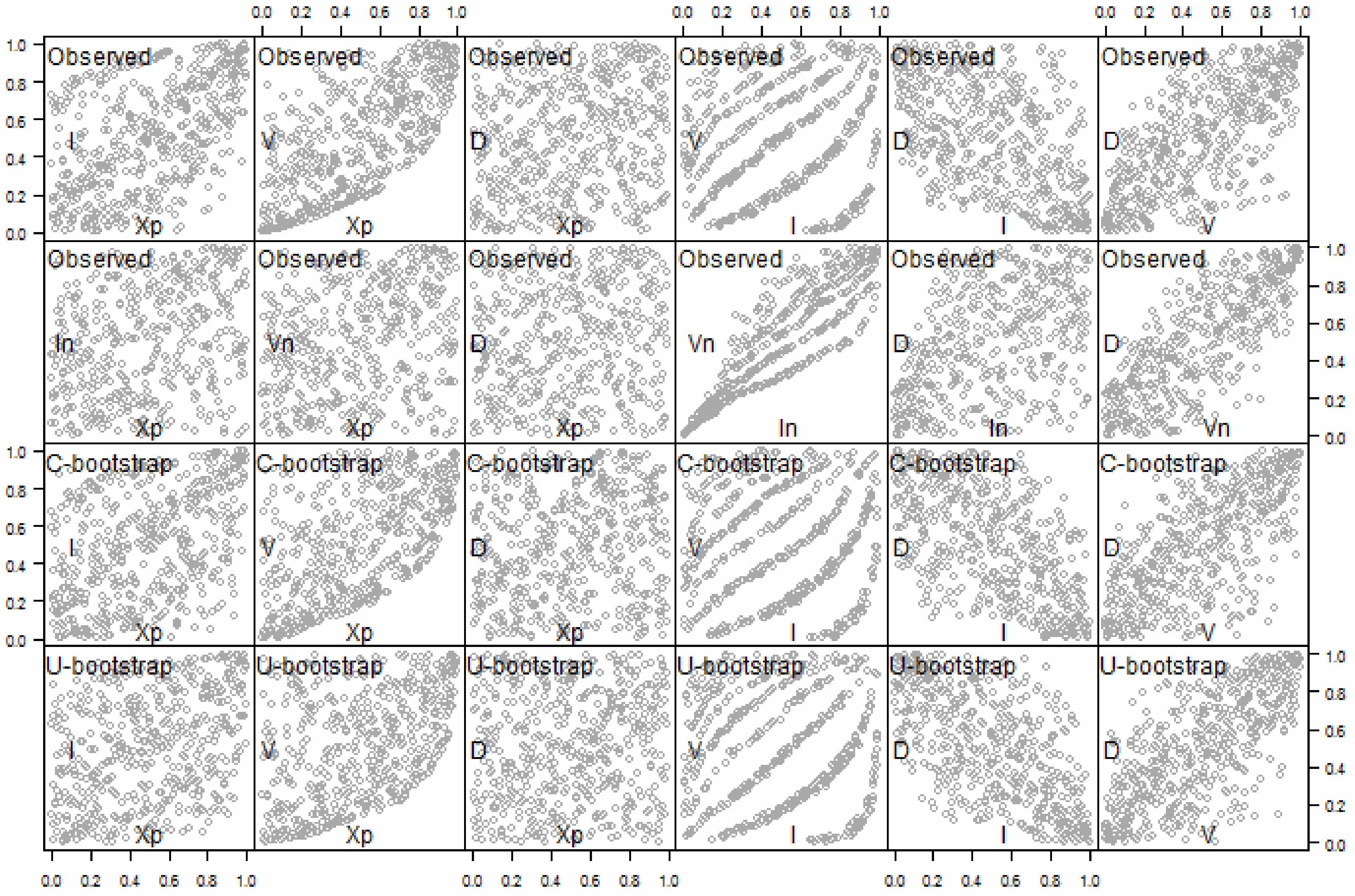

ECA&D ID: 003407

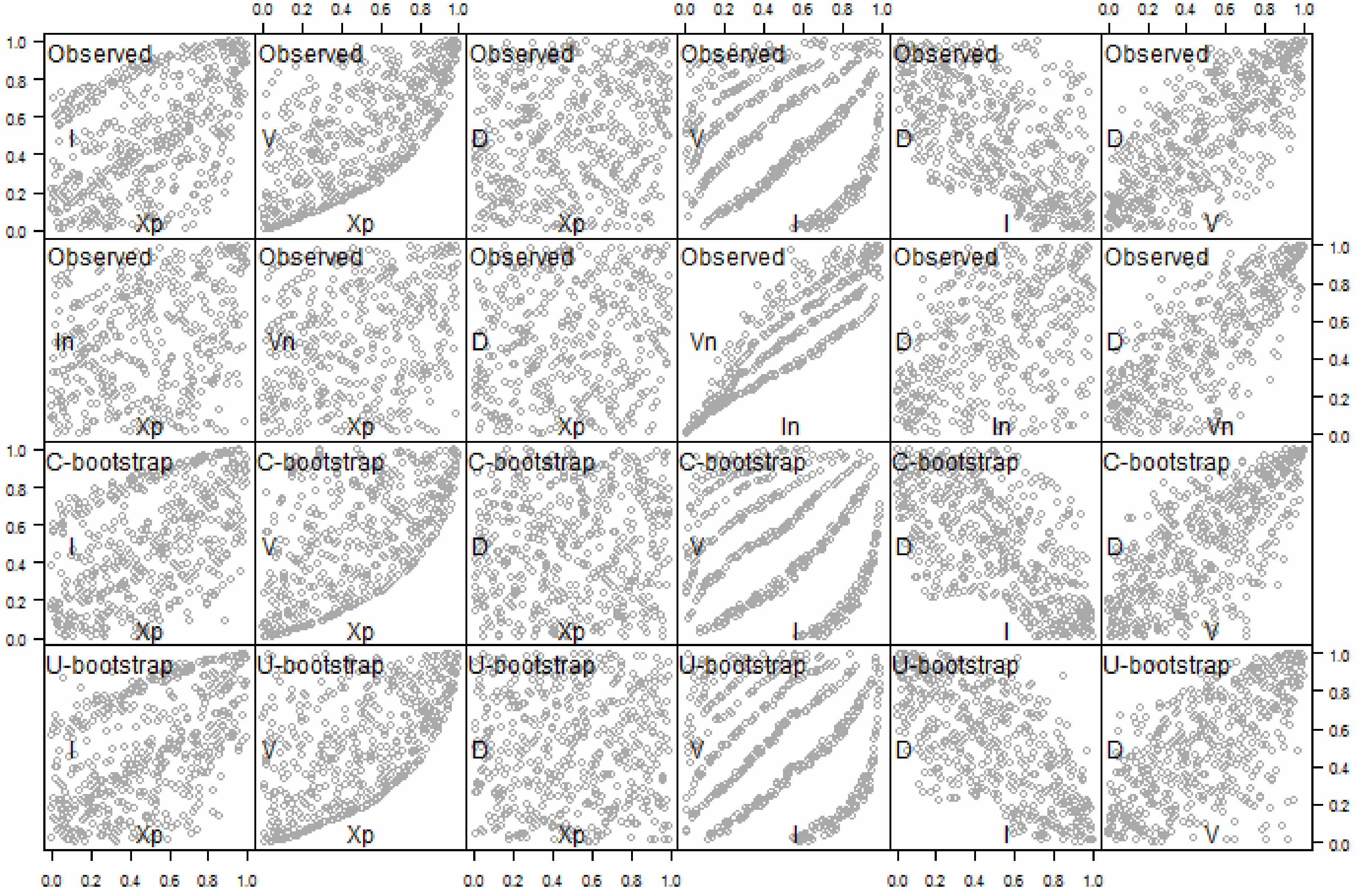

ECA&D ID: 003409

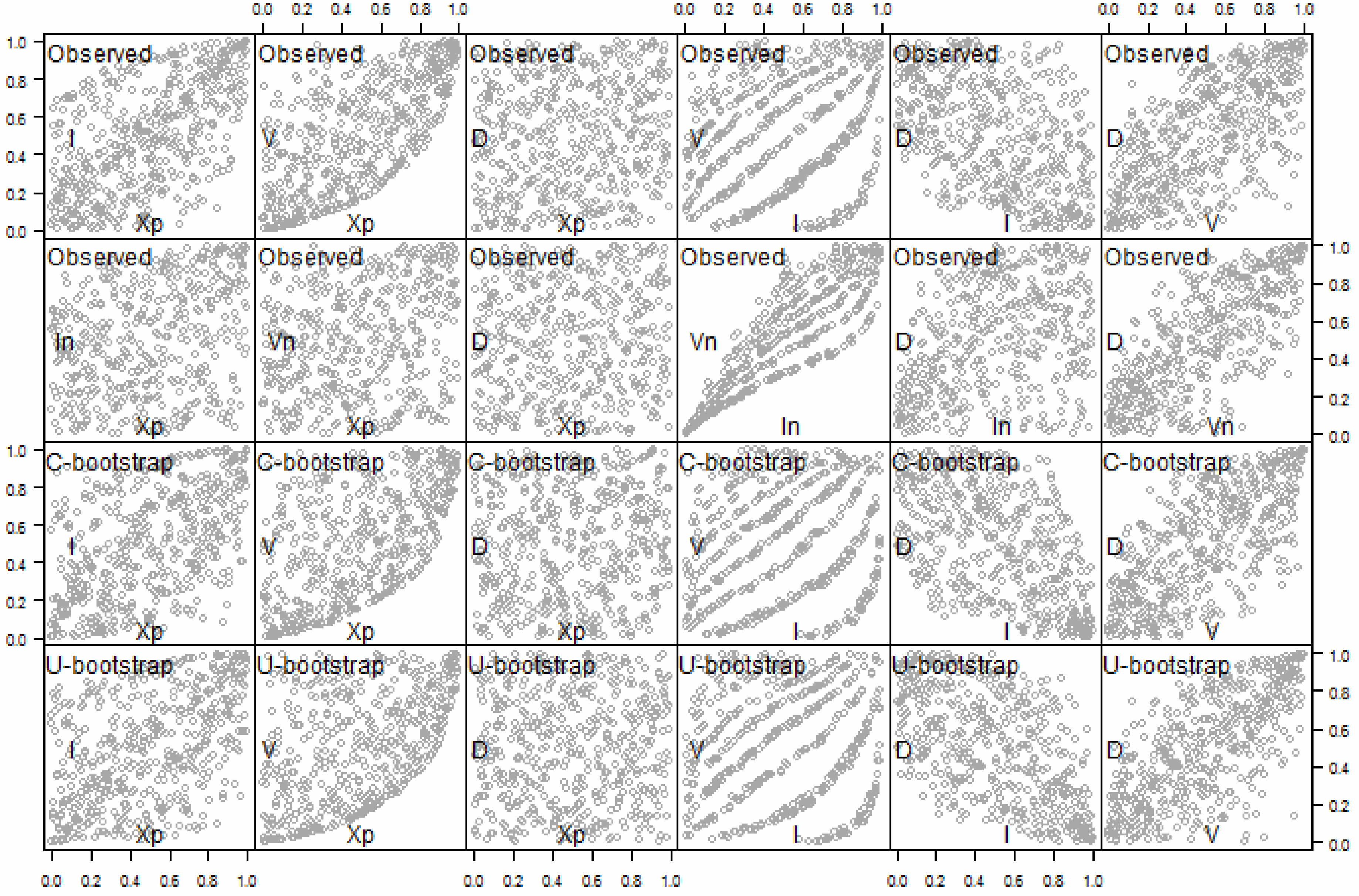

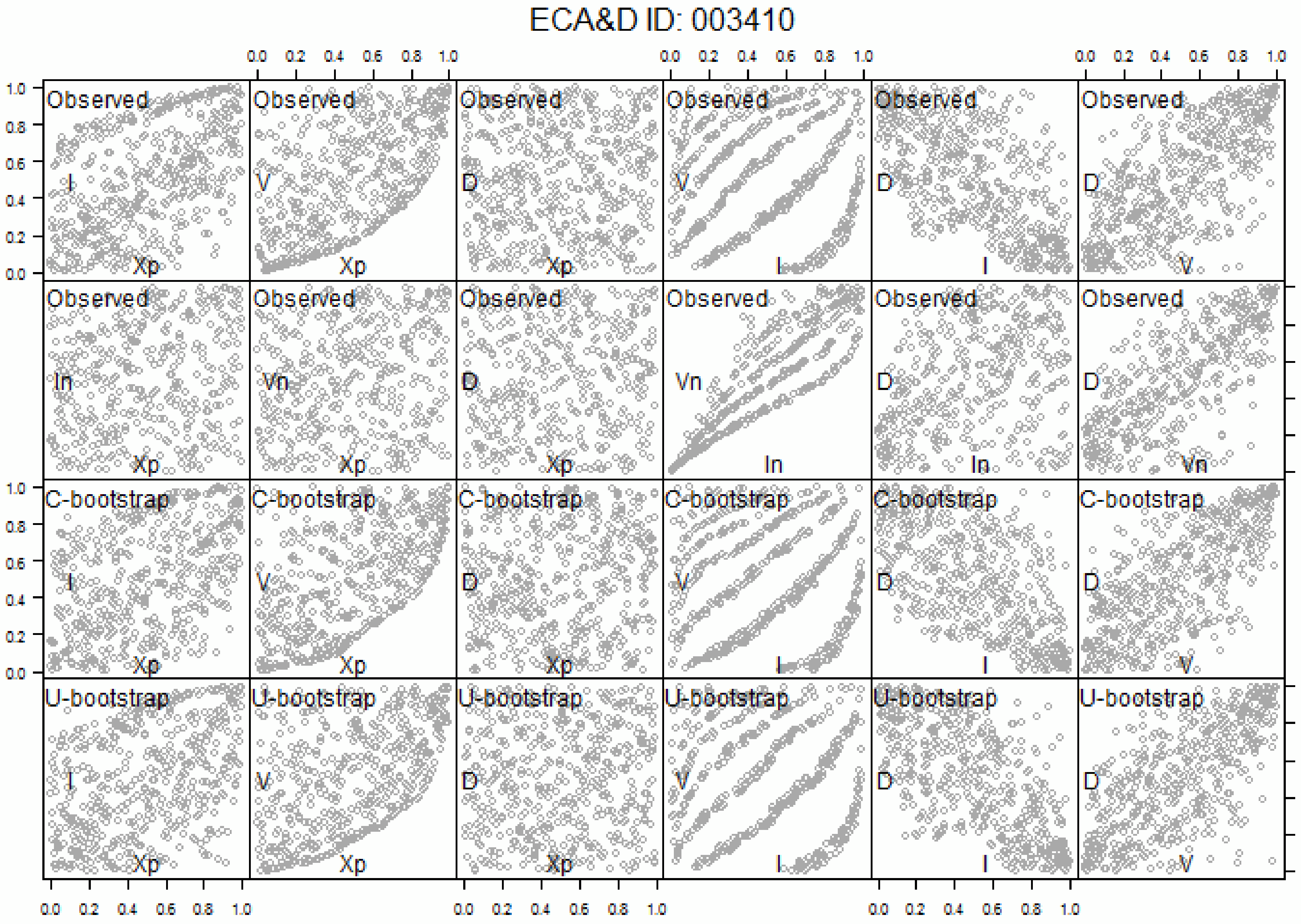

ECA&D ID: 003410
Observed
C-bootstrap
U-bootstrap
Xp
I
V
D
In
Vn
0.0
0.2
0.4
0.6
0.8
1.0

ECA&D ID: 003412

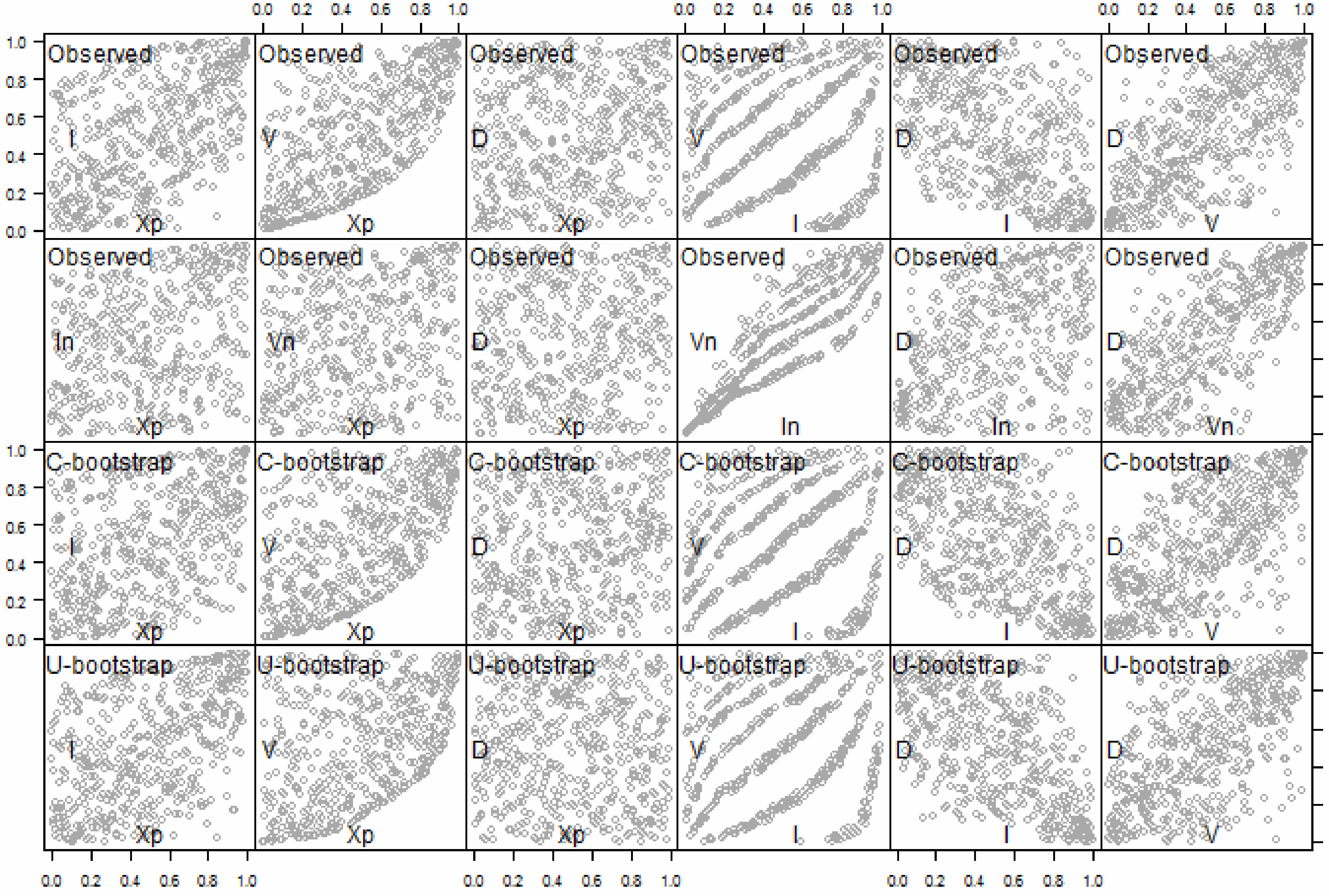

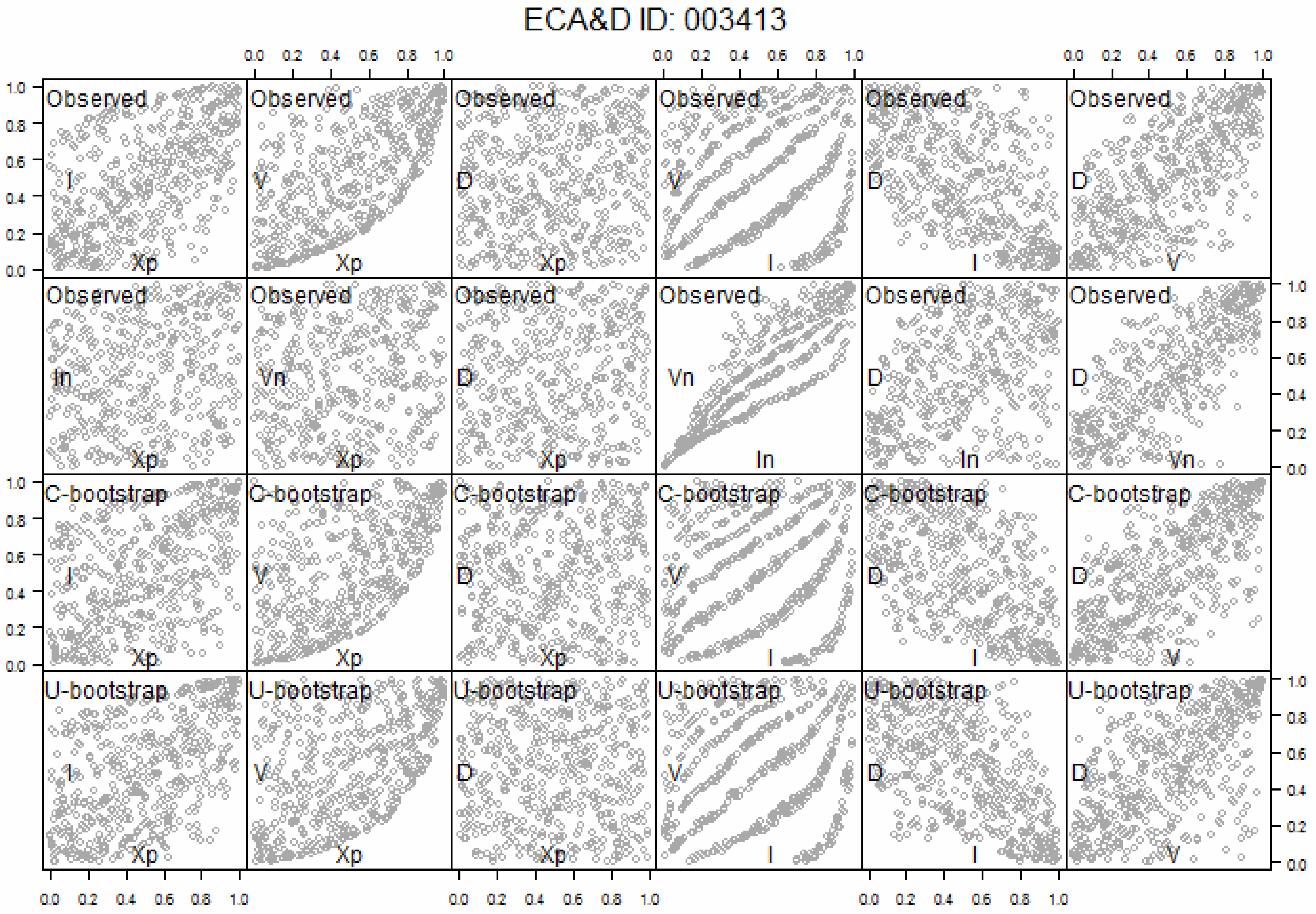
ECA&D ID: 003413
Observed
C-bootstrap
U-bootstrap
Xp
I
V
D
In
Vn
0.0
0.2
0.4
0.6
0.8
1.0

ECA&D ID: 003414

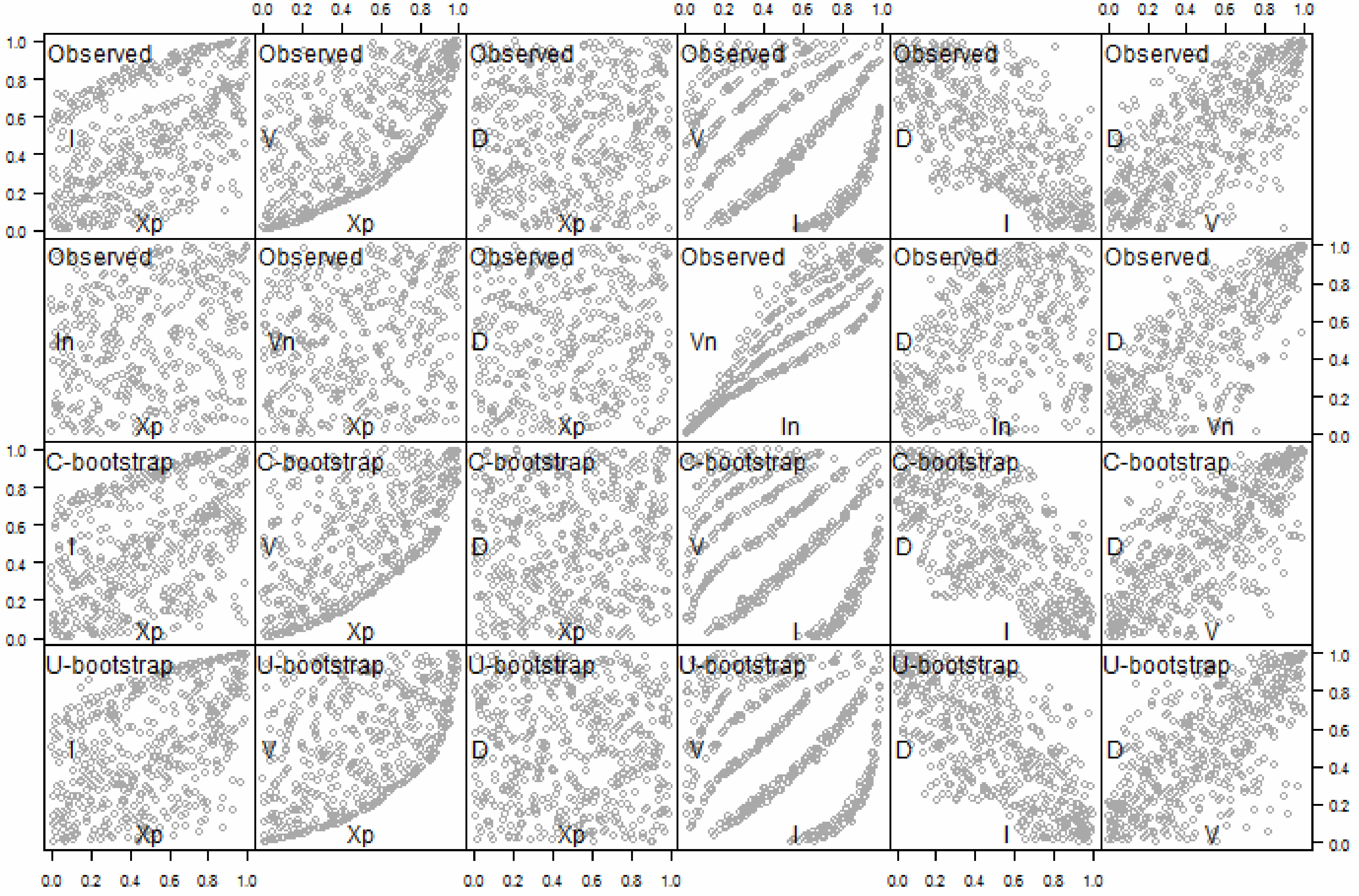

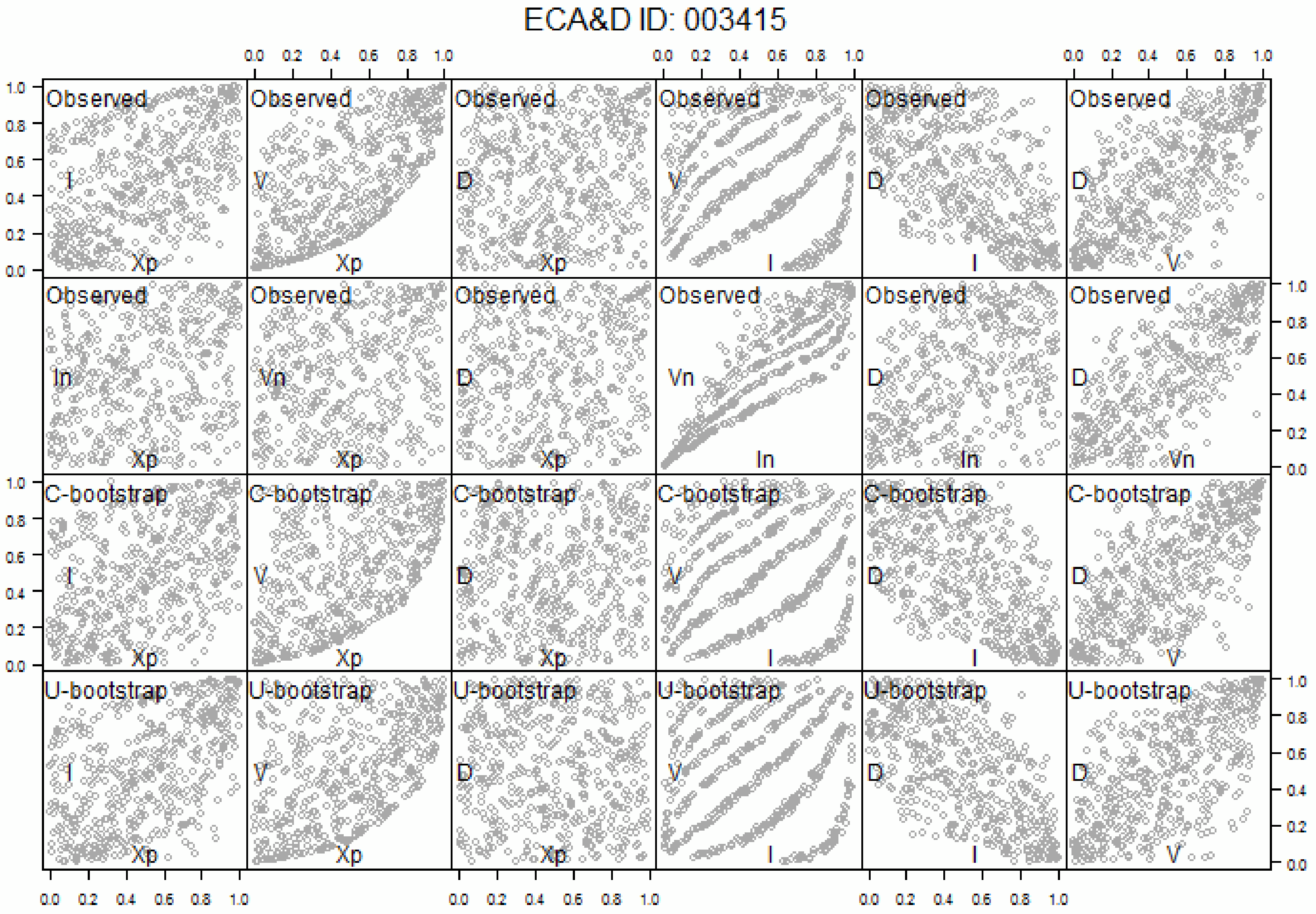
ECA&D ID: 003415
Observed
C-bootstrap
U-bootstrap
Xp
I
V
D
In
Vn
0.0
0.2
0.4
0.6
0.8
1.0

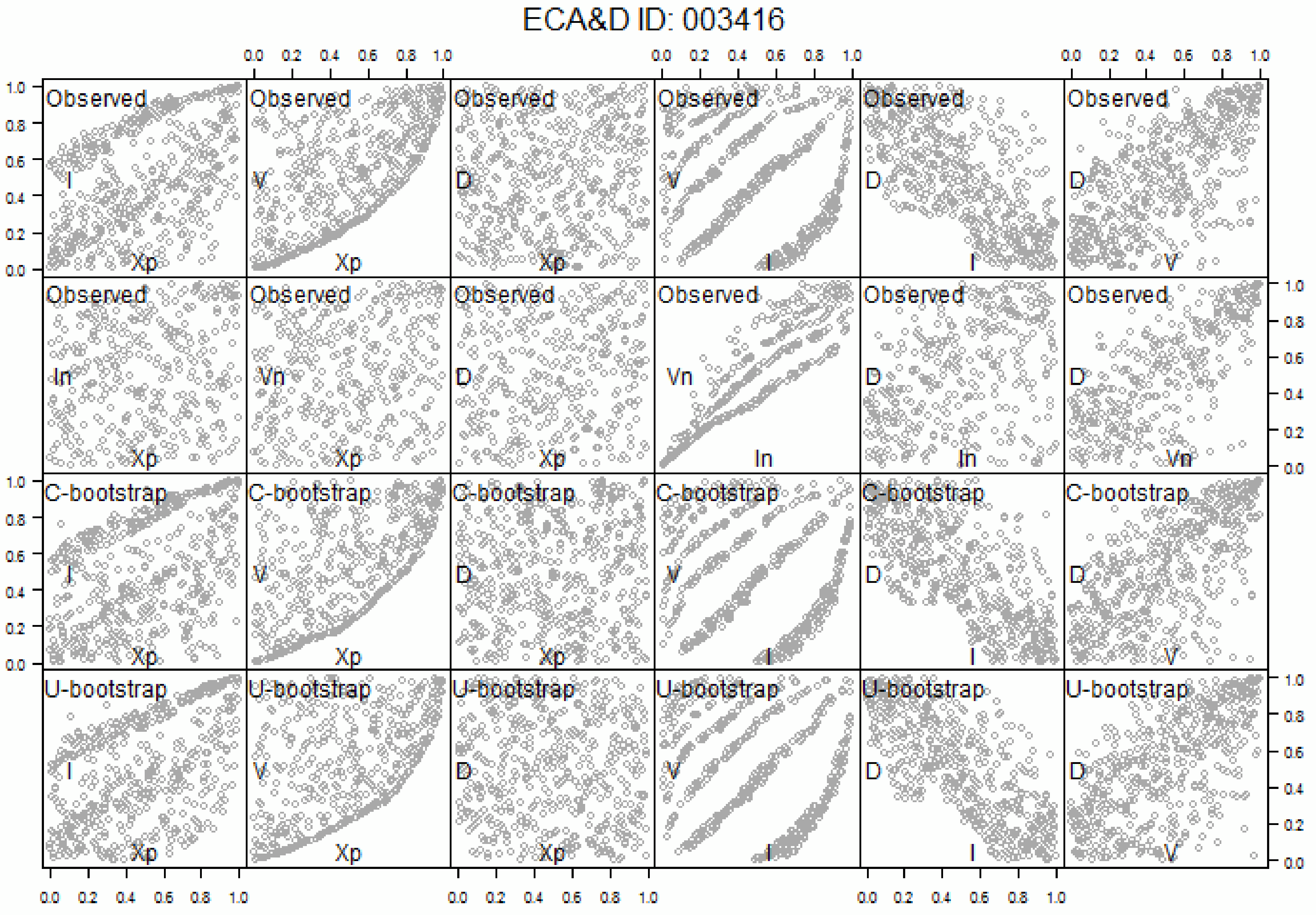
ECA&D ID: 003416
Observed
C-bootstrap
U-bootstrap
Xp
I
V
D
In
Vn
0.0
0.2
0.4
0.6
0.8
1.0

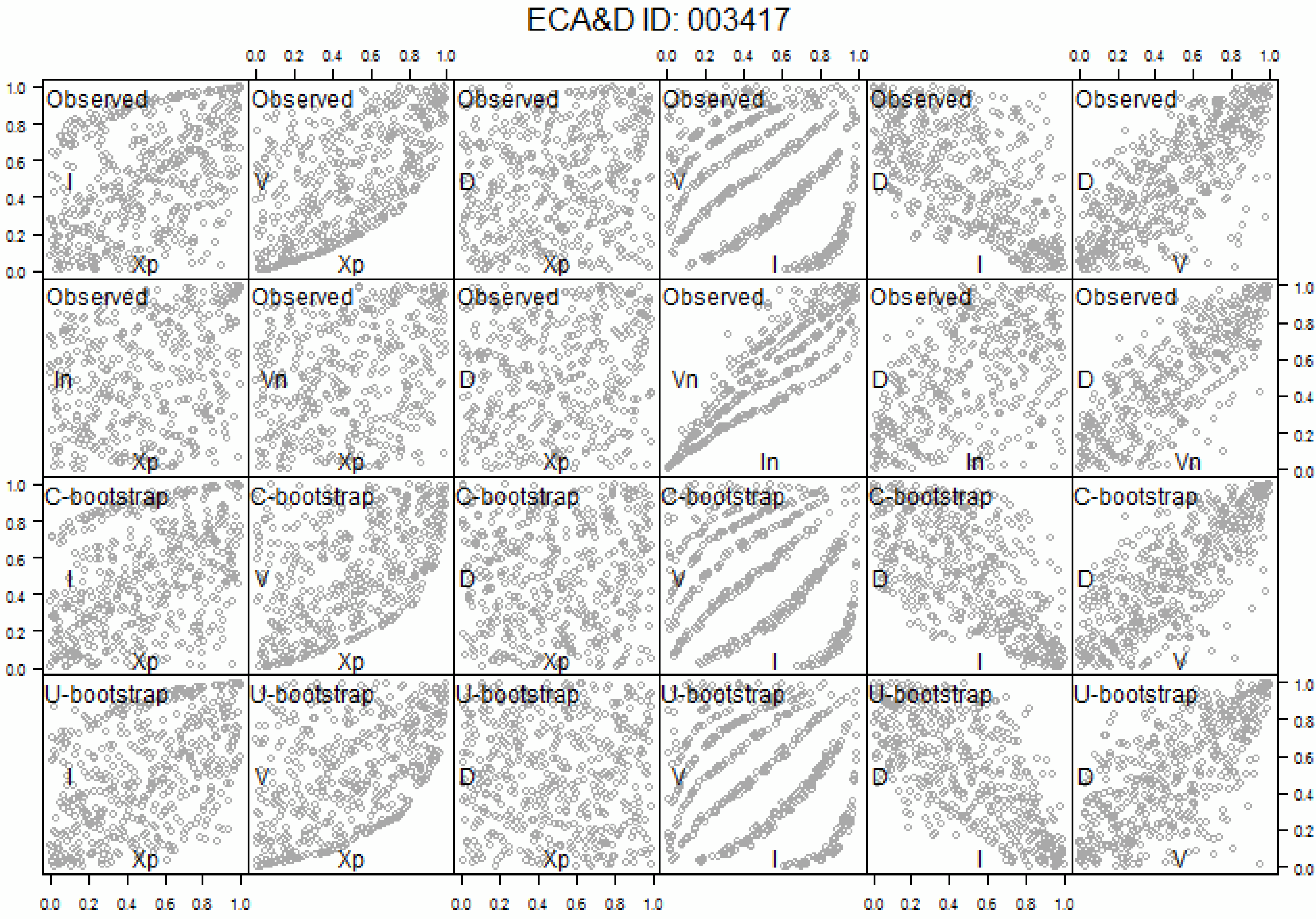
ECA&D ID: 003417
Observed
C-bootstrap
U-bootstrap
Xp
I
V
D
In
Vn
0.0
0.2
0.4
0.6
0.8
1.0

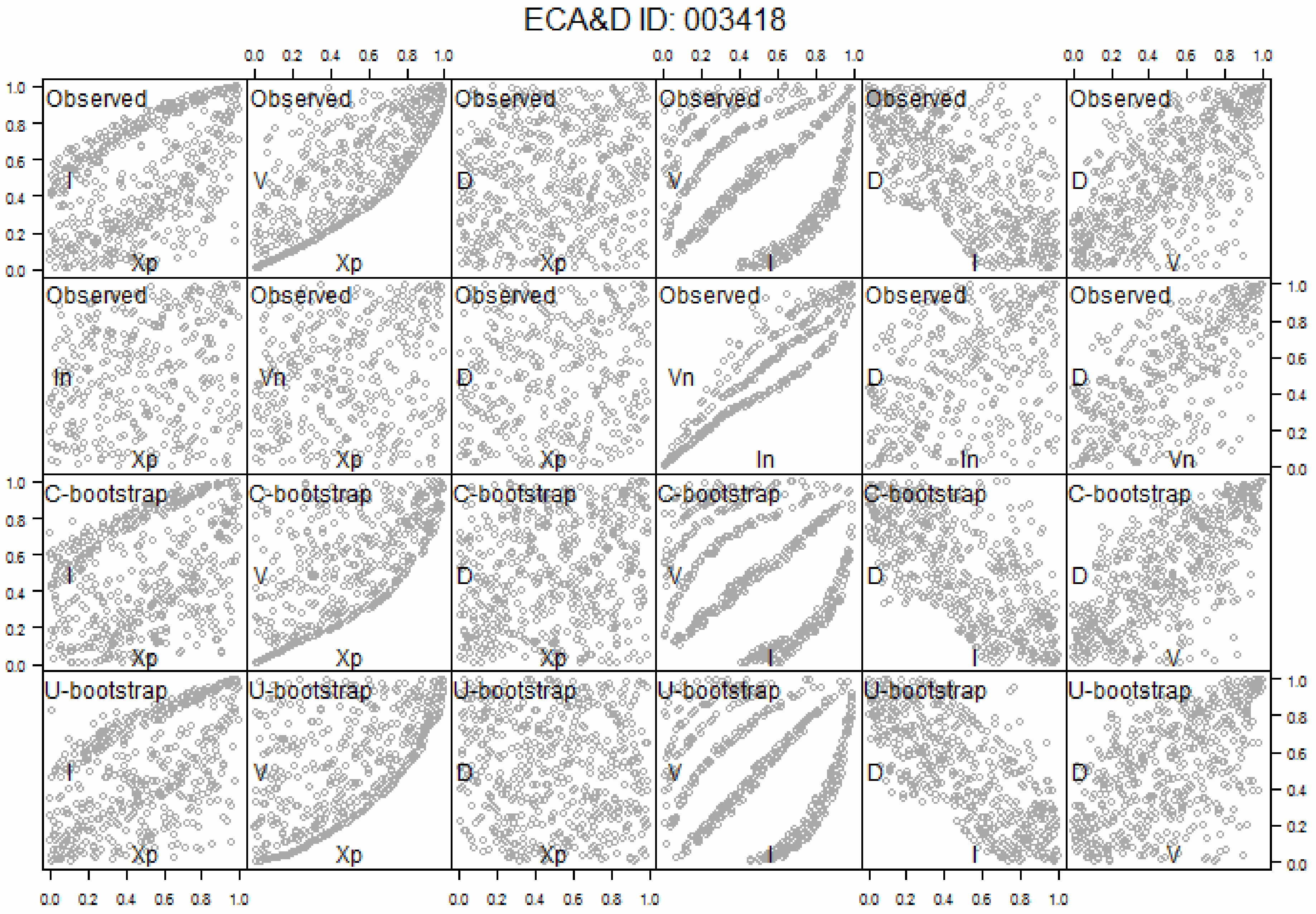
ECA&D ID: 003418
Observed
C-bootstrap
U-bootstrap
Xp
I
V
D
In
Vn
0.0
0.2
0.4
0.6
0.8
1.0

ECA&D ID: 003419

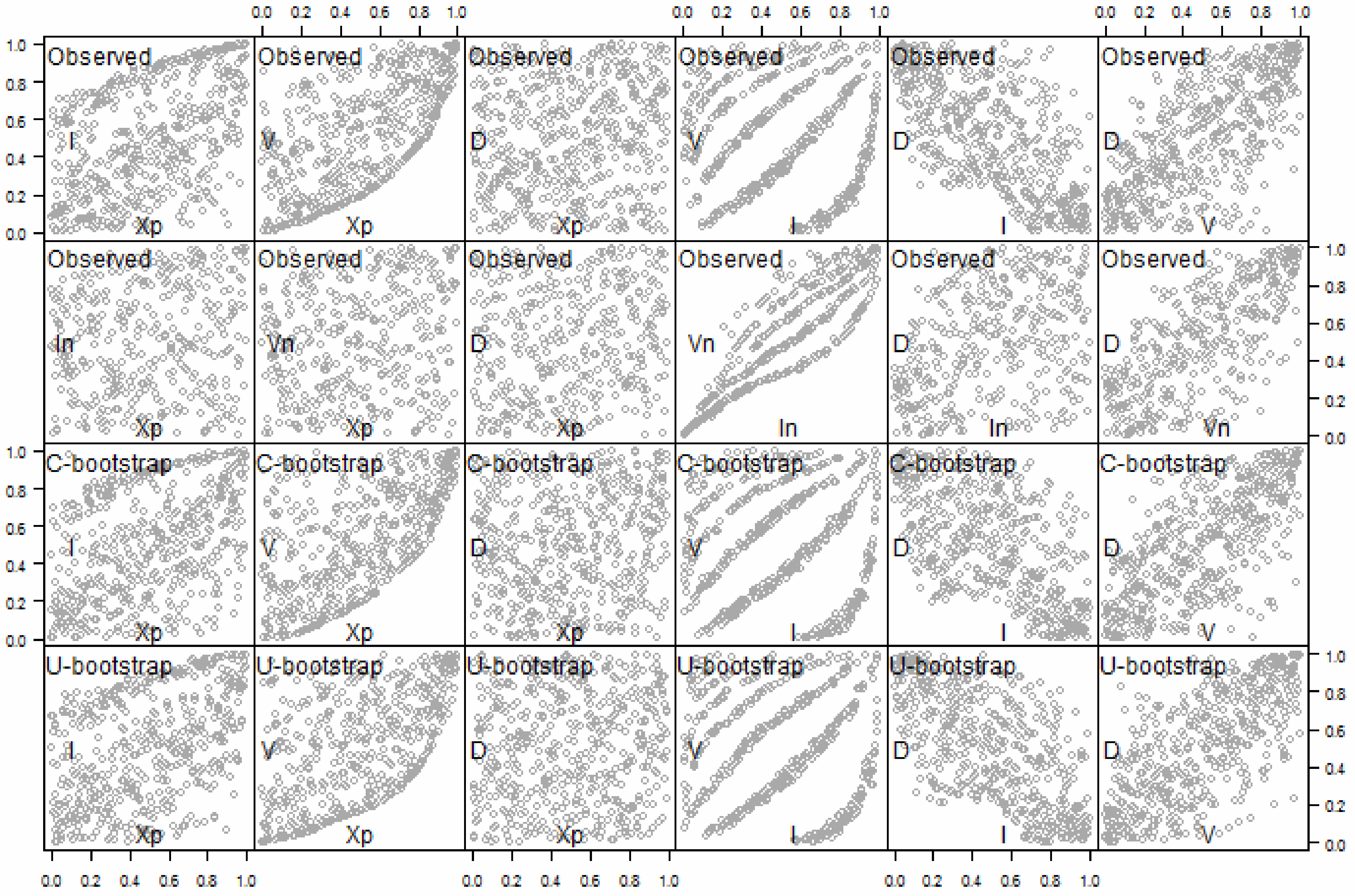

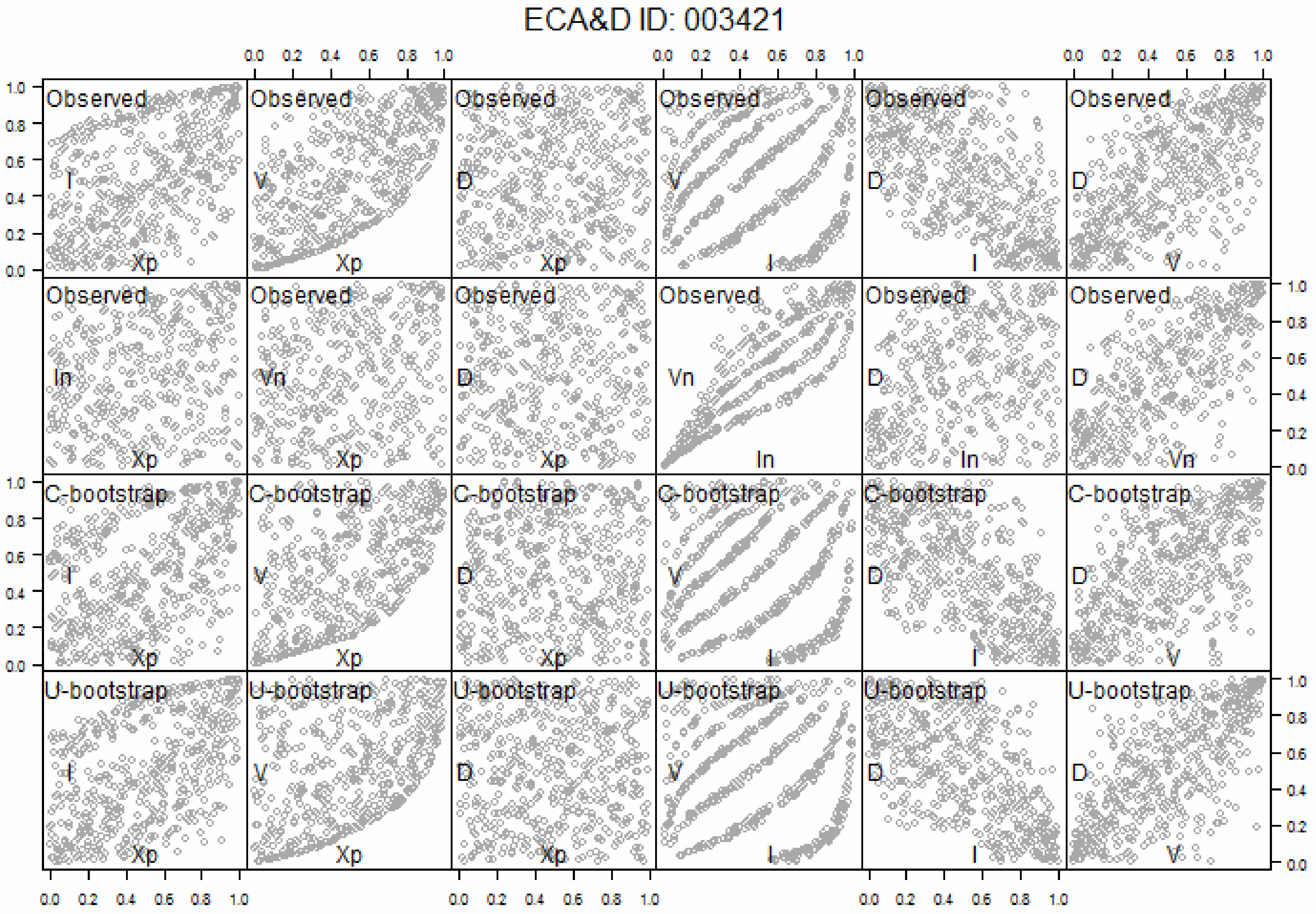
ECA&D ID: 003421
Observed
C-bootstrap
U-bootstrap
Xp
I
V
D
In
Vn
0.0
0.2
0.4
0.6
0.8
1.0

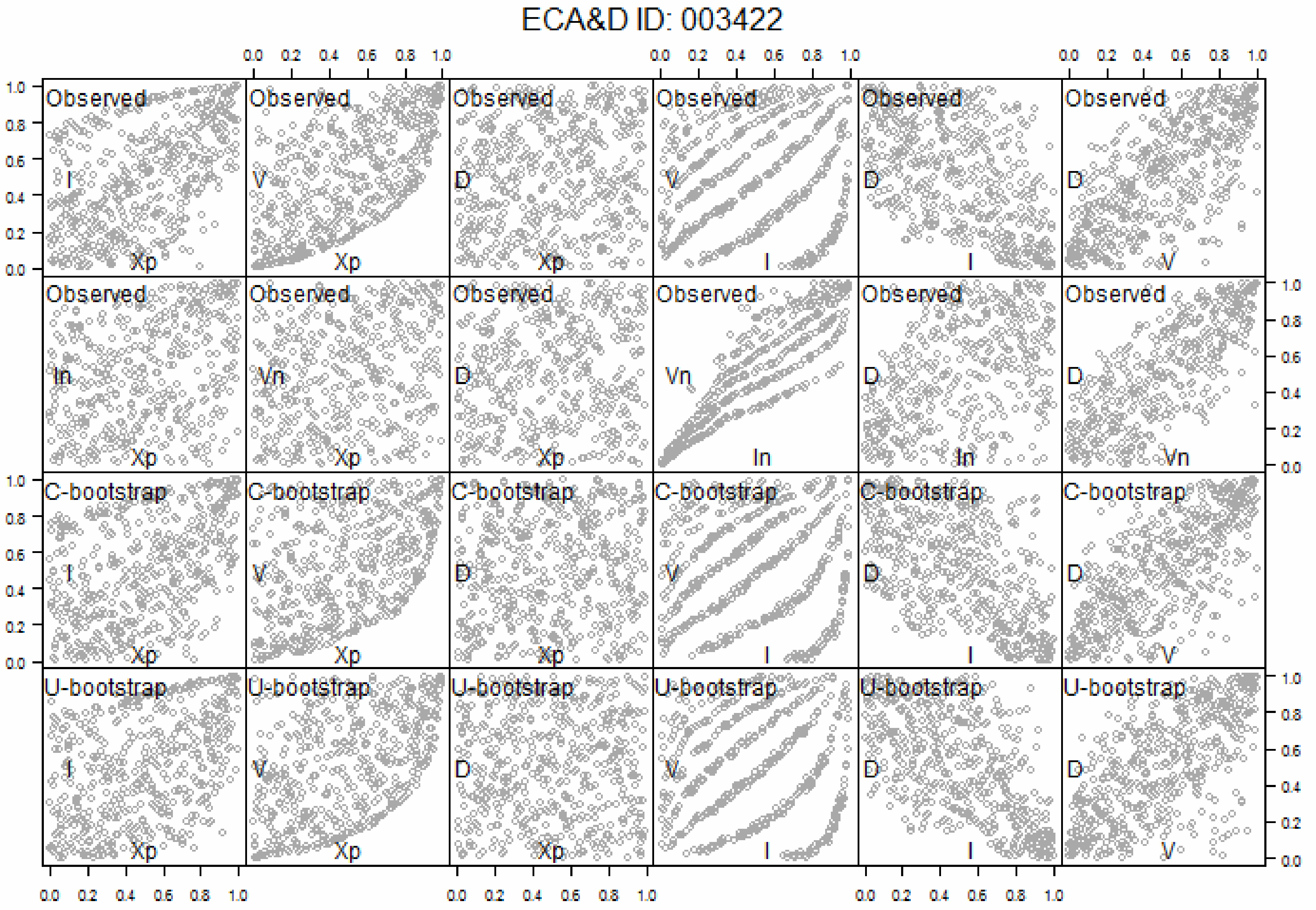
ECA&D ID: 003422
Observed
C-bootstrap
U-bootstrap
Xp
I
V
D
In
Vn
0.0
0.2
0.4
0.6
0.8
1.0

ECA&D ID: 003423

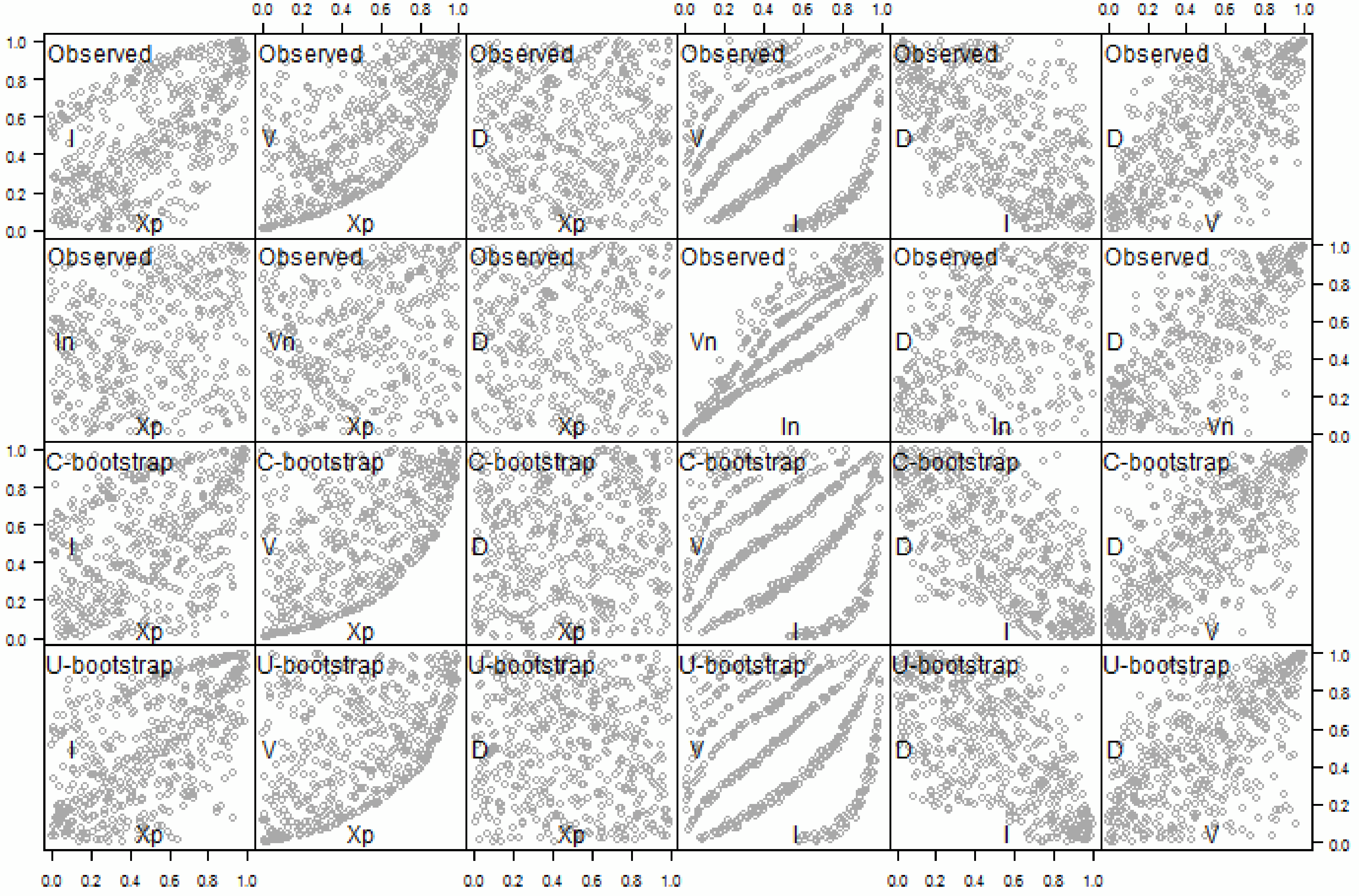

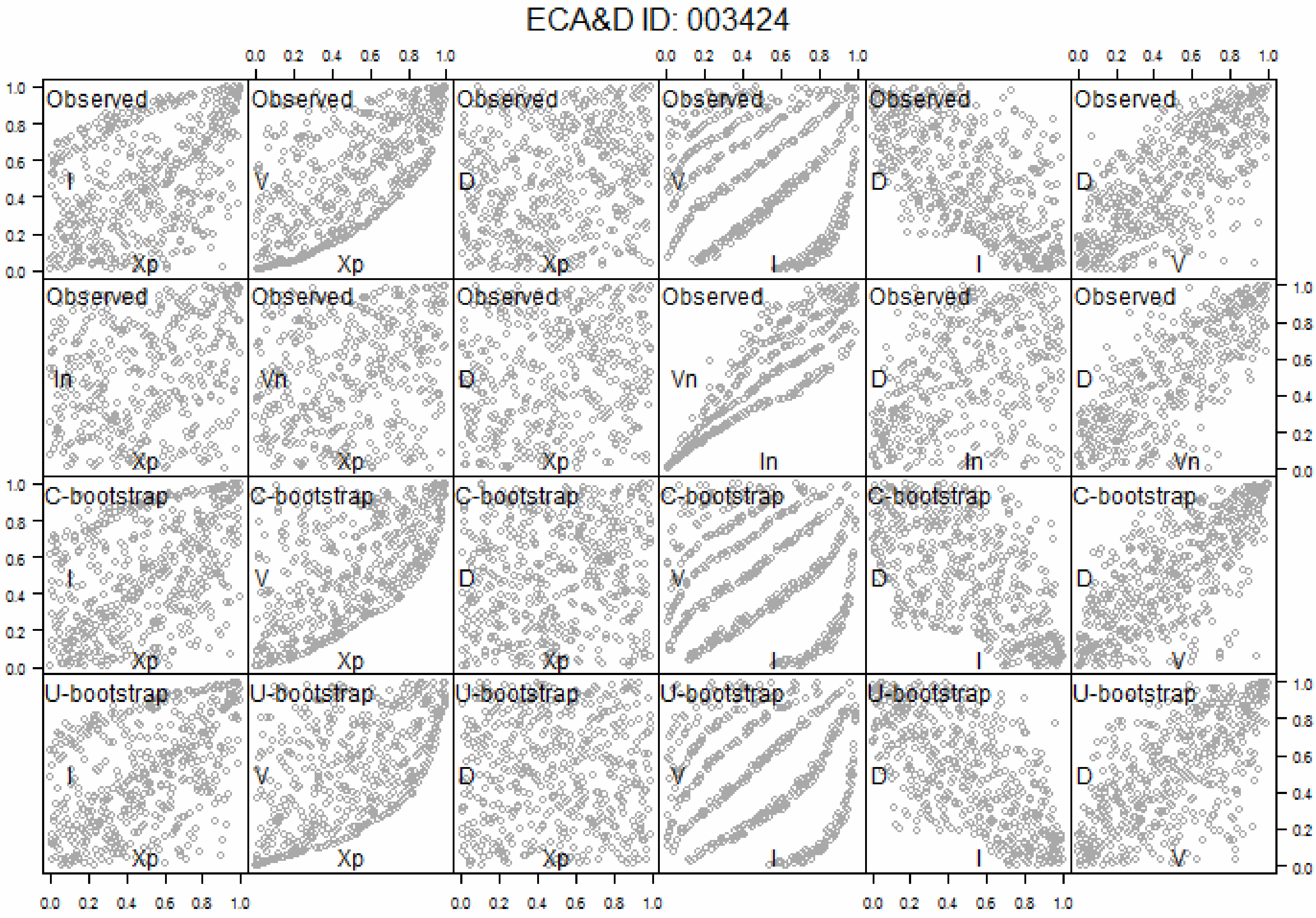
ECA&D ID: 003424
Observed
C-bootstrap
U-bootstrap
Xp
I
V
D
In
Vn
0.0
0.2
0.4
0.6
0.8
1.0

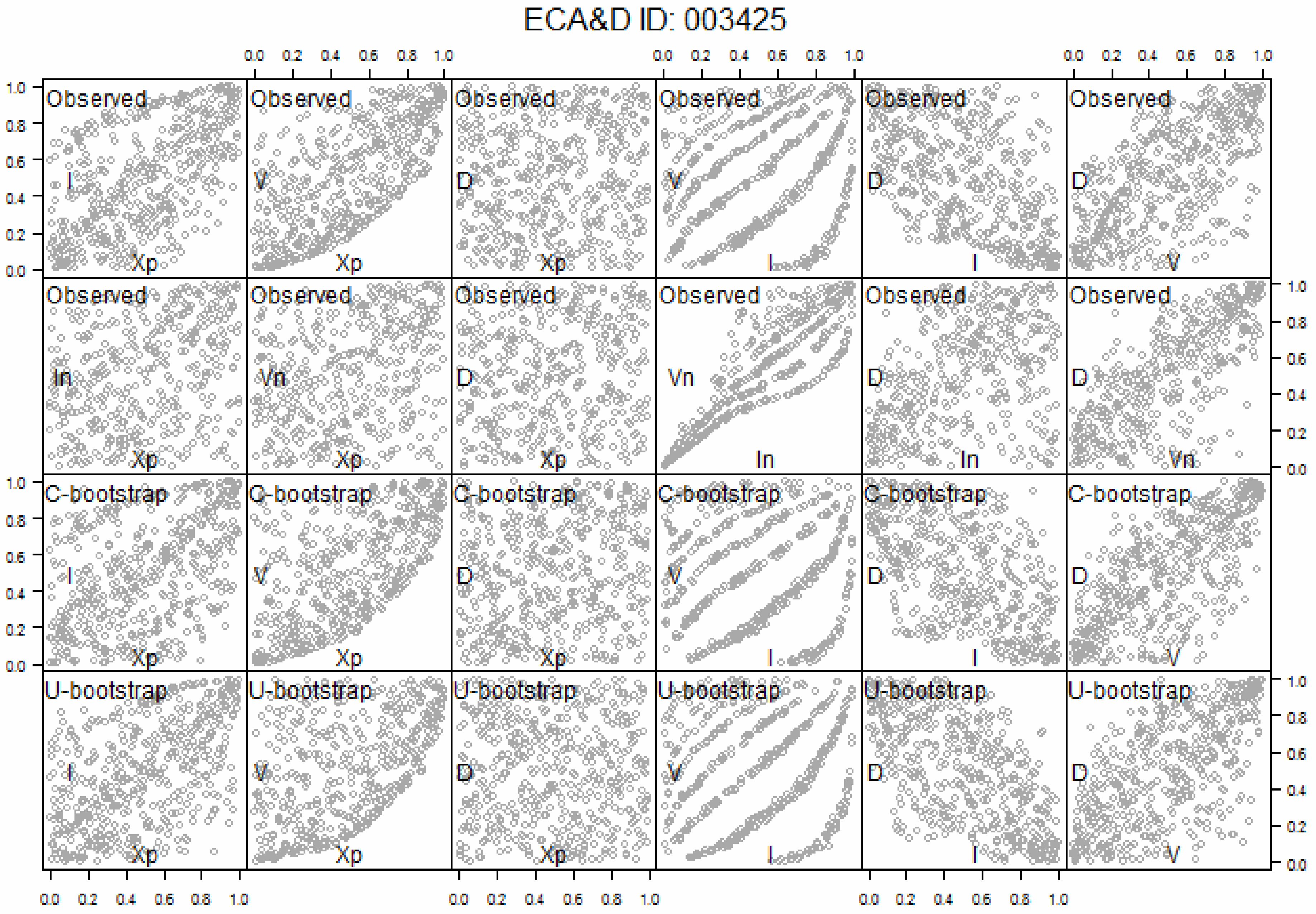
ECA&D ID: 003425
Observed
C-bootstrap
U-bootstrap
Xp
I
V
D
In
Vn
0.0
0.2
0.4
0.6
0.8
1.0

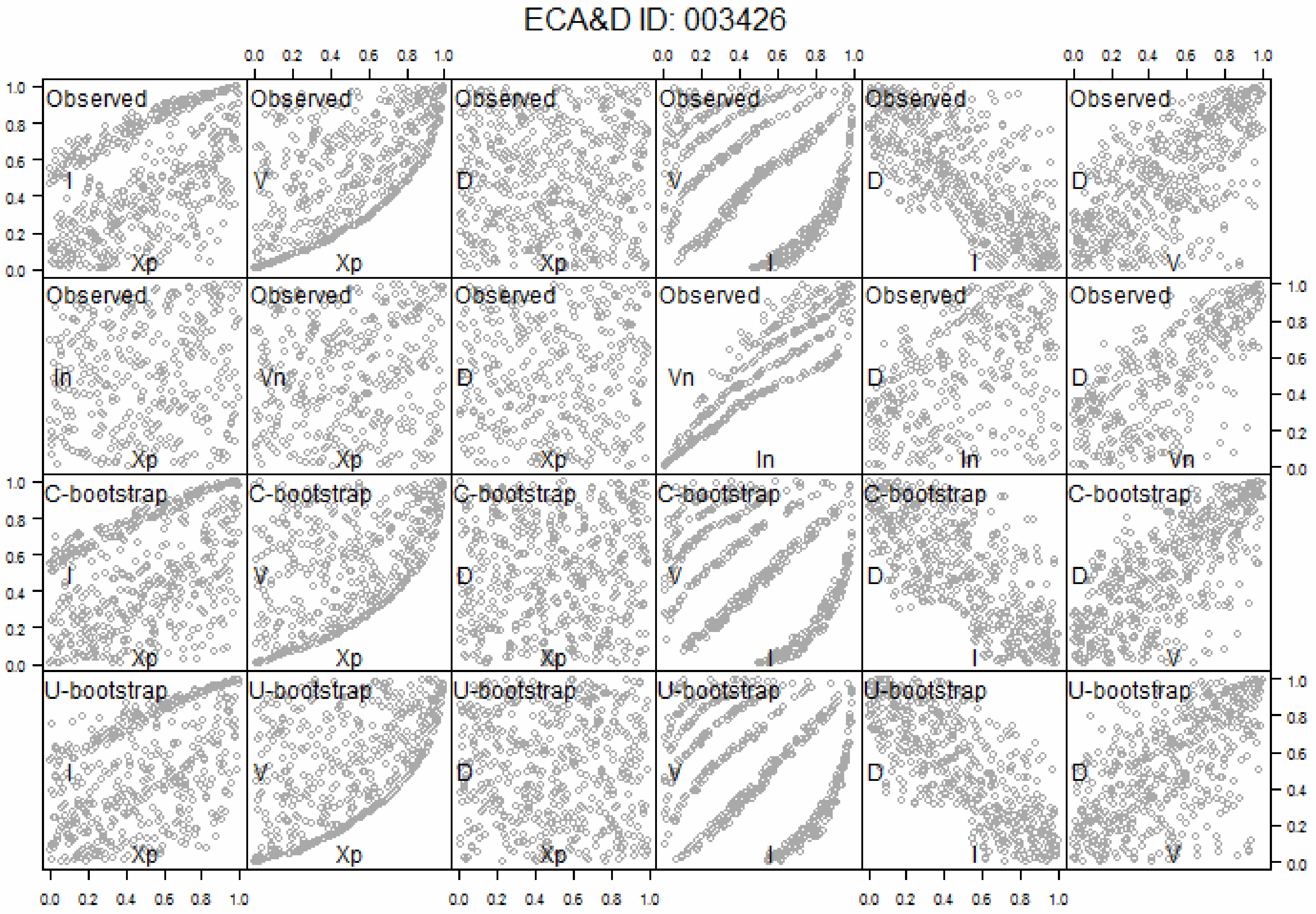

ECA&D ID: 003426
Observed
C-bootstrap
U-bootstrap
Xp
I
V
D
In
Vn
0.0
0.2
0.4
0.6
0.8
1.0

ECA&D ID: 003427

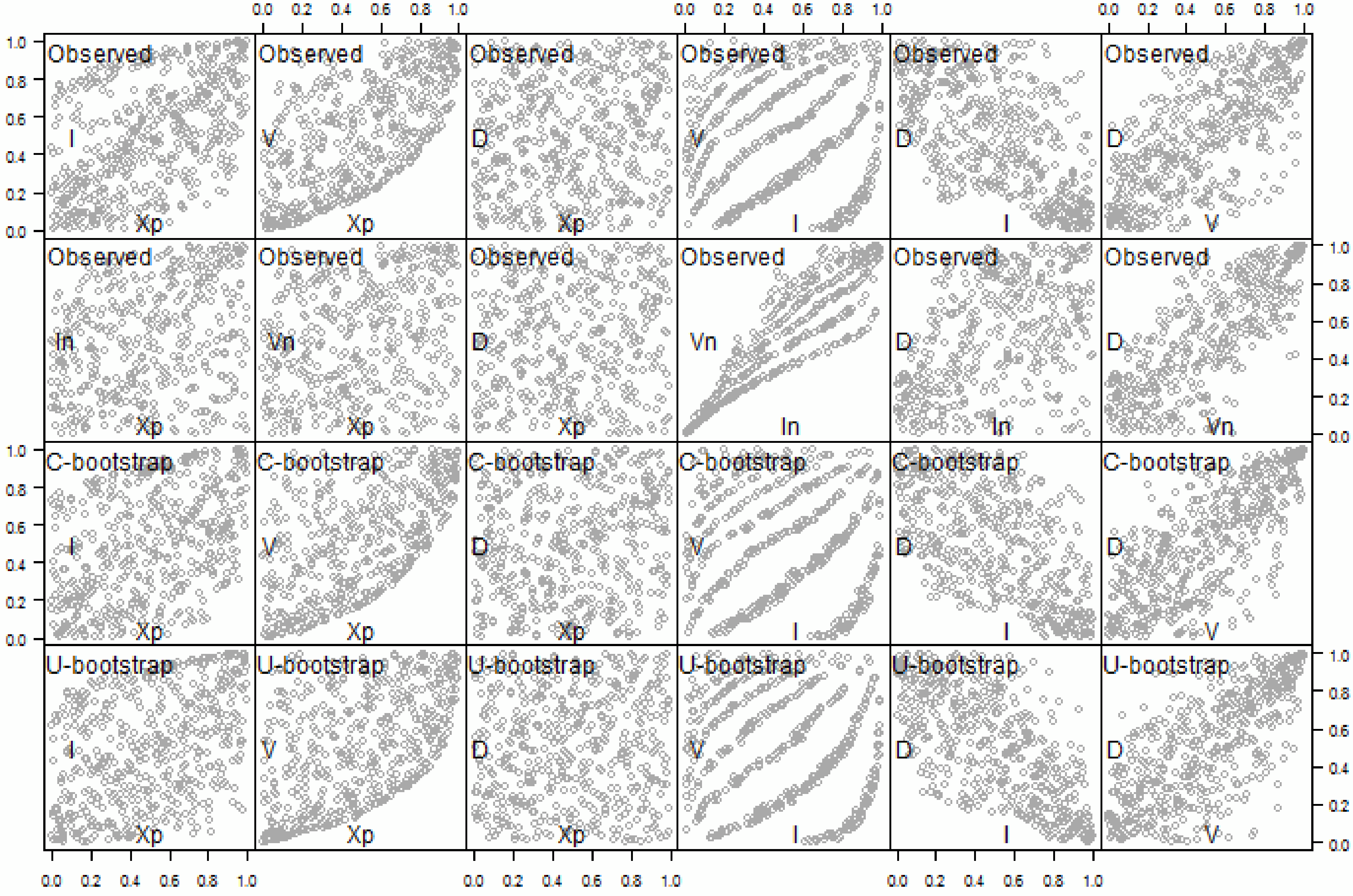

ECA&D ID: 003428

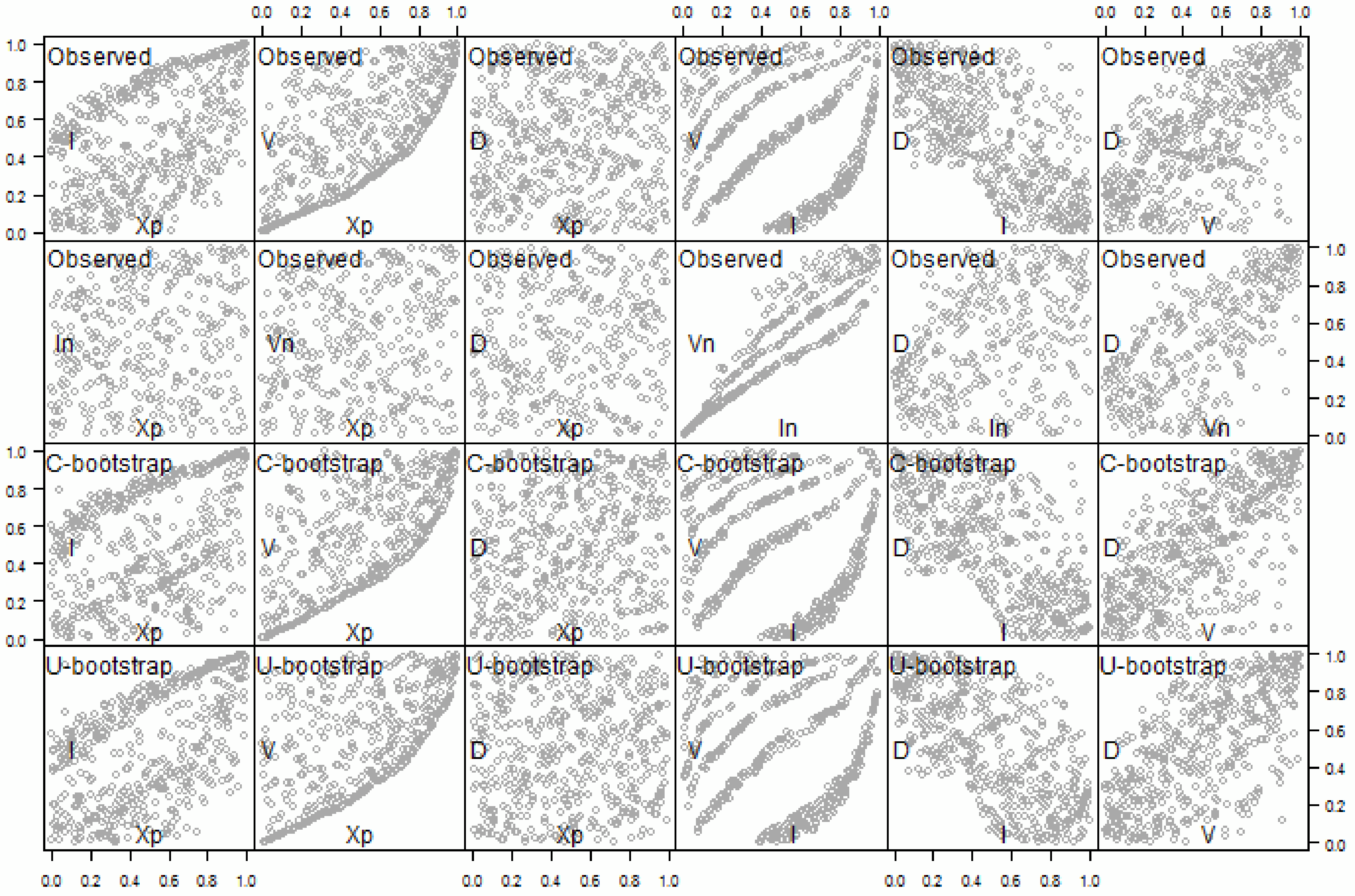

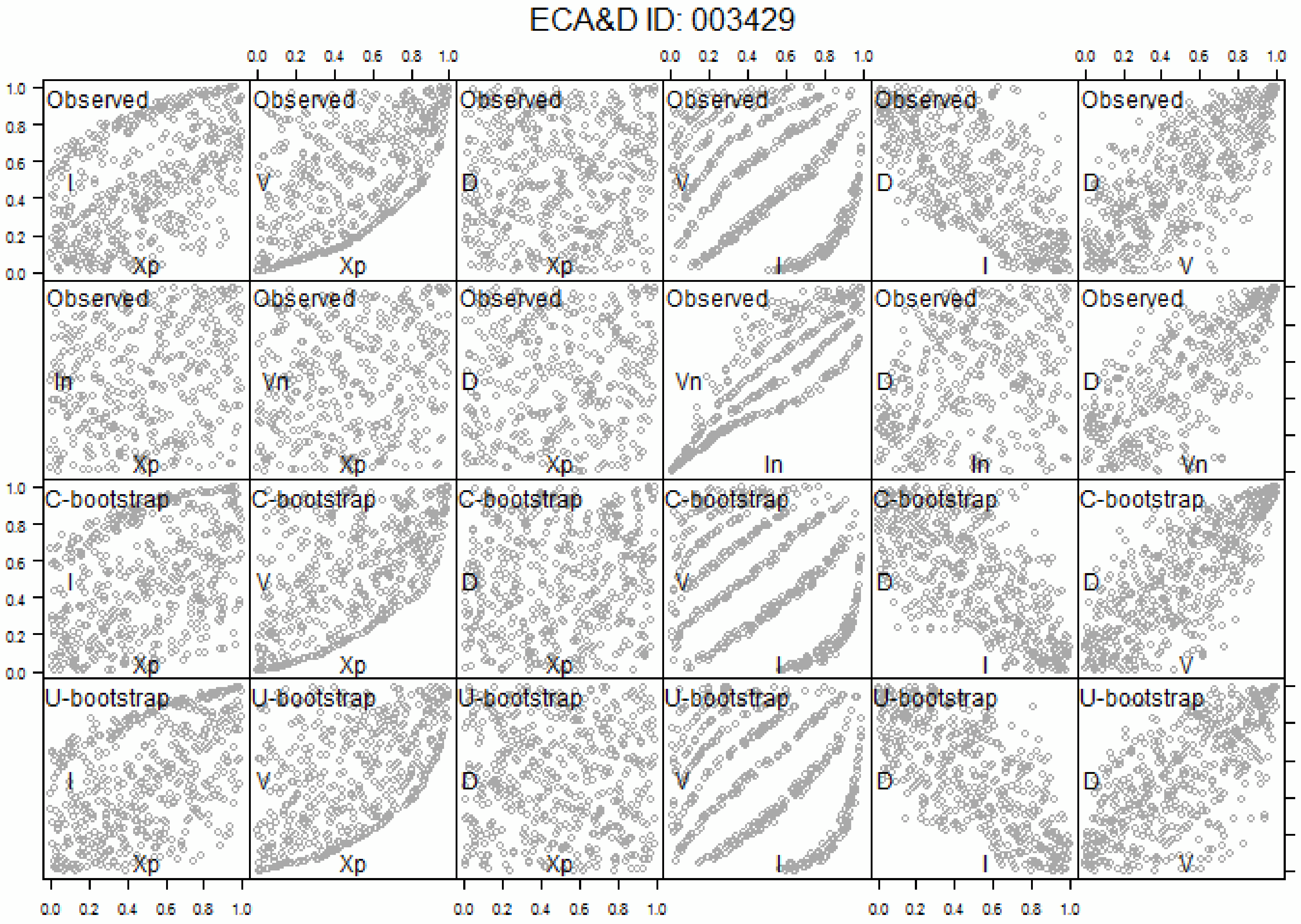

ECA&D ID: 003429
Observed
C-bootstrap
U-bootstrap
Xp
I
V
D
In
Vn
0.0
0.2
0.4
0.6
0.8
1.0

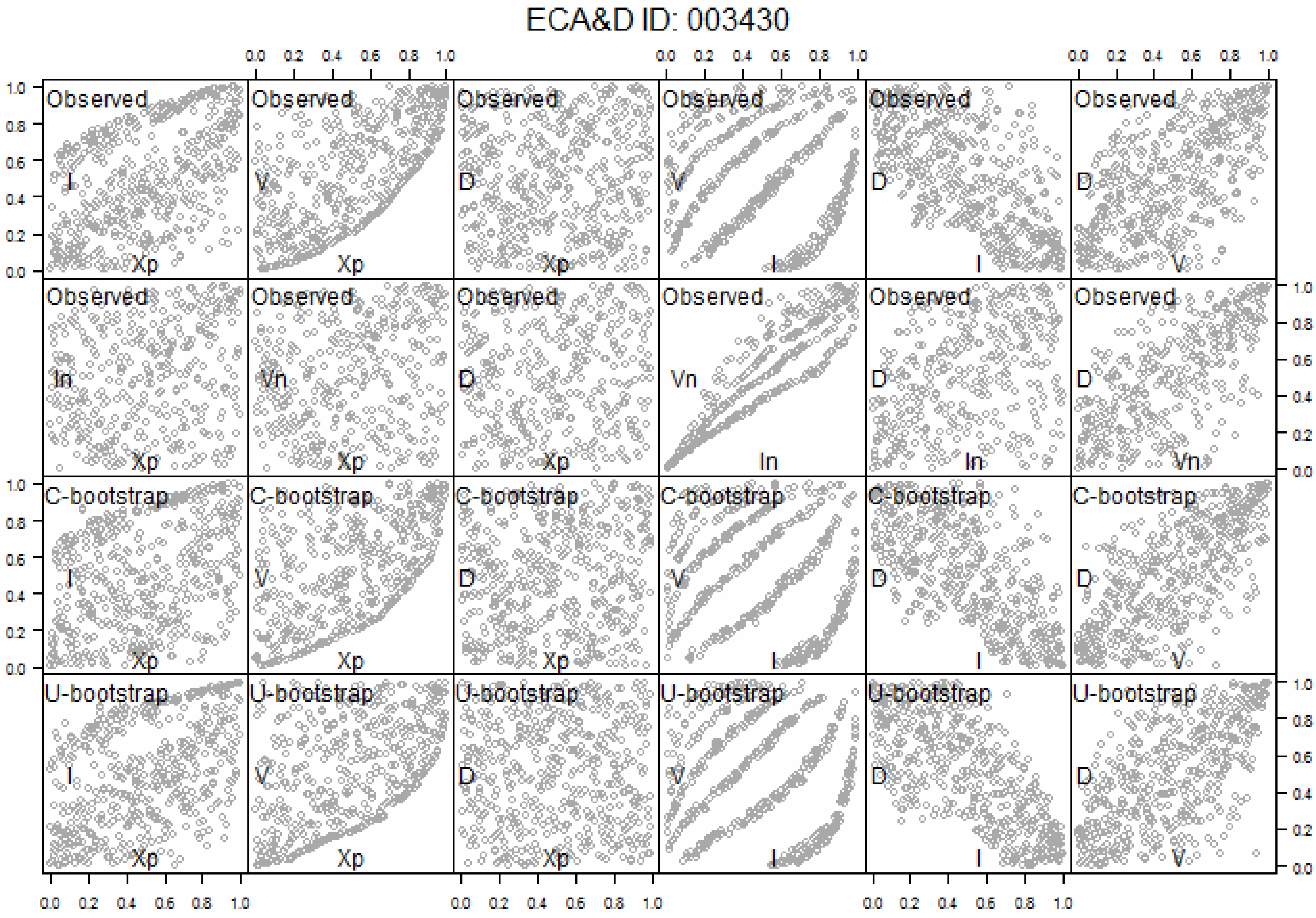
ECA&D ID: 003430
Observed
C-bootstrap
U-bootstrap
Xp
I
V
D
In
Vn
0.0
0.2
0.4
0.6
0.8
1.0

ECA&D ID: 003431

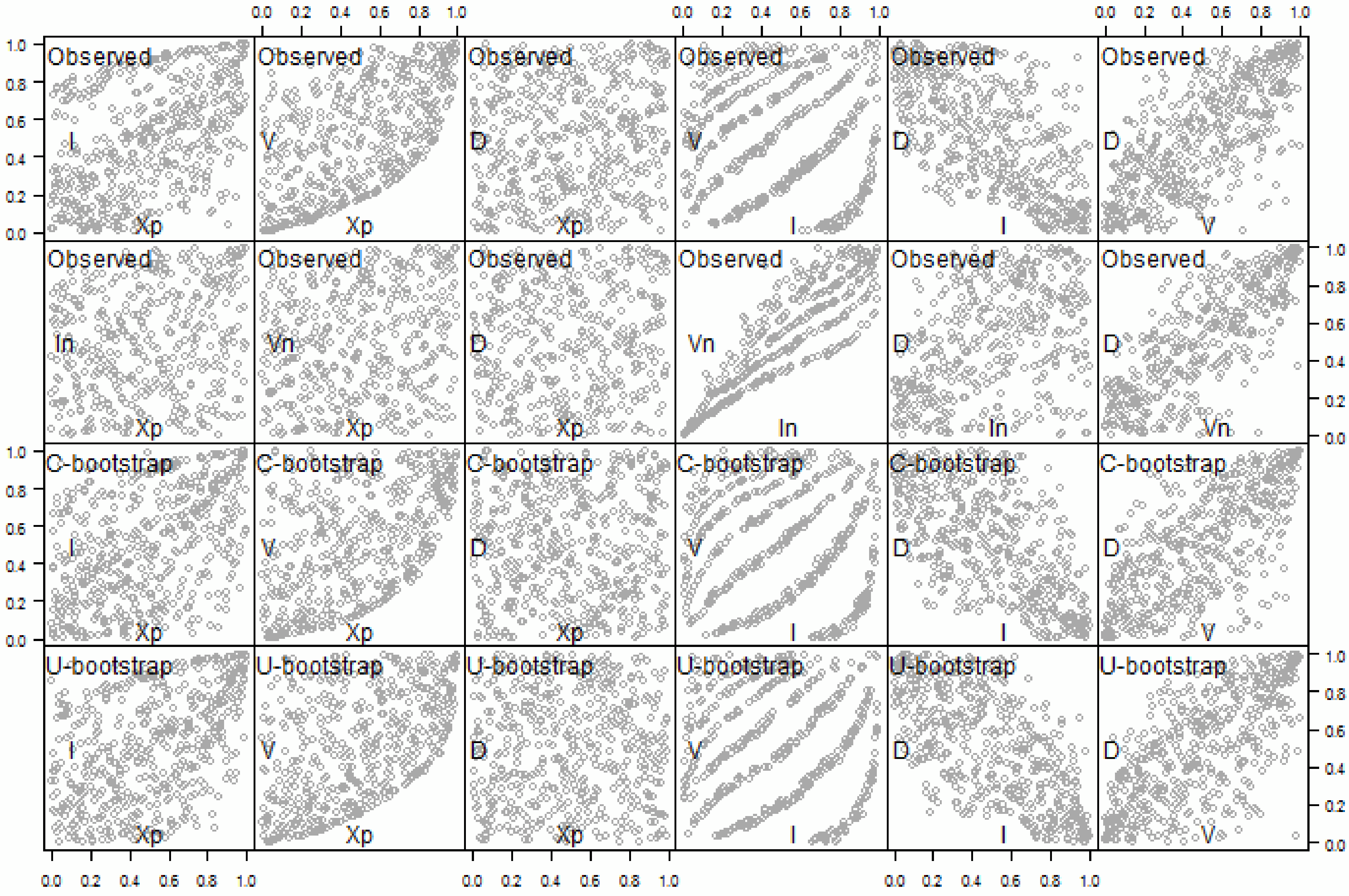

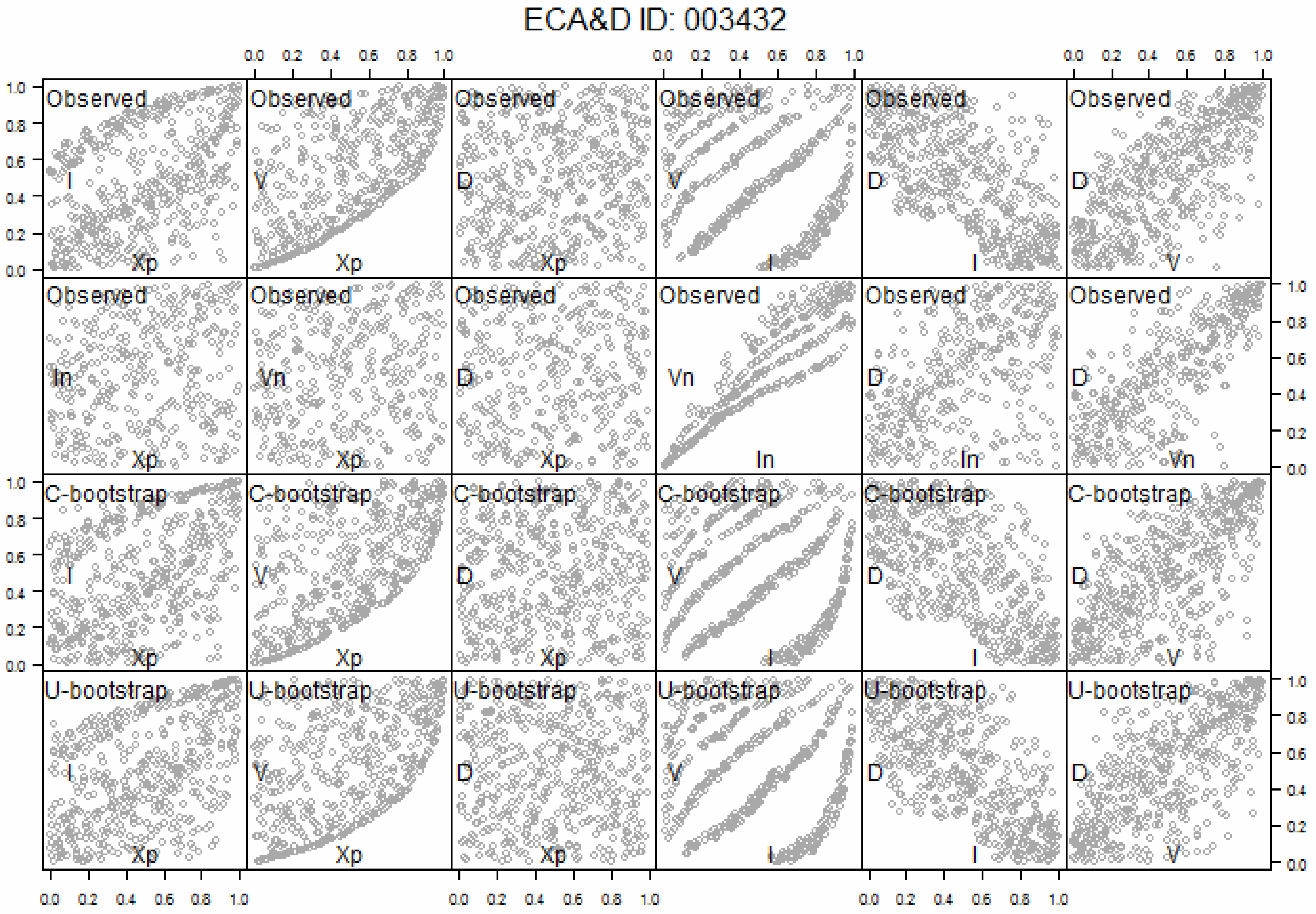
ECA&D ID: 003432
Observed
I
Xp
V
D
In
Vn
C-bootstrap
U-bootstrap
0.0
0.2
0.4
0.6
0.8
1.0

ECA&D ID: 003433

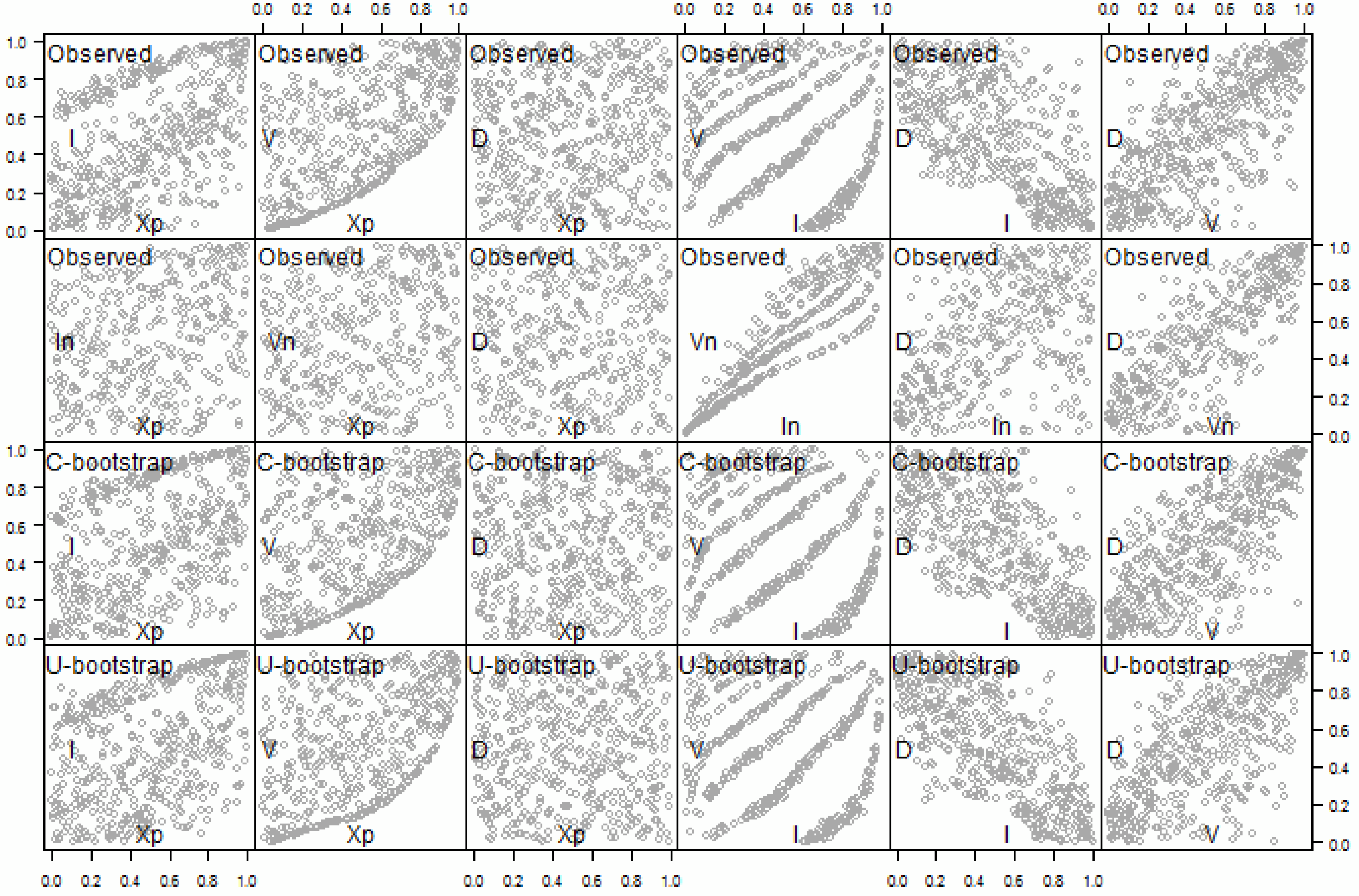

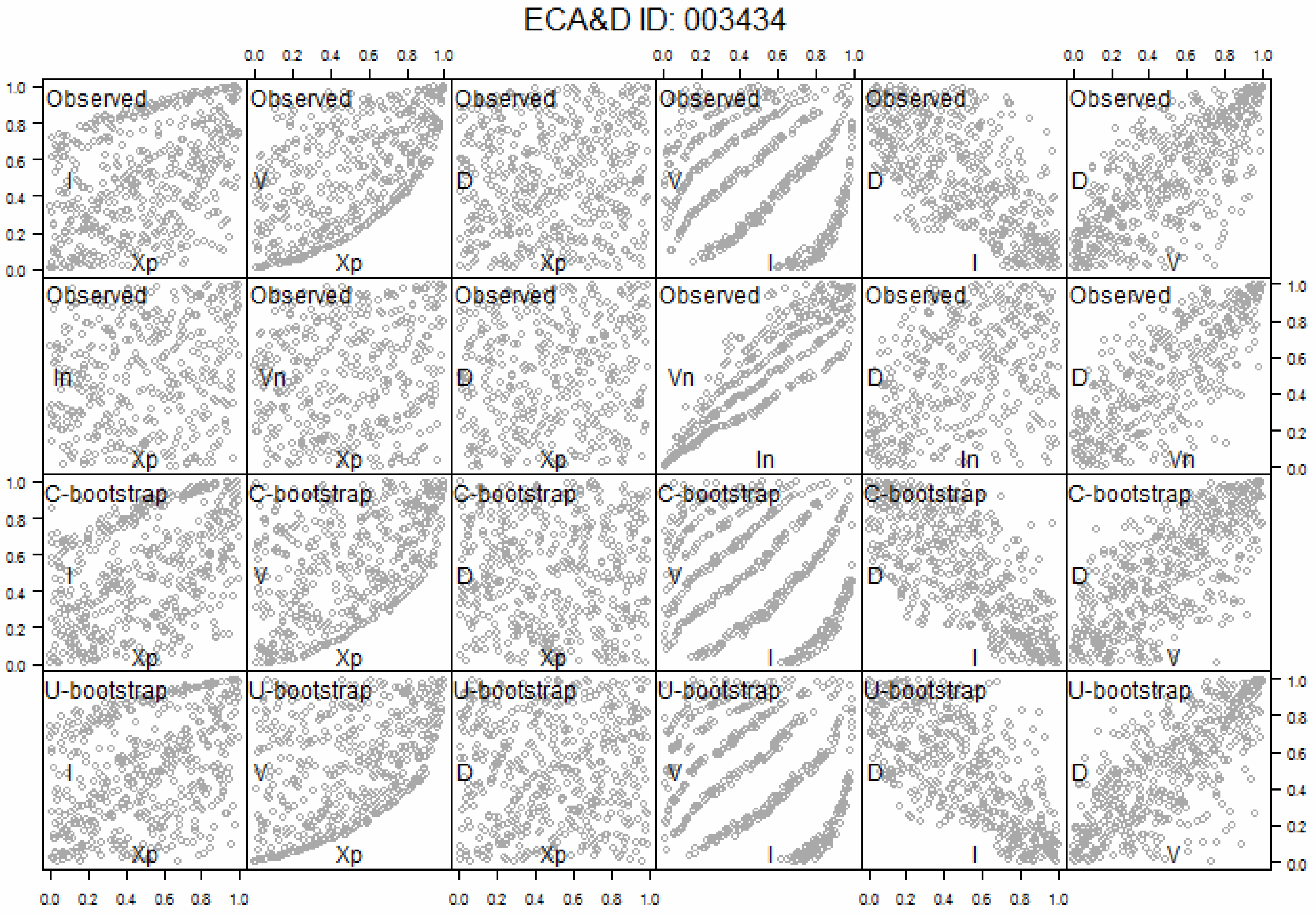
ECA&D ID: 003434
Observed
C-bootstrap
U-bootstrap
Xp
I
V
D
In
Vn
0.0
0.2
0.4
0.6
0.8
1.0

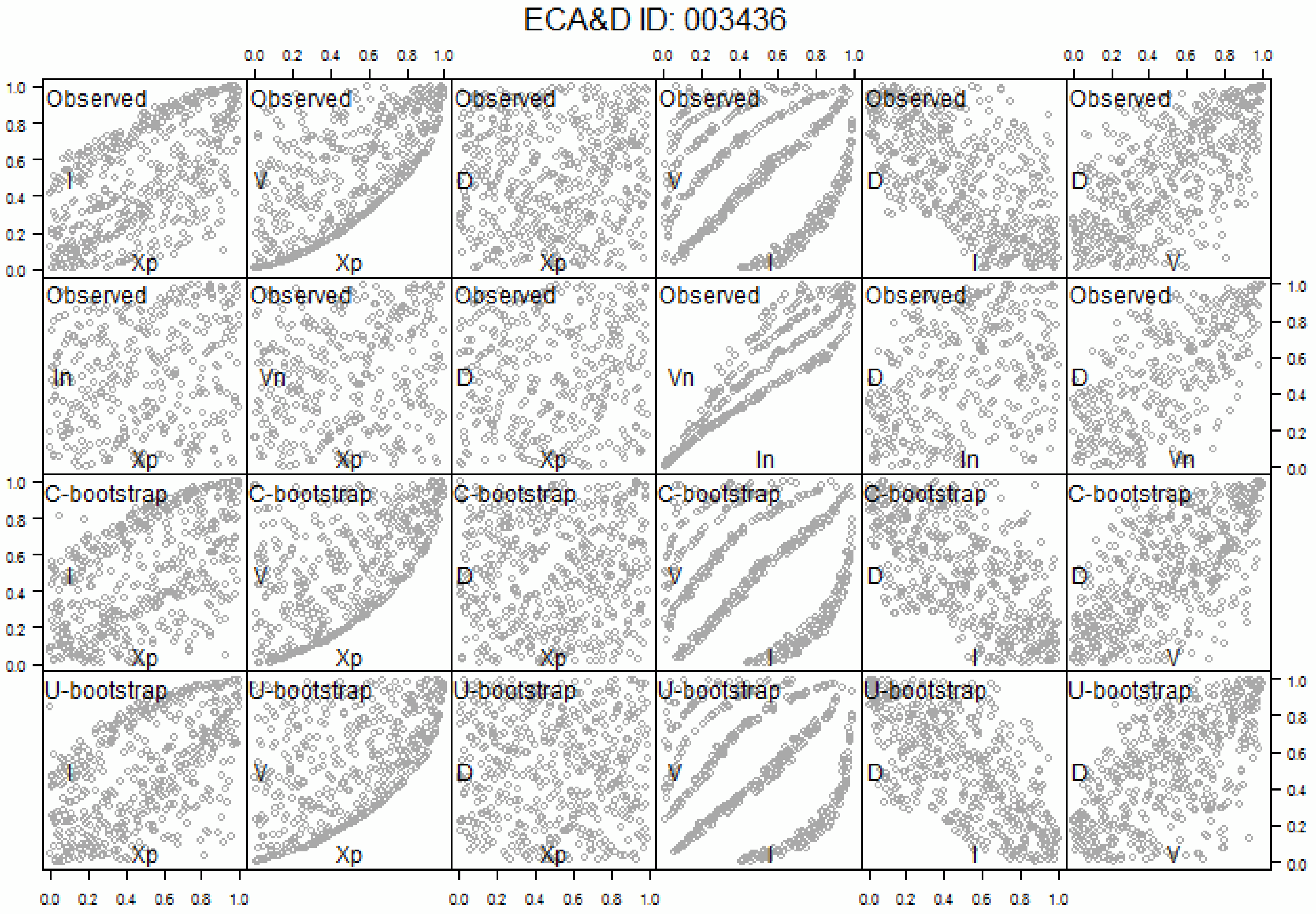
ECA&D ID: 003436
Observed
C-bootstrap
U-bootstrap
Xp
I
V
D
In
Vn

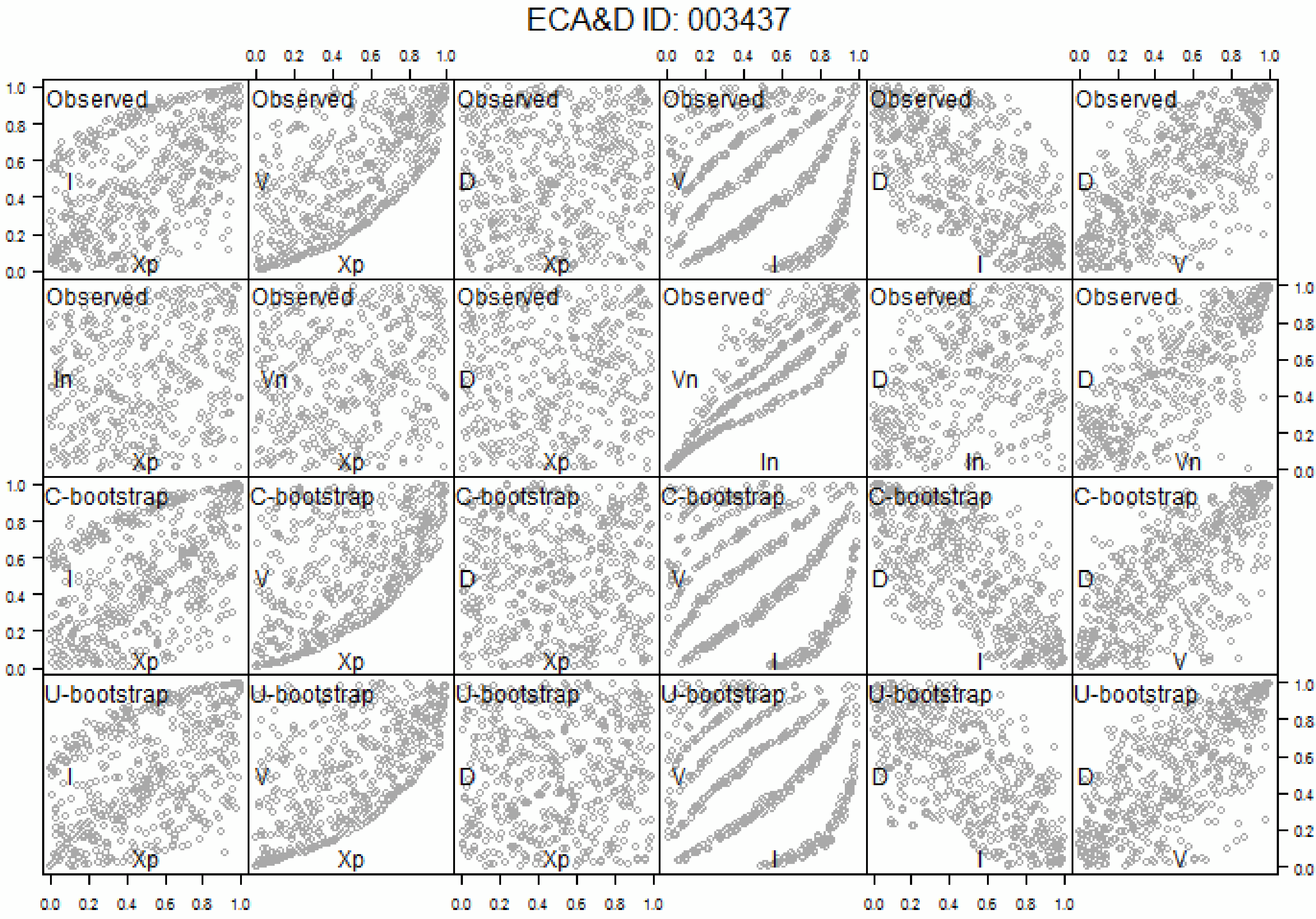
ECA&D ID: 003437
Observed
C-bootstrap
U-bootstrap
Xp
I
V
D
In
Vn
0.0
0.2
0.4
0.6
0.8
1.0

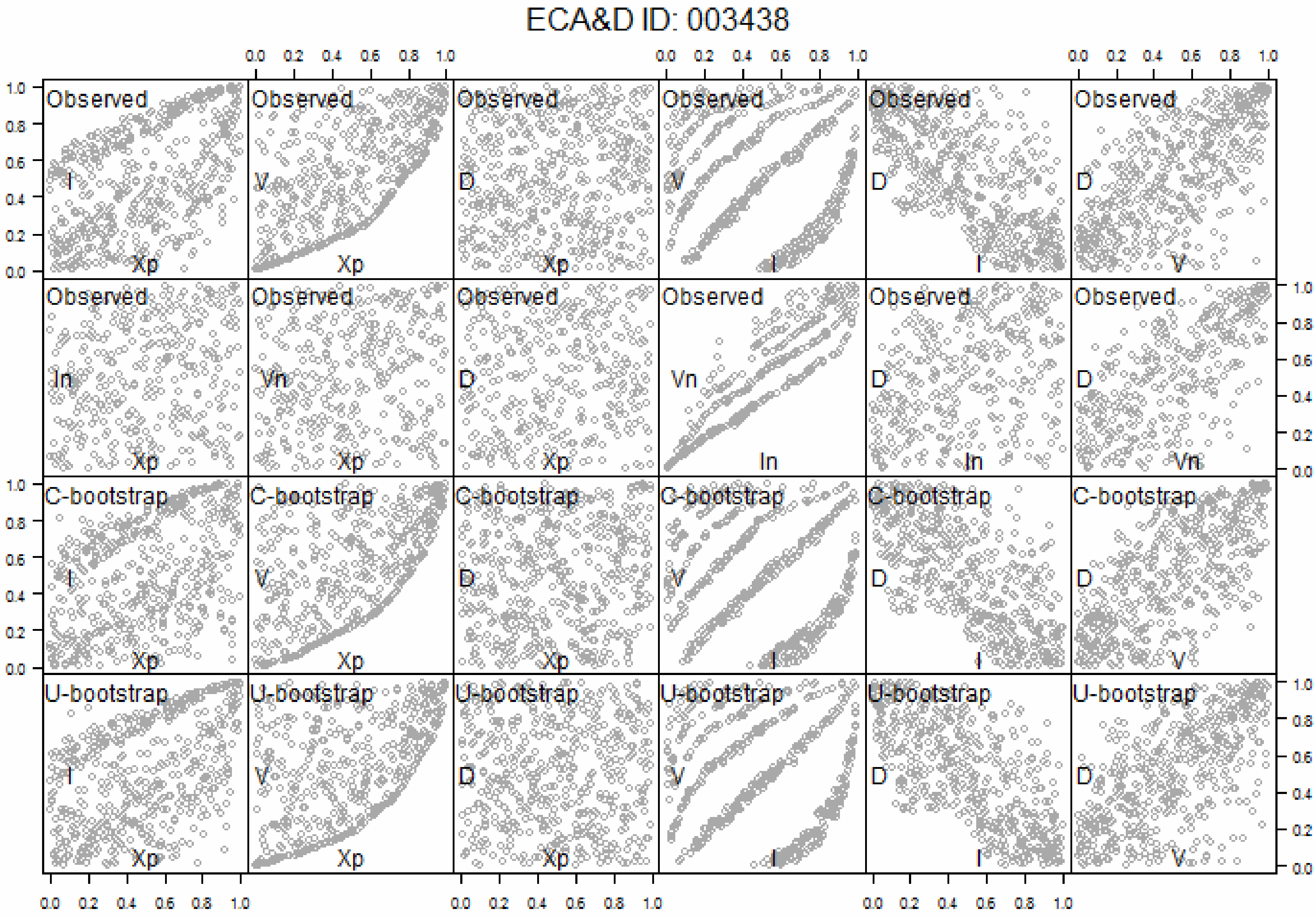
ECA&D ID: 003438
Observed
C-bootstrap
U-bootstrap
Xp
I
V
D
In
Vn
0.0
0.2
0.4
0.6
0.8
1.0

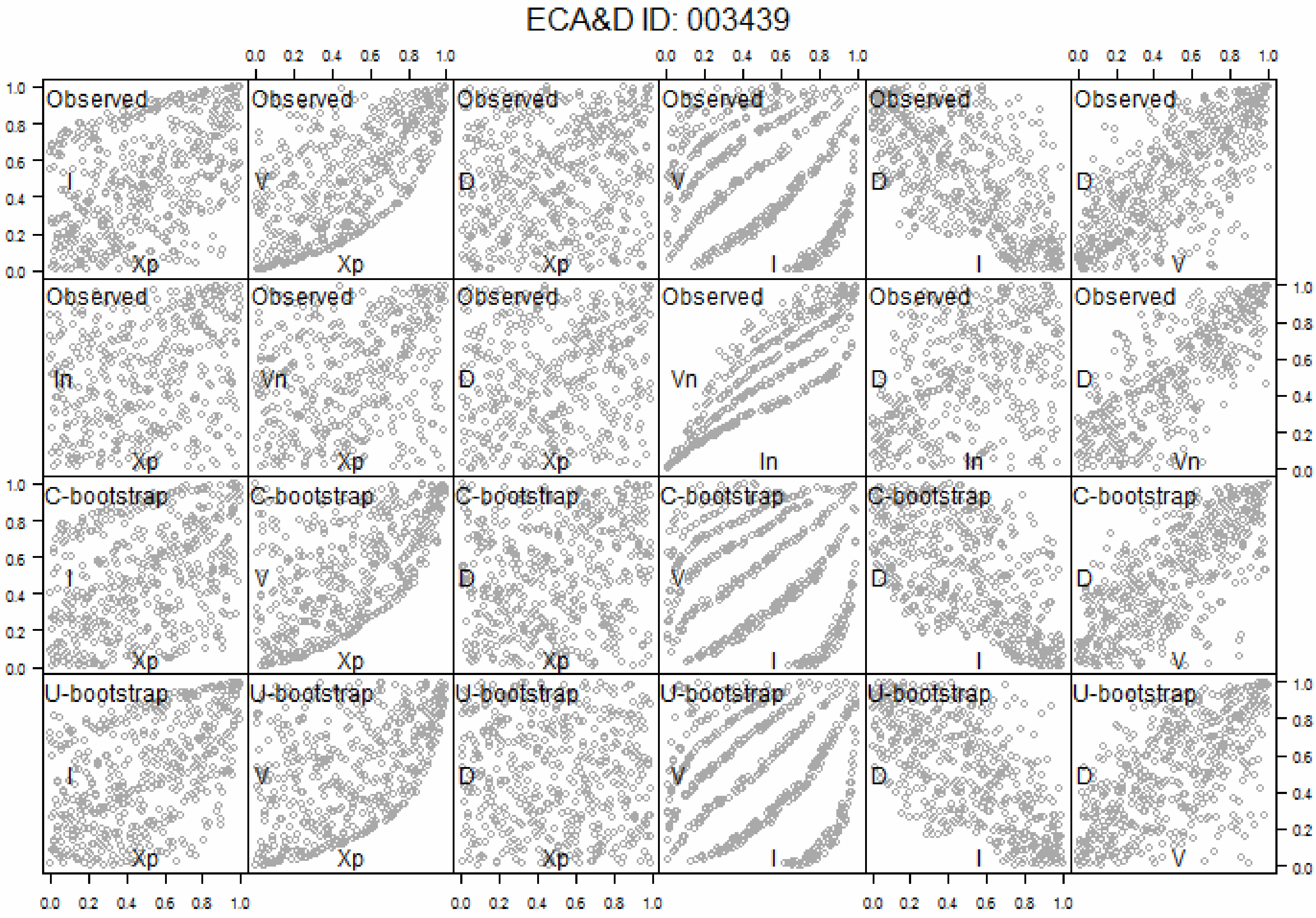
ECA&D ID: 003439
Observed
C-bootstrap
U-bootstrap
Xp
I
V
D
In
Vn
0.0
0.2
0.4
0.6
0.8
1.0

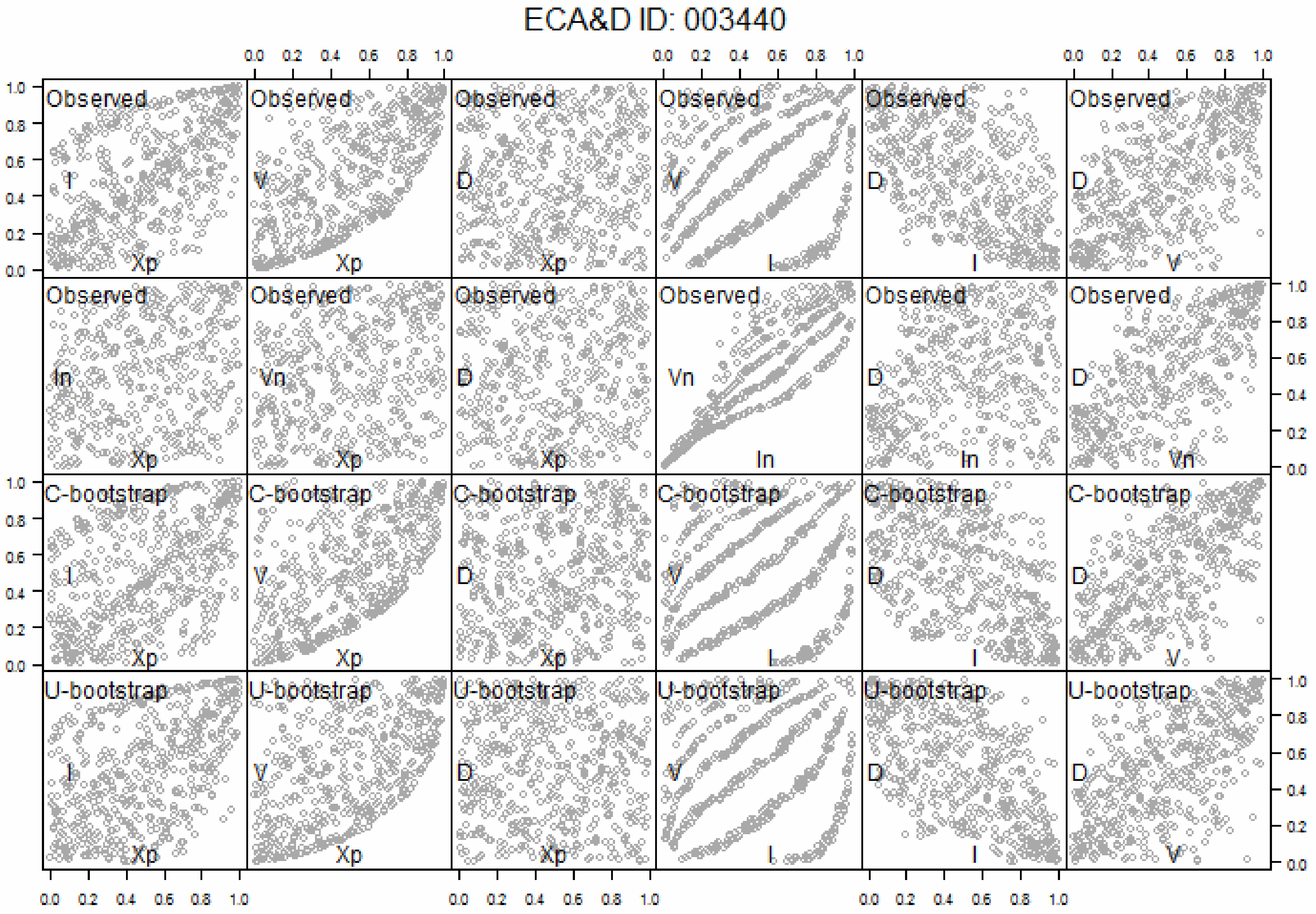
ECA&D ID: 003440
Observed
C-bootstrap
U-bootstrap
Xp
I
V
D
In
Vn
0.0
0.2
0.4
0.6
0.8
1.0

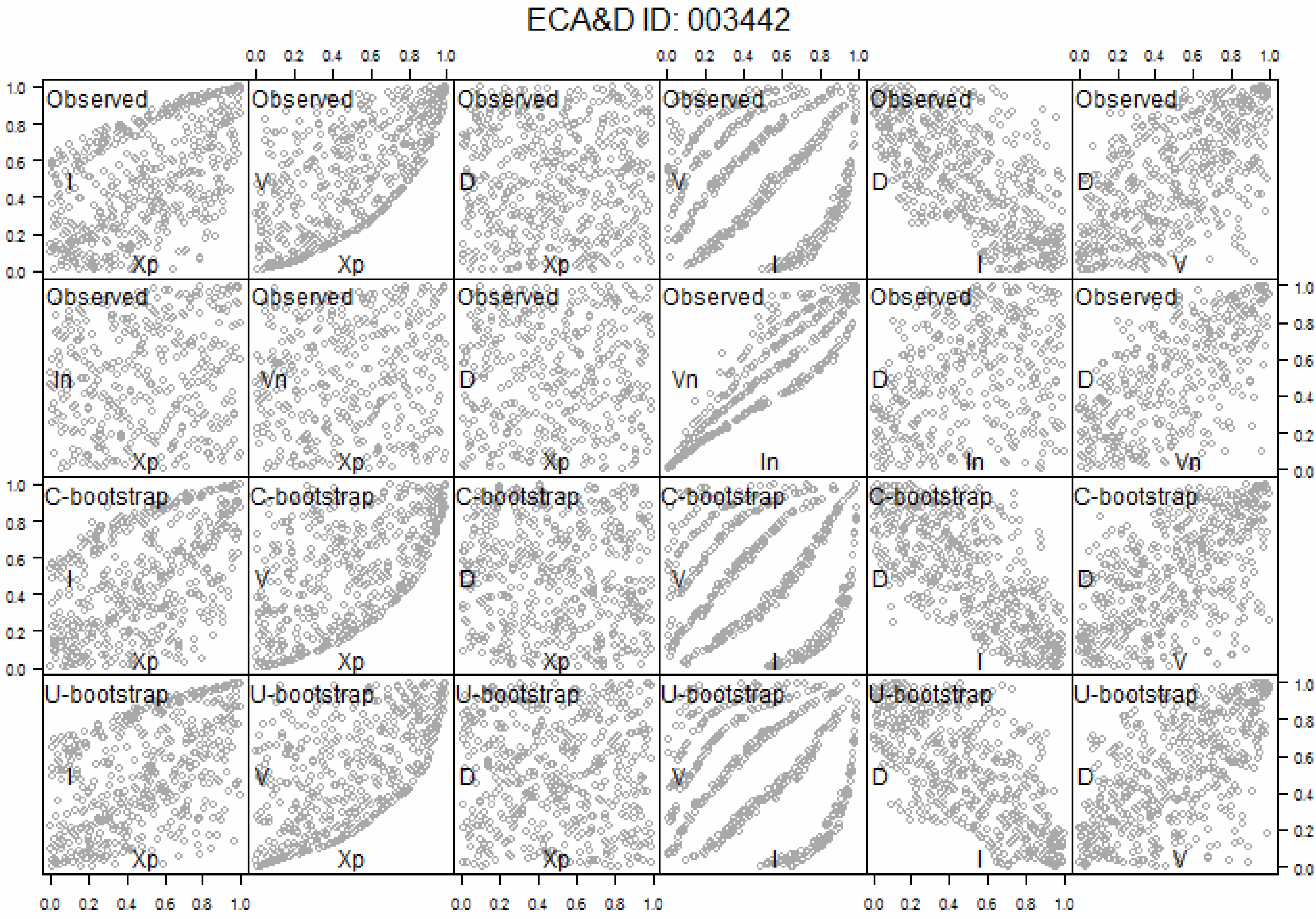
ECA&D ID: 003442
Observed
C-bootstrap
U-bootstrap
Xp
I
V
D
In
Vn
0.0
0.2
0.4
0.6
0.8
1.0

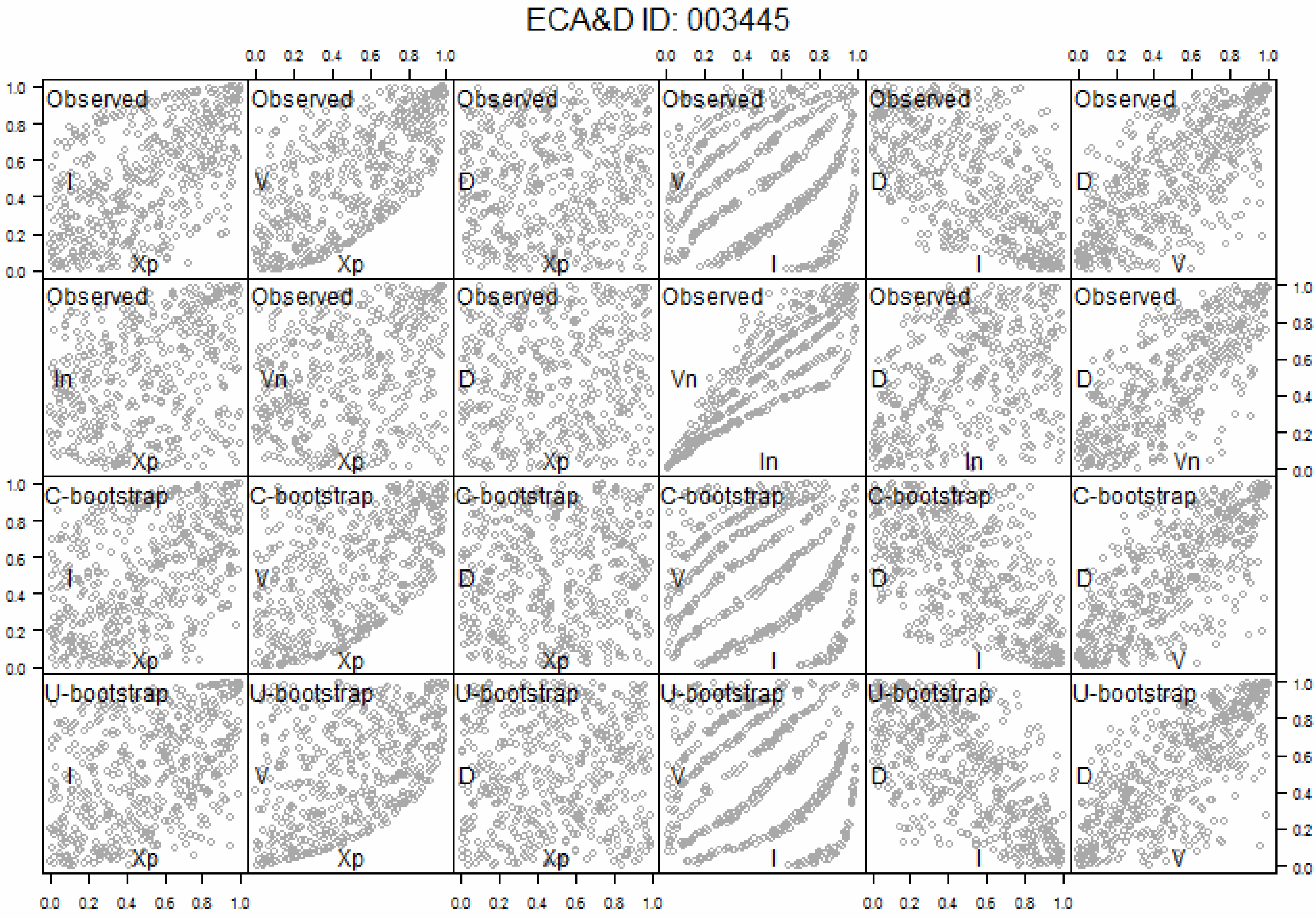

ECA&D ID: 003445
Observed
C-bootstrap
U-bootstrap
I
V
D
In
Vn
Xp
0.0
0.2
0.4
0.6
0.8
1.0

ECA&D ID: 003446

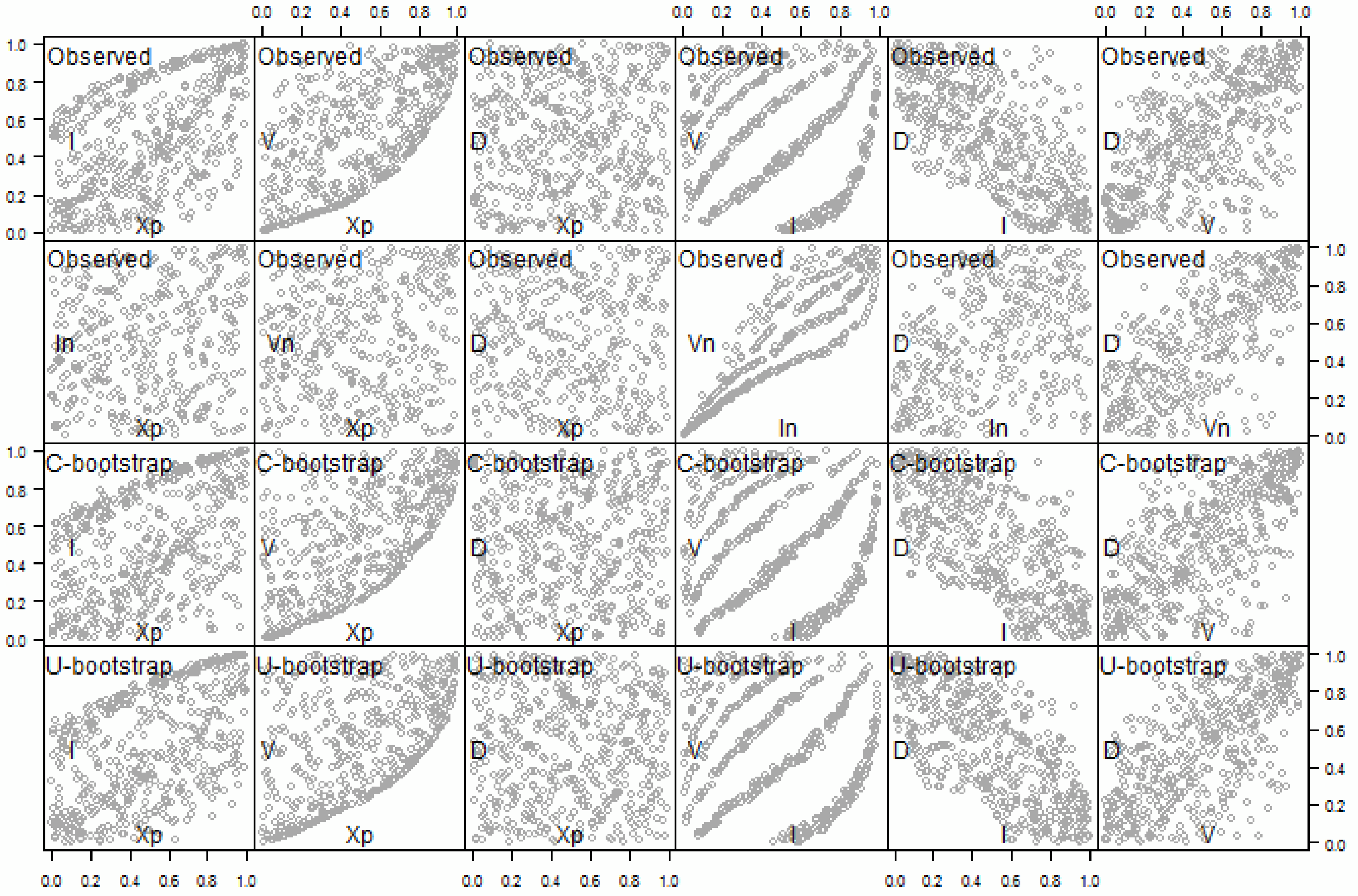

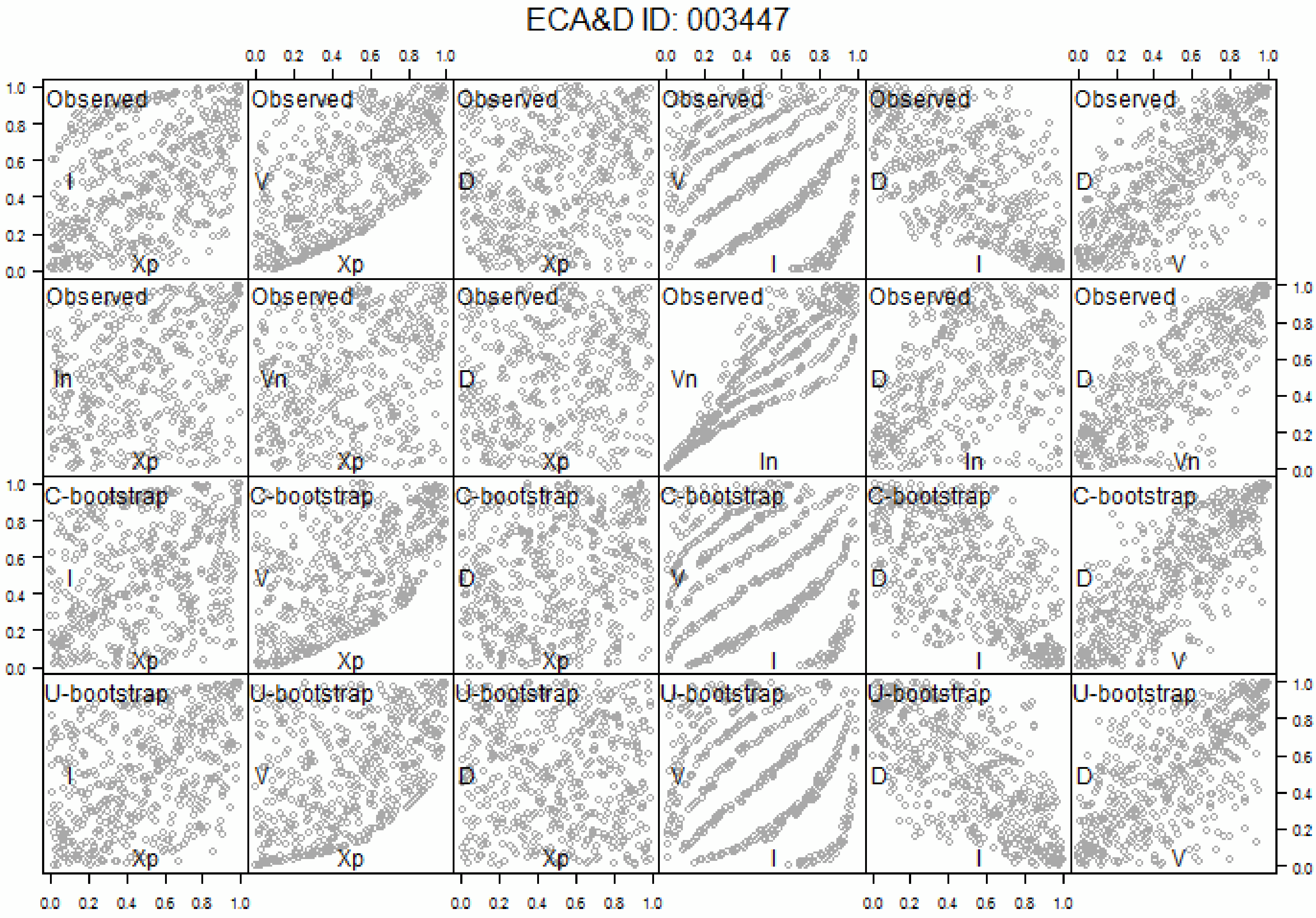
ECA&D ID: 003447
Observed
C-bootstrap
U-bootstrap
Xp
I
V
D
In
Vn
0.0
0.2
0.4
0.6
0.8
1.0

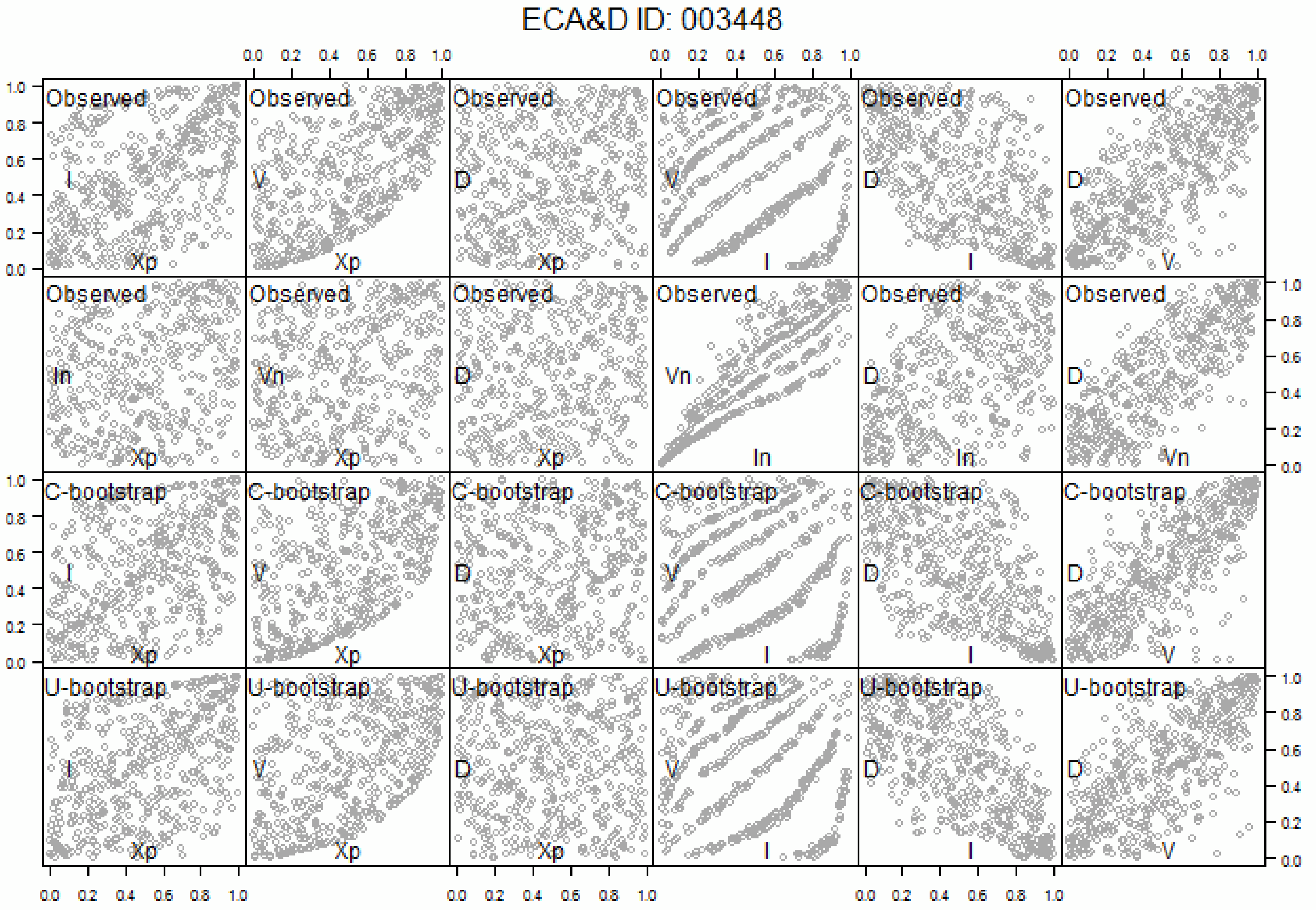
ECA&D ID: 003448
Observed
C-bootstrap
U-bootstrap
Xp
I
V
D
In
Vn
0.0
0.2
0.4
0.6
0.8
1.0

ECA&D ID: 003449

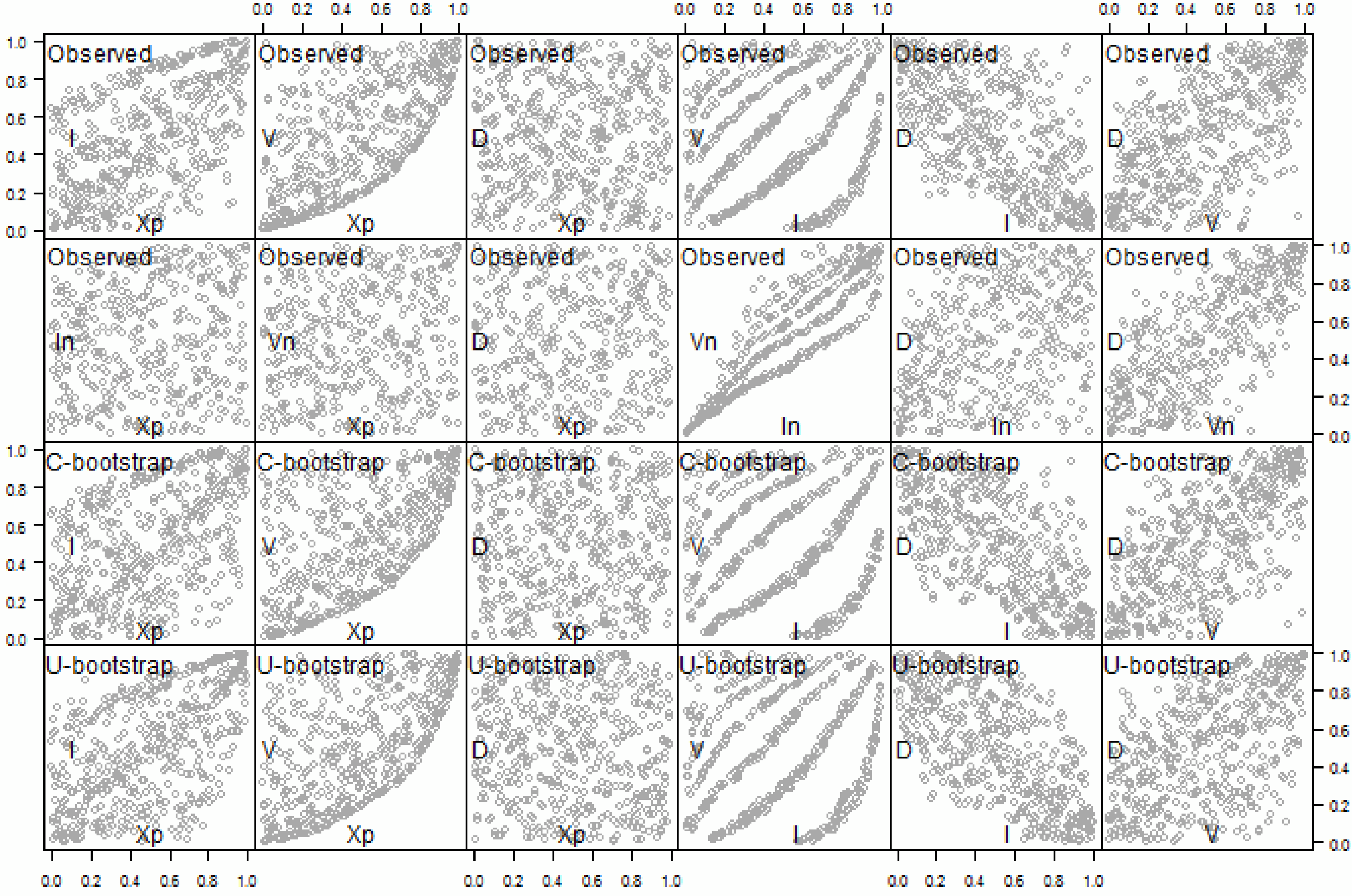

ECA&D ID: 003450

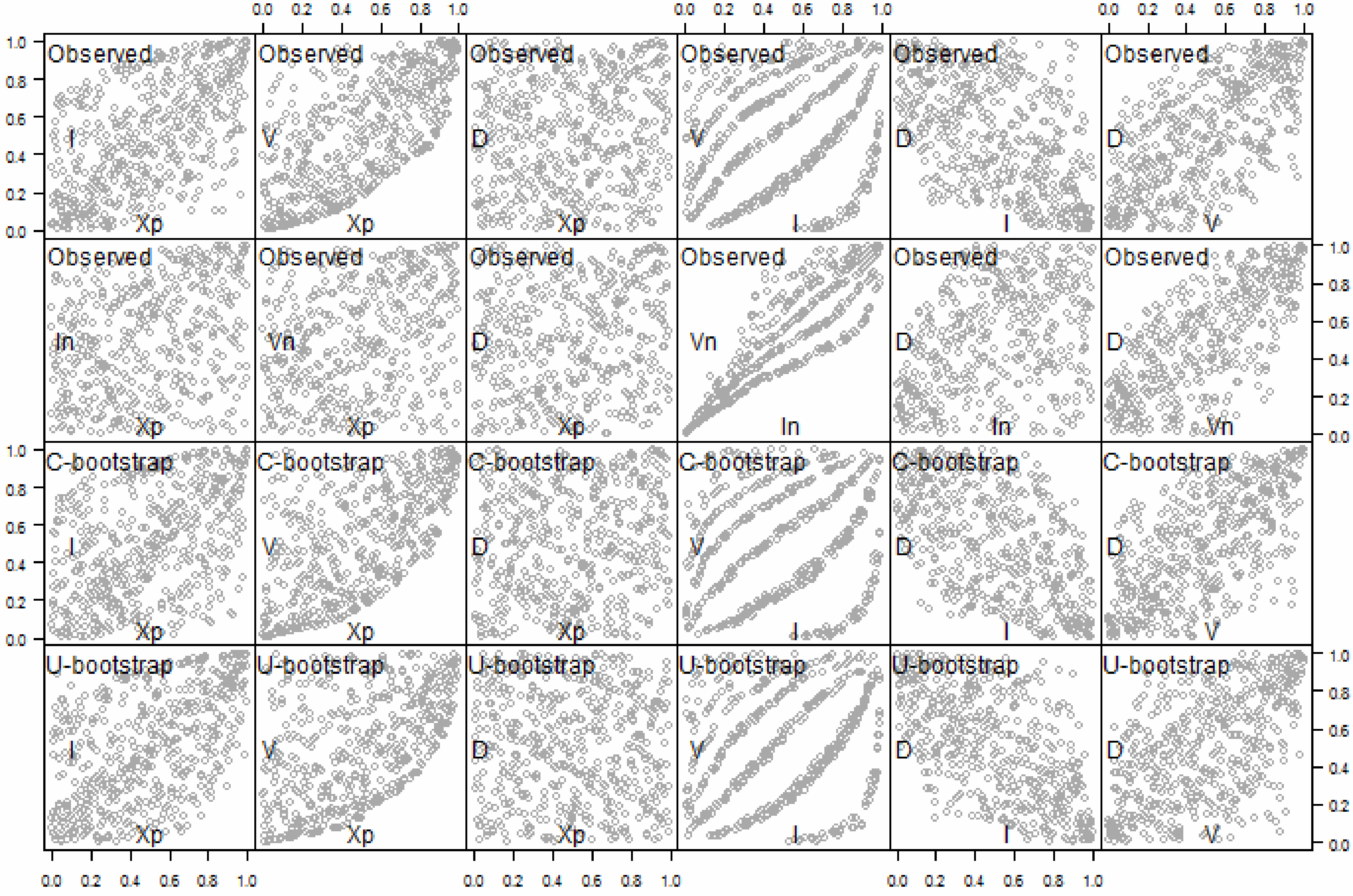

ECA&D ID: 003451

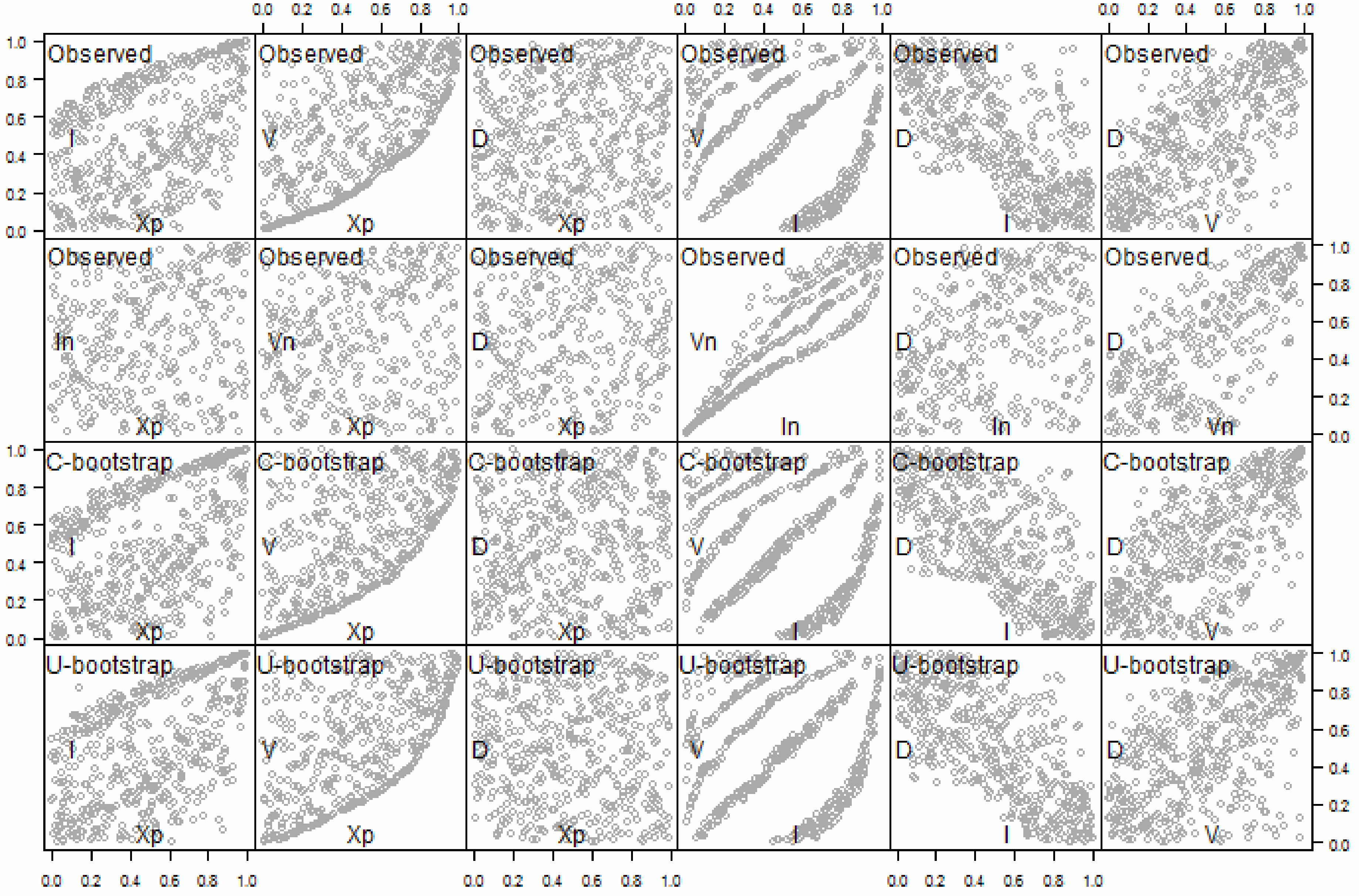

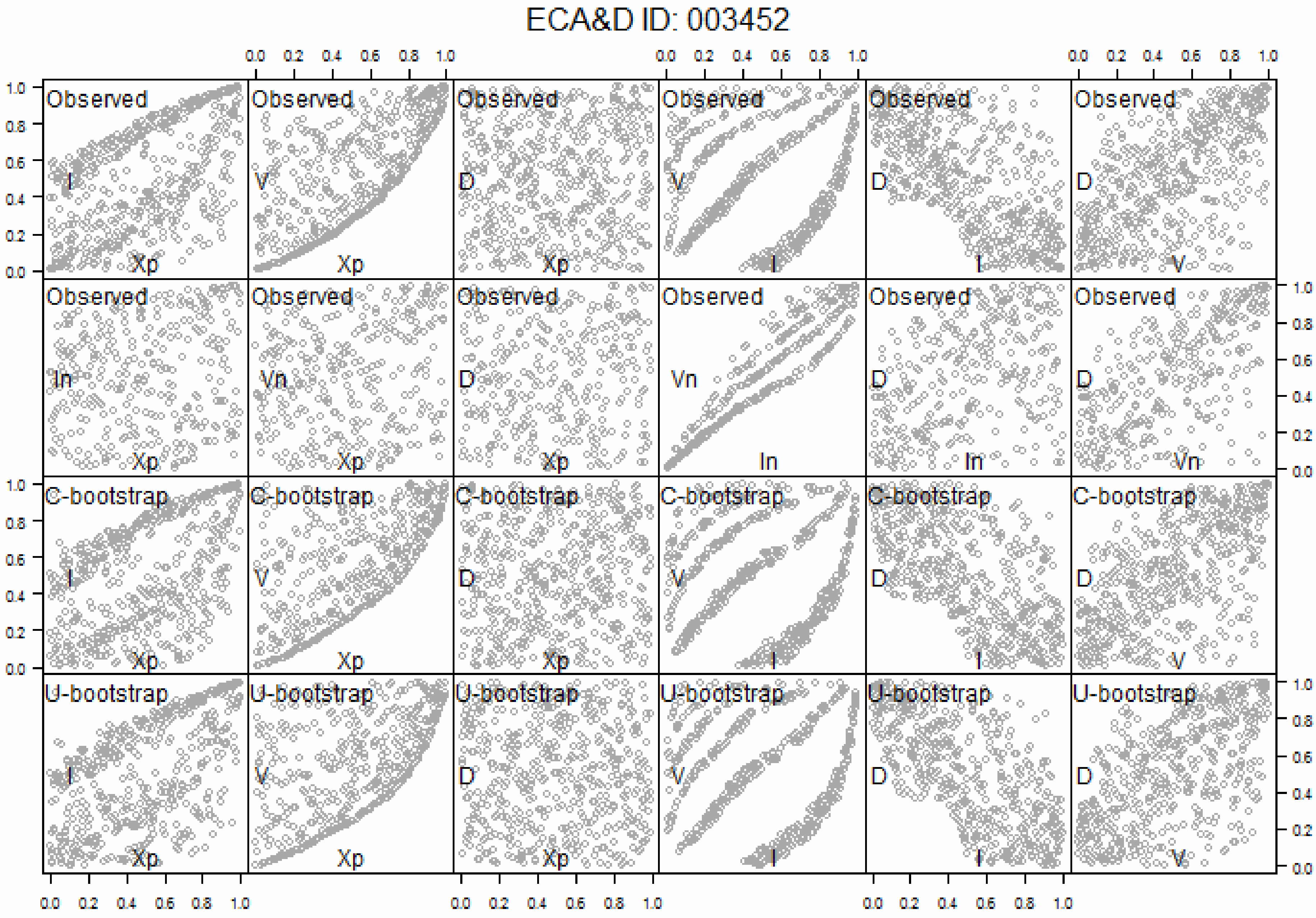

ECA&D ID: 003452
Observed
C-bootstrap
U-bootstrap
Xp
I
V
D
In
Vn
0.0
0.2
0.4
0.6
0.8
1.0

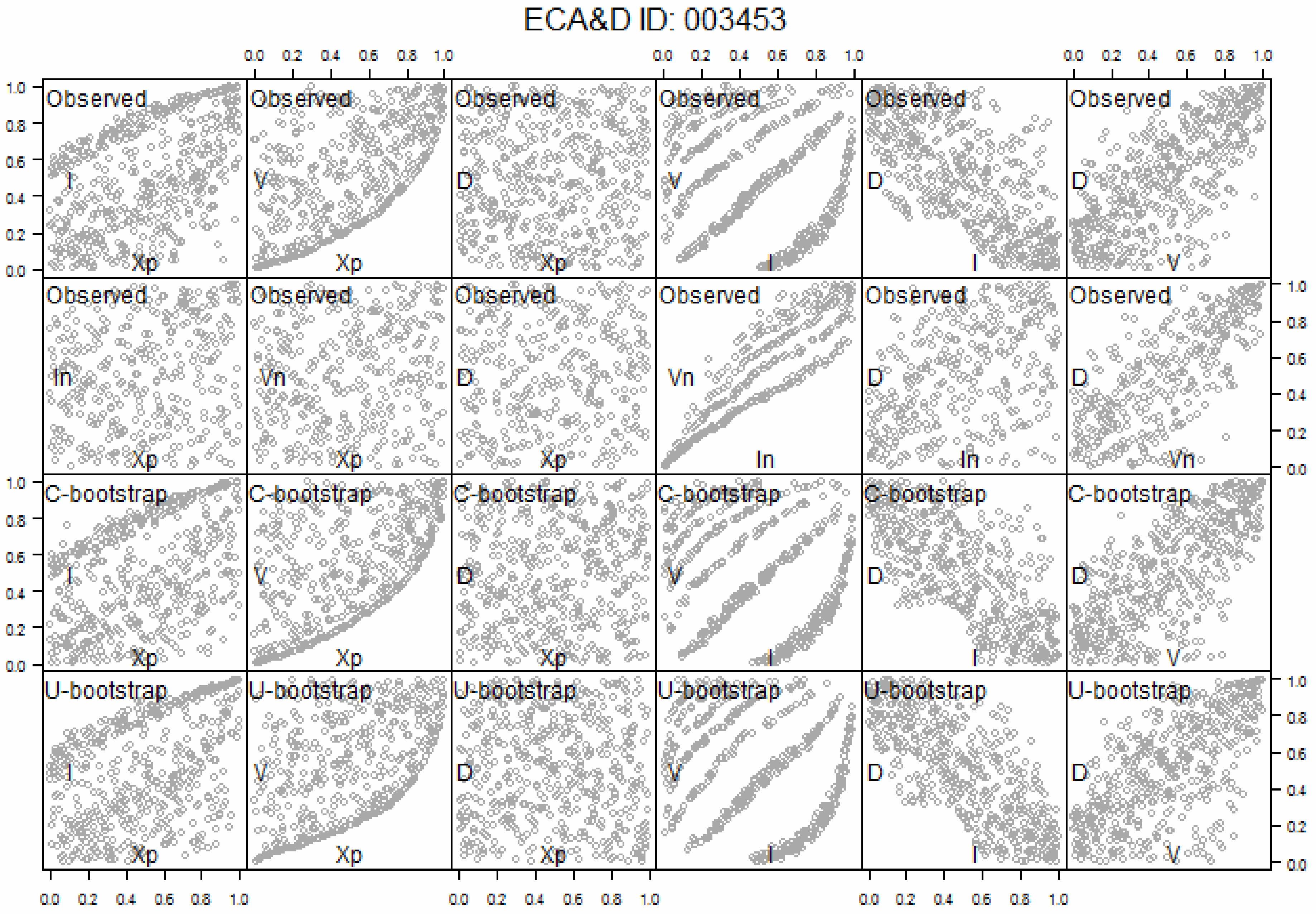
ECA&D ID: 003453
Observed
C-bootstrap
U-bootstrap
Xp
I
V
D
In
Vn
0.0
0.2
0.4
0.6
0.8
1.0

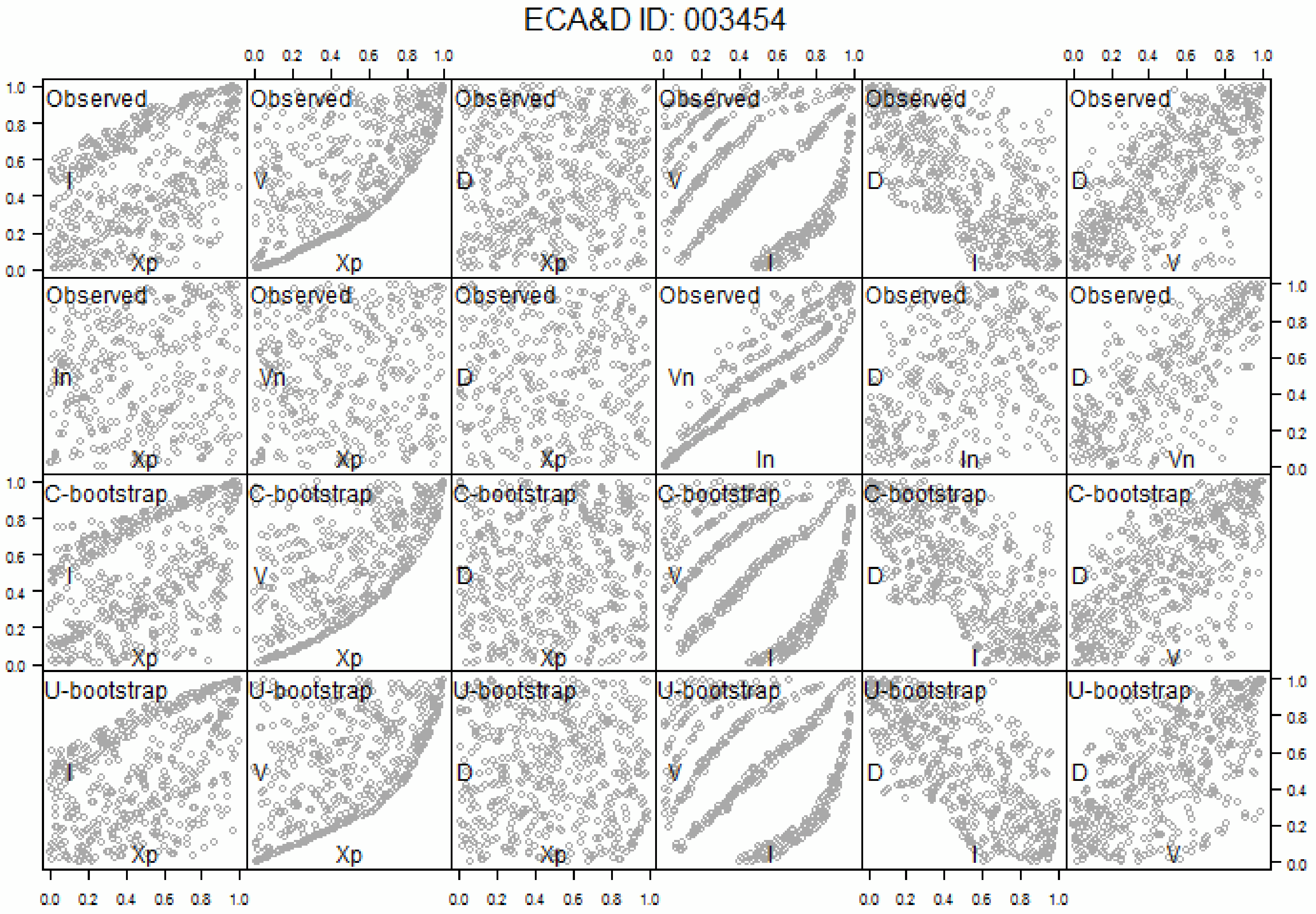
ECA&D ID: 003454
Observed
C-bootstrap
U-bootstrap
Xp
I
V
D
In
Vn
0.0
0.2
0.4
0.6
0.8
1.0

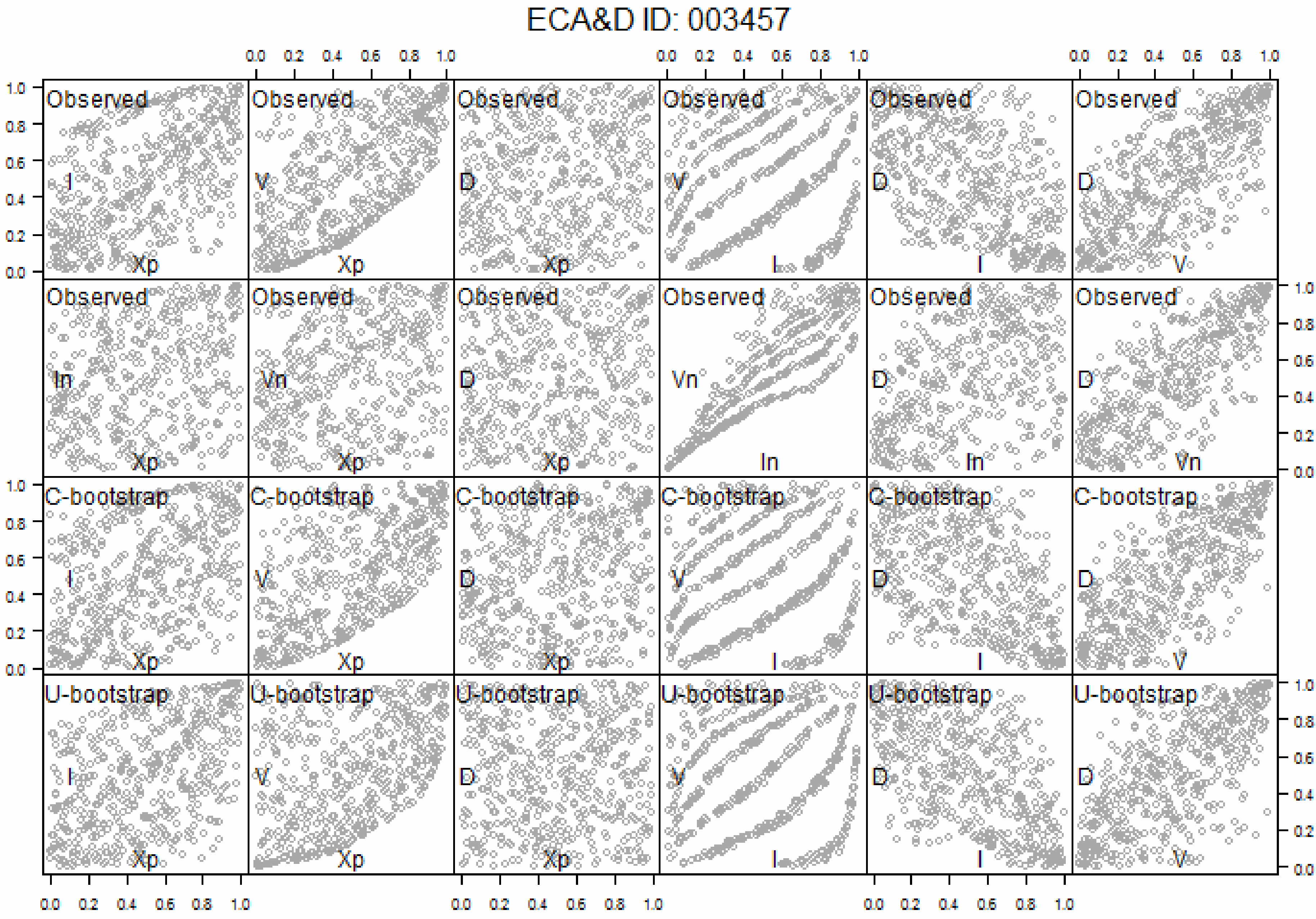
ECA&D ID: 003457
Observed
C-bootstrap
U-bootstrap
Xp
I
V
D
In
Vn
0.0
0.2
0.4
0.6
0.8
1.0

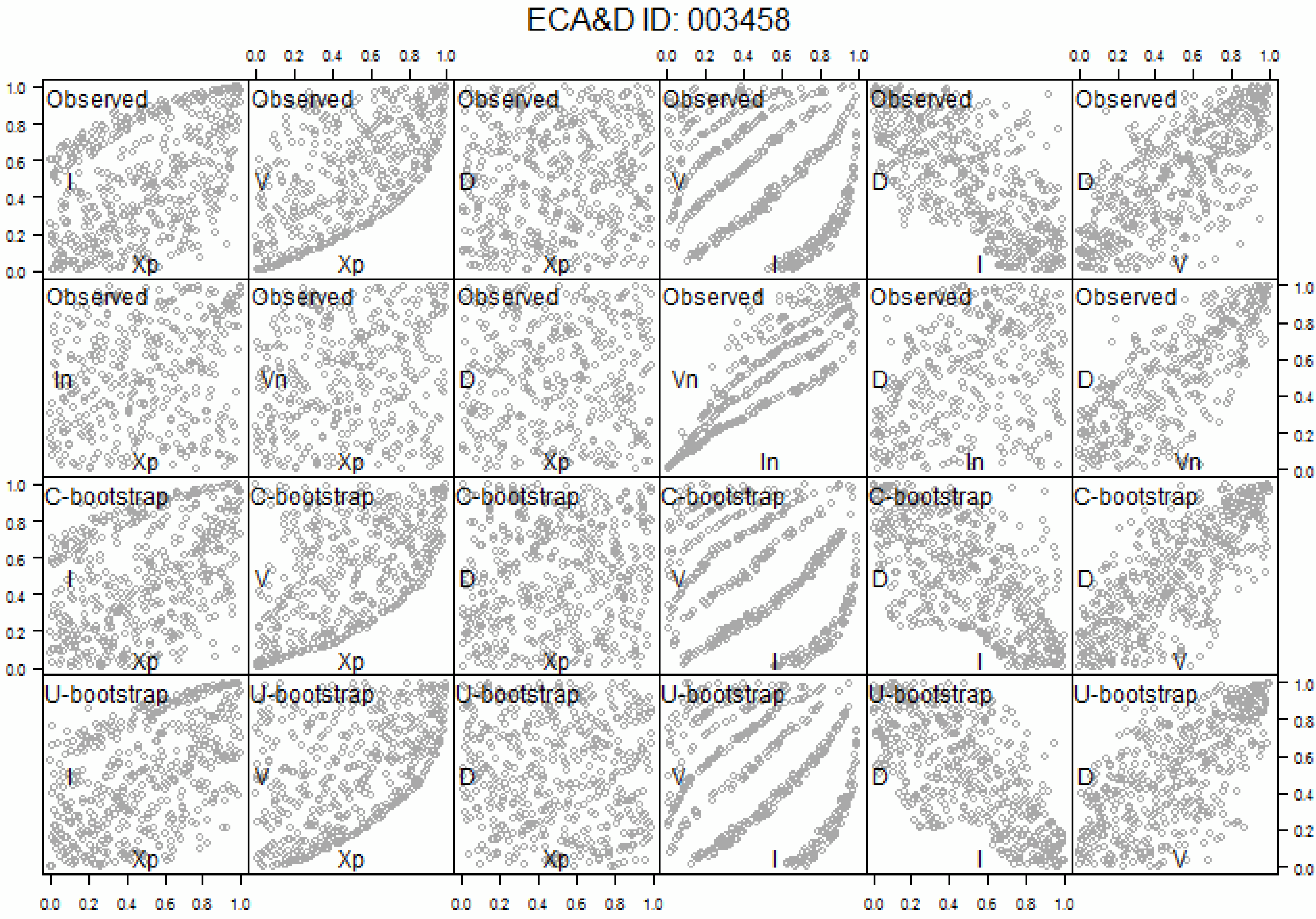
ECA&D ID: 003458
Observed
C-bootstrap
U-bootstrap
Xp
I
V
D
In
Vn
0.0
0.2
0.4
0.6
0.8
1.0

ECA&D ID: 003459

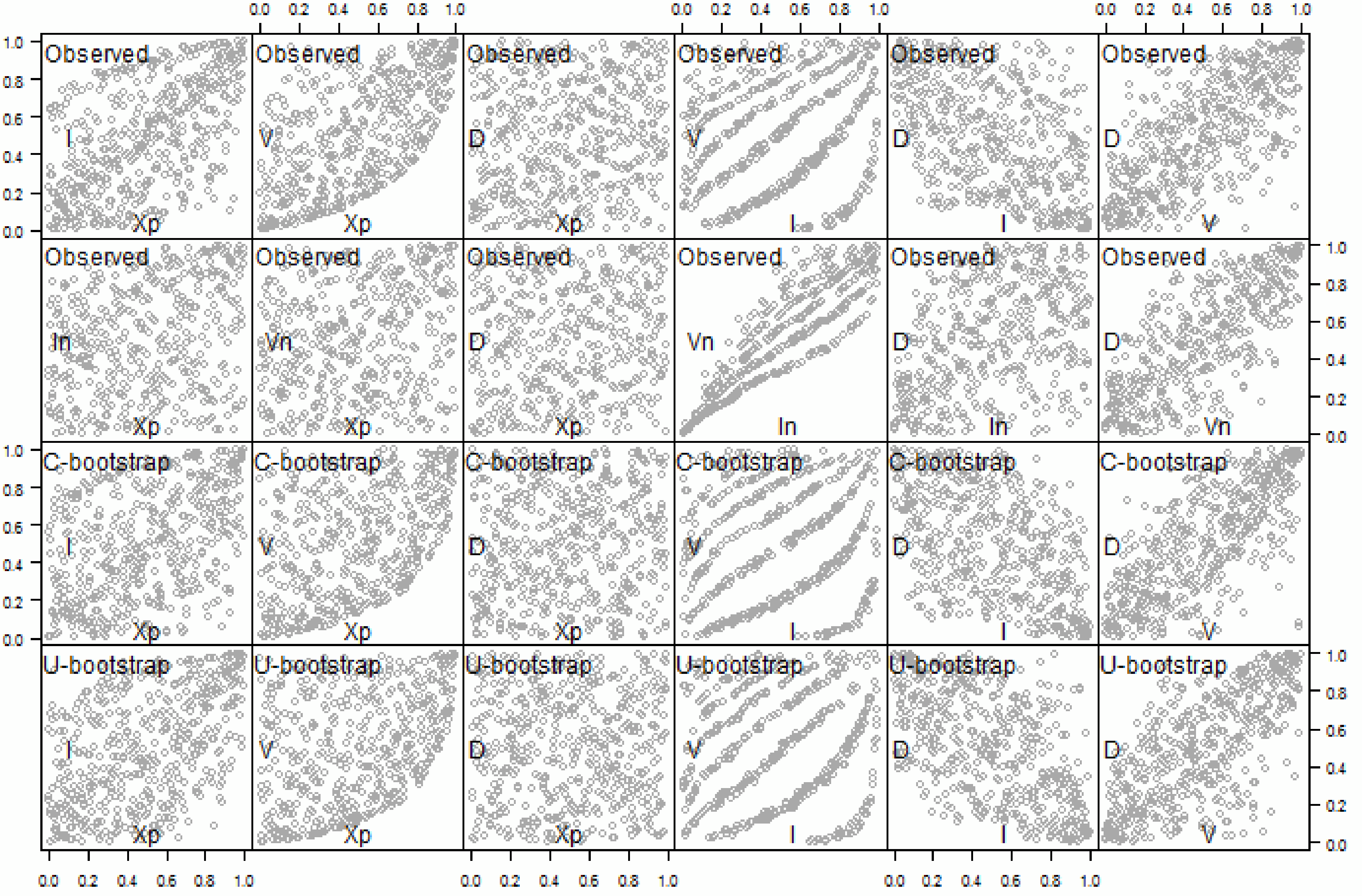

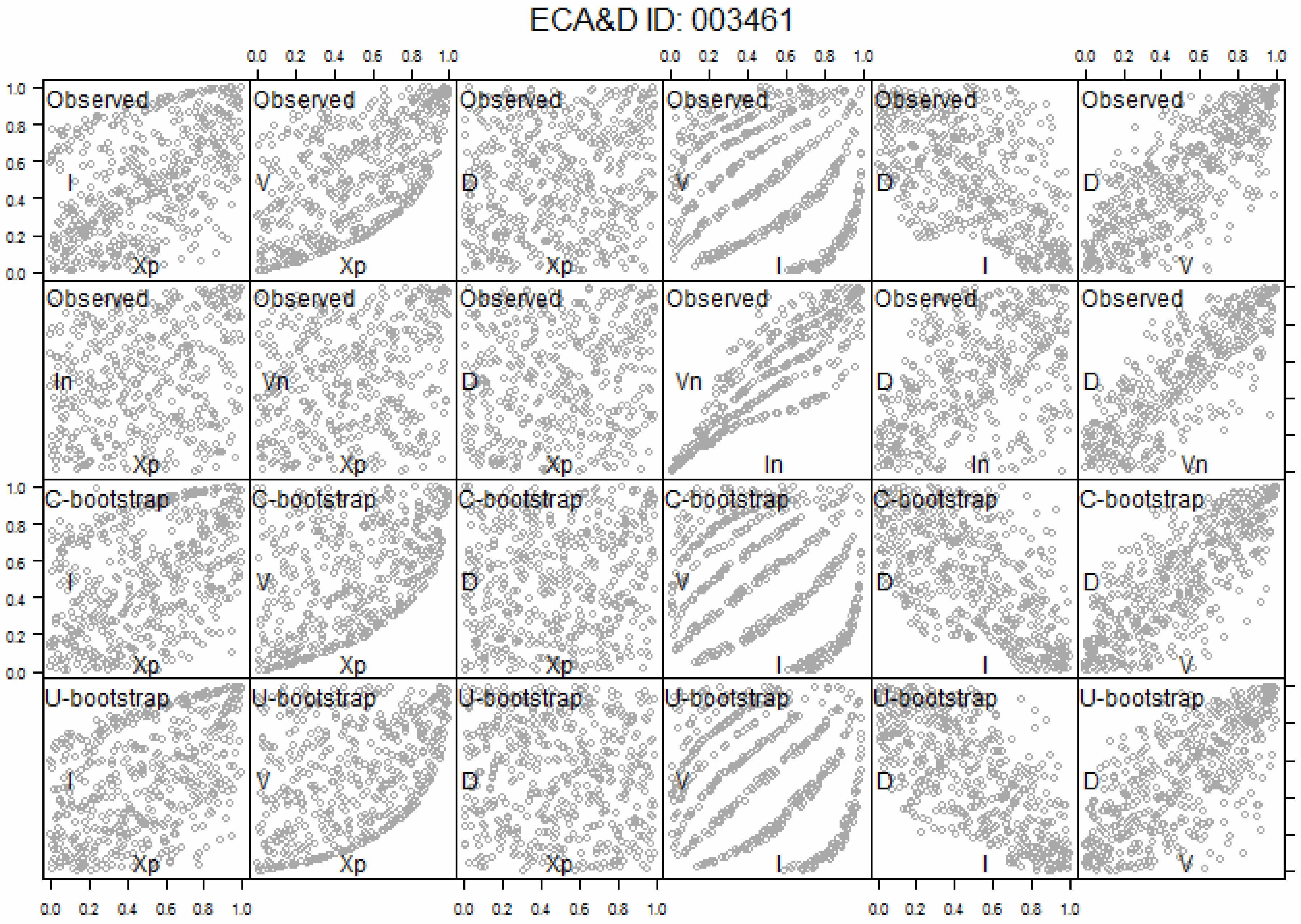
ECA&D ID: 003461
Observed
C-bootstrap
U-bootstrap
Xp
I
V
D
In
Vn
0.0
0.2
0.4
0.6
0.8
1.0

ECA&D ID: 003462

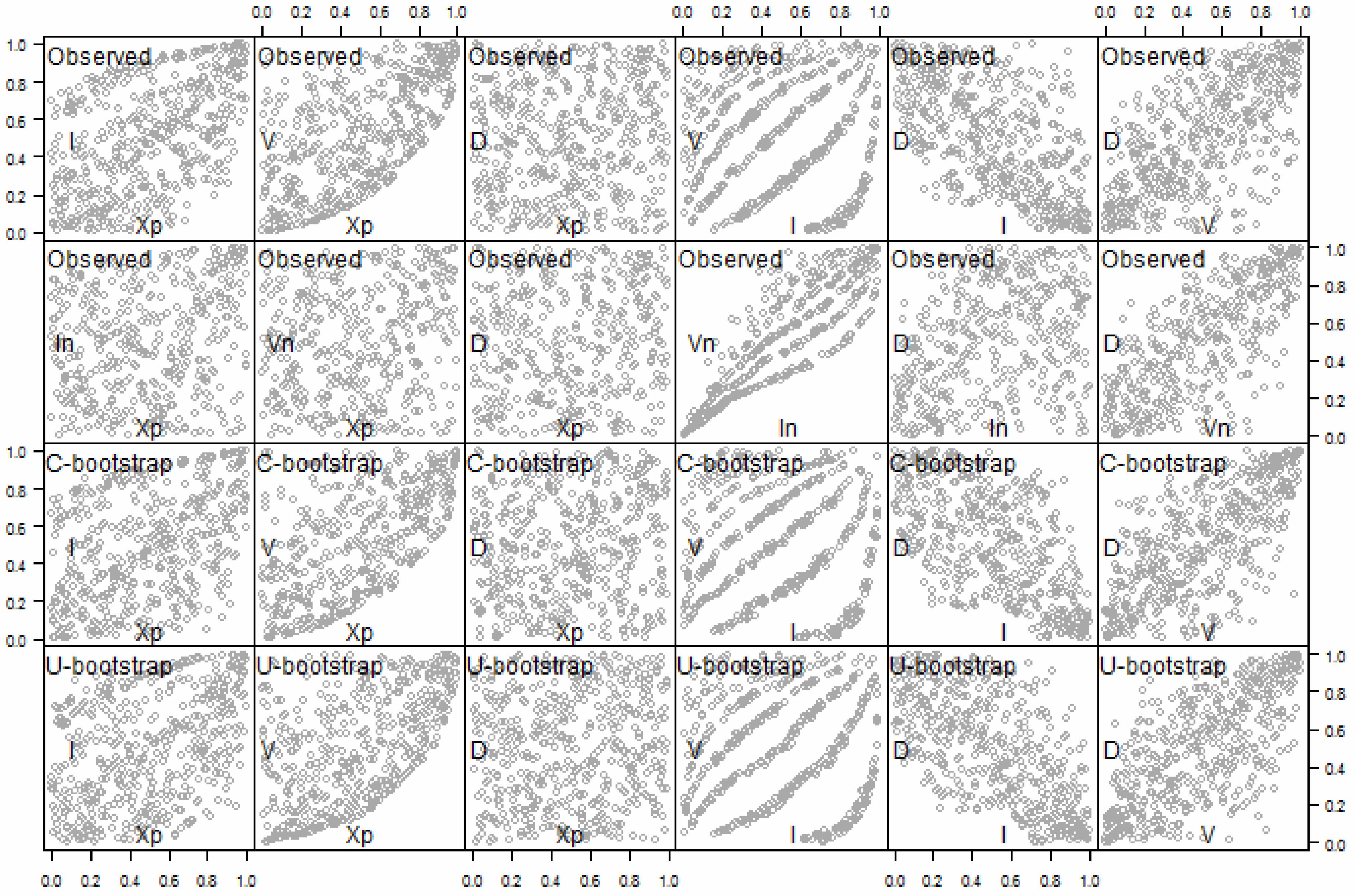

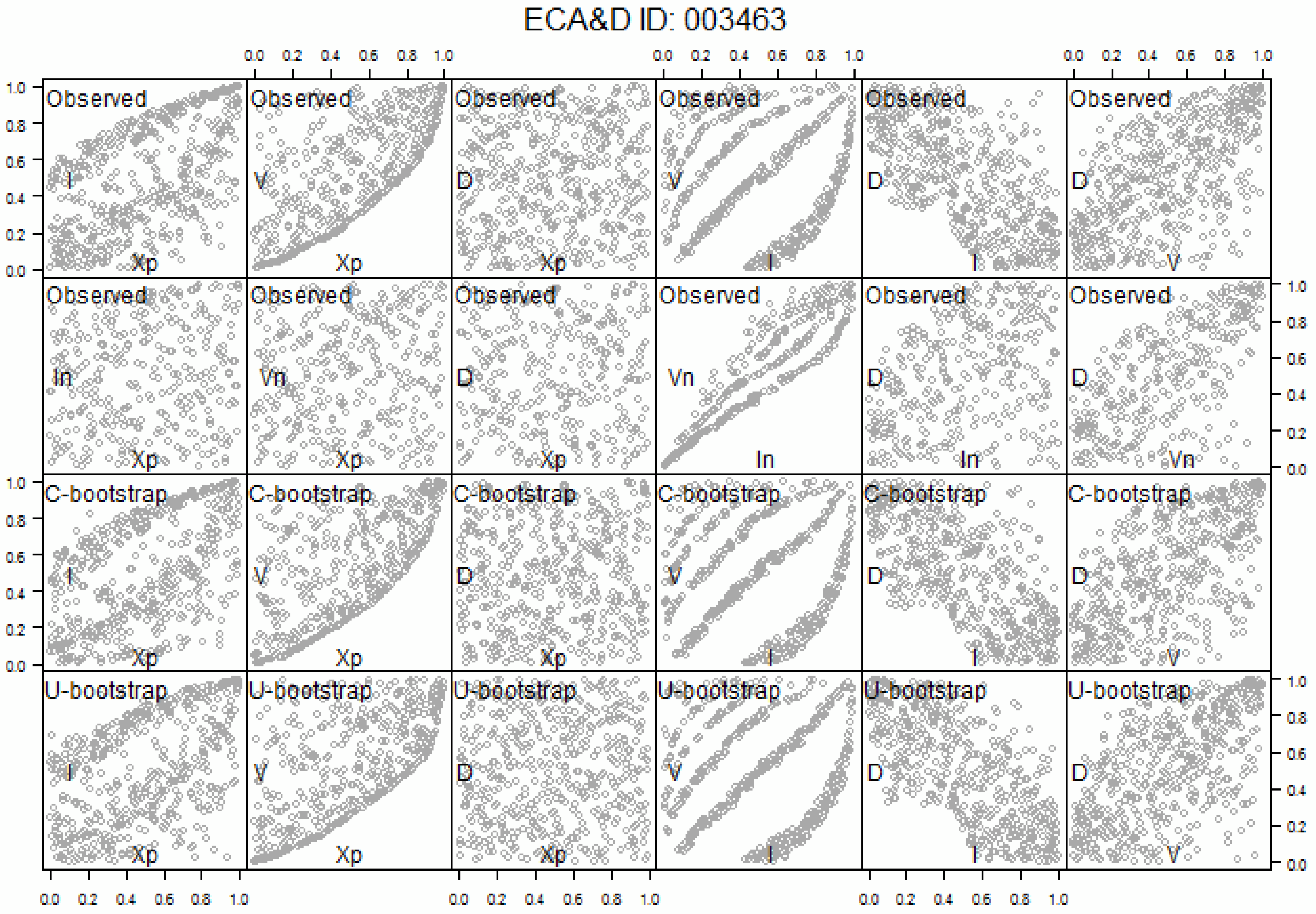

ECA&D ID: 003463
Observed
C-bootstrap
U-bootstrap
Xp
I
V
D
In
Vn
0.0
0.2
0.4
0.6
0.8
1.0

ECA&D ID: 003464

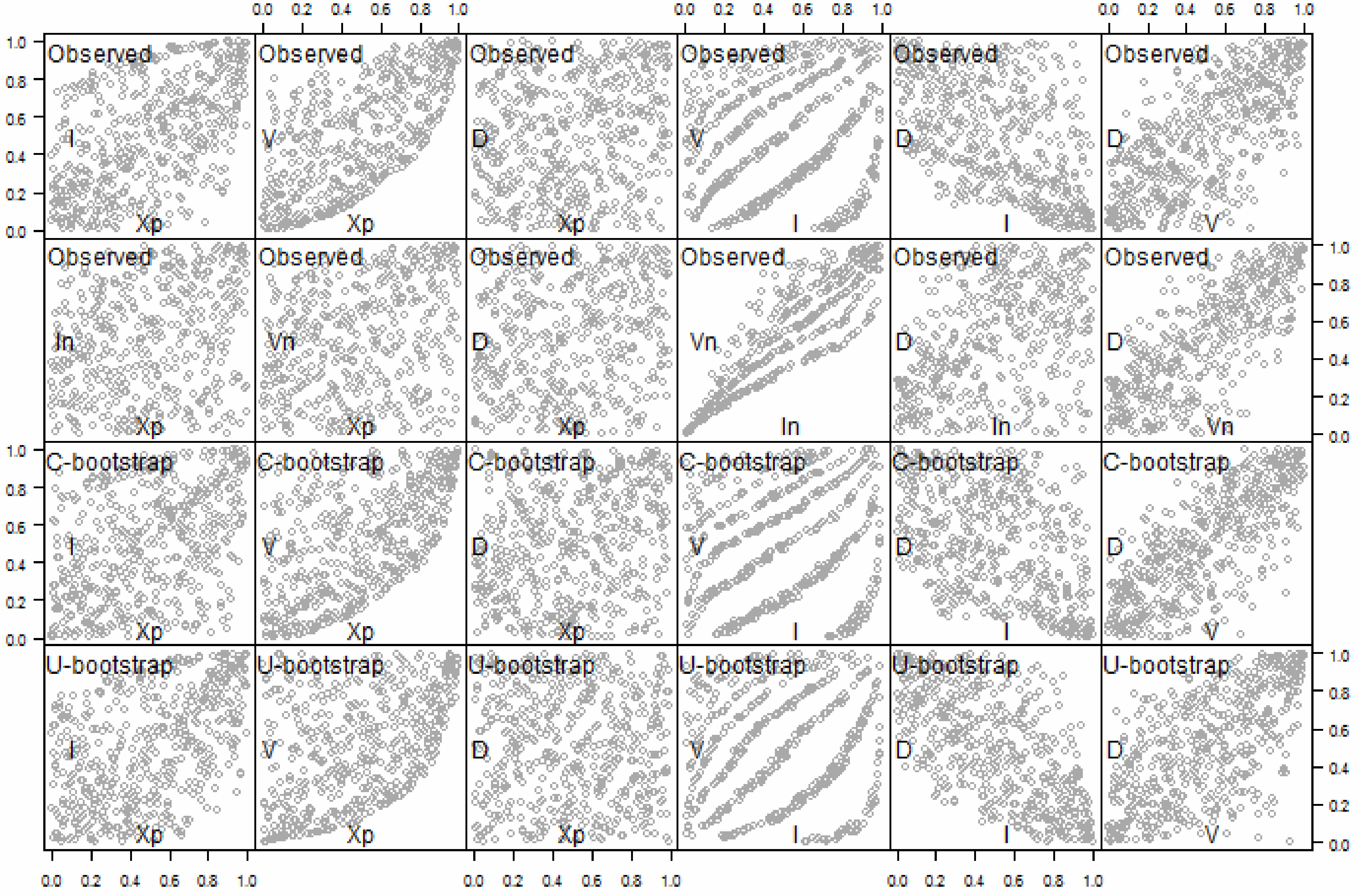

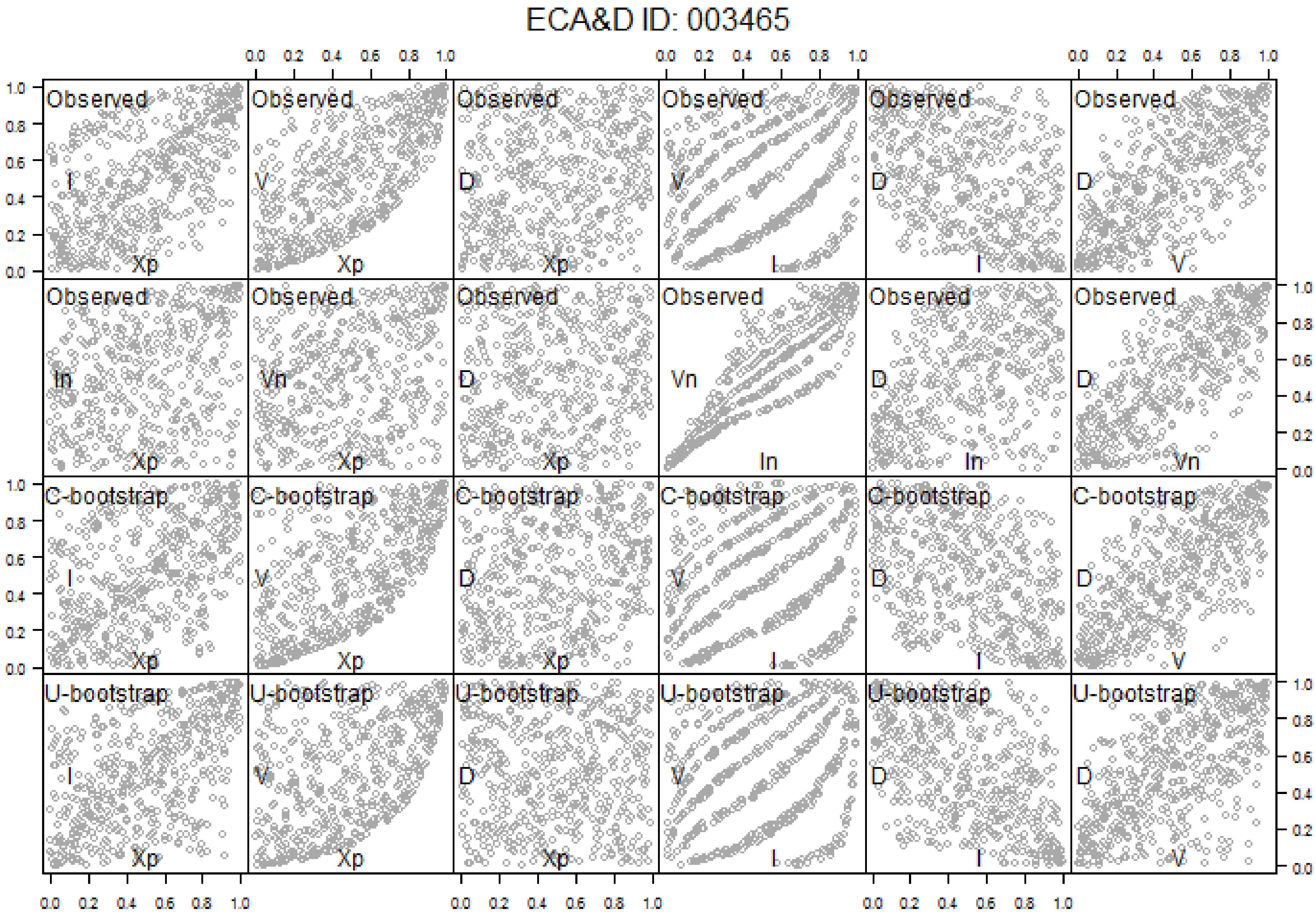
ECA&D ID: 003465
Observed
C-bootstrap
U-bootstrap
Xp
I
V
D
In
Vn
0.0
0.2
0.4
0.6
0.8
1.0

ECA&D ID: 003466

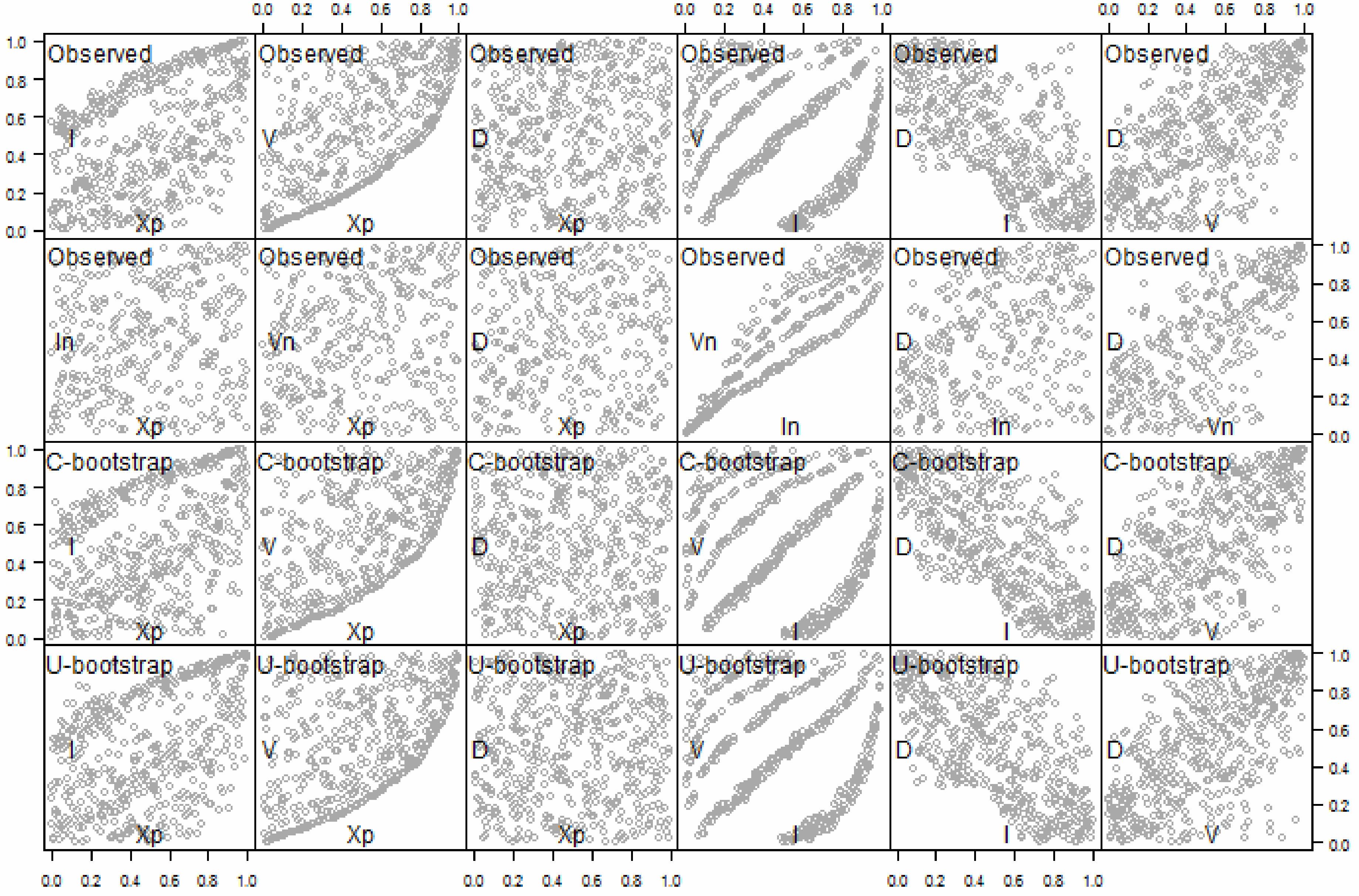

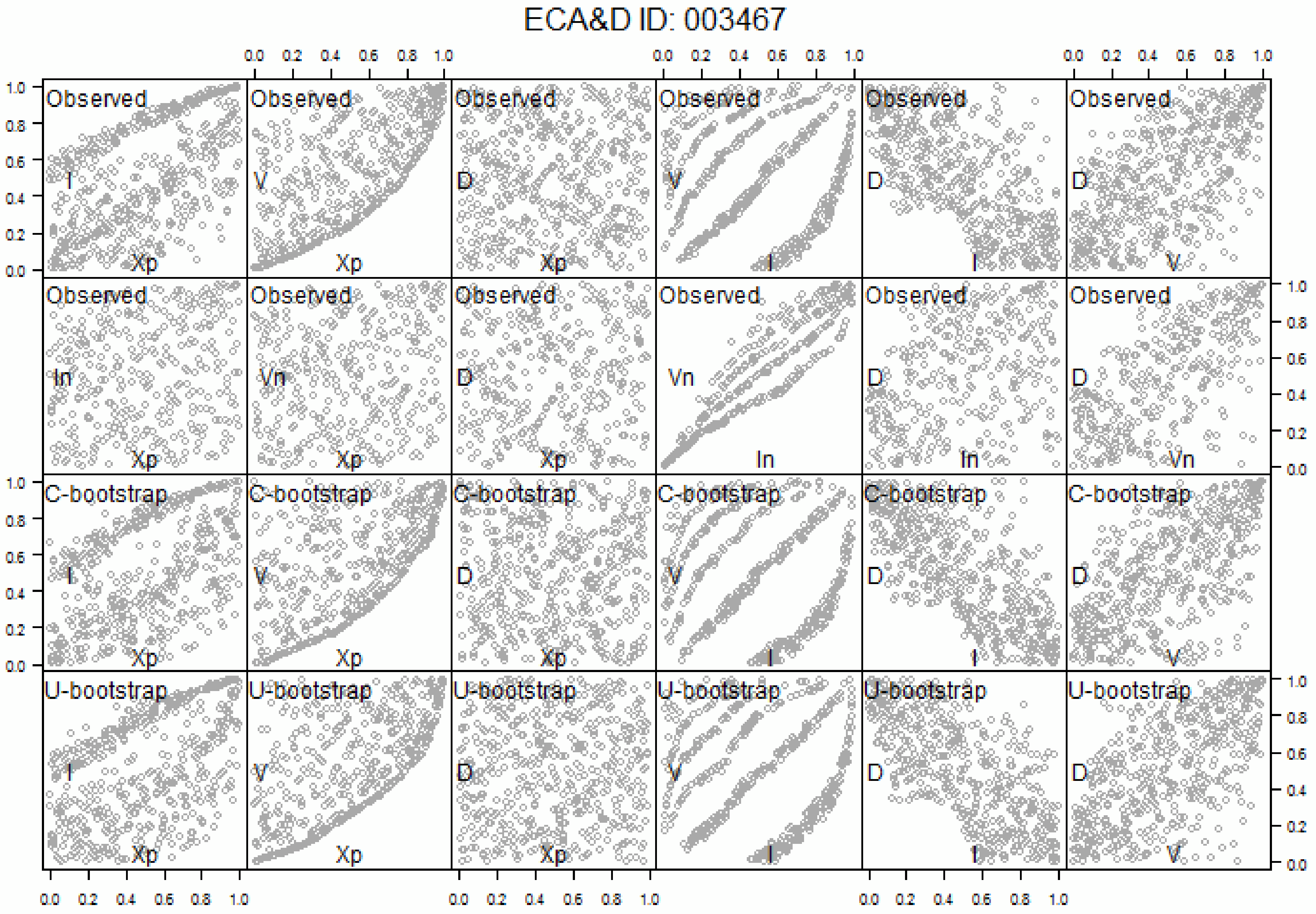

ECA&D ID: 003467
Observed
C-bootstrap
U-bootstrap
Xp
I
V
D
In
Vn
0.0
0.2
0.4
0.6
0.8
1.0

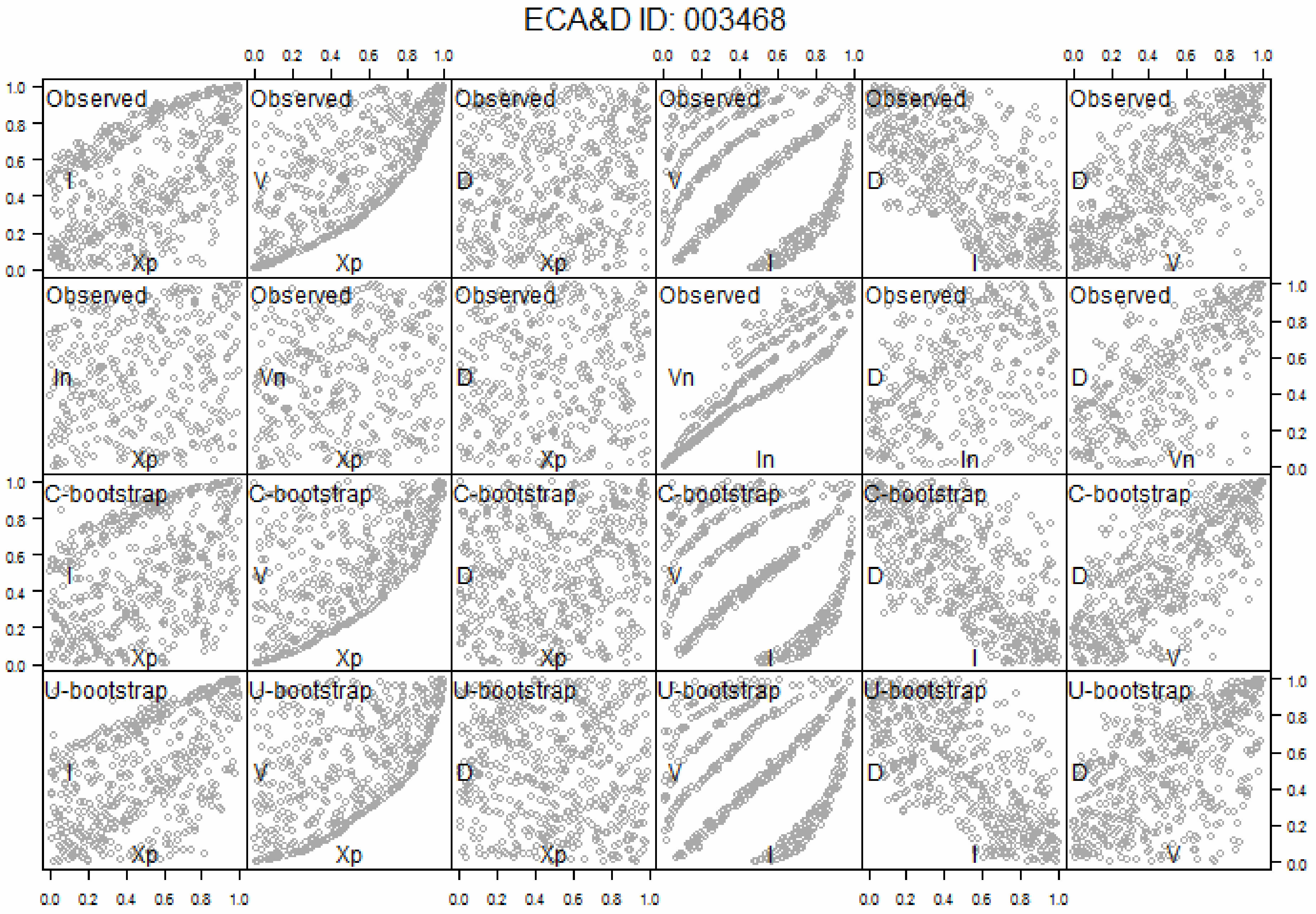

ECA&D ID: 003468
Observed
C-bootstrap
U-bootstrap
Xp
I
V
D
In
Vn
0.0
0.2
0.4
0.6
0.8
1.0

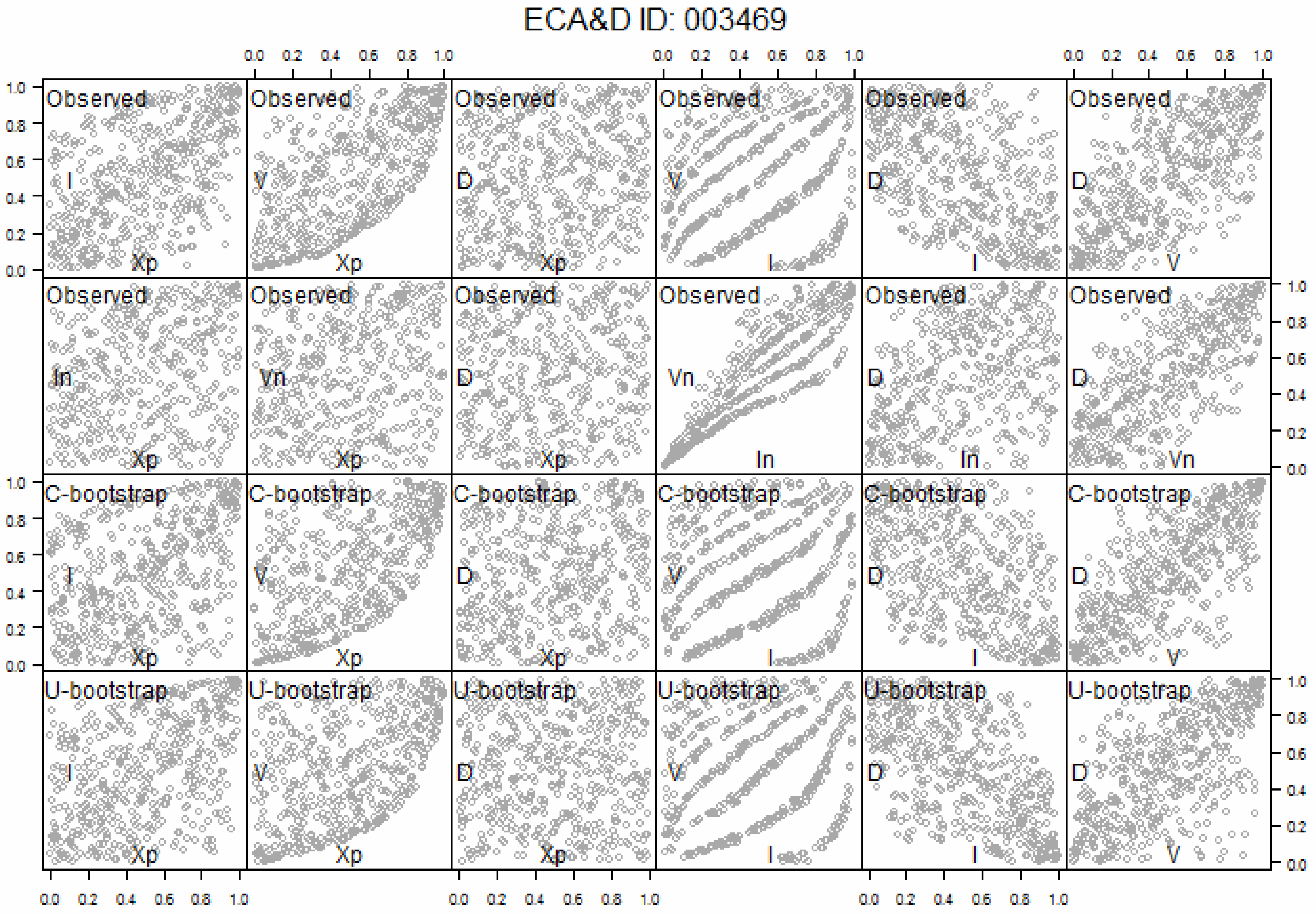

ECA&D ID: 003469
Observed
C-bootstrap
U-bootstrap
Xp
I
V
D
In
Vn
0.0
0.2
0.4
0.6
0.8
1.0

ECA&D ID: 003470

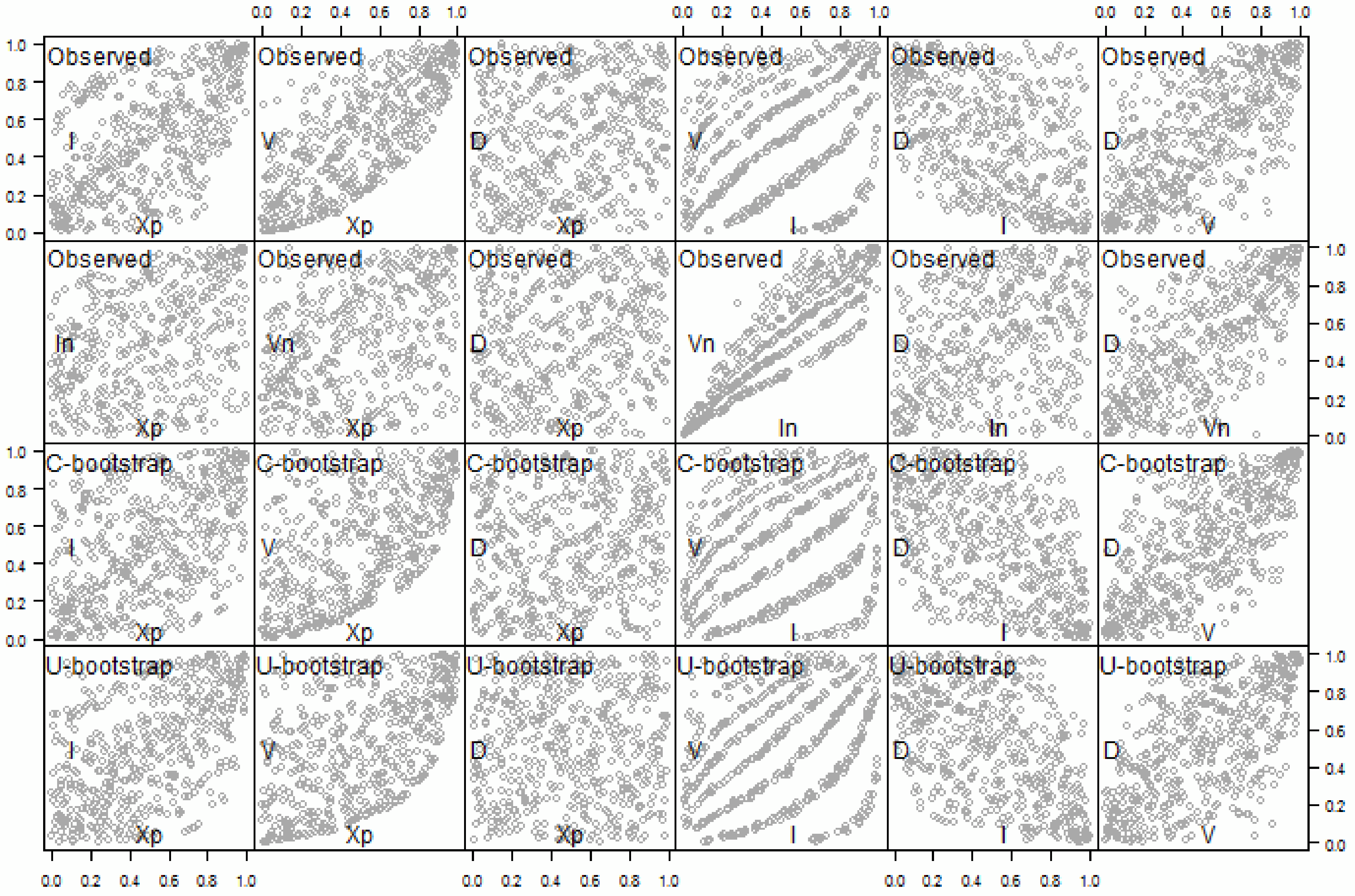

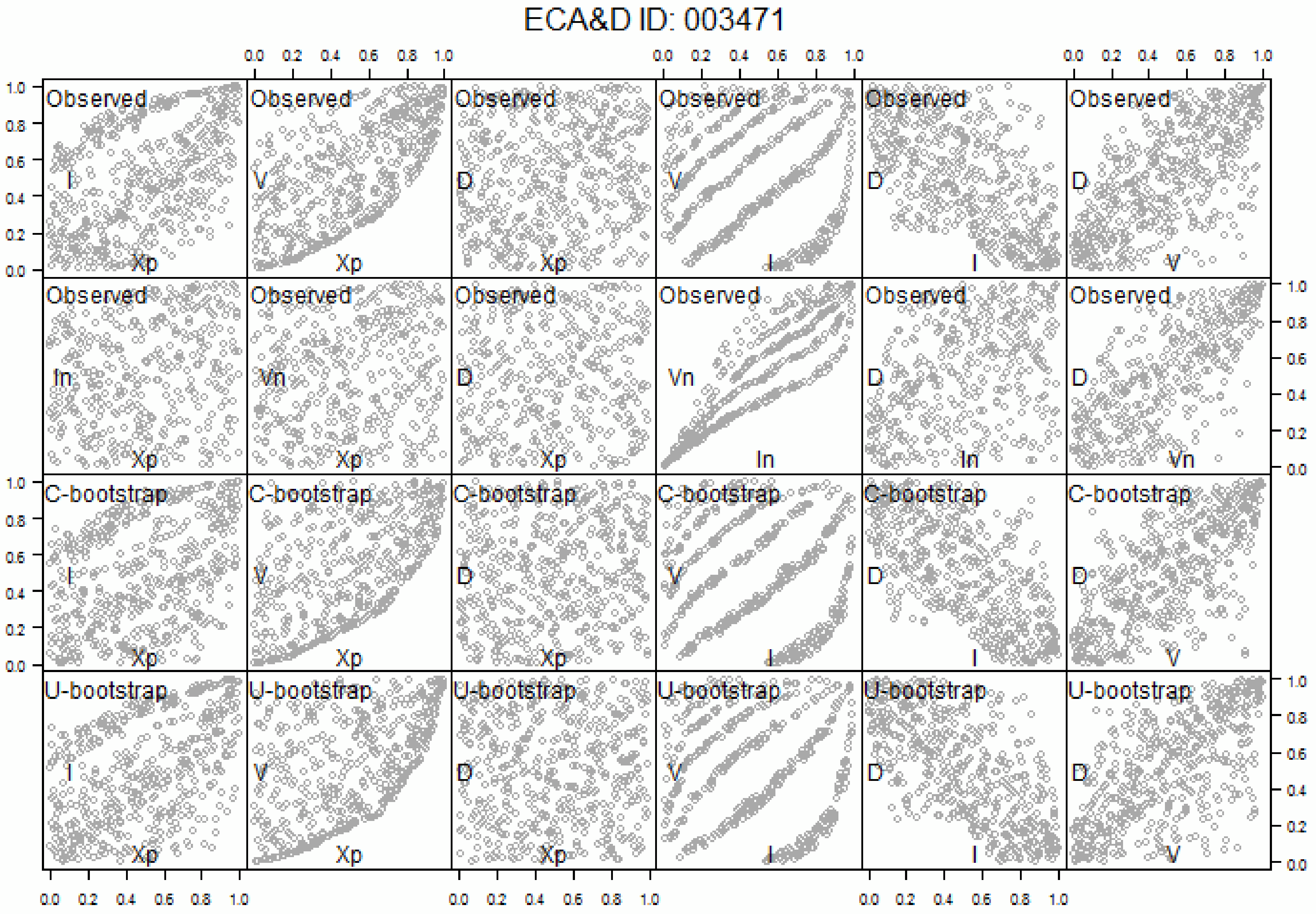
ECA&D ID: 003471
Observed
C-bootstrap
U-bootstrap
Xp
I
V
D
In
Vn
0.0
0.2
0.4
0.6
0.8
1.0

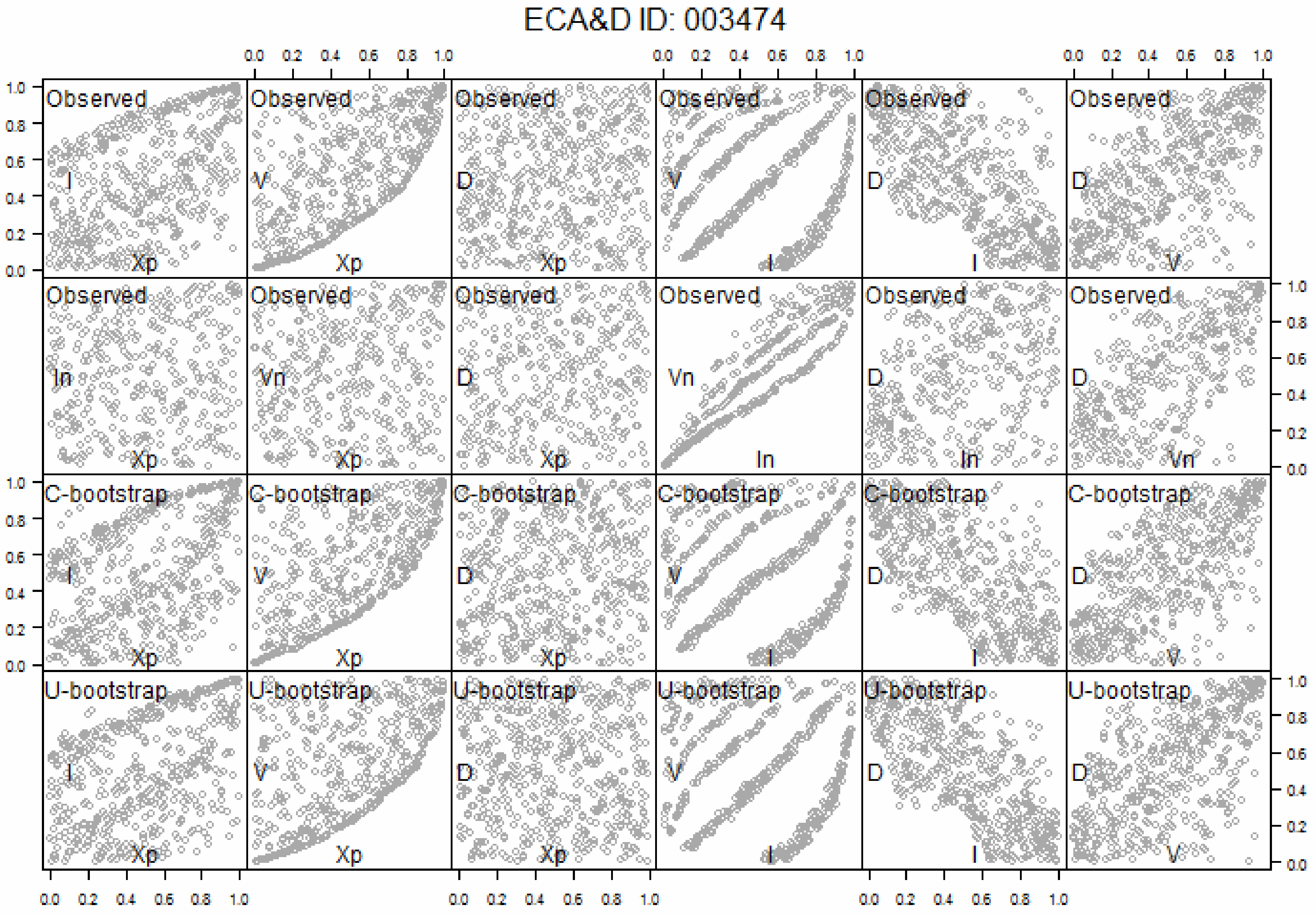
ECA&D ID: 003474
Observed
C-bootstrap
U-bootstrap
Xp
I
V
D
In
Vn
0.0
0.2
0.4
0.6
0.8
1.0

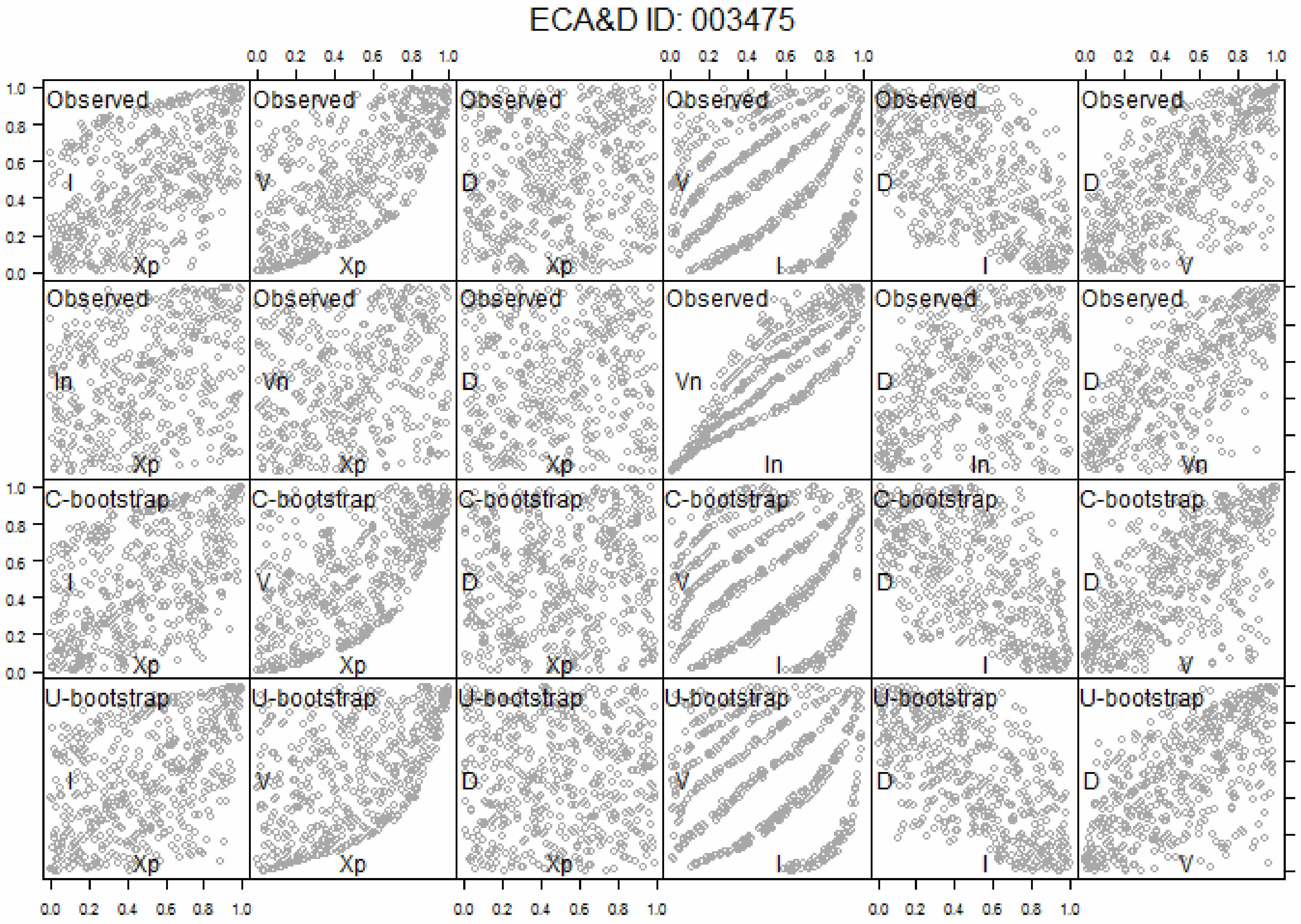
ECA&D ID: 003475
Observed
C-bootstrap
U-bootstrap
Xp
I
V
D
In
Vn
0.0
0.2
0.4
0.6
0.8
1.0

ECA&D ID: 003476

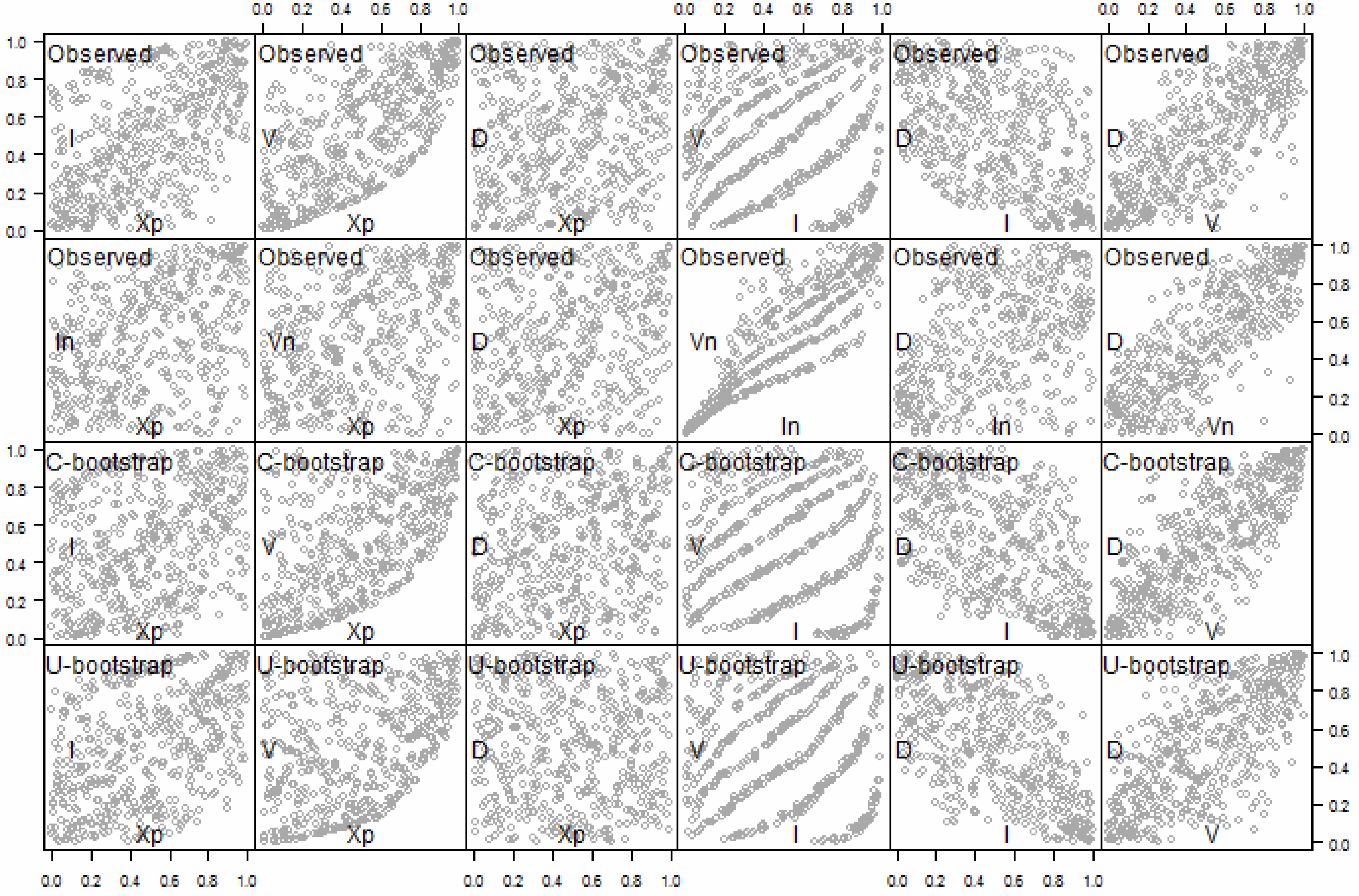

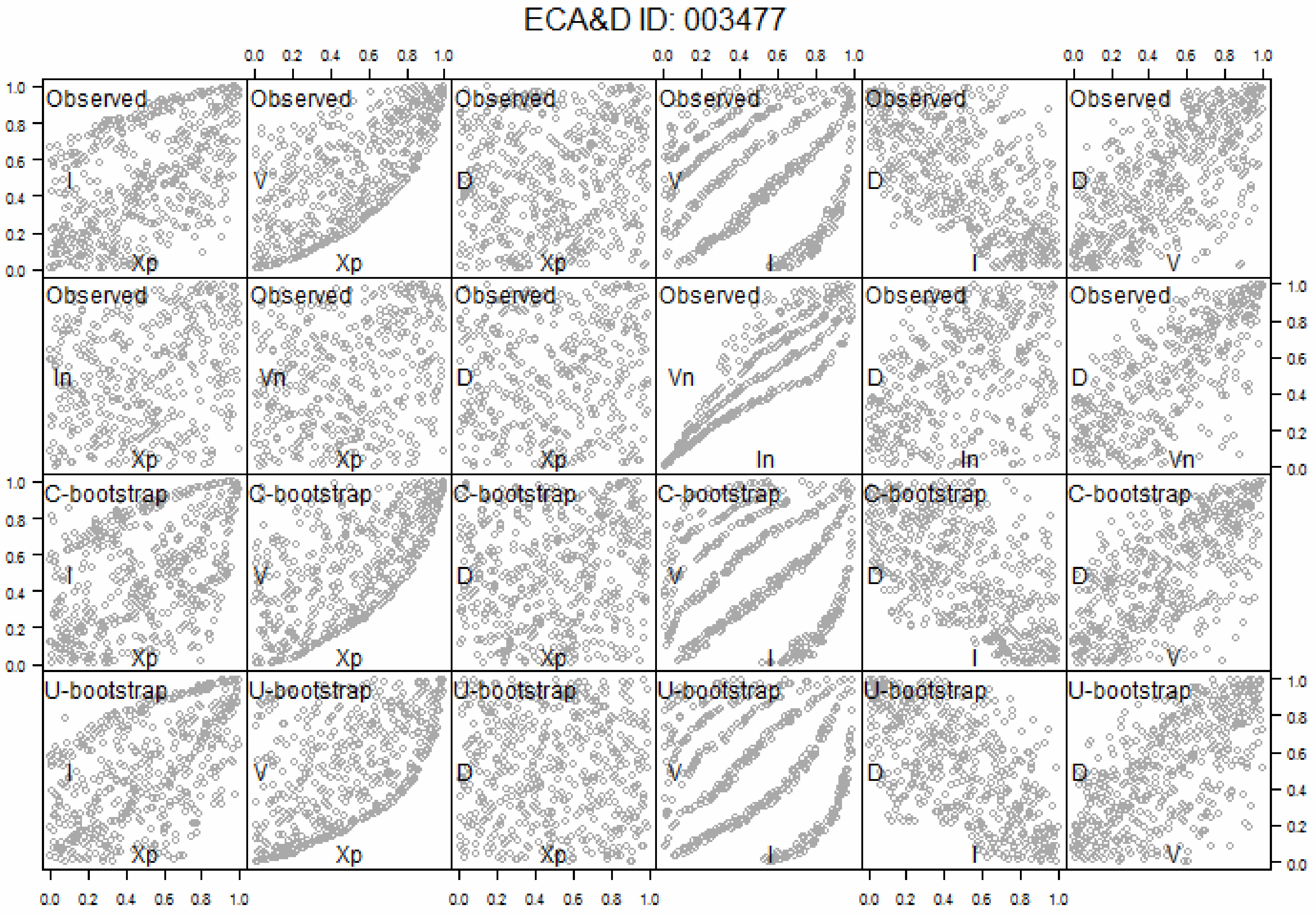

ECA&D ID: 003477
Observed
C-bootstrap
U-bootstrap
Xp
I
V
D
In
Vn
0.0
0.2
0.4
0.6
0.8
1.0

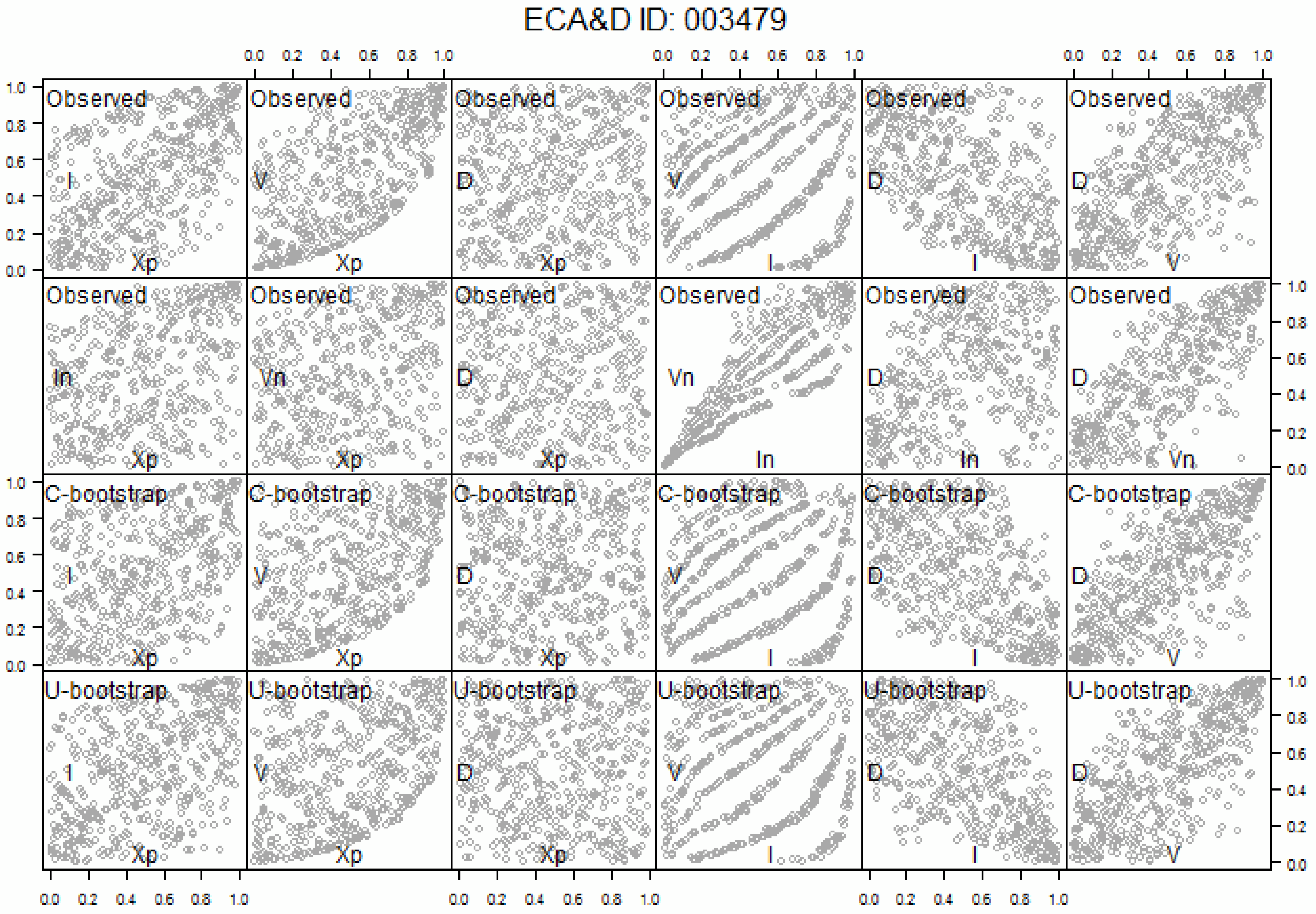

ECA&D ID: 003479
Observed
C-bootstrap
U-bootstrap
Xp
I
V
D
In
Vn
0.0
0.2
0.4
0.6
0.8
1.0

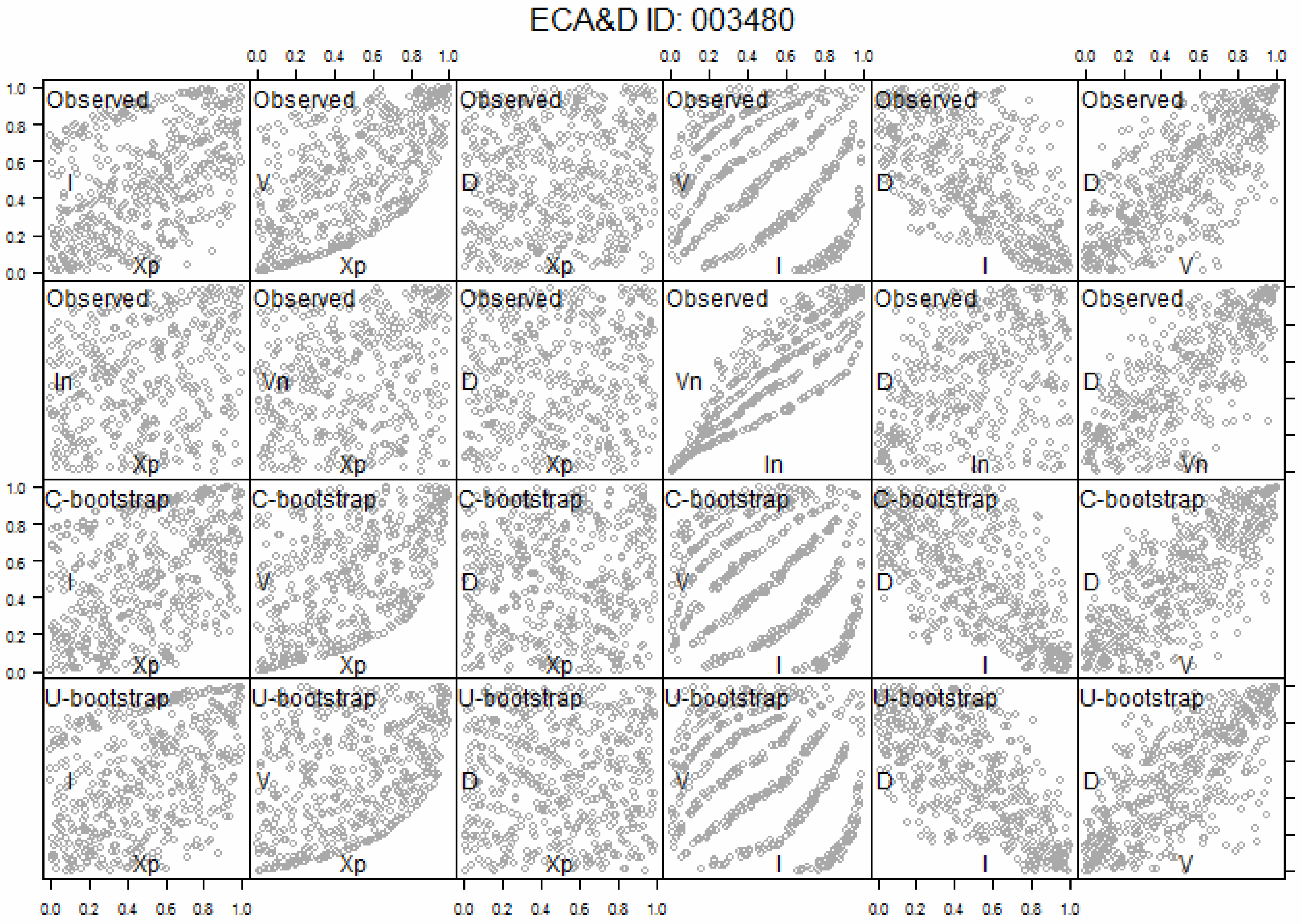
ECA&D ID: 003480
Observed
C-bootstrap
U-bootstrap
Xp
I
V
D
In
Vn
0.0
0.2
0.4
0.6
0.8
1.0

ECA&D ID: 003481

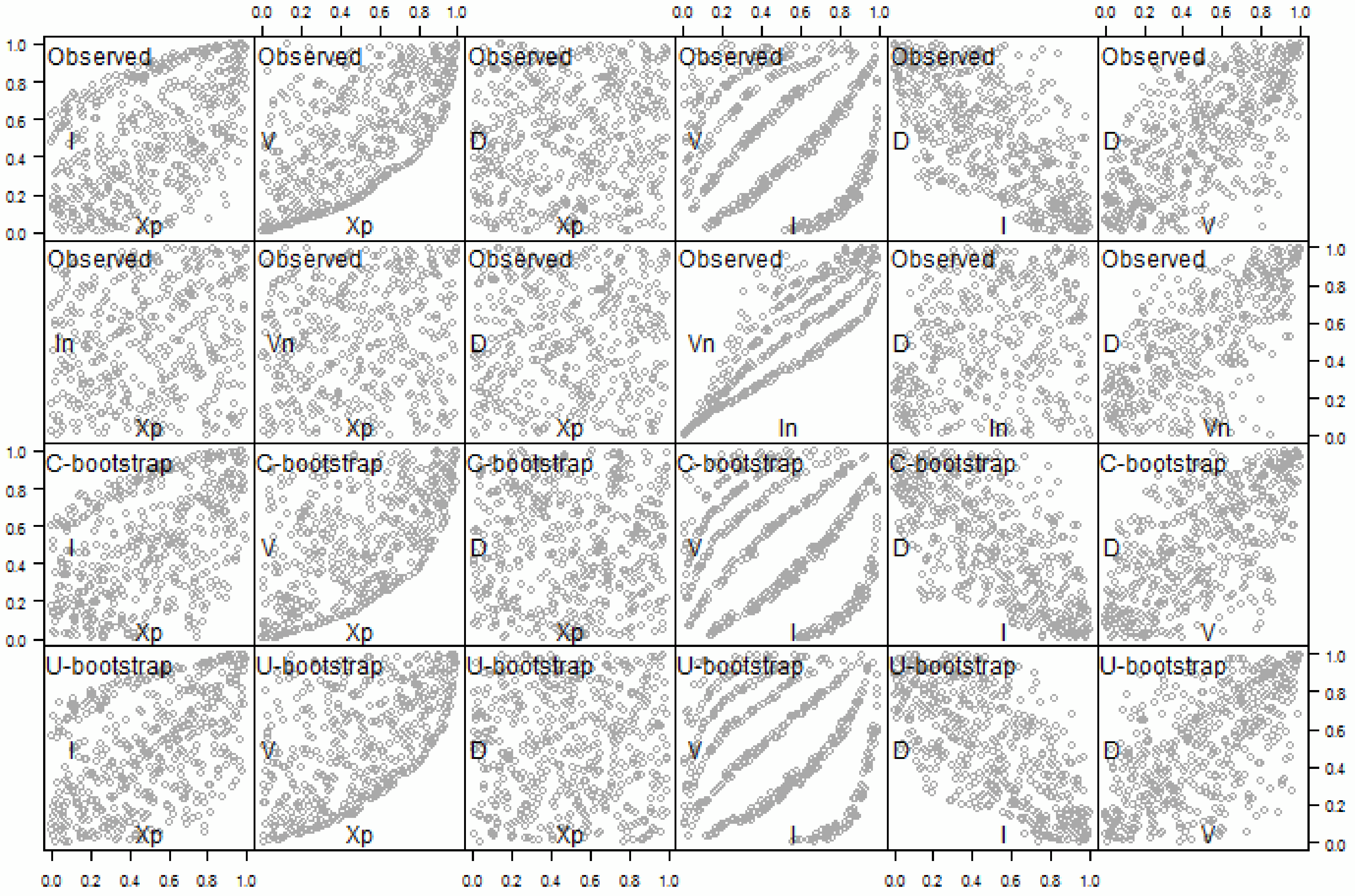

ECA&D ID: 003482

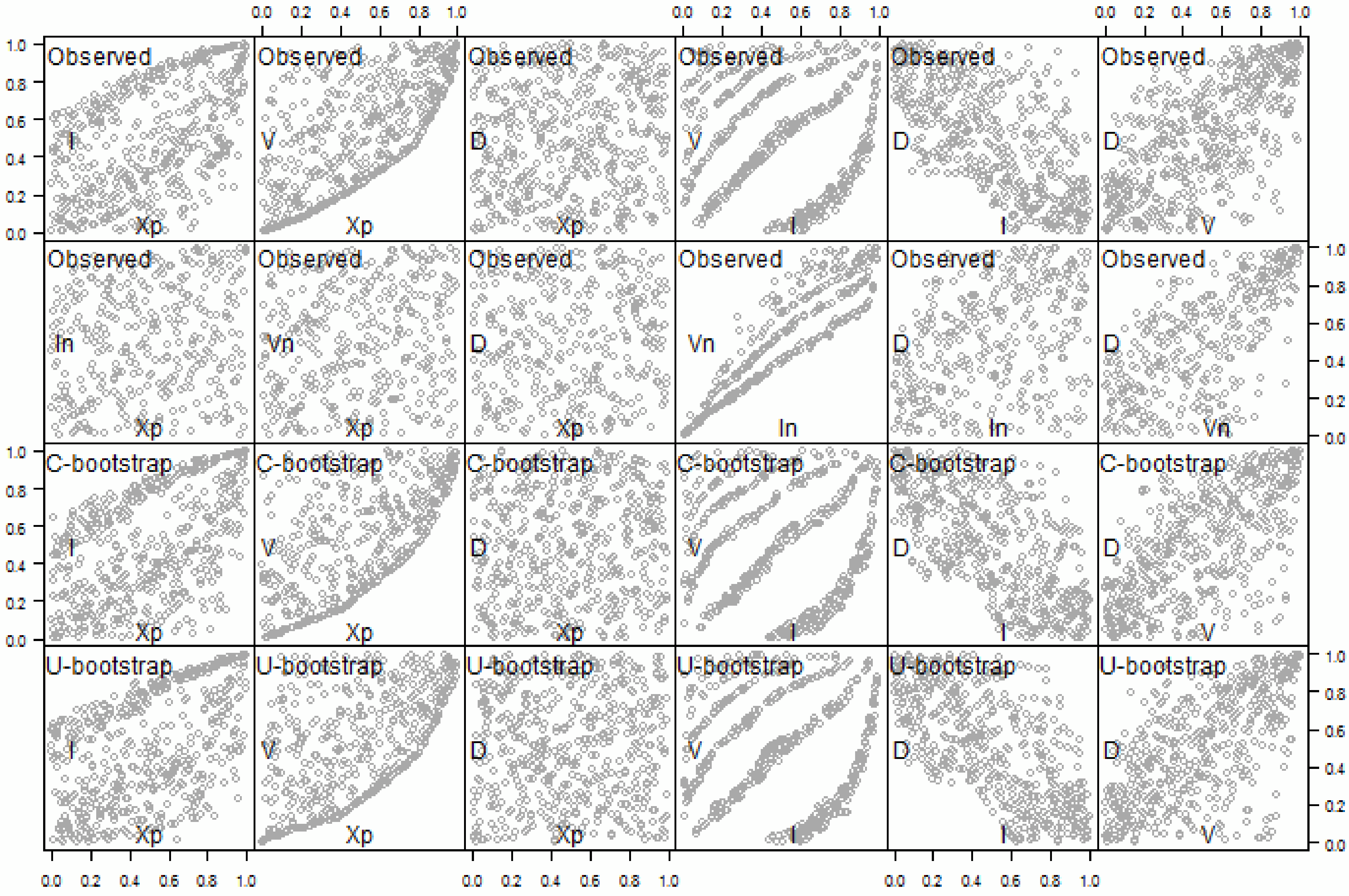

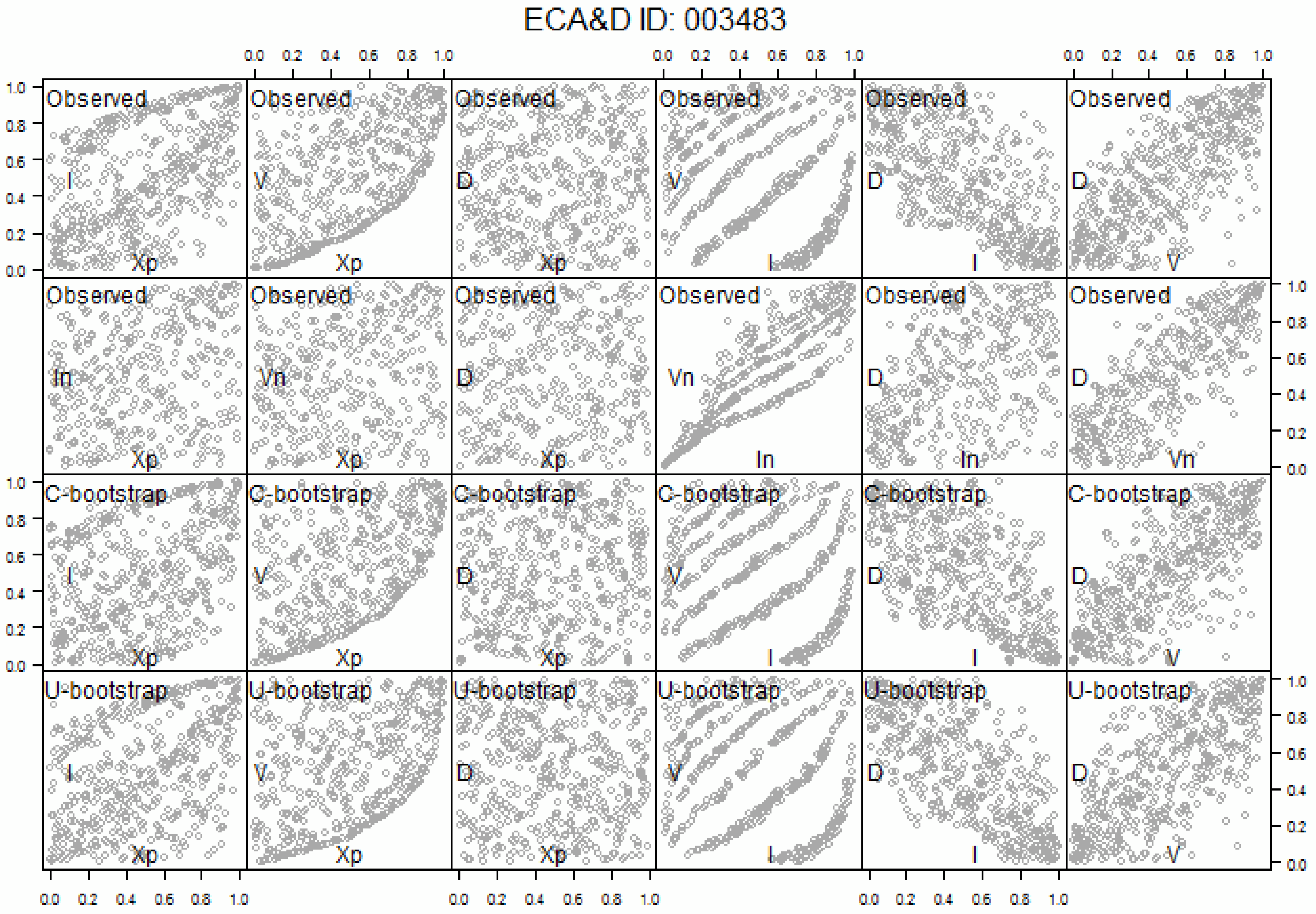
ECA&D ID: 003483
Observed
C-bootstrap
U-bootstrap
Xp
I
V
D
In
Vn
0.0
0.2
0.4
0.6
0.8
1.0

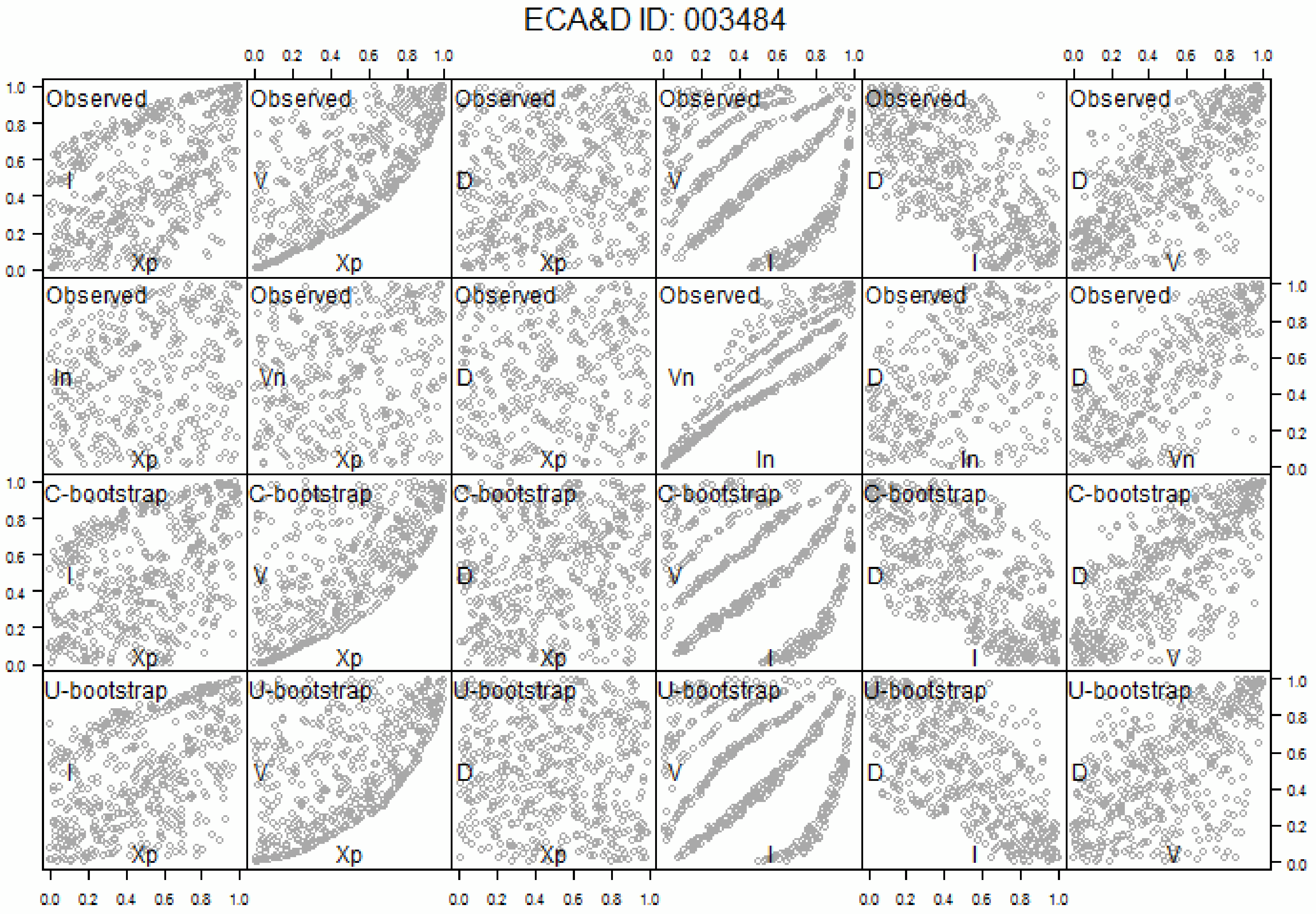
ECA&D ID: 003484
Observed
C-bootstrap
U-bootstrap
Xp
I
V
D
In
Vn
0.0
0.2
0.4
0.6
0.8
1.0

ECA&D ID: 003485

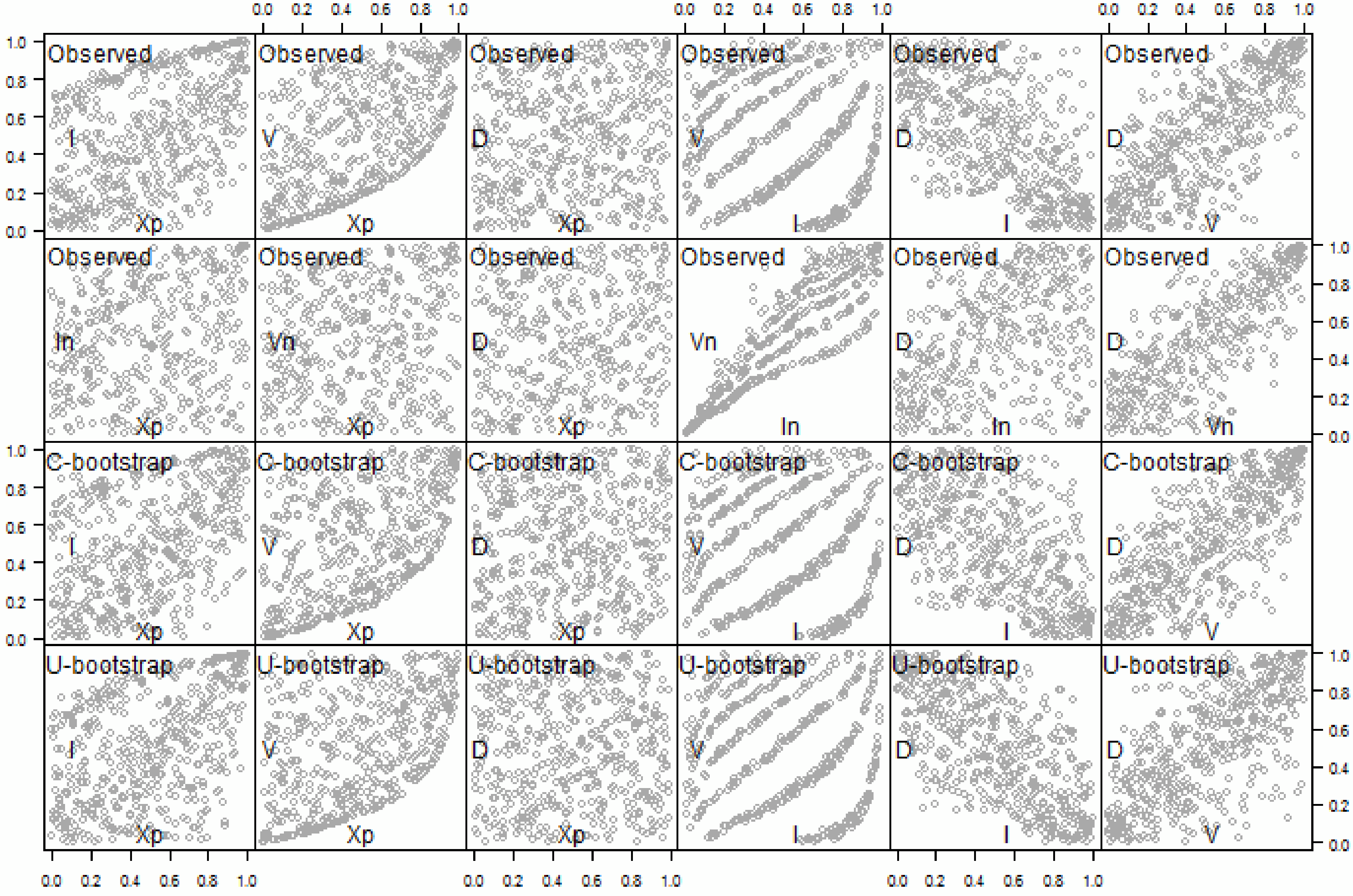

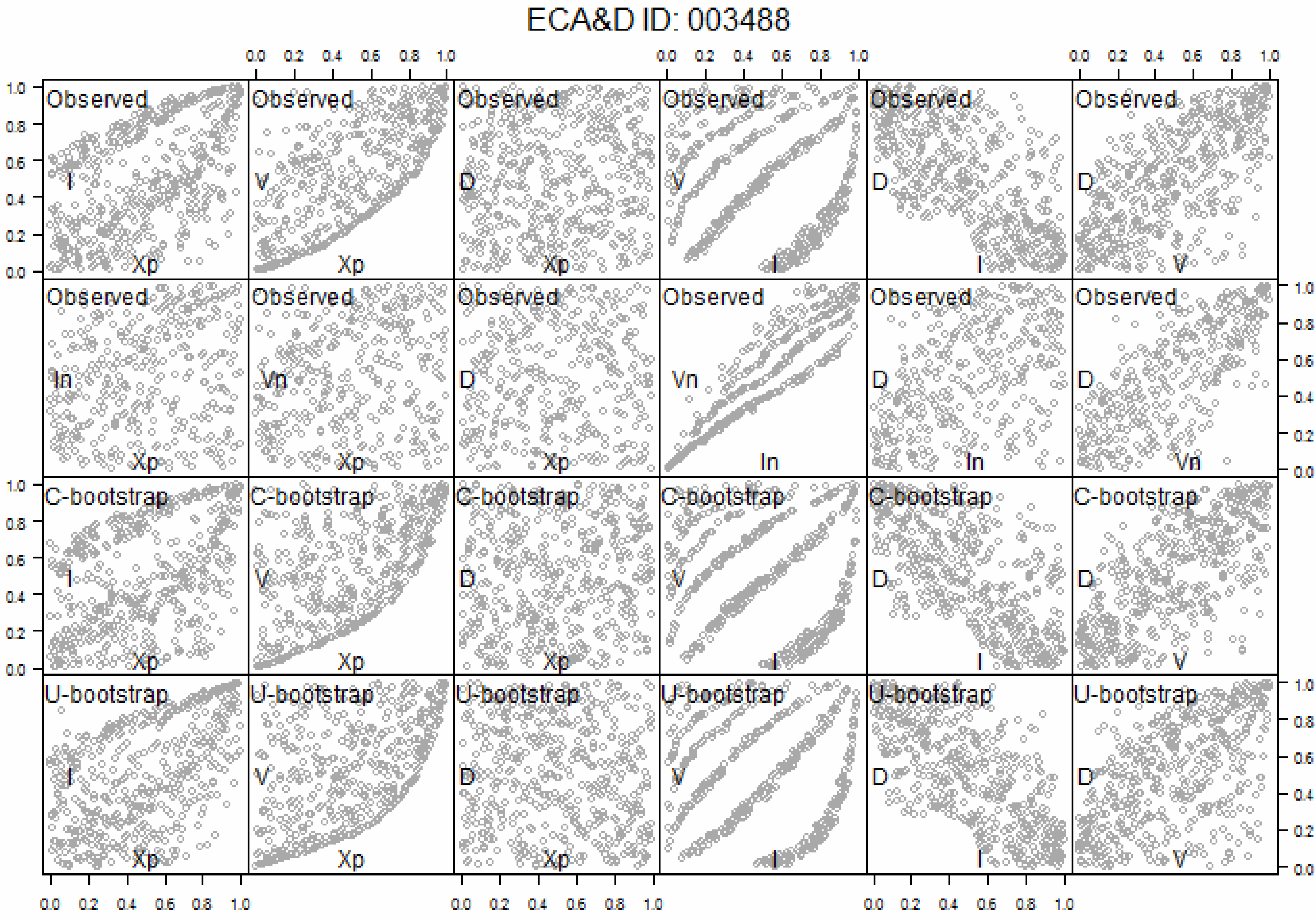

ECA&D ID: 003488
Observed
C-bootstrap
U-bootstrap
Xp
I
V
D
In
Vn
0.0
0.2
0.4
0.6
0.8
1.0

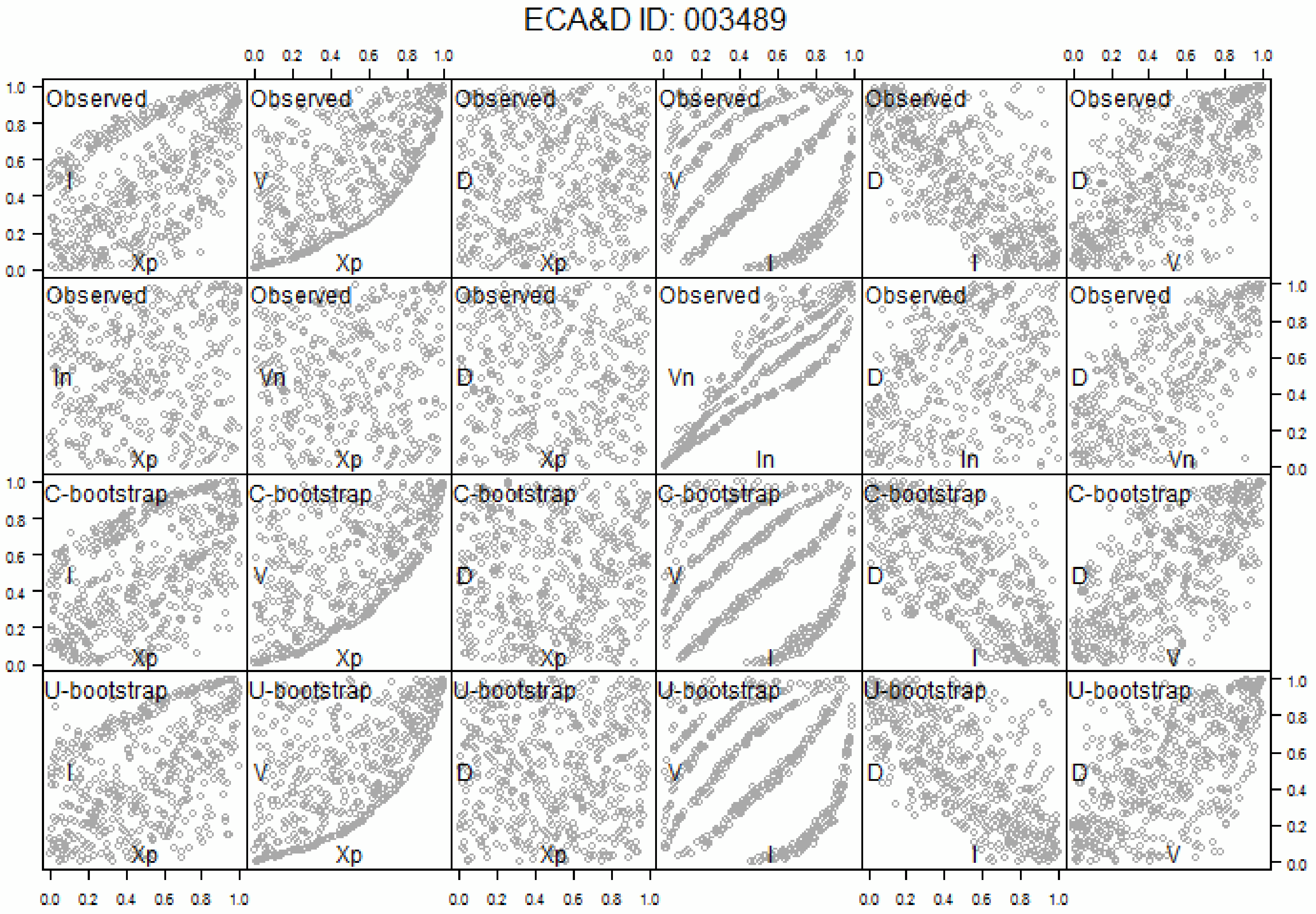
ECA&D ID: 003489
Observed
C-bootstrap
U-bootstrap
Xp
I
V
D
In
Vn
0.0
0.2
0.4
0.6
0.8
1.0

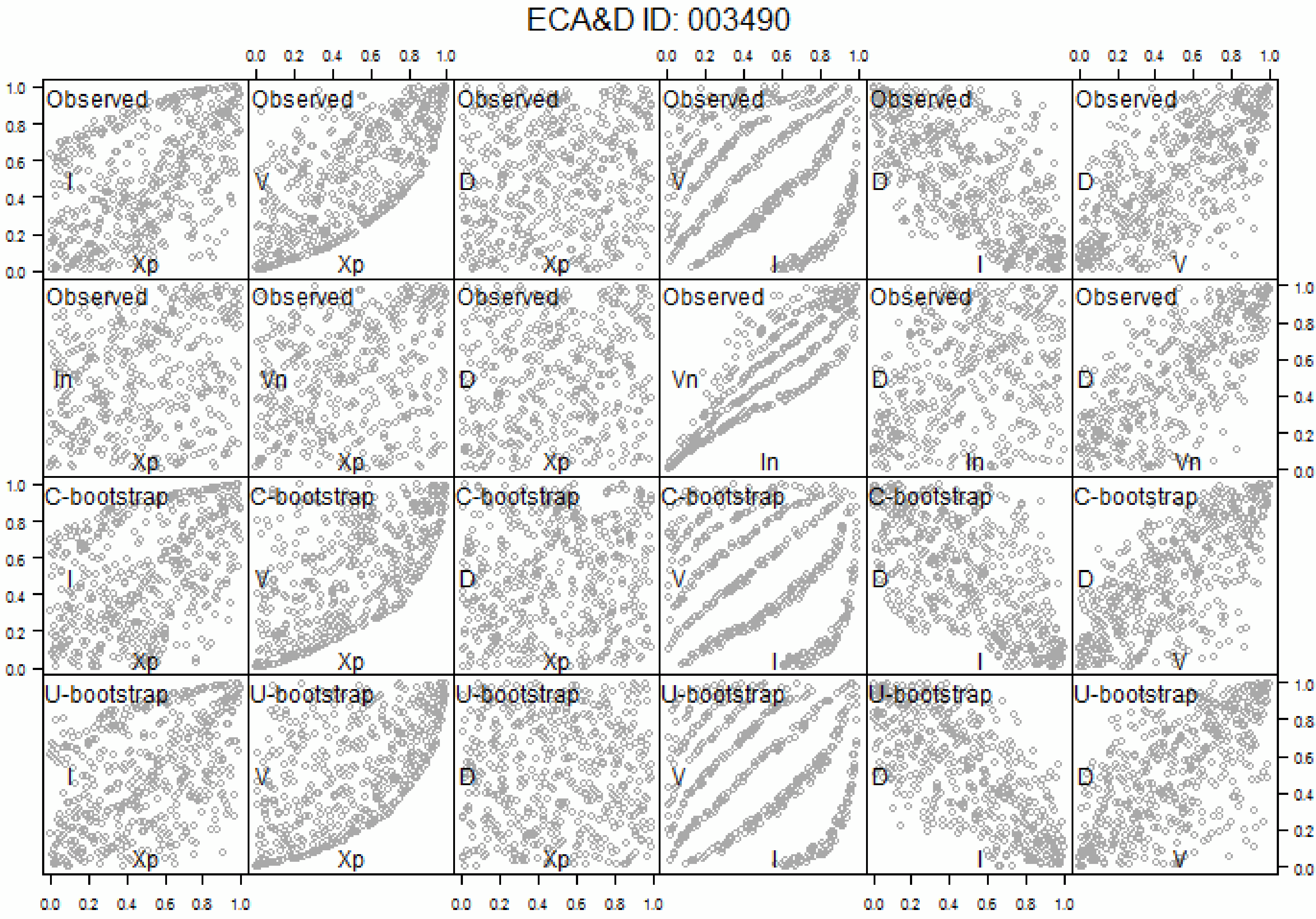
ECA&D ID: 003490
Observed
C-bootstrap
U-bootstrap
Xp
I
V
D
In
Vn
0.0
0.2
0.4
0.6
0.8
1.0

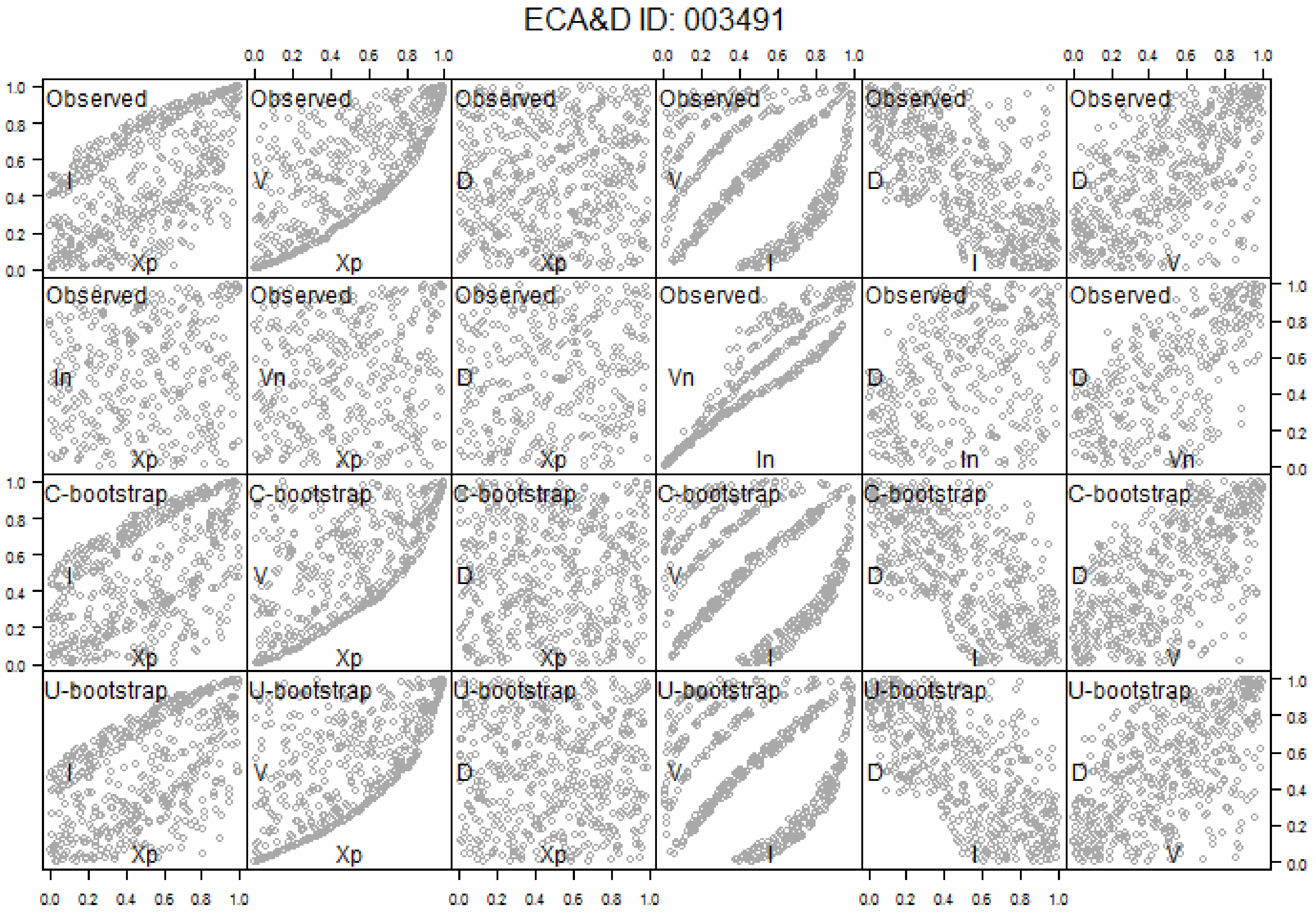

ECA&D ID: 003491
Observed
C-bootstrap
U-bootstrap
Xp
I
V
D
In
Vn
0.0
0.2
0.4
0.6
0.8
1.0

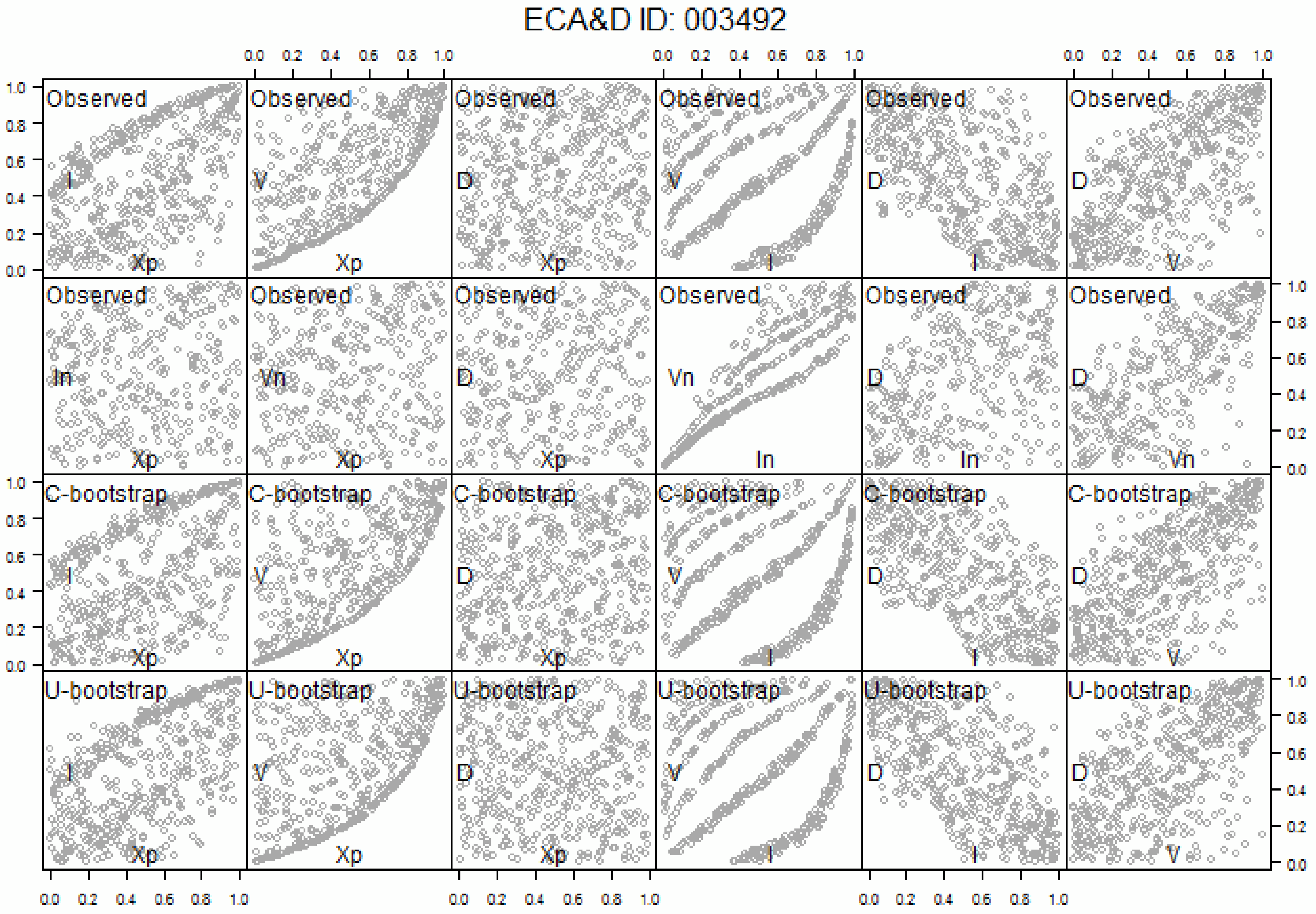
ECA&D ID: 003492
Observed
C-bootstrap
U-bootstrap
Xp
I
V
D
In
Vn
0.0
0.2
0.4
0.6
0.8
1.0

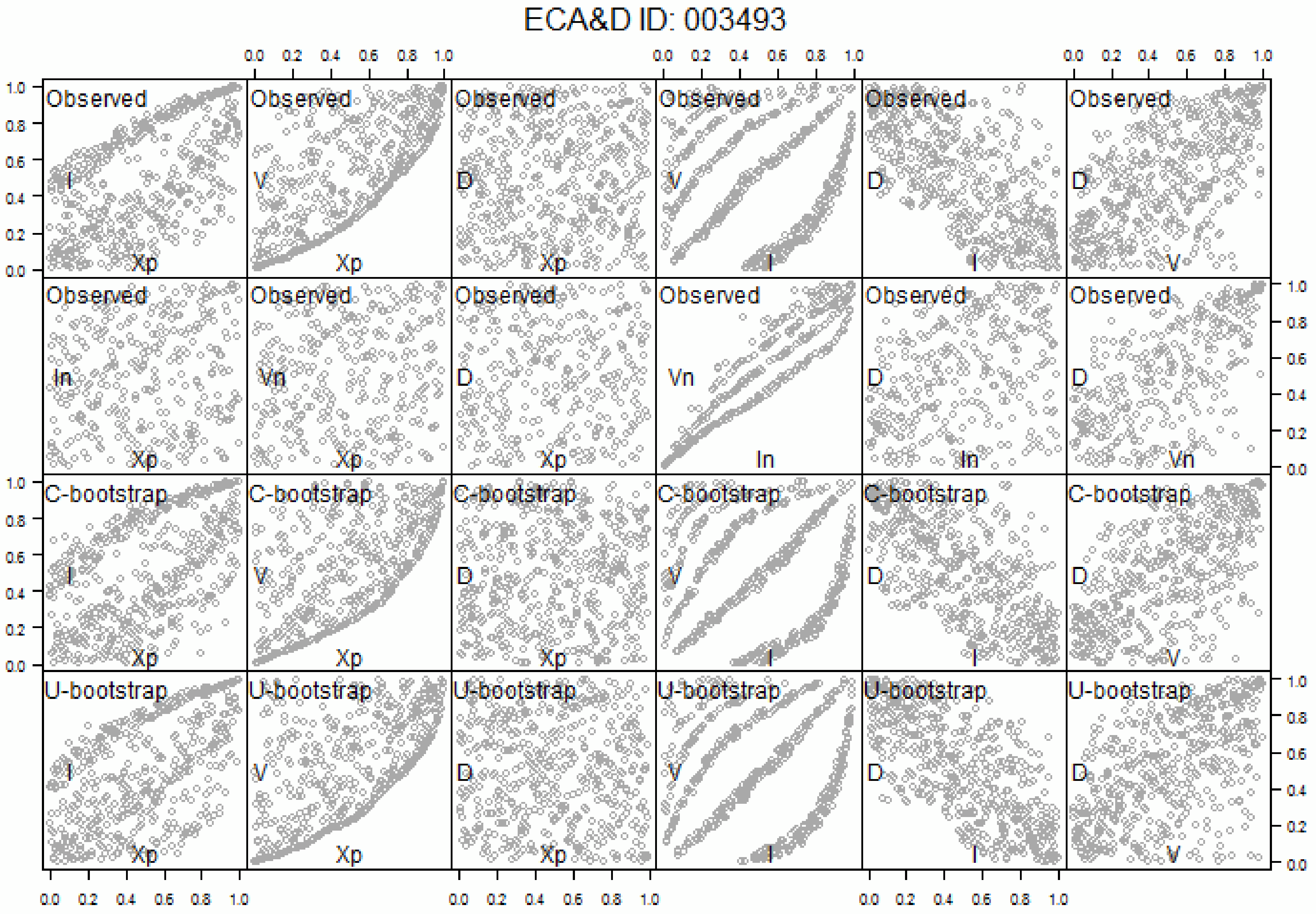

ECA&D ID: 003493
Observed
Xp
I
V
D
In
Vn
C-bootstrap
U-bootstrap
0.0
0.2
0.4
0.6
0.8
1.0

ECA&D ID: 003497

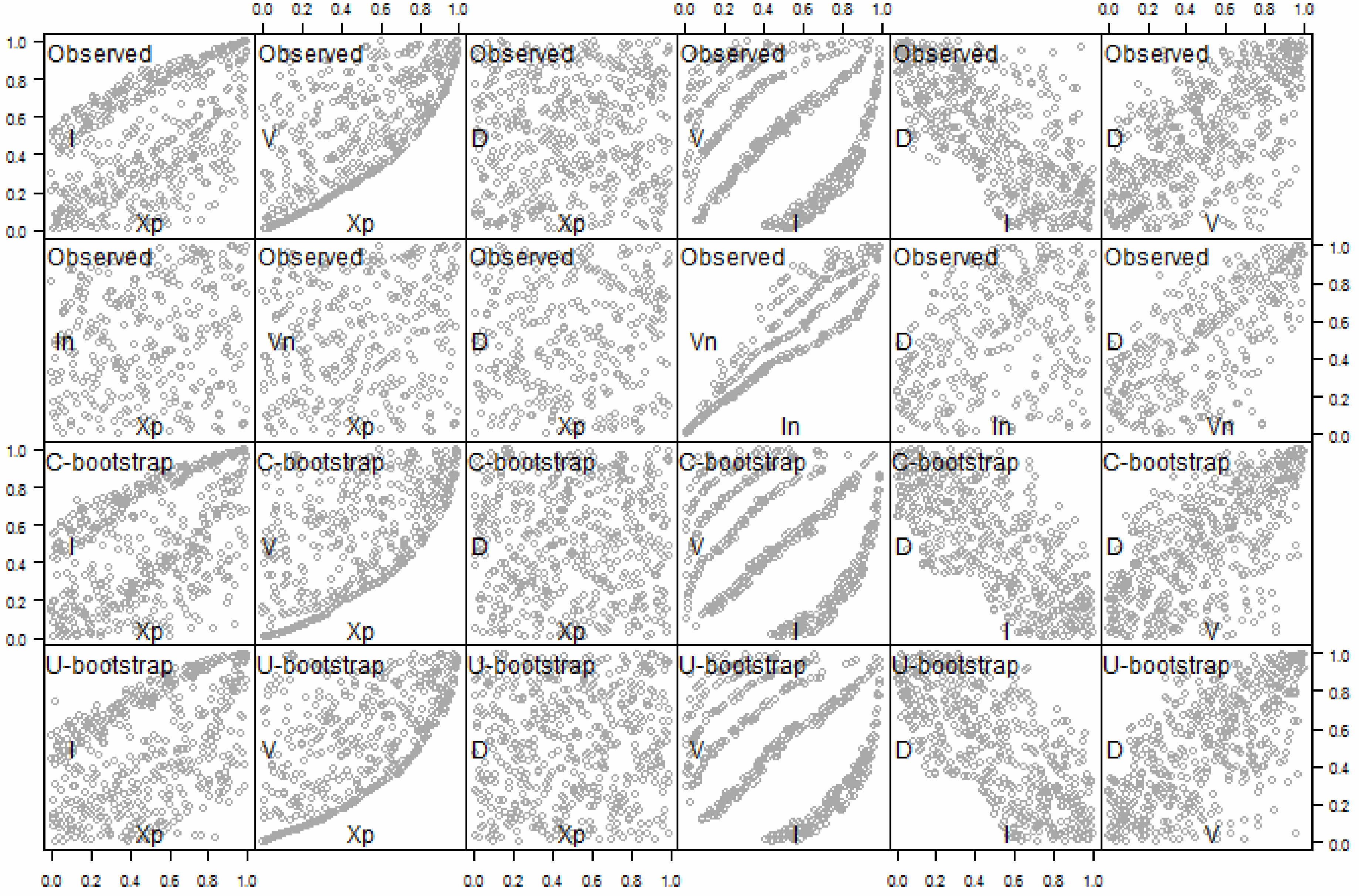

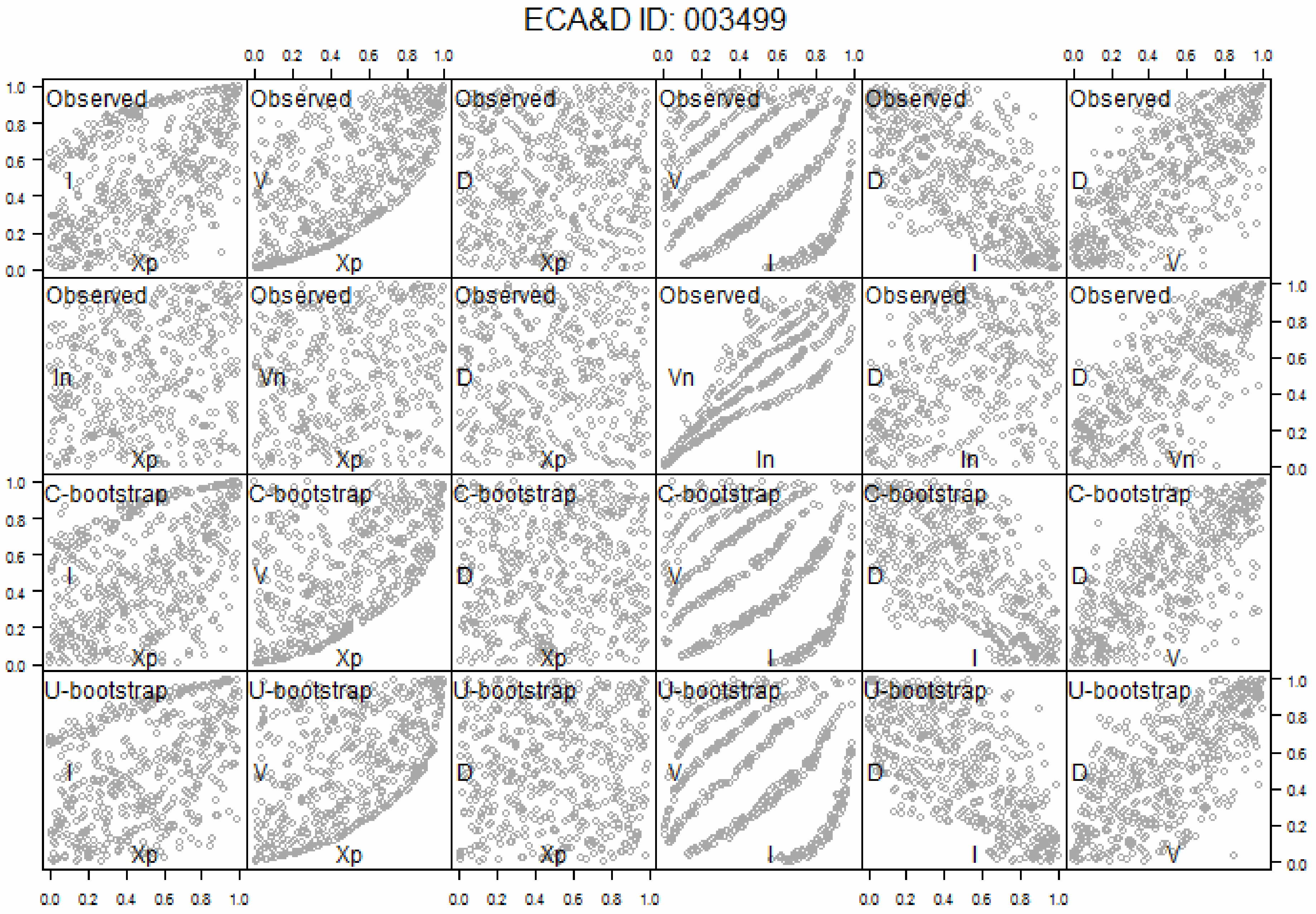
ECA&D ID: 003499
Observed
C-bootstrap
U-bootstrap
Xp
I
V
D
In
Vn
0.0
0.2
0.4
0.6
0.8
1.0

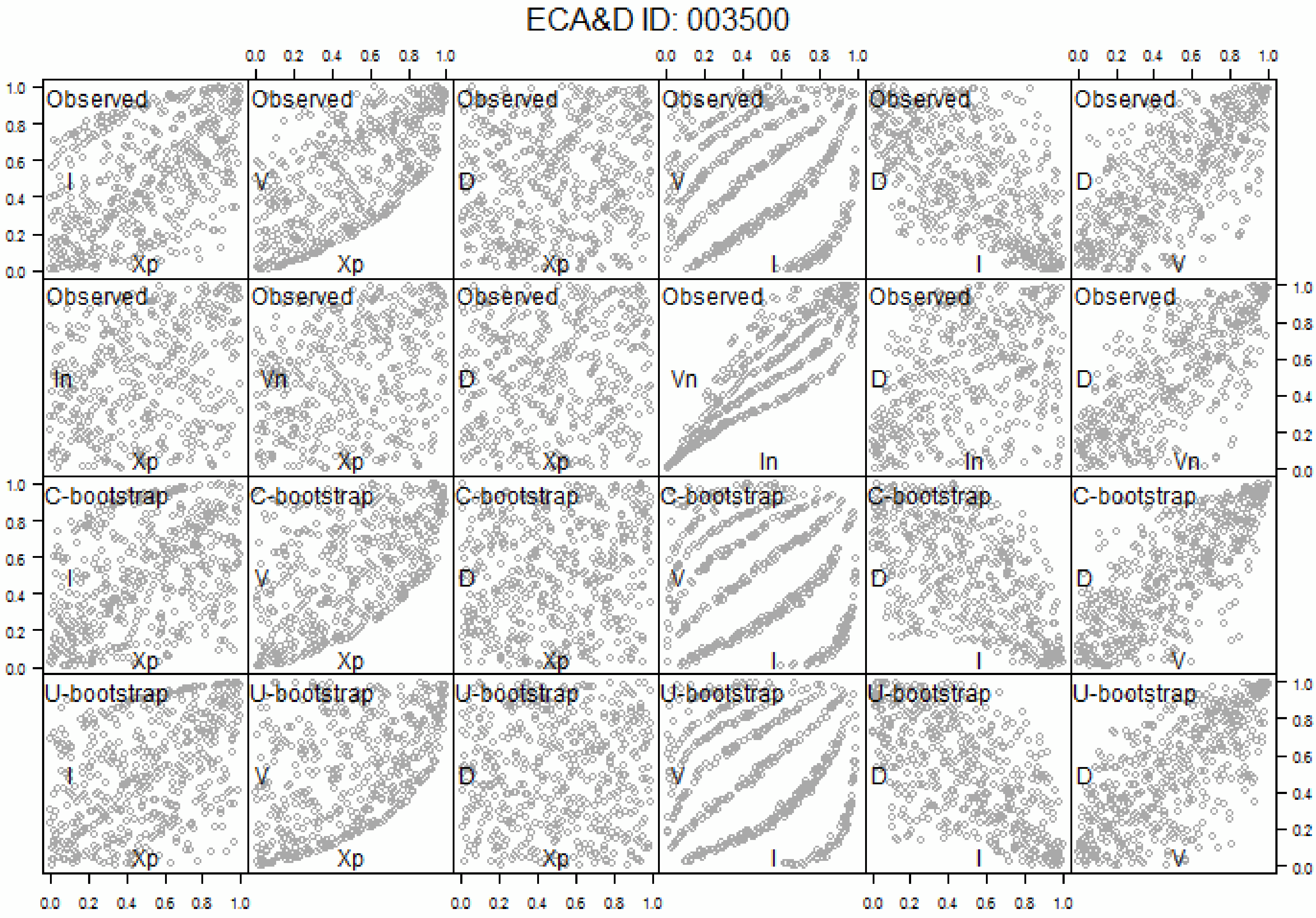

ECA&D ID: 003500
Observed
C-bootstrap
U-bootstrap
Xp
I
V
D
In
Vn
0.0
0.2
0.4
0.6
0.8
1.0

ECA&D ID: 003502

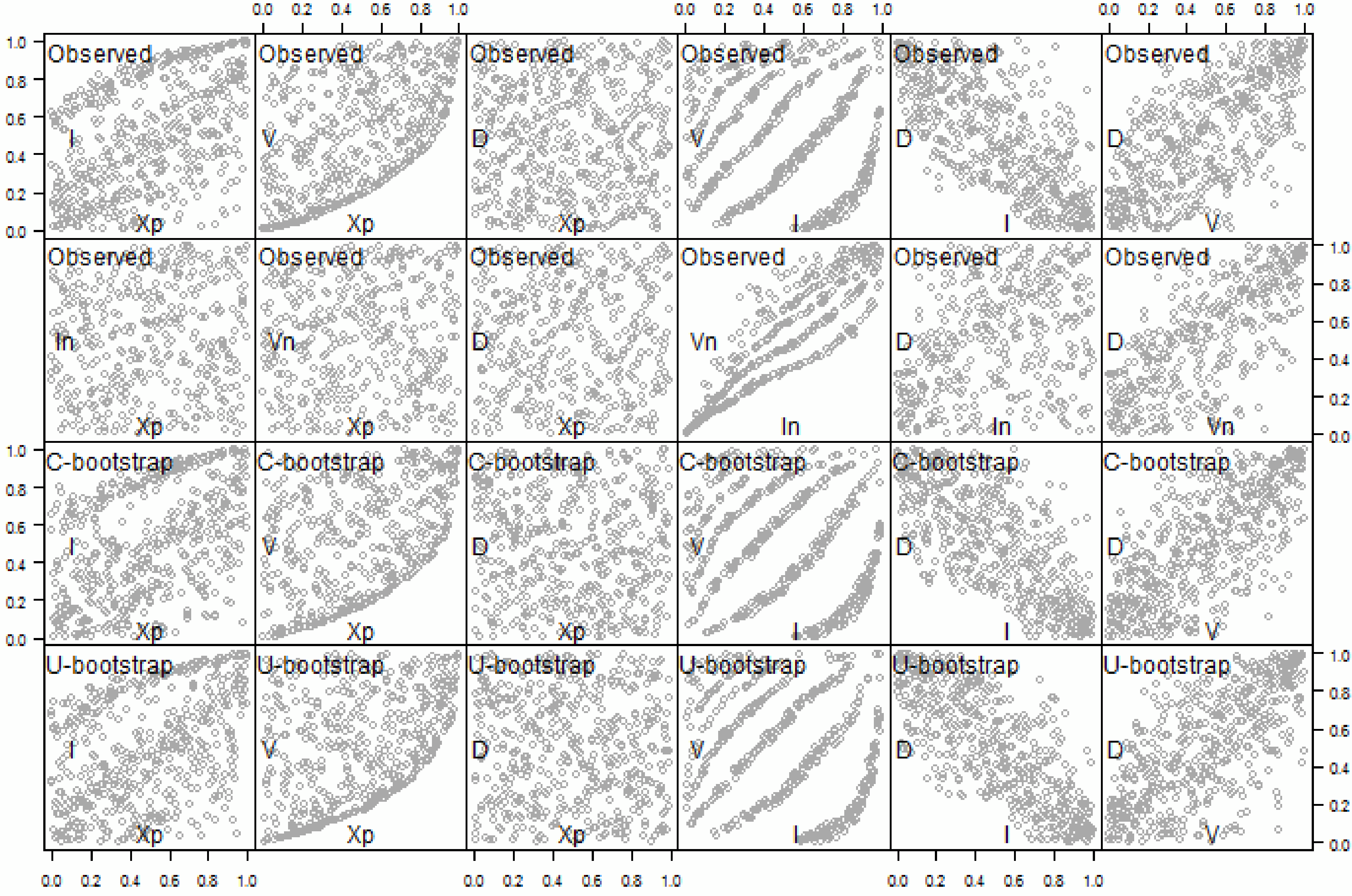

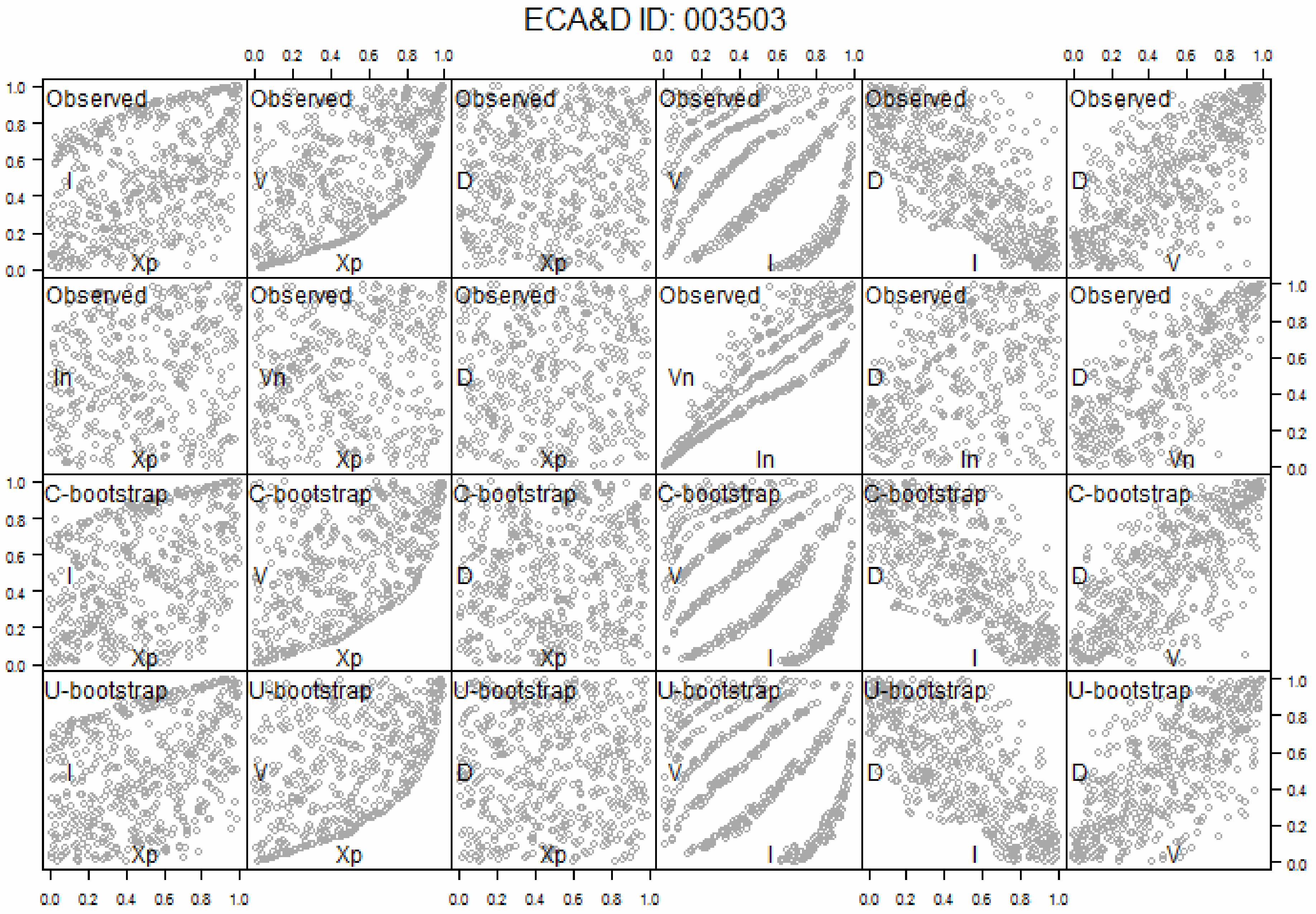
ECA&D ID: 003503
Observed
C-bootstrap
U-bootstrap
Xp
I
V
D
In
Vn
0.0
0.2
0.4
0.6
0.8
1.0

ECA&D ID: 003504

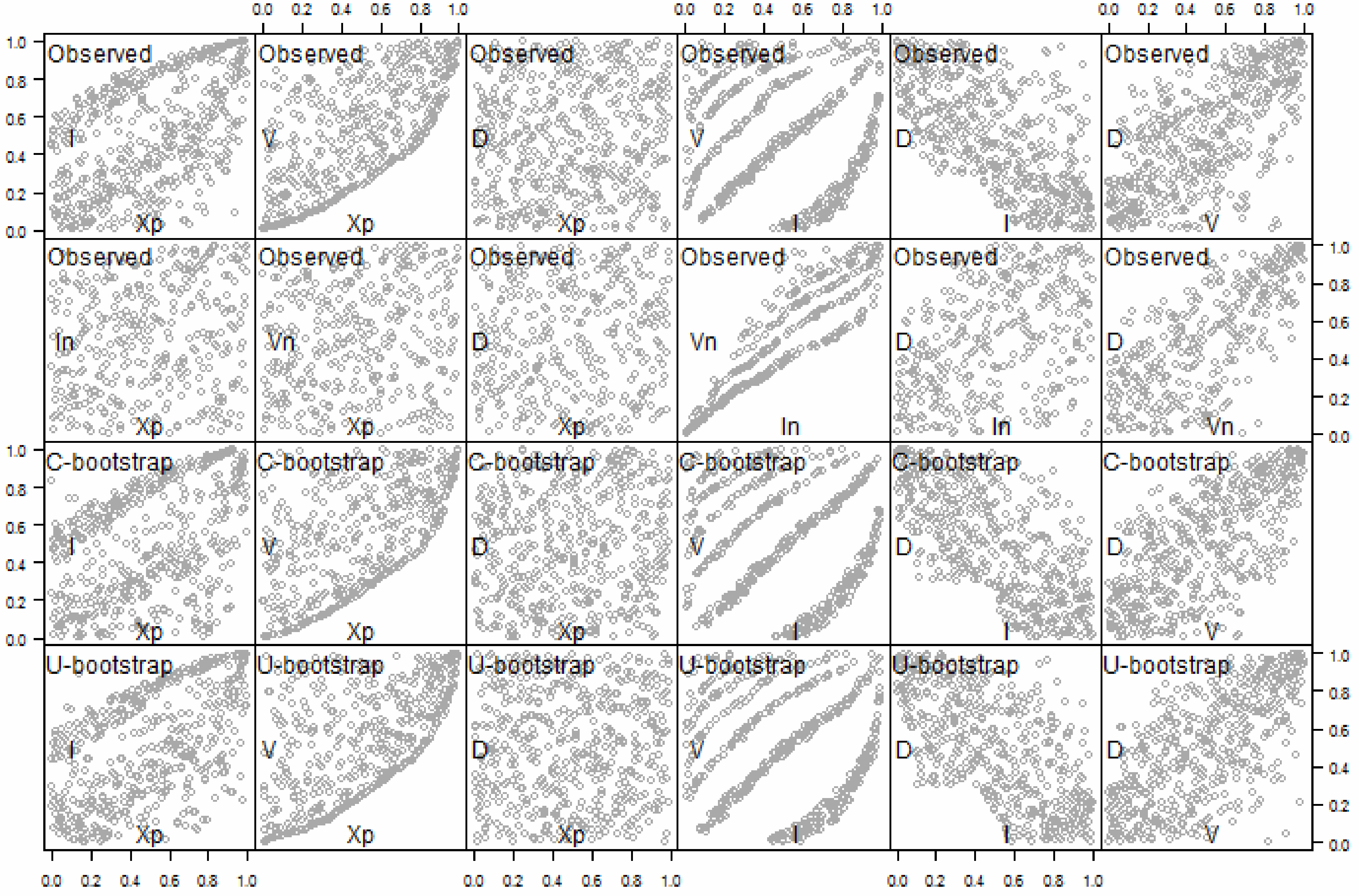

ECA&D ID: 003506

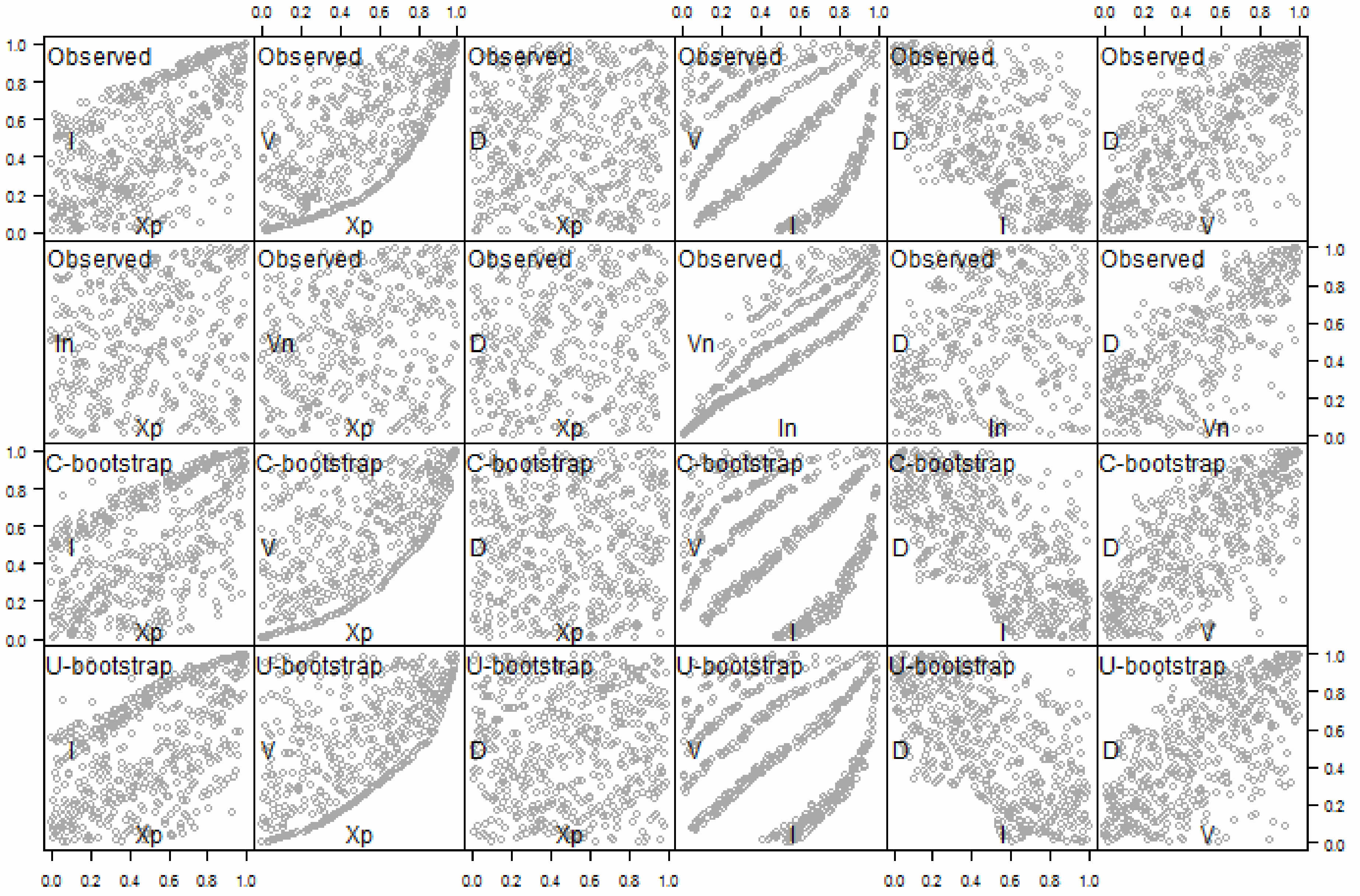

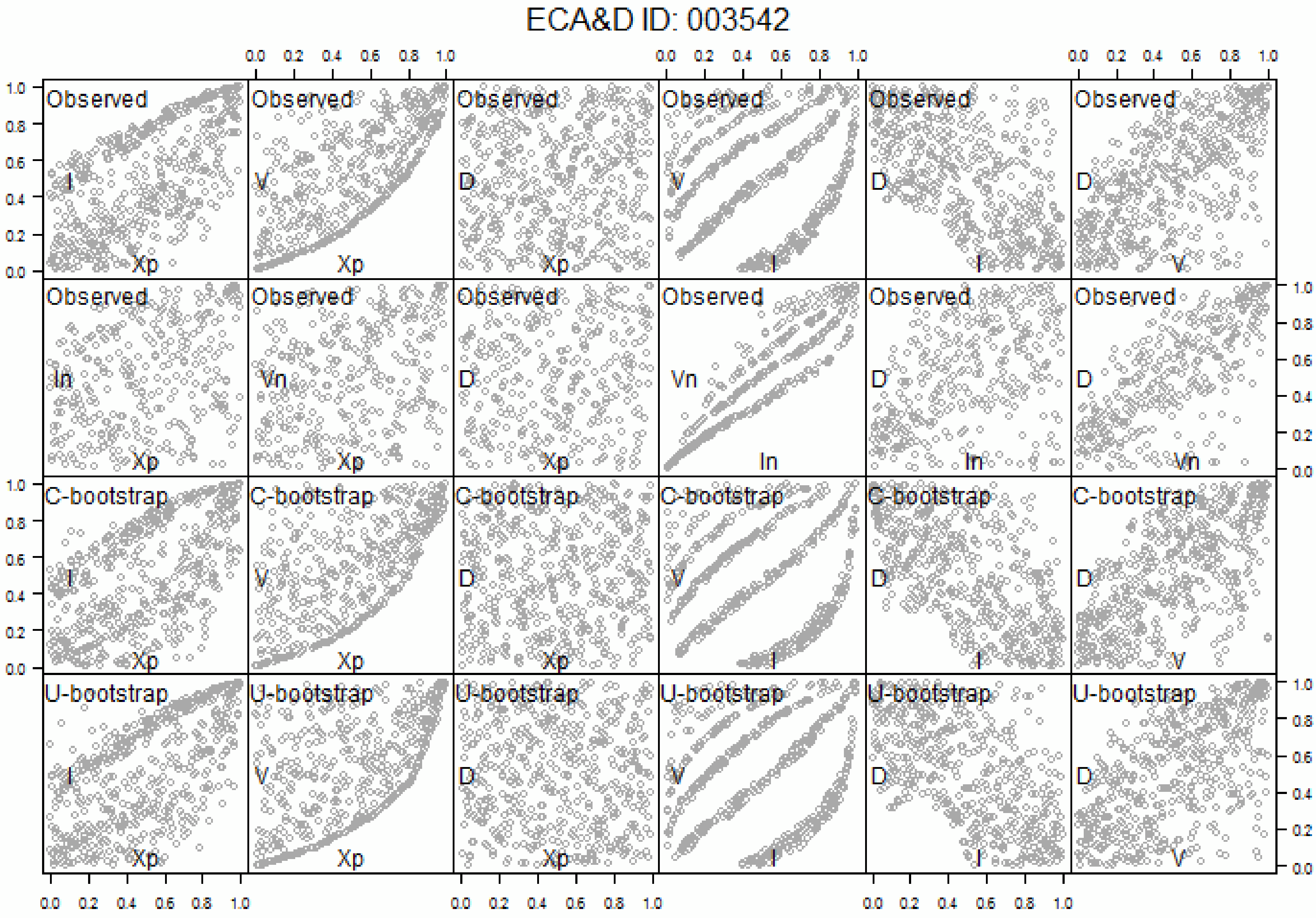
ECA&D ID: 003542
Observed
C-bootstrap
U-bootstrap
Xp
I
V
D
In
Vn
0.0
0.2
0.4
0.6
0.8
1.0

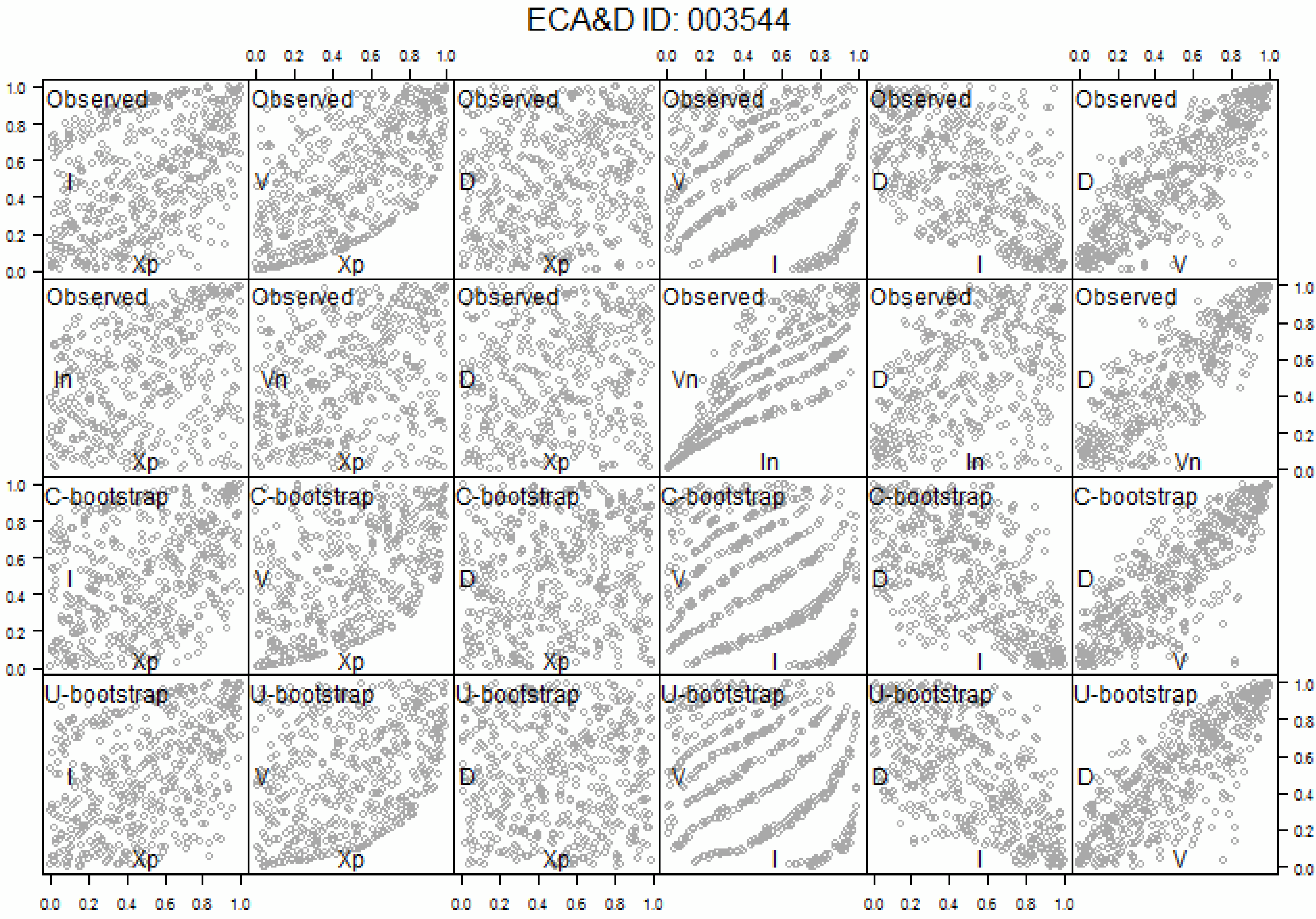

ECA&D ID: 003544
Observed
C-bootstrap
U-bootstrap
Xp
I
V
D
In
Vn
0.0
0.2
0.4
0.6
0.8
1.0

ECA&D ID: 003901

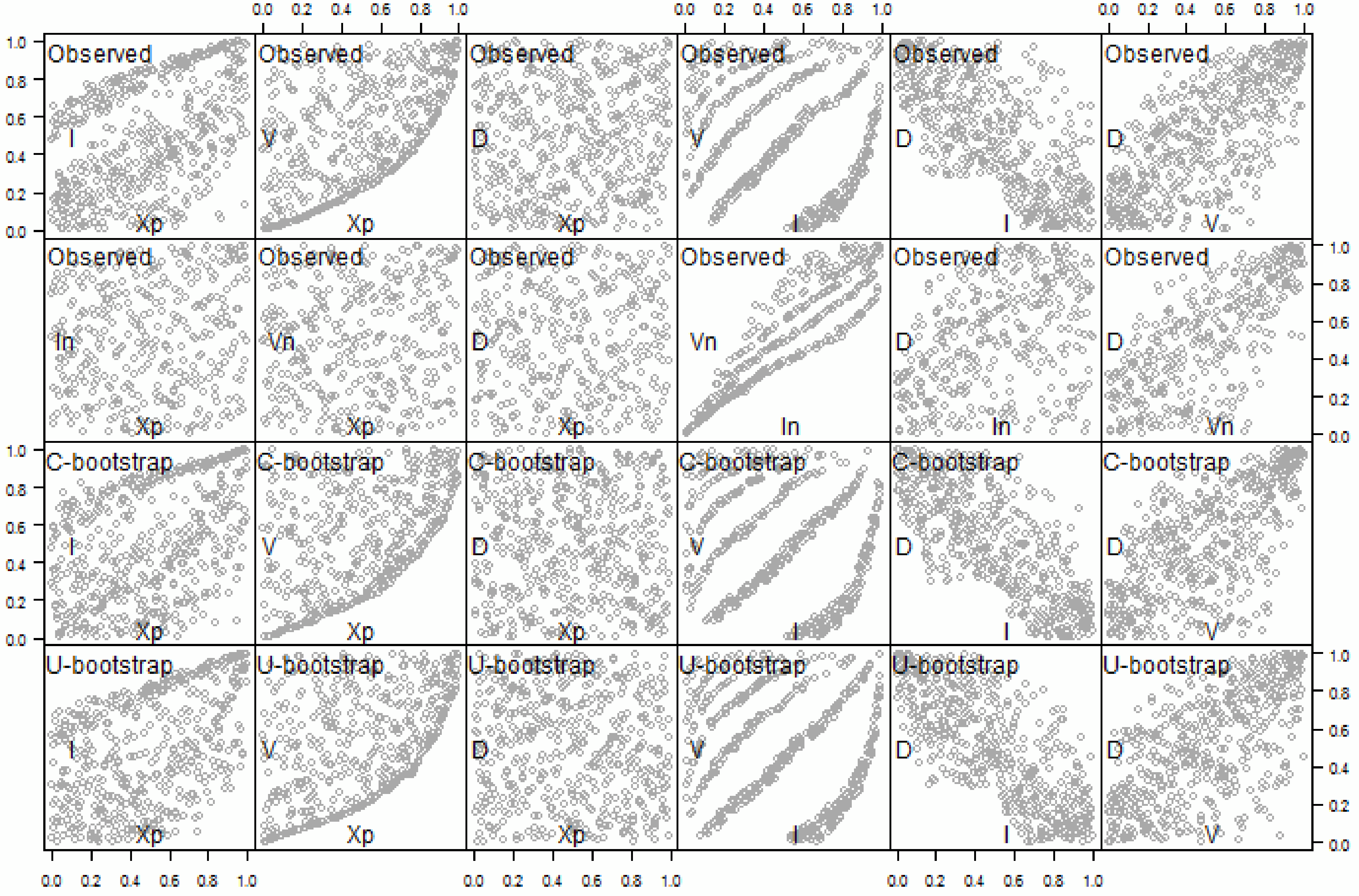

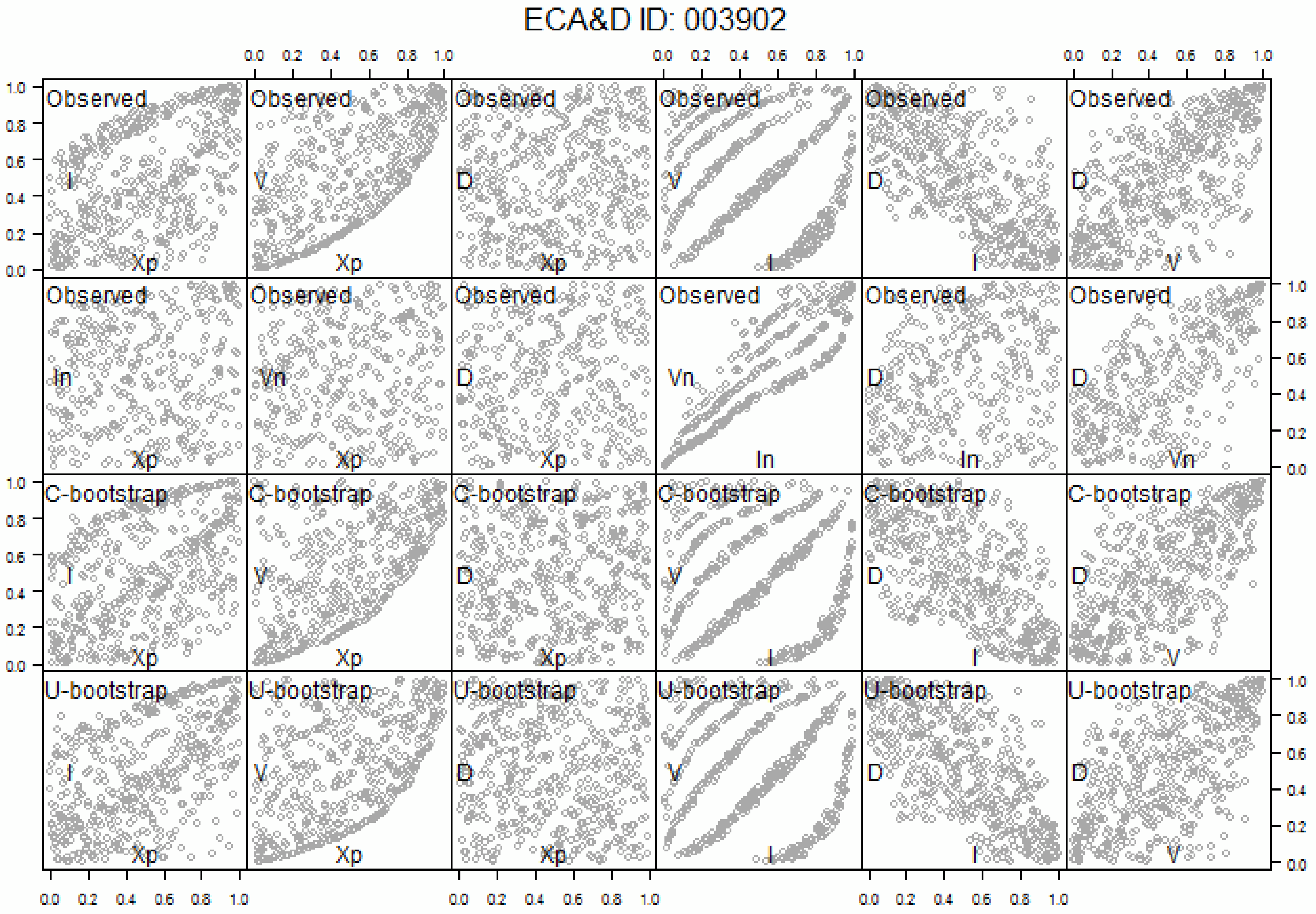
ECA&D ID: 003902
Observed
C-bootstrap
U-bootstrap
Xp
I
V
D
In
Vn
0.0
0.2
0.4
0.6
0.8
1.0

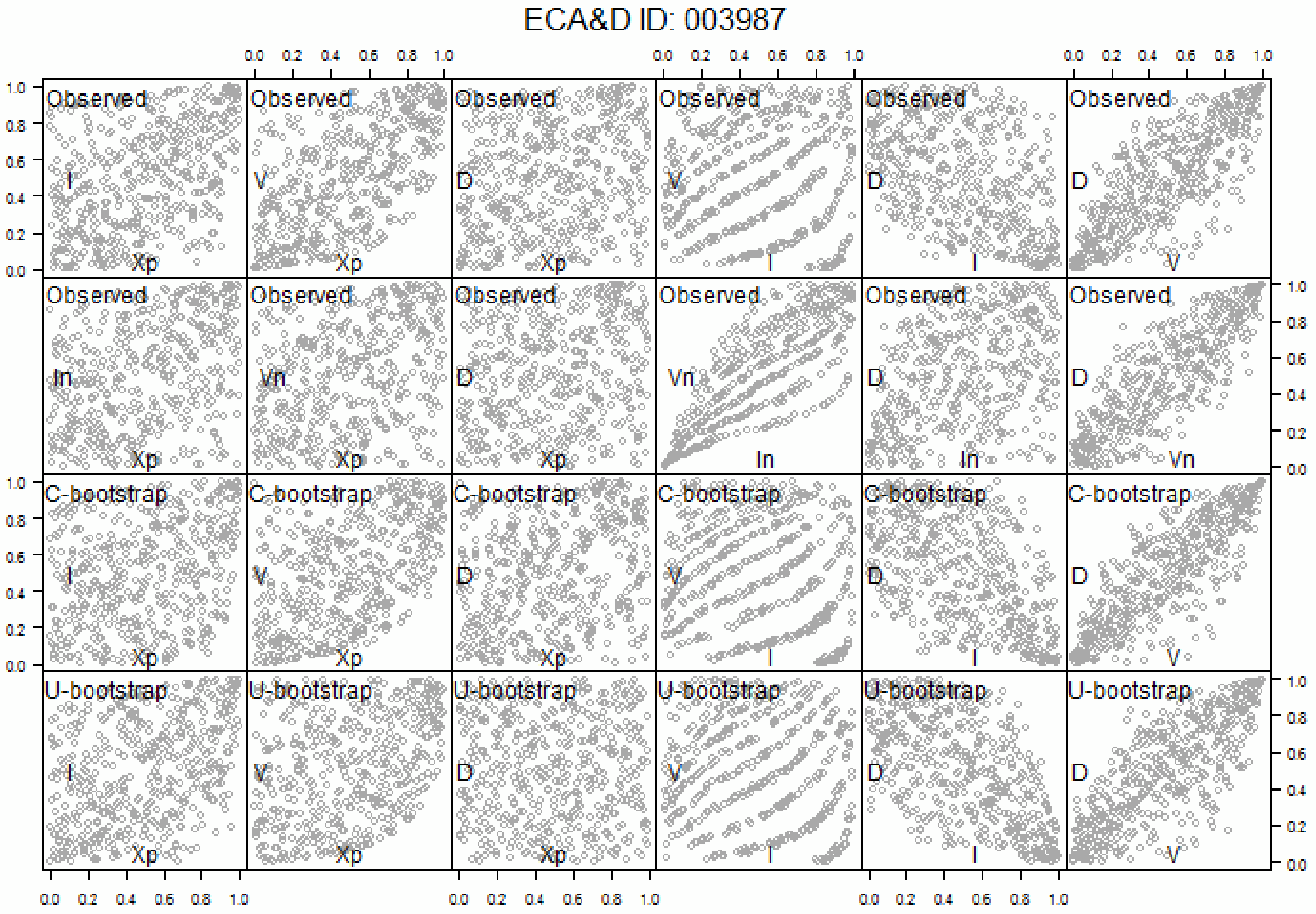
ECA&D ID: 003987
Observed
C-bootstrap
U-bootstrap
Xp
I
V
D
In
Vn
0.0
0.2
0.4
0.6
0.8
1.0

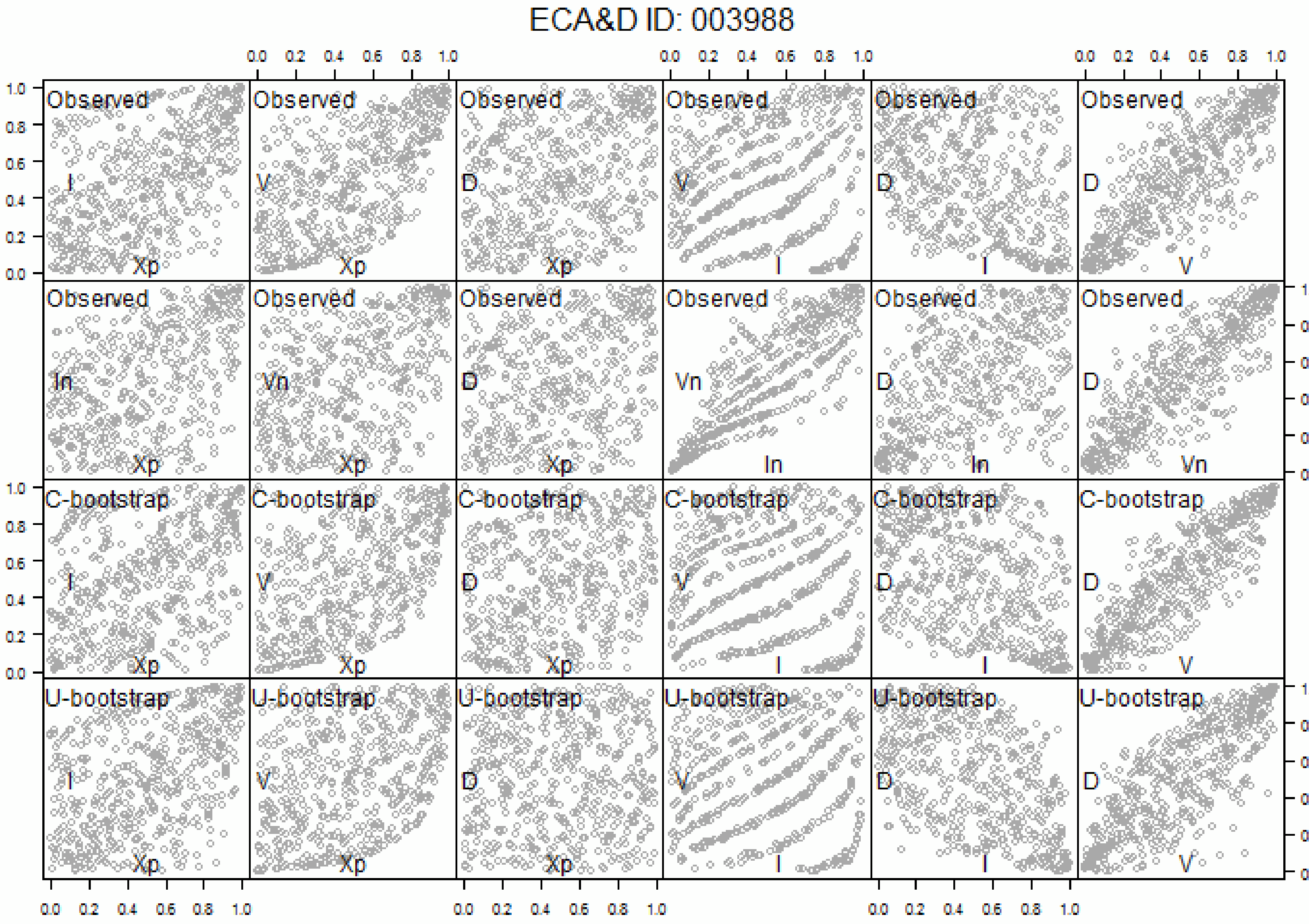
ECA&D ID: 003988
Observed
C-bootstrap
U-bootstrap
Xp
I
V
D
In
Vn
0.0
0.2
0.4
0.6
0.8
1.0

ECA&D ID: 003990

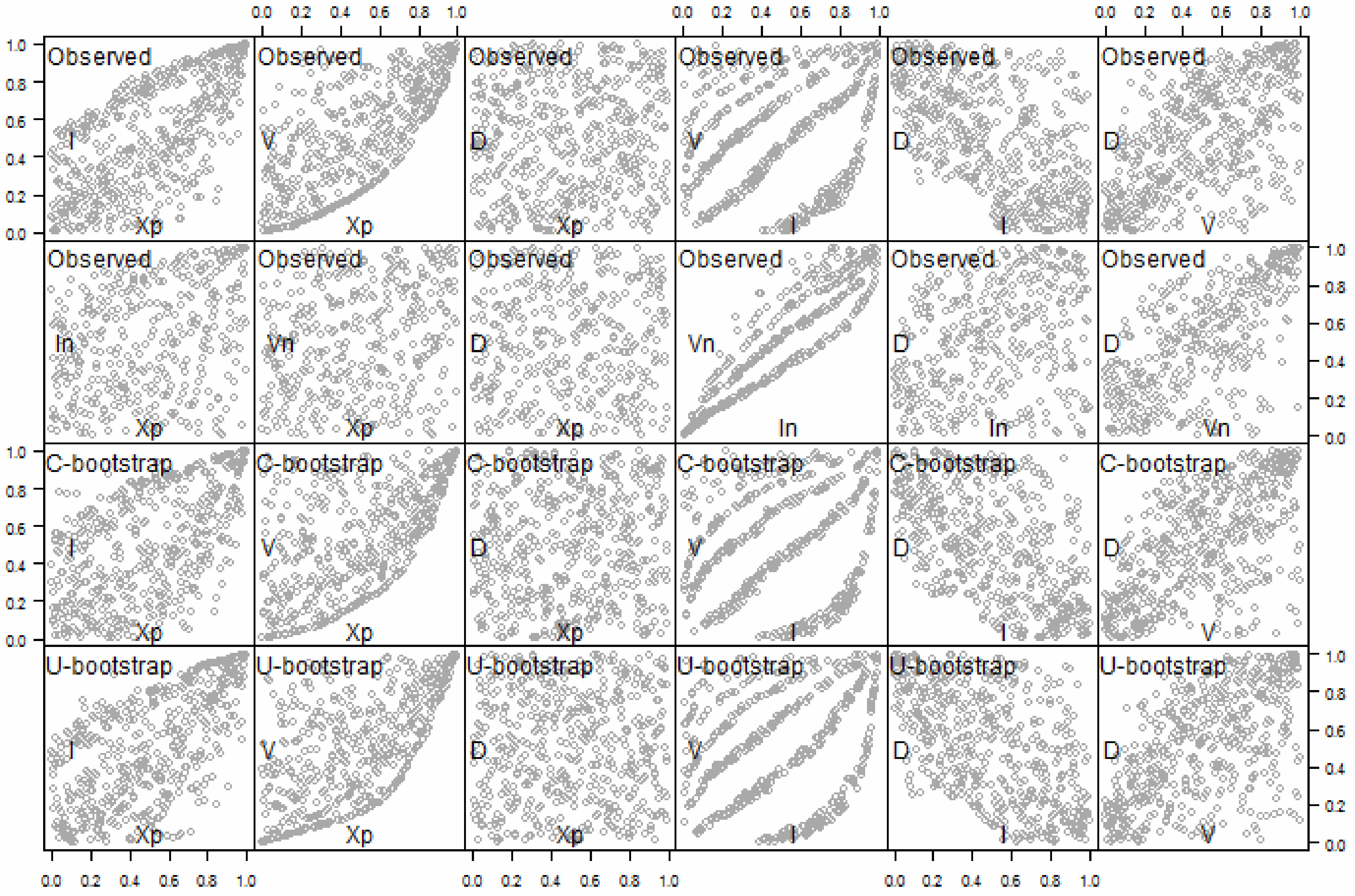

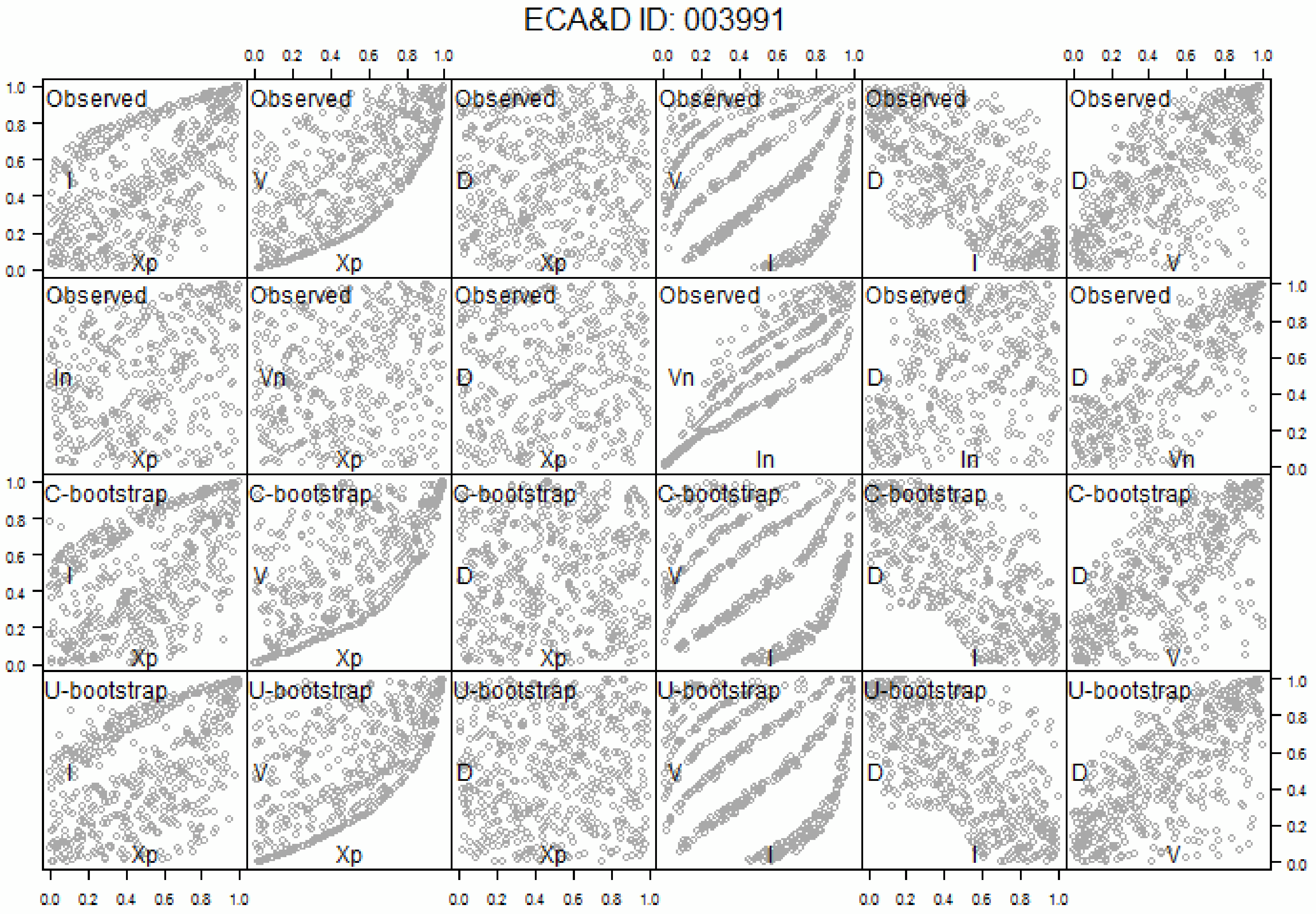

ECA&D ID: 003991
Observed
C-bootstrap
U-bootstrap
Xp
I
V
D
In
Vn
0.0
0.2
0.4
0.6
0.8
1.0

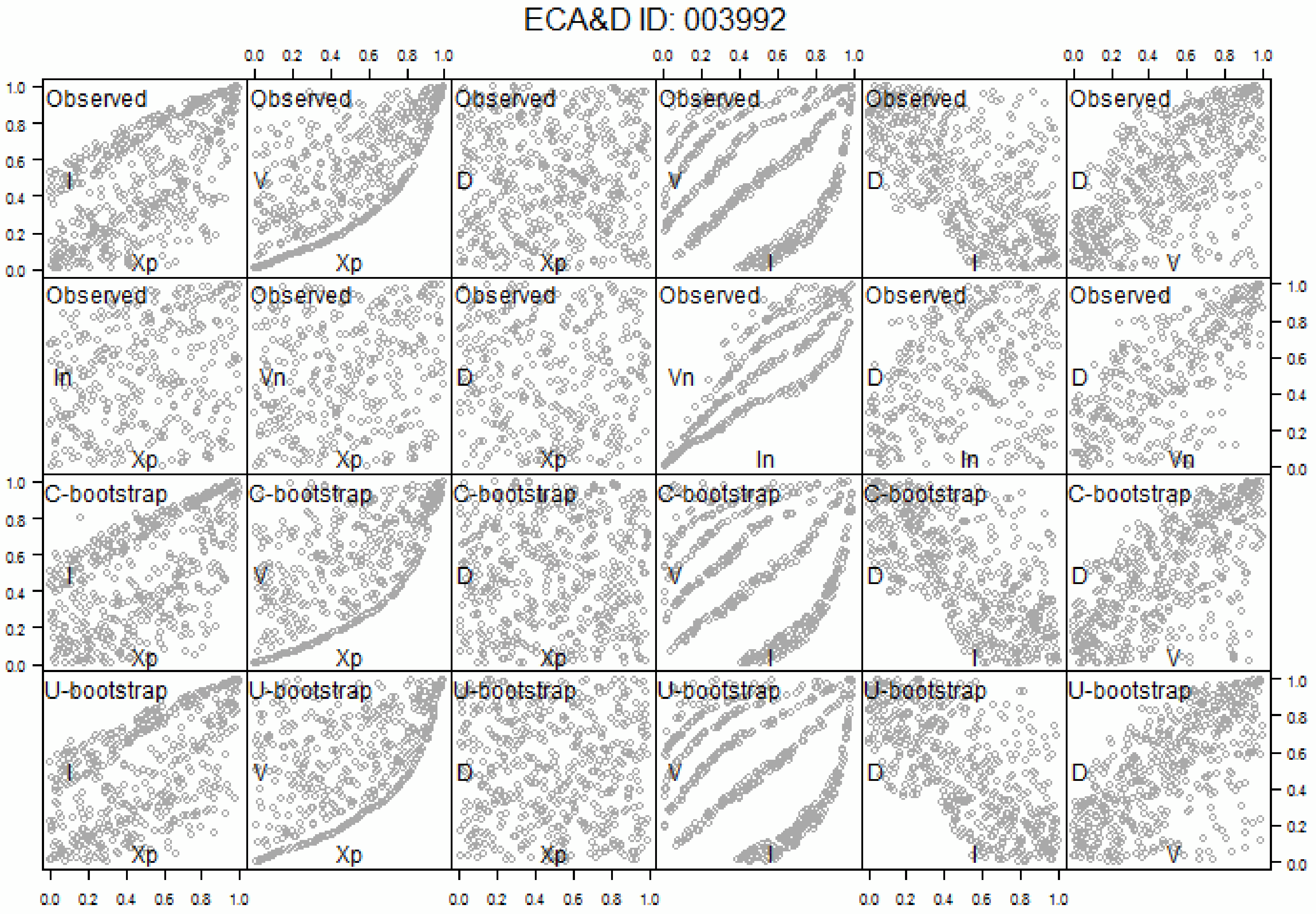
ECA&D ID: 003992
Observed
C-bootstrap
U-bootstrap
Xp
I
V
D
In
Vn
0.0
0.2
0.4
0.6
0.8
1.0

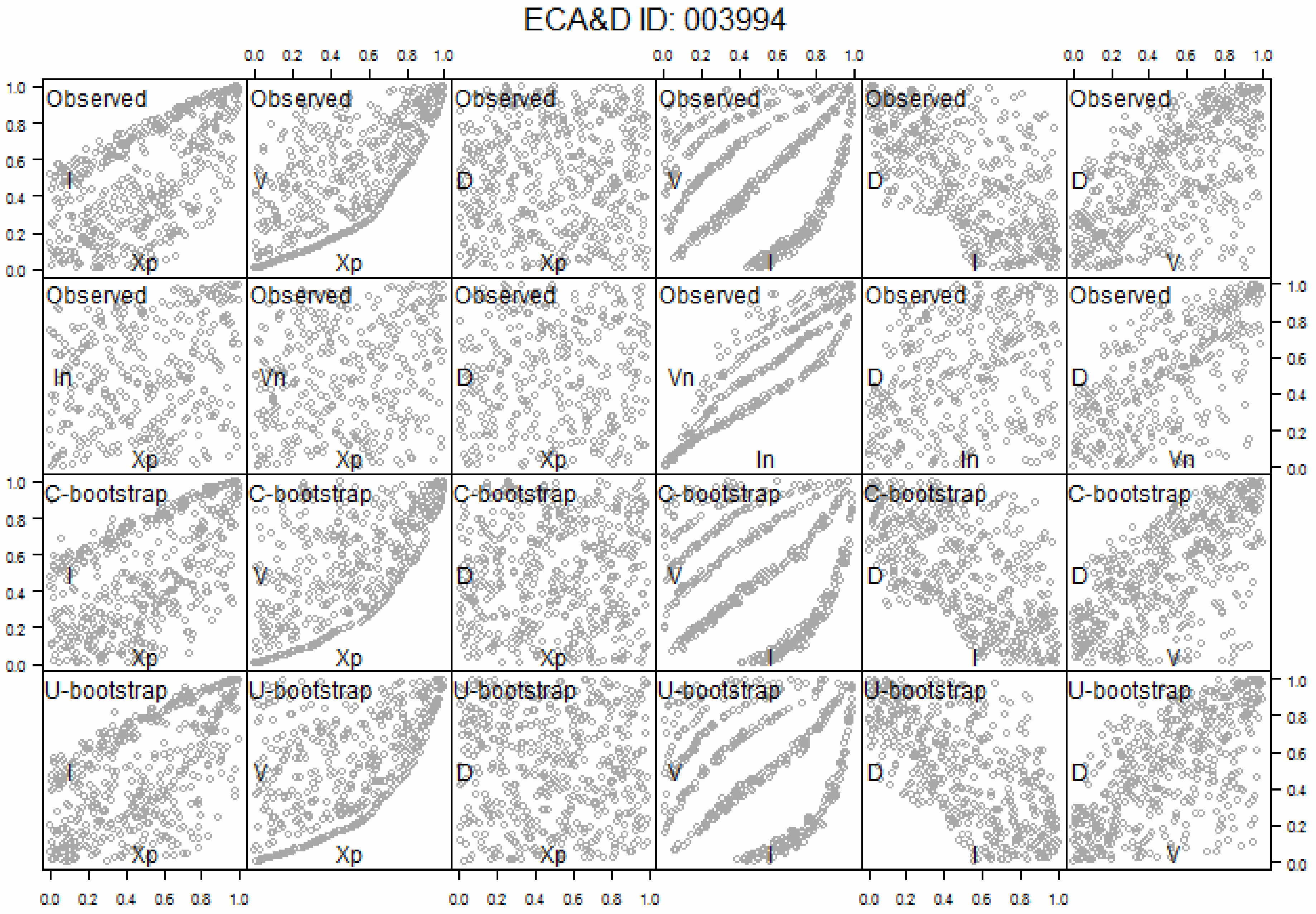
ECA&D ID: 003994
Observed
C-bootstrap
U-bootstrap
Xp
I
V
D
In
Vn
0.0
0.2
0.4
0.6
0.8
1.0

ECA&D ID: 003996

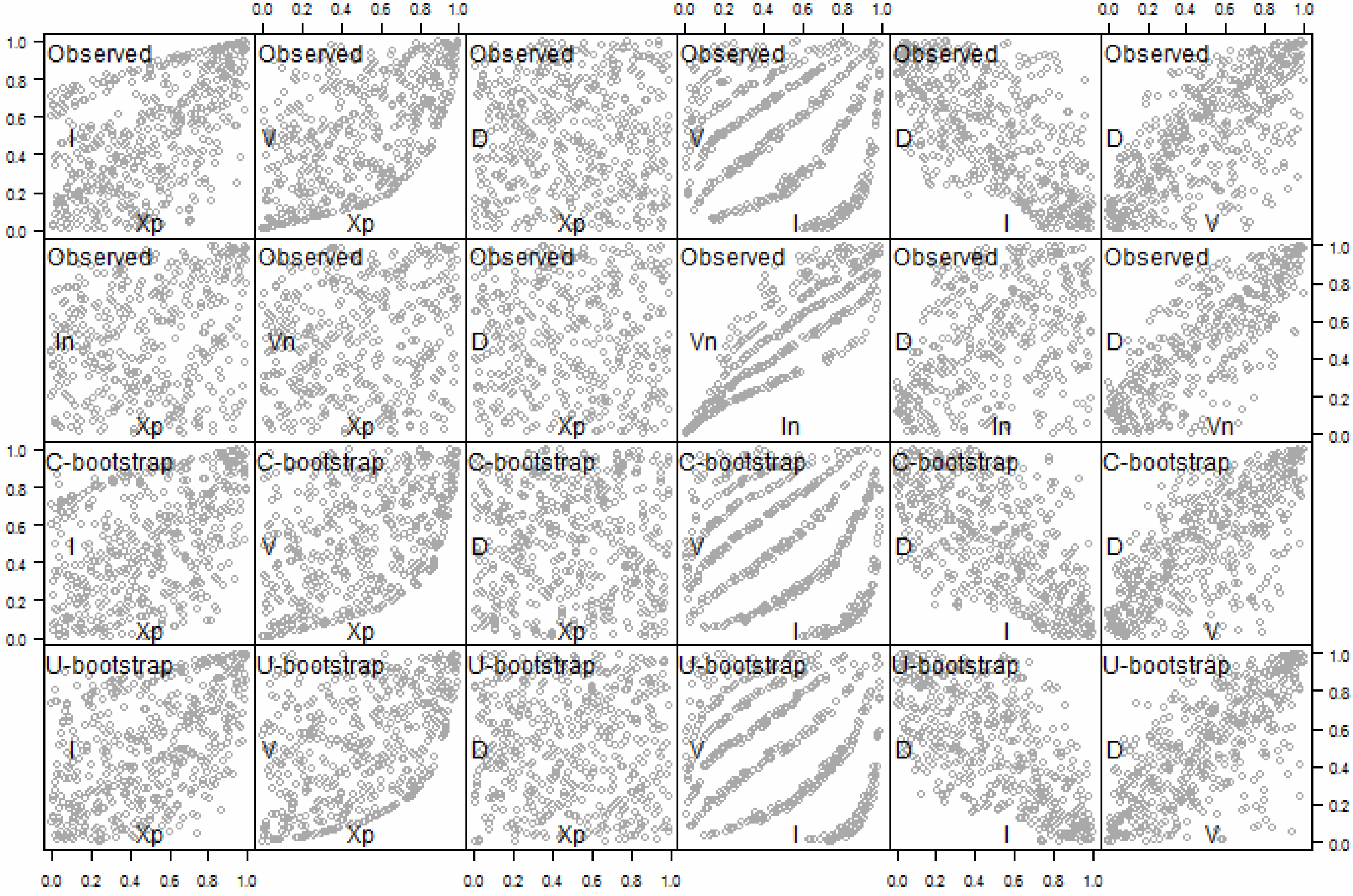

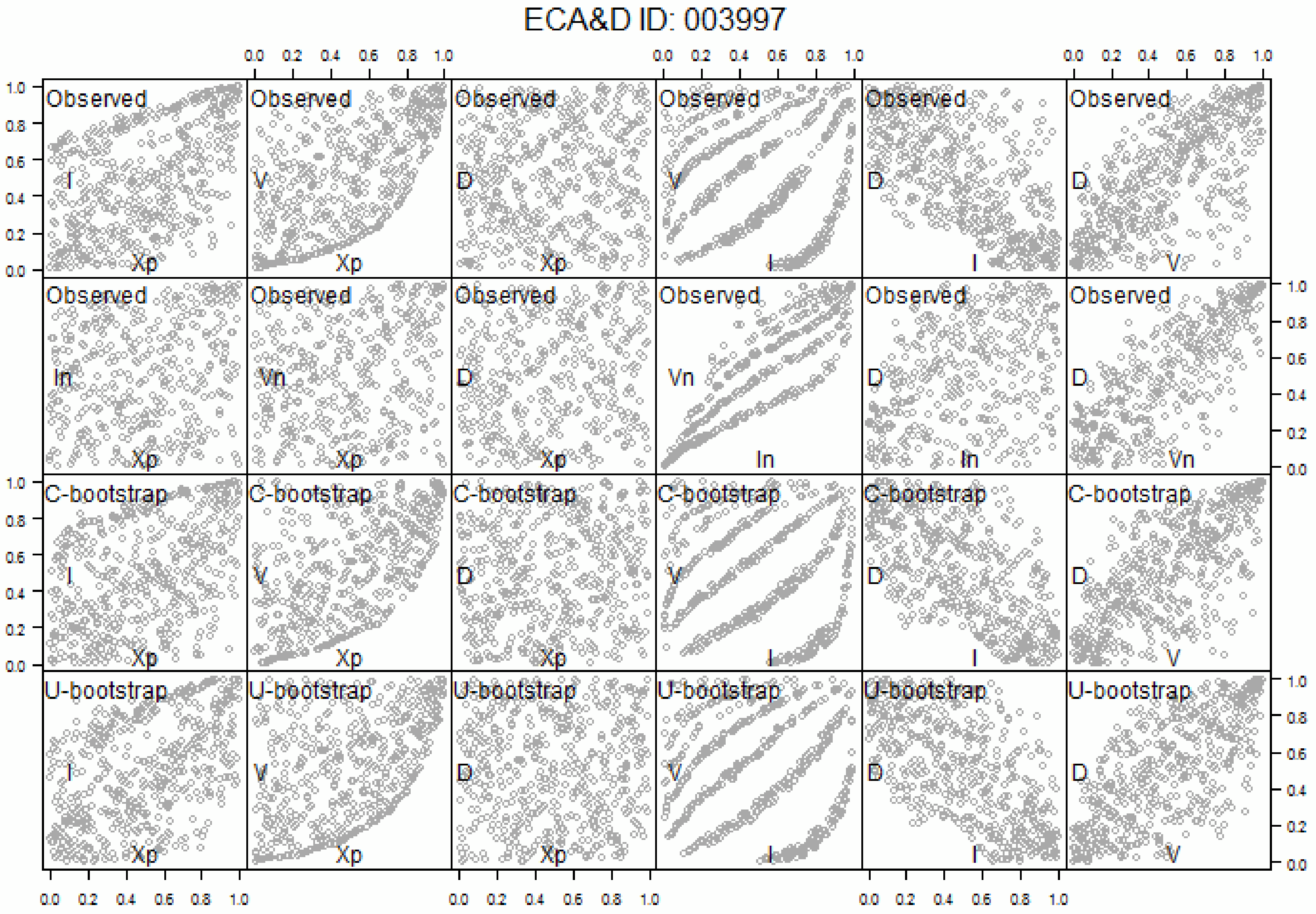
ECA&D ID: 003997
Observed
C-bootstrap
U-bootstrap
Xp
I
V
D
In
Vn
0.0
0.2
0.4
0.6
0.8
1.0

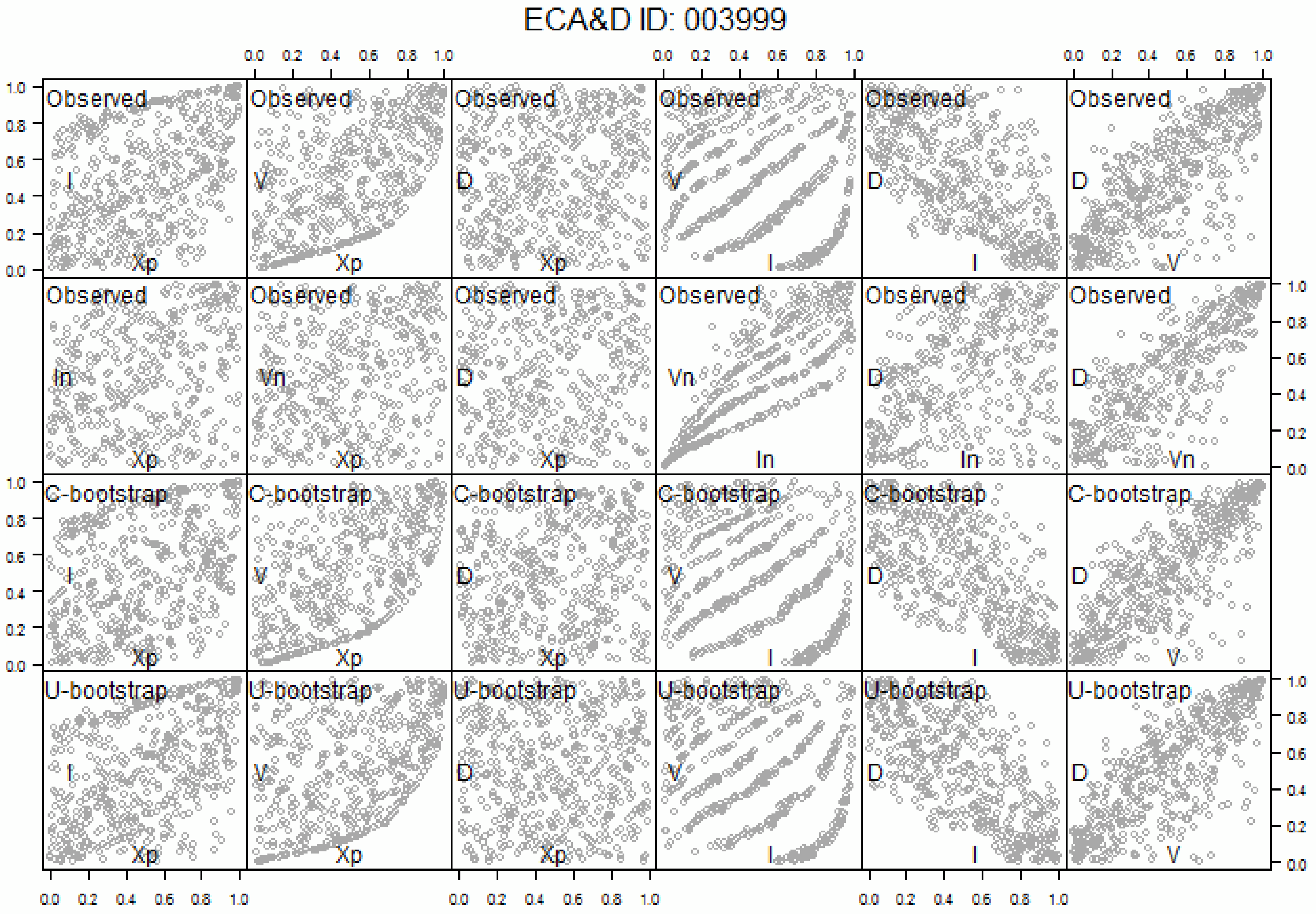
ECA&D ID: 003999
Observed
C-bootstrap
U-bootstrap
Xp
I
V
D
In
Vn

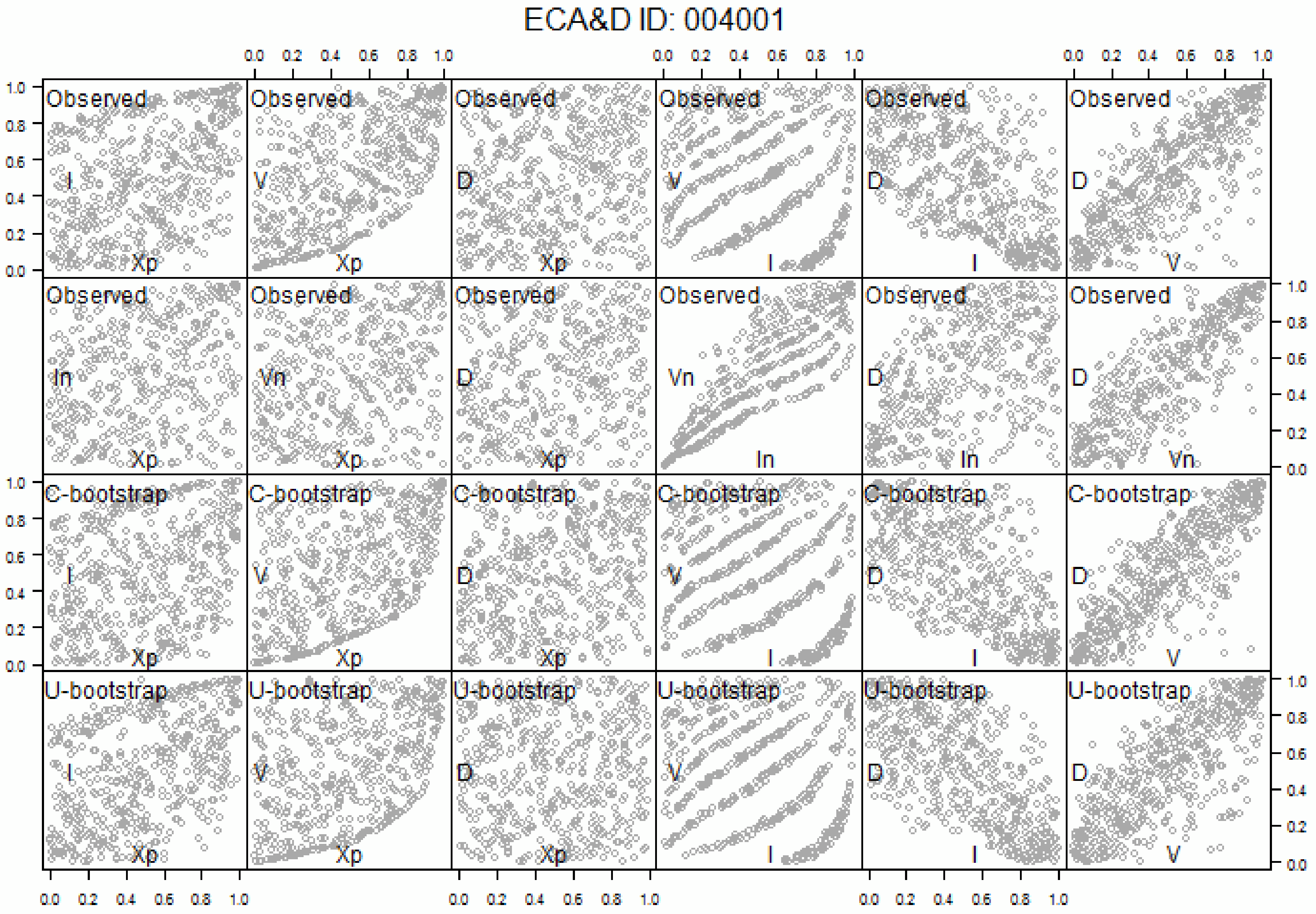
ECA&D ID: 004001
Observed
C-bootstrap
U-bootstrap
Xp
I
V
D
In
Vn
0.0
0.2
0.4
0.6
0.8
1.0

ECA&D ID: 004002

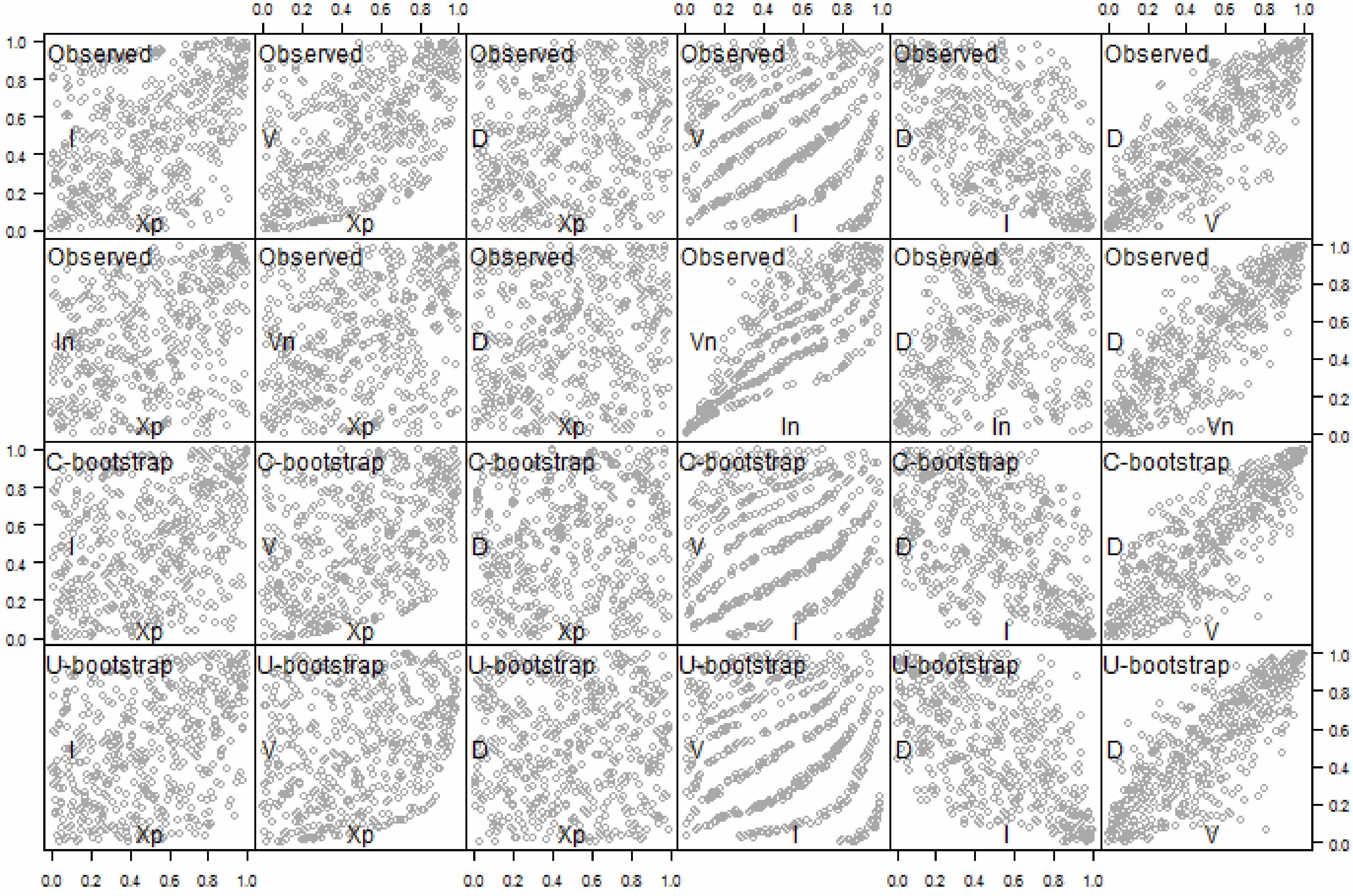

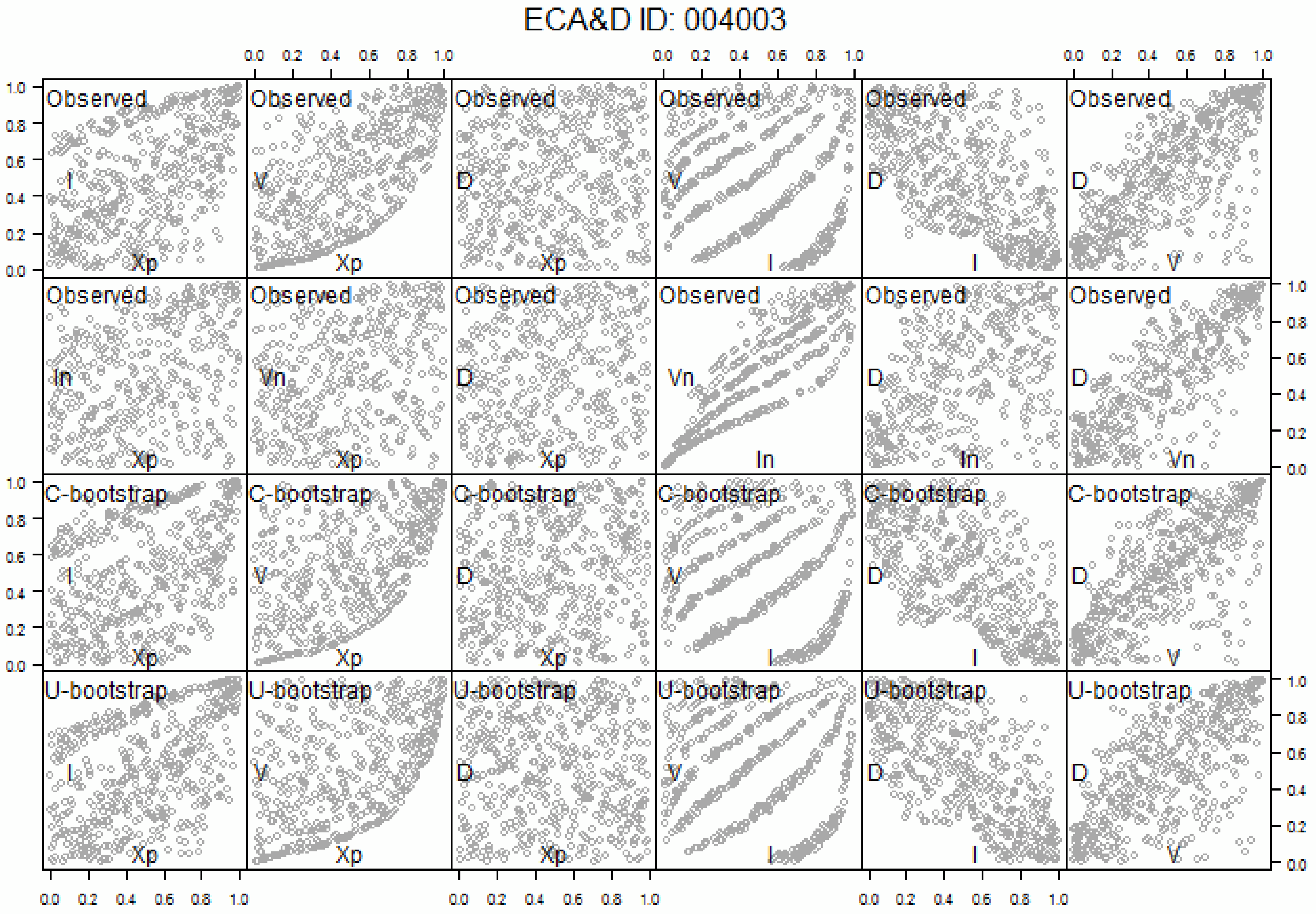
ECA&D ID: 004003
Observed
C-bootstrap
U-bootstrap
Xp
I
V
D
In
Vn
0.0
0.2
0.4
0.6
0.8
1.0

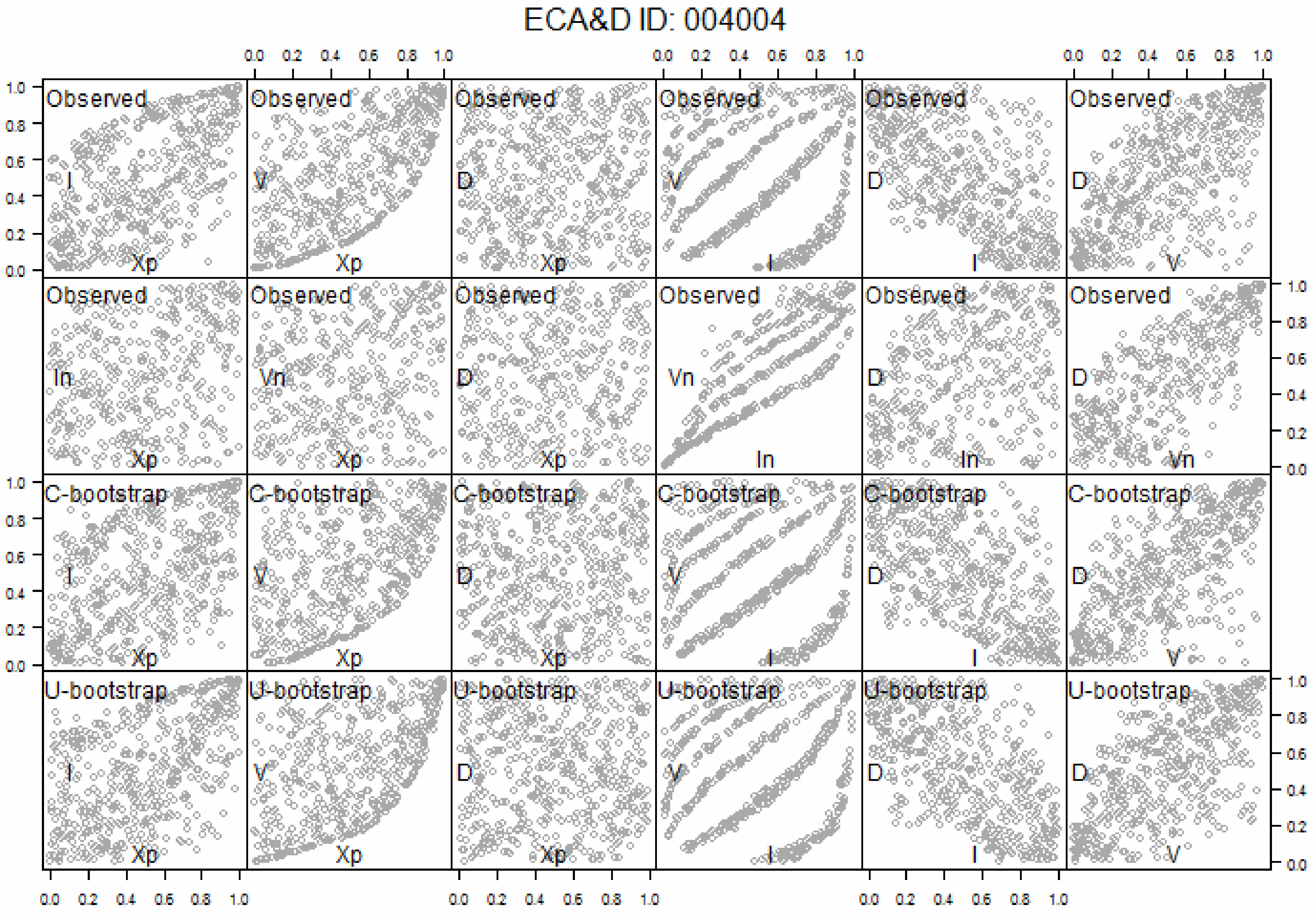
ECA&D ID: 004004
Observed
C-bootstrap
U-bootstrap
Xp
I
V
D
In
Vn
0.0
0.2
0.4
0.6
0.8
1.0

ECA&D ID: 004005

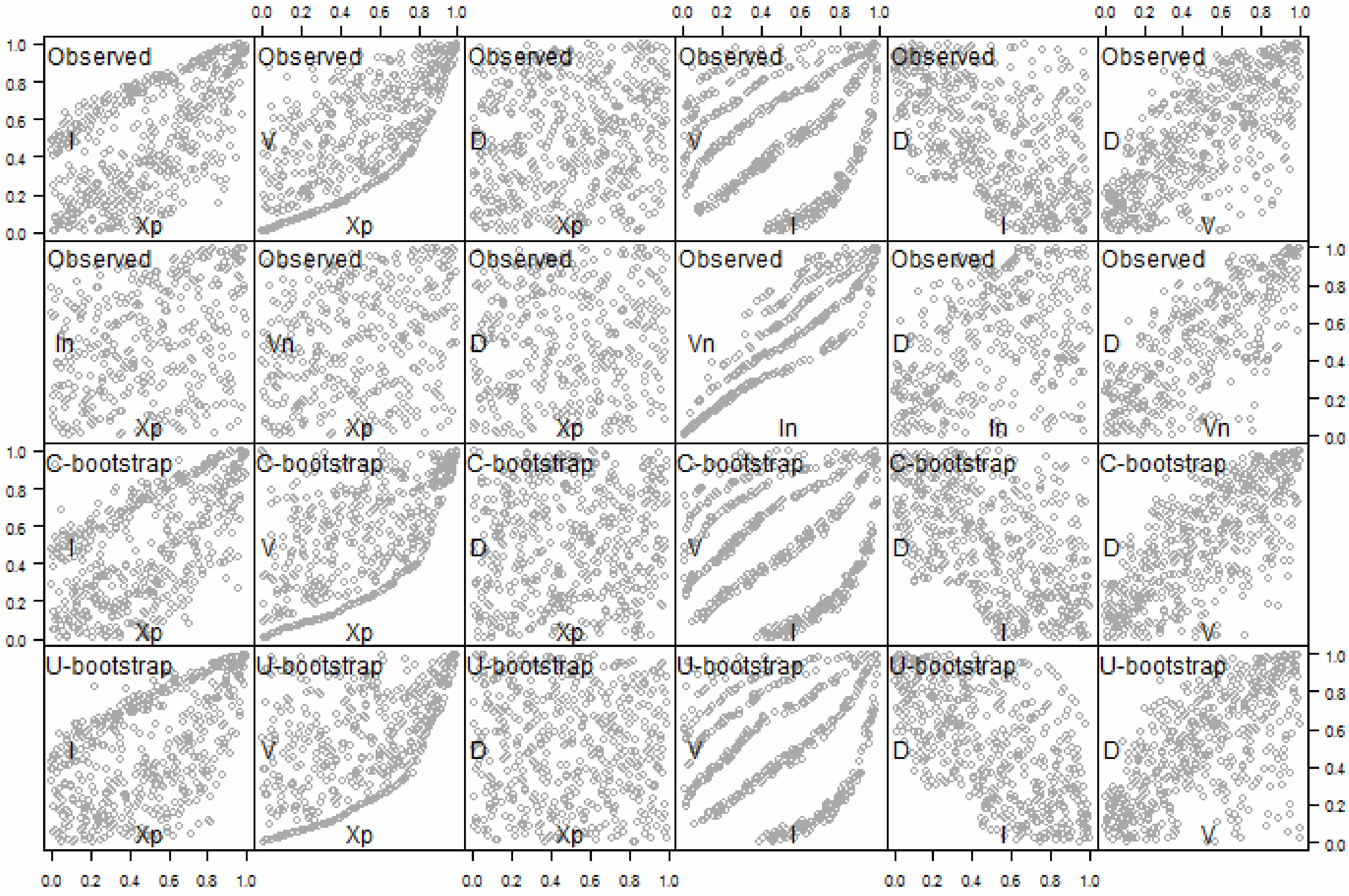

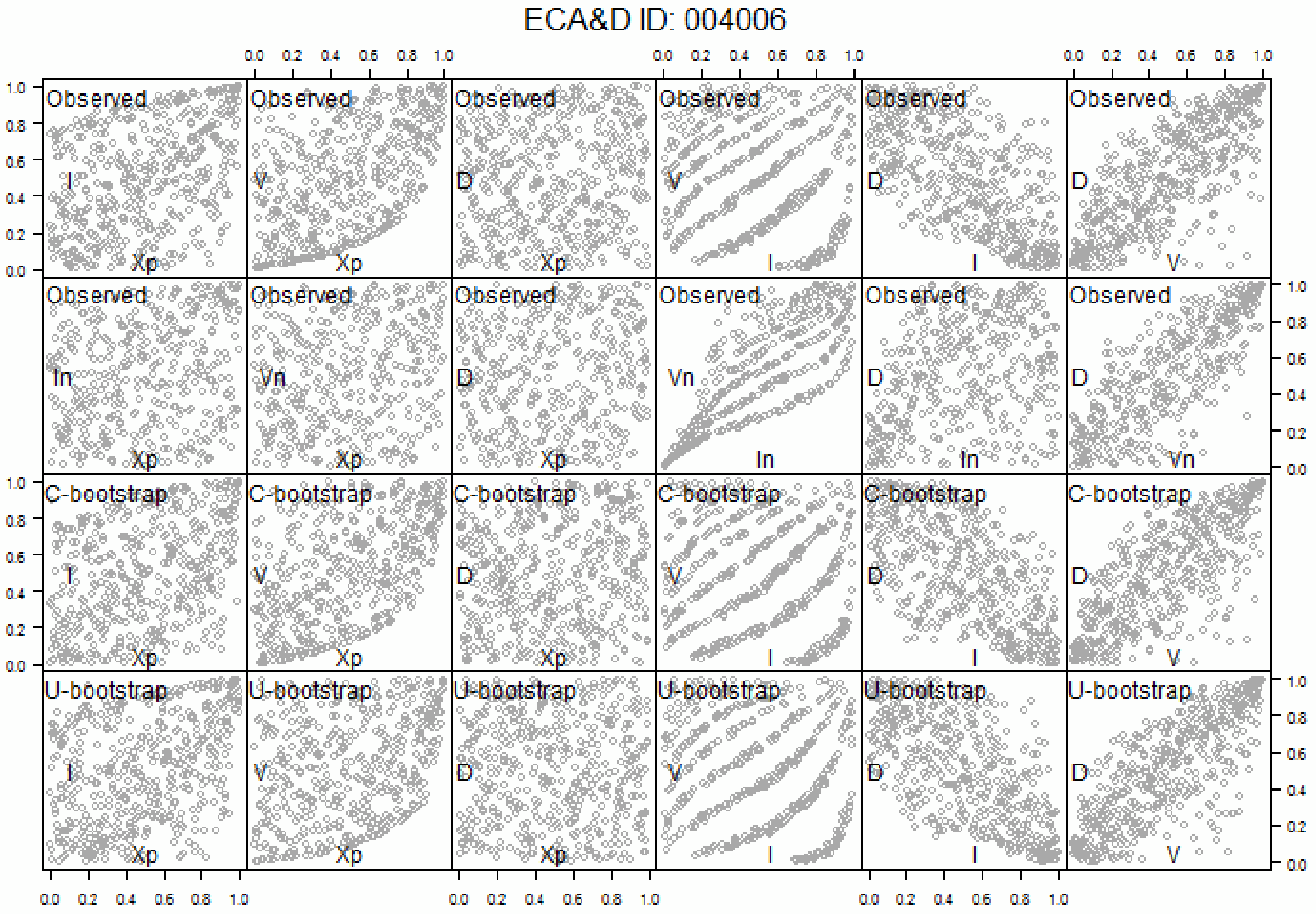

ECA&D ID: 004006
Observed
C-bootstrap
U-bootstrap
Xp
I
V
D
In
Vn
0.0
0.2
0.4
0.6
0.8
1.0

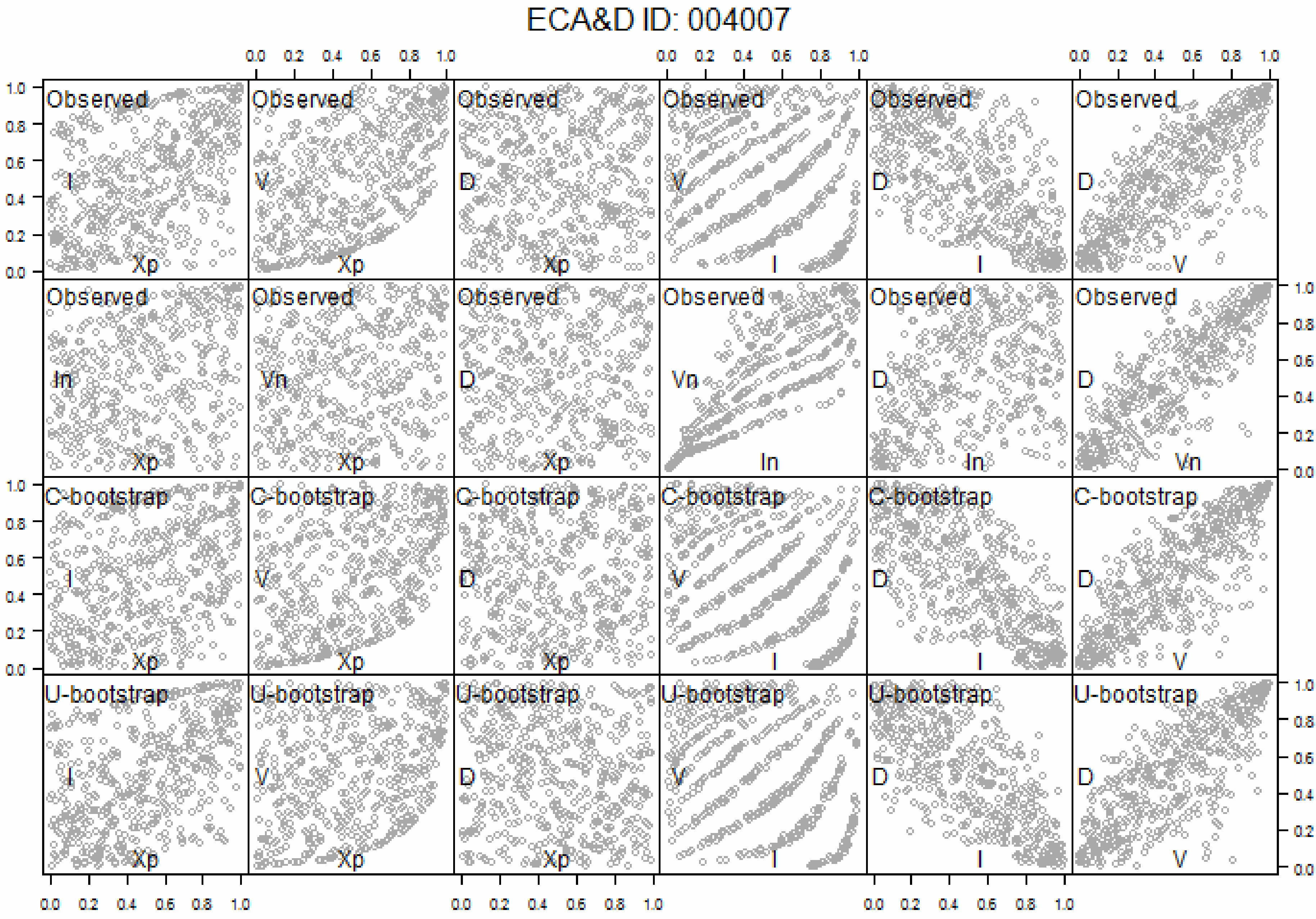

ECA&D ID: 004007
Observed
C-bootstrap
U-bootstrap
Xp
I
V
D
In
Vn
0.0
0.2
0.4
0.6
0.8
1.0

ECA&D ID: 004008

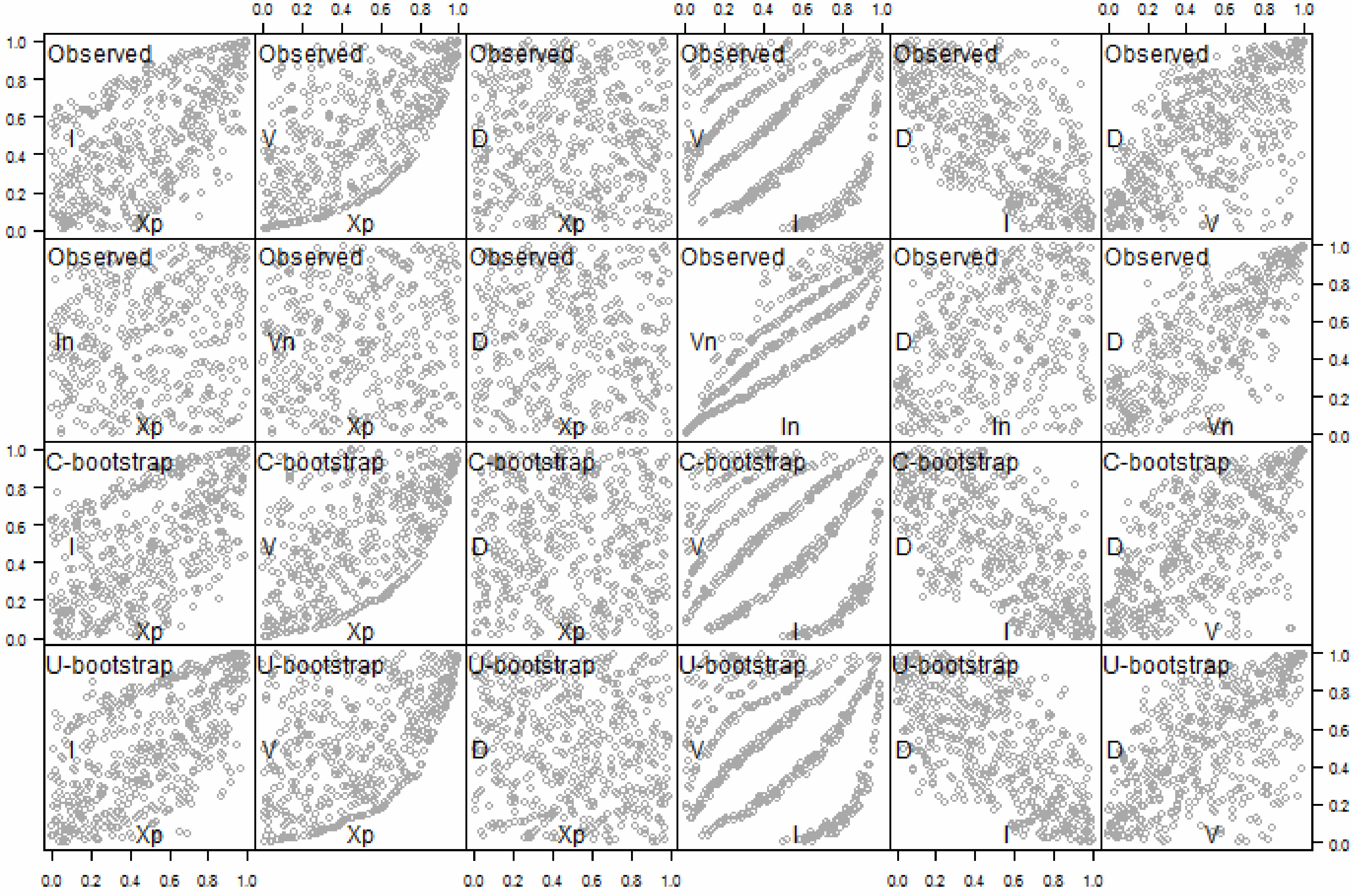

ECA&D ID: 004009

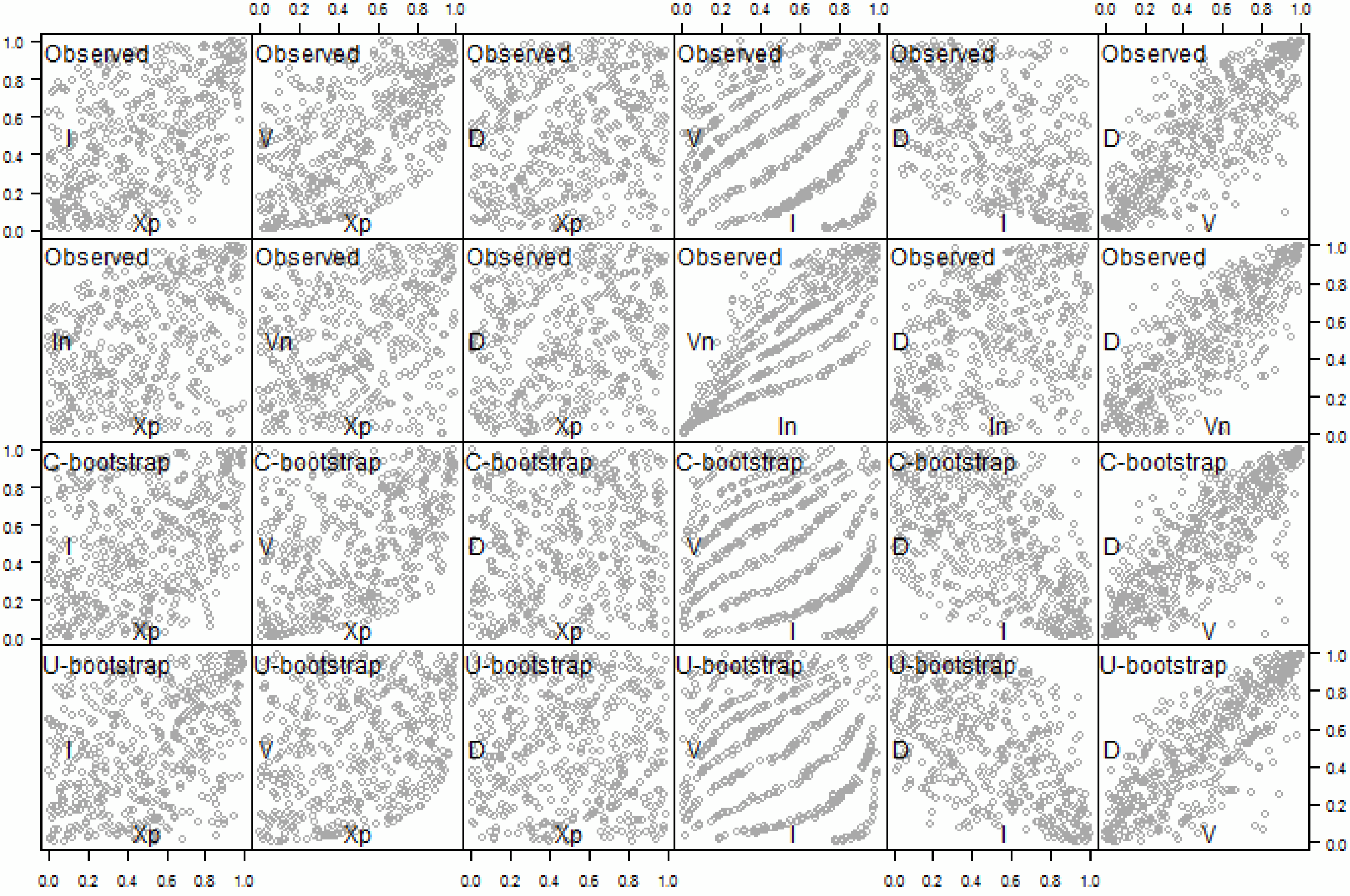

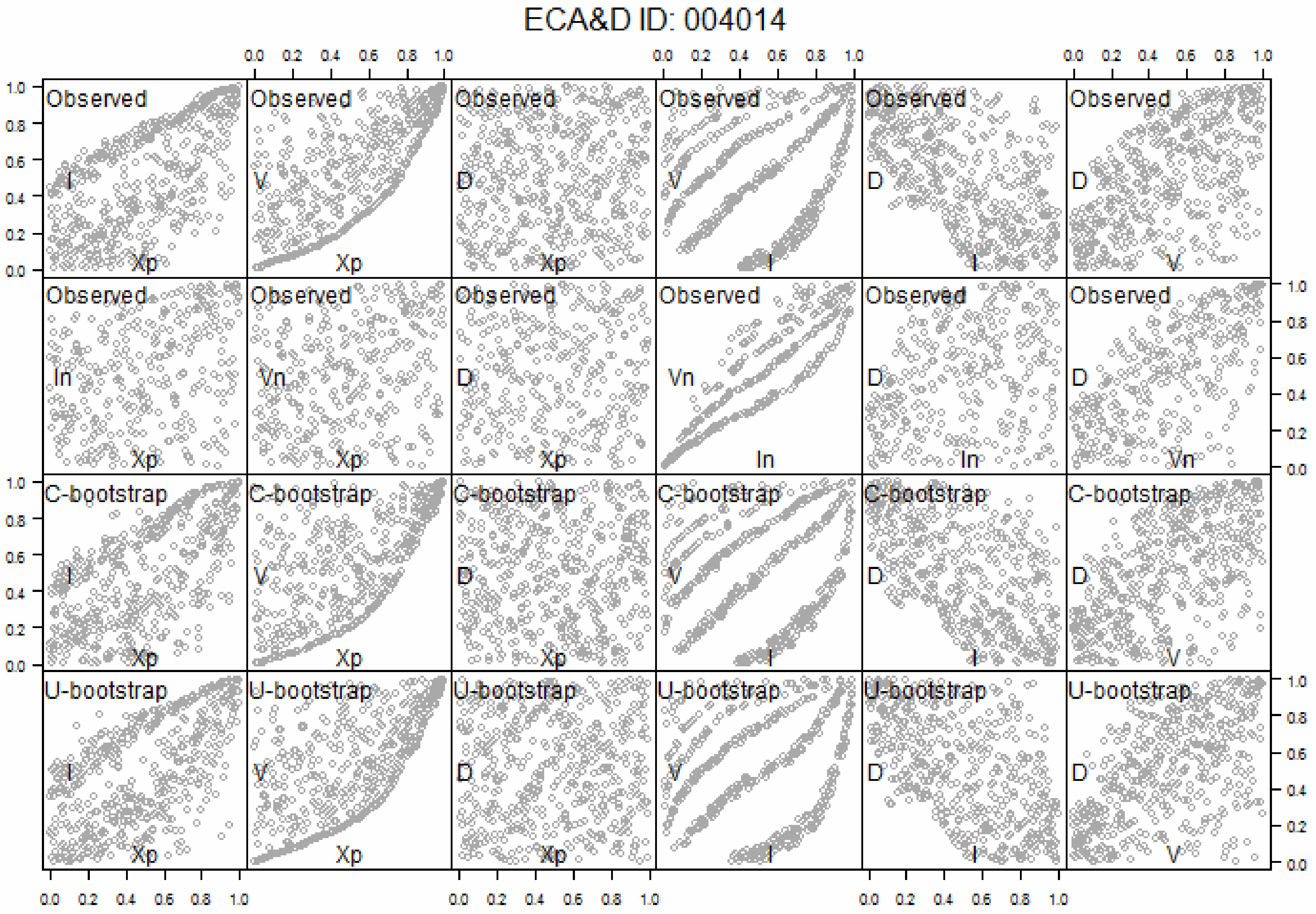
ECA&D ID: 004014
Observed
C-bootstrap
U-bootstrap
Xp
I
V
D
In
Vn
0.0
0.2
0.4
0.6
0.8
1.0

ECA&D ID: 004015

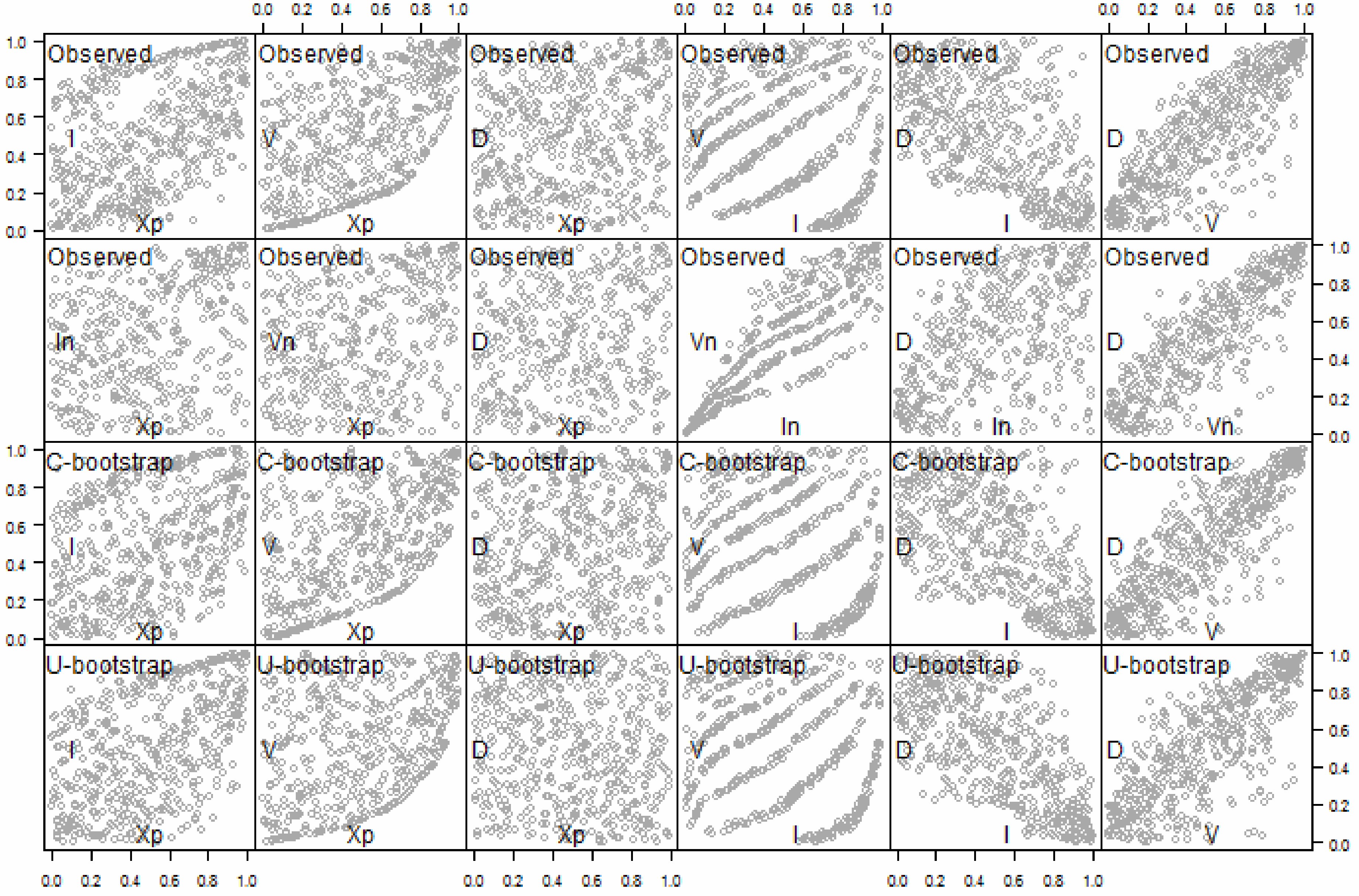

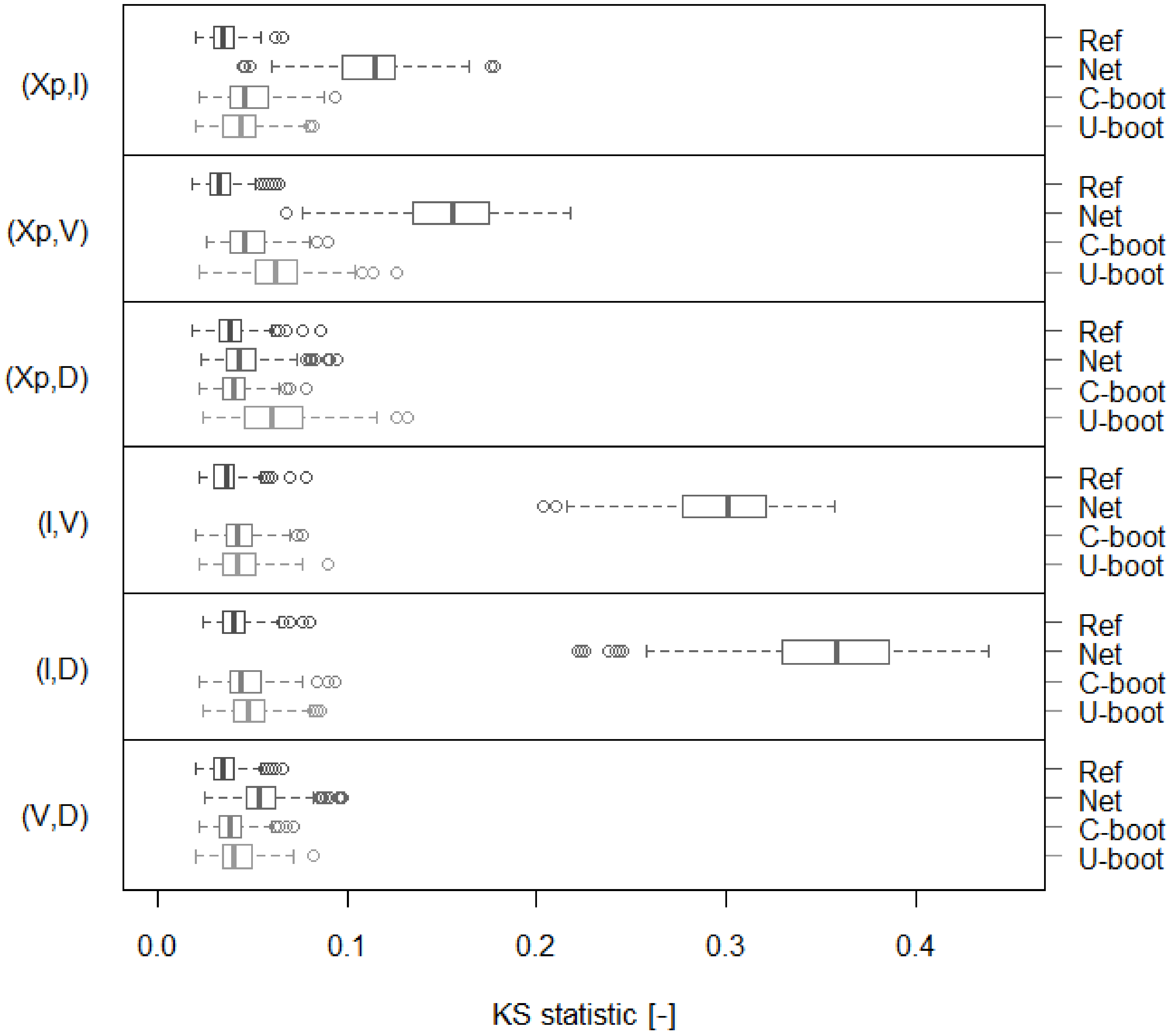
(Xp,I)
(Xp,V)
(Xp,D)
(I,V)
(I,D)
(V,D)
Ref
Net
C-boot
U-boot
0.0
0.1
0.2
0.3
0.4
KS statistic [-]

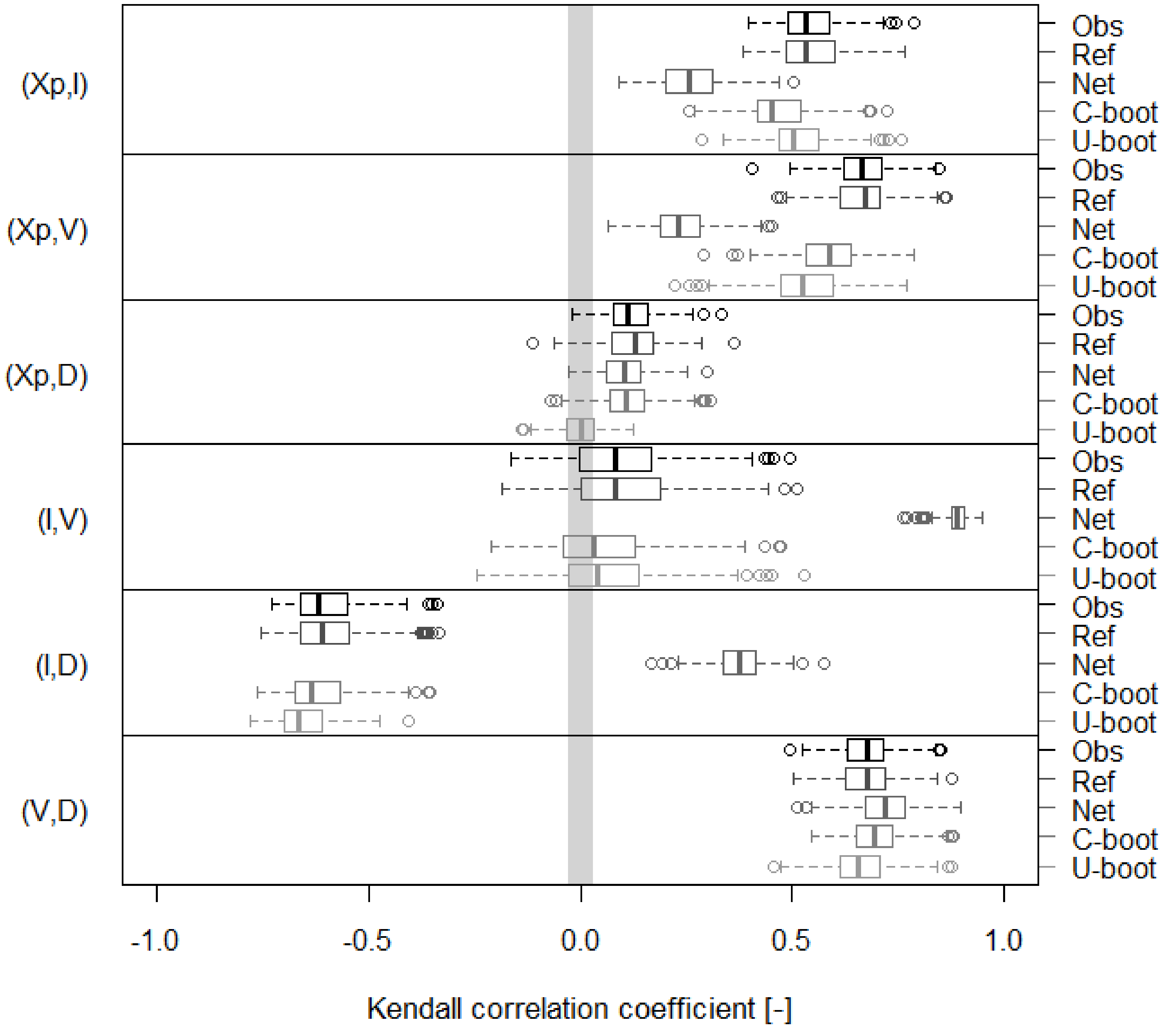

(Xp,I)
(Xp,V)
(Xp,D)
(I,V)
(I,D)
(V,D)
Obs
Ref
Net
C-boot
U-boot
-1.0
-0.5
0.0
0.5
1.0
Kendall correlation coefficient [-]
